# Simple sequence proteins in prokaryotic proteomes

**DOI:** 10.1186/1471-2164-7-141

**Published:** 2006-06-08

**Authors:** Mekapati Bala Subramanyam, Muthiah Gnanamani, Srinivasan Ramachandran

**Affiliations:** 1 G.N. Ramachandran Knowledge Centre for Genome Informatics, Institute of Genomics and Integrative Biology, Mall road, Delhi-110007, India

## Abstract

**Background:**

The structural and functional features associated with Simple Sequence Proteins (SSPs) are non-globularity, disease states, signaling and post-translational modification. SSPs are also an important source of genetic and possibly phenotypic variation. Analysis of 249 prokaryotic proteomes offers a new opportunity to examine the genomic properties of SSPs.

**Results:**

SSPs are a minority but they grow with proteome size. This relationship is exhibited across species varying in genomic GC, mutational bias, life style, and pathogenicity. Their proportion in each proteome is strongly influenced by genomic base compositional bias. In most species simple duplications is favoured, but in a few cases such as Mycobacteria, large families of duplications occur.

Amino acid preference in SSPs exhibits a trend towards low cost of biosynthesis. In SSPs and in non-SSPs, Alanine, Glycine, Leucine, and Valine are abundant in species widely varying in genomic GC whereas Isoleucine and Lysine are rich only in organisms with low genomic GC. Arginine is abundant in SSPs of two species and in the non-SSPs of *Xanthomonas oryzae*. Asparagine is abundant only in SSPs of low GC species. Aspartic acid is abundant only in the non-SSPs of *Halobacterium *sp NRC1. The abundance of Serine in SSPs of 62 species extends over a broader range compared to that of non-SSPs. Threonine(T) is abundant only in SSPs of a couple of species. SSPs exhibit preferential association with Cell surface, Cell membrane and Transport functions and a negative association with Metabolism. Mesophiles and Thermophiles display similar ranges in the content of SSPs.

**Conclusion:**

Although SSPs are a minority, the genomic forces of base compositional bias and duplications influence their growth and pattern in each species. The preferences and abundance of amino acids are governed by low biosynthetic cost, evolutionary age and base composition of codons. Abundance of charged amino acids Arginine and Aspartic acid is severely restricted. SSPs preferentially associate with cell surface and interface functions as opposed to metabolism, wherein proteins of high sequence complexity with globular structures are preferred. Mesophiles and Thermophiles are similar with respect to the content of SSPs. Our analysis serves to expandthe commonly held views on SSPs.

## Background

Simple Sequence Proteins (SSPs) are composed of various types of amino acid repeats such as amino acid runs [1], regular repeats and cryptic repeats [2]. SSPs can be recognized by their compositional bias. Early work by Wootton and Federhen [3] showed that simple sequence segments are either part of non-globular regions or of linkers between structural or functional domains. Following this work, simple sequence segments were usually masked during database searches and therefore they did not receive wide attention for a long time. The observation that expansion of polyglutamine tracts in proteins cause several human neurological diseases led to a surge in interest in investigating the function, distribution and evolution of reiterated sequences in proteins [1,4]. Recent observations suggest that compositionally biased sequences in many proteins are structurally disordered, and these disordered segments participate in important functional roles such as signaling and post-translational modifications [5-7]. Sequence segments such as polyglutamine tracts and proline rich sequences could mediate protein-protein interactions [8-10] and charged segments such as arginine-rich regions are often involved in protein-RNA interactions [11]. Investigation of functional associations of SSPs in yeast revealed that they were preferentially associated with transcription factors and signaling proteins [12]. Analysis of the ratio of non-synonymous (Ka) to synonymous (Ks) divergences of gene sequences encoding SSPs orthologously conserved between human and mouse revealed that these proteins are under strong purifying selection [2]. However, the extent of operation of selective forces may vary depending on the functional role. For example, SSPs functioning in cellular processes display higher degree of conservation that parallels taxonomic divergence patterns compared to those functioning at the interface between the organism and its niche (Transport & membrane proteins) or those that carryout species specific specialized functions [13]. These results strongly suggest that reiterated sequence motifs in proteins are involved in important biological processes and the tempo and mode of their evolution are constrained by their functional roles.

Another major interest in simple sequences stems from the observation that they constitute an important source of genetic (and possibly phenotypic) variation [14]. Sequences composed of simple sequence repeats undergo expansion/contraction polymorphisms due to slippage during replication and also in some cases insertion/deletion polymorphisms due to intra-chromosomal or unequal crossing-over recombinogenic events [15-17]. These molecular events occur in the genomic DNA and therefore early efforts in identifying simple sequences were focused on analyzing nucleic acid sequences. It has now become clear that correlations between simple sequence regions in proteins and the encoding DNA are not always observed [18]. This in principle is due to the codon degeneracy. SSPs encoded by scrambled mixtures of codons are likely to be missed if analysis was restricted to DNA sequence alone. Nonetheless these SSPs are still interesting because their evolution is likely due to selection on protein structure or function.

Previously, we developed a measure to analyze protein sequences and classify proteomes in a binary mode into two categories: SSPs and non-SSPs. This measure considers the entire protein sequence and SSPs are identified according to the proportion of simple sequences carried in them [19]. Our approach using whole protein sequence to identify SSPs is general in that, all forms of repeats are identified, and suited for comparative analysis in the same framework as described recently by Sim and Creamer, [20]. In this work we present the analysis of SSPs from 249 prokaryotes.

## Results and discussion

### Growth of SSPs: proteome size, genomic GC bias and duplications

**Figure 1:**
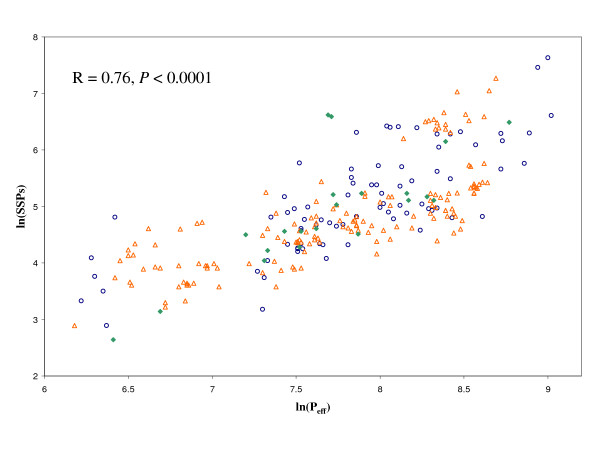
The number of SSPs in each species of prokaryotes grows with its effective proteome size. The correlation coefficient and the *P *value for statistical significance are shown. Open blue circles (○) represent non-pathogens; Open red triangles (△) are pathogens; other prokaryotes are marked by filled green diamonds (◆).

A proteome of a given species can be considered as a collection of protein sequences of that species [21]. From this point of view, proteome size refers to the number of proteins in a given collection. The term effective proteome size (P_eff_) in this work refers to the number of proteins of length greater than 45 amino acids. Because the number of such small proteins is very low, it is unlikely to affect the general analysis. The relationship between the number of SSPs and the effective proteome size from 249 proteomes is shown in Figure 1. It is apparent that this relationship follows a proportionate relation in a log-log scale (Correlation coefficient R = 0.76, *P *< 0.0001). On an average, the number of SSPs in a proteome is approximately proportional to 1/30^th ^of the proteome size although there is considerable variation in the dataset and ranges from 1.62% in *Thermoplasma acidophilum *to as high as 34.1% in *Thermus thermophilus*. These observations show that although SSPs are a minority, they tend to grow with proteome size. This relationship is exhibited across species varying in genomic GC content, mutational bias, life style and pathogenicity (See Additional file 1).

**Figure 2:**
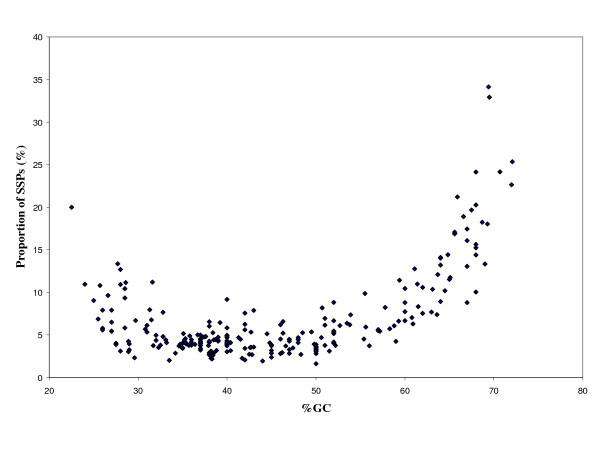
Relationship between the proportion of SSPs (expressed as percent fraction of P_eff_) and the genomic GC content in each species.

Although a general growth trend is apparent, a significant variability can be noticed. Two potential factors contributing to this variability are genomic base composition bias and gene duplications. In order to assess the base bias effect, we determined the relationship between the proportion of SSPs in each species and its genomic GC (Figure 2). It is evident that in species with low or high genomic GC, the proportion of SSPs is higher compared to the species with mid-range GC. These results show that biased genomic base composition results in a higher proportion of SSPs. We examined the relationship between the number of SSPs and the effective proteome size (log-log scale) in species varying in GC in three ranges: 22.5%–32%, 32%–60% and 60%–72%. The correlation coefficients varied from 0.6 to 0.8 and were all highly statistically significant (*P *< 0.0001). These results show that, while there is a general trend of SSPs to grow with proteome size across all types of species, genomic GC bias can strongly influence this trend.

According to the model of Qian et al. [22], genomes evolve from their initial small size using two basic operations: (1) duplication of existing genes to expand the size of existing families, and (2) introduction of new genes by either lateral transfer from other organisms or *ab initio *creation. An approach to examine the role of duplicative processes is by following the growth of the number of paralogous pairs among the SSPs of each species with increasing number of SSPs.

**Figure 3:**
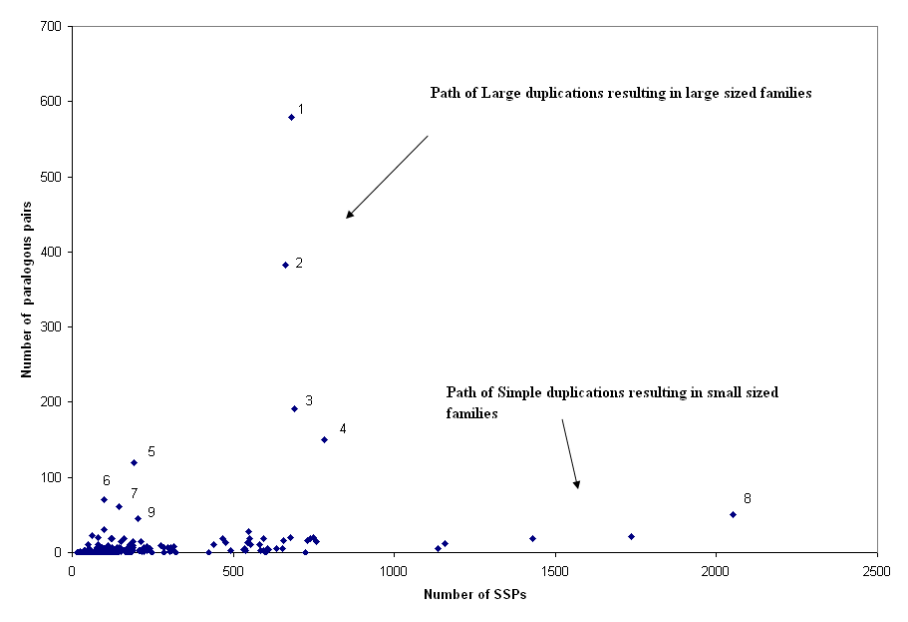
Distribution of the number of paralogs (computed as pairs for ease of visual inspection in distinguishing large duplications from small ones) in SSPs of prokaryotes. Species with large number of paralogous pairs are marked: 1. *Mycobacterium tuberculosis *H37Rv; 2. *Mycobacterium bovis *AF2122/97; 3. *Mycobacterium tuberculosis *CDC1551; 4. *Mycobacterium avium subsp paratuberculosis *K-10; 5. *Borrelia burgdorferi *B31; 6. *Onion yellows phytoplasma *OY-M; 7. *Leptospira interrogans *serovar Lai str56601; 8. *Streptomyces coelicolor *A32; 9. *Escherichia coli *O157H7.

The relationship between the number of paralogous pairs among the SSPs and the total numbers of SSPs of each species is displayed in Figure 3. This method enables ready differentiation of simple duplications from large duplications. Simple duplications result in clusters of small size, usually 2 members per cluster. Large duplications on the other hand yield clusters of large size comprising of more than 2 members per cluster. Large clusters with high number of pairs can be easily separated from small ones by computing the number of pairs of paralogs in each cluster. It is evident that, most species have small sized clusters with low pairs of paralogs indicating that simple duplications is the generally favoured trend. The summit in the path of simple duplications (Figure 3, marked point no. 8) belongs to that of *Streptomyces coelicolor *A3(2) with 80 paralogs.

A few species deviate from this general trend and follow a vertical path (see Figure 3, marked points except no.8). These species have large sized clusters of paralogs resulting in large sized families of SSPs. It is to be noted that, while all of these species are pathogens and most of them have highly skewed base composition in their genomes, these factors do not appear to be sole contributors to large duplications because several other pathogens with skewed genomic base composition do not display this trend. The large duplications in these selected pathogens, particularly Mycobacteria, is perhaps more related to specific host-pathogen interactions, tropisms and lineage specific duplications. Indeed, a large number of these proteins in Mycobacteria belong to PE_PGRS and PPE families with potential role in host-pathogen interactions [23-26]. Many of these proteins are adhesin-like proteins with P_ad _≥ 0.7 (See Additional files 2 and 3). Furthermore, the reiterated sequence parts display similarity to antigens from other species. These results, taken together support the surface characteristics of these proteins.

SSPs grow with proteome size and their proportion in each proteome is strongly influenced by genomic base compositional bias. In most species, simple duplications is the main player. In a few species, SSPs arise from genomic forces of large duplications dedicated to specific host-pathogen interactions.

### Amino acids in SSPs and non-SSPs: similarities and differences

**Figure 4:**
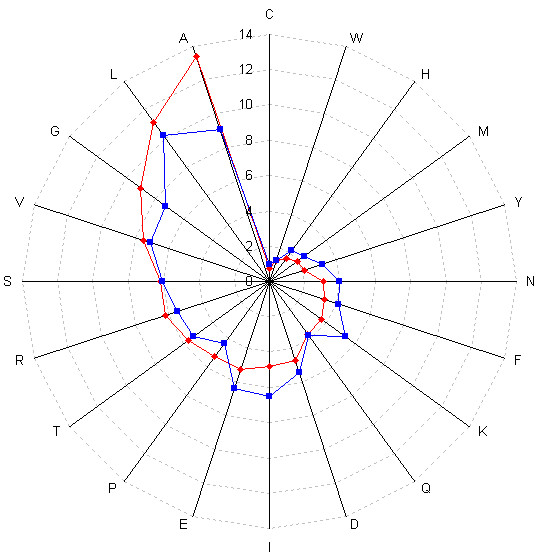
Amino acid content differences between SSPs and non-SSPs. Red contour: SSPs; Blue contour: non-SSPs. The numbers at the concentric circles correspond to percent values of amino acid content. Amino acids are shown using single letter code.

The comparison of amino acid content between the SSPs and non-SSPs is shown in Figure 4. SSPs have higher content of the amino acids Alanine, Leucine, Glycine and Proline whereas the non-SSPs have elevated content in many other amino acids, most strikingly in Glutamic acid, Isoleucine and Lysine. These observations suggest that SSPs prefer amino acids with low biosynthetic cost [27]. In order to examine the relationship between amino acid abundance and genomic GC, we compared the top ranking amino acids of SSPs with the genomic GC bias. The relationships between the top ranking three amino acids in SSPs and non-SSPs and the genomic GC content from various organisms are displayed in Table 1.

It is apparent that the aliphatic amino acids Alanine(A), Glycine(G), Leucine(L) and Valine(V) display similar abundance patterns in SSPs and non-SSPs of organisms varying widely in genomic GC content. Interestingly, the abundance of Glycine in SSPs persists even in low GC (33.5%) species, whereas its abundance in non-SSPs is restricted to the lowest limit GC content of 45%. The abundance of amino acids Isoleucine(I) and Lysine(K) are restricted to organisms with low genomic GC content in both SSPs and non-SSPs. Asparagine is abundant only in SSPs of species of low GC. Arginine(R) is abundant in SSPs of two species and in the non-SSPs of *Xanthomonas oryzae*. Aspartic acid was abundant only in the non-SSPs of *Halobacterium *sp NRC1 (GC 65.9%). On the other hand, Glutamic acid(E) displays similarity in abundance in SSPs and non-SSPs with respect to genomic GC content. The abundance of Serine(S) in SSPs of 62 species extends to an upper limit of 60% GC whereas it is restricted to 50% GC in non-SSPs of 19 species. Threonine(T) is abundant only in SSPs of a couple of species.

The restricted abundance of Isoleucine and Lysine in species of low genomic GC content and of Arginine in species of high genomic GC content correlates positively with the AT rich and GC rich base composition of their respective codons. On the other hand, the abundance of Alanine, Glycine, Leucine and Valine in species varying widely in genomic GC content suggests that this phenomenon relates to the evolutionary age of these amino acids instead of base compositional bias of the genomic DNA. The codons of Alanine (GCN) belong to the family of GCT triplets and those of Glycine (GGN) belong to a point change derivative of the GCT family. It has been proposed that the GCT triplets may have expanded during ancient period of evolution of nucleic acids [28].

It is therefore likely that the observed abundance of Alanine, Glycine, and Valine emerges from the abundance of their respective codons as a consequence of the earliest expansions since Glycine and Alanine co-rank 1^st^(earliest) in the consensus chronological order of amino acids [29]. Persistence of abundance of Glycine in the SSPs of low GC suggests a preference for Glycine in simple sequence regions and likely relates to its conformational flexibility, simple chemical structure and low biosynthetic cost.

The abundance of Leucine presents itself as an interesting case. Majority of the codons (4/6) for Leucine are AT rich and Leucine co-ranks with Glutamic acid at the 5^th ^position in chronological order. Interestingly Glutamic acid displays similar patterns of abundance in SSPs and non-SSPs as does Leucine. Miller's imitation experiment of primordial mixture contained Leucine [30]. Although, this observation points to an old age of Leucine, the preference towards abundance of Leucine over other similarly aged amino acids V, D, P, S, E and T is perhaps due to its wide usage such as its high propensity to be in α-helix, could also be in the core, participates in homo-dimerization and is used in many motifs [31].

The dominance of Asparagine in SSPs of species of low GC mirrors the trend observed in the low complexity sequences of *Plasmodium falciparum*, an AT rich species [32] and points to an early tendency of abundance of Asparagine in simple sequences. Serine is preferred in the SSPs of many species varying in GC content in a broader range compared to the non-SSPs. These features most likely relate to their early history and their characteristic ability to participate in post-translational modifications in regions of compositional bias [5].

### Functional associations of SSPs

In order to examine the preferential association of SSPs towards a specific functional class, the SSPs from all the organisms were classified into seven broad functional classes namely, C: Cell Wall, Cell Membrane and Transporters, D: Cell Division, I: Information (Replication, Transcription, Translation), L: Translocation and secretion, R: Stress, S: Signaling and Communication and M: Metabolism (See Additional file 4). The statistical significance of positive association of SSPs to a functional class in a species was tested against the expected association for the same species computed from its entire proteome. We applied a stringent criterion of *P *<= 0.0001 to avoid potential erroneous conclusions that may arise from small sample sizes.

The number of organisms falling into different functional classes with significant positive association of SSPs is shown in Table 2 (See Additional File 5 for a full list of functional roles of all SSPs from 249 proteomes). It is evident that in a large number of species, SSPs tend to preferentially associate with the functional class of Cell Wall, Cell Membrane and Transporters. In a few species, SSPs associate positively with other functional classes. In the case of metabolism, we observed that SSPs tend to be underrepresented with respect to expected patterns in all species. These observations show that SSPs in general have a preference to be associated or over represented in the class of Cell Wall, Cell Membrane and Transporters. One factor contributing to this trend is the association of simple sequences with membrane spanning segments in transporters and membrane proteins [36].

## Conclusion

The number of Simple Sequence Proteins tends to grow with proteome size and their proportion in each proteome is strongly influenced by genomic base compositional bias. In most species, simple duplications is favoured. In a few species such as Mycobacteria, several SSPs are organized into large sized families with role in host-pathogen interactions. Amino acids with low biosynthetic cost are preferred in SSPs. The abundance of amino acids is controlled by multiple factors including biosynthetic cost, base composition of their respective codons, evolutionary age, wide usage in many biological processes and post-translational modifications. SSPs preferentially associate with Cell Wall, Cell Membrane and Transporters. The proportion of SSPs in a given species does not appear to be governed by its growth temperature (unpublished data) and is in agreement with other observations [33].

SSPs either adopt well structured non-globular shapes or may have a propensity to exhibit disordered conformation [3,34]. The great majority of proteins in any prokaryotic proteome are, however, non-SSPs. This observation suggests that most proteins are likely globular. In this regard, it is interesting to note the preferential association of SSPs with cell surface and concomitant negative association of SSPs with metabolism. Since proteins functioning in metabolic pathways are mostly globular, the negative association of SSPs with metabolism is in agreement with this phenomenon. On the other hand, proteins at the surface have several segments of regular structures such as helices or sheets or disordered regions and with bias in amino acid composition to suit their local environment [35,36].

## Methods

### Identification of SSPs

Complete proteome sequences of 226 bacteria and 23 archaea available in NCBI as on September 2, 2005 were retrieved from the NCBI ftp site [37]. These sequences were processed using ScanCom algorithm [13,19] which classifies protein sequences into either high complexity or low complexity based on a quantitative measure termed F_c_, which is proportional to the fraction of low complexity sequence (simple sequence) present in the protein. Protein sequences with F_c _value ≥ 15 are low complexity proteins and were considered to be SSPs [13,19]. The % (G+C) content and their biological characteristics (pathogenic Vs non-pathogenic) of all the 249 organisms were collected from the NCBI Genome project site [38]. This detailed list is displayed in Additional File 1.

Simple sequences have significant biases in amino acid or nucleotide composition. Collectively, these regions exhibit a very broad range of compositional properties and lengths, and most of them have unknown structures, dynamics and interactions. The sequence simplicity varies from extreme, as in homopolymeric tracts, to very subtle as in some non-globular domains of proteins. Locally abundant residues may be contiguous or loosely clustered, irregularly spaced or periodic. They tend to evolve rapidly, reflecting mutational processes such as replication slippage, unequal crossing-over, and biased nucleotide substitution [3].

**Figure 5:**
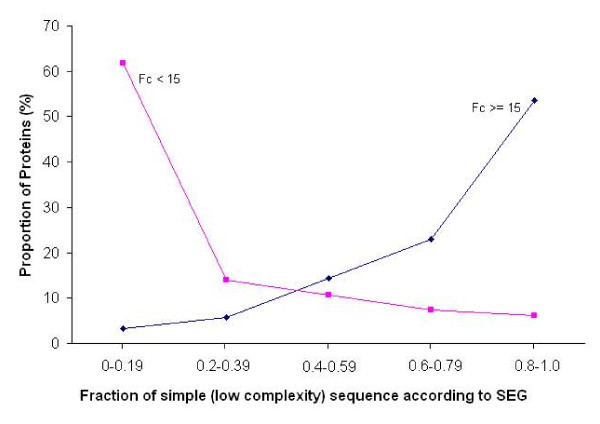
Partition characteristics of two groups of proteins from 249 species based on the content of simple sequences present in them. The fraction of low complexity sequences in proteins was computed using SEG with the default parameters 45, K1 = 3.4, K2 = 3.75. Proteins with F_c _< 15 (pink contour) have lower fraction of low complexity sequences whereas proteins with F_c _≥ 15 (blue contour) have higher fraction of simple sequences. Note that the peaks appear at the extremes. The proportion of proteins in the Y-axis is computed in the respective datasets. For example, the proportion of proteins with a given fraction of low complexity sequence for pink contour is computed with respect to the total number of proteins with F_c _< 15.

**Figure 6:**
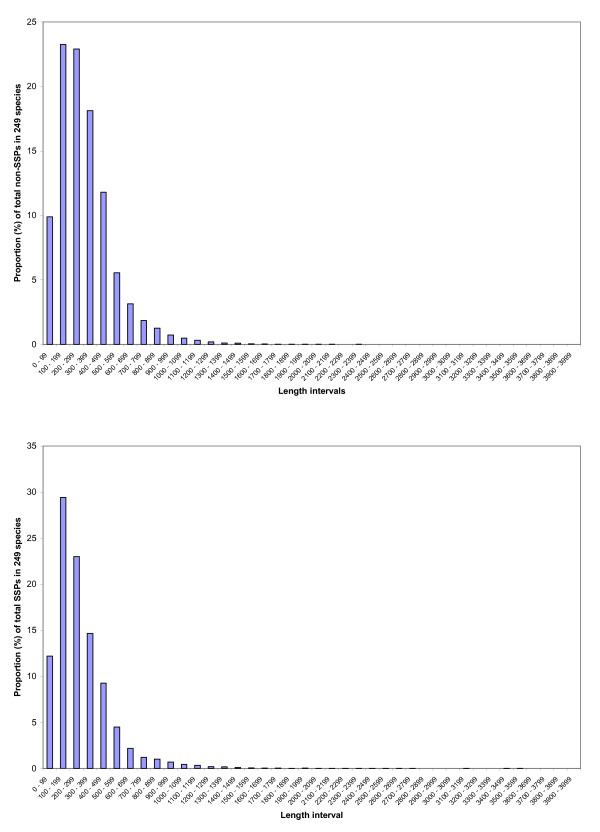
Distribution of proportion of proteins with respect to their lengths in SSPs and non-SSPs from 249 species. Y-axis denotes proportion of the proteins in a given length interval (number of amino acids, X-axis) with respect to the total number of proteins in the respective datasets. Note that both distributions are similar with respect to length bias.

Previously, we had used the structural information available from the non-homologous proteins with high resolution structures in PDB to ascertain the value of F_c _(given by ScanCom algorithm) for identifying a low complexity protein (simple sequence protein). This principle is analogous to that used previously [3]. Proteins with F_c _≥ 15 were observed to be non-globular whereas proteins with lower values of F_c _were globular. The Sensitivity and Specificity of this procedure was 99.4% and 71.4% respectively. Cases of counter-examples were re-examined with the program SEG using default parameters. We found that SEG produced the same inferences and conclusions as ScanCom [13,19] (See also Figures 5 and 6). We were able to identify proteins containing homopolymeric tracts (for example (P)_27_) or charge clusters (for example RDDRPRDDRPRDDRPRDDRPRDDRPRDDRPRD), other types (for example GGAGGAGGKAGLLFGSGGAGGSGGA).

### Identification of paralogs

BLASTCLUST program [39,40] for protein sequence was used with the following parameters: -S (Blast score density) 0.8, -L Minimum length coverage 0.95 (equivalent to 95% coverage of sequence length). Other parameters were used at their default settings: substitution matrix: BLOSUM62, gap opening cost: 11, gap extension cost:1, low complexity filtering: absent, e value threshold 1e^-6^. These parameters were used to meet the clustering of the human hemoglobin proteins, a standard text book example of paralogs, with the following accession numbers: P09105, P69905, P68871, P02042, P02100, P69891, P69892, and Q1W6G9. BLASTCLUST program yields clusters formed from single linkage clustering of pairs of sequences meeting the given parameters. This output can be processed further to distinguish species with large duplications (with large clusters) from small duplications (with small clusters) by computing the number of pairs in each cluster and summing them. The number of pairs in each cluster is given by ^n^C_2 _= {n(n-1)/2} where n is the number of paralogs in a given cluster. Duplex clusters with 2 members will have one pair whereas multiplex clusters will have large number of pairs. Species with paralogs organized predominantly into duplex clusters (simple duplications) will yield low number of pairs, whereas species with paralogs organized into multiplex clusters (large duplications) yield high number of pairs. A plot of the number of total number of paralogous pairs against the total number of SSPs describes the nature of duplications present in the SSPs of a given species.

### Amino acid abundance in SSPs and non-SSPs

The percent amino acid content of all amino acids of SSPs and non-SSPs were computed to examine general preferences in the two datasets. Further, the average percent frequency of amino acids of SSPs or of non-SSPs of each species was computed according to the formula:

fi=(∑j=1Nni(j)/∑j=1Nℓ(j))∗100
 MathType@MTEF@5@5@+=feaafiart1ev1aaatCvAUfKttLearuWrP9MDH5MBPbIqV92AaeXatLxBI9gBaebbnrfifHhDYfgasaacH8akY=wiFfYdH8Gipec8Eeeu0xXdbba9frFj0=OqFfea0dXdd9vqai=hGuQ8kuc9pgc9s8qqaq=dirpe0xb9q8qiLsFr0=vr0=vr0dc8meaabaqaciaacaGaaeqabaqabeGadaaakeaaieWacqWFMbGzdaWgaaWcbaGae8xAaKgabeaakiabg2da9maabmaabaWaaSGbaeaadaaeWaqaaiabd6gaUnaaBaaaleaacqWGPbqAaeqaaOWaaeWaaeaacqWGQbGAaiaawIcacaGLPaaaaSqaaiabdQgaQjabg2da9iabigdaXaqaaiabd6eaobqdcqGHris5aaGcbaWaaabmaeaacqWItecBdaqadaqaaiabdQgaQbGaayjkaiaawMcaaaWcbaGaemOAaOMaeyypa0JaeGymaedabaGaemOta4eaniabggHiLdaaaaGccaGLOaGaayzkaaGaey4fIOIaeGymaeJaeGimaaJaeGimaadaaa@4CF1@

where, *n*_*i*_(*j*) = Number of amino acid of *i*^*th *^type in *j*^*th *^SSP; ℓ(*j*) = length of *j*^th ^protein (SSP or non-SSP); *N *= Total number of proteins (SSP or non-SSP)

The top three ranking amino acids in each of the species were considered for further analysis.

### Functional classifications of SSPs

To investigate the functional association of SSPs, they were first classified into seven basic functional classes C: Cell Wall, Cell Membrane and Transporters, D: Cell Division, I: Information (Replication, Transcription, Translation), L: Translocation and secretion, R: Stress, S: Signaling and Communication and M: Metabolism using an automated open source software program ARC (Automated Resource Classifier for agglomerative functional classification of bacterial proteins using annotation texts, Gnanamani, M., Kumar, N., and Ramachandran, S. Web server in preparation). ARC with its associative keyword library, uses a text word match approach to classify proteins. Since most annotation groups use automated approach in genome centers, the success rates (85%) for classification using our strategy is high. The proteins of *Aeropyrum pernix *K1, *Agrobacterium tumefaciens str*. C58 (Cereon), *Halobacterium sp*. NRC-1, *Listeria innocua *Clip11262, *Listeria monocytogenes *EGD-e, *Mannheimia succiniciproducens *MBEL55E, *Mycobacterium avium subsp. paratuberculosis *K-10, *Mycoplasma gallisepticum *R, *Mycoplasma hyopneumoniae *232, *Mycoplasma hyopneumoniae *7448, *Mycoplasma hyopneumoniae *J, *Nanoarchaeum equitans *Kin4-M, *Onion yellows phytoplasma *OY-M, *Pasteurella multocida subsp. multocida str*. Pm70, *Streptococcus agalactiae *NEM316, *Wigglesworthia glossinidia *endosymbiont of *Glossina brevipalpis *could not be classified by ARC. This is due to either incomplete annotation or rarely used gene symbol annotation. These species were dropped for this analysis. Details are displayed in (See Additional file 4). A full list of functional annotations of all SSPs from 249 species is displayed in Additional file 5.

### Statistical methods

The Correlation coefficient with statistical test was computed to examine the strengths of relationships. Statistically significant positive association (over representation) of SSPs with functional classes for each species were identified by testing the difference between observed proportion and the expected proportion computed from the entire proteome in the same species. Binomial proportions test was used applying a stringent cut off of *P *<= 0.0001 in order to eliminate potential erroneous inferences due to small sample sizes. The interactive statistical calculation page's website [41] was used to perform the statistical tests (Binomial Proportions [42] and Correlation coefficient [43]) using automated scripts.

## Abbreviations

SSPs: Simple Sequence Proteins.

## Authors' contributions

SR conceived the idea, helped in critical assessment and writing the manuscript, MBS implemented and carried out the study, MG assisted in functional classification and manuscript revisions. The contributions of MBS and MG may be considered equal.

## Supplementary Material

Additional File 1 Microbial Organism Information, containing information on the species Taxonomy ID, Organism Name, Super Kingdom, Group Sequence Status, Genome Size, GC Content, Gram Stain, Shape, Arrangment, Endospores, Motility, Salinity, Oxygen Requirement, Habitat, Temperature range, Pathogenic host and Disease caused. Click here for file

Additional File 2 Paralog clusters in species marked in Figure 3 containing information on the paralog proteins and their functions in the various species. Clusters are numbered arbitrarily for convenient post use of data. Click here for file

Additional File 3 additional function predictions of paralogous proteins using other Bioinformatics softwares CDD and SPAAN (see manuscript text for references) detailing the functional characteristics of these proteins in species marked in Figure 3. Click here for file

Additional File 4 Functional class codes, designations and associated keywords used by ARC computer program (see methods section) for functional classification of proteins into the respective functional class. Click here for file

Additional File 5 Adobe Acrobat Document, contains the list of all SSPs analyzed in this work. Click here for file

## Figures and Tables

**Table 1:** Relationships between the top ranking amino acids in SSPs and non-SSPs and the genomic GC content of prokaryotes^1^.

Amino acid	Median GC (SSPs)	Median GC (Non-SSPs)	Variance in GC (SSPs)	Variance in GC (Non-SSPs)	Lowest GC (SSPs)	Lowest GC (Non-SSPs)	Highest GC (SSPs)	Highest GC (Non-SSPs)
L	42.5	42	160.57	158.35	22.5	22.5	72.1	72.1
A	50	51	119.19	112.05	33.5	33.5	72.1	72.1
**G**	55.8	58.5	**98.79**	**62.03**	**33.5**	**45**	72.1	72.1
V	52	51.3	72.82	78.22	38.2	36.7	67.5	67.5
I	31.6	36	33.59	29.49	22.5	22.5	50	50
K	34.45	32	31.92	20.95	22.5	22.5	45	43
E	42	42	41.46	37.58	31.7	35	60	60
**N**	29	-	7.96	-	25.7	-	34.6	-
**R**	66.55	-^2^	4.21	-	65.1	-	68	-
**S**	38	40	34.56	39.69	25.7	27.5	**60**	**50**
**T**	37	-	21.15	-	32	-	46.3	-

^1^: Amino acids displaying differences in abundance patterns between SSPs and non-SSPs are shown in bold face type. Aspartic acid ranked top only in the non-SSPs of *Halobacterium *sp NRC1 (GC 65.9%).

^2^: Arginine ranked top in the non-SSPs of *Xanthomonas oryzae *pv oryzae KACC10331 (GC 63.7%) only.

**Table 2:** Number of species with significant association of SSPs to various functional classes^a^.

CLASS	No. of species with statistically significant association (*P *<= 0.0001)	No. of species with Positive association	No. of species with Negative association
**C**: Cell Wall, Cell Membrane and Transporters	102	102	0
**D**: Cell Division	3	3	0
**I**: Information (Replication, Transcription, Translation)	11	4	7
**L**: Translocation and secretion	7	7	0
**R**: Stress	8	8	0
**S**: Signal and Communication	0	0	0
**M**: Metabolism	80	0	80

^a^: Only those species are listed in which a statistically significant difference was observed between the 'observed proportion' and the 'expected proportion'.
