# Supplementary material for: Simple sequence proteins in prokaryotic proteomes
Source: BMC Genomics. 2006 Jun 8;7:141. doi: 10.1186/1471-2164-7-141 (PMC1524752; doi:10.1186/1471-2164-7-141)
Supplement: Additional File 5 — Adobe Acrobat Document, contains the list of all SSPs analyzed in this work. [file 1471-2164-7-141-S5.pdf]

>gi|72160414|ref|YP_288071.1| putative membrane protein [Thermobifida fusca YX]
>gi|72160419|ref|YP_288076.1| putative integral membrane protein [Thermobifida fusca YX]
>gi|72160420|ref|YP_288077.1| hypothetical protein Tfu_0014 [Thermobifida fusca YX]
>gi|72160429|ref|YP_288086.1| hypothetical protein Tfu_0025 [Thermobifida fusca YX]
>gi|72160430|ref|YP_288087.1| hypothetical protein Tfu_0026 [Thermobifida fusca YX]
>gi|72160435|ref|YP_288092.1| seryl-tRNA synthetase, class IIa [Thermobifida fusca YX]
>gi|72160442|ref|YP_288099.1| hypothetical protein Tfu_0038 [Thermobifida fusca YX]
>gi|72160443|ref|YP_288100.1| hypothetical protein Tfu_0039 [Thermobifida fusca YX]
>gi|72160444|ref|YP_288101.1| hypothetical protein Tfu_0040 [Thermobifida fusca YX]
>gi|72160455|ref|YP_288112.1| ATPase [Thermobifida fusca YX]
>gi|72160456|ref|YP_288113.1| hypothetical protein Tfu_0052 [Thermobifida fusca YX]
>gi|72160460|ref|YP_288117.1| Tyrosine protein kinase:WD-40 repeat:Serine/threonine protein kinase [Thermobifida fusca YX]
>gi|72160461|ref|YP_288118.1| putative regulatory protein [Thermobifida fusca YX]
>gi|72160473|ref|YP_288130.1| putative transcriptional regulator [Thermobifida fusca YX]
>gi|72160475|ref|YP_288132.1| hypothetical protein Tfu_0071 [Thermobifida fusca YX]
>gi|72160477|ref|YP_288134.1| similar to ATPases involved in chromosome partitioning [Thermobifida fusca YX]
>gi|72160482|ref|YP_288139.1| branched-chain amino acid ABC transporter permease protein [Thermobifida fusca YX]
>gi|72160499|ref|YP_288156.1| hypothetical protein Tfu_0095 [Thermobifida fusca YX]
>gi|72160502|ref|YP_288159.1| hypothetical protein Tfu_0098 [Thermobifida fusca YX]
>gi|72160528|ref|YP_288185.1| hypothetical protein Tfu_0124 [Thermobifida fusca YX]
>gi|72160530|ref|YP_288187.1| putative integral membrane protein [Thermobifida fusca YX]
>gi|72160531|ref|YP_288188.1| putative integral membrane protein [Thermobifida fusca YX]
>gi|72160532|ref|YP_288189.1| pilus assembly protein CpaF [Thermobifida fusca YX]
>gi|72160533|ref|YP_288190.1| putative septum site determining protein [Thermobifida fusca YX]
>gi|72160535|ref|YP_288192.1| hypothetical protein Tfu_0131 [Thermobifida fusca YX]
>gi|72160537|ref|YP_288194.1| hypothetical protein Tfu_0133 [Thermobifida fusca YX]
>gi|72160538|ref|YP_288195.1| inorganic H+ pyrophosphatase [Thermobifida fusca YX]
>gi|72160550|ref|YP_288207.1| Surface protein from Gram-positive cocci, anchor region [Thermobifida fusca YX]
>gi|72160551|ref|YP_288208.1| similar to Metal-dependent hydrolase [Thermobifida fusca YX]
>gi|72160553|ref|YP_288210.1| putative integral membrane protein [Thermobifida fusca YX]
>gi|72160559|ref|YP_288216.1| esterase / lipase [Thermobifida fusca YX]
>gi|72160565|ref|YP_288222.1| hypothetical protein Tfu_0161 [Thermobifida fusca YX]
>gi|72160567|ref|YP_288224.1| Tyrosine protein kinase:Serine/threonine protein kinase [Thermobifida fusca YX]
>gi|72160574|ref|YP_288231.1| hypothetical protein Tfu_0170 [Thermobifida fusca YX]
>gi|72160575|ref|YP_288232.1| probable conserved membrane protein [Thermobifida fusca YX]
>gi|72160586|ref|YP_288243.1| putative dihydrolipoamide acyltransferase component [Thermobifida fusca YX]
>gi|72160587|ref|YP_288244.1| exonuclease [Thermobifida fusca YX]
>gi|72160589|ref|YP_288246.1| preprotein translocase YidC subunit [Thermobifida fusca YX]
>gi|72160592|ref|YP_288249.1| 2-dehydro-3-deoxyphosphogluconate aldolase / 4-hydroxy-2-oxoglutarate aldolase [Thermobifida fusca YX]
>gi|72160594|ref|YP_288251.1| similar to Cell wall-associated hydrolases (invasion-associated proteins) [Thermobifida fusca YX]
>gi|72160596|ref|YP_288253.1| hypothetical protein Tfu_0192 [Thermobifida fusca YX]
>gi|72160597|ref|YP_288254.1| similar to Carbonic anhydrases/acetyltransferases isoleucine patch superfamily [Thermobifida fusca YX]
>gi|72160601|ref|YP_288258.1| similar to Molecular chaperone GrpE (heat shock protein) [Thermobifida fusca YX]
>gi|72160602|ref|YP_288259.1| Heat shock protein DnaJ [Thermobifida fusca YX]
>gi|72160611|ref|YP_288268.1| putative transcriptional regulator [Thermobifida fusca YX]
>gi|72160612|ref|YP_288269.1| hypothetical protein Tfu_0208 [Thermobifida fusca YX]
>gi|72160615|ref|YP_288272.1| hypothetical protein Tfu_0211 [Thermobifida fusca YX]
>gi|72160621|ref|YP_288278.1| hypothetical protein Tfu_0217 [Thermobifida fusca YX]
>gi|72160623|ref|YP_288280.1| hypothetical protein Tfu_0219 [Thermobifida fusca YX]
>gi|72160628|ref|YP_288285.1| trehalose-phosphatase:HAD-superfamily hydrolase subfamily IIB [Thermobifida fusca YX]
>gi|72160634|ref|YP_288291.1| hypothetical protein Tfu_0230 [Thermobifida fusca YX]
>gi|72160635|ref|YP_288292.1| hypothetical protein Tfu_0231 [Thermobifida fusca YX]
>gi|72160637|ref|YP_288294.1| hypothetical protein Tfu_0233 [Thermobifida fusca YX]
>gi|72160641|ref|YP_288298.1| hypothetical protein Tfu_0237 [Thermobifida fusca YX]
>gi|72160643|ref|YP_288300.1| hypothetical protein Tfu_0239 [Thermobifida fusca YX]
>gi|72160664|ref|YP_288321.1| hypothetical protein Tfu_0260 [Thermobifida fusca YX]
>gi|72160675|ref|YP_288332.1| Fatty acid synthesis plsX protein [Thermobifida fusca YX]
>gi|72160679|ref|YP_288336.1| putative short chain dehydrogenase [Thermobifida fusca YX]
>gi|72160681|ref|YP_288338.1| Surface protein from Gram-positive cocci, anchor region [Thermobifida fusca YX]
>gi|72160692|ref|YP_288349.1| regulatory protein, LuxR:Response regulator receiver [Thermobifida fusca YX]
>gi|72160693|ref|YP_288350.1| putative two-component system sensor kinase [Thermobifida fusca YX]
>gi|72160694|ref|YP_288351.1| putative integral membrane protein [Thermobifida fusca YX]
>gi|72160702|ref|YP_288359.1| similar to Cu resistance protein CopC [Thermobifida fusca YX]
>gi|72160709|ref|YP_288366.1| amino acid ABC transporter, inner membrane subunit [Thermobifida fusca YX]
>gi|72160712|ref|YP_288369.1| hypothetical protein Tfu_0308 [Thermobifida fusca YX]
>gi|72160715|ref|YP_288372.1| cobyrinic acid a,c-diamide synthase CbiA [Thermobifida fusca YX]
>gi|72160717|ref|YP_288374.1| Mg-chelatase subunit ChlI [Thermobifida fusca YX]
>gi|72160731|ref|YP_288388.1| molybdate ABC transporter, inner membrane subunit [Thermobifida fusca YX]
>gi|72160732|ref|YP_288389.1| molybdenum ABC transporter, periplasmic binding protein [Thermobifida fusca YX]
>gi|72160733|ref|YP_288390.1| signal transduction histidine kinase [Thermobifida fusca YX]

>gi|72160735|ref|YP_288392.1| hypothetical protein Tfu_0331 [Thermobifida fusca YX]
>gi|72160736|ref|YP_288393.1| putative integral membrane protein [Thermobifida fusca YX]
>gi|72160741|ref|YP_288398.1| ABC-type enterobactin transport system inner membrane subunit [Thermobifida fusca YX]
>gi|72160742|ref|YP_288399.1| ABC-type Fe3+-siderophore transport system inner membrane subunit [Thermobifida fusca YX]
>gi|72160743|ref|YP_288400.1| hypothetical protein Tfu_0339 [Thermobifida fusca YX]
>gi|72160746|ref|YP_288403.1| putative membrane protein [Thermobifida fusca YX]
>gi|72160748|ref|YP_288405.1| signal transduction histidine kinase [Thermobifida fusca YX]
>gi|72160750|ref|YP_288407.1| putative cation antiporter subunit [Thermobifida fusca YX]
>gi|72160753|ref|YP_288410.1| similar to Multisubunit Na+/H+ antiporter MnhC subunit [Thermobifida fusca YX]
>gi|72160754|ref|YP_288411.1| NADH dehydrogenase (quinone) [Thermobifida fusca YX]
>gi|72160757|ref|YP_288414.1| trypsin-like serine proteases typically periplasmic contain C-terminal PDZ domain [Thermobifida fusca YX]
>gi|72160759|ref|YP_288416.1| putative integral membrane protein [Thermobifida fusca YX]
>gi|72160760|ref|YP_288417.1| putative integral membrane protein [Thermobifida fusca YX]
>gi|72160765|ref|YP_288422.1| oxidoreductase, short chain dehydrogenase/reductase family [Thermobifida fusca YX]
>gi|72160767|ref|YP_288424.1| hypothetical protein Tfu_0363 [Thermobifida fusca YX]
>gi|72160773|ref|YP_288430.1| hypothetical protein Tfu_0369 [Thermobifida fusca YX]
>gi|72160775|ref|YP_288432.1| hypothetical protein Tfu_0371 [Thermobifida fusca YX]
>gi|72160776|ref|YP_288433.1| hypothetical protein Tfu_0372 [Thermobifida fusca YX]
>gi|72160779|ref|YP_288436.1| molybdopterin cofactor biosynthesis MoaC region [Thermobifida fusca YX]
>gi|72160780|ref|YP_288437.1| molybdopterin binding domain [Thermobifida fusca YX]
>gi|72160782|ref|YP_288439.1| hypothetical protein Tfu_0378 [Thermobifida fusca YX]
>gi|72160786|ref|YP_288443.1| putative integral membrane protein [Thermobifida fusca YX]
>gi|72160789|ref|YP_288446.1| putative integral membrane protein [Thermobifida fusca YX]
>gi|72160794|ref|YP_288451.1| hypothetical protein Tfu_0390 [Thermobifida fusca YX]
>gi|72160810|ref|YP_288467.1| Tyrosine protein kinase:Serine/threonine protein kinase [Thermobifida fusca YX]
>gi|72160811|ref|YP_288468.1| 4-diphosphocytidyl-2C-methyl-D-erythritol kinase [Thermobifida fusca YX]
>gi|72160815|ref|YP_288472.1| regulatory protein, MarR [Thermobifida fusca YX]
>gi|72160825|ref|YP_288482.1| hypothetical protein Tfu_0421 [Thermobifida fusca YX]
>gi|72160833|ref|YP_288490.1| hypothetical protein Tfu_0429 [Thermobifida fusca YX]
>gi|72160839|ref|YP_288496.1| hypothetical protein Tfu_0435 [Thermobifida fusca YX]
>gi|72160841|ref|YP_288498.1| putative secreted protein [Thermobifida fusca YX]
>gi|72160849|ref|YP_288506.1| putative membrane protein [Thermobifida fusca YX]
>gi|72160862|ref|YP_288519.1| secreted protein [Thermobifida fusca YX]
>gi|72160867|ref|YP_288524.1| hypothetical protein Tfu_0463 [Thermobifida fusca YX]
>gi|72160871|ref|YP_288528.1| exonuclease VII, small subunit [Thermobifida fusca YX]
>gi|72160874|ref|YP_288531.1| hypothetical protein Tfu_0470 [Thermobifida fusca YX]
>gi|72160876|ref|YP_288533.1| hypothetical protein Tfu_0472 [Thermobifida fusca YX]
>gi|72160879|ref|YP_288536.1| hypothetical protein Tfu_0475 [Thermobifida fusca YX]
>gi|72160880|ref|YP_288537.1| similar to ATPases involved in chromosome partitioning [Thermobifida fusca YX]
>gi|72160882|ref|YP_288539.1| hypothetical protein Tfu_0478 [Thermobifida fusca YX]
>gi|72160888|ref|YP_288545.1| putative serine protease precursor [Thermobifida fusca YX]
>gi|72160891|ref|YP_288548.1| hypothetical protein Tfu_0487 [Thermobifida fusca YX]
>gi|72160892|ref|YP_288549.1| putative peptidase [Thermobifida fusca YX]
>gi|72160903|ref|YP_288560.1| hypothetical protein Tfu_0499 [Thermobifida fusca YX]
>gi|72160904|ref|YP_288561.1| Tyrosine protein kinase:Serine/threonine protein kinase [Thermobifida fusca YX]
>gi|72160905|ref|YP_288562.1| enoyl-CoA hydratase [Thermobifida fusca YX]
>gi|72160909|ref|YP_288566.1| putative membrane protein [Thermobifida fusca YX]
>gi|72160913|ref|YP_288570.1| hypothetical protein Tfu_0509 [Thermobifida fusca YX]
>gi|72160916|ref|YP_288573.1| similar to Zn-dependent hydrolases including glyoxylases [Thermobifida fusca YX]
>gi|72160921|ref|YP_288578.1| hypothetical protein Tfu_0517 [Thermobifida fusca YX]
>gi|72160923|ref|YP_288580.1| hypothetical protein Tfu_0519 [Thermobifida fusca YX]
>gi|72160925|ref|YP_288582.1| hypothetical protein Tfu_0521 [Thermobifida fusca YX]
>gi|72160929|ref|YP_288586.1| hypothetical protein Tfu_0525 [Thermobifida fusca YX]
>gi|72160942|ref|YP_288599.1| molybdenum cofactor biosynthesis protein E [Thermobifida fusca YX]
>gi|72160956|ref|YP_288613.1| hypothetical protein Tfu_0552 [Thermobifida fusca YX]
>gi|72160962|ref|YP_288619.1| putative integral membrane protein [Thermobifida fusca YX]
>gi|72160974|ref|YP_288631.1| similar to Membrane carboxypeptidase (penicillin-binding protein) [Thermobifida fusca YX]
>gi|72160978|ref|YP_288635.1| similar to DNA-directed RNA polymerase specialized sigma subunit sigma24 -like protein [Thermobifida fusca YX]
>gi|72160982|ref|YP_288639.1| putative reductase [Thermobifida fusca YX]
>gi|72160993|ref|YP_288650.1| ABC-type cobalt transport system [Thermobifida fusca YX]
>gi|72161002|ref|YP_288659.1| similar to Uncharacterized membrane-associated protein [Thermobifida fusca YX]
>gi|72161011|ref|YP_288668.1| Surface protein from Gram-positive cocci, anchor region [Thermobifida fusca YX]
>gi|72161012|ref|YP_288669.1| Surface protein from Gram-positive cocci, anchor region [Thermobifida fusca YX]
>gi|72161022|ref|YP_288679.1| putative membrane protein [Thermobifida fusca YX]
>gi|72161033|ref|YP_288690.1| hypothetical protein Tfu_0629 [Thermobifida fusca YX]
>gi|72161038|ref|YP_288695.1| putative integral membrane protein [Thermobifida fusca YX]
>gi|72161039|ref|YP_288696.1| ABC transporter, ATP-binding protein [Thermobifida fusca YX]
>gi|72161040|ref|YP_288697.1| ABC transporter, ATP-binding protein [Thermobifida fusca YX]
>gi|72161047|ref|YP_288704.1| thiamine-monophosphate kinase [Thermobifida fusca YX]
>gi|72161048|ref|YP_288705.1| cellulose-binding, family II, bacterial type [Thermobifida fusca YX]

>gi|72161051|ref|YP_288708.1| Conserved hypothetical protein 95 [Thermobifida fusca YX]
>gi|72161053|ref|YP_288710.1| hypothetical protein Tfu_0649 [Thermobifida fusca YX]
>gi|72161059|ref|YP_288716.1| Chromosome segregation protein SMC [Thermobifida fusca YX]
>gi|72161061|ref|YP_288718.1| ABC-type transporter, permease component [Thermobifida fusca YX]
>gi|72161062|ref|YP_288719.1| cell division transporter substrate-binding protein FtsY [Thermobifida fusca YX]
>gi|72161077|ref|YP_288734.1| hypothetical protein Tfu_0673 [Thermobifida fusca YX]
>gi|72161079|ref|YP_288736.1| hypothetical protein Tfu_0675 [Thermobifida fusca YX]
>gi|72161084|ref|YP_288741.1| phosphatidate cytidylyltransferase [Thermobifida fusca YX]
>gi|72161104|ref|YP_288761.1| hypothetical protein Tfu_0700 [Thermobifida fusca YX]
>gi|72161108|ref|YP_288765.1| hypothetical protein Tfu_0704 [Thermobifida fusca YX]
>gi|72161127|ref|YP_288784.1| hypothetical protein Tfu_0723 [Thermobifida fusca YX]
>gi|72161130|ref|YP_288787.1| hypothetical protein Tfu_0726 [Thermobifida fusca YX]
>gi|72161147|ref|YP_288804.1| hypothetical protein Tfu_0743 [Thermobifida fusca YX]
>gi|72161149|ref|YP_288806.1| hypothetical protein Tfu_0745 [Thermobifida fusca YX]
>gi|72161169|ref|YP_288826.1| putative cytochrome D ubiquinol oxidase subunit II [Thermobifida fusca YX]
>gi|72161171|ref|YP_288828.1| regulatory protein, LuxR:Response regulator receiver [Thermobifida fusca YX]
>gi|72161173|ref|YP_288830.1| membrane protein, putative [Thermobifida fusca YX]
>gi|72161174|ref|YP_288831.1| hypothetical protein Tfu_0770 [Thermobifida fusca YX]
>gi|72161177|ref|YP_288834.1| hypothetical protein Tfu_0773 [Thermobifida fusca YX]
>gi|72161182|ref|YP_288839.1| initiation factor 2:Small GTP-binding protein domain [Thermobifida fusca YX]
>gi|72161187|ref|YP_288844.1| Ribosomal protein S15, bacterial chloroplast and mitochondrial type [Thermobifida fusca YX]
>gi|72161192|ref|YP_288849.1| 1,4-dihydroxy-2-naphthoate octaprenyltransferase [Thermobifida fusca YX]
>gi|72161198|ref|YP_288855.1| helix-turn-helix motif [Thermobifida fusca YX]
>gi|72161201|ref|YP_288858.1| CinA, C-terminal [Thermobifida fusca YX]
>gi|72161205|ref|YP_288862.1| probable acetyltransferase [Thermobifida fusca YX]
>gi|72161208|ref|YP_288865.1| putative RecX protein [Thermobifida fusca YX]
>gi|72161209|ref|YP_288866.1| metal-dependent phosphohydrolase, HD region [Thermobifida fusca YX]
>gi|72161221|ref|YP_288878.1| ABC peptide/nickel transport system permease protein [Thermobifida fusca YX]
>gi|72161228|ref|YP_288885.1| hypothetical protein Tfu_0824 [Thermobifida fusca YX]
>gi|72161229|ref|YP_288886.1| ComEC/Rec2-related protein [Thermobifida fusca YX]
>gi|72161230|ref|YP_288887.1| helix-hairpin-helix DNA-binding, class 1 [Thermobifida fusca YX]
>gi|72161240|ref|YP_288897.1| hypothetical protein Tfu_0836 [Thermobifida fusca YX]
>gi|72161241|ref|YP_288898.1| hypothetical protein Tfu_0837 [Thermobifida fusca YX]
>gi|72161244|ref|YP_288901.1| Conserved hypothetical protein 46 [Thermobifida fusca YX]
>gi|72161245|ref|YP_288902.1| hypothetical protein Tfu_0841 [Thermobifida fusca YX]
>gi|72161251|ref|YP_288908.1| hypothetical protein Tfu_0847 [Thermobifida fusca YX]
>gi|72161255|ref|YP_288912.1| putative membrane protein [Thermobifida fusca YX]
>gi|72161261|ref|YP_288918.1| ABC zinc/manganese transport system permease protein [Thermobifida fusca YX]
>gi|72161263|ref|YP_288920.1| hypothetical protein Tfu_0859 [Thermobifida fusca YX]
>gi|72161277|ref|YP_288934.1| sulfate permease, SulP family [Thermobifida fusca YX]
>gi|72161288|ref|YP_288945.1| putative integral membrane protein [Thermobifida fusca YX]
>gi|72161296|ref|YP_288953.1| hypothetical protein Tfu_0892 [Thermobifida fusca YX]
>gi|72161300|ref|YP_288957.1| hypothetical protein Tfu_0896 [Thermobifida fusca YX]
>gi|72161301|ref|YP_288958.1| similar to ADP-heptose:LPS heptosyltransferase [Thermobifida fusca YX]
>gi|72161306|ref|YP_288963.1| DNA polymerase III, epsilon subunit [Thermobifida fusca YX]
>gi|72161310|ref|YP_288967.1| hypothetical protein Tfu_0906 [Thermobifida fusca YX]
>gi|72161321|ref|YP_288978.1| hypothetical protein Tfu_0917 [Thermobifida fusca YX]
>gi|72161322|ref|YP_288979.1| hypothetical protein Tfu_0918 [Thermobifida fusca YX]
>gi|72161327|ref|YP_288984.1| hypothetical protein Tfu_0923 [Thermobifida fusca YX]
>gi|72161333|ref|YP_288990.1| hypothetical protein Tfu_0929 [Thermobifida fusca YX]
>gi|72161334|ref|YP_288991.1| hypothetical protein Tfu_0930 [Thermobifida fusca YX]
>gi|72161336|ref|YP_288993.1| putative ABC transporter permease protein [Thermobifida fusca YX]
>gi|72161343|ref|YP_289000.1| hypothetical protein Tfu_0939 [Thermobifida fusca YX]
>gi|72161351|ref|YP_289008.1| putative acetyl/propionyl CoA carboxylase alpha subunit: biotin carboxylase [Thermobifida fusca YX]
>gi|72161357|ref|YP_289014.1| hypothetical protein Tfu_0953 [Thermobifida fusca YX]
>gi|72161359|ref|YP_289016.1| hypothetical protein Tfu_0955 [Thermobifida fusca YX]
>gi|72161366|ref|YP_289023.1| allantoinase [Thermobifida fusca YX]
>gi|72161368|ref|YP_289025.1| hypothetical protein Tfu_0964 [Thermobifida fusca YX]
>gi|72161380|ref|YP_289037.1| hypothetical protein Tfu_0976 [Thermobifida fusca YX]
>gi|72161390|ref|YP_289047.1| glutamate-ammonia-ligase adenylyltransferase [Thermobifida fusca YX]
>gi|72161393|ref|YP_289050.1| hypothetical protein Tfu_0989 [Thermobifida fusca YX]
>gi|72161397|ref|YP_289054.1| dihydrolipoamide S-succinyltransferase [Thermobifida fusca YX]
>gi|72161399|ref|YP_289056.1| leucyl aminopeptidase [Thermobifida fusca YX]
>gi|72161400|ref|YP_289057.1| putative cobalamin (5'-phosphate) synthase : adenosylcobinamide-GDP ribazoletransferase [Thermobifida fusca YX]
>gi|72161403|ref|YP_289060.1| hypothetical protein Tfu_0999 [Thermobifida fusca YX]
>gi|72161405|ref|YP_289062.1| hypothetical protein Tfu_1001 [Thermobifida fusca YX]
>gi|72161406|ref|YP_289063.1| hedgehog/intein hint, N-terminal [Thermobifida fusca YX]
>gi|72161412|ref|YP_289069.1| protease HtpX -like protein (heat shock protein) [Thermobifida fusca YX]
>gi|72161414|ref|YP_289071.1| glycerate kinase [Thermobifida fusca YX]
>gi|72161431|ref|YP_289088.1| DNA polymerase III, epsilon subunit [Thermobifida fusca YX]

>gi|72161432|ref|YP_289089.1| hypothetical protein Tfu_1028 [Thermobifida fusca YX]
>gi|72161433|ref|YP_289090.1| putative secreted protein [Thermobifida fusca YX]
>gi|72161434|ref|YP_289091.1| putative NLP/P60 family secreted protein [Thermobifida fusca YX]
>gi|72161438|ref|YP_289095.1| hypothetical protein Tfu_1034 [Thermobifida fusca YX]
>gi|72161439|ref|YP_289096.1| hypothetical protein Tfu_1035 [Thermobifida fusca YX]
>gi|72161447|ref|YP_289104.1| ThiS, thiamine-biosynthesis [Thermobifida fusca YX]
>gi|72161464|ref|YP_289121.1| orotidine 5'-phosphate decarboxylase [Thermobifida fusca YX]
>gi|72161468|ref|YP_289125.1| DNA/pantothenate metabolism flavoprotein [Thermobifida fusca YX]
>gi|72161470|ref|YP_289127.1| hypothetical protein Tfu_1066 [Thermobifida fusca YX]
>gi|72161477|ref|YP_289134.1| primosomal protein N' (replication factor Y) (superfamily II helicase) [Thermobifida fusca YX]
>gi|72161478|ref|YP_289135.1| cellulose-binding, family II, bacterial type [Thermobifida fusca YX]
>gi|72161485|ref|YP_289142.1| 6,7-dimethyl-8-ribityllumazine synthase [Thermobifida fusca YX]
>gi|72161488|ref|YP_289145.1| serine/threonine protein kinase [Thermobifida fusca YX]
>gi|72161493|ref|YP_289150.1| hypothetical protein Tfu_1089 [Thermobifida fusca YX]
>gi|72161502|ref|YP_289159.1| transglutaminase-like [Thermobifida fusca YX]
>gi|72161513|ref|YP_289170.1| cell division protein FtsW [Thermobifida fusca YX]
>gi|72161516|ref|YP_289173.1| cell division protein FtsQ [Thermobifida fusca YX]
>gi|72161517|ref|YP_289174.1| Cell division protein FtsZ [Thermobifida fusca YX]
>gi|72161518|ref|YP_289175.1| Protein of unknown function DUF152 [Thermobifida fusca YX]
>gi|72161520|ref|YP_289177.1| hypothetical protein Tfu_1116 [Thermobifida fusca YX]
>gi|72161522|ref|YP_289179.1| hypothetical protein Tfu_1118 [Thermobifida fusca YX]
>gi|72161523|ref|YP_289180.1| hypothetical protein Tfu_1119 [Thermobifida fusca YX]
>gi|72161529|ref|YP_289186.1| hypothetical protein Tfu_1125 [Thermobifida fusca YX]
>gi|72161530|ref|YP_289187.1| hypothetical protein Tfu_1126 [Thermobifida fusca YX]
>gi|72161531|ref|YP_289188.1| hypothetical protein Tfu_1127 [Thermobifida fusca YX]
>gi|72161533|ref|YP_289190.1| hypothetical protein Tfu_1129 [Thermobifida fusca YX]
>gi|72161539|ref|YP_289196.1| hypothetical protein Tfu_1135 [Thermobifida fusca YX]
>gi|72161541|ref|YP_289198.1| putative membrane protein [Thermobifida fusca YX]
>gi|72161542|ref|YP_289199.1| putative membrane protein [Thermobifida fusca YX]
>gi|72161550|ref|YP_289207.1| hypothetical protein Tfu_1146 [Thermobifida fusca YX]
>gi|72161551|ref|YP_289208.1| putative transcriptional regulator [Thermobifida fusca YX]
>gi|72161552|ref|YP_289209.1| hypothetical protein Tfu_1148 [Thermobifida fusca YX]
>gi|72161557|ref|YP_289214.1| hypothetical protein Tfu_1153 [Thermobifida fusca YX]
>gi|72161561|ref|YP_289218.1| ABC-type multidrug transport system ATPase and permease components [Thermobifida fusca YX]
>gi|72161564|ref|YP_289221.1| putative membrane protein [Thermobifida fusca YX]
>gi|72161565|ref|YP_289222.1| hypothetical protein Tfu_1161 [Thermobifida fusca YX]
>gi|72161566|ref|YP_289223.1| hypothetical protein Tfu_1162 [Thermobifida fusca YX]
>gi|72161571|ref|YP_289228.1| hypothetical protein Tfu_1167 [Thermobifida fusca YX]
>gi|72161574|ref|YP_289231.1| hypothetical protein Tfu_1170 [Thermobifida fusca YX]
>gi|72161587|ref|YP_289244.1| branched-chain amino acid transport system permease protein [Thermobifida fusca YX]
>gi|72161591|ref|YP_289248.1| hypothetical protein Tfu_1187 [Thermobifida fusca YX]
>gi|72161594|ref|YP_289251.1| hypothetical protein Tfu_1190 [Thermobifida fusca YX]
>gi|72161597|ref|YP_289254.1| similar to GTPase [Thermobifida fusca YX]
>gi|72161598|ref|YP_289255.1| hypothetical protein Tfu_1194 [Thermobifida fusca YX]
>gi|72161599|ref|YP_289256.1| putative regulator [Thermobifida fusca YX]
>gi|72161601|ref|YP_289258.1| hypothetical protein Tfu_1197 [Thermobifida fusca YX]
>gi|72161608|ref|YP_289265.1| hypothetical protein Tfu_1204 [Thermobifida fusca YX]
>gi|72161610|ref|YP_289267.1| pseudouridine synthase, Rsu [Thermobifida fusca YX]
>gi|72161612|ref|YP_289269.1| prephenate dehydrogenase [Thermobifida fusca YX]
>gi|72161624|ref|YP_289281.1| actinorhodin polyketide synthase acyl carrier protein / 3-oxoacyl-ACP synthase acyl carrier protein [Thermobifida fusca YX]
>gi|72161637|ref|YP_289294.1| putative transmembrane efflux protein [Thermobifida fusca YX]
>gi|72161663|ref|YP_289320.1| enoyl-CoA hydratase/isomerase-like protein [Thermobifida fusca YX]
>gi|72161667|ref|YP_289324.1| CBS [Thermobifida fusca YX]
>gi|72161668|ref|YP_289325.1| hypothetical protein Tfu_1264 [Thermobifida fusca YX]
>gi|72161673|ref|YP_289330.1| hypothetical protein Tfu_1269 [Thermobifida fusca YX]
>gi|72161695|ref|YP_289352.1| probable amino-acid transmembrane lipoprotein ABC transporter [Thermobifida fusca YX]
>gi|72161699|ref|YP_289356.1| putative membrane transport protein [Thermobifida fusca YX]
>gi|72161714|ref|YP_289371.1| CBS [Thermobifida fusca YX]
>gi|72161717|ref|YP_289374.1| putative citrate lyase beta subunit [Thermobifida fusca YX]
>gi|72161720|ref|YP_289377.1| 5-amino-6-(5-phosphoribosylamino)uracil reductase [Thermobifida fusca YX]
>gi|72161729|ref|YP_289386.1| hypothetical protein Tfu_1325 [Thermobifida fusca YX]
>gi|72161730|ref|YP_289387.1| hypothetical protein Tfu_1326 [Thermobifida fusca YX]
>gi|72161732|ref|YP_289389.1| hypothetical protein Tfu_1328 [Thermobifida fusca YX]
>gi|72161735|ref|YP_289392.1| hypothetical protein Tfu_1331 [Thermobifida fusca YX]
>gi|72161746|ref|YP_289403.1| Tyrosine protein kinase:Serine/threonine protein kinase [Thermobifida fusca YX]
>gi|72161747|ref|YP_289404.1| putative transport protein [Thermobifida fusca YX]
>gi|72161750|ref|YP_289407.1| hypothetical protein Tfu_1346 [Thermobifida fusca YX]
>gi|72161758|ref|YP_289415.1| transmembrane transport protein [Thermobifida fusca YX]
>gi|72161760|ref|YP_289417.1| hypothetical protein Tfu_1356 [Thermobifida fusca YX]

>gi|72161765|ref|YP_289422.1| hypothetical protein Tfu_1361 [Thermobifida fusca YX]
>gi|72161774|ref|YP_289431.1| membrane protein, putative [Thermobifida fusca YX]
>gi|72161779|ref|YP_289436.1| putative integral membrane transporter [Thermobifida fusca YX]
>gi|72161793|ref|YP_289450.1| carbon starvation-induced protein [Thermobifida fusca YX]
>gi|72161795|ref|YP_289452.1| arsenite-transporting ATPase [Thermobifida fusca YX]
>gi|72161797|ref|YP_289454.1| putative CDP-diacylglycerol--glycerol-3-phosphate 3-phosphatidyl-transferase [Thermobifida fusca YX]
>gi|72161803|ref|YP_289460.1| hypothetical protein Tfu_1399 [Thermobifida fusca YX]
>gi|72161805|ref|YP_289462.1| hypothetical protein Tfu_1401 [Thermobifida fusca YX]
>gi|72161806|ref|YP_289463.1| putative two-component system sensor kinase [Thermobifida fusca YX]
>gi|72161814|ref|YP_289471.1| o-succinylbenzoate-CoA synthase [Thermobifida fusca YX]
>gi|72161815|ref|YP_289472.1| Menaquinone biosynthesis protein [Thermobifida fusca YX]
>gi|72161816|ref|YP_289473.1| hypothetical protein Tfu_1412 [Thermobifida fusca YX]
>gi|72161820|ref|YP_289477.1| putative secreted penicillin binding protein [Thermobifida fusca YX]
>gi|72161823|ref|YP_289480.1| hypothetical protein Tfu_1419 [Thermobifida fusca YX]
>gi|72161829|ref|YP_289486.1| putative integral membrane protein [Thermobifida fusca YX]
>gi|72161831|ref|YP_289488.1| putative DNA repair protein [Thermobifida fusca YX]
>gi|72161836|ref|YP_289493.1| hypothetical protein Tfu_1432 [Thermobifida fusca YX]
>gi|72161838|ref|YP_289495.1| possible solute binding protein of ABC transporter system [Thermobifida fusca YX]
>gi|72161839|ref|YP_289496.1| putative integral membrane protein [Thermobifida fusca YX]
>gi|72161846|ref|YP_289503.1| hypothetical protein Tfu_1442 [Thermobifida fusca YX]
>gi|72161847|ref|YP_289504.1| hypothetical protein Tfu_1443 [Thermobifida fusca YX]
>gi|72161848|ref|YP_289505.1| hypothetical protein Tfu_1444 [Thermobifida fusca YX]
>gi|72161859|ref|YP_289516.1| hypothetical protein Tfu_1455 [Thermobifida fusca YX]
>gi|72161868|ref|YP_289525.1| short-chain alcohol dehydrogenase [Thermobifida fusca YX]
>gi|72161871|ref|YP_289528.1| hypothetical protein Tfu_1469 [Thermobifida fusca YX]
>gi|72161876|ref|YP_289533.1| short chain dehydrogenase [Thermobifida fusca YX]
>gi|72161894|ref|YP_289551.1| putative ferrichrome transport system permease protein [Thermobifida fusca YX]
>gi|72161895|ref|YP_289552.1| putative ferrichrome transport system permease protein [Thermobifida fusca YX]
>gi|72161897|ref|YP_289554.1| hypothetical protein Tfu_1495 [Thermobifida fusca YX]
>gi|72161901|ref|YP_289558.1| hypothetical protein Tfu_1499 [Thermobifida fusca YX]
>gi|72161911|ref|YP_289568.1| hypothetical protein Tfu_1509 [Thermobifida fusca YX]
>gi|72161912|ref|YP_289569.1| phosphoglycolate phosphatase [Thermobifida fusca YX]
>gi|72161925|ref|YP_289582.1| similar to Protein-disulfide isomerase [Thermobifida fusca YX]
>gi|72161926|ref|YP_289583.1| hypothetical protein Tfu_1524 [Thermobifida fusca YX]
>gi|72161927|ref|YP_289584.1| hypothetical protein Tfu_1525 [Thermobifida fusca YX]
>gi|72161929|ref|YP_289586.1| rare lipoprotein A [Thermobifida fusca YX]
>gi|72161933|ref|YP_289590.1| hypothetical protein Tfu_1531 [Thermobifida fusca YX]
>gi|72161934|ref|YP_289591.1| hypothetical protein Tfu_1532 [Thermobifida fusca YX]
>gi|72161936|ref|YP_289593.1| hypothetical protein Tfu_1534 [Thermobifida fusca YX]
>gi|72161938|ref|YP_289595.1| hypothetical protein Tfu_1536 [Thermobifida fusca YX]
>gi|72161947|ref|YP_289604.1| hypothetical protein Tfu_1545 [Thermobifida fusca YX]
>gi|72161952|ref|YP_289609.1| hypothetical protein Tfu_1550 [Thermobifida fusca YX]
>gi|72161969|ref|YP_289626.1| leucine-rich repeat family protein / extensin family protein [Thermobifida fusca YX]
>gi|72161971|ref|YP_289628.1| arsenite efflux pump [Thermobifida fusca YX]
>gi|72161972|ref|YP_289629.1| hypothetical protein Tfu_1570 [Thermobifida fusca YX]
>gi|72161977|ref|YP_289634.1| putative sugar kinase protein [Thermobifida fusca YX]
>gi|72161978|ref|YP_289635.1| Conserved hypothetical protein 374 [Thermobifida fusca YX]
>gi|72161987|ref|YP_289644.1| hypothetical protein Tfu_1585 [Thermobifida fusca YX]
>gi|72162003|ref|YP_289660.1| hypothetical protein Tfu_1602 [Thermobifida fusca YX]
>gi|72162026|ref|YP_289683.1| hypothetical protein Tfu_1625 [Thermobifida fusca YX]
>gi|72162027|ref|YP_289684.1| regulatory protein GntR, HTH [Thermobifida fusca YX]
>gi|72162053|ref|YP_289710.1| hypothetical protein Tfu_1652 [Thermobifida fusca YX]
>gi|72162059|ref|YP_289716.1| Tyrosine protein kinase:Serine/threonine protein kinase:Sel1-like repeat [Thermobifida fusca YX]
>gi|72162068|ref|YP_289725.1| regulatory protein, MerR [Thermobifida fusca YX]
>gi|72162076|ref|YP_289733.1| hypothetical protein Tfu_1675 [Thermobifida fusca YX]
>gi|72162078|ref|YP_289735.1| hypothetical protein Tfu_1677 [Thermobifida fusca YX]
>gi|72162082|ref|YP_289739.1| probable conserved integral membrane protein [Thermobifida fusca YX]
>gi|72162083|ref|YP_289740.1| putative short chain dehydrogenase [Thermobifida fusca YX]
>gi|72162090|ref|YP_289747.1| hypothetical protein Tfu_1689 [Thermobifida fusca YX]
>gi|72162097|ref|YP_289754.1| hypothetical protein Tfu_1696 [Thermobifida fusca YX]
>gi|72162098|ref|YP_289755.1| ABC-type multidrug transport system ATPase and permease components [Thermobifida fusca YX]
>gi|72162101|ref|YP_289758.1| probable 3-oxoacyl-(acyl carrier protein) reductase [Thermobifida fusca YX]
>gi|72162114|ref|YP_289771.1| hypothetical protein Tfu_1713 [Thermobifida fusca YX]
>gi|72162116|ref|YP_289773.1| dihydrodipicolinate synthase [Thermobifida fusca YX]
>gi|72162125|ref|YP_289782.1| hypothetical protein Tfu_1724 [Thermobifida fusca YX]
>gi|72162128|ref|YP_289785.1| HAD-superfamily hydrolase subfamily IIB [Thermobifida fusca YX]
>gi|72162129|ref|YP_289786.1| drug resistance transporter Bcr/CflA subfamily [Thermobifida fusca YX]
>gi|72162135|ref|YP_289792.1| drug resistance transporter Bcr/CflA subfamily [Thermobifida fusca YX]
>gi|72162139|ref|YP_289796.1| hypothetical protein Tfu_1738 [Thermobifida fusca YX]
>gi|72162142|ref|YP_289799.1| threonine ammonia-lyase [Thermobifida fusca YX]

>gi|72162157|ref|YP_289814.1| Tyrosine protein kinase:Serine/threonine protein kinase [Thermobifida fusca YX]
>gi|72162160|ref|YP_289817.1| hypothetical protein Tfu_1759 [Thermobifida fusca YX]
>gi|72162162|ref|YP_289819.1| similar to Protein affecting phage T7 exclusion by the F plasmid [Thermobifida fusca YX]
>gi|72162163|ref|YP_289820.1| Apolipoprotein N-acyltransferase [Thermobifida fusca YX]
>gi|72162165|ref|YP_289822.1| putative glycosyl transferase [Thermobifida fusca YX]
>gi|72162171|ref|YP_289828.1| hypothetical protein Tfu_1770 [Thermobifida fusca YX]
>gi|72162172|ref|YP_289829.1| similar to transcriptional regulator [Thermobifida fusca YX]
>gi|72162173|ref|YP_289830.1| hypothetical protein Tfu_1772 [Thermobifida fusca YX]
>gi|72162181|ref|YP_289838.1| ABC transporter permease protein [Thermobifida fusca YX]
>gi|72162186|ref|YP_289843.1| regulatory protein GntR, HTH [Thermobifida fusca YX]
>gi|72162188|ref|YP_289845.1| regulatory protein GntR, HTH [Thermobifida fusca YX]
>gi|72162196|ref|YP_289853.1| helix-turn-helix, Fis-type [Thermobifida fusca YX]
>gi|72162216|ref|YP_289873.1| regulatory protein, LuxR:Response regulator receiver [Thermobifida fusca YX]
>gi|72162217|ref|YP_289874.1| putative membrane protein [Thermobifida fusca YX]
>gi|72162222|ref|YP_289879.1| putative membrane protein [Thermobifida fusca YX]
>gi|72162226|ref|YP_289883.1| hypothetical protein Tfu_1827 [Thermobifida fusca YX]
>gi|72162227|ref|YP_289884.1| hypothetical protein Tfu_1828 [Thermobifida fusca YX]
>gi|72162228|ref|YP_289885.1| hypothetical protein Tfu_1829 [Thermobifida fusca YX]
>gi|72162233|ref|YP_289890.1| ATPase, E1-E2 type [Thermobifida fusca YX]
>gi|72162239|ref|YP_289896.1| hypothetical protein Tfu_1840 [Thermobifida fusca YX]
>gi|72162248|ref|YP_289905.1| hypothetical protein Tfu_1849 [Thermobifida fusca YX]
>gi|72162250|ref|YP_289907.1| regulatory protein, DeoR [Thermobifida fusca YX]
>gi|72162252|ref|YP_289909.1| putative transporter [Thermobifida fusca YX]
>gi|72162260|ref|YP_289917.1| probable POT family transport protein [Thermobifida fusca YX]
>gi|72162265|ref|YP_289922.1| amino acid adenylation [Thermobifida fusca YX]
>gi|72162272|ref|YP_289929.1| 2,3-dihydro-2,3-dihydroxybenzoate dehydrogenase; RBL00455 [Thermobifida fusca YX]
>gi|72162273|ref|YP_289930.1| putative membrane protein [Thermobifida fusca YX]
>gi|72162274|ref|YP_289931.1| hypothetical protein Tfu_1875 [Thermobifida fusca YX]
>gi|72162277|ref|YP_289934.1| putative enoyl-CoA hydratase/isomerase [Thermobifida fusca YX]
>gi|72162284|ref|YP_289941.1| putative integral membrane protein [Thermobifida fusca YX]
>gi|72162286|ref|YP_289943.1| similar to membrane protein [Thermobifida fusca YX]
>gi|72162289|ref|YP_289946.1| putative secreted protein [Thermobifida fusca YX]
>gi|72162291|ref|YP_289948.1| hypothetical protein Tfu_1892 [Thermobifida fusca YX]
>gi|72162294|ref|YP_289951.1| putative phosphatidylserine synthase [Thermobifida fusca YX]
>gi|72162297|ref|YP_289954.1| putative lipoprotein [Thermobifida fusca YX]
>gi|72162301|ref|YP_289958.1| hypothetical protein Tfu_1902 [Thermobifida fusca YX]
>gi|72162304|ref|YP_289961.1| putative regulatory protein [Thermobifida fusca YX]
>gi|72162311|ref|YP_289968.1| putative membrane protein, MmpL family [Thermobifida fusca YX]
>gi|72162318|ref|YP_289975.1| hypothetical protein Tfu_1919 [Thermobifida fusca YX]
>gi|72162331|ref|YP_289988.1| hypothetical protein Tfu_1932 [Thermobifida fusca YX]
>gi|72162345|ref|YP_290002.1| FAD-linked oxidoreductase [Thermobifida fusca YX]
>gi|72162346|ref|YP_290003.1| hypothetical protein Tfu_1947 [Thermobifida fusca YX]
>gi|72162347|ref|YP_290004.1| putative membrane protein [Thermobifida fusca YX]
>gi|72162350|ref|YP_290007.1| hypothetical protein Tfu_1951 [Thermobifida fusca YX]
>gi|72162353|ref|YP_290010.1| similar to Zn-ribbon protein possibly nucleic acid-binding [Thermobifida fusca YX]
>gi|72162364|ref|YP_290021.1| hypothetical protein Tfu_1965 [Thermobifida fusca YX]
>gi|72162372|ref|YP_290029.1| (Acyl-carrier protein) S-malonyltransferase [Thermobifida fusca YX]
>gi|72162389|ref|YP_290046.1| putative integral membrane transport protein [Thermobifida fusca YX]
>gi|72162390|ref|YP_290047.1| hypothetical protein Tfu_1991 [Thermobifida fusca YX]
>gi|72162393|ref|YP_290050.1| putative integral membrane protein [Thermobifida fusca YX]
>gi|72162394|ref|YP_290051.1| putative integral membrane transport protein [Thermobifida fusca YX]
>gi|72162413|ref|YP_290070.1| preprotein translocase SecG subunit [Thermobifida fusca YX]
>gi|72162425|ref|YP_290082.1| putative glycosyl transferase [Thermobifida fusca YX]
>gi|72162426|ref|YP_290083.1| similar to Uncharacterized membrane protein putative virulence factor [Thermobifida fusca YX]
>gi|72162427|ref|YP_290084.1| hypothetical protein Tfu_2028 [Thermobifida fusca YX]
>gi|72162431|ref|YP_290088.1| DNA repair protein RecN [Thermobifida fusca YX]
>gi|72162435|ref|YP_290092.1| hypothetical protein Tfu_2036 [Thermobifida fusca YX]
>gi|72162440|ref|YP_290097.1| hypothetical protein Tfu_2041 [Thermobifida fusca YX]
>gi|72162466|ref|YP_290123.1| shikimate 5-dehydrogenase [Thermobifida fusca YX]
>gi|72162467|ref|YP_290124.1| putative integral membrane protein [Thermobifida fusca YX]
>gi|72162468|ref|YP_290125.1| Holliday junction resolvase YqgF [Thermobifida fusca YX]
>gi|72162472|ref|YP_290129.1| putative secreted protein [Thermobifida fusca YX]
>gi|72162473|ref|YP_290130.1| Tyrosine protein kinase:Serine/threonine protein kinase [Thermobifida fusca YX]
>gi|72162475|ref|YP_290132.1| hypothetical protein Tfu_2076 [Thermobifida fusca YX]
>gi|72162476|ref|YP_290133.1| hypothetical protein Tfu_2077 [Thermobifida fusca YX]
>gi|72162479|ref|YP_290136.1| hypothetical protein Tfu_2080 [Thermobifida fusca YX]
>gi|72162481|ref|YP_290138.1| phosphoesterase, PA-phosphatase related [Thermobifida fusca YX]
>gi|72162487|ref|YP_290144.1| hypothetical protein Tfu_2088 [Thermobifida fusca YX]
>gi|72162490|ref|YP_290147.1| adenine phosphoribosyl transferase [Thermobifida fusca YX]
>gi|72162497|ref|YP_290154.1| hypothetical protein Tfu_2098 [Thermobifida fusca YX]

>gi|72162499|ref|YP_290156.1| hypothetical protein Tfu_2100 [Thermobifida fusca YX]
>gi|72162503|ref|YP_290160.1| hypothetical protein Tfu_2104 [Thermobifida fusca YX]
>gi|72162509|ref|YP_290166.1| hypothetical protein Tfu_2110 [Thermobifida fusca YX]
>gi|72162518|ref|YP_290175.1| hypothetical protein Tfu_2119 [Thermobifida fusca YX]
>gi|72162522|ref|YP_290179.1| hypothetical protein Tfu_2123 [Thermobifida fusca YX]
>gi|72162536|ref|YP_290193.1| hypothetical protein Tfu_2137 [Thermobifida fusca YX]
>gi|72162538|ref|YP_290195.1| carbamoyl-phosphate synthase L chain, ATP-binding [Thermobifida fusca YX]
>gi|72162540|ref|YP_290197.1| hypothetical protein Tfu_2141 [Thermobifida fusca YX]
>gi|72162552|ref|YP_290209.1| hypothetical protein Tfu_2153 [Thermobifida fusca YX]
>gi|72162553|ref|YP_290210.1| hypothetical protein Tfu_2154 [Thermobifida fusca YX]
>gi|72162554|ref|YP_290211.1| hypothetical protein Tfu_2155 [Thermobifida fusca YX]
>gi|72162561|ref|YP_290218.1| glutamate racemase [Thermobifida fusca YX]
>gi|72162562|ref|YP_290219.1| hypothetical protein Tfu_2163 [Thermobifida fusca YX]
>gi|72162564|ref|YP_290221.1| hypothetical protein Tfu_2165 [Thermobifida fusca YX]
>gi|72162582|ref|YP_290239.1| ribonuclease E and G [Thermobifida fusca YX]
>gi|72162585|ref|YP_290242.1| Sfr protein [Thermobifida fusca YX]
>gi|72162588|ref|YP_290245.1| hypothetical protein Tfu_2189 [Thermobifida fusca YX]
>gi|72162605|ref|YP_290262.1| putative transporter [Thermobifida fusca YX]
>gi|72162613|ref|YP_290270.1| putative transporter [Thermobifida fusca YX]
>gi|72162617|ref|YP_290274.1| GGDEF [Thermobifida fusca YX]
>gi|72162618|ref|YP_290275.1| similar to GTPases [Thermobifida fusca YX]
>gi|72162621|ref|YP_290278.1| nicotinate-nucleotide-dimethylbenzimidazole phosphoribosyltransferase subtype [Thermobifida fusca YX]
>gi|72162623|ref|YP_290280.1| Cobalamin biosynthesis protein CbiB [Thermobifida fusca YX]
>gi|72162625|ref|YP_290282.1| putative integral membrane protein [Thermobifida fusca YX]
>gi|72162626|ref|YP_290283.1| band 7 protein [Thermobifida fusca YX]
>gi|72162631|ref|YP_290288.1| putative two-component sensor kinase [Thermobifida fusca YX]
>gi|72162632|ref|YP_290289.1| regulatory protein, LuxR:Response regulator receiver [Thermobifida fusca YX]
>gi|72162634|ref|YP_290291.1| hypothetical protein Tfu_2235 [Thermobifida fusca YX]
>gi|72162635|ref|YP_290292.1| hypothetical protein Tfu_2236 [Thermobifida fusca YX]
>gi|72162636|ref|YP_290293.1| hypothetical protein Tfu_2237 [Thermobifida fusca YX]
>gi|72162654|ref|YP_290311.1| membrane protein, putative [Thermobifida fusca YX]
>gi|72162655|ref|YP_290312.1| hypothetical protein Tfu_2256 [Thermobifida fusca YX]
>gi|72162657|ref|YP_290314.1| hypothetical protein Tfu_2258 [Thermobifida fusca YX]
>gi|72162667|ref|YP_290324.1| hypothetical protein Tfu_2268 [Thermobifida fusca YX]
>gi|72162669|ref|YP_290326.1| hypothetical protein Tfu_2270 [Thermobifida fusca YX]
>gi|72162672|ref|YP_290329.1| hypothetical protein Tfu_2273 [Thermobifida fusca YX]
>gi|72162676|ref|YP_290333.1| ATP-binding region, ATPase-like [Thermobifida fusca YX]
>gi|72162679|ref|YP_290336.1| hypothetical protein Tfu_2280 [Thermobifida fusca YX]
>gi|72162689|ref|YP_290346.1| hypothetical protein Tfu_2290 [Thermobifida fusca YX]
>gi|72162700|ref|YP_290357.1| Na+/H+ antiporter NhaA [Thermobifida fusca YX]
>gi|72162709|ref|YP_290366.1| putative acyl-CoA dehydrogenase [Thermobifida fusca YX]
>gi|72162710|ref|YP_290367.1| putative secreted protein [Thermobifida fusca YX]
>gi|72162711|ref|YP_290368.1| putative cobalt transport system permease protein [Thermobifida fusca YX]
>gi|72162713|ref|YP_290370.1| similar to Uncharacterized conserved protein [Thermobifida fusca YX]
>gi|72162718|ref|YP_290375.1| hypothetical protein Tfu_2319 [Thermobifida fusca YX]
>gi|72162719|ref|YP_290376.1| putative membrane transport protein [Thermobifida fusca YX]
>gi|72162721|ref|YP_290378.1| similar to N-acetylglucosamine kinase [Thermobifida fusca YX]
>gi|72162729|ref|YP_290386.1| hypothetical protein Tfu_2330 [Thermobifida fusca YX]
>gi|72162746|ref|YP_290403.1| exonuclease [Thermobifida fusca YX]
>gi|72162757|ref|YP_290414.1| hypothetical protein Tfu_2358 [Thermobifida fusca YX]
>gi|72162774|ref|YP_290431.1| Tyrosine protein kinase:Serine/threonine protein kinase [Thermobifida fusca YX]
>gi|72162778|ref|YP_290435.1| hypothetical protein Tfu_2379 [Thermobifida fusca YX]
>gi|72162784|ref|YP_290441.1| FecCD-family membrane transport protein [Thermobifida fusca YX]
>gi|72162786|ref|YP_290443.1| hypothetical protein Tfu_2387 [Thermobifida fusca YX]
>gi|72162795|ref|YP_290452.1| putative cellulose-binding protein [Thermobifida fusca YX]
>gi|72162805|ref|YP_290462.1| H+-transporting two-sector ATPase, delta/epsilon subunit [Thermobifida fusca YX]
>gi|72162807|ref|YP_290464.1| H+-transporting two-sector ATPase, gamma subunit [Thermobifida fusca YX]
>gi|72162810|ref|YP_290467.1| ATP synthase F0, subunit B [Thermobifida fusca YX]
>gi|72162815|ref|YP_290472.1| putative teichoic acid linkage unit synthesis (synthesis of undecaprenylpyrophosphate-N-aetylglucosamine) [Thermobifida fusca YX]
>gi|72162820|ref|YP_290477.1| transcription termination factor Rho [Thermobifida fusca YX]
>gi|72162825|ref|YP_290482.1| similar to Arginyl-tRNA synthetase [Thermobifida fusca YX]
>gi|72162830|ref|YP_290487.1| hypothetical protein Tfu_2431 [Thermobifida fusca YX]
>gi|72162833|ref|YP_290490.1| hypothetical protein Tfu_2434 [Thermobifida fusca YX]
>gi|72162836|ref|YP_290493.1| peptidase M20D, amidohydrolase [Thermobifida fusca YX]
>gi|72162837|ref|YP_290494.1| hypothetical protein Tfu_2438 [Thermobifida fusca YX]
>gi|72162856|ref|YP_290513.1| putative mutT-like protein [Thermobifida fusca YX]
>gi|72162859|ref|YP_290516.1| conserved putative integral membrane protein [Thermobifida fusca YX]
>gi|72162861|ref|YP_290518.1| putative short chain dehydrogenase [Thermobifida fusca YX]
>gi|72162862|ref|YP_290519.1| hypothetical protein Tfu_2463 [Thermobifida fusca YX]

>gi|72162868|ref|YP_290525.1| hypothetical protein Tfu_2469 [Thermobifida fusca YX]
>gi|72162870|ref|YP_290527.1| hypothetical protein Tfu_2471 [Thermobifida fusca YX]
>gi|72162871|ref|YP_290528.1| similar to flavoprotein [Thermobifida fusca YX]
>gi|72162872|ref|YP_290529.1| n-acetylglucosamine-6-phosphate deacetylase [Thermobifida fusca YX]
>gi|72162885|ref|YP_290542.1| beta-N-acetylglucosaminidase (putative secreted protein) [Thermobifida fusca YX]
>gi|72162886|ref|YP_290543.1| phosphoryl transfer system, HPr [Thermobifida fusca YX]
>gi|72162892|ref|YP_290549.1| hypothetical protein Tfu_2493 [Thermobifida fusca YX]
>gi|72162896|ref|YP_290553.1| hypothetical protein Tfu_2497 [Thermobifida fusca YX]
>gi|72162897|ref|YP_290554.1| putative integral membrane protein [Thermobifida fusca YX]
>gi|72162898|ref|YP_290555.1| hypothetical protein Tfu_2499 [Thermobifida fusca YX]
>gi|72162900|ref|YP_290557.1| hypothetical protein Tfu_2501 [Thermobifida fusca YX]
>gi|72162902|ref|YP_290559.1| possible conserved membrane protein [Thermobifida fusca YX]
>gi|72162903|ref|YP_290560.1| putative integral membrane protein [Thermobifida fusca YX]
>gi|72162915|ref|YP_290572.1| hypothetical protein Tfu_2516 [Thermobifida fusca YX]
>gi|72162920|ref|YP_290577.1| hypothetical protein Tfu_2521 [Thermobifida fusca YX]
>gi|72162931|ref|YP_290588.1| putative transferase [Thermobifida fusca YX]
>gi|72162934|ref|YP_290591.1| hypothetical protein Tfu_2535 [Thermobifida fusca YX]
>gi|72162935|ref|YP_290592.1| hypothetical protein Tfu_2536 [Thermobifida fusca YX]
>gi|72162942|ref|YP_290599.1| hypothetical protein Tfu_2543 [Thermobifida fusca YX]
>gi|72162946|ref|YP_290603.1| phosphoribosylaminoimidazole carboxylase, ATPase subunit [Thermobifida fusca YX]
>gi|72162955|ref|YP_290612.1| hypothetical protein Tfu_2556 [Thermobifida fusca YX]
>gi|72162957|ref|YP_290614.1| putative membrane protein [Thermobifida fusca YX]
>gi|72162965|ref|YP_290622.1| galactokinase [Thermobifida fusca YX]
>gi|72162966|ref|YP_290623.1| cell envelope-related transcriptional attenuator [Thermobifida fusca YX]
>gi|72162974|ref|YP_290631.1| hypothetical protein Tfu_2575 [Thermobifida fusca YX]
>gi|72162977|ref|YP_290634.1| hypothetical protein Tfu_2578 [Thermobifida fusca YX]
>gi|72162983|ref|YP_290640.1| hypothetical protein Tfu_2584 [Thermobifida fusca YX]
>gi|72162984|ref|YP_290641.1| hypothetical protein Tfu_2585 [Thermobifida fusca YX]
>gi|72162986|ref|YP_290643.1| molybdopterin dehydrogenase [Thermobifida fusca YX]
>gi|72162987|ref|YP_290644.1| hypothetical protein Tfu_2588 [Thermobifida fusca YX]
>gi|72162999|ref|YP_290656.1| hypothetical protein Tfu_2600 [Thermobifida fusca YX]
>gi|72163000|ref|YP_290657.1| hypothetical protein Tfu_2601 [Thermobifida fusca YX]
>gi|72163005|ref|YP_290662.1| alanine racemase region [Thermobifida fusca YX]
>gi|72163006|ref|YP_290663.1| hypothetical protein Tfu_2607 [Thermobifida fusca YX]
>gi|72163007|ref|YP_290664.1| Protein of unknown function UPF0031:YjeF-related protein, N-terminal [Thermobifida fusca YX]
>gi|72163014|ref|YP_290671.1| hypothetical protein Tfu_2615 [Thermobifida fusca YX]
>gi|72163022|ref|YP_290679.1| hypothetical protein Tfu_2623 [Thermobifida fusca YX]
>gi|72163051|ref|YP_290708.1| hypothetical protein Tfu_2652 [Thermobifida fusca YX]
>gi|72163054|ref|YP_290711.1| Ribosomal protein L7/L12 [Thermobifida fusca YX]
>gi|72163068|ref|YP_290725.1| hypothetical protein Tfu_2669 [Thermobifida fusca YX]
>gi|72163072|ref|YP_290729.1| putative peptidase [Thermobifida fusca YX]
>gi|72163076|ref|YP_290733.1| hypothetical protein Tfu_2677 [Thermobifida fusca YX]
>gi|72163079|ref|YP_290736.1| hypothetical protein Tfu_2680 [Thermobifida fusca YX]
>gi|72163097|ref|YP_290754.1| hypothetical protein Tfu_2698 [Thermobifida fusca YX]
>gi|72163102|ref|YP_290759.1| putative cytochrome C biogenesis membrane protein [Thermobifida fusca YX]
>gi|72163109|ref|YP_290766.1| hypothetical protein Tfu_2710 [Thermobifida fusca YX]
>gi|72163114|ref|YP_290771.1| hypothetical protein Tfu_2715 [Thermobifida fusca YX]
>gi|72163115|ref|YP_290772.1| alanine-rich protein [Thermobifida fusca YX]
>gi|72163120|ref|YP_290777.1| hypothetical protein Tfu_2721 [Thermobifida fusca YX]
>gi|72163137|ref|YP_290794.1| hypothetical protein Tfu_2738 [Thermobifida fusca YX]
>gi|72163138|ref|YP_290795.1| putative acetyltransferase [Thermobifida fusca YX]
>gi|72163140|ref|YP_290797.1| phosphate ABC transporter, permease protein PstC [Thermobifida fusca YX]
>gi|72163155|ref|YP_290812.1| hypothetical protein Tfu_2756 [Thermobifida fusca YX]
>gi|72163160|ref|YP_290817.1| methylmalonyl-CoA mutase [Thermobifida fusca YX]
>gi|72163164|ref|YP_290821.1| Phosphoenolpyruvate-protein phosphotransferase [Thermobifida fusca YX]
>gi|72163168|ref|YP_290825.1| hypothetical protein Tfu_2769 [Thermobifida fusca YX]
>gi|72163180|ref|YP_290837.1| similar to Uncharacterized protein of PSP1 [Thermobifida fusca YX]
>gi|72163181|ref|YP_290838.1| DNA polymerase III delta prime subunit [Thermobifida fusca YX]
>gi|72163182|ref|YP_290839.1| thymidylate kinase [Thermobifida fusca YX]
>gi|72163185|ref|YP_290842.1| hypothetical protein Tfu_2786 [Thermobifida fusca YX]
>gi|72163192|ref|YP_290849.1| regulatory protein, ArsR [Thermobifida fusca YX]
>gi|72163193|ref|YP_290850.1| conserved membrane protein [Thermobifida fusca YX]
>gi|72163205|ref|YP_290862.1| hypothetical protein Tfu_2806 [Thermobifida fusca YX]
>gi|72163206|ref|YP_290863.1| ATP-binding region, ATPase-like [Thermobifida fusca YX]
>gi|72163208|ref|YP_290865.1| regulatory protein, LuxR [Thermobifida fusca YX]
>gi|72163211|ref|YP_290868.1| hypothetical protein Tfu_2812 [Thermobifida fusca YX]
>gi|72163214|ref|YP_290871.1| similar to hydrolases or acyltransferases (alpha/beta hydrolase superfamily) [Thermobifida fusca YX]
>gi|72163220|ref|YP_290877.1| cation-transporting P-ATPase PacL [Thermobifida fusca YX]
>gi|72163231|ref|YP_290888.1| hypothetical protein Tfu_2832 [Thermobifida fusca YX]
>gi|72163232|ref|YP_290889.1| putative secreted protein [Thermobifida fusca YX]

>gi|72163233|ref|YP_290890.1| hypothetical protein Tfu_2834 [Thermobifida fusca YX]
>gi|72163235|ref|YP_290892.1| hypothetical protein Tfu_2836 [Thermobifida fusca YX]
>gi|72163241|ref|YP_290898.1| hypothetical protein Tfu_2842 [Thermobifida fusca YX]
>gi|72163245|ref|YP_290902.1| hypothetical protein Tfu_2846 [Thermobifida fusca YX]
>gi|72163247|ref|YP_290904.1| ATPase, E1-E2 type:Copper-translocating P-type ATPase:Heavy metal translocating P-type ATPase [Thermobifida fusca YX]
>gi|72163248|ref|YP_290905.1| hypothetical protein Tfu_2849 [Thermobifida fusca YX]
>gi|72163249|ref|YP_290906.1| hypothetical protein Tfu_2850 [Thermobifida fusca YX]
>gi|72163250|ref|YP_290907.1| hypothetical protein Tfu_2851 [Thermobifida fusca YX]
>gi|72163253|ref|YP_290910.1| hypothetical protein Tfu_2854 [Thermobifida fusca YX]
>gi|72163256|ref|YP_290913.1| ATP-binding region, ATPase-like [Thermobifida fusca YX]
>gi|72163258|ref|YP_290915.1| hypothetical protein Tfu_2859 [Thermobifida fusca YX]
>gi|72163259|ref|YP_290916.1| putative transmembrane transport protein [Thermobifida fusca YX]
>gi|72163273|ref|YP_290930.1| hypothetical protein Tfu_2874 [Thermobifida fusca YX]
>gi|72163283|ref|YP_290940.1| pantoate-beta-alanine ligase [Thermobifida fusca YX]
>gi|72163286|ref|YP_290943.1| similar to membrane protein [Thermobifida fusca YX]
>gi|72163287|ref|YP_290944.1| similar to Uncharacterized conserved protein [Thermobifida fusca YX]
>gi|72163289|ref|YP_290946.1| 7,8-Dihydro-6-hydroxymethylpterin-pyrophosphokinase, HPPK [Thermobifida fusca YX]
>gi|72163298|ref|YP_290955.1| hypothetical protein Tfu_2899 [Thermobifida fusca YX]
>gi|72163300|ref|YP_290957.1| hypothetical protein Tfu_2901 [Thermobifida fusca YX]
>gi|72163316|ref|YP_290973.1| integral membrane protein [Thermobifida fusca YX]
>gi|72163353|ref|YP_291010.1| hypothetical protein Tfu_2954 [Thermobifida fusca YX]
>gi|72163354|ref|YP_291011.1| hypothetical protein Tfu_2955 [Thermobifida fusca YX]
>gi|72163359|ref|YP_291016.1| ATP-binding region, ATPase-like [Thermobifida fusca YX]
>gi|72163362|ref|YP_291019.1| hypothetical protein Tfu_2963 [Thermobifida fusca YX]
>gi|72163368|ref|YP_291025.1| hypothetical protein Tfu_2969 [Thermobifida fusca YX]
>gi|72163376|ref|YP_291033.1| putative Lsr2-like protein [Thermobifida fusca YX]
>gi|72163381|ref|YP_291038.1| hypothetical protein Tfu_2982 [Thermobifida fusca YX]
>gi|72163385|ref|YP_291042.1| hypothetical protein Tfu_2986 [Thermobifida fusca YX]
>gi|72163389|ref|YP_291046.1| cellulose-binding, family II, bacterial type [Thermobifida fusca YX]
>gi|72163391|ref|YP_291048.1| hypothetical protein Tfu_2992 [Thermobifida fusca YX]
>gi|72163393|ref|YP_291050.1| putative transport protein [Thermobifida fusca YX]
>gi|72163395|ref|YP_291052.1| hypothetical protein Tfu_2996 [Thermobifida fusca YX]
>gi|72163396|ref|YP_291053.1| hypothetical protein Tfu_2997 [Thermobifida fusca YX]
>gi|72163405|ref|YP_291062.1| hypothetical protein Tfu_3006 [Thermobifida fusca YX]
>gi|72163407|ref|YP_291064.1| orotate phosphoribosyl transferase [Thermobifida fusca YX]
>gi|72163410|ref|YP_291067.1| hypothetical protein Tfu_3011 [Thermobifida fusca YX]
>gi|72163419|ref|YP_291076.1| hypothetical protein Tfu_3020 [Thermobifida fusca YX]
>gi|72163428|ref|YP_291085.1| putative integral membrane protein [Thermobifida fusca YX]
>gi|72163429|ref|YP_291086.1| putative integral membrane protein [Thermobifida fusca YX]
>gi|72163431|ref|YP_291088.1| hypothetical protein Tfu_3032 [Thermobifida fusca YX]
>gi|72163440|ref|YP_291097.1| putative stress-inducible protein [Thermobifida fusca YX]
>gi|72163450|ref|YP_291107.1| pyruvate dehydrogenase complex, E2 component, dihydrolipoamide acetyltransferase [Thermobifida fusca YX]
>gi|72163453|ref|YP_291110.1| glycosyltransferases involved in cell wall biogenesis [Thermobifida fusca YX]
>gi|72163464|ref|YP_291121.1| Tyrosine protein kinase:Serine/threonine protein kinase [Thermobifida fusca YX]
>gi|72163480|ref|YP_291137.1| hypothetical protein Tfu_3081 [Thermobifida fusca YX]
>gi|72163483|ref|YP_291140.1| conserved hypothetical membrane permease protein [Thermobifida fusca YX]
>gi|72163493|ref|YP_291150.1| Single-strand binding protein [Thermobifida fusca YX]
>gi|72163495|ref|YP_291152.1| putative integral membrane protein [Thermobifida fusca YX]
>gi|72163496|ref|YP_291153.1| putative penicillin-binding protein [Thermobifida fusca YX]
>gi|72163499|ref|YP_291156.1| hypothetical protein Tfu_3100 [Thermobifida fusca YX]
>gi|72163500|ref|YP_291157.1| putative membrane protein [Thermobifida fusca YX]
>gi|72163506|ref|YP_291163.1| hypothetical protein Tfu_3107 [Thermobifida fusca YX]
>gi|72163516|ref|YP_291173.1| Ribosomal protein L34 [Thermobifida fusca YX]
>gi|29140556|ref|NP_803898.1| DnaJ protein [Salmonella enterica subsp. enterica serovar Typhi Ty2]
>gi|29140700|ref|NP_804042.1| dihydrolipoamide acetyltransferase component (E2) of pyruvate dehydrogenase [Salmonella enterica subsp. enterica serovar Typhi Ty2]
>gi|29140704|ref|NP_804046.1| hypothetical protein t0163 [Salmonella enterica subsp. enterica serovar Typhi Ty2]
>gi|29140707|ref|NP_804049.1| 2-keto-3-deoxygluconate permease [Salmonella enterica subsp. enterica serovar Typhi Ty2]
>gi|29140742|ref|NP_804084.1| ferrichrome transport protein FhuB precursor [Salmonella enterica subsp. enterica serovar Typhi Ty2]
>gi|29140766|ref|NP_804108.1| UDP-3-O-[3-hydroxymyristoyl] glucosamine N-acyltransferase [Salmonella enterica subsp. enterica serovar Typhi Ty2]
>gi|29140807|ref|NP_804149.1| sigma-E factor regulatory protein RseC [Salmonella enterica subsp. enterica serovar Typhi Ty2]
>gi|29140864|ref|NP_804206.1| putative DNA-binding protein [Salmonella enterica subsp. enterica serovar Typhi Ty2]
>gi|29140879|ref|NP_804221.1| hypothetical protein t0350 [Salmonella enterica subsp. enterica serovar Typhi Ty2]
>gi|29140893|ref|NP_804235.1| putative permease [Salmonella enterica subsp. enterica serovar Typhi Ty2]
>gi|29140933|ref|NP_804275.1| putative membrane protein [Salmonella enterica subsp. enterica serovar Typhi Ty2]
>gi|29140937|ref|NP_804279.1| hypothetical protein t0410 [Salmonella enterica subsp. enterica serovar Typhi Ty2]
>gi|29140956|ref|NP_804298.1| cell division protein [Salmonella enterica subsp. enterica serovar Typhi Ty2]
>gi|29140971|ref|NP_804313.1| hypothetical protein t0450 [Salmonella enterica subsp. enterica serovar Typhi Ty2]

>gi|29141015|ref|NP_804357.1| DedD protein [Salmonella enterica subsp. enterica serovar Typhi Ty2]
>gi|29141125|ref|NP_804467.1| heme exporter protein B1 [Salmonella enterica subsp. enterica serovar Typhi Ty2]
>gi|29141127|ref|NP_804469.1| heme exporter protein D1 [Salmonella enterica subsp. enterica serovar Typhi Ty2]
>gi|29141135|ref|NP_804477.1| secreted effector protein [Salmonella enterica subsp. enterica serovar Typhi Ty2]
>gi|29141139|ref|NP_804481.1| putative membrane protein [Salmonella enterica subsp. enterica serovar Typhi Ty2]
>gi|29141199|ref|NP_804541.1| putative permease transmembrane component [Salmonella enterica subsp. enterica serovar Typhi Ty2]
>gi|29141222|ref|NP_804564.1| putative membrane protein [Salmonella enterica subsp. enterica serovar Typhi Ty2]
>gi|29141248|ref|NP_804590.1| putative efflux system protein [Salmonella enterica subsp. enterica serovar Typhi Ty2]
>gi|29141325|ref|NP_804667.1| putative propanediol utilization protein PduK [Salmonella enterica subsp. enterica serovar Typhi Ty2]
>gi|29141326|ref|NP_804668.1| putative propanediol utilization protein PduJ [Salmonella enterica subsp. enterica serovar Typhi Ty2]
>gi|29141350|ref|NP_804692.1| cobalamin (5'-phosphate) synthase [Salmonella enterica subsp. enterica serovar Typhi Ty2]
>gi|29141383|ref|NP_804725.1| flagellar biosynthetic protein FliR [Salmonella enterica subsp. enterica serovar Typhi Ty2]
>gi|29141390|ref|NP_804732.1| flagellar hook-length control protein [Salmonella enterica subsp. enterica serovar Typhi Ty2]
>gi|29141393|ref|NP_804735.1| flagellar assembly protein FliH [Salmonella enterica subsp. enterica serovar Typhi Ty2]
>gi|29141424|ref|NP_804766.1| tyrosine-specific transport protein [Salmonella enterica subsp. enterica serovar Typhi Ty2]
>gi|29141446|ref|NP_804788.1| flagellar biosynthesis protein FlhA [Salmonella enterica subsp. enterica serovar Typhi Ty2]
>gi|29141478|ref|NP_804820.1| hypothetical protein t0997 [Salmonella enterica subsp. enterica serovar Typhi Ty2]
>gi|29141508|ref|NP_804850.1| ProP effector [Salmonella enterica subsp. enterica serovar Typhi Ty2]
>gi|29141514|ref|NP_804856.1| hypothetical protein t1039 [Salmonella enterica subsp. enterica serovar Typhi Ty2]
>gi|29141522|ref|NP_804864.1| phosphotransferase enzyme II, C component [Salmonella enterica subsp. enterica serovar Typhi Ty2]
>gi|29141529|ref|NP_804871.1| hypothetical protein t1054 [Salmonella enterica subsp. enterica serovar Typhi Ty2]
>gi|29141542|ref|NP_804884.1| hypothetical protein t1067 [Salmonella enterica subsp. enterica serovar Typhi Ty2]
>gi|29141555|ref|NP_804897.1| putative membrane protein [Salmonella enterica subsp. enterica serovar Typhi Ty2]
>gi|29141609|ref|NP_804951.1| hypothetical protein t1139 [Salmonella enterica subsp. enterica serovar Typhi Ty2]
>gi|29141619|ref|NP_804961.1| hypothetical protein t1150 [Salmonella enterica subsp. enterica serovar Typhi Ty2]
>gi|29141622|ref|NP_804964.1| putative membrane protein [Salmonella enterica subsp. enterica serovar Typhi Ty2]
>gi|29141627|ref|NP_804969.1| putative membrane protein [Salmonella enterica subsp. enterica serovar Typhi Ty2]
>gi|29141679|ref|NP_805021.1| 50S ribosomal subunit protein L35 [Salmonella enterica subsp. enterica serovar Typhi Ty2]
>gi|29141685|ref|NP_805027.1| vitamin B12 transport system permease [Salmonella enterica subsp. enterica serovar Typhi Ty2]
>gi|29141696|ref|NP_805038.1| putative membrane protein [Salmonella enterica subsp. enterica serovar Typhi Ty2]
>gi|29141699|ref|NP_805041.1| hypothetical protein t1235 [Salmonella enterica subsp. enterica serovar Typhi Ty2]
>gi|29141707|ref|NP_805049.1| major outer membrane lipoprotein [Salmonella enterica subsp. enterica serovar Typhi Ty2]
>gi|29141708|ref|NP_805050.1| major outer membrane lipoprotein [Salmonella enterica subsp. enterica serovar Typhi Ty2]
>gi|29141730|ref|NP_805072.1| putative pathogenicity island effector protein [Salmonella enterica subsp. enterica serovar Typhi Ty2]
>gi|29141759|ref|NP_805101.1| putative secreted protein [Salmonella enterica subsp. enterica serovar Typhi Ty2]
>gi|29141772|ref|NP_805114.1| outer membrane lipoprotein SlyB precursor [Salmonella enterica subsp. enterica serovar Typhi Ty2]
>gi|29141781|ref|NP_805123.1| hypothetical protein t1322 [Salmonella enterica subsp. enterica serovar Typhi Ty2]
>gi|29141784|ref|NP_805126.1| putative NADH reducing dehydrogenase [Salmonella enterica subsp. enterica serovar Typhi Ty2]
>gi|29141787|ref|NP_805129.1| putative membrane protein [Salmonella enterica subsp. enterica serovar Typhi Ty2]
>gi|29141821|ref|NP_805163.1| putative membrane protein [Salmonella enterica subsp. enterica serovar Typhi Ty2]
>gi|29141858|ref|NP_805200.1| putative secreted stress response protein [Salmonella enterica subsp. enterica serovar Typhi Ty2]
>gi|29141877|ref|NP_805219.1| putative membrane protein [Salmonella enterica subsp. enterica serovar Typhi Ty2]
>gi|29141885|ref|NP_805227.1| hypothetical protein t1434 [Salmonella enterica subsp. enterica serovar Typhi Ty2]
>gi|29141889|ref|NP_805231.1| putative membrane protein [Salmonella enterica subsp. enterica serovar Typhi Ty2]
>gi|29141947|ref|NP_805289.1| putative virulence effector protein [Salmonella enterica subsp. enterica serovar Typhi Ty2]
>gi|29141954|ref|NP_805296.1| putative membrane protein [Salmonella enterica subsp. enterica serovar Typhi Ty2]
>gi|29141997|ref|NP_805339.1| putative multidrug transporter [Salmonella enterica subsp. enterica serovar Typhi Ty2]
>gi|29142003|ref|NP_805345.1| O6-methylguanine-DNA-alkyltransferase [Salmonella enterica subsp. enterica serovar Typhi Ty2]
>gi|29142054|ref|NP_805396.1| osmotically inducible lipoprotein B precursor [Salmonella enterica subsp. enterica serovar Typhi Ty2]
>gi|29142076|ref|NP_805418.1| hypothetical protein t1640 [Salmonella enterica subsp. enterica serovar Typhi Ty2]
>gi|29142082|ref|NP_805424.1| putative membrane protein [Salmonella enterica subsp. enterica serovar Typhi Ty2]
>gi|29142085|ref|NP_805427.1| TonB protein [Salmonella enterica subsp. enterica serovar Typhi Ty2]
>gi|29142097|ref|NP_805439.1| DNA-binding protein [Salmonella enterica subsp. enterica serovar Typhi Ty2]
>gi|29142147|ref|NP_805489.1| putative lipoprotein [Salmonella enterica subsp. enterica serovar Typhi Ty2]
>gi|29142167|ref|NP_805509.1| ribonuclease E [Salmonella enterica subsp. enterica serovar Typhi Ty2]
>gi|29142231|ref|NP_805573.1| hypothetical protein t1800 [Salmonella enterica subsp. enterica serovar Typhi Ty2]
>gi|29142238|ref|NP_805580.1| membrane protein, suppressor for copper-sensitivity A [Salmonella enterica subsp. enterica serovar Typhi Ty2]
>gi|29142240|ref|NP_805582.1| hypothetical protein t1809 [Salmonella enterica subsp. enterica serovar Typhi Ty2]
>gi|29142260|ref|NP_805602.1| hypothetical protein t1830 [Salmonella enterica subsp. enterica serovar Typhi Ty2]
>gi|29142312|ref|NP_805654.1| putative bacteriophage protein [Salmonella enterica subsp. enterica serovar Typhi Ty2]
>gi|29142314|ref|NP_805656.1| putative bacteriophage protein [Salmonella enterica subsp. enterica serovar Typhi Ty2]
>gi|29142324|ref|NP_805666.1| putative secreted protein [Salmonella enterica subsp. enterica serovar Typhi Ty2]
>gi|29142327|ref|NP_805669.1| putative prophage membrane protein [Salmonella enterica subsp. enterica serovar Typhi Ty2]
>gi|29142328|ref|NP_805670.1| putative lipoprotein [Salmonella enterica subsp. enterica serovar Typhi Ty2]
>gi|29142331|ref|NP_805673.1| hypothetical prophage protein [Salmonella enterica subsp. enterica serovar Typhi Ty2]
>gi|29142396|ref|NP_805738.1| cell division protein FtsK [Salmonella enterica subsp. enterica serovar Typhi Ty2]
>gi|29142400|ref|NP_805742.1| transport ATP-binding protein CydC [Salmonella enterica subsp. enterica serovar Typhi Ty2]
>gi|29142444|ref|NP_805786.1| putative membrane protein [Salmonella enterica subsp. enterica serovar Typhi Ty2]
>gi|29142477|ref|NP_805819.1| putative membrane protein [Salmonella enterica subsp. enterica serovar Typhi Ty2]
>gi|29142492|ref|NP_805834.1| putative membrane protein [Salmonella enterica subsp. enterica serovar Typhi Ty2]

>gi|29142532|ref|NP_805874.1| ABC transporter permease (FecCD_family) [Salmonella enterica subsp. enterica serovar Typhi Ty2]
>gi|29142534|ref|NP_805876.1| probable secreted protein [Salmonella enterica subsp. enterica serovar Typhi Ty2]
>gi|29142538|ref|NP_805880.1| hypothetical protein t2126 [Salmonella enterica subsp. enterica serovar Typhi Ty2]
>gi|29142541|ref|NP_805883.1| tolA protein [Salmonella enterica subsp. enterica serovar Typhi Ty2]
>gi|29142580|ref|NP_805922.1| potassium-transporting ATPase C chain [Salmonella enterica subsp. enterica serovar Typhi Ty2]
>gi|29142630|ref|NP_805972.1| rare lipoprotein A precursor [Salmonella enterica subsp. enterica serovar Typhi Ty2]
>gi|29142647|ref|NP_805989.1| hypothetical protein t2247 [Salmonella enterica subsp. enterica serovar Typhi Ty2]
>gi|29142654|ref|NP_805996.1| molybdopterin-containing oxidoreductase membrane anchor subunit [Salmonella enterica subsp. enterica serovar Typhi Ty2]
>gi|29142675|ref|NP_806017.1| hypothetical membrane protein p43 [Salmonella enterica subsp. enterica serovar Typhi Ty2]
>gi|29142676|ref|NP_806018.1| ferric enterobactin transport protein FepD [Salmonella enterica subsp. enterica serovar Typhi Ty2]
>gi|29142685|ref|NP_806027.1| putative membrane protein [Salmonella enterica subsp. enterica serovar Typhi Ty2]
>gi|29142742|ref|NP_806084.1| putative membrane protein [Salmonella enterica subsp. enterica serovar Typhi Ty2]
>gi|29142746|ref|NP_806088.1| thioredoxin-like protein [Salmonella enterica subsp. enterica serovar Typhi Ty2]
>gi|29142776|ref|NP_806118.1| maltose O-acetyltransferase [Salmonella enterica subsp. enterica serovar Typhi Ty2]
>gi|29142795|ref|NP_806137.1| hypothetical protein t2407 [Salmonella enterica subsp. enterica serovar Typhi Ty2]
>gi|29142857|ref|NP_806199.1| exonuclease SbcC [Salmonella enterica subsp. enterica serovar Typhi Ty2]
>gi|29142885|ref|NP_806227.1| RhtC-like transporter [Salmonella enterica subsp. enterica serovar Typhi Ty2]
>gi|29142974|ref|NP_806316.1| hypothetical protein t2599 [Salmonella enterica subsp. enterica serovar Typhi Ty2]
>gi|29143031|ref|NP_806373.1| putative bacteriophage tail protein [Salmonella enterica subsp. enterica serovar Typhi Ty2]
>gi|29143073|ref|NP_806415.1| putative transmembrane transport protein [Salmonella enterica subsp. enterica serovar Typhi Ty2]
>gi|29143093|ref|NP_806435.1| glucitol/sorbitol-specific IIBC component of PTS system [Salmonella enterica subsp. enterica serovar Typhi Ty2]
>gi|29143112|ref|NP_806454.1| formate hydrogenlyase subunit 3 [Salmonella enterica subsp. enterica serovar Typhi Ty2]
>gi|29143146|ref|NP_806488.1| pathogenicity island 1 effector protein [Salmonella enterica subsp. enterica serovar Typhi Ty2]
>gi|29143181|ref|NP_806523.1| lipoprotein NlpD precursor [Salmonella enterica subsp. enterica serovar Typhi Ty2]
>gi|29143188|ref|NP_806530.1| hypothetical protein t2833 [Salmonella enterica subsp. enterica serovar Typhi Ty2]
>gi|29143218|ref|NP_806560.1| hypothetical protein t2864 [Salmonella enterica subsp. enterica serovar Typhi Ty2]
>gi|29143300|ref|NP_806642.1| hypothetical protein t2949 [Salmonella enterica subsp. enterica serovar Typhi Ty2]
>gi|29143341|ref|NP_806683.1| putative membrane protein [Salmonella enterica subsp. enterica serovar Typhi Ty2]
>gi|29143417|ref|NP_806759.1| putative membrane protein [Salmonella enterica subsp. enterica serovar Typhi Ty2]
>gi|29143460|ref|NP_806802.1| hypothetical protein t3119 [Salmonella enterica subsp. enterica serovar Typhi Ty2]
>gi|29143491|ref|NP_806833.1| putative membrane protein [Salmonella enterica subsp. enterica serovar Typhi Ty2]
>gi|29143534|ref|NP_806876.1| ATP-dependent RNA helicase [Salmonella enterica subsp. enterica serovar Typhi Ty2]
>gi|29143540|ref|NP_806882.1| protein chain initiation factor 2 [Salmonella enterica subsp. enterica serovar Typhi Ty2]
>gi|29143544|ref|NP_806886.1| export membrane protein [Salmonella enterica subsp. enterica serovar Typhi Ty2]
>gi|29143609|ref|NP_806951.1| hypothetical protein t3276 [Salmonella enterica subsp. enterica serovar Typhi Ty2]
>gi|29143621|ref|NP_806963.1| rod shape-determining protein [Salmonella enterica subsp. enterica serovar Typhi Ty2]
>gi|29143626|ref|NP_806968.1| putative membrane protein [Salmonella enterica subsp. enterica serovar Typhi Ty2]
>gi|29143627|ref|NP_806969.1| biotin carboxyl carrier protein [Salmonella enterica subsp. enterica serovar Typhi Ty2]
>gi|29143649|ref|NP_806991.1| sec-independent protein translocase protein [Salmonella enterica subsp. enterica serovar Typhi Ty2]
>gi|29143650|ref|NP_806992.1| sec-independent protein translocase protein [Salmonella enterica subsp. enterica serovar Typhi Ty2]
>gi|29143670|ref|NP_807012.1| putative membrane protein [Salmonella enterica subsp. enterica serovar Typhi Ty2]
>gi|29143687|ref|NP_807029.1| uroporphyrinogen III methylase [Salmonella enterica subsp. enterica serovar Typhi Ty2]
>gi|29143747|ref|NP_807089.1| putative regulatory protein [Salmonella enterica subsp. enterica serovar Typhi Ty2]
>gi|29143762|ref|NP_807104.1| hypothetical protein t3442 [Salmonella enterica subsp. enterica serovar Typhi Ty2]
>gi|29143789|ref|NP_807131.1| 50S ribosomal subunit protein L7/L12 [Salmonella enterica subsp. enterica serovar Typhi Ty2]
>gi|29143830|ref|NP_807172.1| cell division protein [Salmonella enterica subsp. enterica serovar Typhi Ty2]
>gi|29143847|ref|NP_807189.1| putative ABC transporter, membrane component [Salmonella enterica subsp. enterica serovar Typhi Ty2]
>gi|29143917|ref|NP_807259.1| hypothetical protein t3618 [Salmonella enterica subsp. enterica serovar Typhi Ty2]
>gi|29143931|ref|NP_807273.1| high affinity ribose transport protein [Salmonella enterica subsp. enterica serovar Typhi Ty2]
>gi|29143942|ref|NP_807284.1| ATP synthase protein I. [Salmonella enterica subsp. enterica serovar Typhi Ty2]
>gi|29143944|ref|NP_807286.1| ATP synthase subunit C [Salmonella enterica subsp. enterica serovar Typhi Ty2]
>gi|29143945|ref|NP_807287.1| ATP synthase subunit B [Salmonella enterica subsp. enterica serovar Typhi Ty2]
>gi|29143959|ref|NP_807301.1| probable PTS system permease [Salmonella enterica subsp. enterica serovar Typhi Ty2]
>gi|29143993|ref|NP_807335.1| heme exporter protein B2 [Salmonella enterica subsp. enterica serovar Typhi Ty2]
>gi|29143995|ref|NP_807337.1| heme exporter protein D2 [Salmonella enterica subsp. enterica serovar Typhi Ty2]
>gi|29144020|ref|NP_807362.1| two-component system sensor histidine kinase [Salmonella enterica subsp. enterica serovar Typhi Ty2]
>gi|29144054|ref|NP_807396.1| glutamate permease [Salmonella enterica subsp. enterica serovar Typhi Ty2]
>gi|29144066|ref|NP_807408.1| putative TetR-family transcriptional regulator [Salmonella enterica subsp. enterica serovar Typhi Ty2]
>gi|29144107|ref|NP_807449.1| putative autotransporter [Salmonella enterica subsp. enterica serovar Typhi Ty2]
>gi|29144108|ref|NP_807450.1| putative lipoprotein [Salmonella enterica subsp. enterica serovar Typhi Ty2]
>gi|29144110|ref|NP_807452.1| hypothetical protein t3831 [Salmonella enterica subsp. enterica serovar Typhi Ty2]
>gi|29144150|ref|NP_807492.1| putative outer membrane protein [Salmonella enterica subsp. enterica serovar Typhi Ty2]
>gi|29144161|ref|NP_807503.1| dipeptide transport system permease protein DppC [Salmonella enterica subsp. enterica serovar Typhi Ty2]
>gi|29144216|ref|NP_807558.1| putative membrane protein [Salmonella enterica subsp. enterica serovar Typhi Ty2]
>gi|29144220|ref|NP_807562.1| cell division protein [Salmonella enterica subsp. enterica serovar Typhi Ty2]
>gi|29144237|ref|NP_807579.1| hypothetical protein t3969 [Salmonella enterica subsp. enterica serovar Typhi Ty2]
>gi|29144275|ref|NP_807617.1| putative membrane protein [Salmonella enterica subsp. enterica serovar Typhi Ty2]
>gi|29144283|ref|NP_807625.1| putative membrane protein [Salmonella enterica subsp. enterica serovar Typhi Ty2]
>gi|29144289|ref|NP_807631.1| DamX protein [Salmonella enterica subsp. enterica serovar Typhi Ty2]

>gi|29144315|ref|NP_807657.1| FKBP-type peptidyl-prolyl cis-trans isomerase [Salmonella enterica subsp. enterica serovar Typhi Ty2]
>gi|29144321|ref|NP_807663.1| hypothetical protein t4056 [Salmonella enterica subsp. enterica serovar Typhi Ty2]
>gi|29144338|ref|NP_807680.1| 50S ribosomal subunit protein L29 [Salmonella enterica subsp. enterica serovar Typhi Ty2]
>gi|29144346|ref|NP_807688.1| 50S ribosomal subunit protein L18 [Salmonella enterica subsp. enterica serovar Typhi Ty2]
>gi|29144349|ref|NP_807691.1| 50S ribosomal subunit protein L15 [Salmonella enterica subsp. enterica serovar Typhi Ty2]
>gi|29144387|ref|NP_807729.1| putative membrane protein [Salmonella enterica subsp. enterica serovar Typhi Ty2]
>gi|29144390|ref|NP_807732.1| hypothetical protein t4128 [Salmonella enterica subsp. enterica serovar Typhi Ty2]
>gi|29144412|ref|NP_807754.1| putative membrane protein [Salmonella enterica subsp. enterica serovar Typhi Ty2]
>gi|29144423|ref|NP_807765.1| single-strand DNA-binding protein [Salmonella enterica subsp. enterica serovar Typhi Ty2]
>gi|29144520|ref|NP_807862.1| putative membrane protein [Salmonella enterica subsp. enterica serovar Typhi Ty2]
>gi|29144609|ref|NP_807951.1| hypothetical protein t4358 [Salmonella enterica subsp. enterica serovar Typhi Ty2]
>gi|29144619|ref|NP_807961.1| putative membrane protein [Salmonella enterica subsp. enterica serovar Typhi Ty2]
>gi|29144628|ref|NP_807970.1| FxsA protein [Salmonella enterica subsp. enterica serovar Typhi Ty2]
>gi|29144629|ref|NP_807971.1| putative permease [Salmonella enterica subsp. enterica serovar Typhi Ty2]
>gi|29144661|ref|NP_808003.1| HflK protein [Salmonella enterica subsp. enterica serovar Typhi Ty2]
>gi|29144692|ref|NP_808034.1| hypothetical protein t4447 [Salmonella enterica subsp. enterica serovar Typhi Ty2]
>gi|29144710|ref|NP_808052.1| hypothetical protein t4465 [Salmonella enterica subsp. enterica serovar Typhi Ty2]
>gi|29144807|ref|NP_808149.1| putative membrane protein [Salmonella enterica subsp. enterica serovar Typhi Ty2]
>gi|29144818|ref|NP_808160.1| putative secreted protein [Salmonella enterica subsp. enterica serovar Typhi Ty2]
>gi|29144824|ref|NP_808166.1| conserved hypothetical regulatory protein [Salmonella enterica subsp. enterica serovar Typhi Ty2]
>gi|29144835|ref|NP_808177.1| hypothetical protein t4605 [Salmonella enterica subsp. enterica serovar Typhi Ty2]
>gi|56416386|ref|YP_153460.1| hypothetical protein AM020 [Anaplasma marginale str. St. Maries]
>gi|56416423|ref|YP_153497.1| ORF X [Anaplasma marginale str. St. Maries]
>gi|56416449|ref|YP_153523.1| major surface protein 1B-2 [Anaplasma marginale str. St. Maries]
>gi|56416450|ref|YP_153524.1| major surface protein 1B [Anaplasma marginale str. St. Maries]
>gi|56416481|ref|YP_153555.1| ribosomal protein L9 [Anaplasma marginale str. St. Maries]
>gi|56416534|ref|YP_153608.1| hypothetical protein AM247 [Anaplasma marginale str. St. Maries]
>gi|56416566|ref|YP_153640.1| succinate dehydrogenase cytochrome b556 subunit [Anaplasma marginale str. St. Maries]
>gi|56416623|ref|YP_153697.1| hypothetical protein AM375 [Anaplasma marginale str. St. Maries]
>gi|56416688|ref|YP_153762.1| hypothetical protein AM470 [Anaplasma marginale str. St. Maries]
>gi|56416696|ref|YP_153770.1| hypothetical protein AM480 [Anaplasma marginale str. St. Maries]
>gi|56416726|ref|YP_153800.1| hypothetical protein AM520 [Anaplasma marginale str. St. Maries]
>gi|56416735|ref|YP_153809.1| major surface protein 1a [Anaplasma marginale str. St. Maries]
>gi|56416781|ref|YP_153855.1| hypothetical protein AM594 [Anaplasma marginale str. St. Maries]
>gi|56416792|ref|YP_153866.1| hypothetical protein AM615 [Anaplasma marginale str. St. Maries]
>gi|56416832|ref|YP_153906.1| hypothetical protein AM670 [Anaplasma marginale str. St. Maries]
>gi|56416834|ref|YP_153908.1| hypothetical protein AM673 [Anaplasma marginale str. St. Maries]
>gi|56416836|ref|YP_153910.1| hypothetical protein AM676 [Anaplasma marginale str. St. Maries]
>gi|56416839|ref|YP_153913.1| hypothetical protein AM681 [Anaplasma marginale str. St. Maries]
>gi|56416845|ref|YP_153919.1| hypothetical protein AM688 [Anaplasma marginale str. St. Maries]
>gi|56416856|ref|YP_153930.1| hypothetical protein AM709 [Anaplasma marginale str. St. Maries]
>gi|56416871|ref|YP_153945.1| hypothetical protein AM742 [Anaplasma marginale str. St. Maries]
>gi|56416877|ref|YP_153951.1| NADH dehydrogenase chain J [Anaplasma marginale str. St. Maries]
>gi|56416892|ref|YP_153966.1| hypothetical protein AM773 [Anaplasma marginale str. St. Maries]
>gi|56416902|ref|YP_153976.1| zinc ABC transporter, permease protein [Anaplasma marginale str. St. Maries]
>gi|56416909|ref|YP_153983.1| dihydrolipoamide dehydrogenase [Anaplasma marginale str. St. Maries]
>gi|56416971|ref|YP_154045.1| appendage-associated protein [Anaplasma marginale str. St. Maries]
>gi|56416972|ref|YP_154046.1| hypothetical protein AM879 [Anaplasma marginale str. St. Maries]
>gi|56416973|ref|YP_154047.1| hypothetical protein AM880 [Anaplasma marginale str. St. Maries]
>gi|56416992|ref|YP_154066.1| 50S ribosomal protein L24 [Anaplasma marginale str. St. Maries]
>gi|56417040|ref|YP_154114.1| hypothetical protein AM959 [Anaplasma marginale str. St. Maries]
>gi|56417095|ref|YP_154169.1| hypothetical protein AM1041 [Anaplasma marginale str. St. Maries]
>gi|56417103|ref|YP_154177.1| hypothetical protein AM1056 [Anaplasma marginale str. St. Maries]
>gi|56417108|ref|YP_154182.1| major surface protein 3 [Anaplasma marginale str. St. Maries]
>gi|56417144|ref|YP_154218.1| hypothetical protein AM1111 [Anaplasma marginale str. St. Maries]
>gi|56417170|ref|YP_154244.1| msp2 operon associated gene 1 [Anaplasma marginale str. St. Maries]
>gi|56417224|ref|YP_154298.1| hypothetical protein AM1226 [Anaplasma marginale str. St. Maries]
>gi|56417285|ref|YP_154359.1| VirD4 protein [Anaplasma marginale str. St. Maries]
>gi|16077130|ref|NP_387943.1| cell-division initiation protein [Bacillus subtilis subsp. subtilis str. 168]
>gi|16077168|ref|NP_387981.1| preprotein translocase subunit [Bacillus subtilis subsp. subtilis str. 168]
>gi|16077173|ref|NP_387986.1| ribosomal protein L12 (BL9) [Bacillus subtilis subsp. subtilis str. 168]
>gi|16077271|ref|NP_388084.1| hypothetical protein BSU02020 [Bacillus subtilis subsp. subtilis str. 168]
>gi|16077328|ref|NP_388141.1| hypothetical protein BSU02590 [Bacillus subtilis subsp. subtilis str. 168]
>gi|16077442|ref|NP_388256.1| hypothetical protein BSU03740 [Bacillus subtilis subsp. subtilis str. 168]
>gi|16077491|ref|NP_388305.1| hypothetical protein BSU04240 [Bacillus subtilis subsp. subtilis str. 168]
>gi|16077504|ref|NP_388318.1| hypothetical protein BSU04370 [Bacillus subtilis subsp. subtilis str. 168]
>gi|16077507|ref|NP_388321.1| general stress protein [Bacillus subtilis subsp. subtilis str. 168]
>gi|16077576|ref|NP_388390.1| hypothetical protein BSU05090 [Bacillus subtilis subsp. subtilis str. 168]
>gi|16077581|ref|NP_388395.1| hypothetical protein BSU05140 [Bacillus subtilis subsp. subtilis str. 168]
>gi|16077586|ref|NP_388400.1| hypothetical protein BSU05190 [Bacillus subtilis subsp. subtilis str. 168]

>gi|16077630|ref|NP_388444.1| nuclease inhibitor [Bacillus subtilis subsp. subtilis str. 168]
>gi|50812190|ref|NP_388483.2| class I heat-shock protein (chaperonin) [Bacillus subtilis subsp. subtilis str. 168]
>gi|16077688|ref|NP_388502.1| hypothetical protein BSU06210 [Bacillus subtilis subsp. subtilis str. 168]
>gi|50812196|ref|NP_388567.2| hypothetical protein BSU06850 [Bacillus subtilis subsp. subtilis str. 168]
>gi|16077818|ref|NP_388632.1| hypothetical protein BSU07510 [Bacillus subtilis subsp. subtilis str. 168]
>gi|16077834|ref|NP_388648.1| hypothetical protein BSU07670 [Bacillus subtilis subsp. subtilis str. 168]
>gi|16077879|ref|NP_388693.1| hypothetical protein BSU08120 [Bacillus subtilis subsp. subtilis str. 168]
>gi|16077884|ref|NP_388698.1| hypothetical protein BSU08170 [Bacillus subtilis subsp. subtilis str. 168]
>gi|16077897|ref|NP_388711.1| hypothetical protein BSU08300 [Bacillus subtilis subsp. subtilis str. 168]
>gi|16077932|ref|NP_388746.1| small acid-soluble spore protein (gamma-type SASP) [Bacillus subtilis subsp. subtilis str. 168]
>gi|16077979|ref|NP_388795.1| hypothetical protein BSU09140 [Bacillus subtilis subsp. subtilis str. 168]
>gi|16077980|ref|NP_388796.1| hypothetical protein BSU09150 [Bacillus subtilis subsp. subtilis str. 168]
>gi|16078002|ref|NP_388818.1| gamma-D-glutamate-meso-diaminopimelate muropeptidase (major autolysin) (CWBP49') [Bacillus subtilis subsp. subtilis str. 168]
>gi|16078007|ref|NP_388823.1| cell wall hydrolase; phosphatase-associated protein (major autolysin) (CWBP33) [Bacillus subtilis subsp. subtilis str. 168]
>gi|16078065|ref|NP_388882.1| hypothetical protein BSU10010 [Bacillus subtilis subsp. subtilis str. 168]
>gi|16078083|ref|NP_388900.1| hypothetical protein BSU10190 [Bacillus subtilis subsp. subtilis str. 168]
>gi|16078101|ref|NP_388918.1| hypothetical protein BSU10370 [Bacillus subtilis subsp. subtilis str. 168]
>gi|16078108|ref|NP_388925.1| hypothetical protein BSU10440 [Bacillus subtilis subsp. subtilis str. 168]
>gi|16078135|ref|NP_388952.1| spore germination protein [Bacillus subtilis subsp. subtilis str. 168]
>gi|16078238|ref|NP_389055.1| hypothetical protein BSU11730 [Bacillus subtilis subsp. subtilis str. 168]
>gi|16078241|ref|NP_389058.1| spore coat protein (insoluble fraction) [Bacillus subtilis subsp. subtilis str. 168]
>gi|16078242|ref|NP_389059.1| spore coat protein (insoluble fraction) [Bacillus subtilis subsp. subtilis str. 168]
>gi|16078243|ref|NP_389060.1| spore coat protein (insoluble fraction) [Bacillus subtilis subsp. subtilis str. 168]
>gi|16078248|ref|NP_389065.1| hypothetical protein BSU11830 [Bacillus subtilis subsp. subtilis str. 168]
>gi|16078274|ref|NP_389091.1| spore coat protein (inner) [Bacillus subtilis subsp. subtilis str. 168]
>gi|16078283|ref|NP_389100.1| hypothetical protein BSU12180 [Bacillus subtilis subsp. subtilis str. 168]
>gi|16078341|ref|NP_389159.1| hypothetical protein BSU12760 [Bacillus subtilis subsp. subtilis str. 168]
>gi|16078349|ref|NP_389167.1| low-affinity inorganic phosphate transporter [Bacillus subtilis subsp. subtilis str. 168]
>gi|16078353|ref|NP_389171.1| hypothetical protein BSU12880 [Bacillus subtilis subsp. subtilis str. 168]
>gi|16078355|ref|NP_389173.1| serine protease Do (heat-shock protein) [Bacillus subtilis subsp. subtilis str. 168]
>gi|16078410|ref|NP_389229.1| hypothetical protein BSU13460 [Bacillus subtilis subsp. subtilis str. 168]
>gi|16078415|ref|NP_389234.1| hypothetical protein BSU13510 [Bacillus subtilis subsp. subtilis str. 168]
>gi|16078432|ref|NP_389251.1| motility protein B [Bacillus subtilis subsp. subtilis str. 168]
>gi|16078482|ref|NP_389301.1| hypothetical protein BSU14180 [Bacillus subtilis subsp. subtilis str. 168]
>gi|16078542|ref|NP_389361.1| hypothetical protein BSU14780 [Bacillus subtilis subsp. subtilis str. 168]
>gi|16078655|ref|NP_389474.1| acyl carrier protein [Bacillus subtilis subsp. subtilis str. 168]
>gi|16078660|ref|NP_389479.1| hypothetical protein BSU15970 [Bacillus subtilis subsp. subtilis str. 168]
>gi|16078689|ref|NP_389508.1| hypothetical protein BSU16260 [Bacillus subtilis subsp. subtilis str. 168]
>gi|16078717|ref|NP_389536.1| phosphatidate cytidylyltransferase (CDP-diglyceride synthase) [Bacillus subtilis subsp. subtilis str. 168]
>gi|50812230|ref|NP_389573.2| hypothetical protein BSU16910 [Bacillus subtilis subsp. subtilis str. 168]
>gi|16078761|ref|NP_389580.1| required for dehydratation of the spore core and assembly of the coat (stage V sporulation) [Bacillus subtilis subsp. subtilis str. 168]
>gi|16078766|ref|NP_389585.1| morphogenic protein [Bacillus subtilis subsp. subtilis str. 168]
>gi|16078794|ref|NP_389613.1| hypothetical protein BSU17310 [Bacillus subtilis subsp. subtilis str. 168]
>gi|16078830|ref|NP_389650.1| hypothetical protein BSU17670 [Bacillus subtilis subsp. subtilis str. 168]
>gi|16078833|ref|NP_389653.1| spore coat protein (outer) [Bacillus subtilis subsp. subtilis str. 168]
>gi|16078854|ref|NP_389674.1| hypothetical protein BSU17910 [Bacillus subtilis subsp. subtilis str. 168]
>gi|16078868|ref|NP_389689.1| hypothetical protein BSU18070 [Bacillus subtilis subsp. subtilis str. 168]
>gi|16078981|ref|NP_389802.1| hypothetical protein BSU19210 [Bacillus subtilis subsp. subtilis str. 168]
>gi|16079009|ref|NP_389832.1| hypothetical protein BSU19510 [Bacillus subtilis subsp. subtilis str. 168]
>gi|16079019|ref|NP_389842.1| hypothetical protein BSU19610 [Bacillus subtilis subsp. subtilis str. 168]
>gi|16079055|ref|NP_389878.1| hypothetical protein BSU19970 [Bacillus subtilis subsp. subtilis str. 168]
>gi|16079058|ref|NP_389881.1| hypothetical protein BSU20000 [Bacillus subtilis subsp. subtilis str. 168]
>gi|16079074|ref|NP_389897.1| hypothetical protein BSU20150 [Bacillus subtilis subsp. subtilis str. 168]
>gi|16079101|ref|NP_389924.1| hypothetical protein BSU20420 [Bacillus subtilis subsp. subtilis str. 168]
>gi|16079118|ref|NP_389941.1| hypothetical protein BSU20590 [Bacillus subtilis subsp. subtilis str. 168]
>gi|16079154|ref|NP_389978.1| hypothetical protein BSU20950 [Bacillus subtilis subsp. subtilis str. 168]
>gi|16079159|ref|NP_389983.1| hypothetical protein BSU21000 [Bacillus subtilis subsp. subtilis str. 168]
>gi|16079200|ref|NP_390024.1| N-acetylmuramoyl-L-alanine amidase [Bacillus subtilis subsp. subtilis str. 168]
>gi|16079220|ref|NP_390044.1| hypothetical protein BSU21610 [Bacillus subtilis subsp. subtilis str. 168]
>gi|16079283|ref|NP_390107.1| hypothetical protein BSU22250 [Bacillus subtilis subsp. subtilis str. 168]
>gi|16079357|ref|NP_390181.1| hypothetical protein BSU23000 [Bacillus subtilis subsp. subtilis str. 168]
>gi|16079379|ref|NP_390203.1| hypothetical protein BSU23220 [Bacillus subtilis subsp. subtilis str. 168]
>gi|16079491|ref|NP_390315.1| acetyl-CoA carboxylase subunit (biotin carboxyl carrier subunit) [Bacillus subtilis subsp. subtilis str. 168]
>gi|16079492|ref|NP_390316.1| mutants block sporulation after engulfment (stage III sporulation) [Bacillus subtilis subsp. subtilis str. 168]
>gi|16079542|ref|NP_390366.1| hypothetical protein BSU24860 [Bacillus subtilis subsp. subtilis str. 168]
>gi|16079570|ref|NP_390394.1| hypothetical protein BSU25150 [Bacillus subtilis subsp. subtilis str. 168]
>gi|16079591|ref|NP_390415.1| hypothetical protein BSU25370 [Bacillus subtilis subsp. subtilis str. 168]

>gi|16079593|ref|NP_390417.1| hypothetical protein BSU25390 [Bacillus subtilis subsp. subtilis str. 168]
>gi|16079594|ref|NP_390418.1| hypothetical protein BSU25400 [Bacillus subtilis subsp. subtilis str. 168]
>gi|16079595|ref|NP_390419.1| ribosomal protein S21 [Bacillus subtilis subsp. subtilis str. 168]
>gi|16079600|ref|NP_390424.1| heat-shock protein [Bacillus subtilis subsp. subtilis str. 168]
>gi|16079602|ref|NP_390426.1| heat-shock protein [Bacillus subtilis subsp. subtilis str. 168]
>gi|16079609|ref|NP_390433.1| ribosomal protein S20 (BS20) [Bacillus subtilis subsp. subtilis str. 168]
>gi|16079668|ref|NP_390492.1| hypothetical protein BSU26150 [Bacillus subtilis subsp. subtilis str. 168]
>gi|16079689|ref|NP_390513.1| hypothetical protein BSU26360 [Bacillus subtilis subsp. subtilis str. 168]
>gi|16079736|ref|NP_390560.1| hypothetical protein BSU26830 [Bacillus subtilis subsp. subtilis str. 168]
>gi|16079784|ref|NP_390608.1| hypothetical protein BSU27300 [Bacillus subtilis subsp. subtilis str. 168]
>gi|16079822|ref|NP_390647.1| hypothetical protein BSU27690 [Bacillus subtilis subsp. subtilis str. 168]
>gi|16079835|ref|NP_390661.1| spore cortex protein [Bacillus subtilis subsp. subtilis str. 168]
>gi|16079836|ref|NP_390662.1| morphogenetic protein associated with SpoVID [Bacillus subtilis subsp. subtilis str. 168]
>gi|16079858|ref|NP_390684.1| spatial and temporal regulation of the dissolution of septal peptidoglycan during engulfment (stage II sporulation) [Bacillus subtilis subsp. subtilis str. 168]
>gi|16079863|ref|NP_390689.1| required for assembly of the spore coat (stage VI sporulation) [Bacillus subtilis subsp. subtilis str. 168]
>gi|16079884|ref|NP_390710.1| hypothetical protein BSU28320 [Bacillus subtilis subsp. subtilis str. 168]
>gi|50812278|ref|NP_390712.2| hypothetical protein BSU28340 [Bacillus subtilis subsp. subtilis str. 168]
>gi|16079941|ref|NP_390767.1| hypothetical protein BSU28890 [Bacillus subtilis subsp. subtilis str. 168]
>gi|16080088|ref|NP_390914.1| hypothetical protein BSU30360 [Bacillus subtilis subsp. subtilis str. 168]
>gi|16080104|ref|NP_390930.1| hypothetical protein BSU30520 [Bacillus subtilis subsp. subtilis str. 168]
>gi|16080114|ref|NP_390940.1| hypothetical protein BSU30620 [Bacillus subtilis subsp. subtilis str. 168]
>gi|16080141|ref|NP_390967.1| hypothetical protein BSU30890 [Bacillus subtilis subsp. subtilis str. 168]
>gi|16080153|ref|NP_390979.1| hypothetical protein BSU31010 [Bacillus subtilis subsp. subtilis str. 168]
>gi|16080154|ref|NP_390980.1| hypothetical protein BSU31020 [Bacillus subtilis subsp. subtilis str. 168]
>gi|16080168|ref|NP_390994.1| hypothetical protein BSU31160 [Bacillus subtilis subsp. subtilis str. 168]
>gi|16080259|ref|NP_391086.1| hypothetical protein BSU32060 [Bacillus subtilis subsp. subtilis str. 168]
>gi|50812290|ref|YP_054592.1| small acid-soluble spore protein [Bacillus subtilis subsp. subtilis str. 168]
>gi|16080413|ref|NP_391240.1| tmRNA-binding protein [Bacillus subtilis subsp. subtilis str. 168]
>gi|16080430|ref|NP_391257.1| hypothetical protein BSU33770 [Bacillus subtilis subsp. subtilis str. 168]
>gi|16080477|ref|NP_391304.1| hypothetical protein BSU34240 [Bacillus subtilis subsp. subtilis str. 168]
>gi|16080480|ref|NP_391307.1| hypothetical protein BSU34270 [Bacillus subtilis subsp. subtilis str. 168]
>gi|16080533|ref|NP_391360.1| hypothetical protein BSU34800 [Bacillus subtilis subsp. subtilis str. 168]
>gi|16080563|ref|NP_391390.1| hypothetical protein BSU35100 [Bacillus subtilis subsp. subtilis str. 168]
>gi|16080566|ref|NP_391393.1| hypothetical protein BSU35130 [Bacillus subtilis subsp. subtilis str. 168]
>gi|16080605|ref|NP_391432.1| hypothetical protein BSU35520 [Bacillus subtilis subsp. subtilis str. 168]
>gi|16080642|ref|NP_391470.1| hypothetical protein BSU35890 [Bacillus subtilis subsp. subtilis str. 168]
>gi|16080650|ref|NP_391478.1| hypothetical protein BSU35970 [Bacillus subtilis subsp. subtilis str. 168]
>gi|16080658|ref|NP_391486.1| spore coat protein (outer) [Bacillus subtilis subsp. subtilis str. 168]
>gi|16080660|ref|NP_391488.1| morphogenetic protein [Bacillus subtilis subsp. subtilis str. 168]
>gi|16080707|ref|NP_391535.1| hypothetical protein BSU36540 [Bacillus subtilis subsp. subtilis str. 168]
>gi|16080708|ref|NP_391536.1| required for completion of engulfment [Bacillus subtilis subsp. subtilis str. 168]
>gi|16080711|ref|NP_391539.1| hypothetical protein BSU36580 [Bacillus subtilis subsp. subtilis str. 168]
>gi|16080720|ref|NP_391548.1| sigma-B-controlled gene [Bacillus subtilis subsp. subtilis str. 168]
>gi|16080725|ref|NP_391553.1| hypothetical protein BSU36720 [Bacillus subtilis subsp. subtilis str. 168]
>gi|16080747|ref|NP_391575.1| hypothetical protein BSU36940 [Bacillus subtilis subsp. subtilis str. 168]
>gi|16080769|ref|NP_391597.1| RNA polymerase (delta subunit) [Bacillus subtilis subsp. subtilis str. 168]
>gi|16080843|ref|NP_391671.1| hypothetical protein BSU37920 [Bacillus subtilis subsp. subtilis str. 168]
>gi|16080858|ref|NP_391686.1| transcriptional antiterminator [Bacillus subtilis subsp. subtilis str. 168]
>gi|16080883|ref|NP_391711.1| hypothetical protein BSU38320 [Bacillus subtilis subsp. subtilis str. 168]
>gi|16080930|ref|NP_391758.1| hypothetical protein BSU38790 [Bacillus subtilis subsp. subtilis str. 168]
>gi|16080936|ref|NP_391764.1| hypothetical protein BSU38850 [Bacillus subtilis subsp. subtilis str. 168]
>gi|16081009|ref|NP_391837.1| hypothetical protein BSU39580 [Bacillus subtilis subsp. subtilis str. 168]
>gi|16081010|ref|NP_391838.1| hypothetical protein BSU39590 [Bacillus subtilis subsp. subtilis str. 168]
>gi|16081030|ref|NP_391858.1| hypothetical protein BSU39790 [Bacillus subtilis subsp. subtilis str. 168]
>gi|16081067|ref|NP_391895.1| hypothetical protein BSU40150 [Bacillus subtilis subsp. subtilis str. 168]
>gi|16081078|ref|NP_391906.1| hypothetical protein BSU40260 [Bacillus subtilis subsp. subtilis str. 168]
>gi|16081101|ref|NP_391929.1| hypothetical protein BSU40490 [Bacillus subtilis subsp. subtilis str. 168]
>gi|16081125|ref|NP_391953.1| hypothetical protein BSU40730 [Bacillus subtilis subsp. subtilis str. 168]
>gi|16081137|ref|NP_391965.1| maltose O-acetyltransferase [Bacillus subtilis subsp. subtilis str. 168]
>gi|16081142|ref|NP_391970.1| single-strand DNA-binding protein [Bacillus subtilis subsp. subtilis str. 168]
>gi|15607146|ref|NP_214518.1| hypothetical protein Rv0004 [Mycobacterium tuberculosis H37Rv]
>gi|15607149|ref|NP_214521.1| POSSIBLE CONSERVED MEMBRANE PROTEIN [Mycobacterium tuberculosis H37Rv]
>gi|15607150|ref|NP_214522.1| POSSIBLE MEMBRANE PROTEIN [Mycobacterium tuberculosis H37Rv]
>gi|15607162|ref|NP_214534.1| hypothetical protein Rv0020c [Mycobacterium tuberculosis H37Rv]
>gi|15607166|ref|NP_214538.1| PUTATIVE SECRETED PROTEIN P60-RELATED PROTEIN [Mycobacterium tuberculosis H37Rv]
>gi|15607167|ref|NP_214539.1| hypothetical protein Rv0025 [Mycobacterium tuberculosis H37Rv]
>gi|15607179|ref|NP_214551.1| PROBABLE CONSERVED INTEGRAL MEMBRANE PROTEIN [Mycobacterium tuberculosis H37Rv]
>gi|15607181|ref|NP_214553.1| POSSIBLE CONSERVED TRANSMEMBRANE PROTEIN [Mycobacterium tuberculosis H37Rv]

>gi|57116684|ref|NP_214554.2| SECRETED PROLINE RICH PROTEIN MTC28 (PROLINE RICH 28 KDA ANTIGEN) [Mycobacterium tuberculosis H37Rv]
>gi|15607193|ref|NP_214565.1| PROBABLE CONSERVED TRANSMEMBRANE PROTEIN [Mycobacterium tuberculosis H37Rv]
>gi|15607214|ref|NP_214586.1| PROBABLE GLUTAMINE-TRANSPORT TRANSMEMBRANE PROTEIN ABC TRANSPORTER [Mycobacterium tuberculosis H37Rv]
>gi|15607225|ref|NP_214597.1| PROBABLE OXIDOREDUCTASE [Mycobacterium tuberculosis H37Rv]
>gi|15607226|ref|NP_214598.1| POSSIBLE FORMATE HYDROGENLYASE HYCD (FHL) [Mycobacterium tuberculosis H37Rv]
>gi|15607227|ref|NP_214599.1| POSSIBLE HYDROGENASE HYCP [Mycobacterium tuberculosis H37Rv]
>gi|15607228|ref|NP_214600.1| POSSIBLE HYDROGENASE HYCQ [Mycobacterium tuberculosis H37Rv]
>gi|15607234|ref|NP_214606.1| PROBABLE CATION TRANSPORTER P-TYPE ATPASE A CTPA [Mycobacterium tuberculosis H37Rv]
>gi|57116689|ref|YP_177690.1| PPE FAMILY PROTEIN [Mycobacterium tuberculosis H37Rv]
>gi|57116691|ref|YP_177692.1| PE-PGRS FAMILY PROTEIN [Mycobacterium tuberculosis H37Rv]
>gi|57116692|ref|YP_177693.1| PE-PGRS FAMILY PROTEIN [Mycobacterium tuberculosis H37Rv]
>gi|57116694|ref|YP_177695.1| PE FAMILY PROTEIN [Mycobacterium tuberculosis H37Rv]
>gi|15607297|ref|NP_214669.1| PROBABLE NAD(P) TRANSHYDROGENASE (SUBUNIT ALPHA) PNTAA [FIRST PART; CATALYTIC PART] (PYRIDINE NUCLEOTIDE TRANSHYDROGENASE SUBUNIT ALPHA) (NICOTINAMIDE NUCLEOTIDE TRANSHYDROGENASE SUBUNIT ALPHA) [Mycobacterium tuberculosis H37Rv]
>gi|15607299|ref|NP_214671.1| PROBABLE NAD(P) TRANSHYDROGENASE (SUBUNIT BETA) PNTB [INTEGRAL MEMBRANE PROTEIN] (PYRIDINE NUCLEOTIDE TRANSHYDROGENASE SUBUNIT BETA) (NICOTINAMIDE NUCLEOTIDE TRANSHYDROGENASE SUBUNIT BETA) [Mycobacterium tuberculosis H37Rv]
>gi|57116696|ref|YP_177697.1| PE FAMILY PROTEIN [Mycobacterium tuberculosis H37Rv]
>gi|15607308|ref|NP_214681.1| CONSERVED HYPOTHETICAL INTEGRAL MEMBRANE PROTEIN YRBE1A [Mycobacterium tuberculosis H37Rv]
>gi|57116701|ref|YP_177701.1| MCE-FAMILY PROTEIN MCE1A [Mycobacterium tuberculosis H37Rv]
>gi|15607312|ref|NP_214685.1| MCE-FAMILY PROTEIN MCE1C [Mycobacterium tuberculosis H37Rv]
>gi|15607319|ref|NP_214692.1| PROBABLE CONSERVED MCE ASSOCIATED MEMBRANE PROTEIN [Mycobacterium tuberculosis H37Rv]
>gi|15607328|ref|NP_214701.1| PROBABLE O-METHYLTRANSFERASE [Mycobacterium tuberculosis H37Rv]
>gi|15607332|ref|NP_214705.1| PROBABLE CONSERVED INTEGRAL MEMBRANE PROTEIN [Mycobacterium tuberculosis H37Rv]
>gi|15607333|ref|NP_214706.1| hypothetical protein Rv0192 [Mycobacterium tuberculosis H37Rv]
>gi|57116703|ref|YP_177618.1| CONSERVED SECRETED PROTEIN [Mycobacterium tuberculosis H37Rv]
>gi|15607344|ref|NP_214717.1| POSSIBLE EXPORTED PROTEIN [Mycobacterium tuberculosis H37Rv]
>gi|15607345|ref|NP_214718.1| PROBABLE CONSERVED TRANSMEMBRANE PROTEIN [Mycobacterium tuberculosis H37Rv]
>gi|15607346|ref|NP_214719.1| PROBABLE CONSERVED TRANSMEMBRANE PROTEIN [Mycobacterium tuberculosis H37Rv]
>gi|15607350|ref|NP_214723.1| hypothetical protein Rv0209 [Mycobacterium tuberculosis H37Rv]
>gi|15607351|ref|NP_214724.1| hypothetical protein Rv0210 [Mycobacterium tuberculosis H37Rv]
>gi|15607358|ref|NP_214731.1| POSSIBLE ESTERASE LIPW [Mycobacterium tuberculosis H37Rv]
>gi|15607367|ref|NP_214740.1| PROBABLE CONSERVED TRANSMEMBRANE PROTEIN [Mycobacterium tuberculosis H37Rv]
>gi|15607370|ref|NP_214743.1| POSSIBLE CONSERVED MEMBRANE PROTEIN [Mycobacterium tuberculosis H37Rv]
>gi|57116708|ref|YP_177704.1| PPE FAMILY PROTEIN [Mycobacterium tuberculosis H37Rv]
>gi|15607409|ref|NP_214782.1| hypothetical protein Rv0268c [Mycobacterium tuberculosis H37Rv]
>gi|57116712|ref|YP_177707.1| PE-PGRS FAMILY PROTEIN [Mycobacterium tuberculosis H37Rv]
>gi|57116713|ref|YP_177708.1| PE-PGRS FAMILY PROTEIN [Mycobacterium tuberculosis H37Rv]
>gi|57116714|ref|YP_177709.1| PPE FAMILY PROTEIN [Mycobacterium tuberculosis H37Rv]
>gi|57116715|ref|YP_177710.1| PE FAMILY PROTEIN [Mycobacterium tuberculosis H37Rv]
>gi|57116716|ref|YP_177711.1| PPE FAMILY PROTEIN [Mycobacterium tuberculosis H37Rv]
>gi|15607428|ref|NP_214801.1| ESAT-6 LIKE PROTEIN ESXG (CONSERVED HYPOTHETICAL PROTEIN TB9.8) [Mycobacterium tuberculosis H37Rv]
>gi|15607431|ref|NP_214804.1| PROBABLE CONSERVED TRANSMEMBRANE PROTEIN [Mycobacterium tuberculosis H37Rv]
>gi|57116718|ref|YP_177713.1| PE-PGRS FAMILY PROTEIN [Mycobacterium tuberculosis H37Rv]
>gi|15607441|ref|NP_214814.1| hypothetical protein Rv0300 [Mycobacterium tuberculosis H37Rv]
>gi|57116719|ref|YP_177714.1| PPE FAMILY PROTEIN [Mycobacterium tuberculosis H37Rv]
>gi|57116720|ref|YP_177715.1| PPE FAMILY PROTEIN [Mycobacterium tuberculosis H37Rv]
>gi|15607449|ref|NP_214822.1| PROBABLE CONSERVED INTEGRAL MEMBRANE PROTEIN [Mycobacterium tuberculosis H37Rv]
>gi|15607453|ref|NP_214826.1| CONSERVED HYPOTHETICAL PROLINE AND THREONINE RICH PROTEIN [Mycobacterium tuberculosis H37Rv]
>gi|15607455|ref|NP_214828.1| POSSIBLE CONSERVED MEMBRANE PROTEIN [Mycobacterium tuberculosis H37Rv]
>gi|15607460|ref|NP_214833.1| PROBABLE PYRROLIDONE-CARBOXYLATE PEPTIDASE PCP (5-OXOPROLYL-PEPTIDASE) (PYROGLUTAMYL-PEPTIDASE I) (PGP-I) (PYRASE) [Mycobacterium tuberculosis H37Rv]
>gi|15607461|ref|NP_214834.1| POSSIBLE CONSERVED EXPORTED PROTEIN [Mycobacterium tuberculosis H37Rv]
>gi|15607471|ref|NP_214844.1| hypothetical protein Rv0330c [Mycobacterium tuberculosis H37Rv]
>gi|57116722|ref|YP_177717.1| PE FAMILY PROTEIN [Mycobacterium tuberculosis H37Rv]
>gi|15607479|ref|NP_214852.1| PROBABLE IRON-SULFUR-BINDING REDUCTASE [Mycobacterium tuberculosis H37Rv]
>gi|15607480|ref|NP_214853.1| POSSIBLE TRANSCRIPTIONAL REGULATORY PROTEIN [Mycobacterium tuberculosis H37Rv]
>gi|15607482|ref|NP_214855.1| ISONIAZID INDUCTIBLE GENE PROTEIN INIB [Mycobacterium tuberculosis H37Rv]
>gi|15607485|ref|NP_214858.1| PROBABLE LIPOPROTEIN LPQJ [Mycobacterium tuberculosis H37Rv]
>gi|57116724|ref|YP_177719.1| PROBABLE CHAPERONE PROTEIN DNAJ1 [Mycobacterium tuberculosis H37Rv]
>gi|57116725|ref|YP_177720.1| PPE FAMILY PROTEIN [Mycobacterium tuberculosis H37Rv]
>gi|57116726|ref|YP_177721.1| PPE FAMILY PROTEIN [Mycobacterium tuberculosis H37Rv]
>gi|15607499|ref|NP_214872.1| hypothetical protein Rv0358 [Mycobacterium tuberculosis H37Rv]
>gi|15607519|ref|NP_214892.1| CONSERVED HYPOTHETICAL GLYCINE RICH PROTEIN [Mycobacterium tuberculosis H37Rv]

>gi|15607524|ref|NP_214897.1| POSSIBLE CONSERVED SECRETED PROTEIN [Mycobacterium tuberculosis H37Rv]
>gi|15607527|ref|NP_214900.1| PROBABLE TRANSCRIPTIONAL REGULATORY PROTEIN (PROBABLY LUXR/UHPA-FAMILY) [Mycobacterium tuberculosis H37Rv]
>gi|15607528|ref|NP_214901.1| hypothetical protein Rv0387c [Mycobacterium tuberculosis H37Rv]
>gi|57116729|ref|YP_177724.1| PPE FAMILY PROTEIN [Mycobacterium tuberculosis H37Rv]
>gi|15607534|ref|NP_214907.1| CONSERVED 13E12 REPEAT FAMILY PROTEIN [Mycobacterium tuberculosis H37Rv]
>gi|15607538|ref|NP_214911.1| CONSERVED 13E12 REPEAT FAMILY PROTEIN [Mycobacterium tuberculosis H37Rv]
>gi|15607542|ref|NP_214915.1| PROBABLE CONSERVED TRANSMEMBRANE PROTEIN [Mycobacterium tuberculosis H37Rv]
>gi|15607558|ref|NP_214931.1| PROBABLE THIAMIN BIOSYNTHESIS PROTEIN THIG (THIAZOLE BIOSYNTHESIS PROTEIN) [Mycobacterium tuberculosis H37Rv]
>gi|15607566|ref|NP_214939.1| POSSIBLE METAL CATION TRANSPORTING P-TYPE ATPASE CTPH [Mycobacterium tuberculosis H37Rv]
>gi|15607567|ref|NP_214940.1| POSSIBLE TRANSMEMBRANE PROTEIN [Mycobacterium tuberculosis H37Rv]
>gi|57116731|ref|YP_177726.1| PPE FAMILY PROTEIN [Mycobacterium tuberculosis H37Rv]
>gi|15607585|ref|NP_214958.1| hypothetical protein Rv0444c [Mycobacterium tuberculosis H37Rv]
>gi|15607593|ref|NP_214966.1| POSSIBLE TRANSCRIPTIONAL REGULATORY PROTEIN [Mycobacterium tuberculosis H37Rv]
>gi|57116732|ref|YP_177727.1| PPE FAMILY PROTEIN [Mycobacterium tuberculosis H37Rv]
>gi|15607612|ref|NP_214985.1| hypothetical protein Rv0471c [Mycobacterium tuberculosis H37Rv]
>gi|15607616|ref|NP_214989.1| IRON-REGULATED HEPARIN BINDING HEMAGGLUTININ HBHA (ADHESIN) [Mycobacterium tuberculosis H37Rv]
>gi|15607619|ref|NP_214992.1| PROBABLE DEOXYRIBOSE-PHOSPHATE ALDOLASE DEOC (PHOSPHODEOXYRIBOALDOLASE) (DEOXYRIBOALDOLASE) [Mycobacterium tuberculosis H37Rv]
>gi|15607638|ref|NP_215011.1| PROBABLE CONSERVED TRANSMEMBRANE PROTEIN [Mycobacterium tuberculosis H37Rv]
>gi|15607641|ref|NP_215014.1| PROBABLE PYRROLINE-5-CARBOXYLATE REDUCTASE PROC (P5CR) (P5C REDUCTASE) [Mycobacterium tuberculosis H37Rv]
>gi|15607650|ref|NP_215023.1| PROBABLE GLUTAMYL-TRNA REDUCTASE HEMA (GLUTR) [Mycobacterium tuberculosis H37Rv]
>gi|15607653|ref|NP_215026.1| PROBABLE DELTA-AMINOLEVULINIC ACID DEHYDRATASE HEMB (PORPHOBILINOGEN SYNTHASE) (ALAD) (ALADH) [Mycobacterium tuberculosis H37Rv]
>gi|15607660|ref|NP_215033.1| POSSIBLE CONSERVED MEMBRANE PROTEIN [Mycobacterium tuberculosis H37Rv]
>gi|57116750|ref|YP_177735.1| POSSIBLE CYTOCHROME C-TYPE BIOGENESIS PROTEIN CCDA [Mycobacterium tuberculosis H37Rv]
>gi|15607671|ref|NP_215045.1| POSSIBLE CONSERVED MEMBRANE PROTEIN [Mycobacterium tuberculosis H37Rv]
>gi|57116752|ref|YP_177736.1| PE-PGRS FAMILY PROTEIN [Mycobacterium tuberculosis H37Rv]
>gi|15607678|ref|NP_215052.1| POSSIBLE CONSERVED MEMBRANE PROTEIN [Mycobacterium tuberculosis H37Rv]
>gi|15607680|ref|NP_215054.1| hypothetical protein Rv0540 [Mycobacterium tuberculosis H37Rv]
>gi|15607681|ref|NP_215055.1| PROBABLE CONSERVED INTEGRAL MEMBRANE PROTEIN [Mycobacterium tuberculosis H37Rv]
>gi|15607690|ref|NP_215064.1| hypothetical protein Rv0550c [Mycobacterium tuberculosis H37Rv]
>gi|15607693|ref|NP_215067.1| PROBABLE MUCONATE CYCLOISOMERASE MENC (CIS,CIS-MUCONATE LACTONIZING ENZYME) (MLE) [Mycobacterium tuberculosis H37Rv]
>gi|57116755|ref|YP_177739.1| PE-PGRS FAMILY PROTEIN [Mycobacterium tuberculosis H37Rv]
>gi|15607721|ref|NP_215095.1| hypothetical protein Rv0581 [Mycobacterium tuberculosis H37Rv]
>gi|15607723|ref|NP_215097.1| PROBABLE CONSERVED LIPOPROTEIN LPQN [Mycobacterium tuberculosis H37Rv]
>gi|15607727|ref|NP_215101.1| CONSERVED HYPOTHETICAL INTEGRAL MEMBRANE PROTEIN YRBE2A [Mycobacterium tuberculosis H37Rv]
>gi|15607728|ref|NP_215102.1| CONSERVED HYPOTHETICAL INTEGRAL MEMBRANE PROTEIN YRBE2B [Mycobacterium tuberculosis H37Rv]
>gi|15607731|ref|NP_215105.1| MCE-FAMILY PROTEIN MCE2C [Mycobacterium tuberculosis H37Rv]
>gi|15607738|ref|NP_215112.1| hypothetical protein Rv0598c [Mycobacterium tuberculosis H37Rv]
>gi|15607743|ref|NP_215117.1| POSSIBLE EXPORTED PROTEIN [Mycobacterium tuberculosis H37Rv]
>gi|15607754|ref|NP_215128.1| hypothetical protein Rv0614 [Mycobacterium tuberculosis H37Rv]
>gi|15607755|ref|NP_215129.1| PROBABLE INTEGRAL MEMBRANE PROTEIN [Mycobacterium tuberculosis H37Rv]
>gi|15607763|ref|NP_215137.1| hypothetical protein Rv0623 [Mycobacterium tuberculosis H37Rv]
>gi|15607765|ref|NP_215139.1| PROBABLE CONSERVED TRANSMEMBRANE PROTEIN [Mycobacterium tuberculosis H37Rv]
>gi|15607774|ref|NP_215148.1| POSSIBLE GLYOXALASE II (HYDROXYACYLGLUTATHIONE HYDROLASE) (GLX II) [Mycobacterium tuberculosis H37Rv]
>gi|57116765|ref|YP_177743.1| PROBABLE PREPROTEIN TRANSLOCASE SECE1 [Mycobacterium tuberculosis H37Rv]
>gi|57116766|ref|YP_177744.1| POSSIBLE MALONYL COA-ACYL CARRIER PROTEIN TRANSACYLASE FABD2 (MCT) [Mycobacterium tuberculosis H37Rv]
>gi|15607790|ref|NP_215164.1| POSSIBLE SUGAR KINASE [Mycobacterium tuberculosis H37Rv]
>gi|15607792|ref|NP_215166.1| PROBABLE 50S RIBOSOMAL PROTEIN L7/L12 RPLL (SA1) [Mycobacterium tuberculosis H37Rv]
>gi|15607798|ref|NP_215172.1| PROBABLE CONSERVED INTEGRAL MEMBRANE PROTEIN [Mycobacterium tuberculosis H37Rv]
>gi|15607799|ref|NP_215173.1| hypothetical protein Rv0659c [Mycobacterium tuberculosis H37Rv]
>gi|15607800|ref|NP_215174.1| hypothetical protein Rv0660c [Mycobacterium tuberculosis H37Rv]
>gi|15607804|ref|NP_215178.1| hypothetical protein Rv0664 [Mycobacterium tuberculosis H37Rv]
>gi|57116767|ref|YP_177745.1| PROBABLE ENOYL-CoA HYDRATASE ECHA5 (ENOYL HYDRASE) (UNSATURATED ACYL-CoA HYDRATASE) (CROTONASE) [Mycobacterium tuberculosis H37Rv]
>gi|15607819|ref|NP_215193.1| CONSERVED HYPOTHETICAL THREONINE RICH PROTEIN [Mycobacterium tuberculosis H37Rv]
>gi|15607826|ref|NP_215200.1| PROBABLE MEMBRANE PROTEIN [Mycobacterium tuberculosis H37Rv]
>gi|15607846|ref|NP_215220.1| PROBABLE 50S RIBOSOMAL PROTEIN L22 RPLV [Mycobacterium tuberculosis H37Rv]
>gi|15607850|ref|NP_215224.1| PROBABLE 30S RIBOSOMAL PROTEIN S17 RPSQ [Mycobacterium tuberculosis H37Rv]
>gi|15607870|ref|NP_215244.1| hypothetical protein Rv0730 [Mycobacterium tuberculosis H37Rv]
>gi|57116772|ref|YP_177749.1| PE-PGRS FAMILY PROTEIN [Mycobacterium tuberculosis H37Rv]

>gi|57116773|ref|YP_177750.1| PE-PGRS FAMILY PROTEIN [Mycobacterium tuberculosis H37Rv]
>gi|57116774|ref|YP_177751.1| PE-PGRS FAMILY PROTEIN [Mycobacterium tuberculosis H37Rv]
>gi|15607891|ref|NP_215265.1| PROBABLE 3-HYDROXYISOBUTYRATE DEHYDROGENASE MMSB (HIBADH) [Mycobacterium tuberculosis H37Rv]
>gi|57116776|ref|YP_177752.1| PE-PGRS FAMILY PROTEIN [Mycobacterium tuberculosis H37Rv]
>gi|57116777|ref|YP_177753.1| PPE FAMILY PROTEIN [Mycobacterium tuberculosis H37Rv]
>gi|15607896|ref|NP_215270.1| hypothetical protein Rv0756c [Mycobacterium tuberculosis H37Rv]
>gi|15607909|ref|NP_215283.1| PROBABLE DEHYDROGENASE/REDUCTASE [Mycobacterium tuberculosis H37Rv]
>gi|57116780|ref|NP_215288.2| PROBABLE CONSERVED EXPORTED PROTEIN [Mycobacterium tuberculosis H37Rv]
>gi|15607919|ref|NP_215293.1| POSSIBLE CONSERVED TRANSMEMBRANE PROTEIN [Mycobacterium tuberculosis H37Rv]
>gi|15607926|ref|NP_215300.1| hypothetical protein Rv0786c [Mycobacterium tuberculosis H37Rv]
>gi|15607944|ref|NP_215319.1| hypothetical protein Rv0804 [Mycobacterium tuberculosis H37Rv]
>gi|15607950|ref|NP_215325.1| hypothetical protein Rv0810c [Mycobacterium tuberculosis H37Rv]
>gi|57116787|ref|YP_177759.1| PE-PGRS FAMILY PROTEIN [Mycobacterium tuberculosis H37Rv]
>gi|57116788|ref|YP_177760.1| PE-PGRS FAMILY PROTEIN [Mycobacterium tuberculosis H37Rv]
>gi|57116789|ref|YP_177761.1| PE-PGRS FAMILY PROTEIN [Mycobacterium tuberculosis H37Rv]
>gi|15607982|ref|NP_215357.1| PROBABLE CONSERVED INTEGRAL MEMBRANE PROTEIN [Mycobacterium tuberculosis H37Rv]
>gi|15607987|ref|NP_215362.1| PROBABLE LIPOPROTEIN LPQS [Mycobacterium tuberculosis H37Rv]
>gi|15607989|ref|NP_215364.1| PROBABLE CONSERVED INTEGRAL MEMBRANE TRANSPORT PROTEIN [Mycobacterium tuberculosis H37Rv]
>gi|15608006|ref|NP_215381.1| PROBABLE MOLYBDENUM COFACTOR BIOSYNTHESIS PROTEIN E2 MOAE2 (MOLYBDOPTERIN CONVERTING FACTOR LARGE SUBUNIT) (MOLYBDOPTERIN [MPT] CONVERTING FACTOR, SUBUNIT 2) [Mycobacterium tuberculosis H37Rv]
>gi|15608007|ref|NP_215382.1| POSSIBLE RESUSCITATION-PROMOTING FACTOR RPFA [Mycobacterium tuberculosis H37Rv]
>gi|15608010|ref|NP_215385.1| POSSIBLE CONSERVED INTEGRAL MEMBRANE PROTEIN [Mycobacterium tuberculosis H37Rv]
>gi|57116793|ref|YP_177763.1| PE-PGRS FAMILY PROTEIN [Mycobacterium tuberculosis H37Rv]
>gi|15608016|ref|NP_215391.1| POSSIBLE CONSERVED TRANSMEMBRANE PROTEIN [Mycobacterium tuberculosis H37Rv]
>gi|57116794|ref|YP_177764.1| PPE FAMILY PROTEIN [Mycobacterium tuberculosis H37Rv]
>gi|15608019|ref|NP_215394.1| POSSIBLE CONSERVED TRANSMEMBRANE PROTEIN [Mycobacterium tuberculosis H37Rv]
>gi|15608022|ref|NP_215397.1| PROBABLE TRANSMEMBRANE PROTEIN [Mycobacterium tuberculosis H37Rv]
>gi|15608038|ref|NP_215413.1| hypothetical protein Rv0898c [Mycobacterium tuberculosis H37Rv]
>gi|15608041|ref|NP_215416.1| POSSIBLE CONSERVED EXPORTED OR MEMBRANE PROTEIN [Mycobacterium tuberculosis H37Rv]
>gi|15608044|ref|NP_215419.1| PUTATIVE ACETYL-COENZYME A CARBOXYLASE CARBOXYL TRANSFERASE (SUBUNIT BETA) ACCD3 (ACCASE BETA CHAIN) [Mycobacterium tuberculosis H37Rv]
>gi|57116795|ref|YP_177765.1| PPE FAMILY PROTEIN [Mycobacterium tuberculosis H37Rv]
>gi|57116796|ref|YP_177766.1| PE FAMILY PROTEIN [Mycobacterium tuberculosis H37Rv]
>gi|57116802|ref|YP_177771.1| PHOSPHATE-TRANSPORT INTEGRAL MEMBRANE ABC TRANSPORTER PSTC1 [Mycobacterium tuberculosis H37Rv]
>gi|15608090|ref|NP_215465.1| hypothetical protein Rv0950c [Mycobacterium tuberculosis H37Rv]
>gi|15608094|ref|NP_215469.1| PROBABLE CONSERVED TRANSMEMBRANE PROTEIN [Mycobacterium tuberculosis H37Rv]
>gi|15608095|ref|NP_215470.1| PROBABLE CONSERVED INTEGRAL MEMBRANE PROTEIN [Mycobacterium tuberculosis H37Rv]
>gi|15608104|ref|NP_215479.1| hypothetical protein Rv0964c [Mycobacterium tuberculosis H37Rv]
>gi|15608108|ref|NP_215483.1| hypothetical protein Rv0968 [Mycobacterium tuberculosis H37Rv]
>gi|15608110|ref|NP_215485.1| PROBABLE CONSERVED INTEGRAL MEMBRANE PROTEIN [Mycobacterium tuberculosis H37Rv]
>gi|57116805|ref|YP_177773.1| PE-PGRS FAMILY PROTEIN [Mycobacterium tuberculosis H37Rv]
>gi|57116806|ref|YP_177774.1| PE-PGRS FAMILY PROTEIN [Mycobacterium tuberculosis H37Rv]
>gi|57116809|ref|YP_177775.1| PE-PGRS FAMILY PROTEIN [Mycobacterium tuberculosis H37Rv]
>gi|15608123|ref|NP_215498.1| PROBABLE SERINE PROTEASE PEPD (SERINE PROTEINASE) (MTB32B) [Mycobacterium tuberculosis H37Rv]
>gi|15608130|ref|NP_215505.1| hypothetical protein Rv0990c [Mycobacterium tuberculosis H37Rv]
>gi|15608131|ref|NP_215506.1| CONSERVED HYPOTHETICAL SERINE RICH PROTEIN [Mycobacterium tuberculosis H37Rv]
>gi|15608132|ref|NP_215507.1| hypothetical protein Rv0992c [Mycobacterium tuberculosis H37Rv]
>gi|15608136|ref|NP_215511.1| PROBABLE CONSERVED TRANSMEMBRANE PROTEIN [Mycobacterium tuberculosis H37Rv]
>gi|15608144|ref|NP_215520.1| PROBABLE MEMBRANE PROTEIN [Mycobacterium tuberculosis H37Rv]
>gi|15608148|ref|NP_215524.1| PROBABLE DEOXYRIBONUCLEASE TATD (YJJV PROTEIN) [Mycobacterium tuberculosis H37Rv]
>gi|15608151|ref|NP_215527.1| Probable 4-diphosphocytidyl-2-C-methyl-D-erythritol kinase ISPE (CMK) (4-(cytidine-5'-diphospho)-2-C-methyl-D-erythritol kinase) [Mycobacterium tuberculosis H37Rv]
>gi|15608175|ref|NP_215551.1| PROBABLE TRANSPOSASE (FRAGMENT) [Mycobacterium tuberculosis H37Rv]
>gi|57116813|ref|YP_177778.1| PPE FAMILY PROTEIN [Mycobacterium tuberculosis H37Rv]
>gi|57116814|ref|YP_177779.1| PE FAMILY PROTEIN [Mycobacterium tuberculosis H37Rv]
>gi|15608204|ref|NP_215580.1| POSSIBLE LIPOPROTEIN LPQV [Mycobacterium tuberculosis H37Rv]
>gi|57116818|ref|YP_177780.1| PE-PGRS FAMILY PROTEIN [Mycobacterium tuberculosis H37Rv]
>gi|57116819|ref|YP_177781.1| PE-PGRS FAMILY PROTEIN [Mycobacterium tuberculosis H37Rv]
>gi|15608218|ref|NP_215594.1| Probable Proline-rich antigen homolog pra [Mycobacterium tuberculosis H37Rv]
>gi|57116821|ref|YP_177783.1| PE-PGRS FAMILY PROTEIN [Mycobacterium tuberculosis H37Rv]
>gi|57116823|ref|YP_177784.1| PE FAMILY PROTEIN [Mycobacterium tuberculosis H37Rv]
>gi|57116824|ref|YP_177785.1| PE FAMILY PROTEIN [Mycobacterium tuberculosis H37Rv]
>gi|57116826|ref|YP_177786.1| PE-PGRS FAMILY PROTEIN [Mycobacterium tuberculosis H37Rv]
>gi|15608237|ref|NP_215613.1| PROBABLE MEMBRANE GLYCINE AND PROLINE RICH PROTEIN [Mycobacterium tuberculosis H37Rv]
>gi|15608243|ref|NP_215619.1| hypothetical protein Rv1103c [Mycobacterium tuberculosis H37Rv]

>gi|57116830|ref|NP_215627.2| hypothetical protein Rv1111c [Mycobacterium tuberculosis H37Rv]
>gi|15608254|ref|NP_215630.1| hypothetical protein Rv1114 [Mycobacterium tuberculosis H37Rv]
>gi|15608274|ref|NP_215650.1| hypothetical protein Rv1134 [Mycobacterium tuberculosis H37Rv]
>gi|57116833|ref|YP_177790.1| PPE FAMILY PROTEIN [Mycobacterium tuberculosis H37Rv]
>gi|15608281|ref|NP_215657.1| PROBABLE ENOYL-CoA HYDRATASE ECHA11 (ENOYL HYDRASE) (UNSATURATED ACYL-CoA HYDRATASE) (CROTONASE) [Mycobacterium tuberculosis H37Rv]
>gi|15608286|ref|NP_215662.1| PROBABLE CONSERVED TRANSMEMBRANE TRANSPORT PROTEIN MMPL13B [Mycobacterium tuberculosis H37Rv]
>gi|15608297|ref|NP_215673.1| CONSERVED HYPOTHETICAL ALA-, PRO-RICH PROTEIN [Mycobacterium tuberculosis H37Rv]
>gi|15608298|ref|NP_215674.1| CONSERVED HYPOTHETICAL ALA-, PRO-RICH PROTEIN [Mycobacterium tuberculosis H37Rv]
>gi|57116838|ref|NP_215687.2| hypothetical protein Rv1171 [Mycobacterium tuberculosis H37Rv]
>gi|57116839|ref|YP_177793.1| PE FAMILY PROTEIN [Mycobacterium tuberculosis H37Rv]
>gi|15608314|ref|NP_215690.1| LOW MOLECULAR WEIGHT T-CELL ANTIGEN TB8.4 [Mycobacterium tuberculosis H37Rv]
>gi|15608331|ref|NP_215707.1| hypothetical protein Rv1191 [Mycobacterium tuberculosis H37Rv]
>gi|57116840|ref|YP_177794.1| PE FAMILY PROTEIN [Mycobacterium tuberculosis H37Rv]
>gi|57116841|ref|YP_177795.1| PPE FAMILY PROTEIN [Mycobacterium tuberculosis H37Rv]
>gi|15608343|ref|NP_215719.1| hypothetical protein Rv1203c [Mycobacterium tuberculosis H37Rv]
>gi|15608344|ref|NP_215720.1| hypothetical protein Rv1204c [Mycobacterium tuberculosis H37Rv]
>gi|57116843|ref|YP_177797.1| PE FAMILY PROTEIN [Mycobacterium tuberculosis H37Rv]
>gi|15608357|ref|NP_215733.1| PROBABLE TETRONASIN-TRANSPORT INTEGRAL MEMBRANE PROTEIN ABC TRANSPORTER [Mycobacterium tuberculosis H37Rv]
>gi|15608362|ref|NP_215738.1| hypothetical protein Rv1222 [Mycobacterium tuberculosis H37Rv]
>gi|15608366|ref|NP_215742.1| PROBABLE TRANSMEMBRANE PROTEIN [Mycobacterium tuberculosis H37Rv]
>gi|15608370|ref|NP_215746.1| POSSIBLE MEMBRANE PROTEIN [Mycobacterium tuberculosis H37Rv]
>gi|15608373|ref|NP_215749.1| CONSERVED HYPOTHETICAL MEMBRANE PROTEIN [Mycobacterium tuberculosis H37Rv]
>gi|57116845|ref|YP_177798.1| PE-PGRS FAMILY PROTEIN [Mycobacterium tuberculosis H37Rv]
>gi|15608389|ref|NP_215765.1| POSSIBLE MEMBRANE PROTEIN [Mycobacterium tuberculosis H37Rv]
>gi|15608392|ref|NP_215768.1| PROBABLE LIPOPROTEIN LPRE [Mycobacterium tuberculosis H37Rv]
>gi|15608398|ref|NP_215774.1| PROBABLE CONSERVED INTEGRAL MEMBRANE TRANSPORT PROTEIN [Mycobacterium tuberculosis H37Rv]
>gi|15608406|ref|NP_215782.1| PROBABLE TRANSMEMBRANE SERINE/THREONINE-PROTEIN KINASE H PKNH (PROTEIN KINASE H) (STPK H) [Mycobacterium tuberculosis H37Rv]
>gi|15608409|ref|NP_215785.1| CONSERVED PROBABLE SECRETED PROTEIN [Mycobacterium tuberculosis H37Rv]
>gi|15608418|ref|NP_215794.1| hypothetical protein Rv1278 [Mycobacterium tuberculosis H37Rv]
>gi|15608431|ref|NP_215807.1| CONSERVED HYPOTHETICAL SECRETED PROTEIN [Mycobacterium tuberculosis H37Rv]
>gi|15608443|ref|NP_215819.1| CONSERVED HYPOTHETICAL TRANSMEMBRANE PROTEIN [Mycobacterium tuberculosis H37Rv]
>gi|15608445|ref|NP_215821.1| PROBABLE ATP SYNTHASE C CHAIN ATPE (LIPID-BINDING PROTEIN) (DICYCLOHEXYLCARBODIIMIDE-BINDING PROTEIN) [Mycobacterium tuberculosis H37Rv]
>gi|15608447|ref|NP_215823.1| PROBABLE ATP SYNTHASE DELTA CHAIN ATPH [Mycobacterium tuberculosis H37Rv]
>gi|57116848|ref|YP_177799.1| PE-PGRS FAMILY PROTEIN [Mycobacterium tuberculosis H37Rv]
>gi|57116850|ref|NP_215846.2| hypothetical protein Rv1330c [Mycobacterium tuberculosis H37Rv]
>gi|15608473|ref|NP_215849.1| PROBABLE HYDROLASE [Mycobacterium tuberculosis H37Rv]
>gi|15608488|ref|NP_215864.1| PROBABLE DRUGS-TRANSPORT TRANSMEMBRANE ATP-BINDING PROTEIN ABC TRANSPORTER [Mycobacterium tuberculosis H37Rv]
>gi|15608489|ref|NP_215865.1| PROBABLE DRUGS-TRANSPORT TRANSMEMBRANE ATP-BINDING PROTEIN ABC TRANSPORTER [Mycobacterium tuberculosis H37Rv]
>gi|57116852|ref|YP_177801.1| PPE FAMILY PROTEIN [Mycobacterium tuberculosis H37Rv]
>gi|15608503|ref|NP_215879.1| POSSIBLE MEMBRANE PROTEIN [Mycobacterium tuberculosis H37Rv]
>gi|15608518|ref|NP_215894.1| hypothetical protein Rv1378c [Mycobacterium tuberculosis H37Rv]
>gi|15608524|ref|NP_215901.1| PROBABLE OROTIDINE 5'-PHOSPHATE DECARBOXYLASE PYRF (OMP decarboxylase) (OMPdecase) [Mycobacterium tuberculosis H37Rv]
>gi|57116857|ref|YP_177805.1| PE FAMILY PROTEIN [Mycobacterium tuberculosis H37Rv]
>gi|57116858|ref|YP_177806.1| PPE FAMILY PROTEIN [Mycobacterium tuberculosis H37Rv]
>gi|15608527|ref|NP_215904.1| PUTATIVE INTEGRATION HOST FACTOR MIHF [Mycobacterium tuberculosis H37Rv]
>gi|15608530|ref|NP_215907.1| PROBABLE DNA/PANTOTHENATE METABOLISM FLAVOPROTEIN HOMOLOG DFP [Mycobacterium tuberculosis H37Rv]
>gi|57116861|ref|YP_177809.1| PE-PGRS FAMILY PROTEIN [Mycobacterium tuberculosis H37Rv]
>gi|57116862|ref|YP_177810.1| PE FAMILY PROTEIN [Mycobacterium tuberculosis H37Rv]
>gi|15608572|ref|NP_215950.1| hypothetical protein Rv1434 [Mycobacterium tuberculosis H37Rv]
>gi|15608573|ref|NP_215951.1| Probable conserved Proline, Glycine, Valine-rich secreted protein [Mycobacterium tuberculosis H37Rv]
>gi|57116863|ref|NP_215956.2| PROBABLE PROTEIN-EXPORT MEMBRANE PROTEIN (TRANSLOCASE SUBUNIT) SECG [Mycobacterium tuberculosis H37Rv]
>gi|57116864|ref|YP_177811.1| PE-PGRS FAMILY PROTEIN [Mycobacterium tuberculosis H37Rv]
>gi|15608583|ref|NP_215961.1| PROBABLE 6-PHOSPHOGLUCONOLACTONASE DEVB (6PGL) [Mycobacterium tuberculosis H37Rv]
>gi|57116865|ref|YP_177812.1| PE-PGRS FAMILY PROTEIN [Mycobacterium tuberculosis H37Rv]
>gi|57116866|ref|YP_177813.1| PE-PGRS FAMILY PROTEIN [Mycobacterium tuberculosis H37Rv]
>gi|15608594|ref|NP_215972.1| PROBABLE UNIDENTIFIED ANTIBIOTIC-TRANSPORT INTEGRAL MEMBRANE ABC TRANSPORTER [Mycobacterium tuberculosis H37Rv]
>gi|15608595|ref|NP_215973.1| PROBABLE UNIDENTIFIED ANTIBIOTIC-TRANSPORT INTEGRAL MEMBRANE ABC TRANSPORTER [Mycobacterium tuberculosis H37Rv]

>gi|57116869|ref|YP_177814.1| PE-PGRS FAMILY PROTEIN [Mycobacterium tuberculosis H37Rv]
>gi|15608615|ref|NP_215993.1| HYPOTHETICAL INVASION PROTEIN [Mycobacterium tuberculosis H37Rv]
>gi|15608625|ref|NP_216003.1| CONSERVED MEMBRANE PROTEIN [Mycobacterium tuberculosis H37Rv]
>gi|15608626|ref|NP_216004.1| POSSIBLE EXPORTED CONSERVED PROTEIN [Mycobacterium tuberculosis H37Rv]
>gi|57116876|ref|YP_177646.1| hypothetical protein Rv1489A [Mycobacterium tuberculosis H37Rv]
>gi|15608629|ref|NP_216007.1| CONSERVED MEMBRANE PROTEIN [Mycobacterium tuberculosis H37Rv]
>gi|15608632|ref|NP_216010.1| hypothetical protein Rv1494 [Mycobacterium tuberculosis H37Rv]
>gi|15608648|ref|NP_216026.1| conserved probable membrane protein [Mycobacterium tuberculosis H37Rv]
>gi|15608655|ref|NP_216033.1| CONSERVED HYPOTHETICAL TRANSMEMBRANE PROTEIN [Mycobacterium tuberculosis H37Rv]
>gi|15608668|ref|NP_216046.1| Probable alcohol dehydrogenase adh [Mycobacterium tuberculosis H37Rv]
>gi|57116882|ref|YP_177817.1| PPE FAMILY PROTEIN [Mycobacterium tuberculosis H37Rv]
>gi|15608693|ref|NP_216071.1| PROBABLE FUMARATE REDUCTASE [MEMBRANE ANCHOR SUBUNIT] FRDD (FUMARATE DEHYDROGENASE) (FUMARIC HYDROGENASE) [Mycobacterium tuberculosis H37Rv]
>gi|15608695|ref|NP_216073.1| PROBABLE CONSERVED TRANSMEMBRANE TRANSPORT PROTEIN MMPL6 [Mycobacterium tuberculosis H37Rv]
>gi|15608704|ref|NP_216082.1| Possible inv protein [Mycobacterium tuberculosis H37Rv]
>gi|57116887|ref|YP_177822.1| PROBABLE 8-AMINO-7-OXONONANOATE SYNTHASE BIOF1 (AONS) (8-AMINO-7-KETOPELARGONATE SYNTHASE) (7-KETO-8-AMINO-PELARGONIC ACID SYNTHETASE) (7-KAP SYNTHETASE) (L-ALANINE--PIMELYL CoA LIGASE) [Mycobacterium tuberculosis H37Rv]
>gi|15608708|ref|NP_216086.1| Probable dethiobiotin synthetase bioD [Mycobacterium tuberculosis H37Rv]
>gi|57116890|ref|NP_216103.2| Partial REP13E12 repeat protein [Mycobacterium tuberculosis H37Rv]
>gi|15608726|ref|NP_216104.1| Partial REP13E12 repeat protein [Mycobacterium tuberculosis H37Rv]
>gi|15608745|ref|NP_216123.1| Probable ionic transporter integral membrane protein chaA [Mycobacterium tuberculosis H37Rv]
>gi|15608748|ref|NP_216126.1| POSSIBLE CONSERVED MEMBRANE PROTEIN [Mycobacterium tuberculosis H37Rv]
>gi|15608749|ref|NP_216127.1| Probable indole-3-glycerol phosphate synthase trpC [Mycobacterium tuberculosis H37Rv]
>gi|15608751|ref|NP_216129.1| Probable tryptophan synthase, alpha subunit trpA [Mycobacterium tuberculosis H37Rv]
>gi|15608752|ref|NP_216130.1| Possible prolipoprotein diacylglyceryl transferases Lgt [Mycobacterium tuberculosis H37Rv]
>gi|15608753|ref|NP_216131.1| Probable hypothetical membrane protein [Mycobacterium tuberculosis H37Rv]
>gi|15608758|ref|NP_216136.1| PROBABLE 'COMPONENT LINKED WITH THE ASSEMBLY OF CYTOCHROME' TRANSPORT TRANSMEMBRANE ATP-BINDING PROTEIN ABC TRANSPORTER CYDC [Mycobacterium tuberculosis H37Rv]
>gi|15608762|ref|NP_216140.1| Probable conserved membrane protein [Mycobacterium tuberculosis H37Rv]
>gi|15608770|ref|NP_216148.1| hypothetical protein Rv1632c [Mycobacterium tuberculosis H37Rv]
>gi|15608772|ref|NP_216150.1| Possible drug efflux membrane protein [Mycobacterium tuberculosis H37Rv]
>gi|57116896|ref|YP_177825.1| PE FAMILY PROTEIN [Mycobacterium tuberculosis H37Rv]
>gi|15608786|ref|NP_216164.1| Probable transmembrane protein [Mycobacterium tuberculosis H37Rv]
>gi|15608787|ref|NP_216165.1| Probable phenylalanyl-tRNA synthetase, alpha chain PheS [Mycobacterium tuberculosis H37Rv]
>gi|57116897|ref|YP_177826.1| PE-PGRS FAMILY PROTEIN [Mycobacterium tuberculosis H37Rv]
>gi|15608791|ref|NP_216169.1| Probable Glutamate n-acetyltransferase argJ [Mycobacterium tuberculosis H37Rv]
>gi|15608805|ref|NP_216183.1| PROBABLE SECOND PART OF MACROLIDE-TRANSPORT ATP-BINDING PROTEIN ABC TRANSPORTER [Mycobacterium tuberculosis H37Rv]
>gi|15608810|ref|NP_216188.1| PROBABLE CONSERVED INTEGRAL MEMBRANE TRANSPORT PROTEIN [Mycobacterium tuberculosis H37Rv]
>gi|15608816|ref|NP_216194.1| PROBABLE INTEGRAL MEMBRANE PROTEIN [Mycobacterium tuberculosis H37Rv]
>gi|15608834|ref|NP_216212.1| PROBABLE DNA REPAIR PROTEIN RECN (RECOMBINATION PROTEIN N) [Mycobacterium tuberculosis H37Rv]
>gi|15608842|ref|NP_216220.1| PROBABLE D-SERINE/ALANINE/GLYCINE TRANSPORTER PROTEIN CYCA [Mycobacterium tuberculosis H37Rv]
>gi|57116898|ref|YP_177827.1| PPE FAMILY PROTEIN [Mycobacterium tuberculosis H37Rv]
>gi|57116899|ref|YP_177828.1| PPE FAMILY PROTEIN [Mycobacterium tuberculosis H37Rv]
>gi|15608845|ref|NP_216223.1| PROBABLE CONSERVED TRANSMEMBRANE PROTEIN [Mycobacterium tuberculosis H37Rv]
>gi|57116901|ref|YP_177829.1| PROBABLE 3-HYDROXYBUTYRYL-CoA DEHYDROGENASE FADB3 (BETA-HYDROXYBUTYRYL-CoA DEHYDROGENASE) (BHBD) [Mycobacterium tuberculosis H37Rv]
>gi|15608855|ref|NP_216233.1| hypothetical protein Rv1717 [Mycobacterium tuberculosis H37Rv]
>gi|57116902|ref|NP_216237.2| hypothetical protein Rv1721c [Mycobacterium tuberculosis H37Rv]
>gi|15608877|ref|NP_216255.1| PROBABLE SULPHATE-TRANSPORT TRANSMEMBRANE PROTEIN ABC TRANSPORTER [Mycobacterium tuberculosis H37Rv]
>gi|15608882|ref|NP_216260.1| PROBABLE MEMBRANE PROTEIN [Mycobacterium tuberculosis H37Rv]
>gi|15608884|ref|NP_216262.1| ANCHORED-MEMBRANE SERINE/THREONINE-PROTEIN KINASE PKNF (PROTEIN KINASE F) (STPK F) [Mycobacterium tuberculosis H37Rv]
>gi|57116904|ref|YP_177830.1| PPE FAMILY PROTEIN [Mycobacterium tuberculosis H37Rv]
>gi|15608892|ref|NP_216270.1| hypothetical protein Rv1754c [Mycobacterium tuberculosis H37Rv]
>gi|57116905|ref|YP_177831.1| PE-PGRS FAMILY PROTEIN [Mycobacterium tuberculosis H37Rv]
>gi|57116908|ref|YP_177832.1| PE-PGRS FAMILY PROTEIN [Mycobacterium tuberculosis H37Rv]
>gi|15608911|ref|NP_216289.1| PROBABLE TRANSCRIPTIONAL REGULATORY PROTEIN [Mycobacterium tuberculosis H37Rv]
>gi|15608917|ref|NP_216295.1| HYPOTHETICAL INTEGRAL MEMBRANE PROTEIN [Mycobacterium tuberculosis H37Rv]
>gi|57116909|ref|YP_177833.1| PPE FAMILY PROTEIN [Mycobacterium tuberculosis H37Rv]
>gi|57116910|ref|YP_177834.1| PE FAMILY PROTEIN [Mycobacterium tuberculosis H37Rv]
>gi|57116911|ref|YP_177835.1| PPE FAMILY PROTEIN [Mycobacterium tuberculosis H37Rv]
>gi|57116912|ref|YP_177836.1| PPE FAMILY PROTEIN [Mycobacterium tuberculosis H37Rv]
>gi|57116913|ref|YP_177837.1| PE FAMILY PROTEIN [Mycobacterium tuberculosis H37Rv]

>gi|15608932|ref|NP_216311.1| CONSERVED HYPOTHETICAL MEMBRANE PROTEIN [Mycobacterium tuberculosis H37Rv]
>gi|57116916|ref|YP_177840.1| PPE FAMILY PROTEIN [Mycobacterium tuberculosis H37Rv]
>gi|57116917|ref|YP_177841.1| PPE FAMILY PROTEIN [Mycobacterium tuberculosis H37Rv]
>gi|57116918|ref|YP_177842.1| PE-PGRS FAMILY PROTEIN [Mycobacterium tuberculosis H37Rv]
>gi|57116919|ref|YP_177843.1| PE FAMILY PROTEIN [Mycobacterium tuberculosis H37Rv]
>gi|57116920|ref|YP_177653.1| PPE FAMILY PROTEIN [Mycobacterium tuberculosis H37Rv]
>gi|57116921|ref|YP_177844.1| PPE FAMILY PROTEIN [Mycobacterium tuberculosis H37Rv]
>gi|57116922|ref|YP_177845.1| PPE FAMILY PROTEIN [Mycobacterium tuberculosis H37Rv]
>gi|57116923|ref|YP_177846.1| PE-PGRS FAMILY PROTEIN [Mycobacterium tuberculosis H37Rv]
>gi|15608960|ref|NP_216339.1| hypothetical protein Rv1823 [Mycobacterium tuberculosis H37Rv]
>gi|15608961|ref|NP_216340.1| CONSERVED HYPOTHETICAL MEMBRANE PROTEIN [Mycobacterium tuberculosis H37Rv]
>gi|57116924|ref|YP_177847.1| PE-PGRS FAMILY PROTEIN [Mycobacterium tuberculosis H37Rv]
>gi|15608982|ref|NP_216361.1| CONSERVED HYPOTHETICAL TRANSMEMBRANE PROTEIN [Mycobacterium tuberculosis H37Rv]
>gi|57116926|ref|YP_177849.1| ALANINE AND PROLINE RICH SECRETED PROTEIN APA (FIBRONECTIN ATTACHMENT PROTEIN) (Immunogenic protein MPT32) (Antigen MPT-32) (45-kDa glycoprotein) (45/47 kDa antigen) [Mycobacterium tuberculosis H37Rv]
>gi|15608998|ref|NP_216377.1| PROBABLE CONSERVED TRANSMEMBRANE PROTEIN [Mycobacterium tuberculosis H37Rv]
>gi|15609024|ref|NP_216403.1| hypothetical protein Rv1887 [Mycobacterium tuberculosis H37Rv]
>gi|15609036|ref|NP_216415.1| POSSIBLE LIPOPROTEIN LPPD [Mycobacterium tuberculosis H37Rv]
>gi|57116928|ref|YP_177655.1| PPE FAMILY PROTEIN [Mycobacterium tuberculosis H37Rv]
>gi|57116929|ref|YP_177850.1| PPE FAMILY PROTEIN [Mycobacterium tuberculosis H37Rv]
>gi|15609078|ref|NP_216457.1| PROBABLE SHORT-CHAIN TYPE DEHYDROGENASE/REDUCTASE [Mycobacterium tuberculosis H37Rv]
>gi|15609080|ref|NP_216459.1| hypothetical protein Rv1943c [Mycobacterium tuberculosis H37Rv]
>gi|15609086|ref|NP_216465.1| hypothetical protein Rv1949c [Mycobacterium tuberculosis H37Rv]
>gi|15609088|ref|NP_216467.1| hypothetical protein Rv1951c [Mycobacterium tuberculosis H37Rv]
>gi|15609097|ref|NP_216476.1| hypothetical protein Rv1960c [Mycobacterium tuberculosis H37Rv]
>gi|15609115|ref|NP_216494.1| hypothetical protein Rv1978 [Mycobacterium tuberculosis H37Rv]
>gi|57116933|ref|YP_177854.1| PE-PGRS FAMILY PROTEIN [Mycobacterium tuberculosis H37Rv]
>gi|15609130|ref|NP_216509.1| hypothetical protein Rv1993c [Mycobacterium tuberculosis H37Rv]
>gi|15609136|ref|NP_216515.1| PROBABLE CONSERVED INTEGRAL MEMBRANE PROTEIN [Mycobacterium tuberculosis H37Rv]
>gi|15609160|ref|NP_216539.1| hypothetical protein Rv2023c [Mycobacterium tuberculosis H37Rv]
>gi|15609183|ref|NP_216562.1| Probable lipoprotein lppI [Mycobacterium tuberculosis H37Rv]
>gi|15609186|ref|NP_216565.1| hypothetical protein Rv2049c [Mycobacterium tuberculosis H37Rv]
>gi|15609190|ref|NP_216569.1| PROBABLE TRANSMEMBRANE PROTEIN [Mycobacterium tuberculosis H37Rv]
>gi|15609193|ref|NP_216572.1| Probable ribosomal protein S14 RpsN2 [Mycobacterium tuberculosis H37Rv]
>gi|15609196|ref|NP_216575.1| hypothetical protein Rv2059 [Mycobacterium tuberculosis H37Rv]
>gi|15609197|ref|NP_216576.1| Possible conserved integral membrane protein [Mycobacterium tuberculosis H37Rv]
>gi|57116939|ref|YP_177657.1| hypothetical protein Rv2063 [Mycobacterium tuberculosis H37Rv]
>gi|15609202|ref|NP_216581.1| Probable precorrin-8X methylmutase CobH (aka precorrin isomerase) [Mycobacterium tuberculosis H37Rv]
>gi|15609208|ref|NP_216587.1| Probable precorrin-4 C11-methyltransferase CobM [Mycobacterium tuberculosis H37Rv]
>gi|15609210|ref|NP_216589.1| Probable shortchain dehydrogenase [Mycobacterium tuberculosis H37Rv]
>gi|15609214|ref|NP_216593.1| POSSIBLE CONSERVED TRANSMEMBRANE PROTEIN [Mycobacterium tuberculosis H37Rv]
>gi|57116940|ref|YP_177658.1| hypothetical protein Rv2077A [Mycobacterium tuberculosis H37Rv]
>gi|15609218|ref|NP_216597.1| POSSIBLE TRANSMEMBRANE PROTEIN [Mycobacterium tuberculosis H37Rv]
>gi|15609219|ref|NP_216598.1| hypothetical protein Rv2082 [Mycobacterium tuberculosis H37Rv]
>gi|15609220|ref|NP_216599.1| hypothetical protein Rv2083 [Mycobacterium tuberculosis H37Rv]
>gi|15609221|ref|NP_216600.1| hypothetical protein Rv2084 [Mycobacterium tuberculosis H37Rv]
>gi|15609227|ref|NP_216606.1| Probable 5'-3' exonuclease [Mycobacterium tuberculosis H37Rv]
>gi|15609228|ref|NP_216607.1| Probable membrane protein [Mycobacterium tuberculosis H37Rv]
>gi|15609232|ref|NP_216611.1| hypothetical protein Rv2095c [Mycobacterium tuberculosis H37Rv]
>gi|57116943|ref|YP_177857.1| PE-PGRS FAMILY PROTEIN [Mycobacterium tuberculosis H37Rv]
>gi|57116944|ref|YP_177659.1| PE FAMILY PROTEIN [Mycobacterium tuberculosis H37Rv]
>gi|15609237|ref|NP_216616.1| hypothetical protein Rv2100 [Mycobacterium tuberculosis H37Rv]
>gi|15609248|ref|NP_216627.1| hypothetical protein Rv2111c [Mycobacterium tuberculosis H37Rv]
>gi|15609253|ref|NP_216632.1| Probable conserved lipoprotein lppK [Mycobacterium tuberculosis H37Rv]
>gi|15609257|ref|NP_216636.1| PROBABLE CONSERVED INTEGRAL MEMBRANE PROTEIN [Mycobacterium tuberculosis H37Rv]
>gi|57116948|ref|YP_177861.1| PPE FAMILY PROTEIN [Mycobacterium tuberculosis H37Rv]
>gi|57116949|ref|YP_177862.1| PE-PGRS FAMILY PROTEIN [Mycobacterium tuberculosis H37Rv]
>gi|15609276|ref|NP_216655.1| Probable dihydroorotate dehydrogenase PyrD [Mycobacterium tuberculosis H37Rv]
>gi|15609281|ref|NP_216660.1| Probable transmembrane protein [Mycobacterium tuberculosis H37Rv]
>gi|15609282|ref|NP_216661.1| CONSERVED HYPOTHETICAL PROTEIN WAG31 [Mycobacterium tuberculosis H37Rv]
>gi|15609284|ref|NP_216663.1| hypothetical protein Rv2147c [Mycobacterium tuberculosis H37Rv]
>gi|15609288|ref|NP_216667.1| POSSIBLE CELL DIVISION PROTEIN FTSQ [Mycobacterium tuberculosis H37Rv]
>gi|15609290|ref|NP_216669.1| Probable UPD-N-acetylglucosamine-N-acetylmuramyl-(pentapeptide) pyrophosphoryl-undecaprenol-N-acetylglucosamine transferase MurG [Mycobacterium tuberculosis H37Rv]
>gi|15609294|ref|NP_216673.1| Probable UDP-N-acetylmuramoylalanyl-D-glutamyl-2,6-diaminopimelate- D-alanyl-D-alanyl ligase MurF [Mycobacterium tuberculosis H37Rv]
>gi|15609296|ref|NP_216675.1| hypothetical protein Rv2159c [Mycobacterium tuberculosis H37Rv]
>gi|15609297|ref|NP_216676.1| hypothetical protein Rv2160c [Mycobacterium tuberculosis H37Rv]
>gi|57116953|ref|YP_177865.1| PE-PGRS FAMILY PROTEIN [Mycobacterium tuberculosis H37Rv]
>gi|15609301|ref|NP_216680.1| PROBABLE CONSERVED PROLINE RICH MEMBRANE PROTEIN [Mycobacterium tuberculosis H37Rv]

>gi|15609326|ref|NP_216705.1| hypothetical protein Rv2189c [Mycobacterium tuberculosis H37Rv]
>gi|15609327|ref|NP_216706.1| hypothetical protein Rv2190c [Mycobacterium tuberculosis H37Rv]
>gi|15609335|ref|NP_216714.1| PROBABLE CONSERVED MEMBRANE PROTEIN MMPS3 [Mycobacterium tuberculosis H37Rv]
>gi|15609336|ref|NP_216715.1| Possible conserved integral membrane protein [Mycobacterium tuberculosis H37Rv]
>gi|15609340|ref|NP_216719.1| POSSIBLE CONSERVED MEMBRANE PROTEIN [Mycobacterium tuberculosis H37Rv]
>gi|57116955|ref|NP_216721.2| hypothetical protein Rv2205c [Mycobacterium tuberculosis H37Rv]
>gi|57116956|ref|NP_216722.2| PROBABLE CONSERVED TRANSMEMBRANE PROTEIN [Mycobacterium tuberculosis H37Rv]
>gi|15609345|ref|NP_216724.1| Probable cobalamin 5'-phosphate synthase CobS [Mycobacterium tuberculosis H37Rv]
>gi|15609346|ref|NP_216725.1| Probable conserved integral membrane protein [Mycobacterium tuberculosis H37Rv]
>gi|15609352|ref|NP_216731.1| Probable pyruvate dehydrogenase (E2 component) SucB [Mycobacterium tuberculosis H37Rv]
>gi|57116957|ref|YP_177661.1| PROBABLE CONSERVED MEMBRANE PROTEIN [Mycobacterium tuberculosis H37Rv]
>gi|15609366|ref|NP_216745.1| hypothetical protein Rv2229c [Mycobacterium tuberculosis H37Rv]
>gi|15609367|ref|NP_216746.1| hypothetical protein Rv2230c [Mycobacterium tuberculosis H37Rv]
>gi|15609368|ref|NP_216747.1| Possible aminotransferase CobC [Mycobacterium tuberculosis H37Rv]
>gi|15609377|ref|NP_216756.1| hypothetical protein Rv2240c [Mycobacterium tuberculosis H37Rv]
>gi|57116960|ref|YP_177662.1| POSSIBLE FLAVOPROTEIN [Mycobacterium tuberculosis H37Rv]
>gi|15609391|ref|NP_216770.1| Probable integral membrane protein [Mycobacterium tuberculosis H37Rv]
>gi|15609401|ref|NP_216780.1| conserved hypothetical proline rich protein [Mycobacterium tuberculosis H37Rv]
>gi|15609419|ref|NP_216798.1| Probable transcription regulator (lysR family) [Mycobacterium tuberculosis H37Rv]
>gi|57116962|ref|YP_177663.1| POSSIBLE CONSERVED MEMBRANE PROTEIN [Mycobacterium tuberculosis H37Rv]
>gi|15609447|ref|NP_216826.1| POSSIBLE EXCISIONASE [Mycobacterium tuberculosis H37Rv]
>gi|15609462|ref|NP_216841.1| hypothetical protein Rv2325c [Mycobacterium tuberculosis H37Rv]
>gi|15609464|ref|NP_216843.1| hypothetical protein Rv2327 [Mycobacterium tuberculosis H37Rv]
>gi|57116969|ref|YP_177867.1| PE FAMILY PROTEIN [Mycobacterium tuberculosis H37Rv]
>gi|15609472|ref|NP_216851.1| PROBABLE SERINE ACETYLTRANSFERASE CYSE (SAT) [Mycobacterium tuberculosis H37Rv]
>gi|57116973|ref|YP_177869.1| PE-PGRS FAMILY PROTEIN [Mycobacterium tuberculosis H37Rv]
>gi|15609482|ref|NP_216861.1| POSSIBLE CONSERVED TRANSMEMBRANE PROTEIN [Mycobacterium tuberculosis H37Rv]
>gi|15609485|ref|NP_216864.1| hypothetical protein Rv2348c [Mycobacterium tuberculosis H37Rv]
>gi|57116974|ref|YP_177870.1| PPE FAMILY PROTEIN [Mycobacterium tuberculosis H37Rv]
>gi|57116975|ref|YP_177871.1| PPE FAMILY PROTEIN [Mycobacterium tuberculosis H37Rv]
>gi|57116976|ref|YP_177872.1| PPE FAMILY PROTEIN [Mycobacterium tuberculosis H37Rv]
>gi|15609502|ref|NP_216881.1| hypothetical protein Rv2365c [Mycobacterium tuberculosis H37Rv]
>gi|57116979|ref|YP_177875.1| PE-PGRS FAMILY PROTEIN [Mycobacterium tuberculosis H37Rv]
>gi|15609510|ref|NP_216889.1| PROBABLE CHAPERONE PROTEIN DNAJ2 [Mycobacterium tuberculosis H37Rv]
>gi|15609532|ref|NP_216911.1| PROBABLE CONSERVED INTEGRAL MEMBRANE PROTEIN [Mycobacterium tuberculosis H37Rv]
>gi|57116982|ref|YP_177878.1| PE-PGRS FAMILY PROTEIN [Mycobacterium tuberculosis H37Rv]
>gi|57116985|ref|YP_177670.1| POSSIBLE CONSERVED MEMBRANE PROTEIN [Mycobacterium tuberculosis H37Rv]
>gi|57116986|ref|YP_177880.1| POSSIBLE PE FAMILY-RELATED PROTEIN [Mycobacterium tuberculosis H37Rv]
>gi|15609549|ref|NP_216928.1| PROBABLE 30S RIBOSOMAL PROTEIN S20 RPST [Mycobacterium tuberculosis H37Rv]
>gi|15609550|ref|NP_216929.1| hypothetical protein Rv2413c [Mycobacterium tuberculosis H37Rv]
>gi|15609551|ref|NP_216930.1| hypothetical protein Rv2414c [Mycobacterium tuberculosis H37Rv]
>gi|15609552|ref|NP_216931.1| hypothetical protein Rv2415c [Mycobacterium tuberculosis H37Rv]
>gi|15609554|ref|NP_216933.1| hypothetical protein Rv2417c [Mycobacterium tuberculosis H37Rv]
>gi|15609559|ref|NP_216938.1| hypothetical protein Rv2422 [Mycobacterium tuberculosis H37Rv]
>gi|15609566|ref|NP_216945.1| ALKYL HYDROPEROXIDE REDUCTASE D PROTEIN AHPD (ALKYL HYDROPEROXIDASE D) [Mycobacterium tuberculosis H37Rv]
>gi|15609573|ref|NP_216952.1| RIBOKINASE RBSK [Mycobacterium tuberculosis H37Rv]
>gi|15609576|ref|NP_216955.1| PROBABLE GLUTAMATE 5-KINASE PROTEIN PROB (GAMMA-GLUTAMYL KINASE) (GK) [Mycobacterium tuberculosis H37Rv]
>gi|15609581|ref|NP_216960.1| POSSIBLE RIBONUCLEASE E RNE [Mycobacterium tuberculosis H37Rv]
>gi|15609587|ref|NP_216966.1| PROBABLE RESUSCITATION-PROMOTING FACTOR RPFE [Mycobacterium tuberculosis H37Rv]
>gi|15609593|ref|NP_216972.1| PROBABLE CONSERVED INTEGRAL MEMBRANE TRANSPORT PROTEIN [Mycobacterium tuberculosis H37Rv]
>gi|15609615|ref|NP_216994.1| hypothetical protein Rv2478c [Mycobacterium tuberculosis H37Rv]
>gi|57116995|ref|YP_177886.1| PE-PGRS FAMILY PROTEIN [Mycobacterium tuberculosis H37Rv]
>gi|15609626|ref|NP_217005.1| HYPOTHETICAL ALANINE RICH PROTEIN [Mycobacterium tuberculosis H37Rv]
>gi|57116996|ref|YP_177887.1| PE-PGRS FAMILY PROTEIN [Mycobacterium tuberculosis H37Rv]
>gi|15609644|ref|NP_217023.1| POSSIBLE CONSERVED PROLINE RICH MEMBRANE PROTEIN [Mycobacterium tuberculosis H37Rv]
>gi|15609645|ref|NP_217024.1| PROBABLE CONSERVED INTEGRAL MEMBRANE LEUCINE AND ALANINE RICH PROTEIN [Mycobacterium tuberculosis H37Rv]
>gi|15609646|ref|NP_217025.1| PROBABLE SHORT-CHAIN TYPE DEHYDROGENASE/REDUCTASE [Mycobacterium tuberculosis H37Rv]
>gi|57116998|ref|YP_177888.1| PE FAMILY PROTEIN [Mycobacterium tuberculosis H37Rv]
>gi|15609663|ref|NP_217042.1| hypothetical protein Rv2526 [Mycobacterium tuberculosis H37Rv]
>gi|15609670|ref|NP_217049.1| N UTILIZATION SUBSTANCE PROTEIN NUSB (NUSB PROTEIN) [Mycobacterium tuberculosis H37Rv]
>gi|15609673|ref|NP_217052.1| PROBABLE CONSERVED TRANSMEMBRANE PROTEIN [Mycobacterium tuberculosis H37Rv]
>gi|15609678|ref|NP_217057.1| HYPOTHETICAL ALANINE RICH PROTEIN [Mycobacterium tuberculosis H37Rv]
>gi|15609685|ref|NP_217064.1| hypothetical protein Rv2548 [Mycobacterium tuberculosis H37Rv]
>gi|15609688|ref|NP_217067.1| hypothetical protein Rv2551c [Mycobacterium tuberculosis H37Rv]
>gi|15609689|ref|NP_217068.1| PROBABLE SHIKIMATE 5-DEHYDROGENASE AROE (5-DEHYDROSHIKIMATE REDUCTASE) [Mycobacterium tuberculosis H37Rv]

>gi|15609697|ref|NP_217076.1| PROBABLE PROLINE AND GLYCINE RICH TRANSMEMBRANE PROTEIN [Mycobacterium tuberculosis H37Rv]
>gi|15609708|ref|NP_217087.1| PROBABLE TRANSMEMBRANE ALANINE AND VALINE AND LEUCINE RICH PROTEIN [Mycobacterium tuberculosis H37Rv]
>gi|15609719|ref|NP_217098.1| PROBABLE PEPTIDYL-PROLYL CIS-TRANS ISOMERASE B PPIB (CYCLOPHILIN) (PPIASE) (ROTAMASE) (PEPTIDYLPROLYL ISOMERASE) [Mycobacterium tuberculosis H37Rv]
>gi|15609724|ref|NP_217103.1| PROBABLE PROTEIN-EXPORT MEMBRANE PROTEIN SECD [Mycobacterium tuberculosis H37Rv]
>gi|57117002|ref|YP_177891.1| PE-PGRS FAMILY PROTEIN [Mycobacterium tuberculosis H37Rv]
>gi|15609737|ref|NP_217116.1| PROBABLE CONSERVED INTEGRAL MEMBRANE PROTEIN [Mycobacterium tuberculosis H37Rv]
>gi|57117003|ref|YP_177892.1| PROBABLE SPERMIDINE SYNTHASE SPEE (PUTRESCINE AMINOPROPYLTRANSFERASE) (AMINOPROPYLTRANSFERASE) (SPDSY) [Mycobacterium tuberculosis H37Rv]
>gi|57117005|ref|YP_177893.1| PPE FAMILY PROTEIN [Mycobacterium tuberculosis H37Rv]
>gi|15609746|ref|NP_217125.1| PROBABLE CONSERVED MEMBRANE PROTEIN [Mycobacterium tuberculosis H37Rv]
>gi|57117008|ref|YP_177895.1| PE-PGRS FAMILY PROTEIN [Mycobacterium tuberculosis H37Rv]
>gi|15609757|ref|NP_217136.1| PROBABLE CONSERVED TRANSMEMBRANE PROTEIN [Mycobacterium tuberculosis H37Rv]
>gi|15609762|ref|NP_217141.1| PROBABLE CONSERVED TRANSMEMBRANE ALANINE AND LEUCINE RICH PROTEIN [Mycobacterium tuberculosis H37Rv]
>gi|57117010|ref|YP_177896.1| PE-PGRS FAMILY PROTEIN [Mycobacterium tuberculosis H37Rv]
>gi|15609774|ref|NP_217153.1| POSSIBLE TRANSMEMBRANE PROTEIN DEDA [Mycobacterium tuberculosis H37Rv]
>gi|15609787|ref|NP_217166.1| POSSIBLE phiRv2 PROPHAGE PROTEIN [Mycobacterium tuberculosis H37Rv]
>gi|15609789|ref|NP_217168.1| PROBABLE phiRv2 PROPHAGE PROTEIN [Mycobacterium tuberculosis H37Rv]
>gi|15609791|ref|NP_217170.1| POSSIBLE phiRv2 PROPHAGE PROTEIN [Mycobacterium tuberculosis H37Rv]
>gi|15609793|ref|NP_217172.1| POSSIBLE phiRv2 PROPHAGE PROTEIN [Mycobacterium tuberculosis H37Rv]
>gi|15609798|ref|NP_217177.1| hypothetical protein Rv2661c [Mycobacterium tuberculosis H37Rv]
>gi|15609815|ref|NP_217194.1| PROBABLE UROPORPHYRINOGEN DECARBOXYLASE HEME (UROPORPHYRINOGEN III DECARBOXYLASE) (URO-D) (UPD) [Mycobacterium tuberculosis H37Rv]
>gi|15609832|ref|NP_217211.1| CONSERVED HYPOTHETICAL ALANINE RICH PROTEIN [Mycobacterium tuberculosis H37Rv]
>gi|15609833|ref|NP_217212.1| CONSERVED HYPOTHETICAL ALANINE AND GLYCINE AND VALINE RICH PROTEIN [Mycobacterium tuberculosis H37Rv]
>gi|15609835|ref|NP_217214.1| PROBABLE CONSERVED ALANINE RICH TRANSMEMBRANE PROTEIN [Mycobacterium tuberculosis H37Rv]
>gi|15609836|ref|NP_217215.1| hypothetical protein Rv2699c [Mycobacterium tuberculosis H37Rv]
>gi|15609838|ref|NP_217217.1| POSSIBLE EXTRAGENIC SUPPRESSOR PROTEIN SUHB [Mycobacterium tuberculosis H37Rv]
>gi|15609840|ref|NP_217219.1| RNA POLYMERASE SIGMA FACTOR SIGA (SIGMA-A) [Mycobacterium tuberculosis H37Rv]
>gi|15609846|ref|NP_217225.1| PROBABLE CONSERVED TRANSMEMBRANE PROTEIN [Mycobacterium tuberculosis H37Rv]
>gi|15609849|ref|NP_217228.1| hypothetical protein Rv2712c [Mycobacterium tuberculosis H37Rv]
>gi|15609856|ref|NP_217235.1| POSSIBLE CONSERVED MEMBRANE PROTEIN [Mycobacterium tuberculosis H37Rv]
>gi|15609858|ref|NP_217237.1| POSSIBLE CONSERVED TRANSMEMBRANE ALANINE AND GLYCINE RICH PROTEIN [Mycobacterium tuberculosis H37Rv]
>gi|15609863|ref|NP_217242.1| PROBABLE DIAMINOPIMELATE EPIMERASE DAPF (DAP EPIMERASE) [Mycobacterium tuberculosis H37Rv]
>gi|15609865|ref|NP_217244.1| CONSERVED HYPOTHETICAL ALANINE RICH PROTEIN [Mycobacterium tuberculosis H37Rv]
>gi|15609866|ref|NP_217245.1| PROBABLE CONSERVED INTEGRAL MEMBRANE ALANINE VALINE AND LEUCINE RICH PROTEIN [Mycobacterium tuberculosis H37Rv]
>gi|15609868|ref|NP_217247.1| CONSERVED HYPOTHETICAL ALANINE AND ARGININE RICH PROTEIN [Mycobacterium tuberculosis H37Rv]
>gi|57117018|ref|YP_177902.1| PE-PGRS FAMILY PROTEIN [Mycobacterium tuberculosis H37Rv]
>gi|15609879|ref|NP_217258.1| CONSERVED HYPOTHETICAL ARGININE RICH PROTEIN [Mycobacterium tuberculosis H37Rv]
>gi|15609882|ref|NP_217261.1| POSSIBLE TRANSCRIPTIONAL REGULATORY PROTEIN [Mycobacterium tuberculosis H37Rv]
>gi|57117020|ref|YP_177904.1| POSSIBLE TYPE I RESTRICTION/MODIFICATION SYSTEM SPECIFICITY DETERMINANT (FRAGMENT) HSDS.1 (S PROTEIN) [Mycobacterium tuberculosis H37Rv]
>gi|15609895|ref|NP_217274.1| hypothetical protein Rv2758c [Mycobacterium tuberculosis H37Rv]
>gi|15609897|ref|NP_217276.1| hypothetical protein Rv2760c [Mycobacterium tuberculosis H37Rv]
>gi|15609899|ref|NP_217278.1| hypothetical protein Rv2762c [Mycobacterium tuberculosis H37Rv]
>gi|57117021|ref|YP_177905.1| PROBABLE SHORT-CHAIN TYPE DEHYDROGENASE/REDUCTASE [Mycobacterium tuberculosis H37Rv]
>gi|57117022|ref|YP_177906.1| PPE FAMILY PROTEIN [Mycobacterium tuberculosis H37Rv]
>gi|57117023|ref|YP_177907.1| PE FAMILY PROTEIN [Mycobacterium tuberculosis H37Rv]
>gi|57117024|ref|YP_177677.1| PPE FAMILY PROTEIN [Mycobacterium tuberculosis H37Rv]
>gi|15609909|ref|NP_217288.1| PROBABLE CONSERVED TRANSMEMBRANE PROTEIN [Mycobacterium tuberculosis H37Rv]
>gi|15609934|ref|NP_217313.1| hypothetical protein Rv2797c [Mycobacterium tuberculosis H37Rv]
>gi|15609939|ref|NP_217318.1| HYPOTHETICAL ARGININE AND ALANINE RICH PROTEIN [Mycobacterium tuberculosis H37Rv]
>gi|15609973|ref|NP_217352.1| POSSIBLE DNA-DAMAGE-INDUCIBLE PROTEIN F DINF [Mycobacterium tuberculosis H37Rv]
>gi|15609975|ref|NP_217354.1| PROBABLE RIBOSOME-BINDING FACTOR A RBFA (P15B PROTEIN) [Mycobacterium tuberculosis H37Rv]
>gi|15609976|ref|NP_217355.1| PROBABLE TRANSLATION INITIATION FACTOR IF-2 INFB [Mycobacterium tuberculosis H37Rv]
>gi|15609980|ref|NP_217359.1| PROBABLE CONSERVED TRANSMEMBRANE ALANINE RICH PROTEIN [Mycobacterium tuberculosis H37Rv]
>gi|15609987|ref|NP_217366.1| POSSIBLE MAGNESIUM CHELATASE [Mycobacterium tuberculosis H37Rv]
>gi|57117029|ref|YP_177909.1| PE-PGRS FAMILY PROTEIN [Mycobacterium tuberculosis H37Rv]
>gi|15610010|ref|NP_217389.1| CELL SURFACE LIPOPROTEIN MPT83 (LIPOPROTEIN P23) [Mycobacterium tuberculosis H37Rv]
>gi|15610012|ref|NP_217391.1| MAJOR SECRETED IMMUNOGENIC PROTEIN MPT70 [Mycobacterium tuberculosis H37Rv]

>gi|57117033|ref|YP_177912.1| PROBABLE CONSERVED INTEGRAL MEMBRANE PROTEIN [Mycobacterium tuberculosis H37Rv]
>gi|15610021|ref|NP_217400.1| PROBABLE TRANSCRIPTIONAL REGULATORY PROTEIN [Mycobacterium tuberculosis H37Rv]
>gi|15610024|ref|NP_217403.1| PROBABLE TRANSCRIPTIONAL REGULATORY PROTEIN [Mycobacterium tuberculosis H37Rv]
>gi|15610027|ref|NP_217406.1| PROBABLE 30S RIBOSOMAL PROTEIN S2 RPSB [Mycobacterium tuberculosis H37Rv]
>gi|15610028|ref|NP_217407.1| hypothetical protein Rv2891 [Mycobacterium tuberculosis H37Rv]
>gi|57117034|ref|YP_177913.1| PPE FAMILY PROTEIN [Mycobacterium tuberculosis H37Rv]
>gi|15610031|ref|NP_217410.1| PROBABLE INTEGRASE/RECOMBINASE XERC [Mycobacterium tuberculosis H37Rv]
>gi|15610033|ref|NP_217412.1| hypothetical protein Rv2896c [Mycobacterium tuberculosis H37Rv]
>gi|15610045|ref|NP_217424.1| hypothetical protein Rv2908c [Mycobacterium tuberculosis H37Rv]
>gi|15610046|ref|NP_217425.1| PROBABLE 30S RIBOSOMAL PROTEIN S16 RPSP [Mycobacterium tuberculosis H37Rv]
>gi|15610056|ref|NP_217435.1| PROBABLE NITROGEN REGULATORY PROTEIN P-II GLNB [Mycobacterium tuberculosis H37Rv]
>gi|15610057|ref|NP_217436.1| PROBABLE AMMONIUM-TRANSPORT INTEGRAL MEMBRANE PROTEIN AMT [Mycobacterium tuberculosis H37Rv]
>gi|15610058|ref|NP_217437.1| PROBABLE CELL DIVISION PROTEIN FTSY (SRP RECEPTOR) (SIGNAL RECOGNITION PARTICLE RECEPTOR) [Mycobacterium tuberculosis H37Rv]
>gi|57117036|ref|NP_217438.2| PROBABLE CHROMOSOME PARTITION PROTEIN SMC [Mycobacterium tuberculosis H37Rv]
>gi|15610079|ref|NP_217458.1| CONSERVED TRANSMEMBRANE TRANSPORT PROTEIN MMPL7 [Mycobacterium tuberculosis H37Rv]
>gi|15610111|ref|NP_217490.1| CONSERVED HYPOTHETICAL ALANINE RICH PROTEIN [Mycobacterium tuberculosis H37Rv]
>gi|15610114|ref|NP_217493.1| PROBABLE THIAMINE-MONOPHOSPHATE KINASE THIL (THIAMINE-PHOSPHATE KINASE) [Mycobacterium tuberculosis H37Rv]
>gi|15610117|ref|NP_217496.1| POSSIBLE CONSERVED SECRETED PROTEIN [Mycobacterium tuberculosis H37Rv]
>gi|15610120|ref|NP_217499.1| CONSERVED HYPOTHETICAL ALANINE RICH PROTEIN [Mycobacterium tuberculosis H37Rv]
>gi|15610123|ref|NP_217502.1| PROBABLE DNA-BINDING PROTEIN HU HOMOLOG HUPB (HISTONE-LIKE PROTEIN) (HLP) (21-KDA LAMININ-2-BINDING PROTEIN) [Mycobacterium tuberculosis H37Rv]
>gi|15610137|ref|NP_217516.1| POSSIBLE CONSERVED TRANSMEMBRANE PROTEIN [Mycobacterium tuberculosis H37Rv]
>gi|57117045|ref|YP_177918.1| PPE FAMILY PROTEIN [Mycobacterium tuberculosis H37Rv]
>gi|57117047|ref|YP_177919.1| ESAT-6 LIKE PROTEIN ESXS [Mycobacterium tuberculosis H37Rv]
>gi|57117048|ref|YP_177920.1| PPE FAMILY PROTEIN [Mycobacterium tuberculosis H37Rv]
>gi|57117049|ref|YP_177684.1| PPE FAMILY PROTEIN [Mycobacterium tuberculosis H37Rv]
>gi|57117050|ref|YP_177685.1| PE FAMILY PROTEIN [Mycobacterium tuberculosis H37Rv]
>gi|15610163|ref|NP_217542.1| hypothetical protein Rv3026c [Mycobacterium tuberculosis H37Rv]
>gi|15610165|ref|NP_217544.1| PROBABLE ELECTRON TRANSFER FLAVOPROTEIN (ALPHA-SUBUNIT) FIXB (ALPHA-ETF) (ELECTRON TRANSFER FLAVOPROTEIN LARGE SUBUNIT) (ETFLS) [Mycobacterium tuberculosis H37Rv]
>gi|15610176|ref|NP_217555.1| PROBABLE ENOYL-CoA HYDRATASE ECHA17 (CROTONASE) (UNSATURED ACYL-CoA HYDRATASE) (ENOYL HYDRASE) [Mycobacterium tuberculosis H37Rv]
>gi|15610209|ref|NP_217588.1| hypothetical protein Rv3072c [Mycobacterium tuberculosis H37Rv]
>gi|15610211|ref|NP_217590.1| hypothetical protein Rv3074 [Mycobacterium tuberculosis H37Rv]
>gi|15610229|ref|NP_217608.1| PROBABLE CONSERVED INTEGRAL MEMBRANE PROTEIN [Mycobacterium tuberculosis H37Rv]
>gi|15610230|ref|NP_217609.1| HYPOTHETICAL OXIDOREDUCTASE [Mycobacterium tuberculosis H37Rv]
>gi|15610231|ref|NP_217610.1| hypothetical protein Rv3094c [Mycobacterium tuberculosis H37Rv]
>gi|15610235|ref|NP_217614.1| hypothetical protein Rv3098c [Mycobacterium tuberculosis H37Rv]
>gi|15610240|ref|NP_217619.1| HYPOTHETICAL PROLINE-RICH PROTEIN [Mycobacterium tuberculosis H37Rv]
>gi|57117057|ref|YP_177927.1| PROBABLE MOLYBDENUM COFACTOR BIOSYNTHESIS PROTEIN C MOAC1 [Mycobacterium tuberculosis H37Rv]
>gi|15610260|ref|NP_217639.1| hypothetical protein Rv3123 [Mycobacterium tuberculosis H37Rv]
>gi|57117062|ref|YP_177932.1| PPE FAMILY PROTEIN [Mycobacterium tuberculosis H37Rv]
>gi|57117064|ref|YP_177934.1| PPE FAMILY PROTEIN [Mycobacterium tuberculosis H37Rv]
>gi|57117065|ref|YP_177935.1| PPE FAMILY PROTEIN [Mycobacterium tuberculosis H37Rv]
>gi|57117066|ref|YP_177936.1| PPE-FAMILY PROTEIN [Mycobacterium tuberculosis H37Rv]
>gi|15610290|ref|NP_217670.1| PROBABLE NADH DEHYDROGENASE I (CHAIN J) NUOJ (NADH-UBIQUINONE OXIDOREDUCTASE CHAIN J) [Mycobacterium tuberculosis H37Rv]
>gi|57117067|ref|YP_177937.1| PPE FAMILY PROTEIN [Mycobacterium tuberculosis H37Rv]
>gi|15610298|ref|NP_217678.1| POSSIBLE INTEGRAL MEMBRANE PROTEIN [Mycobacterium tuberculosis H37Rv]
>gi|15610302|ref|NP_217682.1| hypothetical protein Rv3166c [Mycobacterium tuberculosis H37Rv]
>gi|15610303|ref|NP_217683.1| PROBABLE TRANSCRIPTIONAL REGULATORY PROTEIN (PROBABLY TETR-FAMILY) [Mycobacterium tuberculosis H37Rv]
>gi|15610316|ref|NP_217696.1| HYPOTHETICAL ALANINE RICH PROTEIN [Mycobacterium tuberculosis H37Rv]
>gi|15610345|ref|NP_217725.1| CONSERVED HYPOTHETICAL THREONIN AND PROLINE RICH PROTEIN [Mycobacterium tuberculosis H37Rv]
>gi|15610353|ref|NP_217733.1| PROBABLE CONSERVED INTEGRAL MEMBRANE PROTEIN [Mycobacterium tuberculosis H37Rv]
>gi|15610358|ref|NP_217738.1| hypothetical protein Rv3222c [Mycobacterium tuberculosis H37Rv]
>gi|15610364|ref|NP_217745.1| hypothetical protein Rv3228 [Mycobacterium tuberculosis H37Rv]
>gi|15610367|ref|NP_217748.1| hypothetical protein Rv3231c [Mycobacterium tuberculosis H37Rv]
>gi|57117079|ref|YP_177949.1| PROBABLE CONSERVED INTEGRAL MEMBRANE TRANSPORT PROTEIN [Mycobacterium tuberculosis H37Rv]
>gi|15610375|ref|NP_217756.1| PROBABLE CONSERVED TRANSMEMBRANE TRANSPORT PROTEIN [Mycobacterium tuberculosis H37Rv]
>gi|15610383|ref|NP_217764.1| PROBABLE THYMIDYLATE KINASE TMK (dTMP KINASE) (THYMIDYLIC ACID KINASE) (TMPK) [Mycobacterium tuberculosis H37Rv]
>gi|15610392|ref|NP_217773.1| hypothetical protein Rv3256c [Mycobacterium tuberculosis H37Rv]
>gi|15610405|ref|NP_217786.1| hypothetical protein Rv3269 [Mycobacterium tuberculosis H37Rv]

>gi|15610407|ref|NP_217788.1| PROBABLE CONSERVED INTEGRAL MEMBRANE PROTEIN [Mycobacterium tuberculosis H37Rv]
>gi|15610411|ref|NP_217792.1| PROBABLE PHOSPHORIBOSYLAMINOIMIDAZOLE CARBOXYLASE CATALYTIC SUBUNIT PURE (AIR CARBOXYLASE) (AIRC) [Mycobacterium tuberculosis H37Rv]
>gi|15610417|ref|NP_217798.1| hypothetical protein Rv3281 [Mycobacterium tuberculosis H37Rv]
>gi|15610444|ref|NP_217825.1| PROBABLE PHOSPHOMANNOMUTASE PMMB (PHOSPHOMANNOSE MUTASE) [Mycobacterium tuberculosis H37Rv]
>gi|15610447|ref|NP_217828.1| hypothetical protein Rv3311 [Mycobacterium tuberculosis H37Rv]
>gi|15610460|ref|NP_217841.1| PROBABLE MOLYBDENUM COFACTOR BIOSYNTHESIS PROTEIN C 3 MOAC3 [Mycobacterium tuberculosis H37Rv]
>gi|15610467|ref|NP_217848.1| PROBABLE SUGAR-TRANSPORT INTEGRAL MEMBRANE PROTEIN SUGI [Mycobacterium tuberculosis H37Rv]
>gi|15610469|ref|NP_217850.1| HYPOTHETICAL PROLINE RICH PROTEIN [Mycobacterium tuberculosis H37Rv]
>gi|57117092|ref|YP_177960.1| PPE FAMILY PROTEIN [Mycobacterium tuberculosis H37Rv]
>gi|57117093|ref|YP_177961.1| PE-PGRS FAMILY PROTEIN [Mycobacterium tuberculosis H37Rv]
>gi|57117094|ref|YP_177962.1| PE-PGRS FAMILY PROTEIN [Mycobacterium tuberculosis H37Rv]
>gi|15610482|ref|NP_217863.1| hypothetical protein Rv3346c [Mycobacterium tuberculosis H37Rv]
>gi|57117095|ref|YP_177963.1| PPE FAMILY PROTEIN [Mycobacterium tuberculosis H37Rv]
>gi|57117096|ref|YP_177964.1| PPE FAMILY PROTEIN [Mycobacterium tuberculosis H37Rv]
>gi|15610490|ref|NP_217871.1| hypothetical protein Rv3354 [Mycobacterium tuberculosis H37Rv]
>gi|15610491|ref|NP_217872.1| hypothetical protein Rv3355c [Mycobacterium tuberculosis H37Rv]
>gi|15610497|ref|NP_217878.1| hypothetical protein Rv3361c [Mycobacterium tuberculosis H37Rv]
>gi|57117098|ref|YP_177965.1| PE-PGRS FAMILY PROTEIN [Mycobacterium tuberculosis H37Rv]
>gi|15610520|ref|NP_217901.1| hypothetical protein Rv3384c [Mycobacterium tuberculosis H37Rv]
>gi|57117101|ref|YP_177968.1| PE-PGRS FAMILY PROTEIN [Mycobacterium tuberculosis H37Rv]
>gi|57117102|ref|NP_217912.2| hypothetical protein Rv3395c [Mycobacterium tuberculosis H37Rv]
>gi|15610533|ref|NP_217914.1| PROBABLE PHYTOENE SYNTHASE PHYA [Mycobacterium tuberculosis H37Rv]
>gi|15610549|ref|NP_217930.1| HYPOTHETICAL ALANINE AND PROLINE RICH PROTEIN [Mycobacterium tuberculosis H37Rv]
>gi|15610551|ref|NP_217932.1| hypothetical protein Rv3415c [Mycobacterium tuberculosis H37Rv]
>gi|15610569|ref|NP_217950.1| hypothetical protein Rv3433c [Mycobacterium tuberculosis H37Rv]
>gi|15610575|ref|NP_217956.1| CONSERVED HYPOTHETICAL ALANINE AND PROLINE RICH PROTEIN [Mycobacterium tuberculosis H37Rv]
>gi|15610580|ref|NP_217961.1| PUTATIVE ESAT-6 LIKE PROTEIN ESXT [Mycobacterium tuberculosis H37Rv]
>gi|15610582|ref|NP_217963.1| HYPOTHETICAL ALANINE AND VALINE RICH PROTEIN [Mycobacterium tuberculosis H37Rv]
>gi|15610584|ref|NP_217965.1| PROBABLE CONSERVED INTEGRAL MEMBRANE PROTEIN [Mycobacterium tuberculosis H37Rv]
>gi|15610592|ref|NP_217973.1| PROBABLE 50S RIBOSOMAL PROTEIN L17 RPLQ [Mycobacterium tuberculosis H37Rv]
>gi|15610602|ref|NP_217983.1| hypothetical protein Rv3466 [Mycobacterium tuberculosis H37Rv]
>gi|57117110|ref|YP_177975.1| PE FAMILY PROTEIN [Mycobacterium tuberculosis H37Rv]
>gi|57117111|ref|YP_177976.1| PE FAMILY PROTEIN [Mycobacterium tuberculosis H37Rv]
>gi|15610617|ref|NP_217998.1| PROBABLE INTEGRAL MEMBRANE PROTEIN [Mycobacterium tuberculosis H37Rv]
>gi|15610629|ref|NP_218010.1| CONSERVED HYPOTHETICAL MCE ASSOCIATED ALANINE AND VALINE RICH PROTEIN [Mycobacterium tuberculosis H37Rv]
>gi|15610630|ref|NP_218011.1| MCE-FAMILY PROTEIN MCE4F [Mycobacterium tuberculosis H37Rv]
>gi|15610636|ref|NP_218017.1| CONSERVED HYPOTHETICAL INTEGRAL MEMBRANE PROTEIN YRBE4B [Mycobacterium tuberculosis H37Rv]
>gi|15610638|ref|NP_218019.1| PROBABLE SHORT-CHAIN TYPE DEHYDROGENASE/REDUCTASE [Mycobacterium tuberculosis H37Rv]
>gi|57117114|ref|YP_177978.1| PE-PGRS FAMILY PROTEIN [Mycobacterium tuberculosis H37Rv]
>gi|57117115|ref|YP_177979.1| PE-PGRS FAMILY PROTEIN [Mycobacterium tuberculosis H37Rv]
>gi|57117116|ref|YP_177980.1| PE-PGRS FAMILY PROTEIN [Mycobacterium tuberculosis H37Rv]
>gi|57117117|ref|YP_177981.1| PE-PGRS FAMILY PROTEIN [Mycobacterium tuberculosis H37Rv]
>gi|57117118|ref|YP_177982.1| PE-PGRS FAMILY PROTEIN [Mycobacterium tuberculosis H37Rv]
>gi|15610661|ref|NP_218042.1| POSSIBLE SIDEROPHORE-BINDING PROTEIN [Mycobacterium tuberculosis H37Rv]
>gi|57117121|ref|YP_177985.1| PPE FAMILY PROTEIN [Mycobacterium tuberculosis H37Rv]
>gi|57117123|ref|YP_177987.1| PPE FAMILY PROTEIN [Mycobacterium tuberculosis H37Rv]
>gi|57117124|ref|YP_177988.1| PPE FAMILY PROTEIN [Mycobacterium tuberculosis H37Rv]
>gi|15610700|ref|NP_218081.1| PROBABLE ACYL-CoA DEHYDROGENASE FADE33 [Mycobacterium tuberculosis H37Rv]
>gi|15610714|ref|NP_218095.1| POSSIBLE ARSENICAL PUMP INTEGRAL MEMBRANE PROTEIN ARSB2 [Mycobacterium tuberculosis H37Rv]
>gi|15610723|ref|NP_218104.1| PROBABLE CONSERVED MEMBRANE PROTEIN [Mycobacterium tuberculosis H37Rv]
>gi|57117129|ref|YP_177993.1| PE-PGRS FAMILY PROTEIN [Mycobacterium tuberculosis H37Rv]
>gi|57117130|ref|YP_177994.1| PE-PGRS FAMILY PROTEIN [Mycobacterium tuberculosis H37Rv]
>gi|15610733|ref|NP_218114.1| PROBABLE IRON-REGULATED LSR2 PROTEIN PRECURSOR [Mycobacterium tuberculosis H37Rv]
>gi|15610738|ref|NP_218119.1| PROBABLE PANTOATE--BETA-ALANINE LIGASE PANC (PANTOTHENATE SYNTHETASE) (PANTOATE ACTIVATING ENZYME) [Mycobacterium tuberculosis H37Rv]
>gi|15610739|ref|NP_218120.1| CONSERVED HYPOTHETICAL ALANINE AND LEUCINE RICH PROTEIN [Mycobacterium tuberculosis H37Rv]
>gi|57117132|ref|NP_218121.2| PROBABLE CONSERVED TRANSMEMBRANE PROTEIN RICH IN ALANINE AND ARGININE AND PROLINE [Mycobacterium tuberculosis H37Rv]
>gi|15610752|ref|NP_218133.1| CONSERVED HYPOTHETICAL ALANINE AND GLYCINE RICH PROTEIN [Mycobacterium tuberculosis H37Rv]
>gi|57117135|ref|YP_177998.1| PPE FAMILY PROTEIN [Mycobacterium tuberculosis H37Rv]

>gi|57117136|ref|YP_177999.1| PE FAMILY PROTEIN [Mycobacterium tuberculosis H37Rv]
>gi|15610763|ref|NP_218144.1| hypothetical protein Rv3627c [Mycobacterium tuberculosis H37Rv]
>gi|15610766|ref|NP_218147.1| PROBABLE CONSERVED INTEGRAL MEMBRANE PROTEIN [Mycobacterium tuberculosis H37Rv]
>gi|15610778|ref|NP_218159.1| hypothetical protein Rv3642c [Mycobacterium tuberculosis H37Rv]
>gi|15610780|ref|NP_218161.1| POSSIBLE DNA POLYMERASE [Mycobacterium tuberculosis H37Rv]
>gi|57117138|ref|YP_178000.1| PE FAMILY PROTEIN [Mycobacterium tuberculosis H37Rv]
>gi|57117139|ref|YP_178001.1| PE-PGRS FAMILY-RELATED PROTEIN [Mycobacterium tuberculosis H37Rv]
>gi|57117140|ref|YP_178002.1| PE-PGRS FAMILY-RELATED PROTEIN [Mycobacterium tuberculosis H37Rv]
>gi|15610790|ref|NP_218171.1| hypothetical protein Rv3654c [Mycobacterium tuberculosis H37Rv]
>gi|15610791|ref|NP_218172.1| hypothetical protein Rv3655c [Mycobacterium tuberculosis H37Rv]
>gi|15610793|ref|NP_218174.1| POSSIBLE CONSERVED ALANINE RICH MEMBRANE PROTEIN [Mycobacterium tuberculosis H37Rv]
>gi|15610794|ref|NP_218175.1| PROBABLE CONSERVED TRANSMEMBRANE PROTEIN [Mycobacterium tuberculosis H37Rv]
>gi|57117141|ref|YP_178003.1| hypothetical protein Rv3659c [Mycobacterium tuberculosis H37Rv]
>gi|15610796|ref|NP_218177.1| hypothetical protein Rv3660c [Mycobacterium tuberculosis H37Rv]
>gi|15610799|ref|NP_218180.1| PROBABLE DIPEPTIDE-TRANSPORT ATP-BINDING PROTEIN ABC TRANSPORTER DPPD [Mycobacterium tuberculosis H37Rv]
>gi|15610811|ref|NP_218192.1| POSSIBLE MEMBRANE PROTEIN [Mycobacterium tuberculosis H37Rv]
>gi|15610822|ref|NP_218203.1| hypothetical protein Rv3686c [Mycobacterium tuberculosis H37Rv]
>gi|15610825|ref|NP_218206.1| PROBABLE CONSERVED TRANSMEMBRANE PROTEIN [Mycobacterium tuberculosis H37Rv]
>gi|15610827|ref|NP_218208.1| hypothetical protein Rv3691 [Mycobacterium tuberculosis H37Rv]
>gi|15610831|ref|NP_218212.1| POSSIBLE CONSERVED MEMBRANE PROTEIN [Mycobacterium tuberculosis H37Rv]
>gi|57117146|ref|YP_178006.1| CONSERVED HYPOTHETICAL PROLINE RICH PROTEIN [Mycobacterium tuberculosis H37Rv]
>gi|15610842|ref|NP_218223.1| CONSERVED HYPOTHETICAL PROLINE RICH PROTEIN [Mycobacterium tuberculosis H37Rv]
>gi|15610848|ref|NP_218229.1| POSSIBLE LIGASE [Mycobacterium tuberculosis H37Rv]
>gi|15610852|ref|NP_218233.1| hypothetical protein Rv3716c [Mycobacterium tuberculosis H37Rv]
>gi|15610859|ref|NP_218240.1| PROBABLE CONSERVED TRANSMEMBRANE PROTEIN [Mycobacterium tuberculosis H37Rv]
>gi|57117149|ref|YP_178008.1| PROBABLE CUTINASE [SECOND PART] CUT5B [Mycobacterium tuberculosis H37Rv]
>gi|57117150|ref|YP_178009.1| PPE FAMILY PROTEIN [Mycobacterium tuberculosis H37Rv]
>gi|57117151|ref|YP_178010.1| PPE FAMILY PROTEIN [Mycobacterium tuberculosis H37Rv]
>gi|57117152|ref|YP_178011.1| PROBABLE PE FAMILY PROTEIN (PE FAMILY-RELATED PROTEIN) [Mycobacterium tuberculosis H37Rv]
>gi|15610889|ref|NP_218270.1| hypothetical protein Rv3753c [Mycobacterium tuberculosis H37Rv]
>gi|15610890|ref|NP_218271.1| PREPHENATE DEHYDROGENASE TYRA (PDH) (HYDROXYPHENYLPYRUVATE SYNTHASE) [Mycobacterium tuberculosis H37Rv]
>gi|15610892|ref|NP_218273.1| POSSIBLE OSMOPROTECTANT (GLYCINE BETAINE/CARNITINE/CHOLINE/L-PROLINE) TRANSPORT INTEGRAL MEMBRANE PROTEIN ABC TRANSPORTER PROZ [Mycobacterium tuberculosis H37Rv]
>gi|15610896|ref|NP_218277.1| POSSIBLE CONSERVED MEMBRANE PROTEIN [Mycobacterium tuberculosis H37Rv]
>gi|15610906|ref|NP_218287.1| HYPOTHETICAL LEUCINE RICH PROTEIN [Mycobacterium tuberculosis H37Rv]
>gi|15610907|ref|NP_218288.1| hypothetical protein Rv3771c [Mycobacterium tuberculosis H37Rv]
>gi|15610915|ref|NP_218296.1| PROBABLE CONSERVED TRANSMEMBRANE PROTEIN ALANINE AND LEUCINE RICH [Mycobacterium tuberculosis H37Rv]
>gi|15610943|ref|NP_218324.1| POSSIBLE CONSERVED TRANSMEMBRANE PROTEIN [Mycobacterium tuberculosis H37Rv]
>gi|15610946|ref|NP_218327.1| EXPORTED REPETITIVE PROTEIN PRECURSOR PIRG (CELL SURFACE PROTEIN) (EXP53) [Mycobacterium tuberculosis H37Rv]
>gi|57117161|ref|YP_178019.1| PE-PGRS FAMILY PROTEIN [Mycobacterium tuberculosis H37Rv]
>gi|15610967|ref|NP_218348.1| hypothetical protein Rv3831 [Mycobacterium tuberculosis H37Rv]
>gi|15610971|ref|NP_218352.1| PROBABLE CONSERVED MEMBRANE PROTEIN [Mycobacterium tuberculosis H37Rv]
>gi|15610973|ref|NP_218354.1| PROBABLE PHOSPHOGLYCERATE MUTASE (PHOSPHOGLYCEROMUTASE) (PHOSPHOGLYCERATE PHOSPHOMUTASE) [Mycobacterium tuberculosis H37Rv]
>gi|15610974|ref|NP_218355.1| POSSIBLE PREPHENATE DEHYDRATASE PHEA [Mycobacterium tuberculosis H37Rv]
>gi|15610979|ref|NP_218360.1| PROBABLE CONSERVED TRANSMEMBRANE PROTEIN [Mycobacterium tuberculosis H37Rv]
>gi|15610984|ref|NP_218365.1| PROBABLE CONSERVED TRANSMEMBRANE PROTEIN [Mycobacterium tuberculosis H37Rv]
>gi|15610986|ref|NP_218367.1| hypothetical protein Rv3850 [Mycobacterium tuberculosis H37Rv]
>gi|15610987|ref|NP_218368.1| POSSIBLE MEMBRANE PROTEIN [Mycobacterium tuberculosis H37Rv]
>gi|15610988|ref|NP_218369.1| POSSIBLE HISTONE-LIKE PROTEIN HNS [Mycobacterium tuberculosis H37Rv]
>gi|15610999|ref|NP_218380.1| HYPOTHETICAL ALANINE RICH PROTEIN [Mycobacterium tuberculosis H37Rv]
>gi|15611000|ref|NP_218381.1| hypothetical protein Rv3864 [Mycobacterium tuberculosis H37Rv]
>gi|15611003|ref|NP_218384.1| hypothetical protein Rv3867 [Mycobacterium tuberculosis H37Rv]
>gi|57117163|ref|YP_178021.1| PE FAMILY-RELATED PROTEIN [Mycobacterium tuberculosis H37Rv]
>gi|57117164|ref|YP_178022.1| PPE FAMILY PROTEIN [Mycobacterium tuberculosis H37Rv]
>gi|15611012|ref|NP_218393.1| CONSERVED HYPOTHETICAL PROLINE AND ALANINE RICH PROTEIN [Mycobacterium tuberculosis H37Rv]
>gi|15611014|ref|NP_218395.1| CONSERVED HYPOTHETICAL ALANINE RICH PROTEIN [Mycobacterium tuberculosis H37Rv]
>gi|15611015|ref|NP_218396.1| HYPOTHETICAL ALANINE AND PROLINE RICH PROTEIN [Mycobacterium tuberculosis H37Rv]
>gi|15611016|ref|NP_218397.1| hypothetical protein Rv3880c [Mycobacterium tuberculosis H37Rv]
>gi|15611017|ref|NP_218398.1| CONSERVED HYPOTHETICAL ALANINE AND GLYCINE RICH PROTEIN [Mycobacterium tuberculosis H37Rv]
>gi|15611022|ref|NP_218403.1| PROBABLE ALANINE AND PROLINE RICH MEMBRANE-ANCHORED MYCOSIN MYCP2 (SERINE PROTEASE) (SUBTILISIN-LIKE PROTEASE) (SUBTILASE-LIKE) (MYCOSIN-2) [Mycobacterium tuberculosis H37Rv]
>gi|15611023|ref|NP_218404.1| PROBABLE CONSERVED TRANSMEMBRANE PROTEIN [Mycobacterium tuberculosis H37Rv]
>gi|57117166|ref|YP_178024.1| PPE FAMILY PROTEIN [Mycobacterium tuberculosis H37Rv]

>gi|57117167|ref|YP_178025.1| PE FAMILY PROTEIN [Mycobacterium tuberculosis H37Rv]
>gi|15611032|ref|NP_218413.1| hypothetical protein Rv3896c [Mycobacterium tuberculosis H37Rv]
>gi|15611033|ref|NP_218414.1| hypothetical protein Rv3897c [Mycobacterium tuberculosis H37Rv]
>gi|15611034|ref|NP_218415.1| hypothetical protein Rv3898c [Mycobacterium tuberculosis H37Rv]
>gi|15611035|ref|NP_218416.1| hypothetical protein Rv3899c [Mycobacterium tuberculosis H37Rv]
>gi|15611037|ref|NP_218418.1| POSSIBLE MEMBRANE PROTEIN [Mycobacterium tuberculosis H37Rv]
>gi|15611039|ref|NP_218420.1| HYPOTHETICAL ALANINE AND PROLINE RICH PROTEIN [Mycobacterium tuberculosis H37Rv]
>gi|15611040|ref|NP_218421.1| PUTATIVE ESAT-6 LIKE PROTEIN ESXE (HYPOTHETICAL ALANINE RICH PROTEIN) (ESAT-6 LIKE PROTEIN 12) [Mycobacterium tuberculosis H37Rv]
>gi|15611041|ref|NP_218422.1| PUTATIVE ESAT-6 LIKE PROTEIN ESXF (HYPOTHETICAL ALANINE AND GLYCINE RICH PROTEIN) (ESAT-6 LIKE PROTEIN 13) [Mycobacterium tuberculosis H37Rv]
>gi|15611042|ref|NP_218423.1| hypothetical protein Rv3906c [Mycobacterium tuberculosis H37Rv]
>gi|15611048|ref|NP_218429.1| HYPOTHETICAL ALANINE RICH PROTEIN [Mycobacterium tuberculosis H37Rv]
>gi|15611056|ref|NP_218437.1| HYPOTHETICAL PROTEIN SIMILAR TO JAG PROTEIN [Mycobacterium tuberculosis H37Rv]
>gi|33862279|ref|NP_893839.1| hypothetical protein PMT0006 [Prochlorococcus marinus str. MIT 9313]
>gi|33862281|ref|NP_893841.1| hypothetical protein PMT0008 [Prochlorococcus marinus str. MIT 9313]
>gi|33862287|ref|NP_893847.1| RNA-binding region RNP-1 (RNA recognition motif) [Prochlorococcus marinus str. MIT 9313]
>gi|33862305|ref|NP_893865.1| Biotin / Lipoyl attachment:Acetyl-CoA biotin carboxyl carrier... [Prochlorococcus marinus str. MIT 9313]
>gi|33862312|ref|NP_893872.1| hypothetical protein PMT0039 [Prochlorococcus marinus str. MIT 9313]
>gi|33862343|ref|NP_893903.1| hypothetical protein PMT0070 [Prochlorococcus marinus str. MIT 9313]
>gi|33862363|ref|NP_893923.1| hypothetical protein PMT0090 [Prochlorococcus marinus str. MIT 9313]
>gi|33862397|ref|NP_893957.1| hypothetical protein PMT0124 [Prochlorococcus marinus str. MIT 9313]
>gi|33862404|ref|NP_893964.1| hypothetical protein PMT0131 [Prochlorococcus marinus str. MIT 9313]
>gi|33862413|ref|NP_893973.1| DedA family; putative alkaline phosphatase-like protein [Prochlorococcus marinus str. MIT 9313]
>gi|33862417|ref|NP_893977.1| hypothetical protein PMT0144 [Prochlorococcus marinus str. MIT 9313]
>gi|33862449|ref|NP_894009.1| hypothetical protein PMT0176 [Prochlorococcus marinus str. MIT 9313]
>gi|33862463|ref|NP_894023.1| possible LysM domain [Prochlorococcus marinus str. MIT 9313]
>gi|33862472|ref|NP_894032.1| hypothetical protein PMT0199 [Prochlorococcus marinus str. MIT 9313]
>gi|33862477|ref|NP_894037.1| possible cobalt transport protein [Prochlorococcus marinus str. MIT 9313]
>gi|33862489|ref|NP_894049.1| hypothetical protein PMT0216 [Prochlorococcus marinus str. MIT 9313]
>gi|33862493|ref|NP_894053.1| Dihydrolipoamide S-acetyltransferase component (E2), pyruvate de [Prochlorococcus marinus str. MIT 9313]
>gi|33862505|ref|NP_894065.1| putative hydrogenase accessory protein [Prochlorococcus marinus str. MIT 9313]
>gi|33862510|ref|NP_894070.1| hypothetical protein PMT0237 [Prochlorococcus marinus str. MIT 9313]
>gi|33862529|ref|NP_894089.1| Hemolysin-type calcium-binding region:RTX N-terminal domain [Prochlorococcus marinus str. MIT 9313]
>gi|33862539|ref|NP_894099.1| putative ABC transporter, oligopeptides [Prochlorococcus marinus str. MIT 9313]
>gi|33862548|ref|NP_894108.1| SAM (and some other nucleotide) binding motif [Prochlorococcus marinus str. MIT 9313]
>gi|33862566|ref|NP_894126.1| hypothetical protein PMT0293 [Prochlorococcus marinus str. MIT 9313]
>gi|33862569|ref|NP_894129.1| TPR repeat [Prochlorococcus marinus str. MIT 9313]
>gi|33862574|ref|NP_894134.1| possible Fanconi anaemia group C protein [Prochlorococcus marinus str. MIT 9313]
>gi|33862589|ref|NP_894149.1| hypothetical protein PMT0316 [Prochlorococcus marinus str. MIT 9313]
>gi|33862601|ref|NP_894161.1| possible bromodomain adjacent to zinc finger domain, 2B... [Prochlorococcus marinus str. MIT 9313]
>gi|33862611|ref|NP_894171.1| marine cyanobacterial conserved hypothetical [Prochlorococcus marinus str. MIT 9313]
>gi|33862616|ref|NP_894176.1| possible apolipoprotein n-acyltransferase [Prochlorococcus marinus str. MIT 9313]
>gi|33862623|ref|NP_894183.1| 30S Ribosomal protein S16 [Prochlorococcus marinus str. MIT 9313]
>gi|33862625|ref|NP_894185.1| hypothetical protein PMT0352 [Prochlorococcus marinus str. MIT 9313]
>gi|33862626|ref|NP_894186.1| possible sodium dependent transporter [Prochlorococcus marinus str. MIT 9313]
>gi|33862634|ref|NP_894194.1| hypothetical protein PMT0361 [Prochlorococcus marinus str. MIT 9313]
>gi|33862645|ref|NP_894205.1| Hemolysin-type calcium-binding protein [Prochlorococcus marinus str. MIT 9313]
>gi|33862675|ref|NP_894235.1| hypothetical protein PMT0402 [Prochlorococcus marinus str. MIT 9313]
>gi|33862694|ref|NP_894254.1| hypothetical protein PMT0421 [Prochlorococcus marinus str. MIT 9313]
>gi|33862705|ref|NP_894265.1| hypothetical protein PMT0432 [Prochlorococcus marinus str. MIT 9313]
>gi|33862709|ref|NP_894269.1| Gamma-glutamyl phosphate reductase [Prochlorococcus marinus str. MIT 9313]
>gi|33862713|ref|NP_894273.1| hypothetical protein PMT0440 [Prochlorococcus marinus str. MIT 9313]
>gi|33862730|ref|NP_894290.1| hypothetical protein PMT0457 [Prochlorococcus marinus str. MIT 9313]
>gi|33862742|ref|NP_894302.1| putative CaCA family, sodium/calcium exchanger [Prochlorococcus marinus str. MIT 9313]
>gi|33862748|ref|NP_894308.1| GCN5-related N-acetyltransferase [Prochlorococcus marinus str. MIT 9313]
>gi|33862756|ref|NP_894316.1| possible Malic enzyme [Prochlorococcus marinus str. MIT 9313]
>gi|33862762|ref|NP_894322.1| hypothetical protein PMT0489 [Prochlorococcus marinus str. MIT 9313]
>gi|33862766|ref|NP_894326.1| hypothetical protein PMT0493 [Prochlorococcus marinus str. MIT 9313]
>gi|33862789|ref|NP_894349.1| Proline-rich region [Prochlorococcus marinus str. MIT 9313]
>gi|33862791|ref|NP_894351.1| CDP-diacylglycerol-glycerol-3-phosphate 3-phosphatidyltransferase [Prochlorococcus marinus str. MIT 9313]
>gi|33862803|ref|NP_894363.1| UDP-3-O-[3-hydroxymyristoyl] glucosamine N-acyltransferase [Prochlorococcus marinus str. MIT 9313]
>gi|33862806|ref|NP_894366.1| putative type 4 prepilin peptidase [Prochlorococcus marinus str. MIT 9313]
>gi|33862813|ref|NP_894373.1| hypothetical protein PMT0540 [Prochlorococcus marinus str. MIT 9313]
>gi|33862815|ref|NP_894375.1| possible Na+/H+ antiporter, CPA1 family [Prochlorococcus marinus str. MIT 9313]
>gi|33862823|ref|NP_894383.1| hypothetical protein PMT0550 [Prochlorococcus marinus str. MIT 9313]
>gi|33862827|ref|NP_894387.1| ABC transporter,membrane component, glycine betaine/proline family [Prochlorococcus marinus str. MIT 9313]
>gi|33862867|ref|NP_894427.1| hypothetical protein PMT0594 [Prochlorococcus marinus str. MIT 9313]
>gi|33862875|ref|NP_894435.1| hypothetical protein PMT0602 [Prochlorococcus marinus str. MIT 9313]
>gi|33862876|ref|NP_894436.1| hypothetical protein PMT0603 [Prochlorococcus marinus str. MIT 9313]

>gi|33862899|ref|NP_894459.1| hypothetical protein PMT0626 [Prochlorococcus marinus str. MIT 9313]
>gi|33862902|ref|NP_894462.1| hypothetical protein PMT0629 [Prochlorococcus marinus str. MIT 9313]
>gi|33862915|ref|NP_894475.1| hypothetical protein PMT0642 [Prochlorococcus marinus str. MIT 9313]
>gi|33862923|ref|NP_894483.1| tRNA/rRNA methyltransferase (SpoU):RNA methyltransferase TrmH... [Prochlorococcus marinus str. MIT 9313]
>gi|33862927|ref|NP_894487.1| putative Anthranilate synthase component II [Prochlorococcus marinus str. MIT 9313]
>gi|33862952|ref|NP_894512.1| hypothetical protein PMT0680 [Prochlorococcus marinus str. MIT 9313]
>gi|33862964|ref|NP_894524.1| ABC transporter component, likely for sugar transport [Prochlorococcus marinus str. MIT 9313]
>gi|33862993|ref|NP_894553.1| hypothetical protein PMT0721 [Prochlorococcus marinus str. MIT 9313]
>gi|33863034|ref|NP_894594.1| possible chloride channel [Prochlorococcus marinus str. MIT 9313]
>gi|33863036|ref|NP_894596.1| hypothetical protein PMT0764 [Prochlorococcus marinus str. MIT 9313]
>gi|33863037|ref|NP_894597.1| hypothetical protein PMT0765 [Prochlorococcus marinus str. MIT 9313]
>gi|33863057|ref|NP_894617.1| hypothetical protein PMT0785 [Prochlorococcus marinus str. MIT 9313]
>gi|33863067|ref|NP_894627.1| hypothetical protein PMT0795 [Prochlorococcus marinus str. MIT 9313]
>gi|33863079|ref|NP_894639.1| hypothetical protein PMT0807 [Prochlorococcus marinus str. MIT 9313]
>gi|33863124|ref|NP_894684.1| possible HAMP domain [Prochlorococcus marinus str. MIT 9313]
>gi|33863131|ref|NP_894691.1| hypothetical protein PMT0859 [Prochlorococcus marinus str. MIT 9313]
>gi|33863156|ref|NP_894716.1| putative RIKEN cDNA 1200003J11; EST AA930106 [Mus muscu... [Prochlorococcus marinus str. MIT 9313]
>gi|33863159|ref|NP_894719.1| possible SAP domain [Prochlorococcus marinus str. MIT 9313]
>gi|33863166|ref|NP_894726.1| ABC transporter for amino acids, membrane component [Prochlorococcus marinus str. MIT 9313]
>gi|33863167|ref|NP_894727.1| hypothetical protein PMT0895 [Prochlorococcus marinus str. MIT 9313]
>gi|33863168|ref|NP_894728.1| ABC transporter for amino acids, membrane component [Prochlorococcus marinus str. MIT 9313]
>gi|33863189|ref|NP_894749.1| hypothetical protein PMT0917 [Prochlorococcus marinus str. MIT 9313]
>gi|33863201|ref|NP_894761.1| Hemolysin-type calcium-binding region:RTX N-terminal domain [Prochlorococcus marinus str. MIT 9313]
>gi|33863210|ref|NP_894770.1| TPR repeat [Prochlorococcus marinus str. MIT 9313]
>gi|33863352|ref|NP_894912.1| RNA-binding protein RbpD [Prochlorococcus marinus str. MIT 9313]
>gi|33863359|ref|NP_894919.1| hypothetical protein PMT1088 [Prochlorococcus marinus str. MIT 9313]
>gi|33863377|ref|NP_894937.1| possible Cytochrome C biogenesis protein trans [Prochlorococcus marinus str. MIT 9313]
>gi|33863384|ref|NP_894944.1| possible Hepatitis C virus core protein [Prochlorococcus marinus str. MIT 9313]
>gi|33863386|ref|NP_894946.1| possible Transcription initiation factor IIA, gam [Prochlorococcus marinus str. MIT 9313]
>gi|33863396|ref|NP_894956.1| Proline-rich region [Prochlorococcus marinus str. MIT 9313]
>gi|33863420|ref|NP_894980.1| Type I antifreeze protein [Prochlorococcus marinus str. MIT 9313]
>gi|33863436|ref|NP_894996.1| putative nicotinamide nucleotide transhydrogenase, subunit alpha 1 (A1) [Prochlorococcus marinus str. MIT 9313]
>gi|33863469|ref|NP_895029.1| carboxysome structural protein CsoS1 [Prochlorococcus marinus str. MIT 9313]
>gi|33863490|ref|NP_895050.1| Domain of unknown function DUF81 [Prochlorococcus marinus str. MIT 9313]
>gi|33863493|ref|NP_895053.1| hypothetical protein PMT1225 [Prochlorococcus marinus str. MIT 9313]
>gi|33863519|ref|NP_895079.1| Possible membrane associated protease [Prochlorococcus marinus str. MIT 9313]
>gi|33863537|ref|NP_895097.1| hypothetical protein PMT1269 [Prochlorococcus marinus str. MIT 9313]
>gi|33863540|ref|NP_895100.1| Putative principal RNA polymerase sigma factor [Prochlorococcus marinus str. MIT 9313]
>gi|33863542|ref|NP_895102.1| hypothetical protein PMT1274 [Prochlorococcus marinus str. MIT 9313]
>gi|33863546|ref|NP_895106.1| possible Paralytic/GBP/PSP peptide [Prochlorococcus marinus str. MIT 9313]
>gi|33863567|ref|NP_895127.1| Band 7 protein [Prochlorococcus marinus str. MIT 9313]
>gi|33863577|ref|NP_895137.1| putative Na+/H+ antiporter, CPA1 family [Prochlorococcus marinus str. MIT 9313]
>gi|33863588|ref|NP_895148.1| hypothetical protein PMT1321 [Prochlorococcus marinus str. MIT 9313]
>gi|33863595|ref|NP_895155.1| probable 4-hydroxybenzoate-octaprenyltransferase [Prochlorococcus marinus str. MIT 9313]
>gi|33863604|ref|NP_895164.1| putative multidrug efflux ABC transporter [Prochlorococcus marinus str. MIT 9313]
>gi|33863607|ref|NP_895167.1| hypothetical protein PMT1340 [Prochlorococcus marinus str. MIT 9313]
>gi|33863618|ref|NP_895178.1| hypothetical protein PMT1351 [Prochlorococcus marinus str. MIT 9313]
>gi|33863635|ref|NP_895195.1| 50S ribosomal protein L34 [Prochlorococcus marinus str. MIT 9313]
>gi|33863648|ref|NP_895208.1| hypothetical protein PMT1381 [Prochlorococcus marinus str. MIT 9313]
>gi|33863651|ref|NP_895211.1| hypothetical protein PMT1384 [Prochlorococcus marinus str. MIT 9313]
>gi|33863655|ref|NP_895215.1| hypothetical protein PMT1388 [Prochlorococcus marinus str. MIT 9313]
>gi|33863668|ref|NP_895228.1| hypothetical protein PMT1401 [Prochlorococcus marinus str. MIT 9313]
>gi|33863682|ref|NP_895242.1| hypothetical protein PMT1415 [Prochlorococcus marinus str. MIT 9313]
>gi|33863714|ref|NP_895274.1| hypothetical protein PMT1447 [Prochlorococcus marinus str. MIT 9313]
>gi|33863726|ref|NP_895286.1| hypothetical protein PMT1459 [Prochlorococcus marinus str. MIT 9313]
>gi|33863729|ref|NP_895289.1| Alanine dehydrogenase [Prochlorococcus marinus str. MIT 9313]
>gi|33863737|ref|NP_895297.1| ATP synthase B/B' CF(0) [Prochlorococcus marinus str. MIT 9313]
>gi|33863738|ref|NP_895298.1| Eubacterial and plasma membrane ATP synthase subunit C:ATP sy... [Prochlorococcus marinus str. MIT 9313]
>gi|33863740|ref|NP_895300.1| possible H+-transporting ATP synthase [Prochlorococcus marinus str. MIT 9313]
>gi|33863743|ref|NP_895303.1| putative c-type cytochrome biogenesis protein CcdA [Prochlorococcus marinus str. MIT 9313]
>gi|33863769|ref|NP_895329.1| Sodium:solute symporter family, possible glucose transporter [Prochlorococcus marinus str. MIT 9313]
>gi|33863785|ref|NP_895345.1| hypothetical protein PMT1518 [Prochlorococcus marinus str. MIT 9313]
>gi|33863795|ref|NP_895355.1| Translation initiation factor IF-2 [Prochlorococcus marinus str. MIT 9313]
>gi|33863797|ref|NP_895357.1| hypothetical protein PMT1530 [Prochlorococcus marinus str. MIT 9313]
>gi|33863806|ref|NP_895366.1| conserved hypothetical membrane protein [Prochlorococcus marinus str. MIT 9313]
>gi|33863811|ref|NP_895371.1| GTP1/OBG family:Hemolysin-type calcium-binding region [Prochlorococcus marinus str. MIT 9313]
>gi|33863815|ref|NP_895375.1| DnaJ3 protein [Prochlorococcus marinus str. MIT 9313]
>gi|33863820|ref|NP_895380.1| possible sodium:solute symporter, ESS family [Prochlorococcus marinus str. MIT 9313]
>gi|33863847|ref|NP_895407.1| hypothetical protein PMT1580 [Prochlorococcus marinus str. MIT 9313]
>gi|33863851|ref|NP_895411.1| putative chromosome segregation protein, SMC ATPase superfamily [Prochlorococcus marinus str. MIT 9313]

>gi|33863864|ref|NP_895424.1| hypothetical protein PMT1597 [Prochlorococcus marinus str. MIT 9313]
>gi|33863894|ref|NP_895454.1| putative sodium/sulfate transporter, DASS family [Prochlorococcus marinus str. MIT 9313]
>gi|33863899|ref|NP_895459.1| hypothetical protein PMT1632 [Prochlorococcus marinus str. MIT 9313]
>gi|33863910|ref|NP_895470.1| hypothetical protein PMT1643 [Prochlorococcus marinus str. MIT 9313]
>gi|33863911|ref|NP_895471.1| possible Zinc finger, C3HC4 type (RING finger) [Prochlorococcus marinus str. MIT 9313]
>gi|33863912|ref|NP_895472.1| possible Glypican [Prochlorococcus marinus str. MIT 9313]
>gi|33863913|ref|NP_895473.1| similar to serum resistance locus BrkB [Prochlorococcus marinus str. MIT 9313]
>gi|33863923|ref|NP_895483.1| hypothetical protein PMT1656 [Prochlorococcus marinus str. MIT 9313]
>gi|33863962|ref|NP_895522.1| TPR repeat [Prochlorococcus marinus str. MIT 9313]
>gi|33863975|ref|NP_895535.1| hypothetical protein PMT1708 [Prochlorococcus marinus str. MIT 9313]
>gi|33863982|ref|NP_895542.1| putative DNA repair and genetic recombination protein RecF [Prochlorococcus marinus str. MIT 9313]
>gi|33863991|ref|NP_895551.1| Prephenate dehydrogenase [Prochlorococcus marinus str. MIT 9313]
>gi|33864015|ref|NP_895575.1| 30S ribosomal protein S5 [Prochlorococcus marinus str. MIT 9313]
>gi|33864038|ref|NP_895598.1| Proline-rich region [Prochlorococcus marinus str. MIT 9313]
>gi|33864060|ref|NP_895620.1| Abortive infection protein [Prochlorococcus marinus str. MIT 9313]
>gi|33864063|ref|NP_895623.1| hypothetical protein PMT1796 [Prochlorococcus marinus str. MIT 9313]
>gi|33864088|ref|NP_895648.1| possible bicarbonate transporter, ICT family [Prochlorococcus marinus str. MIT 9313]
>gi|33864092|ref|NP_895652.1| hypothetical protein PMT1825 [Prochlorococcus marinus str. MIT 9313]
>gi|33864109|ref|NP_895669.1| Pentapeptide repeats [Prochlorococcus marinus str. MIT 9313]
>gi|33864113|ref|NP_895673.1| Glycosyl transferase, family 4 [Prochlorococcus marinus str. MIT 9313]
>gi|33864118|ref|NP_895678.1| hypothetical protein PMT1851 [Prochlorococcus marinus str. MIT 9313]
>gi|33864127|ref|NP_895687.1| two-component sensor histidine kinase [Prochlorococcus marinus str. MIT 9313]
>gi|33864128|ref|NP_895688.1| Cobalamin-5-phosphate synthase CobS [Prochlorococcus marinus str. MIT 9313]
>gi|33864140|ref|NP_895700.1| possible transporter, MFS family [Prochlorococcus marinus str. MIT 9313]
>gi|33864144|ref|NP_895704.1| Hemolysin-type calcium-binding protein [Prochlorococcus marinus str. MIT 9313]
>gi|33864225|ref|NP_895785.1| hypothetical protein PMT1960 [Prochlorococcus marinus str. MIT 9313]
>gi|33864236|ref|NP_895796.1| hypothetical protein PMT1971 [Prochlorococcus marinus str. MIT 9313]
>gi|33864239|ref|NP_895799.1| putative Na+/H+ antiporter, CPA1 family [Prochlorococcus marinus str. MIT 9313]
>gi|33864241|ref|NP_895801.1| hypothetical protein PMT1976 [Prochlorococcus marinus str. MIT 9313]
>gi|33864249|ref|NP_895809.1| hypothetical protein PMT1984 [Prochlorococcus marinus str. MIT 9313]
>gi|33864267|ref|NP_895827.1| RNA-binding region RNP-1 (RNA recognition motif) [Prochlorococcus marinus str. MIT 9313]
>gi|33864283|ref|NP_895843.1| putative NADH Dehydrogenase (complex I) subunit (chain 6) [Prochlorococcus marinus str. MIT 9313]
>gi|33864285|ref|NP_895845.1| putative respiratory-chain NADH dehydrogenase subunit [Prochlorococcus marinus str. MIT 9313]
>gi|33864295|ref|NP_895855.1| Domain of unknown function DUF20 [Prochlorococcus marinus str. MIT 9313]
>gi|33864297|ref|NP_895857.1| Haloacid dehalogenase/epoxide hydrolase family [Prochlorococcus marinus str. MIT 9313]
>gi|33864298|ref|NP_895858.1| hypothetical protein PMT2033 [Prochlorococcus marinus str. MIT 9313]
>gi|33864311|ref|NP_895871.1| hypothetical protein PMT2046 [Prochlorococcus marinus str. MIT 9313]
>gi|33864312|ref|NP_895872.1| hypothetical protein PMT2047 [Prochlorococcus marinus str. MIT 9313]
>gi|33864323|ref|NP_895883.1| putative O-succinylbenzoate synthase [Prochlorococcus marinus str. MIT 9313]
>gi|33864324|ref|NP_895884.1| 1,4-dihydroxy-2-naphthoate (DHNA) octaprenyltransferase; UbiA prenyltranferase family [Prochlorococcus marinus str. MIT 9313]
>gi|33864331|ref|NP_895891.1| Prokaryotic diacylglycerol kinase [Prochlorococcus marinus str. MIT 9313]
>gi|33864337|ref|NP_895897.1| Nicotinate-nucleotide pyrophosphorylase:Quinolinate phosphoriobsyl transferase [Prochlorococcus marinus str. MIT 9313]
>gi|33864344|ref|NP_895904.1| Proline-rich region [Prochlorococcus marinus str. MIT 9313]
>gi|33864350|ref|NP_895910.1| putative preprotein translocase, SecE subunit [Prochlorococcus marinus str. MIT 9313]
>gi|33864355|ref|NP_895915.1| 50S ribosomal protein L7/L12 [Prochlorococcus marinus str. MIT 9313]
>gi|33864360|ref|NP_895920.1| hypothetical protein PMT2096 [Prochlorococcus marinus str. MIT 9313]
>gi|33864363|ref|NP_895923.1| hypothetical protein PMT2099 [Prochlorococcus marinus str. MIT 9313]
>gi|33864365|ref|NP_895925.1| Undecaprenyl-PP-MurNAc-pentapeptide-UDPGlcNAc GlcNAc transferase [Prochlorococcus marinus str. MIT 9313]
>gi|33864372|ref|NP_895932.1| hypothetical protein PMT2108 [Prochlorococcus marinus str. MIT 9313]
>gi|33864380|ref|NP_895940.1| hypothetical protein PMT2116 [Prochlorococcus marinus str. MIT 9313]
>gi|33864392|ref|NP_895952.1| possible (AF314193) Toutatis [Drosophila melanogaster] [Prochlorococcus marinus str. MIT 9313]
>gi|33864417|ref|NP_895977.1| hypothetical protein PMT2153 [Prochlorococcus marinus str. MIT 9313]
>gi|33864465|ref|NP_896025.1| ABC transporter component, possibly Mn transport [Prochlorococcus marinus str. MIT 9313]
>gi|33864468|ref|NP_896028.1| hypothetical protein PMT2204 [Prochlorococcus marinus str. MIT 9313]
>gi|33864471|ref|NP_896031.1| possible permease [Prochlorococcus marinus str. MIT 9313]
>gi|33864474|ref|NP_896034.1| hypothetical protein PMT2210 [Prochlorococcus marinus str. MIT 9313]
>gi|33864526|ref|NP_896086.1| possible NADH-Ubiquinone/plastoquinone (complex I [Prochlorococcus marinus str. MIT 9313]
>gi|33864527|ref|NP_896087.1| hypothetical protein PMT2263 [Prochlorococcus marinus str. MIT 9313]
>gi|73747964|ref|YP_307203.1| conserved hypothetical membrane protein [Dehalococcoides sp. CBDB1]
>gi|73747969|ref|YP_307208.1| hypothetical protein cbdb_A15 [Dehalococcoides sp. CBDB1]
>gi|73748023|ref|YP_307262.1| putative reductive dehalogenase anchoring protein [Dehalococcoides sp. CBDB1]
>gi|73748062|ref|YP_307301.1| probable endonuclease [Dehalococcoides sp. CBDB1]
>gi|73748067|ref|YP_307306.1| ABC transporter, permease protein [Dehalococcoides sp. CBDB1]
>gi|73748084|ref|YP_307323.1| rhomboid family protein [Dehalococcoides sp. CBDB1]
>gi|73748105|ref|YP_307344.1| conserved hypothetical membrane protein [Dehalococcoides sp. CBDB1]
>gi|73748108|ref|YP_307347.1| hypothetical protein cbdb_A183 [Dehalococcoides sp. CBDB1]
>gi|73748132|ref|YP_307371.1| hypothetical membrane protein [Dehalococcoides sp. CBDB1]

>gi|73748146|ref|YP_307385.1| hypothetical protein cbdb_A230 [Dehalococcoides sp. CBDB1]
>gi|73748147|ref|YP_307386.1| hypothetical protein cbdb_A232 [Dehalococcoides sp. CBDB1]
>gi|73748200|ref|YP_307439.1| ribosomal protein S21 [Dehalococcoides sp. CBDB1]
>gi|73748201|ref|YP_307440.1| hypothetical protein cbdb_A294 [Dehalococcoides sp. CBDB1]
>gi|73748250|ref|YP_307489.1| cation channel family protein [Dehalococcoides sp. CBDB1]
>gi|73748342|ref|YP_307581.1| ribosomal protein S11 [Dehalococcoides sp. CBDB1]
>gi|73748349|ref|YP_307588.1| hypothetical protein cbdb_A471 [Dehalococcoides sp. CBDB1]
>gi|73748352|ref|YP_307591.1| hypothetical protein cbdb_A475 [Dehalococcoides sp. CBDB1]
>gi|73748382|ref|YP_307621.1| ribosomal protein L9 [Dehalococcoides sp. CBDB1]
>gi|73748396|ref|YP_307635.1| hypothetical protein cbdb_A531 [Dehalococcoides sp. CBDB1]
>gi|73748398|ref|YP_307637.1| ATP synthase F0, C subunit [Dehalococcoides sp. CBDB1]
>gi|73748413|ref|YP_307652.1| hypothetical protein cbdb_A549 [Dehalococcoides sp. CBDB1]
>gi|73748427|ref|YP_307666.1| hypothetical protein cbdb_A566 [Dehalococcoides sp. CBDB1]
>gi|73748449|ref|YP_307688.1| conserved hypothetical membrane protein [Dehalococcoides sp. CBDB1]
>gi|73748459|ref|YP_307698.1| conserved hypothetical membrane protein [Dehalococcoides sp. CBDB1]
>gi|73748471|ref|YP_307710.1| cation ABC transporter, permease protein [Dehalococcoides sp. CBDB1]
>gi|73748545|ref|YP_307784.1| hypothetical protein cbdb_A706 [Dehalococcoides sp. CBDB1]
>gi|73748566|ref|YP_307805.1| hypothetical protein cbdb_A727 [Dehalococcoides sp. CBDB1]
>gi|73748567|ref|YP_307806.1| hypothetical protein cbdb_A728 [Dehalococcoides sp. CBDB1]
>gi|73748577|ref|YP_307816.1| ATPase, C subunit family protein [Dehalococcoides sp. CBDB1]
>gi|73748633|ref|YP_307872.1| membrane protein, MgtC [Dehalococcoides sp. CBDB1]
>gi|73748691|ref|YP_307930.1| proton-translocating NADH-quinone oxidoreductase, A subunit [Dehalococcoides sp. CBDB1]
>gi|73748757|ref|YP_307996.1| ribosomal protein L7 [Dehalococcoides sp. CBDB1]
>gi|73748789|ref|YP_308028.1| hypothetical protein cbdb_A995 [Dehalococcoides sp. CBDB1]
>gi|73748811|ref|YP_308050.1| hypothetical protein cbdb_A1024 [Dehalococcoides sp. CBDB1]
>gi|73748815|ref|YP_308054.1| hypothetical protein cbdb_A1030 [Dehalococcoides sp. CBDB1]
>gi|73748910|ref|YP_308149.1| hypothetical protein cbdb_A1139 [Dehalococcoides sp. CBDB1]
>gi|73748935|ref|YP_308174.1| hypothetical membrane protein [Dehalococcoides sp. CBDB1]
>gi|73748999|ref|YP_308238.1| hypothetical protein cbdb_A1256 [Dehalococcoides sp. CBDB1]
>gi|73749001|ref|YP_308240.1| hypothetical protein cbdb_A1259 [Dehalococcoides sp. CBDB1]
>gi|73749045|ref|YP_308284.1| general secretion family protein [Dehalococcoides sp. CBDB1]
>gi|73749143|ref|YP_308382.1| integral membrane protein TIGR01906 [Dehalococcoides sp. CBDB1]
>gi|73749146|ref|YP_308385.1| hypothetical protein cbdb_A1440 [Dehalococcoides sp. CBDB1]
>gi|73749158|ref|YP_308397.1| putative reductive dehalogenase anchoring protein [Dehalococcoides sp. CBDB1]
>gi|73749314|ref|YP_308553.1| putative reductive dehalogenase anchoring protein [Dehalococcoides sp. CBDB1]
>gi|73749346|ref|YP_308585.1| putative hydrogenase subunit [Dehalococcoides sp. CBDB1]
>gi|73749374|ref|YP_308613.1| putative twin-arginine translocation protein TatB [Dehalococcoides sp. CBDB1]
>gi|73749394|ref|YP_308633.1| putative ABC-transporter, permease component [Dehalococcoides sp. CBDB1]
>gi|15674263|ref|NP_268436.1| putative secreted protein [Streptococcus pyogenes M1 GAS]
>gi|15674288|ref|NP_268461.1| 50S ribosomal protein L4 [Streptococcus pyogenes M1 GAS]
>gi|15674295|ref|NP_268468.1| 50S ribosomal protein L29 [Streptococcus pyogenes M1 GAS]
>gi|15674360|ref|NP_268534.1| putative V-type Na+ -ATPase subunit E [Streptococcus pyogenes M1 GAS]
>gi|15674373|ref|NP_268547.1| hypothetical protein SPy0168 [Streptococcus pyogenes M1 GAS]
>gi|15674405|ref|NP_268579.1| putative biotin synthase [Streptococcus pyogenes M1 GAS]
>gi|15674436|ref|NP_268610.1| hypothetical protein SPy0256 [Streptococcus pyogenes M1 GAS]
>gi|15674492|ref|NP_268666.1| putative heat shock protein [Streptococcus pyogenes M1 GAS]
>gi|15674522|ref|NP_268696.1| hypothetical protein SPy0374 [Streptococcus pyogenes M1 GAS]
>gi|15674529|ref|NP_268703.1| putative ferrichrome ABC transporter (permease) [Streptococcus pyogenes M1 GAS]
>gi|15674558|ref|NP_268732.1| hypothetical protein SPy0432 [Streptococcus pyogenes M1 GAS]
>gi|15674582|ref|NP_268756.1| putative ribosome recycling factor [Streptococcus pyogenes M1 GAS]
>gi|15674586|ref|NP_268760.1| putative 42 kDa protein [Streptococcus pyogenes M1 GAS]
>gi|15674595|ref|NP_268769.1| conserved hypothetical protein - bacteriocin like peptide associated [Streptococcus pyogenes M1 GAS]
>gi|15674596|ref|NP_268770.1| hypothetical protein SPy0480 [Streptococcus pyogenes M1 GAS]
>gi|15674597|ref|NP_268771.1| conserved hypothetical protein - bacteriocin like peptide associated [Streptococcus pyogenes M1 GAS]
>gi|15674626|ref|NP_268800.1| hypothetical protein SPy0526 [Streptococcus pyogenes M1 GAS]
>gi|15674666|ref|NP_268840.1| hypothetical protein SPy0578 [Streptococcus pyogenes M1 GAS]
>gi|15674670|ref|NP_268844.1| hypothetical protein SPy0583 [Streptococcus pyogenes M1 GAS]
>gi|15674685|ref|NP_268859.1| hypothetical protein SPy0603 [Streptococcus pyogenes M1 GAS]
>gi|15674703|ref|NP_268877.1| putative PTS dependent N-acetyl-galactosamine-IIC component [Streptococcus pyogenes M1 GAS]
>gi|15674764|ref|NP_268938.1| hypothetical protein SPy0703 [Streptococcus pyogenes M1 GAS]
>gi|15674767|ref|NP_268941.1| putative holin, phage associated [Streptococcus pyogenes M1 GAS]
>gi|15674788|ref|NP_268962.1| putative extracellular matrix binding protein [Streptococcus pyogenes M1 GAS]
>gi|15674789|ref|NP_268963.1| streptolysin S associated protein [Streptococcus pyogenes M1 GAS]
>gi|15674794|ref|NP_268968.1| hypothetical protein SPy0743 [Streptococcus pyogenes M1 GAS]
>gi|15674824|ref|NP_268998.1| 30S ribosomal protein S21 [Streptococcus pyogenes M1 GAS]
>gi|15674844|ref|NP_269018.1| hypothetical protein SPy0802 [Streptococcus pyogenes M1 GAS]
>gi|15674856|ref|NP_269030.1| hypothetical protein SPy0815 [Streptococcus pyogenes M1 GAS]
>gi|15674860|ref|NP_269034.1| 50S ribosomal protein L21 [Streptococcus pyogenes M1 GAS]
>gi|15674888|ref|NP_269062.1| putative fructose-specific enzyme II, PTS system BC component [Streptococcus pyogenes M1 GAS]
>gi|15674929|ref|NP_269103.1| hypothetical protein SPy0908 [Streptococcus pyogenes M1 GAS]

>gi|15674982|ref|NP_269156.1| hypothetical protein SPy0970 [Streptococcus pyogenes M1 GAS]
>gi|15674994|ref|NP_269168.1| hypothetical protein SPy0985 [Streptococcus pyogenes M1 GAS]
>gi|15675002|ref|NP_269176.1| putative minor tail protein - phage associated [Streptococcus pyogenes M1 GAS]
>gi|15675020|ref|NP_269194.1| putative ABC transporter (ATP-binding protein) [Streptococcus pyogenes M1 GAS]
>gi|15675044|ref|NP_269218.1| hypothetical protein SPy1049 [Streptococcus pyogenes M1 GAS]
>gi|15675046|ref|NP_269220.1| putative collagen-like protein [Streptococcus pyogenes M1 GAS]
>gi|15675065|ref|NP_269239.1| 50S ribosomal protein L7/L12 [Streptococcus pyogenes M1 GAS]
>gi|15675074|ref|NP_269248.1| conserved hypothetical protein - lantibiotic associated [Streptococcus pyogenes M1 GAS]
>gi|15675075|ref|NP_269249.1| conserved hypothetical protein - lantibiotics associated [Streptococcus pyogenes M1 GAS]
>gi|15675095|ref|NP_269269.1| hypothetical protein SPy1114 [Streptococcus pyogenes M1 GAS]
>gi|15675147|ref|NP_269321.1| hypothetical protein SPy1175 [Streptococcus pyogenes M1 GAS]
>gi|15675154|ref|NP_269328.1| putative decarboxylase, gamma chain [Streptococcus pyogenes M1 GAS]
>gi|15675187|ref|NP_269361.1| hypothetical protein SPy1223 [Streptococcus pyogenes M1 GAS]
>gi|15675196|ref|NP_269370.1| 30S ribosomal protein S20 [Streptococcus pyogenes M1 GAS]
>gi|15675220|ref|NP_269394.1| hypothetical protein SPy1261 [Streptococcus pyogenes M1 GAS]
>gi|15675222|ref|NP_269396.1| hypothetical protein SPy1263 [Streptococcus pyogenes M1 GAS]
>gi|15675224|ref|NP_269398.1| hypothetical protein SPy1265 [Streptococcus pyogenes M1 GAS]
>gi|15675290|ref|NP_269464.1| protein GRAB (protein G-related alpha 2M-binding protein) [Streptococcus pyogenes M1 GAS]
>gi|15675295|ref|NP_269469.1| hypothetical protein SPy1363 [Streptococcus pyogenes M1 GAS]
>gi|15675341|ref|NP_269515.1| hypothetical protein SPy1425 [Streptococcus pyogenes M1 GAS]
>gi|15675347|ref|NP_269521.1| hypothetical protein SPy1437 [Streptococcus pyogenes M1 GAS]
>gi|15675349|ref|NP_269523.1| putative holin - phage associated [Streptococcus pyogenes M1 GAS]
>gi|15675352|ref|NP_269526.1| hypothetical protein SPy1443 [Streptococcus pyogenes M1 GAS]
>gi|15675431|ref|NP_269605.1| hypothetical protein SPy1532 [Streptococcus pyogenes M1 GAS]
>gi|15675433|ref|NP_269607.1| hypothetical protein SPy1534 [Streptococcus pyogenes M1 GAS]
>gi|15675451|ref|NP_269625.1| hypothetical protein SPy1558 [Streptococcus pyogenes M1 GAS]
>gi|15675500|ref|NP_269674.1| hypothetical protein SPy1623 [Streptococcus pyogenes M1 GAS]
>gi|15675506|ref|NP_269680.1| hypothetical protein SPy1630 [Streptococcus pyogenes M1 GAS]
>gi|15675543|ref|NP_269717.1| putative glycerol uptake facilitator [Streptococcus pyogenes M1 GAS]
>gi|15675554|ref|NP_269728.1| hypothetical protein SPy1697 [Streptococcus pyogenes M1 GAS]
>gi|15675578|ref|NP_269752.1| putative ABC transporter (permease) [Streptococcus pyogenes M1 GAS]
>gi|15675585|ref|NP_269759.1| hypothetical protein SPy1736 [Streptococcus pyogenes M1 GAS]
>gi|15675607|ref|NP_269781.1| putative Hsp-70 cofactor [Streptococcus pyogenes M1 GAS]
>gi|15675627|ref|NP_269801.1| hypothetical protein SPy1788 [Streptococcus pyogenes M1 GAS]
>gi|15675632|ref|NP_269806.1| putative ABC transporter (permease) [Streptococcus pyogenes M1 GAS]
>gi|15675684|ref|NP_269858.1| hypothetical protein SPy1865 [Streptococcus pyogenes M1 GAS]
>gi|15675708|ref|NP_269882.1| putative DNA-directed RNA polymerase (delta subunit) [Streptococcus pyogenes M1 GAS]
>gi|15675773|ref|NP_269947.1| collagen-like surface protei [Streptococcus pyogenes M1 GAS]
>gi|15675792|ref|NP_269966.1| hypothetical protein SPy2005 [Streptococcus pyogenes M1 GAS]
>gi|15675795|ref|NP_269969.1| hypothetical protein SPy2009 [Streptococcus pyogenes M1 GAS]
>gi|15675798|ref|NP_269972.1| inhibitor of complement-mediated lysis [Streptococcus pyogenes M1 GAS]
>gi|15675799|ref|NP_269973.1| M protein type 1 [Streptococcus pyogenes M1 GAS]
>gi|15675802|ref|NP_269976.1| immunogenic secreted protein precursor [Streptococcus pyogenes M1 GAS]
>gi|15675808|ref|NP_269982.1| hypothetical protein SPy2033 [Streptococcus pyogenes M1 GAS]
>gi|15675833|ref|NP_270007.1| heat shock protein - cochaperonin [Streptococcus pyogenes M1 GAS]
>gi|15675902|ref|NP_270076.1| putative cadmium resistance protein [Streptococcus pyogenes M1 GAS]
>gi|15675912|ref|NP_270086.1| hypothetical protein SPy2176 [Streptococcus pyogenes M1 GAS]
>gi|15675918|ref|NP_270092.1| 50S ribosomal protein L9 [Streptococcus pyogenes M1 GAS]
>gi|71891796|ref|YP_277525.1| ATP synthase, F0 sector, subunit c [Candidatus Blochmannia pennsylvanicus str. BPEN]
>gi|71891811|ref|YP_277540.1| probable transmembrane protein [Candidatus Blochmannia pennsylvanicus str. BPEN]
>gi|71891845|ref|YP_277574.1| putative membrane protein [Candidatus Blochmannia pennsylvanicus str. BPEN]
>gi|71891881|ref|YP_277611.1| 50S ribosomal subunit protein L21 [Candidatus Blochmannia pennsylvanicus str. BPEN]
>gi|71891894|ref|YP_277624.1| 30S ribosomal subunit protein S15 [Candidatus Blochmannia pennsylvanicus str. BPEN]
>gi|71891922|ref|YP_277652.1| cell division protein [Candidatus Blochmannia pennsylvanicus str. BPEN]
>gi|71891971|ref|YP_277701.1| biotin-[acetylCoA carboxylase] holoenzyme synthetase [Candidatus Blochmannia pennsylvanicus str. BPEN]
>gi|71891993|ref|YP_277723.1| 50S ribosomal subunit protein L15 [Candidatus Blochmannia pennsylvanicus str. BPEN]
>gi|71892063|ref|YP_277793.1| UDP-3-O-(3-hydroxymyristoyl)-glucosamine N-acyltransferase [Candidatus Blochmannia pennsylvanicus str. BPEN]
>gi|71892078|ref|YP_277808.1| rod shape-determining protein [Candidatus Blochmannia pennsylvanicus str. BPEN]
>gi|71892115|ref|YP_277847.1| TolA [Candidatus Blochmannia pennsylvanicus str. BPEN]
>gi|71892132|ref|YP_277864.1| 50S ribosomal subunit protein L35 [Candidatus Blochmannia pennsylvanicus str. BPEN]
>gi|71892206|ref|YP_277939.1| putative membrane protein, transport [Candidatus Blochmannia pennsylvanicus str. BPEN]
>gi|71892257|ref|YP_277991.1| NADH dehydrogenase I chain K [Candidatus Blochmannia pennsylvanicus str. BPEN]
>gi|49474841|ref|YP_032882.1| Lipoprotein signal peptidase [Bartonella henselae str. Houston-1]
>gi|49474918|ref|YP_032959.1| hypothetical protein BH00960 [Bartonella henselae str. Houston-1]
>gi|49474923|ref|YP_032964.1| hypothetical protein BH01030 [Bartonella henselae str. Houston-1]
>gi|49474932|ref|YP_032973.1| Heme exporter protein B [Bartonella henselae str. Houston-1]
>gi|49474943|ref|YP_032984.1| Cytochrome o ubiquinol oxidase subunit IV [Bartonella henselae str. Houston-1]
>gi|49474950|ref|YP_032991.1| 30S ribosomal protein s21 [Bartonella henselae str. Houston-1]
>gi|49475088|ref|YP_033129.1| Biotin synthase [Bartonella henselae str. Houston-1]

>gi|49475099|ref|YP_033140.1| hypothetical prophage protein [Bartonella henselae str. Houston-1]
>gi|49475117|ref|YP_033158.1| hypothetical prophage protein [Bartonella henselae str. Houston-1]
>gi|49475122|ref|YP_033163.1| Phage-related baseplate assembly protein [Bartonella henselae str. Houston-1]
>gi|49475133|ref|YP_033174.1| hypothetical protein BH03310 [Bartonella henselae str. Houston-1]
>gi|49475143|ref|YP_033184.1| hypothetical prophage protein [Bartonella henselae str. Houston-1]
>gi|49475171|ref|YP_033212.1| Phage related protein [Bartonella henselae str. Houston-1]
>gi|49475186|ref|YP_033227.1| Large-conductance mechanosensitive channel [Bartonella henselae str. Houston-1]
>gi|49475212|ref|YP_033253.1| ATP synthase B chain [Bartonella henselae str. Houston-1]
>gi|49475298|ref|YP_033339.1| DNA-directed RNA polymerase omega subunit [Bartonella henselae str. Houston-1]
>gi|49475317|ref|YP_033358.1| Phosphatidylserine synthase [Bartonella henselae str. Houston-1]
>gi|49475320|ref|YP_033361.1| Colicin v production protein [Bartonella henselae str. Houston-1]
>gi|49475362|ref|YP_033403.1| Protein-export membrane protein [Bartonella henselae str. Houston-1]
>gi|49475397|ref|YP_033438.1| 50S ribosomal protein l7 /l12 [Bartonella henselae str. Houston-1]
>gi|49475424|ref|YP_033465.1| ABC transporter, permease protein [Bartonella henselae str. Houston-1]
>gi|49475472|ref|YP_033513.1| phage related protein [Bartonella henselae str. Houston-1]
>gi|49475619|ref|YP_033660.1| hypothetical protein BH08560 [Bartonella henselae str. Houston-1]
>gi|49475649|ref|YP_033690.1| NADH dehydrogenase I, J subunit [Bartonella henselae str. Houston-1]
>gi|49475676|ref|YP_033717.1| hypothetical genomic island protein [Bartonella henselae str. Houston-1]
>gi|49475682|ref|YP_033723.1| hypothetical genomic island protein [Bartonella henselae str. Houston-1]
>gi|49475686|ref|YP_033727.1| hypothetical genomic island protein [Bartonella henselae str. Houston-1]
>gi|49475819|ref|YP_033860.1| Potassium-efflux system protein [Bartonella henselae str. Houston-1]
>gi|49475882|ref|YP_033923.1| hypothetical protein BH11570 [Bartonella henselae str. Houston-1]
>gi|49475896|ref|YP_033937.1| hypothetical protein BH11730 [Bartonella henselae str. Houston-1]
>gi|49475939|ref|YP_033980.1| hypothetical protein BH12250 [Bartonella henselae str. Houston-1]
>gi|49475999|ref|YP_034040.1| hypothetical protein BH13120 [Bartonella henselae str. Houston-1]
>gi|49476013|ref|YP_034054.1| 17-kDa antigen precursor [Bartonella henselae str. Houston-1]
>gi|49476025|ref|YP_034066.1| hypothetical protein BH13410 [Bartonella henselae str. Houston-1]
>gi|49476027|ref|YP_034068.1| hypothetical protein BH13430 [Bartonella henselae str. Houston-1]
>gi|49476042|ref|YP_034083.1| hypothetical protein BH13620 [Bartonella henselae str. Houston-1]
>gi|49476079|ref|YP_034120.1| hypothetical protein BH14150 [Bartonella henselae str. Houston-1]
>gi|49476081|ref|YP_034122.1| hypothetical protein BH14170 [Bartonella henselae str. Houston-1]
>gi|49476086|ref|YP_034127.1| hypothetical protein BH14220 [Bartonella henselae str. Houston-1]
>gi|49476105|ref|YP_034146.1| hypothetical protein BH14430 [Bartonella henselae str. Houston-1]
>gi|49476112|ref|YP_034153.1| hypothetical protein BH14510 [Bartonella henselae str. Houston-1]
>gi|49476113|ref|YP_034154.1| phage related protein [Bartonella henselae str. Houston-1]
>gi|49476136|ref|YP_034177.1| hypothetical protein BH14780 [Bartonella henselae str. Houston-1]
>gi|49476153|ref|YP_034194.1| hypothetical protein BH14960 [Bartonella henselae str. Houston-1]
>gi|49476173|ref|YP_034214.1| hypothetical protein BH15180 [Bartonella henselae str. Houston-1]
>gi|49476219|ref|YP_034260.1| trwL8 protein [Bartonella henselae str. Houston-1]
>gi|21229485|ref|NP_635402.1| hypothetical protein XCC0007 [Xanthomonas campestris pv. campestris str. ATCC 33913]
>gi|21229486|ref|NP_635403.1| TonB protein [Xanthomonas campestris pv. campestris str. ATCC 33913]
>gi|21229491|ref|NP_635408.1| hypothetical protein XCC0013 [Xanthomonas campestris pv. campestris str. ATCC 33913]
>gi|21229496|ref|NP_635413.1| hypothetical protein XCC0018 [Xanthomonas campestris pv. campestris str. ATCC 33913]
>gi|21229499|ref|NP_635416.1| carboxyl-terminal protease [Xanthomonas campestris pv. campestris str. ATCC 33913]
>gi|21229500|ref|NP_635417.1| hypothetical protein XCC0022 [Xanthomonas campestris pv. campestris str. ATCC 33913]
>gi|21229501|ref|NP_635418.1| hypothetical protein XCC0023 [Xanthomonas campestris pv. campestris str. ATCC 33913]
>gi|21229511|ref|NP_635428.1| hypothetical protein XCC0033 [Xanthomonas campestris pv. campestris str. ATCC 33913]
>gi|21229518|ref|NP_635435.1| transcriptional regulator lysR family [Xanthomonas campestris pv. campestris str. ATCC 33913]
>gi|21229524|ref|NP_635441.1| ankyrin-like protein [Xanthomonas campestris pv. campestris str. ATCC 33913]
>gi|21229525|ref|NP_635442.1| hypothetical protein XCC0047 [Xanthomonas campestris pv. campestris str. ATCC 33913]
>gi|21229547|ref|NP_635464.1| hypothetical protein XCC0069 [Xanthomonas campestris pv. campestris str. ATCC 33913]
>gi|21229550|ref|NP_635467.1| hypothetical protein XCC0072 [Xanthomonas campestris pv. campestris str. ATCC 33913]
>gi|21229551|ref|NP_635468.1| hypothetical protein XCC0073 [Xanthomonas campestris pv. campestris str. ATCC 33913]
>gi|21229557|ref|NP_635474.1| hypothetical protein XCC0079 [Xanthomonas campestris pv. campestris str. ATCC 33913]
>gi|21229564|ref|NP_635481.1| hypothetical protein XCC0086 [Xanthomonas campestris pv. campestris str. ATCC 33913]
>gi|21229565|ref|NP_635482.1| hypothetical protein XCC0087 [Xanthomonas campestris pv. campestris str. ATCC 33913]
>gi|21229566|ref|NP_635483.1| hypothetical protein XCC0088 [Xanthomonas campestris pv. campestris str. ATCC 33913]
>gi|21229567|ref|NP_635484.1| hypothetical protein XCC0089 [Xanthomonas campestris pv. campestris str. ATCC 33913]
>gi|21229583|ref|NP_635500.1| ATP-dependent DNA ligase [Xanthomonas campestris pv. campestris str. ATCC 33913]
>gi|21229586|ref|NP_635503.1| two-component system sensor protein [Xanthomonas campestris pv. campestris str. ATCC 33913]
>gi|21229592|ref|NP_635509.1| transcriptional regulator [Xanthomonas campestris pv. campestris str. ATCC 33913]
>gi|21229602|ref|NP_635519.1| hypothetical protein XCC0124 [Xanthomonas campestris pv. campestris str. ATCC 33913]
>gi|21229615|ref|NP_635532.1| hypothetical protein XCC0137 [Xanthomonas campestris pv. campestris str. ATCC 33913]
>gi|21229618|ref|NP_635535.1| hypothetical protein XCC0140 [Xanthomonas campestris pv. campestris str. ATCC 33913]
>gi|21229620|ref|NP_635537.1| hypothetical protein XCC0142 [Xanthomonas campestris pv. campestris str. ATCC 33913]
>gi|21229626|ref|NP_635543.1| C4-dicarboxylate transport protein [Xanthomonas campestris pv. campestris str. ATCC 33913]
>gi|21229650|ref|NP_635567.1| hypothetical protein XCC0172 [Xanthomonas campestris pv. campestris str. ATCC 33913]
>gi|21229661|ref|NP_635578.1| hypothetical protein XCC0183 [Xanthomonas campestris pv. campestris str. ATCC 33913]
>gi|21229665|ref|NP_635582.1| ammonium transporter [Xanthomonas campestris pv. campestris str. ATCC 33913]
>gi|21229666|ref|NP_635583.1| two-component system sensor protein [Xanthomonas campestris pv. campestris str. ATCC 33913]

>gi|21229676|ref|NP_635593.1| uroporphyrinogen-III synthase [Xanthomonas campestris pv. campestris str. ATCC 33913]
>gi|21229678|ref|NP_635595.1| hypothetical protein XCC0200 [Xanthomonas campestris pv. campestris str. ATCC 33913]
>gi|21229679|ref|NP_635596.1| hypothetical protein XCC0201 [Xanthomonas campestris pv. campestris str. ATCC 33913]
>gi|21229686|ref|NP_635603.1| MFS transporter [Xanthomonas campestris pv. campestris str. ATCC 33913]
>gi|21229688|ref|NP_635605.1| hypothetical protein XCC0210 [Xanthomonas campestris pv. campestris str. ATCC 33913]
>gi|21229699|ref|NP_635616.1| acetyltransferase, GNAT family [Xanthomonas campestris pv. campestris str. ATCC 33913]
>gi|21229704|ref|NP_635621.1| hypothetical protein XCC0226 [Xanthomonas campestris pv. campestris str. ATCC 33913]
>gi|21229727|ref|NP_635644.1| hypothetical protein XCC0249 [Xanthomonas campestris pv. campestris str. ATCC 33913]
>gi|21229728|ref|NP_635645.1| hypothetical protein XCC0250 [Xanthomonas campestris pv. campestris str. ATCC 33913]
>gi|21229732|ref|NP_635649.1| cytochrome C5 [Xanthomonas campestris pv. campestris str. ATCC 33913]
>gi|21229734|ref|NP_635651.1| hypothetical protein XCC0256 [Xanthomonas campestris pv. campestris str. ATCC 33913]
>gi|21229736|ref|NP_635653.1| hypothetical protein XCC0258 [Xanthomonas campestris pv. campestris str. ATCC 33913]
>gi|21229737|ref|NP_635654.1| integral membrane protein [Xanthomonas campestris pv. campestris str. ATCC 33913]
>gi|21229752|ref|NP_635669.1| hypothetical protein XCC0274 [Xanthomonas campestris pv. campestris str. ATCC 33913]
>gi|21229753|ref|NP_635670.1| ATP-dependent RNA helicase [Xanthomonas campestris pv. campestris str. ATCC 33913]
>gi|21229754|ref|NP_635671.1| chemotaxis protein [Xanthomonas campestris pv. campestris str. ATCC 33913]
>gi|21229756|ref|NP_635673.1| hypothetical protein XCC0278 [Xanthomonas campestris pv. campestris str. ATCC 33913]
>gi|21229766|ref|NP_635683.1| transcriptional regulator [Xanthomonas campestris pv. campestris str. ATCC 33913]
>gi|21229770|ref|NP_635687.1| Glu-tRNAGln amidotransferase A subunit [Xanthomonas campestris pv. campestris str. ATCC 33913]
>gi|21229773|ref|NP_635690.1| xanthine/uracil permease [Xanthomonas campestris pv. campestris str. ATCC 33913]
>gi|21229774|ref|NP_635691.1| vanillate O-demethylase oxidoreductase [Xanthomonas campestris pv. campestris str. ATCC 33913]
>gi|21229780|ref|NP_635697.1| MFS transporter [Xanthomonas campestris pv. campestris str. ATCC 33913]
>gi|21229789|ref|NP_635706.1| transcriptional regulator [Xanthomonas campestris pv. campestris str. ATCC 33913]
>gi|21229799|ref|NP_635716.1| oxidoreductase [Xanthomonas campestris pv. campestris str. ATCC 33913]
>gi|21229801|ref|NP_635718.1| hypothetical protein XCC0323 [Xanthomonas campestris pv. campestris str. ATCC 33913]
>gi|21229802|ref|NP_635719.1| chemotaxis protein [Xanthomonas campestris pv. campestris str. ATCC 33913]
>gi|21229809|ref|NP_635726.1| hypothetical protein XCC0331 [Xanthomonas campestris pv. campestris str. ATCC 33913]
>gi|21229827|ref|NP_635744.1| MFS transporter [Xanthomonas campestris pv. campestris str. ATCC 33913]
>gi|21229847|ref|NP_635764.1| 3-carboxy-cis,cis-muconate cycloisomerase [Xanthomonas campestris pv. campestris str. ATCC 33913]
>gi|21229856|ref|NP_635773.1| hypothetical protein XCC0378 [Xanthomonas campestris pv. campestris str. ATCC 33913]
>gi|21229857|ref|NP_635774.1| threonine dehydratase [Xanthomonas campestris pv. campestris str. ATCC 33913]
>gi|21229863|ref|NP_635780.1| biotin biosynthesis protein [Xanthomonas campestris pv. campestris str. ATCC 33913]
>gi|21229867|ref|NP_635784.1| competence protein F [Xanthomonas campestris pv. campestris str. ATCC 33913]
>gi|21229884|ref|NP_635801.1| hypothetical protein XCC0407 [Xanthomonas campestris pv. campestris str. ATCC 33913]
>gi|21229896|ref|NP_635813.1| outer membrane protein [Xanthomonas campestris pv. campestris str. ATCC 33913]
>gi|21229898|ref|NP_635815.1| component of multidrug efflux system [Xanthomonas campestris pv. campestris str. ATCC 33913]
>gi|21229904|ref|NP_635821.1| dihydrolipoamide acyltransferase [Xanthomonas campestris pv. campestris str. ATCC 33913]
>gi|21229911|ref|NP_635828.1| hypothetical protein XCC0434 [Xanthomonas campestris pv. campestris str. ATCC 33913]
>gi|21229916|ref|NP_635833.1| hypothetical protein XCC0440 [Xanthomonas campestris pv. campestris str. ATCC 33913]
>gi|21229917|ref|NP_635834.1| hypothetical protein XCC0439 [Xanthomonas campestris pv. campestris str. ATCC 33913]
>gi|21229921|ref|NP_635838.1| PhaD protein [Xanthomonas campestris pv. campestris str. ATCC 33913]
>gi|21229925|ref|NP_635842.1| hypothetical protein XCC0448 [Xanthomonas campestris pv. campestris str. ATCC 33913]
>gi|21229928|ref|NP_635845.1| hypothetical protein XCC0451 [Xanthomonas campestris pv. campestris str. ATCC 33913]
>gi|21229931|ref|NP_635848.1| hypothetical protein XCC0454 [Xanthomonas campestris pv. campestris str. ATCC 33913]
>gi|21229937|ref|NP_635854.1| threonine aldolase [Xanthomonas campestris pv. campestris str. ATCC 33913]
>gi|21229951|ref|NP_635868.1| SugE protein [Xanthomonas campestris pv. campestris str. ATCC 33913]
>gi|21229960|ref|NP_635877.1| hypothetical protein XCC0485 [Xanthomonas campestris pv. campestris str. ATCC 33913]
>gi|21229962|ref|NP_635879.1| hypothetical protein XCC0487 [Xanthomonas campestris pv. campestris str. ATCC 33913]
>gi|21229965|ref|NP_635882.1| transmembrane protein [Xanthomonas campestris pv. campestris str. ATCC 33913]
>gi|21229970|ref|NP_635887.1| MFS transporter [Xanthomonas campestris pv. campestris str. ATCC 33913]
>gi|21229976|ref|NP_635893.1| hypothetical protein XCC0501 [Xanthomonas campestris pv. campestris str. ATCC 33913]
>gi|21229978|ref|NP_635895.1| hypothetical protein XCC0503 [Xanthomonas campestris pv. campestris str. ATCC 33913]
>gi|21229979|ref|NP_635896.1| CDP-diacylglycerol-glycerol-3-phosphate 3-phosphatidyltransferase-related protein [Xanthomonas campestris pv. campestris str. ATCC 33913]
>gi|21229982|ref|NP_635899.1| ice nucleation protein [Xanthomonas campestris pv. campestris str. ATCC 33913]
>gi|21229984|ref|NP_635901.1| hypothetical protein XCC0509 [Xanthomonas campestris pv. campestris str. ATCC 33913]
>gi|21229986|ref|NP_635903.1| hypothetical protein XCC0511 [Xanthomonas campestris pv. campestris str. ATCC 33913]
>gi|21229988|ref|NP_635905.1| hypothetical protein XCC0513 [Xanthomonas campestris pv. campestris str. ATCC 33913]
>gi|21229992|ref|NP_635909.1| biotin carboxyl carrier protein of acetyl-CoA [Xanthomonas campestris pv. campestris str. ATCC 33913]
>gi|21229994|ref|NP_635911.1| c-type cytochrome biogenesis protein [Xanthomonas campestris pv. campestris str. ATCC 33913]
>gi|21230022|ref|NP_635939.1| hypothetical protein XCC0547 [Xanthomonas campestris pv. campestris str. ATCC 33913]
>gi|21230026|ref|NP_635943.1| ATP synthase delta chain [Xanthomonas campestris pv. campestris str. ATCC 33913]
>gi|21230031|ref|NP_635948.1| chorismate mutase/prephenate dehydratase [Xanthomonas campestris pv. campestris str. ATCC 33913]
>gi|21230039|ref|NP_635956.1| hypothetical protein XCC0564 [Xanthomonas campestris pv. campestris str. ATCC 33913]
>gi|21230051|ref|NP_635968.1| copper resistance protein B precursor [Xanthomonas campestris pv. campestris str. ATCC 33913]
>gi|21230053|ref|NP_635970.1| hypothetical protein XCC0579 [Xanthomonas campestris pv. campestris str. ATCC 33913]
>gi|21230062|ref|NP_635979.1| dethiobiotin synthetase [Xanthomonas campestris pv. campestris str. ATCC 33913]
>gi|21230069|ref|NP_635986.1| outer membrane protein [Xanthomonas campestris pv. campestris str. ATCC 33913]
>gi|21230070|ref|NP_635987.1| outer membrane protein [Xanthomonas campestris pv. campestris str. ATCC 33913]
>gi|21230090|ref|NP_636007.1| bifunctional acetyl transferase/isomerase [Xanthomonas campestris pv. campestris str. ATCC 33913]

>gi|21230094|ref|NP_636011.1| electron transfer flavoprotein alpha subunit [Xanthomonas campestris pv. campestris str. ATCC 33913]
>gi|21230109|ref|NP_636026.1| ABC transporter ATP-binding protein [Xanthomonas campestris pv. campestris str. ATCC 33913]
>gi|21230112|ref|NP_636029.1| DNA-binding protein [Xanthomonas campestris pv. campestris str. ATCC 33913]
>gi|21230117|ref|NP_636034.1| hypothetical protein XCC0642 [Xanthomonas campestris pv. campestris str. ATCC 33913]
>gi|21230133|ref|NP_636050.1| outer membrane protein [Xanthomonas campestris pv. campestris str. ATCC 33913]
>gi|21230138|ref|NP_636055.1| general secretion pathway protein H [Xanthomonas campestris pv. campestris str. ATCC 33913]
>gi|21230142|ref|NP_636059.1| general secretion pathway protein L [Xanthomonas campestris pv. campestris str. ATCC 33913]
>gi|21230144|ref|NP_636061.1| general secretion pathway protein N [Xanthomonas campestris pv. campestris str. ATCC 33913]
>gi|21230150|ref|NP_636067.1| hypothetical protein XCC0675 [Xanthomonas campestris pv. campestris str. ATCC 33913]
>gi|21230154|ref|NP_636071.1| hypothetical protein XCC0679 [Xanthomonas campestris pv. campestris str. ATCC 33913]
>gi|21230157|ref|NP_636074.1| cellulose synthase subunit C [Xanthomonas campestris pv. campestris str. ATCC 33913]
>gi|21230160|ref|NP_636077.1| hypothetical protein XCC0685 [Xanthomonas campestris pv. campestris str. ATCC 33913]
>gi|21230164|ref|NP_636081.1| hypothetical protein XCC0689 [Xanthomonas campestris pv. campestris str. ATCC 33913]
>gi|21230167|ref|NP_636084.1| acetyltransferase [Xanthomonas campestris pv. campestris str. ATCC 33913]
>gi|21230178|ref|NP_636095.1| potassium-transporting ATPase B chain [Xanthomonas campestris pv. campestris str. ATCC 33913]
>gi|21230180|ref|NP_636097.1| two-component system sensor protein [Xanthomonas campestris pv. campestris str. ATCC 33913]
>gi|21230185|ref|NP_636102.1| hypothetical protein XCC0710 [Xanthomonas campestris pv. campestris str. ATCC 33913]
>gi|21230186|ref|NP_636103.1| hypothetical protein XCC0711 [Xanthomonas campestris pv. campestris str. ATCC 33913]
>gi|21230196|ref|NP_636113.1| UDP-N-acetylmuramoylalanyl-D-glutamyl-2, 6-diaminopimelate-D-alanyl-D-alanyl ligase [Xanthomonas campestris pv. campestris str. ATCC 33913]
>gi|21230199|ref|NP_636116.1| UDP-N-acetylglucosamine-N- acetylmuramyl-(pentapeptide) pyrophosphoryl-undecaprenol [Xanthomonas campestris pv. campestris str. ATCC 33913]
>gi|21230207|ref|NP_636124.1| peptidase [Xanthomonas campestris pv. campestris str. ATCC 33913]
>gi|21230212|ref|NP_636129.1| hypothetical protein XCC0738 [Xanthomonas campestris pv. campestris str. ATCC 33913]
>gi|21230217|ref|NP_636134.1| hypothetical protein XCC0743 [Xanthomonas campestris pv. campestris str. ATCC 33913]
>gi|21230220|ref|NP_636137.1| hypothetical protein XCC0746 [Xanthomonas campestris pv. campestris str. ATCC 33913]
>gi|21230232|ref|NP_636149.1| hypothetical protein XCC0758 [Xanthomonas campestris pv. campestris str. ATCC 33913]
>gi|21230238|ref|NP_636155.1| ribokinase [Xanthomonas campestris pv. campestris str. ATCC 33913]
>gi|21230243|ref|NP_636160.1| hypothetical protein XCC0769 [Xanthomonas campestris pv. campestris str. ATCC 33913]
>gi|21230250|ref|NP_636167.1| oxidoreductase [Xanthomonas campestris pv. campestris str. ATCC 33913]
>gi|21230256|ref|NP_636173.1| diacylglycerol kinase [Xanthomonas campestris pv. campestris str. ATCC 33913]
>gi|21230258|ref|NP_636175.1| glycine rich protein [Xanthomonas campestris pv. campestris str. ATCC 33913]
>gi|21230293|ref|NP_636210.1| integral membrane protein [Xanthomonas campestris pv. campestris str. ATCC 33913]
>gi|21230319|ref|NP_636236.1| hypothetical protein XCC0845 [Xanthomonas campestris pv. campestris str. ATCC 33913]
>gi|21230320|ref|NP_636237.1| hypothetical protein XCC0846 [Xanthomonas campestris pv. campestris str. ATCC 33913]
>gi|21230322|ref|NP_636239.1| hypothetical protein XCC0848 [Xanthomonas campestris pv. campestris str. ATCC 33913]
>gi|21230325|ref|NP_636242.1| extracellular protease [Xanthomonas campestris pv. campestris str. ATCC 33913]
>gi|21230326|ref|NP_636243.1| extracellular protease [Xanthomonas campestris pv. campestris str. ATCC 33913]
>gi|21230332|ref|NP_636249.1| hypothetical protein XCC0858 [Xanthomonas campestris pv. campestris str. ATCC 33913]
>gi|21230335|ref|NP_636252.1| thioredoxin [Xanthomonas campestris pv. campestris str. ATCC 33913]
>gi|21230343|ref|NP_636260.1| hypothetical protein XCC0869 [Xanthomonas campestris pv. campestris str. ATCC 33913]
>gi|21230356|ref|NP_636273.1| 50S ribosomal protein L7/L12 [Xanthomonas campestris pv. campestris str. ATCC 33913]
>gi|21230366|ref|NP_636283.1| 50S ribosomal protein L23 [Xanthomonas campestris pv. campestris str. ATCC 33913]
>gi|21230383|ref|NP_636300.1| 50S ribosomal protein L15 [Xanthomonas campestris pv. campestris str. ATCC 33913]
>gi|21230385|ref|NP_636302.1| 30S ribosomal protein S13 [Xanthomonas campestris pv. campestris str. ATCC 33913]
>gi|21230401|ref|NP_636318.1| hypothetical protein XCC0932 [Xanthomonas campestris pv. campestris str. ATCC 33913]
>gi|21230405|ref|NP_636322.1| hypothetical protein XCC0936 [Xanthomonas campestris pv. campestris str. ATCC 33913]
>gi|21230417|ref|NP_636334.1| phosphoglycerate mutase [Xanthomonas campestris pv. campestris str. ATCC 33913]
>gi|21230419|ref|NP_636336.1| folylpolyglutamate synthase/dihydrofolate synthase [Xanthomonas campestris pv. campestris str. ATCC 33913]
>gi|21230420|ref|NP_636337.1| hypothetical protein XCC0951 [Xanthomonas campestris pv. campestris str. ATCC 33913]
>gi|21230425|ref|NP_636342.1| hypothetical protein XCC0956 [Xanthomonas campestris pv. campestris str. ATCC 33913]
>gi|21230460|ref|NP_636377.1| DNA polymerase III tau and gamma subunits [Xanthomonas campestris pv. campestris str. ATCC 33913]
>gi|21230466|ref|NP_636383.1| hypothetical protein XCC1007 [Xanthomonas campestris pv. campestris str. ATCC 33913]
>gi|21230469|ref|NP_636386.1| hypothetical protein XCC1010 [Xanthomonas campestris pv. campestris str. ATCC 33913]
>gi|21230471|ref|NP_636388.1| hypothetical protein XCC1012 [Xanthomonas campestris pv. campestris str. ATCC 33913]
>gi|21230477|ref|NP_636394.1| 3-oxoacyl-[ACP] reductase [Xanthomonas campestris pv. campestris str. ATCC 33913]
>gi|21230501|ref|NP_636418.1| hypothetical protein XCC1043 [Xanthomonas campestris pv. campestris str. ATCC 33913]
>gi|21230506|ref|NP_636423.1| curved DNA binding protein [Xanthomonas campestris pv. campestris str. ATCC 33913]
>gi|21230528|ref|NP_636445.1| hypothetical protein XCC1070 [Xanthomonas campestris pv. campestris str. ATCC 33913]
>gi|21230531|ref|NP_636448.1| hypothetical protein XCC1073 [Xanthomonas campestris pv. campestris str. ATCC 33913]
>gi|21230555|ref|NP_636472.1| hypothetical protein XCC1097 [Xanthomonas campestris pv. campestris str. ATCC 33913]
>gi|21230556|ref|NP_636473.1| LexA repressor [Xanthomonas campestris pv. campestris str. ATCC 33913]
>gi|21230565|ref|NP_636482.1| hypothetical protein XCC1107 [Xanthomonas campestris pv. campestris str. ATCC 33913]
>gi|21230577|ref|NP_636494.1| hypothetical protein XCC1119 [Xanthomonas campestris pv. campestris str. ATCC 33913]
>gi|21230583|ref|NP_636500.1| cell division inhibitor [Xanthomonas campestris pv. campestris str. ATCC 33913]
>gi|21230588|ref|NP_636505.1| hypothetical protein XCC1130 [Xanthomonas campestris pv. campestris str. ATCC 33913]
>gi|21230593|ref|NP_636510.1| hypothetical protein XCC1135 [Xanthomonas campestris pv. campestris str. ATCC 33913]
>gi|21230600|ref|NP_636517.1| hypothetical protein XCC1142 [Xanthomonas campestris pv. campestris str. ATCC 33913]
>gi|21230601|ref|NP_636518.1| RNA polymerase sigma factor [Xanthomonas campestris pv. campestris str. ATCC 33913]
>gi|21230602|ref|NP_636519.1| pathogenicity-related protein [Xanthomonas campestris pv. campestris str. ATCC 33913]

>gi|21230631|ref|NP_636548.1| two-component system regulatory protein [Xanthomonas campestris pv. campestris str. ATCC 33913]
>gi|21230650|ref|NP_636567.1| hypothetical protein XCC1193 [Xanthomonas campestris pv. campestris str. ATCC 33913]
>gi|21230654|ref|NP_636571.1| 2-nitropropane dioxygenase [Xanthomonas campestris pv. campestris str. ATCC 33913]
>gi|21230663|ref|NP_636580.1| hypothetical protein XCC1206 [Xanthomonas campestris pv. campestris str. ATCC 33913]
>gi|21230669|ref|NP_636586.1| isopenicillin N epimerase [Xanthomonas campestris pv. campestris str. ATCC 33913]
>gi|21230673|ref|NP_636590.1| hypothetical protein XCC1216 [Xanthomonas campestris pv. campestris str. ATCC 33913]
>gi|21230676|ref|NP_636593.1| HrpW protein [Xanthomonas campestris pv. campestris str. ATCC 33913]
>gi|21230682|ref|NP_636599.1| HrcS protein [Xanthomonas campestris pv. campestris str. ATCC 33913]
>gi|21230684|ref|NP_636601.1| HrcQ protein [Xanthomonas campestris pv. campestris str. ATCC 33913]
>gi|21230688|ref|NP_636605.1| HrpB1 protein [Xanthomonas campestris pv. campestris str. ATCC 33913]
>gi|21230690|ref|NP_636607.1| HrcJ protein [Xanthomonas campestris pv. campestris str. ATCC 33913]
>gi|21230692|ref|NP_636609.1| HrpB5 protein [Xanthomonas campestris pv. campestris str. ATCC 33913]
>gi|21230694|ref|NP_636611.1| HrpB7 protein [Xanthomonas campestris pv. campestris str. ATCC 33913]
>gi|21230725|ref|NP_636642.1| regulatory protein [Xanthomonas campestris pv. campestris str. ATCC 33913]
>gi|21230732|ref|NP_636649.1| DNA repair protein [Xanthomonas campestris pv. campestris str. ATCC 33913]
>gi|21230735|ref|NP_636652.1| hypothetical protein XCC1278 [Xanthomonas campestris pv. campestris str. ATCC 33913]
>gi|21230737|ref|NP_636654.1| polyketide synthase [Xanthomonas campestris pv. campestris str. ATCC 33913]
>gi|21230750|ref|NP_636667.1| hypothetical protein XCC1293 [Xanthomonas campestris pv. campestris str. ATCC 33913]
>gi|21230752|ref|NP_636669.1| hypothetical protein XCC1295 [Xanthomonas campestris pv. campestris str. ATCC 33913]
>gi|21230753|ref|NP_636670.1| outer membrane protein [Xanthomonas campestris pv. campestris str. ATCC 33913]
>gi|21230759|ref|NP_636676.1| hypothetical protein XCC1302 [Xanthomonas campestris pv. campestris str. ATCC 33913]
>gi|21230764|ref|NP_636681.1| hypothetical protein XCC1307 [Xanthomonas campestris pv. campestris str. ATCC 33913]
>gi|21230765|ref|NP_636682.1| hypothetical protein XCC1308 [Xanthomonas campestris pv. campestris str. ATCC 33913]
>gi|21230774|ref|NP_636691.1| hypothetical protein XCC1317 [Xanthomonas campestris pv. campestris str. ATCC 33913]
>gi|21230781|ref|NP_636698.1| hypothetical protein XCC1324 [Xanthomonas campestris pv. campestris str. ATCC 33913]
>gi|21230785|ref|NP_636702.1| hypothetical protein XCC1328 [Xanthomonas campestris pv. campestris str. ATCC 33913]
>gi|21230790|ref|NP_636707.1| hypothetical protein XCC1333 [Xanthomonas campestris pv. campestris str. ATCC 33913]
>gi|21230805|ref|NP_636722.1| hypothetical protein XCC1348 [Xanthomonas campestris pv. campestris str. ATCC 33913]
>gi|21230809|ref|NP_636726.1| sensor protein [Xanthomonas campestris pv. campestris str. ATCC 33913]
>gi|21230820|ref|NP_636737.1| UDP-3-O-(R-3-hydroxymyristoyl)-glucosamine N-acyltransferase [Xanthomonas campestris pv. campestris str. ATCC 33913]
>gi|21230824|ref|NP_636741.1| 1-deoxy-D-xylulose 5-phosphate reductoisomerase [Xanthomonas campestris pv. campestris str. ATCC 33913]
>gi|21230825|ref|NP_636742.1| phosphatidate cytidylyltransferase [Xanthomonas campestris pv. campestris str. ATCC 33913]
>gi|21230834|ref|NP_636751.1| hypothetical protein XCC1377 [Xanthomonas campestris pv. campestris str. ATCC 33913]
>gi|21230838|ref|NP_636755.1| protein U [Xanthomonas campestris pv. campestris str. ATCC 33913]
>gi|21230841|ref|NP_636758.1| 2,3,4,5-tetrahydropyridine-2-carboxylate N-succin [Xanthomonas campestris pv. campestris str. ATCC 33913]
>gi|21230859|ref|NP_636776.1| hypothetical protein XCC1402 [Xanthomonas campestris pv. campestris str. ATCC 33913]
>gi|21230866|ref|NP_636783.1| hypothetical protein XCC1410 [Xanthomonas campestris pv. campestris str. ATCC 33913]
>gi|21230877|ref|NP_636794.1| hypothetical protein XCC1421 [Xanthomonas campestris pv. campestris str. ATCC 33913]
>gi|21230879|ref|NP_636796.1| peptidoglycan-associated outer membrane lipoprotein [Xanthomonas campestris pv. campestris str. ATCC 33913]
>gi|21230884|ref|NP_636801.1| hypothetical protein XCC1428 [Xanthomonas campestris pv. campestris str. ATCC 33913]
>gi|21230886|ref|NP_636803.1| hypothetical protein XCC1430 [Xanthomonas campestris pv. campestris str. ATCC 33913]
>gi|21230894|ref|NP_636811.1| aklaviketone reductase [Xanthomonas campestris pv. campestris str. ATCC 33913]
>gi|21230918|ref|NP_636835.1| hypothetical protein XCC1462 [Xanthomonas campestris pv. campestris str. ATCC 33913]
>gi|21230930|ref|NP_636847.1| DnaJ protein [Xanthomonas campestris pv. campestris str. ATCC 33913]
>gi|21230943|ref|NP_636860.1| hypothetical protein XCC1488 [Xanthomonas campestris pv. campestris str. ATCC 33913]
>gi|21230944|ref|NP_636861.1| hypothetical protein XCC1489 [Xanthomonas campestris pv. campestris str. ATCC 33913]
>gi|21230959|ref|NP_636876.1| hypothetical protein XCC1504 [Xanthomonas campestris pv. campestris str. ATCC 33913]
>gi|21230960|ref|NP_636877.1| hypothetical protein XCC1505 [Xanthomonas campestris pv. campestris str. ATCC 33913]
>gi|21230976|ref|NP_636893.1| hypothetical protein XCC1522 [Xanthomonas campestris pv. campestris str. ATCC 33913]
>gi|21230987|ref|NP_636904.1| hypothetical protein XCC1533 [Xanthomonas campestris pv. campestris str. ATCC 33913]
>gi|21230998|ref|NP_636915.1| ABC transporter ATP-binding protein [Xanthomonas campestris pv. campestris str. ATCC 33913]
>gi|21231008|ref|NP_636925.1| hypothetical protein XCC1554 [Xanthomonas campestris pv. campestris str. ATCC 33913]
>gi|21231020|ref|NP_636937.1| hypothetical protein XCC1566 [Xanthomonas campestris pv. campestris str. ATCC 33913]
>gi|21231023|ref|NP_636940.1| aminotransferase [Xanthomonas campestris pv. campestris str. ATCC 33913]
>gi|21231034|ref|NP_636951.1| imidazolonepropionase [Xanthomonas campestris pv. campestris str. ATCC 33913]
>gi|21231037|ref|NP_636954.1| hypothetical protein XCC1583 [Xanthomonas campestris pv. campestris str. ATCC 33913]
>gi|21231038|ref|NP_636955.1| poly(hydroxyalcanoate) granule associated protein [Xanthomonas campestris pv. campestris str. ATCC 33913]
>gi|21231047|ref|NP_636964.1| hypothetical protein XCC1593 [Xanthomonas campestris pv. campestris str. ATCC 33913]
>gi|21231057|ref|NP_636974.1| hypothetical protein XCC1604 [Xanthomonas campestris pv. campestris str. ATCC 33913]
>gi|21231070|ref|NP_636987.1| hypothetical protein XCC1617 [Xanthomonas campestris pv. campestris str. ATCC 33913]
>gi|21231078|ref|NP_636995.1| unknown acidic aa rich protein [Xanthomonas campestris pv. campestris str. ATCC 33913]
>gi|21231096|ref|NP_637013.1| glycosyl transferase-related protein [Xanthomonas campestris pv. campestris str. ATCC 33913]
>gi|21231109|ref|NP_637026.1| hypothetical protein XCC1656 [Xanthomonas campestris pv. campestris str. ATCC 33913]
>gi|21231114|ref|NP_637031.1| hypothetical protein XCC1661 [Xanthomonas campestris pv. campestris str. ATCC 33913]
>gi|21231122|ref|NP_637039.1| cytochrome like B561 [Xanthomonas campestris pv. campestris str. ATCC 33913]
>gi|21231125|ref|NP_637042.1| lipopolysaccharide biosynthesis protein [Xanthomonas campestris pv. campestris str. ATCC 33913]
>gi|21231130|ref|NP_637047.1| hypothetical protein XCC1677 [Xanthomonas campestris pv. campestris str. ATCC 33913]
>gi|21231133|ref|NP_637050.1| hexosyltransferase [Xanthomonas campestris pv. campestris str. ATCC 33913]
>gi|21231137|ref|NP_637054.1| ABC transporter ATP-binding protein [Xanthomonas campestris pv. campestris str. ATCC 33913]

>gi|21231138|ref|NP_637055.1| MFS transporter [Xanthomonas campestris pv. campestris str. ATCC 33913]
>gi|21231141|ref|NP_637058.1| ExoD protein [Xanthomonas campestris pv. campestris str. ATCC 33913]
>gi|21231149|ref|NP_637066.1| hypothetical protein XCC1696 [Xanthomonas campestris pv. campestris str. ATCC 33913]
>gi|21231152|ref|NP_637069.1| hypothetical protein XCC1699 [Xanthomonas campestris pv. campestris str. ATCC 33913]
>gi|21231154|ref|NP_637071.1| hypothetical protein XCC1701 [Xanthomonas campestris pv. campestris str. ATCC 33913]
>gi|21231167|ref|NP_637084.1| dihydropteroate synthase [Xanthomonas campestris pv. campestris str. ATCC 33913]
>gi|21231174|ref|NP_637091.1| LexA repressor [Xanthomonas campestris pv. campestris str. ATCC 33913]
>gi|21231183|ref|NP_637100.1| hypothetical protein XCC1731 [Xanthomonas campestris pv. campestris str. ATCC 33913]
>gi|21231213|ref|NP_637130.1| N-acetylmuramoyl-L-alanine amidase [Xanthomonas campestris pv. campestris str. ATCC 33913]
>gi|21231229|ref|NP_637146.1| two-component system sensor protein [Xanthomonas campestris pv. campestris str. ATCC 33913]
>gi|21231232|ref|NP_637149.1| Prop transport protein [Xanthomonas campestris pv. campestris str. ATCC 33913]
>gi|21231242|ref|NP_637159.1| filamentous haemagglutinin [Xanthomonas campestris pv. campestris str. ATCC 33913]
>gi|21231249|ref|NP_637166.1| homoserine kinase [Xanthomonas campestris pv. campestris str. ATCC 33913]
>gi|21231252|ref|NP_637169.1| hypothetical protein XCC1804 [Xanthomonas campestris pv. campestris str. ATCC 33913]
>gi|21231253|ref|NP_637170.1| fumarate and nitrate reduction regulatory protein [Xanthomonas campestris pv. campestris str. ATCC 33913]
>gi|21231275|ref|NP_637192.1| hypothetical protein XCC1827 [Xanthomonas campestris pv. campestris str. ATCC 33913]
>gi|21231277|ref|NP_637194.1| hypothetical protein XCC1829 [Xanthomonas campestris pv. campestris str. ATCC 33913]
>gi|21231279|ref|NP_637196.1| 3-hydroxyacyl-CoA dehydrogenase type II [Xanthomonas campestris pv. campestris str. ATCC 33913]
>gi|21231281|ref|NP_637198.1| enoyl-CoA hydratase [Xanthomonas campestris pv. campestris str. ATCC 33913]
>gi|21231293|ref|NP_637210.1| regulatory protein [Xanthomonas campestris pv. campestris str. ATCC 33913]
>gi|21231296|ref|NP_637213.1| inner membrane protein [Xanthomonas campestris pv. campestris str. ATCC 33913]
>gi|21231298|ref|NP_637215.1| inner membrane protein [Xanthomonas campestris pv. campestris str. ATCC 33913]
>gi|21231310|ref|NP_637227.1| hypothetical protein XCC1862 [Xanthomonas campestris pv. campestris str. ATCC 33913]
>gi|21231323|ref|NP_637240.1| chemotaxis protein [Xanthomonas campestris pv. campestris str. ATCC 33913]
>gi|21231336|ref|NP_637253.1| chemotaxis protein [Xanthomonas campestris pv. campestris str. ATCC 33913]
>gi|21231344|ref|NP_637261.1| hypothetical protein XCC1896 [Xanthomonas campestris pv. campestris str. ATCC 33913]
>gi|21231345|ref|NP_637262.1| hypothetical protein XCC1897 [Xanthomonas campestris pv. campestris str. ATCC 33913]
>gi|21231348|ref|NP_637265.1| hypothetical protein XCC1900 [Xanthomonas campestris pv. campestris str. ATCC 33913]
>gi|21231356|ref|NP_637273.1| flagellar biosynthetic protein [Xanthomonas campestris pv. campestris str. ATCC 33913]
>gi|21231362|ref|NP_637279.1| flagellar biosynthetic protein [Xanthomonas campestris pv. campestris str. ATCC 33913]
>gi|21231363|ref|NP_637280.1| flagellar biosynthesis [Xanthomonas campestris pv. campestris str. ATCC 33913]
>gi|21231364|ref|NP_637281.1| flagellar biosynthetic protein [Xanthomonas campestris pv. campestris str. ATCC 33913]
>gi|21231365|ref|NP_637282.1| flagellar protein [Xanthomonas campestris pv. campestris str. ATCC 33913]
>gi|21231369|ref|NP_637286.1| flagellar protein [Xanthomonas campestris pv. campestris str. ATCC 33913]
>gi|21231383|ref|NP_637300.1| RNA polymerase sigma-54 factor [Xanthomonas campestris pv. campestris str. ATCC 33913]
>gi|21231385|ref|NP_637302.1| hypothetical protein XCC1937 [Xanthomonas campestris pv. campestris str. ATCC 33913]
>gi|21231389|ref|NP_637306.1| flagellar protein [Xanthomonas campestris pv. campestris str. ATCC 33913]
>gi|21231392|ref|NP_637309.1| flagellar protein [Xanthomonas campestris pv. campestris str. ATCC 33913]
>gi|21231409|ref|NP_637326.1| hypothetical protein XCC1961 [Xanthomonas campestris pv. campestris str. ATCC 33913]
>gi|21231442|ref|NP_637359.1| hypothetical protein XCC1994 [Xanthomonas campestris pv. campestris str. ATCC 33913]
>gi|21231446|ref|NP_637363.1| exodeoxyribonuclease III [Xanthomonas campestris pv. campestris str. ATCC 33913]
>gi|21231454|ref|NP_637371.1| nitrate transporter [Xanthomonas campestris pv. campestris str. ATCC 33913]
>gi|21231458|ref|NP_637375.1| uroporphyrin-III C-methyltransferase [Xanthomonas campestris pv. campestris str. ATCC 33913]
>gi|21231459|ref|NP_637376.1| exopolysaccharide biosynthesis protein [Xanthomonas campestris pv. campestris str. ATCC 33913]
>gi|21231472|ref|NP_637389.1| YapH protein [Xanthomonas campestris pv. campestris str. ATCC 33913]
>gi|21231473|ref|NP_637390.1| serine protease [Xanthomonas campestris pv. campestris str. ATCC 33913]
>gi|21231485|ref|NP_637402.1| hypothetical protein XCC2037 [Xanthomonas campestris pv. campestris str. ATCC 33913]
>gi|21231487|ref|NP_637404.1| hypothetical protein XCC2039 [Xanthomonas campestris pv. campestris str. ATCC 33913]
>gi|21231488|ref|NP_637405.1| transport protein [Xanthomonas campestris pv. campestris str. ATCC 33913]
>gi|21231500|ref|NP_637417.1| hypothetical protein XCC2052 [Xanthomonas campestris pv. campestris str. ATCC 33913]
>gi|21231511|ref|NP_637428.1| adsorption protein [Xanthomonas campestris pv. campestris str. ATCC 33913]
>gi|21231515|ref|NP_637432.1| phage-related protein [Xanthomonas campestris pv. campestris str. ATCC 33913]
>gi|21231518|ref|NP_637435.1| adsorption protein [Xanthomonas campestris pv. campestris str. ATCC 33913]
>gi|21231526|ref|NP_637443.1| hypothetical protein XCC2078 [Xanthomonas campestris pv. campestris str. ATCC 33913]
>gi|21231529|ref|NP_637446.1| hypothetical protein XCC2081 [Xanthomonas campestris pv. campestris str. ATCC 33913]
>gi|21231530|ref|NP_637447.1| hypothetical protein XCC2082 [Xanthomonas campestris pv. campestris str. ATCC 33913]
>gi|21231532|ref|NP_637449.1| ribonuclease E [Xanthomonas campestris pv. campestris str. ATCC 33913]
>gi|21231567|ref|NP_637484.1| DNA uptake/competence protein [Xanthomonas campestris pv. campestris str. ATCC 33913]
>gi|21231578|ref|NP_637495.1| hypothetical protein XCC2133 [Xanthomonas campestris pv. campestris str. ATCC 33913]
>gi|21231599|ref|NP_637516.1| PHA synthase subunit [Xanthomonas campestris pv. campestris str. ATCC 33913]
>gi|21231601|ref|NP_637518.1| D-beta-hydroxybutyrate dehydrogenase [Xanthomonas campestris pv. campestris str. ATCC 33913]
>gi|21231610|ref|NP_637527.1| manganese transport protein [Xanthomonas campestris pv. campestris str. ATCC 33913]
>gi|21231613|ref|NP_637530.1| MFS transporter [Xanthomonas campestris pv. campestris str. ATCC 33913]
>gi|21231615|ref|NP_637532.1| hypothetical protein XCC2176 [Xanthomonas campestris pv. campestris str. ATCC 33913]
>gi|21231621|ref|NP_637538.1| hypothetical protein XCC2182 [Xanthomonas campestris pv. campestris str. ATCC 33913]
>gi|21231639|ref|NP_637556.1| phosphinothricin N-acetyltransferase [Xanthomonas campestris pv. campestris str. ATCC 33913]
>gi|21231652|ref|NP_637569.1| hypothetical protein XCC2213 [Xanthomonas campestris pv. campestris str. ATCC 33913]
>gi|21231653|ref|NP_637570.1| pseudouridylate synthase [Xanthomonas campestris pv. campestris str. ATCC 33913]
>gi|21231659|ref|NP_637576.1| ABC transporter heme permease [Xanthomonas campestris pv. campestris str. ATCC 33913]
>gi|21231663|ref|NP_637580.1| c-type cytochrome biogenesis membrane protein [Xanthomonas campestris pv. campestris str. ATCC 33913]

>gi|21231669|ref|NP_637586.1| ABC transporter ATP-binding protein [Xanthomonas campestris pv. campestris str. ATCC 33913]
>gi|21231670|ref|NP_637587.1| transport protein [Xanthomonas campestris pv. campestris str. ATCC 33913]
>gi|21231672|ref|NP_637589.1| cytochrome D ubiquinol oxidase subunit II [Xanthomonas campestris pv. campestris str. ATCC 33913]
>gi|21231674|ref|NP_637591.1| MFS transporter [Xanthomonas campestris pv. campestris str. ATCC 33913]
>gi|21231682|ref|NP_637599.1| amino-acid acetyltransferase [Xanthomonas campestris pv. campestris str. ATCC 33913]
>gi|21231692|ref|NP_637609.1| hypothetical protein XCC2254 [Xanthomonas campestris pv. campestris str. ATCC 33913]
>gi|21231705|ref|NP_637622.1| hypothetical protein XCC2267 [Xanthomonas campestris pv. campestris str. ATCC 33913]
>gi|21231709|ref|NP_637626.1| hypothetical protein XCC2271 [Xanthomonas campestris pv. campestris str. ATCC 33913]
>gi|21231714|ref|NP_637631.1| tRNA/rRNA methyltransferase [Xanthomonas campestris pv. campestris str. ATCC 33913]
>gi|21231718|ref|NP_637635.1| hypothetical protein XCC2280 [Xanthomonas campestris pv. campestris str. ATCC 33913]
>gi|21231737|ref|NP_637654.1| N-acetylmuramoyl-L-alanine amidase [Xanthomonas campestris pv. campestris str. ATCC 33913]
>gi|21231741|ref|NP_637658.1| exodeoxyribonuclease VII large subunit [Xanthomonas campestris pv. campestris str. ATCC 33913]
>gi|21231762|ref|NP_637679.1| soluble lytic murein transglycosylase [Xanthomonas campestris pv. campestris str. ATCC 33913]
>gi|21231763|ref|NP_637680.1| repressor [Xanthomonas campestris pv. campestris str. ATCC 33913]
>gi|21231778|ref|NP_637695.1| outer membrane protein OprN precursor [Xanthomonas campestris pv. campestris str. ATCC 33913]
>gi|21231780|ref|NP_637697.1| transport protein [Xanthomonas campestris pv. campestris str. ATCC 33913]
>gi|21231795|ref|NP_637712.1| hypothetical protein XCC2358 [Xanthomonas campestris pv. campestris str. ATCC 33913]
>gi|21231804|ref|NP_637721.1| multidrug resistance protein [Xanthomonas campestris pv. campestris str. ATCC 33913]
>gi|21231808|ref|NP_637725.1| 1-phosphofructokinase [Xanthomonas campestris pv. campestris str. ATCC 33913]
>gi|21231809|ref|NP_637726.1| PTS system,fructose-specificIIBC component [Xanthomonas campestris pv. campestris str. ATCC 33913]
>gi|21231840|ref|NP_637757.1| hypothetical protein XCC2403 [Xanthomonas campestris pv. campestris str. ATCC 33913]
>gi|21231847|ref|NP_637764.1| ketoglutarate semialdehyde dehydrogenase [Xanthomonas campestris pv. campestris str. ATCC 33913]
>gi|21231849|ref|NP_637766.1| oxidoreductase [Xanthomonas campestris pv. campestris str. ATCC 33913]
>gi|21231854|ref|NP_637771.1| cell division protein [Xanthomonas campestris pv. campestris str. ATCC 33913]
>gi|21231865|ref|NP_637782.1| hypothetical protein XCC2428 [Xanthomonas campestris pv. campestris str. ATCC 33913]
>gi|21231873|ref|NP_637790.1| hypothetical protein XCC2437 [Xanthomonas campestris pv. campestris str. ATCC 33913]
>gi|21231916|ref|NP_637833.1| VirB8 protein [Xanthomonas campestris pv. campestris str. ATCC 33913]
>gi|21231917|ref|NP_637834.1| hypothetical protein XCC2482 [Xanthomonas campestris pv. campestris str. ATCC 33913]
>gi|21231932|ref|NP_637849.1| short chain dehydrogenase [Xanthomonas campestris pv. campestris str. ATCC 33913]
>gi|21231951|ref|NP_637868.1| NADH-ubiquinone oxidoreductase NQO10 subunit [Xanthomonas campestris pv. campestris str. ATCC 33913]
>gi|21231961|ref|NP_637878.1| protein-export membrane protein [Xanthomonas campestris pv. campestris str. ATCC 33913]
>gi|21231972|ref|NP_637889.1| tryptophan synthase alpha chain [Xanthomonas campestris pv. campestris str. ATCC 33913]
>gi|21231973|ref|NP_637890.1| hypothetical protein XCC2542 [Xanthomonas campestris pv. campestris str. ATCC 33913]
>gi|21231979|ref|NP_637896.1| FimV protein [Xanthomonas campestris pv. campestris str. ATCC 33913]
>gi|21231983|ref|NP_637900.1| hypothetical protein XCC2552 [Xanthomonas campestris pv. campestris str. ATCC 33913]
>gi|21231991|ref|NP_637908.1| hypothetical protein XCC2560 [Xanthomonas campestris pv. campestris str. ATCC 33913]
>gi|21232000|ref|NP_637917.1| hypothetical protein XCC2569 [Xanthomonas campestris pv. campestris str. ATCC 33913]
>gi|21232009|ref|NP_637926.1| methylated-DNA-protein-cysteine S-methyltransferase related protein [Xanthomonas campestris pv. campestris str. ATCC 33913]
>gi|21232012|ref|NP_637929.1| transport protein [Xanthomonas campestris pv. campestris str. ATCC 33913]
>gi|21232014|ref|NP_637931.1| lipoprotein [Xanthomonas campestris pv. campestris str. ATCC 33913]
>gi|21232015|ref|NP_637932.1| hypothetical protein XCC2584 [Xanthomonas campestris pv. campestris str. ATCC 33913]
>gi|21232025|ref|NP_637942.1| hypothetical protein XCC2594 [Xanthomonas campestris pv. campestris str. ATCC 33913]
>gi|21232029|ref|NP_637946.1| cell cycle protein [Xanthomonas campestris pv. campestris str. ATCC 33913]
>gi|21232043|ref|NP_637960.1| TonB like protein [Xanthomonas campestris pv. campestris str. ATCC 33913]
>gi|21232052|ref|NP_637969.1| hypothetical protein XCC2621 [Xanthomonas campestris pv. campestris str. ATCC 33913]
>gi|21232055|ref|NP_637972.1| hypothetical protein XCC2624 [Xanthomonas campestris pv. campestris str. ATCC 33913]
>gi|21232062|ref|NP_637979.1| hypothetical protein XCC2631 [Xanthomonas campestris pv. campestris str. ATCC 33913]
>gi|21232070|ref|NP_637987.1| hypothetical protein XCC2639 [Xanthomonas campestris pv. campestris str. ATCC 33913]
>gi|21232080|ref|NP_637997.1| hypothetical protein XCC2649 [Xanthomonas campestris pv. campestris str. ATCC 33913]
>gi|21232087|ref|NP_638004.1| hypothetical protein XCC2656 [Xanthomonas campestris pv. campestris str. ATCC 33913]
>gi|21232088|ref|NP_638005.1| hypothetical protein XCC2657 [Xanthomonas campestris pv. campestris str. ATCC 33913]
>gi|21232091|ref|NP_638008.1| transmembrane transport protein [Xanthomonas campestris pv. campestris str. ATCC 33913]
>gi|21232092|ref|NP_638009.1| cytochrome D oxidase subunit B [Xanthomonas campestris pv. campestris str. ATCC 33913]
>gi|21232099|ref|NP_638016.1| hypothetical protein XCC2668 [Xanthomonas campestris pv. campestris str. ATCC 33913]
>gi|21232101|ref|NP_638018.1| oxidoreductase [Xanthomonas campestris pv. campestris str. ATCC 33913]
>gi|21232103|ref|NP_638020.1| MFS transporter [Xanthomonas campestris pv. campestris str. ATCC 33913]
>gi|21232111|ref|NP_638028.1| transcriptional regulator [Xanthomonas campestris pv. campestris str. ATCC 33913]
>gi|21232121|ref|NP_638038.1| hypothetical protein XCC2690 [Xanthomonas campestris pv. campestris str. ATCC 33913]
>gi|21232125|ref|NP_638042.1| two-component system sensor protein [Xanthomonas campestris pv. campestris str. ATCC 33913]
>gi|21232142|ref|NP_638059.1| hypothetical protein XCC2711 [Xanthomonas campestris pv. campestris str. ATCC 33913]
>gi|21232156|ref|NP_638073.1| hypothetical protein XCC2725 [Xanthomonas campestris pv. campestris str. ATCC 33913]
>gi|21232160|ref|NP_638077.1| oxidoreductase [Xanthomonas campestris pv. campestris str. ATCC 33913]
>gi|21232166|ref|NP_638083.1| single-stranded DNA binding protein [Xanthomonas campestris pv. campestris str. ATCC 33913]
>gi|21232171|ref|NP_638088.1| hypothetical protein XCC2740 [Xanthomonas campestris pv. campestris str. ATCC 33913]
>gi|21232173|ref|NP_638090.1| hypothetical protein XCC2742 [Xanthomonas campestris pv. campestris str. ATCC 33913]
>gi|21232175|ref|NP_638092.1| hypothetical protein XCC2744 [Xanthomonas campestris pv. campestris str. ATCC 33913]
>gi|21232180|ref|NP_638097.1| hypothetical protein XCC2749 [Xanthomonas campestris pv. campestris str. ATCC 33913]
>gi|21232189|ref|NP_638106.1| histone-like protein [Xanthomonas campestris pv. campestris str. ATCC 33913]
>gi|21232192|ref|NP_638109.1| hypothetical protein XCC2761 [Xanthomonas campestris pv. campestris str. ATCC 33913]

>gi|21232205|ref|NP_638122.1| hypothetical protein XCC2774 [Xanthomonas campestris pv. campestris str. ATCC 33913]
>gi|21232214|ref|NP_638131.1| hypothetical protein XCC2783 [Xanthomonas campestris pv. campestris str. ATCC 33913]
>gi|21232215|ref|NP_638132.1| hypothetical protein XCC2784 [Xanthomonas campestris pv. campestris str. ATCC 33913]
>gi|21232223|ref|NP_638140.1| hypothetical protein XCC2792 [Xanthomonas campestris pv. campestris str. ATCC 33913]
>gi|21232228|ref|NP_638145.1| polysialic acid capsule expression protein [Xanthomonas campestris pv. campestris str. ATCC 33913]
>gi|21232249|ref|NP_638166.1| transcriptional regulator ahyR/asaR family [Xanthomonas campestris pv. campestris str. ATCC 33913]
>gi|21232254|ref|NP_638171.1| hypothetical protein XCC2823 [Xanthomonas campestris pv. campestris str. ATCC 33913]
>gi|21232261|ref|NP_638178.1| hypothetical protein XCC2830 [Xanthomonas campestris pv. campestris str. ATCC 33913]
>gi|21232263|ref|NP_638180.1| MFS transporter [Xanthomonas campestris pv. campestris str. ATCC 33913]
>gi|21232265|ref|NP_638182.1| hypothetical protein XCC2834 [Xanthomonas campestris pv. campestris str. ATCC 33913]
>gi|21232305|ref|NP_638222.1| hypothetical protein XCC2874 [Xanthomonas campestris pv. campestris str. ATCC 33913]
>gi|21232306|ref|NP_638223.1| histone H1 [Xanthomonas campestris pv. campestris str. ATCC 33913]
>gi|21232307|ref|NP_638224.1| glycine cleavage H protein [Xanthomonas campestris pv. campestris str. ATCC 33913]
>gi|21232309|ref|NP_638226.1| hypothetical protein XCC2878 [Xanthomonas campestris pv. campestris str. ATCC 33913]
>gi|21232310|ref|NP_638227.1| hypothetical protein XCC2879 [Xanthomonas campestris pv. campestris str. ATCC 33913]
>gi|21232311|ref|NP_638228.1| hypothetical protein XCC2880 [Xanthomonas campestris pv. campestris str. ATCC 33913]
>gi|21232321|ref|NP_638238.1| beta-hexosaminidase [Xanthomonas campestris pv. campestris str. ATCC 33913]
>gi|21232339|ref|NP_638256.1| RebB protein [Xanthomonas campestris pv. campestris str. ATCC 33913]
>gi|21232348|ref|NP_638265.1| antifreeze glycopeptide AFGP related protein [Xanthomonas campestris pv. campestris str. ATCC 33913]
>gi|21232352|ref|NP_638269.1| PilL protein [Xanthomonas campestris pv. campestris str. ATCC 33913]
>gi|21232360|ref|NP_638277.1| hypothetical protein XCC2929 [Xanthomonas campestris pv. campestris str. ATCC 33913]
>gi|21232362|ref|NP_638279.1| hypothetical protein XCC2931 [Xanthomonas campestris pv. campestris str. ATCC 33913]
>gi|21232365|ref|NP_638282.1| hypothetical protein XCC2934 [Xanthomonas campestris pv. campestris str. ATCC 33913]
>gi|21232366|ref|NP_638283.1| hypothetical protein XCC2935 [Xanthomonas campestris pv. campestris str. ATCC 33913]
>gi|21232387|ref|NP_638304.1| chloride channel [Xanthomonas campestris pv. campestris str. ATCC 33913]
>gi|21232410|ref|NP_638327.1| hypothetical protein XCC2979 [Xanthomonas campestris pv. campestris str. ATCC 33913]
>gi|21232415|ref|NP_638332.1| phage-related tail fiber protein [Xanthomonas campestris pv. campestris str. ATCC 33913]
>gi|21232417|ref|NP_638334.1| phage-related baseplate protein [Xanthomonas campestris pv. campestris str. ATCC 33913]
>gi|21232418|ref|NP_638335.1| phage-related baseplate protein [Xanthomonas campestris pv. campestris str. ATCC 33913]
>gi|21232423|ref|NP_638340.1| phage-related tail protein [Xanthomonas campestris pv. campestris str. ATCC 33913]
>gi|21232446|ref|NP_638363.1| hypothetical protein XCC3016 [Xanthomonas campestris pv. campestris str. ATCC 33913]
>gi|21232449|ref|NP_638366.1| TolA protein [Xanthomonas campestris pv. campestris str. ATCC 33913]
>gi|21232461|ref|NP_638378.1| hypothetical protein XCC3031 [Xanthomonas campestris pv. campestris str. ATCC 33913]
>gi|21232463|ref|NP_638380.1| hypothetical protein XCC3033 [Xanthomonas campestris pv. campestris str. ATCC 33913]
>gi|21232470|ref|NP_638387.1| transcriptional activator ampR family [Xanthomonas campestris pv. campestris str. ATCC 33913]
>gi|21232471|ref|NP_638388.1| hypothetical protein XCC3041 [Xanthomonas campestris pv. campestris str. ATCC 33913]
>gi|21232478|ref|NP_638395.1| cation:proton antiporter [Xanthomonas campestris pv. campestris str. ATCC 33913]
>gi|21232482|ref|NP_638399.1| hypothetical protein XCC3052 [Xanthomonas campestris pv. campestris str. ATCC 33913]
>gi|21232483|ref|NP_638400.1| transport protein [Xanthomonas campestris pv. campestris str. ATCC 33913]
>gi|21232484|ref|NP_638401.1| iron transporter [Xanthomonas campestris pv. campestris str. ATCC 33913]
>gi|21232487|ref|NP_638404.1| cobalamin synthase [Xanthomonas campestris pv. campestris str. ATCC 33913]
>gi|21232492|ref|NP_638409.1| cobalamin biosynthesis protein [Xanthomonas campestris pv. campestris str. ATCC 33913]
>gi|21232495|ref|NP_638412.1| hypothetical protein XCC3065 [Xanthomonas campestris pv. campestris str. ATCC 33913]
>gi|21232501|ref|NP_638418.1| ABC transporter ATP-binding subunit [Xanthomonas campestris pv. campestris str. ATCC 33913]
>gi|21232504|ref|NP_638421.1| hypothetical protein XCC3074 [Xanthomonas campestris pv. campestris str. ATCC 33913]
>gi|21232505|ref|NP_638422.1| hypothetical protein XCC3075 [Xanthomonas campestris pv. campestris str. ATCC 33913]
>gi|21232506|ref|NP_638423.1| hypothetical protein XCC3076 [Xanthomonas campestris pv. campestris str. ATCC 33913]
>gi|21232517|ref|NP_638434.1| hypothetical protein XCC3087 [Xanthomonas campestris pv. campestris str. ATCC 33913]
>gi|21232594|ref|NP_638511.1| tRNA/rRNA methyltransferase [Xanthomonas campestris pv. campestris str. ATCC 33913]
>gi|21232605|ref|NP_638522.1| hypothetical protein XCC3176 [Xanthomonas campestris pv. campestris str. ATCC 33913]
>gi|21232612|ref|NP_638529.1| hypothetical protein XCC3183 [Xanthomonas campestris pv. campestris str. ATCC 33913]
>gi|21232619|ref|NP_638536.1| hypothetical protein XCC3190 [Xanthomonas campestris pv. campestris str. ATCC 33913]
>gi|21232626|ref|NP_638543.1| beta-lactamase [Xanthomonas campestris pv. campestris str. ATCC 33913]
>gi|21232640|ref|NP_638557.1| hypothetical protein XCC3211 [Xanthomonas campestris pv. campestris str. ATCC 33913]
>gi|21232651|ref|NP_638568.1| hypothetical protein XCC3222 [Xanthomonas campestris pv. campestris str. ATCC 33913]
>gi|21232652|ref|NP_638569.1| hypothetical protein XCC3223 [Xanthomonas campestris pv. campestris str. ATCC 33913]
>gi|21232654|ref|NP_638571.1| hypothetical protein XCC3225 [Xanthomonas campestris pv. campestris str. ATCC 33913]
>gi|21232660|ref|NP_638577.1| fimbrial assembly membrane protein [Xanthomonas campestris pv. campestris str. ATCC 33913]
>gi|21232661|ref|NP_638578.1| fimbrial assembly membrane protein [Xanthomonas campestris pv. campestris str. ATCC 33913]
>gi|21232666|ref|NP_638583.1| inosine-uridine preferring nucleoside hydrolase [Xanthomonas campestris pv. campestris str. ATCC 33913]
>gi|21232677|ref|NP_638594.1| RNA polymerase omega subunit [Xanthomonas campestris pv. campestris str. ATCC 33913]
>gi|21232684|ref|NP_638601.1| hypothetical protein XCC3255 [Xanthomonas campestris pv. campestris str. ATCC 33913]
>gi|21232689|ref|NP_638606.1| hypothetical protein XCC3260 [Xanthomonas campestris pv. campestris str. ATCC 33913]
>gi|21232690|ref|NP_638607.1| hypothetical protein XCC3261 [Xanthomonas campestris pv. campestris str. ATCC 33913]
>gi|21232698|ref|NP_638615.1| thiamin-phosphate pyrophosphorylase [Xanthomonas campestris pv. campestris str. ATCC 33913]
>gi|21232709|ref|NP_638626.1| hypothetical protein XCC3280 [Xanthomonas campestris pv. campestris str. ATCC 33913]
>gi|21232710|ref|NP_638627.1| hypothetical protein XCC3281 [Xanthomonas campestris pv. campestris str. ATCC 33913]
>gi|21232769|ref|NP_638686.1| glycosyl transferase [Xanthomonas campestris pv. campestris str. ATCC 33913]
>gi|21232803|ref|NP_638720.1| hypothetical protein XCC3374 [Xanthomonas campestris pv. campestris str. ATCC 33913]
>gi|21232821|ref|NP_638738.1| putative malic acid transport protein [Xanthomonas campestris pv. campestris str. ATCC 33913]

>gi|21232829|ref|NP_638746.1| thiol:disulfide interchange protein [Xanthomonas campestris pv. campestris str. ATCC 33913]
>gi|21232840|ref|NP_638757.1| glucosamine-fructose-6-phosphate aminotransferase [Xanthomonas campestris pv. campestris str. ATCC 33913]
>gi|21232844|ref|NP_638761.1| type II secretion system protein N [Xanthomonas campestris pv. campestris str. ATCC 33913]
>gi|21232845|ref|NP_638762.1| type II secretion system protein M [Xanthomonas campestris pv. campestris str. ATCC 33913]
>gi|21232846|ref|NP_638763.1| type II secretion system protein L [Xanthomonas campestris pv. campestris str. ATCC 33913]
>gi|21232848|ref|NP_638765.1| type II secretion system protein J [Xanthomonas campestris pv. campestris str. ATCC 33913]
>gi|21232849|ref|NP_638766.1| type II secretion system protein I [Xanthomonas campestris pv. campestris str. ATCC 33913]
>gi|21232855|ref|NP_638772.1| type II secretion system protein C [Xanthomonas campestris pv. campestris str. ATCC 33913]
>gi|21232858|ref|NP_638775.1| hypothetical protein XCC3429 [Xanthomonas campestris pv. campestris str. ATCC 33913]
>gi|21232870|ref|NP_638787.1| hypothetical protein XCC3441 [Xanthomonas campestris pv. campestris str. ATCC 33913]
>gi|21232871|ref|NP_638788.1| hypothetical protein XCC3442 [Xanthomonas campestris pv. campestris str. ATCC 33913]
>gi|21232877|ref|NP_638794.1| transcriptional regulator [Xanthomonas campestris pv. campestris str. ATCC 33913]
>gi|21232878|ref|NP_638795.1| hypothetical protein XCC3449 [Xanthomonas campestris pv. campestris str. ATCC 33913]
>gi|21232898|ref|NP_638815.1| rod shape-determining protein [Xanthomonas campestris pv. campestris str. ATCC 33913]
>gi|21232906|ref|NP_638823.1| hypothetical protein XCC3477 [Xanthomonas campestris pv. campestris str. ATCC 33913]
>gi|21232907|ref|NP_638824.1| MoxJ protein [Xanthomonas campestris pv. campestris str. ATCC 33913]
>gi|21232908|ref|NP_638825.1| methanol dehydrogenase heavy chain [Xanthomonas campestris pv. campestris str. ATCC 33913]
>gi|21232912|ref|NP_638829.1| hypothetical protein XCC3483 [Xanthomonas campestris pv. campestris str. ATCC 33913]
>gi|21232913|ref|NP_638830.1| hypothetical protein XCC3484 [Xanthomonas campestris pv. campestris str. ATCC 33913]
>gi|21232918|ref|NP_638835.1| multidrug resistance efflux pump [Xanthomonas campestris pv. campestris str. ATCC 33913]
>gi|21232921|ref|NP_638838.1| hypothetical protein XCC3492 [Xanthomonas campestris pv. campestris str. ATCC 33913]
>gi|21232929|ref|NP_638846.1| hypothetical protein XCC3500 [Xanthomonas campestris pv. campestris str. ATCC 33913]
>gi|21232945|ref|NP_638862.1| sulfur deprivation response regulator [Xanthomonas campestris pv. campestris str. ATCC 33913]
>gi|21232949|ref|NP_638866.1| sulfate permease [Xanthomonas campestris pv. campestris str. ATCC 33913]
>gi|21232950|ref|NP_638867.1| cellulase [Xanthomonas campestris pv. campestris str. ATCC 33913]
>gi|21232955|ref|NP_638872.1| hypothetical protein XCC3526 [Xanthomonas campestris pv. campestris str. ATCC 33913]
>gi|21232957|ref|NP_638874.1| hypothetical protein XCC3528 [Xanthomonas campestris pv. campestris str. ATCC 33913]
>gi|21232965|ref|NP_638882.1| hypothetical protein XCC3536 [Xanthomonas campestris pv. campestris str. ATCC 33913]
>gi|21232969|ref|NP_638886.1| hypothetical protein XCC3540 [Xanthomonas campestris pv. campestris str. ATCC 33913]
>gi|21232970|ref|NP_638887.1| hypothetical protein XCC3541 [Xanthomonas campestris pv. campestris str. ATCC 33913]
>gi|21232975|ref|NP_638892.1| hypothetical protein XCC3546 [Xanthomonas campestris pv. campestris str. ATCC 33913]
>gi|21232981|ref|NP_638898.1| hypothetical protein XCC3552 [Xanthomonas campestris pv. campestris str. ATCC 33913]
>gi|21232988|ref|NP_638905.1| hypothetical protein XCC3559 [Xanthomonas campestris pv. campestris str. ATCC 33913]
>gi|21233003|ref|NP_638920.1| phage-related baseplate assembly protein [Xanthomonas campestris pv. campestris str. ATCC 33913]
>gi|21233019|ref|NP_638936.1| hypothetical protein XCC3590 [Xanthomonas campestris pv. campestris str. ATCC 33913]
>gi|21233022|ref|NP_638939.1| RNA polymerase sigma factor [Xanthomonas campestris pv. campestris str. ATCC 33913]
>gi|21233026|ref|NP_638943.1| hypothetical protein XCC3597 [Xanthomonas campestris pv. campestris str. ATCC 33913]
>gi|21233027|ref|NP_638944.1| hypothetical protein XCC3598 [Xanthomonas campestris pv. campestris str. ATCC 33913]
>gi|21233035|ref|NP_638952.1| hypothetical protein XCC3606 [Xanthomonas campestris pv. campestris str. ATCC 33913]
>gi|21233043|ref|NP_638960.1| CitG protein [Xanthomonas campestris pv. campestris str. ATCC 33913]
>gi|21233045|ref|NP_638962.1| dicarboxylate carrier protein [Xanthomonas campestris pv. campestris str. ATCC 33913]
>gi|21233058|ref|NP_638975.1| ABC transporter permease [Xanthomonas campestris pv. campestris str. ATCC 33913]
>gi|21233079|ref|NP_638996.1| hypothetical protein XCC3650 [Xanthomonas campestris pv. campestris str. ATCC 33913]
>gi|21233081|ref|NP_638998.1| hypothetical protein XCC3652 [Xanthomonas campestris pv. campestris str. ATCC 33913]
>gi|21233083|ref|NP_639000.1| chemotaxis MotB protein [Xanthomonas campestris pv. campestris str. ATCC 33913]
>gi|21233084|ref|NP_639001.1| hypothetical protein XCC3655 [Xanthomonas campestris pv. campestris str. ATCC 33913]
>gi|21233086|ref|NP_639003.1| hypothetical protein XCC3657 [Xanthomonas campestris pv. campestris str. ATCC 33913]
>gi|21233090|ref|NP_639007.1| Na+:H+ antiporter [Xanthomonas campestris pv. campestris str. ATCC 33913]
>gi|21233113|ref|NP_639030.1| hypothetical protein XCC3684 [Xanthomonas campestris pv. campestris str. ATCC 33913]
>gi|21233116|ref|NP_639033.1| regulatory protein [Xanthomonas campestris pv. campestris str. ATCC 33913]
>gi|21233122|ref|NP_639039.1| hypothetical protein XCC3693 [Xanthomonas campestris pv. campestris str. ATCC 33913]
>gi|21233140|ref|NP_639057.1| hypothetical protein XCC3711 [Xanthomonas campestris pv. campestris str. ATCC 33913]
>gi|21233142|ref|NP_639059.1| hypothetical protein XCC3713 [Xanthomonas campestris pv. campestris str. ATCC 33913]
>gi|21233144|ref|NP_639061.1| hypothetical protein XCC3715 [Xanthomonas campestris pv. campestris str. ATCC 33913]
>gi|21233145|ref|NP_639062.1| hypothetical protein XCC3716 [Xanthomonas campestris pv. campestris str. ATCC 33913]
>gi|21233152|ref|NP_639069.1| hypothetical protein XCC3723 [Xanthomonas campestris pv. campestris str. ATCC 33913]
>gi|21233185|ref|NP_639102.1| hypothetical protein XCC3757 [Xanthomonas campestris pv. campestris str. ATCC 33913]
>gi|21233186|ref|NP_639103.1| hypothetical protein XCC3758 [Xanthomonas campestris pv. campestris str. ATCC 33913]
>gi|21233191|ref|NP_639108.1| hypothetical protein XCC3763 [Xanthomonas campestris pv. campestris str. ATCC 33913]
>gi|21233192|ref|NP_639109.1| hypothetical protein XCC3764 [Xanthomonas campestris pv. campestris str. ATCC 33913]
>gi|21233202|ref|NP_639119.1| cell division protein [Xanthomonas campestris pv. campestris str. ATCC 33913]
>gi|21233204|ref|NP_639121.1| ATP-dependent RNA helicase [Xanthomonas campestris pv. campestris str. ATCC 33913]
>gi|21233211|ref|NP_639128.1| hypothetical protein XCC3783 [Xanthomonas campestris pv. campestris str. ATCC 33913]
>gi|21233222|ref|NP_639139.1| hypothetical protein XCC3794 [Xanthomonas campestris pv. campestris str. ATCC 33913]
>gi|21233223|ref|NP_639140.1| hypothetical protein XCC3795 [Xanthomonas campestris pv. campestris str. ATCC 33913]
>gi|21233225|ref|NP_639142.1| hypothetical protein XCC3797 [Xanthomonas campestris pv. campestris str. ATCC 33913]
>gi|21233226|ref|NP_639143.1| hypothetical protein XCC3798 [Xanthomonas campestris pv. campestris str. ATCC 33913]
>gi|21233231|ref|NP_639148.1| hypothetical protein XCC3803 [Xanthomonas campestris pv. campestris str. ATCC 33913]
>gi|21233234|ref|NP_639151.1| hypothetical protein XCC3806 [Xanthomonas campestris pv. campestris str. ATCC 33913]
>gi|21233237|ref|NP_639154.1| cationic amino acid transporter [Xanthomonas campestris pv. campestris str. ATCC 33913]

>gi|21233238|ref|NP_639155.1| hypothetical protein XCC3810 [Xanthomonas campestris pv. campestris str. ATCC 33913]
>gi|21233240|ref|NP_639157.1| membrane protein [Xanthomonas campestris pv. campestris str. ATCC 33913]
>gi|21233245|ref|NP_639162.1| 30S ribosomal protein S21 [Xanthomonas campestris pv. campestris str. ATCC 33913]
>gi|21233246|ref|NP_639163.1| hypothetical protein XCC3818 [Xanthomonas campestris pv. campestris str. ATCC 33913]
>gi|21233253|ref|NP_639170.1| hypothetical protein XCC3825 [Xanthomonas campestris pv. campestris str. ATCC 33913]
>gi|21233255|ref|NP_639172.1| hypothetical protein XCC3827 [Xanthomonas campestris pv. campestris str. ATCC 33913]
>gi|21233273|ref|NP_639190.1| chromosome partitioning protein [Xanthomonas campestris pv. campestris str. ATCC 33913]
>gi|21233282|ref|NP_639199.1| DNA/pantothenate metabolism flavoprotein [Xanthomonas campestris pv. campestris str. ATCC 33913]
>gi|21233285|ref|NP_639202.1| hypothetical protein XCC3862 [Xanthomonas campestris pv. campestris str. ATCC 33913]
>gi|21233288|ref|NP_639205.1| acyltransferase [Xanthomonas campestris pv. campestris str. ATCC 33913]
>gi|21233302|ref|NP_639219.1| oxidoreductase [Xanthomonas campestris pv. campestris str. ATCC 33913]
>gi|21233307|ref|NP_639224.1| hypothetical protein XCC3884 [Xanthomonas campestris pv. campestris str. ATCC 33913]
>gi|21233312|ref|NP_639229.1| hypothetical protein XCC3889 [Xanthomonas campestris pv. campestris str. ATCC 33913]
>gi|21233315|ref|NP_639232.1| hypothetical protein XCC3892 [Xanthomonas campestris pv. campestris str. ATCC 33913]
>gi|21233318|ref|NP_639235.1| hypothetical protein XCC3895 [Xanthomonas campestris pv. campestris str. ATCC 33913]
>gi|21233320|ref|NP_639237.1| histone H1 [Xanthomonas campestris pv. campestris str. ATCC 33913]
>gi|21233322|ref|NP_639239.1| hypothetical protein XCC3899 [Xanthomonas campestris pv. campestris str. ATCC 33913]
>gi|21233324|ref|NP_639241.1| hypothetical protein XCC3901 [Xanthomonas campestris pv. campestris str. ATCC 33913]
>gi|21233329|ref|NP_639246.1| cytochrome B561 [Xanthomonas campestris pv. campestris str. ATCC 33913]
>gi|21233333|ref|NP_639250.1| two-component system sensor protein [Xanthomonas campestris pv. campestris str. ATCC 33913]
>gi|21233354|ref|NP_639271.1| thymidylate kinase [Xanthomonas campestris pv. campestris str. ATCC 33913]
>gi|21233358|ref|NP_639275.1| hypothetical protein XCC3936 [Xanthomonas campestris pv. campestris str. ATCC 33913]
>gi|21233360|ref|NP_639277.1| bifunctional transcriptional repressor of the biotin operon/biotin acetyl-CoA-carboxylase synthetase [Xanthomonas campestris pv. campestris str. ATCC 33913]
>gi|21233374|ref|NP_639291.1| hypothetical protein XCC3952 [Xanthomonas campestris pv. campestris str. ATCC 33913]
>gi|21233380|ref|NP_639297.1| hypothetical protein XCC3958 [Xanthomonas campestris pv. campestris str. ATCC 33913]
>gi|21233389|ref|NP_639306.1| TonB-like protein [Xanthomonas campestris pv. campestris str. ATCC 33913]
>gi|21233394|ref|NP_639311.1| hypothetical protein XCC3972 [Xanthomonas campestris pv. campestris str. ATCC 33913]
>gi|21233396|ref|NP_639313.1| heavy metal transporter [Xanthomonas campestris pv. campestris str. ATCC 33913]
>gi|21233418|ref|NP_639335.1| ankyrin-like protein [Xanthomonas campestris pv. campestris str. ATCC 33913]
>gi|21233419|ref|NP_639336.1| hypothetical protein XCC3997 [Xanthomonas campestris pv. campestris str. ATCC 33913]
>gi|21233421|ref|NP_639338.1| hypothetical protein XCC3999 [Xanthomonas campestris pv. campestris str. ATCC 33913]
>gi|21233425|ref|NP_639342.1| 3-oxoacyl-[ACP] reductase [Xanthomonas campestris pv. campestris str. ATCC 33913]
>gi|21233427|ref|NP_639344.1| membrane protein [Xanthomonas campestris pv. campestris str. ATCC 33913]
>gi|21233428|ref|NP_639345.1| membrane protein [Xanthomonas campestris pv. campestris str. ATCC 33913]
>gi|21233439|ref|NP_639356.1| hypothetical protein XCC4017 [Xanthomonas campestris pv. campestris str. ATCC 33913]
>gi|21233440|ref|NP_639357.1| hypothetical protein XCC4018 [Xanthomonas campestris pv. campestris str. ATCC 33913]
>gi|21233443|ref|NP_639360.1| hypothetical protein XCC4021 [Xanthomonas campestris pv. campestris str. ATCC 33913]
>gi|21233447|ref|NP_639364.1| nodulation protein [Xanthomonas campestris pv. campestris str. ATCC 33913]
>gi|21233457|ref|NP_639374.1| hypothetical protein XCC4035 [Xanthomonas campestris pv. campestris str. ATCC 33913]
>gi|21233460|ref|NP_639377.1| cation efflux system protein [Xanthomonas campestris pv. campestris str. ATCC 33913]
>gi|21233461|ref|NP_639378.1| hypothetical protein XCC4039 [Xanthomonas campestris pv. campestris str. ATCC 33913]
>gi|21233470|ref|NP_639387.1| phospholipase A1 [Xanthomonas campestris pv. campestris str. ATCC 33913]
>gi|21233473|ref|NP_639390.1| hypothetical protein XCC4051 [Xanthomonas campestris pv. campestris str. ATCC 33913]
>gi|21233476|ref|NP_639393.1| hypothetical protein XCC4054 [Xanthomonas campestris pv. campestris str. ATCC 33913]
>gi|21233487|ref|NP_639404.1| oxidoreductase [Xanthomonas campestris pv. campestris str. ATCC 33913]
>gi|21233489|ref|NP_639406.1| oxidoreductase [Xanthomonas campestris pv. campestris str. ATCC 33913]
>gi|21233512|ref|NP_639429.1| hypothetical protein XCC4090 [Xanthomonas campestris pv. campestris str. ATCC 33913]
>gi|21233514|ref|NP_639431.1| sec-independent protein translocase [Xanthomonas campestris pv. campestris str. ATCC 33913]
>gi|21233519|ref|NP_639436.1| hypothetical protein XCC4097 [Xanthomonas campestris pv. campestris str. ATCC 33913]
>gi|21233533|ref|NP_639450.1| histone [Xanthomonas campestris pv. campestris str. ATCC 33913]
>gi|21233557|ref|NP_639474.1| acid phosphatase [Xanthomonas campestris pv. campestris str. ATCC 33913]
>gi|21233558|ref|NP_639475.1| hypothetical protein XCC4136 [Xanthomonas campestris pv. campestris str. ATCC 33913]
>gi|21233578|ref|NP_639495.1| hypothetical protein XCC4161 [Xanthomonas campestris pv. campestris str. ATCC 33913]
>gi|21233580|ref|NP_639497.1| hypothetical protein XCC4163 [Xanthomonas campestris pv. campestris str. ATCC 33913]
>gi|21233585|ref|NP_639502.1| hypothetical protein XCC4168 [Xanthomonas campestris pv. campestris str. ATCC 33913]
>gi|21233588|ref|NP_639505.1| hypothetical protein XCC4171 [Xanthomonas campestris pv. campestris str. ATCC 33913]
>gi|21233594|ref|NP_639511.1| hypothetical protein XCC4178 [Xanthomonas campestris pv. campestris str. ATCC 33913]
>gi|21233596|ref|NP_639513.1| hypothetical protein XCC4179 [Xanthomonas campestris pv. campestris str. ATCC 33913]
>gi|21233615|ref|NP_639532.1| exodeoxyribonuclease V alpha chain [Xanthomonas campestris pv. campestris str. ATCC 33913]
>gi|21233616|ref|NP_639533.1| exodeoxyribonuclease V beta chain [Xanthomonas campestris pv. campestris str. ATCC 33913]
>gi|21233618|ref|NP_639535.1| hemagglutinin [Xanthomonas campestris pv. campestris str. ATCC 33913]
>gi|21233620|ref|NP_639537.1| microcystin dependent protein [Xanthomonas campestris pv. campestris str. ATCC 33913]
>gi|21233628|ref|NP_639545.1| hypothetical protein XCC4211 [Xanthomonas campestris pv. campestris str. ATCC 33913]
>gi|21233629|ref|NP_639546.1| lipoprotein [Xanthomonas campestris pv. campestris str. ATCC 33913]
>gi|21233631|ref|NP_639548.1| hypothetical protein XCC4214 [Xanthomonas campestris pv. campestris str. ATCC 33913]
>gi|21233636|ref|NP_639553.1| hypothetical protein XCC4219 [Xanthomonas campestris pv. campestris str. ATCC 33913]
>gi|21233640|ref|NP_639557.1| hypothetical protein XCC4223 [Xanthomonas campestris pv. campestris str. ATCC 33913]
>gi|21233643|ref|NP_639560.1| glycerate kinase [Xanthomonas campestris pv. campestris str. ATCC 33913]
>gi|21233644|ref|NP_639561.1| MFS transporter [Xanthomonas campestris pv. campestris str. ATCC 33913]

>gi|21233650|ref|NP_639567.1| hypothetical protein XCC4233 [Xanthomonas campestris pv. campestris str. ATCC 33913]
>gi|72080353|ref|YP_287411.1| heat shock protein [Mycoplasma hyopneumoniae 7448]
>gi|72080358|ref|YP_287416.1| hypothetical protein MHP7448_0016 [Mycoplasma hyopneumoniae 7448]
>gi|72080359|ref|YP_287417.1| hypothetical protein MHP7448_0017 [Mycoplasma hyopneumoniae 7448]
>gi|72080360|ref|YP_287418.1| hypothetical protein MHP7448_0018 [Mycoplasma hyopneumoniae 7448]
>gi|72080378|ref|YP_287436.1| hypothetical protein MHP7448_0036 [Mycoplasma hyopneumoniae 7448]
>gi|72080388|ref|YP_287446.1| hypothetical protein MHP7448_0046 [Mycoplasma hyopneumoniae 7448]
>gi|72080414|ref|YP_287472.1| hypothetical protein MHP7448_0072 [Mycoplasma hyopneumoniae 7448]
>gi|72080422|ref|YP_287480.1| hypothetical protein MHP7448_0080 [Mycoplasma hyopneumoniae 7448]
>gi|72080427|ref|YP_287485.1| hypothetical protein MHP7448_0085 [Mycoplasma hyopneumoniae 7448]
>gi|72080434|ref|YP_287492.1| hypothetical protein MHP7448_0092 [Mycoplasma hyopneumoniae 7448]
>gi|72080436|ref|YP_287494.1| hypothetical protein MHP7448_0094 [Mycoplasma hyopneumoniae 7448]
>gi|72080452|ref|YP_287510.1| hypothetical protein MHP7448_0112 [Mycoplasma hyopneumoniae 7448]
>gi|72080481|ref|YP_287539.1| hypothetical protein MHP7448_0142 [Mycoplasma hyopneumoniae 7448]
>gi|72080485|ref|YP_287543.1| chromate transport protein [Mycoplasma hyopneumoniae 7448]
>gi|72080501|ref|YP_287559.1| hypothetical protein MHP7448_0162 [Mycoplasma hyopneumoniae 7448]
>gi|72080515|ref|YP_287573.1| 50S ribosomal protein L15 [Mycoplasma hyopneumoniae 7448]
>gi|72080525|ref|YP_287583.1| 50S ribosomal protein L29 [Mycoplasma hyopneumoniae 7448]
>gi|72080531|ref|YP_287589.1| 50S ribosomal protein L23 [Mycoplasma hyopneumoniae 7448]
>gi|72080535|ref|YP_287593.1| hypothetical protein MHP7448_0196 [Mycoplasma hyopneumoniae 7448]
>gi|72080536|ref|YP_287594.1| hypothetical protein MHP7448_0197 [Mycoplasma hyopneumoniae 7448]
>gi|72080544|ref|YP_287602.1| hypothetical protein MHP7448_0205 [Mycoplasma hyopneumoniae 7448]
>gi|72080558|ref|YP_287616.1| hypothetical protein MHP7448_0219 [Mycoplasma hyopneumoniae 7448]
>gi|72080559|ref|YP_287617.1| hypothetical protein MHP7448_0220 [Mycoplasma hyopneumoniae 7448]
>gi|72080576|ref|YP_287634.1| hypothetical protein MHP7448_0237 [Mycoplasma hyopneumoniae 7448]
>gi|72080584|ref|YP_287642.1| 30S ribosomal protein S20 [Mycoplasma hyopneumoniae 7448]
>gi|72080587|ref|YP_287645.1| hypothetical protein MHP7448_0248 [Mycoplasma hyopneumoniae 7448]
>gi|72080604|ref|YP_287662.1| ABC transporter permease protein [Mycoplasma hyopneumoniae 7448]
>gi|72080620|ref|YP_287678.1| CDP-diacylglycerol--glycerol-3-phosphate 3-phosphatidyltransferase [Mycoplasma hyopneumoniae 7448]
>gi|72080634|ref|YP_287692.1| 30S ribosomal protein S6 [Mycoplasma hyopneumoniae 7448]
>gi|72080636|ref|YP_287694.1| hypothetical protein MHP7448_0298 [Mycoplasma hyopneumoniae 7448]
>gi|72080667|ref|YP_287725.1| hypothetical protein MHP7448_0330 [Mycoplasma hyopneumoniae 7448]
>gi|72080683|ref|YP_287741.1| hypothetical protein MHP7448_0347 [Mycoplasma hyopneumoniae 7448]
>gi|72080686|ref|YP_287744.1| hypothetical protein MHP7448_0350 [Mycoplasma hyopneumoniae 7448]
>gi|72080694|ref|YP_287752.1| glycerol uptake facilitator protein [Mycoplasma hyopneumoniae 7448]
>gi|72080698|ref|YP_287756.1| ABC transport system permease protein p69 - like [Mycoplasma hyopneumoniae 7448]
>gi|72080699|ref|YP_287757.1| hypothetical protein MHP7448_0363 [Mycoplasma hyopneumoniae 7448]
>gi|72080700|ref|YP_287758.1| hypothetical protein MHP7448_0364 [Mycoplasma hyopneumoniae 7448]
>gi|72080705|ref|YP_287763.1| ABC transporter permease protein [Mycoplasma hyopneumoniae 7448]
>gi|72080717|ref|YP_287775.1| ABC transporter permease protein [Mycoplasma hyopneumoniae 7448]
>gi|72080718|ref|YP_287776.1| proline dipeptidase [Mycoplasma hyopneumoniae 7448]
>gi|72080722|ref|YP_287780.1| hypothetical protein MHP7448_0387 [Mycoplasma hyopneumoniae 7448]
>gi|72080731|ref|YP_287789.1| hypothetical protein MHP7448_0397 [Mycoplasma hyopneumoniae 7448]
>gi|72080736|ref|YP_287794.1| hypothetical protein MHP7448_0402 [Mycoplasma hyopneumoniae 7448]
>gi|72080765|ref|YP_287823.1| hypothetical protein MHP7448_0432 [Mycoplasma hyopneumoniae 7448]
>gi|72080773|ref|YP_287831.1| hypothetical protein MHP7448_0440 [Mycoplasma hyopneumoniae 7448]
>gi|72080778|ref|YP_287836.1| hypothetical protein MHP7448_0446 [Mycoplasma hyopneumoniae 7448]
>gi|72080793|ref|YP_287851.1| hypothetical protein MHP7448_0461 [Mycoplasma hyopneumoniae 7448]
>gi|72080798|ref|YP_287856.1| hypothetical protein MHP7448_0466 [Mycoplasma hyopneumoniae 7448]
>gi|72080812|ref|YP_287870.1| hypothetical protein MHP7448_0481 [Mycoplasma hyopneumoniae 7448]
>gi|72080828|ref|YP_287886.1| p76 membrane protein precursor [Mycoplasma hyopneumoniae 7448]
>gi|72080829|ref|YP_287887.1| hypothetical protein MHP7448_0498 [Mycoplasma hyopneumoniae 7448]
>gi|72080834|ref|YP_287892.1| oligopeptide transport system permease protein [Mycoplasma hyopneumoniae 7448]
>gi|72080846|ref|YP_287904.1| xylose ABC transporter permease protein [Mycoplasma hyopneumoniae 7448]
>gi|72080849|ref|YP_287907.1| hypothetical protein MHP7448_0518 [Mycoplasma hyopneumoniae 7448]
>gi|72080850|ref|YP_287908.1| hypothetical protein MHP7448_0519 [Mycoplasma hyopneumoniae 7448]
>gi|72080864|ref|YP_287922.1| phosphatidate cytidylyltransferase synthase [Mycoplasma hyopneumoniae 7448]
>gi|72080874|ref|YP_287932.1| hypothetical protein MHP7448_0543 [Mycoplasma hyopneumoniae 7448]
>gi|72080886|ref|YP_287944.1| hypothetical protein MHP7448_0555 [Mycoplasma hyopneumoniae 7448]
>gi|72080887|ref|YP_287945.1| hypothetical protein MHP7448_0556 [Mycoplasma hyopneumoniae 7448]
>gi|72080909|ref|YP_287967.1| 5'-3' exonuclease [Mycoplasma hyopneumoniae 7448]
>gi|72080913|ref|YP_287971.1| hypothetical protein MHP7448_0585 [Mycoplasma hyopneumoniae 7448]
>gi|72080934|ref|YP_287992.1| ABC transporter protein [Mycoplasma hyopneumoniae 7448]
>gi|72080946|ref|YP_288004.1| 50S ribosomal protein L7/L12 [Mycoplasma hyopneumoniae 7448]
>gi|72080954|ref|YP_288012.1| ABC transporter ATP-binding - Pr1-like [Mycoplasma hyopneumoniae 7448]
>gi|72080955|ref|YP_288013.1| ABC transporter ATP-binding - Pr2 - like [Mycoplasma hyopneumoniae 7448]
>gi|72080972|ref|YP_288030.1| 50S ribosomal protein L9 [Mycoplasma hyopneumoniae 7448]
>gi|72080989|ref|YP_288047.1| hypothetical protein MHP7448_0662 [Mycoplasma hyopneumoniae 7448]
>gi|72080990|ref|YP_288048.1| adhesin like-protein P146 [Mycoplasma hyopneumoniae 7448]
>gi|72080993|ref|YP_288051.1| hypothetical protein MHP7448_0666 [Mycoplasma hyopneumoniae 7448]

>gi|15925788|ref|NP_373321.1| hypothetical protein SA0080 [Staphylococcus aureus subsp. aureus N315]
>gi|15925815|ref|NP_373348.1| Immunoglobulin G binding protein A precursor [Staphylococcus aureus subsp. aureus N315]
>gi|15925818|ref|NP_373351.1| lipoprotein [Staphylococcus aureus subsp. aureus N315]
>gi|15925829|ref|NP_373362.1| hypothetical protein SA0121 [Staphylococcus aureus subsp. aureus N315]
>gi|15925838|ref|NP_373371.1| hypothetical protein SA0129 [Staphylococcus aureus subsp. aureus N315]
>gi|15925980|ref|NP_373513.1| hypothetical protein SA0267 [Staphylococcus aureus subsp. aureus N315]
>gi|15925984|ref|NP_373517.1| hypothetical protein SA0271 [Staphylococcus aureus subsp. aureus N315]
>gi|15925997|ref|NP_373530.1| hypothetical protein SA0284 [Staphylococcus aureus subsp. aureus N315]
>gi|15926051|ref|NP_373584.1| hypothetical protein SA0338 [Staphylococcus aureus subsp. aureus N315]
>gi|15926075|ref|NP_373608.1| hypothetical protein SA0360 [Staphylococcus aureus subsp. aureus N315]
>gi|15926078|ref|NP_373611.1| hypothetical protein SA0362 [Staphylococcus aureus subsp. aureus N315]
>gi|15926101|ref|NP_373634.1| exotoxin 8 [Staphylococcus aureus subsp. aureus N315]
>gi|15926102|ref|NP_373635.1| exotoxin 9 [Staphylococcus aureus subsp. aureus N315]
>gi|15926206|ref|NP_373739.1| hypothetical protein SA0487 [Staphylococcus aureus subsp. aureus N315]
>gi|15926218|ref|NP_373751.1| 50S ribosomal protein L7/L12 [Staphylococcus aureus subsp. aureus N315]
>gi|15926239|ref|NP_373772.1| Ser-Asp rich fibrinogen-binding, bone sialoprotein-binding protein [Staphylococcus aureus subsp. aureus N315]
>gi|15926240|ref|NP_373773.1| Ser-Asp rich fibrinogen-binding, bone sialoprotein-binding protein [Staphylococcus aureus subsp. aureus N315]
>gi|15926241|ref|NP_373774.1| Ser-Asp rich fibrinogen-binding, bone sialoprotein-binding protein [Staphylococcus aureus subsp. aureus N315]
>gi|15926271|ref|NP_373804.1| hypothetical protein SA0550 [Staphylococcus aureus subsp. aureus N315]
>gi|15926288|ref|NP_373821.1| hypothetical protein SA0567 [Staphylococcus aureus subsp. aureus N315]
>gi|15926295|ref|NP_373828.1| hypothetical protein SA0574 [Staphylococcus aureus subsp. aureus N315]
>gi|15926325|ref|NP_373858.1| ferrichrome transport permease [Staphylococcus aureus subsp. aureus N315]
>gi|15926354|ref|NP_373887.1| hypothetical protein SA0632 [Staphylococcus aureus subsp. aureus N315]
>gi|15926359|ref|NP_373892.1| hypothetical protein SA0637 [Staphylococcus aureus subsp. aureus N315]
>gi|15926386|ref|NP_373919.1| hypothetical protein SA0664 [Staphylococcus aureus subsp. aureus N315]
>gi|15926393|ref|NP_373926.1| hypothetical protein SA0671 [Staphylococcus aureus subsp. aureus N315]
>gi|15926411|ref|NP_373944.1| hypothetical protein SA0689 [Staphylococcus aureus subsp. aureus N315]
>gi|15926424|ref|NP_373957.1| lipophilic protein affecting bacterial lysis rate and methicillin resistance level [Staphylococcus aureus subsp. aureus N315]
>gi|15926432|ref|NP_373965.1| hypothetical protein SA0710 [Staphylococcus aureus subsp. aureus N315]
>gi|15926447|ref|NP_373980.1| hypothetical protein SA0725 [Staphylococcus aureus subsp. aureus N315]
>gi|15926461|ref|NP_373994.1| hypothetical protein SA0739 [Staphylococcus aureus subsp. aureus N315]
>gi|15926464|ref|NP_373997.1| fibrinogen-binding protein A, clumping factor [Staphylococcus aureus subsp. aureus N315]
>gi|15926498|ref|NP_374031.1| hypothetical protein SA0770 [Staphylococcus aureus subsp. aureus N315]
>gi|15926536|ref|NP_374069.1| Na+/H+ antiporter subunit [Staphylococcus aureus subsp. aureus N315]
>gi|15926688|ref|NP_374221.1| hypothetical protein SA0954 [Staphylococcus aureus subsp. aureus N315]
>gi|15926690|ref|NP_374223.1| hypothetical protein SA0956 [Staphylococcus aureus subsp. aureus N315]
>gi|15926713|ref|NP_374246.1| hypothetical protein SA0976 [Staphylococcus aureus subsp. aureus N315]
>gi|15926714|ref|NP_374247.1| cell surface protein [Staphylococcus aureus subsp. aureus N315]
>gi|15926740|ref|NP_374273.1| hypothetical protein SA1002 [Staphylococcus aureus subsp. aureus N315]
>gi|15926904|ref|NP_374437.1| hypothetical protein SAS041 [Staphylococcus aureus subsp. aureus N315]
>gi|15926924|ref|NP_374457.1| hypothetical protein SA1178 [Staphylococcus aureus subsp. aureus N315]
>gi|15926956|ref|NP_374489.1| hypothetical protein SA1208 [Staphylococcus aureus subsp. aureus N315]
>gi|15926977|ref|NP_374510.1| tetrahydrodipicolinate acetyltransferase [Staphylococcus aureus subsp. aureus N315]
>gi|15927062|ref|NP_374595.1| elastin binding protein [Staphylococcus aureus subsp. aureus N315]
>gi|15927109|ref|NP_374642.1| translation elongation factor EF-P [Staphylococcus aureus subsp. aureus N315]
>gi|15927112|ref|NP_374645.1| hypothetical protein SA1362 [Staphylococcus aureus subsp. aureus N315]
>gi|15927149|ref|NP_374682.1| hypothetical protein SA1398 [Staphylococcus aureus subsp. aureus N315]
>gi|15927155|ref|NP_374688.1| 30S ribosomal protein S21 [Staphylococcus aureus subsp. aureus N315]
>gi|15927166|ref|NP_374699.1| 30S ribosomal protein S20 [Staphylococcus aureus subsp. aureus N315]
>gi|15927184|ref|NP_374717.1| hypothetical protein SA1432 [Staphylococcus aureus subsp. aureus N315]
>gi|15927195|ref|NP_374728.1| hypothetical protein SA1443 [Staphylococcus aureus subsp. aureus N315]
>gi|15927204|ref|NP_374737.1| hypothetical protein SAS049 [Staphylococcus aureus subsp. aureus N315]
>gi|15927205|ref|NP_374738.1| sigmaB-controlled gene product [Staphylococcus aureus subsp. aureus N315]
>gi|15927231|ref|NP_374764.1| hypothetical protein SA1477 [Staphylococcus aureus subsp. aureus N315]
>gi|15927308|ref|NP_374841.1| hypothetical protein SA1552 [Staphylococcus aureus subsp. aureus N315]
>gi|15927315|ref|NP_374848.1| hypothetical protein SA1559 [Staphylococcus aureus subsp. aureus N315]
>gi|15927356|ref|NP_374889.1| hypothetical protein SA1600 [Staphylococcus aureus subsp. aureus N315]
>gi|15927373|ref|NP_374906.1| hypothetical protein SA1617 [Staphylococcus aureus subsp. aureus N315]
>gi|15927374|ref|NP_374907.1| hypothetical protein SA1618 [Staphylococcus aureus subsp. aureus N315]
>gi|15927396|ref|NP_374929.1| hypothetical protein SA1640 [Staphylococcus aureus subsp. aureus N315]
>gi|15927415|ref|NP_374948.1| hypothetical protein SA1659 [Staphylococcus aureus subsp. aureus N315]
>gi|15927419|ref|NP_374952.1| hypothetical protein SA1663 [Staphylococcus aureus subsp. aureus N315]
>gi|15927501|ref|NP_375034.1| hypothetical protein SA1741 [Staphylococcus aureus subsp. aureus N315]
>gi|15927504|ref|NP_375037.1| hypothetical protein SA1744 [Staphylococcus aureus subsp. aureus N315]
>gi|15927516|ref|NP_375049.1| hypothetical protein SA1754 [Staphylococcus aureus subsp. aureus N315]
>gi|15927554|ref|NP_375087.1| hypothetical protein SA1788 [Staphylococcus aureus subsp. aureus N315]
>gi|15927586|ref|NP_375119.1| hypothetical protein SA1818 [Staphylococcus aureus subsp. aureus N315]
>gi|15927599|ref|NP_375132.1| hypothetical protein SA1831 [Staphylococcus aureus subsp. aureus N315]
>gi|15927607|ref|NP_375140.1| hypothetical protein SA1839 [Staphylococcus aureus subsp. aureus N315]

>gi|15927611|ref|NP_375144.1| accessory gene regulator B [Staphylococcus aureus subsp. aureus N315]
>gi|15927702|ref|NP_375235.1| probable DNA-directed RNA polymerase delta subunit [Staphylococcus aureus subsp. aureus N315]
>gi|15927728|ref|NP_375261.1| lytic regulatory protein truncated with Tn554 [Staphylococcus aureus subsp. aureus N315]
>gi|15927741|ref|NP_375274.1| FmtB protein [Staphylococcus aureus subsp. aureus N315]
>gi|15927755|ref|NP_375288.1| hypothetical protein SA1977 [Staphylococcus aureus subsp. aureus N315]
>gi|15927843|ref|NP_375376.1| hypothetical protein SA2058 [Staphylococcus aureus subsp. aureus N315]
>gi|15927862|ref|NP_375395.1| hypothetical protein SA2077 [Staphylococcus aureus subsp. aureus N315]
>gi|15927879|ref|NP_375412.1| hypothetical protein SA2093 [Staphylococcus aureus subsp. aureus N315]
>gi|15927883|ref|NP_375416.1| hypothetical protein SA2096 [Staphylococcus aureus subsp. aureus N315]
>gi|15927884|ref|NP_375417.1| hypothetical protein SA2097 [Staphylococcus aureus subsp. aureus N315]
>gi|15927897|ref|NP_375430.1| hypothetical protein SA2110 [Staphylococcus aureus subsp. aureus N315]
>gi|15927907|ref|NP_375440.1| hypothetical protein SA2118 [Staphylococcus aureus subsp. aureus N315]
>gi|15927933|ref|NP_375466.1| hypothetical protein SA2143 [Staphylococcus aureus subsp. aureus N315]
>gi|15927948|ref|NP_375481.1| hypothetical protein SA2158 [Staphylococcus aureus subsp. aureus N315]
>gi|15927961|ref|NP_375494.1| hypothetical protein SA2171 [Staphylococcus aureus subsp. aureus N315]
>gi|15927996|ref|NP_375529.1| IgG-binding protein SBI [Staphylococcus aureus subsp. aureus N315]
>gi|15928000|ref|NP_375533.1| hypothetical protein SA2210 [Staphylococcus aureus subsp. aureus N315]
>gi|15928011|ref|NP_375544.1| hypothetical protein SA2221 [Staphylococcus aureus subsp. aureus N315]
>gi|15928017|ref|NP_375550.1| hypothetical protein SA2227 [Staphylococcus aureus subsp. aureus N315]
>gi|15928073|ref|NP_375606.1| hypothetical protein SA2282 [Staphylococcus aureus subsp. aureus N315]
>gi|15928081|ref|NP_375614.1| hypothetical protein SA2290 [Staphylococcus aureus subsp. aureus N315]
>gi|15928082|ref|NP_375615.1| hypothetical protein SA2291 [Staphylococcus aureus subsp. aureus N315]
>gi|15928094|ref|NP_375627.1| hypothetical protein SA2303 [Staphylococcus aureus subsp. aureus N315]
>gi|15928106|ref|NP_375639.1| hypothetical protein SA2315 [Staphylococcus aureus subsp. aureus N315]
>gi|15928107|ref|NP_375640.1| sortase [Staphylococcus aureus subsp. aureus N315]
>gi|15928109|ref|NP_375642.1| hypothetical protein SA2318 [Staphylococcus aureus subsp. aureus N315]
>gi|15928145|ref|NP_375678.1| hypothetical protein SA2353 [Staphylococcus aureus subsp. aureus N315]
>gi|15928148|ref|NP_375681.1| immunodominant antigen A [Staphylococcus aureus subsp. aureus N315]
>gi|15928216|ref|NP_375749.1| Clumping factor B [Staphylococcus aureus subsp. aureus N315]
>gi|15928225|ref|NP_375758.1| hypothetical protein SA2432 [Staphylococcus aureus subsp. aureus N315]
>gi|15928230|ref|NP_375763.1| hypothetical protein SA2437 [Staphylococcus aureus subsp. aureus N315]
>gi|15928240|ref|NP_375773.1| hypothetical protein SA2447 [Staphylococcus aureus subsp. aureus N315]
>gi|15928277|ref|NP_375810.1| hypothetical protein SA2483 [Staphylococcus aureus subsp. aureus N315]
>gi|15928285|ref|NP_375818.1| hypothetical protein SA2491 [Staphylococcus aureus subsp. aureus N315]
>gi|15928292|ref|NP_375825.1| hypothetical protein SA2497 [Staphylococcus aureus subsp. aureus N315]
>gi|15928299|ref|NP_375832.1| 50S ribosomal protein L34 [Staphylococcus aureus subsp. aureus N315]
>gi|16119203|ref|NP_395539.1| hypothetical protein SAP003 [Staphylococcus aureus subsp. aureus N315]
>gi|16119204|ref|NP_395540.1| hypothetical protein SAP004 [Staphylococcus aureus subsp. aureus N315]
>gi|58584285|ref|YP_197858.1| HesB/YadR/YfhF family protein [Wolbachia endosymbiont strain TRS of Brugia malayi]
>gi|58584337|ref|YP_197910.1| WAS family protein [Wolbachia endosymbiont strain TRS of Brugia malayi]
>gi|58584342|ref|YP_197915.1| hypothetical protein Wbm0081 [Wolbachia endosymbiont strain TRS of Brugia malayi]
>gi|58584400|ref|YP_197973.1| hypothetical protein Wbm0140 [Wolbachia endosymbiont strain TRS of Brugia malayi]
>gi|58584423|ref|YP_197996.1| hypothetical protein Wbm0163 [Wolbachia endosymbiont strain TRS of Brugia malayi]
>gi|58584424|ref|YP_197997.1| hypothetical protein Wbm0164 [Wolbachia endosymbiont strain TRS of Brugia malayi]
>gi|58584425|ref|YP_197998.1| hypothetical protein Wbm0165 [Wolbachia endosymbiont strain TRS of Brugia malayi]
>gi|58584435|ref|YP_198008.1| ABC-type Mn2+/Zn2+ transport system, permease component [Wolbachia endosymbiont strain TRS of Brugia malayi]
>gi|58584528|ref|YP_198101.1| hypothetical protein Wbm0270 [Wolbachia endosymbiont strain TRS of Brugia malayi]
>gi|58584549|ref|YP_198122.1| Predicted membrane protein WF-2 [Wolbachia endosymbiont strain TRS of Brugia malayi]
>gi|58584554|ref|YP_198127.1| Ankyrin repeat-containing protein [Wolbachia endosymbiont strain TRS of Brugia malayi]
>gi|58584581|ref|YP_198154.1| Ribosomal protein L15 [Wolbachia endosymbiont strain TRS of Brugia malayi]
>gi|58584724|ref|YP_198297.1| hypothetical protein Wbm0467 [Wolbachia endosymbiont strain TRS of Brugia malayi]
>gi|58584778|ref|YP_198351.1| Ribosomal protein S21 [Wolbachia endosymbiont strain TRS of Brugia malayi]
>gi|58584836|ref|YP_198409.1| DnaK suppressor protein [Wolbachia endosymbiont strain TRS of Brugia malayi]
>gi|58584867|ref|YP_198440.1| hypothetical protein Wbm0610 [Wolbachia endosymbiont strain TRS of Brugia malayi]
>gi|58584929|ref|YP_198502.1| hypothetical protein Wbm0672 [Wolbachia endosymbiont strain TRS of Brugia malayi]
>gi|58584936|ref|YP_198509.1| Uncharacterized ABC-type transport system, permease component [Wolbachia endosymbiont strain TRS of Brugia malayi]
>gi|58585005|ref|YP_198578.1| hypothetical protein Wbm0748 [Wolbachia endosymbiont strain TRS of Brugia malayi]
>gi|58585022|ref|YP_198595.1| hypothetical protein Wbm0765 [Wolbachia endosymbiont strain TRS of Brugia malayi]
>gi|58585029|ref|YP_198602.1| hypothetical protein Wbm0772 [Wolbachia endosymbiont strain TRS of Brugia malayi]
>gi|58585038|ref|YP_198611.1| hypothetical protein Wbm0781 [Wolbachia endosymbiont strain TRS of Brugia malayi]
>gi|58585060|ref|YP_198633.1| CDP-diglyceride synthetase [Wolbachia endosymbiont strain TRS of Brugia malayi]
>gi|68248645|ref|YP_247757.1| conserved putative lipoprotein [Haemophilus influenzae 86-028NP]
>gi|68248703|ref|YP_247815.1| hypothetical protein NTHI0171 [Haemophilus influenzae 86-028NP]
>gi|68248732|ref|YP_247844.1| high-affinity zinc uptake system protein ZnuA [Haemophilus influenzae 86-028NP]
>gi|68248743|ref|YP_247855.1| probable sugar efflux transporter [Haemophilus influenzae 86-028NP]
>gi|68248757|ref|YP_247869.1| putative protein-S-isoprenylcysteine methyltransferase [Haemophilus influenzae 86-028NP]
>gi|68248792|ref|YP_247904.1| Sec-independent protein translocase protein TatB [Haemophilus influenzae 86-028NP]
>gi|68248858|ref|YP_247970.1| TonB [Haemophilus influenzae 86-028NP]

>gi|68248909|ref|YP_248021.1| hypothetical protein NTHI0420 [Haemophilus influenzae 86-028NP]
>gi|68248959|ref|YP_248071.1| putative ABC-type nitrate/sulfonate/bicarbonate transport system, permease component [Haemophilus influenzae 86-028NP]
>gi|68248966|ref|YP_248078.1| hypothetical protein NTHI0482 [Haemophilus influenzae 86-028NP]
>gi|68248986|ref|YP_248098.1| TolA [Haemophilus influenzae 86-028NP]
>gi|68249045|ref|YP_248157.1| protein-export membrane protein SecG [Haemophilus influenzae 86-028NP]
>gi|68249128|ref|YP_248240.1| 30S ribosomal protein S21 [Haemophilus influenzae 86-028NP]
>gi|68249141|ref|YP_248253.1| 50S ribosomal protein L9 [Haemophilus influenzae 86-028NP]
>gi|68249147|ref|YP_248259.1| UDP-Gal--lipooligosaccharide galactosyltransferase [Haemophilus influenzae 86-028NP]
>gi|68249156|ref|YP_248268.1| hypothetical protein NTHI0688 [Haemophilus influenzae 86-028NP]
>gi|68249192|ref|YP_248304.1| DNA transformation protein TfoX [Haemophilus influenzae 86-028NP]
>gi|68249219|ref|YP_248331.1| 50S ribosomal protein L7/L12 [Haemophilus influenzae 86-028NP]
>gi|68249300|ref|YP_248412.1| PsiE [Haemophilus influenzae 86-028NP]
>gi|68249352|ref|YP_248464.1| predicted membrane-bound metallopeptidase [Haemophilus influenzae 86-028NP]
>gi|68249390|ref|YP_248502.1| 50S ribosomal protein L18 [Haemophilus influenzae 86-028NP]
>gi|68249393|ref|YP_248505.1| 50S ribosomal protein L15 [Haemophilus influenzae 86-028NP]
>gi|68249419|ref|YP_248531.1| galactoside transport system permease protein MglC [Haemophilus influenzae 86-028NP]
>gi|68249501|ref|YP_248613.1| UDP-3-O-[3-hydroxymyristoyl] glucosamine N-acyltransferase [Haemophilus influenzae 86-028NP]
>gi|68249511|ref|YP_248623.1| hypothetical protein NTHI1094 [Haemophilus influenzae 86-028NP]
>gi|68249553|ref|YP_248665.1| 30S ribosomal protein S20 [Haemophilus influenzae 86-028NP]
>gi|68249558|ref|YP_248670.1| biotin carboxyl carrier protein of acetyl-CoA carboxylase [Haemophilus influenzae 86-028NP]
>gi|68249599|ref|YP_248711.1| magnesium and cobalt transport protein CorA [Haemophilus influenzae 86-028NP]
>gi|68249615|ref|YP_248727.1| conserved putative gamma-carboxymuconolactone decarboxylase subunit [Haemophilus influenzae 86-028NP]
>gi|68249647|ref|YP_248759.1| heme exporter protein B [Haemophilus influenzae 86-028NP]
>gi|68249809|ref|YP_248921.1| hypothetical protein NTHI1441 [Haemophilus influenzae 86-028NP]
>gi|68249836|ref|YP_248948.1| Outer membrane lipoprotein PCP precursor [Haemophilus influenzae 86-028NP]
>gi|68249850|ref|YP_248962.1| hypothetical protein NTHI1489 [Haemophilus influenzae 86-028NP]
>gi|68249903|ref|YP_249015.1| hypothetical protein NTHI1548 [Haemophilus influenzae 86-028NP]
>gi|68249919|ref|YP_249031.1| hypothetical protein NTHI1567 [Haemophilus influenzae 86-028NP]
>gi|68249923|ref|YP_249035.1| hypothetical protein NTHI1571 [Haemophilus influenzae 86-028NP]
>gi|68249966|ref|YP_249078.1| phosphatidylglycerophosphatase A [Haemophilus influenzae 86-028NP]
>gi|68249988|ref|YP_249100.1| 50S ribosomal protein L35 [Haemophilus influenzae 86-028NP]
>gi|68249996|ref|YP_249108.1| hypothetical protein NTHI1651 [Haemophilus influenzae 86-028NP]
>gi|68250008|ref|YP_249120.1| hypothetical protein NTHI1667 [Haemophilus influenzae 86-028NP]
>gi|68250010|ref|YP_249122.1| hypothetical protein NTHI1669 [Haemophilus influenzae 86-028NP]
>gi|68250072|ref|YP_249184.1| hypothetical protein NTHI1737 [Haemophilus influenzae 86-028NP]
>gi|68250158|ref|YP_249270.1| translation initiation factor IF-2 [Haemophilus influenzae 86-028NP]
>gi|68250172|ref|YP_249284.1| predicted phage P2-like baseplate assembly protein [Haemophilus influenzae 86-028NP]
>gi|68250178|ref|YP_249290.1| hypothetical protein NTHI1869 [Haemophilus influenzae 86-028NP]
>gi|68250227|ref|YP_249339.1| hypothetical protein NTHI1924 [Haemophilus influenzae 86-028NP]
>gi|68250230|ref|YP_249342.1| chaperone protein DnaJ [Haemophilus influenzae 86-028NP]
>gi|68250235|ref|YP_249347.1| dihydrolipoamide acetyltransferase component of pyruvate dehydrogenase complex [Haemophilus influenzae 86-028NP]
>gi|68250270|ref|YP_249382.1| predicted activator of osmoprotectant transporter ProP [Haemophilus influenzae 86-028NP]
>gi|68250287|ref|YP_249399.1| predicted NADH:ubiquinone oxidoreductase, subunit RnfA [Haemophilus influenzae 86-028NP]
>gi|68250335|ref|YP_249447.1| predicted branched-chain amino acid permease [Haemophilus influenzae 86-028NP]
>gi|28492979|ref|NP_787140.1| hypothetical protein TWT012 [Tropheryma whipplei str. Twist]
>gi|28492995|ref|NP_787156.1| hypothetical protein TWT028 [Tropheryma whipplei str. Twist]
>gi|28492996|ref|NP_787157.1| dTDP-4-dehydrorhamnose reductase [Tropheryma whipplei str. Twist]
>gi|28493008|ref|NP_787169.1| hypothetical protein TWT041 [Tropheryma whipplei str. Twist]
>gi|28493009|ref|NP_787170.1| hypothetical protein TWT042 [Tropheryma whipplei str. Twist]
>gi|28493052|ref|NP_787213.1| hypothetical protein TWT085 [Tropheryma whipplei str. Twist]
>gi|28493066|ref|NP_787227.1| hypothetical protein TWT099 [Tropheryma whipplei str. Twist]
>gi|28493072|ref|NP_787233.1| 50S ribosomal protein L9 [Tropheryma whipplei str. Twist]
>gi|28493080|ref|NP_787241.1| 50S ribosomal protein L28 [Tropheryma whipplei str. Twist]
>gi|28493101|ref|NP_787262.1| hypothetical protein TWT134 [Tropheryma whipplei str. Twist]
>gi|28493115|ref|NP_787276.1| hypothetical protein TWT148 [Tropheryma whipplei str. Twist]
>gi|28493118|ref|NP_787279.1| hypothetical protein TWT151 [Tropheryma whipplei str. Twist]
>gi|28493120|ref|NP_787281.1| hypothetical protein TWT153 [Tropheryma whipplei str. Twist]
>gi|28493125|ref|NP_787286.1| hypothetical protein TWT158 [Tropheryma whipplei str. Twist]
>gi|28493143|ref|NP_787304.1| hypothetical protein TWT176 [Tropheryma whipplei str. Twist]
>gi|28493148|ref|NP_787309.1| hypothetical protein TWT181 [Tropheryma whipplei str. Twist]
>gi|28493152|ref|NP_787313.1| hypothetical protein TWT185 [Tropheryma whipplei str. Twist]
>gi|28493164|ref|NP_787325.1| multidrug efflux protein [Tropheryma whipplei str. Twist]
>gi|28493169|ref|NP_787330.1| hypothetical protein TWT202 [Tropheryma whipplei str. Twist]
>gi|28493178|ref|NP_787339.1| biotin synthesis BioY protein [Tropheryma whipplei str. Twist]
>gi|28493179|ref|NP_787340.1| hypothetical protein TWT212 [Tropheryma whipplei str. Twist]
>gi|28493200|ref|NP_787361.1| hypothetical protein TWT233 [Tropheryma whipplei str. Twist]
>gi|28493207|ref|NP_787368.1| hypothetical protein TWT240 [Tropheryma whipplei str. Twist]
>gi|28493237|ref|NP_787398.1| hypothetical protein TWT270 [Tropheryma whipplei str. Twist]

>gi|28493270|ref|NP_787431.1| preprotein translocase SecG subunit [Tropheryma whipplei str. Twist]
>gi|28493273|ref|NP_787434.1| hypothetical protein TWT306 [Tropheryma whipplei str. Twist]
>gi|28493290|ref|NP_787451.1| hypothetical protein TWT323 [Tropheryma whipplei str. Twist]
>gi|28493311|ref|NP_787472.1| hypothetical protein TWT344 [Tropheryma whipplei str. Twist]
>gi|28493376|ref|NP_787537.1| hypothetical protein TWT409 [Tropheryma whipplei str. Twist]
>gi|28493428|ref|NP_787589.1| 30S ribosomal protein S16 [Tropheryma whipplei str. Twist]
>gi|28493449|ref|NP_787610.1| hypothetical protein TWT482 [Tropheryma whipplei str. Twist]
>gi|28493480|ref|NP_787641.1| cell division protein DivIVA-like protein [Tropheryma whipplei str. Twist]
>gi|28493494|ref|NP_787655.1| 50S ribosomal protein L17 [Tropheryma whipplei str. Twist]
>gi|28493505|ref|NP_787666.1| 30S ribosomal protein S5 [Tropheryma whipplei str. Twist]
>gi|28493511|ref|NP_787672.1| 50S ribosomal protein L14 [Tropheryma whipplei str. Twist]
>gi|28493527|ref|NP_787688.1| branched-chain amino acid ABC transporter permease protein [Tropheryma whipplei str. Twist]
>gi|28493536|ref|NP_787697.1| hypothetical protein TWT569 [Tropheryma whipplei str. Twist]
>gi|28493539|ref|NP_787700.1| hypothetical protein TWT572 [Tropheryma whipplei str. Twist]
>gi|28493547|ref|NP_787708.1| hypothetical protein TWT580 [Tropheryma whipplei str. Twist]
>gi|28493552|ref|NP_787713.1| hypothetical protein TWT585 [Tropheryma whipplei str. Twist]
>gi|28493575|ref|NP_787736.1| hypothetical protein TWT608 [Tropheryma whipplei str. Twist]
>gi|28493580|ref|NP_787741.1| hypothetical protein TWT613 [Tropheryma whipplei str. Twist]
>gi|28493584|ref|NP_787745.1| hypothetical protein TWT617 [Tropheryma whipplei str. Twist]
>gi|28493591|ref|NP_787752.1| hypothetical protein TWT624 [Tropheryma whipplei str. Twist]
>gi|28493640|ref|NP_787801.1| hypothetical protein TWT673 [Tropheryma whipplei str. Twist]
>gi|28493678|ref|NP_787839.1| 50S ribosomal protein L7/L12 [Tropheryma whipplei str. Twist]
>gi|28493728|ref|NP_787889.1| hypothetical protein TWT761 [Tropheryma whipplei str. Twist]
>gi|28493734|ref|NP_787895.1| hypothetical protein TWT767 [Tropheryma whipplei str. Twist]
>gi|28493746|ref|NP_787907.1| hypothetical protein TWT779 [Tropheryma whipplei str. Twist]
>gi|28493751|ref|NP_787912.1| hypothetical protein TWT784 [Tropheryma whipplei str. Twist]
>gi|34495463|ref|NP_899678.1| hypothetical protein CV0008 [Chromobacterium violaceum ATCC 12472]
>gi|34495475|ref|NP_899690.1| probable MFS transporter [Chromobacterium violaceum ATCC 12472]
>gi|34495477|ref|NP_899692.1| hypothetical protein CV0022 [Chromobacterium violaceum ATCC 12472]
>gi|34495492|ref|NP_899707.1| hypothetical protein CV0037 [Chromobacterium violaceum ATCC 12472]
>gi|34495494|ref|NP_899709.1| hypothetical protein CV0039 [Chromobacterium violaceum ATCC 12472]
>gi|34495496|ref|NP_899711.1| probable transmembrane protein [Chromobacterium violaceum ATCC 12472]
>gi|34495498|ref|NP_899713.1| hypothetical protein CV0043 [Chromobacterium violaceum ATCC 12472]
>gi|34495501|ref|NP_899716.1| pleD gene product [Chromobacterium violaceum ATCC 12472]
>gi|34495502|ref|NP_899717.1| probable transcriptional regulator, MarR family [Chromobacterium violaceum ATCC 12472]
>gi|34495503|ref|NP_899718.1| probable transport transmembrane protein [Chromobacterium violaceum ATCC 12472]
>gi|34495505|ref|NP_899720.1| protein porphyrin biosynthesis [Chromobacterium violaceum ATCC 12472]
>gi|34495507|ref|NP_899722.1| uroporphyrinogen-III synthase [Chromobacterium violaceum ATCC 12472]
>gi|34495513|ref|NP_899728.1| hypothetical protein CV0058 [Chromobacterium violaceum ATCC 12472]
>gi|34495516|ref|NP_899731.1| probable transcriptional regulator, LysR family [Chromobacterium violaceum ATCC 12472]
>gi|34495517|ref|NP_899732.1| probable transmembrane protein [Chromobacterium violaceum ATCC 12472]
>gi|34495519|ref|NP_899734.1| hypothetical protein CV0064 [Chromobacterium violaceum ATCC 12472]
>gi|34495536|ref|NP_899751.1| probable cytochrome c4 [Chromobacterium violaceum ATCC 12472]
>gi|34495537|ref|NP_899752.1| hypothetical protein CV0082 [Chromobacterium violaceum ATCC 12472]
>gi|34495538|ref|NP_899753.1| hypothetical protein CV0083 [Chromobacterium violaceum ATCC 12472]
>gi|34495553|ref|NP_899768.1| hypothetical protein CV0098 [Chromobacterium violaceum ATCC 12472]
>gi|34495556|ref|NP_899771.1| NAD(P) transhydrogenase, alpha subunit [Chromobacterium violaceum ATCC 12472]
>gi|34495558|ref|NP_899773.1| probable two-component system sensor protein [Chromobacterium violaceum ATCC 12472]
>gi|34495563|ref|NP_899778.1| hypothetical protein CV0108 [Chromobacterium violaceum ATCC 12472]
>gi|34495573|ref|NP_899788.1| glutamate/aspartate transport system permease protein [Chromobacterium violaceum ATCC 12472]
>gi|34495577|ref|NP_899792.1| probable multidrug resistance protein [Chromobacterium violaceum ATCC 12472]
>gi|34495586|ref|NP_899801.1| probable benzoate membrane transport protein [Chromobacterium violaceum ATCC 12472]
>gi|34495590|ref|NP_899805.1| hypothetical protein CV0135 [Chromobacterium violaceum ATCC 12472]
>gi|34495596|ref|NP_899811.1| probable transmembrane efflux protein [Chromobacterium violaceum ATCC 12472]
>gi|34495602|ref|NP_899817.1| glucokinase [Chromobacterium violaceum ATCC 12472]
>gi|34495605|ref|NP_899820.1| probable thiamine-phosphate diphosphorylase [Chromobacterium violaceum ATCC 12472]
>gi|34495610|ref|NP_899825.1| hypothetical protein CV0155 [Chromobacterium violaceum ATCC 12472]
>gi|34495612|ref|NP_899827.1| hypothetical protein CV0157 [Chromobacterium violaceum ATCC 12472]
>gi|34495614|ref|NP_899829.1| sulfite dehydrogenase - subunitB [Chromobacterium violaceum ATCC 12472]
>gi|34495615|ref|NP_899830.1| hypothetical protein CV0160 [Chromobacterium violaceum ATCC 12472]
>gi|34495626|ref|NP_899841.1| hypothetical protein CV0171 [Chromobacterium violaceum ATCC 12472]
>gi|34495627|ref|NP_899842.1| poly (3-hydroxybutyrate) depolymerase [Chromobacterium violaceum ATCC 12472]
>gi|34495629|ref|NP_899844.1| DnaK suppressor protein [Chromobacterium violaceum ATCC 12472]
>gi|34495631|ref|NP_899846.1| hypothetical protein CV0176 [Chromobacterium violaceum ATCC 12472]
>gi|34495633|ref|NP_899848.1| hypothetical protein CV0178 [Chromobacterium violaceum ATCC 12472]
>gi|34495638|ref|NP_899853.1| 2'-5' RNA ligase [Chromobacterium violaceum ATCC 12472]
>gi|34495647|ref|NP_899862.1| hypothetical protein CV0192 [Chromobacterium violaceum ATCC 12472]
>gi|34495653|ref|NP_899868.1| hypothetical protein CV0198 [Chromobacterium violaceum ATCC 12472]
>gi|34495654|ref|NP_899869.1| hypothetical protein CV0199 [Chromobacterium violaceum ATCC 12472]
>gi|34495655|ref|NP_899870.1| hypothetical protein CV0200 [Chromobacterium violaceum ATCC 12472]

>gi|34495669|ref|NP_899884.1| hypothetical protein CV0214 [Chromobacterium violaceum ATCC 12472]
>gi|34495674|ref|NP_899889.1| probable transmembrane protein [Chromobacterium violaceum ATCC 12472]
>gi|34495676|ref|NP_899891.1| hypothetical protein CV0221 [Chromobacterium violaceum ATCC 12472]
>gi|34495684|ref|NP_899899.1| hypothetical protein CV0229 [Chromobacterium violaceum ATCC 12472]
>gi|34495696|ref|NP_899911.1| probable pts system, nitrogen regulatory IIA component [Chromobacterium violaceum ATCC 12472]
>gi|34495697|ref|NP_899912.1| hypothetical protein CV0242 [Chromobacterium violaceum ATCC 12472]
>gi|34495727|ref|NP_899942.1| hypothetical protein CV0272 [Chromobacterium violaceum ATCC 12472]
>gi|34495736|ref|NP_899951.1| hypothetical protein CV0281 [Chromobacterium violaceum ATCC 12472]
>gi|34495737|ref|NP_899952.1| hypothetical protein CV0282 [Chromobacterium violaceum ATCC 12472]
>gi|34495738|ref|NP_899953.1| hypothetical protein CV0283 [Chromobacterium violaceum ATCC 12472]
>gi|34495739|ref|NP_899954.1| hypothetical protein CV0284 [Chromobacterium violaceum ATCC 12472]
>gi|34495751|ref|NP_899966.1| hypothetical protein CV0296 [Chromobacterium violaceum ATCC 12472]
>gi|34495754|ref|NP_899969.1| probable transcriptional regulator [Chromobacterium violaceum ATCC 12472]
>gi|34495756|ref|NP_899971.1| hypothetical protein CV0301 [Chromobacterium violaceum ATCC 12472]
>gi|34495759|ref|NP_899974.1| rarD protein, chloamphenicol sensitive [Chromobacterium violaceum ATCC 12472]
>gi|34495765|ref|NP_899980.1| hypothetical protein CV0310 [Chromobacterium violaceum ATCC 12472]
>gi|34495767|ref|NP_899982.1| hypothetical protein CV0312 [Chromobacterium violaceum ATCC 12472]
>gi|34495774|ref|NP_899989.1| hypothetical protein CV0319 [Chromobacterium violaceum ATCC 12472]
>gi|34495787|ref|NP_900002.1| hypothetical protein CV0332 [Chromobacterium violaceum ATCC 12472]
>gi|34495791|ref|NP_900006.1| hypothetical protein CV0336 [Chromobacterium violaceum ATCC 12472]
>gi|34495794|ref|NP_900009.1| probable bacteriophge tail fiber protein [Chromobacterium violaceum ATCC 12472]
>gi|34495800|ref|NP_900015.1| probable bacteriophage base plate protein [Chromobacterium violaceum ATCC 12472]
>gi|34495803|ref|NP_900018.1| hypothetical protein CV0348 [Chromobacterium violaceum ATCC 12472]
>gi|34495805|ref|NP_900020.1| probable phage sheath protein [Chromobacterium violaceum ATCC 12472]
>gi|34495826|ref|NP_900041.1| hypothetical protein CV0371 [Chromobacterium violaceum ATCC 12472]
>gi|34495838|ref|NP_900053.1| ATP-dependent RNA helicase [Chromobacterium violaceum ATCC 12472]
>gi|34495843|ref|NP_900058.1| hypothetical protein CV0388 [Chromobacterium violaceum ATCC 12472]
>gi|34495847|ref|NP_900062.1| probable transcription regulator protein [Chromobacterium violaceum ATCC 12472]
>gi|34495853|ref|NP_900068.1| biopolymer transport exbD transmembrane protein [Chromobacterium violaceum ATCC 12472]
>gi|34495854|ref|NP_900069.1| probable exbB-like biopolymer transport [Chromobacterium violaceum ATCC 12472]
>gi|34495855|ref|NP_900070.1| hypothetical protein CV0400 [Chromobacterium violaceum ATCC 12472]
>gi|34495868|ref|NP_900083.1| hypothetical protein CV0413 [Chromobacterium violaceum ATCC 12472]
>gi|34495872|ref|NP_900087.1| probable phage baseplate component [Chromobacterium violaceum ATCC 12472]
>gi|34495874|ref|NP_900089.1| probable tail fiber assembly-like protein [Chromobacterium violaceum ATCC 12472]
>gi|34495888|ref|NP_900103.1| outer membrane efflux protein [Chromobacterium violaceum ATCC 12472]
>gi|34495900|ref|NP_900115.1| hypothetical protein CV0445 [Chromobacterium violaceum ATCC 12472]
>gi|34495916|ref|NP_900131.1| probable cytochrome P-450 [Chromobacterium violaceum ATCC 12472]
>gi|34495918|ref|NP_900133.1| hypothetical protein CV0463 [Chromobacterium violaceum ATCC 12472]
>gi|34495919|ref|NP_900134.1| hypothetical protein CV0464 [Chromobacterium violaceum ATCC 12472]
>gi|34495922|ref|NP_900137.1| hypothetical protein CV0467 [Chromobacterium violaceum ATCC 12472]
>gi|34495926|ref|NP_900141.1| competence protein [Chromobacterium violaceum ATCC 12472]
>gi|34495929|ref|NP_900144.1| hypothetical protein CV0474 [Chromobacterium violaceum ATCC 12472]
>gi|34495943|ref|NP_900158.1| hypothetical protein CV0488 [Chromobacterium violaceum ATCC 12472]
>gi|34495946|ref|NP_900161.1| cobalamin (5'-phosphate) synthase [Chromobacterium violaceum ATCC 12472]
>gi|34495951|ref|NP_900166.1| probable substrate-binding periplasmatic (PBP) ABC transporter protein [Chromobacterium violaceum ATCC 12472]
>gi|34495954|ref|NP_900169.1| probable 5-formyltetrahydrofolate cyclo-ligase [Chromobacterium violaceum ATCC 12472]
>gi|34495956|ref|NP_900171.1| hypothetical protein CV0501 [Chromobacterium violaceum ATCC 12472]
>gi|34495958|ref|NP_900173.1| hypothetical protein CV0503 [Chromobacterium violaceum ATCC 12472]
>gi|34495963|ref|NP_900178.1| probable MFS transporter [Chromobacterium violaceum ATCC 12472]
>gi|34495966|ref|NP_900181.1| probable MFS permease [Chromobacterium violaceum ATCC 12472]
>gi|34495971|ref|NP_900186.1| probable calcium binding hemolysin [Chromobacterium violaceum ATCC 12472]
>gi|34495977|ref|NP_900192.1| hypothetical protein CV0522 [Chromobacterium violaceum ATCC 12472]
>gi|34495979|ref|NP_900194.1| probable zinc metalloprotease [Chromobacterium violaceum ATCC 12472]
>gi|34495982|ref|NP_900197.1| dihydrolipoamide S-acetyltransferase [Chromobacterium violaceum ATCC 12472]
>gi|34495994|ref|NP_900209.1| probable transcriptional regulator, MarR-family [Chromobacterium violaceum ATCC 12472]
>gi|34495996|ref|NP_900211.1| hypothetical protein CV0541 [Chromobacterium violaceum ATCC 12472]
>gi|34496013|ref|NP_900228.1| probable phosphoenolpyruvate-protein phosphotransferase [Chromobacterium violaceum ATCC 12472]
>gi|34496014|ref|NP_900229.1| protein-N p-phosphohistidine-sugar phosphotransferase [Chromobacterium violaceum ATCC 12472]
>gi|34496049|ref|NP_900264.1| probable two-component sensor histidine kinase protein [Chromobacterium violaceum ATCC 12472]
>gi|34496061|ref|NP_900276.1| cytochrome aa3 oxidase assembly protein [Chromobacterium violaceum ATCC 12472]
>gi|34496062|ref|NP_900277.1| protoheme IX farnesyltransferase [Chromobacterium violaceum ATCC 12472]
>gi|34496079|ref|NP_900294.1| Sec-independent protein translocase protein TatB [Chromobacterium violaceum ATCC 12472]
>gi|34496081|ref|NP_900296.1| hypothetical protein CV0626 [Chromobacterium violaceum ATCC 12472]
>gi|34496087|ref|NP_900302.1| probable transmembrane protein [Chromobacterium violaceum ATCC 12472]
>gi|34496095|ref|NP_900310.1| hypothetical protein CV0640 [Chromobacterium violaceum ATCC 12472]
>gi|34496108|ref|NP_900323.1| hypothetical protein CV0653 [Chromobacterium violaceum ATCC 12472]
>gi|34496113|ref|NP_900328.1| hypothetical protein CV0658 [Chromobacterium violaceum ATCC 12472]
>gi|34496120|ref|NP_900335.1| hypothetical protein CV0665 [Chromobacterium violaceum ATCC 12472]
>gi|34496123|ref|NP_900338.1| ATP synthase F0, B subunit [Chromobacterium violaceum ATCC 12472]

>gi|34496138|ref|NP_900353.1| hypothetical protein CV0683 [Chromobacterium violaceum ATCC 12472]
>gi|34496139|ref|NP_900354.1| hypothetical protein CV0684 [Chromobacterium violaceum ATCC 12472]
>gi|34496147|ref|NP_900362.1| probable transport portein [Chromobacterium violaceum ATCC 12472]
>gi|34496148|ref|NP_900363.1| transcription regulator protein [Chromobacterium violaceum ATCC 12472]
>gi|34496152|ref|NP_900367.1| adenylosuccinate synthetase [Chromobacterium violaceum ATCC 12472]
>gi|34496155|ref|NP_900370.1| probable multidrug/chloramphenicol efflux transporter [Chromobacterium violaceum ATCC 12472]
>gi|34496158|ref|NP_900373.1| probable branched-chain amino acid permease, AzlC family protein [Chromobacterium violaceum ATCC 12472]
>gi|34496164|ref|NP_900379.1| probable multidrug resistance transmembrane protein [Chromobacterium violaceum ATCC 12472]
>gi|34496165|ref|NP_900380.1| probable outer membrane multidrug resistance lipoprotein [Chromobacterium violaceum ATCC 12472]
>gi|34496174|ref|NP_900389.1| methylenomycin A resistance protein [Chromobacterium violaceum ATCC 12472]
>gi|34496180|ref|NP_900395.1| hypothetical protein CV0725 [Chromobacterium violaceum ATCC 12472]
>gi|34496183|ref|NP_900398.1| hypothetical protein CV0728 [Chromobacterium violaceum ATCC 12472]
>gi|34496187|ref|NP_900402.1| hypothetical protein CV0732 [Chromobacterium violaceum ATCC 12472]
>gi|34496207|ref|NP_900422.1| hypothetical protein CV0752 [Chromobacterium violaceum ATCC 12472]
>gi|34496208|ref|NP_900423.1| hypothetical protein CV0753 [Chromobacterium violaceum ATCC 12472]
>gi|34496230|ref|NP_900445.1| hypothetical protein CV0775 [Chromobacterium violaceum ATCC 12472]
>gi|34496232|ref|NP_900447.1| hypothetical protein CV0777 [Chromobacterium violaceum ATCC 12472]
>gi|34496236|ref|NP_900451.1| hypothetical protein CV0781 [Chromobacterium violaceum ATCC 12472]
>gi|34496238|ref|NP_900453.1| MFS family, muropeptide transporter [Chromobacterium violaceum ATCC 12472]
>gi|34496268|ref|NP_900483.1| uroporphyrin-III C-methyltransferase [Chromobacterium violaceum ATCC 12472]
>gi|34496292|ref|NP_900507.1| hypothetical protein CV0837 [Chromobacterium violaceum ATCC 12472]
>gi|34496306|ref|NP_900521.1| hypothetical protein CV0851 [Chromobacterium violaceum ATCC 12472]
>gi|34496312|ref|NP_900527.1| hypothetical protein CV0857 [Chromobacterium violaceum ATCC 12472]
>gi|34496314|ref|NP_900529.1| hypothetical protein CV0859 [Chromobacterium violaceum ATCC 12472]
>gi|34496321|ref|NP_900536.1| probable transcriptional regulator, MarR family [Chromobacterium violaceum ATCC 12472]
>gi|34496328|ref|NP_900543.1| probable methyl-accepting chemotaxis protein [Chromobacterium violaceum ATCC 12472]
>gi|34496330|ref|NP_900545.1| probable carbonic anhydrase, family 3 [Chromobacterium violaceum ATCC 12472]
>gi|34496337|ref|NP_900552.1| probable sensor/response regulator hybrid protein [Chromobacterium violaceum ATCC 12472]
>gi|34496340|ref|NP_900555.1| probable alginate regulatory protein [Chromobacterium violaceum ATCC 12472]
>gi|34496345|ref|NP_900560.1| hypothetical protein CV0890 [Chromobacterium violaceum ATCC 12472]
>gi|34496346|ref|NP_900561.1| Transcriptional regulatory protein qseB [Chromobacterium violaceum ATCC 12472]
>gi|34496351|ref|NP_900566.1| probable two-component sensor [Chromobacterium violaceum ATCC 12472]
>gi|34496355|ref|NP_900570.1| hypothetical protein CV0900 [Chromobacterium violaceum ATCC 12472]
>gi|34496361|ref|NP_900576.1| phenylacetic acid permease [Chromobacterium violaceum ATCC 12472]
>gi|34496362|ref|NP_900577.1| hypothetical protein CV0907 [Chromobacterium violaceum ATCC 12472]
>gi|34496369|ref|NP_900584.1| probable transmembrane protein [Chromobacterium violaceum ATCC 12472]
>gi|34496410|ref|NP_900625.1| hypothetical protein CV0955 [Chromobacterium violaceum ATCC 12472]
>gi|34496430|ref|NP_900645.1| hypothetical protein CV0975 [Chromobacterium violaceum ATCC 12472]
>gi|34496436|ref|NP_900651.1| hypothetical protein CV0981 [Chromobacterium violaceum ATCC 12472]
>gi|34496438|ref|NP_900653.1| probable transmembrane protein [Chromobacterium violaceum ATCC 12472]
>gi|34496441|ref|NP_900656.1| acetyl-CoA carboxylase biotin carboxyl carrier protein [Chromobacterium violaceum ATCC 12472]
>gi|34496445|ref|NP_900660.1| hypothetical protein CV0990 [Chromobacterium violaceum ATCC 12472]
>gi|34496448|ref|NP_900663.1| 4-hydroxybenzoate transporter [Chromobacterium violaceum ATCC 12472]
>gi|34496455|ref|NP_900670.1| negative regulator of flagellin synthesis [Chromobacterium violaceum ATCC 12472]
>gi|34496461|ref|NP_900676.1| hypothetical protein CV1006 [Chromobacterium violaceum ATCC 12472]
>gi|34496468|ref|NP_900683.1| probable methyl-accepting chemotaxis protein II [Chromobacterium violaceum ATCC 12472]
>gi|34496469|ref|NP_900684.1| chemotaxis protein CheA [Chromobacterium violaceum ATCC 12472]
>gi|34496483|ref|NP_900698.1| dihydrofolate reductase [Chromobacterium violaceum ATCC 12472]
>gi|34496497|ref|NP_900712.1| hypothetical protein CV1042 [Chromobacterium violaceum ATCC 12472]
>gi|34496498|ref|NP_900713.1| probable transmembrane protein [Chromobacterium violaceum ATCC 12472]
>gi|34496504|ref|NP_900719.1| hypothetical protein CV1049 [Chromobacterium violaceum ATCC 12472]
>gi|34496527|ref|NP_900742.1| dihydrolipoamide succinyltransferase E2 component [Chromobacterium violaceum ATCC 12472]
>gi|34496533|ref|NP_900748.1| multidrug resistence transmembrane protein [Chromobacterium violaceum ATCC 12472]
>gi|34496534|ref|NP_900749.1| probable transcription regulator protein, LysR family [Chromobacterium violaceum ATCC 12472]
>gi|34496545|ref|NP_900760.1| hypothetical protein CV1090 [Chromobacterium violaceum ATCC 12472]
>gi|34496546|ref|NP_900761.1| chaperone protein hscB [Chromobacterium violaceum ATCC 12472]
>gi|34496560|ref|NP_900775.1| sodium/glutamate symport carrier protein [Chromobacterium violaceum ATCC 12472]
>gi|34496572|ref|NP_900787.1| probable chromate transporter protein [Chromobacterium violaceum ATCC 12472]
>gi|34496573|ref|NP_900788.1| probable transmembrane protein [Chromobacterium violaceum ATCC 12472]
>gi|34496580|ref|NP_900795.1| serine-type D-Ala-D-Ala carboxypeptidase [Chromobacterium violaceum ATCC 12472]
>gi|34496586|ref|NP_900801.1| hypothetical protein CV1131 [Chromobacterium violaceum ATCC 12472]
>gi|34496591|ref|NP_900806.1| hypothetical protein CV1136 [Chromobacterium violaceum ATCC 12472]
>gi|34496596|ref|NP_900811.1| hypothetical protein CV1141 [Chromobacterium violaceum ATCC 12472]
>gi|34496602|ref|NP_900817.1| hypothetical protein CV1147 [Chromobacterium violaceum ATCC 12472]
>gi|34496606|ref|NP_900821.1| probable murein hydrolase exporter [Chromobacterium violaceum ATCC 12472]
>gi|34496609|ref|NP_900824.1| lead,cadmium,zinc and mercury transporting ATPase [Chromobacterium violaceum ATCC 12472]
>gi|34496623|ref|NP_900838.1| probable MFS transporter [Chromobacterium violaceum ATCC 12472]
>gi|34496634|ref|NP_900849.1| probable multidrug resistance protein [Chromobacterium violaceum ATCC 12472]
>gi|34496639|ref|NP_900854.1| ethanolamine ammonia-lyase light chain [Chromobacterium violaceum ATCC 12472]
>gi|34496646|ref|NP_900861.1| urease accessory protein [Chromobacterium violaceum ATCC 12472]

>gi|34496654|ref|NP_900869.1| hypothetical protein CV1199 [Chromobacterium violaceum ATCC 12472]
>gi|34496663|ref|NP_900878.1| hypothetical protein CV1208 [Chromobacterium violaceum ATCC 12472]
>gi|34496686|ref|NP_900901.1| hypothetical protein CV1231 [Chromobacterium violaceum ATCC 12472]
>gi|34496687|ref|NP_900902.1| hypothetical protein CV1232 [Chromobacterium violaceum ATCC 12472]
>gi|34496708|ref|NP_900923.1| peptidoglycan N-acetylmuramoylhydrolase [Chromobacterium violaceum ATCC 12472]
>gi|34496714|ref|NP_900929.1| 2-C-methyl-d-erythritol-2,4-cyclodiphosphate synthase [Chromobacterium violaceum ATCC 12472]
>gi|34496723|ref|NP_900938.1| hypothetical protein CV1268 [Chromobacterium violaceum ATCC 12472]
>gi|34496726|ref|NP_900941.1| probable chromate ion transporter [Chromobacterium violaceum ATCC 12472]
>gi|34496728|ref|NP_900943.1| glutamine transport system permease protein [Chromobacterium violaceum ATCC 12472]
>gi|34496729|ref|NP_900944.1| probable permease of ABC transporter [Chromobacterium violaceum ATCC 12472]
>gi|34496734|ref|NP_900949.1| probable multidrug transmembrane resistance signal peptide protein [Chromobacterium violaceum ATCC 12472]
>gi|34496737|ref|NP_900952.1| probable transcriptional regulator [Chromobacterium violaceum ATCC 12472]
>gi|34496759|ref|NP_900974.1| hypothetical protein CV1304 [Chromobacterium violaceum ATCC 12472]
>gi|34496767|ref|NP_900982.1| hypothetical protein CV1312 [Chromobacterium violaceum ATCC 12472]
>gi|34496772|ref|NP_900987.1| hypothetical protein CV1317 [Chromobacterium violaceum ATCC 12472]
>gi|34496776|ref|NP_900991.1| hypothetical protein CV1321 [Chromobacterium violaceum ATCC 12472]
>gi|34496778|ref|NP_900993.1| probable hydrolase/nitrilase [Chromobacterium violaceum ATCC 12472]
>gi|34496805|ref|NP_901020.1| 50S ribosomal protein L35 [Chromobacterium violaceum ATCC 12472]
>gi|34496821|ref|NP_901036.1| probable phasin [Chromobacterium violaceum ATCC 12472]
>gi|34496840|ref|NP_901055.1| probable transmembrane protein [Chromobacterium violaceum ATCC 12472]
>gi|34496844|ref|NP_901059.1| hypothetical protein CV1389 [Chromobacterium violaceum ATCC 12472]
>gi|34496848|ref|NP_901063.1| probable homoserine/homoserine lactone efflux protein [Chromobacterium violaceum ATCC 12472]
>gi|34496853|ref|NP_901068.1| hypothetical protein CV1398 [Chromobacterium violaceum ATCC 12472]
>gi|34496874|ref|NP_901089.1| probable transmembrane sensor histidine kinase transcription regulator protein [Chromobacterium violaceum ATCC 12472]
>gi|34496889|ref|NP_901104.1| hypothetical protein CV1434 [Chromobacterium violaceum ATCC 12472]
>gi|34496898|ref|NP_901113.1| hypothetical protein CV1443 [Chromobacterium violaceum ATCC 12472]
>gi|34496908|ref|NP_901123.1| hypothetical protein CV1453 [Chromobacterium violaceum ATCC 12472]
>gi|34496917|ref|NP_901132.1| translation initiation factor IF-2 [Chromobacterium violaceum ATCC 12472]
>gi|34496926|ref|NP_901141.1| probable adhesin [Chromobacterium violaceum ATCC 12472]
>gi|34496941|ref|NP_901156.1| enterobactin synthetase component F [Chromobacterium violaceum ATCC 12472]
>gi|34496943|ref|NP_901158.1| ferric citrate transport system permease protein [Chromobacterium violaceum ATCC 12472]
>gi|34496965|ref|NP_901180.1| probable multidrug resistance protein [Chromobacterium violaceum ATCC 12472]
>gi|34496974|ref|NP_901189.1| hypothetical protein CV1519 [Chromobacterium violaceum ATCC 12472]
>gi|34496976|ref|NP_901191.1| probable two-component sensor [Chromobacterium violaceum ATCC 12472]
>gi|34496977|ref|NP_901192.1| hypothetical protein CV1522 [Chromobacterium violaceum ATCC 12472]
>gi|34496979|ref|NP_901194.1| probable methyl-accepting chemotaxis protein II [Chromobacterium violaceum ATCC 12472]
>gi|34496984|ref|NP_901199.1| probable transmembrane protein [Chromobacterium violaceum ATCC 12472]
>gi|34496991|ref|NP_901206.1| hypothetical protein CV1536 [Chromobacterium violaceum ATCC 12472]
>gi|34497003|ref|NP_901218.1| hypothetical protein CV1548 [Chromobacterium violaceum ATCC 12472]
>gi|34497009|ref|NP_901224.1| hypothetical protein CV1554 [Chromobacterium violaceum ATCC 12472]
>gi|34497010|ref|NP_901225.1| probable oxidoreductase protein [Chromobacterium violaceum ATCC 12472]
>gi|34497011|ref|NP_901226.1| cobyrinic acid A,C-diamide synthase [Chromobacterium violaceum ATCC 12472]
>gi|34497013|ref|NP_901228.1| vitamin B12 transport system permease protein [Chromobacterium violaceum ATCC 12472]
>gi|34497030|ref|NP_901245.1| cobalamin biosynthetic protein [Chromobacterium violaceum ATCC 12472]
>gi|34497032|ref|NP_901247.1| inositol monophosphatase family protein [Chromobacterium violaceum ATCC 12472]
>gi|34497044|ref|NP_901259.1| probable integral membrane protein [Chromobacterium violaceum ATCC 12472]
>gi|34497045|ref|NP_901260.1| probable transmembrane protein [Chromobacterium violaceum ATCC 12472]
>gi|34497056|ref|NP_901271.1| probable transmembrane protein [Chromobacterium violaceum ATCC 12472]
>gi|34497065|ref|NP_901280.1| DNA polymerase subunits gamma and tau [Chromobacterium violaceum ATCC 12472]
>gi|34497078|ref|NP_901293.1| hypothetical protein CV1623 [Chromobacterium violaceum ATCC 12472]
>gi|34497107|ref|NP_901322.1| probable methyl-accepting chemotaxis protein [Chromobacterium violaceum ATCC 12472]
>gi|34497108|ref|NP_901323.1| protoporphyrinogen oxidase [Chromobacterium violaceum ATCC 12472]
>gi|34497114|ref|NP_901329.1| hypothetical protein CV1659 [Chromobacterium violaceum ATCC 12472]
>gi|34497124|ref|NP_901339.1| hypothetical protein CV1669 [Chromobacterium violaceum ATCC 12472]
>gi|34497127|ref|NP_901342.1| hypothetical protein CV1672 [Chromobacterium violaceum ATCC 12472]
>gi|34497131|ref|NP_901346.1| hypothetical protein CV1676 [Chromobacterium violaceum ATCC 12472]
>gi|34497135|ref|NP_901350.1| probable permease of ABC transporter [Chromobacterium violaceum ATCC 12472]
>gi|34497141|ref|NP_901356.1| hypothetical protein CV1686 [Chromobacterium violaceum ATCC 12472]
>gi|34497142|ref|NP_901357.1| L-asparaginase I [Chromobacterium violaceum ATCC 12472]
>gi|34497151|ref|NP_901366.1| probable integral membrane protein [Chromobacterium violaceum ATCC 12472]
>gi|34497155|ref|NP_901370.1| probable flagella basal body P-ring formation protein flgA [Chromobacterium violaceum ATCC 12472]
>gi|34497158|ref|NP_901373.1| basal-body rod modification protein FlgD [Chromobacterium violaceum ATCC 12472]
>gi|34497170|ref|NP_901385.1| hypothetical protein CV1715 [Chromobacterium violaceum ATCC 12472]
>gi|34497177|ref|NP_901392.1| probable sulfate permease family protein [Chromobacterium violaceum ATCC 12472]
>gi|34497178|ref|NP_901393.1| hypothetical protein CV1723 [Chromobacterium violaceum ATCC 12472]
>gi|34497180|ref|NP_901395.1| hypothetical protein CV1725 [Chromobacterium violaceum ATCC 12472]
>gi|34497181|ref|NP_901396.1| probable integral membrane protein [Chromobacterium violaceum ATCC 12472]
>gi|34497184|ref|NP_901399.1| hypothetical protein CV1729 [Chromobacterium violaceum ATCC 12472]
>gi|34497203|ref|NP_901418.1| hypothetical protein CV1748 [Chromobacterium violaceum ATCC 12472]

>gi|34497204|ref|NP_901419.1| hypothetical protein CV1749 [Chromobacterium violaceum ATCC 12472]
>gi|34497205|ref|NP_901420.1| hypothetical protein CV1750 [Chromobacterium violaceum ATCC 12472]
>gi|34497216|ref|NP_901431.1| hypothetical protein CV1761 [Chromobacterium violaceum ATCC 12472]
>gi|34497220|ref|NP_901435.1| hypothetical protein CV1765 [Chromobacterium violaceum ATCC 12472]
>gi|34497227|ref|NP_901442.1| hypothetical protein CV1772 [Chromobacterium violaceum ATCC 12472]
>gi|34497232|ref|NP_901447.1| probable regulator protein pecM [Chromobacterium violaceum ATCC 12472]
>gi|34497234|ref|NP_901449.1| probable transcriptional regulator, MerR family [Chromobacterium violaceum ATCC 12472]
>gi|34497236|ref|NP_901451.1| hypothetical protein CV1781 [Chromobacterium violaceum ATCC 12472]
>gi|34497237|ref|NP_901452.1| hypothetical protein CV1782 [Chromobacterium violaceum ATCC 12472]
>gi|34497249|ref|NP_901464.1| hemin transport system permease protein [Chromobacterium violaceum ATCC 12472]
>gi|34497263|ref|NP_901478.1| hypothetical protein CV1808 [Chromobacterium violaceum ATCC 12472]
>gi|34497266|ref|NP_901481.1| PAL cross-reacting lipoprotein precursor [Chromobacterium violaceum ATCC 12472]
>gi|34497275|ref|NP_901490.1| probable ribonuclease E [Chromobacterium violaceum ATCC 12472]
>gi|34497278|ref|NP_901493.1| probable transmembrane transporter protein [Chromobacterium violaceum ATCC 12472]
>gi|34497283|ref|NP_901498.1| sulfate transport ATP-binding ABC transporter protein [Chromobacterium violaceum ATCC 12472]
>gi|34497285|ref|NP_901500.1| sulfate transport system permease protein CysU [Chromobacterium violaceum ATCC 12472]
>gi|34497290|ref|NP_901505.1| hypothetical protein CV1835 [Chromobacterium violaceum ATCC 12472]
>gi|34497292|ref|NP_901507.1| probable transcriptional regulator, TetR family [Chromobacterium violaceum ATCC 12472]
>gi|34497300|ref|NP_901515.1| phosphonate metabolism protein PhnH [Chromobacterium violaceum ATCC 12472]
>gi|34497308|ref|NP_901523.1| hypothetical protein CV1853 [Chromobacterium violaceum ATCC 12472]
>gi|34497312|ref|NP_901527.1| probable ABC transporter permease protein [Chromobacterium violaceum ATCC 12472]
>gi|34497314|ref|NP_901529.1| hypothetical protein CV1859 [Chromobacterium violaceum ATCC 12472]
>gi|34497316|ref|NP_901531.1| probable transport protein [Chromobacterium violaceum ATCC 12472]
>gi|34497344|ref|NP_901559.1| single-strand DNA-binding protein [Chromobacterium violaceum ATCC 12472]
>gi|34497345|ref|NP_901560.1| hypothetical protein CV1890 [Chromobacterium violaceum ATCC 12472]
>gi|34497346|ref|NP_901561.1| probable outer membrane protein [Chromobacterium violaceum ATCC 12472]
>gi|34497359|ref|NP_901574.1| probable transmembrane protein, DMT Superfamily [Chromobacterium violaceum ATCC 12472]
>gi|34497360|ref|NP_901575.1| probable transcription regulator [Chromobacterium violaceum ATCC 12472]
>gi|34497373|ref|NP_901588.1| probable hemolysin [Chromobacterium violaceum ATCC 12472]
>gi|34497391|ref|NP_901606.1| hypothetical protein CV1936 [Chromobacterium violaceum ATCC 12472]
>gi|34497402|ref|NP_901617.1| two-component response regulator [Chromobacterium violaceum ATCC 12472]
>gi|34497404|ref|NP_901619.1| hypothetical protein CV1949 [Chromobacterium violaceum ATCC 12472]
>gi|34497405|ref|NP_901620.1| hypothetical protein CV1950 [Chromobacterium violaceum ATCC 12472]
>gi|34497408|ref|NP_901623.1| hypothetical protein CV1953 [Chromobacterium violaceum ATCC 12472]
>gi|34497421|ref|NP_901636.1| hypothetical protein CV1966 [Chromobacterium violaceum ATCC 12472]
>gi|34497426|ref|NP_901641.1| hypothetical protein CV1971 [Chromobacterium violaceum ATCC 12472]
>gi|34497438|ref|NP_901653.1| hypothetical protein CV1983 [Chromobacterium violaceum ATCC 12472]
>gi|34497450|ref|NP_901665.1| probable membrane protein [Chromobacterium violaceum ATCC 12472]
>gi|34497451|ref|NP_901666.1| hypothetical protein CV1996 [Chromobacterium violaceum ATCC 12472]
>gi|34497453|ref|NP_901668.1| hypothetical protein CV1998 [Chromobacterium violaceum ATCC 12472]
>gi|34497470|ref|NP_901685.1| probable transcriptional regulator [Chromobacterium violaceum ATCC 12472]
>gi|34497483|ref|NP_901698.1| hypothetical protein CV2028 [Chromobacterium violaceum ATCC 12472]
>gi|34497501|ref|NP_901716.1| hypothetical protein CV2046 [Chromobacterium violaceum ATCC 12472]
>gi|34497526|ref|NP_901741.1| hypothetical protein CV2071 [Chromobacterium violaceum ATCC 12472]
>gi|34497527|ref|NP_901742.1| holo-[acyl-carrier-protein] synthase [Chromobacterium violaceum ATCC 12472]
>gi|34497529|ref|NP_901744.1| hypothetical protein CV2074 [Chromobacterium violaceum ATCC 12472]
>gi|34497536|ref|NP_901751.1| 3-hydroxyisobutyrate dehydrogenase [Chromobacterium violaceum ATCC 12472]
>gi|34497545|ref|NP_901760.1| hypothetical protein CV2090 [Chromobacterium violaceum ATCC 12472]
>gi|34497562|ref|NP_901777.1| hypothetical protein CV2107 [Chromobacterium violaceum ATCC 12472]
>gi|34497575|ref|NP_901790.1| hypothetical protein CV2120 [Chromobacterium violaceum ATCC 12472]
>gi|34497577|ref|NP_901792.1| hypothetical protein CV2122 [Chromobacterium violaceum ATCC 12472]
>gi|34497587|ref|NP_901802.1| hypothetical protein CV2132 [Chromobacterium violaceum ATCC 12472]
>gi|34497599|ref|NP_901814.1| hypothetical protein CV2144 [Chromobacterium violaceum ATCC 12472]
>gi|34497600|ref|NP_901815.1| hypothetical protein CV2145 [Chromobacterium violaceum ATCC 12472]
>gi|34497618|ref|NP_901833.1| DNA-binding protein hu-beta [Chromobacterium violaceum ATCC 12472]
>gi|34497633|ref|NP_901848.1| probable transmembrane efflux protein [Chromobacterium violaceum ATCC 12472]
>gi|34497642|ref|NP_901857.1| hypothetical protein CV2187 [Chromobacterium violaceum ATCC 12472]
>gi|34497645|ref|NP_901860.1| hypothetical protein CV2190 [Chromobacterium violaceum ATCC 12472]
>gi|34497650|ref|NP_901865.1| hypothetical protein CV2195 [Chromobacterium violaceum ATCC 12472]
>gi|34497661|ref|NP_901876.1| UDP-3-O-[3-hydroxymyristoyl] glucosamine N-acyltransferase (firA protein) [Chromobacterium violaceum ATCC 12472]
>gi|34497666|ref|NP_901881.1| hypothetical protein CV2211 [Chromobacterium violaceum ATCC 12472]
>gi|34497677|ref|NP_901892.1| hypothetical protein CV2222 [Chromobacterium violaceum ATCC 12472]
>gi|34497682|ref|NP_901897.1| nitrate transporter protein [Chromobacterium violaceum ATCC 12472]
>gi|34497683|ref|NP_901898.1| nitrate/nitrite transporter [Chromobacterium violaceum ATCC 12472]
>gi|34497690|ref|NP_901905.1| enterobactin-iron transport system permease protein [Chromobacterium violaceum ATCC 12472]
>gi|34497691|ref|NP_901906.1| enterobactin-iron transport system permease protein [Chromobacterium violaceum ATCC 12472]
>gi|34497692|ref|NP_901907.1| probable POT family transport protein [Chromobacterium violaceum ATCC 12472]
>gi|34497697|ref|NP_901912.1| outer membrane drug efflux lipoprotein [Chromobacterium violaceum ATCC 12472]
>gi|34497710|ref|NP_901925.1| maltose O-acetyltransferase [Chromobacterium violaceum ATCC 12472]

>gi|34497717|ref|NP_901932.1| hypothetical protein CV2262 [Chromobacterium violaceum ATCC 12472]
>gi|34497731|ref|NP_901946.1| hypothetical protein CV2276 [Chromobacterium violaceum ATCC 12472]
>gi|34497746|ref|NP_901961.1| probable ABC transporter permease protein [Chromobacterium violaceum ATCC 12472]
>gi|34497760|ref|NP_901975.1| probable flagellar protein [Chromobacterium violaceum ATCC 12472]
>gi|34497791|ref|NP_902006.1| probable ThiJ/PfpI family protein [Chromobacterium violaceum ATCC 12472]
>gi|34497798|ref|NP_902013.1| hypothetical protein CV2343 [Chromobacterium violaceum ATCC 12472]
>gi|34497807|ref|NP_902022.1| hypothetical protein CV2352 [Chromobacterium violaceum ATCC 12472]
>gi|34497808|ref|NP_902023.1| Thiol:disulfide interchange protein DsbD [Chromobacterium violaceum ATCC 12472]
>gi|34497812|ref|NP_902027.1| hypothetical protein CV2357 [Chromobacterium violaceum ATCC 12472]
>gi|34497813|ref|NP_902028.1| probable phospho-2-dehydro-3-deoxyheptonate aldolase [Chromobacterium violaceum ATCC 12472]
>gi|34497822|ref|NP_902037.1| probable transmembrane protease [Chromobacterium violaceum ATCC 12472]
>gi|34497847|ref|NP_902062.1| thiamine-phosphate kinase [Chromobacterium violaceum ATCC 12472]
>gi|34497849|ref|NP_902064.1| probable cell division ftsk transmembrane protein [Chromobacterium violaceum ATCC 12472]
>gi|34497857|ref|NP_902072.1| hypothetical protein CV2402 [Chromobacterium violaceum ATCC 12472]
>gi|34497859|ref|NP_902074.1| probable KrfA protein [Chromobacterium violaceum ATCC 12472]
>gi|34497869|ref|NP_902084.1| hypothetical protein CV2414 [Chromobacterium violaceum ATCC 12472]
>gi|34497872|ref|NP_902087.1| hypothetical protein CV2417 [Chromobacterium violaceum ATCC 12472]
>gi|34497873|ref|NP_902088.1| probable oxygen-regulated invasion protein - cell invasion protein [Chromobacterium violaceum ATCC 12472]
>gi|34497885|ref|NP_902100.1| hypothetical protein CV2430 [Chromobacterium violaceum ATCC 12472]
>gi|34497898|ref|NP_902113.1| hypothetical protein CV2443 [Chromobacterium violaceum ATCC 12472]
>gi|34497900|ref|NP_902115.1| hypothetical protein CV2445 [Chromobacterium violaceum ATCC 12472]
>gi|34497902|ref|NP_902117.1| probable dehydrogenase [Chromobacterium violaceum ATCC 12472]
>gi|34497905|ref|NP_902120.1| hypothetical protein CV2450 [Chromobacterium violaceum ATCC 12472]
>gi|34497906|ref|NP_902121.1| hypothetical protein CV2451 [Chromobacterium violaceum ATCC 12472]
>gi|34497907|ref|NP_902122.1| probable linoleoyl-CoA desaturase [Chromobacterium violaceum ATCC 12472]
>gi|34497910|ref|NP_902125.1| cation (Na+-coupled) multidrug resistance efflux pump [Chromobacterium violaceum ATCC 12472]
>gi|34497920|ref|NP_902135.1| hypothetical protein CV2465 [Chromobacterium violaceum ATCC 12472]
>gi|34497938|ref|NP_902153.1| hypothetical protein CV2483 [Chromobacterium violaceum ATCC 12472]
>gi|34497939|ref|NP_902154.1| hypothetical protein CV2484 [Chromobacterium violaceum ATCC 12472]
>gi|34497945|ref|NP_902160.1| hypothetical protein CV2490 [Chromobacterium violaceum ATCC 12472]
>gi|34497950|ref|NP_902165.1| flagellin [Chromobacterium violaceum ATCC 12472]
>gi|34497971|ref|NP_902186.1| probable colicin V production protein [Chromobacterium violaceum ATCC 12472]
>gi|34497972|ref|NP_902187.1| lipoprotein [Chromobacterium violaceum ATCC 12472]
>gi|34497982|ref|NP_902197.1| transmembrane regulator PrtR [Chromobacterium violaceum ATCC 12472]
>gi|34497985|ref|NP_902200.1| hypothetical protein CV2530 [Chromobacterium violaceum ATCC 12472]
>gi|34497986|ref|NP_902201.1| probable transmembrane protein [Chromobacterium violaceum ATCC 12472]
>gi|34498006|ref|NP_902221.1| probable transmembrane protein [Chromobacterium violaceum ATCC 12472]
>gi|34498018|ref|NP_902233.1| hypothetical protein CV2563 [Chromobacterium violaceum ATCC 12472]
>gi|34498021|ref|NP_902236.1| probable peroxide-inducible genes activator [Chromobacterium violaceum ATCC 12472]
>gi|34498027|ref|NP_902242.1| purine cytosine permease-like protein [Chromobacterium violaceum ATCC 12472]
>gi|34498031|ref|NP_902246.1| hypothetical protein CV2576 [Chromobacterium violaceum ATCC 12472]
>gi|34498046|ref|NP_902261.1| probable cytochrome b561 [Chromobacterium violaceum ATCC 12472]
>gi|34498063|ref|NP_902278.1| type III secretion apparatus protein EscS [Chromobacterium violaceum ATCC 12472]
>gi|34498072|ref|NP_902287.1| pathogenicity island 1 effector protein [Chromobacterium violaceum ATCC 12472]
>gi|34498074|ref|NP_902289.1| cell invasion protein [Chromobacterium violaceum ATCC 12472]
>gi|34498077|ref|NP_902292.1| surface presentation of antigens; secretory proteins [Chromobacterium violaceum ATCC 12472]
>gi|34498081|ref|NP_902296.1| surface presentation of antigens; secretory proteins [Chromobacterium violaceum ATCC 12472]
>gi|34498082|ref|NP_902297.1| secretory protein, associated with virulence [Chromobacterium violaceum ATCC 12472]
>gi|34498089|ref|NP_902304.1| two-component sensor kinase [Chromobacterium violaceum ATCC 12472]
>gi|34498092|ref|NP_902307.1| thiol:disulfide interchange protein DsbG [Chromobacterium violaceum ATCC 12472]
>gi|34498093|ref|NP_902308.1| hypothetical protein CV2638 [Chromobacterium violaceum ATCC 12472]
>gi|34498094|ref|NP_902309.1| hypothetical protein CV2639 [Chromobacterium violaceum ATCC 12472]
>gi|34498098|ref|NP_902313.1| probable sensor/response regulator hybrid [Chromobacterium violaceum ATCC 12472]
>gi|34498109|ref|NP_902324.1| probable transcriptional regulator [Chromobacterium violaceum ATCC 12472]
>gi|34498116|ref|NP_902331.1| hypothetical protein CV2661 [Chromobacterium violaceum ATCC 12472]
>gi|34498117|ref|NP_902332.1| calcium/proton antiporter [Chromobacterium violaceum ATCC 12472]
>gi|34498129|ref|NP_902344.1| hypothetical protein CV2674 [Chromobacterium violaceum ATCC 12472]
>gi|34498130|ref|NP_902345.1| cellulose synthase, subunit C [Chromobacterium violaceum ATCC 12472]
>gi|34498136|ref|NP_902351.1| hypothetical protein CV2681 [Chromobacterium violaceum ATCC 12472]
>gi|34498154|ref|NP_902369.1| probable transcriptional regulator, TetR family [Chromobacterium violaceum ATCC 12472]
>gi|34498158|ref|NP_902373.1| probable outer membrane protein [Chromobacterium violaceum ATCC 12472]
>gi|34498160|ref|NP_902375.1| probable membrane protein [Chromobacterium violaceum ATCC 12472]
>gi|34498162|ref|NP_902377.1| probable short chain dehydrogenase [Chromobacterium violaceum ATCC 12472]
>gi|34498164|ref|NP_902379.1| hypothetical protein CV2709 [Chromobacterium violaceum ATCC 12472]
>gi|34498165|ref|NP_902380.1| hypothetical protein CV2710 [Chromobacterium violaceum ATCC 12472]
>gi|34498166|ref|NP_902381.1| hypothetical protein CV2711 [Chromobacterium violaceum ATCC 12472]
>gi|34498168|ref|NP_902383.1| lipase modulator protein [Chromobacterium violaceum ATCC 12472]
>gi|34498190|ref|NP_902405.1| hypothetical protein CV2735 [Chromobacterium violaceum ATCC 12472]
>gi|34498203|ref|NP_902418.1| probable cation transport P-type ATPase [Chromobacterium violaceum ATCC 12472]
>gi|34498220|ref|NP_902435.1| probable transmembrane protein [Chromobacterium violaceum ATCC 12472]

>gi|34498221|ref|NP_902436.1| probable transmembrane protein [Chromobacterium violaceum ATCC 12472]
>gi|34498222|ref|NP_902437.1| aspartate-semialdehyde dehydrogenase [Chromobacterium violaceum ATCC 12472]
>gi|34498224|ref|NP_902439.1| hypothetical protein CV2769 [Chromobacterium violaceum ATCC 12472]
>gi|34498234|ref|NP_902449.1| hypothetical protein CV2779 [Chromobacterium violaceum ATCC 12472]
>gi|34498253|ref|NP_902468.1| probable macrolide efflux protein [Chromobacterium violaceum ATCC 12472]
>gi|34498262|ref|NP_902477.1| hypothetical protein CV2807 [Chromobacterium violaceum ATCC 12472]
>gi|34498279|ref|NP_902494.1| probable ketoglutarate semialdehyde dehydrogenase [Chromobacterium violaceum ATCC 12472]
>gi|34498280|ref|NP_902495.1| probable dihydroxydipicolinate synthase [Chromobacterium violaceum ATCC 12472]
>gi|34498294|ref|NP_902509.1| probable transporter transmembrane protein [Chromobacterium violaceum ATCC 12472]
>gi|34498302|ref|NP_902517.1| probable fusaric acid resistance protein FusE II [Chromobacterium violaceum ATCC 12472]
>gi|34498303|ref|NP_902518.1| probable outer membrane component of multidrug efflux pump [Chromobacterium violaceum ATCC 12472]
>gi|34498311|ref|NP_902526.1| taurine transport system permease protein [Chromobacterium violaceum ATCC 12472]
>gi|34498321|ref|NP_902536.1| hypothetical protein CV2866 [Chromobacterium violaceum ATCC 12472]
>gi|34498341|ref|NP_902556.1| basal-body rod modification protein FlgD [Chromobacterium violaceum ATCC 12472]
>gi|34498350|ref|NP_902565.1| probable transcriptional regulator protein [Chromobacterium violaceum ATCC 12472]
>gi|34498355|ref|NP_902570.1| hypothetical protein CV2900 [Chromobacterium violaceum ATCC 12472]
>gi|34498358|ref|NP_902573.1| probable Na/H+ antiporter [Chromobacterium violaceum ATCC 12472]
>gi|34498363|ref|NP_902578.1| hypothetical protein CV2908 [Chromobacterium violaceum ATCC 12472]
>gi|34498366|ref|NP_902581.1| hypothetical protein CV2911 [Chromobacterium violaceum ATCC 12472]
>gi|34498367|ref|NP_902582.1| hypothetical protein CV2912 [Chromobacterium violaceum ATCC 12472]
>gi|34498402|ref|NP_902617.1| hypothetical protein CV2947 [Chromobacterium violaceum ATCC 12472]
>gi|34498421|ref|NP_902636.1| hypothetical protein CV2966 [Chromobacterium violaceum ATCC 12472]
>gi|34498423|ref|NP_902638.1| hypothetical protein CV2968 [Chromobacterium violaceum ATCC 12472]
>gi|34498426|ref|NP_902641.1| hypothetical protein CV2971 [Chromobacterium violaceum ATCC 12472]
>gi|34498437|ref|NP_902652.1| probable amino-acid transport system permease ABC transporter protein [Chromobacterium violaceum ATCC 12472]
>gi|34498440|ref|NP_902655.1| hypothetical protein CV2985 [Chromobacterium violaceum ATCC 12472]
>gi|34498441|ref|NP_902656.1| hypothetical protein CV2986 [Chromobacterium violaceum ATCC 12472]
>gi|34498445|ref|NP_902660.1| RNA polymerase sigma factor for flagellar operon [Chromobacterium violaceum ATCC 12472]
>gi|34498447|ref|NP_902662.1| hypothetical protein CV2992 [Chromobacterium violaceum ATCC 12472]
>gi|34498450|ref|NP_902665.1| hypothetical protein CV2995 [Chromobacterium violaceum ATCC 12472]
>gi|34498452|ref|NP_902667.1| flagellar assembly protein [Chromobacterium violaceum ATCC 12472]
>gi|34498458|ref|NP_902673.1| flagellar biosynthetic protein fliP [Chromobacterium violaceum ATCC 12472]
>gi|34498459|ref|NP_902674.1| flagellar biosynthetic protein FliQ [Chromobacterium violaceum ATCC 12472]
>gi|34498460|ref|NP_902675.1| flagellar biosynthetic protein FliR [Chromobacterium violaceum ATCC 12472]
>gi|34498461|ref|NP_902676.1| flagellar biosynthetic protein flhB [Chromobacterium violaceum ATCC 12472]
>gi|34498466|ref|NP_902681.1| flagellin D [Chromobacterium violaceum ATCC 12472]
>gi|34498479|ref|NP_902694.1| probable glutathione S-transferase [Chromobacterium violaceum ATCC 12472]
>gi|34498499|ref|NP_902714.1| hypothetical protein CV3044 [Chromobacterium violaceum ATCC 12472]
>gi|34498505|ref|NP_902720.1| cytochrome b561 [Chromobacterium violaceum ATCC 12472]
>gi|34498506|ref|NP_902721.1| probable transcriptional regulator lacI family [Chromobacterium violaceum ATCC 12472]
>gi|34498508|ref|NP_902723.1| 1-phosphofructokinase [Chromobacterium violaceum ATCC 12472]
>gi|34498509|ref|NP_902724.1| protein-N p-phosphohistidine-sugar phosphotransferase [Chromobacterium violaceum ATCC 12472]
>gi|34498513|ref|NP_902728.1| hypothetical protein CV3058 [Chromobacterium violaceum ATCC 12472]
>gi|34498517|ref|NP_902732.1| probable enoyl-CoA hydratase [Chromobacterium violaceum ATCC 12472]
>gi|34498518|ref|NP_902733.1| hypothetical protein CV3063 [Chromobacterium violaceum ATCC 12472]
>gi|34498520|ref|NP_902735.1| high-affinity zinc transport system permease protein [Chromobacterium violaceum ATCC 12472]
>gi|34498530|ref|NP_902745.1| hypothetical protein CV3075 [Chromobacterium violaceum ATCC 12472]
>gi|34498531|ref|NP_902746.1| hypothetical protein CV3076 [Chromobacterium violaceum ATCC 12472]
>gi|34498545|ref|NP_902760.1| hypothetical protein CV3090 [Chromobacterium violaceum ATCC 12472]
>gi|34498563|ref|NP_902778.1| TerC family protein [Chromobacterium violaceum ATCC 12472]
>gi|34498567|ref|NP_902782.1| type-4 fimbrial biogenesis PilV transmembrane protein [Chromobacterium violaceum ATCC 12472]
>gi|34498573|ref|NP_902788.1| hypothetical protein CV3118 [Chromobacterium violaceum ATCC 12472]
>gi|34498576|ref|NP_902791.1| 2-octaprenyl-6-methoxyphenol hydroxylase oxidoreductase protein [Chromobacterium violaceum ATCC 12472]
>gi|34498580|ref|NP_902795.1| flagellar biosynthetic protein FliQ [Chromobacterium violaceum ATCC 12472]
>gi|34498583|ref|NP_902798.1| probable Flagellar protein FliO [Chromobacterium violaceum ATCC 12472]
>gi|34498587|ref|NP_902802.1| hypothetical protein CV3132 [Chromobacterium violaceum ATCC 12472]
>gi|34498595|ref|NP_902810.1| probable protein-tyrosine-phosphatase [Chromobacterium violaceum ATCC 12472]
>gi|34498599|ref|NP_902814.1| probable transcriptional regulator LysR family [Chromobacterium violaceum ATCC 12472]
>gi|34498600|ref|NP_902815.1| hypothetical protein CV3145 [Chromobacterium violaceum ATCC 12472]
>gi|34498603|ref|NP_902818.1| hypothetical protein CV3148 [Chromobacterium violaceum ATCC 12472]
>gi|34498611|ref|NP_902826.1| hypothetical protein CV3156 [Chromobacterium violaceum ATCC 12472]
>gi|34498615|ref|NP_902830.1| hypothetical protein CV3160 [Chromobacterium violaceum ATCC 12472]
>gi|34498617|ref|NP_902832.1| probable thiamine transport system permease protein [Chromobacterium violaceum ATCC 12472]
>gi|34498626|ref|NP_902841.1| hypothetical protein CV3171 [Chromobacterium violaceum ATCC 12472]
>gi|34498629|ref|NP_902844.1| hypothetical protein CV3174 [Chromobacterium violaceum ATCC 12472]
>gi|34498630|ref|NP_902845.1| hypothetical protein CV3175 [Chromobacterium violaceum ATCC 12472]
>gi|34498632|ref|NP_902847.1| hypothetical protein CV3177 [Chromobacterium violaceum ATCC 12472]
>gi|34498650|ref|NP_902865.1| DNA-3-methyladenine glycosidase II [Chromobacterium violaceum ATCC 12472]
>gi|34498664|ref|NP_902879.1| osmotically inducible lipoprotein [Chromobacterium violaceum ATCC 12472]

>gi|34498669|ref|NP_902884.1| hypothetical protein CV3214 [Chromobacterium violaceum ATCC 12472]
>gi|34498670|ref|NP_902885.1| hypothetical protein CV3215 [Chromobacterium violaceum ATCC 12472]
>gi|34498676|ref|NP_902891.1| probable NADH-ubiquinone oxidoreductase, chain L [Chromobacterium violaceum ATCC 12472]
>gi|34498682|ref|NP_902897.1| hypothetical protein CV3227 [Chromobacterium violaceum ATCC 12472]
>gi|34498693|ref|NP_902908.1| probable oxidoreductase protein (frameshift) [Chromobacterium violaceum ATCC 12472]
>gi|34498695|ref|NP_902910.1| Threonine efflux protein [Chromobacterium violaceum ATCC 12472]
>gi|34498698|ref|NP_902913.1| transmembrane multidrug resistance efflux protein [Chromobacterium violaceum ATCC 12472]
>gi|34498702|ref|NP_902917.1| hypothetical protein CV3247 [Chromobacterium violaceum ATCC 12472]
>gi|34498704|ref|NP_902919.1| ornithine cyclodeaminase [Chromobacterium violaceum ATCC 12472]
>gi|34498708|ref|NP_902923.1| probable pirin-related protein [Chromobacterium violaceum ATCC 12472]
>gi|34498719|ref|NP_902934.1| hypothetical protein CV3264 [Chromobacterium violaceum ATCC 12472]
>gi|34498724|ref|NP_902939.1| hypothetical protein CV3269 [Chromobacterium violaceum ATCC 12472]
>gi|34498741|ref|NP_902956.1| hypothetical protein CV3286 [Chromobacterium violaceum ATCC 12472]
>gi|34498744|ref|NP_902959.1| probable transcriptional regulator, LysR family [Chromobacterium violaceum ATCC 12472]
>gi|34498747|ref|NP_902962.1| Electron transport complex protein [Chromobacterium violaceum ATCC 12472]
>gi|34498769|ref|NP_902984.1| probable membrane transport protein [Chromobacterium violaceum ATCC 12472]
>gi|34498770|ref|NP_902985.1| hypothetical protein CV3315 [Chromobacterium violaceum ATCC 12472]
>gi|34498774|ref|NP_902989.1| hypothetical protein CV3319 [Chromobacterium violaceum ATCC 12472]
>gi|34498779|ref|NP_902994.1| probable Cytochrome b561 [Chromobacterium violaceum ATCC 12472]
>gi|34498787|ref|NP_903002.1| RNA polymerase N (sigma54) factor [Chromobacterium violaceum ATCC 12472]
>gi|34498793|ref|NP_903008.1| hypothetical protein CV3338 [Chromobacterium violaceum ATCC 12472]
>gi|34498794|ref|NP_903009.1| hypothetical protein CV3339 [Chromobacterium violaceum ATCC 12472]
>gi|34498799|ref|NP_903014.1| 3-deoxy-manno-octulosonate cytidylyltransferase [Chromobacterium violaceum ATCC 12472]
>gi|34498802|ref|NP_903017.1| biopolymer transport exbD-related transmembrane protein [Chromobacterium violaceum ATCC 12472]
>gi|34498808|ref|NP_903023.1| hypothetical protein CV3353 [Chromobacterium violaceum ATCC 12472]
>gi|34498813|ref|NP_903028.1| hypothetical protein CV3358 [Chromobacterium violaceum ATCC 12472]
>gi|34498817|ref|NP_903032.1| probable lipoprotein [Chromobacterium violaceum ATCC 12472]
>gi|34498818|ref|NP_903033.1| hypothetical protein CV3363 [Chromobacterium violaceum ATCC 12472]
>gi|34498820|ref|NP_903035.1| hypothetical protein CV3365 [Chromobacterium violaceum ATCC 12472]
>gi|34498821|ref|NP_903036.1| fumarate reductase, subunit D [Chromobacterium violaceum ATCC 12472]
>gi|34498830|ref|NP_903045.1| Cell division inhibitor [Chromobacterium violaceum ATCC 12472]
>gi|34498847|ref|NP_903062.1| hypothetical protein CV3392 [Chromobacterium violaceum ATCC 12472]
>gi|34498855|ref|NP_903070.1| hypothetical protein CV3400 [Chromobacterium violaceum ATCC 12472]
>gi|34498858|ref|NP_903073.1| probable MFS transporter [Chromobacterium violaceum ATCC 12472]
>gi|34498864|ref|NP_903079.1| proton/sodium-glutamate symport protein [Chromobacterium violaceum ATCC 12472]
>gi|34498865|ref|NP_903080.1| 4-amino-4-deoxychorismate lyase [Chromobacterium violaceum ATCC 12472]
>gi|34498870|ref|NP_903085.1| malonyl CoA-acyl carrier protein transacylase [Chromobacterium violaceum ATCC 12472]
>gi|34498894|ref|NP_903109.1| hypothetical protein CV3439 [Chromobacterium violaceum ATCC 12472]
>gi|34498895|ref|NP_903110.1| hypothetical protein CV3440 [Chromobacterium violaceum ATCC 12472]
>gi|34498905|ref|NP_903120.1| probable chemotaxis protein CheA [Chromobacterium violaceum ATCC 12472]
>gi|34498913|ref|NP_903128.1| probable phosphopyruvate hydratase [Chromobacterium violaceum ATCC 12472]
>gi|34498923|ref|NP_903138.1| hypothetical protein CV3468 [Chromobacterium violaceum ATCC 12472]
>gi|34498924|ref|NP_903139.1| hypothetical protein CV3469 [Chromobacterium violaceum ATCC 12472]
>gi|34498925|ref|NP_903140.1| hypothetical protein CV3470 [Chromobacterium violaceum ATCC 12472]
>gi|34498934|ref|NP_903149.1| hypothetical protein CV3479 [Chromobacterium violaceum ATCC 12472]
>gi|34498938|ref|NP_903153.1| hypothetical protein CV3483 [Chromobacterium violaceum ATCC 12472]
>gi|34498940|ref|NP_903155.1| hypothetical protein CV3485 [Chromobacterium violaceum ATCC 12472]
>gi|34498944|ref|NP_903159.1| probable chitin-binding protein [Chromobacterium violaceum ATCC 12472]
>gi|34498946|ref|NP_903161.1| hypothetical protein CV3491 [Chromobacterium violaceum ATCC 12472]
>gi|34498948|ref|NP_903163.1| probable acetyltransferase protein [Chromobacterium violaceum ATCC 12472]
>gi|34498969|ref|NP_903184.1| hypothetical protein CV3514 [Chromobacterium violaceum ATCC 12472]
>gi|34498970|ref|NP_903185.1| hypothetical protein CV3515 [Chromobacterium violaceum ATCC 12472]
>gi|34498972|ref|NP_903187.1| hypothetical protein CV3517 [Chromobacterium violaceum ATCC 12472]
>gi|34498978|ref|NP_903193.1| probable ribonuclease R [Chromobacterium violaceum ATCC 12472]
>gi|34498991|ref|NP_903206.1| hypothetical protein CV3536 [Chromobacterium violaceum ATCC 12472]
>gi|34498994|ref|NP_903209.1| hypothetical protein CV3539 [Chromobacterium violaceum ATCC 12472]
>gi|34498998|ref|NP_903213.1| hypothetical protein CV3543 [Chromobacterium violaceum ATCC 12472]
>gi|34499011|ref|NP_903226.1| hypothetical protein CV3556 [Chromobacterium violaceum ATCC 12472]
>gi|34499012|ref|NP_903227.1| hypothetical protein CV3557 [Chromobacterium violaceum ATCC 12472]
>gi|34499015|ref|NP_903230.1| hypothetical protein CV3560 [Chromobacterium violaceum ATCC 12472]
>gi|34499018|ref|NP_903233.1| hypothetical protein CV3563 [Chromobacterium violaceum ATCC 12472]
>gi|34499035|ref|NP_903250.1| hypothetical protein CV3580 [Chromobacterium violaceum ATCC 12472]
>gi|34499038|ref|NP_903253.1| hypothetical protein CV3583 [Chromobacterium violaceum ATCC 12472]
>gi|34499052|ref|NP_903267.1| probable cysteine synthase B [Chromobacterium violaceum ATCC 12472]
>gi|34499055|ref|NP_903270.1| 3-hydroxyphenylpropionic acid transporter [Chromobacterium violaceum ATCC 12472]
>gi|34499057|ref|NP_903272.1| hypothetical protein CV3602 [Chromobacterium violaceum ATCC 12472]
>gi|34499061|ref|NP_903276.1| hypothetical protein CV3606 [Chromobacterium violaceum ATCC 12472]
>gi|34499062|ref|NP_903277.1| hypothetical protein CV3607 [Chromobacterium violaceum ATCC 12472]
>gi|34499063|ref|NP_903278.1| 4-hydroxybenzoate octaprenyltransferase [Chromobacterium violaceum ATCC 12472]
>gi|34499064|ref|NP_903279.1| hypothetical protein CV3609 [Chromobacterium violaceum ATCC 12472]

>gi|34499071|ref|NP_903286.1| phosphoribosylglycinamide formyltransferase [Chromobacterium violaceum ATCC 12472]
>gi|34499072|ref|NP_903287.1| hypothetical protein CV3617 [Chromobacterium violaceum ATCC 12472]
>gi|34499077|ref|NP_903292.1| hypothetical protein CV3622 [Chromobacterium violaceum ATCC 12472]
>gi|34499091|ref|NP_903306.1| hypothetical protein CV3636 [Chromobacterium violaceum ATCC 12472]
>gi|34499101|ref|NP_903316.1| hypothetical protein CV3646 [Chromobacterium violaceum ATCC 12472]
>gi|34499112|ref|NP_903327.1| cytochrome bd-I oxidase subunit II [Chromobacterium violaceum ATCC 12472]
>gi|34499132|ref|NP_903347.1| probable cation efflux system [Chromobacterium violaceum ATCC 12472]
>gi|34499136|ref|NP_903351.1| probable lipoprotein nlpD precursor [Chromobacterium violaceum ATCC 12472]
>gi|34499138|ref|NP_903353.1| hypothetical protein CV3683 [Chromobacterium violaceum ATCC 12472]
>gi|34499139|ref|NP_903354.1| hypothetical protein CV3684 [Chromobacterium violaceum ATCC 12472]
>gi|34499143|ref|NP_903358.1| hypothetical protein CV3688 [Chromobacterium violaceum ATCC 12472]
>gi|34499159|ref|NP_903374.1| hypothetical protein CV3704 [Chromobacterium violaceum ATCC 12472]
>gi|34499168|ref|NP_903383.1| probable transcriptional regulator [Chromobacterium violaceum ATCC 12472]
>gi|34499174|ref|NP_903389.1| hypothetical protein CV3719 [Chromobacterium violaceum ATCC 12472]
>gi|34499183|ref|NP_903398.1| hypothetical protein CV3728 [Chromobacterium violaceum ATCC 12472]
>gi|34499184|ref|NP_903399.1| probable flagellar protein [Chromobacterium violaceum ATCC 12472]
>gi|34499185|ref|NP_903400.1| probable transcriptional regulator, GntR family [Chromobacterium violaceum ATCC 12472]
>gi|34499186|ref|NP_903401.1| probable MFS transporter [Chromobacterium violaceum ATCC 12472]
>gi|34499187|ref|NP_903402.1| hypothetical protein CV3732 [Chromobacterium violaceum ATCC 12472]
>gi|34499188|ref|NP_903403.1| probable oxidoreductase [Chromobacterium violaceum ATCC 12472]
>gi|34499200|ref|NP_903415.1| probable member of the acetate kinase family [Chromobacterium violaceum ATCC 12472]
>gi|34499202|ref|NP_903417.1| 30S ribosomal protein S20 [Chromobacterium violaceum ATCC 12472]
>gi|34499216|ref|NP_903431.1| hypothetical protein CV3761 [Chromobacterium violaceum ATCC 12472]
>gi|34499220|ref|NP_903435.1| 30S ribosomal protein S21 [Chromobacterium violaceum ATCC 12472]
>gi|34499229|ref|NP_903444.1| hypothetical protein CV3774 [Chromobacterium violaceum ATCC 12472]
>gi|34499240|ref|NP_903455.1| hypothetical protein CV3785 [Chromobacterium violaceum ATCC 12472]
>gi|34499270|ref|NP_903485.1| hypothetical protein CV3815 [Chromobacterium violaceum ATCC 12472]
>gi|34499272|ref|NP_903487.1| probable electron transfer flavoprotein alpha subunit [Chromobacterium violaceum ATCC 12472]
>gi|34499274|ref|NP_903489.1| probable maoC-like dehydratase [Chromobacterium violaceum ATCC 12472]
>gi|34499281|ref|NP_903496.1| probable type IV prepilin-like proteins leader peptide processing enzyme [Chromobacterium violaceum ATCC 12472]
>gi|34499287|ref|NP_903502.1| hypothetical protein CV3832 [Chromobacterium violaceum ATCC 12472]
>gi|34499293|ref|NP_903508.1| probable outer membrane drug efflux lipoprotein [Chromobacterium violaceum ATCC 12472]
>gi|34499297|ref|NP_903512.1| formate dehydrogenase formation protein FdhE [Chromobacterium violaceum ATCC 12472]
>gi|34499300|ref|NP_903515.1| hypothetical protein CV3845 [Chromobacterium violaceum ATCC 12472]
>gi|34499322|ref|NP_903537.1| hypothetical protein CV3867 [Chromobacterium violaceum ATCC 12472]
>gi|34499332|ref|NP_903547.1| Flagellin protein [Chromobacterium violaceum ATCC 12472]
>gi|34499333|ref|NP_903548.1| flagellin [Chromobacterium violaceum ATCC 12472]
>gi|34499334|ref|NP_903549.1| flagellin [Chromobacterium violaceum ATCC 12472]
>gi|34499353|ref|NP_903568.1| probable permease of ABC transporter [Chromobacterium violaceum ATCC 12472]
>gi|34499354|ref|NP_903569.1| ferric citrate transport system ATP-binding protein [Chromobacterium violaceum ATCC 12472]
>gi|34499361|ref|NP_903576.1| hypothetical protein CV3906 [Chromobacterium violaceum ATCC 12472]
>gi|34499363|ref|NP_903578.1| probable chromosome segregation protein [Chromobacterium violaceum ATCC 12472]
>gi|34499370|ref|NP_903585.1| hypothetical protein CV3915 [Chromobacterium violaceum ATCC 12472]
>gi|34499374|ref|NP_903589.1| probable transcriptional regulator, AsnC family [Chromobacterium violaceum ATCC 12472]
>gi|34499378|ref|NP_903593.1| probable transport/efflux transmembrane protein [Chromobacterium violaceum ATCC 12472]
>gi|34499395|ref|NP_903610.1| hypothetical protein CV3940 [Chromobacterium violaceum ATCC 12472]
>gi|34499400|ref|NP_903615.1| probable glutathione-dependent aldehyde dehydrogenase [Chromobacterium violaceum ATCC 12472]
>gi|34499403|ref|NP_903618.1| probable 3-oxoacyl-[acyl-carrier-protein] synthase II [Chromobacterium violaceum ATCC 12472]
>gi|34499410|ref|NP_903625.1| hypothetical protein CV3955 [Chromobacterium violaceum ATCC 12472]
>gi|34499419|ref|NP_903634.1| hypothetical protein CV3964 [Chromobacterium violaceum ATCC 12472]
>gi|34499420|ref|NP_903635.1| probable ClpA/B-type chaperone [Chromobacterium violaceum ATCC 12472]
>gi|34499431|ref|NP_903646.1| hypothetical protein CV3976 [Chromobacterium violaceum ATCC 12472]
>gi|34499440|ref|NP_903655.1| hypothetical protein CV3985 [Chromobacterium violaceum ATCC 12472]
>gi|34499454|ref|NP_903669.1| hypothetical protein CV3999 [Chromobacterium violaceum ATCC 12472]
>gi|34499470|ref|NP_903685.1| chaperonin 10kD subunit [Chromobacterium violaceum ATCC 12472]
>gi|34499499|ref|NP_903714.1| probable transporter, LysE family [Chromobacterium violaceum ATCC 12472]
>gi|34499501|ref|NP_903716.1| hypothetical protein CV4046 [Chromobacterium violaceum ATCC 12472]
>gi|34499502|ref|NP_903717.1| probable L-asparaginase [Chromobacterium violaceum ATCC 12472]
>gi|34499507|ref|NP_903722.1| probable amino acid ABC transporter, permease protein [Chromobacterium violaceum ATCC 12472]
>gi|34499524|ref|NP_903739.1| probable cytochrome c5 [Chromobacterium violaceum ATCC 12472]
>gi|34499535|ref|NP_903750.1| hypothetical protein CV4080 [Chromobacterium violaceum ATCC 12472]
>gi|34499555|ref|NP_903770.1| polyamine transport protein PotC [Chromobacterium violaceum ATCC 12472]
>gi|34499560|ref|NP_903775.1| hypothetical protein CV4105 [Chromobacterium violaceum ATCC 12472]
>gi|34499569|ref|NP_903784.1| hypothetical protein CV4114 [Chromobacterium violaceum ATCC 12472]
>gi|34499595|ref|NP_903810.1| hypothetical protein CV4140 [Chromobacterium violaceum ATCC 12472]
>gi|34499600|ref|NP_903815.1| hypothetical protein CV4145 [Chromobacterium violaceum ATCC 12472]
>gi|34499602|ref|NP_903817.1| probable cation-efflux system transmembrane protein [Chromobacterium violaceum ATCC 12472]
>gi|34499609|ref|NP_903824.1| apolipoprotein N-acyltransferase [Chromobacterium violaceum ATCC 12472]
>gi|34499633|ref|NP_903848.1| 50S ribosomal protein L29 [Chromobacterium violaceum ATCC 12472]

>gi|34499649|ref|NP_903864.1| 50S ribosomal protein L7/L12 [Chromobacterium violaceum ATCC 12472]
>gi|34499654|ref|NP_903869.1| preprotein translocase transmembrane,secE subunit [Chromobacterium violaceum ATCC 12472]
>gi|34499660|ref|NP_903875.1| cell division protein FtsX, ABC transporter integral membrane protein [Chromobacterium violaceum ATCC 12472]
>gi|34499663|ref|NP_903878.1| hypothetical protein CV4208 [Chromobacterium violaceum ATCC 12472]
>gi|34499666|ref|NP_903881.1| hypothetical protein CV4211 [Chromobacterium violaceum ATCC 12472]
>gi|34499669|ref|NP_903884.1| hypothetical protein CV4214 [Chromobacterium violaceum ATCC 12472]
>gi|34499673|ref|NP_903888.1| probable two-component system sensor kinase [Chromobacterium violaceum ATCC 12472]
>gi|34499674|ref|NP_903889.1| hypothetical protein CV4219 [Chromobacterium violaceum ATCC 12472]
>gi|34499676|ref|NP_903891.1| hypothetical protein CV4221 [Chromobacterium violaceum ATCC 12472]
>gi|34499686|ref|NP_903901.1| pyridoxal phosphate biosynthesis protein [Chromobacterium violaceum ATCC 12472]
>gi|34499687|ref|NP_903902.1| hypothetical protein CV4232 [Chromobacterium violaceum ATCC 12472]
>gi|34499691|ref|NP_903906.1| probable transcription regulator, MerR family [Chromobacterium violaceum ATCC 12472]
>gi|34499696|ref|NP_903911.1| probable acid shock protein [Chromobacterium violaceum ATCC 12472]
>gi|34499704|ref|NP_903919.1| hypothetical protein CV4249 [Chromobacterium violaceum ATCC 12472]
>gi|34499705|ref|NP_903920.1| hypothetical protein CV4250 [Chromobacterium violaceum ATCC 12472]
>gi|34499707|ref|NP_903922.1| hypothetical protein CV4252 [Chromobacterium violaceum ATCC 12472]
>gi|34499709|ref|NP_903924.1| probable TonB protein [Chromobacterium violaceum ATCC 12472]
>gi|34499722|ref|NP_903937.1| smf protein [Chromobacterium violaceum ATCC 12472]
>gi|34499728|ref|NP_903943.1| hypothetical protein CV4273 [Chromobacterium violaceum ATCC 12472]
>gi|34499729|ref|NP_903944.1| diacylglycerol kinase [Chromobacterium violaceum ATCC 12472]
>gi|34499747|ref|NP_903962.1| hypothetical protein CV4292 [Chromobacterium violaceum ATCC 12472]
>gi|34499756|ref|NP_903971.1| probable hemolysin [Chromobacterium violaceum ATCC 12472]
>gi|34499762|ref|NP_903977.1| hypothetical protein CV4307 [Chromobacterium violaceum ATCC 12472]
>gi|34499765|ref|NP_903980.1| probable transcription regulator, TetR family [Chromobacterium violaceum ATCC 12472]
>gi|34499772|ref|NP_903987.1| probable multidrug translocase [Chromobacterium violaceum ATCC 12472]
>gi|34499774|ref|NP_903989.1| hypothetical protein CV4319 [Chromobacterium violaceum ATCC 12472]
>gi|34499776|ref|NP_903991.1| hypothetical protein CV4321 [Chromobacterium violaceum ATCC 12472]
>gi|34499814|ref|NP_904029.1| rod shape-determining protein MreD [Chromobacterium violaceum ATCC 12472]
>gi|34499818|ref|NP_904033.1| DNA-damage-inducible protein P [Chromobacterium violaceum ATCC 12472]
>gi|34499822|ref|NP_904037.1| hypothetical protein CV4367 [Chromobacterium violaceum ATCC 12472]
>gi|34499826|ref|NP_904041.1| hypothetical protein CV4371 [Chromobacterium violaceum ATCC 12472]
>gi|34499848|ref|NP_904063.1| probable ABC transporter, permease protein [Chromobacterium violaceum ATCC 12472]
>gi|34499850|ref|NP_904065.1| probable ABC transporter system, amino acid permease [Chromobacterium violaceum ATCC 12472]
>gi|34499851|ref|NP_904066.1| probable ABC transporter, permease protein [Chromobacterium violaceum ATCC 12472]
>gi|34499855|ref|NP_904070.1| probable colicin V secretion ABC transporter ATP-binding protein [Chromobacterium violaceum ATCC 12472]
>gi|34499856|ref|NP_904071.1| hypothetical protein CV4401 [Chromobacterium violaceum ATCC 12472]
>gi|71280048|ref|YP_266826.1| ATP synthase F0, B subunit [Colwellia psychrerythraea 34H]
>gi|71278528|ref|YP_266831.1| ATP synthase F1, epsilon subunit [Colwellia psychrerythraea 34H]
>gi|71278112|ref|YP_266840.1| putative uroporphyrin-III C-methyltransferase [Colwellia psychrerythraea 34H]
>gi|71281635|ref|YP_266891.1| putative glpG protein [Colwellia psychrerythraea 34H]
>gi|71280517|ref|YP_266900.1| hypothetical protein CPS_0132 [Colwellia psychrerythraea 34H]
>gi|71281073|ref|YP_266920.1| hypothetical protein CPS_0152 [Colwellia psychrerythraea 34H]
>gi|71278165|ref|YP_266925.1| signal recognition particle-docking protein FtsY [Colwellia psychrerythraea 34H]
>gi|71278104|ref|YP_266988.1| hypothetical protein CPS_0221 [Colwellia psychrerythraea 34H]
>gi|71278737|ref|YP_267031.1| hypothetical protein CPS_0264 [Colwellia psychrerythraea 34H]
>gi|71279896|ref|YP_267042.1| hypothetical protein CPS_0275 [Colwellia psychrerythraea 34H]
>gi|71278139|ref|YP_267049.1| cytochrome c family protein [Colwellia psychrerythraea 34H]
>gi|71279284|ref|YP_267122.1| hypothetical protein CPS_0364 [Colwellia psychrerythraea 34H]
>gi|71281997|ref|YP_267173.1| ribosomal protein S6 [Colwellia psychrerythraea 34H]
>gi|71278855|ref|YP_267236.1| hypothetical protein CPS_0478 [Colwellia psychrerythraea 34H]
>gi|71281019|ref|YP_267320.1| putative lipoprotein [Colwellia psychrerythraea 34H]
>gi|71279625|ref|YP_267332.1| polysaccharide biosynthesis protein [Colwellia psychrerythraea 34H]
>gi|71279982|ref|YP_267370.1| ribosomal protein L15 [Colwellia psychrerythraea 34H]
>gi|71277934|ref|YP_267451.1| putative membrane protein [Colwellia psychrerythraea 34H]
>gi|71281079|ref|YP_267470.1| hemolysin III [Colwellia psychrerythraea 34H]
>gi|71278203|ref|YP_267633.1| hypothetical protein CPS_0884 [Colwellia psychrerythraea 34H]
>gi|71281502|ref|YP_267641.1| hypothetical protein CPS_0892 [Colwellia psychrerythraea 34H]
>gi|71278777|ref|YP_267677.1| hypothetical protein CPS_0928 [Colwellia psychrerythraea 34H]
>gi|71277762|ref|YP_267679.1| single-strand binding protein [Colwellia psychrerythraea 34H]
>gi|71279126|ref|YP_267696.1| acetyl-CoA carboxylase, biotin carboxyl carrier protein [Colwellia psychrerythraea 34H]
>gi|71280868|ref|YP_267708.1| hypothetical protein CPS_0959 [Colwellia psychrerythraea 34H]
>gi|71280313|ref|YP_267749.1| hypothetical protein CPS_1000 [Colwellia psychrerythraea 34H]
>gi|71277758|ref|YP_267780.1| heme exporter protein CcmD [Colwellia psychrerythraea 34H]
>gi|71279810|ref|YP_267782.1| heme exporter protein CcmB [Colwellia psychrerythraea 34H]
>gi|71280130|ref|YP_267794.1| oxaloacetate decarboxylase, beta subunit [Colwellia psychrerythraea 34H]
>gi|71281316|ref|YP_267798.1| hypothetical protein CPS_1054 [Colwellia psychrerythraea 34H]
>gi|71282541|ref|YP_267828.1| NADH:ubiquinone oxidoreductase, Na(+)-translocating, E subunit [Colwellia psychrerythraea 34H]
>gi|71278087|ref|YP_267834.1| exodeoxyribonuclease VII, small subunit [Colwellia psychrerythraea 34H]
>gi|71278387|ref|YP_268136.1| hypothetical protein CPS_1393 [Colwellia psychrerythraea 34H]
>gi|71282510|ref|YP_268138.1| putative membrane protein [Colwellia psychrerythraea 34H]

>gi|71279744|ref|YP_268185.1| hypothetical protein CPS_1442 [Colwellia psychrerythraea 34H]
>gi|71281328|ref|YP_268214.1| negative regulator of flagellin synthesis FlgM [Colwellia psychrerythraea 34H]
>gi|71280733|ref|YP_268222.1| flagellar hook protein FlgE [Colwellia psychrerythraea 34H]
>gi|71281535|ref|YP_268230.1| flagellin [Colwellia psychrerythraea 34H]
>gi|71278945|ref|YP_268232.1| flagellin [Colwellia psychrerythraea 34H]
>gi|71278505|ref|YP_268236.1| hypothetical protein CPS_1494 [Colwellia psychrerythraea 34H]
>gi|71279599|ref|YP_268246.1| flagellar assembly protein FliH [Colwellia psychrerythraea 34H]
>gi|71279116|ref|YP_268280.1| hypothetical protein CPS_1538 [Colwellia psychrerythraea 34H]
>gi|71278708|ref|YP_268305.1| UDP-3-O-[3-hydroxymyristoyl] glucosamine N-acyltransferase [Colwellia psychrerythraea 34H]
>gi|71281708|ref|YP_268428.1| SCP-like extracellular protein [Colwellia psychrerythraea 34H]
>gi|71278157|ref|YP_268439.1| ribonuclease HI [Colwellia psychrerythraea 34H]
>gi|71282545|ref|YP_268457.1| hypothetical protein CPS_1719 [Colwellia psychrerythraea 34H]
>gi|71278760|ref|YP_268468.1| putative tolA protein [Colwellia psychrerythraea 34H]
>gi|71281739|ref|YP_268528.1| putative membrane protein [Colwellia psychrerythraea 34H]
>gi|71278176|ref|YP_268580.1| putative tonB protein [Colwellia psychrerythraea 34H]
>gi|71281801|ref|YP_268598.1| perfringolysin O regulator protein [Colwellia psychrerythraea 34H]
>gi|71279727|ref|YP_268629.1| hypothetical protein CPS_1899 [Colwellia psychrerythraea 34H]
>gi|71279563|ref|YP_268647.1| hypothetical protein CPS_1917 [Colwellia psychrerythraea 34H]
>gi|71282135|ref|YP_268654.1| hypothetical protein CPS_1924 [Colwellia psychrerythraea 34H]
>gi|71277847|ref|YP_268686.1| putative RND efflux system protein [Colwellia psychrerythraea 34H]
>gi|71278520|ref|YP_268747.1| hypothetical protein CPS_2017 [Colwellia psychrerythraea 34H]
>gi|71281532|ref|YP_268817.1| chain length determinant family protein [Colwellia psychrerythraea 34H]
>gi|71279978|ref|YP_268830.1| putative membrane protein [Colwellia psychrerythraea 34H]
>gi|71282616|ref|YP_268877.1| thrombospondin type 3 repeat family protein/Calx-beta domain protein [Colwellia psychrerythraea 34H]
>gi|71278141|ref|YP_268899.1| hypothetical protein CPS_2174 [Colwellia psychrerythraea 34H]
>gi|71280860|ref|YP_268901.1| hypothetical protein CPS_2176 [Colwellia psychrerythraea 34H]
>gi|71278124|ref|YP_268909.1| hypothetical protein CPS_2184 [Colwellia psychrerythraea 34H]
>gi|71281638|ref|YP_268922.1| electron transport complex, RnfABCDGE type, C subunit [Colwellia psychrerythraea 34H]
>gi|71278447|ref|YP_268928.1| translation initiation factor IF-2 [Colwellia psychrerythraea 34H]
>gi|71280135|ref|YP_268958.1| hypothetical protein CPS_2233 [Colwellia psychrerythraea 34H]
>gi|71281458|ref|YP_269032.1| hypothetical protein CPS_2312 [Colwellia psychrerythraea 34H]
>gi|71281603|ref|YP_269091.1| hypothetical protein CPS_2373 [Colwellia psychrerythraea 34H]
>gi|71280887|ref|YP_269156.1| rarD protein [Colwellia psychrerythraea 34H]
>gi|71278130|ref|YP_269355.1| putative ABC transporter, permease protein [Colwellia psychrerythraea 34H]
>gi|71279125|ref|YP_269523.1| ProP effector [Colwellia psychrerythraea 34H]
>gi|71278430|ref|YP_269567.1| hypothetical protein CPS_2862 [Colwellia psychrerythraea 34H]
>gi|71282403|ref|YP_269666.1| hypothetical protein CPS_2966 [Colwellia psychrerythraea 34H]
>gi|71282432|ref|YP_269675.1| putative bax protein [Colwellia psychrerythraea 34H]
>gi|71282202|ref|YP_269676.1| putative membrane protein [Colwellia psychrerythraea 34H]
>gi|71281017|ref|YP_269785.1| transporter, LysE family [Colwellia psychrerythraea 34H]
>gi|71282583|ref|YP_269859.1| TPR domain protein [Colwellia psychrerythraea 34H]
>gi|71279797|ref|YP_269909.1| hypothetical protein CPS_3213 [Colwellia psychrerythraea 34H]
>gi|71279334|ref|YP_269938.1| putative polysaccharide biosynthesis glycosyltransferase [Colwellia psychrerythraea 34H]
>gi|71278480|ref|YP_269960.1| hypothetical protein CPS_3278 [Colwellia psychrerythraea 34H]
>gi|71281537|ref|YP_270066.1| membrane protein [Colwellia psychrerythraea 34H]
>gi|71278727|ref|YP_270110.1| hypothetical protein CPS_3435 [Colwellia psychrerythraea 34H]
>gi|71277852|ref|YP_270143.1| hypothetical protein CPS_3469 [Colwellia psychrerythraea 34H]
>gi|71281649|ref|YP_270219.1| hypothetical protein CPS_3545 [Colwellia psychrerythraea 34H]
>gi|71281184|ref|YP_270326.1| cobalamin 5'-phosphate synthase [Colwellia psychrerythraea 34H]
>gi|71281838|ref|YP_270371.1| hypothetical protein CPS_3704 [Colwellia psychrerythraea 34H]
>gi|71280152|ref|YP_270417.1| hypothetical protein CPS_3750 [Colwellia psychrerythraea 34H]
>gi|71280326|ref|YP_270419.1| putative TonB protein [Colwellia psychrerythraea 34H]
>gi|71280410|ref|YP_270463.1| dedD protein [Colwellia psychrerythraea 34H]
>gi|71279596|ref|YP_270467.1| hypothetical protein CPS_3804 [Colwellia psychrerythraea 34H]
>gi|71282171|ref|YP_270483.1| chaperone protein DnaJ [Colwellia psychrerythraea 34H]
>gi|71279763|ref|YP_270512.1| hypothetical protein CPS_3850 [Colwellia psychrerythraea 34H]
>gi|71279842|ref|YP_270544.1| leucine rich repeat protein [Colwellia psychrerythraea 34H]
>gi|71281356|ref|YP_270630.1| putative TonB protein [Colwellia psychrerythraea 34H]
>gi|71278768|ref|YP_270637.1| putative lipoprotein [Colwellia psychrerythraea 34H]
>gi|71282196|ref|YP_270739.1| putative granule-associated protein [Colwellia psychrerythraea 34H]
>gi|71279966|ref|YP_270741.1| putative granule-associated protein [Colwellia psychrerythraea 34H]
>gi|71277956|ref|YP_270755.1| hypothetical protein CPS_4100 [Colwellia psychrerythraea 34H]
>gi|71278367|ref|YP_270781.1| sigma-E factor regulatory protein RseC [Colwellia psychrerythraea 34H]
>gi|71278080|ref|YP_270783.1| sigma-E factor negative regulatory protein RseA [Colwellia psychrerythraea 34H]
>gi|71279157|ref|YP_270837.1| putative lipoprotein [Colwellia psychrerythraea 34H]
>gi|71280671|ref|YP_270845.1| putative major outer membrane lipoprotein [Colwellia psychrerythraea 34H]
>gi|71278988|ref|YP_270904.1| lysM domain protein [Colwellia psychrerythraea 34H]
>gi|71281120|ref|YP_270921.1| hypothetical protein CPS_4271 [Colwellia psychrerythraea 34H]
>gi|71281567|ref|YP_270948.1| hypothetical protein CPS_4298 [Colwellia psychrerythraea 34H]
>gi|71282071|ref|YP_270972.1| putative zinc transporter ZitB [Colwellia psychrerythraea 34H]

>gi|71277960|ref|YP_270986.1| ribosomal protein S21 [Colwellia psychrerythraea 34H]
>gi|71278378|ref|YP_271041.1| peptidase, M23/37 family [Colwellia psychrerythraea 34H]
>gi|71282451|ref|YP_271052.1| hypothetical protein CPS_4403 [Colwellia psychrerythraea 34H]
>gi|71281497|ref|YP_271073.1| hypothetical protein CPS_4424 [Colwellia psychrerythraea 34H]
>gi|71279014|ref|YP_271108.1| hypothetical protein CPS_4460 [Colwellia psychrerythraea 34H]
>gi|71281202|ref|YP_271195.1| phosphocarrier protein NPr [Colwellia psychrerythraea 34H]
>gi|71280480|ref|YP_271258.1| multidrug resistance transporter, Bcr/CflA family [Colwellia psychrerythraea 34H]
>gi|71279055|ref|YP_271273.1| hypothetical protein CPS_4626 [Colwellia psychrerythraea 34H]
>gi|71278071|ref|YP_271341.1| hypothetical protein CPS_4697 [Colwellia psychrerythraea 34H]
>gi|71280423|ref|YP_271351.1| hypothetical protein CPS_4707 [Colwellia psychrerythraea 34H]
>gi|71277897|ref|YP_271352.1| hypothetical protein CPS_4708 [Colwellia psychrerythraea 34H]
>gi|71279472|ref|YP_271379.1| copper ABC transporter, permease protein [Colwellia psychrerythraea 34H]
>gi|71279728|ref|YP_271385.1| putative membrane protein [Colwellia psychrerythraea 34H]
>gi|71279061|ref|YP_271397.1| hypothetical protein CPS_4753 [Colwellia psychrerythraea 34H]
>gi|71279888|ref|YP_271414.1| ribosomal protein L7/L12 [Colwellia psychrerythraea 34H]
>gi|71278450|ref|YP_271445.1| pyruvate dehydrogenase complex, E2 component, dihydrolipoamide acetyltransferase [Colwellia psychrerythraea 34H]
>gi|71281113|ref|YP_271476.1| SPFH domain/Band 7 domain protein [Colwellia psychrerythraea 34H]
>gi|11497062|ref|NP_051200.1| ErpB [Borrelia burgdorferi B31]
>gi|11497065|ref|NP_051168.1| hypothetical protein BBP07 [Borrelia burgdorferi B31]
>gi|11497073|ref|NP_051184.1| pore-forming hemolysin [Borrelia burgdorferi B31]
>gi|11497077|ref|NP_051188.1| rev protein [Borrelia burgdorferi B31]
>gi|11497079|ref|NP_051190.1| hypothetical protein BBP29 [Borrelia burgdorferi B31]
>gi|11497083|ref|NP_051194.1| hypothetical protein BBP33 [Borrelia burgdorferi B31]
>gi|11497084|ref|NP_051195.1| BdrA [Borrelia burgdorferi B31]
>gi|11497091|ref|NP_051163.1| hypothetical protein BBP02 [Borrelia burgdorferi B31]
>gi|11497106|ref|NP_051226.1| pore-forming hemolysin [Borrelia burgdorferi B31]
>gi|11497110|ref|NP_051232.1| BdrF [Borrelia burgdorferi B31]
>gi|11497118|ref|NP_051240.1| BdrE [Borrelia burgdorferi B31]
>gi|11497121|ref|NP_051243.1| BppC [Borrelia burgdorferi B31]
>gi|11497126|ref|NP_051210.1| hypothetical protein BBS07 [Borrelia burgdorferi B31]
>gi|11497137|ref|NP_051205.1| hypothetical protein BBS02 [Borrelia burgdorferi B31]
>gi|11497151|ref|NP_051288.1| ErpY [Borrelia burgdorferi B31]
>gi|11497154|ref|NP_051254.1| hypothetical protein BBR07 [Borrelia burgdorferi B31]
>gi|11497162|ref|NP_051270.1| pore-forming hemolysin [Borrelia burgdorferi B31]
>gi|11497166|ref|NP_051274.1| BdrH [Borrelia burgdorferi B31]
>gi|11497168|ref|NP_051276.1| hypothetical protein BBR29 [Borrelia burgdorferi B31]
>gi|11497198|ref|NP_051314.1| pore-forming hemolysin [Borrelia burgdorferi B31]
>gi|11497202|ref|NP_051318.1| rev protein [Borrelia burgdorferi B31]
>gi|11497209|ref|NP_051325.1| BdrK [Borrelia burgdorferi B31]
>gi|11497213|ref|NP_051329.1| ErpK [Borrelia burgdorferi B31]
>gi|11497241|ref|NP_051367.1| BdrM [Borrelia burgdorferi B31]
>gi|11497245|ref|NP_051372.1| ErpL [Borrelia burgdorferi B31]
>gi|11497246|ref|NP_051373.1| ErpM [Borrelia burgdorferi B31]
>gi|11497249|ref|NP_051340.1| hypothetical protein BBO07 [Borrelia burgdorferi B31]
>gi|11497257|ref|NP_051356.1| pore-forming hemolysin [Borrelia burgdorferi B31]
>gi|11497261|ref|NP_051360.1| BdrN [Borrelia burgdorferi B31]
>gi|11497262|ref|NP_051361.1| lipoprotein [Borrelia burgdorferi B31]
>gi|11497268|ref|NP_051335.1| hypothetical protein BBO02 [Borrelia burgdorferi B31]
>gi|11497280|ref|NP_051362.1| hypothetical protein BBO29 [Borrelia burgdorferi B31]
>gi|11497286|ref|NP_051412.1| BdrO [Borrelia burgdorferi B31]
>gi|11497291|ref|NP_051417.1| ErpO [Borrelia burgdorferi B31]
>gi|11497294|ref|NP_051384.1| hypothetical protein BBL07 [Borrelia burgdorferi B31]
>gi|11497302|ref|NP_051400.1| pore forming hemolysin [Borrelia burgdorferi B31]
>gi|11497306|ref|NP_051404.1| BdrP [Borrelia burgdorferi B31]
>gi|11497308|ref|NP_051406.1| hypothetical protein BBL29 [Borrelia burgdorferi B31]
>gi|11497313|ref|NP_051379.1| hypothetical protein BBL02 [Borrelia burgdorferi B31]
>gi|11497326|ref|NP_051425.1| hypothetical protein BBN07 [Borrelia burgdorferi B31]
>gi|11497332|ref|NP_051436.1| pore-forming hemolysin [Borrelia burgdorferi B31]
>gi|11497336|ref|NP_051440.1| BdrR [Borrelia burgdorferi B31]
>gi|11497342|ref|NP_051446.1| BdrQ [Borrelia burgdorferi B31]
>gi|11497346|ref|NP_051450.1| ErpQ [Borrelia burgdorferi B31]
>gi|11497363|ref|NP_051460.1| hypothetical protein BBU06 [Borrelia burgdorferi B31]
>gi|11497364|ref|NP_051461.1| hypothetical protein BBU07 [Borrelia burgdorferi B31]
>gi|11497388|ref|NP_051492.1| pore-forming hemolysin [Borrelia burgdorferi B31]
>gi|11497392|ref|NP_051496.1| BdrW [Borrelia burgdorferi B31]
>gi|11497399|ref|NP_051504.1| BdrV [Borrelia burgdorferi B31]
>gi|11497403|ref|NP_051509.1| ErpX [Borrelia burgdorferi B31]
>gi|11497433|ref|NP_051525.1| hypothetical protein BBQ68 [Borrelia burgdorferi B31]
>gi|11497438|ref|NP_051530.1| hypothetical protein BBQ82 [Borrelia burgdorferi B31]

>gi|11497442|ref|NP_051535.1| hypothetical protein BBQ87 [Borrelia burgdorferi B31]
>gi|15594359|ref|NP_212147.1| hypothetical protein BB0013 [Borrelia burgdorferi B31]
>gi|15594363|ref|NP_212151.1| conserved hypothetical integral membrane protein [Borrelia burgdorferi B31]
>gi|15594408|ref|NP_212196.1| hypothetical protein BB0062 [Borrelia burgdorferi B31]
>gi|15594417|ref|NP_212205.1| hypothetical protein BB0071 [Borrelia burgdorferi B31]
>gi|15594419|ref|NP_212207.1| hypothetical protein BB0073 [Borrelia burgdorferi B31]
>gi|15594421|ref|NP_212209.1| hypothetical protein BB0075 [Borrelia burgdorferi B31]
>gi|15594422|ref|NP_212210.1| cell division protein, putative [Borrelia burgdorferi B31]
>gi|15594424|ref|NP_212212.1| hypothetical protein BB0078 [Borrelia burgdorferi B31]
>gi|15594425|ref|NP_212213.1| hypothetical protein BB0079 [Borrelia burgdorferi B31]
>gi|15594427|ref|NP_212215.1| hypothetical protein BB0081 [Borrelia burgdorferi B31]
>gi|15594436|ref|NP_212224.1| V-type ATPase, subunit K, putative [Borrelia burgdorferi B31]
>gi|15594442|ref|NP_212230.1| V-type ATPase, subunit E, putative [Borrelia burgdorferi B31]
>gi|15594448|ref|NP_212236.1| hypothetical protein BB0102 [Borrelia burgdorferi B31]
>gi|15594469|ref|NP_212258.1| hypothetical protein BB0124 [Borrelia burgdorferi B31]
>gi|15594475|ref|NP_212264.1| hypothetical protein BB0130 [Borrelia burgdorferi B31]
>gi|15594483|ref|NP_212272.1| hypothetical protein BB0138 [Borrelia burgdorferi B31]
>gi|15594501|ref|NP_212290.1| hypothetical protein BB0156 [Borrelia burgdorferi B31]
>gi|15594509|ref|NP_212298.1| Na+/Ca+ exchange protein, putative [Borrelia burgdorferi B31]
>gi|15594522|ref|NP_212311.1| glucose inhibited division protein B (gidB) [Borrelia burgdorferi B31]
>gi|15594529|ref|NP_212318.1| carbon storage regulator (csrA) [Borrelia burgdorferi B31]
>gi|15594534|ref|NP_212323.1| ribosomal protein L35 (rpmI) [Borrelia burgdorferi B31]
>gi|15594537|ref|NP_212326.1| hypothetical protein BB0192 [Borrelia burgdorferi B31]
>gi|15594538|ref|NP_212327.1| hypothetical protein BB0193 [Borrelia burgdorferi B31]
>gi|15594541|ref|NP_212330.1| peptide chain release factor 1 (prfA) [Borrelia burgdorferi B31]
>gi|15594544|ref|NP_212333.1| hypothetical protein BB0199 [Borrelia burgdorferi B31]
>gi|15594557|ref|NP_212346.1| hypothetical protein BB0212 [Borrelia burgdorferi B31]
>gi|15594579|ref|NP_212368.1| conserved hypothetical integral membrane protein [Borrelia burgdorferi B31]
>gi|15594594|ref|NP_212383.1| phosphatidyltransferase [Borrelia burgdorferi B31]
>gi|15594595|ref|NP_212384.1| dedA protein (dedA) [Borrelia burgdorferi B31]
>gi|15594607|ref|NP_212396.1| hypothetical protein BB0262 [Borrelia burgdorferi B31]
>gi|15594610|ref|NP_212399.1| hypothetical protein BB0265 [Borrelia burgdorferi B31]
>gi|15594618|ref|NP_212407.1| flagellar biosynthesis protein (fliR) [Borrelia burgdorferi B31]
>gi|15594619|ref|NP_212408.1| flagellar biosynthesis protein (fliQ) [Borrelia burgdorferi B31]
>gi|15594630|ref|NP_212419.1| flagellar protein (flbC) [Borrelia burgdorferi B31]
>gi|15594631|ref|NP_212420.1| flagellar protein (flbB) [Borrelia burgdorferi B31]
>gi|15594634|ref|NP_212423.1| flagellar assembly protein (fliH) [Borrelia burgdorferi B31]
>gi|15594647|ref|NP_212436.1| cell division protein (ftsW) [Borrelia burgdorferi B31]
>gi|15594661|ref|NP_212450.1| conserved hypothetical integral membrane protein [Borrelia burgdorferi B31]
>gi|15594662|ref|NP_212451.1| conserved hypothetical integral membrane protein [Borrelia burgdorferi B31]
>gi|15594676|ref|NP_212465.1| hypothetical protein BB0331 [Borrelia burgdorferi B31]
>gi|15594688|ref|NP_212477.1| glu-tRNA amidotransferase, subunit C (gluC) [Borrelia burgdorferi B31]
>gi|15594694|ref|NP_212483.1| hypothetical protein BB0349 [Borrelia burgdorferi B31]
>gi|15594699|ref|NP_212488.1| hypothetical protein BB0354 [Borrelia burgdorferi B31]
>gi|15594705|ref|NP_212494.1| hypothetical protein BB0360 [Borrelia burgdorferi B31]
>gi|15594712|ref|NP_212501.1| hypothetical protein BB0367 [Borrelia burgdorferi B31]
>gi|15594744|ref|NP_212533.1| hypothetical protein BB0399 [Borrelia burgdorferi B31]
>gi|15594749|ref|NP_212538.1| hypothetical protein BB0404 [Borrelia burgdorferi B31]
>gi|15594757|ref|NP_212546.1| hypothetical protein BB0412 [Borrelia burgdorferi B31]
>gi|15594796|ref|NP_212585.1| chromate transport protein, putative [Borrelia burgdorferi B31]
>gi|15594801|ref|NP_212590.1| hypothetical protein BB0456 [Borrelia burgdorferi B31]
>gi|15594815|ref|NP_212604.1| hypothetical protein BB0470 [Borrelia burgdorferi B31]
>gi|15594832|ref|NP_212621.1| ribosomal protein S17 (rpsQ) [Borrelia burgdorferi B31]
>gi|15594841|ref|NP_212630.1| ribosomal protein L30 (rpmD) [Borrelia burgdorferi B31]
>gi|15594842|ref|NP_212631.1| ribosomal protein L15 (rplO) [Borrelia burgdorferi B31]
>gi|15594852|ref|NP_212641.1| hypothetical protein BB0507 [Borrelia burgdorferi B31]
>gi|15594883|ref|NP_212672.1| hypothetical protein BB0538 [Borrelia burgdorferi B31]
>gi|15594891|ref|NP_212680.1| hypothetical protein BB0546 [Borrelia burgdorferi B31]
>gi|15594892|ref|NP_212681.1| hypothetical protein BB0547 [Borrelia burgdorferi B31]
>gi|15594894|ref|NP_212683.1| hypothetical protein BB0549 [Borrelia burgdorferi B31]
>gi|15594898|ref|NP_212687.1| hypothetical protein BB0553 [Borrelia burgdorferi B31]
>gi|15594907|ref|NP_212696.1| hypothetical protein BB0562 [Borrelia burgdorferi B31]
>gi|15594919|ref|NP_212708.1| conserved hypothetical integral membrane protein [Borrelia burgdorferi B31]
>gi|15594921|ref|NP_212710.1| hypothetical protein BB0576 [Borrelia burgdorferi B31]
>gi|15594925|ref|NP_212714.1| conserved hypothetical integral membrane protein [Borrelia burgdorferi B31]
>gi|15594936|ref|NP_212725.1| competence locus E, putative [Borrelia burgdorferi B31]
>gi|15594937|ref|NP_212726.1| hypothetical protein BB0592 [Borrelia burgdorferi B31]
>gi|15594986|ref|NP_212775.1| spermidine/putrescine ABC transporter, permease protein (potB) [Borrelia burgdorferi B31]
>gi|15595012|ref|NP_212801.1| hypothetical protein BB0667 [Borrelia burgdorferi B31]
>gi|15595019|ref|NP_212808.1| hypothetical protein BB0674 [Borrelia burgdorferi B31]

>gi|15595046|ref|NP_212835.1| hypothetical protein BB0701 [Borrelia burgdorferi B31]
>gi|15595053|ref|NP_212842.1| hypothetical protein BB0708 [Borrelia burgdorferi B31]
>gi|15595062|ref|NP_212851.1| conserved hypothetical integral membrane protein [Borrelia burgdorferi B31]
>gi|15595068|ref|NP_212857.1| hypothetical protein BB0723 [Borrelia burgdorferi B31]
>gi|15595085|ref|NP_212874.1| hypothetical protein BB0740 [Borrelia burgdorferi B31]
>gi|15595095|ref|NP_212884.1| hypothetical protein BB0750 [Borrelia burgdorferi B31]
>gi|15595104|ref|NP_212893.1| hypothetical protein BB0759 [Borrelia burgdorferi B31]
>gi|15595111|ref|NP_212900.1| colicin V production protein, putative [Borrelia burgdorferi B31]
>gi|15595146|ref|NP_212935.1| translation initiation factor 2 (infB) [Borrelia burgdorferi B31]
>gi|15595152|ref|NP_212941.1| conserved hypothetical integral membrane protein [Borrelia burgdorferi B31]
>gi|15595158|ref|NP_212947.1| hypothetical protein BB0813 [Borrelia burgdorferi B31]
>gi|15595171|ref|NP_212960.1| hypothetical protein BB0826 [Borrelia burgdorferi B31]
>gi|15595197|ref|NP_212253.1| phosphatidate cytidylyltransferase [Borrelia burgdorferi B31]
>gi|11496591|ref|NP_045398.1| hypothetical protein BBD14 [Borrelia burgdorferi B31]
>gi|11496593|ref|NP_045395.1| hypothetical protein BBD11 [Borrelia burgdorferi B31]
>gi|11496597|ref|NP_045406.1| hypothetical protein BBD24 [Borrelia burgdorferi B31]
>gi|11496599|ref|NP_045386.1| hypothetical protein BBD02 [Borrelia burgdorferi B31]
>gi|11496609|ref|NP_045428.1| hypothetical protein BBE21 [Borrelia burgdorferi B31]
>gi|11496622|ref|NP_045433.1| hypothetical protein BBE27 [Borrelia burgdorferi B31]
>gi|11496641|ref|NP_045439.1| hypothetical protein BBF03 [Borrelia burgdorferi B31]
>gi|11496642|ref|NP_045438.1| hypothetical protein BBF02 [Borrelia burgdorferi B31]
>gi|11496643|ref|NP_045437.1| erpD protein, putative [Borrelia burgdorferi B31]
>gi|11496644|ref|NP_045459.1| hypothetical protein BBF26 [Borrelia burgdorferi B31]
>gi|11496650|ref|NP_045451.1| hypothetical protein BBF16 [Borrelia burgdorferi B31]
>gi|11496655|ref|NP_045444.1| hypothetical protein BBF08 [Borrelia burgdorferi B31]
>gi|11496658|ref|NP_045440.1| hypothetical protein BBF04 [Borrelia burgdorferi B31]
>gi|11496675|ref|NP_045493.1| hypothetical protein BBG33 [Borrelia burgdorferi B31]
>gi|11496694|ref|NP_045488.1| hypothetical protein BBG28 [Borrelia burgdorferi B31]
>gi|11496699|ref|NP_045506.1| hypothetical protein BBH13 [Borrelia burgdorferi B31]
>gi|11496709|ref|NP_045497.1| hypothetical protein BBH03 [Borrelia burgdorferi B31]
>gi|11496723|ref|NP_045517.1| hypothetical protein BBH26 [Borrelia burgdorferi B31]
>gi|11496737|ref|NP_045550.1| hypothetical protein BBI19 [Borrelia burgdorferi B31]
>gi|11496748|ref|NP_045567.1| antigen, P35, putative [Borrelia burgdorferi B31]
>gi|11496750|ref|NP_045540.1| hypothetical protein BBI09 [Borrelia burgdorferi B31]
>gi|11496751|ref|NP_045541.1| hypothetical protein BBI10 [Borrelia burgdorferi B31]
>gi|11496755|ref|NP_045546.1| hypothetical protein BBI15 [Borrelia burgdorferi B31]
>gi|11496756|ref|NP_045547.1| hypothetical protein BBI16 [Borrelia burgdorferi B31]
>gi|11496761|ref|NP_045569.1| hypothetical protein BBI38 [Borrelia burgdorferi B31]
>gi|11496762|ref|NP_045570.1| hypothetical protein BBI39 [Borrelia burgdorferi B31]
>gi|11496771|ref|NP_045559.1| hypothetical protein BBI28 [Borrelia burgdorferi B31]
>gi|11496791|ref|NP_045598.1| hypothetical protein BBK24 [Borrelia burgdorferi B31]
>gi|11496792|ref|NP_045612.1| hypothetical protein BBK40 [Borrelia burgdorferi B31]
>gi|11496805|ref|NP_045584.1| hypothetical protein BBK10 [Borrelia burgdorferi B31]
>gi|11496808|ref|NP_045606.1| hypothetical protein BBK33 [Borrelia burgdorferi B31]
>gi|11496809|ref|NP_045600.1| hypothetical protein BBK27 [Borrelia burgdorferi B31]
>gi|11496814|ref|NP_045607.1| hypothetical protein BBK34 [Borrelia burgdorferi B31]
>gi|11496820|ref|NP_045593.1| hypothetical protein BBK19 [Borrelia burgdorferi B31]
>gi|11496822|ref|NP_045597.1| hypothetical protein BBK23 [Borrelia burgdorferi B31]
>gi|11496827|ref|NP_045614.1| hypothetical protein BBK42 [Borrelia burgdorferi B31]
>gi|11496832|ref|NP_045633.1| outer surface protein D (ospD) [Borrelia burgdorferi B31]
>gi|11496837|ref|NP_045643.1| hypothetical protein BBJ19 [Borrelia burgdorferi B31]
>gi|11496839|ref|NP_045665.1| antigen, P35, putative [Borrelia burgdorferi B31]
>gi|11496843|ref|NP_045631.1| hypothetical protein BBJ07 [Borrelia burgdorferi B31]
>gi|11496859|ref|NP_045661.1| hypothetical protein BBJ37 [Borrelia burgdorferi B31]
>gi|11496863|ref|NP_045627.1| hypothetical protein BBJ02 [Borrelia burgdorferi B31]
>gi|11496876|ref|NP_045670.1| hypothetical protein BBJ46 [Borrelia burgdorferi B31]
>gi|11496904|ref|NP_045725.1| outer membrane protein [Borrelia burgdorferi B31]
>gi|11496905|ref|NP_045732.1| lipoprotein [Borrelia burgdorferi B31]
>gi|11496906|ref|NP_045733.1| surface lipoprotein P27 [Borrelia burgdorferi B31]
>gi|11496912|ref|NP_045691.1| hypothetical protein BBA18 [Borrelia burgdorferi B31]
>gi|11496944|ref|NP_045717.1| hypothetical protein BBA44 [Borrelia burgdorferi B31]
>gi|11496949|ref|NP_045723.1| hypothetical protein BBA50 [Borrelia burgdorferi B31]
>gi|11496954|ref|NP_045742.1| hypothetical protein BBA69 [Borrelia burgdorferi B31]
>gi|11497012|ref|NP_046999.1| hypothetical protein BBB13 [Borrelia burgdorferi B31]
>gi|11497015|ref|NP_046996.1| hypothetical protein BBB10 [Borrelia burgdorferi B31]
>gi|11497024|ref|NP_047005.1| outer surface protein C (ospC) [Borrelia burgdorferi B31]
>gi|11497053|ref|NP_047023.1| hypothetical protein BBC08 [Borrelia burgdorferi B31]
>gi|11497055|ref|NP_047025.1| hypothetical protein BBC11 [Borrelia burgdorferi B31]
>gi|11497057|ref|NP_047016.1| hypothetical protein BBC01 [Borrelia burgdorferi B31]
>gi|60115472|ref|YP_209264.1| hypothetical protein YeeJ [Salmonella enterica subsp. enterica serovar Choleraesuis]

>gi|60115515|ref|YP_209306.1| hypothetical protein SC001 [Salmonella enterica subsp. enterica serovar Choleraesuis]
>gi|60115520|ref|YP_209311.1| relaxase/helicase [Salmonella enterica subsp. enterica serovar Choleraesuis]
>gi|60115535|ref|YP_209326.1| MerD [Salmonella enterica subsp. enterica serovar Choleraesuis]
>gi|60115536|ref|YP_209327.1| MerE [Salmonella enterica subsp. enterica serovar Choleraesuis]
>gi|60115586|ref|YP_209377.1| TraL-like protein [Salmonella enterica subsp. enterica serovar Choleraesuis]
>gi|60115618|ref|YP_209409.1| hypothetical protein SC107 [Salmonella enterica subsp. enterica serovar Choleraesuis]
>gi|60115630|ref|YP_209421.1| single-stranded DNA binding protein [Salmonella enterica subsp. enterica serovar Choleraesuis]
>gi|60115631|ref|YP_209422.1| hypothetical protein SC121 [Salmonella enterica subsp. enterica serovar Choleraesuis]
>gi|62178722|ref|YP_215139.1| pyruvate dehydrogenase, dihydrolipoyltransacetylase component [Salmonella enterica subsp. enterica serovar Choleraesuis str. SC-B67]
>gi|62178726|ref|YP_215143.1| putative outer membrane protein [Salmonella enterica subsp. enterica serovar Choleraesuis str. SC-B67]
>gi|62178731|ref|YP_215148.1| 2-keto-3-deoxygluconate permease [Salmonella enterica subsp. enterica serovar Choleraesuis str. SC-B67]
>gi|62178764|ref|YP_215181.1| ABC superfamily (membrane), hydroxamate-dependent iron uptake [Salmonella enterica subsp. enterica serovar Choleraesuis str. SC-B67]
>gi|62178770|ref|YP_215187.1| putative minor fimbrial subunit; putative adhesin [Salmonella enterica subsp. enterica serovar Choleraesuis str. SC-B67]
>gi|62178932|ref|YP_215349.1| DNA transfer protein gp7 precursor [Salmonella enterica subsp. enterica serovar Choleraesuis str. SC-B67]
>gi|62179006|ref|YP_215423.1| ATP-dependent dsDNA exonuclease [Salmonella enterica subsp. enterica serovar Choleraesuis str. SC-B67]
>gi|62179065|ref|YP_215482.1| putative DNA uptake protein and related DNA-binding proteins [Salmonella enterica subsp. enterica serovar Choleraesuis str. SC-B67]
>gi|62179112|ref|YP_215529.1| putative inner membrane protein [Salmonella enterica subsp. enterica serovar Choleraesuis str. SC-B67]
>gi|62179119|ref|YP_215536.1| putative inner membrane protein [Salmonella enterica subsp. enterica serovar Choleraesuis str. SC-B67]
>gi|62179183|ref|YP_215600.1| putative inner membrane protein [Salmonella enterica subsp. enterica serovar Choleraesuis str. SC-B67]
>gi|62179193|ref|YP_215610.1| ABC superfamily (membrane), ferric enterobactin (enterochelin) transporter [Salmonella enterica subsp. enterica serovar Choleraesuis str. SC-B67]
>gi|62179194|ref|YP_215611.1| putative POT family transport protein [Salmonella enterica subsp. enterica serovar Choleraesuis str. SC-B67]
>gi|62179238|ref|YP_215655.1| minor lipoprotein [Salmonella enterica subsp. enterica serovar Choleraesuis str. SC-B67]
>gi|62179294|ref|YP_215711.1| P-type ATPase, high-affinity potassium transport system, C chain [Salmonella enterica subsp. enterica serovar Choleraesuis str. SC-B67]
>gi|62179321|ref|YP_215738.1| tol protein, membrane spanning protein [Salmonella enterica subsp. enterica serovar Choleraesuis str. SC-B67]
>gi|62179324|ref|YP_215741.1| putative periplasmic protein [Salmonella enterica subsp. enterica serovar Choleraesuis str. SC-B67]
>gi|62179328|ref|YP_215745.1| putative homeobox protein [Salmonella enterica subsp. enterica serovar Choleraesuis str. SC-B67]
>gi|62179338|ref|YP_215755.1| putative ABC transport protein [Salmonella enterica subsp. enterica serovar Choleraesuis str. SC-B67]
>gi|62179381|ref|YP_215798.1| putative inner membrane protein [Salmonella enterica subsp. enterica serovar Choleraesuis str. SC-B67]
>gi|62179398|ref|YP_215815.1| putative permease [Salmonella enterica subsp. enterica serovar Choleraesuis str. SC-B67]
>gi|62179484|ref|YP_215901.1| cell division protein, required for cell division and chromosome partitioning [Salmonella enterica subsp. enterica serovar Choleraesuis str. SC-B67]
>gi|62179572|ref|YP_215989.1| Gifsy-2 prophage probable tail fiber protein [Salmonella enterica subsp. enterica serovar Choleraesuis str. SC-B67]
>gi|62179632|ref|YP_216049.1| putative cytoplasmic protein [Salmonella enterica subsp. enterica serovar Choleraesuis str. SC-B67]
>gi|62179634|ref|YP_216051.1| Suppression of copper sensitivity: putative copper binding protein [Salmonella enterica subsp. enterica serovar Choleraesuis str. SC-B67]
>gi|62179702|ref|YP_216119.1| RNase E [Salmonella enterica subsp. enterica serovar Choleraesuis str. SC-B67]
>gi|62179779|ref|YP_216196.1| putative prophage membrane protein [Salmonella enterica subsp. enterica serovar Choleraesuis str. SC-B67]
>gi|62179782|ref|YP_216199.1| Protein gp55 precursor [Salmonella enterica subsp. enterica serovar Choleraesuis str. SC-B67]
>gi|62179806|ref|YP_216223.1| side tail fiber protein [Salmonella enterica subsp. enterica serovar Choleraesuis str. SC-B67]
>gi|62179807|ref|YP_216224.1| unknown protein encoded by prophage CP-933X [Salmonella enterica subsp. enterica serovar Choleraesuis str. SC-B67]
>gi|62179855|ref|YP_216272.1| putative inner membrane protein [Salmonella enterica subsp. enterica serovar Choleraesuis str. SC-B67]
>gi|62179859|ref|YP_216276.1| putative MFS family transport protein (amino acid/amine transport) [Salmonella enterica subsp. enterica serovar Choleraesuis str. SC-B67]
>gi|62179924|ref|YP_216341.1| 50S ribosomal protein L35 [Salmonella enterica subsp. enterica serovar Choleraesuis str. SC-B67]
>gi|62179929|ref|YP_216346.1| ABC superfamily (membrane), vitamin B12 transport protein [Salmonella enterica subsp. enterica serovar Choleraesuis str. SC-B67]
>gi|62179957|ref|YP_216374.1| putative cytoplasmic protein [Salmonella enterica subsp. enterica serovar Choleraesuis str. SC-B67]
>gi|62179967|ref|YP_216384.1| putative methyl-accepting chemotaxis protein [Salmonella enterica subsp. enterica serovar Choleraesuis str. SC-B67]
>gi|62179968|ref|YP_216385.1| murein lipoprotein, links outer and inner membranes [Salmonella enterica subsp. enterica serovar Choleraesuis str. SC-B67]
>gi|62179992|ref|YP_216409.1| Secretion system effector SseD [Salmonella enterica subsp. enterica serovar Choleraesuis str. SC-B67]
>gi|62180033|ref|YP_216450.1| putative outer membrane lipoprotein [Salmonella enterica subsp. enterica serovar Choleraesuis str. SC-B67]
>gi|62180042|ref|YP_216459.1| putative NADH-quinone reductase, membrane protein [Salmonella enterica subsp. enterica serovar Choleraesuis str. SC-B67]
>gi|62180045|ref|YP_216462.1| putative respiratory-chain NADH dehydrogenase [Salmonella enterica subsp. enterica serovar Choleraesuis str. SC-B67]
>gi|62180048|ref|YP_216465.1| putative inner membrane protein [Salmonella enterica subsp. enterica serovar Choleraesuis str. SC-B67]
>gi|62180073|ref|YP_216490.1| acid shock protein [Salmonella enterica subsp. enterica serovar Choleraesuis str. SC-B67]
>gi|62180091|ref|YP_216508.1| putative inner membrane lipoprotein [Salmonella enterica subsp. enterica serovar Choleraesuis str. SC-B67]
>gi|62180100|ref|YP_216517.1| putative cytoplasmic protein [Salmonella enterica subsp. enterica serovar Choleraesuis str. SC-B67]
>gi|62180160|ref|YP_216577.1| ssrAB activated gene [Salmonella enterica subsp. enterica serovar Choleraesuis str. SC-B67]
>gi|62180168|ref|YP_216585.1| putative membrane protein UgtL [Salmonella enterica subsp. enterica serovar Choleraesuis str. SC-B67]
>gi|62180270|ref|YP_216687.1| osmotically inducible lipoprotein [Salmonella enterica subsp. enterica serovar Choleraesuis str. SC-B67]

>gi|62180294|ref|YP_216711.1| putative cytoplasmic protein [Salmonella enterica subsp. enterica serovar Choleraesuis str. SC-B67]
>gi|62180300|ref|YP_216717.1| putative inner membrane protein [Salmonella enterica subsp. enterica serovar Choleraesuis str. SC-B67]
>gi|62180303|ref|YP_216720.1| energy transducer; uptake of iron, cyanocobalimin; sensitivity to phages, colicins [Salmonella enterica subsp. enterica serovar Choleraesuis str. SC-B67]
>gi|62180316|ref|YP_216733.1| DNA-binding protein HLP-II (HU, BH2, HD, NS); pleiotropic regulator [Salmonella enterica subsp. enterica serovar Choleraesuis str. SC-B67]
>gi|62180373|ref|YP_216790.1| putative cytoplasmic protein [Salmonella enterica subsp. enterica serovar Choleraesuis str. SC-B67]
>gi|62180387|ref|YP_216804.1| putative cytoplasmic protein [Salmonella enterica subsp. enterica serovar Choleraesuis str. SC-B67]
>gi|62180405|ref|YP_216822.1| putative periplasmic or exported protein [Salmonella enterica subsp. enterica serovar Choleraesuis str. SC-B67]
>gi|62180412|ref|YP_216829.1| activator of proP [Salmonella enterica subsp. enterica serovar Choleraesuis str. SC-B67]
>gi|62180455|ref|YP_216872.1| putative inner membrane protein [Salmonella enterica subsp. enterica serovar Choleraesuis str. SC-B67]
>gi|62180534|ref|YP_216951.1| flagellar biosynthesis; filament capping protein; enables filament assembly [Salmonella enterica subsp. enterica serovar Choleraesuis str. SC-B67]
>gi|62180546|ref|YP_216963.1| flagellar biosynthesis; possible export of flagellar proteins [Salmonella enterica subsp. enterica serovar Choleraesuis str. SC-B67]
>gi|62180549|ref|YP_216966.1| flagellar hook-length control protein [Salmonella enterica subsp. enterica serovar Choleraesuis str. SC-B67]
>gi|62180556|ref|YP_216973.1| putative flagellar biosynthetic protein [Salmonella enterica subsp. enterica serovar Choleraesuis str. SC-B67]
>gi|62180616|ref|YP_217033.1| Propanediol utilization: polyhedral bodies [Salmonella enterica subsp. enterica serovar Choleraesuis str. SC-B67]
>gi|62180623|ref|YP_217040.1| Propanediol utilization: polyhedral bodies [Salmonella enterica subsp. enterica serovar Choleraesuis str. SC-B67]
>gi|62180699|ref|YP_217116.1| putative HlyD family secretion protein [Salmonella enterica subsp. enterica serovar Choleraesuis str. SC-B67]
>gi|62180705|ref|YP_217122.1| putative inner membrane protein [Salmonella enterica subsp. enterica serovar Choleraesuis str. SC-B67]
>gi|62180756|ref|YP_217173.1| putative DedA family, membrane protein [Salmonella enterica subsp. enterica serovar Choleraesuis str. SC-B67]
>gi|62180818|ref|YP_217235.1| hypothetical protein SC2248 [Salmonella enterica subsp. enterica serovar Choleraesuis str. SC-B67]
>gi|62180826|ref|YP_217243.1| heme exporter protein C, cytochrome c-type biogenesis protein [Salmonella enterica subsp. enterica serovar Choleraesuis str. SC-B67]
>gi|62180828|ref|YP_217245.1| ABC superfamily (membrane) heme exporter protein, cytochrome c-type biogenesis protein [Salmonella enterica subsp. enterica serovar Choleraesuis str. SC-B67]
>gi|62180874|ref|YP_217291.1| putative inner membrane protein [Salmonella enterica subsp. enterica serovar Choleraesuis str. SC-B67]
>gi|62180936|ref|YP_217353.1| paral putative lipoprotein [Salmonella enterica subsp. enterica serovar Choleraesuis str. SC-B67]
>gi|62180980|ref|YP_217397.1| putative periplasmic protein [Salmonella enterica subsp. enterica serovar Choleraesuis str. SC-B67]
>gi|62180996|ref|YP_217413.1| cell division protein involved in FtsZ ring [Salmonella enterica subsp. enterica serovar Choleraesuis str. SC-B67]
>gi|62181031|ref|YP_217448.1| putative detox protein in ethanolamine utilization [Salmonella enterica subsp. enterica serovar Choleraesuis str. SC-B67]
>gi|62181091|ref|YP_217508.1| paral putative membrane protein [Salmonella enterica subsp. enterica serovar Choleraesuis str. SC-B67]
>gi|62181187|ref|YP_217604.1| Gifsy-2 prophage protein [Salmonella enterica subsp. enterica serovar Choleraesuis str. SC-B67]
>gi|62181212|ref|YP_217629.1| regulator of sigma E (sigma 24) factor [Salmonella enterica subsp. enterica serovar Choleraesuis str. SC-B67]
>gi|62181387|ref|YP_217804.1| cell invasion protein [Salmonella enterica subsp. enterica serovar Choleraesuis str. SC-B67]
>gi|62181542|ref|YP_217959.1| hypothetical protein SC2972 [Salmonella enterica subsp. enterica serovar Choleraesuis str. SC-B67]
>gi|62181572|ref|YP_217989.1| putative ligase [Salmonella enterica subsp. enterica serovar Choleraesuis str. SC-B67]
>gi|62181584|ref|YP_218001.1| putative inner membrane protein [Salmonella enterica subsp. enterica serovar Choleraesuis str. SC-B67]
>gi|62181665|ref|YP_218082.1| putative membrane-associated protein [Salmonella enterica subsp. enterica serovar Choleraesuis str. SC-B67]
>gi|62181716|ref|YP_218133.1| paral putative membrane protein [Salmonella enterica subsp. enterica serovar Choleraesuis str. SC-B67]
>gi|62181749|ref|YP_218166.1| putative membrane-associated protein [Salmonella enterica subsp. enterica serovar Choleraesuis str. SC-B67]
>gi|62181791|ref|YP_218208.1| cysteine sulfinate desulfinase [Salmonella enterica subsp. enterica serovar Choleraesuis str. SC-B67]
>gi|62181797|ref|YP_218214.1| protein chain initiation factor IF-2 [Salmonella enterica subsp. enterica serovar Choleraesuis str. SC-B67]
>gi|62181802|ref|YP_218219.1| preprotein translocase IISP family, auxillary membrane component [Salmonella enterica subsp. enterica serovar Choleraesuis str. SC-B67]
>gi|62181869|ref|YP_218286.1| putative outer membrane protein [Salmonella enterica subsp. enterica serovar Choleraesuis str. SC-B67]
>gi|62181886|ref|YP_218303.1| putative inner membrane protein [Salmonella enterica subsp. enterica serovar Choleraesuis str. SC-B67]
>gi|62181887|ref|YP_218304.1| acetylCoA carboxylase, BCCP subunit, carrier of biotin [Salmonella enterica subsp. enterica serovar Choleraesuis str. SC-B67]
>gi|62181925|ref|YP_218342.1| 50S ribosomal subunit protein L15 [Salmonella enterica subsp. enterica serovar Choleraesuis str. SC-B67]
>gi|62181936|ref|YP_218353.1| 50S ribosomal subunit protein L29 [Salmonella enterica subsp. enterica serovar Choleraesuis str. SC-B67]
>gi|62181953|ref|YP_218370.1| putative oxidation of intracellular sulfur [Salmonella enterica subsp. enterica serovar Choleraesuis str. SC-B67]
>gi|62181959|ref|YP_218376.1| FKBP-type peptidyl prolyl cis-trans isomerase (rotamase) [Salmonella enterica subsp. enterica serovar Choleraesuis str. SC-B67]
>gi|62181987|ref|YP_218404.1| membrane protein [Salmonella enterica subsp. enterica serovar Choleraesuis str. SC-B67]
>gi|62181993|ref|YP_218410.1| putative inner membrane protein [Salmonella enterica subsp. enterica serovar Choleraesuis str. SC-B67]
>gi|62182001|ref|YP_218418.1| putative inner membrane protein [Salmonella enterica subsp. enterica serovar Choleraesuis str. SC-B67]
>gi|62182051|ref|YP_218468.1| putative outer membrane protein [Salmonella enterica subsp. enterica serovar Choleraesuis str. SC-B67]
>gi|62182070|ref|YP_218487.1| GTPase domain of cell division membrane protein [Salmonella enterica subsp. enterica serovar Choleraesuis str. SC-B67]
>gi|62182074|ref|YP_218491.1| putative inner membrane protein [Salmonella enterica subsp. enterica serovar Choleraesuis str. SC-B67]
>gi|62182129|ref|YP_218546.1| ABC superfamily (membrane), dipeptide transport protein 2 [Salmonella enterica subsp. enterica serovar Choleraesuis str. SC-B67]
>gi|62182147|ref|YP_218564.1| putative outer membrane lipoprotein [Salmonella enterica subsp. enterica serovar Choleraesuis str. SC-B67]
>gi|62182182|ref|YP_218599.1| putative cytoplasmic protein [Salmonella enterica subsp. enterica serovar Choleraesuis str. SC-B67]
>gi|62182184|ref|YP_218601.1| putative inner membrane lipoprotein [Salmonella enterica subsp. enterica serovar Choleraesuis str. SC-B67]
>gi|62182185|ref|YP_218602.1| putative inner membrane protein [Salmonella enterica subsp. enterica serovar Choleraesuis str. SC-B67]
>gi|62182240|ref|YP_218657.1| GltS family, glutamate transport protein [Salmonella enterica subsp. enterica serovar Choleraesuis str. SC-B67]

>gi|62182253|ref|YP_218670.1| putative inner membrane protein [Salmonella enterica subsp. enterica serovar Choleraesuis str. SC-B67]
>gi|62182279|ref|YP_218696.1| sensory histidine kinase in two-component regulatory sytem with UhpA [Salmonella enterica subsp. enterica serovar Choleraesuis str. SC-B67]
>gi|62182297|ref|YP_218714.1| putative inner membrane protein [Salmonella enterica subsp. enterica serovar Choleraesuis str. SC-B67]
>gi|62182351|ref|YP_218768.1| membrane-bound ATP synthase, F0 sector, subunit b [Salmonella enterica subsp. enterica serovar Choleraesuis str. SC-B67]
>gi|62182352|ref|YP_218769.1| ATP synthase C chain [Salmonella enterica subsp. enterica serovar Choleraesuis str. SC-B67]
>gi|62182354|ref|YP_218771.1| membrane-bound ATP synthase subunit, F1-F0-type proton-ATPase [Salmonella enterica subsp. enterica serovar Choleraesuis str. SC-B67]
>gi|62182412|ref|YP_218829.1| putative inner membrane protein [Salmonella enterica subsp. enterica serovar Choleraesuis str. SC-B67]
>gi|62182441|ref|YP_218858.1| component of Sec-independent protein secretion pathway [Salmonella enterica subsp. enterica serovar Choleraesuis str. SC-B67]
>gi|62182465|ref|YP_218882.1| putative cytoplasmic protein [Salmonella enterica subsp. enterica serovar Choleraesuis str. SC-B67]
>gi|62182535|ref|YP_218952.1| putative ABC superfamily (membrane), sugar transport protein [Salmonella enterica subsp. enterica serovar Choleraesuis str. SC-B67]
>gi|62182552|ref|YP_218969.1| essential cell division protein [Salmonella enterica subsp. enterica serovar Choleraesuis str. SC-B67]
>gi|62182606|ref|YP_219023.1| 50S ribosomal subunit protein L7/L12 [Salmonella enterica subsp. enterica serovar Choleraesuis str. SC-B67]
>gi|62182650|ref|YP_219067.1| putative phage tail fiber protein H [Salmonella enterica subsp. enterica serovar Choleraesuis str. SC-B67]
>gi|62182656|ref|YP_219073.1| putative phage baseplate component [Salmonella enterica subsp. enterica serovar Choleraesuis str. SC-B67]
>gi|62182671|ref|YP_219088.1| putative outer membrane protein [Salmonella enterica subsp. enterica serovar Choleraesuis str. SC-B67]
>gi|62182685|ref|YP_219102.1| diacylglycerol kinase [Salmonella enterica subsp. enterica serovar Choleraesuis str. SC-B67]
>gi|62182687|ref|YP_219104.1| DNA-damage-inducible protein F, induced by UV and mitomycin C; SOS, lexA regulon [Salmonella enterica subsp. enterica serovar Choleraesuis str. SC-B67]
>gi|62182693|ref|YP_219110.1| putative inner membrane protein [Salmonella enterica subsp. enterica serovar Choleraesuis str. SC-B67]
>gi|62182702|ref|YP_219119.1| putative outer membrane lipoprotein [Salmonella enterica subsp. enterica serovar Choleraesuis str. SC-B67]
>gi|62182704|ref|YP_219121.1| ssDNA-binding protein controls activity of RecBCD nuclease [Salmonella enterica subsp. enterica serovar Choleraesuis str. SC-B67]
>gi|62182776|ref|YP_219193.1| suppresses F exclusion of bacteriophage T7 [Salmonella enterica subsp. enterica serovar Choleraesuis str. SC-B67]
>gi|62182856|ref|YP_219273.1| putative cytoplasmic protein [Salmonella enterica subsp. enterica serovar Choleraesuis str. SC-B67]
>gi|62182940|ref|YP_219357.1| putative sugar transporter [Salmonella enterica subsp. enterica serovar Choleraesuis str. SC-B67]
>gi|62182948|ref|YP_219365.1| putative inner membrane protein [Salmonella enterica subsp. enterica serovar Choleraesuis str. SC-B67]
>gi|62182950|ref|YP_219367.1| transporter, LysE family [Salmonella enterica subsp. enterica serovar Choleraesuis str. SC-B67]
>gi|62182964|ref|YP_219381.1| putative outer membrane protein [Salmonella enterica subsp. enterica serovar Choleraesuis str. SC-B67]
>gi|62182980|ref|YP_219397.1| putative inner membrane protein [Salmonella enterica subsp. enterica serovar Choleraesuis str. SC-B67]
>gi|15603890|ref|NP_220405.1| UDP-3-O-[3-HYDROXYMYRISTOYL] GLUCOSAMINE N-ACYLTRANSFERASE (lpxD) [Rickettsia prowazekii str. Madrid E]
>gi|15603928|ref|NP_220443.1| CDP-DIACYLGLYCEROL--GLYCEROL-3-PHOSPHATE 3-PHOSPHATIDYLTRANSFERASE (pgsA) [Rickettsia prowazekii str. Madrid E]
>gi|15603960|ref|NP_220475.1| hypothetical protein RP082 [Rickettsia prowazekii str. Madrid E]
>gi|15603962|ref|NP_220477.1| hypothetical protein RP084 [Rickettsia prowazekii str. Madrid E]
>gi|15603976|ref|NP_220491.1| hypothetical protein RP098 [Rickettsia prowazekii str. Madrid E]
>gi|15604015|ref|NP_220530.1| 50S RIBOSOMAL PROTEIN L7/L12 (rplL) [Rickettsia prowazekii str. Madrid E]
>gi|15604044|ref|NP_220559.1| hypothetical protein RP169 [Rickettsia prowazekii str. Madrid E]
>gi|15604102|ref|NP_220617.1| hypothetical protein RP231 [Rickettsia prowazekii str. Madrid E]
>gi|15604125|ref|NP_220640.1| hypothetical protein RP255 [Rickettsia prowazekii str. Madrid E]
>gi|15604136|ref|NP_220651.1| hypothetical protein RP266 [Rickettsia prowazekii str. Madrid E]
>gi|15604180|ref|NP_220695.1| hypothetical protein RP311 [Rickettsia prowazekii str. Madrid E]
>gi|15604215|ref|NP_220730.1| OUTER MEMBRANE ASSEMBLY PROTEIN (asmA) [Rickettsia prowazekii str. Madrid E]
>gi|15604230|ref|NP_220746.1| hypothetical protein RP363 [Rickettsia prowazekii str. Madrid E]
>gi|15604235|ref|NP_220751.1| hypothetical protein RP368 [Rickettsia prowazekii str. Madrid E]
>gi|15604237|ref|NP_220753.1| hypothetical protein RP370 [Rickettsia prowazekii str. Madrid E]
>gi|15604257|ref|NP_220773.1| hypothetical protein RP392 [Rickettsia prowazekii str. Madrid E]
>gi|15604278|ref|NP_220794.1| hypothetical protein RP413 [Rickettsia prowazekii str. Madrid E]
>gi|15604350|ref|NP_220866.1| hypothetical protein RP489 [Rickettsia prowazekii str. Madrid E]
>gi|15604356|ref|NP_220872.1| hypothetical protein RP496 [Rickettsia prowazekii str. Madrid E]
>gi|15604417|ref|NP_220935.1| hypothetical protein RP563 [Rickettsia prowazekii str. Madrid E]
>gi|15604462|ref|NP_220980.1| hypothetical protein RP612 [Rickettsia prowazekii str. Madrid E]
>gi|15604470|ref|NP_220988.1| hypothetical protein RP621 [Rickettsia prowazekii str. Madrid E]
>gi|15604477|ref|NP_220995.1| PUTATIVE PERMEASE PERM HOMOLOG (perM) [Rickettsia prowazekii str. Madrid E]
>gi|15604497|ref|NP_221015.1| 50S RIBOSOMAL PROTEIN L29 (rpmC) [Rickettsia prowazekii str. Madrid E]
>gi|15604503|ref|NP_221021.1| 50S RIBOSOMAL PROTEIN L23 (rplW) [Rickettsia prowazekii str. Madrid E]
>gi|15604614|ref|NP_221132.1| hypothetical protein RP782 [Rickettsia prowazekii str. Madrid E]
>gi|15604664|ref|NP_221182.1| 17 KD SURFACE ANTIGEN PRECURSOR (omp) [Rickettsia prowazekii str. Madrid E]
>gi|16759006|ref|NP_454623.1| DnaJ protein [Salmonella enterica subsp. enterica serovar Typhi str. CT18]
>gi|16759150|ref|NP_454767.1| dihydrolipoamide acetyltransferase component (E2) of pyruvate dehydrogenase [Salmonella enterica subsp. enterica serovar Typhi str. CT18]
>gi|16759154|ref|NP_454771.1| hypothetical protein STY0180 [Salmonella enterica subsp. enterica serovar Typhi str. CT18]
>gi|16759157|ref|NP_454774.1| 2-keto-3-deoxygluconate permease [Salmonella enterica subsp. enterica serovar Typhi str. CT18]
>gi|16759192|ref|NP_454809.1| ferrichrome transport protein FhuB precursor [Salmonella enterica subsp. enterica serovar Typhi str. CT18]

>gi|16759216|ref|NP_454833.1| UDP-3-O-[3-hydroxymyristoyl] glucosamine N-acyltransferase [Salmonella enterica subsp. enterica serovar Typhi str. CT18]
>gi|16759251|ref|NP_454868.1| hypothetical protein STY0286 [Salmonella enterica subsp. enterica serovar Typhi str. CT18]
>gi|16759343|ref|NP_454960.1| RhtC-like transporter [Salmonella enterica subsp. enterica serovar Typhi str. CT18]
>gi|16759372|ref|NP_454989.1| exonuclease SbcC [Salmonella enterica subsp. enterica serovar Typhi str. CT18]
>gi|16759433|ref|NP_455050.1| hypothetical protein STY0495 [Salmonella enterica subsp. enterica serovar Typhi str. CT18]
>gi|16759452|ref|NP_455069.1| maltose O-acetyltransferase [Salmonella enterica subsp. enterica serovar Typhi str. CT18]
>gi|16759482|ref|NP_455099.1| thioredoxin-like protein [Salmonella enterica subsp. enterica serovar Typhi str. CT18]
>gi|16759486|ref|NP_455103.1| putative membrane protein [Salmonella enterica subsp. enterica serovar Typhi str. CT18]
>gi|16759542|ref|NP_455159.1| putative membrane protein [Salmonella enterica subsp. enterica serovar Typhi str. CT18]
>gi|16759551|ref|NP_455168.1| ferric enterobactin transport protein FepD [Salmonella enterica subsp. enterica serovar Typhi str. CT18]
>gi|16759552|ref|NP_455169.1| hypothetical membrane protein p43 [Salmonella enterica subsp. enterica serovar Typhi str. CT18]
>gi|16759573|ref|NP_455190.1| molybdopterin-containing oxidoreductase membrane anchor subunit [Salmonella enterica subsp. enterica serovar Typhi str. CT18]
>gi|16759580|ref|NP_455197.1| hypothetical protein STY0669 [Salmonella enterica subsp. enterica serovar Typhi str. CT18]
>gi|16759597|ref|NP_455214.1| rare lipoprotein A precursor [Salmonella enterica subsp. enterica serovar Typhi str. CT18]
>gi|16759647|ref|NP_455264.1| potassium-transporting ATPase C chain [Salmonella enterica subsp. enterica serovar Typhi str. CT18]
>gi|16759686|ref|NP_455303.1| tolA protein [Salmonella enterica subsp. enterica serovar Typhi str. CT18]
>gi|16759689|ref|NP_455306.1| hypothetical protein STY0796 [Salmonella enterica subsp. enterica serovar Typhi str. CT18]
>gi|16759693|ref|NP_455310.1| probable secreted protein [Salmonella enterica subsp. enterica serovar Typhi str. CT18]
>gi|16759695|ref|NP_455312.1| ABC transporter permease (FecCD_family) [Salmonella enterica subsp. enterica serovar Typhi str. CT18]
>gi|16759735|ref|NP_455352.1| putative membrane protein [Salmonella enterica subsp. enterica serovar Typhi str. CT18]
>gi|16759750|ref|NP_455367.1| putative membrane protein [Salmonella enterica subsp. enterica serovar Typhi str. CT18]
>gi|16759783|ref|NP_455400.1| putative membrane protein [Salmonella enterica subsp. enterica serovar Typhi str. CT18]
>gi|16759827|ref|NP_455444.1| transport ATP-binding protein CydC [Salmonella enterica subsp. enterica serovar Typhi str. CT18]
>gi|16759831|ref|NP_455448.1| cell division protein FtsK [Salmonella enterica subsp. enterica serovar Typhi str. CT18]
>gi|16759897|ref|NP_455514.1| hypothetical prophage protein [Salmonella enterica subsp. enterica serovar Typhi str. CT18]
>gi|16759900|ref|NP_455517.1| putative lipoprotein [Salmonella enterica subsp. enterica serovar Typhi str. CT18]
>gi|16759901|ref|NP_455518.1| putative prophage membrane protein [Salmonella enterica subsp. enterica serovar Typhi str. CT18]
>gi|16759904|ref|NP_455521.1| putative secreted protein [Salmonella enterica subsp. enterica serovar Typhi str. CT18]
>gi|16759914|ref|NP_455531.1| putative bacteriophage protein [Salmonella enterica subsp. enterica serovar Typhi str. CT18]
>gi|16759969|ref|NP_455586.1| hypothetical protein STY1117 [Salmonella enterica subsp. enterica serovar Typhi str. CT18]
>gi|16759989|ref|NP_455606.1| hypothetical protein STY1147 [Salmonella enterica subsp. enterica serovar Typhi str. CT18]
>gi|16759991|ref|NP_455608.1| membrane protein, suppressor for copper-sensitivity A [Salmonella enterica subsp. enterica serovar Typhi str. CT18]
>gi|16759998|ref|NP_455615.1| hypothetical protein STY1156 [Salmonella enterica subsp. enterica serovar Typhi str. CT18]
>gi|16760062|ref|NP_455679.1| ribonuclease E [Salmonella enterica subsp. enterica serovar Typhi str. CT18]
>gi|16760082|ref|NP_455699.1| putative lipoprotein [Salmonella enterica subsp. enterica serovar Typhi str. CT18]
>gi|16760132|ref|NP_455749.1| DNA-binding protein (histone-like protein Hlp-II) [Salmonella enterica subsp. enterica serovar Typhi str. CT18]
>gi|16760144|ref|NP_455761.1| TonB protein [Salmonella enterica subsp. enterica serovar Typhi str. CT18]
>gi|16760147|ref|NP_455764.1| putative membrane protein [Salmonella enterica subsp. enterica serovar Typhi str. CT18]
>gi|16760153|ref|NP_455770.1| hypothetical protein STY1323 [Salmonella enterica subsp. enterica serovar Typhi str. CT18]
>gi|16760175|ref|NP_455792.1| osmotically inducible lipoprotein B precursor [Salmonella enterica subsp. enterica serovar Typhi str. CT18]
>gi|16760228|ref|NP_455845.1| O6-methylguanine-DNA-alkyltransferase [Salmonella enterica subsp. enterica serovar Typhi str. CT18]
>gi|16760234|ref|NP_455851.1| putative multidrug transporter [Salmonella enterica subsp. enterica serovar Typhi str. CT18]
>gi|16760279|ref|NP_455896.1| putative membrane protein [Salmonella enterica subsp. enterica serovar Typhi str. CT18]
>gi|16760286|ref|NP_455903.1| putative virulence effector protein [Salmonella enterica subsp. enterica serovar Typhi str. CT18]
>gi|16760319|ref|NP_455936.1| putative transport protein (pseudogene) [Salmonella enterica subsp. enterica serovar Typhi str. CT18]
>gi|16760346|ref|NP_455963.1| putative membrane protein [Salmonella enterica subsp. enterica serovar Typhi str. CT18]
>gi|16760350|ref|NP_455967.1| hypothetical protein STY1548 [Salmonella enterica subsp. enterica serovar Typhi str. CT18]
>gi|16760359|ref|NP_455976.1| putative membrane protein [Salmonella enterica subsp. enterica serovar Typhi str. CT18]
>gi|16760378|ref|NP_455995.1| putative secreted stress response protein [Salmonella enterica subsp. enterica serovar Typhi str. CT18]
>gi|16760415|ref|NP_456032.1| putative membrane protein [Salmonella enterica subsp. enterica serovar Typhi str. CT18]
>gi|16760453|ref|NP_456070.1| putative membrane protein [Salmonella enterica subsp. enterica serovar Typhi str. CT18]
>gi|16760456|ref|NP_456073.1| putative NADH reducing dehydrogenase [Salmonella enterica subsp. enterica serovar Typhi str. CT18]
>gi|16760459|ref|NP_456076.1| hypothetical protein STY1668 [Salmonella enterica subsp. enterica serovar Typhi str. CT18]
>gi|16760468|ref|NP_456085.1| outer membrane lipoprotein SlyB precursor [Salmonella enterica subsp. enterica serovar Typhi str. CT18]
>gi|16760481|ref|NP_456098.1| putative secreted protein [Salmonella enterica subsp. enterica serovar Typhi str. CT18]
>gi|16760510|ref|NP_456127.1| putative pathogenicity island effector protein [Salmonella enterica subsp. enterica serovar Typhi str. CT18]
>gi|16760531|ref|NP_456148.1| major outer membrane lipoprotein [Salmonella enterica subsp. enterica serovar Typhi str. CT18]
>gi|16760532|ref|NP_456149.1| major outer membrane lipoprotein [Salmonella enterica subsp. enterica serovar Typhi str. CT18]
>gi|16760540|ref|NP_456157.1| hypothetical protein STY1756 [Salmonella enterica subsp. enterica serovar Typhi str. CT18]
>gi|16760543|ref|NP_456160.1| putative membrane protein [Salmonella enterica subsp. enterica serovar Typhi str. CT18]
>gi|16760554|ref|NP_456171.1| vitamin B12 transport system permease [Salmonella enterica subsp. enterica serovar Typhi str. CT18]
>gi|16760560|ref|NP_456177.1| 50S ribosomal subunit protein L35 [Salmonella enterica subsp. enterica serovar Typhi str. CT18]
>gi|16760614|ref|NP_456231.1| putative membrane protein [Salmonella enterica subsp. enterica serovar Typhi str. CT18]
>gi|16760619|ref|NP_456236.1| putative membrane protein [Salmonella enterica subsp. enterica serovar Typhi str. CT18]
>gi|16760622|ref|NP_456239.1| hypothetical protein STY1849 [Salmonella enterica subsp. enterica serovar Typhi str. CT18]
>gi|16760632|ref|NP_456249.1| hypothetical protein STY1859 [Salmonella enterica subsp. enterica serovar Typhi str. CT18]
>gi|16760687|ref|NP_456304.1| putative membrane protein [Salmonella enterica subsp. enterica serovar Typhi str. CT18]
>gi|16760700|ref|NP_456317.1| hypothetical protein STY1940 [Salmonella enterica subsp. enterica serovar Typhi str. CT18]

>gi|16760713|ref|NP_456330.1| hypothetical protein STY1953 [Salmonella enterica subsp. enterica serovar Typhi str. CT18]
>gi|16760720|ref|NP_456337.1| phosphotransferase enzyme II, C component [Salmonella enterica subsp. enterica serovar Typhi str. CT18]
>gi|16760728|ref|NP_456345.1| hypothetical protein STY1969 [Salmonella enterica subsp. enterica serovar Typhi str. CT18]
>gi|16760734|ref|NP_456351.1| ProP effector [Salmonella enterica subsp. enterica serovar Typhi str. CT18]
>gi|16760775|ref|NP_456392.1| putative bacteriophage protein [Salmonella enterica subsp. enterica serovar Typhi str. CT18]
>gi|16760777|ref|NP_456394.1| putative bacteriophage protein [Salmonella enterica subsp. enterica serovar Typhi str. CT18]
>gi|16760788|ref|NP_456405.1| putative bacteriophage protein [Salmonella enterica subsp. enterica serovar Typhi str. CT18]
>gi|16760831|ref|NP_456448.1| hypothetical protein STY2086 [Salmonella enterica subsp. enterica serovar Typhi str. CT18]
>gi|16760862|ref|NP_456479.1| flagellar biosynthesis protein FlhA [Salmonella enterica subsp. enterica serovar Typhi str. CT18]
>gi|16760884|ref|NP_456501.1| tyrosine-specific transport protein [Salmonella enterica subsp. enterica serovar Typhi str. CT18]
>gi|16760915|ref|NP_456532.1| flagellar assembly protein FliH [Salmonella enterica subsp. enterica serovar Typhi str. CT18]
>gi|16760918|ref|NP_456535.1| flagellar hook-length control protein [Salmonella enterica subsp. enterica serovar Typhi str. CT18]
>gi|16760925|ref|NP_456542.1| flagellar biosynthetic protein FliR [Salmonella enterica subsp. enterica serovar Typhi str. CT18]
>gi|16760954|ref|NP_456571.1| cobalamin (5'-phosphate) synthase [Salmonella enterica subsp. enterica serovar Typhi str. CT18]
>gi|16760978|ref|NP_456595.1| putative propanediol utilization protein PduJ [Salmonella enterica subsp. enterica serovar Typhi str. CT18]
>gi|16760979|ref|NP_456596.1| putative propanediol utilization protein PduK [Salmonella enterica subsp. enterica serovar Typhi str. CT18]
>gi|16761055|ref|NP_456672.1| putative efflux system protein [Salmonella enterica subsp. enterica serovar Typhi str. CT18]
>gi|16761081|ref|NP_456698.1| putative membrane protein [Salmonella enterica subsp. enterica serovar Typhi str. CT18]
>gi|16761105|ref|NP_456722.1| putative permease transmembrane component [Salmonella enterica subsp. enterica serovar Typhi str. CT18]
>gi|16761166|ref|NP_456783.1| putative membrane protein [Salmonella enterica subsp. enterica serovar Typhi str. CT18]
>gi|16761170|ref|NP_456787.1| secreted effector protein [Salmonella enterica subsp. enterica serovar Typhi str. CT18]
>gi|16761179|ref|NP_456796.1| heme exporter protein D1 [Salmonella enterica subsp. enterica serovar Typhi str. CT18]
>gi|16761181|ref|NP_456798.1| heme exporter protein B1 [Salmonella enterica subsp. enterica serovar Typhi str. CT18]
>gi|16761289|ref|NP_456906.1| DedD protein [Salmonella enterica subsp. enterica serovar Typhi str. CT18]
>gi|16761333|ref|NP_456950.1| hypothetical protein STY2648 [Salmonella enterica subsp. enterica serovar Typhi str. CT18]
>gi|16761348|ref|NP_456965.1| cell division protein [Salmonella enterica subsp. enterica serovar Typhi str. CT18]
>gi|16761366|ref|NP_456983.1| hypothetical protein STY2685 [Salmonella enterica subsp. enterica serovar Typhi str. CT18]
>gi|16761370|ref|NP_456987.1| putative membrane protein [Salmonella enterica subsp. enterica serovar Typhi str. CT18]
>gi|16761410|ref|NP_457027.1| putative permease [Salmonella enterica subsp. enterica serovar Typhi str. CT18]
>gi|16761424|ref|NP_457041.1| hypothetical protein STY2748 [Salmonella enterica subsp. enterica serovar Typhi str. CT18]
>gi|16761439|ref|NP_457056.1| putative DNA-binding protein [Salmonella enterica subsp. enterica serovar Typhi str. CT18]
>gi|16761496|ref|NP_457113.1| sigma-E factor regulatory protein RseC [Salmonella enterica subsp. enterica serovar Typhi str. CT18]
>gi|16761550|ref|NP_457167.1| putative bacteriophage tail protein [Salmonella enterica subsp. enterica serovar Typhi str. CT18]
>gi|16761593|ref|NP_457210.1| putative transmembrane transport protein [Salmonella enterica subsp. enterica serovar Typhi str. CT18]
>gi|16761609|ref|NP_457226.1| PTS system, glucitol/sorbitol-specific IIBC component [Salmonella enterica subsp. enterica serovar Typhi str. CT18]
>gi|16761628|ref|NP_457245.1| formate hydrogenlyase subunit 3 [Salmonella enterica subsp. enterica serovar Typhi str. CT18]
>gi|16761662|ref|NP_457279.1| pathogenicity island 1 effector protein [Salmonella enterica subsp. enterica serovar Typhi str. CT18]
>gi|16761697|ref|NP_457314.1| lipoprotein NlpD precursor [Salmonella enterica subsp. enterica serovar Typhi str. CT18]
>gi|16761704|ref|NP_457321.1| hypothetical protein STY3057 [Salmonella enterica subsp. enterica serovar Typhi str. CT18]
>gi|16761734|ref|NP_457351.1| hypothetical protein STY3093 [Salmonella enterica subsp. enterica serovar Typhi str. CT18]
>gi|16761854|ref|NP_457471.1| putative membrane protein [Salmonella enterica subsp. enterica serovar Typhi str. CT18]
>gi|16761930|ref|NP_457547.1| putative membrane protein [Salmonella enterica subsp. enterica serovar Typhi str. CT18]
>gi|16761973|ref|NP_457590.1| hypothetical protein STY3378 [Salmonella enterica subsp. enterica serovar Typhi str. CT18]
>gi|16762004|ref|NP_457621.1| putative membrane protein [Salmonella enterica subsp. enterica serovar Typhi str. CT18]
>gi|16762045|ref|NP_457662.1| ATP-dependent RNA helicase (dead-box protein) [Salmonella enterica subsp. enterica serovar Typhi str. CT18]
>gi|16762051|ref|NP_457668.1| protein chain initiation factor 2 [Salmonella enterica subsp. enterica serovar Typhi str. CT18]
>gi|16762055|ref|NP_457672.1| protein-export membrane protein [Salmonella enterica subsp. enterica serovar Typhi str. CT18]
>gi|16762132|ref|NP_457749.1| rod shape-determining protein [Salmonella enterica subsp. enterica serovar Typhi str. CT18]
>gi|16762137|ref|NP_457754.1| putative membrane protein [Salmonella enterica subsp. enterica serovar Typhi str. CT18]
>gi|16762138|ref|NP_457755.1| biotin carboxyl carrier protein [Salmonella enterica subsp. enterica serovar Typhi str. CT18]
>gi|16762160|ref|NP_457777.1| sec-independent protein translocase protein [Salmonella enterica subsp. enterica serovar Typhi str. CT18]
>gi|16762161|ref|NP_457778.1| sec-independent protein translocase protein [Salmonella enterica subsp. enterica serovar Typhi str. CT18]
>gi|16762181|ref|NP_457798.1| putative membrane protein [Salmonella enterica subsp. enterica serovar Typhi str. CT18]
>gi|16762198|ref|NP_457815.1| uroporphyrinogen III methylase [Salmonella enterica subsp. enterica serovar Typhi str. CT18]
>gi|16762258|ref|NP_457875.1| putative regulatory protein [Salmonella enterica subsp. enterica serovar Typhi str. CT18]
>gi|16762274|ref|NP_457891.1| hypothetical protein STY3700 [Salmonella enterica subsp. enterica serovar Typhi str. CT18]
>gi|16762301|ref|NP_457918.1| 50S ribosomal subunit protein L7/L12 [Salmonella enterica subsp. enterica serovar Typhi str. CT18]
>gi|16762342|ref|NP_457959.1| cell division protein [Salmonella enterica subsp. enterica serovar Typhi str. CT18]
>gi|16762359|ref|NP_457976.1| putative ABC transporter, membrane component [Salmonella enterica subsp. enterica serovar Typhi str. CT18]
>gi|16762429|ref|NP_458046.1| hypothetical protein STY3878 [Salmonella enterica subsp. enterica serovar Typhi str. CT18]
>gi|16762443|ref|NP_458060.1| high affinity ribose transport protein [Salmonella enterica subsp. enterica serovar Typhi str. CT18]
>gi|16762454|ref|NP_458071.1| ATP synthase protein I. [Salmonella enterica subsp. enterica serovar Typhi str. CT18]
>gi|16762456|ref|NP_458073.1| ATP synthase subunit C [Salmonella enterica subsp. enterica serovar Typhi str. CT18]
>gi|16762457|ref|NP_458074.1| ATP synthase subunit B [Salmonella enterica subsp. enterica serovar Typhi str. CT18]
>gi|16762471|ref|NP_458088.1| probable PTS system permease [Salmonella enterica subsp. enterica serovar Typhi str. CT18]
>gi|16762504|ref|NP_458121.1| heme exporter protein B2 [Salmonella enterica subsp. enterica serovar Typhi str. CT18]
>gi|16762506|ref|NP_458123.1| heme exporter protein D2 [Salmonella enterica subsp. enterica serovar Typhi str. CT18]
>gi|16762531|ref|NP_458148.1| two-component system sensor histidine kinase [Salmonella enterica subsp. enterica serovar Typhi str. CT18]
>gi|16762565|ref|NP_458182.1| glutamate permease [Salmonella enterica subsp. enterica serovar Typhi str. CT18]
>gi|16762577|ref|NP_458194.1| putative TetR-family transcriptional regulator [Salmonella enterica subsp. enterica serovar Typhi str. CT18]

>gi|16762618|ref|NP_458235.1| putative autotransporter [Salmonella enterica subsp. enterica serovar Typhi str. CT18]
>gi|16762619|ref|NP_458236.1| putative lipoprotein [Salmonella enterica subsp. enterica serovar Typhi str. CT18]
>gi|16762621|ref|NP_458238.1| hypothetical protein STY4108 [Salmonella enterica subsp. enterica serovar Typhi str. CT18]
>gi|16762663|ref|NP_458280.1| putative outer membrane protein [Salmonella enterica subsp. enterica serovar Typhi str. CT18]
>gi|16762674|ref|NP_458291.1| dipeptide transport system permease protein DppC [Salmonella enterica subsp. enterica serovar Typhi str. CT18]
>gi|16762729|ref|NP_458346.1| putative membrane protein [Salmonella enterica subsp. enterica serovar Typhi str. CT18]
>gi|16762733|ref|NP_458350.1| cell division protein [Salmonella enterica subsp. enterica serovar Typhi str. CT18]
>gi|16762750|ref|NP_458367.1| hypothetical protein STY4259 [Salmonella enterica subsp. enterica serovar Typhi str. CT18]
>gi|16762788|ref|NP_458405.1| putative membrane protein [Salmonella enterica subsp. enterica serovar Typhi str. CT18]
>gi|16762796|ref|NP_458413.1| putative membrane protein [Salmonella enterica subsp. enterica serovar Typhi str. CT18]
>gi|16762802|ref|NP_458419.1| DamX protein [Salmonella enterica subsp. enterica serovar Typhi str. CT18]
>gi|16762828|ref|NP_458445.1| FKBP-type peptidyl-prolyl cis-trans isomerase [Salmonella enterica subsp. enterica serovar Typhi str. CT18]
>gi|16762834|ref|NP_458451.1| hypothetical protein STY4349 [Salmonella enterica subsp. enterica serovar Typhi str. CT18]
>gi|16762851|ref|NP_458468.1| 50S ribosomal subunit protein L29 [Salmonella enterica subsp. enterica serovar Typhi str. CT18]
>gi|16762859|ref|NP_458476.1| 50S ribosomal subunit protein L18 [Salmonella enterica subsp. enterica serovar Typhi str. CT18]
>gi|16762862|ref|NP_458479.1| 50S ribosomal subunit protein L15 [Salmonella enterica subsp. enterica serovar Typhi str. CT18]
>gi|16762900|ref|NP_458517.1| putative membrane protein [Salmonella enterica subsp. enterica serovar Typhi str. CT18]
>gi|16762903|ref|NP_458520.1| hypothetical protein STY4418 [Salmonella enterica subsp. enterica serovar Typhi str. CT18]
>gi|16762925|ref|NP_458542.1| putative membrane protein [Salmonella enterica subsp. enterica serovar Typhi str. CT18]
>gi|16762936|ref|NP_458553.1| single-strand DNA-binding protein [Salmonella enterica subsp. enterica serovar Typhi str. CT18]
>gi|16763035|ref|NP_458652.1| putative membrane protein [Salmonella enterica subsp. enterica serovar Typhi str. CT18]
>gi|16763129|ref|NP_458746.1| hypothetical protein STY4667 [Salmonella enterica subsp. enterica serovar Typhi str. CT18]
>gi|16763139|ref|NP_458756.1| putative membrane protein [Salmonella enterica subsp. enterica serovar Typhi str. CT18]
>gi|16763149|ref|NP_458766.1| FxsA protein [Salmonella enterica subsp. enterica serovar Typhi str. CT18]
>gi|16763150|ref|NP_458767.1| putative permease [Salmonella enterica subsp. enterica serovar Typhi str. CT18]
>gi|16763182|ref|NP_458799.1| HflK protein [Salmonella enterica subsp. enterica serovar Typhi str. CT18]
>gi|16763213|ref|NP_458830.1| hypothetical protein STY4752 [Salmonella enterica subsp. enterica serovar Typhi str. CT18]
>gi|16763231|ref|NP_458848.1| hypothetical protein STY4770 [Salmonella enterica subsp. enterica serovar Typhi str. CT18]
>gi|16763329|ref|NP_458946.1| putative membrane protein [Salmonella enterica subsp. enterica serovar Typhi str. CT18]
>gi|16763340|ref|NP_458957.1| putative secreted protein [Salmonella enterica subsp. enterica serovar Typhi str. CT18]
>gi|16763346|ref|NP_458963.1| conserved hypothetical regulatory protein [Salmonella enterica subsp. enterica serovar Typhi str. CT18]
>gi|16763357|ref|NP_458974.1| hypothetical protein STY4912 [Salmonella enterica subsp. enterica serovar Typhi str. CT18]
>gi|18466438|ref|NP_569246.1| putative membrane protein [Salmonella enterica subsp. enterica serovar Typhi str. CT18]
>gi|18466553|ref|NP_569361.1| putative mercuric transport protein [Salmonella enterica subsp. enterica serovar Typhi str. CT18]
>gi|18466557|ref|NP_569365.1| putative mercuric resistance operon coregulator [Salmonella enterica subsp. enterica serovar Typhi str. CT18]
>gi|18466558|ref|NP_569366.1| hypothetical protein HCM1.160 [Salmonella enterica subsp. enterica serovar Typhi str. CT18]
>gi|18466577|ref|NP_569385.1| hypothetical protein HCM1.183 [Salmonella enterica subsp. enterica serovar Typhi str. CT18]
>gi|18466611|ref|NP_569419.1| hypothetical protein HCM1.229c [Salmonella enterica subsp. enterica serovar Typhi str. CT18]
>gi|18466612|ref|NP_569420.1| putative mercuric resistance operon coregulator [Salmonella enterica subsp. enterica serovar Typhi str. CT18]
>gi|18466615|ref|NP_569423.1| putative mercuric transport protein periplasmic binding protein [Salmonella enterica subsp. enterica serovar Typhi str. CT18]
>gi|18466616|ref|NP_569424.1| putative mercuric transport protein [Salmonella enterica subsp. enterica serovar Typhi str. CT18]
>gi|18466654|ref|NP_569462.1| putative membrane protein [Salmonella enterica subsp. enterica serovar Typhi str. CT18]
>gi|18466672|ref|NP_569479.1| putative membrane protein [Salmonella enterica subsp. enterica serovar Typhi str. CT18]
>gi|18466676|ref|NP_569483.1| hypothetical protein HCM2.0011c [Salmonella enterica subsp. enterica serovar Typhi str. CT18]
>gi|18466731|ref|NP_569538.1| hypothetical protein HCM2.0066c [Salmonella enterica subsp. enterica serovar Typhi str. CT18]
>gi|18466733|ref|NP_569540.1| hypothetical protein HCM2.0068c [Salmonella enterica subsp. enterica serovar Typhi str. CT18]
>gi|29899129|ref|NP_829906.1| hypothetical cytosolic protein [Bacillus cereus ATCC 14579]
>gi|30018330|ref|NP_829961.1| Spore cortex biosynthesis protein [Bacillus cereus ATCC 14579]
>gi|30018365|ref|NP_829996.1| Protein translocase subunit SecE [Bacillus cereus ATCC 14579]
>gi|30018370|ref|NP_830001.1| LSU ribosomal protein L12P (L7/L12) [Bacillus cereus ATCC 14579]
>gi|30018420|ref|NP_830051.1| hypothetical protein BC0172 [Bacillus cereus ATCC 14579]
>gi|30018433|ref|NP_830064.1| ABC transporter permease protein [Bacillus cereus ATCC 14579]
>gi|30018449|ref|NP_830080.1| hypothetical protein BC0212 [Bacillus cereus ATCC 14579]
>gi|30018477|ref|NP_830108.1| hypothetical protein BC0240 [Bacillus cereus ATCC 14579]
>gi|30018544|ref|NP_830175.1| Somatin-like protein [Bacillus cereus ATCC 14579]
>gi|30018590|ref|NP_830221.1| Ferrichrome transport system permease protein fhuB [Bacillus cereus ATCC 14579]
>gi|30018592|ref|NP_830223.1| hypothetical protein BC0384 [Bacillus cereus ATCC 14579]
>gi|30018640|ref|NP_830271.1| hypothetical protein BC0432 [Bacillus cereus ATCC 14579]
>gi|30018648|ref|NP_830279.1| surface protein [Bacillus cereus ATCC 14579]
>gi|30018653|ref|NP_830284.1| TerC-like protein [Bacillus cereus ATCC 14579]
>gi|30018712|ref|NP_830343.1| Small, acid-soluble spore protein gamma-type [Bacillus cereus ATCC 14579]
>gi|30018740|ref|NP_830371.1| Internalin protein [Bacillus cereus ATCC 14579]
>gi|30018744|ref|NP_830375.1| Flottilin [Bacillus cereus ATCC 14579]
>gi|30018760|ref|NP_830391.1| hypothetical Membrane Spanning Protein [Bacillus cereus ATCC 14579]
>gi|30018812|ref|NP_830443.1| hypothetical protein BC0627 [Bacillus cereus ATCC 14579]
>gi|30018861|ref|NP_830492.1| Cell wall-binding protein [Bacillus cereus ATCC 14579]
>gi|30018865|ref|NP_830496.1| hypothetical protein BC0683 [Bacillus cereus ATCC 14579]
>gi|30018925|ref|NP_830556.1| Methylthioribose transport system permease protein [Bacillus cereus ATCC 14579]
>gi|30018972|ref|NP_830603.1| enterotoxin / cell-wall binding protein [Bacillus cereus ATCC 14579]

>gi|30018989|ref|NP_830620.1| hypothetical protein BC0830 [Bacillus cereus ATCC 14579]
>gi|30018999|ref|NP_830630.1| hypothetical Membrane Spanning Protein [Bacillus cereus ATCC 14579]
>gi|30019087|ref|NP_830718.1| hypothetical protein BC0932 [Bacillus cereus ATCC 14579]
>gi|30019109|ref|NP_830740.1| tcdA-E operon negative regulator [Bacillus cereus ATCC 14579]
>gi|30019120|ref|NP_830751.1| hypothetical protein BC0965 [Bacillus cereus ATCC 14579]
>gi|30019125|ref|NP_830756.1| hypothetical protein BC0970 [Bacillus cereus ATCC 14579]
>gi|30019147|ref|NP_830778.1| hypothetical protein BC0992 [Bacillus cereus ATCC 14579]
>gi|30019154|ref|NP_830785.1| hypothetical protein BC0999 [Bacillus cereus ATCC 14579]
>gi|30019165|ref|NP_830796.1| hypothetical protein BC1010 [Bacillus cereus ATCC 14579]
>gi|30019168|ref|NP_830799.1| hypothetical protein BC1013 [Bacillus cereus ATCC 14579]
>gi|30019185|ref|NP_830816.1| hypothetical protein BC1030 [Bacillus cereus ATCC 14579]
>gi|30019201|ref|NP_830832.1| hypothetical protein BC1046 [Bacillus cereus ATCC 14579]
>gi|30019233|ref|NP_830864.1| Phage infection protein [Bacillus cereus ATCC 14579]
>gi|30019303|ref|NP_830934.1| hypothetical protein BC1148 [Bacillus cereus ATCC 14579]
>gi|30019332|ref|NP_830963.1| hypothetical protein BC1178 [Bacillus cereus ATCC 14579]
>gi|30019343|ref|NP_830974.1| Integral membrane protein [Bacillus cereus ATCC 14579]
>gi|30019370|ref|NP_831001.1| hypothetical protein BC1217 [Bacillus cereus ATCC 14579]
>gi|30019373|ref|NP_831004.1| hypothetical protein BC1220 [Bacillus cereus ATCC 14579]
>gi|30019392|ref|NP_831023.1| hypothetical Membrane Spanning Protein [Bacillus cereus ATCC 14579]
>gi|30019404|ref|NP_831035.1| Dihydrolipoamide succinyltransferase component (E2) of 2-oxoglutarate dehydrogenase complex [Bacillus cereus ATCC 14579]
>gi|30019431|ref|NP_831062.1| hypothetical protein BC1280 [Bacillus cereus ATCC 14579]
>gi|30019445|ref|NP_831076.1| hypothetical Membrane Spanning Protein [Bacillus cereus ATCC 14579]
>gi|30019446|ref|NP_831077.1| hypothetical protein BC1295 [Bacillus cereus ATCC 14579]
>gi|30019536|ref|NP_831167.1| hypothetical protein BC1386 [Bacillus cereus ATCC 14579]
>gi|30019538|ref|NP_831169.1| hypothetical protein BC1388 [Bacillus cereus ATCC 14579]
>gi|30019541|ref|NP_831172.1| hypothetical protein BC1391 [Bacillus cereus ATCC 14579]
>gi|30019547|ref|NP_831178.1| Acetolactate synthase small subunit [Bacillus cereus ATCC 14579]
>gi|30019580|ref|NP_831211.1| Cell wall endopeptidase, family M23/M37 [Bacillus cereus ATCC 14579]
>gi|30019697|ref|NP_831328.1| Multimodular transpeptidase-transglycosylase PBP 1A [Bacillus cereus ATCC 14579]
>gi|30019706|ref|NP_831337.1| Spore coat protein [Bacillus cereus ATCC 14579]
>gi|30019707|ref|NP_831338.1| Spore coat protein D [Bacillus cereus ATCC 14579]
>gi|30019725|ref|NP_831356.1| hypothetical protein BC1578 [Bacillus cereus ATCC 14579]
>gi|30019730|ref|NP_831361.1| O-acetyl transferase [Bacillus cereus ATCC 14579]
>gi|30019731|ref|NP_831362.1| Capsular polysaccharide protein CpsC [Bacillus cereus ATCC 14579]
>gi|30019777|ref|NP_831408.1| hypothetical protein BC1630 [Bacillus cereus ATCC 14579]
>gi|30019786|ref|NP_831417.1| Flagellar protein fliS [Bacillus cereus ATCC 14579]
>gi|30019791|ref|NP_831422.1| Flagellar M-ring protein fliF [Bacillus cereus ATCC 14579]
>gi|30019793|ref|NP_831424.1| hypothetical protein BC1646 [Bacillus cereus ATCC 14579]
>gi|30019820|ref|NP_831451.1| hypothetical protein BC1676 [Bacillus cereus ATCC 14579]
>gi|30019826|ref|NP_831457.1| hypothetical protein BC1682 [Bacillus cereus ATCC 14579]
>gi|30019839|ref|NP_831470.1| Transcriptional regulator, MarR family [Bacillus cereus ATCC 14579]
>gi|30019973|ref|NP_831604.1| hypothetical protein BC1831 [Bacillus cereus ATCC 14579]
>gi|30019996|ref|NP_831627.1| hypothetical Cytosolic Protein [Bacillus cereus ATCC 14579]
>gi|30020035|ref|NP_831666.1| Scaffold protein [Bacillus cereus ATCC 14579]
>gi|30020047|ref|NP_831678.1| Phage protein [Bacillus cereus ATCC 14579]
>gi|30020049|ref|NP_831680.1| XpaF1 protein [Bacillus cereus ATCC 14579]
>gi|30020092|ref|NP_831723.1| Enterotoxin [Bacillus cereus ATCC 14579]
>gi|30020114|ref|NP_831745.1| hypothetical protein BC1975 [Bacillus cereus ATCC 14579]
>gi|30020145|ref|NP_831776.1| hypothetical protein BC2008 [Bacillus cereus ATCC 14579]
>gi|30020167|ref|NP_831798.1| Spore coat protein G [Bacillus cereus ATCC 14579]
>gi|30020194|ref|NP_831825.1| Stomatin like protein [Bacillus cereus ATCC 14579]
>gi|30020198|ref|NP_831829.1| Multidrug resistance protein B [Bacillus cereus ATCC 14579]
>gi|30020216|ref|NP_831847.1| hypothetical Membrane Associated Protein [Bacillus cereus ATCC 14579]
>gi|30020277|ref|NP_831908.1| Stage V sporulation protein S [Bacillus cereus ATCC 14579]
>gi|30020284|ref|NP_831915.1| hypothetical protein BC2149 [Bacillus cereus ATCC 14579]
>gi|30020356|ref|NP_831987.1| Oligopeptide transport system permease protein oppB [Bacillus cereus ATCC 14579]
>gi|30020389|ref|NP_832020.1| hypothetical protein BC2257 [Bacillus cereus ATCC 14579]
>gi|30020397|ref|NP_832028.1| hypothetical protein BC2265 [Bacillus cereus ATCC 14579]
>gi|30020413|ref|NP_832044.1| Multimodular transpeptidase-transglycosylase PBP 1A [Bacillus cereus ATCC 14579]
>gi|30020465|ref|NP_832096.1| hypothetical protein BC2333 [Bacillus cereus ATCC 14579]
>gi|30020475|ref|NP_832106.1| hypothetical protein BC2344 [Bacillus cereus ATCC 14579]
>gi|30020507|ref|NP_832138.1| hypothetical protein BC2376 [Bacillus cereus ATCC 14579]
>gi|30020511|ref|NP_832142.1| hypothetical Membrane Spanning Protein [Bacillus cereus ATCC 14579]
>gi|30020512|ref|NP_832143.1| Collagen triple helix repeat protein [Bacillus cereus ATCC 14579]
>gi|30020513|ref|NP_832144.1| hypothetical protein BC2382 [Bacillus cereus ATCC 14579]
>gi|30020529|ref|NP_832160.1| putative integral membrane protein [Bacillus cereus ATCC 14579]
>gi|30020546|ref|NP_832177.1| Collagen triple helix repeat protein [Bacillus cereus ATCC 14579]
>gi|30020550|ref|NP_832181.1| Phage protein [Bacillus cereus ATCC 14579]
>gi|30020610|ref|NP_832241.1| hypothetical protein BC2481 [Bacillus cereus ATCC 14579]

>gi|30020620|ref|NP_832251.1| hypothetical protein BC2492 [Bacillus cereus ATCC 14579]
>gi|30020659|ref|NP_832290.1| hypothetical Membrane Associated Protein [Bacillus cereus ATCC 14579]
>gi|30020680|ref|NP_832311.1| hypothetical protein BC2553 [Bacillus cereus ATCC 14579]
>gi|30020696|ref|NP_832327.1| Collagen triple helix repeat protein [Bacillus cereus ATCC 14579]
>gi|30020697|ref|NP_832328.1| Collagen triple helix repeat protein [Bacillus cereus ATCC 14579]
>gi|30020700|ref|NP_832331.1| Phage protein [Bacillus cereus ATCC 14579]
>gi|30020703|ref|NP_832334.1| hypothetical protein BC2576 [Bacillus cereus ATCC 14579]
>gi|30020728|ref|NP_832359.1| hypothetical protein BC2601 [Bacillus cereus ATCC 14579]
>gi|30020762|ref|NP_832393.1| Collagen triple helix repeat protein [Bacillus cereus ATCC 14579]
>gi|30020779|ref|NP_832410.1| hypothetical protein BC2653 [Bacillus cereus ATCC 14579]
>gi|30020811|ref|NP_832442.1| hypothetical protein BC2687 [Bacillus cereus ATCC 14579]
>gi|30020909|ref|NP_832540.1| hypothetical protein BC2788 [Bacillus cereus ATCC 14579]
>gi|30020961|ref|NP_832592.1| hypothetical protein BC2842 [Bacillus cereus ATCC 14579]
>gi|30020975|ref|NP_832606.1| hypothetical protein BC2857 [Bacillus cereus ATCC 14579]
>gi|30020989|ref|NP_832620.1| Spore coat protein X [Bacillus cereus ATCC 14579]
>gi|30020991|ref|NP_832622.1| Spore coat protein X [Bacillus cereus ATCC 14579]
>gi|30021005|ref|NP_832636.1| Integral membrane protein [Bacillus cereus ATCC 14579]
>gi|30021068|ref|NP_832699.1| enterotoxin / cell-wall binding protein [Bacillus cereus ATCC 14579]
>gi|30021077|ref|NP_832708.1| Sugar transport system permease protein [Bacillus cereus ATCC 14579]
>gi|30021104|ref|NP_832735.1| hypothetical protein BC2989 [Bacillus cereus ATCC 14579]
>gi|30021117|ref|NP_832748.1| hypothetical protein BC3002 [Bacillus cereus ATCC 14579]
>gi|30021255|ref|NP_832886.1| hypothetical protein BC3145 [Bacillus cereus ATCC 14579]
>gi|30021280|ref|NP_832911.1| Transposase [Bacillus cereus ATCC 14579]
>gi|30021296|ref|NP_832927.1| hypothetical protein BC3186 [Bacillus cereus ATCC 14579]
>gi|30021331|ref|NP_832962.1| surface protein [Bacillus cereus ATCC 14579]
>gi|30021430|ref|NP_833061.1| hypothetical protein BC3322 [Bacillus cereus ATCC 14579]
>gi|30021434|ref|NP_833065.1| hypothetical protein BC3326 [Bacillus cereus ATCC 14579]
>gi|30021451|ref|NP_833082.1| Integral membrane protein [Bacillus cereus ATCC 14579]
>gi|30021453|ref|NP_833084.1| Collagen-like triple helix repeat protein [Bacillus cereus ATCC 14579]
>gi|30021454|ref|NP_833085.1| Collagen-like triple helix repeat protein [Bacillus cereus ATCC 14579]
>gi|30021474|ref|NP_833105.1| oxetanocin A resistance protein [Bacillus cereus ATCC 14579]
>gi|30021528|ref|NP_833159.1| hypothetical Membrane Associated Protein [Bacillus cereus ATCC 14579]
>gi|30021535|ref|NP_833166.1| hypothetical Membrane Spanning Protein [Bacillus cereus ATCC 14579]
>gi|30021546|ref|NP_833177.1| hypothetical Membrane Spanning Protein [Bacillus cereus ATCC 14579]
>gi|30021560|ref|NP_833191.1| hypothetical protein BC3457 [Bacillus cereus ATCC 14579]
>gi|30021584|ref|NP_833215.1| Collagen triple helix repeat protein [Bacillus cereus ATCC 14579]
>gi|30021636|ref|NP_833267.1| IG hypothetical 17193 [Bacillus cereus ATCC 14579]
>gi|30021663|ref|NP_833294.1| hypothetical protein BC3561 [Bacillus cereus ATCC 14579]
>gi|30021683|ref|NP_833314.1| hypothetical protein BC3582 [Bacillus cereus ATCC 14579]
>gi|30021727|ref|NP_833358.1| BioY protein [Bacillus cereus ATCC 14579]
>gi|30021733|ref|NP_833364.1| hypothetical protein BC3635 [Bacillus cereus ATCC 14579]
>gi|30021737|ref|NP_833368.1| hypothetical Membrane Spanning Protein [Bacillus cereus ATCC 14579]
>gi|30021777|ref|NP_833408.1| hypothetical protein BC3680 [Bacillus cereus ATCC 14579]
>gi|30021794|ref|NP_833425.1| hypothetical protein BC3697 [Bacillus cereus ATCC 14579]
>gi|30021807|ref|NP_833438.1| hypothetical Membrane Spanning Protein [Bacillus cereus ATCC 14579]
>gi|30021812|ref|NP_833443.1| PTS system, fructose-specific IIABC component [Bacillus cereus ATCC 14579]
>gi|30021848|ref|NP_833479.1| Murein hydrolase exporter [Bacillus cereus ATCC 14579]
>gi|30021868|ref|NP_833499.1| Stage V sporulation protein S [Bacillus cereus ATCC 14579]
>gi|30021934|ref|NP_833565.1| Signal recognition particle associated protein [Bacillus cereus ATCC 14579]
>gi|30022045|ref|NP_833676.1| hypothetical protein BC3957 [Bacillus cereus ATCC 14579]
>gi|30022069|ref|NP_833700.1| Tetrahydrodipicolinate N-acetyltransferase [Bacillus cereus ATCC 14579]
>gi|30022080|ref|NP_833711.1| hypothetical protein BC3992 [Bacillus cereus ATCC 14579]
>gi|30022084|ref|NP_833715.1| hypothetical protein BC3996 [Bacillus cereus ATCC 14579]
>gi|30022207|ref|NP_833838.1| hypothetical protein BC4120 [Bacillus cereus ATCC 14579]
>gi|30022219|ref|NP_833850.1| L-serine dehydratase [Bacillus cereus ATCC 14579]
>gi|30022267|ref|NP_833898.1| Biotin carboxyl carrier protein of acetyl-CoA carboxylase [Bacillus cereus ATCC 14579]
>gi|30022269|ref|NP_833900.1| Stage III sporulation protein AH [Bacillus cereus ATCC 14579]
>gi|30022273|ref|NP_833904.1| Stage III sporulation protein AD [Bacillus cereus ATCC 14579]
>gi|30022285|ref|NP_833916.1| hypothetical Membrane Spanning Protein [Bacillus cereus ATCC 14579]
>gi|30022291|ref|NP_833922.1| hypothetical protein BC4208 [Bacillus cereus ATCC 14579]
>gi|30022296|ref|NP_833927.1| Quaternary ammonium compound-resistance protein [Bacillus cereus ATCC 14579]
>gi|30022298|ref|NP_833929.1| hypothetical protein BC4215 [Bacillus cereus ATCC 14579]
>gi|30022305|ref|NP_833936.1| Transcriptional regulator, GntR family [Bacillus cereus ATCC 14579]
>gi|30022342|ref|NP_833973.1| hypothetical Membrane Spanning Protein [Bacillus cereus ATCC 14579]
>gi|30022366|ref|NP_833997.1| VrrA protein [Bacillus cereus ATCC 14579]
>gi|30022394|ref|NP_834025.1| GrpE protein [Bacillus cereus ATCC 14579]
>gi|30022418|ref|NP_834049.1| hypothetical Membrane Spanning Protein [Bacillus cereus ATCC 14579]
>gi|30022436|ref|NP_834067.1| hypothetical Membrane Spanning Protein [Bacillus cereus ATCC 14579]
>gi|30022452|ref|NP_834083.1| hypothetical protein BC4371 [Bacillus cereus ATCC 14579]
>gi|30022454|ref|NP_834085.1| hypothetical protein BC4373 [Bacillus cereus ATCC 14579]

>gi|30022500|ref|NP_834131.1| hypothetical protein BC4419 [Bacillus cereus ATCC 14579]
>gi|30022501|ref|NP_834132.1| SpoVID-dependent spore coat assembly factor SafA [Bacillus cereus ATCC 14579]
>gi|30022548|ref|NP_834179.1| Stage VI sporulation protein D [Bacillus cereus ATCC 14579]
>gi|30022576|ref|NP_834207.1| Tetracycline resistence protein TETA(L)/TETK [Bacillus cereus ATCC 14579]
>gi|30022604|ref|NP_834235.1| hypothetical Membrane Spanning Protein [Bacillus cereus ATCC 14579]
>gi|30022623|ref|NP_834254.1| Cell surface protein [Bacillus cereus ATCC 14579]
>gi|30022641|ref|NP_834272.1| hypothetical Membrane Spanning Protein [Bacillus cereus ATCC 14579]
>gi|30022642|ref|NP_834273.1| hypothetical Membrane Spanning Protein [Bacillus cereus ATCC 14579]
>gi|30022671|ref|NP_834302.1| hypothetical Membrane Spanning Protein [Bacillus cereus ATCC 14579]
>gi|30022697|ref|NP_834328.1| hypothetical protein BC4622 [Bacillus cereus ATCC 14579]
>gi|30022708|ref|NP_834339.1| hypothetical Membrane Spanning Protein [Bacillus cereus ATCC 14579]
>gi|30022715|ref|NP_834346.1| hypothetical protein BC4640 [Bacillus cereus ATCC 14579]
>gi|30022733|ref|NP_834364.1| Maltose O-acetyltransferase [Bacillus cereus ATCC 14579]
>gi|30022761|ref|NP_834392.1| Cell division protein ftsK [Bacillus cereus ATCC 14579]
>gi|30022762|ref|NP_834393.1| N-acetylmuramoyl-L-alanine amidase [Bacillus cereus ATCC 14579]
>gi|30022798|ref|NP_834429.1| hypothetical Membrane Spanning Protein [Bacillus cereus ATCC 14579]
>gi|30022804|ref|NP_834435.1| Spore germination protein IA [Bacillus cereus ATCC 14579]
>gi|30022809|ref|NP_834440.1| VrrB [Bacillus cereus ATCC 14579]
>gi|30022818|ref|NP_834449.1| hypothetical protein BC4745 [Bacillus cereus ATCC 14579]
>gi|30022837|ref|NP_834468.1| hypothetical protein BC4764 [Bacillus cereus ATCC 14579]
>gi|30022842|ref|NP_834473.1| Collagen triple helix repeat protein [Bacillus cereus ATCC 14579]
>gi|30022869|ref|NP_834500.1| hypothetical protein BC4798 [Bacillus cereus ATCC 14579]
>gi|30022879|ref|NP_834510.1| Two-component response regulator [Bacillus cereus ATCC 14579]
>gi|30022881|ref|NP_834512.1| Cell surface protein [Bacillus cereus ATCC 14579]
>gi|30022888|ref|NP_834519.1| hypothetical protein BC4819 [Bacillus cereus ATCC 14579]
>gi|30023026|ref|NP_834657.1| ABC transporter permease protein [Bacillus cereus ATCC 14579]
>gi|30023054|ref|NP_834685.1| hypothetical exported repetitive protein [Bacillus cereus ATCC 14579]
>gi|30023086|ref|NP_834717.1| IG hypothetical 16995 [Bacillus cereus ATCC 14579]
>gi|30023105|ref|NP_834736.1| CrcB family protein [Bacillus cereus ATCC 14579]
>gi|30023111|ref|NP_834742.1| Transcriptional regulator, MerR family [Bacillus cereus ATCC 14579]
>gi|30023124|ref|NP_834755.1| putative lantibiotic precursor peptide [Bacillus cereus ATCC 14579]
>gi|30023125|ref|NP_834756.1| putative lantibiotic precursor peptide [Bacillus cereus ATCC 14579]
>gi|30023126|ref|NP_834757.1| putative lantibiotic precursor peptide [Bacillus cereus ATCC 14579]
>gi|30023127|ref|NP_834758.1| putative lantibiotic precursor peptide [Bacillus cereus ATCC 14579]
>gi|30023153|ref|NP_834784.1| hypothetical protein BC5116 [Bacillus cereus ATCC 14579]
>gi|30023207|ref|NP_834838.1| hypothetical Membrane Spanning Protein [Bacillus cereus ATCC 14579]
>gi|30023229|ref|NP_834860.1| N-acetylmuramoyl-L-alanine amidase [Bacillus cereus ATCC 14579]
>gi|30023247|ref|NP_834878.1| PTS system, lichenan oligosaccharide-specific IIA component [Bacillus cereus ATCC 14579]
>gi|30023266|ref|NP_834897.1| N-acetylmuramoyl-L-alanine amidase [Bacillus cereus ATCC 14579]
>gi|30023271|ref|NP_834902.1| enterotoxin / cell-wall binding protein [Bacillus cereus ATCC 14579]
>gi|30023296|ref|NP_834927.1| EPSX protein [Bacillus cereus ATCC 14579]
>gi|30023331|ref|NP_834962.1| NADH-quinone oxidoreductase chain C [Bacillus cereus ATCC 14579]
>gi|30023335|ref|NP_834966.1| Integral membrane protein [Bacillus cereus ATCC 14579]
>gi|30023358|ref|NP_834989.1| Stage II sporulation protein R [Bacillus cereus ATCC 14579]
>gi|30023370|ref|NP_835001.1| DNA-directed RNA polymerase delta chain [Bacillus cereus ATCC 14579]
>gi|30023387|ref|NP_835018.1| Collagen adhesion protein [Bacillus cereus ATCC 14579]
>gi|30023413|ref|NP_835044.1| Ferrichrome transport system permease protein fhuB [Bacillus cereus ATCC 14579]
>gi|30023421|ref|NP_835052.1| hypothetical protein BC5391 [Bacillus cereus ATCC 14579]
>gi|30023452|ref|NP_835083.1| hypothetical protein BC5422 [Bacillus cereus ATCC 14579]
>gi|30023493|ref|NP_835124.1| LSU ribosomal protein L9P [Bacillus cereus ATCC 14579]
>gi|30023497|ref|NP_835128.1| Single-strand DNA binding protein [Bacillus cereus ATCC 14579]
>gi|30061576|ref|NP_835747.1| hypothetical protein S0005 [Shigella flexneri 2a str. 2457T]
>gi|30061582|ref|NP_835753.1| positive regulator for sigma 32 heat shock promoters [Shigella flexneri 2a str. 2457T]
>gi|30061585|ref|NP_835756.1| chaperone with DnaK; heat shock protein [Shigella flexneri 2a str. 2457T]
>gi|30061587|ref|NP_835758.1| Na+/H+ antiporter [Shigella flexneri 2a str. 2457T]
>gi|30061681|ref|NP_835852.1| pyruvate dehydrogenase (dihydrolipoyltransacetylase component) [Shigella flexneri 2a str. 2457T]
>gi|30061798|ref|NP_835969.1| putative DNA transfer protein [Shigella flexneri 2a str. 2457T]
>gi|30061893|ref|NP_836064.1| ATP-dependent dsDNA exonuclease [Shigella flexneri 2a str. 2457T]
>gi|30061944|ref|NP_836115.1| hypothetical protein S0393 [Shigella flexneri 2a str. 2457T]
>gi|30062034|ref|NP_836205.1| hypothetical protein S0495 [Shigella flexneri 2a str. 2457T]
>gi|30062048|ref|NP_836219.1| ferric enterobactin (enterochelin) transport protein [Shigella flexneri 2a str. 2457T]
>gi|30062049|ref|NP_836220.1| hypothetical membrane protein P43 [Shigella flexneri 2a str. 2457T]
>gi|30062091|ref|NP_836262.1| putative homeobox protein [Shigella flexneri 2a str. 2457T]
>gi|30062095|ref|NP_836266.1| hypothetical protein S0568 [Shigella flexneri 2a str. 2457T]
>gi|30062098|ref|NP_836269.1| membrane spanning protein [Shigella flexneri 2a str. 2457T]
>gi|30062140|ref|NP_836311.1| hypothetical protein S0619 [Shigella flexneri 2a str. 2457T]
>gi|30062184|ref|NP_836355.1| hypothetical protein S0676 [Shigella flexneri 2a str. 2457T]
>gi|30062231|ref|NP_836402.1| endopeptidase [Shigella flexneri 2a str. 2457T]
>gi|30062255|ref|NP_836426.1| putative tail component encoded by cryptic prophage CP-933M [Shigella flexneri 2a str. 2457T]
>gi|30062276|ref|NP_836447.1| hypothetical protein S0782 [Shigella flexneri 2a str. 2457T]

>gi|30062280|ref|NP_836451.1| putative membrane protein [Shigella flexneri 2a str. 2457T]
>gi|30062297|ref|NP_836468.1| putative transmembrane protein subunit [Shigella flexneri 2a str. 2457T]
>gi|30062325|ref|NP_836496.1| hypothetical protein S0839 [Shigella flexneri 2a str. 2457T]
>gi|30062332|ref|NP_836503.1| hypothetical protein S0846 [Shigella flexneri 2a str. 2457T]
>gi|30062361|ref|NP_836532.1| transmembrane water channel AqpZ protein [Shigella flexneri 2a str. 2457T]
>gi|30062377|ref|NP_836548.1| cell division protein [Shigella flexneri 2a str. 2457T]
>gi|30062426|ref|NP_836597.1| ISSfl3 orfC [Shigella flexneri 2a str. 2457T]
>gi|30062524|ref|NP_836695.1| hypothetical protein S1056 [Shigella flexneri 2a str. 2457T]
>gi|30062618|ref|NP_836789.1| RNase E [Shigella flexneri 2a str. 2457T]
>gi|30062638|ref|NP_836809.1| hypothetical protein S1189 [Shigella flexneri 2a str. 2457T]
>gi|30062689|ref|NP_836860.1| hypothetical protein S1245 [Shigella flexneri 2a str. 2457T]
>gi|30062703|ref|NP_836874.1| Na+/H+ antiporter [Shigella flexneri 2a str. 2457T]
>gi|30062708|ref|NP_836879.1| invasion plasmid antigen fragment [Shigella flexneri 2a str. 2457T]
>gi|30062713|ref|NP_836884.1| hypothetical protein S1273 [Shigella flexneri 2a str. 2457T]
>gi|30062717|ref|NP_836888.1| putative iron compound ABC transporter permease protein [Shigella flexneri 2a str. 2457T]
>gi|30062757|ref|NP_836928.1| DNA-binding protein HLP-II (HU, BH2, HD, NS); pleiotropic regulator [Shigella flexneri 2a str. 2457T]
>gi|30062775|ref|NP_836946.1| membrane protein, energy transducer [Shigella flexneri 2a str. 2457T]
>gi|30062778|ref|NP_836949.1| hypothetical protein S1344 [Shigella flexneri 2a str. 2457T]
>gi|30062782|ref|NP_836953.1| hypothetical protein S1348 [Shigella flexneri 2a str. 2457T]
>gi|30062805|ref|NP_836976.1| osmotically inducible lipoprotein [Shigella flexneri 2a str. 2457T]
>gi|30062824|ref|NP_836995.1| phage shock protein, inner membrane protein [Shigella flexneri 2a str. 2457T]
>gi|30062846|ref|NP_837017.1| ISSfl4 orf [Shigella flexneri 2a str. 2457T]
>gi|30062879|ref|NP_837050.1| probable enzyme [Shigella flexneri 2a str. 2457T]
>gi|30062950|ref|NP_837121.1| putative amino acid/amine transport protein [Shigella flexneri 2a str. 2457T]
>gi|30062979|ref|NP_837150.1| putative cytochrome oxidase [Shigella flexneri 2a str. 2457T]
>gi|30063016|ref|NP_837187.1| 50S ribosomal subunit protein A [Shigella flexneri 2a str. 2457T]
>gi|30063022|ref|NP_837193.1| Vitamin B12 transport permease protein [Shigella flexneri 2a str. 2457T]
>gi|30063085|ref|NP_837256.1| putative transport system permease protein [Shigella flexneri 2a str. 2457T]
>gi|30063111|ref|NP_837282.1| acid shock protein [Shigella flexneri 2a str. 2457T]
>gi|30063113|ref|NP_837284.1| possible chaperone [Shigella flexneri 2a str. 2457T]
>gi|30063115|ref|NP_837286.1| putative transport protein [Shigella flexneri 2a str. 2457T]
>gi|30063141|ref|NP_837312.1| hypothetical protein S1783 [Shigella flexneri 2a str. 2457T]
>gi|30063144|ref|NP_837315.1| putative membrane protein [Shigella flexneri 2a str. 2457T]
>gi|30063147|ref|NP_837318.1| hypothetical protein S1789 [Shigella flexneri 2a str. 2457T]
>gi|30063158|ref|NP_837329.1| putative outer membrane protein [Shigella flexneri 2a str. 2457T]
>gi|30063171|ref|NP_837342.1| putative lipoprotein [Shigella flexneri 2a str. 2457T]
>gi|30063191|ref|NP_837362.1| murein lipoprotein [Shigella flexneri 2a str. 2457T]
>gi|30063246|ref|NP_837417.1| putative transport system permease protein [Shigella flexneri 2a str. 2457T]
>gi|30063258|ref|NP_837429.1| hypothetical protein S1922 [Shigella flexneri 2a str. 2457T]
>gi|30063339|ref|NP_837510.1| positive regulator of CheA protein activity [Shigella flexneri 2a str. 2457T]
>gi|30063380|ref|NP_837551.1| flagellar assembly protein FliH [Shigella flexneri 2a str. 2457T]
>gi|30063386|ref|NP_837557.1| flagellar protein FliO [Shigella flexneri 2a str. 2457T]
>gi|30063388|ref|NP_837559.1| flagellar biosynthetic protein FliR [Shigella flexneri 2a str. 2457T]
>gi|30063394|ref|NP_837565.1| putative transmembrane subunit [Shigella flexneri 2a str. 2457T]
>gi|30063409|ref|NP_837580.1| hypothetical protein S2116 [Shigella flexneri 2a str. 2457T]
>gi|30063444|ref|NP_837615.1| cobalamin 5-phosphate synthase [Shigella flexneri 2a str. 2457T]
>gi|30063457|ref|NP_837628.1| ISSfl3 orfC [Shigella flexneri 2a str. 2457T]
>gi|30063484|ref|NP_837655.1| glycosyl translocase [Shigella flexneri 2a str. 2457T]
>gi|30063566|ref|NP_837737.1| putative phage tail fiber protein [Shigella flexneri 2a str. 2457T]
>gi|30063640|ref|NP_837811.1| heme exporter protein B, cytochrome c-type biogenesis protein [Shigella flexneri 2a str. 2457T]
>gi|30063740|ref|NP_837911.1| putative lipoprotein [Shigella flexneri 2a str. 2457T]
>gi|30063794|ref|NP_837965.1| hypothetical protein S2595 [Shigella flexneri 2a str. 2457T]
>gi|30063795|ref|NP_837966.1| hypothetical protein S2596 [Shigella flexneri 2a str. 2457T]
>gi|30063802|ref|NP_837973.1| hypothetical protein S2609 [Shigella flexneri 2a str. 2457T]
>gi|30063806|ref|NP_837977.1| cell division protein involved in FtsZ ring [Shigella flexneri 2a str. 2457T]
>gi|30063825|ref|NP_837996.1| hypothetical protein S2634 [Shigella flexneri 2a str. 2457T]
>gi|30063863|ref|NP_838034.1| hydrogenase 4 membrane subunit [Shigella flexneri 2a str. 2457T]
>gi|30063880|ref|NP_838051.1| putative outer membrane lipoprotein [Shigella flexneri 2a str. 2457T]
>gi|30063881|ref|NP_838052.1| putative membrane protein [Shigella flexneri 2a str. 2457T]
>gi|30063891|ref|NP_838062.1| hypothetical protein S2715 [Shigella flexneri 2a str. 2457T]
>gi|30063893|ref|NP_838064.1| putative mating pair formation protein [Shigella flexneri 2a str. 2457T]
>gi|30063905|ref|NP_838076.1| hypothetical protein S2731 [Shigella flexneri 2a str. 2457T]
>gi|30063908|ref|NP_838079.1| putative membrane protein [Shigella flexneri 2a str. 2457T]
>gi|30063972|ref|NP_838143.1| sigma-E factor [Shigella flexneri 2a str. 2457T]
>gi|30064037|ref|NP_838208.1| DNA-binding protein; H-NS-like protein [Shigella flexneri 2a str. 2457T]
>gi|30064062|ref|NP_838233.1| PTS system, glucitol/sorbitol-specific IIB component and second of two IIC components [Shigella flexneri 2a str. 2457T]
>gi|30064077|ref|NP_838248.1| membrane-spanning protein of hydrogenase 3 (part of FHL complex) [Shigella flexneri 2a str. 2457T]
>gi|30064099|ref|NP_838270.1| lipoprotein [Shigella flexneri 2a str. 2457T]
>gi|30064119|ref|NP_838290.1| putative transport protein [Shigella flexneri 2a str. 2457T]

>gi|30064165|ref|NP_838336.1| prepilin peptidase dependent protein C [Shigella flexneri 2a str. 2457T]
>gi|30064237|ref|NP_838408.1| putative inner membrane protein [Shigella flexneri 2a str. 2457T]
>gi|30064247|ref|NP_838418.1| putative serine protease [Shigella flexneri 2a str. 2457T]
>gi|30064406|ref|NP_838577.1| putative kinase [Shigella flexneri 2a str. 2457T]
>gi|30064417|ref|NP_838588.1| hypothetical protein S3318 [Shigella flexneri 2a str. 2457T]
>gi|30064471|ref|NP_838642.1| N-acetylgalactosameine-specific IIC component 2 [Shigella flexneri 2a str. 2457T]
>gi|30064502|ref|NP_838673.1| inducible ATP-independent RNA helicase [Shigella flexneri 2a str. 2457T]
>gi|30064508|ref|NP_838679.1| protein chain initiation factor IF-2 [Shigella flexneri 2a str. 2457T]
>gi|30064513|ref|NP_838684.1| protein translocase membrane component [Shigella flexneri 2a str. 2457T]
>gi|30064581|ref|NP_838752.1| rod shape-determining protein [Shigella flexneri 2a str. 2457T]
>gi|30064586|ref|NP_838757.1| acetyl CoA carboxylase, carrier of biotin, BCCP subunit [Shigella flexneri 2a str. 2457T]
>gi|30064617|ref|NP_838788.1| hypothetical protein S3552 [Shigella flexneri 2a str. 2457T]
>gi|30064648|ref|NP_838819.1| ssDNA-binding protein [Shigella flexneri 2a str. 2457T]
>gi|30064731|ref|NP_838902.1| 50S ribosomal subunit protein L7/L12 [Shigella flexneri 2a str. 2457T]
>gi|30064774|ref|NP_838945.1| essential cell division protein [Shigella flexneri 2a str. 2457T]
>gi|30064798|ref|NP_838969.1| 2-keto-3-deoxy-D-gluconate transport protein [Shigella flexneri 2a str. 2457T]
>gi|30064845|ref|NP_839016.1| hypothetical protein S3810 [Shigella flexneri 2a str. 2457T]
>gi|30064866|ref|NP_839037.1| hypothetical protein S3838 [Shigella flexneri 2a str. 2457T]
>gi|30064867|ref|NP_839038.1| hypothetical protein S3840 [Shigella flexneri 2a str. 2457T]
>gi|30064886|ref|NP_839057.1| hypothetical protein S3860 [Shigella flexneri 2a str. 2457T]
>gi|30064904|ref|NP_839075.1| uroporphyrinogen III methylase [Shigella flexneri 2a str. 2457T]
>gi|30064943|ref|NP_839114.1| D-ribose high-affinity transport protein [Shigella flexneri 2a str. 2457T]
>gi|30064956|ref|NP_839127.1| membrane-bound ATP synthase [Shigella flexneri 2a str. 2457T]
>gi|30064958|ref|NP_839129.1| membrane-bound ATP synthase, F0 sector, subunit c [Shigella flexneri 2a str. 2457T]
>gi|30064959|ref|NP_839130.1| membrane-bound ATP synthase, F0 sector, subunit b [Shigella flexneri 2a str. 2457T]
>gi|30064981|ref|NP_839152.1| sensor histidine protein kinase, phosphorylates UhpA [Shigella flexneri 2a str. 2457T]
>gi|30065027|ref|NP_839198.1| hypothetical protein S4028 [Shigella flexneri 2a str. 2457T]
>gi|30065062|ref|NP_839233.1| hypothetical protein S4831 [Shigella flexneri 2a str. 2457T]
>gi|30065169|ref|NP_839340.1| putative outer membrane protein [Shigella flexneri 2a str. 2457T]
>gi|30065178|ref|NP_839349.1| dipeptide transport system permease protein 2 [Shigella flexneri 2a str. 2457T]
>gi|30065238|ref|NP_839409.1| putative membrane protein [Shigella flexneri 2a str. 2457T]
>gi|30065255|ref|NP_839426.1| putative enzyme [Shigella flexneri 2a str. 2457T]
>gi|30065259|ref|NP_839430.1| cell division membrane protein [Shigella flexneri 2a str. 2457T]
>gi|30065274|ref|NP_839445.1| hypothetical protein S4297 [Shigella flexneri 2a str. 2457T]
>gi|30065323|ref|NP_839494.1| hypothetical protein S4350 [Shigella flexneri 2a str. 2457T]
>gi|30065328|ref|NP_839499.1| putative membrane protein [Shigella flexneri 2a str. 2457T]
>gi|30065365|ref|NP_839536.1| FKBP-type peptidyl-prolyl cis-trans isomerase (rotamase) [Shigella flexneri 2a str. 2457T]
>gi|30065387|ref|NP_839558.1| 50S ribosomal subunit protein L29 [Shigella flexneri 2a str. 2457T]
>gi|30065395|ref|NP_839566.1| 50S ribosomal subunit protein L18 [Shigella flexneri 2a str. 2457T]
>gi|30065398|ref|NP_839569.1| 50S ribosomal subunit protein L15 [Shigella flexneri 2a str. 2457T]
>gi|30065408|ref|NP_839579.1| ISSfl3 orfC [Shigella flexneri 2a str. 2457T]
>gi|30065515|ref|NP_839686.1| hypothetical protein S4561 [Shigella flexneri 2a str. 2457T]
>gi|30065546|ref|NP_839717.1| protease specific for phage lambda cII repressor [Shigella flexneri 2a str. 2457T]
>gi|30260207|ref|NP_842584.1| hypothetical protein BA0013 [Bacillus anthracis str. Ames]
>gi|30260250|ref|NP_842627.1| membrane protein, putative [Bacillus anthracis str. Ames]
>gi|30260291|ref|NP_842668.1| ribosomal protein L7/L12 [Bacillus anthracis str. Ames]
>gi|30260341|ref|NP_842718.1| hypothetical protein BA0151 [Bacillus anthracis str. Ames]
>gi|30260359|ref|NP_842736.1| lipoprotein, putative [Bacillus anthracis str. Ames]
>gi|30260408|ref|NP_842785.1| hypothetical protein BA0229 [Bacillus anthracis str. Ames]
>gi|30260457|ref|NP_842834.1| bacitracin ABC transporter, permease protein, putative [Bacillus anthracis str. Ames]
>gi|30260474|ref|NP_842851.1| SPFH domain/band 7 family protein [Bacillus anthracis str. Ames]
>gi|30260520|ref|NP_842897.1| iron compound ABC transporter, permease protein [Bacillus anthracis str. Ames]
>gi|30260555|ref|NP_842932.1| hypothetical protein BA0388 [Bacillus anthracis str. Ames]
>gi|30260564|ref|NP_842941.1| LPXTG-motif cell wall anchor domain protein [Bacillus anthracis str. Ames]
>gi|30260644|ref|NP_843021.1| hypothetical protein BA0482 [Bacillus anthracis str. Ames]
>gi|30260651|ref|NP_843028.1| hypothetical protein BA0490 [Bacillus anthracis str. Ames]
>gi|30260683|ref|NP_843060.1| small acid-soluble spore protein, gamma-type [Bacillus anthracis str. Ames]
>gi|30260710|ref|NP_843087.1| internalin, putative [Bacillus anthracis str. Ames]
>gi|30260715|ref|NP_843092.1| SPFH domain/band 7 family protein [Bacillus anthracis str. Ames]
>gi|30260730|ref|NP_843107.1| hypothetical protein BA0573 [Bacillus anthracis str. Ames]
>gi|30260780|ref|NP_843157.1| hypothetical protein BA0626 [Bacillus anthracis str. Ames]
>gi|30260781|ref|NP_843158.1| hypothetical protein BA0627 [Bacillus anthracis str. Ames]
>gi|30260835|ref|NP_843212.1| hypothetical protein BA0685 [Bacillus anthracis str. Ames]
>gi|30260938|ref|NP_843315.1| hypothetical protein BA0796 [Bacillus anthracis str. Ames]
>gi|30260953|ref|NP_843330.1| hypothetical protein BA0812 [Bacillus anthracis str. Ames]
>gi|30260966|ref|NP_843343.1| hypothetical protein BA0825 [Bacillus anthracis str. Ames]
>gi|30261001|ref|NP_843378.1| hypothetical protein BA0865 [Bacillus anthracis str. Ames]
>gi|30261045|ref|NP_843422.1| hypothetical protein BA0915 [Bacillus anthracis str. Ames]
>gi|30261061|ref|NP_843438.1| hypothetical protein BA0932 [Bacillus anthracis str. Ames]
>gi|30261065|ref|NP_843442.1| lipoprotein, putative [Bacillus anthracis str. Ames]

>gi|30261066|ref|NP_843443.1| hypothetical protein BA0937 [Bacillus anthracis str. Ames]
>gi|30261076|ref|NP_843453.1| hypothetical protein BA0948 [Bacillus anthracis str. Ames]
>gi|30261077|ref|NP_843454.1| protein-export membrane protein-related protein [Bacillus anthracis str. Ames]
>gi|30261085|ref|NP_843462.1| hypothetical protein BA0957 [Bacillus anthracis str. Ames]
>gi|30261097|ref|NP_843474.1| CAAX amino terminal protease family protein [Bacillus anthracis str. Ames]
>gi|30261111|ref|NP_843488.1| hypothetical protein BA0987 [Bacillus anthracis str. Ames]
>gi|30261123|ref|NP_843500.1| hypothetical protein BA0999 [Bacillus anthracis str. Ames]
>gi|30261127|ref|NP_843504.1| hypothetical protein BA1003 [Bacillus anthracis str. Ames]
>gi|30261166|ref|NP_843543.1| hypothetical protein BA1044 [Bacillus anthracis str. Ames]
>gi|30261173|ref|NP_843550.1| hypothetical protein BA1051 [Bacillus anthracis str. Ames]
>gi|30261180|ref|NP_843557.1| lipoprotein, putative [Bacillus anthracis str. Ames]
>gi|30261289|ref|NP_843666.1| hypothetical protein BA1189 [Bacillus anthracis str. Ames]
>gi|30261299|ref|NP_843676.1| membrane protein, putative [Bacillus anthracis str. Ames]
>gi|30261318|ref|NP_843695.1| hypothetical protein BA1222 [Bacillus anthracis str. Ames]
>gi|30261328|ref|NP_843705.1| hypothetical protein BA1233 [Bacillus anthracis str. Ames]
>gi|30261331|ref|NP_843708.1| hypothetical protein BA1236 [Bacillus anthracis str. Ames]
>gi|30261350|ref|NP_843727.1| hypothetical protein BA1255 [Bacillus anthracis str. Ames]
>gi|30261396|ref|NP_843773.1| hypothetical protein BA1306 [Bacillus anthracis str. Ames]
>gi|30261485|ref|NP_843862.1| hypothetical protein BA1405 [Bacillus anthracis str. Ames]
>gi|30261487|ref|NP_843864.1| hypothetical protein BA1407 [Bacillus anthracis str. Ames]
>gi|30261528|ref|NP_843905.1| peptidase, M23/M37 family [Bacillus anthracis str. Ames]
>gi|30261542|ref|NP_843919.1| hypothetical protein BA1464 [Bacillus anthracis str. Ames]
>gi|30261652|ref|NP_844029.1| hypothetical protein BA1580 [Bacillus anthracis str. Ames]
>gi|30261653|ref|NP_844030.1| spore coat protein D, putative [Bacillus anthracis str. Ames]
>gi|30261673|ref|NP_844050.1| lipoprotein, putative [Bacillus anthracis str. Ames]
>gi|30261679|ref|NP_844056.1| acyltransferase family protein [Bacillus anthracis str. Ames]
>gi|30261719|ref|NP_844096.1| membrane protein, putative [Bacillus anthracis str. Ames]
>gi|30261724|ref|NP_844101.1| hypothetical protein BA1654 [Bacillus anthracis str. Ames]
>gi|30261732|ref|NP_844109.1| hypothetical protein BA1663 [Bacillus anthracis str. Ames]
>gi|30261746|ref|NP_844123.1| hypothetical protein BA1680 [Bacillus anthracis str. Ames]
>gi|30261766|ref|NP_844143.1| flagellin [Bacillus anthracis str. Ames]
>gi|30261790|ref|NP_844167.1| lipoprotein, putative [Bacillus anthracis str. Ames]
>gi|30261938|ref|NP_844315.1| membrane protein, putative [Bacillus anthracis str. Ames]
>gi|30261984|ref|NP_844361.1| NLP/P60 family protein [Bacillus anthracis str. Ames]
>gi|30262021|ref|NP_844398.1| hypothetical protein BA1993 [Bacillus anthracis str. Ames]
>gi|30262037|ref|NP_844414.1| hypothetical protein BA2010 [Bacillus anthracis str. Ames]
>gi|30262052|ref|NP_844429.1| hypothetical protein BA2025 [Bacillus anthracis str. Ames]
>gi|30262056|ref|NP_844433.1| hypothetical protein BA2029 [Bacillus anthracis str. Ames]
>gi|30262070|ref|NP_844447.1| hypothetical protein BA2045 [Bacillus anthracis str. Ames]
>gi|30262098|ref|NP_844475.1| SPFH domain/Band 7 family protein [Bacillus anthracis str. Ames]
>gi|30262152|ref|NP_844529.1| hypothetical protein BA2131 [Bacillus anthracis str. Ames]
>gi|30262170|ref|NP_844547.1| hypothetical protein BA2149 [Bacillus anthracis str. Ames]
>gi|30262175|ref|NP_844552.1| stage V sporulation protein S [Bacillus anthracis str. Ames]
>gi|30262180|ref|NP_844557.1| hypothetical protein BA2162 [Bacillus anthracis str. Ames]
>gi|30262314|ref|NP_844691.1| hypothetical protein BA2306 [Bacillus anthracis str. Ames]
>gi|30262340|ref|NP_844717.1| hypothetical protein BA2333 [Bacillus anthracis str. Ames]
>gi|30262351|ref|NP_844728.1| penicillin-binding protein 1A [Bacillus anthracis str. Ames]
>gi|30262402|ref|NP_844779.1| hypothetical protein BA2402 [Bacillus anthracis str. Ames]
>gi|30262433|ref|NP_844810.1| hypothetical protein BA2434 [Bacillus anthracis str. Ames]
>gi|30262444|ref|NP_844821.1| hypothetical protein BA2445 [Bacillus anthracis str. Ames]
>gi|30262448|ref|NP_844825.1| hypothetical protein BA2449 [Bacillus anthracis str. Ames]
>gi|30262449|ref|NP_844826.1| hypothetical protein BA2450 [Bacillus anthracis str. Ames]
>gi|30262466|ref|NP_844843.1| membrane protein, putative [Bacillus anthracis str. Ames]
>gi|30262494|ref|NP_844871.1| hypothetical protein BA2496 [Bacillus anthracis str. Ames]
>gi|30262536|ref|NP_844913.1| hypothetical protein BA2545 [Bacillus anthracis str. Ames]
>gi|30262599|ref|NP_844976.1| hypothetical protein BA2614 [Bacillus anthracis str. Ames]
>gi|30262609|ref|NP_844986.1| hypothetical protein BA2624 [Bacillus anthracis str. Ames]
>gi|30262618|ref|NP_844995.1| hypothetical protein BA2633 [Bacillus anthracis str. Ames]
>gi|30262628|ref|NP_845005.1| hypothetical protein BA2643 [Bacillus anthracis str. Ames]
>gi|30262634|ref|NP_845011.1| permease, putative [Bacillus anthracis str. Ames]
>gi|30262659|ref|NP_845036.1| hypothetical protein BA2677 [Bacillus anthracis str. Ames]
>gi|30262718|ref|NP_845095.1| lipoprotein, putative [Bacillus anthracis str. Ames]
>gi|30262756|ref|NP_845133.1| hypothetical protein BA2784 [Bacillus anthracis str. Ames]
>gi|30262808|ref|NP_845185.1| hypothetical protein BA2840 [Bacillus anthracis str. Ames]
>gi|30262821|ref|NP_845198.1| hypothetical protein BA2856 [Bacillus anthracis str. Ames]
>gi|30262824|ref|NP_845201.1| hypothetical protein BA2859 [Bacillus anthracis str. Ames]
>gi|30262850|ref|NP_845227.1| hypothetical protein BA2887 [Bacillus anthracis str. Ames]
>gi|30262859|ref|NP_845236.1| hypothetical protein BA2897 [Bacillus anthracis str. Ames]
>gi|30262892|ref|NP_845269.1| lipoprotein, putative [Bacillus anthracis str. Ames]
>gi|30262901|ref|NP_845278.1| lipoprotein, putative [Bacillus anthracis str. Ames]

>gi|30262924|ref|NP_845301.1| hypothetical protein BA2967 [Bacillus anthracis str. Ames]
>gi|30262933|ref|NP_845310.1| ribose ABC transporter, permease protein, putative [Bacillus anthracis str. Ames]
>gi|30262959|ref|NP_845336.1| hypothetical protein BA3004 [Bacillus anthracis str. Ames]
>gi|30262972|ref|NP_845349.1| hypothetical protein BA3018 [Bacillus anthracis str. Ames]
>gi|30262977|ref|NP_845354.1| hypothetical protein BA3023 [Bacillus anthracis str. Ames]
>gi|30263012|ref|NP_845389.1| hypothetical protein BA3059 [Bacillus anthracis str. Ames]
>gi|30263087|ref|NP_845464.1| pyrroline-5-carboxylate reductase [Bacillus anthracis str. Ames]
>gi|30263088|ref|NP_845465.1| hypothetical protein BA3144 [Bacillus anthracis str. Ames]
>gi|30263149|ref|NP_845526.1| hypothetical protein BA3219 [Bacillus anthracis str. Ames]
>gi|30263154|ref|NP_845531.1| hypothetical protein BA3225 [Bacillus anthracis str. Ames]
>gi|30263160|ref|NP_845537.1| hypothetical protein BA3231 [Bacillus anthracis str. Ames]
>gi|30263163|ref|NP_845540.1| hypothetical protein BA3235 [Bacillus anthracis str. Ames]
>gi|30263167|ref|NP_845544.1| hypothetical protein BA3242 [Bacillus anthracis str. Ames]
>gi|30263177|ref|NP_845554.1| LPXTG-motif cell wall anchor domain protein [Bacillus anthracis str. Ames]
>gi|30263195|ref|NP_845572.1| hypothetical protein BA3278 [Bacillus anthracis str. Ames]
>gi|30263256|ref|NP_845633.1| hypothetical protein BA3347 [Bacillus anthracis str. Ames]
>gi|30263261|ref|NP_845638.1| hypothetical protein BA3352 [Bacillus anthracis str. Ames]
>gi|30263275|ref|NP_845652.1| lipoprotein, putative [Bacillus anthracis str. Ames]
>gi|30263290|ref|NP_845667.1| hypothetical protein BA3381 [Bacillus anthracis str. Ames]
>gi|30263292|ref|NP_845669.1| lipoprotein, putative [Bacillus anthracis str. Ames]
>gi|30263318|ref|NP_845695.1| hypothetical protein BA3411 [Bacillus anthracis str. Ames]
>gi|30263332|ref|NP_845709.1| pentapeptide repeats domain protein [Bacillus anthracis str. Ames]
>gi|30263386|ref|NP_845763.1| hypothetical protein BA3482 [Bacillus anthracis str. Ames]
>gi|30263391|ref|NP_845768.1| hypothetical protein BA3488 [Bacillus anthracis str. Ames]
>gi|30263393|ref|NP_845770.1| transcriptional regulator, DeoR family [Bacillus anthracis str. Ames]
>gi|30263395|ref|NP_845772.1| ABC transporter, efflux permease protein [Bacillus anthracis str. Ames]
>gi|30263447|ref|NP_845824.1| hypothetical protein BA3550 [Bacillus anthracis str. Ames]
>gi|30263467|ref|NP_845844.1| bile acid transporter family protein [Bacillus anthracis str. Ames]
>gi|30263482|ref|NP_845859.1| hypothetical protein BA3589 [Bacillus anthracis str. Ames]
>gi|30263531|ref|NP_845908.1| hypothetical protein BA3640 [Bacillus anthracis str. Ames]
>gi|30263575|ref|NP_845952.1| bioY family protein [Bacillus anthracis str. Ames]
>gi|30263580|ref|NP_845957.1| hypothetical protein BA3694 [Bacillus anthracis str. Ames]
>gi|30263586|ref|NP_845963.1| membrane protein, putative [Bacillus anthracis str. Ames]
>gi|30263610|ref|NP_845987.1| hypothetical protein BA3724 [Bacillus anthracis str. Ames]
>gi|30263611|ref|NP_845988.1| conserved repeat domain protein [Bacillus anthracis str. Ames]
>gi|30263626|ref|NP_846003.1| conserved hypothetical protein UPF0154 [Bacillus anthracis str. Ames]
>gi|30263676|ref|NP_846053.1| hypothetical protein BA3800 [Bacillus anthracis str. Ames]
>gi|30263684|ref|NP_846061.1| hypothetical protein BA3809 [Bacillus anthracis str. Ames]
>gi|30263699|ref|NP_846076.1| hypothetical protein BA3824 [Bacillus anthracis str. Ames]
>gi|30263715|ref|NP_846092.1| hypothetical protein BA3841 [Bacillus anthracis str. Ames]
>gi|30263719|ref|NP_846096.1| hypothetical protein BA3845 [Bacillus anthracis str. Ames]
>gi|30263720|ref|NP_846097.1| PTS system, fructose-specific IIABC component [Bacillus anthracis str. Ames]
>gi|30263755|ref|NP_846132.1| hypothetical protein BA3885 [Bacillus anthracis str. Ames]
>gi|30263782|ref|NP_846159.1| stage V sporulation protein S [Bacillus anthracis str. Ames]
>gi|30263788|ref|NP_846165.1| hypothetical protein BA3918 [Bacillus anthracis str. Ames]
>gi|30263846|ref|NP_846223.1| KH domain protein [Bacillus anthracis str. Ames]
>gi|30263849|ref|NP_846226.1| hypothetical protein BA3984 [Bacillus anthracis str. Ames]
>gi|30263994|ref|NP_846371.1| hypothetical protein BA4132 [Bacillus anthracis str. Ames]
>gi|30263995|ref|NP_846372.1| hypothetical protein BA4133 [Bacillus anthracis str. Ames]
>gi|30264028|ref|NP_846405.1| hypothetical protein BA4167 [Bacillus anthracis str. Ames]
>gi|30264040|ref|NP_846417.1| hypothetical protein BA4179 [Bacillus anthracis str. Ames]
>gi|30264045|ref|NP_846422.1| hypothetical protein BA4186 [Bacillus anthracis str. Ames]
>gi|30264053|ref|NP_846430.1| 2,3,4,5-tetrahydropyridine-2-carboxylate N-succinyltransferase, putative [Bacillus anthracis str. Ames]
>gi|30264065|ref|NP_846442.1| hypothetical protein BA4207 [Bacillus anthracis str. Ames]
>gi|30264069|ref|NP_846446.1| lipoprotein, putative [Bacillus anthracis str. Ames]
>gi|30264177|ref|NP_846554.1| hypothetical protein BA4321 [Bacillus anthracis str. Ames]
>gi|30264198|ref|NP_846575.1| hypothetical protein BA4342 [Bacillus anthracis str. Ames]
>gi|30264204|ref|NP_846581.1| hypothetical protein BA4349 [Bacillus anthracis str. Ames]
>gi|30264214|ref|NP_846591.1| L-serine dehydratase, iron-sulfur-dependent, alpha subunit [Bacillus anthracis str. Ames]
>gi|30264260|ref|NP_846637.1| acetyl-CoA carboxylase, biotin carboxyl carrier protein [Bacillus anthracis str. Ames]
>gi|30264261|ref|NP_846638.1| stage III sporulation protein AH [Bacillus anthracis str. Ames]
>gi|30264265|ref|NP_846642.1| stage III sporulation protein AD [Bacillus anthracis str. Ames]
>gi|30264281|ref|NP_846658.1| hypothetical protein BA4430 [Bacillus anthracis str. Ames]
>gi|30264286|ref|NP_846663.1| sugE protein [Bacillus anthracis str. Ames]
>gi|30264288|ref|NP_846665.1| hypothetical protein BA4437 [Bacillus anthracis str. Ames]
>gi|30264309|ref|NP_846686.1| hypothetical protein BA4460 [Bacillus anthracis str. Ames]
>gi|30264334|ref|NP_846711.1| hypothetical protein BA4486 [Bacillus anthracis str. Ames]
>gi|30264380|ref|NP_846757.1| ribosomal protein S21 [Bacillus anthracis str. Ames]
>gi|30264386|ref|NP_846763.1| GrpE protein [Bacillus anthracis str. Ames]
>gi|30264412|ref|NP_846789.1| hypothetical protein BA4567 [Bacillus anthracis str. Ames]

>gi|30264431|ref|NP_846808.1| hypothetical protein BA4589 [Bacillus anthracis str. Ames]
>gi|30264447|ref|NP_846824.1| hypothetical protein BA4605 [Bacillus anthracis str. Ames]
>gi|30264496|ref|NP_846873.1| forespore-specific protein, putative [Bacillus anthracis str. Ames]
>gi|30264497|ref|NP_846874.1| lysM domain protein [Bacillus anthracis str. Ames]
>gi|30264528|ref|NP_846905.1| stage VI sporulation protein D, putative [Bacillus anthracis str. Ames]
>gi|30264557|ref|NP_846934.1| hypothetical protein BA4723 [Bacillus anthracis str. Ames]
>gi|30264570|ref|NP_846947.1| membrane protein, putative [Bacillus anthracis str. Ames]
>gi|30264593|ref|NP_846970.1| hypothetical protein BA4764 [Bacillus anthracis str. Ames]
>gi|30264606|ref|NP_846983.1| hypothetical protein BA4778 [Bacillus anthracis str. Ames]
>gi|30264615|ref|NP_846992.1| hypothetical protein BA4787 [Bacillus anthracis str. Ames]
>gi|30264637|ref|NP_847014.1| hypothetical protein BA4811 [Bacillus anthracis str. Ames]
>gi|30264655|ref|NP_847032.1| membrane protein, putative [Bacillus anthracis str. Ames]
>gi|30264666|ref|NP_847043.1| hypothetical protein BA4840 [Bacillus anthracis str. Ames]
>gi|30264694|ref|NP_847071.1| hypothetical protein BA4870 [Bacillus anthracis str. Ames]
>gi|30264714|ref|NP_847091.1| hypothetical protein BA4891 [Bacillus anthracis str. Ames]
>gi|30264761|ref|NP_847138.1| FtsK/SpoIIIE family protein [Bacillus anthracis str. Ames]
>gi|30264784|ref|NP_847161.1| hypothetical protein BA4966 [Bacillus anthracis str. Ames]
>gi|30264795|ref|NP_847172.1| collagen triple helix repeat domain protein [Bacillus anthracis str. Ames]
>gi|30264799|ref|NP_847176.1| hypothetical protein BA4982 [Bacillus anthracis str. Ames]
>gi|30264801|ref|NP_847178.1| spore germination protein GerHA [Bacillus anthracis str. Ames]
>gi|30264806|ref|NP_847183.1| vrrB protein [Bacillus anthracis str. Ames]
>gi|30264817|ref|NP_847194.1| hypothetical protein BA5000 [Bacillus anthracis str. Ames]
>gi|30264872|ref|NP_847249.1| lipoprotein, putative [Bacillus anthracis str. Ames]
>gi|30264882|ref|NP_847259.1| DNA-binding response regulator [Bacillus anthracis str. Ames]
>gi|30264884|ref|NP_847261.1| cell wall surface anchor family protein [Bacillus anthracis str. Ames]
>gi|30264890|ref|NP_847267.1| hypothetical protein BA5077 [Bacillus anthracis str. Ames]
>gi|30264904|ref|NP_847281.1| hypothetical protein BA5093 [Bacillus anthracis str. Ames]
>gi|30264988|ref|NP_847365.1| hypothetical protein BA5182 [Bacillus anthracis str. Ames]
>gi|30265025|ref|NP_847402.1| ABC transporter, permease protein [Bacillus anthracis str. Ames]
>gi|30265064|ref|NP_847441.1| hypothetical protein BA5262 [Bacillus anthracis str. Ames]
>gi|30265069|ref|NP_847446.1| hypothetical protein BA5267 [Bacillus anthracis str. Ames]
>gi|30265073|ref|NP_847450.1| hypothetical protein BA5271 [Bacillus anthracis str. Ames]
>gi|30265119|ref|NP_847496.1| crcB protein [Bacillus anthracis str. Ames]
>gi|30265218|ref|NP_847595.1| endopeptidase lytE, putative [Bacillus anthracis str. Ames]
>gi|30265266|ref|NP_847643.1| hypothetical protein BA5481 [Bacillus anthracis str. Ames]
>gi|30265321|ref|NP_847698.1| NADH dehydrogenase I, C subunit, putative [Bacillus anthracis str. Ames]
>gi|30265350|ref|NP_847727.1| stage II sporulation protein R, putative [Bacillus anthracis str. Ames]
>gi|30265363|ref|NP_847740.1| DNA-directed RNA polymerase, delta subunit [Bacillus anthracis str. Ames]
>gi|30265413|ref|NP_847790.1| hypothetical protein BA5641 [Bacillus anthracis str. Ames]
>gi|30265491|ref|NP_847868.1| single-stranded DNA-binding protein [Bacillus anthracis str. Ames]
>gi|15604723|ref|NP_219507.1| hypothetical protein CT005 [Chlamydia trachomatis D/UW-3/CX]
>gi|15604765|ref|NP_219549.1| Histone-like Protein 2 [Chlamydia trachomatis D/UW-3/CX]
>gi|15604769|ref|NP_219553.1| hypothetical protein CT050 [Chlamydia trachomatis D/UW-3/CX]
>gi|15604770|ref|NP_219554.1| hypothetical protein CT051 [Chlamydia trachomatis D/UW-3/CX]
>gi|15604789|ref|NP_219573.1| Integral Membrane Protein [Chlamydia trachomatis D/UW-3/CX]
>gi|15604834|ref|NP_219618.1| hypothetical protein CT115 [Chlamydia trachomatis D/UW-3/CX]
>gi|15604836|ref|NP_219620.1| hypothetical protein CT117 [Chlamydia trachomatis D/UW-3/CX]
>gi|15604837|ref|NP_219621.1| hypothetical protein CT118 [Chlamydia trachomatis D/UW-3/CX]
>gi|15604838|ref|NP_219622.1| Inclusion Membrane Protein A [Chlamydia trachomatis D/UW-3/CX]
>gi|15604875|ref|NP_219659.1| hypothetical protein CT156 [Chlamydia trachomatis D/UW-3/CX]
>gi|15604883|ref|NP_219667.1| hypothetical protein CT164 [Chlamydia trachomatis D/UW-3/CX]
>gi|15604943|ref|NP_219727.1| CHLTR hypothetical protein [Chlamydia trachomatis D/UW-3/CX]
>gi|15604946|ref|NP_219730.1| hypothetical protein CT225 [Chlamydia trachomatis D/UW-3/CX]
>gi|15604947|ref|NP_219731.1| hypothetical protein CT226 [Chlamydia trachomatis D/UW-3/CX]
>gi|15604950|ref|NP_219734.1| hypothetical protein CT229 [Chlamydia trachomatis D/UW-3/CX]
>gi|15604951|ref|NP_219735.1| Neutral Amino Acid (Glutamate) Transporter [Chlamydia trachomatis D/UW-3/CX]
>gi|15604954|ref|NP_219738.1| Inclusion Membrane Protein C [Chlamydia trachomatis D/UW-3/CX]
>gi|15604964|ref|NP_219748.1| UDP-3-O-[3-hydroxymyristoyl] glucosamine N-acyltransferase [Chlamydia trachomatis D/UW-3/CX]
>gi|15604970|ref|NP_219754.1| hypothetical protein CT249 [Chlamydia trachomatis D/UW-3/CX]
>gi|15604975|ref|NP_219759.1| hypothetical protein CT254 [Chlamydia trachomatis D/UW-3/CX]
>gi|15604992|ref|NP_219776.1| hypothetical protein CT271 [Chlamydia trachomatis D/UW-3/CX]
>gi|15605037|ref|NP_219821.1| L7/L12 Ribosomal Protein [Chlamydia trachomatis D/UW-3/CX]
>gi|15605068|ref|NP_219852.1| hypothetical protein CT345 [Chlamydia trachomatis D/UW-3/CX]
>gi|15605081|ref|NP_219866.1| hypothetical protein CT357 [Chlamydia trachomatis D/UW-3/CX]
>gi|15605082|ref|NP_219867.1| hypothetical protein CT358 [Chlamydia trachomatis D/UW-3/CX]
>gi|15605084|ref|NP_219869.1| hypothetical protein CT360 [Chlamydia trachomatis D/UW-3/CX]
>gi|15605089|ref|NP_219874.1| hypothetical protein CT365 [Chlamydia trachomatis D/UW-3/CX]
>gi|15605113|ref|NP_219898.1| hypothetical protein CT388 [Chlamydia trachomatis D/UW-3/CX]
>gi|15605176|ref|NP_219962.1| hypothetical protein CT449 [Chlamydia trachomatis D/UW-3/CX]
>gi|15605183|ref|NP_219969.1| hypothetical protein CT456 [Chlamydia trachomatis D/UW-3/CX]

>gi|15605208|ref|NP_219994.1| hypothetical protein CT480.1 [Chlamydia trachomatis D/UW-3/CX]
>gi|15605237|ref|NP_220023.1| S11 Ribosomal Protein [Chlamydia trachomatis D/UW-3/CX]
>gi|15605240|ref|NP_220026.1| L15 Ribosomal Protein [Chlamydia trachomatis D/UW-3/CX]
>gi|15605307|ref|NP_220093.1| hypothetical protein CT578 [Chlamydia trachomatis D/UW-3/CX]
>gi|15605308|ref|NP_220094.1| hypothetical protein CT579 [Chlamydia trachomatis D/UW-3/CX]
>gi|15605328|ref|NP_220114.1| hypothetical protein CT598 [Chlamydia trachomatis D/UW-3/CX]
>gi|15605397|ref|NP_220183.1| (FHA domain; homology to adenylate cyclase) [Chlamydia trachomatis D/UW-3/CX]
>gi|15605403|ref|NP_220189.1| hypothetical protein CT670 [Chlamydia trachomatis D/UW-3/CX]
>gi|15605404|ref|NP_220190.1| hypothetical protein CT671 [Chlamydia trachomatis D/UW-3/CX]
>gi|15605451|ref|NP_220237.1| hypothetical protein CT718 [Chlamydia trachomatis D/UW-3/CX]
>gi|15605457|ref|NP_220243.1| hypothetical protein CT724 [Chlamydia trachomatis D/UW-3/CX]
>gi|15605476|ref|NP_220262.1| Histone-Like Developmental Protein [Chlamydia trachomatis D/UW-3/CX]
>gi|15605492|ref|NP_220278.1| Muramidase (invasin repeat family) [Chlamydia trachomatis D/UW-3/CX]
>gi|15605520|ref|NP_220306.1| S14 Ribosomal Protein [Chlamydia trachomatis D/UW-3/CX]
>gi|15605528|ref|NP_220314.1| hypothetical protein CT794.1 [Chlamydia trachomatis D/UW-3/CX]
>gi|15605547|ref|NP_220333.1| hypothetical protein CT813 [Chlamydia trachomatis D/UW-3/CX]
>gi|15605561|ref|NP_220347.1| CDP-diacylglycerol-serine-O-phosphatidyltransferase [Chlamydia trachomatis D/UW-3/CX]
>gi|15605588|ref|NP_220374.1| YhgN family [Chlamydia trachomatis D/UW-3/CX]
>gi|15605589|ref|NP_220375.1| hypothetical protein CT853 [Chlamydia trachomatis D/UW-3/CX]
>gi|15605596|ref|NP_220382.1| hypothetical protein CT860 [Chlamydia trachomatis D/UW-3/CX]
>gi|15605597|ref|NP_220383.1| hypothetical protein CT861 [Chlamydia trachomatis D/UW-3/CX]
>gi|15605604|ref|NP_220390.1| Membrane Thiol Protease (predicted) [Chlamydia trachomatis D/UW-3/CX]
>gi|55979973|ref|YP_143270.1| hypothetical protein TTHA0004 [Thermus thermophilus HB8]
>gi|55979977|ref|YP_143274.1| phage shock protein A [Thermus thermophilus HB8]
>gi|55979981|ref|YP_143278.1| conserved hypothetical protein, integral membrane protein [Thermus thermophilus HB8]
>gi|55979982|ref|YP_143279.1| geranylgeranyl diphosphate synthetase [Thermus thermophilus HB8]
>gi|55979983|ref|YP_143280.1| hypothetical protein TTHA0014 [Thermus thermophilus HB8]
>gi|55979986|ref|YP_143283.1| probable tetratricopeptide repeat family protein [Thermus thermophilus HB8]
>gi|55979988|ref|YP_143285.1| hypothetical protein TTHA0019 [Thermus thermophilus HB8]
>gi|55979989|ref|YP_143286.1| response regulator [Thermus thermophilus HB8]
>gi|55979990|ref|YP_143287.1| putative NADPH oxidoreductase [Thermus thermophilus HB8]
>gi|55979992|ref|YP_143289.1| putative ATP-dependent protease La [Thermus thermophilus HB8]
>gi|55979994|ref|YP_143291.1| hypothetical protein TTHA0025 [Thermus thermophilus HB8]
>gi|55979997|ref|YP_143294.1| putative macrolide-efflux protein [Thermus thermophilus HB8]
>gi|55979998|ref|YP_143295.1| hypothetical protein TTHA0029 [Thermus thermophilus HB8]
>gi|55979999|ref|YP_143296.1| hypothetical protein TTHA0030 [Thermus thermophilus HB8]
>gi|55980000|ref|YP_143297.1| conserved hypothetical membrane protein [Thermus thermophilus HB8]
>gi|55980004|ref|YP_143301.1| hypothetical membrane protein [Thermus thermophilus HB8]
>gi|55980006|ref|YP_143303.1| hypothetical protein TTHA0037 [Thermus thermophilus HB8]
>gi|55980007|ref|YP_143304.1| hypothetical protein TTHA0038 [Thermus thermophilus HB8]
>gi|55980008|ref|YP_143305.1| hypothetical protein TTHA0039 [Thermus thermophilus HB8]
>gi|55980012|ref|YP_143309.1| lipoprotein signal peptidase [Thermus thermophilus HB8]
>gi|55980013|ref|YP_143310.1| putative Na(+)/H(+) antiporter [Thermus thermophilus HB8]
>gi|55980016|ref|YP_143313.1| probable nucleotidyltransferase [Thermus thermophilus HB8]
>gi|55980024|ref|YP_143321.1| ATP-dependent DNA helicase RecG-related protein [Thermus thermophilus HB8]
>gi|55980025|ref|YP_143322.1| ammonium transporter [Thermus thermophilus HB8]
>gi|55980027|ref|YP_143324.1| transcriptional repressor of class I heat-shock genes [Thermus thermophilus HB8]
>gi|55980029|ref|YP_143326.1| hypothetical protein TTHA0060 [Thermus thermophilus HB8]
>gi|55980031|ref|YP_143328.1| alanine racemase [Thermus thermophilus HB8]
>gi|55980033|ref|YP_143330.1| hypothetical protein TTHA0064 [Thermus thermophilus HB8]
>gi|55980034|ref|YP_143331.1| glycolate oxidase subunit GlcE [Thermus thermophilus HB8]
>gi|55980035|ref|YP_143332.1| hypothetical protein TTHA0066 [Thermus thermophilus HB8]
>gi|55980038|ref|YP_143335.1| ABC transporter, periplasmic binding protein [Thermus thermophilus HB8]
>gi|55980039|ref|YP_143336.1| ABC transporter permease protein [Thermus thermophilus HB8]
>gi|55980040|ref|YP_143337.1| ABC-transporter, ATP-binding subunit [Thermus thermophilus HB8]
>gi|55980047|ref|YP_143344.1| hypothetical protein TTHA0078 [Thermus thermophilus HB8]
>gi|55980049|ref|YP_143346.1| hypothetical protein TTHA0080 [Thermus thermophilus HB8]
>gi|55980050|ref|YP_143347.1| hypothetical protein TTHA0081 [Thermus thermophilus HB8]
>gi|55980053|ref|YP_143350.1| NADH-quinone oxidoreductase chain 7 [Thermus thermophilus HB8]
>gi|55980060|ref|YP_143357.1| NADH-quinone oxidoreductase chain 8 [Thermus thermophilus HB8]
>gi|55980062|ref|YP_143359.1| NADH-quinone oxidoreductase chain 10 [Thermus thermophilus HB8]
>gi|55980064|ref|YP_143361.1| NADH-quinone oxidoreductase chain 12 [Thermus thermophilus HB8]
>gi|55980065|ref|YP_143362.1| NADH-quinone oxidoreductase chain 13 [Thermus thermophilus HB8]
>gi|55980066|ref|YP_143363.1| NADH-quinone oxidoreductase chain 14 [Thermus thermophilus HB8]
>gi|55980068|ref|YP_143365.1| serine protease [Thermus thermophilus HB8]
>gi|55980071|ref|YP_143368.1| hypothetical protein TTHA0102 [Thermus thermophilus HB8]
>gi|55980072|ref|YP_143369.1| putative oxidoreductase [Thermus thermophilus HB8]
>gi|55980076|ref|YP_143373.1| hypothetical protein TTHA0107 [Thermus thermophilus HB8]
>gi|55980078|ref|YP_143375.1| ATP-dependent RNA helicase [Thermus thermophilus HB8]
>gi|55980080|ref|YP_143377.1| hypothetical protein TTHA0111 [Thermus thermophilus HB8]

>gi|55980090|ref|YP_143387.1| competence protein DprA (SMF protein (DNA processing chain A)) [Thermus thermophilus HB8]
>gi|55980098|ref|YP_143395.1| alternative histidyl-tRNA synthetase [Thermus thermophilus HB8]
>gi|55980110|ref|YP_143407.1| hypothetical protein TTHA0141 [Thermus thermophilus HB8]
>gi|55980113|ref|YP_143410.1| sensor histidine kinase [Thermus thermophilus HB8]
>gi|55980114|ref|YP_143411.1| phosphate regulon transcriptional regulatory protein PhoB [Thermus thermophilus HB8]
>gi|55980115|ref|YP_143412.1| alanyl-tRNA synthetase-related protein [Thermus thermophilus HB8]
>gi|55980123|ref|YP_143420.1| hypothetical protein TTHA0154 [Thermus thermophilus HB8]
>gi|55980124|ref|YP_143421.1| hypothetical protein TTHA0155 [Thermus thermophilus HB8]
>gi|55980129|ref|YP_143426.1| hypothetical protein TTHA0160 [Thermus thermophilus HB8]
>gi|55980132|ref|YP_143429.1| hypothetical protein TTHA0163 [Thermus thermophilus HB8]
>gi|55980134|ref|YP_143431.1| hypothetical protein TTHA0165 [Thermus thermophilus HB8]
>gi|55980135|ref|YP_143432.1| hypothetical protein TTHA0166 [Thermus thermophilus HB8]
>gi|55980143|ref|YP_143440.1| alternative chaperone protein DnaJ [Thermus thermophilus HB8]
>gi|55980147|ref|YP_143444.1| hypothetical protein TTHA0178 [Thermus thermophilus HB8]
>gi|55980148|ref|YP_143445.1| hypothetical protein TTHA0179 [Thermus thermophilus HB8]
>gi|55980151|ref|YP_143448.1| hypothetical protein TTHA0182 [Thermus thermophilus HB8]
>gi|55980153|ref|YP_143450.1| pyruvate dehydrogenase complex, dihydrolipoamide acetyltransferase E2 component [Thermus thermophilus HB8]
>gi|55980155|ref|YP_143452.1| probable transcriptional regulator [Thermus thermophilus HB8]
>gi|55980162|ref|YP_143459.1| putative membrane protein [Thermus thermophilus HB8]
>gi|55980164|ref|YP_143461.1| probable tripartite transporter, large subunit [Thermus thermophilus HB8]
>gi|55980167|ref|YP_143464.1| ribonuclease HII [Thermus thermophilus HB8]
>gi|55980171|ref|YP_143468.1| hypothetical protein TTHA0202 [Thermus thermophilus HB8]
>gi|55980172|ref|YP_143469.1| hypothetical protein TTHA0203 [Thermus thermophilus HB8]
>gi|55980173|ref|YP_143470.1| conserved hypothetical protein, integral membrane protein [Thermus thermophilus HB8]
>gi|55980174|ref|YP_143471.1| hypothetical protein TTHA0205 [Thermus thermophilus HB8]
>gi|55980179|ref|YP_143476.1| 50S ribosomal protein L12 [Thermus thermophilus HB8]
>gi|55980180|ref|YP_143477.1| lipoprotein releasing system transmembrane protein [Thermus thermophilus HB8]
>gi|55980183|ref|YP_143480.1| probable kinase [Thermus thermophilus HB8]
>gi|55980187|ref|YP_143484.1| enoyl-CoA hydratase [Thermus thermophilus HB8]
>gi|55980192|ref|YP_143489.1| hypothetical protein TTHA0223 [Thermus thermophilus HB8]
>gi|55980193|ref|YP_143490.1| glucose-1-phosphate thymidylyltransferase [Thermus thermophilus HB8]
>gi|55980194|ref|YP_143491.1| biotin operon repressor/biotin-[acetyl-CoA-carboxylase] synthetase [Thermus thermophilus HB8]
>gi|55980204|ref|YP_143501.1| hypothetical protein TTHA0235 [Thermus thermophilus HB8]
>gi|55980205|ref|YP_143502.1| hypothetical protein TTHA0236 [Thermus thermophilus HB8]
>gi|55980207|ref|YP_143504.1| hypothetical protein TTHA0238 [Thermus thermophilus HB8]
>gi|55980210|ref|YP_143507.1| oxidoreductase, short-chain dehydrogenase/reductase family [Thermus thermophilus HB8]
>gi|55980211|ref|YP_143508.1| 50S ribosomal protein L9 [Thermus thermophilus HB8]
>gi|55980223|ref|YP_143520.1| hypothetical protein TTHA0254 [Thermus thermophilus HB8]
>gi|55980225|ref|YP_143522.1| leucine aminopeptidase [Thermus thermophilus HB8]
>gi|55980232|ref|YP_143529.1| multidrug-efflux transporter [Thermus thermophilus HB8]
>gi|55980233|ref|YP_143530.1| RecF protein [Thermus thermophilus HB8]
>gi|55980235|ref|YP_143532.1| cell wall-binding endopeptidase-related protein [Thermus thermophilus HB8]
>gi|55980236|ref|YP_143533.1| oxygen-insensitive NADPH nitroreductase [Thermus thermophilus HB8]
>gi|55980238|ref|YP_143535.1| type IV prepilin peptidase (PilD) [Thermus thermophilus HB8]
>gi|55980242|ref|YP_143539.1| hypothetical protein (transposase related protein) [Thermus thermophilus HB8]
>gi|55980244|ref|YP_143541.1| RNA 2'-O ribose methyltransferase [Thermus thermophilus HB8]
>gi|55980248|ref|YP_143545.1| putative threonine dehydratase [Thermus thermophilus HB8]
>gi|55980252|ref|YP_143549.1| argininosuccinate lyase [Thermus thermophilus HB8]
>gi|55980257|ref|YP_143554.1| 2-oxoglutarate dehydrogenase E2 component (dihydrolipoamide succinyltransferase) [Thermus thermophilus HB8]
>gi|55980259|ref|YP_143556.1| probable enoyl-CoA hydratase [Thermus thermophilus HB8]
>gi|55980260|ref|YP_143557.1| Holliday junction DNA helicase RuvA [Thermus thermophilus HB8]
>gi|55980261|ref|YP_143558.1| probable hydrolase [Thermus thermophilus HB8]
>gi|55980262|ref|YP_143559.1| hypothetical protein TTHA0293 [Thermus thermophilus HB8]
>gi|55980265|ref|YP_143562.1| RNA methyltransferase, TrmH family [Thermus thermophilus HB8]
>gi|55980268|ref|YP_143565.1| glucokinase [Thermus thermophilus HB8]
>gi|55980269|ref|YP_143566.1| hypothetical protein TTHA0300 [Thermus thermophilus HB8]
>gi|55980270|ref|YP_143567.1| hypothetical protein TTHA0301 [Thermus thermophilus HB8]
>gi|55980272|ref|YP_143569.1| hypothetical protein TTHA0303 [Thermus thermophilus HB8]
>gi|55980278|ref|YP_143575.1| hypothetical protein TTHA0309 [Thermus thermophilus HB8]
>gi|55980279|ref|YP_143576.1| cytochrome c oxidase assembly factor (CtaA) + protoheme IX farnesyltransferase (CtaB) [Thermus thermophilus HB8]
>gi|55980282|ref|YP_143579.1| hypothetical protein TTHA0313 [Thermus thermophilus HB8]
>gi|55980283|ref|YP_143580.1| conserved hypothetical membrane protein [Thermus thermophilus HB8]
>gi|55980284|ref|YP_143581.1| hypothetical protein TTHA0315 [Thermus thermophilus HB8]
>gi|55980287|ref|YP_143584.1| hypothetical protein TTHA0318 [Thermus thermophilus HB8]
>gi|55980288|ref|YP_143585.1| hypothetical protein TTHA0319 [Thermus thermophilus HB8]
>gi|55980289|ref|YP_143586.1| methionyl-tRNA formyltransferase [Thermus thermophilus HB8]
>gi|55980291|ref|YP_143588.1| hypothetical protein TTHA0322 [Thermus thermophilus HB8]
>gi|55980292|ref|YP_143589.1| hypothetical protein TTHA0323 [Thermus thermophilus HB8]
>gi|55980294|ref|YP_143591.1| hypothetical protein TTHA0325 [Thermus thermophilus HB8]

>gi|55980299|ref|YP_143596.1| hypothetical protein TTHA0330 [Thermus thermophilus HB8]
>gi|55980301|ref|YP_143598.1| hypothetical protein TTHA0332 [Thermus thermophilus HB8]
>gi|55980304|ref|YP_143601.1| hypothetical protein TTHA0335 [Thermus thermophilus HB8]
>gi|55980306|ref|YP_143603.1| hypothetical protein TTHA0337 [Thermus thermophilus HB8]
>gi|55980307|ref|YP_143604.1| hypothetical protein TTHA0338 [Thermus thermophilus HB8]
>gi|55980308|ref|YP_143605.1| hypothetical protein TTHA0339 [Thermus thermophilus HB8]
>gi|55980309|ref|YP_143606.1| hypothetical protein TTHA0340 [Thermus thermophilus HB8]
>gi|55980312|ref|YP_143609.1| folyl-polyglutamate synthetase [Thermus thermophilus HB8]
>gi|55980317|ref|YP_143614.1| hypothetical protein TTHA0348 [Thermus thermophilus HB8]
>gi|55980319|ref|YP_143616.1| hypothetical protein TTHA0350 [Thermus thermophilus HB8]
>gi|55980320|ref|YP_143617.1| probable efflux protein (fosmidomycin resistance) [Thermus thermophilus HB8]
>gi|55980327|ref|YP_143624.1| hypothetical protein TTHA0358 [Thermus thermophilus HB8]
>gi|55980335|ref|YP_143632.1| aspartyl/glutamyl-tRNA(Asn/Gln) amidotransferase subunit B [Thermus thermophilus HB8]
>gi|55980340|ref|YP_143637.1| probable serine/threonine-protein kinase [Thermus thermophilus HB8]
>gi|55980344|ref|YP_143641.1| putative glycoprotein endopeptidase [Thermus thermophilus HB8]
>gi|55980345|ref|YP_143642.1| sugar ABC transporter, permease protein [Thermus thermophilus HB8]
>gi|55980350|ref|YP_143647.1| hypothetical protein TTHA0381 [Thermus thermophilus HB8]
>gi|55980351|ref|YP_143648.1| hypothetical protein TTHA0382 [Thermus thermophilus HB8]
>gi|55980355|ref|YP_143652.1| Sec-independent protein translocase protein TatA [Thermus thermophilus HB8]
>gi|55980356|ref|YP_143653.1| Sec-independent protein translocase protein TatC [Thermus thermophilus HB8]
>gi|55980361|ref|YP_143658.1| probable methylmalonyl-CoA epimerase [Thermus thermophilus HB8]
>gi|55980362|ref|YP_143659.1| hypothetical protein TTHA0393 [Thermus thermophilus HB8]
>gi|55980366|ref|YP_143663.1| hypothetical protein TTHA0397 [Thermus thermophilus HB8]
>gi|55980367|ref|YP_143664.1| hypothetical protein TTHA0398 [Thermus thermophilus HB8]
>gi|55980368|ref|YP_143665.1| hypothetical protein TTHA0399 [Thermus thermophilus HB8]
>gi|55980371|ref|YP_143668.1| hypothetical protein TTHA0402 [Thermus thermophilus HB8]
>gi|55980373|ref|YP_143670.1| hypothetical protein TTHA0404 [Thermus thermophilus HB8]
>gi|55980374|ref|YP_143671.1| hypothetical protein TTHA0405 [Thermus thermophilus HB8]
>gi|55980381|ref|YP_143678.1| deoxyguanosinetriphosphate triphosphohydrolase, putative [Thermus thermophilus HB8]
>gi|55980390|ref|YP_143687.1| hypothetical protein TTHA0421 [Thermus thermophilus HB8]
>gi|55980392|ref|YP_143689.1| hypothetical protein TTHA0423 [Thermus thermophilus HB8]
>gi|55980393|ref|YP_143690.1| thiamin-monophosphate kinase [Thermus thermophilus HB8]
>gi|55980394|ref|YP_143691.1| NADH oxidase (H2O2-forming) [Thermus thermophilus HB8]
>gi|55980396|ref|YP_143693.1| hypothetical protein TTHA0427 [Thermus thermophilus HB8]
>gi|55980398|ref|YP_143695.1| Imidazoleglycerol-phosphate dehydratase [Thermus thermophilus HB8]
>gi|55980402|ref|YP_143699.1| hypothetical protein TTHA0433 [Thermus thermophilus HB8]
>gi|55980403|ref|YP_143700.1| hypothetical protein TTHA0434 [Thermus thermophilus HB8]
>gi|55980406|ref|YP_143703.1| hypothetical protein TTHA0437 [Thermus thermophilus HB8]
>gi|55980409|ref|YP_143706.1| hypothetical protein TTHA0440 [Thermus thermophilus HB8]
>gi|55980410|ref|YP_143707.1| methyltransferase, HemK family [Thermus thermophilus HB8]
>gi|55980411|ref|YP_143708.1| probable DNA/RNA-binding protein (Jag-related protein) [Thermus thermophilus HB8]
>gi|55980418|ref|YP_143715.1| branched-chain amino acid ABC transporter, permease protein [Thermus thermophilus HB8]
>gi|55980423|ref|YP_143720.1| Mn2+/Zn2+ ABC transporter, permease protein [Thermus thermophilus HB8]
>gi|55980425|ref|YP_143722.1| cysteine desulfurase/cysteine sulfinate desulfinase [Thermus thermophilus HB8]
>gi|55980427|ref|YP_143724.1| cytidylate kinase [Thermus thermophilus HB8]
>gi|55980428|ref|YP_143725.1| hypothetical protein TTHA0459 [Thermus thermophilus HB8]
>gi|55980433|ref|YP_143730.1| hypothetical protein TTHA0464 [Thermus thermophilus HB8]
>gi|55980436|ref|YP_143733.1| hypothetical protein TTHA0467 [Thermus thermophilus HB8]
>gi|55980437|ref|YP_143734.1| hypothetical protein TTHA0468 [Thermus thermophilus HB8]
>gi|55980440|ref|YP_143737.1| peptide ABC transporter, permease protein [Thermus thermophilus HB8]
>gi|55980443|ref|YP_143740.1| acetoin utilization protein AcuB (acetoin dehydrogenase) [Thermus thermophilus HB8]
>gi|55980445|ref|YP_143742.1| hypothetical protein TTHA0476 [Thermus thermophilus HB8]
>gi|55980448|ref|YP_143745.1| trehalose-6-phosphate phosphatase [Thermus thermophilus HB8]
>gi|55980453|ref|YP_143750.1| apolipoprotein N-acyltransferase [Thermus thermophilus HB8]
>gi|55980456|ref|YP_143753.1| hypothetical protein TTHA0487 [Thermus thermophilus HB8]
>gi|55980458|ref|YP_143755.1| homoserine dehydrogenase [Thermus thermophilus HB8]
>gi|55980462|ref|YP_143759.1| hypothetical protein TTHA0493 [Thermus thermophilus HB8]
>gi|55980463|ref|YP_143760.1| hypothetical protein TTHA0494 [Thermus thermophilus HB8]
>gi|55980467|ref|YP_143764.1| hypothetical protein TTHA0498 [Thermus thermophilus HB8]
>gi|55980473|ref|YP_143770.1| glycolate oxidase subunit GlcE [Thermus thermophilus HB8]
>gi|55980483|ref|YP_143780.1| phosphoribosylaminoimidazole carboxylase, catalytic subunit (PurE) [Thermus thermophilus HB8]
>gi|55980484|ref|YP_143781.1| hypothetical protein TTHA0515 [Thermus thermophilus HB8]
>gi|55980486|ref|YP_143783.1| DedA family protein [Thermus thermophilus HB8]
>gi|55980490|ref|YP_143787.1| hypothetical protein TTHA0521 [Thermus thermophilus HB8]
>gi|55980496|ref|YP_143793.1| riboflavin kinase/FMN adenylyltransferase [Thermus thermophilus HB8]
>gi|55980500|ref|YP_143797.1| hypothetical protein TTHA0531 [Thermus thermophilus HB8]
>gi|55980509|ref|YP_143806.1| hypothetical protein TTHA0540 [Thermus thermophilus HB8]
>gi|55980510|ref|YP_143807.1| DNA repair protein RadA [Thermus thermophilus HB8]
>gi|55980513|ref|YP_143810.1| hypothetical protein TTHA0544 [Thermus thermophilus HB8]
>gi|55980515|ref|YP_143812.1| hemolysin [Thermus thermophilus HB8]

>gi|55980517|ref|YP_143814.1| hypothetical protein TTHA0548 [Thermus thermophilus HB8]
>gi|55980523|ref|YP_143820.1| small multidrug export protein [Thermus thermophilus HB8]
>gi|55980524|ref|YP_143821.1| hypothetical protein TTHA0555 [Thermus thermophilus HB8]
>gi|55980525|ref|YP_143822.1| hypothetical protein TTHA0556 [Thermus thermophilus HB8]
>gi|55980533|ref|YP_143830.1| cell division initiation protein DivIVA [Thermus thermophilus HB8]
>gi|55980537|ref|YP_143834.1| hypothetical protein TTHA0568 [Thermus thermophilus HB8]
>gi|55980543|ref|YP_143840.1| probable DNA polymerase III epsilon subunit [Thermus thermophilus HB8]
>gi|55980544|ref|YP_143841.1| hypothetical protein TTHA0575 [Thermus thermophilus HB8]
>gi|55980545|ref|YP_143842.1| putative pyruvyl-transferase [Thermus thermophilus HB8]
>gi|55980546|ref|YP_143843.1| hypothetical protein TTHA0577 [Thermus thermophilus HB8]
>gi|55980549|ref|YP_143846.1| hypothetical protein TTHA0580 [Thermus thermophilus HB8]
>gi|55980550|ref|YP_143847.1| hypothetical protein TTHA0581 [Thermus thermophilus HB8]
>gi|55980554|ref|YP_143851.1| branched-chain amino acid ABC transporter, permease protein [Thermus thermophilus HB8]
>gi|55980556|ref|YP_143853.1| branched-chain amino acid ABC transporter, ATP-binding protein [Thermus thermophilus HB8]
>gi|55980558|ref|YP_143855.1| branched-chain amino acid ABC transporter permease protein [Thermus thermophilus HB8]
>gi|55980561|ref|YP_143858.1| hypothetical protein TTHA0592 [Thermus thermophilus HB8]
>gi|55980567|ref|YP_143864.1| putative transport integral membrane protein (chloride channel) [Thermus thermophilus HB8]
>gi|55980571|ref|YP_143868.1| peptidyl-prolyl cis-trans isomerase [Thermus thermophilus HB8]
>gi|55980572|ref|YP_143869.1| hypothetical protein TTHA0603 [Thermus thermophilus HB8]
>gi|55980574|ref|YP_143871.1| 4-hydroxybenzoate octaprenyltransferase [Thermus thermophilus HB8]
>gi|55980583|ref|YP_143880.1| trigger factor [Thermus thermophilus HB8]
>gi|55980590|ref|YP_143887.1| putative 4-amino-4-deoxychorismate lyase [Thermus thermophilus HB8]
>gi|55980592|ref|YP_143889.1| DNA repair protein RecO [Thermus thermophilus HB8]
>gi|55980593|ref|YP_143890.1| hypothetical protein TTHA0624 [Thermus thermophilus HB8]
>gi|55980595|ref|YP_143892.1| phosphoenolpyruvate carboxylase [Thermus thermophilus HB8]
>gi|55980598|ref|YP_143895.1| putative O-linked GlcNAc transferase (TPR repeat) [Thermus thermophilus HB8]
>gi|55980604|ref|YP_143901.1| probable nucleotidyltransferase [Thermus thermophilus HB8]
>gi|55980610|ref|YP_143907.1| hypothetical protein TTHA0641 [Thermus thermophilus HB8]
>gi|55980611|ref|YP_143908.1| putative glycosyltransferase [Thermus thermophilus HB8]
>gi|55980612|ref|YP_143909.1| putative glycosyltransferase [Thermus thermophilus HB8]
>gi|55980615|ref|YP_143912.1| hypothetical protein TTHA0646 [Thermus thermophilus HB8]
>gi|55980618|ref|YP_143915.1| putative O-antigen transporter [Thermus thermophilus HB8]
>gi|55980622|ref|YP_143919.1| hypothetical protein TTHA0653 [Thermus thermophilus HB8]
>gi|55980624|ref|YP_143921.1| hypothetical protein TTHA0655 [Thermus thermophilus HB8]
>gi|55980627|ref|YP_143924.1| hypothetical protein TTHA0658 [Thermus thermophilus HB8]
>gi|55980628|ref|YP_143925.1| hypothetical protein TTHA0659 [Thermus thermophilus HB8]
>gi|55980630|ref|YP_143927.1| hypothetical protein TTHA0661 [Thermus thermophilus HB8]
>gi|55980633|ref|YP_143930.1| NADPH quinone oxidoreductase [Thermus thermophilus HB8]
>gi|55980637|ref|YP_143934.1| hypothetical protein TTHA0668 [Thermus thermophilus HB8]
>gi|55980640|ref|YP_143937.1| probable uroporphyrinogen-III synthase [Thermus thermophilus HB8]
>gi|55980642|ref|YP_143939.1| hypothetical protein TTHA0673 [Thermus thermophilus HB8]
>gi|55980646|ref|YP_143943.1| putative oxidoreductase [Thermus thermophilus HB8]
>gi|55980648|ref|YP_143945.1| putative transport protein [Thermus thermophilus HB8]
>gi|55980649|ref|YP_143946.1| phosphomethylpyrimidine kinase (ThiD) [Thermus thermophilus HB8]
>gi|55980651|ref|YP_143948.1| hypothetical protein TTHA0682 [Thermus thermophilus HB8]
>gi|55980652|ref|YP_143949.1| probable TolR-type transport protein [Thermus thermophilus HB8]
>gi|55980656|ref|YP_143953.1| sugar ABC transporter, permease protein [N-terminal] [Thermus thermophilus HB8]
>gi|55980658|ref|YP_143955.1| putative xylose repressor [C-terminal] [Thermus thermophilus HB8]
>gi|55980663|ref|YP_143960.1| branched-chain amino acid ABC transporter, ATP-binding protein [Thermus thermophilus HB8]
>gi|55980664|ref|YP_143961.1| amino acid ABC transporter ATP-binding protein [C-terminal] [Thermus thermophilus HB8]
>gi|55980665|ref|YP_143962.1| hypothetical protein TTHA0696 [Thermus thermophilus HB8]
>gi|55980667|ref|YP_143964.1| hypothetical protein TTHA0698 [Thermus thermophilus HB8]
>gi|55980674|ref|YP_143971.1| transcriptional repressor SmtB [Thermus thermophilus HB8]
>gi|55980675|ref|YP_143972.1| cation-transporting ATPase [Thermus thermophilus HB8]
>gi|55980676|ref|YP_143973.1| glutamine amidotransferase (pyridoxine biosynthesis) [Thermus thermophilus HB8]
>gi|55980678|ref|YP_143975.1| putative hydrolase [Thermus thermophilus HB8]
>gi|55980681|ref|YP_143978.1| histidyl-tRNA synthetase [Thermus thermophilus HB8]
>gi|55980682|ref|YP_143979.1| hypothetical protein TTHA0713 [Thermus thermophilus HB8]
>gi|55980683|ref|YP_143980.1| hypothetical protein TTHA0714 [Thermus thermophilus HB8]
>gi|55980684|ref|YP_143981.1| ABC transporter ATP-binding protein [Thermus thermophilus HB8]
>gi|55980685|ref|YP_143982.1| molybdenum ABC transporter, permease protein [Thermus thermophilus HB8]
>gi|55980686|ref|YP_143983.1| molybdenum ABC transporter molybdate-binding protein [Thermus thermophilus HB8]
>gi|55980688|ref|YP_143985.1| hypothetical protein TTHA0719 [Thermus thermophilus HB8]
>gi|55980689|ref|YP_143986.1| hypothetical protein TTHA0720 [Thermus thermophilus HB8]
>gi|55980690|ref|YP_143987.1| hypothetical protein TTHA0721 [Thermus thermophilus HB8]
>gi|55980694|ref|YP_143991.1| membrane-bound protein LytR [Thermus thermophilus HB8]
>gi|55980697|ref|YP_143994.1| hypothetical protein TTHA0728 [Thermus thermophilus HB8]
>gi|55980698|ref|YP_143995.1| probable efflux transporter, AcrB/AcrD/AcrF family [Thermus thermophilus HB8]
>gi|55980699|ref|YP_143996.1| hypothetical protein TTHA0730 [Thermus thermophilus HB8]
>gi|55980700|ref|YP_143997.1| hypothetical protein TTHA0731 [Thermus thermophilus HB8]

>gi|55980701|ref|YP_143998.1| hypothetical protein TTHA0732 [Thermus thermophilus HB8]
>gi|55980702|ref|YP_143999.1| transcriptional regulator MarR family [Thermus thermophilus HB8]
>gi|55980703|ref|YP_144000.1| hemolysin-related protein [Thermus thermophilus HB8]
>gi|55980705|ref|YP_144002.1| hypothetical protein TTHA0736 [Thermus thermophilus HB8]
>gi|55980707|ref|YP_144004.1| hypothetical protein TTHA0738 [Thermus thermophilus HB8]
>gi|55980709|ref|YP_144006.1| sensor histidine kinase [Thermus thermophilus HB8]
>gi|55980711|ref|YP_144008.1| hypothetical protein TTHA0742 [Thermus thermophilus HB8]
>gi|55980713|ref|YP_144010.1| hypothetical protein TTHA0744 [Thermus thermophilus HB8]
>gi|55980714|ref|YP_144011.1| hypothetical protein TTHA0745 [Thermus thermophilus HB8]
>gi|55980715|ref|YP_144012.1| iron ABC transporter, substrate-binding protein [Thermus thermophilus HB8]
>gi|55980716|ref|YP_144013.1| iron ABC transporter, permease protein [Thermus thermophilus HB8]
>gi|55980717|ref|YP_144014.1| iron ABC transporter, ATP-binding protein [Thermus thermophilus HB8]
>gi|55980720|ref|YP_144017.1| hypothetical protein TTHA0751 [Thermus thermophilus HB8]
>gi|55980730|ref|YP_144027.1| iron-sulfur cluster biosynthesis protein SufE [Thermus thermophilus HB8]
>gi|55980732|ref|YP_144029.1| zinc-dependent dehydrogenase [Thermus thermophilus HB8]
>gi|55980734|ref|YP_144031.1| probable hydrolase [Thermus thermophilus HB8]
>gi|55980744|ref|YP_144041.1| hypothetical protein TTHA0775 [Thermus thermophilus HB8]
>gi|55980746|ref|YP_144043.1| hypothetical protein TTHA0777 [Thermus thermophilus HB8]
>gi|55980750|ref|YP_144047.1| dihydroorotase (PyrC) [Thermus thermophilus HB8]
>gi|55980753|ref|YP_144050.1| hypothetical protein TTHA0784 [Thermus thermophilus HB8]
>gi|55980754|ref|YP_144051.1| hypothetical protein TTHA0785 [Thermus thermophilus HB8]
>gi|55980757|ref|YP_144054.1| DNA polymerase III, delta subunit [Thermus thermophilus HB8]
>gi|55980759|ref|YP_144056.1| putative acetyltransferase [Thermus thermophilus HB8]
>gi|55980760|ref|YP_144057.1| hypothetical protein TTHA0791 [Thermus thermophilus HB8]
>gi|55980761|ref|YP_144058.1| hypothetical protein TTHA0792 [Thermus thermophilus HB8]
>gi|55980766|ref|YP_144063.1| (s)-2-hydroxy-acid oxidase subunit (GlcD) [Thermus thermophilus HB8]
>gi|55980767|ref|YP_144064.1| GGDEF domain protein [Thermus thermophilus HB8]
>gi|55980772|ref|YP_144069.1| hypothetical protein TTHA0803 [Thermus thermophilus HB8]
>gi|55980775|ref|YP_144072.1| hypothetical protein TTHA0806 [Thermus thermophilus HB8]
>gi|55980780|ref|YP_144077.1| phosphoribosylglycinamide formyltransferase (PurD) [Thermus thermophilus HB8]
>gi|55980782|ref|YP_144079.1| DNA polymerase III epsilon chain-like protein [Thermus thermophilus HB8]
>gi|55980783|ref|YP_144080.1| putative response regulator [Thermus thermophilus HB8]
>gi|55980784|ref|YP_144081.1| sensor histidine kinase [Thermus thermophilus HB8]
>gi|55980785|ref|YP_144082.1| hypothetical protein TTHA0816 [Thermus thermophilus HB8]
>gi|55980787|ref|YP_144084.1| hypothetical protein TTHA0818 [Thermus thermophilus HB8]
>gi|55980791|ref|YP_144088.1| probable potassium efflux transporter [Thermus thermophilus HB8]
>gi|55980795|ref|YP_144092.1| cell division protein FtsK [Thermus thermophilus HB8]
>gi|55980797|ref|YP_144094.1| hypothetical protein TTHA0828 [Thermus thermophilus HB8]
>gi|55980799|ref|YP_144096.1| hypothetical protein TTHA0830 [Thermus thermophilus HB8]
>gi|55980800|ref|YP_144097.1| polyA polymerase family protein [Thermus thermophilus HB8]
>gi|55980804|ref|YP_144101.1| conserved hypothetical membrane protein [Thermus thermophilus HB8]
>gi|55980809|ref|YP_144106.1| probable thiol:disulfide interchange protein [Thermus thermophilus HB8]
>gi|55980811|ref|YP_144108.1| hypothetical protein TTHA0842 [Thermus thermophilus HB8]
>gi|55980813|ref|YP_144110.1| CAAX amino terminal protease family protein [Thermus thermophilus HB8]
>gi|55980815|ref|YP_144112.1| metallo-beta-lactamase family protein [Thermus thermophilus HB8]
>gi|55980817|ref|YP_144114.1| RecX protein [Thermus thermophilus HB8]
>gi|55980819|ref|YP_144116.1| stage V sporulation protein S (SpoVS) related protein [Thermus thermophilus HB8]
>gi|55980820|ref|YP_144117.1| Sun protein (RNA methyltransferase) [Thermus thermophilus HB8]
>gi|55980822|ref|YP_144119.1| hypothetical protein TTHA0853 [Thermus thermophilus HB8]
>gi|55980824|ref|YP_144121.1| membrane-associated Zn-dependent protease [Thermus thermophilus HB8]
>gi|55980825|ref|YP_144122.1| 1-deoxy-D-xylulose-5-phosphate reductoisomerase [Thermus thermophilus HB8]
>gi|55980839|ref|YP_144136.1| hypothetical protein TTHA0870 [Thermus thermophilus HB8]
>gi|55980840|ref|YP_144137.1| hypothetical protein TTHA0871 [Thermus thermophilus HB8]
>gi|55980845|ref|YP_144142.1| glutamyl-tRNA(Gln) amidotransferase subunit C [Thermus thermophilus HB8]
>gi|55980846|ref|YP_144143.1| hypothetical protein TTHA0877 [Thermus thermophilus HB8]
>gi|55980847|ref|YP_144144.1| hypothetical protein TTHA0878 [Thermus thermophilus HB8]
>gi|55980849|ref|YP_144146.1| multidrug resistance protein-related protein [Thermus thermophilus HB8]
>gi|55980856|ref|YP_144153.1| glycosyltransferase [Thermus thermophilus HB8]
>gi|55980863|ref|YP_144160.1| 33 kDa chaperonin (Heat shock protein 33 homolog) (HSP33) [Thermus thermophilus HB8]
>gi|55980864|ref|YP_144161.1| universal stress protein family [Thermus thermophilus HB8]
>gi|55980865|ref|YP_144162.1| Zn-dependent protease [Thermus thermophilus HB8]
>gi|55980866|ref|YP_144163.1| hypothetical protein TTHA0897 [Thermus thermophilus HB8]
>gi|55980868|ref|YP_144165.1| chromosome segregation SMC protein [Thermus thermophilus HB8]
>gi|55980870|ref|YP_144167.1| hypothetical protein TTHA0901 [Thermus thermophilus HB8]
>gi|55980872|ref|YP_144169.1| probable hydrolase [Thermus thermophilus HB8]
>gi|55980873|ref|YP_144170.1| biotin biosynthesis protein BioY [Thermus thermophilus HB8]
>gi|55980876|ref|YP_144173.1| ribosome-binding factor A [Thermus thermophilus HB8]
>gi|55980880|ref|YP_144177.1| hypothetical protein TTHA0911 [Thermus thermophilus HB8]
>gi|55980885|ref|YP_144182.1| hypothetical protein TTHA0916 [Thermus thermophilus HB8]
>gi|55980889|ref|YP_144186.1| hypothetical protein TTHA0920 [Thermus thermophilus HB8]

>gi|55980893|ref|YP_144190.1| hypothetical protein TTHA0924 [Thermus thermophilus HB8]
>gi|55980894|ref|YP_144191.1| poly(A) polymerase family protein [Thermus thermophilus HB8]
>gi|55980895|ref|YP_144192.1| dephospho-CoA kinase (dephosphocoenzyme A kinase) [Thermus thermophilus HB8]
>gi|55980896|ref|YP_144193.1| hypothetical protein TTHA0927 [Thermus thermophilus HB8]
>gi|55980900|ref|YP_144197.1| thiophene and furan oxidation protein [Thermus thermophilus HB8]
>gi|55980902|ref|YP_144199.1| hypothetical protein TTHA0933 [Thermus thermophilus HB8]
>gi|55980904|ref|YP_144201.1| quaternary ammonium compound-resistance protein [Thermus thermophilus HB8]
>gi|55980905|ref|YP_144202.1| CBS domain protein [Thermus thermophilus HB8]
>gi|55980907|ref|YP_144204.1| pyruvate dehydrogenase E1 component, beta subunit [Thermus thermophilus HB8]
>gi|55980910|ref|YP_144207.1| ABC transporter ATP-binding protein [Thermus thermophilus HB8]
>gi|55980911|ref|YP_144208.1| hypothetical protein TTHA0942 [Thermus thermophilus HB8]
>gi|55980914|ref|YP_144211.1| hypothetical protein TTHA0945 [Thermus thermophilus HB8]
>gi|55980924|ref|YP_144221.1| mannosyl-3-phosphoglycerate phosphatase [Thermus thermophilus HB8]
>gi|55980925|ref|YP_144222.1| hypothetical protein TTHA0956 [Thermus thermophilus HB8]
>gi|55980930|ref|YP_144227.1| hypothetical protein TTHA0961 [Thermus thermophilus HB8]
>gi|55980932|ref|YP_144229.1| ABC tranpsorter ATP binding protein related protein [Thermus thermophilus HB8]
>gi|55980938|ref|YP_144235.1| phenylacetic acid degradation protein PaaD [Thermus thermophilus HB8]
>gi|55980952|ref|YP_144249.1| L-aspartate oxidase [Thermus thermophilus HB8]
>gi|55980954|ref|YP_144251.1| nicotinate-nucleotide pyrophosphorylase [carboxylating] [Thermus thermophilus HB8]
>gi|55980957|ref|YP_144254.1| hypothetical protein TTHA0988 [Thermus thermophilus HB8]
>gi|55980958|ref|YP_144255.1| GGDEF domain protein [Thermus thermophilus HB8]
>gi|55980959|ref|YP_144256.1| hypothetical protein TTHA0990 [Thermus thermophilus HB8]
>gi|55980961|ref|YP_144258.1| putative fructokinase [Thermus thermophilus HB8]
>gi|55980967|ref|YP_144264.1| ATP-dependent DNA helicase [Thermus thermophilus HB8]
>gi|55980968|ref|YP_144265.1| hypothetical protein TTHA0999 [Thermus thermophilus HB8]
>gi|55980969|ref|YP_144266.1| hypothetical protein TTHA1000 [Thermus thermophilus HB8]
>gi|55980970|ref|YP_144267.1| hypothetical protein TTHA1001 [Thermus thermophilus HB8]
>gi|55980972|ref|YP_144269.1| sensor histidine kinase [Thermus thermophilus HB8]
>gi|55980973|ref|YP_144270.1| conserved hypothetical membrane protein [Thermus thermophilus HB8]
>gi|55980974|ref|YP_144271.1| hypothetical protein TTHA1005 [Thermus thermophilus HB8]
>gi|55980975|ref|YP_144272.1| hypothetical protein TTHA1006 [Thermus thermophilus HB8]
>gi|55980978|ref|YP_144275.1| hypothetical protein TTHA1009 [Thermus thermophilus HB8]
>gi|55980980|ref|YP_144277.1| hypothetical protein TTHA1011 [Thermus thermophilus HB8]
>gi|55980982|ref|YP_144279.1| hypothetical protein TTHA1013 [Thermus thermophilus HB8]
>gi|55980992|ref|YP_144289.1| hypothetical protein TTHA1023 [Thermus thermophilus HB8]
>gi|55981000|ref|YP_144297.1| 50S ribosomal protein L19 [Thermus thermophilus HB8]
>gi|55981003|ref|YP_144300.1| hypothetical protein TTHA1034 [Thermus thermophilus HB8]
>gi|55981009|ref|YP_144306.1| cation efflux system membrane protein [Thermus thermophilus HB8]
>gi|55981011|ref|YP_144308.1| anti-cleavage anti-GreA transcription factor Gfh1 [Thermus thermophilus HB8]
>gi|55981013|ref|YP_144310.1| diacylglycerol kinase [Thermus thermophilus HB8]
>gi|55981016|ref|YP_144313.1| hypothetical protein TTHA1047 [Thermus thermophilus HB8]
>gi|55981018|ref|YP_144315.1| hypothetical protein TTHA1049 [Thermus thermophilus HB8]
>gi|55981020|ref|YP_144317.1| hypothetical protein TTHA1051 [Thermus thermophilus HB8]
>gi|55981021|ref|YP_144318.1| hypothetical protein TTHA1052 [Thermus thermophilus HB8]
>gi|55981022|ref|YP_144319.1| hypothetical protein TTHA1053 [Thermus thermophilus HB8]
>gi|55981028|ref|YP_144325.1| ADP-ribosylglycohydrolase [Thermus thermophilus HB8]
>gi|55981030|ref|YP_144327.1| hypothetical protein TTHA1061 [Thermus thermophilus HB8]
>gi|55981033|ref|YP_144330.1| diaminohydroxyphosphoribosylaminopyrimidine deaminase + 5-amino-6-(5-phosphoribosylamino)uracil reductase (RibD) [Thermus thermophilus HB8]
>gi|55981037|ref|YP_144334.1| proline dipeptidase [Thermus thermophilus HB8]
>gi|55981038|ref|YP_144335.1| cell division protein FtsX [Thermus thermophilus HB8]
>gi|55981039|ref|YP_144336.1| hypothetical protein TTHA1070 [Thermus thermophilus HB8]
>gi|55981040|ref|YP_144337.1| competence protein ComF [Thermus thermophilus HB8]
>gi|55981043|ref|YP_144340.1| hypothetical protein TTHA1074 [Thermus thermophilus HB8]
>gi|55981047|ref|YP_144344.1| penicillin-binding protein [Thermus thermophilus HB8]
>gi|55981049|ref|YP_144346.1| hypothetical protein TTHA1080 [Thermus thermophilus HB8]
>gi|55981052|ref|YP_144349.1| cell division protein, FtsW/RodA/SpoVE family [Thermus thermophilus HB8]
>gi|55981053|ref|YP_144350.1| UDP-N-acetylglucosamine--N-acetylmuramyl- (pentapeptide) pyrophosphoryl-undecaprenol N-acetylglucosamine transferase [Thermus thermophilus HB8]
>gi|55981071|ref|YP_144368.1| hypothetical protein TTHA1102 [Thermus thermophilus HB8]
>gi|55981074|ref|YP_144371.1| hypothetical protein TTHA1105 [Thermus thermophilus HB8]
>gi|55981075|ref|YP_144372.1| hypothetical protein TTHA1106 [Thermus thermophilus HB8]
>gi|55981076|ref|YP_144373.1| hypothetical protein TTHA1107 [Thermus thermophilus HB8]
>gi|55981077|ref|YP_144374.1| hypothetical protein TTHA1108 [Thermus thermophilus HB8]
>gi|55981078|ref|YP_144375.1| putative glycosyltransferase [Thermus thermophilus HB8]
>gi|55981079|ref|YP_144376.1| hypothetical protein TTHA1110 [Thermus thermophilus HB8]
>gi|55981081|ref|YP_144378.1| hypothetical protein TTHA1112 [Thermus thermophilus HB8]
>gi|55981084|ref|YP_144381.1| integral membrane protein TerC family [Thermus thermophilus HB8]
>gi|55981087|ref|YP_144384.1| hypothetical protein TTHA1118 [Thermus thermophilus HB8]
>gi|55981088|ref|YP_144385.1| hypothetical protein TTHA1119 [Thermus thermophilus HB8]

>gi|55981093|ref|YP_144390.1| acetyl-CoA carboxylase, biotin carboxyl carrier protein [Thermus thermophilus HB8]
>gi|55981096|ref|YP_144393.1| hypothetical protein TTHA1127 [Thermus thermophilus HB8]
>gi|55981105|ref|YP_144402.1| hypothetical protein TTHA1136 [Thermus thermophilus HB8]
>gi|55981106|ref|YP_144403.1| major facilitator superfamily transporter [Thermus thermophilus HB8]
>gi|55981110|ref|YP_144407.1| cation-transporting ATPase [Thermus thermophilus HB8]
>gi|55981112|ref|YP_144409.1| sensor histidine kinase [Thermus thermophilus HB8]
>gi|55981114|ref|YP_144411.1| electron transfer flavoprotein, beta subunit [Thermus thermophilus HB8]
>gi|55981116|ref|YP_144413.1| hypothetical protein TTHA1147 [Thermus thermophilus HB8]
>gi|55981120|ref|YP_144417.1| hypothetical protein TTHA1151 [Thermus thermophilus HB8]
>gi|55981122|ref|YP_144419.1| mercuric reductase [Thermus thermophilus HB8]
>gi|55981124|ref|YP_144421.1| mercuric resistance operon regulatory protein (MerR) [Thermus thermophilus HB8]
>gi|55981127|ref|YP_144424.1| hypothetical protein TTHA1158 [Thermus thermophilus HB8]
>gi|55981129|ref|YP_144426.1| hypothetical protein TTHA1160 [Thermus thermophilus HB8]
>gi|55981130|ref|YP_144427.1| hypothetical protein TTHA1161 [Thermus thermophilus HB8]
>gi|55981131|ref|YP_144428.1| excisionase domain protein [Thermus thermophilus HB8]
>gi|55981133|ref|YP_144430.1| indole-3-glycerol phosphate synthase [Thermus thermophilus HB8]
>gi|55981134|ref|YP_144431.1| phosphoribosylformimino-5-aminoimidazole carboxamide ribotide isomerase [Thermus thermophilus HB8]
>gi|55981135|ref|YP_144432.1| hypothetical protein TTHA1166 [Thermus thermophilus HB8]
>gi|55981142|ref|YP_144439.1| Trk system potassium uptake protein (TrkG) [Thermus thermophilus HB8]
>gi|55981145|ref|YP_144442.1| hypothetical protein TTHA1176 [Thermus thermophilus HB8]
>gi|55981147|ref|YP_144444.1| hypothetical protein TTHA1178 [Thermus thermophilus HB8]
>gi|55981150|ref|YP_144447.1| sensor histidine kinase [Thermus thermophilus HB8]
>gi|55981154|ref|YP_144451.1| GTP-binding protein [Thermus thermophilus HB8]
>gi|55981158|ref|YP_144455.1| rod shape-determining protein MreC [Thermus thermophilus HB8]
>gi|55981159|ref|YP_144456.1| rod shape-determining protein MreD [Thermus thermophilus HB8]
>gi|55981161|ref|YP_144458.1| septum site-determining protein MinC [Thermus thermophilus HB8]
>gi|55981164|ref|YP_144461.1| hypothetical protein TTHA1195 [Thermus thermophilus HB8]
>gi|55981173|ref|YP_144470.1| hypothetical protein TTHA1204 [Thermus thermophilus HB8]
>gi|55981175|ref|YP_144472.1| hypothetical protein TTHA1206 [Thermus thermophilus HB8]
>gi|55981188|ref|YP_144485.1| probable general secretion pathway protein J [Thermus thermophilus HB8]
>gi|55981192|ref|YP_144489.1| hypothetical protein TTHA1223 [Thermus thermophilus HB8]
>gi|55981193|ref|YP_144490.1| hypothetical protein TTHA1224 [Thermus thermophilus HB8]
>gi|55981195|ref|YP_144492.1| hypothetical protein TTHA1226 [Thermus thermophilus HB8]
>gi|55981202|ref|YP_144499.1| short-chain dehydrogenases/reductases family protein [Thermus thermophilus HB8]
>gi|55981207|ref|YP_144504.1| spermidine/putrescine ABC transporter, permease protein [Thermus thermophilus HB8]
>gi|55981208|ref|YP_144505.1| spermidine/putrescine ABC transporter, permease protein [Thermus thermophilus HB8]
>gi|55981210|ref|YP_144507.1| rod shape determining protein RodA [Thermus thermophilus HB8]
>gi|55981214|ref|YP_144511.1| hypothetical protein TTHA1245 [Thermus thermophilus HB8]
>gi|55981222|ref|YP_144519.1| hypothetical protein TTHA1253 [Thermus thermophilus HB8]
>gi|55981228|ref|YP_144525.1| adenylate cyclase related protein [Thermus thermophilus HB8]
>gi|55981229|ref|YP_144526.1| DNA primase [Thermus thermophilus HB8]
>gi|55981232|ref|YP_144529.1| hypothetical protein TTHA1263 [Thermus thermophilus HB8]
>gi|55981236|ref|YP_144533.1| hypothetical protein TTHA1267 [Thermus thermophilus HB8]
>gi|55981243|ref|YP_144540.1| V-type ATP synthase subunit F [Thermus thermophilus HB8]
>gi|55981244|ref|YP_144541.1| V-type ATP synthase subunit [Thermus thermophilus HB8]
>gi|55981245|ref|YP_144542.1| V-type ATP synthase subunit E [Thermus thermophilus HB8]
>gi|55981246|ref|YP_144543.1| V-type ATP synthase, subunit K [Thermus thermophilus HB8]
>gi|55981247|ref|YP_144544.1| V-type ATP synthase subunit I [Thermus thermophilus HB8]
>gi|55981248|ref|YP_144545.1| V-type ATP synthase, subunit (VAPC-THERM) [Thermus thermophilus HB8]
>gi|55981250|ref|YP_144547.1| hypothetical protein TTHA1281 [Thermus thermophilus HB8]
>gi|55981251|ref|YP_144548.1| hypothetical protein TTHA1282 [Thermus thermophilus HB8]
>gi|55981255|ref|YP_144552.1| virulence factor-related protein [Thermus thermophilus HB8]
>gi|55981256|ref|YP_144553.1| hypothetical protein TTHA1287 [Thermus thermophilus HB8]
>gi|55981257|ref|YP_144554.1| exonuclease SbcC [Thermus thermophilus HB8]
>gi|55981259|ref|YP_144556.1| putative membrane protein [Thermus thermophilus HB8]
>gi|55981260|ref|YP_144557.1| putative membrane protein [C-terminal] [Thermus thermophilus HB8]
>gi|55981271|ref|YP_144568.1| sugar ABC transporter, permease protein [Thermus thermophilus HB8]
>gi|55981272|ref|YP_144569.1| sugar ABC transporter, permease protein [Thermus thermophilus HB8]
>gi|55981275|ref|YP_144572.1| hypothetical protein TTHA1306 [Thermus thermophilus HB8]
>gi|55981276|ref|YP_144573.1| hypothetical protein TTHA1307 [Thermus thermophilus HB8]
>gi|55981282|ref|YP_144579.1| hypothetical protein TTHA1313 [Thermus thermophilus HB8]
>gi|55981284|ref|YP_144581.1| putative integral membrane efflux protein [Thermus thermophilus HB8]
>gi|55981285|ref|YP_144582.1| hypothetical protein TTHA1316 [Thermus thermophilus HB8]
>gi|55981287|ref|YP_144584.1| hypothetical protein TTHA1318 [Thermus thermophilus HB8]
>gi|55981288|ref|YP_144585.1| probable acetyltransferase [Thermus thermophilus HB8]
>gi|55981292|ref|YP_144589.1| DNA mismatch repair protein MutL [Thermus thermophilus HB8]
>gi|55981293|ref|YP_144590.1| DNA mismatch repair protein MutS [Thermus thermophilus HB8]
>gi|55981296|ref|YP_144593.1| hypothetical protein TTHA1327 [Thermus thermophilus HB8]
>gi|55981301|ref|YP_144598.1| branched-chain amino acid ABC transporter, permease protein [Thermus thermophilus HB8]
>gi|55981302|ref|YP_144599.1| hypothetical protein TTHA1333 [Thermus thermophilus HB8]

>gi|55981303|ref|YP_144600.1| branched-chain amino acid ABC transporter, ATP-binding protein [Thermus thermophilus HB8]
>gi|55981309|ref|YP_144606.1| folyl-polyglutamate synthetase [Thermus thermophilus HB8]
>gi|55981316|ref|YP_144613.1| endonuclease V [Thermus thermophilus HB8]
>gi|55981320|ref|YP_144617.1| peptidase, M20/M25/M40 family [Thermus thermophilus HB8]
>gi|55981322|ref|YP_144619.1| probable RNA methyltransferase [Thermus thermophilus HB8]
>gi|55981330|ref|YP_144627.1| hypothetical protein TTHA1361 [Thermus thermophilus HB8]
>gi|55981332|ref|YP_144629.1| sensor histidine kinase [Thermus thermophilus HB8]
>gi|55981336|ref|YP_144633.1| hypothetical protein TTHA1367 [Thermus thermophilus HB8]
>gi|55981341|ref|YP_144638.1| N-acetylmuramoyl-L-alanine amidase [Thermus thermophilus HB8]
>gi|55981346|ref|YP_144643.1| hypothetical protein TTHA1377 [Thermus thermophilus HB8]
>gi|55981350|ref|YP_144647.1| competence protein PilO [Thermus thermophilus HB8]
>gi|55981351|ref|YP_144648.1| competence protein PilW [Thermus thermophilus HB8]
>gi|55981355|ref|YP_144652.1| 3-dehydroquinate synthase [Thermus thermophilus HB8]
>gi|55981356|ref|YP_144653.1| probable rRNA methylase [Thermus thermophilus HB8]
>gi|55981358|ref|YP_144655.1| hypothetical protein TTHA1389 [Thermus thermophilus HB8]
>gi|55981362|ref|YP_144659.1| hypothetical protein TTHA1393 [Thermus thermophilus HB8]
>gi|55981363|ref|YP_144660.1| homoserine kinase [Thermus thermophilus HB8]
>gi|55981366|ref|YP_144663.1| 30S ribosomal protein S20 [Thermus thermophilus HB8]
>gi|55981370|ref|YP_144667.1| cytochrome c family protein [Thermus thermophilus HB8]
>gi|55981373|ref|YP_144670.1| cytochrome c-type biogenesis protein CcmF [Thermus thermophilus HB8]
>gi|55981376|ref|YP_144673.1| cytochrome c-type biogenesis protein, heme exporter protein B [Thermus thermophilus HB8]
>gi|55981377|ref|YP_144674.1| ABC transporter, ATP-binding protein [Thermus thermophilus HB8]
>gi|55981378|ref|YP_144675.1| cytochrome c-type biogenesis protein CcdA [Thermus thermophilus HB8]
>gi|55981391|ref|YP_144688.1| thioredoxin [Thermus thermophilus HB8]
>gi|55981393|ref|YP_144690.1| ABC transporter ATP-binding protein (CycB) [Thermus thermophilus HB8]
>gi|55981394|ref|YP_144691.1| putative thiamine pyrophosphokinase [Thermus thermophilus HB8]
>gi|55981395|ref|YP_144692.1| hypothetical protein TTHA1426 [Thermus thermophilus HB8]
>gi|55981397|ref|YP_144694.1| putative phosphoglucomutase/phosphomannomutase [Thermus thermophilus HB8]
>gi|55981403|ref|YP_144700.1| 3-hydroxybutyryl-CoA dehydratase [Thermus thermophilus HB8]
>gi|55981405|ref|YP_144702.1| ABC-type transporter, ATP-binding protein [Thermus thermophilus HB8]
>gi|55981406|ref|YP_144703.1| transcription regulator, Crp family [Thermus thermophilus HB8]
>gi|55981407|ref|YP_144704.1| hypothetical protein TTHA1438 [Thermus thermophilus HB8]
>gi|55981408|ref|YP_144705.1| probable sugar aminotransferase [Thermus thermophilus HB8]
>gi|55981410|ref|YP_144707.1| hypothetical protein TTHA1441 [Thermus thermophilus HB8]
>gi|55981416|ref|YP_144713.1| alanine dehydrogenase [Thermus thermophilus HB8]
>gi|55981418|ref|YP_144715.1| putative hydrolase, TatD family [Thermus thermophilus HB8]
>gi|55981429|ref|YP_144726.1| ABC transporter, permease protein [Thermus thermophilus HB8]
>gi|55981431|ref|YP_144728.1| putative phosphoribosyltransferase [Thermus thermophilus HB8]
>gi|55981436|ref|YP_144733.1| hypothetical protein TTHA1467 [Thermus thermophilus HB8]
>gi|55981440|ref|YP_144737.1| putative transglycosylase [Thermus thermophilus HB8]
>gi|55981441|ref|YP_144738.1| Na(+)/H(+) antiporter [Thermus thermophilus HB8]
>gi|55981445|ref|YP_144742.1| probable Na(+)/H(+) antiporter [Thermus thermophilus HB8]
>gi|55981446|ref|YP_144743.1| hypothetical protein TTHA1477 [Thermus thermophilus HB8]
>gi|55981447|ref|YP_144744.1| hypothetical protein TTHA1478 [Thermus thermophilus HB8]
>gi|55981455|ref|YP_144752.1| hypothetical protein TTHA1486 [Thermus thermophilus HB8]
>gi|55981459|ref|YP_144756.1| GrpE protein (HSP-70 cofactor) [Thermus thermophilus HB8]
>gi|55981462|ref|YP_144759.1| hypothetical protein TTHA1493 [Thermus thermophilus HB8]
>gi|55981470|ref|YP_144767.1| putative hemolysin [Thermus thermophilus HB8]
>gi|55981473|ref|YP_144770.1| hypothetical protein TTHA1504 [Thermus thermophilus HB8]
>gi|55981474|ref|YP_144771.1| cytochrome c assembly protein-related protein [Thermus thermophilus HB8]
>gi|55981476|ref|YP_144773.1| probable uroporphyrinogen III synthase [Thermus thermophilus HB8]
>gi|55981490|ref|YP_144787.1| hypothetical protein TTHA1521 [Thermus thermophilus HB8]
>gi|55981491|ref|YP_144788.1| probable GTP-binding protein [Thermus thermophilus HB8]
>gi|55981492|ref|YP_144789.1| hypothetical protein TTHA1523 [Thermus thermophilus HB8]
>gi|55981494|ref|YP_144791.1| DNA repair protein RecN [Thermus thermophilus HB8]
>gi|55981498|ref|YP_144795.1| hypothetical protein TTHA1529 [Thermus thermophilus HB8]
>gi|55981503|ref|YP_144800.1| ribonuclease II family protein [Thermus thermophilus HB8]
>gi|55981510|ref|YP_144807.1| hypothetical protein TTHA1541 [Thermus thermophilus HB8]
>gi|55981511|ref|YP_144808.1| probable cell cycle protein MesJ [Thermus thermophilus HB8]
>gi|55981512|ref|YP_144809.1| hypothetical protein TTHA1543 [Thermus thermophilus HB8]
>gi|55981513|ref|YP_144810.1| hypothetical protein TTHA1544 [Thermus thermophilus HB8]
>gi|55981514|ref|YP_144811.1| hypothetical protein TTHA1545 [Thermus thermophilus HB8]
>gi|55981516|ref|YP_144813.1| putative peptidase [Thermus thermophilus HB8]
>gi|55981535|ref|YP_144832.1| hypothetical protein TTHA1566 [Thermus thermophilus HB8]
>gi|55981536|ref|YP_144833.1| transcriptional regulatory protein [Thermus thermophilus HB8]
>gi|55981538|ref|YP_144835.1| hypothetical protein TTHA1569 [Thermus thermophilus HB8]
>gi|55981542|ref|YP_144839.1| hypothetical protein TTHA1573 [Thermus thermophilus HB8]
>gi|55981549|ref|YP_144846.1| transcriptional regulator, GntR family [Thermus thermophilus HB8]
>gi|55981554|ref|YP_144851.1| hypothetical protein TTHA1585 [Thermus thermophilus HB8]
>gi|55981559|ref|YP_144856.1| hypothetical protein TTHA1590 [Thermus thermophilus HB8]

>gi|55981560|ref|YP_144857.1| hypothetical protein TTHA1591 [Thermus thermophilus HB8]
>gi|55981561|ref|YP_144858.1| sensor histidine kinase [Thermus thermophilus HB8]
>gi|55981562|ref|YP_144859.1| hypothetical protein TTHA1593 [Thermus thermophilus HB8]
>gi|55981565|ref|YP_144862.1| hypothetical protein TTHA1596 [Thermus thermophilus HB8]
>gi|55981566|ref|YP_144863.1| putative transporter [Thermus thermophilus HB8]
>gi|55981569|ref|YP_144866.1| RecR protein [Thermus thermophilus HB8]
>gi|55981570|ref|YP_144867.1| hypothetical protein TTHA1601 [Thermus thermophilus HB8]
>gi|55981571|ref|YP_144868.1| hypothetical protein TTHA1602 [Thermus thermophilus HB8]
>gi|55981572|ref|YP_144869.1| hypothetical protein TTHA1603 [Thermus thermophilus HB8]
>gi|55981578|ref|YP_144875.1| hypothetical protein TTHA1609 [Thermus thermophilus HB8]
>gi|55981581|ref|YP_144878.1| putative hydrolase [Thermus thermophilus HB8]
>gi|55981593|ref|YP_144890.1| hypothetical protein TTHA1624 [Thermus thermophilus HB8]
>gi|55981595|ref|YP_144892.1| hypothetical protein TTHA1626 [Thermus thermophilus HB8]
>gi|55981596|ref|YP_144893.1| hypothetical protein TTHA1627 [Thermus thermophilus HB8]
>gi|55981598|ref|YP_144895.1| iron ABC transporter, permease protein [Thermus thermophilus HB8]
>gi|55981600|ref|YP_144897.1| tRNA pseudouridine synthase A (pseudouridylate synthase I) [Thermus thermophilus HB8]
>gi|55981608|ref|YP_144905.1| hypothetical protein TTHA1639 [Thermus thermophilus HB8]
>gi|55981614|ref|YP_144911.1| MutS2 protein [Thermus thermophilus HB8]
>gi|55981615|ref|YP_144912.1| hypothetical protein TTHA1646 [Thermus thermophilus HB8]
>gi|55981618|ref|YP_144915.1| nucleotidyltransferase [Thermus thermophilus HB8]
>gi|55981619|ref|YP_144916.1| maltose ABC transporter, permease protein [Thermus thermophilus HB8]
>gi|55981620|ref|YP_144917.1| maltose ABC transporter, permease protein [Thermus thermophilus HB8]
>gi|55981624|ref|YP_144921.1| octaprenyl-diphosphate synthase [Thermus thermophilus HB8]
>gi|55981627|ref|YP_144924.1| hypothetical protein TTHA1658 [Thermus thermophilus HB8]
>gi|55981628|ref|YP_144925.1| tetratricopeptide repeat family protein [Thermus thermophilus HB8]
>gi|55981630|ref|YP_144927.1| conserved hypothetical protein, integral membrane protein [Thermus thermophilus HB8]
>gi|55981631|ref|YP_144928.1| hypothetical protein TTHA1662 [Thermus thermophilus HB8]
>gi|55981640|ref|YP_144937.1| adenylate kinase [Thermus thermophilus HB8]
>gi|55981645|ref|YP_144942.1| 50S ribosomal protein L18 [Thermus thermophilus HB8]
>gi|55981670|ref|YP_144967.1| hypothetical protein TTHA1701 [Thermus thermophilus HB8]
>gi|55981672|ref|YP_144969.1| sodium-alanine symporter family protein [Thermus thermophilus HB8]
>gi|55981673|ref|YP_144970.1| hypothetical protein TTHA1704 [Thermus thermophilus HB8]
>gi|55981675|ref|YP_144972.1| putative divalent heavy-metal cation transporter [Thermus thermophilus HB8]
>gi|55981676|ref|YP_144973.1| sugar fermentation stimulation protein family protein [Thermus thermophilus HB8]
>gi|55981678|ref|YP_144975.1| hypothetical protein TTHA1709 [Thermus thermophilus HB8]
>gi|55981681|ref|YP_144978.1| hypothetical protein TTHA1712 [Thermus thermophilus HB8]
>gi|55981682|ref|YP_144979.1| hypothetical protein TTHA1713 [Thermus thermophilus HB8]
>gi|55981684|ref|YP_144981.1| hypothetical protein TTHA1715 [Thermus thermophilus HB8]
>gi|55981685|ref|YP_144982.1| hypothetical protein TTHA1716 [Thermus thermophilus HB8]
>gi|55981687|ref|YP_144984.1| heavy metal binding protein [Thermus thermophilus HB8]
>gi|55981689|ref|YP_144986.1| cation-transporting ATPase [Thermus thermophilus HB8]
>gi|55981692|ref|YP_144989.1| sensor histidine kinase [Thermus thermophilus HB8]
>gi|55981693|ref|YP_144990.1| hypothetical protein TTHA1724 [Thermus thermophilus HB8]
>gi|55981696|ref|YP_144993.1| hypothetical protein TTHA1727 [Thermus thermophilus HB8]
>gi|55981699|ref|YP_144996.1| hypothetical protein TTHA1730 [Thermus thermophilus HB8]
>gi|55981700|ref|YP_144997.1| hypothetical protein TTHA1731 [Thermus thermophilus HB8]
>gi|55981703|ref|YP_145000.1| hypothetical protein TTHA1734 [Thermus thermophilus HB8]
>gi|55981710|ref|YP_145007.1| hypothetical protein TTHA1741 [Thermus thermophilus HB8]
>gi|55981711|ref|YP_145008.1| orotate phosphoribosyltransferase [Thermus thermophilus HB8]
>gi|55981718|ref|YP_145015.1| 2-amino-4-hydroxy-6- hydroxymethyldihydropteridine pyrophosphokinase (FolK) [Thermus thermophilus HB8]
>gi|55981721|ref|YP_145018.1| hypothetical protein TTHA1752 [Thermus thermophilus HB8]
>gi|55981722|ref|YP_145019.1| probable glycosyltransferase [Thermus thermophilus HB8]
>gi|55981723|ref|YP_145020.1| hypothetical protein TTHA1754 [Thermus thermophilus HB8]
>gi|55981727|ref|YP_145024.1| GGDEF domain protein [Thermus thermophilus HB8]
>gi|55981729|ref|YP_145026.1| hypothetical protein TTHA1760 [Thermus thermophilus HB8]
>gi|55981733|ref|YP_145030.1| molybdopterin oxidoreductase, iron-sulfur binding subunit [Thermus thermophilus HB8]
>gi|55981738|ref|YP_145035.1| hypothetical protein TTHA1769 [Thermus thermophilus HB8]
>gi|55981739|ref|YP_145036.1| putative endopeptidase, family M23/M37 [Thermus thermophilus HB8]
>gi|55981740|ref|YP_145037.1| pyrimidine-nucleoside (thymidine) phosphorylase [Thermus thermophilus HB8]
>gi|55981745|ref|YP_145042.1| hypothetical protein TTHA1776 [Thermus thermophilus HB8]
>gi|55981750|ref|YP_145047.1| probable GTP-binding protein [Thermus thermophilus HB8]
>gi|55981752|ref|YP_145049.1| 50S ribosomal protein L21 [Thermus thermophilus HB8]
>gi|55981755|ref|YP_145052.1| hypothetical protein TTHA1786 [Thermus thermophilus HB8]
>gi|55981756|ref|YP_145053.1| hypothetical protein TTHA1787 [Thermus thermophilus HB8]
>gi|55981761|ref|YP_145058.1| hypothetical protein TTHA1792 [Thermus thermophilus HB8]
>gi|55981762|ref|YP_145059.1| hypothetical protein TTHA1793 [Thermus thermophilus HB8]
>gi|55981766|ref|YP_145063.1| probable amidase [Thermus thermophilus HB8]
>gi|55981767|ref|YP_145064.1| transport protein [Thermus thermophilus HB8]
>gi|55981769|ref|YP_145066.1| hypothetical protein TTHA1800 [Thermus thermophilus HB8]
>gi|55981770|ref|YP_145067.1| hypothetical protein TTHA1801 [Thermus thermophilus HB8]

>gi|55981773|ref|YP_145070.1| probable acyl-CoA thioesterase [Thermus thermophilus HB8]
>gi|55981774|ref|YP_145071.1| hypothetical protein TTHA1805 [Thermus thermophilus HB8]
>gi|55981776|ref|YP_145073.1| ABC transporter, permease protein [Thermus thermophilus HB8]
>gi|55981779|ref|YP_145076.1| hypothetical protein TTHA1810 [Thermus thermophilus HB8]
>gi|55981783|ref|YP_145080.1| hypothetical protein TTHA1814 [Thermus thermophilus HB8]
>gi|55981786|ref|YP_145083.1| putative hydrolase (HD domain) [Thermus thermophilus HB8]
>gi|55981790|ref|YP_145087.1| hypothetical protein TTHA1821 [Thermus thermophilus HB8]
>gi|55981791|ref|YP_145088.1| probable transporter [Thermus thermophilus HB8]
>gi|55981794|ref|YP_145091.1| hypothetical protein TTHA1825 [Thermus thermophilus HB8]
>gi|55981795|ref|YP_145092.1| hypothetical protein TTHA1826 [Thermus thermophilus HB8]
>gi|55981796|ref|YP_145093.1| sugar ABC transporter permease protein [Thermus thermophilus HB8]
>gi|55981799|ref|YP_145096.1| hypothetical protein TTHA1830 [Thermus thermophilus HB8]
>gi|55981802|ref|YP_145099.1| ABC transporter, permease protein [Thermus thermophilus HB8]
>gi|55981806|ref|YP_145103.1| hypothetical protein TTHA1837 [Thermus thermophilus HB8]
>gi|55981811|ref|YP_145108.1| anthranilate phosphoribosyltransferase (TrpD) [Thermus thermophilus HB8]
>gi|55981816|ref|YP_145113.1| hypothetical protein TTHA1847 [Thermus thermophilus HB8]
>gi|55981823|ref|YP_145120.1| ubiquinone/menaquinone biosynthesis methyltransferase [Thermus thermophilus HB8]
>gi|55981824|ref|YP_145121.1| ABC transporter, permease protein [Thermus thermophilus HB8]
>gi|55981828|ref|YP_145125.1| PSP1-related protein [Thermus thermophilus HB8]
>gi|55981829|ref|YP_145126.1| DNA polymerase III delta prime subunit [Thermus thermophilus HB8]
>gi|55981833|ref|YP_145130.1| S-layer protein-related protein [Thermus thermophilus HB8]
>gi|55981839|ref|YP_145136.1| hypothetical protein TTHA1870 [Thermus thermophilus HB8]
>gi|55981848|ref|YP_145145.1| ferripyochelin-binding protein [Thermus thermophilus HB8]
>gi|55981852|ref|YP_145149.1| hypothetical protein TTHA1883 [Thermus thermophilus HB8]
>gi|55981853|ref|YP_145150.1| probable ATPase [Thermus thermophilus HB8]
>gi|55981854|ref|YP_145151.1| hypothetical protein TTHA1885 [Thermus thermophilus HB8]
>gi|55981855|ref|YP_145152.1| ABC transporter, permease protein [Thermus thermophilus HB8]
>gi|55981858|ref|YP_145155.1| hypothetical protein TTHA1889 [Thermus thermophilus HB8]
>gi|55981860|ref|YP_145157.1| hypothetical protein TTHA1891 [Thermus thermophilus HB8]
>gi|55981863|ref|YP_145160.1| hypothetical protein TTHA1894 [Thermus thermophilus HB8]
>gi|55981864|ref|YP_145161.1| hypothetical protein TTHA1895 [Thermus thermophilus HB8]
>gi|55981870|ref|YP_145167.1| carbon-nitrogen hydrolase family protein [Thermus thermophilus HB8]
>gi|55981875|ref|YP_145172.1| hypothetical protein TTHA1906 [Thermus thermophilus HB8]
>gi|55981877|ref|YP_145174.1| hypothetical protein TTHA1908 [Thermus thermophilus HB8]
>gi|55981878|ref|YP_145175.1| hypothetical protein TTHA1909 [Thermus thermophilus HB8]
>gi|55981888|ref|YP_145185.1| hypothetical protein TTHA1919 [Thermus thermophilus HB8]
>gi|55981890|ref|YP_145187.1| hypothetical protein TTHA1921 [Thermus thermophilus HB8]
>gi|55981891|ref|YP_145188.1| hypothetical protein TTHA1922 [Thermus thermophilus HB8]
>gi|55981892|ref|YP_145189.1| sodium ABC transporter, permease protein NatB [Thermus thermophilus HB8]
>gi|55981898|ref|YP_145195.1| hypothetical protein TTHA1929 [Thermus thermophilus HB8]
>gi|55981903|ref|YP_145200.1| hypothetical protein TTHA1934 [Thermus thermophilus HB8]
>gi|55981910|ref|YP_145207.1| ZIP zinc transporter family protein [Thermus thermophilus HB8]
>gi|55981913|ref|YP_145210.1| hypothetical protein TTHA1944 [Thermus thermophilus HB8]
>gi|55981914|ref|YP_145211.1| hypothetical protein TTHA1945 [Thermus thermophilus HB8]
>gi|55981917|ref|YP_145214.1| hypothetical protein TTHA1948 [Thermus thermophilus HB8]
>gi|55981919|ref|YP_145216.1| N-acetylglutamate kinase (ArgB-2) [Thermus thermophilus HB8]
>gi|55981920|ref|YP_145217.1| hypothetical protein TTHA1951 [Thermus thermophilus HB8]
>gi|55981921|ref|YP_145218.1| DNA polymerase III holoenzyme tau subunit [Thermus thermophilus HB8]
>gi|55981927|ref|YP_145224.1| phenylalanyl-tRNA synthetase alpha chain [Thermus thermophilus HB8]
>gi|55981930|ref|YP_145227.1| hypothetical protein TTHA1961 [Thermus thermophilus HB8]
>gi|55981933|ref|YP_145230.1| hypothetical protein TTHA1964 [Thermus thermophilus HB8]
>gi|55981935|ref|YP_145232.1| hypothetical protein TTHA1966 [Thermus thermophilus HB8]
>gi|55981937|ref|YP_145234.1| competence protein ComEC [Thermus thermophilus HB8]
>gi|55981940|ref|YP_145237.1| glucose inhibited division protein B [Thermus thermophilus HB8]
>gi|55978184|ref|YP_145240.1| hypothetical protein TTHB001 [Thermus thermophilus HB8]
>gi|55978190|ref|YP_145246.1| hypothetical protein TTHB007 [Thermus thermophilus HB8]
>gi|55978196|ref|YP_145252.1| hypothetical protein TTHB013 [Thermus thermophilus HB8]
>gi|55978203|ref|YP_145259.1| 3-oxoacyl-[acyl carrier protein] reductase [Thermus thermophilus HB8]
>gi|55978213|ref|YP_145269.1| probable sugar transporter [Thermus thermophilus HB8]
>gi|55978216|ref|YP_145272.1| alpha-glucosidase [Thermus thermophilus HB8]
>gi|55978219|ref|YP_145275.1| probable sugar transporter [Thermus thermophilus HB8]
>gi|55978220|ref|YP_145276.1| hypothetical protein TTHB037 [Thermus thermophilus HB8]
>gi|55978222|ref|YP_145278.1| hypothetical protein TTHB039 [Thermus thermophilus HB8]
>gi|55978224|ref|YP_145280.1| hypothetical protein TTHB041 [Thermus thermophilus HB8]
>gi|55978226|ref|YP_145282.1| cobyrinic acid a,c-diamide synthase [Thermus thermophilus HB8]
>gi|55978228|ref|YP_145284.1| repeat motif-containing protein [Thermus thermophilus HB8]
>gi|55978229|ref|YP_145285.1| cobalamin (5'-phosphate) synthase [Thermus thermophilus HB8]
>gi|55978230|ref|YP_145286.1| hypothetical protein TTHB047 [Thermus thermophilus HB8]
>gi|55978231|ref|YP_145287.1| nicotinate-nucleotide--dimethylbenzimidazole phosphoribosyltransferase [Thermus thermophilus HB8]
>gi|55978233|ref|YP_145289.1| probable high-affinity nickel permease [Thermus thermophilus HB8]

>gi|55978238|ref|YP_145294.1| precorrin-4 C11-methyltransferase [Thermus thermophilus HB8]
>gi|55978241|ref|YP_145297.1| cobalamin biosynthesis protein CbiX [Thermus thermophilus HB8]
>gi|55978242|ref|YP_145298.1| hypothetical protein TTHB059 [Thermus thermophilus HB8]
>gi|55978244|ref|YP_145300.1| cobalamin biosynthesis nitroreductase BluB [Thermus thermophilus HB8]
>gi|55978245|ref|YP_145301.1| cobalamin biosynthesis protein CbiB [Thermus thermophilus HB8]
>gi|55978254|ref|YP_145310.1| hypothetical protein TTHB071 [Thermus thermophilus HB8]
>gi|55978255|ref|YP_145311.1| 4-hydroxy-2-oxoglutarate aldolase/2-deydro-3-deoxyphosphogluconate aldolase [Thermus thermophilus HB8]
>gi|55978259|ref|YP_145315.1| putative C4-dicarboxylate transporter, large permease protein [Thermus thermophilus HB8]
>gi|55978262|ref|YP_145318.1| 2-keto-3-deoxy-gluconate kinase [Thermus thermophilus HB8]
>gi|55978266|ref|YP_145322.1| sugar ABC transporter, permease protein [Thermus thermophilus HB8]
>gi|55978273|ref|YP_145329.1| hypothetical protein TTHB090 [Thermus thermophilus HB8]
>gi|55978274|ref|YP_145330.1| hypothetical protein TTHB091 [Thermus thermophilus HB8]
>gi|55978276|ref|YP_145332.1| putative oxidoreductase [Thermus thermophilus HB8]
>gi|55978277|ref|YP_145333.1| putative short-chain oxidoreductase [Thermus thermophilus HB8]
>gi|55978279|ref|YP_145335.1| hypothetical protein TTHB096 [Thermus thermophilus HB8]
>gi|55978283|ref|YP_145339.1| transcriptional regulator, MerR family [Thermus thermophilus HB8]
>gi|55978285|ref|YP_145341.1| DNA photolyase [Thermus thermophilus HB8]
>gi|55978287|ref|YP_145343.1| phytoene synthase-related protein [Thermus thermophilus HB8]
>gi|55978289|ref|YP_145345.1| probable glycosyltransferase [Thermus thermophilus HB8]
>gi|55978296|ref|YP_145352.1| hypothetical protein TTHB113 [Thermus thermophilus HB8]
>gi|55978317|ref|YP_145373.1| hypothetical protein TTHB134 [Thermus thermophilus HB8]
>gi|55978318|ref|YP_145374.1| hypothetical protein TTHB135 [Thermus thermophilus HB8]
>gi|55978319|ref|YP_145375.1| probable transcriptional regulator, CopG family [Thermus thermophilus HB8]
>gi|55978320|ref|YP_145376.1| hypothetical protein TTHB137 [Thermus thermophilus HB8]
>gi|55978322|ref|YP_145378.1| permease of the major facilitator superfamily [Thermus thermophilus HB8]
>gi|55978323|ref|YP_145379.1| hypothetical protein TTHB140 [Thermus thermophilus HB8]
>gi|55978330|ref|YP_145386.1| hypothetical protein TTHB147 [Thermus thermophilus HB8]
>gi|55978334|ref|YP_145390.1| hypothetical protein TTHB151 [Thermus thermophilus HB8]
>gi|55978335|ref|YP_145391.1| hypothetical protein TTHB152 [Thermus thermophilus HB8]
>gi|55978336|ref|YP_145392.1| hypothetical protein TTHB153 [Thermus thermophilus HB8]
>gi|55978342|ref|YP_145398.1| hypothetical protein TTHB159 [Thermus thermophilus HB8]
>gi|55978344|ref|YP_145400.1| hypothetical protein TTHB161 [Thermus thermophilus HB8]
>gi|55978347|ref|YP_145403.1| hypothetical protein TTHB164 [Thermus thermophilus HB8]
>gi|55978354|ref|YP_145410.1| hypothetical protein TTHB171 [Thermus thermophilus HB8]
>gi|55978355|ref|YP_145411.1| reverse gyrase [Thermus thermophilus HB8]
>gi|55978356|ref|YP_145412.1| response regulator [Thermus thermophilus HB8]
>gi|55978357|ref|YP_145413.1| sensor histidine kinase-like protein [Thermus thermophilus HB8]
>gi|55978363|ref|YP_145419.1| hypothetical protein TTHB180 [Thermus thermophilus HB8]
>gi|55978367|ref|YP_145423.1| hypothetical protein TTHB184 [Thermus thermophilus HB8]
>gi|55978368|ref|YP_145424.1| hypothetical protein TTHB185 [Thermus thermophilus HB8]
>gi|55978369|ref|YP_145425.1| putative transcriptional regulator [Thermus thermophilus HB8]
>gi|55978379|ref|YP_145435.1| putative protein required for formate dehydrogenase activity [Thermus thermophilus HB8]
>gi|55978382|ref|YP_145438.1| hypothetical protein TTHB199 [Thermus thermophilus HB8]
>gi|55978385|ref|YP_145441.1| hypothetical protein TTHB202 [Thermus thermophilus HB8]
>gi|55978389|ref|YP_145445.1| hypothetical protein TTHB206 [Thermus thermophilus HB8]
>gi|55978391|ref|YP_145447.1| ribonucleoside-diphosphate reductase, beta subunit [Thermus thermophilus HB8]
>gi|55978400|ref|YP_145456.1| hypothetical protein TTHB217 [Thermus thermophilus HB8]
>gi|55978402|ref|YP_145458.1| hemin ABC transporter, permease protein [Thermus thermophilus HB8]
>gi|55978409|ref|YP_145465.1| hypothetical protein TTHB226 [Thermus thermophilus HB8]
>gi|55978414|ref|YP_145470.1| hypothetical protein TTHB231 [Thermus thermophilus HB8]
>gi|55978416|ref|YP_145472.1| hypothetical protein TTHB233 [Thermus thermophilus HB8]
>gi|55978417|ref|YP_145473.1| hypothetical protein TTHB234 [Thermus thermophilus HB8]
>gi|55978426|ref|YP_145482.1| hypothetical protein TTHB243 [Thermus thermophilus HB8]
>gi|55978428|ref|YP_145484.1| hypothetical protein TTHB245 [Thermus thermophilus HB8]
>gi|55978447|ref|YP_145502.1| anti-toxin-like protein [Thermus thermophilus HB8]
>gi|55978449|ref|YP_145504.1| hypothetical protein TTHC014 [Thermus thermophilus HB8]
>gi|23500939|ref|NP_697066.1| hypothetical protein BR0024 [Brucella suis 1330]
>gi|23500941|ref|NP_697068.1| cytidylate kinase [Brucella suis 1330]
>gi|23500945|ref|NP_697072.1| membrane protein, putative [Brucella suis 1330]
>gi|23500962|ref|NP_697089.1| hypothetical protein BR0047 [Brucella suis 1330]
>gi|23500986|ref|NP_697113.1| hypothetical protein BR0071 [Brucella suis 1330]
>gi|23501001|ref|NP_697128.1| ABC transporter, permease protein [Brucella suis 1330]
>gi|23501009|ref|NP_697136.1| heme exporter protein CcmB [Brucella suis 1330]
>gi|23501023|ref|NP_697150.1| sulfate ABC transporter, permease protein [Brucella suis 1330]
>gi|23501078|ref|NP_697205.1| hypothetical protein BR0167 [Brucella suis 1330]
>gi|23501082|ref|NP_697209.1| heat shock protein GrpE [Brucella suis 1330]
>gi|23501098|ref|NP_697225.1| hypothetical protein BR0187 [Brucella suis 1330]
>gi|23501139|ref|NP_697266.1| sarcosine oxidase, gamma subunit [Brucella suis 1330]
>gi|23501162|ref|NP_697289.1| hypothetical protein BR0255 [Brucella suis 1330]
>gi|23501170|ref|NP_697297.1| hypothetical protein BR0263 [Brucella suis 1330]

>gi|23501217|ref|NP_697344.1| DNA-damage-inducible protein F, putative [Brucella suis 1330]
>gi|23501225|ref|NP_697352.1| large conductance mechanosensitive channel protein [Brucella suis 1330]
>gi|23501232|ref|NP_697359.1| hypothetical protein BR0325 [Brucella suis 1330]
>gi|23501278|ref|NP_697405.1| hypothetical protein BR0374 [Brucella suis 1330]
>gi|23501288|ref|NP_697415.1| ATP synthase F0, B subunit, putative [Brucella suis 1330]
>gi|23501289|ref|NP_697416.1| ATP synthase F0, B subunit [Brucella suis 1330]
>gi|23501295|ref|NP_697422.1| hypothetical protein BR0391 [Brucella suis 1330]
>gi|23501303|ref|NP_697430.1| hypothetical protein BR0399 [Brucella suis 1330]
>gi|23501321|ref|NP_697448.1| chaperone protein DnaJ, putative [Brucella suis 1330]
>gi|23501334|ref|NP_697461.1| transglycosylase-associated protein, putative [Brucella suis 1330]
>gi|23501335|ref|NP_697462.1| hypothetical protein BR0434 [Brucella suis 1330]
>gi|23501342|ref|NP_697469.1| LysM domain protein [Brucella suis 1330]
>gi|23501349|ref|NP_697476.1| colicin V production protein, putative [Brucella suis 1330]
>gi|23501356|ref|NP_697483.1| ribosomal protein S6 [Brucella suis 1330]
>gi|23501374|ref|NP_697501.1| hypothetical protein BR0473 [Brucella suis 1330]
>gi|23501398|ref|NP_697525.1| SMC family protein [Brucella suis 1330]
>gi|23501411|ref|NP_697538.1| glycosyl transferase, group 4 family protein [Brucella suis 1330]
>gi|23501456|ref|NP_697583.1| hypothetical protein BR0567 [Brucella suis 1330]
>gi|23501476|ref|NP_697603.1| hypothetical protein BR0587 [Brucella suis 1330]
>gi|23501483|ref|NP_697610.1| hypothetical protein BR0594 [Brucella suis 1330]
>gi|23501520|ref|NP_697647.1| hypothetical protein BR0633 [Brucella suis 1330]
>gi|23501538|ref|NP_697665.1| DNA-directed RNA polymerase, omega subunit [Brucella suis 1330]
>gi|23501591|ref|NP_697718.1| hypothetical protein BR0704 [Brucella suis 1330]
>gi|23501600|ref|NP_697727.1| permease, putative [Brucella suis 1330]
>gi|23501603|ref|NP_697730.1| hypothetical protein BR0716 [Brucella suis 1330]
>gi|23501623|ref|NP_697750.1| hypothetical protein BR0736 [Brucella suis 1330]
>gi|23501624|ref|NP_697751.1| lipoprotein, putative [Brucella suis 1330]
>gi|23501681|ref|NP_697808.1| hypothetical protein BR0794 [Brucella suis 1330]
>gi|23501698|ref|NP_697825.1| NADH dehydrogenase I, J subunit [Brucella suis 1330]
>gi|23501715|ref|NP_697842.1| hypothetical protein BR0828 [Brucella suis 1330]
>gi|23501725|ref|NP_697852.1| lipoprotein, putative [Brucella suis 1330]
>gi|23501730|ref|NP_697857.1| hypothetical protein BR0843 [Brucella suis 1330]
>gi|23501743|ref|NP_697870.1| hypothetical protein BR0856 [Brucella suis 1330]
>gi|23501752|ref|NP_697879.1| cobalamin 5'-phosphate synthase [Brucella suis 1330]
>gi|23501764|ref|NP_697891.1| hypothetical protein BR0878 [Brucella suis 1330]
>gi|23501769|ref|NP_697896.1| mttA/Hcf106 family protein [Brucella suis 1330]
>gi|23501774|ref|NP_697901.1| peptidase, M23/M37 family [Brucella suis 1330]
>gi|23501793|ref|NP_697920.1| acetyl-CoA carboxylase, biotin carboxyl carrier protein [Brucella suis 1330]
>gi|23501798|ref|NP_697925.1| ribonuclease, Rne/Rng domain protein [Brucella suis 1330]
>gi|23501812|ref|NP_697939.1| quaternary ammonium compound-resistance protein [Brucella suis 1330]
>gi|23501826|ref|NP_697953.1| heavy-metal-associated domain protein [Brucella suis 1330]
>gi|23501847|ref|NP_697974.1| hypothetical protein BR0964 [Brucella suis 1330]
>gi|23501894|ref|NP_698021.1| diacylglycerol kinase [Brucella suis 1330]
>gi|23501917|ref|NP_698044.1| hypothetical protein BR1038 [Brucella suis 1330]
>gi|23501980|ref|NP_698107.1| single-stranded DNA-binding protein family [Brucella suis 1330]
>gi|23502003|ref|NP_698130.1| hypothetical protein BR1125 [Brucella suis 1330]
>gi|23502005|ref|NP_698132.1| pyruvate dehydrogenase complex, E2 component, dihydrolipoamide acetyltransferase [Brucella suis 1330]
>gi|23502015|ref|NP_698142.1| preprotein translocase, SecG subunit, putative [Brucella suis 1330]
>gi|23502039|ref|NP_698166.1| translation elongation factor Ts [Brucella suis 1330]
>gi|23502081|ref|NP_698208.1| OmpA family protein [Brucella suis 1330]
>gi|23502094|ref|NP_698221.1| ribosomal protein L18 [Brucella suis 1330]
>gi|23502109|ref|NP_698236.1| ribosomal protein L4 [Brucella suis 1330]
>gi|23502122|ref|NP_698249.1| ribosomal protein L7/L12 [Brucella suis 1330]
>gi|23502136|ref|NP_698263.1| ferripyochelin-binding protein, putative [Brucella suis 1330]
>gi|23502145|ref|NP_698272.1| hypothetical protein BR1269 [Brucella suis 1330]
>gi|23502179|ref|NP_698306.1| hypothetical protein BR1303 [Brucella suis 1330]
>gi|23502185|ref|NP_698312.1| hypothetical protein BR1309 [Brucella suis 1330]
>gi|23502205|ref|NP_698332.1| dedA family protein [Brucella suis 1330]
>gi|23502208|ref|NP_698335.1| acid-shock protein, putative [Brucella suis 1330]
>gi|23502217|ref|NP_698344.1| iron compound ABC transporter, permease protein [Brucella suis 1330]
>gi|23502223|ref|NP_698350.1| HlyD family secretion protein [Brucella suis 1330]
>gi|23502224|ref|NP_698351.1| hypothetical protein BR1352 [Brucella suis 1330]
>gi|23502235|ref|NP_698362.1| urea transporter, putative [Brucella suis 1330]
>gi|23502237|ref|NP_698364.1| cobalamin biosynthesis protein CbiM [Brucella suis 1330]
>gi|23502286|ref|NP_698413.1| antibiotic acetyltransferase [Brucella suis 1330]
>gi|23502296|ref|NP_698423.1| cell division protein FtsZ [Brucella suis 1330]
>gi|23502317|ref|NP_698444.1| hypothetical protein BR1446 [Brucella suis 1330]
>gi|23502341|ref|NP_698468.1| hypothetical protein BR1472 [Brucella suis 1330]
>gi|23502345|ref|NP_698472.1| hypothetical protein BR1476 [Brucella suis 1330]
>gi|23502380|ref|NP_698507.1| hypothetical protein BR1511 [Brucella suis 1330]

>gi|23502384|ref|NP_698511.1| hypothetical protein BR1516 [Brucella suis 1330]
>gi|23502386|ref|NP_698513.1| hypothetical protein BR1518 [Brucella suis 1330]
>gi|23502484|ref|NP_698611.1| hypothetical protein BR1621 [Brucella suis 1330]
>gi|23502515|ref|NP_698642.1| hypothetical protein BR1656 [Brucella suis 1330]
>gi|23502519|ref|NP_698646.1| hypothetical protein BR1660 [Brucella suis 1330]
>gi|23502527|ref|NP_698654.1| TonB-dependent receptor [Brucella suis 1330]
>gi|23502544|ref|NP_698671.1| hypothetical protein BR1686 [Brucella suis 1330]
>gi|23502546|ref|NP_698673.1| sodium/bile acid transporter family protein [Brucella suis 1330]
>gi|23502556|ref|NP_698683.1| tolA protein [Brucella suis 1330]
>gi|23502572|ref|NP_698699.1| OmpA family protein [Brucella suis 1330]
>gi|23502601|ref|NP_698728.1| phosphoribosylaminoimidazole carboxylase, catalytic subunit [Brucella suis 1330]
>gi|23502604|ref|NP_698731.1| TPR domain protein [Brucella suis 1330]
>gi|23502612|ref|NP_698739.1| lipoprotein, putative [Brucella suis 1330]
>gi|23502649|ref|NP_698776.1| transcriptional regulator, MarR family [Brucella suis 1330]
>gi|23502677|ref|NP_698804.1| ribosomal protein S16 [Brucella suis 1330]
>gi|23502679|ref|NP_698806.1| signal recognition particle protein [Brucella suis 1330]
>gi|23502687|ref|NP_698814.1| membrane protein, putative [Brucella suis 1330]
>gi|23502691|ref|NP_698818.1| hypothetical protein BR1838 [Brucella suis 1330]
>gi|23502699|ref|NP_698826.1| hypothetical protein BR1846 [Brucella suis 1330]
>gi|23502703|ref|NP_698830.1| ribosomal protein L21 [Brucella suis 1330]
>gi|23502706|ref|NP_698833.1| AzlC family protein [Brucella suis 1330]
>gi|23502718|ref|NP_698845.1| hypothetical protein BR1865 [Brucella suis 1330]
>gi|23502726|ref|NP_698853.1| membrane protein, putative [Brucella suis 1330]
>gi|23502747|ref|NP_698874.1| ammonium transporter [Brucella suis 1330]
>gi|23502772|ref|NP_698899.1| 2-oxoglutarate dehydrogenase, E2 component, dihydrolipoamide succinyltransferase [Brucella suis 1330]
>gi|23502784|ref|NP_698911.1| signal recognition particle-docking protein FtsY [Brucella suis 1330]
>gi|23502815|ref|NP_698942.1| universal stress protein family [Brucella suis 1330]
>gi|23502818|ref|NP_698945.1| electron transfer flavoprotein, alpha subunit [Brucella suis 1330]
>gi|23502861|ref|NP_698988.1| outer membrane autotransporter [Brucella suis 1330]
>gi|23502865|ref|NP_698992.1| transcriptional regulator, MerR family [Brucella suis 1330]
>gi|23502878|ref|NP_699005.1| hypothetical protein BR2030 [Brucella suis 1330]
>gi|23502959|ref|NP_699086.1| N-(5'phosphoribosyl)anthranilate isomerase [Brucella suis 1330]
>gi|23502962|ref|NP_699089.1| hypothetical protein BR2114 [Brucella suis 1330]
>gi|23502967|ref|NP_699094.1| ribosomal protein L35 [Brucella suis 1330]
>gi|23502993|ref|NP_699120.1| lipoprotein, putative [Brucella suis 1330]
>gi|23503012|ref|NP_699139.1| hypothetical protein BR2164 [Brucella suis 1330]
>gi|23503013|ref|NP_699140.1| translation initiation factor IF-2 [Brucella suis 1330]
>gi|23499774|ref|NP_699214.1| ABC transporter, permease protein [Brucella suis 1330]
>gi|23499787|ref|NP_699227.1| hypothetical protein BRA0020 [Brucella suis 1330]
>gi|23499789|ref|NP_699229.1| hypothetical protein BRA0022 [Brucella suis 1330]
>gi|23499800|ref|NP_699240.1| acetoin dehydrogenase complex, E2 component, dihydrolipoamide acetyltransferase, putative [Brucella suis 1330]
>gi|23499824|ref|NP_699264.1| hypothetical protein BRA0057 [Brucella suis 1330]
>gi|23499827|ref|NP_699267.1| type IV secretion system protein VirB10 [Brucella suis 1330]
>gi|23499828|ref|NP_699268.1| type IV secretion system protein VirB9 [Brucella suis 1330]
>gi|23499831|ref|NP_699271.1| type IV secretion system protein VirB6 [Brucella suis 1330]
>gi|23499842|ref|NP_699282.1| SPFH domain/Band 7 family protein [Brucella suis 1330]
>gi|23499850|ref|NP_699290.1| hypothetical protein BRA0083 [Brucella suis 1330]
>gi|23499919|ref|NP_699359.1| hypothetical protein BRA0157 [Brucella suis 1330]
>gi|23499934|ref|NP_699374.1| hypothetical protein BRA0172 [Brucella suis 1330]
>gi|23499935|ref|NP_699375.1| outer membrane autotransporter [Brucella suis 1330]
>gi|23499989|ref|NP_699429.1| hypothetical protein BRA0232 [Brucella suis 1330]
>gi|23500010|ref|NP_699450.1| hypothetical protein BRA0253 [Brucella suis 1330]
>gi|23500024|ref|NP_699464.1| sugar ABC transporter, permease protein [Brucella suis 1330]
>gi|23500034|ref|NP_699474.1| nitrous-oxide reductase, nosY component [Brucella suis 1330]
>gi|23500080|ref|NP_699520.1| hypothetical protein BRA0325 [Brucella suis 1330]
>gi|23500096|ref|NP_699536.1| hypothetical protein BRA0343 [Brucella suis 1330]
>gi|23500119|ref|NP_699559.1| trbL protein [Brucella suis 1330]
>gi|23500198|ref|NP_699638.1| 3-hydroxyacyl-CoA dehydrogenase, putative [Brucella suis 1330]
>gi|23500206|ref|NP_699646.1| hypothetical protein BRA0457 [Brucella suis 1330]
>gi|23500212|ref|NP_699652.1| HlyD family secretion protein [Brucella suis 1330]
>gi|23500251|ref|NP_699691.1| monovalent cation/proton antiporter, MnhF/PhaF subunit [Brucella suis 1330]
>gi|23500256|ref|NP_699696.1| ABC transporter, ATP-binding/permease protein [Brucella suis 1330]
>gi|23500277|ref|NP_699717.1| cell division protein FtsK, putative [Brucella suis 1330]
>gi|23500312|ref|NP_699752.1| ABC transporter, permease protein [Brucella suis 1330]
>gi|23500331|ref|NP_699771.1| hypothetical protein BRA0587 [Brucella suis 1330]
>gi|23500345|ref|NP_699785.1| membrane protein, putative [Brucella suis 1330]
>gi|23500371|ref|NP_699811.1| hypothetical protein BRA0627 [Brucella suis 1330]
>gi|23500394|ref|NP_699834.1| branched-chain amino acid ABC transporter, permease protein, [Brucella suis 1330]
>gi|23500405|ref|NP_699845.1| uracil-xanthine permease, putative [Brucella suis 1330]
>gi|23500446|ref|NP_699886.1| major facilitator family transporter [Brucella suis 1330]

>gi|23500472|ref|NP_699912.1| immunoreactive 14 kDa protein BA14k [Brucella suis 1330]
>gi|23500523|ref|NP_699963.1| hypothetical protein BRA0789 [Brucella suis 1330]
>gi|23500548|ref|NP_699988.1| crcB family protein [Brucella suis 1330]
>gi|23500692|ref|NP_700132.1| ribosomal protein S21 [Brucella suis 1330]
>gi|23500697|ref|NP_700137.1| NAD(P) transhydrogenase, beta subunit [Brucella suis 1330]
>gi|23500698|ref|NP_700138.1| NAD(P) transhydrogenase, alpha2 subunit [Brucella suis 1330]
>gi|23500699|ref|NP_700139.1| NAD(P) transhydrogenase, alpha subunit [Brucella suis 1330]
>gi|23500711|ref|NP_700151.1| cobalamin synthesis protein/P47K family protein [Brucella suis 1330]
>gi|23500717|ref|NP_700157.1| ribose ABC transporter, permease protein [Brucella suis 1330]
>gi|23500736|ref|NP_700176.1| oxidoreductase, short chain dehydrogenase/reductase family [Brucella suis 1330]
>gi|23500758|ref|NP_700198.1| hypothetical protein BRA1036 [Brucella suis 1330]
>gi|23500779|ref|NP_700219.1| hypothetical protein BRA1057 [Brucella suis 1330]
>gi|23500807|ref|NP_700247.1| enoyl-CoA hydratase/isomerase family protein [Brucella suis 1330]
>gi|23500822|ref|NP_700262.1| polyamine ABC transporter, permease protein [Brucella suis 1330]
>gi|23500839|ref|NP_700279.1| zinc ABC transporter, permease protein [Brucella suis 1330]
>gi|23500845|ref|NP_700285.1| hypothetical protein BRA1130 [Brucella suis 1330]
>gi|23500848|ref|NP_700288.1| flagellar biosynthesis protein FliQ [Brucella suis 1330]
>gi|23500857|ref|NP_700297.1| motD-related protein [Brucella suis 1330]
>gi|23500862|ref|NP_700302.1| outer membrane autotransporter [Brucella suis 1330]
>gi|23500866|ref|NP_700306.1| D-xylose ABC transporter, permease protein [Brucella suis 1330]
>gi|23500883|ref|NP_700323.1| amino acid permease family protein [Brucella suis 1330]
>gi|23500887|ref|NP_700327.1| branched-chain amino acid ABC transporter, permease protein [Brucella suis 1330]
>gi|15834666|ref|NP_296425.1| hypothetical protein TC0041 [Chlamydia muridarum Nigg]
>gi|15834743|ref|NP_296502.1| histone H1-like protein HC1 [Chlamydia muridarum Nigg]
>gi|15834764|ref|NP_296523.1| hypothetical protein TC0144 [Chlamydia muridarum Nigg]
>gi|15834780|ref|NP_296539.1| hypothetical protein TC0160 [Chlamydia muridarum Nigg]
>gi|15834790|ref|NP_296549.1| ribosomal protein S14 [Chlamydia muridarum Nigg]
>gi|15834819|ref|NP_296578.1| hypothetical protein TC0199 [Chlamydia muridarum Nigg]
>gi|15834833|ref|NP_296592.1| CDP-diacylglycerol--serine O-phosphatidyltransferase, putative [Chlamydia muridarum Nigg]
>gi|15834859|ref|NP_296618.1| hypothetical protein TC0239 [Chlamydia muridarum Nigg]
>gi|15834936|ref|NP_296695.1| Hc2 nucleoprotein [Chlamydia muridarum Nigg]
>gi|15834941|ref|NP_296700.1| hypothetical protein TC0321 [Chlamydia muridarum Nigg]
>gi|15834943|ref|NP_296702.1| hypothetical protein TC0323 [Chlamydia muridarum Nigg]
>gi|15834948|ref|NP_296707.1| hypothetical protein TC0328 [Chlamydia muridarum Nigg]
>gi|15834971|ref|NP_296730.1| hypothetical protein TC0352 [Chlamydia muridarum Nigg]
>gi|15835011|ref|NP_296770.1| hypothetical protein TC0392 [Chlamydia muridarum Nigg]
>gi|15835013|ref|NP_296772.1| hypothetical protein TC0394 [Chlamydia muridarum Nigg]
>gi|15835029|ref|NP_296788.1| hypothetical protein TC0411 [Chlamydia muridarum Nigg]
>gi|15835115|ref|NP_296874.1| hypothetical protein TC0497 [Chlamydia muridarum Nigg]
>gi|15835116|ref|NP_296875.1| hypothetical protein TC0498 [Chlamydia muridarum Nigg]
>gi|15835117|ref|NP_296876.1| hypothetical protein TC0499 [Chlamydia muridarum Nigg]
>gi|15835118|ref|NP_296877.1| hypothetical protein TC0500 [Chlamydia muridarum Nigg]
>gi|15835121|ref|NP_296880.1| inclusion membrane protein B [Chlamydia muridarum Nigg]
>gi|15835132|ref|NP_296891.1| UDP-3-O-(R-3-hydroxymyristoyl)-glucosamine N-acyltransferase [Chlamydia muridarum Nigg]
>gi|15835138|ref|NP_296897.1| hypothetical protein TC0520 [Chlamydia muridarum Nigg]
>gi|15835207|ref|NP_296966.1| ribosomal protein L7/L12 [Chlamydia muridarum Nigg]
>gi|15835239|ref|NP_296998.1| hypothetical protein TC0624 [Chlamydia muridarum Nigg]
>gi|15835252|ref|NP_297011.1| hypothetical protein TC0637 [Chlamydia muridarum Nigg]
>gi|15835253|ref|NP_297012.1| BioY family protein [Chlamydia muridarum Nigg]
>gi|15835254|ref|NP_297013.1| hypothetical protein TC0639 [Chlamydia muridarum Nigg]
>gi|15835259|ref|NP_297018.1| hypothetical protein TC0644 [Chlamydia muridarum Nigg]
>gi|15835356|ref|NP_297115.1| hypothetical protein TC0741 [Chlamydia muridarum Nigg]
>gi|15835412|ref|NP_297171.1| ribosomal protein L15 [Chlamydia muridarum Nigg]
>gi|15835459|ref|NP_297218.1| hypothetical protein TC0845 [Chlamydia muridarum Nigg]
>gi|15835481|ref|NP_297240.1| hypothetical protein TC0867 [Chlamydia muridarum Nigg]
>gi|15835482|ref|NP_297241.1| hypothetical protein TC0868 [Chlamydia muridarum Nigg]
>gi|15835501|ref|NP_297260.1| hypothetical protein TC0887 [Chlamydia muridarum Nigg]
>gi|15835510|ref|NP_297269.1| hypothetical protein TC0896 [Chlamydia muridarum Nigg]
>gi|67458400|ref|YP_246024.1| UDP-3-O-[3-hydroxymyristoyl] glucosamine N-acyltransferase [Rickettsia felis URRWXCal2]
>gi|67458439|ref|YP_246063.1| Cell surface antigen-like protein Sca10 [Rickettsia felis URRWXCal2]
>gi|67458474|ref|YP_246098.1| hypothetical protein RF_0082 [Rickettsia felis URRWXCal2]
>gi|67458506|ref|YP_246130.1| hypothetical protein RF_0114 [Rickettsia felis URRWXCal2]
>gi|67458522|ref|YP_246146.1| CDP-diacylglycerol--glycerol-3-phosphate 3-phosphatidyltransferase [Rickettsia felis URRWXCal2]
>gi|67458541|ref|YP_246165.1| ClpB protein [Rickettsia felis URRWXCal2]
>gi|67458581|ref|YP_246205.1| hypothetical protein RF_0189 [Rickettsia felis URRWXCal2]
>gi|67458672|ref|YP_246296.1| 50S ribosomal protein L23 [Rickettsia felis URRWXCal2]
>gi|67458678|ref|YP_246302.1| 50S ribosomal protein L29 [Rickettsia felis URRWXCal2]
>gi|67458689|ref|YP_246313.1| 50S ribosomal protein L15 [Rickettsia felis URRWXCal2]
>gi|67458709|ref|YP_246333.1| hypothetical protein RF_0317 [Rickettsia felis URRWXCal2]
>gi|67458737|ref|YP_246361.1| hypothetical protein RF_0345 [Rickettsia felis URRWXCal2]

>gi|67458738|ref|YP_246362.1| hypothetical protein RF_0346 [Rickettsia felis URRWXCal2]
>gi|67458763|ref|YP_246387.1| Actin polymerization protein RickA [Rickettsia felis URRWXCal2]
>gi|67458793|ref|YP_246417.1| Permease PerM homolog [Rickettsia felis URRWXCal2]
>gi|67458807|ref|YP_246431.1| Zinc/manganese ABC transporter permease protein [Rickettsia felis URRWXCal2]
>gi|67458826|ref|YP_246450.1| hypothetical protein RF_0434 [Rickettsia felis URRWXCal2]
>gi|67458835|ref|YP_246459.1| hypothetical protein RF_0443 [Rickettsia felis URRWXCal2]
>gi|67458850|ref|YP_246474.1| NADH dehydrogenase I chain L [Rickettsia felis URRWXCal2]
>gi|67458859|ref|YP_246483.1| VirB10 protein [Rickettsia felis URRWXCal2]
>gi|67458898|ref|YP_246522.1| Periplasmic protein TonB [Rickettsia felis URRWXCal2]
>gi|67459066|ref|YP_246690.1| hypothetical protein RF_0674 [Rickettsia felis URRWXCal2]
>gi|67459085|ref|YP_246709.1| Cell surface antigen Sca3 [Rickettsia felis URRWXCal2]
>gi|67459119|ref|YP_246743.1| Ribonuclease BN, putative [Rickettsia felis URRWXCal2]
>gi|67459134|ref|YP_246758.1| Predicted esterase of the alpha-beta hydrolase superfamily [Rickettsia felis URRWXCal2]
>gi|67459160|ref|YP_246784.1| hypothetical protein RF_0768 [Rickettsia felis URRWXCal2]
>gi|67459225|ref|YP_246849.1| hypothetical protein RF_0833 [Rickettsia felis URRWXCal2]
>gi|67459241|ref|YP_246865.1| hypothetical protein RF_0849 [Rickettsia felis URRWXCal2]
>gi|67459256|ref|YP_246880.1| Uncharacterized low-complexity protein [Rickettsia felis URRWXCal2]
>gi|67459262|ref|YP_246886.1| hypothetical protein RF_0870 [Rickettsia felis URRWXCal2]
>gi|67459271|ref|YP_246895.1| hypothetical protein RF_0879 [Rickettsia felis URRWXCal2]
>gi|67459275|ref|YP_246899.1| hypothetical protein RF_0883 [Rickettsia felis URRWXCal2]
>gi|67459288|ref|YP_246912.1| Uncharacterized low-complexity protein [Rickettsia felis URRWXCal2]
>gi|67459321|ref|YP_246945.1| hypothetical protein RF_0929 [Rickettsia felis URRWXCal2]
>gi|67459344|ref|YP_246968.1| hypothetical protein RF_0952 [Rickettsia felis URRWXCal2]
>gi|67459393|ref|YP_247017.1| Peptide chain release factor RF-2 [Rickettsia felis URRWXCal2]
>gi|67459418|ref|YP_247042.1| hypothetical protein RF_1026 [Rickettsia felis URRWXCal2]
>gi|67459419|ref|YP_247043.1| hypothetical protein RF_1027 [Rickettsia felis URRWXCal2]
>gi|67459433|ref|YP_247057.1| hypothetical protein RF_1041 [Rickettsia felis URRWXCal2]
>gi|67459508|ref|YP_247132.1| hypothetical protein RF_1116 [Rickettsia felis URRWXCal2]
>gi|67459539|ref|YP_247163.1| 50S ribosomal protein L7/L12 [Rickettsia felis URRWXCal2]
>gi|67459618|ref|YP_247242.1| hypothetical protein RF_1226 [Rickettsia felis URRWXCal2]
>gi|67459640|ref|YP_247264.1| Sec-independent protein translocase protein TatC [Rickettsia felis URRWXCal2]
>gi|67459681|ref|YP_247305.1| hypothetical protein RF_1289 [Rickettsia felis URRWXCal2]
>gi|67459682|ref|YP_247306.1| Cell surface antigen-like protein Sca13 [Rickettsia felis URRWXCal2]
>gi|67459695|ref|YP_247319.1| hypothetical protein RF_1303 [Rickettsia felis URRWXCal2]
>gi|67459697|ref|YP_247321.1| Outer memberane protein rOmpA [Rickettsia felis URRWXCal2]
>gi|67459705|ref|YP_247329.1| hypothetical protein RF_1313 [Rickettsia felis URRWXCal2]
>gi|67459713|ref|YP_247337.1| 17 kDa surface antigen precursor [Rickettsia felis URRWXCal2]
>gi|67459721|ref|YP_247345.1| hypothetical protein RF_1329 [Rickettsia felis URRWXCal2]
>gi|67459723|ref|YP_247347.1| hypothetical protein RF_1331 [Rickettsia felis URRWXCal2]
>gi|67459770|ref|YP_247394.1| Type I restriction-modification system methyltransferase subunit [Rickettsia felis URRWXCal2]
>gi|67459786|ref|YP_247410.1| TPR [Rickettsia felis URRWXCal2]
>gi|67459800|ref|YP_247423.1| Type I restriction-modification system methyltransferase subunit [Rickettsia felis URRWXCal2]
>gi|67459802|ref|YP_247425.1| hypothetical protein RF_p09 [Rickettsia felis URRWXCal2]
>gi|67459869|ref|YP_247491.1| Type I restriction-modification system methyltransferase subunit [Rickettsia felis URRWXCal2]
>gi|67459871|ref|YP_247493.1| hypothetical protein RF_pd09 [Rickettsia felis URRWXCal2]
>gi|15611104|ref|NP_222755.1| hypothetical protein jhp0033 [Helicobacter pylori J99]
>gi|15611121|ref|NP_222772.1| hypothetical protein jhp0050 [Helicobacter pylori J99]
>gi|15611123|ref|NP_222774.1| hypothetical protein jhp0052 [Helicobacter pylori J99]
>gi|15611124|ref|NP_222775.1| hypothetical protein jhp0053 [Helicobacter pylori J99]
>gi|15611131|ref|NP_222782.1| hypothetical protein jhp0060 [Helicobacter pylori J99]
>gi|15611145|ref|NP_222796.1| hypothetical protein jhp0074 [Helicobacter pylori J99]
>gi|15611180|ref|NP_222831.1| hypothetical protein jhp0110 [Helicobacter pylori J99]
>gi|15611209|ref|NP_222860.1| hypothetical protein jhp0139 [Helicobacter pylori J99]
>gi|15611213|ref|NP_222864.1| hypothetical protein jhp0143 [Helicobacter pylori J99]
>gi|15611226|ref|NP_222877.1| hypothetical protein jhp0156 [Helicobacter pylori J99]
>gi|15611239|ref|NP_222890.1| hypothetical protein jhp0169 [Helicobacter pylori J99]
>gi|15611243|ref|NP_222894.1| hypothetical protein jhp0173 [Helicobacter pylori J99]
>gi|15611252|ref|NP_222903.1| UDP-3-O-[3-hydroxymyristoyl [Helicobacter pylori J99]
>gi|15611281|ref|NP_222932.1| hypothetical protein jhp0211 [Helicobacter pylori J99]
>gi|15611287|ref|NP_222938.1| motility protein [Helicobacter pylori J99]
>gi|15611297|ref|NP_222948.1| hypothetical protein jhp0227 [Helicobacter pylori J99]
>gi|15611304|ref|NP_222955.1| hypothetical protein jhp0234 [Helicobacter pylori J99]
>gi|15611310|ref|NP_222961.1| hypothetical protein jhp0240 [Helicobacter pylori J99]
>gi|15611311|ref|NP_222962.1| hypothetical protein jhp0241 [Helicobacter pylori J99]
>gi|15611322|ref|NP_222973.1| hypothetical protein jhp0252 [Helicobacter pylori J99]
>gi|15611362|ref|NP_223013.1| hypothetical protein jhp0293 [Helicobacter pylori J99]
>gi|15611368|ref|NP_223019.1| hypothetical protein jhp0299 [Helicobacter pylori J99]
>gi|15611374|ref|NP_223025.1| hypothetical protein jhp0305 [Helicobacter pylori J99]
>gi|15611387|ref|NP_223038.1| hypothetical protein jhp0319 [Helicobacter pylori J99]
>gi|15611388|ref|NP_223039.1| hypothetical protein jhp0320 [Helicobacter pylori J99]

>gi|15611392|ref|NP_223043.1| hypothetical protein jhp0324 [Helicobacter pylori J99]
>gi|15611475|ref|NP_223126.1| PHOSPHATIDYLGLYCEROPHOSPHATE SYNTHASE [Helicobacter pylori J99]
>gi|15611476|ref|NP_223127.1| hypothetical protein jhp0408 [Helicobacter pylori J99]
>gi|15611490|ref|NP_223141.1| GLUTATHIONE-REGULATED POTASSIUM-EFFLUX SYSTEM PROTEIN [Helicobacter pylori J99]
>gi|15611525|ref|NP_223176.1| hypothetical protein jhp0458 [Helicobacter pylori J99]
>gi|15611530|ref|NP_223181.1| 50S RIBOSOMAL PROTEIN L9 [Helicobacter pylori J99]
>gi|15611538|ref|NP_223189.1| cag island protein [Helicobacter pylori J99]
>gi|15611543|ref|NP_223194.1| cag island protein [Helicobacter pylori J99]
>gi|15611585|ref|NP_223236.1| hypothetical protein jhp0518 [Helicobacter pylori J99]
>gi|15611593|ref|NP_223244.1| hypothetical protein jhp0526 [Helicobacter pylori J99]
>gi|15611596|ref|NP_223247.1| putative SIDEROPHORE-MEDIATED IRON TRANSPORT PROTEIN [Helicobacter pylori J99]
>gi|15611616|ref|NP_223267.1| ENDONUCLEASE III [Helicobacter pylori J99]
>gi|15611624|ref|NP_223275.1| hypothetical protein jhp0557 [Helicobacter pylori J99]
>gi|15611646|ref|NP_223297.1| hypothetical protein jhp0579 [Helicobacter pylori J99]
>gi|15611647|ref|NP_223298.1| hypothetical protein jhp0580 [Helicobacter pylori J99]
>gi|15611686|ref|NP_223337.1| hypothetical protein jhp0619 [Helicobacter pylori J99]
>gi|15611690|ref|NP_223341.1| hypothetical protein jhp0623 [Helicobacter pylori J99]
>gi|15611723|ref|NP_223374.1| hypothetical protein jhp0656 [Helicobacter pylori J99]
>gi|15611725|ref|NP_223376.1| hypothetical protein jhp0658 [Helicobacter pylori J99]
>gi|15611758|ref|NP_223409.1| hypothetical protein jhp0691 [Helicobacter pylori J99]
>gi|15611760|ref|NP_223411.1| hypothetical protein jhp0693 [Helicobacter pylori J99]
>gi|15611776|ref|NP_223427.1| putative PROBABLE N-ACETYLMURAMOYL-L-ALANINE AMIDASE [Helicobacter pylori J99]
>gi|15611789|ref|NP_223440.1| hypothetical protein jhp0722 [Helicobacter pylori J99]
>gi|15611826|ref|NP_223477.1| hypothetical protein jhp0759 [Helicobacter pylori J99]
>gi|15611884|ref|NP_223535.1| hypothetical protein jhp0817 [Helicobacter pylori J99]
>gi|15611889|ref|NP_223540.1| IRON(III) DICITRATE TRANSPORT SYSTEM PERMEASE PROTEIN [Helicobacter pylori J99]
>gi|15611909|ref|NP_223560.1| hypothetical protein jhp0842 [Helicobacter pylori J99]
>gi|15611910|ref|NP_223561.1| putative FLAGELLAR BIOSYNTHESIS PROTEIN [Helicobacter pylori J99]
>gi|15611923|ref|NP_223574.1| putative vacuolating cytotoxin (VacA) paralog [Helicobacter pylori J99]
>gi|15611959|ref|NP_223610.1| hypothetical protein jhp0892 [Helicobacter pylori J99]
>gi|15611989|ref|NP_223641.1| hypothetical protein jhp0924 [Helicobacter pylori J99]
>gi|15611990|ref|NP_223642.1| hypothetical protein jhp0925 [Helicobacter pylori J99]
>gi|15611994|ref|NP_223646.1| hypothetical protein jhp0929 [Helicobacter pylori J99]
>gi|15612010|ref|NP_223662.1| hypothetical protein jhp0945 [Helicobacter pylori J99]
>gi|15612011|ref|NP_223663.1| hypothetical protein jhp0946 [Helicobacter pylori J99]
>gi|15612017|ref|NP_223669.1| hypothetical protein jhp0952 [Helicobacter pylori J99]
>gi|15612018|ref|NP_223670.1| hypothetical protein jhp0953 [Helicobacter pylori J99]
>gi|15612061|ref|NP_223713.1| hypothetical protein jhp0996 [Helicobacter pylori J99]
>gi|15612064|ref|NP_223716.1| putative ZINC-METALLO PROTEASE [Helicobacter pylori J99]
>gi|15612107|ref|NP_223759.1| hypothetical protein jhp1042 [Helicobacter pylori J99]
>gi|15612121|ref|NP_223773.1| hypothetical protein jhp1056 [Helicobacter pylori J99]
>gi|15612136|ref|NP_223788.1| hypothetical protein jhp1071 [Helicobacter pylori J99]
>gi|15612149|ref|NP_223801.1| putative Outer membrane protein [Helicobacter pylori J99]
>gi|15612157|ref|NP_223809.1| hypothetical protein jhp1092 [Helicobacter pylori J99]
>gi|15612178|ref|NP_223830.1| hypothetical protein jhp1113 [Helicobacter pylori J99]
>gi|15612182|ref|NP_223834.1| putative motility protein [Helicobacter pylori J99]
>gi|15612198|ref|NP_223851.1| O-SERINE ACETYLTRANSFERASE [Helicobacter pylori J99]
>gi|15612200|ref|NP_223853.1| ATP synthase F0, subunit c [Helicobacter pylori J99]
>gi|15612216|ref|NP_223869.1| hypothetical protein jhp1151 [Helicobacter pylori J99]
>gi|15612236|ref|NP_223889.1| hypothetical protein jhp1171 [Helicobacter pylori J99]
>gi|15612241|ref|NP_223894.1| PROTEIN-EXPORT MEMBRANE PROTEIN [Helicobacter pylori J99]
>gi|15612273|ref|NP_223926.1| hypothetical protein jhp1208 [Helicobacter pylori J99]
>gi|15612293|ref|NP_223946.1| 50S RIBOSOMAL PROTEIN L24 [Helicobacter pylori J99]
>gi|15612325|ref|NP_223978.1| SIDEROPHORE-MEDIATED IRON TRANSPORT PROTEIN [Helicobacter pylori J99]
>gi|15612342|ref|NP_223995.1| hypothetical protein jhp1277 [Helicobacter pylori J99]
>gi|15612344|ref|NP_223997.1| DNA transfer protein [Helicobacter pylori J99]
>gi|15612364|ref|NP_224017.1| putative ribonuclease N [Helicobacter pylori J99]
>gi|15612365|ref|NP_224018.1| hypothetical protein jhp1300 [Helicobacter pylori J99]
>gi|15612385|ref|NP_224038.1| putative histidine-rich metal-binding protein [Helicobacter pylori J99]
>gi|15612386|ref|NP_224039.1| putative histidine and glutamine-rich metal-binding protein [Helicobacter pylori J99]
>gi|15612409|ref|NP_224062.1| hypothetical protein jhp1344 [Helicobacter pylori J99]
>gi|15612449|ref|NP_224102.1| putative PHOSPHATE PERMEASE [Helicobacter pylori J99]
>gi|15612499|ref|NP_224152.1| DNA REPAIR PROTEIN(RECOMBINATION PROTEIN N) [Helicobacter pylori J99]
>gi|15612539|ref|NP_224192.1| hypothetical protein jhp1474 [Helicobacter pylori J99]
>gi|46578426|ref|YP_009234.1| TRAP transporter, DctM subunit [Desulfovibrio vulgaris subsp. vulgaris str. Hildenborough]
>gi|46578449|ref|YP_009257.1| hypothetical protein DVU0032 [Desulfovibrio vulgaris subsp. vulgaris str. Hildenborough]
>gi|46578459|ref|YP_009267.1| RNA methyltransferase, TrmH family, group 3 [Desulfovibrio vulgaris subsp. vulgaris str. Hildenborough]
>gi|46578461|ref|YP_009269.1| flagellar biosynthetic protein fliP [Desulfovibrio vulgaris subsp. vulgaris str. Hildenborough]
>gi|46578462|ref|YP_009270.1| flagellar biosynthesis protein, FliO, putative [Desulfovibrio vulgaris subsp. vulgaris str. Hildenborough]
>gi|46578475|ref|YP_009283.1| efflux transporter, RND family, MFP subunit [Desulfovibrio vulgaris subsp. vulgaris str. Hildenborough]

>gi|46578486|ref|YP_009294.1| hypothetical protein DVU0069 [Desulfovibrio vulgaris subsp. vulgaris str. Hildenborough]
>gi|46578496|ref|YP_009304.1| ZIP zinc transporter family protein [Desulfovibrio vulgaris subsp. vulgaris str. Hildenborough]
>gi|46578511|ref|YP_009319.1| methyl-accepting chemotaxis protein [Desulfovibrio vulgaris subsp. vulgaris str. Hildenborough]
>gi|46578516|ref|YP_009324.1| TonB domain protein [Desulfovibrio vulgaris subsp. vulgaris str. Hildenborough]
>gi|46578521|ref|YP_009329.1| cation ABC transporter, permease protein, putative [Desulfovibrio vulgaris subsp. vulgaris str. Hildenborough]
>gi|46578542|ref|YP_009350.1| hypothetical protein DVU0125 [Desulfovibrio vulgaris subsp. vulgaris str. Hildenborough]
>gi|46578543|ref|YP_009351.1| ABC transporter, ATP-binding protein [Desulfovibrio vulgaris subsp. vulgaris str. Hildenborough]
>gi|46578545|ref|YP_009353.1| membrane protein, putative [Desulfovibrio vulgaris subsp. vulgaris str. Hildenborough]
>gi|46578550|ref|YP_009358.1| hypothetical protein DVU0133 [Desulfovibrio vulgaris subsp. vulgaris str. Hildenborough]
>gi|46578566|ref|YP_009374.1| membrane protein, putative [Desulfovibrio vulgaris subsp. vulgaris str. Hildenborough]
>gi|46578576|ref|YP_009384.1| thioesterase family protein [Desulfovibrio vulgaris subsp. vulgaris str. Hildenborough]
>gi|46578594|ref|YP_009402.1| molybdenum ABC transporter, periplasmic molybdenum-binding protein [Desulfovibrio vulgaris subsp. vulgaris str. Hildenborough]
>gi|46578600|ref|YP_009408.1| methyl-accepting chemotaxis protein [Desulfovibrio vulgaris subsp. vulgaris str. Hildenborough]
>gi|46578631|ref|YP_009439.1| tail/DNA circulation protein, putative [Desulfovibrio vulgaris subsp. vulgaris str. Hildenborough]
>gi|46578635|ref|YP_009443.1| tail protein, putative [Desulfovibrio vulgaris subsp. vulgaris str. Hildenborough]
>gi|46578638|ref|YP_009446.1| tail fiber assembly protein, putative [Desulfovibrio vulgaris subsp. vulgaris str. Hildenborough]
>gi|46578661|ref|YP_009469.1| hypothetical protein DVU0244 [Desulfovibrio vulgaris subsp. vulgaris str. Hildenborough]
>gi|46578710|ref|YP_009518.1| glycosyl transferase, group 2 family protein [Desulfovibrio vulgaris subsp. vulgaris str. Hildenborough]
>gi|46578727|ref|YP_009535.1| flagellar assembly protein FliH, putative [Desulfovibrio vulgaris subsp. vulgaris str. Hildenborough]
>gi|46578734|ref|YP_009542.1| TPR domain protein [Desulfovibrio vulgaris subsp. vulgaris str. Hildenborough]
>gi|46578761|ref|YP_009569.1| hypothetical protein DVU0345 [Desulfovibrio vulgaris subsp. vulgaris str. Hildenborough]
>gi|46578770|ref|YP_009578.1| hypothetical protein DVU0354 [Desulfovibrio vulgaris subsp. vulgaris str. Hildenborough]
>gi|46578782|ref|YP_009590.1| 5-formyltetrahydrofolate cyclo-ligase family protein [Desulfovibrio vulgaris subsp. vulgaris str. Hildenborough]
>gi|46578815|ref|YP_009623.1| hypothetical protein DVU0399 [Desulfovibrio vulgaris subsp. vulgaris str. Hildenborough]
>gi|46578825|ref|YP_009633.1| hypothetical protein DVU0409 [Desulfovibrio vulgaris subsp. vulgaris str. Hildenborough]
>gi|46578860|ref|YP_009668.1| CBS domain protein [Desulfovibrio vulgaris subsp. vulgaris str. Hildenborough]
>gi|46578868|ref|YP_009676.1| hypothetical protein DVU0452 [Desulfovibrio vulgaris subsp. vulgaris str. Hildenborough]
>gi|46578884|ref|YP_009692.1| indole-3-glycerol phosphate synthase [Desulfovibrio vulgaris subsp. vulgaris str. Hildenborough]
>gi|46578894|ref|YP_009702.1| Ser/Thr protein phosphatase family [Desulfovibrio vulgaris subsp. vulgaris str. Hildenborough]
>gi|46578903|ref|YP_009711.1| phosphoribosylaminoimidazole carboxylase, catalytic subunit [Desulfovibrio vulgaris subsp. vulgaris str. Hildenborough]
>gi|46578917|ref|YP_009725.1| hypothetical protein DVU0502 [Desulfovibrio vulgaris subsp. vulgaris str. Hildenborough]
>gi|46578923|ref|YP_009731.1| translation initiation factor IF-2 [Desulfovibrio vulgaris subsp. vulgaris str. Hildenborough]
>gi|46578932|ref|YP_009740.1| peptidase, M23/M37 family [Desulfovibrio vulgaris subsp. vulgaris str. Hildenborough]
>gi|46578950|ref|YP_009758.1| hmc operon protein 2 [Desulfovibrio vulgaris subsp. vulgaris str. Hildenborough]
>gi|46578964|ref|YP_009772.1| high-affinity branched-chain amino acid ABC transporter, permease protein [Desulfovibrio vulgaris subsp. vulgaris str. Hildenborough]
>gi|46578985|ref|YP_009793.1| alanine dehydrogenase [Desulfovibrio vulgaris subsp. vulgaris str. Hildenborough]
>gi|46578988|ref|YP_009796.1| hypothetical protein DVU0574 [Desulfovibrio vulgaris subsp. vulgaris str. Hildenborough]
>gi|46579017|ref|YP_009825.1| hypothetical protein DVU0603 [Desulfovibrio vulgaris subsp. vulgaris str. Hildenborough]
>gi|46579041|ref|YP_009849.1| phosphotransbutyrylase [Desulfovibrio vulgaris subsp. vulgaris str. Hildenborough]
>gi|46579062|ref|YP_009870.1| iron compound ABC transporter, ATP-binding protein [Desulfovibrio vulgaris subsp. vulgaris str. Hildenborough]
>gi|46579063|ref|YP_009871.1| iron compound ABC transporter, permease protein [Desulfovibrio vulgaris subsp. vulgaris str. Hildenborough]
>gi|46579065|ref|YP_009873.1| membrane protein, putative [Desulfovibrio vulgaris subsp. vulgaris str. Hildenborough]
>gi|46579082|ref|YP_009890.1| methyl-accepting chemotaxis protein [Desulfovibrio vulgaris subsp. vulgaris str. Hildenborough]
>gi|46579088|ref|YP_009896.1| ABC transporter, permease protein, His/Glu/Gln/Arg/opine family [Desulfovibrio vulgaris subsp. vulgaris str. Hildenborough]
>gi|46579090|ref|YP_009898.1| amino acid ABC transporter, permease protein, His/Glu/Gln/Arg/opine family [Desulfovibrio vulgaris subsp. vulgaris str. Hildenborough]
>gi|46579126|ref|YP_009934.1| branched-chain amino acid ABC transporter, permease protein [Desulfovibrio vulgaris subsp. vulgaris str. Hildenborough]
>gi|46579162|ref|YP_009970.1| DNA-binding response regulator [Desulfovibrio vulgaris subsp. vulgaris str. Hildenborough]
>gi|46579173|ref|YP_009981.1| hypothetical protein DVU0760 [Desulfovibrio vulgaris subsp. vulgaris str. Hildenborough]
>gi|46579187|ref|YP_009995.1| ATP synthase, F1 epsilon subunit [Desulfovibrio vulgaris subsp. vulgaris str. Hildenborough]
>gi|46579193|ref|YP_010001.1| ATP synthase F0, B' subunit, putative [Desulfovibrio vulgaris subsp. vulgaris str. Hildenborough]
>gi|46579197|ref|YP_010005.1| hypothetical protein DVU0784 [Desulfovibrio vulgaris subsp. vulgaris str. Hildenborough]
>gi|46579200|ref|YP_010008.1| hypothetical protein DVU0787 [Desulfovibrio vulgaris subsp. vulgaris str. Hildenborough]
>gi|46579216|ref|YP_010024.1| sensor histidine kinase [Desulfovibrio vulgaris subsp. vulgaris str. Hildenborough]
>gi|46579231|ref|YP_010039.1| hypothetical protein DVU0818 [Desulfovibrio vulgaris subsp. vulgaris str. Hildenborough]
>gi|46579243|ref|YP_010051.1| phosphocarrier protein HPr [Desulfovibrio vulgaris subsp. vulgaris str. Hildenborough]
>gi|46579251|ref|YP_010059.1| hypothetical protein DVU0838 [Desulfovibrio vulgaris subsp. vulgaris str. Hildenborough]
>gi|46579256|ref|YP_010064.1| hypothetical protein DVU0843 [Desulfovibrio vulgaris subsp. vulgaris str. Hildenborough]
>gi|46579264|ref|YP_010072.1| hypothetical protein DVU0851 [Desulfovibrio vulgaris subsp. vulgaris str. Hildenborough]
>gi|46579272|ref|YP_010080.1| hypothetical protein DVU0859 [Desulfovibrio vulgaris subsp. vulgaris str. Hildenborough]
>gi|46579275|ref|YP_010083.1| flagellar protein FliS/hypothetical protein, fusion [Desulfovibrio vulgaris subsp. vulgaris str. Hildenborough]
>gi|46579295|ref|YP_010103.1| hypothetical protein DVU0882 [Desulfovibrio vulgaris subsp. vulgaris str. Hildenborough]
>gi|46579320|ref|YP_010128.1| hypothetical protein DVU0907 [Desulfovibrio vulgaris subsp. vulgaris str. Hildenborough]
>gi|46579325|ref|YP_010133.1| hypothetical protein DVU0912 [Desulfovibrio vulgaris subsp. vulgaris str. Hildenborough]
>gi|46579326|ref|YP_010134.1| hypothetical protein DVU0913 [Desulfovibrio vulgaris subsp. vulgaris str. Hildenborough]

>gi|46579330|ref|YP_010138.1| ATP synthase F0, C subunit [Desulfovibrio vulgaris subsp. vulgaris str. Hildenborough]
>gi|46579382|ref|YP_010190.1| efflux protein, LysE family [Desulfovibrio vulgaris subsp. vulgaris str. Hildenborough]
>gi|46579393|ref|YP_010201.1| DAK2 domain protein [Desulfovibrio vulgaris subsp. vulgaris str. Hildenborough]
>gi|46579394|ref|YP_010202.1| multiphosphoryl transfer protein, putative [Desulfovibrio vulgaris subsp. vulgaris str. Hildenborough]
>gi|46579407|ref|YP_010215.1| hypothetical protein DVU0994 [Desulfovibrio vulgaris subsp. vulgaris str. Hildenborough]
>gi|46579416|ref|YP_010224.1| dnaJ domain protein [Desulfovibrio vulgaris subsp. vulgaris str. Hildenborough]
>gi|46579418|ref|YP_010226.1| hypothetical protein DVU1005 [Desulfovibrio vulgaris subsp. vulgaris str. Hildenborough]
>gi|46579422|ref|YP_010230.1| hypothetical protein DVU1009 [Desulfovibrio vulgaris subsp. vulgaris str. Hildenborough]
>gi|46579440|ref|YP_010248.1| hypothetical protein DVU1027 [Desulfovibrio vulgaris subsp. vulgaris str. Hildenborough]
>gi|46579445|ref|YP_010253.1| hypothetical protein DVU1032 [Desulfovibrio vulgaris subsp. vulgaris str. Hildenborough]
>gi|46579446|ref|YP_010254.1| competence/damage-inducible protein CinA protein, truncation [Desulfovibrio vulgaris subsp. vulgaris str. Hildenborough]
>gi|46579455|ref|YP_010263.1| twin-arginine translocation protein TatB [Desulfovibrio vulgaris subsp. vulgaris str. Hildenborough]
>gi|46579461|ref|YP_010269.1| cytochrome c-type biogenesis protein CcmB [Desulfovibrio vulgaris subsp. vulgaris str. Hildenborough]
>gi|46579470|ref|YP_010278.1| cobalt ABC transporter, permease protein, putative [Desulfovibrio vulgaris subsp. vulgaris str. Hildenborough]
>gi|46579491|ref|YP_010299.1| R3H domain protein [Desulfovibrio vulgaris subsp. vulgaris str. Hildenborough]
>gi|46579510|ref|YP_010318.1| hypothetical protein DVU1097 [Desulfovibrio vulgaris subsp. vulgaris str. Hildenborough]
>gi|46579512|ref|YP_010320.1| tail fiber assembly protein, putative [Desulfovibrio vulgaris subsp. vulgaris str. Hildenborough]
>gi|46579525|ref|YP_010333.1| hypothetical protein DVU1112 [Desulfovibrio vulgaris subsp. vulgaris str. Hildenborough]
>gi|46579534|ref|YP_010342.1| hypothetical protein DVU1121 [Desulfovibrio vulgaris subsp. vulgaris str. Hildenborough]
>gi|46579540|ref|YP_010348.1| hypothetical protein DVU1127 [Desulfovibrio vulgaris subsp. vulgaris str. Hildenborough]
>gi|46579596|ref|YP_010404.1| colicin V production family protein [Desulfovibrio vulgaris subsp. vulgaris str. Hildenborough]
>gi|46579598|ref|YP_010406.1| hypothetical protein DVU1187 [Desulfovibrio vulgaris subsp. vulgaris str. Hildenborough]
>gi|46579600|ref|YP_010408.1| hypothetical protein DVU1189 [Desulfovibrio vulgaris subsp. vulgaris str. Hildenborough]
>gi|46579603|ref|YP_010411.1| acylphosphatase [Desulfovibrio vulgaris subsp. vulgaris str. Hildenborough]
>gi|46579604|ref|YP_010412.1| DNA repair protein RadC [Desulfovibrio vulgaris subsp. vulgaris str. Hildenborough]
>gi|46579623|ref|YP_010431.1| fxsA protein [Desulfovibrio vulgaris subsp. vulgaris str. Hildenborough]
>gi|46579625|ref|YP_010433.1| dolichyl-phosphate-mannose-protein mannosyltransferase family protein [Desulfovibrio vulgaris subsp. vulgaris str. Hildenborough]
>gi|46579645|ref|YP_010453.1| membrane protein, putative [Desulfovibrio vulgaris subsp. vulgaris str. Hildenborough]
>gi|46579658|ref|YP_010466.1| hypothetical protein DVU1247 [Desulfovibrio vulgaris subsp. vulgaris str. Hildenborough]
>gi|46579660|ref|YP_010468.1| malonyl CoA-acyl carrier protein transacylase [Desulfovibrio vulgaris subsp. vulgaris str. Hildenborough]
>gi|46579676|ref|YP_010484.1| hypothetical protein DVU1265 [Desulfovibrio vulgaris subsp. vulgaris str. Hildenborough]
>gi|46579678|ref|YP_010486.1| hypothetical protein DVU1267 [Desulfovibrio vulgaris subsp. vulgaris str. Hildenborough]
>gi|46579752|ref|YP_010560.1| cation ABC transporter, permease protein [Desulfovibrio vulgaris subsp. vulgaris str. Hildenborough]
>gi|46579768|ref|YP_010576.1| hypothetical protein DVU1357 [Desulfovibrio vulgaris subsp. vulgaris str. Hildenborough]
>gi|46579794|ref|YP_010602.1| hypothetical protein DVU1383 [Desulfovibrio vulgaris subsp. vulgaris str. Hildenborough]
>gi|46579797|ref|YP_010605.1| membrane protein, putative [Desulfovibrio vulgaris subsp. vulgaris str. Hildenborough]
>gi|46579803|ref|YP_010611.1| NLP/P60 family protein [Desulfovibrio vulgaris subsp. vulgaris str. Hildenborough]
>gi|46579816|ref|YP_010624.1| hypothetical protein DVU1405 [Desulfovibrio vulgaris subsp. vulgaris str. Hildenborough]
>gi|46579844|ref|YP_010652.1| hypothetical protein DVU1433 [Desulfovibrio vulgaris subsp. vulgaris str. Hildenborough]
>gi|46579845|ref|YP_010653.1| hypothetical protein DVU1434 [Desulfovibrio vulgaris subsp. vulgaris str. Hildenborough]
>gi|46579860|ref|YP_010668.1| anti-anti-sigma factor, putative [Desulfovibrio vulgaris subsp. vulgaris str. Hildenborough]
>gi|46579894|ref|YP_010702.1| tail fiber assembly protein, putative [Desulfovibrio vulgaris subsp. vulgaris str. Hildenborough]
>gi|46579945|ref|YP_010753.1| membrane protein, putative [Desulfovibrio vulgaris subsp. vulgaris str. Hildenborough]
>gi|46579953|ref|YP_010761.1| hypothetical protein DVU1542 [Desulfovibrio vulgaris subsp. vulgaris str. Hildenborough]
>gi|46579962|ref|YP_010770.1| HD domain protein [Desulfovibrio vulgaris subsp. vulgaris str. Hildenborough]
>gi|46579965|ref|YP_010773.1| radical SAM domain protein [Desulfovibrio vulgaris subsp. vulgaris str. Hildenborough]
>gi|46580032|ref|YP_010840.1| hypothetical protein DVU1621 [Desulfovibrio vulgaris subsp. vulgaris str. Hildenborough]
>gi|46580050|ref|YP_010858.1| hypothetical protein DVU1639 [Desulfovibrio vulgaris subsp. vulgaris str. Hildenborough]
>gi|46580061|ref|YP_010869.1| hypothetical protein DVU1651 [Desulfovibrio vulgaris subsp. vulgaris str. Hildenborough]
>gi|46580072|ref|YP_010880.1| permease, putative [Desulfovibrio vulgaris subsp. vulgaris str. Hildenborough]
>gi|46580105|ref|YP_010913.1| tail fiber assembly protein, putative [Desulfovibrio vulgaris subsp. vulgaris str. Hildenborough]
>gi|46580123|ref|YP_010931.1| hypothetical protein DVU1713 [Desulfovibrio vulgaris subsp. vulgaris str. Hildenborough]
>gi|46580126|ref|YP_010934.1| hypothetical protein DVU1716 [Desulfovibrio vulgaris subsp. vulgaris str. Hildenborough]
>gi|46580129|ref|YP_010937.1| hypothetical protein DVU1719 [Desulfovibrio vulgaris subsp. vulgaris str. Hildenborough]
>gi|46580153|ref|YP_010961.1| hypothetical protein DVU1743 [Desulfovibrio vulgaris subsp. vulgaris str. Hildenborough]
>gi|46580162|ref|YP_010970.1| hypothetical protein DVU1752 [Desulfovibrio vulgaris subsp. vulgaris str. Hildenborough]
>gi|46580202|ref|YP_011010.1| ribosomal protein S21 [Desulfovibrio vulgaris subsp. vulgaris str. Hildenborough]
>gi|46580223|ref|YP_011031.1| hypothetical protein DVU1813 [Desulfovibrio vulgaris subsp. vulgaris str. Hildenborough]
>gi|46580265|ref|YP_011073.1| hypothetical protein DVU1856 [Desulfovibrio vulgaris subsp. vulgaris str. Hildenborough]
>gi|46580274|ref|YP_011082.1| hypothetical protein DVU1865 [Desulfovibrio vulgaris subsp. vulgaris str. Hildenborough]
>gi|46580281|ref|YP_011089.1| hypothetical protein DVU1872 [Desulfovibrio vulgaris subsp. vulgaris str. Hildenborough]
>gi|46580285|ref|YP_011093.1| dnaJ protein, putative [Desulfovibrio vulgaris subsp. vulgaris str. Hildenborough]
>gi|46580289|ref|YP_011097.1| hypothetical protein DVU1880 [Desulfovibrio vulgaris subsp. vulgaris str. Hildenborough]
>gi|46580297|ref|YP_011105.1| ATP-NAD kinase domain protein [Desulfovibrio vulgaris subsp. vulgaris str. Hildenborough]
>gi|46580361|ref|YP_011169.1| hypothetical protein DVU1952 [Desulfovibrio vulgaris subsp. vulgaris str. Hildenborough]
>gi|46580375|ref|YP_011183.1| hypothetical protein DVU1966 [Desulfovibrio vulgaris subsp. vulgaris str. Hildenborough]
>gi|46580387|ref|YP_011195.1| Na+/H+ antiporter family protein [Desulfovibrio vulgaris subsp. vulgaris str. Hildenborough]
>gi|46580391|ref|YP_011199.1| ATP-dependent RNA helicase RhlE [Desulfovibrio vulgaris subsp. vulgaris str. Hildenborough]

>gi|46580462|ref|YP_011270.1| hypothetical protein DVU2057 [Desulfovibrio vulgaris subsp. vulgaris str. Hildenborough]
>gi|46580475|ref|YP_011283.1| TPR domain protein [Desulfovibrio vulgaris subsp. vulgaris str. Hildenborough]
>gi|46580476|ref|YP_011284.1| membrane protein, putative [Desulfovibrio vulgaris subsp. vulgaris str. Hildenborough]
>gi|46580477|ref|YP_011285.1| chemotaxis protein CheA [Desulfovibrio vulgaris subsp. vulgaris str. Hildenborough]
>gi|46580486|ref|YP_011294.1| hypothetical protein DVU2081 [Desulfovibrio vulgaris subsp. vulgaris str. Hildenborough]
>gi|46580495|ref|YP_011303.1| EF hand domain protein [Desulfovibrio vulgaris subsp. vulgaris str. Hildenborough]
>gi|46580506|ref|YP_011314.1| hypothetical protein DVU2101 [Desulfovibrio vulgaris subsp. vulgaris str. Hildenborough]
>gi|46580510|ref|YP_011318.1| hypothetical protein DVU2105 [Desulfovibrio vulgaris subsp. vulgaris str. Hildenborough]
>gi|46580530|ref|YP_011338.1| TPR domain protein [Desulfovibrio vulgaris subsp. vulgaris str. Hildenborough]
>gi|46580538|ref|YP_011346.1| membrane protein, putative [Desulfovibrio vulgaris subsp. vulgaris str. Hildenborough]
>gi|46580540|ref|YP_011348.1| hypothetical protein DVU2135 [Desulfovibrio vulgaris subsp. vulgaris str. Hildenborough]
>gi|46580591|ref|YP_011399.1| hypothetical protein DVU2186 [Desulfovibrio vulgaris subsp. vulgaris str. Hildenborough]
>gi|46580612|ref|YP_011420.1| hypothetical protein DVU2207 [Desulfovibrio vulgaris subsp. vulgaris str. Hildenborough]
>gi|46580627|ref|YP_011435.1| single-strand binding protein [Desulfovibrio vulgaris subsp. vulgaris str. Hildenborough]
>gi|46580637|ref|YP_011445.1| hypothetical protein DVU2232 [Desulfovibrio vulgaris subsp. vulgaris str. Hildenborough]
>gi|46580642|ref|YP_011450.1| cobalamin biosynthesis protein CobD, putative [Desulfovibrio vulgaris subsp. vulgaris str. Hildenborough]
>gi|46580683|ref|YP_011491.1| membrane protein, putative [Desulfovibrio vulgaris subsp. vulgaris str. Hildenborough]
>gi|46580731|ref|YP_011539.1| hypothetical protein DVU2326 [Desulfovibrio vulgaris subsp. vulgaris str. Hildenborough]
>gi|46580742|ref|YP_011550.1| peptidase, M23/M37 family [Desulfovibrio vulgaris subsp. vulgaris str. Hildenborough]
>gi|46580750|ref|YP_011558.1| hypothetical protein DVU2345 [Desulfovibrio vulgaris subsp. vulgaris str. Hildenborough]
>gi|46580768|ref|YP_011576.1| hydroxyethylthiazole kinase [Desulfovibrio vulgaris subsp. vulgaris str. Hildenborough]
>gi|46580774|ref|YP_011582.1| UDP-3-O-(R-3-hydroxymyristoyl)-glucosamine N-acyltransferase [Desulfovibrio vulgaris subsp. vulgaris str. Hildenborough]
>gi|46580775|ref|YP_011583.1| outer membrane protein OmpH, putative [Desulfovibrio vulgaris subsp. vulgaris str. Hildenborough]
>gi|46580786|ref|YP_011594.1| hypothetical protein DVU2381 [Desulfovibrio vulgaris subsp. vulgaris str. Hildenborough]
>gi|46580795|ref|YP_011603.1| TonB domain protein [Desulfovibrio vulgaris subsp. vulgaris str. Hildenborough]
>gi|46580816|ref|YP_011624.1| EF hand domain protein [Desulfovibrio vulgaris subsp. vulgaris str. Hildenborough]
>gi|46580824|ref|YP_011632.1| hypothetical protein DVU2419 [Desulfovibrio vulgaris subsp. vulgaris str. Hildenborough]
>gi|46580829|ref|YP_011637.1| membrane protein, putative [Desulfovibrio vulgaris subsp. vulgaris str. Hildenborough]
>gi|46580833|ref|YP_011641.1| lipoprotein, putative [Desulfovibrio vulgaris subsp. vulgaris str. Hildenborough]
>gi|46580839|ref|YP_011647.1| hypothetical protein DVU2434 [Desulfovibrio vulgaris subsp. vulgaris str. Hildenborough]
>gi|46580871|ref|YP_011679.1| ribonuclease R [Desulfovibrio vulgaris subsp. vulgaris str. Hildenborough]
>gi|46580878|ref|YP_011686.1| hypothetical protein DVU2474 [Desulfovibrio vulgaris subsp. vulgaris str. Hildenborough]
>gi|46580899|ref|YP_011707.1| thioesterase family protein [Desulfovibrio vulgaris subsp. vulgaris str. Hildenborough]
>gi|46580948|ref|YP_011756.1| iron-sulfur cluster-binding protein [Desulfovibrio vulgaris subsp. vulgaris str. Hildenborough]
>gi|46580951|ref|YP_011759.1| transcriptional regulator, putative [Desulfovibrio vulgaris subsp. vulgaris str. Hildenborough]
>gi|46580960|ref|YP_011768.1| hypothetical protein DVU2556 [Desulfovibrio vulgaris subsp. vulgaris str. Hildenborough]
>gi|46580965|ref|YP_011773.1| oxidoreductase, short chain dehydrogenase/reductase family [Desulfovibrio vulgaris subsp. vulgaris str. Hildenborough]
>gi|46580974|ref|YP_011782.1| GGDEF domain/HAMP domain protein [Desulfovibrio vulgaris subsp. vulgaris str. Hildenborough]
>gi|46580977|ref|YP_011785.1| hypothetical protein DVU2573 [Desulfovibrio vulgaris subsp. vulgaris str. Hildenborough]
>gi|46580984|ref|YP_011792.1| response regulator [Desulfovibrio vulgaris subsp. vulgaris str. Hildenborough]
>gi|46580995|ref|YP_011803.1| tail fiber assembly protein, putative [Desulfovibrio vulgaris subsp. vulgaris str. Hildenborough]
>gi|46580999|ref|YP_011807.1| hypothetical protein DVU2595 [Desulfovibrio vulgaris subsp. vulgaris str. Hildenborough]
>gi|46581003|ref|YP_011811.1| hypothetical protein DVU2599 [Desulfovibrio vulgaris subsp. vulgaris str. Hildenborough]
>gi|46581005|ref|YP_011813.1| hypothetical protein DVU2601 [Desulfovibrio vulgaris subsp. vulgaris str. Hildenborough]
>gi|46581014|ref|YP_011822.1| hypothetical protein DVU2610 [Desulfovibrio vulgaris subsp. vulgaris str. Hildenborough]
>gi|46581034|ref|YP_011842.1| lipoprotein, putative [Desulfovibrio vulgaris subsp. vulgaris str. Hildenborough]
>gi|46581038|ref|YP_011846.1| hypothetical protein DVU2634 [Desulfovibrio vulgaris subsp. vulgaris str. Hildenborough]
>gi|46581040|ref|YP_011848.1| hypothetical protein DVU2636 [Desulfovibrio vulgaris subsp. vulgaris str. Hildenborough]
>gi|46581042|ref|YP_011850.1| hypothetical protein DVU2638 [Desulfovibrio vulgaris subsp. vulgaris str. Hildenborough]
>gi|46581059|ref|YP_011867.1| D-alanyl-D-alanine carboxypeptidase family protein [Desulfovibrio vulgaris subsp. vulgaris str. Hildenborough]
>gi|46581069|ref|YP_011877.1| phosphate ABC transporter, permease protein, putative [Desulfovibrio vulgaris subsp. vulgaris str. Hildenborough]
>gi|46581073|ref|YP_011881.1| hypothetical protein DVU2669 [Desulfovibrio vulgaris subsp. vulgaris str. Hildenborough]
>gi|46581075|ref|YP_011883.1| HDIG/HD/KH domain protein [Desulfovibrio vulgaris subsp. vulgaris str. Hildenborough]
>gi|46581076|ref|YP_011884.1| membrane protein, putative [Desulfovibrio vulgaris subsp. vulgaris str. Hildenborough]
>gi|46581087|ref|YP_011895.1| L-lactate permease family protein [Desulfovibrio vulgaris subsp. vulgaris str. Hildenborough]
>gi|46581104|ref|YP_011912.1| hypothetical protein DVU2700 [Desulfovibrio vulgaris subsp. vulgaris str. Hildenborough]
>gi|46581107|ref|YP_011915.1| hypothetical protein DVU2703 [Desulfovibrio vulgaris subsp. vulgaris str. Hildenborough]
>gi|46581110|ref|YP_011918.1| hypothetical protein DVU2706 [Desulfovibrio vulgaris subsp. vulgaris str. Hildenborough]
>gi|46581114|ref|YP_011922.1| hypothetical protein DVU2710 [Desulfovibrio vulgaris subsp. vulgaris str. Hildenborough]
>gi|46581116|ref|YP_011924.1| hypothetical protein DVU2712 [Desulfovibrio vulgaris subsp. vulgaris str. Hildenborough]
>gi|46581117|ref|YP_011925.1| hypothetical protein DVU2713 [Desulfovibrio vulgaris subsp. vulgaris str. Hildenborough]
>gi|46581120|ref|YP_011928.1| tail sheath protein, putative [Desulfovibrio vulgaris subsp. vulgaris str. Hildenborough]
>gi|46581125|ref|YP_011933.1| phage tail tape measure protein, TP901 family, putative [Desulfovibrio vulgaris subsp. vulgaris str. Hildenborough]
>gi|46581132|ref|YP_011940.1| tail protein, putative [Desulfovibrio vulgaris subsp. vulgaris str. Hildenborough]
>gi|46581135|ref|YP_011943.1| tail fiber assembly protein, putative [Desulfovibrio vulgaris subsp. vulgaris str. Hildenborough]
>gi|46581140|ref|YP_011948.1| hypothetical protein DVU2736 [Desulfovibrio vulgaris subsp. vulgaris str. Hildenborough]
>gi|46581154|ref|YP_011962.1| cobalamin biosynthesis protein CbiD [Desulfovibrio vulgaris subsp. vulgaris str. Hildenborough]
>gi|46581155|ref|YP_011963.1| hypothetical protein DVU2751 [Desulfovibrio vulgaris subsp. vulgaris str. Hildenborough]

>gi|46581157|ref|YP_011965.1| C_GCAxxG_C_C family protein [Desulfovibrio vulgaris subsp. vulgaris str. Hildenborough]
>gi|46581166|ref|YP_011974.1| membrane protein, putative [Desulfovibrio vulgaris subsp. vulgaris str. Hildenborough]
>gi|46581175|ref|YP_011983.1| hypothetical protein DVU2771 [Desulfovibrio vulgaris subsp. vulgaris str. Hildenborough]
>gi|46581184|ref|YP_011992.1| membrane protein, putative [Desulfovibrio vulgaris subsp. vulgaris str. Hildenborough]
>gi|46581193|ref|YP_012001.1| Gpr1/Fun34/YaaH family protein [Desulfovibrio vulgaris subsp. vulgaris str. Hildenborough]
>gi|46581198|ref|YP_012006.1| electron transport complex protein RnfG, putative [Desulfovibrio vulgaris subsp. vulgaris str. Hildenborough]
>gi|46581199|ref|YP_012007.1| electron transport complex protein RnfE, putative [Desulfovibrio vulgaris subsp. vulgaris str. Hildenborough]
>gi|46581210|ref|YP_012018.1| MotA/TolQ/ExbB proton channel family protein [Desulfovibrio vulgaris subsp. vulgaris str. Hildenborough]
>gi|46581219|ref|YP_012027.1| outer membrane efflux protein [Desulfovibrio vulgaris subsp. vulgaris str. Hildenborough]
>gi|46581227|ref|YP_012035.1| TRAP transporter, DctMQ subunit [Desulfovibrio vulgaris subsp. vulgaris str. Hildenborough]
>gi|46581252|ref|YP_012060.1| tail fiber assembly protein, putative [Desulfovibrio vulgaris subsp. vulgaris str. Hildenborough]
>gi|46581255|ref|YP_012063.1| tail protein, putative [Desulfovibrio vulgaris subsp. vulgaris str. Hildenborough]
>gi|46581259|ref|YP_012067.1| tail/DNA circulation protein, putative [Desulfovibrio vulgaris subsp. vulgaris str. Hildenborough]
>gi|46581272|ref|YP_012080.1| hypothetical protein DVU2868 [Desulfovibrio vulgaris subsp. vulgaris str. Hildenborough]
>gi|46581292|ref|YP_012100.1| cobalt ABC transporter, ATP-binding protein, putative [Desulfovibrio vulgaris subsp. vulgaris str. Hildenborough]
>gi|46581293|ref|YP_012101.1| BioY family protein [Desulfovibrio vulgaris subsp. vulgaris str. Hildenborough]
>gi|46581300|ref|YP_012108.1| TPR domain protein [Desulfovibrio vulgaris subsp. vulgaris str. Hildenborough]
>gi|46581309|ref|YP_012117.1| alcohol dehydrogenase, iron-containing [Desulfovibrio vulgaris subsp. vulgaris str. Hildenborough]
>gi|46581312|ref|YP_012120.1| hypothetical protein DVU2908 [Desulfovibrio vulgaris subsp. vulgaris str. Hildenborough]
>gi|46581319|ref|YP_012127.1| hypothetical protein DVU2915 [Desulfovibrio vulgaris subsp. vulgaris str. Hildenborough]
>gi|46581331|ref|YP_012139.1| ribosomal protein L7/L12 [Desulfovibrio vulgaris subsp. vulgaris str. Hildenborough]
>gi|46581345|ref|YP_012153.1| hypothetical protein DVU2941 [Desulfovibrio vulgaris subsp. vulgaris str. Hildenborough]
>gi|46581349|ref|YP_012157.1| hypothetical protein DVU2945 [Desulfovibrio vulgaris subsp. vulgaris str. Hildenborough]
>gi|46581352|ref|YP_012160.1| bacterial flagellin N-terminal domain protein [Desulfovibrio vulgaris subsp. vulgaris str. Hildenborough]
>gi|46581355|ref|YP_012163.1| membrane protein, putative [Desulfovibrio vulgaris subsp. vulgaris str. Hildenborough]
>gi|46581361|ref|YP_012169.1| membrane protein, putative [Desulfovibrio vulgaris subsp. vulgaris str. Hildenborough]
>gi|46581368|ref|YP_012176.1| hypothetical protein DVU2965 [Desulfovibrio vulgaris subsp. vulgaris str. Hildenborough]
>gi|46581378|ref|YP_012186.1| hydrolase, putative [Desulfovibrio vulgaris subsp. vulgaris str. Hildenborough]
>gi|46581391|ref|YP_012199.1| phage shock protein A [Desulfovibrio vulgaris subsp. vulgaris str. Hildenborough]
>gi|46581454|ref|YP_012262.1| mutator mutT protein [Desulfovibrio vulgaris subsp. vulgaris str. Hildenborough]
>gi|46581457|ref|YP_012265.1| radical SAM domain protein [Desulfovibrio vulgaris subsp. vulgaris str. Hildenborough]
>gi|46581466|ref|YP_012274.1| integral membrane protein MviN [Desulfovibrio vulgaris subsp. vulgaris str. Hildenborough]
>gi|46581472|ref|YP_012280.1| conserved hypothetical protein TIGR00247 [Desulfovibrio vulgaris subsp. vulgaris str. Hildenborough]
>gi|46581475|ref|YP_012283.1| ABC transporter, permease protein, putative [Desulfovibrio vulgaris subsp. vulgaris str. Hildenborough]
>gi|46581477|ref|YP_012285.1| membrane protein, putative [Desulfovibrio vulgaris subsp. vulgaris str. Hildenborough]
>gi|46581482|ref|YP_012290.1| glyoxalase family protein [Desulfovibrio vulgaris subsp. vulgaris str. Hildenborough]
>gi|46581492|ref|YP_012300.1| hypothetical protein DVU3089 [Desulfovibrio vulgaris subsp. vulgaris str. Hildenborough]
>gi|46581494|ref|YP_012302.1| hypothetical protein DVU3091 [Desulfovibrio vulgaris subsp. vulgaris str. Hildenborough]
>gi|46581504|ref|YP_012312.1| tonB protein, putative [Desulfovibrio vulgaris subsp. vulgaris str. Hildenborough]
>gi|46581520|ref|YP_012328.1| hypothetical protein DVU3117 [Desulfovibrio vulgaris subsp. vulgaris str. Hildenborough]
>gi|46581561|ref|YP_012369.1| glycerol-3-phosphate dehydrogenase (NAD(P)+) [Desulfovibrio vulgaris subsp. vulgaris str. Hildenborough]
>gi|46581574|ref|YP_012382.1| hypothetical protein DVU3172 [Desulfovibrio vulgaris subsp. vulgaris str. Hildenborough]
>gi|46581598|ref|YP_012406.1| twin-arginine translocation pathway signal sequence domain protein [Desulfovibrio vulgaris subsp. vulgaris str. Hildenborough]
>gi|46581600|ref|YP_012408.1| DNA polymerase III, gamma and tau subunits, putative [Desulfovibrio vulgaris subsp. vulgaris str. Hildenborough]
>gi|46581610|ref|YP_012418.1| membrane protein, putative [Desulfovibrio vulgaris subsp. vulgaris str. Hildenborough]
>gi|46581627|ref|YP_012435.1| hypothetical protein DVU3225 [Desulfovibrio vulgaris subsp. vulgaris str. Hildenborough]
>gi|46581636|ref|YP_012444.1| flagellar biosynthetic protein FliR [Desulfovibrio vulgaris subsp. vulgaris str. Hildenborough]
>gi|46581641|ref|YP_012449.1| PAP2 family protein [Desulfovibrio vulgaris subsp. vulgaris str. Hildenborough]
>gi|46581653|ref|YP_012461.1| membrane protein, HPP family [Desulfovibrio vulgaris subsp. vulgaris str. Hildenborough]
>gi|46581659|ref|YP_012467.1| DNA internalization-related competence protein ComEC/Rec2 [Desulfovibrio vulgaris subsp. vulgaris str. Hildenborough]
>gi|46581663|ref|YP_012471.1| fumarate reductase, cytochrome b subunit [Desulfovibrio vulgaris subsp. vulgaris str. Hildenborough]
>gi|46581702|ref|YP_012510.1| hypothetical protein DVU3301 [Desulfovibrio vulgaris subsp. vulgaris str. Hildenborough]
>gi|46581709|ref|YP_012517.1| endo/excinuclease amino terminal domain protein [Desulfovibrio vulgaris subsp. vulgaris str. Hildenborough]
>gi|46581726|ref|YP_012534.1| multidrug resistance protein, Smr family [Desulfovibrio vulgaris subsp. vulgaris str. Hildenborough]
>gi|46581730|ref|YP_012538.1| hypothetical protein DVU3330 [Desulfovibrio vulgaris subsp. vulgaris str. Hildenborough]
>gi|46581731|ref|YP_012539.1| hypothetical protein DVU3331 [Desulfovibrio vulgaris subsp. vulgaris str. Hildenborough]
>gi|46581746|ref|YP_012554.1| hypothetical protein DVU3345 [Desulfovibrio vulgaris subsp. vulgaris str. Hildenborough]
>gi|46581775|ref|YP_012583.1| permease, putative [Desulfovibrio vulgaris subsp. vulgaris str. Hildenborough]
>gi|46581778|ref|YP_012586.1| diacylglycerol kinase [Desulfovibrio vulgaris subsp. vulgaris str. Hildenborough]
>gi|46581785|ref|YP_012593.1| zinc resistance-associated protein [Desulfovibrio vulgaris subsp. vulgaris str. Hildenborough]
>gi|46581787|ref|YP_012595.1| permease, putative [Desulfovibrio vulgaris subsp. vulgaris str. Hildenborough]
>gi|46581788|ref|YP_012596.1| hypothetical protein DVU3387 [Desulfovibrio vulgaris subsp. vulgaris str. Hildenborough]
>gi|46581791|ref|YP_012599.1| hypothetical protein DVU3390 [Desulfovibrio vulgaris subsp. vulgaris str. Hildenborough]
>gi|46562134|ref|YP_009167.1| hypothetical protein DVUA0127 [Desulfovibrio vulgaris subsp. vulgaris str. Hildenborough]
>gi|46562136|ref|YP_009165.1| transglycosylase, SLT family [Desulfovibrio vulgaris subsp. vulgaris str. Hildenborough]
>gi|46562140|ref|YP_009161.1| type III secretion system protein, YopQ family [Desulfovibrio vulgaris subsp. vulgaris str. Hildenborough]
>gi|46562142|ref|YP_009157.1| type III secretion lipoprotein [Desulfovibrio vulgaris subsp. vulgaris str. Hildenborough]
>gi|46562146|ref|YP_009153.1| type III secretion protein, YscD family [Desulfovibrio vulgaris subsp. vulgaris str. Hildenborough]

>gi|46562157|ref|YP_009140.1| sigma-54 dependent transcriptional regulator [Desulfovibrio vulgaris subsp. vulgaris str. Hildenborough]
>gi|46562162|ref|YP_009135.1| hypothetical protein DVUA0095 [Desulfovibrio vulgaris subsp. vulgaris str. Hildenborough]
>gi|46562166|ref|YP_009130.1| membrane protein, putative [Desulfovibrio vulgaris subsp. vulgaris str. Hildenborough]
>gi|46562186|ref|YP_009106.1| phospholipase, patatin family [Desulfovibrio vulgaris subsp. vulgaris str. Hildenborough]
>gi|46562188|ref|YP_009104.1| hypothetical protein DVUA0064 [Desulfovibrio vulgaris subsp. vulgaris str. Hildenborough]
>gi|46562190|ref|YP_009102.1| hypothetical protein DVUA0062 [Desulfovibrio vulgaris subsp. vulgaris str. Hildenborough]
>gi|46562192|ref|YP_009100.1| membrane protein, putative [Desulfovibrio vulgaris subsp. vulgaris str. Hildenborough]
>gi|46562194|ref|YP_009098.1| BNR/Asp-box repeat protein [Desulfovibrio vulgaris subsp. vulgaris str. Hildenborough]
>gi|46562197|ref|YP_009095.1| membrane protein, putative [Desulfovibrio vulgaris subsp. vulgaris str. Hildenborough]
>gi|46562198|ref|YP_009094.1| glycosyl transferase, group 1 family protein [Desulfovibrio vulgaris subsp. vulgaris str. Hildenborough]
>gi|46562200|ref|YP_009088.1| exopolysaccharide production protein, putative [Desulfovibrio vulgaris subsp. vulgaris str. Hildenborough]
>gi|46562202|ref|YP_009086.1| glycosyl transferase, group 2 family protein [Desulfovibrio vulgaris subsp. vulgaris str. Hildenborough]
>gi|46562203|ref|YP_009085.1| aminotransferase, DegT/DnrJ/EryC1/StrS family [Desulfovibrio vulgaris subsp. vulgaris str. Hildenborough]
>gi|46562206|ref|YP_009081.1| hypothetical protein DVUA0041 [Desulfovibrio vulgaris subsp. vulgaris str. Hildenborough]
>gi|46562207|ref|YP_009080.1| polysaccharide biosynthesis protein, putative [Desulfovibrio vulgaris subsp. vulgaris str. Hildenborough]
>gi|46562211|ref|YP_009076.1| TPR domain protein [Desulfovibrio vulgaris subsp. vulgaris str. Hildenborough]
>gi|46562218|ref|YP_009069.1| hypothetical protein DVUA0029 [Desulfovibrio vulgaris subsp. vulgaris str. Hildenborough]
>gi|46562238|ref|YP_009048.1| nitrogenase molybdenum-iron cofactor biosynthesis protein NifN, putative [Desulfovibrio vulgaris subsp. vulgaris str. Hildenborough]
>gi|46562242|ref|YP_009043.1| hypothetical protein DVUA0003 [Desulfovibrio vulgaris subsp. vulgaris str. Hildenborough]
>gi|46562247|ref|YP_009189.1| hypothetical protein DVUA0149 [Desulfovibrio vulgaris subsp. vulgaris str. Hildenborough]
>gi|46562248|ref|YP_009188.1| major facilitator superfamily protein [Desulfovibrio vulgaris subsp. vulgaris str. Hildenborough]
>gi|46562254|ref|YP_009180.1| hypothetical protein DVUA0140 [Desulfovibrio vulgaris subsp. vulgaris str. Hildenborough]
>gi|46562261|ref|YP_009158.1| type III secretion protein, YopL family [Desulfovibrio vulgaris subsp. vulgaris str. Hildenborough]
>gi|46562274|ref|YP_009160.1| type III secretion protein, putative [Desulfovibrio vulgaris subsp. vulgaris str. Hildenborough]
>gi|62289013|ref|YP_220806.1| hypothetical protein BruAb1_0024 [Brucella abortus biovar 1 str. 9-941]
>gi|62289015|ref|YP_220808.1| Cmk, cytidylate kinase [Brucella abortus biovar 1 str. 9-941]
>gi|62289057|ref|YP_220850.1| hypothetical protein BruAb1_0071 [Brucella abortus biovar 1 str. 9-941]
>gi|62289070|ref|YP_220863.1| ABC transporter, permease protein [Brucella abortus biovar 1 str. 9-941]
>gi|62289076|ref|YP_220869.1| CcmB, heme exporter protein [Brucella abortus biovar 1 str. 9-941]
>gi|62289090|ref|YP_220883.1| CysW-1, sulfate ABC transporter, permease protein [Brucella abortus biovar 1 str. 9-941]
>gi|62289143|ref|YP_220936.1| hypothetical protein BruAb1_0163 [Brucella abortus biovar 1 str. 9-941]
>gi|62289162|ref|YP_220955.1| hypothetical protein BruAb1_0183 [Brucella abortus biovar 1 str. 9-941]
>gi|62289202|ref|YP_220995.1| SoxG, sarcosine oxidase, gamma subunit [Brucella abortus biovar 1 str. 9-941]
>gi|62289256|ref|YP_221049.1| hypothetical protein BruAb1_0283 [Brucella abortus biovar 1 str. 9-941]
>gi|62289261|ref|YP_221054.1| hypothetical protein BruAb1_0290 [Brucella abortus biovar 1 str. 9-941]
>gi|62289304|ref|YP_221097.1| DNA-damage-inducible protein F, hypothetical [Brucella abortus biovar 1 str. 9-941]
>gi|62289312|ref|YP_221105.1| MscL, large conductance mechanosensitive channel protein [Brucella abortus biovar 1 str. 9-941]
>gi|62289319|ref|YP_221112.1| hypothetical protein BruAb1_0351 [Brucella abortus biovar 1 str. 9-941]
>gi|62289365|ref|YP_221158.1| hypothetical protein BruAb1_0400 [Brucella abortus biovar 1 str. 9-941]
>gi|62289374|ref|YP_221167.1| ATP synthase F0, B subunit, hypothetical [Brucella abortus biovar 1 str. 9-941]
>gi|62289375|ref|YP_221168.1| AtpF, ATP synthase F0, B subunit [Brucella abortus biovar 1 str. 9-941]
>gi|62289380|ref|YP_221173.1| hypothetical protein BruAb1_0415 [Brucella abortus biovar 1 str. 9-941]
>gi|62289386|ref|YP_221179.1| hypothetical protein BruAb1_0422 [Brucella abortus biovar 1 str. 9-941]
>gi|62289405|ref|YP_221198.1| chaperone protein DnaJ, hypothetical [Brucella abortus biovar 1 str. 9-941]
>gi|62289418|ref|YP_221211.1| transglycosylase-associated protein, hypothetical [Brucella abortus biovar 1 str. 9-941]
>gi|62289419|ref|YP_221212.1| hypothetical protein BruAb1_0456 [Brucella abortus biovar 1 str. 9-941]
>gi|62289433|ref|YP_221226.1| colicin V production protein, hypothetical [Brucella abortus biovar 1 str. 9-941]
>gi|62289440|ref|YP_221233.1| RpsF, ribosomal protein S6 [Brucella abortus biovar 1 str. 9-941]
>gi|62289478|ref|YP_221271.1| SMC family protein [Brucella abortus biovar 1 str. 9-941]
>gi|62289490|ref|YP_221283.1| glycosyl transferase, group 4 family protein [Brucella abortus biovar 1 str. 9-941]
>gi|62289535|ref|YP_221328.1| hypothetical protein BruAb1_0589 [Brucella abortus biovar 1 str. 9-941]
>gi|62289554|ref|YP_221347.1| hypothetical protein BruAb1_0608 [Brucella abortus biovar 1 str. 9-941]
>gi|62289580|ref|YP_221373.1| hypothetical membrane protein [Brucella abortus biovar 1 str. 9-941]
>gi|62289594|ref|YP_221387.1| hypothetical protein BruAb1_0651 [Brucella abortus biovar 1 str. 9-941]
>gi|62289611|ref|YP_221404.1| RpoZ, DNA-directed RNA polymerase, omega subunit [Brucella abortus biovar 1 str. 9-941]
>gi|62289666|ref|YP_221459.1| hypothetical protein BruAb1_0723 [Brucella abortus biovar 1 str. 9-941]
>gi|62289675|ref|YP_221468.1| hypothetical protein BruAb1_0733 [Brucella abortus biovar 1 str. 9-941]
>gi|62289693|ref|YP_221486.1| lipoprotein, hypothetical [Brucella abortus biovar 1 str. 9-941]
>gi|62289760|ref|YP_221553.1| NuoJ, NADH dehydrogenase I, J subunit [Brucella abortus biovar 1 str. 9-941]
>gi|62289785|ref|YP_221578.1| hypothetical lipoprotein [Brucella abortus biovar 1 str. 9-941]
>gi|62289790|ref|YP_221583.1| hypothetical protein BruAb1_0856 [Brucella abortus biovar 1 str. 9-941]
>gi|62289802|ref|YP_221595.1| hypothetical protein BruAb1_0868 [Brucella abortus biovar 1 str. 9-941]
>gi|62289812|ref|YP_221605.1| CobS, cobalamin 5-phosphate synthase [Brucella abortus biovar 1 str. 9-941]
>gi|62289823|ref|YP_221616.1| hypothetical protein BruAb1_0890 [Brucella abortus biovar 1 str. 9-941]
>gi|62289828|ref|YP_221621.1| mttA/Hcf106 family protein [Brucella abortus biovar 1 str. 9-941]
>gi|62289833|ref|YP_221626.1| peptidase, M23/M37 family [Brucella abortus biovar 1 str. 9-941]
>gi|62289850|ref|YP_221643.1| AccB, acetyl-CoA carboxylase, biotin carboxyl carrier protein [Brucella abortus biovar 1 str. 9-941]
>gi|62289855|ref|YP_221648.1| ribonuclease, Rne/Rng domain protein [Brucella abortus biovar 1 str. 9-941]
>gi|62289869|ref|YP_221662.1| QacH, quaternary ammonium compound-resistance protein [Brucella abortus biovar 1 str. 9-941]

>gi|62289882|ref|YP_221675.1| heavy-metal-associated domain protein [Brucella abortus biovar 1 str. 9-941]
>gi|62289893|ref|YP_221686.1| hypothetical protein BruAb1_0970 [Brucella abortus biovar 1 str. 9-941]
>gi|62289937|ref|YP_221730.1| DgkA, diacylglycerol kinase [Brucella abortus biovar 1 str. 9-941]
>gi|62289960|ref|YP_221753.1| hypothetical protein BruAb1_1043 [Brucella abortus biovar 1 str. 9-941]
>gi|62290017|ref|YP_221810.1| single-stranded DNA-binding protein family [Brucella abortus biovar 1 str. 9-941]
>gi|62290038|ref|YP_221831.1| hypothetical protein BruAb1_1131 [Brucella abortus biovar 1 str. 9-941]
>gi|62290040|ref|YP_221833.1| AceF, pyruvate dehydrogenase complex, E2 component, dihydrolipoamide acetyltransferase [Brucella abortus biovar 1 str. 9-941]
>gi|62290050|ref|YP_221843.1| preprotein translocase, SecG subunit, hypothetical [Brucella abortus biovar 1 str. 9-941]
>gi|62290074|ref|YP_221867.1| Tsf, translation elongation factor Ts [Brucella abortus biovar 1 str. 9-941]
>gi|62290115|ref|YP_221908.1| OmpA family protein [Brucella abortus biovar 1 str. 9-941]
>gi|62290128|ref|YP_221921.1| RplR, ribosomal protein L18 [Brucella abortus biovar 1 str. 9-941]
>gi|62290143|ref|YP_221936.1| RplD, ribosomal protein L4 [Brucella abortus biovar 1 str. 9-941]
>gi|62290155|ref|YP_221948.1| RplL, ribosomal protein L7/L12 [Brucella abortus biovar 1 str. 9-941]
>gi|62290169|ref|YP_221962.1| ferripyochelin-binding protein, hypothetical [Brucella abortus biovar 1 str. 9-941]
>gi|62290207|ref|YP_222000.1| hypothetical protein BruAb1_1304 [Brucella abortus biovar 1 str. 9-941]
>gi|62290213|ref|YP_222006.1| hypothetical protein BruAb1_1310 [Brucella abortus biovar 1 str. 9-941]
>gi|62290234|ref|YP_222027.1| dedA family protein [Brucella abortus biovar 1 str. 9-941]
>gi|62290235|ref|YP_222028.1| hypothetical acid-shock protein [Brucella abortus biovar 1 str. 9-941]
>gi|62290243|ref|YP_222036.1| iron compound ABC transporter, permease protein [Brucella abortus biovar 1 str. 9-941]
>gi|62290248|ref|YP_222041.1| HlyD family secretion protein [Brucella abortus biovar 1 str. 9-941]
>gi|62290259|ref|YP_222052.1| hypothetical urea transporter [Brucella abortus biovar 1 str. 9-941]
>gi|62290261|ref|YP_222054.1| CbiM, cobalamin biosynthetesis protein [Brucella abortus biovar 1 str. 9-941]
>gi|62290308|ref|YP_222101.1| antibiotic acetyltransferase [Brucella abortus biovar 1 str. 9-941]
>gi|62290318|ref|YP_222111.1| FtsZ, cell division protein FtsZ [Brucella abortus biovar 1 str. 9-941]
>gi|62290339|ref|YP_222132.1| hypothetical protein BruAb1_1441 [Brucella abortus biovar 1 str. 9-941]
>gi|62290342|ref|YP_222135.1| hypothetical transporter [Brucella abortus biovar 1 str. 9-941]
>gi|62290363|ref|YP_222156.1| hypothetical protein BruAb1_1467 [Brucella abortus biovar 1 str. 9-941]
>gi|62290367|ref|YP_222160.1| hypothetical protein BruAb1_1471 [Brucella abortus biovar 1 str. 9-941]
>gi|62290403|ref|YP_222196.1| hypothetical protein BruAb1_1507 [Brucella abortus biovar 1 str. 9-941]
>gi|62290405|ref|YP_222198.1| hypothetical protein BruAb1_1509 [Brucella abortus biovar 1 str. 9-941]
>gi|62290497|ref|YP_222290.1| hypothetical protein BruAb1_1607 [Brucella abortus biovar 1 str. 9-941]
>gi|62290531|ref|YP_222324.1| hypothetical protein BruAb1_1643 [Brucella abortus biovar 1 str. 9-941]
>gi|62290535|ref|YP_222328.1| hypothetical protein BruAb1_1647 [Brucella abortus biovar 1 str. 9-941]
>gi|62290541|ref|YP_222334.1| ExbB, biopolymer transport protein ExbB [Brucella abortus biovar 1 str. 9-941]
>gi|62290543|ref|YP_222336.1| TonB-dependent receptor [Brucella abortus biovar 1 str. 9-941]
>gi|62290560|ref|YP_222353.1| sodium/bile acid transporter family protein [Brucella abortus biovar 1 str. 9-941]
>gi|62290570|ref|YP_222363.1| TolA, tolA protein [Brucella abortus biovar 1 str. 9-941]
>gi|62290586|ref|YP_222379.1| OmpA family protein [Brucella abortus biovar 1 str. 9-941]
>gi|62290615|ref|YP_222408.1| PurE, phosphoribosylaminoimidazole carboxylase, catalytic subunit [Brucella abortus biovar 1 str. 9-941]
>gi|62290618|ref|YP_222411.1| TPR domain protein [Brucella abortus biovar 1 str. 9-941]
>gi|62290626|ref|YP_222419.1| lipoprotein, hypothetical [Brucella abortus biovar 1 str. 9-941]
>gi|62290640|ref|YP_222433.1| ATP-dependent helicase [Brucella abortus biovar 1 str. 9-941]
>gi|62290661|ref|YP_222454.1| transcriptional regulator, MarR family [Brucella abortus biovar 1 str. 9-941]
>gi|62290689|ref|YP_222482.1| Ffh, signal recognition particle protein [Brucella abortus biovar 1 str. 9-941]
>gi|62290696|ref|YP_222489.1| hypothetical membrane protein [Brucella abortus biovar 1 str. 9-941]
>gi|62290700|ref|YP_222493.1| hypothetical protein BruAb1_1817 [Brucella abortus biovar 1 str. 9-941]
>gi|62290712|ref|YP_222505.1| RplU, ribosomal protein L21 [Brucella abortus biovar 1 str. 9-941]
>gi|62290715|ref|YP_222508.1| AzlC family protein [Brucella abortus biovar 1 str. 9-941]
>gi|62290726|ref|YP_222519.1| hypothetical protein BruAb1_1844 [Brucella abortus biovar 1 str. 9-941]
>gi|62290739|ref|YP_222532.1| hypothetical protein BruAb1_1858 [Brucella abortus biovar 1 str. 9-941]
>gi|62290753|ref|YP_222546.1| Amt, ammonium transporter [Brucella abortus biovar 1 str. 9-941]
>gi|62290776|ref|YP_222569.1| SucB, 2-oxoglutarate dehydrogenase, E2 dihydrolipoamide succinyltransferase [Brucella abortus biovar 1 str. 9-941]
>gi|62290788|ref|YP_222581.1| FtsY, signal recognition particle-docking protein [Brucella abortus biovar 1 str. 9-941]
>gi|62290816|ref|YP_222609.1| universal stress protein family [Brucella abortus biovar 1 str. 9-941]
>gi|62290818|ref|YP_222611.1| EtfA, electron transfer flavoprotein, alpha subunit [Brucella abortus biovar 1 str. 9-941]
>gi|62290862|ref|YP_222655.1| transcriptional regulator, MerR family [Brucella abortus biovar 1 str. 9-941]
>gi|62290875|ref|YP_222668.1| hypothetical protein BruAb1_2005 [Brucella abortus biovar 1 str. 9-941]
>gi|62290953|ref|YP_222746.1| TrpF, N-(5phosphoribosyl)anthranilate isomerase [Brucella abortus biovar 1 str. 9-941]
>gi|62290959|ref|YP_222752.1| RpmI, ribosomal protein L35 [Brucella abortus biovar 1 str. 9-941]
>gi|62290984|ref|YP_222777.1| lipoprotein, hypothetical [Brucella abortus biovar 1 str. 9-941]
>gi|62291001|ref|YP_222794.1| hypothetical protein BruAb1_2137 [Brucella abortus biovar 1 str. 9-941]
>gi|62291002|ref|YP_222795.1| InfB, translation initiation factor IF-2 [Brucella abortus biovar 1 str. 9-941]
>gi|62316968|ref|YP_222821.1| ABC transporter, permease protein [Brucella abortus biovar 1 str. 9-941]
>gi|62316980|ref|YP_222833.1| hypothetical protein BruAb2_0020 [Brucella abortus biovar 1 str. 9-941]
>gi|62316982|ref|YP_222835.1| hypothetical protein BruAb2_0022 [Brucella abortus biovar 1 str. 9-941]
>gi|62316993|ref|YP_222846.1| hypothetical acetoin dehydrogenase complex, E2 component, dihydrolipoamide acetyltransferase [Brucella abortus biovar 1 str. 9-941]
>gi|62317016|ref|YP_222869.1| hypothetical protein BruAb2_0057 [Brucella abortus biovar 1 str. 9-941]
>gi|62317019|ref|YP_222872.1| type IV secretion system protein VirB10 [Brucella abortus biovar 1 str. 9-941]

>gi|62317020|ref|YP_222873.1| type IV secretion system protein VirB9 [Brucella abortus biovar 1 str. 9-941]
>gi|62317023|ref|YP_222876.1| type IV secretion system protein VirB6 [Brucella abortus biovar 1 str. 9-941]
>gi|62317034|ref|YP_222887.1| SPFH domain/Band 7 family protein [Brucella abortus biovar 1 str. 9-941]
>gi|62317042|ref|YP_222895.1| hypothetical protein BruAb2_0083 [Brucella abortus biovar 1 str. 9-941]
>gi|62317053|ref|YP_222906.1| hypothetical protein BruAb2_0094 [Brucella abortus biovar 1 str. 9-941]
>gi|62317105|ref|YP_222958.1| hypothetical protein BruAb2_0153 [Brucella abortus biovar 1 str. 9-941]
>gi|62317119|ref|YP_222972.1| hypothetical protein BruAb2_0167 [Brucella abortus biovar 1 str. 9-941]
>gi|62317120|ref|YP_222973.1| outermembrane transporter [Brucella abortus biovar 1 str. 9-941]
>gi|62317153|ref|YP_223006.1| RNA pseudouridylate synthase family protein [Brucella abortus biovar 1 str. 9-941]
>gi|62317176|ref|YP_223029.1| hypothetical protein BruAb2_0227 [Brucella abortus biovar 1 str. 9-941]
>gi|62317186|ref|YP_223039.1| RbsC-4, ribose ABC transporter, permease protein [Brucella abortus biovar 1 str. 9-941]
>gi|62317192|ref|YP_223045.1| cobalamin synthesis protein/P47K family protein [Brucella abortus biovar 1 str. 9-941]
>gi|62317203|ref|YP_223056.1| PntA, NAD(P) transhydrogenase, alpha subunit [Brucella abortus biovar 1 str. 9-941]
>gi|62317204|ref|YP_223057.1| PntAB, NAD(P) transhydrogenase, alpha2 subunit [Brucella abortus biovar 1 str. 9-941]
>gi|62317205|ref|YP_223058.1| PntB, NAD(P) transhydrogenase, beta subunit [Brucella abortus biovar 1 str. 9-941]
>gi|62317209|ref|YP_223062.1| ribosomal protein S21 [Brucella abortus biovar 1 str. 9-941]
>gi|62317244|ref|YP_223097.1| HutH, histidine ammonia-lyase [Brucella abortus biovar 1 str. 9-941]
>gi|62317351|ref|YP_223204.1| CrcB family protein [Brucella abortus biovar 1 str. 9-941]
>gi|62317358|ref|YP_223211.1| ABC transporter, membrane spanning protein [Brucella abortus biovar 1 str. 9-941]
>gi|62317375|ref|YP_223228.1| hypothetical protein BruAb2_0443 [Brucella abortus biovar 1 str. 9-941]
>gi|62317424|ref|YP_223277.1| immunoreactive 14 kDa protein BA14k [Brucella abortus biovar 1 str. 9-941]
>gi|62317451|ref|YP_223304.1| major facilitator family transporter [Brucella abortus biovar 1 str. 9-941]
>gi|62317488|ref|YP_223341.1| hypothetical protein BruAb2_0563 [Brucella abortus biovar 1 str. 9-941]
>gi|62317519|ref|YP_223372.1| hypothetical protein BruAb2_0599 [Brucella abortus biovar 1 str. 9-941]
>gi|62317542|ref|YP_223395.1| hypothetical membrane protein [Brucella abortus biovar 1 str. 9-941]
>gi|62317556|ref|YP_223409.1| hypothetical protein BruAb2_0637 [Brucella abortus biovar 1 str. 9-941]
>gi|62317574|ref|YP_223427.1| ABC transporter, permease protein [Brucella abortus biovar 1 str. 9-941]
>gi|62317627|ref|YP_223480.1| ABC transporter, ATP-binding/permease protein [Brucella abortus biovar 1 str. 9-941]
>gi|62317632|ref|YP_223485.1| monovalent cation/proton antiporter, MnhF/PhaF subunit [Brucella abortus biovar 1 str. 9-941]
>gi|62317668|ref|YP_223521.1| HlyD family secretion protein [Brucella abortus biovar 1 str. 9-941]
>gi|62317674|ref|YP_223527.1| hypothetical protein BruAb2_0765 [Brucella abortus biovar 1 str. 9-941]
>gi|62317739|ref|YP_223592.1| hypothetical protein BruAb2_0838 [Brucella abortus biovar 1 str. 9-941]
>gi|62317755|ref|YP_223608.1| hypothetical protein BruAb2_0856 [Brucella abortus biovar 1 str. 9-941]
>gi|62317797|ref|YP_223650.1| NosY, nitrous-oxide reductase, nosY component [Brucella abortus biovar 1 str. 9-941]
>gi|62317808|ref|YP_223661.1| sugar ABC transporter permease [Brucella abortus biovar 1 str. 9-941]
>gi|62317820|ref|YP_223673.1| hypothetical protein BruAb2_0926 [Brucella abortus biovar 1 str. 9-941]
>gi|62317843|ref|YP_223696.1| oxidoreductase, short-chain dehydrogenase/reductase family [Brucella abortus biovar 1 str. 9-941]
>gi|62317864|ref|YP_223717.1| hypothetical protein BruAb2_0976 [Brucella abortus biovar 1 str. 9-941]
>gi|62317884|ref|YP_223737.1| hypothetical protein BruAb2_0997 [Brucella abortus biovar 1 str. 9-941]
>gi|62317912|ref|YP_223765.1| enoyl-CoA hydratase/isomerase family protein [Brucella abortus biovar 1 str. 9-941]
>gi|62317928|ref|YP_223781.1| polyamine ABC transporter, permease protein [Brucella abortus biovar 1 str. 9-941]
>gi|62317943|ref|YP_223796.1| ZnuB, zinc ABC transporter, permease protein [Brucella abortus biovar 1 str. 9-941]
>gi|62317951|ref|YP_223804.1| FliQ, flagellar biosynthesis protein [Brucella abortus biovar 1 str. 9-941]
>gi|62317960|ref|YP_223813.1| MotD-related protein [Brucella abortus biovar 1 str. 9-941]
>gi|62317966|ref|YP_223819.1| outer membrane autotransporter [Brucella abortus biovar 1 str. 9-941]
>gi|62317970|ref|YP_223823.1| XylH, D-xylose ABC transporter, permease protein [Brucella abortus biovar 1 str. 9-941]
>gi|62317986|ref|YP_223839.1| amino acid permease family protein [Brucella abortus biovar 1 str. 9-941]
>gi|62317990|ref|YP_223843.1| branched-chain amino acid ABC transporter, permease protein [Brucella abortus biovar 1 str. 9-941]
>gi|33151307|ref|NP_872660.1| fumarate reductase, 13 kD hydrophobic protein [Haemophilus ducreyi 35000HP]
>gi|33151368|ref|NP_872721.1| hypothetical protein HD0107 [Haemophilus ducreyi 35000HP]
>gi|33151443|ref|NP_872796.1| hypothetical protein HD0192 [Haemophilus ducreyi 35000HP]
>gi|33151449|ref|NP_872802.1| periplasmic zinc transporter [Haemophilus ducreyi 35000HP]
>gi|33151505|ref|NP_872858.1| 15kd outer membrane lipoprotein [Haemophilus ducreyi 35000HP]
>gi|33151549|ref|NP_872902.1| dipeptide transport system permease protein [Haemophilus ducreyi 35000HP]
>gi|33151562|ref|NP_872915.1| TobB energy transducing protein [Haemophilus ducreyi 35000HP]
>gi|33151621|ref|NP_872974.1| probable Na+-translocating NADH-quinone oxidoreductase [Haemophilus ducreyi 35000HP]
>gi|33151623|ref|NP_872976.1| putative iron-sulfur binding NADH dehydrogenase [Haemophilus ducreyi 35000HP]
>gi|33151626|ref|NP_872979.1| probable Na-translocating NADH-quinone reductase [Haemophilus ducreyi 35000HP]
>gi|33151655|ref|NP_873008.1| hypothetical protein HD0432 [Haemophilus ducreyi 35000HP]
>gi|33151678|ref|NP_873031.1| conserved probable membrane protein [Haemophilus ducreyi 35000HP]
>gi|33151817|ref|NP_873170.1| probable biotin carboxyl carrier protein of acetyl-CoA carboxylase (BCCP) [Haemophilus ducreyi 35000HP]
>gi|33151848|ref|NP_873201.1| hypothetical protein HD0665 [Haemophilus ducreyi 35000HP]
>gi|33151866|ref|NP_873219.1| 30S ribosomal protein s21 [Haemophilus ducreyi 35000HP]
>gi|33151942|ref|NP_873295.1| alkylphosphonate uptake protein [Haemophilus ducreyi 35000HP]
>gi|33151948|ref|NP_873301.1| cytochrome c-type biogenesis protein; heme exporter protein B [Haemophilus ducreyi 35000HP]
>gi|33152146|ref|NP_873499.1| possible ProP effector [Haemophilus ducreyi 35000HP]
>gi|33152180|ref|NP_873533.1| 50S ribosomal protein L9 [Haemophilus ducreyi 35000HP]
>gi|33152219|ref|NP_873572.1| hypothetical protein HD1095 [Haemophilus ducreyi 35000HP]
>gi|33152247|ref|NP_873600.1| hypothetical protein HD1126 [Haemophilus ducreyi 35000HP]
>gi|33152359|ref|NP_873712.1| preprotein translocase SecG subunit [Haemophilus ducreyi 35000HP]

>gi|33152369|ref|NP_873722.1| hypothetical protein HD1289 [Haemophilus ducreyi 35000HP]
>gi|33152376|ref|NP_873729.1| high-affinity zinc uptake system membrane protein [Haemophilus ducreyi 35000HP]
>gi|33152474|ref|NP_873827.1| hypothetical protein HD1409 [Haemophilus ducreyi 35000HP]
>gi|33152481|ref|NP_873834.1| hypothetical protein HD1417 [Haemophilus ducreyi 35000HP]
>gi|33152503|ref|NP_873856.1| hypothetical protein HD1441 [Haemophilus ducreyi 35000HP]
>gi|33152507|ref|NP_873860.1| RstR-like phage repressor protein [Haemophilus ducreyi 35000HP]
>gi|33152511|ref|NP_873864.1| hypothetical protein HD1449 [Haemophilus ducreyi 35000HP]
>gi|33152515|ref|NP_873868.1| RstR-like phage repressor protein [Haemophilus ducreyi 35000HP]
>gi|33152601|ref|NP_873954.1| hypothetical protein HD1559 [Haemophilus ducreyi 35000HP]
>gi|33152809|ref|NP_874162.1| 50S ribosomal protein L35 [Haemophilus ducreyi 35000HP]
>gi|33152841|ref|NP_874194.1| putative polysaccharide biosynthesis protein [Haemophilus ducreyi 35000HP]
>gi|33152868|ref|NP_874221.1| 50S ribosomal protein L7/L12 [Haemophilus ducreyi 35000HP]
>gi|33152935|ref|NP_874288.1| 50S ribosomal protein L15 [Haemophilus ducreyi 35000HP]
>gi|33152978|ref|NP_874331.1| hypothetical protein HD2011 [Haemophilus ducreyi 35000HP]
>gi|29374669|ref|NP_813821.1| single-strand binding protein [Enterococcus faecalis V583]
>gi|29374680|ref|NP_813832.1| PTS system, mannose-specific IIC component [Enterococcus faecalis V583]
>gi|29374684|ref|NP_813836.1| hypothetical protein EF0026 [Enterococcus faecalis V583]
>gi|29374731|ref|NP_813883.1| hypothetical protein EF0078 [Enterococcus faecalis V583]
>gi|29374734|ref|NP_813886.1| membrane protein, putative [Enterococcus faecalis V583]
>gi|29374744|ref|NP_813896.1| cell wall surface anchor family protein [Enterococcus faecalis V583]
>gi|29374750|ref|NP_813902.1| L-serine dehydratase, iron-sulfur-dependent, alpha subunit [Enterococcus faecalis V583]
>gi|29374773|ref|NP_813925.1| hypothetical protein EF0122 [Enterococcus faecalis V583]
>gi|29374797|ref|NP_813949.1| surface exclusion protein, putative [Enterococcus faecalis V583]
>gi|29374803|ref|NP_813955.1| cell wall surface anchor family protein [Enterococcus faecalis V583]
>gi|29374852|ref|NP_814005.1| ribosomal protein L4 [Enterococcus faecalis V583]
>gi|29374862|ref|NP_814015.1| ribosomal protein L24 [Enterococcus faecalis V583]
>gi|29374894|ref|NP_814047.1| N-acetylmuramoyl-L-alanine amidase, family 4 [Enterococcus faecalis V583]
>gi|29374938|ref|NP_814091.1| copper transport protein CopZ [Enterococcus faecalis V583]
>gi|29374952|ref|NP_814105.1| hypothetical protein EF0313 [Enterococcus faecalis V583]
>gi|29374957|ref|NP_814110.1| hypothetical protein EF0318 [Enterococcus faecalis V583]
>gi|29374977|ref|NP_814130.1| scaffold protein [Enterococcus faecalis V583]
>gi|29374985|ref|NP_814138.1| hypothetical protein EF0346 [Enterococcus faecalis V583]
>gi|29375029|ref|NP_814182.1| hypothetical protein EF0393 [Enterococcus faecalis V583]
>gi|29375030|ref|NP_814183.1| secreted antigen, putative [Enterococcus faecalis V583]
>gi|29375036|ref|NP_814189.1| hypothetical protein EF0400 [Enterococcus faecalis V583]
>gi|29375074|ref|NP_814227.1| hypothetical protein EF0442 [Enterococcus faecalis V583]
>gi|29375075|ref|NP_814228.1| LysM domain protein [Enterococcus faecalis V583]
>gi|29375118|ref|NP_814271.1| cell wall surface anchor family protein [Enterococcus faecalis V583]
>gi|29375124|ref|NP_814277.1| hypothetical protein EF0496 [Enterococcus faecalis V583]
>gi|29375125|ref|NP_814278.1| hypothetical protein EF0497 [Enterococcus faecalis V583]
>gi|29375169|ref|NP_814322.1| PTS system, IIC component [Enterococcus faecalis V583]
>gi|29375177|ref|NP_814330.1| hypothetical protein EF0563 [Enterococcus faecalis V583]
>gi|29375205|ref|NP_814358.1| hypothetical protein EF0599 [Enterococcus faecalis V583]
>gi|29375238|ref|NP_814391.1| hypothetical protein EF0637 [Enterococcus faecalis V583]
>gi|29375313|ref|NP_814467.1| PTS system, fructose-specific family, IIABC components [Enterococcus faecalis V583]
>gi|29375347|ref|NP_814501.1| hypothetical protein EF0754 [Enterococcus faecalis V583]
>gi|29375369|ref|NP_814523.1| hypothetical protein EF0778 [Enterococcus faecalis V583]
>gi|29375391|ref|NP_814545.1| hypothetical protein EF0802 [Enterococcus faecalis V583]
>gi|29375398|ref|NP_814552.1| membrane protein, putative [Enterococcus faecalis V583]
>gi|29375421|ref|NP_814575.1| hypothetical protein EF0833 [Enterococcus faecalis V583]
>gi|29375435|ref|NP_814589.1| hypothetical protein EF0847 [Enterococcus faecalis V583]
>gi|29375440|ref|NP_814594.1| hypothetical protein EF0852 [Enterococcus faecalis V583]
>gi|29375481|ref|NP_814635.1| preprotein translocase, YajC subunit, putative [Enterococcus faecalis V583]
>gi|29375516|ref|NP_814670.1| hypothetical protein EF0932 [Enterococcus faecalis V583]
>gi|29375528|ref|NP_814682.1| extracellular protein, putative [Enterococcus faecalis V583]
>gi|29375546|ref|NP_814700.1| hypothetical protein EF0963 [Enterococcus faecalis V583]
>gi|29375548|ref|NP_814702.1| hypothetical protein EF0965 [Enterococcus faecalis V583]
>gi|29375551|ref|NP_814705.1| ribosomal protein L21 [Enterococcus faecalis V583]
>gi|29375554|ref|NP_814708.1| hypothetical protein EF0971 [Enterococcus faecalis V583]
>gi|29375595|ref|NP_814749.1| hypothetical protein EF1015 [Enterococcus faecalis V583]
>gi|29375612|ref|NP_814766.1| 6-aminohexanoate-cyclic-dimer hydrolase, putative [Enterococcus faecalis V583]
>gi|29375614|ref|NP_814768.1| lipoprotein, putative [Enterococcus faecalis V583]
>gi|29375662|ref|NP_814816.1| hypothetical protein EF1085 [Enterococcus faecalis V583]
>gi|29375671|ref|NP_814825.1| hypothetical protein EF1095 [Enterococcus faecalis V583]
>gi|29375675|ref|NP_814829.1| collagen adhesin protein [Enterococcus faecalis V583]
>gi|29375681|ref|NP_814835.1| hypothetical protein EF1105 [Enterococcus faecalis V583]
>gi|29375709|ref|NP_814863.1| 2,3,4,5-tetrahydropyridine-2-carboxylate N-succinyltransferase [Enterococcus faecalis V583]
>gi|29375722|ref|NP_814876.1| DNA-directed RNA polymerase, delta subunit, putative [Enterococcus faecalis V583]
>gi|29375755|ref|NP_814909.1| hypothetical protein EF1180 [Enterococcus faecalis V583]
>gi|29375808|ref|NP_814962.1| hypothetical protein EF1235 [Enterococcus faecalis V583]

>gi|29375825|ref|NP_814979.1| ABC transporter, permease protein [Enterococcus faecalis V583]
>gi|29375827|ref|NP_814981.1| hypothetical protein EF1258 [Enterococcus faecalis V583]
>gi|29375843|ref|NP_814997.1| translation initiation factor IF-2 [Enterococcus faecalis V583]
>gi|29375857|ref|NP_815011.1| hypothetical protein EF1288 [Enterococcus faecalis V583]
>gi|29375868|ref|NP_815022.1| cell division protein, FtsW/RodA/SpovE family [Enterococcus faecalis V583]
>gi|29375878|ref|NP_815032.1| dnaJ protein [Enterococcus faecalis V583]
>gi|29375922|ref|NP_815076.1| pyruvate dehydrogenase complex E2 component, dihydrolipoamide acetyltransferase [Enterococcus faecalis V583]
>gi|29375933|ref|NP_815087.1| hypothetical protein EF1366 [Enterococcus faecalis V583]
>gi|29375942|ref|NP_815096.1| hypothetical protein EF1375 [Enterococcus faecalis V583]
>gi|29375970|ref|NP_815124.1| hypothetical protein EF1403 [Enterococcus faecalis V583]
>gi|29376018|ref|NP_815172.1| hypothetical protein EF1451 [Enterococcus faecalis V583]
>gi|29376025|ref|NP_815179.1| hypothetical protein EF1458 [Enterococcus faecalis V583]
>gi|29376049|ref|NP_815203.1| hypothetical protein EF1482 [Enterococcus faecalis V583]
>gi|29376062|ref|NP_815216.1| V-type ATPase, subunit E [Enterococcus faecalis V583]
>gi|29376073|ref|NP_815227.1| hypothetical protein EF1506 [Enterococcus faecalis V583]
>gi|29376088|ref|NP_815242.1| conserved domainl protein [Enterococcus faecalis V583]
>gi|29376109|ref|NP_815263.1| LysM domain protein [Enterococcus faecalis V583]
>gi|29376156|ref|NP_815310.1| MutT/nudix family protein [Enterococcus faecalis V583]
>gi|29376177|ref|NP_815331.1| ethanolamine utilization protein EutH [Enterococcus faecalis V583]
>gi|29376218|ref|NP_815372.1| hypothetical protein EF1664 [Enterococcus faecalis V583]
>gi|29376231|ref|NP_815385.1| lipoprotein, putative [Enterococcus faecalis V583]
>gi|29376293|ref|NP_815447.1| general stress protein, putative [Enterococcus faecalis V583]
>gi|29376294|ref|NP_815448.1| hypothetical protein EF1745 [Enterococcus faecalis V583]
>gi|29376341|ref|NP_815495.1| lipoprotein, putative [Enterococcus faecalis V583]
>gi|29376368|ref|NP_815522.1| hypothetical protein EF1825 [Enterococcus faecalis V583]
>gi|29376374|ref|NP_815528.1| hypothetical protein EF1833 [Enterococcus faecalis V583]
>gi|29376443|ref|NP_815597.1| hypothetical protein EF1915 [Enterococcus faecalis V583]
>gi|29376454|ref|NP_815608.1| glycerol uptake facilitator protein [Enterococcus faecalis V583]
>gi|29376503|ref|NP_815657.1| hypothetical protein EF1981 [Enterococcus faecalis V583]
>gi|29376527|ref|NP_815681.1| hypothetical protein EF2012 [Enterococcus faecalis V583]
>gi|29376541|ref|NP_815695.1| hypothetical protein EF2027 [Enterococcus faecalis V583]
>gi|29376547|ref|NP_815701.1| hypothetical protein EF2036 [Enterococcus faecalis V583]
>gi|29376599|ref|NP_815753.1| hypothetical protein EF2090 [Enterococcus faecalis V583]
>gi|29376614|ref|NP_815768.1| hypothetical protein EF2105 [Enterococcus faecalis V583]
>gi|29376616|ref|NP_815770.1| hypothetical protein EF2107 [Enterococcus faecalis V583]
>gi|29376648|ref|NP_815802.1| hypothetical protein EF2139 [Enterococcus faecalis V583]
>gi|29376692|ref|NP_815846.1| hypothetical protein EF2184 [Enterococcus faecalis V583]
>gi|29376697|ref|NP_815851.1| hypothetical protein EF2189 [Enterococcus faecalis V583]
>gi|29376710|ref|NP_815864.1| tspO protein, putative [Enterococcus faecalis V583]
>gi|29376753|ref|NP_815907.1| hypothetical protein EF2248 [Enterococcus faecalis V583]
>gi|29376789|ref|NP_815943.1| ParB-like nuclease domain protein [Enterococcus faecalis V583]
>gi|29376790|ref|NP_815944.1| hypothetical protein EF2287 [Enterococcus faecalis V583]
>gi|29376816|ref|NP_815970.1| bacteriocin, putative [Enterococcus faecalis V583]
>gi|29376866|ref|NP_816020.1| hypothetical protein EF2368 [Enterococcus faecalis V583]
>gi|29376899|ref|NP_816053.1| hypothetical protein EF2404 [Enterococcus faecalis V583]
>gi|29376911|ref|NP_816065.1| ribosomal protein S21 [Enterococcus faecalis V583]
>gi|29376973|ref|NP_816127.1| hypothetical protein EF2480 [Enterococcus faecalis V583]
>gi|29376989|ref|NP_816143.1| ABC transporter, permease protein [Enterococcus faecalis V583]
>gi|29377005|ref|NP_816159.1| membrane protein, putative [Enterococcus faecalis V583]
>gi|29377025|ref|NP_816179.1| hypothetical protein EF2536 [Enterococcus faecalis V583]
>gi|29377027|ref|NP_816181.1| hypothetical protein EF2538 [Enterococcus faecalis V583]
>gi|29377049|ref|NP_816203.1| flavodoxin [Enterococcus faecalis V583]
>gi|29377071|ref|NP_816225.1| hypothetical protein EF2584 [Enterococcus faecalis V583]
>gi|29377075|ref|NP_816229.1| hypothetical protein EF2588 [Enterococcus faecalis V583]
>gi|29377114|ref|NP_816268.1| hypothetical protein EF2629 [Enterococcus faecalis V583]
>gi|29377119|ref|NP_816273.1| chaperonin, 10 kDa [Enterococcus faecalis V583]
>gi|29377121|ref|NP_816275.1| abortive infection protein [Enterococcus faecalis V583]
>gi|29377169|ref|NP_816323.1| exonuclease SbcC [Enterococcus faecalis V583]
>gi|29377175|ref|NP_816329.1| hypothetical protein EF2695 [Enterococcus faecalis V583]
>gi|29377188|ref|NP_816342.1| membran protein, putative [Enterococcus faecalis V583]
>gi|29377193|ref|NP_816347.1| cell wall surface anchor family protein [Enterococcus faecalis V583]
>gi|29377194|ref|NP_816348.1| ribosomal protein L7/L12 [Enterococcus faecalis V583]
>gi|29377200|ref|NP_816354.1| L-serine dehydratase, iron-sulfur-dependent, alpha subunit [Enterococcus faecalis V583]
>gi|29377265|ref|NP_816419.1| hypothetical protein EF2793 [Enterococcus faecalis V583]
>gi|29377269|ref|NP_816423.1| hypothetical protein EF2797 [Enterococcus faecalis V583]
>gi|29377322|ref|NP_816476.1| ion transporter, putative [Enterococcus faecalis V583]
>gi|29377345|ref|NP_816499.1| acetyl-CoA carboxylase, biotin carboxyl carrier protein [Enterococcus faecalis V583]
>gi|29377382|ref|NP_816536.1| bacterial transferase, putative [Enterococcus faecalis V583]
>gi|29377405|ref|NP_816559.1| hypothetical protein EF2941 [Enterococcus faecalis V583]
>gi|29377410|ref|NP_816564.1| hypothetical protein EF2946 [Enterococcus faecalis V583]

>gi|29377464|ref|NP_816618.1| lipoprotein, putative [Enterococcus faecalis V583]
>gi|29377512|ref|NP_816666.1| lipoprotein, putative [Enterococcus faecalis V583]
>gi|29377518|ref|NP_816672.1| secreted lipase, putative [Enterococcus faecalis V583]
>gi|29377541|ref|NP_816695.1| iron compound ABC transporter, permease protein [Enterococcus faecalis V583]
>gi|29377551|ref|NP_816705.1| signal recognition particle-docking protein FtsY [Enterococcus faecalis V583]
>gi|29377558|ref|NP_816712.1| hypothetical protein EF3102 [Enterococcus faecalis V583]
>gi|29377559|ref|NP_816713.1| membrane protein, putative [Enterococcus faecalis V583]
>gi|29377581|ref|NP_816735.1| DNA-directed RNA polymerase, omega subunit [Enterococcus faecalis V583]
>gi|29377612|ref|NP_816766.1| hypothetical protein EF3161 [Enterococcus faecalis V583]
>gi|29377623|ref|NP_816777.1| hypothetical protein EF3173 [Enterococcus faecalis V583]
>gi|29377632|ref|NP_816786.1| hypothetical protein EF3182 [Enterococcus faecalis V583]
>gi|29377634|ref|NP_816788.1| hypothetical protein EF3184 [Enterococcus faecalis V583]
>gi|29377755|ref|NP_816909.1| cell wall surface anchor family protein [Enterococcus faecalis V583]
>gi|29377766|ref|NP_816920.1| sodium ion-translocating decarboxylase, biotin carboxyl carrier protein [Enterococcus faecalis V583]
>gi|29377767|ref|NP_816921.1| hypothetical protein EF3326 [Enterococcus faecalis V583]
>gi|29377771|ref|NP_816925.1| jag protein, putative [Enterococcus faecalis V583]
>gi|29377826|ref|NP_816954.1| hypothetical protein EFA0029 [Enterococcus faecalis V583]
>gi|29377832|ref|NP_816960.1| hypothetical protein EFA0035 [Enterococcus faecalis V583]
>gi|29377833|ref|NP_816961.1| hypothetical protein EFA0036 [Enterococcus faecalis V583]
>gi|29377839|ref|NP_816967.1| cell wall surface anchor signal protein [Enterococcus faecalis V583]
>gi|29377848|ref|NP_816976.1| surface exclusion protein Sea1 [Enterococcus faecalis V583]
>gi|29377904|ref|NP_817030.1| surface exclusion protein PrgA [Enterococcus faecalis V583]
>gi|29377906|ref|NP_817032.1| surface protein PrgC [Enterococcus faecalis V583]
>gi|29377917|ref|NP_817043.1| hypothetical protein EFB0023 [Enterococcus faecalis V583]
>gi|29377924|ref|NP_817050.1| hypothetical protein EFB0031 [Enterococcus faecalis V583]
>gi|15672023|ref|NP_266197.1| prophage ps1 protein 17 [Lactococcus lactis subsp. lactis Il1403]
>gi|15672037|ref|NP_266211.1| hypothetical protein L55129 [Lactococcus lactis subsp. lactis Il1403]
>gi|15672051|ref|NP_266225.1| hypothetical protein L70747 [Lactococcus lactis subsp. lactis Il1403]
>gi|15672057|ref|NP_266231.1| hypothetical protein L1010 [Lactococcus lactis subsp. lactis Il1403]
>gi|15672063|ref|NP_266237.1| transporter [Lactococcus lactis subsp. lactis Il1403]
>gi|15672101|ref|NP_266275.1| hypothetical protein L122461 [Lactococcus lactis subsp. lactis Il1403]
>gi|15672115|ref|NP_266289.1| hypothetical protein L135555 [Lactococcus lactis subsp. lactis Il1403]
>gi|15672139|ref|NP_266313.1| hypothetical protein L160332 [Lactococcus lactis subsp. lactis Il1403]
>gi|15672140|ref|NP_266314.1| hypothetical protein L160556 [Lactococcus lactis subsp. lactis Il1403]
>gi|15672160|ref|NP_266334.1| hypothetical protein L183216 [Lactococcus lactis subsp. lactis Il1403]
>gi|15672167|ref|NP_266341.1| hypothetical protein L189326 [Lactococcus lactis subsp. lactis Il1403]
>gi|15672192|ref|NP_266366.1| hypothetical protein L11986 [Lactococcus lactis subsp. lactis Il1403]
>gi|15672222|ref|NP_266396.1| 30S ribosomal protein S21 [Lactococcus lactis subsp. lactis Il1403]
>gi|15672223|ref|NP_266397.1| hypothetical protein L37573 [Lactococcus lactis subsp. lactis Il1403]
>gi|15672225|ref|NP_266399.1| hypothetical protein L41256 [Lactococcus lactis subsp. lactis Il1403]
>gi|15672233|ref|NP_266407.1| glycerol uptake facilitator [Lactococcus lactis subsp. lactis Il1403]
>gi|15672254|ref|NP_266428.1| N-acetylmuramidase [Lactococcus lactis subsp. lactis Il1403]
>gi|15672263|ref|NP_266437.1| acetyltransferase [Lactococcus lactis subsp. lactis Il1403]
>gi|15672271|ref|NP_266445.1| hypothetical protein L88801 [Lactococcus lactis subsp. lactis Il1403]
>gi|30023986|ref|NP_266470.2| hypothetical protein L114632 [Lactococcus lactis subsp. lactis Il1403]
>gi|15672366|ref|NP_266540.1| hypothetical protein L189448 [Lactococcus lactis subsp. lactis Il1403]
>gi|15672413|ref|NP_266587.1| hypothetical protein L43651 [Lactococcus lactis subsp. lactis Il1403]
>gi|15672460|ref|NP_266634.1| prophage pi1 protein 43 [Lactococcus lactis subsp. lactis Il1403]
>gi|15672506|ref|NP_266680.1| hypothetical protein L114078 [Lactococcus lactis subsp. lactis Il1403]
>gi|15672507|ref|NP_266681.1| hypothetical protein L114419 [Lactococcus lactis subsp. lactis Il1403]
>gi|15672559|ref|NP_266733.1| hypothetical protein L171552 [Lactococcus lactis subsp. lactis Il1403]
>gi|30024002|ref|NP_266764.2| flavodoxin [Lactococcus lactis subsp. lactis Il1403]
>gi|15672597|ref|NP_266771.1| hypothetical protein L3407 [Lactococcus lactis subsp. lactis Il1403]
>gi|15672616|ref|NP_266790.1| DNA-directed RNA polymerase delta chain [Lactococcus lactis subsp. lactis Il1403]
>gi|15672617|ref|NP_266791.1| hypothetical protein L24228 [Lactococcus lactis subsp. lactis Il1403]
>gi|15672721|ref|NP_266895.1| flotillin-like protein [Lactococcus lactis subsp. lactis Il1403]
>gi|15672727|ref|NP_266901.1| 50S ribosomal protein L9 [Lactococcus lactis subsp. lactis Il1403]
>gi|30024009|ref|NP_835302.1| hypothetical protein L200028 [Lactococcus lactis subsp. lactis Il1403]
>gi|15672739|ref|NP_266913.1| hypothetical protein L164132 [Lactococcus lactis subsp. lactis Il1403]
>gi|15672740|ref|NP_266914.1| hypothetical protein L167770 [Lactococcus lactis subsp. lactis Il1403]
>gi|15672749|ref|NP_266923.1| translation initiation factor IF-2 [Lactococcus lactis subsp. lactis Il1403]
>gi|15672758|ref|NP_266932.1| biotin carboxyl carrier protein of acetyl-CoA carboxylase [Lactococcus lactis subsp. lactis Il1403]
>gi|15672765|ref|NP_266939.1| hypothetical protein L193031 [Lactococcus lactis subsp. lactis Il1403]
>gi|15672790|ref|NP_266964.1| hypothetical protein L17781 [Lactococcus lactis subsp. lactis Il1403]
>gi|15672795|ref|NP_266969.1| FtsY [Lactococcus lactis subsp. lactis Il1403]
>gi|30024010|ref|NP_835303.1| hypothetical protein L200030 [Lactococcus lactis subsp. lactis Il1403]
>gi|15672834|ref|NP_267008.1| hypothetical protein L73264 [Lactococcus lactis subsp. lactis Il1403]
>gi|15672852|ref|NP_267026.1| ABC transporter permease protein [Lactococcus lactis subsp. lactis Il1403]
>gi|15672857|ref|NP_267031.1| hypothetical protein L98046 [Lactococcus lactis subsp. lactis Il1403]
>gi|15672864|ref|NP_267038.1| hypothetical protein L103741 [Lactococcus lactis subsp. lactis Il1403]

>gi|15672889|ref|NP_267063.1| hypothetical protein L131027 [Lactococcus lactis subsp. lactis Il1403]
>gi|15672898|ref|NP_267072.1| teichoic acid ABC transporter permease protein [Lactococcus lactis subsp. lactis Il1403]
>gi|15672933|ref|NP_267107.1| hypothetical protein L175880 [Lactococcus lactis subsp. lactis Il1403]
>gi|15673007|ref|NP_267181.1| prophage pi2 protein 18 [Lactococcus lactis subsp. lactis Il1403]
>gi|15673040|ref|NP_267214.1| holin [Lactococcus lactis subsp. lactis Il1403]
>gi|15673046|ref|NP_267220.1| hypothetical protein L76119 [Lactococcus lactis subsp. lactis Il1403]
>gi|15673047|ref|NP_267221.1| hypothetical protein L77017 [Lactococcus lactis subsp. lactis Il1403]
>gi|15673074|ref|NP_267248.1| hypothetical protein L98876 [Lactococcus lactis subsp. lactis Il1403]
>gi|15673107|ref|NP_267281.1| hypothetical protein L139276 [Lactococcus lactis subsp. lactis Il1403]
>gi|15673145|ref|NP_267319.1| spermidine/putrescine ABC transporter ATP-binding protein [Lactococcus lactis subsp. lactis Il1403]
>gi|15673163|ref|NP_267337.1| hypothetical protein L197593 [Lactococcus lactis subsp. lactis Il1403]
>gi|15673167|ref|NP_267341.1| hypothetical protein L2081 [Lactococcus lactis subsp. lactis Il1403]
>gi|15673201|ref|NP_267375.1| hypothetical protein L42099 [Lactococcus lactis subsp. lactis Il1403]
>gi|15673224|ref|NP_267398.1| hypothetical protein L67002 [Lactococcus lactis subsp. lactis Il1403]
>gi|15673250|ref|NP_267424.1| 50S ribosomal protein L7/L12 [Lactococcus lactis subsp. lactis Il1403]
>gi|15673307|ref|NP_267481.1| hypothetical protein L159364 [Lactococcus lactis subsp. lactis Il1403]
>gi|15673343|ref|NP_267517.1| hypothetical protein L196808 [Lactococcus lactis subsp. lactis Il1403]
>gi|15673351|ref|NP_267525.1| hypothetical protein L7415 [Lactococcus lactis subsp. lactis Il1403]
>gi|15673394|ref|NP_267568.1| prophage pi3 protein 39 [Lactococcus lactis subsp. lactis Il1403]
>gi|15673395|ref|NP_267569.1| prophage pi3 protein 40 [Lactococcus lactis subsp. lactis Il1403]
>gi|15673402|ref|NP_267576.1| replisome organiser [Lactococcus lactis subsp. lactis Il1403]
>gi|15673427|ref|NP_267601.1| hypothetical protein L67275 [Lactococcus lactis subsp. lactis Il1403]
>gi|15673440|ref|NP_267614.1| hypothetical protein L84260 [Lactococcus lactis subsp. lactis Il1403]
>gi|15673472|ref|NP_267646.1| hypothetical protein L121071 [Lactococcus lactis subsp. lactis Il1403]
>gi|15673524|ref|NP_267698.1| hypothetical protein L183494 [Lactococcus lactis subsp. lactis Il1403]
>gi|15673530|ref|NP_267704.1| hypothetical protein L187666 [Lactococcus lactis subsp. lactis Il1403]
>gi|15673539|ref|NP_267713.1| hypothetical protein L199664 [Lactococcus lactis subsp. lactis Il1403]
>gi|15673577|ref|NP_267751.1| hypothetical protein L39365 [Lactococcus lactis subsp. lactis Il1403]
>gi|15673587|ref|NP_267761.1| hypothetical protein L48287 [Lactococcus lactis subsp. lactis Il1403]
>gi|15673595|ref|NP_267769.1| amino acid permease [Lactococcus lactis subsp. lactis Il1403]
>gi|15673599|ref|NP_267773.1| hypothetical protein L58460 [Lactococcus lactis subsp. lactis Il1403]
>gi|15673616|ref|NP_267790.1| multidrug transporter [Lactococcus lactis subsp. lactis Il1403]
>gi|15673632|ref|NP_267806.1| hypothetical protein L98109 [Lactococcus lactis subsp. lactis Il1403]
>gi|15673686|ref|NP_267860.1| hypothetical protein L144535 [Lactococcus lactis subsp. lactis Il1403]
>gi|15673721|ref|NP_267895.1| 30S ribosomal protein S20 [Lactococcus lactis subsp. lactis Il1403]
>gi|15673745|ref|NP_267919.1| ATP synthase epsilon subunit [Lactococcus lactis subsp. lactis Il1403]
>gi|15674249|ref|NP_267926.1| F0F1-type ATP synthase c subunit [Lactococcus lactis subsp. lactis Il1403]
>gi|15673752|ref|NP_267927.1| hypothetical protein L12489 [Lactococcus lactis subsp. lactis Il1403]
>gi|15673766|ref|NP_267941.1| hypothetical protein L27103 [Lactococcus lactis subsp. lactis Il1403]
>gi|15673809|ref|NP_267984.1| hypothetical protein L77841 [Lactococcus lactis subsp. lactis Il1403]
>gi|15673824|ref|NP_267999.1| 50S ribosomal protein L20 [Lactococcus lactis subsp. lactis Il1403]
>gi|15673846|ref|NP_268021.1| highly hypothetical protein [Lactococcus lactis subsp. lactis Il1403]
>gi|15673849|ref|NP_268024.1| hypothetical protein L119731 [Lactococcus lactis subsp. lactis Il1403]
>gi|15673880|ref|NP_268055.1| hypothetical protein L149295 [Lactococcus lactis subsp. lactis Il1403]
>gi|15673887|ref|NP_268062.1| hypothetical protein L157730 [Lactococcus lactis subsp. lactis Il1403]
>gi|15673889|ref|NP_268064.1| N-acetylmuramidase [Lactococcus lactis subsp. lactis Il1403]
>gi|15673926|ref|NP_268101.1| prophage ps3 protein 12 [Lactococcus lactis subsp. lactis Il1403]
>gi|15673935|ref|NP_268110.1| transport protein [Lactococcus lactis subsp. lactis Il1403]
>gi|15673953|ref|NP_268128.1| hypothetical protein L30285 [Lactococcus lactis subsp. lactis Il1403]
>gi|15673956|ref|NP_268131.1| hypothetical protein L34138 [Lactococcus lactis subsp. lactis Il1403]
>gi|15673972|ref|NP_268147.1| hypothetical protein L53755 [Lactococcus lactis subsp. lactis Il1403]
>gi|15674010|ref|NP_268185.1| type 4 prepilin-like protein specific leader peptidase [Lactococcus lactis subsp. lactis Il1403]
>gi|15674061|ref|NP_268236.1| 50S ribosomal protein L15 [Lactococcus lactis subsp. lactis Il1403]
>gi|15674067|ref|NP_268242.1| hypothetical protein L146261 [Lactococcus lactis subsp. lactis Il1403]
>gi|15674107|ref|NP_268282.1| hypothetical protein L177685 [Lactococcus lactis subsp. lactis Il1403]
>gi|15674118|ref|NP_268293.1| exported serine protease [Lactococcus lactis subsp. lactis Il1403]
>gi|15674134|ref|NP_268309.1| elongation factor Ts [Lactococcus lactis subsp. lactis Il1403]
>gi|15674191|ref|NP_268366.1| hypothetical protein L75803 [Lactococcus lactis subsp. lactis Il1403]
>gi|15674201|ref|NP_268376.1| hypothetical protein L84494 [Lactococcus lactis subsp. lactis Il1403]
>gi|15674211|ref|NP_268386.1| hypothetical protein L96658 [Lactococcus lactis subsp. lactis Il1403]
>gi|71902683|ref|YP_279486.1| secreted protein [Streptococcus pyogenes MGAS6180]
>gi|71902687|ref|YP_279490.1| acyl carrier protein [Streptococcus pyogenes MGAS6180]
>gi|71902710|ref|YP_279513.1| LSU ribosomal protein L1E [Streptococcus pyogenes MGAS6180]
>gi|71902717|ref|YP_279520.1| LSU ribosomal protein L29P [Streptococcus pyogenes MGAS6180]
>gi|71902771|ref|YP_279574.1| fibronectin binding protein 1 [Streptococcus pyogenes MGAS6180]
>gi|71902792|ref|YP_279595.1| V-type sodium ATP synthase subunit E [Streptococcus pyogenes MGAS6180]
>gi|71902806|ref|YP_279609.1| hypothetical protein M28_Spy0140 [Streptococcus pyogenes MGAS6180]
>gi|71902841|ref|YP_279644.1| BioY [Streptococcus pyogenes MGAS6180]
>gi|71902876|ref|YP_279679.1| hypothetical membrane spanning protein [Streptococcus pyogenes MGAS6180]
>gi|71902898|ref|YP_279701.1| hypothetical protein M28_Spy0232 [Streptococcus pyogenes MGAS6180]

>gi|71902938|ref|YP_279741.1| heat shock protein HtpX [Streptococcus pyogenes MGAS6180]
>gi|71902969|ref|YP_279772.1| phosphatidylglycerophosphatase B-like protein [Streptococcus pyogenes MGAS6180]
>gi|71902976|ref|YP_279779.1| ferrichrome transport system permease protein [Streptococcus pyogenes MGAS6180]
>gi|71903008|ref|YP_279811.1| hypothetical membrane spanning protein [Streptococcus pyogenes MGAS6180]
>gi|71903012|ref|YP_279815.1| hypothetical protein M28_Spy0346 [Streptococcus pyogenes MGAS6180]
>gi|71903036|ref|YP_279839.1| surface antigen [Streptococcus pyogenes MGAS6180]
>gi|71903045|ref|YP_279848.1| hypothetical protein M28_Spy0379 [Streptococcus pyogenes MGAS6180]
>gi|71903046|ref|YP_279849.1| hypothetical protein M28_Spy0380 [Streptococcus pyogenes MGAS6180]
>gi|71903049|ref|YP_279852.1| bacteriocin [Streptococcus pyogenes MGAS6180]
>gi|71903050|ref|YP_279853.1| hypothetical membrane associated protein [Streptococcus pyogenes MGAS6180]
>gi|71903126|ref|YP_279929.1| hypothetical membrane spanning protein [Streptococcus pyogenes MGAS6180]
>gi|71903130|ref|YP_279933.1| stress-responsive transcriptional regulator PspC [Streptococcus pyogenes MGAS6180]
>gi|71903139|ref|YP_279942.1| hypothetical protein M28_Spy0473 [Streptococcus pyogenes MGAS6180]
>gi|71903146|ref|YP_279949.1| integral membrane protein [Streptococcus pyogenes MGAS6180]
>gi|71903205|ref|YP_280008.1| putative extracellular matrix binding protein [Streptococcus pyogenes MGAS6180]
>gi|71903206|ref|YP_280009.1| streptolysin S precursor [Streptococcus pyogenes MGAS6180]
>gi|71903211|ref|YP_280014.1| streptolysin S biosynthesis protein [Streptococcus pyogenes MGAS6180]
>gi|71903238|ref|YP_280041.1| neutral zinc metallopeptidase family [Streptococcus pyogenes MGAS6180]
>gi|71903242|ref|YP_280045.1| SSU ribosomal protein S21P [Streptococcus pyogenes MGAS6180]
>gi|71903262|ref|YP_280065.1| hypothetical membrane associated protein [Streptococcus pyogenes MGAS6180]
>gi|71903275|ref|YP_280078.1| hypothetical protein M28_Spy0609 [Streptococcus pyogenes MGAS6180]
>gi|71903279|ref|YP_280082.1| LSU ribosomal protein L21P [Streptococcus pyogenes MGAS6180]
>gi|71903309|ref|YP_280112.1| PTS system, fructose-specific IIABC component [Streptococcus pyogenes MGAS6180]
>gi|71903355|ref|YP_280158.1| hypothetical membrane spanning protein [Streptococcus pyogenes MGAS6180]
>gi|71903390|ref|YP_280193.1| ABC transporter ATP-binding protein [Streptococcus pyogenes MGAS6180]
>gi|71903419|ref|YP_280222.1| collagen-like surface protein [Streptococcus pyogenes MGAS6180]
>gi|71903435|ref|YP_280238.1| LSU ribosomal protein L7/L12P [Streptococcus pyogenes MGAS6180]
>gi|71903451|ref|YP_280254.1| lantibiotic transport permease protein [Streptococcus pyogenes MGAS6180]
>gi|71903452|ref|YP_280255.1| lantibiotic transport permease protein [Streptococcus pyogenes MGAS6180]
>gi|71903478|ref|YP_280281.1| chloride channel protein [Streptococcus pyogenes MGAS6180]
>gi|71903517|ref|YP_280320.1| hypothetical protein M28_Spy0852 [Streptococcus pyogenes MGAS6180]
>gi|71903533|ref|YP_280336.1| hypothetical membrane associated protein [Streptococcus pyogenes MGAS6180]
>gi|71903540|ref|YP_280343.1| biotin carboxyl carrier protein of oxaloacetate decarboxylase [Streptococcus pyogenes MGAS6180]
>gi|71903574|ref|YP_280377.1| transporter [Streptococcus pyogenes MGAS6180]
>gi|71903583|ref|YP_280386.1| SSU ribosomal protein S20P [Streptococcus pyogenes MGAS6180]
>gi|71903609|ref|YP_280412.1| hypothetical protein M28_Spy0944 [Streptococcus pyogenes MGAS6180]
>gi|71903611|ref|YP_280414.1| hypothetical protein M28_Spy0946 [Streptococcus pyogenes MGAS6180]
>gi|71903613|ref|YP_280416.1| integral membrane protein [Streptococcus pyogenes MGAS6180]
>gi|71903645|ref|YP_280448.1| phage protein [Streptococcus pyogenes phage 6180.1]
>gi|71903653|ref|YP_280456.1| phage protein [Streptococcus pyogenes phage 6180.1]
>gi|71903746|ref|YP_280549.1| putative phage-associated protein [Streptococcus pyogenes MGAS6180]
>gi|71903747|ref|YP_280550.1| hypothetical protein M28_Spy1082 [Streptococcus pyogenes MGAS6180]
>gi|71903748|ref|YP_280551.1| nickase [Streptococcus pyogenes MGAS6180]
>gi|71903751|ref|YP_280554.1| hypothetical protein M28_Spy1086 [Streptococcus pyogenes MGAS6180]
>gi|71903763|ref|YP_280566.1| protein G-related alpha 2M-binding protein [Streptococcus pyogenes MGAS6180]
>gi|71903768|ref|YP_280571.1| hypothetical protein M28_Spy1103 [Streptococcus pyogenes MGAS6180]
>gi|71903804|ref|YP_280607.1| ComE operon protein 3 [Streptococcus pyogenes MGAS6180]
>gi|71903818|ref|YP_280621.1| hypothetical protein M28_Spy1156 [Streptococcus pyogenes MGAS6180]
>gi|71903828|ref|YP_280631.1| hypothetical protein M28_Spy1166 [Streptococcus pyogenes MGAS6180]
>gi|71903861|ref|YP_280664.1| prepilin peptidase family [Streptococcus pyogenes MGAS6180]
>gi|71903863|ref|YP_280666.1| transcriptional regulator [Streptococcus pyogenes MGAS6180]
>gi|71903883|ref|YP_280686.1| hypothetical protein M28_Spy1221 [Streptococcus pyogenes phage 6180.2]
>gi|71903892|ref|YP_280695.1| phage protein [Streptococcus pyogenes phage 6180.2]
>gi|71903932|ref|YP_280735.1| phage protein [Streptococcus pyogenes phage 6180.2]
>gi|71903949|ref|YP_280752.1| thiol:disulfide interchange protein [Streptococcus pyogenes MGAS6180]
>gi|71903968|ref|YP_280771.1| putative cell surface protein [Streptococcus pyogenes MGAS6180]
>gi|71903987|ref|YP_280790.1| putative cell surface protein [Streptococcus pyogenes MGAS6180]
>gi|71903988|ref|YP_280791.1| putative cell surface protein [Streptococcus pyogenes MGAS6180]
>gi|71903998|ref|YP_280801.1| R28 protein [Streptococcus pyogenes MGAS6180]
>gi|71904037|ref|YP_280840.1| transporter [Streptococcus pyogenes MGAS6180]
>gi|71904043|ref|YP_280846.1| DNA-directed RNA polymerase omega chain [Streptococcus pyogenes MGAS6180]
>gi|71904084|ref|YP_280887.1| glycerol uptake facilitator protein [Streptococcus pyogenes MGAS6180]
>gi|71904095|ref|YP_280898.1| hypothetical protein M28_Spy1433 [Streptococcus pyogenes MGAS6180]
>gi|71904121|ref|YP_280924.1| EcsB [Streptococcus pyogenes MGAS6180]
>gi|71904128|ref|YP_280931.1| guanine-hypoxanthine permease [Streptococcus pyogenes MGAS6180]
>gi|71904150|ref|YP_280953.1| hypothetical protein M28_Spy1488 [Streptococcus pyogenes MGAS6180]
>gi|71904174|ref|YP_280977.1| cobalt transport protein cbiQ [Streptococcus pyogenes MGAS6180]
>gi|71904179|ref|YP_280982.1| ferrichrome transport system permease protein fhuB [Streptococcus pyogenes MGAS6180]
>gi|71904212|ref|YP_281015.1| colicin V production protein [Streptococcus pyogenes MGAS6180]
>gi|71904233|ref|YP_281036.1| hypothetical cytosolic protein [Streptococcus pyogenes MGAS6180]

>gi|71904238|ref|YP_281041.1| hypothetical protein M28_Spy1576 [Streptococcus pyogenes MGAS6180]
>gi|71904262|ref|YP_281065.1| DNA-directed RNA polymerase delta chain [Streptococcus pyogenes MGAS6180]
>gi|71904337|ref|YP_281140.1| collagen-like surface protein A [Streptococcus pyogenes MGAS6180]
>gi|71904356|ref|YP_281159.1| hypothetical protein M28_Spy1694 [Streptococcus pyogenes MGAS6180]
>gi|71904361|ref|YP_281164.1| cell surface protein [Streptococcus pyogenes MGAS6180]
>gi|71904363|ref|YP_281166.1| enn protein [Streptococcus pyogenes MGAS6180]
>gi|71904364|ref|YP_281167.1| emm28 protein [Streptococcus pyogenes MGAS6180]
>gi|71904365|ref|YP_281168.1| emm-like protein [Streptococcus pyogenes MGAS6180]
>gi|71904369|ref|YP_281172.1| immunogenic secreted protein [Streptococcus pyogenes MGAS6180]
>gi|71904410|ref|YP_281213.1| 10 kDa chaperonin [Streptococcus pyogenes MGAS6180]
>gi|71904460|ref|YP_281263.1| phage protein [Streptococcus pyogenes phage 6180.3]
>gi|71904488|ref|YP_281291.1| cadmium resistance protein [Streptococcus pyogenes MGAS6180]
>gi|71904500|ref|YP_281303.1| phage protein [Streptococcus pyogenes MGAS6180]
>gi|71904518|ref|YP_281321.1| phage protein [Streptococcus pyogenes phage 6180.4]
>gi|71904530|ref|YP_281333.1| LSU ribosomal protein L9P [Streptococcus pyogenes MGAS6180]
>gi|71904537|ref|YP_281340.1| transglycosylase SLT domain family protein [Streptococcus pyogenes MGAS6180]
>gi|71904545|ref|YP_281348.1| hyaluronan synthase [Streptococcus pyogenes MGAS6180]
>gi|66766359|ref|YP_241121.1| hypothetical protein XC_0007 [Xanthomonas campestris pv. campestris str. 8004]
>gi|66766360|ref|YP_241122.1| TonB protein [Xanthomonas campestris pv. campestris str. 8004]
>gi|66766365|ref|YP_241127.1| hypothetical protein XC_0013 [Xanthomonas campestris pv. campestris str. 8004]
>gi|66766370|ref|YP_241132.1| hypothetical protein XC_0018 [Xanthomonas campestris pv. campestris str. 8004]
>gi|66766373|ref|YP_241135.1| carboxyl-terminal protease [Xanthomonas campestris pv. campestris str. 8004]
>gi|66766374|ref|YP_241136.1| hypothetical protein XC_0022 [Xanthomonas campestris pv. campestris str. 8004]
>gi|66766375|ref|YP_241137.1| hypothetical protein XC_0023 [Xanthomonas campestris pv. campestris str. 8004]
>gi|66766385|ref|YP_241147.1| hypothetical protein XC_0033 [Xanthomonas campestris pv. campestris str. 8004]
>gi|66766390|ref|YP_241152.1| transcriptional regulator lysR family [Xanthomonas campestris pv. campestris str. 8004]
>gi|66766396|ref|YP_241158.1| ankyrin-like protein [Xanthomonas campestris pv. campestris str. 8004]
>gi|66766397|ref|YP_241159.1| hypothetical protein XC_0047 [Xanthomonas campestris pv. campestris str. 8004]
>gi|66766420|ref|YP_241182.1| hypothetical protein XC_0070 [Xanthomonas campestris pv. campestris str. 8004]
>gi|66766423|ref|YP_241185.1| hypothetical protein XC_0073 [Xanthomonas campestris pv. campestris str. 8004]
>gi|66766424|ref|YP_241186.1| hypothetical protein XC_0074 [Xanthomonas campestris pv. campestris str. 8004]
>gi|66766430|ref|YP_241192.1| hypothetical protein XC_0080 [Xanthomonas campestris pv. campestris str. 8004]
>gi|66766437|ref|YP_241199.1| hypothetical protein XC_0087 [Xanthomonas campestris pv. campestris str. 8004]
>gi|66766438|ref|YP_241200.1| hypothetical protein XC_0088 [Xanthomonas campestris pv. campestris str. 8004]
>gi|66766439|ref|YP_241201.1| hypothetical protein XC_0089 [Xanthomonas campestris pv. campestris str. 8004]
>gi|66766440|ref|YP_241202.1| hypothetical protein XC_0090 [Xanthomonas campestris pv. campestris str. 8004]
>gi|66766458|ref|YP_241220.1| ATP-dependent DNA ligase [Xanthomonas campestris pv. campestris str. 8004]
>gi|66766460|ref|YP_241222.1| hypothetical protein XC_0111 [Xanthomonas campestris pv. campestris str. 8004]
>gi|66766462|ref|YP_241224.1| two-component system sensor protein [Xanthomonas campestris pv. campestris str. 8004]
>gi|66766467|ref|YP_241229.1| transcriptional regulator [Xanthomonas campestris pv. campestris str. 8004]
>gi|66766477|ref|YP_241239.1| hypothetical protein XC_0128 [Xanthomonas campestris pv. campestris str. 8004]
>gi|66766478|ref|YP_241240.1| hypothetical protein XC_0129 [Xanthomonas campestris pv. campestris str. 8004]
>gi|66766480|ref|YP_241242.1| hypothetical protein XC_0131 [Xanthomonas campestris pv. campestris str. 8004]
>gi|66766491|ref|YP_241253.1| hypothetical protein XC_0146 [Xanthomonas campestris pv. campestris str. 8004]
>gi|66766494|ref|YP_241256.1| hypothetical protein XC_0149 [Xanthomonas campestris pv. campestris str. 8004]
>gi|66766496|ref|YP_241258.1| hypothetical protein XC_0151 [Xanthomonas campestris pv. campestris str. 8004]
>gi|66766502|ref|YP_241264.1| C4-dicarboxylate transport protein [Xanthomonas campestris pv. campestris str. 8004]
>gi|66766526|ref|YP_241288.1| hypothetical protein XC_0181 [Xanthomonas campestris pv. campestris str. 8004]
>gi|66766537|ref|YP_241299.1| hypothetical protein XC_0192 [Xanthomonas campestris pv. campestris str. 8004]
>gi|66766541|ref|YP_241303.1| ammonium transporter [Xanthomonas campestris pv. campestris str. 8004]
>gi|66766542|ref|YP_241304.1| two-component system sensor protein [Xanthomonas campestris pv. campestris str. 8004]
>gi|66766553|ref|YP_241315.1| uroporphyrinogen-III synthase [Xanthomonas campestris pv. campestris str. 8004]
>gi|66766555|ref|YP_241317.1| hypothetical protein XC_0210 [Xanthomonas campestris pv. campestris str. 8004]
>gi|66766556|ref|YP_241318.1| hypothetical protein XC_0211 [Xanthomonas campestris pv. campestris str. 8004]
>gi|66766563|ref|YP_241325.1| MFS transporter [Xanthomonas campestris pv. campestris str. 8004]
>gi|66766565|ref|YP_241327.1| hypothetical protein XC_0220 [Xanthomonas campestris pv. campestris str. 8004]
>gi|66766576|ref|YP_241338.1| acetyltransferase, GNAT family [Xanthomonas campestris pv. campestris str. 8004]
>gi|66766581|ref|YP_241343.1| hypothetical protein XC_0236 [Xanthomonas campestris pv. campestris str. 8004]
>gi|66766604|ref|YP_241366.1| hypothetical protein XC_0259 [Xanthomonas campestris pv. campestris str. 8004]
>gi|66766605|ref|YP_241367.1| hypothetical protein XC_0260 [Xanthomonas campestris pv. campestris str. 8004]
>gi|66766609|ref|YP_241371.1| cytochrome C5 [Xanthomonas campestris pv. campestris str. 8004]
>gi|66766611|ref|YP_241373.1| hypothetical protein XC_0266 [Xanthomonas campestris pv. campestris str. 8004]
>gi|66766613|ref|YP_241375.1| hypothetical protein XC_0268 [Xanthomonas campestris pv. campestris str. 8004]
>gi|66766614|ref|YP_241376.1| integral membrane protein [Xanthomonas campestris pv. campestris str. 8004]
>gi|66766628|ref|YP_241390.1| hypothetical protein XC_0284 [Xanthomonas campestris pv. campestris str. 8004]
>gi|66766629|ref|YP_241391.1| ATP-dependent RNA helicase [Xanthomonas campestris pv. campestris str. 8004]
>gi|66766630|ref|YP_241392.1| chemotaxis protein [Xanthomonas campestris pv. campestris str. 8004]
>gi|66766632|ref|YP_241394.1| hypothetical protein XC_0288 [Xanthomonas campestris pv. campestris str. 8004]
>gi|66766642|ref|YP_241404.1| transcriptional regulator [Xanthomonas campestris pv. campestris str. 8004]
>gi|66766646|ref|YP_241408.1| Glu-tRNAGln amidotransferase A subunit [Xanthomonas campestris pv. campestris str. 8004]

>gi|66766649|ref|YP_241411.1| xanthine/uracil permease [Xanthomonas campestris pv. campestris str. 8004]
>gi|66766651|ref|YP_241413.1| vanillate O-demethylase oxidoreductase [Xanthomonas campestris pv. campestris str. 8004]
>gi|66766657|ref|YP_241419.1| MFS transporter [Xanthomonas campestris pv. campestris str. 8004]
>gi|66766666|ref|YP_241428.1| transcriptional regulator [Xanthomonas campestris pv. campestris str. 8004]
>gi|66766676|ref|YP_241438.1| oxidoreductase [Xanthomonas campestris pv. campestris str. 8004]
>gi|66766678|ref|YP_241440.1| hypothetical protein XC_0335 [Xanthomonas campestris pv. campestris str. 8004]
>gi|66766679|ref|YP_241441.1| chemotaxis protein [Xanthomonas campestris pv. campestris str. 8004]
>gi|66766686|ref|YP_241448.1| hypothetical protein XC_0343 [Xanthomonas campestris pv. campestris str. 8004]
>gi|66766704|ref|YP_241466.1| MFS transporter [Xanthomonas campestris pv. campestris str. 8004]
>gi|66766724|ref|YP_241486.1| 3-carboxy-cis,cis-muconate cycloisomerase [Xanthomonas campestris pv. campestris str. 8004]
>gi|66766733|ref|YP_241495.1| hypothetical protein XC_0390 [Xanthomonas campestris pv. campestris str. 8004]
>gi|66766734|ref|YP_241496.1| threonine dehydratase [Xanthomonas campestris pv. campestris str. 8004]
>gi|66766740|ref|YP_241502.1| biotin biosynthesis protein [Xanthomonas campestris pv. campestris str. 8004]
>gi|66766744|ref|YP_241506.1| competence protein F [Xanthomonas campestris pv. campestris str. 8004]
>gi|66766762|ref|YP_241524.1| hypothetical protein XC_0420 [Xanthomonas campestris pv. campestris str. 8004]
>gi|66766774|ref|YP_241536.1| outer membrane protein [Xanthomonas campestris pv. campestris str. 8004]
>gi|66766776|ref|YP_241538.1| component of multidrug efflux system [Xanthomonas campestris pv. campestris str. 8004]
>gi|66766781|ref|YP_241543.1| dihydrolipoamide acyltransferase [Xanthomonas campestris pv. campestris str. 8004]
>gi|66766788|ref|YP_241550.1| hypothetical protein XC_0448 [Xanthomonas campestris pv. campestris str. 8004]
>gi|66766793|ref|YP_241555.1| hypothetical protein XC_0454 [Xanthomonas campestris pv. campestris str. 8004]
>gi|66766794|ref|YP_241556.1| hypothetical protein XC_0453 [Xanthomonas campestris pv. campestris str. 8004]
>gi|66766798|ref|YP_241560.1| PhaD protein [Xanthomonas campestris pv. campestris str. 8004]
>gi|66766802|ref|YP_241564.1| hypothetical protein XC_0462 [Xanthomonas campestris pv. campestris str. 8004]
>gi|66766804|ref|YP_241566.1| hypothetical protein XC_0465 [Xanthomonas campestris pv. campestris str. 8004]
>gi|66766807|ref|YP_241569.1| hypothetical protein XC_0468 [Xanthomonas campestris pv. campestris str. 8004]
>gi|66766813|ref|YP_241575.1| threonine aldolase [Xanthomonas campestris pv. campestris str. 8004]
>gi|66766827|ref|YP_241589.1| SugE protein [Xanthomonas campestris pv. campestris str. 8004]
>gi|66766836|ref|YP_241598.1| hypothetical protein XC_0497 [Xanthomonas campestris pv. campestris str. 8004]
>gi|66766838|ref|YP_241600.1| hypothetical protein XC_0499 [Xanthomonas campestris pv. campestris str. 8004]
>gi|66766841|ref|YP_241603.1| transmembrane protein [Xanthomonas campestris pv. campestris str. 8004]
>gi|66766846|ref|YP_241608.1| MFS transporter [Xanthomonas campestris pv. campestris str. 8004]
>gi|66766852|ref|YP_241614.1| hypothetical protein XC_0513 [Xanthomonas campestris pv. campestris str. 8004]
>gi|66766854|ref|YP_241616.1| hypothetical protein XC_0515 [Xanthomonas campestris pv. campestris str. 8004]
>gi|66766855|ref|YP_241617.1| CDP-diacylglycerol-glycerol-3-phosphate 3-phosphatidyltransferase-related protein [Xanthomonas campestris pv. campestris str. 8004]
>gi|66766858|ref|YP_241620.1| ice nucleation protein [Xanthomonas campestris pv. campestris str. 8004]
>gi|66766860|ref|YP_241622.1| hypothetical protein XC_0521 [Xanthomonas campestris pv. campestris str. 8004]
>gi|66766862|ref|YP_241624.1| hypothetical protein XC_0523 [Xanthomonas campestris pv. campestris str. 8004]
>gi|66766864|ref|YP_241626.1| hypothetical protein XC_0525 [Xanthomonas campestris pv. campestris str. 8004]
>gi|66766868|ref|YP_241630.1| biotin carboxyl carrier protein of acetyl-CoA [Xanthomonas campestris pv. campestris str. 8004]
>gi|66766870|ref|YP_241632.1| c-type cytochrome biogenesis protein [Xanthomonas campestris pv. campestris str. 8004]
>gi|66766888|ref|YP_241650.1| hypothetical protein XC_0549 [Xanthomonas campestris pv. campestris str. 8004]
>gi|66766891|ref|YP_241653.1| hypothetical protein XC_0552 [Xanthomonas campestris pv. campestris str. 8004]
>gi|66766894|ref|YP_241656.1| hypothetical protein XC_0554 [Xanthomonas campestris pv. campestris str. 8004]
>gi|66766895|ref|YP_241657.1| RNA polymerase sigma factor [Xanthomonas campestris pv. campestris str. 8004]
>gi|66766899|ref|YP_241661.1| hypothetical protein XC_0560 [Xanthomonas campestris pv. campestris str. 8004]
>gi|66766900|ref|YP_241662.1| hypothetical protein XC_0561 [Xanthomonas campestris pv. campestris str. 8004]
>gi|66766908|ref|YP_241670.1| hypothetical protein XC_0569 [Xanthomonas campestris pv. campestris str. 8004]
>gi|66766916|ref|YP_241678.1| CitG protein [Xanthomonas campestris pv. campestris str. 8004]
>gi|66766918|ref|YP_241680.1| dicarboxylate carrier protein [Xanthomonas campestris pv. campestris str. 8004]
>gi|66766938|ref|YP_241700.1| hypothetical protein XC_0599 [Xanthomonas campestris pv. campestris str. 8004]
>gi|66766945|ref|YP_241707.1| hypothetical protein XC_0606 [Xanthomonas campestris pv. campestris str. 8004]
>gi|66766952|ref|YP_241714.1| hypothetical protein XC_0613 [Xanthomonas campestris pv. campestris str. 8004]
>gi|66766957|ref|YP_241719.1| hypothetical protein XC_0619 [Xanthomonas campestris pv. campestris str. 8004]
>gi|66766958|ref|YP_241720.1| hypothetical protein XC_0620 [Xanthomonas campestris pv. campestris str. 8004]
>gi|66766962|ref|YP_241724.1| hypothetical protein XC_0624 [Xanthomonas campestris pv. campestris str. 8004]
>gi|66766970|ref|YP_241732.1| hypothetical protein XC_0632 [Xanthomonas campestris pv. campestris str. 8004]
>gi|66766972|ref|YP_241734.1| hypothetical protein XC_0634 [Xanthomonas campestris pv. campestris str. 8004]
>gi|66766977|ref|YP_241739.1| cellulase [Xanthomonas campestris pv. campestris str. 8004]
>gi|66766978|ref|YP_241740.1| sulfate permease [Xanthomonas campestris pv. campestris str. 8004]
>gi|66766982|ref|YP_241744.1| sulfur deprivation response regulator [Xanthomonas campestris pv. campestris str. 8004]
>gi|66766998|ref|YP_241760.1| hypothetical protein XC_0661 [Xanthomonas campestris pv. campestris str. 8004]
>gi|66767006|ref|YP_241768.1| hypothetical protein XC_0669 [Xanthomonas campestris pv. campestris str. 8004]
>gi|66767009|ref|YP_241771.1| multidrug resistance efflux pump [Xanthomonas campestris pv. campestris str. 8004]
>gi|66767014|ref|YP_241776.1| hypothetical protein XC_0677 [Xanthomonas campestris pv. campestris str. 8004]
>gi|66767015|ref|YP_241777.1| hypothetical protein XC_0678 [Xanthomonas campestris pv. campestris str. 8004]
>gi|66767019|ref|YP_241781.1| methanol dehydrogenase heavy chain [Xanthomonas campestris pv. campestris str. 8004]
>gi|66767020|ref|YP_241782.1| MoxJ protein [Xanthomonas campestris pv. campestris str. 8004]
>gi|66767021|ref|YP_241783.1| hypothetical protein XC_0684 [Xanthomonas campestris pv. campestris str. 8004]
>gi|66767029|ref|YP_241791.1| rod shape-determining protein [Xanthomonas campestris pv. campestris str. 8004]

>gi|66767051|ref|YP_241813.1| hypothetical protein XC_0715 [Xanthomonas campestris pv. campestris str. 8004]
>gi|66767052|ref|YP_241814.1| transcriptional regulator [Xanthomonas campestris pv. campestris str. 8004]
>gi|66767058|ref|YP_241820.1| hypothetical protein XC_0722 [Xanthomonas campestris pv. campestris str. 8004]
>gi|66767059|ref|YP_241821.1| hypothetical protein XC_0723 [Xanthomonas campestris pv. campestris str. 8004]
>gi|66767071|ref|YP_241833.1| hypothetical protein XC_0735 [Xanthomonas campestris pv. campestris str. 8004]
>gi|66767074|ref|YP_241836.1| type II secretion system protein C [Xanthomonas campestris pv. campestris str. 8004]
>gi|66767080|ref|YP_241842.1| type II secretion system protein I [Xanthomonas campestris pv. campestris str. 8004]
>gi|66767081|ref|YP_241843.1| type II secretion system protein J [Xanthomonas campestris pv. campestris str. 8004]
>gi|66767083|ref|YP_241845.1| type II secretion system protein L [Xanthomonas campestris pv. campestris str. 8004]
>gi|66767085|ref|YP_241847.1| type II secretion system protein N [Xanthomonas campestris pv. campestris str. 8004]
>gi|66767100|ref|YP_241862.1| thiol:disulfide interchange protein [Xanthomonas campestris pv. campestris str. 8004]
>gi|66767108|ref|YP_241870.1| putative malic acid transport protein [Xanthomonas campestris pv. campestris str. 8004]
>gi|66767114|ref|YP_241876.1| transcriptional regulator [Xanthomonas campestris pv. campestris str. 8004]
>gi|66767123|ref|YP_241885.1| hypothetical protein XC_0790 [Xanthomonas campestris pv. campestris str. 8004]
>gi|66767157|ref|YP_241919.1| glycosyl transferase [Xanthomonas campestris pv. campestris str. 8004]
>gi|66767215|ref|YP_241977.1| hypothetical protein XC_0883 [Xanthomonas campestris pv. campestris str. 8004]
>gi|66767216|ref|YP_241978.1| hypothetical protein XC_0884 [Xanthomonas campestris pv. campestris str. 8004]
>gi|66767227|ref|YP_241989.1| thiamin-phosphate pyrophosphorylase [Xanthomonas campestris pv. campestris str. 8004]
>gi|66767235|ref|YP_241997.1| hypothetical protein XC_0903 [Xanthomonas campestris pv. campestris str. 8004]
>gi|66767236|ref|YP_241998.1| hypothetical protein XC_0904 [Xanthomonas campestris pv. campestris str. 8004]
>gi|66767239|ref|YP_242001.1| beta-lactamase [Xanthomonas campestris pv. campestris str. 8004]
>gi|66767253|ref|YP_242015.1| hypothetical protein XC_0921 [Xanthomonas campestris pv. campestris str. 8004]
>gi|66767263|ref|YP_242025.1| hypothetical protein XC_0931 [Xanthomonas campestris pv. campestris str. 8004]
>gi|66767264|ref|YP_242026.1| hypothetical protein XC_0932 [Xanthomonas campestris pv. campestris str. 8004]
>gi|66767266|ref|YP_242028.1| hypothetical protein XC_0934 [Xanthomonas campestris pv. campestris str. 8004]
>gi|66767272|ref|YP_242034.1| fimbrial assembly membrane protein [Xanthomonas campestris pv. campestris str. 8004]
>gi|66767273|ref|YP_242035.1| fimbrial assembly membrane protein [Xanthomonas campestris pv. campestris str. 8004]
>gi|66767289|ref|YP_242051.1| RNA polymerase omega subunit [Xanthomonas campestris pv. campestris str. 8004]
>gi|66767296|ref|YP_242058.1| hypothetical protein XC_0964 [Xanthomonas campestris pv. campestris str. 8004]
>gi|66767306|ref|YP_242068.1| hypothetical protein XC_0974 [Xanthomonas campestris pv. campestris str. 8004]
>gi|66767313|ref|YP_242075.1| hypothetical protein XC_0981 [Xanthomonas campestris pv. campestris str. 8004]
>gi|66767319|ref|YP_242081.1| hypothetical protein XC_0986 [Xanthomonas campestris pv. campestris str. 8004]
>gi|66767321|ref|YP_242083.1| hypothetical protein XC_0989 [Xanthomonas campestris pv. campestris str. 8004]
>gi|66767332|ref|YP_242094.1| tRNA/rRNA methyltransferase [Xanthomonas campestris pv. campestris str. 8004]
>gi|66767399|ref|YP_242161.1| hypothetical protein XC_1071 [Xanthomonas campestris pv. campestris str. 8004]
>gi|66767410|ref|YP_242172.1| hypothetical protein XC_1082 [Xanthomonas campestris pv. campestris str. 8004]
>gi|66767411|ref|YP_242173.1| hypothetical protein XC_1083 [Xanthomonas campestris pv. campestris str. 8004]
>gi|66767412|ref|YP_242174.1| hypothetical protein XC_1084 [Xanthomonas campestris pv. campestris str. 8004]
>gi|66767415|ref|YP_242177.1| ABC transporter ATP-binding subunit [Xanthomonas campestris pv. campestris str. 8004]
>gi|66767424|ref|YP_242186.1| cobalamin biosynthesis protein [Xanthomonas campestris pv. campestris str. 8004]
>gi|66767429|ref|YP_242191.1| cobalamin synthase [Xanthomonas campestris pv. campestris str. 8004]
>gi|66767432|ref|YP_242194.1| iron transporter [Xanthomonas campestris pv. campestris str. 8004]
>gi|66767433|ref|YP_242195.1| transport protein [Xanthomonas campestris pv. campestris str. 8004]
>gi|66767434|ref|YP_242196.1| hypothetical protein XC_1106 [Xanthomonas campestris pv. campestris str. 8004]
>gi|66767438|ref|YP_242200.1| cation:proton antiporter [Xanthomonas campestris pv. campestris str. 8004]
>gi|66767445|ref|YP_242207.1| hypothetical protein XC_1117 [Xanthomonas campestris pv. campestris str. 8004]
>gi|66767446|ref|YP_242208.1| transcriptional activator ampR family [Xanthomonas campestris pv. campestris str. 8004]
>gi|66767453|ref|YP_242215.1| hypothetical protein XC_1125 [Xanthomonas campestris pv. campestris str. 8004]
>gi|66767455|ref|YP_242217.1| hypothetical protein XC_1127 [Xanthomonas campestris pv. campestris str. 8004]
>gi|66767468|ref|YP_242230.1| TolA protein [Xanthomonas campestris pv. campestris str. 8004]
>gi|66767471|ref|YP_242233.1| hypothetical protein XC_1143 [Xanthomonas campestris pv. campestris str. 8004]
>gi|66767480|ref|YP_242242.1| chloride channel [Xanthomonas campestris pv. campestris str. 8004]
>gi|66767502|ref|YP_242264.1| hypothetical protein XC_1174 [Xanthomonas campestris pv. campestris str. 8004]
>gi|66767505|ref|YP_242267.1| hypothetical protein XC_1177 [Xanthomonas campestris pv. campestris str. 8004]
>gi|66767507|ref|YP_242269.1| hypothetical protein XC_1179 [Xanthomonas campestris pv. campestris str. 8004]
>gi|66767515|ref|YP_242277.1| PilL protein [Xanthomonas campestris pv. campestris str. 8004]
>gi|66767519|ref|YP_242281.1| antifreeze glycopeptide AFGP related protein [Xanthomonas campestris pv. campestris str. 8004]
>gi|66767528|ref|YP_242290.1| RebB protein [Xanthomonas campestris pv. campestris str. 8004]
>gi|66767556|ref|YP_242318.1| hypothetical protein XC_1229 [Xanthomonas campestris pv. campestris str. 8004]
>gi|66767557|ref|YP_242319.1| hypothetical protein XC_1230 [Xanthomonas campestris pv. campestris str. 8004]
>gi|66767558|ref|YP_242320.1| hypothetical protein XC_1231 [Xanthomonas campestris pv. campestris str. 8004]
>gi|66767560|ref|YP_242322.1| glycine cleavage H protein [Xanthomonas campestris pv. campestris str. 8004]
>gi|66767561|ref|YP_242323.1| histone H1 [Xanthomonas campestris pv. campestris str. 8004]
>gi|66767594|ref|YP_242356.1| pyridoxamine 5'-phosphate oxidase [Xanthomonas campestris pv. campestris str. 8004]
>gi|66767603|ref|YP_242365.1| hypothetical protein XC_1276 [Xanthomonas campestris pv. campestris str. 8004]
>gi|66767606|ref|YP_242368.1| MFS transporter [Xanthomonas campestris pv. campestris str. 8004]
>gi|66767608|ref|YP_242370.1| hypothetical protein XC_1281 [Xanthomonas campestris pv. campestris str. 8004]
>gi|66767616|ref|YP_242378.1| hypothetical protein XC_1289 [Xanthomonas campestris pv. campestris str. 8004]
>gi|66767643|ref|YP_242405.1| polysialic acid capsule expression protein [Xanthomonas campestris pv. campestris str. 8004]
>gi|66767648|ref|YP_242410.1| hypothetical protein XC_1321 [Xanthomonas campestris pv. campestris str. 8004]

>gi|66767656|ref|YP_242418.1| hypothetical protein XC_1329 [Xanthomonas campestris pv. campestris str. 8004]
>gi|66767657|ref|YP_242419.1| hypothetical protein XC_1330 [Xanthomonas campestris pv. campestris str. 8004]
>gi|66767666|ref|YP_242428.1| hypothetical protein XC_1339 [Xanthomonas campestris pv. campestris str. 8004]
>gi|66767679|ref|YP_242441.1| hypothetical protein XC_1352 [Xanthomonas campestris pv. campestris str. 8004]
>gi|66767682|ref|YP_242444.1| histone-like protein [Xanthomonas campestris pv. campestris str. 8004]
>gi|66767691|ref|YP_242453.1| hypothetical protein XC_1364 [Xanthomonas campestris pv. campestris str. 8004]
>gi|66767696|ref|YP_242458.1| hypothetical protein XC_1369 [Xanthomonas campestris pv. campestris str. 8004]
>gi|66767698|ref|YP_242460.1| hypothetical protein XC_1371 [Xanthomonas campestris pv. campestris str. 8004]
>gi|66767700|ref|YP_242462.1| hypothetical protein XC_1373 [Xanthomonas campestris pv. campestris str. 8004]
>gi|66767705|ref|YP_242467.1| single-stranded DNA binding protein [Xanthomonas campestris pv. campestris str. 8004]
>gi|66767712|ref|YP_242474.1| oxidoreductase [Xanthomonas campestris pv. campestris str. 8004]
>gi|66767716|ref|YP_242478.1| hypothetical protein XC_1389 [Xanthomonas campestris pv. campestris str. 8004]
>gi|66767731|ref|YP_242493.1| hypothetical protein XC_1405 [Xanthomonas campestris pv. campestris str. 8004]
>gi|66767746|ref|YP_242508.1| hypothetical protein XC_1420 [Xanthomonas campestris pv. campestris str. 8004]
>gi|66767747|ref|YP_242509.1| two-component system sensor protein [Xanthomonas campestris pv. campestris str. 8004]
>gi|66767751|ref|YP_242513.1| hypothetical protein XC_1425 [Xanthomonas campestris pv. campestris str. 8004]
>gi|66767766|ref|YP_242528.1| hypothetical protein XC_1440 [Xanthomonas campestris pv. campestris str. 8004]
>gi|66767770|ref|YP_242532.1| MFS transporter [Xanthomonas campestris pv. campestris str. 8004]
>gi|66767772|ref|YP_242534.1| oxidoreductase [Xanthomonas campestris pv. campestris str. 8004]
>gi|66767774|ref|YP_242536.1| hypothetical protein XC_1448 [Xanthomonas campestris pv. campestris str. 8004]
>gi|66767781|ref|YP_242543.1| cytochrome D oxidase subunit B [Xanthomonas campestris pv. campestris str. 8004]
>gi|66767782|ref|YP_242544.1| transmembrane transport protein [Xanthomonas campestris pv. campestris str. 8004]
>gi|66767786|ref|YP_242548.1| hypothetical protein XC_1460 [Xanthomonas campestris pv. campestris str. 8004]
>gi|66767787|ref|YP_242549.1| hypothetical protein XC_1461 [Xanthomonas campestris pv. campestris str. 8004]
>gi|66767794|ref|YP_242556.1| hypothetical protein XC_1468 [Xanthomonas campestris pv. campestris str. 8004]
>gi|66767812|ref|YP_242574.1| hypothetical protein XC_1486 [Xanthomonas campestris pv. campestris str. 8004]
>gi|66767819|ref|YP_242581.1| hypothetical protein XC_1493 [Xanthomonas campestris pv. campestris str. 8004]
>gi|66767822|ref|YP_242584.1| hypothetical protein XC_1496 [Xanthomonas campestris pv. campestris str. 8004]
>gi|66767830|ref|YP_242592.1| TonB-like protein [Xanthomonas campestris pv. campestris str. 8004]
>gi|66767844|ref|YP_242606.1| cell cycle protein [Xanthomonas campestris pv. campestris str. 8004]
>gi|66767848|ref|YP_242610.1| hypothetical protein XC_1522 [Xanthomonas campestris pv. campestris str. 8004]
>gi|66767858|ref|YP_242620.1| hypothetical protein XC_1534 [Xanthomonas campestris pv. campestris str. 8004]
>gi|66767859|ref|YP_242621.1| lipoprotein [Xanthomonas campestris pv. campestris str. 8004]
>gi|66767861|ref|YP_242623.1| transport protein [Xanthomonas campestris pv. campestris str. 8004]
>gi|66767864|ref|YP_242626.1| methylated-DNA-protein-cysteine S-methyltransferase related protein [Xanthomonas campestris pv. campestris str. 8004]
>gi|66767873|ref|YP_242635.1| hypothetical protein XC_1549 [Xanthomonas campestris pv. campestris str. 8004]
>gi|66767882|ref|YP_242644.1| hypothetical protein XC_1558 [Xanthomonas campestris pv. campestris str. 8004]
>gi|66767890|ref|YP_242652.1| hypothetical protein XC_1566 [Xanthomonas campestris pv. campestris str. 8004]
>gi|66767894|ref|YP_242656.1| FimV protein [Xanthomonas campestris pv. campestris str. 8004]
>gi|66767900|ref|YP_242662.1| hypothetical protein XC_1576 [Xanthomonas campestris pv. campestris str. 8004]
>gi|66767901|ref|YP_242663.1| tryptophan synthase alpha chain [Xanthomonas campestris pv. campestris str. 8004]
>gi|66767912|ref|YP_242674.1| protein-export membrane protein [Xanthomonas campestris pv. campestris str. 8004]
>gi|66767922|ref|YP_242684.1| NADH-ubiquinone oxidoreductase NQO10 subunit [Xanthomonas campestris pv. campestris str. 8004]
>gi|66767941|ref|YP_242703.1| short chain dehydrogenase [Xanthomonas campestris pv. campestris str. 8004]
>gi|66767955|ref|YP_242717.1| hypothetical protein XC_1631 [Xanthomonas campestris pv. campestris str. 8004]
>gi|66767956|ref|YP_242718.1| VirB8 protein [Xanthomonas campestris pv. campestris str. 8004]
>gi|66767999|ref|YP_242761.1| hypothetical protein XC_1675 [Xanthomonas campestris pv. campestris str. 8004]
>gi|66768007|ref|YP_242769.1| hypothetical protein XC_1683 [Xanthomonas campestris pv. campestris str. 8004]
>gi|66768019|ref|YP_242781.1| cell division protein [Xanthomonas campestris pv. campestris str. 8004]
>gi|66768024|ref|YP_242786.1| oxidoreductase [Xanthomonas campestris pv. campestris str. 8004]
>gi|66768026|ref|YP_242788.1| ketoglutarate semialdehyde dehydrogenase [Xanthomonas campestris pv. campestris str. 8004]
>gi|66768033|ref|YP_242795.1| hypothetical protein XC_1709 [Xanthomonas campestris pv. campestris str. 8004]
>gi|66768065|ref|YP_242827.1| PTS system,fructose-specificIIBC component [Xanthomonas campestris pv. campestris str. 8004]
>gi|66768066|ref|YP_242828.1| 1-phosphofructokinase [Xanthomonas campestris pv. campestris str. 8004]
>gi|66768070|ref|YP_242832.1| multidrug resistance protein [Xanthomonas campestris pv. campestris str. 8004]
>gi|66768079|ref|YP_242841.1| hypothetical protein XC_1758 [Xanthomonas campestris pv. campestris str. 8004]
>gi|66768094|ref|YP_242856.1| transport protein [Xanthomonas campestris pv. campestris str. 8004]
>gi|66768096|ref|YP_242858.1| outer membrane protein OprN precursor [Xanthomonas campestris pv. campestris str. 8004]
>gi|66768111|ref|YP_242873.1| repressor [Xanthomonas campestris pv. campestris str. 8004]
>gi|66768112|ref|YP_242874.1| soluble lytic murein transglycosylase [Xanthomonas campestris pv. campestris str. 8004]
>gi|66768133|ref|YP_242895.1| exodeoxyribonuclease VII large subunit [Xanthomonas campestris pv. campestris str. 8004]
>gi|66768137|ref|YP_242899.1| N-acetylmuramoyl-L-alanine amidase [Xanthomonas campestris pv. campestris str. 8004]
>gi|66768156|ref|YP_242918.1| hypothetical protein XC_1835 [Xanthomonas campestris pv. campestris str. 8004]
>gi|66768160|ref|YP_242922.1| tRNA/rRNA methyltransferase [Xanthomonas campestris pv. campestris str. 8004]
>gi|66768165|ref|YP_242927.1| hypothetical protein XC_1844 [Xanthomonas campestris pv. campestris str. 8004]
>gi|66768169|ref|YP_242931.1| hypothetical protein XC_1848 [Xanthomonas campestris pv. campestris str. 8004]
>gi|66768181|ref|YP_242943.1| hypothetical protein XC_1860 [Xanthomonas campestris pv. campestris str. 8004]
>gi|66768183|ref|YP_242945.1| hypothetical protein XC_1862 [Xanthomonas campestris pv. campestris str. 8004]
>gi|66768195|ref|YP_242957.1| amino-acid acetyltransferase [Xanthomonas campestris pv. campestris str. 8004]

>gi|66768201|ref|YP_242963.1| gamma-glutamyl phosphate reductase [Xanthomonas campestris pv. campestris str. 8004]
>gi|66768203|ref|YP_242965.1| MFS transporter [Xanthomonas campestris pv. campestris str. 8004]
>gi|66768205|ref|YP_242967.1| cytochrome D ubiquinol oxidase subunit II [Xanthomonas campestris pv. campestris str. 8004]
>gi|66768207|ref|YP_242969.1| transport protein [Xanthomonas campestris pv. campestris str. 8004]
>gi|66768208|ref|YP_242970.1| ABC transporter ATP-binding protein [Xanthomonas campestris pv. campestris str. 8004]
>gi|66768214|ref|YP_242976.1| c-type cytochrome biogenesis membrane protein [Xanthomonas campestris pv. campestris str. 8004]
>gi|66768218|ref|YP_242980.1| ABC transporter heme permease [Xanthomonas campestris pv. campestris str. 8004]
>gi|66768225|ref|YP_242987.1| pseudouridylate synthase [Xanthomonas campestris pv. campestris str. 8004]
>gi|66768226|ref|YP_242988.1| hypothetical protein XC_1905 [Xanthomonas campestris pv. campestris str. 8004]
>gi|66768239|ref|YP_243001.1| phosphinothricin N-acetyltransferase [Xanthomonas campestris pv. campestris str. 8004]
>gi|66768257|ref|YP_243019.1| hypothetical protein XC_1936 [Xanthomonas campestris pv. campestris str. 8004]
>gi|66768263|ref|YP_243025.1| hypothetical protein XC_1942 [Xanthomonas campestris pv. campestris str. 8004]
>gi|66768265|ref|YP_243027.1| MFS transporter [Xanthomonas campestris pv. campestris str. 8004]
>gi|66768268|ref|YP_243030.1| manganese transport protein [Xanthomonas campestris pv. campestris str. 8004]
>gi|66768277|ref|YP_243039.1| D-beta-hydroxybutyrate dehydrogenase [Xanthomonas campestris pv. campestris str. 8004]
>gi|66768279|ref|YP_243041.1| PHA synthase subunit [Xanthomonas campestris pv. campestris str. 8004]
>gi|66768300|ref|YP_243062.1| hypothetical protein XC_1980 [Xanthomonas campestris pv. campestris str. 8004]
>gi|66768313|ref|YP_243075.1| DNA uptake/competence protein [Xanthomonas campestris pv. campestris str. 8004]
>gi|66768324|ref|YP_243086.1| cointegrate resolution protein T [Xanthomonas campestris pv. campestris str. 8004]
>gi|66768342|ref|YP_243104.1| hypothetical protein XC_2023 [Xanthomonas campestris pv. campestris str. 8004]
>gi|66768412|ref|YP_243174.1| ribonuclease E [Xanthomonas campestris pv. campestris str. 8004]
>gi|66768414|ref|YP_243176.1| hypothetical protein XC_2100 [Xanthomonas campestris pv. campestris str. 8004]
>gi|66768415|ref|YP_243177.1| hypothetical protein XC_2101 [Xanthomonas campestris pv. campestris str. 8004]
>gi|66768418|ref|YP_243180.1| hypothetical protein XC_2104 [Xanthomonas campestris pv. campestris str. 8004]
>gi|66768426|ref|YP_243188.1| adsorption protein [Xanthomonas campestris pv. campestris str. 8004]
>gi|66768429|ref|YP_243191.1| hypothetical protein XC_2115 [Xanthomonas campestris pv. campestris str. 8004]
>gi|66768433|ref|YP_243195.1| adsorption protein [Xanthomonas campestris pv. campestris str. 8004]
>gi|66768439|ref|YP_243201.1| hypothetical protein XC_2126 [Xanthomonas campestris pv. campestris str. 8004]
>gi|66768440|ref|YP_243202.1| 18.2K protein [Xanthomonas campestris pv. campestris str. 8004]
>gi|66768445|ref|YP_243207.1| hypothetical protein XC_2131 [Xanthomonas campestris pv. campestris str. 8004]
>gi|66768457|ref|YP_243219.1| transport protein [Xanthomonas campestris pv. campestris str. 8004]
>gi|66768458|ref|YP_243220.1| hypothetical protein XC_2144 [Xanthomonas campestris pv. campestris str. 8004]
>gi|66768460|ref|YP_243222.1| hypothetical protein XC_2146 [Xanthomonas campestris pv. campestris str. 8004]
>gi|66768471|ref|YP_243233.1| transcriptional regulator [Xanthomonas campestris pv. campestris str. 8004]
>gi|66768473|ref|YP_243235.1| serine protease [Xanthomonas campestris pv. campestris str. 8004]
>gi|66768474|ref|YP_243236.1| YapH protein [Xanthomonas campestris pv. campestris str. 8004]
>gi|66768487|ref|YP_243249.1| exopolysaccharide biosynthesis protein [Xanthomonas campestris pv. campestris str. 8004]
>gi|66768488|ref|YP_243250.1| uroporphyrin-III C-methyltransferase [Xanthomonas campestris pv. campestris str. 8004]
>gi|66768492|ref|YP_243254.1| nitrate transporter [Xanthomonas campestris pv. campestris str. 8004]
>gi|66768500|ref|YP_243262.1| exodeoxyribonuclease III [Xanthomonas campestris pv. campestris str. 8004]
>gi|66768504|ref|YP_243266.1| hypothetical protein XC_2190 [Xanthomonas campestris pv. campestris str. 8004]
>gi|66768537|ref|YP_243299.1| hypothetical protein XC_2224 [Xanthomonas campestris pv. campestris str. 8004]
>gi|66768538|ref|YP_243300.1| hypothetical protein XC_2225 [Xanthomonas campestris pv. campestris str. 8004]
>gi|66768555|ref|YP_243317.1| flagellar protein [Xanthomonas campestris pv. campestris str. 8004]
>gi|66768558|ref|YP_243320.1| flagellar protein [Xanthomonas campestris pv. campestris str. 8004]
>gi|66768562|ref|YP_243324.1| hypothetical protein XC_2249 [Xanthomonas campestris pv. campestris str. 8004]
>gi|66768564|ref|YP_243326.1| RNA polymerase sigma-54 factor [Xanthomonas campestris pv. campestris str. 8004]
>gi|66768578|ref|YP_243340.1| flagellar protein [Xanthomonas campestris pv. campestris str. 8004]
>gi|66768582|ref|YP_243344.1| flagellar protein [Xanthomonas campestris pv. campestris str. 8004]
>gi|66768583|ref|YP_243345.1| flagellar biosynthetic protein [Xanthomonas campestris pv. campestris str. 8004]
>gi|66768584|ref|YP_243346.1| flagellar biosynthesis [Xanthomonas campestris pv. campestris str. 8004]
>gi|66768585|ref|YP_243347.1| flagellar biosynthetic protein [Xanthomonas campestris pv. campestris str. 8004]
>gi|66768591|ref|YP_243353.1| flagellar biosynthetic protein [Xanthomonas campestris pv. campestris str. 8004]
>gi|66768599|ref|YP_243361.1| hypothetical protein XC_2287 [Xanthomonas campestris pv. campestris str. 8004]
>gi|66768603|ref|YP_243365.1| hypothetical protein XC_2291 [Xanthomonas campestris pv. campestris str. 8004]
>gi|66768612|ref|YP_243374.1| chemotaxis protein [Xanthomonas campestris pv. campestris str. 8004]
>gi|66768617|ref|YP_243379.1| hypothetical protein XC_2305 [Xanthomonas campestris pv. campestris str. 8004]
>gi|66768625|ref|YP_243387.1| chemotaxis protein [Xanthomonas campestris pv. campestris str. 8004]
>gi|66768638|ref|YP_243400.1| hypothetical protein XC_2327 [Xanthomonas campestris pv. campestris str. 8004]
>gi|66768650|ref|YP_243412.1| inner membrane protein [Xanthomonas campestris pv. campestris str. 8004]
>gi|66768652|ref|YP_243414.1| inner membrane protein [Xanthomonas campestris pv. campestris str. 8004]
>gi|66768655|ref|YP_243417.1| regulatory protein rpfE [Xanthomonas campestris pv. campestris str. 8004]
>gi|66768667|ref|YP_243429.1| enoyl-CoA hydratase [Xanthomonas campestris pv. campestris str. 8004]
>gi|66768669|ref|YP_243431.1| 3-hydroxyacyl-CoA dehydrogenase type II [Xanthomonas campestris pv. campestris str. 8004]
>gi|66768671|ref|YP_243433.1| hypothetical protein XC_2360 [Xanthomonas campestris pv. campestris str. 8004]
>gi|66768673|ref|YP_243435.1| hypothetical protein XC_2362 [Xanthomonas campestris pv. campestris str. 8004]
>gi|66768695|ref|YP_243457.1| fumarate and nitrate reduction regulatory protein [Xanthomonas campestris pv. campestris str. 8004]
>gi|66768696|ref|YP_243458.1| hypothetical protein XC_2385 [Xanthomonas campestris pv. campestris str. 8004]
>gi|66768699|ref|YP_243461.1| homoserine kinase [Xanthomonas campestris pv. campestris str. 8004]
>gi|66768706|ref|YP_243468.1| filamentous haemagglutinin [Xanthomonas campestris pv. campestris str. 8004]

>gi|66768713|ref|YP_243475.1| putative transport transmembrane protein [Xanthomonas campestris pv. campestris str. 8004]
>gi|66768716|ref|YP_243478.1| hydroxyproline-rich glycoprotein DZ-HRGP [Xanthomonas campestris pv. campestris str. 8004]
>gi|66768722|ref|YP_243484.1| hypothetical protein XC_2414 [Xanthomonas campestris pv. campestris str. 8004]
>gi|66768750|ref|YP_243512.1| hypothetical protein XC_2442 [Xanthomonas campestris pv. campestris str. 8004]
>gi|66768752|ref|YP_243514.1| hypothetical protein XC_2444 [Xanthomonas campestris pv. campestris str. 8004]
>gi|66768761|ref|YP_243523.1| Prop transport protein [Xanthomonas campestris pv. campestris str. 8004]
>gi|66768764|ref|YP_243526.1| two-component system sensor protein [Xanthomonas campestris pv. campestris str. 8004]
>gi|66768780|ref|YP_243542.1| N-acetylmuramoyl-L-alanine amidase [Xanthomonas campestris pv. campestris str. 8004]
>gi|66768809|ref|YP_243571.1| hypothetical protein XC_2501 [Xanthomonas campestris pv. campestris str. 8004]
>gi|66768818|ref|YP_243580.1| LexA [Xanthomonas campestris pv. campestris str. 8004]
>gi|66768825|ref|YP_243587.1| dihydropteroate synthase [Xanthomonas campestris pv. campestris str. 8004]
>gi|66768838|ref|YP_243600.1| hypothetical protein XC_2530 [Xanthomonas campestris pv. campestris str. 8004]
>gi|66768840|ref|YP_243602.1| hypothetical protein XC_2532 [Xanthomonas campestris pv. campestris str. 8004]
>gi|66768843|ref|YP_243605.1| hypothetical protein XC_2535 [Xanthomonas campestris pv. campestris str. 8004]
>gi|66768851|ref|YP_243613.1| ExoD protein [Xanthomonas campestris pv. campestris str. 8004]
>gi|66768854|ref|YP_243616.1| MFS transporter [Xanthomonas campestris pv. campestris str. 8004]
>gi|66768855|ref|YP_243617.1| ABC transporter ATP-binding protein [Xanthomonas campestris pv. campestris str. 8004]
>gi|66768859|ref|YP_243621.1| hexosyltransferase [Xanthomonas campestris pv. campestris str. 8004]
>gi|66768862|ref|YP_243624.1| hypothetical protein XC_2554 [Xanthomonas campestris pv. campestris str. 8004]
>gi|66768867|ref|YP_243629.1| lipopolysaccharide biosynthesis protein [Xanthomonas campestris pv. campestris str. 8004]
>gi|66768870|ref|YP_243632.1| cytochrome like B561 [Xanthomonas campestris pv. campestris str. 8004]
>gi|66768878|ref|YP_243640.1| hypothetical protein XC_2570 [Xanthomonas campestris pv. campestris str. 8004]
>gi|66768883|ref|YP_243645.1| hypothetical protein XC_2575 [Xanthomonas campestris pv. campestris str. 8004]
>gi|66768896|ref|YP_243658.1| glycosyl transferase-related protein [Xanthomonas campestris pv. campestris str. 8004]
>gi|66768913|ref|YP_243675.1| unknown acidic aa rich protein [Xanthomonas campestris pv. campestris str. 8004]
>gi|66768921|ref|YP_243683.1| hypothetical protein XC_2614 [Xanthomonas campestris pv. campestris str. 8004]
>gi|66768938|ref|YP_243700.1| hypothetical protein XC_2631 [Xanthomonas campestris pv. campestris str. 8004]
>gi|66768948|ref|YP_243710.1| hypothetical protein XC_2641 [Xanthomonas campestris pv. campestris str. 8004]
>gi|66768957|ref|YP_243719.1| poly(hydroxyalcanoate) granule associated protein [Xanthomonas campestris pv. campestris str. 8004]
>gi|66768958|ref|YP_243720.1| hypothetical protein XC_2651 [Xanthomonas campestris pv. campestris str. 8004]
>gi|66768961|ref|YP_243723.1| imidazolonepropionase [Xanthomonas campestris pv. campestris str. 8004]
>gi|66768972|ref|YP_243734.1| aminotransferase [Xanthomonas campestris pv. campestris str. 8004]
>gi|66768975|ref|YP_243737.1| hypothetical protein XC_2668 [Xanthomonas campestris pv. campestris str. 8004]
>gi|66768987|ref|YP_243749.1| hypothetical protein XC_2680 [Xanthomonas campestris pv. campestris str. 8004]
>gi|66768997|ref|YP_243759.1| ABC transporter ATP-binding protein [Xanthomonas campestris pv. campestris str. 8004]
>gi|66769008|ref|YP_243770.1| hypothetical protein XC_2701 [Xanthomonas campestris pv. campestris str. 8004]
>gi|66769020|ref|YP_243782.1| hypothetical protein XC_2713 [Xanthomonas campestris pv. campestris str. 8004]
>gi|66769038|ref|YP_243800.1| hypothetical protein XC_2731 [Xanthomonas campestris pv. campestris str. 8004]
>gi|66769039|ref|YP_243801.1| hypothetical protein XC_2732 [Xanthomonas campestris pv. campestris str. 8004]
>gi|66769054|ref|YP_243816.1| hypothetical protein XC_2747 [Xanthomonas campestris pv. campestris str. 8004]
>gi|66769055|ref|YP_243817.1| hypothetical protein XC_2748 [Xanthomonas campestris pv. campestris str. 8004]
>gi|66769069|ref|YP_243831.1| DnaJ protein [Xanthomonas campestris pv. campestris str. 8004]
>gi|66769083|ref|YP_243845.1| hypothetical protein XC_2776 [Xanthomonas campestris pv. campestris str. 8004]
>gi|66769107|ref|YP_243869.1| aklaviketone reductase [Xanthomonas campestris pv. campestris str. 8004]
>gi|66769115|ref|YP_243877.1| hypothetical protein XC_2808 [Xanthomonas campestris pv. campestris str. 8004]
>gi|66769117|ref|YP_243879.1| hypothetical protein XC_2810 [Xanthomonas campestris pv. campestris str. 8004]
>gi|66769122|ref|YP_243884.1| peptidoglycan-associated outer membrane lipoprotein [Xanthomonas campestris pv. campestris str. 8004]
>gi|66769124|ref|YP_243886.1| hypothetical protein XC_2817 [Xanthomonas campestris pv. campestris str. 8004]
>gi|66769135|ref|YP_243897.1| hypothetical protein XC_2828 [Xanthomonas campestris pv. campestris str. 8004]
>gi|66769142|ref|YP_243904.1| hypothetical protein XC_2835 [Xanthomonas campestris pv. campestris str. 8004]
>gi|66769160|ref|YP_243922.1| 2,3,4,5-tetrahydropyridine-2-carboxylate N-succin [Xanthomonas campestris pv. campestris str. 8004]
>gi|66769163|ref|YP_243925.1| protein U [Xanthomonas campestris pv. campestris str. 8004]
>gi|66769167|ref|YP_243929.1| hypothetical protein XC_2861 [Xanthomonas campestris pv. campestris str. 8004]
>gi|66769176|ref|YP_243938.1| phosphatidate cytidylyltransferase [Xanthomonas campestris pv. campestris str. 8004]
>gi|66769177|ref|YP_243939.1| 1-deoxy-D-xylulose 5-phosphate reductoisomerase [Xanthomonas campestris pv. campestris str. 8004]
>gi|66769181|ref|YP_243943.1| UDP-3-O-(R-3-hydroxymyristoyl)-glucosamine N-acyltransferase [Xanthomonas campestris pv. campestris str. 8004]
>gi|66769192|ref|YP_243954.1| sensor protein [Xanthomonas campestris pv. campestris str. 8004]
>gi|66769196|ref|YP_243958.1| hypothetical protein XC_2890 [Xanthomonas campestris pv. campestris str. 8004]
>gi|66769212|ref|YP_243974.1| hypothetical protein XC_2906 [Xanthomonas campestris pv. campestris str. 8004]
>gi|66769217|ref|YP_243979.1| hypothetical protein XC_2911 [Xanthomonas campestris pv. campestris str. 8004]
>gi|66769221|ref|YP_243983.1| hypothetical protein XC_2915 [Xanthomonas campestris pv. campestris str. 8004]
>gi|66769228|ref|YP_243990.1| hypothetical protein XC_2922 [Xanthomonas campestris pv. campestris str. 8004]
>gi|66769238|ref|YP_244000.1| hypothetical protein XC_2932 [Xanthomonas campestris pv. campestris str. 8004]
>gi|66769239|ref|YP_244001.1| hypothetical protein XC_2933 [Xanthomonas campestris pv. campestris str. 8004]
>gi|66769244|ref|YP_244006.1| hypothetical protein XC_2938 [Xanthomonas campestris pv. campestris str. 8004]
>gi|66769250|ref|YP_244012.1| outer membrane protein [Xanthomonas campestris pv. campestris str. 8004]
>gi|66769251|ref|YP_244013.1| hypothetical protein XC_2945 [Xanthomonas campestris pv. campestris str. 8004]
>gi|66769253|ref|YP_244015.1| hypothetical protein XC_2947 [Xanthomonas campestris pv. campestris str. 8004]
>gi|66769256|ref|YP_244018.1| hypothetical protein XC_2950 [Xanthomonas campestris pv. campestris str. 8004]

>gi|66769267|ref|YP_244029.1| polyketide synthase [Xanthomonas campestris pv. campestris str. 8004]
>gi|66769269|ref|YP_244031.1| hypothetical protein XC_2963 [Xanthomonas campestris pv. campestris str. 8004]
>gi|66769272|ref|YP_244034.1| DNA repair protein [Xanthomonas campestris pv. campestris str. 8004]
>gi|66769279|ref|YP_244041.1| regulatory protein [Xanthomonas campestris pv. campestris str. 8004]
>gi|66769311|ref|YP_244073.1| HrpB7 protein [Xanthomonas campestris pv. campestris str. 8004]
>gi|66769313|ref|YP_244075.1| HrpB5 protein [Xanthomonas campestris pv. campestris str. 8004]
>gi|66769315|ref|YP_244077.1| HrcJ protein [Xanthomonas campestris pv. campestris str. 8004]
>gi|66769317|ref|YP_244079.1| HrpB1 protein [Xanthomonas campestris pv. campestris str. 8004]
>gi|66769321|ref|YP_244083.1| HrcQ protein [Xanthomonas campestris pv. campestris str. 8004]
>gi|66769323|ref|YP_244085.1| HrcS protein [Xanthomonas campestris pv. campestris str. 8004]
>gi|66769329|ref|YP_244091.1| HrpW protein [Xanthomonas campestris pv. campestris str. 8004]
>gi|66769332|ref|YP_244094.1| hypothetical protein XC_3026 [Xanthomonas campestris pv. campestris str. 8004]
>gi|66769336|ref|YP_244098.1| isopenicillin N epimerase [Xanthomonas campestris pv. campestris str. 8004]
>gi|66769342|ref|YP_244104.1| hypothetical protein XC_3036 [Xanthomonas campestris pv. campestris str. 8004]
>gi|66769351|ref|YP_244113.1| 2-nitropropane dioxygenase [Xanthomonas campestris pv. campestris str. 8004]
>gi|66769355|ref|YP_244117.1| hypothetical protein XC_3049 [Xanthomonas campestris pv. campestris str. 8004]
>gi|66769373|ref|YP_244135.1| two-component system regulatory protein [Xanthomonas campestris pv. campestris str. 8004]
>gi|66769402|ref|YP_244164.1| pathogenicity-related protein [Xanthomonas campestris pv. campestris str. 8004]
>gi|66769403|ref|YP_244165.1| RNA polymerase sigma factor [Xanthomonas campestris pv. campestris str. 8004]
>gi|66769404|ref|YP_244166.1| hypothetical protein XC_3100 [Xanthomonas campestris pv. campestris str. 8004]
>gi|66769412|ref|YP_244174.1| hypothetical protein XC_3108 [Xanthomonas campestris pv. campestris str. 8004]
>gi|66769417|ref|YP_244179.1| hypothetical protein XC_3113 [Xanthomonas campestris pv. campestris str. 8004]
>gi|66769430|ref|YP_244192.1| hypothetical protein XC_3127 [Xanthomonas campestris pv. campestris str. 8004]
>gi|66769444|ref|YP_244206.1| hypothetical protein XC_3142 [Xanthomonas campestris pv. campestris str. 8004]
>gi|66769453|ref|YP_244215.1| LexA repressor [Xanthomonas campestris pv. campestris str. 8004]
>gi|66769454|ref|YP_244216.1| hypothetical protein XC_3152 [Xanthomonas campestris pv. campestris str. 8004]
>gi|66769478|ref|YP_244240.1| hypothetical protein XC_3176 [Xanthomonas campestris pv. campestris str. 8004]
>gi|66769481|ref|YP_244243.1| hypothetical protein XC_3179 [Xanthomonas campestris pv. campestris str. 8004]
>gi|66769500|ref|YP_244262.1| curved DNA binding protein [Xanthomonas campestris pv. campestris str. 8004]
>gi|66769505|ref|YP_244267.1| hypothetical protein XC_3203 [Xanthomonas campestris pv. campestris str. 8004]
>gi|66769529|ref|YP_244291.1| 3-oxoacyl-[ACP] reductase [Xanthomonas campestris pv. campestris str. 8004]
>gi|66769535|ref|YP_244297.1| hypothetical protein XC_3233 [Xanthomonas campestris pv. campestris str. 8004]
>gi|66769537|ref|YP_244299.1| hypothetical protein XC_3235 [Xanthomonas campestris pv. campestris str. 8004]
>gi|66769540|ref|YP_244302.1| hypothetical protein XC_3238 [Xanthomonas campestris pv. campestris str. 8004]
>gi|66769546|ref|YP_244308.1| DNA polymerase III tau and gamma subunits [Xanthomonas campestris pv. campestris str. 8004]
>gi|66769581|ref|YP_244343.1| hypothetical protein XC_3279 [Xanthomonas campestris pv. campestris str. 8004]
>gi|66769586|ref|YP_244348.1| hypothetical protein XC_3284 [Xanthomonas campestris pv. campestris str. 8004]
>gi|66769587|ref|YP_244349.1| folylpolyglutamate synthase/dihydrofolate synthase [Xanthomonas campestris pv. campestris str. 8004]
>gi|66769589|ref|YP_244351.1| phosphoglycerate mutase [Xanthomonas campestris pv. campestris str. 8004]
>gi|66769601|ref|YP_244363.1| hypothetical protein XC_3299 [Xanthomonas campestris pv. campestris str. 8004]
>gi|66769605|ref|YP_244367.1| hypothetical protein XC_3303 [Xanthomonas campestris pv. campestris str. 8004]
>gi|66769621|ref|YP_244383.1| 30S ribosomal protein S13 [Xanthomonas campestris pv. campestris str. 8004]
>gi|66769623|ref|YP_244385.1| 50S ribosomal protein L15 [Xanthomonas campestris pv. campestris str. 8004]
>gi|66769640|ref|YP_244402.1| 50S ribosomal protein L23 [Xanthomonas campestris pv. campestris str. 8004]
>gi|66769650|ref|YP_244412.1| 50S ribosomal protein L7/L12 [Xanthomonas campestris pv. campestris str. 8004]
>gi|66769663|ref|YP_244425.1| hypothetical protein XC_3361 [Xanthomonas campestris pv. campestris str. 8004]
>gi|66769671|ref|YP_244433.1| thioredoxin [Xanthomonas campestris pv. campestris str. 8004]
>gi|66769674|ref|YP_244436.1| hypothetical protein XC_3372 [Xanthomonas campestris pv. campestris str. 8004]
>gi|66769679|ref|YP_244441.1| extracellular protease [Xanthomonas campestris pv. campestris str. 8004]
>gi|66769681|ref|YP_244443.1| extracellular protease [Xanthomonas campestris pv. campestris str. 8004]
>gi|66769684|ref|YP_244446.1| hypothetical protein XC_3382 [Xanthomonas campestris pv. campestris str. 8004]
>gi|66769686|ref|YP_244448.1| hypothetical protein XC_3384 [Xanthomonas campestris pv. campestris str. 8004]
>gi|66769687|ref|YP_244449.1| hypothetical protein XC_3385 [Xanthomonas campestris pv. campestris str. 8004]
>gi|66769713|ref|YP_244475.1| integral membrane protein [Xanthomonas campestris pv. campestris str. 8004]
>gi|66769749|ref|YP_244511.1| glycine rich protein [Xanthomonas campestris pv. campestris str. 8004]
>gi|66769751|ref|YP_244513.1| diacylglycerol kinase [Xanthomonas campestris pv. campestris str. 8004]
>gi|66769757|ref|YP_244519.1| oxidoreductase [Xanthomonas campestris pv. campestris str. 8004]
>gi|66769764|ref|YP_244526.1| hypothetical protein XC_3462 [Xanthomonas campestris pv. campestris str. 8004]
>gi|66769770|ref|YP_244532.1| ribokinase [Xanthomonas campestris pv. campestris str. 8004]
>gi|66769777|ref|YP_244539.1| hypothetical protein XC_3475 [Xanthomonas campestris pv. campestris str. 8004]
>gi|66769790|ref|YP_244552.1| hypothetical protein XC_3489 [Xanthomonas campestris pv. campestris str. 8004]
>gi|66769793|ref|YP_244555.1| hypothetical protein XC_3492 [Xanthomonas campestris pv. campestris str. 8004]
>gi|66769798|ref|YP_244560.1| hypothetical protein XC_3497 [Xanthomonas campestris pv. campestris str. 8004]
>gi|66769803|ref|YP_244565.1| peptidase [Xanthomonas campestris pv. campestris str. 8004]
>gi|66769811|ref|YP_244573.1| UDP-N-acetylglucosamine-N- acetylmuramyl-(pentapeptide) pyrophosphoryl-undecaprenol N-acetylglucosamine transferase [Xanthomonas campestris pv. campestris str. 8004]
>gi|66769814|ref|YP_244576.1| UDP-N-acetylmuramoylalanyl-D-glutamyl-2, 6-diaminopimelate-D-alanyl-D-alanyl ligase [Xanthomonas campestris pv. campestris str. 8004]
>gi|66769824|ref|YP_244586.1| hypothetical protein XC_3523 [Xanthomonas campestris pv. campestris str. 8004]
>gi|66769825|ref|YP_244587.1| hypothetical protein XC_3524 [Xanthomonas campestris pv. campestris str. 8004]

>gi|66769830|ref|YP_244592.1| two-component system sensor protein [Xanthomonas campestris pv. campestris str. 8004]
>gi|66769832|ref|YP_244594.1| potassium-transporting ATPase B chain [Xanthomonas campestris pv. campestris str. 8004]
>gi|66769843|ref|YP_244605.1| acetyltransferase [Xanthomonas campestris pv. campestris str. 8004]
>gi|66769846|ref|YP_244608.1| hypothetical protein XC_3545 [Xanthomonas campestris pv. campestris str. 8004]
>gi|66769850|ref|YP_244612.1| hypothetical protein XC_3549 [Xanthomonas campestris pv. campestris str. 8004]
>gi|66769852|ref|YP_244614.1| cellulose synthase subunit C [Xanthomonas campestris pv. campestris str. 8004]
>gi|66769855|ref|YP_244617.1| hypothetical protein XC_3554 [Xanthomonas campestris pv. campestris str. 8004]
>gi|66769859|ref|YP_244621.1| hypothetical protein XC_3558 [Xanthomonas campestris pv. campestris str. 8004]
>gi|66769865|ref|YP_244627.1| general secretion pathway protein N [Xanthomonas campestris pv. campestris str. 8004]
>gi|66769867|ref|YP_244629.1| general secretion pathway protein L [Xanthomonas campestris pv. campestris str. 8004]
>gi|66769871|ref|YP_244633.1| general secretion pathway protein H [Xanthomonas campestris pv. campestris str. 8004]
>gi|66769876|ref|YP_244638.1| outer membrane protein [Xanthomonas campestris pv. campestris str. 8004]
>gi|66769893|ref|YP_244655.1| hypothetical protein XC_3593 [Xanthomonas campestris pv. campestris str. 8004]
>gi|66769897|ref|YP_244659.1| DNA-binding protein [Xanthomonas campestris pv. campestris str. 8004]
>gi|66769900|ref|YP_244662.1| ABC transporter ATP-binding protein [Xanthomonas campestris pv. campestris str. 8004]
>gi|66769915|ref|YP_244677.1| electron transfer flavoprotein alpha subunit [Xanthomonas campestris pv. campestris str. 8004]
>gi|66769919|ref|YP_244681.1| acetyl transferase/isomerase [Xanthomonas campestris pv. campestris str. 8004]
>gi|66769937|ref|YP_244699.1| outer membrane protein [Xanthomonas campestris pv. campestris str. 8004]
>gi|66769938|ref|YP_244700.1| outer membrane protein [Xanthomonas campestris pv. campestris str. 8004]
>gi|66769945|ref|YP_244707.1| dethiobiotin synthetase [Xanthomonas campestris pv. campestris str. 8004]
>gi|66769954|ref|YP_244716.1| hypothetical protein XC_3654 [Xanthomonas campestris pv. campestris str. 8004]
>gi|66769956|ref|YP_244718.1| copper resistance protein B precursor [Xanthomonas campestris pv. campestris str. 8004]
>gi|66769967|ref|YP_244729.1| hypothetical protein XC_3668 [Xanthomonas campestris pv. campestris str. 8004]
>gi|66769975|ref|YP_244737.1| chorismate mutase/prephenate dehydratase [Xanthomonas campestris pv. campestris str. 8004]
>gi|66769980|ref|YP_244742.1| ATP synthase delta chain [Xanthomonas campestris pv. campestris str. 8004]
>gi|66769984|ref|YP_244746.1| hypothetical protein XC_3685 [Xanthomonas campestris pv. campestris str. 8004]
>gi|66769987|ref|YP_244749.1| dihydrolipoamide dehydrogenase [Xanthomonas campestris pv. campestris str. 8004]
>gi|66769998|ref|YP_244760.1| ABC transporter permease [Xanthomonas campestris pv. campestris str. 8004]
>gi|66770019|ref|YP_244781.1| hypothetical protein XC_3721 [Xanthomonas campestris pv. campestris str. 8004]
>gi|66770021|ref|YP_244783.1| hypothetical protein XC_3723 [Xanthomonas campestris pv. campestris str. 8004]
>gi|66770023|ref|YP_244785.1| chemotaxis MotB protein [Xanthomonas campestris pv. campestris str. 8004]
>gi|66770024|ref|YP_244786.1| hypothetical protein XC_3726 [Xanthomonas campestris pv. campestris str. 8004]
>gi|66770026|ref|YP_244788.1| hypothetical protein XC_3728 [Xanthomonas campestris pv. campestris str. 8004]
>gi|66770030|ref|YP_244792.1| Na+:H+ antiporter [Xanthomonas campestris pv. campestris str. 8004]
>gi|66770053|ref|YP_244815.1| hypothetical protein XC_3755 [Xanthomonas campestris pv. campestris str. 8004]
>gi|66770056|ref|YP_244818.1| regulatory protein [Xanthomonas campestris pv. campestris str. 8004]
>gi|66770080|ref|YP_244842.1| hypothetical protein XC_3782 [Xanthomonas campestris pv. campestris str. 8004]
>gi|66770082|ref|YP_244844.1| hypothetical protein XC_3784 [Xanthomonas campestris pv. campestris str. 8004]
>gi|66770084|ref|YP_244846.1| hypothetical protein XC_3786 [Xanthomonas campestris pv. campestris str. 8004]
>gi|66770085|ref|YP_244847.1| hypothetical protein XC_3787 [Xanthomonas campestris pv. campestris str. 8004]
>gi|66770092|ref|YP_244854.1| hypothetical protein XC_3794 [Xanthomonas campestris pv. campestris str. 8004]
>gi|66770125|ref|YP_244887.1| hypothetical protein XC_3827 [Xanthomonas campestris pv. campestris str. 8004]
>gi|66770126|ref|YP_244888.1| hypothetical protein XC_3828 [Xanthomonas campestris pv. campestris str. 8004]
>gi|66770132|ref|YP_244894.1| hypothetical protein XC_3834 [Xanthomonas campestris pv. campestris str. 8004]
>gi|66770133|ref|YP_244895.1| hypothetical protein XC_3835 [Xanthomonas campestris pv. campestris str. 8004]
>gi|66770136|ref|YP_244898.1| hypothetical protein XC_3838 [Xanthomonas campestris pv. campestris str. 8004]
>gi|66770144|ref|YP_244906.1| cell division protein [Xanthomonas campestris pv. campestris str. 8004]
>gi|66770146|ref|YP_244908.1| ATP-dependent RNA helicase [Xanthomonas campestris pv. campestris str. 8004]
>gi|66770153|ref|YP_244915.1| hypothetical protein XC_3855 [Xanthomonas campestris pv. campestris str. 8004]
>gi|66770164|ref|YP_244926.1| hypothetical protein XC_3866 [Xanthomonas campestris pv. campestris str. 8004]
>gi|66770165|ref|YP_244927.1| hypothetical protein XC_3867 [Xanthomonas campestris pv. campestris str. 8004]
>gi|66770167|ref|YP_244929.1| hypothetical protein XC_3869 [Xanthomonas campestris pv. campestris str. 8004]
>gi|66770168|ref|YP_244930.1| hypothetical protein XC_3870 [Xanthomonas campestris pv. campestris str. 8004]
>gi|66770173|ref|YP_244935.1| hypothetical protein XC_3875 [Xanthomonas campestris pv. campestris str. 8004]
>gi|66770176|ref|YP_244938.1| hypothetical protein XC_3878 [Xanthomonas campestris pv. campestris str. 8004]
>gi|66770179|ref|YP_244941.1| cationic amino acid transporter [Xanthomonas campestris pv. campestris str. 8004]
>gi|66770180|ref|YP_244942.1| hypothetical protein XC_3882 [Xanthomonas campestris pv. campestris str. 8004]
>gi|66770182|ref|YP_244944.1| membrane protein [Xanthomonas campestris pv. campestris str. 8004]
>gi|66770187|ref|YP_244949.1| 30S ribosomal protein S21 [Xanthomonas campestris pv. campestris str. 8004]
>gi|66770188|ref|YP_244950.1| hypothetical protein XC_3890 [Xanthomonas campestris pv. campestris str. 8004]
>gi|66770195|ref|YP_244957.1| hypothetical protein XC_3897 [Xanthomonas campestris pv. campestris str. 8004]
>gi|66770197|ref|YP_244959.1| hypothetical protein XC_3899 [Xanthomonas campestris pv. campestris str. 8004]
>gi|66770214|ref|YP_244976.1| phage-related baseplate assembly protein [Xanthomonas campestris pv. campestris str. 8004]
>gi|66770231|ref|YP_244993.1| chromosome partitioning protein [Xanthomonas campestris pv. campestris str. 8004]
>gi|66770240|ref|YP_245002.1| DNA/pantothenate metabolism flavoprotein [Xanthomonas campestris pv. campestris str. 8004]
>gi|66770243|ref|YP_245005.1| hypothetical protein XC_3946 [Xanthomonas campestris pv. campestris str. 8004]
>gi|66770246|ref|YP_245008.1| acyltransferase [Xanthomonas campestris pv. campestris str. 8004]
>gi|66770262|ref|YP_245024.1| oxidoreductase [Xanthomonas campestris pv. campestris str. 8004]
>gi|66770268|ref|YP_245030.1| hypothetical protein XC_3971 [Xanthomonas campestris pv. campestris str. 8004]
>gi|66770274|ref|YP_245036.1| hypothetical protein XC_3977 [Xanthomonas campestris pv. campestris str. 8004]

>gi|66770277|ref|YP_245039.1| hypothetical protein XC_3980 [Xanthomonas campestris pv. campestris str. 8004]
>gi|66770280|ref|YP_245042.1| hypothetical protein XC_3983 [Xanthomonas campestris pv. campestris str. 8004]
>gi|66770282|ref|YP_245044.1| histone H1 [Xanthomonas campestris pv. campestris str. 8004]
>gi|66770284|ref|YP_245046.1| hypothetical protein XC_3987 [Xanthomonas campestris pv. campestris str. 8004]
>gi|66770286|ref|YP_245048.1| hypothetical protein XC_3989 [Xanthomonas campestris pv. campestris str. 8004]
>gi|66770291|ref|YP_245053.1| cytochrome B561 [Xanthomonas campestris pv. campestris str. 8004]
>gi|66770295|ref|YP_245057.1| two-component system sensor protein [Xanthomonas campestris pv. campestris str. 8004]
>gi|66770317|ref|YP_245079.1| thymidylate kinase [Xanthomonas campestris pv. campestris str. 8004]
>gi|66770321|ref|YP_245083.1| hypothetical protein XC_4024 [Xanthomonas campestris pv. campestris str. 8004]
>gi|66770323|ref|YP_245085.1| biotin acetyl-CoA-carboxylase synthetase [Xanthomonas campestris pv. campestris str. 8004]
>gi|66770337|ref|YP_245099.1| hypothetical protein XC_4040 [Xanthomonas campestris pv. campestris str. 8004]
>gi|66770340|ref|YP_245102.1| hypothetical protein XC_4043 [Xanthomonas campestris pv. campestris str. 8004]
>gi|66770344|ref|YP_245106.1| hypothetical protein XC_4047 [Xanthomonas campestris pv. campestris str. 8004]
>gi|66770354|ref|YP_245116.1| TonB-like protein [Xanthomonas campestris pv. campestris str. 8004]
>gi|66770359|ref|YP_245121.1| hypothetical protein XC_4062 [Xanthomonas campestris pv. campestris str. 8004]
>gi|66770361|ref|YP_245123.1| heavy metal transporter [Xanthomonas campestris pv. campestris str. 8004]
>gi|66770382|ref|YP_245144.1| ankyrin-like protein [Xanthomonas campestris pv. campestris str. 8004]
>gi|66770385|ref|YP_245147.1| hypothetical protein XC_4088 [Xanthomonas campestris pv. campestris str. 8004]
>gi|66770389|ref|YP_245151.1| 3-oxoacyl-[ACP] reductase [Xanthomonas campestris pv. campestris str. 8004]
>gi|66770391|ref|YP_245153.1| membrane protein [Xanthomonas campestris pv. campestris str. 8004]
>gi|66770392|ref|YP_245154.1| membrane protein [Xanthomonas campestris pv. campestris str. 8004]
>gi|66770403|ref|YP_245165.1| hypothetical protein XC_4106 [Xanthomonas campestris pv. campestris str. 8004]
>gi|66770404|ref|YP_245166.1| hypothetical protein XC_4107 [Xanthomonas campestris pv. campestris str. 8004]
>gi|66770407|ref|YP_245169.1| hypothetical protein XC_4110 [Xanthomonas campestris pv. campestris str. 8004]
>gi|66770411|ref|YP_245173.1| nodulation protein [Xanthomonas campestris pv. campestris str. 8004]
>gi|66770421|ref|YP_245183.1| hypothetical protein XC_4124 [Xanthomonas campestris pv. campestris str. 8004]
>gi|66770424|ref|YP_245186.1| cation efflux system protein [Xanthomonas campestris pv. campestris str. 8004]
>gi|66770425|ref|YP_245187.1| hypothetical protein XC_4128 [Xanthomonas campestris pv. campestris str. 8004]
>gi|66770434|ref|YP_245196.1| phospholipase A1 [Xanthomonas campestris pv. campestris str. 8004]
>gi|66770437|ref|YP_245199.1| hypothetical protein XC_4140 [Xanthomonas campestris pv. campestris str. 8004]
>gi|66770440|ref|YP_245202.1| hypothetical protein XC_4143 [Xanthomonas campestris pv. campestris str. 8004]
>gi|66770452|ref|YP_245214.1| oxidoreductase [Xanthomonas campestris pv. campestris str. 8004]
>gi|66770454|ref|YP_245216.1| oxidoreductase [Xanthomonas campestris pv. campestris str. 8004]
>gi|66770477|ref|YP_245239.1| hypothetical protein XC_4181 [Xanthomonas campestris pv. campestris str. 8004]
>gi|66770479|ref|YP_245241.1| sec-independent protein translocase [Xanthomonas campestris pv. campestris str. 8004]
>gi|66770484|ref|YP_245246.1| hypothetical protein XC_4188 [Xanthomonas campestris pv. campestris str. 8004]
>gi|66770499|ref|YP_245261.1| histone [Xanthomonas campestris pv. campestris str. 8004]
>gi|66770523|ref|YP_245285.1| acid phosphatase [Xanthomonas campestris pv. campestris str. 8004]
>gi|66770524|ref|YP_245286.1| hypothetical protein XC_4228 [Xanthomonas campestris pv. campestris str. 8004]
>gi|66770544|ref|YP_245306.1| hypothetical protein XC_4248 [Xanthomonas campestris pv. campestris str. 8004]
>gi|66770546|ref|YP_245308.1| hypothetical protein XC_4250 [Xanthomonas campestris pv. campestris str. 8004]
>gi|66770551|ref|YP_245313.1| hypothetical protein XC_4255 [Xanthomonas campestris pv. campestris str. 8004]
>gi|66770554|ref|YP_245316.1| hypothetical protein XC_4258 [Xanthomonas campestris pv. campestris str. 8004]
>gi|66770560|ref|YP_245322.1| hypothetical protein XC_4264 [Xanthomonas campestris pv. campestris str. 8004]
>gi|66770562|ref|YP_245324.1| hypothetical protein XC_4266 [Xanthomonas campestris pv. campestris str. 8004]
>gi|66770580|ref|YP_245342.1| exodeoxyribonuclease V alpha chain [Xanthomonas campestris pv. campestris str. 8004]
>gi|66770581|ref|YP_245343.1| exodeoxyribonuclease V beta chain [Xanthomonas campestris pv. campestris str. 8004]
>gi|66770583|ref|YP_245345.1| hemagglutinin [Xanthomonas campestris pv. campestris str. 8004]
>gi|66770585|ref|YP_245347.1| microcystin dependent protein [Xanthomonas campestris pv. campestris str. 8004]
>gi|66770593|ref|YP_245355.1| hypothetical protein XC_4300 [Xanthomonas campestris pv. campestris str. 8004]
>gi|66770594|ref|YP_245356.1| lipoprotein [Xanthomonas campestris pv. campestris str. 8004]
>gi|66770596|ref|YP_245358.1| hypothetical protein XC_4303 [Xanthomonas campestris pv. campestris str. 8004]
>gi|66770601|ref|YP_245363.1| hypothetical protein XC_4308 [Xanthomonas campestris pv. campestris str. 8004]
>gi|66770605|ref|YP_245367.1| hypothetical protein XC_4312 [Xanthomonas campestris pv. campestris str. 8004]
>gi|66770608|ref|YP_245370.1| glycerate kinase [Xanthomonas campestris pv. campestris str. 8004]
>gi|66770609|ref|YP_245371.1| MFS transporter [Xanthomonas campestris pv. campestris str. 8004]
>gi|66770615|ref|YP_245377.1| hypothetical protein XC_4322 [Xanthomonas campestris pv. campestris str. 8004]
>gi|54295860|ref|YP_122172.1| hypothetical protein plpp0017 [Legionella pneumophila str. Paris]
>gi|54295862|ref|YP_122174.1| hypothetical protein plpp0019 [Legionella pneumophila str. Paris]
>gi|54295866|ref|YP_122178.1| hypothetical protein plpp0023 [Legionella pneumophila str. Paris]
>gi|54296006|ref|YP_122375.1| hypothetical protein lpp0023 [Legionella pneumophila str. Paris]
>gi|54296017|ref|YP_122386.1| hypothetical protein lpp0034 [Legionella pneumophila str. Paris]
>gi|54296051|ref|YP_122420.1| hypothetical protein lpp0069 [Legionella pneumophila str. Paris]
>gi|54296056|ref|YP_122425.1| hypothetical protein lpp0074 [Legionella pneumophila str. Paris]
>gi|54296201|ref|YP_122570.1| hypothetical protein lpp0227 [Legionella pneumophila str. Paris]
>gi|54296203|ref|YP_122572.1| hypothetical protein lpp0229 [Legionella pneumophila str. Paris]
>gi|54296246|ref|YP_122615.1| hypothetical protein lpp0273 [Legionella pneumophila str. Paris]
>gi|54296357|ref|YP_122726.1| 50S ribosomal subunit protein L7/L12 [Legionella pneumophila str. Paris]
>gi|54296367|ref|YP_122736.1| 50S ribosomal subunit protein L23 [Legionella pneumophila str. Paris]
>gi|54296476|ref|YP_122845.1| hypothetical protein lpp0507 [Legionella pneumophila str. Paris]

>gi|54296486|ref|YP_122855.1| hypothetical protein lpp0517 [Legionella pneumophila str. Paris]
>gi|54296539|ref|YP_122908.1| hypothetical protein lpp0570 [Legionella pneumophila str. Paris]
>gi|54296540|ref|YP_122909.1| UDP-3-O-[3-hydroxymyristoyl] glucosamine N-acyltransferase [Legionella pneumophila str. Paris]
>gi|54296613|ref|YP_122982.1| hypothetical protein lpp0644 [Legionella pneumophila str. Paris]
>gi|54296633|ref|YP_123002.1| hypothetical protein lpp0664 [Legionella pneumophila str. Paris]
>gi|54296691|ref|YP_123060.1| hypothetical protein lpp0722 [Legionella pneumophila str. Paris]
>gi|54296711|ref|YP_123080.1| 10 kDa chaperonin (Protein Cpn10) (groES protein) (Heat shock protein A) [Legionella pneumophila str. Paris]
>gi|54296732|ref|YP_123101.1| hypothetical protein lpp0763 [Legionella pneumophila str. Paris]
>gi|54296740|ref|YP_123109.1| hypothetical protein lpp0779 [Legionella pneumophila str. Paris]
>gi|54296750|ref|YP_123119.1| hypothetical protein lpp0789 [Legionella pneumophila str. Paris]
>gi|54296766|ref|YP_123135.1| hypothetical protein lpp0805 [Legionella pneumophila str. Paris]
>gi|54296781|ref|YP_123150.1| hypothetical protein lpp0820 [Legionella pneumophila str. Paris]
>gi|54296834|ref|YP_123203.1| Rod shape-determining protein MreD [Legionella pneumophila str. Paris]
>gi|54296845|ref|YP_123214.1| hypothetical protein lpp0886 [Legionella pneumophila str. Paris]
>gi|54296875|ref|YP_123244.1| hypothetical protein lpp0916 [Legionella pneumophila str. Paris]
>gi|54296878|ref|YP_123247.1| heme exporter protein CcmB [Legionella pneumophila str. Paris]
>gi|54296903|ref|YP_123272.1| hypothetical protein lpp0944 [Legionella pneumophila str. Paris]
>gi|54297023|ref|YP_123392.1| hypothetical protein lpp1065 [Legionella pneumophila str. Paris]
>gi|54297027|ref|YP_123396.1| hypothetical protein lpp1069 [Legionella pneumophila str. Paris]
>gi|54297052|ref|YP_123421.1| hypothetical protein lpp1094 [Legionella pneumophila str. Paris]
>gi|54297054|ref|YP_123423.1| hypothetical protein lpp1096 [Legionella pneumophila str. Paris]
>gi|54297101|ref|YP_123470.1| hypothetical protein lpp1146 [Legionella pneumophila str. Paris]
>gi|54297106|ref|YP_123475.1| hypothetical protein lpp1151 [Legionella pneumophila str. Paris]
>gi|54297168|ref|YP_123537.1| hypothetical protein lpp1213 [Legionella pneumophila str. Paris]
>gi|54297218|ref|YP_123587.1| FimV protein [Legionella pneumophila str. Paris]
>gi|54297249|ref|YP_123618.1| flagelline [Legionella pneumophila str. Paris]
>gi|54297338|ref|YP_123707.1| hypothetical protein lpp1383 [Legionella pneumophila str. Paris]
>gi|54297485|ref|YP_123854.1| hypothetical protein lpp1530 [Legionella pneumophila str. Paris]
>gi|54297601|ref|YP_123970.1| hypothetical protein lpp1652 [Legionella pneumophila str. Paris]
>gi|54297629|ref|YP_123998.1| hypothetical protein lpp1680 [Legionella pneumophila str. Paris]
>gi|54297655|ref|YP_124024.1| hypothetical protein lpp1706 [Legionella pneumophila str. Paris]
>gi|54297689|ref|YP_124058.1| hypothetical protein lpp1740 [Legionella pneumophila str. Paris]
>gi|54297701|ref|YP_124070.1| Flagellar biosynthetic protein FliQ [Legionella pneumophila str. Paris]
>gi|54297703|ref|YP_124072.1| Flagellar protein fliO [Legionella pneumophila str. Paris]
>gi|54297710|ref|YP_124079.1| hypothetical protein lpp1761 [Legionella pneumophila str. Paris]
>gi|54297711|ref|YP_124080.1| hypothetical protein lpp1762 [Legionella pneumophila str. Paris]
>gi|54297803|ref|YP_124172.1| hypothetical protein lpp1854 [Legionella pneumophila str. Paris]
>gi|54297838|ref|YP_124207.1| hypothetical protein lpp1890 [Legionella pneumophila str. Paris]
>gi|54297917|ref|YP_124286.1| hypothetical protein lpp1972 [Legionella pneumophila str. Paris]
>gi|54297977|ref|YP_124346.1| hypothetical protein lpp2032 [Legionella pneumophila str. Paris]
>gi|54298013|ref|YP_124382.1| hypothetical protein lpp2070 [Legionella pneumophila str. Paris]
>gi|54298087|ref|YP_124456.1| hypothetical protein lpp2144 [Legionella pneumophila str. Paris]
>gi|54298152|ref|YP_124521.1| hypothetical protein lpp2209 [Legionella pneumophila str. Paris]
>gi|54298205|ref|YP_124574.1| hypothetical protein lpp2263 [Legionella pneumophila str. Paris]
>gi|54298217|ref|YP_124586.1| hypothetical protein lpp2275 [Legionella pneumophila str. Paris]
>gi|54298269|ref|YP_124638.1| hypothetical protein lpp2327 [Legionella pneumophila str. Paris]
>gi|54298284|ref|YP_124653.1| hypothetical protein lpp2342 [Legionella pneumophila str. Paris]
>gi|54298285|ref|YP_124654.1| hypothetical protein lpp2343 [Legionella pneumophila str. Paris]
>gi|54298299|ref|YP_124668.1| hypothetical protein lpp2357 [Legionella pneumophila str. Paris]
>gi|54298346|ref|YP_124715.1| hypothetical protein lpp2405 [Legionella pneumophila str. Paris]
>gi|54298384|ref|YP_124753.1| hypothetical protein lpp2448 [Legionella pneumophila str. Paris]
>gi|54298451|ref|YP_124820.1| hypothetical protein lpp2515 [Legionella pneumophila str. Paris]
>gi|54298491|ref|YP_124860.1| effector protein B, substrate of the Dot/Icm secretion system [Legionella pneumophila str. Paris]
>gi|54298496|ref|YP_124865.1| hypothetical protein lpp2560 [Legionella pneumophila str. Paris]
>gi|54298525|ref|YP_124894.1| hypothetical protein lpp2589 [Legionella pneumophila str. Paris]
>gi|54298572|ref|YP_124941.1| hypothetical protein lpp2636 [Legionella pneumophila str. Paris]
>gi|54298625|ref|YP_124994.1| 30S ribosomal subunit protein S20 [Legionella pneumophila str. Paris]
>gi|54298633|ref|YP_125002.1| hypothetical protein lpp2697 [Legionella pneumophila str. Paris]
>gi|54298644|ref|YP_125013.1| hypothetical protein lpp2708 [Legionella pneumophila str. Paris]
>gi|54298726|ref|YP_125095.1| hypothetical protein lpp2790 [Legionella pneumophila str. Paris]
>gi|54298745|ref|YP_125114.1| hypothetical protein lpp2809 [Legionella pneumophila str. Paris]
>gi|54298763|ref|YP_125132.1| NADH-quinone oxidoreductase chain J [Legionella pneumophila str. Paris]
>gi|54298836|ref|YP_125205.1| hypothetical protein lpp2903 [Legionella pneumophila str. Paris]
>gi|54298845|ref|YP_125214.1| hypothetical protein lpp2912 [Legionella pneumophila str. Paris]
>gi|54298876|ref|YP_125245.1| hypothetical protein lpp2943 [Legionella pneumophila str. Paris]
>gi|54298877|ref|YP_125246.1| hypothetical protein lpp2944 [Legionella pneumophila str. Paris]
>gi|54298911|ref|YP_125280.1| hypothetical protein lpp2978 [Legionella pneumophila str. Paris]
>gi|54298916|ref|YP_125285.1| hypothetical protein lpp2983 [Legionella pneumophila str. Paris]
>gi|54298985|ref|YP_125354.1| hypothetical protein lpp3052 [Legionella pneumophila str. Paris]
>gi|54298990|ref|YP_125359.1| hypothetical protein lpp3057 [Legionella pneumophila str. Paris]

>gi|33356657|ref|NP_877597.1| hypothetical protein PAB0004.1n [Pyrococcus abyssi GE5]
>gi|14520273|ref|NP_125748.1| hypothetical protein PAB2311 [Pyrococcus abyssi GE5]
>gi|14520290|ref|NP_125765.1| hypothetical protein PAB0037 [Pyrococcus abyssi GE5]
>gi|14520305|ref|NP_125780.1| hypothetical protein PAB2287 [Pyrococcus abyssi GE5]
>gi|14520313|ref|NP_125788.1| hypothetical protein PAB0055 [Pyrococcus abyssi GE5]
>gi|14520349|ref|NP_125824.1| hypothetical protein PAB0081 [Pyrococcus abyssi GE5]
>gi|14520358|ref|NP_125833.1| hypothetical protein PAB0089 [Pyrococcus abyssi GE5]
>gi|14520373|ref|NP_125848.1| biotin synthase, putative. [Pyrococcus abyssi GE5]
>gi|14520378|ref|NP_125853.1| hypothetical protein PAB0107 [Pyrococcus abyssi GE5]
>gi|14520417|ref|NP_125892.1| hypothetical protein PAB0131 [Pyrococcus abyssi GE5]
>gi|33356670|ref|NP_877605.1| hypothetical protein PAB0133.1n [Pyrococcus abyssi GE5]
>gi|14520466|ref|NP_125941.1| lactoylglutathione lyase methylglyoxalase) (aldoketomutase) (glyoxalase i). [Pyrococcus abyssi GE5]
>gi|14520470|ref|NP_125945.1| hypothetical protein PAB2186 [Pyrococcus abyssi GE5]
>gi|33356674|ref|NP_125950.2| hypothetical protein PAB2181 [Pyrococcus abyssi GE5]
>gi|33356676|ref|NP_125975.2| hypothetical protein PAB2166 [Pyrococcus abyssi GE5]
>gi|14520501|ref|NP_125976.1| hypothetical protein PAB2165 [Pyrococcus abyssi GE5]
>gi|14520529|ref|NP_126004.1| polyferredoxin related [Pyrococcus abyssi GE5]
>gi|14520539|ref|NP_126014.1| LSU ribosomal protein L19E [Pyrococcus abyssi GE5]
>gi|14520574|ref|NP_126049.1| hypothetical protein PAB2110 [Pyrococcus abyssi GE5]
>gi|14520575|ref|NP_126050.1| chromosome segregation protein smc1 [Pyrococcus abyssi GE5]
>gi|14520576|ref|NP_126051.1| hypothetical protein PAB0239 [Pyrococcus abyssi GE5]
>gi|14520591|ref|NP_126066.1| adenylyl cyclase related protein [Pyrococcus abyssi GE5]
>gi|14520597|ref|NP_126072.1| hypothetical protein PAB0254 [Pyrococcus abyssi GE5]
>gi|33356683|ref|NP_877610.1| hypothetical protein PAB2407.1n [Pyrococcus abyssi GE5]
>gi|14520607|ref|NP_126082.1| hypothetical protein PAB2408 [Pyrococcus abyssi GE5]
>gi|14520620|ref|NP_126095.1| hypothetical protein PAB3103 [Pyrococcus abyssi GE5]
>gi|33356689|ref|NP_877614.1| hypothetical protein PAB0289.1n [Pyrococcus abyssi GE5]
>gi|33356690|ref|NP_877615.1| hypothetical protein PAB0294.1n [Pyrococcus abyssi GE5]
>gi|14520670|ref|NP_126145.1| ribose ABC transporter, permease protein [Pyrococcus abyssi GE5]
>gi|14520682|ref|NP_126157.1| hypothetical protein PAB2041 [Pyrococcus abyssi GE5]
>gi|14520688|ref|NP_126163.1| archaeal histone A2/archaeal histone A1 [Pyrococcus abyssi GE5]
>gi|14520702|ref|NP_126177.1| hypothetical protein PAB0325 [Pyrococcus abyssi GE5]
>gi|33356697|ref|NP_877621.1| hypothetical protein PAB0328.2n [Pyrococcus abyssi GE5]
>gi|14520718|ref|NP_126193.1| signal recognition particle receptor [Pyrococcus abyssi GE5]
>gi|14520740|ref|NP_126215.1| hypothetical protein PAB2010 [Pyrococcus abyssi GE5]
>gi|14520756|ref|NP_126231.1| hypothetical protein PAB0371 [Pyrococcus abyssi GE5]
>gi|14520762|ref|NP_126237.1| stage ii sporulation protein m related [Pyrococcus abyssi GE5]
>gi|33356699|ref|NP_877623.1| hypothetical protein PAB0385.1n [Pyrococcus abyssi GE5]
>gi|14520787|ref|NP_126262.1| ferripyochelin binding protein [Pyrococcus abyssi GE5]
>gi|14520792|ref|NP_126267.1| hypothetical protein PAB1972 [Pyrococcus abyssi GE5]
>gi|14520840|ref|NP_126315.1| hypothetical protein PAB1946 [Pyrococcus abyssi GE5]
>gi|14520881|ref|NP_126356.1| hypothetical protein PAB1920 [Pyrococcus abyssi GE5]
>gi|14520883|ref|NP_126358.1| SSU ribosomal protein S28E [Pyrococcus abyssi GE5]
>gi|14520896|ref|NP_126371.1| hypothetical protein PAB0470 [Pyrococcus abyssi GE5]
>gi|14520913|ref|NP_126388.1| hypothetical protein PAB3171 [Pyrococcus abyssi GE5]
>gi|14520916|ref|NP_126391.1| long hypothetical protein [Pyrococcus abyssi GE5]
>gi|33356715|ref|NP_877636.1| hypothetical protein PAB1886.2n [Pyrococcus abyssi GE5]
>gi|33356716|ref|NP_126412.2| hypothetical protein PAB3177 [Pyrococcus abyssi GE5]
>gi|14520945|ref|NP_126420.1| hypothetical protein PAB0503 [Pyrococcus abyssi GE5]
>gi|14520949|ref|NP_126424.1| hypothetical protein PAB1876 [Pyrococcus abyssi GE5]
>gi|14520972|ref|NP_126447.1| hypothetical protein PAB1855 [Pyrococcus abyssi GE5]
>gi|33356717|ref|NP_126458.2| hypothetical protein PAB1851 [Pyrococcus abyssi GE5]
>gi|14520986|ref|NP_126461.1| hypothetical protein PAB1848 [Pyrococcus abyssi GE5]
>gi|14520998|ref|NP_126473.1| hypothetical protein PAB0535 [Pyrococcus abyssi GE5]
>gi|14521034|ref|NP_126509.1| hypothetical protein PAB1809 [Pyrococcus abyssi GE5]
>gi|14521036|ref|NP_126511.1| glycine cleavage system protein h [Pyrococcus abyssi GE5]
>gi|14521066|ref|NP_126541.1| LSU ribosomal protein L15E [Pyrococcus abyssi GE5]
>gi|14521080|ref|NP_126555.1| hypothetical protein PAB8218 [Pyrococcus abyssi GE5]
>gi|33356730|ref|NP_877647.1| hypothetical protein PAB0593.1n [Pyrococcus abyssi GE5]
>gi|14521093|ref|NP_126568.1| methylmalonyl-coa decarboxylase gamma chain [Pyrococcus abyssi GE5]
>gi|14521094|ref|NP_126569.1| hypothetical protein PAB1770 [Pyrococcus abyssi GE5]
>gi|14521108|ref|NP_126583.1| hypothetical protein PAB0603 [Pyrococcus abyssi GE5]
>gi|14521131|ref|NP_126606.1| hypothetical protein PAB0618 [Pyrococcus abyssi GE5]
>gi|14521133|ref|NP_126608.1| hypothetical protein PAB0620 [Pyrococcus abyssi GE5]
>gi|14521137|ref|NP_126612.1| hypothetical protein PAB0624 [Pyrococcus abyssi GE5]
>gi|33356735|ref|NP_126636.2| hypothetical protein PAB0643 [Pyrococcus abyssi GE5]
>gi|33356736|ref|NP_877652.1| hypothetical protein PAB0644.1n [Pyrococcus abyssi GE5]
>gi|14521168|ref|NP_126643.1| hypothetical protein PAB0649 [Pyrococcus abyssi GE5]
>gi|14521179|ref|NP_126654.1| hypothetical protein PAB0655 [Pyrococcus abyssi GE5]
>gi|14521192|ref|NP_126667.1| hypothetical protein PAB1715 [Pyrococcus abyssi GE5]

>gi|14521217|ref|NP_126692.1| hypothetical protein PAB1698 [Pyrococcus abyssi GE5]
>gi|14521218|ref|NP_126693.1| hypothetical protein PAB1697 [Pyrococcus abyssi GE5]
>gi|33356739|ref|NP_877653.1| hypothetical protein PAB1693.1n [Pyrococcus abyssi GE5]
>gi|14521223|ref|NP_126698.1| hypothetical protein PAB1692 [Pyrococcus abyssi GE5]
>gi|33356741|ref|NP_877655.1| hypothetical protein PAB1672.1n [Pyrococcus abyssi GE5]
>gi|14521264|ref|NP_126739.1| hypothetical protein PAB0705 [Pyrococcus abyssi GE5]
>gi|14521274|ref|NP_126749.1| hypothetical protein PAB0711 [Pyrococcus abyssi GE5]
>gi|14521285|ref|NP_126760.1| hypothetical protein PAB0720 [Pyrococcus abyssi GE5]
>gi|14521311|ref|NP_126786.1| hypothetical protein PAB0732 [Pyrococcus abyssi GE5]
>gi|14521316|ref|NP_126791.1| hypothetical protein PAB0736 [Pyrococcus abyssi GE5]
>gi|14521319|ref|NP_126794.1| hypothetical protein PAB1631 [Pyrococcus abyssi GE5]
>gi|33356747|ref|NP_126810.2| hypothetical protein PAB0747 [Pyrococcus abyssi GE5]
>gi|33356748|ref|NP_877660.1| hypothetical protein PAB1612.1n [Pyrococcus abyssi GE5]
>gi|14521366|ref|NP_126842.1| putative acetyltransferase [Pyrococcus abyssi GE5]
>gi|33356751|ref|NP_877662.1| hypothetical protein PAB0776.3n [Pyrococcus abyssi GE5]
>gi|33356754|ref|NP_877665.1| hypothetical protein PAB1598.2n [Pyrococcus abyssi GE5]
>gi|33356756|ref|NP_877667.1| hypothetical protein PAB1598.4n [Pyrococcus abyssi GE5]
>gi|33356757|ref|NP_877668.1| hypothetical protein PAB1595.1n [Pyrococcus abyssi GE5]
>gi|33356762|ref|NP_877673.1| hypothetical polysaccharide biosynthesis related protein [Pyrococcus abyssi GE5]
>gi|33356763|ref|NP_877674.1| hypothetical protein PAB2422.2n [Pyrococcus abyssi GE5]
>gi|14521425|ref|NP_126901.1| purine NTPase, putative [Pyrococcus abyssi GE5]
>gi|33356779|ref|NP_877688.1| hypothetical protein PAB1533.2n [Pyrococcus abyssi GE5]
>gi|14521479|ref|NP_126955.1| hypothetical protein PAB3319 [Pyrococcus abyssi GE5]
>gi|14521488|ref|NP_126964.1| hypothetical protein PAB0850 [Pyrococcus abyssi GE5]
>gi|14521537|ref|NP_127013.1| hypothetical protein PAB1493 [Pyrococcus abyssi GE5]
>gi|14521541|ref|NP_127017.1| hypothetical protein PAB0885 [Pyrococcus abyssi GE5]
>gi|14521588|ref|NP_127064.1| hypothetical protein PAB1458 [Pyrococcus abyssi GE5]
>gi|14521591|ref|NP_127067.1| hypothetical protein PAB1455 [Pyrococcus abyssi GE5]
>gi|14521614|ref|NP_127090.1| dna damage-inducible protein. [Pyrococcus abyssi GE5]
>gi|14521629|ref|NP_127105.1| hypothetical protein PAB1429 [Pyrococcus abyssi GE5]
>gi|14521632|ref|NP_127108.1| hypothetical protein PAB1428 [Pyrococcus abyssi GE5]
>gi|33356793|ref|NP_877699.1| hypothetical protein PAB1399.1n [Pyrococcus abyssi GE5]
>gi|33356794|ref|NP_877700.1| hypothetical protein PAB1398.1n [Pyrococcus abyssi GE5]
>gi|14521673|ref|NP_127149.1| hydrogenase-4 component c [Pyrococcus abyssi GE5]
>gi|14521690|ref|NP_127166.1| putative flagella-related protein d or e [Pyrococcus abyssi GE5]
>gi|14521691|ref|NP_127167.1| flagella-related protein c [Pyrococcus abyssi GE5]
>gi|14521697|ref|NP_127173.1| hypothetical protein PAB0993 [Pyrococcus abyssi GE5]
>gi|14521718|ref|NP_127194.1| LSU ribosomal protein L39E [Pyrococcus abyssi GE5]
>gi|14521752|ref|NP_127228.1| chemotaxis histidine kinase [Pyrococcus abyssi GE5]
>gi|14521754|ref|NP_127230.1| chemotaxis response regulator [Pyrococcus abyssi GE5]
>gi|14521787|ref|NP_127263.1| O-sialoglycoprotein endopeptidase, putative [Pyrococcus abyssi GE5]
>gi|33356810|ref|NP_127304.2| hypothetical protein PAB1278 [Pyrococcus abyssi GE5]
>gi|14521963|ref|NP_127440.1| H+-transporting ATP synthase, subunit E [Pyrococcus abyssi GE5]
>gi|14521964|ref|NP_127441.1| probable atpase proteolipid chain [Pyrococcus abyssi GE5]
>gi|14521966|ref|NP_127443.1| hypothetical protein PAB1179 [Pyrococcus abyssi GE5]
>gi|14521972|ref|NP_127449.1| hypothetical protein PAB1174 [Pyrococcus abyssi GE5]
>gi|14521981|ref|NP_127458.1| LSU ribosomal protein L12A [Pyrococcus abyssi GE5]
>gi|14521982|ref|NP_127459.1| LSU ribosomal protein L10E [Pyrococcus abyssi GE5]
>gi|38232650|ref|NP_938417.1| hypothetical protein DIP0008 [Corynebacterium diphtheriae NCTC 13129]
>gi|38232666|ref|NP_938433.1| Putative membrane protein [Corynebacterium diphtheriae NCTC 13129]
>gi|38232690|ref|NP_938457.1| probable serine/threonine-protein kinase [Corynebacterium diphtheriae NCTC 13129]
>gi|38232696|ref|NP_938463.1| Putative cation-transporting ATPase [Corynebacterium diphtheriae NCTC 13129]
>gi|38232700|ref|NP_938467.1| hypothetical protein DIP0066 [Corynebacterium diphtheriae NCTC 13129]
>gi|38232723|ref|NP_938490.1| Putative membrane protein [Corynebacterium diphtheriae NCTC 13129]
>gi|38232725|ref|NP_938492.1| choline transport system permease protein [Corynebacterium diphtheriae NCTC 13129]
>gi|38232730|ref|NP_938497.1| hypothetical protein DIP0100 [Corynebacterium diphtheriae NCTC 13129]
>gi|38232738|ref|NP_938505.1| Membrane protein permease Irp6B [Corynebacterium diphtheriae NCTC 13129]
>gi|38232745|ref|NP_938512.1| Putative membrane protein [Corynebacterium diphtheriae NCTC 13129]
>gi|38232750|ref|NP_938517.1| Putative siderophore binding protein [Corynebacterium diphtheriae NCTC 13129]
>gi|38232760|ref|NP_938527.1| Putative sugar acetyltransferase [Corynebacterium diphtheriae NCTC 13129]
>gi|38232772|ref|NP_938539.1| Putative integral membrane protein [Corynebacterium diphtheriae NCTC 13129]
>gi|38232824|ref|NP_938591.1| hypothetical protein DIP0198 [Corynebacterium diphtheriae NCTC 13129]
>gi|38232838|ref|NP_938605.1| immunity-specific protein Beta241 [Corynebacterium diphtheriae NCTC 13129]
>gi|38232842|ref|NP_938609.1| Putative phage tail fiber protein [Corynebacterium diphtheriae NCTC 13129]
>gi|38232861|ref|NP_938628.1| Putative surface anchored protein [Corynebacterium diphtheriae NCTC 13129]
>gi|38232876|ref|NP_938643.1| gluconate permease [Corynebacterium diphtheriae NCTC 13129]
>gi|38232882|ref|NP_938649.1| DNA polymerase III subunit gamma/tau [Corynebacterium diphtheriae NCTC 13129]
>gi|38232896|ref|NP_938663.1| Putative membrane protein [Corynebacterium diphtheriae NCTC 13129]
>gi|38232915|ref|NP_938682.1| Putative membrane protein [Corynebacterium diphtheriae NCTC 13129]
>gi|38232918|ref|NP_938685.1| hypothetical protein DIP0297 [Corynebacterium diphtheriae NCTC 13129]

>gi|38232930|ref|NP_938697.1| Putative membrane protein [Corynebacterium diphtheriae NCTC 13129]
>gi|38232935|ref|NP_938702.1| Putative membrane protein [Corynebacterium diphtheriae NCTC 13129]
>gi|38232937|ref|NP_938704.1| Putative membrane protein [Corynebacterium diphtheriae NCTC 13129]
>gi|38232939|ref|NP_938706.1| Putative secreted protein [Corynebacterium diphtheriae NCTC 13129]
>gi|38232957|ref|NP_938724.1| hypothetical protein DIP0336 [Corynebacterium diphtheriae NCTC 13129]
>gi|38232961|ref|NP_938728.1| hypothetical protein DIP0343 [Corynebacterium diphtheriae NCTC 13129]
>gi|38232989|ref|NP_938756.1| Putative membrane protein [Corynebacterium diphtheriae NCTC 13129]
>gi|38233007|ref|NP_938774.1| Putative membrane protein [Corynebacterium diphtheriae NCTC 13129]
>gi|38233013|ref|NP_938780.1| hypothetical protein DIP0399 [Corynebacterium diphtheriae NCTC 13129]
>gi|38233051|ref|NP_938818.1| 50S ribosomal protein L7/L12 [Corynebacterium diphtheriae NCTC 13129]
>gi|38233058|ref|NP_938825.1| Putative membrane protein [Corynebacterium diphtheriae NCTC 13129]
>gi|38233153|ref|NP_938920.1| Putative secreted protein [Corynebacterium diphtheriae NCTC 13129]
>gi|38233159|ref|NP_938926.1| 50S ribosomal protein L17 [Corynebacterium diphtheriae NCTC 13129]
>gi|38233174|ref|NP_938941.1| hypothetical protein DIP0565 [Corynebacterium diphtheriae NCTC 13129]
>gi|38233187|ref|NP_938954.1| hypothetical protein DIP0578 [Corynebacterium diphtheriae NCTC 13129]
>gi|38233192|ref|NP_938959.1| Putative iron transport system membrane protein [Corynebacterium diphtheriae NCTC 13129]
>gi|38233193|ref|NP_938960.1| Putative iron transport system membrane protein [Corynebacterium diphtheriae NCTC 13129]
>gi|38233196|ref|NP_938963.1| Putative membrane protein [Corynebacterium diphtheriae NCTC 13129]
>gi|38233209|ref|NP_938976.1| Putative membrane protein [Corynebacterium diphtheriae NCTC 13129]
>gi|38233214|ref|NP_938981.1| Putative membrane protein [Corynebacterium diphtheriae NCTC 13129]
>gi|38233225|ref|NP_938992.1| Putative ABC transport system membrane protein [Corynebacterium diphtheriae NCTC 13129]
>gi|38233241|ref|NP_939008.1| Putative membrane protein [Corynebacterium diphtheriae NCTC 13129]
>gi|38233255|ref|NP_939022.1| hypothetical protein DIP0648 [Corynebacterium diphtheriae NCTC 13129]
>gi|38233266|ref|NP_939033.1| Putative membrane protein [Corynebacterium diphtheriae NCTC 13129]
>gi|38233297|ref|NP_939064.1| Putative secreted protein [Corynebacterium diphtheriae NCTC 13129]
>gi|38233337|ref|NP_939104.1| hypothetical protein DIP0731 [Corynebacterium diphtheriae NCTC 13129]
>gi|38233340|ref|NP_939107.1| Putative membrane protein [Corynebacterium diphtheriae NCTC 13129]
>gi|38233342|ref|NP_939109.1| Putative protease [Corynebacterium diphtheriae NCTC 13129]
>gi|38233369|ref|NP_939136.1| transposase A [Corynebacterium diphtheriae NCTC 13129]
>gi|38233392|ref|NP_939159.1| Putative secreted protein [Corynebacterium diphtheriae NCTC 13129]
>gi|38233399|ref|NP_939166.1| hypothetical protein DIP0800 [Corynebacterium diphtheriae NCTC 13129]
>gi|38233434|ref|NP_939201.1| Putative membrane protein [Corynebacterium diphtheriae NCTC 13129]
>gi|38233437|ref|NP_939204.1| Putative membrane protein [Corynebacterium diphtheriae NCTC 13129]
>gi|38233457|ref|NP_939224.1| Putative membrane protein [Corynebacterium diphtheriae NCTC 13129]
>gi|38233462|ref|NP_939229.1| Putative membrane protein [Corynebacterium diphtheriae NCTC 13129]
>gi|38233463|ref|NP_939230.1| Putative membrane protein [Corynebacterium diphtheriae NCTC 13129]
>gi|38233489|ref|NP_939256.1| Putative membrane protein [Corynebacterium diphtheriae NCTC 13129]
>gi|38233497|ref|NP_939264.1| Putative 50S ribosomal protein L25 [Corynebacterium diphtheriae NCTC 13129]
>gi|38233547|ref|NP_939314.1| Putative membrane protein [Corynebacterium diphtheriae NCTC 13129]
>gi|38233548|ref|NP_939315.1| Putative chloride channel related membrane protein [Corynebacterium diphtheriae NCTC 13129]
>gi|38233553|ref|NP_939320.1| Putative peptide transport system membrane protein [Corynebacterium diphtheriae NCTC 13129]
>gi|38233581|ref|NP_939348.1| Putative secreted protein [Corynebacterium diphtheriae NCTC 13129]
>gi|38233604|ref|NP_939371.1| hypothetical protein DIP1009 [Corynebacterium diphtheriae NCTC 13129]
>gi|38233635|ref|NP_939402.1| peptide chain release factor 1 (RF-1) [Corynebacterium diphtheriae NCTC 13129]
>gi|38233639|ref|NP_939406.1| Hypothetical integral membrane protein [Corynebacterium diphtheriae NCTC 13129]
>gi|38233642|ref|NP_939409.1| ATP synthase B chain [Corynebacterium diphtheriae NCTC 13129]
>gi|38233677|ref|NP_939444.1| Conserved putative integral membrane protein [Corynebacterium diphtheriae NCTC 13129]
>gi|38233689|ref|NP_939456.1| Conserved hypothetical integral membrane protein [Corynebacterium diphtheriae NCTC 13129]
>gi|38233739|ref|NP_939506.1| Putative transport protein [Corynebacterium diphtheriae NCTC 13129]
>gi|38233778|ref|NP_939545.1| hypothetical protein DIP1188 [Corynebacterium diphtheriae NCTC 13129]
>gi|38233830|ref|NP_939597.1| Sec-independent twin-arginine translocase system protein [Corynebacterium diphtheriae NCTC 13129]
>gi|38233860|ref|NP_939627.1| Putative DNA-binding protein [Corynebacterium diphtheriae NCTC 13129]
>gi|38233861|ref|NP_939628.1| Putative secreted protein [Corynebacterium diphtheriae NCTC 13129]
>gi|38233866|ref|NP_939633.1| Putative invasion protein [Corynebacterium diphtheriae NCTC 13129]
>gi|38233867|ref|NP_939634.1| hypothetical protein DIP1282 [Corynebacterium diphtheriae NCTC 13129]
>gi|38233968|ref|NP_939735.1| Putative secreted protein [Corynebacterium diphtheriae NCTC 13129]
>gi|38233991|ref|NP_939758.1| RNA polymerase sigma factor [Corynebacterium diphtheriae NCTC 13129]
>gi|38234007|ref|NP_939774.1| Putative membrane protein [Corynebacterium diphtheriae NCTC 13129]
>gi|38234033|ref|NP_939800.1| Putative membrane protein [Corynebacterium diphtheriae NCTC 13129]
>gi|38234058|ref|NP_939825.1| translation initiation factor IF-2 [Corynebacterium diphtheriae NCTC 13129]
>gi|38234086|ref|NP_939853.1| elongation factor TS [Corynebacterium diphtheriae NCTC 13129]
>gi|38234109|ref|NP_939876.1| 30S ribosomal protein S16 [Corynebacterium diphtheriae NCTC 13129]
>gi|38234113|ref|NP_939880.1| Putative cell division protein [Corynebacterium diphtheriae NCTC 13129]
>gi|38234121|ref|NP_939888.1| hypothetical protein DIP1546 [Corynebacterium diphtheriae NCTC 13129]
>gi|38234131|ref|NP_939898.1| Putative membrane protein [Corynebacterium diphtheriae NCTC 13129]
>gi|38234138|ref|NP_939905.1| Putative membrane protein [Corynebacterium diphtheriae NCTC 13129]
>gi|38234145|ref|NP_939912.1| Putative secreted protein [Corynebacterium diphtheriae NCTC 13129]
>gi|38234152|ref|NP_939919.1| Putative secreted protein [Corynebacterium diphtheriae NCTC 13129]
>gi|38234156|ref|NP_939923.1| Putative membrane protein [Corynebacterium diphtheriae NCTC 13129]
>gi|38234161|ref|NP_939928.1| Putative secreted protein [Corynebacterium diphtheriae NCTC 13129]

>gi|38234165|ref|NP_939932.1| Antigen 84 [Corynebacterium diphtheriae NCTC 13129]
>gi|38234180|ref|NP_939947.1| Putative membrane protein [Corynebacterium diphtheriae NCTC 13129]
>gi|38234197|ref|NP_939964.1| hypothetical protein DIP1622 [Corynebacterium diphtheriae NCTC 13129]
>gi|38234225|ref|NP_939992.1| Putative secreted protein [Corynebacterium diphtheriae NCTC 13129]
>gi|38234256|ref|NP_940023.1| Putative cobalamin biosynthesis protein [Corynebacterium diphtheriae NCTC 13129]
>gi|38234269|ref|NP_940036.1| Putative thiamine biosynthesis related protein [Corynebacterium diphtheriae NCTC 13129]
>gi|38234323|ref|NP_940090.1| Putative antibiotic resistance related transport protein [Corynebacterium diphtheriae NCTC 13129]
>gi|38234325|ref|NP_940092.1| Putative membrane protein [Corynebacterium diphtheriae NCTC 13129]
>gi|38234337|ref|NP_940104.1| 30S ribosomal protein S20 [Corynebacterium diphtheriae NCTC 13129]
>gi|38234342|ref|NP_940109.1| Putative membrane protein [Corynebacterium diphtheriae NCTC 13129]
>gi|38234353|ref|NP_940120.1| Putative RNA-associated protein [Corynebacterium diphtheriae NCTC 13129]
>gi|38234381|ref|NP_940148.1| Putative membrane protein [Corynebacterium diphtheriae NCTC 13129]
>gi|38234398|ref|NP_940165.1| hypothetical protein DIP1827 [Corynebacterium diphtheriae NCTC 13129]
>gi|38234405|ref|NP_940172.1| hypothetical protein DIP1834 [Corynebacterium diphtheriae NCTC 13129]
>gi|38234407|ref|NP_940174.1| collagen-like repeat protein [Corynebacterium diphtheriae NCTC 13129]
>gi|38234425|ref|NP_940192.1| hypothetical protein DIP1855 [Corynebacterium diphtheriae NCTC 13129]
>gi|38234453|ref|NP_940220.1| Putative membrane protein [Corynebacterium diphtheriae NCTC 13129]
>gi|38234469|ref|NP_940236.1| Putative cytochrome ubiquinol oxidase subunit [Corynebacterium diphtheriae NCTC 13129]
>gi|38234488|ref|NP_940255.1| hypothetical protein DIP1921 [Corynebacterium diphtheriae NCTC 13129]
>gi|38234498|ref|NP_940265.1| sodium transport protein [Corynebacterium diphtheriae NCTC 13129]
>gi|38234505|ref|NP_940272.1| Putative membrane protein [Corynebacterium diphtheriae NCTC 13129]
>gi|38234528|ref|NP_940295.1| Putative amino acid export carrier protein [Corynebacterium diphtheriae NCTC 13129]
>gi|38234529|ref|NP_940296.1| hypothetical protein DIP1965 [Corynebacterium diphtheriae NCTC 13129]
>gi|38234539|ref|NP_940306.1| hypothetical protein DIP1975 [Corynebacterium diphtheriae NCTC 13129]
>gi|38234542|ref|NP_940309.1| hypothetical protein DIP1978 [Corynebacterium diphtheriae NCTC 13129]
>gi|38234543|ref|NP_940310.1| Putative carbonic anhydrase [Corynebacterium diphtheriae NCTC 13129]
>gi|38234559|ref|NP_940326.1| Putative membrane protein [Corynebacterium diphtheriae NCTC 13129]
>gi|38234575|ref|NP_940342.1| Putative surface anchored protein [Corynebacterium diphtheriae NCTC 13129]
>gi|38234594|ref|NP_940361.1| Putative membrane protein [Corynebacterium diphtheriae NCTC 13129]
>gi|38234600|ref|NP_940367.1| Putative secreted protein [Corynebacterium diphtheriae NCTC 13129]
>gi|38234652|ref|NP_940419.1| Putative membrane protein [Corynebacterium diphtheriae NCTC 13129]
>gi|38234667|ref|NP_940434.1| chaperone protein cofactor 1 [Corynebacterium diphtheriae NCTC 13129]
>gi|38234706|ref|NP_940473.1| Putative ABC transport system permease protein [Corynebacterium diphtheriae NCTC 13129]
>gi|38234707|ref|NP_940474.1| Putative ABC transport system permease protein [Corynebacterium diphtheriae NCTC 13129]
>gi|38234709|ref|NP_940476.1| Putative ABC transport system permease protein [Corynebacterium diphtheriae NCTC 13129]
>gi|38234740|ref|NP_940507.1| Putative conserved integral membrane protein [Corynebacterium diphtheriae NCTC 13129]
>gi|38234757|ref|NP_940524.1| single-strand DNA binding protein [Corynebacterium diphtheriae NCTC 13129]
>gi|38234811|ref|NP_940578.1| hypothetical protein DIP2275 [Corynebacterium diphtheriae NCTC 13129]
>gi|38234817|ref|NP_940584.1| hypothetical protein DIP2281 [Corynebacterium diphtheriae NCTC 13129]
>gi|38234818|ref|NP_940585.1| hypothetical protein DIP2282 [Corynebacterium diphtheriae NCTC 13129]
>gi|38234825|ref|NP_940592.1| single-strand DNA binding protein [Corynebacterium diphtheriae NCTC 13129]
>gi|38234827|ref|NP_940594.1| Putative membrane protein [Corynebacterium diphtheriae NCTC 13129]
>gi|38234828|ref|NP_940595.1| Putative integral membrane protein [Corynebacterium diphtheriae NCTC 13129]
>gi|38234853|ref|NP_940620.1| Putative copper zinc superoxide dismutase [Corynebacterium diphtheriae NCTC 13129]
>gi|38234858|ref|NP_940625.1| Putative ABC transport system integral membrane protein [Corynebacterium diphtheriae NCTC 13129]
>gi|38234862|ref|NP_940629.1| Conserved integral membrane protein [Corynebacterium diphtheriae NCTC 13129]
>gi|38234863|ref|NP_940630.1| Conserved integral membrane protein [Corynebacterium diphtheriae NCTC 13129]
>gi|38234869|ref|NP_940636.1| Putative dihydroxyacetone kinase sununit [Corynebacterium diphtheriae NCTC 13129]
>gi|38234870|ref|NP_940637.1| hypothetical protein DIP2336 [Corynebacterium diphtheriae NCTC 13129]
>gi|38234875|ref|NP_940642.1| Putative integral membrane transport protein [Corynebacterium diphtheriae NCTC 13129]
>gi|38234902|ref|NP_940669.1| hypothetical protein DIP2369 [Corynebacterium diphtheriae NCTC 13129]
>gi|15789344|ref|NP_279168.1| hypothetical protein VNG0005H [Halobacterium sp. NRC-1]
>gi|15789400|ref|NP_279224.1| hypothetical protein VNG0066H [Halobacterium sp. NRC-1]
>gi|15789405|ref|NP_279229.1| hypothetical protein VNG0072H [Halobacterium sp. NRC-1]
>gi|15789412|ref|NP_279236.1| MoaE [Halobacterium sp. NRC-1]
>gi|15789418|ref|NP_279242.1| hypothetical protein VNG0091C [Halobacterium sp. NRC-1]
>gi|15789427|ref|NP_279251.1| SerA3 [Halobacterium sp. NRC-1]
>gi|15789435|ref|NP_279259.1| YusZ1 [Halobacterium sp. NRC-1]
>gi|15789441|ref|NP_279265.1| hypothetical protein VNG0121H [Halobacterium sp. NRC-1]
>gi|15789443|ref|NP_279267.1| hypothetical protein VNG0124C [Halobacterium sp. NRC-1]
>gi|15789444|ref|NP_279268.1| hypothetical protein VNG0125H [Halobacterium sp. NRC-1]
>gi|15789447|ref|NP_279271.1| Hsp4 [Halobacterium sp. NRC-1]
>gi|15789449|ref|NP_279273.1| hypothetical protein VNG0132C [Halobacterium sp. NRC-1]
>gi|15789454|ref|NP_279278.1| hypothetical protein VNG0138H [Halobacterium sp. NRC-1]
>gi|15789465|ref|NP_279289.1| hypothetical protein VNG0150H [Halobacterium sp. NRC-1]
>gi|15789470|ref|NP_279294.1| hypothetical protein VNG0156C [Halobacterium sp. NRC-1]
>gi|15789471|ref|NP_279295.1| OxlT [Halobacterium sp. NRC-1]
>gi|15789472|ref|NP_279296.1| 2-keto-3-deoxygluconate kinase [Halobacterium sp. NRC-1]
>gi|15789473|ref|NP_279297.1| DNA mismatch repair protein [Halobacterium sp. NRC-1]
>gi|15789477|ref|NP_279301.1| MutS1 [Halobacterium sp. NRC-1]

>gi|15789478|ref|NP_279302.1| Hef [Halobacterium sp. NRC-1]
>gi|15789484|ref|NP_279308.1| MutS2 [Halobacterium sp. NRC-1]
>gi|15789489|ref|NP_279313.1| hypothetical protein VNG0178H [Halobacterium sp. NRC-1]
>gi|15789494|ref|NP_279318.1| hypothetical protein VNG0184H [Halobacterium sp. NRC-1]
>gi|15789496|ref|NP_279320.1| hypothetical protein VNG0187H [Halobacterium sp. NRC-1]
>gi|15789504|ref|NP_279328.1| hypothetical protein VNG0197C [Halobacterium sp. NRC-1]
>gi|15789505|ref|NP_279329.1| hypothetical protein VNG0198H [Halobacterium sp. NRC-1]
>gi|15789507|ref|NP_279331.1| hypothetical protein VNG0200C [Halobacterium sp. NRC-1]
>gi|15789508|ref|NP_279332.1| hypothetical protein VNG0204H [Halobacterium sp. NRC-1]
>gi|15789509|ref|NP_279333.1| hypothetical protein VNG0203C [Halobacterium sp. NRC-1]
>gi|15789510|ref|NP_279334.1| hypothetical protein VNG0205H [Halobacterium sp. NRC-1]
>gi|15789522|ref|NP_279346.1| hypothetical protein VNG0219H [Halobacterium sp. NRC-1]
>gi|15789524|ref|NP_279348.1| hypothetical protein VNG0222C [Halobacterium sp. NRC-1]
>gi|15789525|ref|NP_279349.1| MoxR [Halobacterium sp. NRC-1]
>gi|15789531|ref|NP_279355.1| hypothetical protein VNG0233H [Halobacterium sp. NRC-1]
>gi|15789538|ref|NP_279362.1| hypothetical protein VNG0244H [Halobacterium sp. NRC-1]
>gi|15789541|ref|NP_279365.1| hypothetical protein VNG0248C [Halobacterium sp. NRC-1]
>gi|15789542|ref|NP_279366.1| Fbr [Halobacterium sp. NRC-1]
>gi|15789544|ref|NP_279368.1| hypothetical protein VNG0251C [Halobacterium sp. NRC-1]
>gi|15789548|ref|NP_279372.1| hypothetical protein VNG0256H [Halobacterium sp. NRC-1]
>gi|15789551|ref|NP_279375.1| hypothetical protein VNG0261H [Halobacterium sp. NRC-1]
>gi|15789555|ref|NP_279379.1| hypothetical protein VNG0266H [Halobacterium sp. NRC-1]
>gi|15789556|ref|NP_279380.1| hypothetical protein VNG0267H [Halobacterium sp. NRC-1]
>gi|15789558|ref|NP_279382.1| hypothetical protein VNG0270C [Halobacterium sp. NRC-1]
>gi|15789560|ref|NP_279384.1| hypothetical protein VNG0272H [Halobacterium sp. NRC-1]
>gi|15789563|ref|NP_279387.1| hypothetical protein VNG0280H [Halobacterium sp. NRC-1]
>gi|15789564|ref|NP_279388.1| SoxB [Halobacterium sp. NRC-1]
>gi|15789572|ref|NP_279396.1| hypothetical protein VNG0291H [Halobacterium sp. NRC-1]
>gi|15789577|ref|NP_279401.1| hypothetical protein VNG0298H [Halobacterium sp. NRC-1]
>gi|15789582|ref|NP_279406.1| TrpC [Halobacterium sp. NRC-1]
>gi|15789589|ref|NP_279413.1| 3-dehydroquinate dehydratase [Halobacterium sp. NRC-1]
>gi|15789596|ref|NP_279420.1| hypothetical protein VNG0322H [Halobacterium sp. NRC-1]
>gi|15789607|ref|NP_279431.1| hypothetical protein VNG0339H [Halobacterium sp. NRC-1]
>gi|15789608|ref|NP_279432.1| hypothetical protein VNG0340C [Halobacterium sp. NRC-1]
>gi|15789609|ref|NP_279433.1| Smc1 [Halobacterium sp. NRC-1]
>gi|15789610|ref|NP_279434.1| hypothetical protein VNG0343H [Halobacterium sp. NRC-1]
>gi|15789612|ref|NP_279436.1| hypothetical protein VNG0346H [Halobacterium sp. NRC-1]
>gi|15789618|ref|NP_279442.1| Htr14 [Halobacterium sp. NRC-1]
>gi|15789619|ref|NP_279443.1| hypothetical protein VNG0357H [Halobacterium sp. NRC-1]
>gi|15789630|ref|NP_279454.1| hypothetical protein VNG0373H [Halobacterium sp. NRC-1]
>gi|15789635|ref|NP_279459.1| Caa [Halobacterium sp. NRC-1]
>gi|15789636|ref|NP_279460.1| AroE [Halobacterium sp. NRC-1]
>gi|15789637|ref|NP_279461.1| hypothetical protein VNG0383H [Halobacterium sp. NRC-1]
>gi|15789645|ref|NP_279469.1| hypothetical protein VNG0393C [Halobacterium sp. NRC-1]
>gi|15789646|ref|NP_279470.1| hypothetical protein VNG0394C [Halobacterium sp. NRC-1]
>gi|15789647|ref|NP_279471.1| hypothetical protein VNG0396C [Halobacterium sp. NRC-1]
>gi|15789648|ref|NP_279472.1| NthA1 [Halobacterium sp. NRC-1]
>gi|15789656|ref|NP_279480.1| hypothetical protein VNG0407H [Halobacterium sp. NRC-1]
>gi|15789657|ref|NP_279481.1| hypothetical protein VNG0408H [Halobacterium sp. NRC-1]
>gi|15789661|ref|NP_279485.1| PurH [Halobacterium sp. NRC-1]
>gi|15789665|ref|NP_279489.1| hypothetical protein VNG0420H [Halobacterium sp. NRC-1]
>gi|15789668|ref|NP_279492.1| hypothetical protein VNG0424C [Halobacterium sp. NRC-1]
>gi|15789669|ref|NP_279493.1| L-isoaspartyl protein carboxyl methyltransferase [Halobacterium sp. NRC-1]
>gi|15789671|ref|NP_279495.1| YfmO2 [Halobacterium sp. NRC-1]
>gi|15789682|ref|NP_279506.1| hypothetical protein VNG0440C [Halobacterium sp. NRC-1]
>gi|15789683|ref|NP_279507.1| hypothetical protein VNG0441H [Halobacterium sp. NRC-1]
>gi|15789685|ref|NP_279509.1| DapA [Halobacterium sp. NRC-1]
>gi|15789686|ref|NP_279510.1| hypothetical protein VNG0447H [Halobacterium sp. NRC-1]
>gi|15789692|ref|NP_279516.1| PstA2 [Halobacterium sp. NRC-1]
>gi|15789699|ref|NP_279523.1| hypothetical protein VNG0463C [Halobacterium sp. NRC-1]
>gi|15789701|ref|NP_279525.1| hypothetical protein VNG0466C [Halobacterium sp. NRC-1]
>gi|15789704|ref|NP_279528.1| hypothetical protein VNG0469H [Halobacterium sp. NRC-1]
>gi|15789710|ref|NP_279534.1| hypothetical protein VNG0475C [Halobacterium sp. NRC-1]
>gi|15789720|ref|NP_279544.1| hypothetical protein VNG0488H [Halobacterium sp. NRC-1]
>gi|15789721|ref|NP_279545.1| DnaJ [Halobacterium sp. NRC-1]
>gi|15789722|ref|NP_279546.1| DnaK [Halobacterium sp. NRC-1]
>gi|15789724|ref|NP_279548.1| GrpE [Halobacterium sp. NRC-1]
>gi|15789737|ref|NP_279561.1| hypothetical protein VNG0509H [Halobacterium sp. NRC-1]
>gi|15789741|ref|NP_279565.1| hypothetical protein VNG0514C [Halobacterium sp. NRC-1]
>gi|15789742|ref|NP_279566.1| hypothetical protein VNG0516H [Halobacterium sp. NRC-1]

>gi|15789752|ref|NP_279576.1| hypothetical protein VNG0532H [Halobacterium sp. NRC-1]
>gi|15789753|ref|NP_279577.1| hypothetical protein VNG0533H [Halobacterium sp. NRC-1]
>gi|15789757|ref|NP_279581.1| hypothetical protein VNG0537C [Halobacterium sp. NRC-1]
>gi|15789763|ref|NP_279587.1| hypothetical protein VNG0544H [Halobacterium sp. NRC-1]
>gi|15789769|ref|NP_279593.1| hypothetical protein VNG0553C [Halobacterium sp. NRC-1]
>gi|15789770|ref|NP_279594.1| hypothetical protein VNG0555C [Halobacterium sp. NRC-1]
>gi|15789771|ref|NP_279595.1| Sgb [Halobacterium sp. NRC-1]
>gi|15789784|ref|NP_279608.1| hypothetical protein VNG0573C [Halobacterium sp. NRC-1]
>gi|15789795|ref|NP_279619.1| hypothetical protein VNG0586C [Halobacterium sp. NRC-1]
>gi|15789804|ref|NP_279628.1| hypothetical protein VNG0597H [Halobacterium sp. NRC-1]
>gi|15789807|ref|NP_279631.1| hypothetical protein VNG0599C [Halobacterium sp. NRC-1]
>gi|15789814|ref|NP_279638.1| 4-hydroxybenzoate octaprenyltransferase [Halobacterium sp. NRC-1]
>gi|15789824|ref|NP_279648.1| hypothetical protein VNG0624H [Halobacterium sp. NRC-1]
>gi|15789829|ref|NP_279653.1| RibE [Halobacterium sp. NRC-1]
>gi|15789830|ref|NP_279654.1| hypothetical protein VNG0631C [Halobacterium sp. NRC-1]
>gi|15789839|ref|NP_279663.1| hypothetical protein VNG0642C [Halobacterium sp. NRC-1]
>gi|15789852|ref|NP_279676.1| hypothetical protein VNG0660H [Halobacterium sp. NRC-1]
>gi|15789856|ref|NP_279680.1| CtaB [Halobacterium sp. NRC-1]
>gi|15789857|ref|NP_279681.1| ABC transporter, ATP-binding protein homolog [Halobacterium sp. NRC-1]
>gi|15789859|ref|NP_279683.1| hypothetical protein VNG0669H [Halobacterium sp. NRC-1]
>gi|15789860|ref|NP_279684.1| hypothetical protein VNG0670H [Halobacterium sp. NRC-1]
>gi|15789871|ref|NP_279695.1| hypothetical protein VNG0682C [Halobacterium sp. NRC-1]
>gi|15789875|ref|NP_279699.1| hypothetical protein VNG0688H [Halobacterium sp. NRC-1]
>gi|15789877|ref|NP_279701.1| hypothetical protein VNG0690C [Halobacterium sp. NRC-1]
>gi|15789878|ref|NP_279702.1| hypothetical protein VNG0692C [Halobacterium sp. NRC-1]
>gi|15789882|ref|NP_279706.1| YvgX [Halobacterium sp. NRC-1]
>gi|15789888|ref|NP_279712.1| hypothetical protein VNG0708H [Halobacterium sp. NRC-1]
>gi|15789891|ref|NP_279715.1| hypothetical protein VNG0713C [Halobacterium sp. NRC-1]
>gi|15789893|ref|NP_279717.1| AfsQ2 [Halobacterium sp. NRC-1]
>gi|15789896|ref|NP_279720.1| AraL [Halobacterium sp. NRC-1]
>gi|15789897|ref|NP_279721.1| DNA damage-inducible protein [Halobacterium sp. NRC-1]
>gi|15789898|ref|NP_279722.1| PepQ1 [Halobacterium sp. NRC-1]
>gi|15789904|ref|NP_279728.1| hypothetical protein VNG0730C [Halobacterium sp. NRC-1]
>gi|15789905|ref|NP_279729.1| hypothetical protein VNG0731H [Halobacterium sp. NRC-1]
>gi|15789906|ref|NP_279730.1| RNA 3'-terminal phosphate cyclase [Halobacterium sp. NRC-1]
>gi|15789909|ref|NP_279733.1| Hlx2 [Halobacterium sp. NRC-1]
>gi|15789912|ref|NP_279736.1| hypothetical protein VNG0737H [Halobacterium sp. NRC-1]
>gi|15789914|ref|NP_279738.1| hypothetical protein VNG0741H [Halobacterium sp. NRC-1]
>gi|15789925|ref|NP_279749.1| hypothetical protein VNG0754C [Halobacterium sp. NRC-1]
>gi|15789936|ref|NP_279760.1| hypothetical protein VNG0767H [Halobacterium sp. NRC-1]
>gi|15789946|ref|NP_279770.1| hypothetical protein VNG0782H [Halobacterium sp. NRC-1]
>gi|15789947|ref|NP_279771.1| CDP-diacylglycerol-serine O-phosphatidyltransferase [Halobacterium sp. NRC-1]
>gi|15789950|ref|NP_279774.1| hypothetical protein VNG0788H [Halobacterium sp. NRC-1]
>gi|15789951|ref|NP_279775.1| hypothetical protein VNG0789C [Halobacterium sp. NRC-1]
>gi|15789953|ref|NP_279777.1| Htr6 [Halobacterium sp. NRC-1]
>gi|15789954|ref|NP_279778.1| ABC transporter (lipoprotein) [Halobacterium sp. NRC-1]
>gi|15789955|ref|NP_279779.1| HcpC [Halobacterium sp. NRC-1]
>gi|15789958|ref|NP_279782.1| hypothetical protein VNG0799C [Halobacterium sp. NRC-1]
>gi|15789959|ref|NP_279783.1| hypothetical protein VNG0800H [Halobacterium sp. NRC-1]
>gi|15789960|ref|NP_279784.1| hypothetical protein VNG0801C [Halobacterium sp. NRC-1]
>gi|15789962|ref|NP_279786.1| Htr4 [Halobacterium sp. NRC-1]
>gi|15789968|ref|NP_279792.1| hypothetical protein VNG0814C [Halobacterium sp. NRC-1]
>gi|15789976|ref|NP_279800.1| Gdb [Halobacterium sp. NRC-1]
>gi|15789977|ref|NP_279801.1| hypothetical protein VNG0825C [Halobacterium sp. NRC-1]
>gi|15789978|ref|NP_279802.1| hypothetical protein VNG0826C [Halobacterium sp. NRC-1]
>gi|15789992|ref|NP_279816.1| hypothetical protein VNG0846C [Halobacterium sp. NRC-1]
>gi|15789994|ref|NP_279818.1| hypothetical protein VNG0849C [Halobacterium sp. NRC-1]
>gi|15790000|ref|NP_279824.1| hypothetical protein VNG0858C [Halobacterium sp. NRC-1]
>gi|15790005|ref|NP_279829.1| PurL [Halobacterium sp. NRC-1]
>gi|15790007|ref|NP_279831.1| AsnA [Halobacterium sp. NRC-1]
>gi|15790013|ref|NP_279837.1| TraB [Halobacterium sp. NRC-1]
>gi|15790015|ref|NP_279839.1| PurM [Halobacterium sp. NRC-1]
>gi|15790028|ref|NP_279852.1| hypothetical protein VNG0892H [Halobacterium sp. NRC-1]
>gi|15790031|ref|NP_279855.1| RbsC1 [Halobacterium sp. NRC-1]
>gi|15790032|ref|NP_279856.1| RbsC2 [Halobacterium sp. NRC-1]
>gi|15790035|ref|NP_279859.1| Pmu2 [Halobacterium sp. NRC-1]
>gi|15790036|ref|NP_279860.1| hypothetical protein VNG0906H [Halobacterium sp. NRC-1]
>gi|15790044|ref|NP_279868.1| Ark [Halobacterium sp. NRC-1]
>gi|15790048|ref|NP_279872.1| hypothetical protein VNG0920H [Halobacterium sp. NRC-1]
>gi|15790054|ref|NP_279878.1| hypothetical protein VNG0927C [Halobacterium sp. NRC-1]

>gi|15790060|ref|NP_279884.1| hypothetical protein VNG0934H [Halobacterium sp. NRC-1]
>gi|15790062|ref|NP_279886.1| hypothetical protein VNG0936C [Halobacterium sp. NRC-1]
>gi|15790074|ref|NP_279898.1| hypothetical protein VNG0953C [Halobacterium sp. NRC-1]
>gi|15790075|ref|NP_279899.1| hypothetical protein VNG0954C [Halobacterium sp. NRC-1]
>gi|15790089|ref|NP_279913.1| CheA [Halobacterium sp. NRC-1]
>gi|15790093|ref|NP_279917.1| hypothetical protein VNG0978H [Halobacterium sp. NRC-1]
>gi|15790094|ref|NP_279918.1| hypothetical protein VNG0979H [Halobacterium sp. NRC-1]
>gi|15790095|ref|NP_279919.1| hypothetical protein VNG0981C [Halobacterium sp. NRC-1]
>gi|15790109|ref|NP_279933.1| Boa4 [Halobacterium sp. NRC-1]
>gi|15790112|ref|NP_279936.1| hypothetical protein VNG0999H [Halobacterium sp. NRC-1]
>gi|15790115|ref|NP_279939.1| hypothetical protein VNG1002H [Halobacterium sp. NRC-1]
>gi|15790124|ref|NP_279948.1| Htr13 [Halobacterium sp. NRC-1]
>gi|15790126|ref|NP_279950.1| hypothetical protein VNG1015H [Halobacterium sp. NRC-1]
>gi|15790134|ref|NP_279958.1| hypothetical protein VNG1026H [Halobacterium sp. NRC-1]
>gi|15790135|ref|NP_279959.1| TpiA [Halobacterium sp. NRC-1]
>gi|15790136|ref|NP_279960.1| hypothetical protein VNG1029C [Halobacterium sp. NRC-1]
>gi|15790137|ref|NP_279961.1| CDP-diacylglycerol-glycerol-3-phosphate 3-phosphatidyltransferase [Halobacterium sp. NRC-1]
>gi|15790142|ref|NP_279966.1| hypothetical protein VNG1036H [Halobacterium sp. NRC-1]
>gi|15790146|ref|NP_279970.1| hypothetical protein VNG1041H [Halobacterium sp. NRC-1]
>gi|15790168|ref|NP_279992.1| hypothetical protein VNG1069C [Halobacterium sp. NRC-1]
>gi|15790171|ref|NP_279995.1| Lfl1 [Halobacterium sp. NRC-1]
>gi|15790172|ref|NP_279996.1| YkfB2 [Halobacterium sp. NRC-1]
>gi|15790176|ref|NP_280000.1| 2-succinyl-6-hydroxy-2,4-cyclohexadiene-1-carboxylate synthase [Halobacterium sp. NRC-1]
>gi|15790181|ref|NP_280005.1| hypothetical protein VNG1087C [Halobacterium sp. NRC-1]
>gi|15790193|ref|NP_280017.1| hypothetical protein VNG1101C [Halobacterium sp. NRC-1]
>gi|15790194|ref|NP_280018.1| 50S ribosomal protein L12P [Halobacterium sp. NRC-1]
>gi|15790195|ref|NP_280019.1| 50S ribosomal protein L10P [Halobacterium sp. NRC-1]
>gi|15790196|ref|NP_280020.1| 50S ribosomal protein L1P [Halobacterium sp. NRC-1]
>gi|15790200|ref|NP_280024.1| hypothetical protein VNG1112H [Halobacterium sp. NRC-1]
>gi|15790205|ref|NP_280029.1| hypothetical protein VNG1119H [Halobacterium sp. NRC-1]
>gi|15790214|ref|NP_280038.1| 30S ribosomal protein S4P [Halobacterium sp. NRC-1]
>gi|15790222|ref|NP_280046.1| Eno [Halobacterium sp. NRC-1]
>gi|15790225|ref|NP_280049.1| Mvk [Halobacterium sp. NRC-1]
>gi|15790229|ref|NP_280053.1| hypothetical protein VNG1151H [Halobacterium sp. NRC-1]
>gi|15790231|ref|NP_280055.1| hypothetical protein VNG1154H [Halobacterium sp. NRC-1]
>gi|15790232|ref|NP_280056.1| Hsp3 [Halobacterium sp. NRC-1]
>gi|15790233|ref|NP_280057.1| 30S ribosomal protein S14P [Halobacterium sp. NRC-1]
>gi|15790242|ref|NP_280066.1| hypothetical protein VNG1169C [Halobacterium sp. NRC-1]
>gi|15790247|ref|NP_280071.1| Nop56/58 [Halobacterium sp. NRC-1]
>gi|15790250|ref|NP_280074.1| hypothetical protein VNG1179C [Halobacterium sp. NRC-1]
>gi|15790259|ref|NP_280083.1| hypothetical protein VNG1189H [Halobacterium sp. NRC-1]
>gi|15790269|ref|NP_280093.1| hypothetical protein VNG1202C [Halobacterium sp. NRC-1]
>gi|15790272|ref|NP_280096.1| hypothetical protein VNG1207C [Halobacterium sp. NRC-1]
>gi|15790275|ref|NP_280099.1| HutI [Halobacterium sp. NRC-1]
>gi|15790281|ref|NP_280105.1| hypothetical protein VNG1218C [Halobacterium sp. NRC-1]
>gi|15790286|ref|NP_280110.1| hypothetical protein VNG1226H [Halobacterium sp. NRC-1]
>gi|15790288|ref|NP_280112.1| hypothetical protein VNG1229H [Halobacterium sp. NRC-1]
>gi|15790293|ref|NP_280117.1| hypothetical protein VNG1235C [Halobacterium sp. NRC-1]
>gi|15790294|ref|NP_280118.1| hypothetical protein VNG1236C [Halobacterium sp. NRC-1]
>gi|15790298|ref|NP_280122.1| YhdG [Halobacterium sp. NRC-1]
>gi|15790301|ref|NP_280125.1| hypothetical protein VNG1245C [Halobacterium sp. NRC-1]
>gi|15790303|ref|NP_280127.1| ABC-type transport protein [Halobacterium sp. NRC-1]
>gi|15790304|ref|NP_280128.1| hypothetical protein VNG1249C [Halobacterium sp. NRC-1]
>gi|15790308|ref|NP_280132.1| hypothetical protein VNG1253C [Halobacterium sp. NRC-1]
>gi|15790319|ref|NP_280143.1| hypothetical protein VNG1268H [Halobacterium sp. NRC-1]
>gi|15790322|ref|NP_280146.1| hypothetical protein VNG1272C [Halobacterium sp. NRC-1]
>gi|15790326|ref|NP_280150.1| hypothetical protein VNG1279H [Halobacterium sp. NRC-1]
>gi|15790337|ref|NP_280161.1| SlyD [Halobacterium sp. NRC-1]
>gi|15790338|ref|NP_280162.1| hypothetical protein VNG1295H [Halobacterium sp. NRC-1]
>gi|15790341|ref|NP_280165.1| hypothetical protein VNG1299C [Halobacterium sp. NRC-1]
>gi|15790345|ref|NP_280169.1| hypothetical protein VNG1303C [Halobacterium sp. NRC-1]
>gi|15790346|ref|NP_280170.1| PurD [Halobacterium sp. NRC-1]
>gi|15790349|ref|NP_280173.1| SdhD [Halobacterium sp. NRC-1]
>gi|15790354|ref|NP_280178.1| hypothetical protein VNG1315H [Halobacterium sp. NRC-1]
>gi|15790355|ref|NP_280179.1| hypothetical protein VNG1317H [Halobacterium sp. NRC-1]
>gi|15790356|ref|NP_280180.1| hypothetical protein VNG1318H [Halobacterium sp. NRC-1]
>gi|15790357|ref|NP_280181.1| hypothetical protein VNG1319H [Halobacterium sp. NRC-1]
>gi|15790358|ref|NP_280182.1| Cbp [Halobacterium sp. NRC-1]
>gi|15790359|ref|NP_280183.1| hypothetical protein VNG1323C [Halobacterium sp. NRC-1]
>gi|15790363|ref|NP_280187.1| FprA [Halobacterium sp. NRC-1]

>gi|15790382|ref|NP_280206.1| hypothetical protein VNG1353C [Halobacterium sp. NRC-1]
>gi|15790383|ref|NP_280207.1| hypothetical protein VNG1355H [Halobacterium sp. NRC-1]
>gi|15790390|ref|NP_280214.1| hypothetical protein VNG1365C [Halobacterium sp. NRC-1]
>gi|15790394|ref|NP_280218.1| HemU [Halobacterium sp. NRC-1]
>gi|15790396|ref|NP_280220.1| hypothetical protein VNG1372C [Halobacterium sp. NRC-1]
>gi|15790404|ref|NP_280228.1| hypothetical protein VNG1382H [Halobacterium sp. NRC-1]
>gi|15790407|ref|NP_280231.1| YvoF [Halobacterium sp. NRC-1]
>gi|15790408|ref|NP_280232.1| hypothetical protein VNG1387H [Halobacterium sp. NRC-1]
>gi|15790409|ref|NP_280233.1| hypothetical protein VNG1388H [Halobacterium sp. NRC-1]
>gi|15790411|ref|NP_280235.1| hypothetical protein VNG1390H [Halobacterium sp. NRC-1]
>gi|15790413|ref|NP_280237.1| Htr9 [Halobacterium sp. NRC-1]
>gi|15790424|ref|NP_280248.1| hypothetical protein VNG1409C [Halobacterium sp. NRC-1]
>gi|15790425|ref|NP_280249.1| hypothetical protein VNG1410H [Halobacterium sp. NRC-1]
>gi|15790434|ref|NP_280258.1| hypothetical protein VNG1423H [Halobacterium sp. NRC-1]
>gi|15790446|ref|NP_280270.1| hypothetical protein VNG1440H [Halobacterium sp. NRC-1]
>gi|15790447|ref|NP_280271.1| Htr12 [Halobacterium sp. NRC-1]
>gi|15790448|ref|NP_280272.1| HisD [Halobacterium sp. NRC-1]
>gi|15790450|ref|NP_280274.1| hypothetical protein VNG1447H [Halobacterium sp. NRC-1]
>gi|15790457|ref|NP_280281.1| hypothetical protein VNG1455H [Halobacterium sp. NRC-1]
>gi|15790462|ref|NP_280286.1| hypothetical protein VNG1461H [Halobacterium sp. NRC-1]
>gi|15790463|ref|NP_280287.1| Cdc48a [Halobacterium sp. NRC-1]
>gi|15790466|ref|NP_280290.1| Brp [Halobacterium sp. NRC-1]
>gi|15790469|ref|NP_280293.1| hypothetical protein VNG1468H [Halobacterium sp. NRC-1]
>gi|15790471|ref|NP_280295.1| hypothetical protein VNG1471C [Halobacterium sp. NRC-1]
>gi|15790480|ref|NP_280304.1| Sat [Halobacterium sp. NRC-1]
>gi|15790482|ref|NP_280306.1| hypothetical protein VNG1483C [Halobacterium sp. NRC-1]
>gi|15790483|ref|NP_280307.1| hypothetical protein VNG1484H [Halobacterium sp. NRC-1]
>gi|15790488|ref|NP_280312.1| hypothetical protein VNG1492C [Halobacterium sp. NRC-1]
>gi|15790492|ref|NP_280316.1| CelM [Halobacterium sp. NRC-1]
>gi|15790501|ref|NP_280325.1| hypothetical protein VNG1511C [Halobacterium sp. NRC-1]
>gi|15790506|ref|NP_280330.1| hypothetical protein VNG1519H [Halobacterium sp. NRC-1]
>gi|15790508|ref|NP_280332.1| Htr8 [Halobacterium sp. NRC-1]
>gi|15790510|ref|NP_280334.1| hypothetical protein VNG1525C [Halobacterium sp. NRC-1]
>gi|15790514|ref|NP_280338.1| hypothetical protein VNG1530H [Halobacterium sp. NRC-1]
>gi|15790529|ref|NP_280353.1| CbiT [Halobacterium sp. NRC-1]
>gi|15790535|ref|NP_280359.1| hypothetical protein VNG1558H [Halobacterium sp. NRC-1]
>gi|15790538|ref|NP_280362.1| hypothetical protein VNG1562H [Halobacterium sp. NRC-1]
>gi|15790539|ref|NP_280363.1| hypothetical protein VNG1564H [Halobacterium sp. NRC-1]
>gi|15790540|ref|NP_280364.1| HmcA [Halobacterium sp. NRC-1]
>gi|15790542|ref|NP_280366.1| CbiC [Halobacterium sp. NRC-1]
>gi|15790544|ref|NP_280368.1| hypothetical protein VNG1570H [Halobacterium sp. NRC-1]
>gi|15790545|ref|NP_280369.1| hypothetical protein VNG1572C [Halobacterium sp. NRC-1]
>gi|15790549|ref|NP_280373.1| hypothetical protein VNG1577C [Halobacterium sp. NRC-1]
>gi|15790550|ref|NP_280374.1| hypothetical protein VNG1578H [Halobacterium sp. NRC-1]
>gi|15790551|ref|NP_280375.1| hypothetical protein VNG1580H [Halobacterium sp. NRC-1]
>gi|15790552|ref|NP_280376.1| hypothetical protein VNG1581C [Halobacterium sp. NRC-1]
>gi|15790554|ref|NP_280378.1| hypothetical protein VNG1583C [Halobacterium sp. NRC-1]
>gi|15790557|ref|NP_280381.1| hypothetical protein VNG1589C [Halobacterium sp. NRC-1]
>gi|15790560|ref|NP_280384.1| CysT2 [Halobacterium sp. NRC-1]
>gi|15790571|ref|NP_280395.1| hypothetical protein VNG1611C [Halobacterium sp. NRC-1]
>gi|15790577|ref|NP_280401.1| hypothetical protein VNG1619H [Halobacterium sp. NRC-1]
>gi|15790583|ref|NP_280407.1| hypothetical protein VNG1626C [Halobacterium sp. NRC-1]
>gi|15790587|ref|NP_280411.1| CbiQ [Halobacterium sp. NRC-1]
>gi|15790591|ref|NP_280415.1| hypothetical protein VNG1638H [Halobacterium sp. NRC-1]
>gi|15790593|ref|NP_280417.1| hypothetical protein VNG1641H [Halobacterium sp. NRC-1]
>gi|15790596|ref|NP_280420.1| hypothetical protein VNG1645H [Halobacterium sp. NRC-1]
>gi|15790599|ref|NP_280423.1| TrpF [Halobacterium sp. NRC-1]
>gi|15790609|ref|NP_280433.1| Htr1 [Halobacterium sp. NRC-1]
>gi|15790617|ref|NP_280441.1| hypothetical protein VNG1670C [Halobacterium sp. NRC-1]
>gi|15790618|ref|NP_280442.1| hypothetical protein VNG1672H [Halobacterium sp. NRC-1]
>gi|15790621|ref|NP_280445.1| hypothetical protein VNG1675H [Halobacterium sp. NRC-1]
>gi|15790623|ref|NP_280447.1| hypothetical protein VNG1678H [Halobacterium sp. NRC-1]
>gi|15790624|ref|NP_280448.1| hypothetical protein VNG1679H [Halobacterium sp. NRC-1]
>gi|15790627|ref|NP_280451.1| hypothetical protein VNG1682C [Halobacterium sp. NRC-1]
>gi|15790629|ref|NP_280453.1| Mch [Halobacterium sp. NRC-1]
>gi|15790638|ref|NP_280462.1| 30S ribosomal protein S3P [Halobacterium sp. NRC-1]
>gi|15790643|ref|NP_280467.1| 50S ribosomal protein L24P [Halobacterium sp. NRC-1]
>gi|15790649|ref|NP_280473.1| 50S ribosomal protein L32E [Halobacterium sp. NRC-1]
>gi|15790669|ref|NP_280493.1| hypothetical protein VNG1740C [Halobacterium sp. NRC-1]
>gi|15790670|ref|NP_280494.1| hypothetical protein VNG1743C [Halobacterium sp. NRC-1]

>gi|15790671|ref|NP_280495.1| hypothetical protein VNG1744H [Halobacterium sp. NRC-1]
>gi|15790675|ref|NP_280499.1| hypothetical protein VNG1751H [Halobacterium sp. NRC-1]
>gi|15790680|ref|NP_280504.1| hypothetical protein VNG1758H [Halobacterium sp. NRC-1]
>gi|15790681|ref|NP_280505.1| Htr7 [Halobacterium sp. NRC-1]
>gi|15790682|ref|NP_280506.1| Htr5 [Halobacterium sp. NRC-1]
>gi|15790683|ref|NP_280507.1| ProX [Halobacterium sp. NRC-1]
>gi|15790685|ref|NP_280509.1| Htr2 [Halobacterium sp. NRC-1]
>gi|15790695|ref|NP_280519.1| hypothetical protein VNG1775C [Halobacterium sp. NRC-1]
>gi|15790699|ref|NP_280523.1| hypothetical protein VNG1781C [Halobacterium sp. NRC-1]
>gi|15790700|ref|NP_280524.1| hypothetical protein VNG1782C [Halobacterium sp. NRC-1]
>gi|15790701|ref|NP_280525.1| hypothetical protein VNG1784C [Halobacterium sp. NRC-1]
>gi|15790710|ref|NP_280534.1| hypothetical protein VNG1794C [Halobacterium sp. NRC-1]
>gi|15790711|ref|NP_280535.1| ThrB [Halobacterium sp. NRC-1]
>gi|15790713|ref|NP_280537.1| hypothetical protein VNG1796H [Halobacterium sp. NRC-1]
>gi|15790716|ref|NP_280540.1| Hsp1 [Halobacterium sp. NRC-1]
>gi|15790717|ref|NP_280541.1| hypothetical protein VNG1802H [Halobacterium sp. NRC-1]
>gi|15790719|ref|NP_280543.1| hypothetical protein VNG1806H [Halobacterium sp. NRC-1]
>gi|15790720|ref|NP_280544.1| hypothetical protein VNG1807H [Halobacterium sp. NRC-1]
>gi|15790729|ref|NP_280553.1| MoaB [Halobacterium sp. NRC-1]
>gi|15790732|ref|NP_280556.1| hypothetical protein VNG1827H [Halobacterium sp. NRC-1]
>gi|15790737|ref|NP_280561.1| hypothetical protein VNG1833C [Halobacterium sp. NRC-1]
>gi|15790742|ref|NP_280566.1| hypothetical protein VNG1839H [Halobacterium sp. NRC-1]
>gi|15790744|ref|NP_280568.1| hypothetical protein VNG1842H [Halobacterium sp. NRC-1]
>gi|15790747|ref|NP_280571.1| hypothetical protein VNG1845C [Halobacterium sp. NRC-1]
>gi|15790748|ref|NP_280572.1| hypothetical protein VNG1846C [Halobacterium sp. NRC-1]
>gi|15790749|ref|NP_280573.1| hypothetical protein VNG1848H [Halobacterium sp. NRC-1]
>gi|15790750|ref|NP_280574.1| PchA [Halobacterium sp. NRC-1]
>gi|15790751|ref|NP_280575.1| hypothetical protein VNG1849H [Halobacterium sp. NRC-1]
>gi|15790754|ref|NP_280578.1| hypothetical protein VNG1852H [Halobacterium sp. NRC-1]
>gi|15790756|ref|NP_280580.1| Htr3 [Halobacterium sp. NRC-1]
>gi|15790758|ref|NP_280582.1| DeoC [Halobacterium sp. NRC-1]
>gi|15790760|ref|NP_280584.1| Cef [Halobacterium sp. NRC-1]
>gi|15790764|ref|NP_280588.1| PotC [Halobacterium sp. NRC-1]
>gi|15790768|ref|NP_280592.1| hypothetical protein VNG1872C [Halobacterium sp. NRC-1]
>gi|15790770|ref|NP_280594.1| hypothetical protein VNG1874C [Halobacterium sp. NRC-1]
>gi|15790771|ref|NP_280595.1| 3-hydroxy-3-methylglutaryl-coenzyme A reductase [Halobacterium sp. NRC-1]
>gi|15790777|ref|NP_280601.1| NadC [Halobacterium sp. NRC-1]
>gi|15790784|ref|NP_280608.1| hypothetical protein VNG1894C [Halobacterium sp. NRC-1]
>gi|15790791|ref|NP_280615.1| hypothetical protein VNG1902H [Halobacterium sp. NRC-1]
>gi|15790794|ref|NP_280618.1| hypothetical protein VNG1905C [Halobacterium sp. NRC-1]
>gi|15790795|ref|NP_280619.1| hypothetical protein VNG1906H [Halobacterium sp. NRC-1]
>gi|15790802|ref|NP_280626.1| hypothetical protein VNG1917H [Halobacterium sp. NRC-1]
>gi|15790804|ref|NP_280628.1| hypothetical protein VNG1919H [Halobacterium sp. NRC-1]
>gi|15790805|ref|NP_280629.1| hypothetical protein VNG1921H [Halobacterium sp. NRC-1]
>gi|15790816|ref|NP_280640.1| hypothetical protein VNG1935C [Halobacterium sp. NRC-1]
>gi|15790822|ref|NP_280646.1| hypothetical protein VNG1942H [Halobacterium sp. NRC-1]
>gi|15790830|ref|NP_280654.1| hypothetical protein VNG1952H [Halobacterium sp. NRC-1]
>gi|15790831|ref|NP_280655.1| hypothetical protein VNG1953C [Halobacterium sp. NRC-1]
>gi|15790838|ref|NP_280662.1| hypothetical protein VNG1963H [Halobacterium sp. NRC-1]
>gi|15790843|ref|NP_280667.1| GpdB [Halobacterium sp. NRC-1]
>gi|15790851|ref|NP_280675.1| hypothetical protein VNG1983H [Halobacterium sp. NRC-1]
>gi|15790853|ref|NP_280677.1| RNAase H [Halobacterium sp. NRC-1]
>gi|15790860|ref|NP_280684.1| Pgi [Halobacterium sp. NRC-1]
>gi|15790861|ref|NP_280685.1| hypothetical protein VNG1993H [Halobacterium sp. NRC-1]
>gi|15790873|ref|NP_280697.1| hypothetical protein VNG2008H [Halobacterium sp. NRC-1]
>gi|15790875|ref|NP_280699.1| ThiL [Halobacterium sp. NRC-1]
>gi|15790882|ref|NP_280706.1| hypothetical protein VNG2021C [Halobacterium sp. NRC-1]
>gi|15790884|ref|NP_280708.1| hypothetical protein VNG2024H [Halobacterium sp. NRC-1]
>gi|15790888|ref|NP_280712.1| hypothetical protein VNG2029H [Halobacterium sp. NRC-1]
>gi|15790894|ref|NP_280718.1| hypothetical protein VNG2037C [Halobacterium sp. NRC-1]
>gi|15790896|ref|NP_280720.1| hypothetical protein VNG2041H [Halobacterium sp. NRC-1]
>gi|15790911|ref|NP_280735.1| hypothetical protein VNG2064H [Halobacterium sp. NRC-1]
>gi|15790924|ref|NP_280748.1| hypothetical protein VNG2080C [Halobacterium sp. NRC-1]
>gi|15790933|ref|NP_280757.1| hypothetical protein VNG2091H [Halobacterium sp. NRC-1]
>gi|15790937|ref|NP_280761.1| hypothetical protein VNG2097C [Halobacterium sp. NRC-1]
>gi|15790938|ref|NP_280762.1| hypothetical protein VNG2098H [Halobacterium sp. NRC-1]
>gi|15790946|ref|NP_280770.1| ThrC3 [Halobacterium sp. NRC-1]
>gi|15790948|ref|NP_280772.1| hypothetical protein VNG2110C [Halobacterium sp. NRC-1]
>gi|15790955|ref|NP_280779.1| hypothetical protein VNG2119C [Halobacterium sp. NRC-1]
>gi|15790965|ref|NP_280789.1| MinD2 [Halobacterium sp. NRC-1]

>gi|15790968|ref|NP_280792.1| hypothetical protein VNG2133H [Halobacterium sp. NRC-1]
>gi|15790974|ref|NP_280798.1| AtpF [Halobacterium sp. NRC-1]
>gi|15790976|ref|NP_280800.1| AtpE [Halobacterium sp. NRC-1]
>gi|15790977|ref|NP_280801.1| AtpK [Halobacterium sp. NRC-1]
>gi|15790979|ref|NP_280803.1| hypothetical protein VNG2146H [Halobacterium sp. NRC-1]
>gi|15790981|ref|NP_280805.1| hypothetical protein VNG2148H [Halobacterium sp. NRC-1]
>gi|15790985|ref|NP_280809.1| hypothetical protein VNG2152C [Halobacterium sp. NRC-1]
>gi|15790986|ref|NP_280810.1| Fps [Halobacterium sp. NRC-1]
>gi|15790987|ref|NP_280811.1| hypothetical protein VNG2154C [Halobacterium sp. NRC-1]
>gi|15790990|ref|NP_280814.1| hypothetical protein VNG2157C [Halobacterium sp. NRC-1]
>gi|15790991|ref|NP_280815.1| OxrA [Halobacterium sp. NRC-1]
>gi|15790993|ref|NP_280817.1| hypothetical protein VNG2160C [Halobacterium sp. NRC-1]
>gi|15790994|ref|NP_280818.1| hypothetical protein VNG2162C [Halobacterium sp. NRC-1]
>gi|15790997|ref|NP_280821.1| hypothetical protein VNG2165H [Halobacterium sp. NRC-1]
>gi|15790999|ref|NP_280823.1| DNA binding protein eukaryotic-like [Halobacterium sp. NRC-1]
>gi|15791001|ref|NP_280825.1| Adh1 [Halobacterium sp. NRC-1]
>gi|15791010|ref|NP_280834.1| hypothetical protein VNG2179H [Halobacterium sp. NRC-1]
>gi|15791014|ref|NP_280838.1| hypothetical protein VNG2183H [Halobacterium sp. NRC-1]
>gi|15791017|ref|NP_280841.1| Hit1 [Halobacterium sp. NRC-1]
>gi|15791021|ref|NP_280845.1| hypothetical protein VNG2191H [Halobacterium sp. NRC-1]
>gi|15791024|ref|NP_280848.1| HcpB [Halobacterium sp. NRC-1]
>gi|15791026|ref|NP_280850.1| hypothetical protein VNG2199H [Halobacterium sp. NRC-1]
>gi|15791027|ref|NP_280851.1| Cpx [Halobacterium sp. NRC-1]
>gi|15791028|ref|NP_280852.1| hypothetical protein VNG2202H [Halobacterium sp. NRC-1]
>gi|15791030|ref|NP_280854.1| hypothetical protein VNG2204H [Halobacterium sp. NRC-1]
>gi|15791033|ref|NP_280857.1| hypothetical protein VNG2207H [Halobacterium sp. NRC-1]
>gi|15791038|ref|NP_280862.1| DNA damage-inducible protein [Halobacterium sp. NRC-1]
>gi|15791042|ref|NP_280866.1| Dsa [Halobacterium sp. NRC-1]
>gi|15791050|ref|NP_280874.1| hypothetical protein VNG2231C [Halobacterium sp. NRC-1]
>gi|15791062|ref|NP_280886.1| hypothetical protein VNG2244H [Halobacterium sp. NRC-1]
>gi|15791063|ref|NP_280887.1| hypothetical protein VNG2246H [Halobacterium sp. NRC-1]
>gi|15791065|ref|NP_280889.1| hypothetical protein VNG2248H [Halobacterium sp. NRC-1]
>gi|15791069|ref|NP_280893.1| hypothetical protein VNG2253H [Halobacterium sp. NRC-1]
>gi|15791071|ref|NP_280895.1| hypothetical protein VNG2255C [Halobacterium sp. NRC-1]
>gi|15791075|ref|NP_280899.1| hypothetical protein VNG2260H [Halobacterium sp. NRC-1]
>gi|15791080|ref|NP_280904.1| TatC2 [Halobacterium sp. NRC-1]
>gi|15791084|ref|NP_280908.1| Rpi [Halobacterium sp. NRC-1]
>gi|15791085|ref|NP_280909.1| hypothetical protein VNG2273H [Halobacterium sp. NRC-1]
>gi|15791088|ref|NP_280912.1| hypothetical protein VNG2277H [Halobacterium sp. NRC-1]
>gi|15791093|ref|NP_280917.1| AlaS [Halobacterium sp. NRC-1]
>gi|15791095|ref|NP_280919.1| hypothetical protein VNG2285C [Halobacterium sp. NRC-1]
>gi|15791096|ref|NP_280920.1| MamA [Halobacterium sp. NRC-1]
>gi|15791101|ref|NP_280925.1| hypothetical protein VNG2292H [Halobacterium sp. NRC-1]
>gi|15791105|ref|NP_280929.1| hypothetical protein VNG2297H [Halobacterium sp. NRC-1]
>gi|15791114|ref|NP_280938.1| hypothetical protein VNG2307C [Halobacterium sp. NRC-1]
>gi|15791120|ref|NP_280944.1| hypothetical protein VNG2314H [Halobacterium sp. NRC-1]
>gi|15791121|ref|NP_280945.1| hypothetical protein VNG2315H [Halobacterium sp. NRC-1]
>gi|15791122|ref|NP_280946.1| hypothetical protein VNG2316C [Halobacterium sp. NRC-1]
>gi|15791128|ref|NP_280952.1| hypothetical protein VNG2324H [Halobacterium sp. NRC-1]
>gi|15791130|ref|NP_280954.1| hypothetical protein VNG2328H [Halobacterium sp. NRC-1]
>gi|15791131|ref|NP_280955.1| hypothetical protein VNG2329C [Halobacterium sp. NRC-1]
>gi|15791135|ref|NP_280959.1| hypothetical protein VNG2333C [Halobacterium sp. NRC-1]
>gi|15791151|ref|NP_280975.1| hypothetical protein VNG2353H [Halobacterium sp. NRC-1]
>gi|15791158|ref|NP_280982.1| hypothetical protein VNG2366C [Halobacterium sp. NRC-1]
>gi|15791160|ref|NP_280984.1| hypothetical protein VNG2370C [Halobacterium sp. NRC-1]
>gi|15791162|ref|NP_280986.1| hypothetical protein VNG2369C [Halobacterium sp. NRC-1]
>gi|15791163|ref|NP_280987.1| hypothetical protein VNG2371C [Halobacterium sp. NRC-1]
>gi|15791168|ref|NP_280992.1| NosY [Halobacterium sp. NRC-1]
>gi|15791172|ref|NP_280996.1| UvrC [Halobacterium sp. NRC-1]
>gi|15791176|ref|NP_281000.1| hypothetical protein VNG2386C [Halobacterium sp. NRC-1]
>gi|15791177|ref|NP_281001.1| hypothetical protein VNG2387H [Halobacterium sp. NRC-1]
>gi|15791179|ref|NP_281003.1| hypothetical protein VNG2392H [Halobacterium sp. NRC-1]
>gi|15791186|ref|NP_281010.1| hypothetical protein VNG2399H [Halobacterium sp. NRC-1]
>gi|15791191|ref|NP_281015.1| hypothetical protein VNG2406C [Halobacterium sp. NRC-1]
>gi|15791195|ref|NP_281019.1| hypothetical protein VNG2412H [Halobacterium sp. NRC-1]
>gi|15791198|ref|NP_281022.1| hypothetical protein VNG2415H [Halobacterium sp. NRC-1]
>gi|15791201|ref|NP_281025.1| AspC1 [Halobacterium sp. NRC-1]
>gi|15791202|ref|NP_281026.1| hypothetical protein VNG2419C [Halobacterium sp. NRC-1]
>gi|15791206|ref|NP_281030.1| SerB [Halobacterium sp. NRC-1]
>gi|15791207|ref|NP_281031.1| SerA1 [Halobacterium sp. NRC-1]

>gi|15791209|ref|NP_281033.1| D-lactate dehydrogenase [Halobacterium sp. NRC-1]
>gi|15791213|ref|NP_281037.1| hypothetical protein VNG2433H [Halobacterium sp. NRC-1]
>gi|15791214|ref|NP_281038.1| ArgH [Halobacterium sp. NRC-1]
>gi|15791216|ref|NP_281040.1| hypothetical protein VNG2439H [Halobacterium sp. NRC-1]
>gi|15791217|ref|NP_281041.1| hypothetical protein VNG2440H [Halobacterium sp. NRC-1]
>gi|15791221|ref|NP_281045.1| hypothetical protein VNG2444C [Halobacterium sp. NRC-1]
>gi|15791222|ref|NP_281046.1| hypothetical protein VNG2445C [Halobacterium sp. NRC-1]
>gi|15791224|ref|NP_281048.1| Lta [Halobacterium sp. NRC-1]
>gi|15791229|ref|NP_281053.1| hypothetical protein VNG2455C [Halobacterium sp. NRC-1]
>gi|15791231|ref|NP_281055.1| hypothetical protein VNG2458C [Halobacterium sp. NRC-1]
>gi|15791234|ref|NP_281058.1| Dpa [Halobacterium sp. NRC-1]
>gi|15791235|ref|NP_281059.1| hypothetical protein VNG2465C [Halobacterium sp. NRC-1]
>gi|15791239|ref|NP_281063.1| hypothetical protein VNG2468C [Halobacterium sp. NRC-1]
>gi|15791250|ref|NP_281074.1| PstA1 [Halobacterium sp. NRC-1]
>gi|15791256|ref|NP_281080.1| hypothetical protein VNG2495H [Halobacterium sp. NRC-1]
>gi|15791268|ref|NP_281092.1| hypothetical protein VNG2510H [Halobacterium sp. NRC-1]
>gi|15791274|ref|NP_281098.1| hypothetical protein VNG2516C [Halobacterium sp. NRC-1]
>gi|15791275|ref|NP_281099.1| hypothetical protein VNG2518C [Halobacterium sp. NRC-1]
>gi|15791287|ref|NP_281111.1| hypothetical protein VNG2532H [Halobacterium sp. NRC-1]
>gi|15791289|ref|NP_281113.1| hypothetical protein VNG2536C [Halobacterium sp. NRC-1]
>gi|15791290|ref|NP_281114.1| hypothetical protein VNG2534C [Halobacterium sp. NRC-1]
>gi|15791300|ref|NP_281124.1| FhuG [Halobacterium sp. NRC-1]
>gi|15791303|ref|NP_281127.1| hypothetical protein VNG2554H [Halobacterium sp. NRC-1]
>gi|15791304|ref|NP_281128.1| hypothetical protein VNG2555C [Halobacterium sp. NRC-1]
>gi|15791318|ref|NP_281142.1| hypothetical protein VNG2576H [Halobacterium sp. NRC-1]
>gi|15791320|ref|NP_281144.1| Idr1 [Halobacterium sp. NRC-1]
>gi|15791322|ref|NP_281146.1| hypothetical protein VNG2581H [Halobacterium sp. NRC-1]
>gi|15791325|ref|NP_281149.1| hypothetical protein VNG2585H [Halobacterium sp. NRC-1]
>gi|15791328|ref|NP_281152.1| hypothetical protein VNG2589C [Halobacterium sp. NRC-1]
>gi|15791329|ref|NP_281153.1| hypothetical protein VNG2591C [Halobacterium sp. NRC-1]
>gi|15791330|ref|NP_281154.1| hypothetical protein VNG2593H [Halobacterium sp. NRC-1]
>gi|15791332|ref|NP_281156.1| hypothetical protein VNG2594C [Halobacterium sp. NRC-1]
>gi|15791342|ref|NP_281166.1| hypothetical protein VNG2607C [Halobacterium sp. NRC-1]
>gi|15791354|ref|NP_281178.1| hypothetical protein VNG2622H [Halobacterium sp. NRC-1]
>gi|15791356|ref|NP_281180.1| hypothetical protein VNG2625C [Halobacterium sp. NRC-1]
>gi|15791361|ref|NP_281185.1| hypothetical protein VNG2631H [Halobacterium sp. NRC-1]
>gi|15791362|ref|NP_281186.1| YusZ2 [Halobacterium sp. NRC-1]
>gi|15791366|ref|NP_281190.1| hypothetical protein VNG2637H [Halobacterium sp. NRC-1]
>gi|15791369|ref|NP_281193.1| GcvT2 [Halobacterium sp. NRC-1]
>gi|15791371|ref|NP_281195.1| hypothetical protein VNG2642H [Halobacterium sp. NRC-1]
>gi|15791396|ref|NP_281220.1| hypothetical protein VNG2677H [Halobacterium sp. NRC-1]
>gi|16554462|ref|NP_444186.1| hypothetical protein VNG0287a [Halobacterium sp. NRC-1]
>gi|16554471|ref|NP_444195.1| Multisubunit Na+/H+ antiporter, MnhG subunit [Halobacterium sp. NRC-1]
>gi|16554473|ref|NP_444197.1| Predicted RNA methylase [Halobacterium sp. NRC-1]
>gi|16554476|ref|NP_444200.1| p-nitrophenyl phosphatase [Halobacterium sp. NRC-1]
>gi|16554477|ref|NP_444201.1| hypothetical protein VNG0892a [Halobacterium sp. NRC-1]
>gi|16554494|ref|NP_444218.1| Iron (III) ABC transporter ATPase [Halobacterium sp. NRC-1]
>gi|16554504|ref|NP_444228.1| Membrane transporter of cationic drugs [Halobacterium sp. NRC-1]
>gi|16554507|ref|NP_444231.1| Enoyl-CoA hydratase [Halobacterium sp. NRC-1]
>gi|16554509|ref|NP_444233.1| 3,4-dihydroxy-2-butanone 4-phosphate synthase [Halobacterium sp. NRC-1]
>gi|16554511|ref|NP_444235.1| Mannose-1-phosphate guanylyltransferase [Halobacterium sp. NRC-1]
>gi|16554514|ref|NP_444238.1| hypothetical protein VNG2298a [Halobacterium sp. NRC-1]
>gi|16554517|ref|NP_444241.1| Permease, similar to cation transporters [Halobacterium sp. NRC-1]
>gi|16554519|ref|NP_444243.1| hypothetical protein VNG2480Hm [Halobacterium sp. NRC-1]
>gi|16119995|ref|NP_395583.1| GvpK protein, cluster A [Halobacterium sp. NRC-1]
>gi|16119998|ref|NP_395586.1| GvpH protein, cluster A [Halobacterium sp. NRC-1]
>gi|16120004|ref|NP_395592.1| GvpC protein, cluster A [Halobacterium sp. NRC-1]
>gi|16120024|ref|NP_395612.1| CydB_1 [Halobacterium sp. NRC-1]
>gi|16120030|ref|NP_395618.1| PhoT1_1 [Halobacterium sp. NRC-1]
>gi|16120074|ref|NP_395662.1| hypothetical protein VNG6120H [Halobacterium sp. NRC-1]
>gi|16120079|ref|NP_395667.1| hypothetical protein VNG6127H [Halobacterium sp. NRC-1]
>gi|16120086|ref|NP_395674.1| hypothetical protein VNG6134H [Halobacterium sp. NRC-1]
>gi|16120113|ref|NP_395701.1| hypothetical protein VNG6165H [Halobacterium sp. NRC-1]
>gi|16120118|ref|NP_395706.1| hypothetical protein VNG6173C [Halobacterium sp. NRC-1]
>gi|16120132|ref|NP_395720.1| hypothetical protein VNG6188H [Halobacterium sp. NRC-1]
>gi|16120138|ref|NP_395726.1| PhoT2 [Halobacterium sp. NRC-1]
>gi|16120140|ref|NP_395728.1| hypothetical protein VNG6198H [Halobacterium sp. NRC-1]
>gi|16120144|ref|NP_395732.1| hypothetical protein VNG6204H [Halobacterium sp. NRC-1]
>gi|16120162|ref|NP_395750.1| hypothetical protein VNG6226H [Halobacterium sp. NRC-1]
>gi|16120164|ref|NP_395752.1| GvpK protein, cluster B [Halobacterium sp. NRC-1]

>gi|16120166|ref|NP_395754.1| GvpI protein, cluster B [Halobacterium sp. NRC-1]
>gi|16120173|ref|NP_395761.1| GvpC protein, cluster B [Halobacterium sp. NRC-1]
>gi|16120177|ref|NP_395765.1| PhoT3 [Halobacterium sp. NRC-1]
>gi|16120178|ref|NP_395766.1| hypothetical protein VNG6251H [Halobacterium sp. NRC-1]
>gi|16120180|ref|NP_395768.1| hypothetical protein VNG6255C [Halobacterium sp. NRC-1]
>gi|16120184|ref|NP_395772.1| YocR [Halobacterium sp. NRC-1]
>gi|16120185|ref|NP_395773.1| ABC transporter, permease protein [Halobacterium sp. NRC-1]
>gi|16120193|ref|NP_395781.1| hypothetical protein VNG6275H [Halobacterium sp. NRC-1]
>gi|16120230|ref|NP_395818.1| hypothetical protein VNG6322H [Halobacterium sp. NRC-1]
>gi|16120234|ref|NP_395822.1| hypothetical protein VNG6327H [Halobacterium sp. NRC-1]
>gi|16120240|ref|NP_395828.1| hypothetical protein VNG6335H [Halobacterium sp. NRC-1]
>gi|16120245|ref|NP_395833.1| hypothetical protein VNG6343H [Halobacterium sp. NRC-1]
>gi|16120253|ref|NP_395841.1| hypothetical protein VNG6353H [Halobacterium sp. NRC-1]
>gi|16120257|ref|NP_395845.1| hypothetical protein VNG6359H [Halobacterium sp. NRC-1]
>gi|16120265|ref|NP_395853.1| hypothetical protein VNG6368H [Halobacterium sp. NRC-1]
>gi|16120286|ref|NP_395874.1| hypothetical protein VNG6400H [Halobacterium sp. NRC-1]
>gi|16120290|ref|NP_395878.1| hypothetical protein VNG6404H [Halobacterium sp. NRC-1]
>gi|16120304|ref|NP_395892.1| hypothetical protein VNG6427H [Halobacterium sp. NRC-1]
>gi|16120313|ref|NP_395901.1| hypothetical protein VNG6441H [Halobacterium sp. NRC-1]
>gi|16120332|ref|NP_395920.1| PhoT1_2 [Halobacterium sp. NRC-1]
>gi|16120338|ref|NP_395926.1| CydB_2 [Halobacterium sp. NRC-1]
>gi|10803564|ref|NP_045962.1| hypothetical protein VNG7017 [Halobacterium salinarum NRC-1]
>gi|10803567|ref|NP_045965.1| hypothetical protein VNG7020 [Halobacterium salinarum NRC-1]
>gi|10803573|ref|NP_045971.1| hypothetical protein VNG7026 [Halobacterium salinarum NRC-1]
>gi|10803587|ref|NP_045985.1| hypothetical protein VNG7040 [Halobacterium salinarum NRC-1]
>gi|10803594|ref|NP_045992.1| hypothetical protein VNG7047 [Halobacterium salinarum NRC-1]
>gi|10803597|ref|NP_045995.1| hypothetical protein VNG7050 [Halobacterium salinarum NRC-1]
>gi|10803604|ref|NP_046002.1| hypothetical protein VNG7057 [Halobacterium salinarum NRC-1]
>gi|10803635|ref|NP_046033.1| hypothetical protein VNG7088 [Halobacterium salinarum NRC-1]
>gi|10803637|ref|NP_046035.1| hypothetical protein VNG7090 [Halobacterium salinarum NRC-1]
>gi|10803660|ref|NP_046058.1| hypothetical protein VNG7113 [Halobacterium salinarum NRC-1]
>gi|10803668|ref|NP_046066.1| hypothetical protein VNG7121 [Halobacterium salinarum NRC-1]
>gi|10803672|ref|NP_046070.1| hypothetical protein VNG7125 [Halobacterium salinarum NRC-1]
>gi|10803677|ref|NP_046075.1| hypothetical protein VNG7130 [Halobacterium salinarum NRC-1]
>gi|10803678|ref|NP_046076.1| hypothetical protein VNG7131 [Halobacterium salinarum NRC-1]
>gi|10803680|ref|NP_046078.1| hypothetical protein VNG7133 [Halobacterium salinarum NRC-1]
>gi|10803699|ref|NP_046097.1| hypothetical protein VNG7152 [Halobacterium salinarum NRC-1]
>gi|10803706|ref|NP_046104.1| hypothetical protein VNG7159 [Halobacterium salinarum NRC-1]
>gi|10803709|ref|NP_046107.1| hypothetical protein VNG7162 [Halobacterium salinarum NRC-1]
>gi|10803716|ref|NP_046114.1| hypothetical protein VNG7169 [Halobacterium salinarum NRC-1]
>gi|55822010|ref|YP_140451.1| IS861, transposase (orf1), IS3 family, truncated [Streptococcus thermophilus CNRZ1066]
>gi|55822013|ref|YP_140454.1| rod shape-determining protein MreC [Streptococcus thermophilus CNRZ1066]
>gi|55822015|ref|YP_140456.1| glucan binding protein [Streptococcus thermophilus CNRZ1066]
>gi|55822092|ref|YP_140533.1| integrase/recombinase, phage integrase family, truncated [Streptococcus thermophilus CNRZ1066]
>gi|55822108|ref|YP_140549.1| heat shock protein, chaperonin [Streptococcus thermophilus CNRZ1066]
>gi|55822122|ref|YP_140563.1| DNA-directed RNA polymerase delta subunit [Streptococcus thermophilus CNRZ1066]
>gi|55822137|ref|YP_140578.1| 1,6-alpha-glucanhydrolase (dextranase), truncated [Streptococcus thermophilus CNRZ1066]
>gi|55822143|ref|YP_140584.1| hypothetical protein str0155 [Streptococcus thermophilus CNRZ1066]
>gi|55822191|ref|YP_140632.1| chaperonin, 10 kDa [Streptococcus thermophilus CNRZ1066]
>gi|55822196|ref|YP_140637.1| hypothetical protein str0208 [Streptococcus thermophilus CNRZ1066]
>gi|55822209|ref|YP_140650.1| hypothetical protein str0221 [Streptococcus thermophilus CNRZ1066]
>gi|55822221|ref|YP_140662.1| hypothetical protein str0233 [Streptococcus thermophilus CNRZ1066]
>gi|55822275|ref|YP_140716.1| cobalt ABC transporter permease protein [Streptococcus thermophilus CNRZ1066]
>gi|55822293|ref|YP_140734.1| hypothetical protein str0316 [Streptococcus thermophilus CNRZ1066]
>gi|55822309|ref|YP_140750.1| mannose PTS system component IIC [Streptococcus thermophilus CNRZ1066]
>gi|55822321|ref|YP_140762.1| translation initiation factor IF-2 [Streptococcus thermophilus CNRZ1066]
>gi|55822338|ref|YP_140779.1| branched-chain amino acid ABC transporter permease protein [Streptococcus thermophilus CNRZ1066]
>gi|55822365|ref|YP_140806.1| biotoin carboxyl carrier protein [Streptococcus thermophilus CNRZ1066]
>gi|55822381|ref|YP_140822.1| PTS fructose-specific enzyme IIABC components, truncated [Streptococcus thermophilus CNRZ1066]
>gi|55822385|ref|YP_140826.1| hypothetical protein str0410 [Streptococcus thermophilus CNRZ1066]
>gi|55822420|ref|YP_140861.1| surface immunogenic protein, truncated [Streptococcus thermophilus CNRZ1066]
>gi|55822424|ref|YP_140865.1| hypothetical protein str0450 [Streptococcus thermophilus CNRZ1066]
>gi|55822458|ref|YP_140899.1| proton-translocating ATPase, epsilon subunit [Streptococcus thermophilus CNRZ1066]
>gi|55822487|ref|YP_140928.1| hypothetical protein str0516 [Streptococcus thermophilus CNRZ1066]
>gi|55822492|ref|YP_140933.1| prolipoprotein signal peptidase (spase II) [Streptococcus thermophilus CNRZ1066]
>gi|55822507|ref|YP_140948.1| 50S ribosomal protein L7/L12 [Streptococcus thermophilus CNRZ1066]
>gi|55822620|ref|YP_141061.1| exfoliative exotoxin B, putative [Streptococcus thermophilus CNRZ1066]
>gi|55822663|ref|YP_141104.1| hypothetical protein str0694 [Streptococcus thermophilus CNRZ1066]
>gi|55822700|ref|YP_141141.1| cell division protein [Streptococcus thermophilus CNRZ1066]
>gi|55822765|ref|YP_141206.1| 30S ribosomal protein S20 [Streptococcus thermophilus CNRZ1066]

>gi|55822844|ref|YP_141285.1| alcohol dehydrogenase I, truncated [Streptococcus thermophilus CNRZ1066]
>gi|55822880|ref|YP_141321.1| hypothetical protein str0922 [Streptococcus thermophilus CNRZ1066]
>gi|55822931|ref|YP_141372.1| hypothetical protein str0978 [Streptococcus thermophilus CNRZ1066]
>gi|55822998|ref|YP_141439.1| hypothetical protein str1047 [Streptococcus thermophilus CNRZ1066]
>gi|55823025|ref|YP_141466.1| hypothetical protein str1075 [Streptococcus thermophilus CNRZ1066]
>gi|55823058|ref|YP_141499.1| hypothetical protein str1136 [Streptococcus thermophilus CNRZ1066]
>gi|55823064|ref|YP_141505.1| hypothetical protein str1142 [Streptococcus thermophilus CNRZ1066]
>gi|55823099|ref|YP_141540.1| conserved hypothetical protein, voltage-gated chloride channel family [Streptococcus thermophilus CNRZ1066]
>gi|55823102|ref|YP_141543.1| flavodoxin [Streptococcus thermophilus CNRZ1066]
>gi|55823114|ref|YP_141555.1| hypothetical protein str1195 [Streptococcus thermophilus CNRZ1066]
>gi|55823165|ref|YP_141606.1| hypothetical protein str1246 [Streptococcus thermophilus CNRZ1066]
>gi|55823223|ref|YP_141664.1| biotin synthase [Streptococcus thermophilus CNRZ1066]
>gi|55823253|ref|YP_141694.1| nucleobase:cation symporter for xanthine, truncated [Streptococcus thermophilus CNRZ1066]
>gi|55823256|ref|YP_141697.1| MATE efflux family protein (Na(+)/drug antiporter) [Streptococcus thermophilus CNRZ1066]
>gi|55823272|ref|YP_141713.1| hypothetical protein str1360 [Streptococcus thermophilus CNRZ1066]
>gi|55823298|ref|YP_141739.1| hypothetical protein str1386 [Streptococcus thermophilus CNRZ1066]
>gi|55823395|ref|YP_141836.1| 30S ribosomal protein S21 [Streptococcus thermophilus CNRZ1066]
>gi|55823408|ref|YP_141849.1| hypothetical protein str1503 [Streptococcus thermophilus CNRZ1066]
>gi|55823455|ref|YP_141896.1| hypothetical protein, citrulline cluster-linked gene [Streptococcus thermophilus CNRZ1066]
>gi|55823466|ref|YP_141907.1| late competence protein required for DNA binding and uptake [Streptococcus thermophilus CNRZ1066]
>gi|55823486|ref|YP_141927.1| amino acid (glutamine) ABC transporter permease protein [Streptococcus thermophilus CNRZ1066]
>gi|55823506|ref|YP_141947.1| hypothetical protein str1602 [Streptococcus thermophilus CNRZ1066]
>gi|55823543|ref|YP_141984.1| hypothetical protein str1639 [Streptococcus thermophilus CNRZ1066]
>gi|55823567|ref|YP_142008.1| hypothetical protein str1664 [Streptococcus thermophilus CNRZ1066]
>gi|55823579|ref|YP_142020.1| pore-forming peptide, putative bacteriocin [Streptococcus thermophilus CNRZ1066]
>gi|55823598|ref|YP_142039.1| hypothetical protein str1704 [Streptococcus thermophilus CNRZ1066]
>gi|55823601|ref|YP_142042.1| hypothetical protein str1707 [Streptococcus thermophilus CNRZ1066]
>gi|55823603|ref|YP_142044.1| hypothetical protein str1709 [Streptococcus thermophilus CNRZ1066]
>gi|55823615|ref|YP_142056.1| hypothetical protein str1721 [Streptococcus thermophilus CNRZ1066]
>gi|55823645|ref|YP_142086.1| single strand binding protein [Streptococcus thermophilus CNRZ1066]
>gi|55823676|ref|YP_142117.1| IS861, transposase (orf1), IS3 family, truncated [Streptococcus thermophilus CNRZ1066]
>gi|55823727|ref|YP_142168.1| 2,3,4,5-tetrahydropyridine-2-carboxylate N-succinyltransferase, putative [Streptococcus thermophilus CNRZ1066]
>gi|55823783|ref|YP_142224.1| hypothetical protein str1896 [Streptococcus thermophilus CNRZ1066]
>gi|55823813|ref|YP_142254.1| 50S ribosomal protein L29 [Streptococcus thermophilus CNRZ1066]
>gi|55823820|ref|YP_142261.1| 50S ribosomal protein L4 [Streptococcus thermophilus CNRZ1066]
>gi|55823864|ref|YP_142305.1| conserved hypothetical protein, truncated [Streptococcus thermophilus CNRZ1066]
>gi|55823868|ref|YP_142309.1| hypothetical protein str1982 [Streptococcus thermophilus CNRZ1066]
>gi|55823872|ref|YP_142313.1| hypothetical protein str1987 [Streptococcus thermophilus CNRZ1066]
>gi|55823876|ref|YP_142317.1| hypothetical protein str1991 [Streptococcus thermophilus CNRZ1066]
>gi|55823878|ref|YP_142319.1| hypothetical protein str1993 [Streptococcus thermophilus CNRZ1066]
>gi|45439927|ref|NP_991466.1| putative membrane protein [Yersinia pestis biovar Medievalis str. 91001]
>gi|45439965|ref|NP_991504.1| hypothetical protein YP0102 [Yersinia pestis biovar Medievalis str. 91001]
>gi|45439972|ref|NP_991511.1| cell division protein [Yersinia pestis biovar Medievalis str. 91001]
>gi|45440010|ref|NP_991549.1| putative membrane protein [Yersinia pestis biovar Medievalis str. 91001]
>gi|45440013|ref|NP_991552.1| hypothetical protein YP0151 [Yersinia pestis biovar Medievalis str. 91001]
>gi|45440058|ref|NP_991597.1| hypothetical protein YP0198 [Yersinia pestis biovar Medievalis str. 91001]
>gi|45440067|ref|NP_991606.1| 50S ribosomal protein L3 [Yersinia pestis biovar Medievalis str. 91001]
>gi|45440075|ref|NP_991614.1| 50S ribosomal protein L29 [Yersinia pestis biovar Medievalis str. 91001]
>gi|45440126|ref|NP_991665.1| dihydrolipoamide acetyltransferase component of pyruvate dehydrogenase complex [Yersinia pestis biovar Medievalis str. 91001]
>gi|45440127|ref|NP_991666.1| hypothetical protein YP0267 [Yersinia pestis biovar Medievalis str. 91001]
>gi|45440154|ref|NP_991693.1| ferrichrome transport system permease protein FhuB [Yersinia pestis biovar Medievalis str. 91001]
>gi|45440156|ref|NP_991695.1| putative chloride channel protein [Yersinia pestis biovar Medievalis str. 91001]
>gi|45440170|ref|NP_991709.1| superoxide dismutase [Cu-Zn] precursor [Yersinia pestis biovar Medievalis str. 91001]
>gi|45440206|ref|NP_991745.1| Acetyltransferases (the isoleucine patch superfamily) [Yersinia pestis biovar Medievalis str. 91001]
>gi|45440213|ref|NP_991752.1| putative sugar ABC transporter, permease protein [Yersinia pestis biovar Medievalis str. 91001]
>gi|45440237|ref|NP_991776.1| putative regulatory protein [Yersinia pestis biovar Medievalis str. 91001]
>gi|45440241|ref|NP_991780.1| putative membrane protein [Yersinia pestis biovar Medievalis str. 91001]
>gi|45440273|ref|NP_991812.1| hypothetical protein YP0418 [Yersinia pestis biovar Medievalis str. 91001]
>gi|45440274|ref|NP_991813.1| putative type III secretion system component [Yersinia pestis biovar Medievalis str. 91001]
>gi|45440277|ref|NP_991816.1| type III secretion system apparatus protein [Yersinia pestis biovar Medievalis str. 91001]
>gi|45440290|ref|NP_991829.1| hemin transport system permease protein HmuU [Yersinia pestis biovar Medievalis str. 91001]
>gi|45440327|ref|NP_991866.1| hypothetical protein YP0473 [Yersinia pestis biovar Medievalis str. 91001]
>gi|45440334|ref|NP_991873.1| single-strand binding protein [Yersinia pestis biovar Medievalis str. 91001]
>gi|45440344|ref|NP_991883.1| hypothetical protein YP0490 [Yersinia pestis biovar Medievalis str. 91001]
>gi|45440358|ref|NP_991897.1| putative membrane protein [Yersinia pestis biovar Medievalis str. 91001]
>gi|45440362|ref|NP_991901.1| putative lipoprotein [Yersinia pestis biovar Medievalis str. 91001]
>gi|45440385|ref|NP_991924.1| putative membrane protein [Yersinia pestis biovar Medievalis str. 91001]
>gi|45440438|ref|NP_991977.1| protein-export membrane protein [Yersinia pestis biovar Medievalis str. 91001]
>gi|45440449|ref|NP_991988.1| inducible ATP-independent RNA helicase [Yersinia pestis biovar Medievalis str. 91001]

>gi|45440485|ref|NP_992024.1| putative ABC transporter, ATP-binding protein [Yersinia pestis biovar Medievalis str. 91001]
>gi|45440513|ref|NP_992052.1| putative tRNA/rRNA methyltransferase [Yersinia pestis biovar Medievalis str. 91001]
>gi|45440575|ref|NP_992114.1| ATP-dependent dsDNA exonuclease [Yersinia pestis biovar Medievalis str. 91001]
>gi|45440683|ref|NP_992222.1| putative membrane protein [Yersinia pestis biovar Medievalis str. 91001]
>gi|45440684|ref|NP_992223.1| Membrane protease subunits, stomatin/prohibitin homologs [Yersinia pestis biovar Medievalis str. 91001]
>gi|45440685|ref|NP_992224.1| putative thioredoxin [Yersinia pestis biovar Medievalis str. 91001]
>gi|45440689|ref|NP_992228.1| putative permease [Yersinia pestis biovar Medievalis str. 91001]
>gi|45440779|ref|NP_992318.1| putative ABC transport integral membrane subunit [Yersinia pestis biovar Medievalis str. 91001]
>gi|45440785|ref|NP_992324.1| putative Branched-chain amino acid transport system, permease component [Yersinia pestis biovar Medievalis str. 91001]
>gi|45440786|ref|NP_992325.1| putative Branched-chain amino acid transport system, permease component [Yersinia pestis biovar Medievalis str. 91001]
>gi|45440793|ref|NP_992332.1| putative membrane protein [Yersinia pestis biovar Medievalis str. 91001]
>gi|45440802|ref|NP_992341.1| hypothetical protein YP0964 [Yersinia pestis biovar Medievalis str. 91001]
>gi|45440805|ref|NP_992344.1| hypothetical protein YP0969 [Yersinia pestis biovar Medievalis str. 91001]
>gi|45440806|ref|NP_992345.1| Molecular chaperones (contain C-terminal Zn finger domain) [Yersinia pestis biovar Medievalis str. 91001]
>gi|45440807|ref|NP_992346.1| hypothetical protein YP0971 [Yersinia pestis biovar Medievalis str. 91001]
>gi|45440854|ref|NP_992393.1| putative membrane protein [Yersinia pestis biovar Medievalis str. 91001]
>gi|45440864|ref|NP_992403.1| hypothetical protein YP1030 [Yersinia pestis biovar Medievalis str. 91001]
>gi|45440867|ref|NP_992406.1| TolA colicin import membrane protein [Yersinia pestis biovar Medievalis str. 91001]
>gi|45440947|ref|NP_992486.1| sec-independent protein translocase protein [Yersinia pestis biovar Medievalis str. 91001]
>gi|45440962|ref|NP_992501.1| sugar transport system permease protein [Yersinia pestis biovar Medievalis str. 91001]
>gi|45440998|ref|NP_992537.1| putative lipoprotein protein [Yersinia pestis biovar Medievalis str. 91001]
>gi|45441032|ref|NP_992571.1| hypothetical protein YP1205 [Yersinia pestis biovar Medievalis str. 91001]
>gi|45441033|ref|NP_992572.1| hypothetical protein YP1206 [Yersinia pestis biovar Medievalis str. 91001]
>gi|45441086|ref|NP_992625.1| putrescine transport system permease protein [Yersinia pestis biovar Medievalis str. 91001]
>gi|45441154|ref|NP_992693.1| putative cell division inhibitor [Yersinia pestis biovar Medievalis str. 91001]
>gi|45441203|ref|NP_992742.1| hypothetical protein YP1378 [Yersinia pestis biovar Medievalis str. 91001]
>gi|45441249|ref|NP_992788.1| putative iron-siderophore transport system, transmembrane component [Yersinia pestis biovar Medievalis str. 91001]
>gi|45441279|ref|NP_992818.1| hypothetical protein YP1456 [Yersinia pestis biovar Medievalis str. 91001]
>gi|45441304|ref|NP_992843.1| hypothetical protein YP1481 [Yersinia pestis biovar Medievalis str. 91001]
>gi|45441308|ref|NP_992847.1| hypothetical protein YP1485 [Yersinia pestis biovar Medievalis str. 91001]
>gi|45441310|ref|NP_992849.1| hypothetical protein YP1487 [Yersinia pestis biovar Medievalis str. 91001]
>gi|45441341|ref|NP_992880.1| hypothetical protein YP1521 [Yersinia pestis biovar Medievalis str. 91001]
>gi|45441385|ref|NP_992924.1| flagellar hook-length control protein FliK [Yersinia pestis biovar Medievalis str. 91001]
>gi|45441389|ref|NP_992928.1| flagellar biosynthesis protein [Yersinia pestis biovar Medievalis str. 91001]
>gi|45441391|ref|NP_992930.1| flagellar biosynthetic protein FliQ [Yersinia pestis biovar Medievalis str. 91001]
>gi|45441426|ref|NP_992965.1| hypothetical protein YP1609 [Yersinia pestis biovar Medievalis str. 91001]
>gi|45441472|ref|NP_993011.1| putative signal transducer [Yersinia pestis biovar Medievalis str. 91001]
>gi|45441498|ref|NP_993037.1| hypothetical protein YP1684 [Yersinia pestis biovar Medievalis str. 91001]
>gi|45441537|ref|NP_993076.1| hypothetical protein YP1725 [Yersinia pestis biovar Medievalis str. 91001]
>gi|45441541|ref|NP_993080.1| ProP effector homologue [Yersinia pestis biovar Medievalis str. 91001]
>gi|45441544|ref|NP_993083.1| putative fimbrial protein [Yersinia pestis biovar Medievalis str. 91001]
>gi|45441564|ref|NP_993103.1| hypothetical protein YP1753 [Yersinia pestis biovar Medievalis str. 91001]
>gi|45441593|ref|NP_993132.1| hypothetical protein YP1782 [Yersinia pestis biovar Medievalis str. 91001]
>gi|45441604|ref|NP_993143.1| chemotaxis MotB protein [Yersinia pestis biovar Medievalis str. 91001]
>gi|45441686|ref|NP_993225.1| putative membrane protein [Yersinia pestis biovar Medievalis str. 91001]
>gi|45441753|ref|NP_993292.1| putative membrane protein [Yersinia pestis biovar Medievalis str. 91001]
>gi|45441775|ref|NP_993314.1| DNA-binding protein HLP-II (HU, BH2, HD, NS) [Yersinia pestis biovar Medievalis str. 91001]
>gi|45441793|ref|NP_993332.1| TonB protein [Yersinia pestis biovar Medievalis str. 91001]
>gi|45441828|ref|NP_993367.1| osmotically inducible lipoprotein B precursor [Yersinia pestis biovar Medievalis str. 91001]
>gi|45441838|ref|NP_993377.1| putative membrane protein [Yersinia pestis biovar Medievalis str. 91001]
>gi|45441842|ref|NP_993381.1| putative iron-sulfur binding NADH dehydrogenase [Yersinia pestis biovar Medievalis str. 91001]
>gi|45441845|ref|NP_993384.1| putative membrane protein [Yersinia pestis biovar Medievalis str. 91001]
>gi|45441898|ref|NP_993437.1| putative acid shock protein [Yersinia pestis biovar Medievalis str. 91001]
>gi|45441910|ref|NP_993449.1| putative lipoprotein [Yersinia pestis biovar Medievalis str. 91001]
>gi|45441935|ref|NP_993474.1| hypothetical protein YP2143 [Yersinia pestis biovar Medievalis str. 91001]
>gi|45441951|ref|NP_993490.1| putative lipoprotein [Yersinia pestis biovar Medievalis str. 91001]
>gi|45441963|ref|NP_993502.1| hypothetical protein YP2171 [Yersinia pestis biovar Medievalis str. 91001]
>gi|45441973|ref|NP_993512.1| major outer membrane lipoprotein [Yersinia pestis biovar Medievalis str. 91001]
>gi|45441993|ref|NP_993532.1| putative membrane protein [Yersinia pestis biovar Medievalis str. 91001]
>gi|45442003|ref|NP_993542.1| hypothetical protein YP2213 [Yersinia pestis biovar Medievalis str. 91001]
>gi|45442009|ref|NP_993548.1| 50S ribosomal protein L35 [Yersinia pestis biovar Medievalis str. 91001]
>gi|45442031|ref|NP_993570.1| putative lipoprotein [Yersinia pestis biovar Medievalis str. 91001]
>gi|45442038|ref|NP_993577.1| DNA polymerase III, delta' subunit [Yersinia pestis biovar Medievalis str. 91001]
>gi|45442053|ref|NP_993592.1| ribonuclease E [Yersinia pestis biovar Medievalis str. 91001]
>gi|45442059|ref|NP_993598.1| putative LuxR-family regulatory protein [Yersinia pestis biovar Medievalis str. 91001]
>gi|45442146|ref|NP_993685.1| NADH dehydrogenase i chain k [Yersinia pestis biovar Medievalis str. 91001]
>gi|45442173|ref|NP_993712.1| Predicted phosphoesterase [Yersinia pestis biovar Medievalis str. 91001]
>gi|45442183|ref|NP_993722.1| putative membrane protein [Yersinia pestis biovar Medievalis str. 91001]

>gi|45442199|ref|NP_993738.1| putative membrane protein [Yersinia pestis biovar Medievalis str. 91001]
>gi|45442204|ref|NP_993743.1| hypothetical protein YP2415 [Yersinia pestis biovar Medievalis str. 91001]
>gi|45442216|ref|NP_993755.1| putative heme exporter protein D [Yersinia pestis biovar Medievalis str. 91001]
>gi|45442218|ref|NP_993757.1| heme exporter protein B [Yersinia pestis biovar Medievalis str. 91001]
>gi|45442228|ref|NP_993767.1| hypothetical protein YP2441 [Yersinia pestis biovar Medievalis str. 91001]
>gi|45442239|ref|NP_993778.1| putative acid shock protein [Yersinia pestis biovar Medievalis str. 91001]
>gi|45442273|ref|NP_993812.1| putative membrane protein [Yersinia pestis biovar Medievalis str. 91001]
>gi|45442286|ref|NP_993825.1| putative lactam utilization protein [Yersinia pestis biovar Medievalis str. 91001]
>gi|45442300|ref|NP_993839.1| sigma E factor regulatory protein [Yersinia pestis biovar Medievalis str. 91001]
>gi|45442323|ref|NP_993862.1| hypothetical protein YP2542 [Yersinia pestis biovar Medievalis str. 91001]
>gi|45442356|ref|NP_993895.1| Uncharacterized protein involved in chromosome partitioning [Yersinia pestis biovar Medievalis str. 91001]
>gi|45442365|ref|NP_993904.1| Hemolysin-coregulated protein (uncharacterized) [Yersinia pestis biovar Medievalis str. 91001]
>gi|45442383|ref|NP_993922.1| hypothetical protein YP2606 [Yersinia pestis biovar Medievalis str. 91001]
>gi|45442416|ref|NP_993955.1| putative sugar transporter [Yersinia pestis biovar Medievalis str. 91001]
>gi|45442421|ref|NP_993960.1| putative membrane protein [Yersinia pestis biovar Medievalis str. 91001]
>gi|45442426|ref|NP_993965.1| hypothetical protein YP2650 [Yersinia pestis biovar Medievalis str. 91001]
>gi|45442444|ref|NP_993983.1| putative membrane protein [Yersinia pestis biovar Medievalis str. 91001]
>gi|45442453|ref|NP_993992.1| putative membrane protein [Yersinia pestis biovar Medievalis str. 91001]
>gi|45442501|ref|NP_994040.1| putative membrane protein [Yersinia pestis biovar Medievalis str. 91001]
>gi|45442502|ref|NP_994041.1| hypothetical protein YP2730 [Yersinia pestis biovar Medievalis str. 91001]
>gi|45442505|ref|NP_994044.1| putative membrane protein [Yersinia pestis biovar Medievalis str. 91001]
>gi|45442510|ref|NP_994049.1| exodeoxyribonuclease VII large subunit [Yersinia pestis biovar Medievalis str. 91001]
>gi|45442524|ref|NP_994063.1| putative autotransporter protein [Yersinia pestis biovar Medievalis str. 91001]
>gi|45442564|ref|NP_994103.1| UDP-3-o-[3-hydroxymyristoyl] glucosamine N-acyltransferase [Yersinia pestis biovar Medievalis str. 91001]
>gi|45442589|ref|NP_994128.1| Uncharacterized small membrane protein [Yersinia pestis biovar Medievalis str. 91001]
>gi|45442646|ref|NP_994185.1| putative prepilin peptidase dependent protein [Yersinia pestis biovar Medievalis str. 91001]
>gi|45442653|ref|NP_994192.1| putative membrane protein [Yersinia pestis biovar Medievalis str. 91001]
>gi|45442655|ref|NP_994194.1| hypothetical protein YP2889 [Yersinia pestis biovar Medievalis str. 91001]
>gi|45442658|ref|NP_994197.1| hypothetical protein YP2892 [Yersinia pestis biovar Medievalis str. 91001]
>gi|45442676|ref|NP_994215.1| putative membrane protein [Yersinia pestis biovar Medievalis str. 91001]
>gi|45442757|ref|NP_994296.1| putative DedA-family membrane protein [Yersinia pestis biovar Medievalis str. 91001]
>gi|45442759|ref|NP_994298.1| TonB complex protein [Yersinia pestis biovar Medievalis str. 91001]
>gi|45442793|ref|NP_994332.1| flagellar basal-body rod protein FlgB [Yersinia pestis biovar Medievalis str. 91001]
>gi|45442809|ref|NP_994348.1| putative flagellar hook-length control protein [Yersinia pestis biovar Medievalis str. 91001]
>gi|45442865|ref|NP_994404.1| Ribosomal protein L7/L12 [Yersinia pestis biovar Medievalis str. 91001]
>gi|45442866|ref|NP_994405.1| 50S ribosomal protein L10 [Yersinia pestis biovar Medievalis str. 91001]
>gi|45442870|ref|NP_994409.1| preprotein translocase SecE subunit [Yersinia pestis biovar Medievalis str. 91001]
>gi|45442897|ref|NP_994436.1| putative sugar transport system permease [Yersinia pestis biovar Medievalis str. 91001]
>gi|45442947|ref|NP_994486.1| uroporphyrinogen III methylase [Yersinia pestis biovar Medievalis str. 91001]
>gi|45442981|ref|NP_994520.1| putative membrane protein [Yersinia pestis biovar Medievalis str. 91001]
>gi|45442983|ref|NP_994522.1| putative membrane protein [Yersinia pestis biovar Medievalis str. 91001]
>gi|45442987|ref|NP_994526.1| cell division protein [Yersinia pestis biovar Medievalis str. 91001]
>gi|45442995|ref|NP_994534.1| high-affinity branched-chain amino acid transport system, permease protein [Yersinia pestis biovar Medievalis str. 91001]
>gi|45443018|ref|NP_994557.1| putative membrane protein [Yersinia pestis biovar Medievalis str. 91001]
>gi|45443019|ref|NP_994558.1| putative DNA recombination protein [Yersinia pestis biovar Medievalis str. 91001]
>gi|45443024|ref|NP_994563.1| Sec-independent protein translocase protein TatB [Yersinia pestis biovar Medievalis str. 91001]
>gi|45443061|ref|NP_994600.1| putative membrane protein [Yersinia pestis biovar Medievalis str. 91001]
>gi|45443091|ref|NP_994630.1| GntP family permease [Yersinia pestis biovar Medievalis str. 91001]
>gi|45443111|ref|NP_994650.1| dipeptide transport system permease protein [Yersinia pestis biovar Medievalis str. 91001]
>gi|45443118|ref|NP_994657.1| Signal transduction histidine kinase, glucose-6-phosphate specific [Yersinia pestis biovar Medievalis str. 91001]
>gi|45443130|ref|NP_994669.1| putative membrane protein [Yersinia pestis biovar Medievalis str. 91001]
>gi|45443160|ref|NP_994699.1| putative autotransporter protein [Yersinia pestis biovar Medievalis str. 91001]
>gi|45443162|ref|NP_994701.1| putative antigenic leucine-rich repeat protein [Yersinia pestis biovar Medievalis str. 91001]
>gi|45443180|ref|NP_994719.1| putative virG protein [Yersinia pestis biovar Medievalis str. 91001]
>gi|45443194|ref|NP_994733.1| putative membrane protein [Yersinia pestis biovar Medievalis str. 91001]
>gi|45443230|ref|NP_994769.1| hypothetical protein YP3491 [Yersinia pestis biovar Medievalis str. 91001]
>gi|45443237|ref|NP_994776.1| putative membrane protein [Yersinia pestis biovar Medievalis str. 91001]
>gi|45443279|ref|NP_994818.1| hypothetical protein YP3542 [Yersinia pestis biovar Medievalis str. 91001]
>gi|45443325|ref|NP_994864.1| putative membrane protein [Yersinia pestis biovar Medievalis str. 91001]
>gi|45443333|ref|NP_994872.1| hypothetical protein YP3598 [Yersinia pestis biovar Medievalis str. 91001]
>gi|45443404|ref|NP_994943.1| hypothetical protein YP3672 [Yersinia pestis biovar Medievalis str. 91001]
>gi|45443443|ref|NP_994982.1| chaperone protein DnaJ [Yersinia pestis biovar Medievalis str. 91001]
>gi|45443478|ref|NP_995017.1| putative Na+ dependent nucleosidetransporter-family protein [Yersinia pestis biovar Medievalis str. 91001]
>gi|45443487|ref|NP_995026.1| Permeases of the major facilitator superfamily [Yersinia pestis biovar Medievalis str. 91001]
>gi|45443500|ref|NP_995039.1| putative membrane protein [Yersinia pestis biovar Medievalis str. 91001]
>gi|45443502|ref|NP_995041.1| putative ABC transporter permease protein [Yersinia pestis biovar Medievalis str. 91001]
>gi|45443598|ref|NP_995137.1| hypothetical protein YP3869 [Yersinia pestis biovar Medievalis str. 91001]
>gi|45443614|ref|NP_995153.1| putative membrane protein [Yersinia pestis biovar Medievalis str. 91001]
>gi|45443616|ref|NP_995155.1| biotin carboxyl carrier protein of acetyl-CoA carboxylase [Yersinia pestis biovar Medievalis str. 91001]

>gi|45443630|ref|NP_995169.1| outer membrane lipoprotein [Yersinia pestis biovar Medievalis str. 91001]
>gi|45443689|ref|NP_995228.1| putative membrane protein [Yersinia pestis biovar Medievalis str. 91001]
>gi|45443710|ref|NP_995249.1| putative lipoprotein [Yersinia pestis biovar Medievalis str. 91001]
>gi|45443735|ref|NP_995274.1| hypothetical protein YP4012 [Yersinia pestis biovar Medievalis str. 91001]
>gi|45443755|ref|NP_995294.1| ATP synthase subunit B protein [Yersinia pestis biovar Medievalis str. 91001]
>gi|45443756|ref|NP_995295.1| ATP synthase subunit C protein [Yersinia pestis biovar Medievalis str. 91001]
>gi|45478549|ref|NP_995376.1| putative type III secretion protein YscY [Yersinia pestis biovar Medievalis str. 91001]
>gi|45478559|ref|NP_995386.1| putative targeted effector protein YopM [Yersinia pestis biovar Medievalis str. 91001]
>gi|45478570|ref|NP_995397.1| hypothetical protein pCD76 [Yersinia pestis biovar Medievalis str. 91001]
>gi|45478577|ref|NP_995404.1| hypothetical protein pCD84 [Yersinia pestis biovar Medievalis str. 91001]
>gi|45478582|ref|NP_995409.1| hypothetical protein pCD90 [Yersinia pestis biovar Medievalis str. 91001]
>gi|45478593|ref|NP_995449.1| phage lambda-related protein [Yersinia pestis biovar Medievalis str. 91001]
>gi|45478611|ref|NP_995467.1| hypothetical protein pMT023 [Yersinia pestis biovar Medievalis str. 91001]
>gi|45478655|ref|NP_995511.1| hypothetical protein pMT069 [Yersinia pestis biovar Medievalis str. 91001]
>gi|45478662|ref|NP_995518.1| putative porphyrin biosynthetic protein [Yersinia pestis biovar Medievalis str. 91001]
>gi|45478667|ref|NP_995523.1| F1 capsule antigen [Yersinia pestis biovar Medievalis str. 91001]
>gi|45478691|ref|NP_995547.1| hypothetical protein pMT107 [Yersinia pestis biovar Medievalis str. 91001]
>gi|45478708|ref|NP_995564.1| hypothetical protein pMT124 [Yersinia pestis biovar Medievalis str. 91001]
>gi|28209877|ref|NP_780821.1| conserved membrane protein [Clostridium tetani E88]
>gi|28209883|ref|NP_780827.1| LSU ribosomal protein L9P [Clostridium tetani E88]
>gi|28209916|ref|NP_780860.1| cytochrome c-type biogenesis protein ccdA [Clostridium tetani E88]
>gi|28209927|ref|NP_780871.1| hypothetical protein CTC00156 [Clostridium tetani E88]
>gi|28209942|ref|NP_780886.1| hypothetical protein CTC00172 [Clostridium tetani E88]
>gi|28209947|ref|NP_780891.1| transporter [Clostridium tetani E88]
>gi|28209989|ref|NP_780933.1| Tpl protein [Clostridium tetani E88]
>gi|28210019|ref|NP_780963.1| conserved membrane protein [Clostridium tetani E88]
>gi|28210076|ref|NP_781020.1| stage II sporulation protein D [Clostridium tetani E88]
>gi|28210108|ref|NP_781052.1| serine acetyltransferase [Clostridium tetani E88]
>gi|28210115|ref|NP_781059.1| hypothetical protein CTC00359 [Clostridium tetani E88]
>gi|28210215|ref|NP_781159.1| putative S-layer protein [Clostridium tetani E88]
>gi|28210229|ref|NP_781173.1| putative mechanosensitive ion-channel [Clostridium tetani E88]
>gi|28210240|ref|NP_781184.1| internalin A-like protein/putative S-layer protein [Clostridium tetani E88]
>gi|28210241|ref|NP_781185.1| internalin A-like protein/putative S-layer protein [Clostridium tetani E88]
>gi|28210245|ref|NP_781189.1| hypothetical protein CTC00500 [Clostridium tetani E88]
>gi|28210262|ref|NP_781206.1| cwp66 homolog/N-acetylmuramoyl-L-alanine amidase [Clostridium tetani E88]
>gi|28210285|ref|NP_781229.1| hypothetical protein CTC00546 [Clostridium tetani E88]
>gi|28210291|ref|NP_781235.1| hypothetical protein CTC00552 [Clostridium tetani E88]
>gi|28210293|ref|NP_781237.1| hypothetical protein CTC00555 [Clostridium tetani E88]
>gi|28210315|ref|NP_781259.1| exonuclease sbcC [Clostridium tetani E88]
>gi|28210336|ref|NP_781280.1| hypothetical protein CTC00602 [Clostridium tetani E88]
>gi|28210374|ref|NP_781318.1| hypothetical protein CTC00646 [Clostridium tetani E88]
>gi|28210376|ref|NP_781320.1| hypothetical protein CTC00648 [Clostridium tetani E88]
>gi|28210384|ref|NP_781328.1| hypothetical protein CTC00658 [Clostridium tetani E88]
>gi|28210393|ref|NP_781337.1| hypothetical protein CTC00669 [Clostridium tetani E88]
>gi|28210405|ref|NP_781349.1| hypothetical protein CTC00681 [Clostridium tetani E88]
>gi|28210406|ref|NP_781350.1| hypothetical protein CTC00682 [Clostridium tetani E88]
>gi|28210445|ref|NP_781389.1| cobalt transport protein cbiQ [Clostridium tetani E88]
>gi|28210475|ref|NP_781419.1| putative peptidoglycan N-acetylglucosamine deacetylase [Clostridium tetani E88]
>gi|28210481|ref|NP_781425.1| membrane-associated protein [Clostridium tetani E88]
>gi|28210483|ref|NP_781427.1| hypothetical protein CTC00763 [Clostridium tetani E88]
>gi|28210489|ref|NP_781433.1| putative surface/cell-adhesion protein [Clostridium tetani E88]
>gi|28210490|ref|NP_781434.1| putative surface/cell-adhesion protein [Clostridium tetani E88]
>gi|28210516|ref|NP_781460.1| hypothetical protein CTC00799 [Clostridium tetani E88]
>gi|28210517|ref|NP_781461.1| hypothetical protein CTC00800 [Clostridium tetani E88]
>gi|28210518|ref|NP_781462.1| hypothetical protein CTC00801 [Clostridium tetani E88]
>gi|28210519|ref|NP_781463.1| hypothetical protein CTC00802 [Clostridium tetani E88]
>gi|28210525|ref|NP_781469.1| hypothetical protein CTC00808 [Clostridium tetani E88]
>gi|28210564|ref|NP_781508.1| ethanolamine utilization protein [Clostridium tetani E88]
>gi|28210650|ref|NP_781594.1| transporter [Clostridium tetani E88]
>gi|28210686|ref|NP_781630.1| membrane spanning protein [Clostridium tetani E88]
>gi|28210699|ref|NP_781643.1| V-type sodium ATP synthase subunit G [Clostridium tetani E88]
>gi|28210702|ref|NP_781646.1| V-type sodium ATP synthase subunit E [Clostridium tetani E88]
>gi|28210719|ref|NP_781663.1| hypothetical protein CTC01014 [Clostridium tetani E88]
>gi|28210756|ref|NP_781700.1| permease [Clostridium tetani E88]
>gi|28210770|ref|NP_781714.1| hypothetical protein CTC01068 [Clostridium tetani E88]
>gi|28210784|ref|NP_781728.1| hypothetical protein CTC01083 [Clostridium tetani E88]
>gi|28210820|ref|NP_781764.1| putative sporulation sigma-E factor processing peptidase [Clostridium tetani E88]
>gi|28210881|ref|NP_781825.1| tonB-dependent receptor protein [Clostridium tetani E88]
>gi|28210882|ref|NP_781826.1| hypothetical protein CTC01193 [Clostridium tetani E88]
>gi|28210883|ref|NP_781827.1| hypothetical protein CTC01194 [Clostridium tetani E88]

>gi|28210932|ref|NP_781876.1| chromosome segregation protein smc2 [Clostridium tetani E88]
>gi|28210934|ref|NP_781878.1| signal recognition particle associated protein [Clostridium tetani E88]
>gi|28210973|ref|NP_781917.1| CDP-diacylglycerol--glycerol-3-phosphate 3-phosphatidyltransferase [Clostridium tetani E88]
>gi|28210982|ref|NP_781926.1| hypothetical protein CTC01297 [Clostridium tetani E88]
>gi|28211030|ref|NP_781974.1| membrane spanning protein [Clostridium tetani E88]
>gi|28211108|ref|NP_782052.1| membrane spanning protein [Clostridium tetani E88]
>gi|28211114|ref|NP_782058.1| hypothetical protein CTC01437 [Clostridium tetani E88]
>gi|28211132|ref|NP_782076.1| hypothetical protein CTC01455 [Clostridium tetani E88]
>gi|28211136|ref|NP_782080.1| membrane associated protein [Clostridium tetani E88]
>gi|28211140|ref|NP_782084.1| hypothetical protein CTC01464 [Clostridium tetani E88]
>gi|28211172|ref|NP_782116.1| hypothetical protein CTC01497 [Clostridium tetani E88]
>gi|28211178|ref|NP_782122.1| phospholipase-subfamily protein [Clostridium tetani E88]
>gi|28211188|ref|NP_782132.1| hypothetical protein CTC01517 [Clostridium tetani E88]
>gi|28211198|ref|NP_782142.1| hypothetical protein CTC01528 [Clostridium tetani E88]
>gi|28211210|ref|NP_782154.1| phage pre-neck appendage-like protein [Clostridium tetani E88]
>gi|28211213|ref|NP_782157.1| hypothetical protein CTC01546 [Clostridium tetani E88]
>gi|28211214|ref|NP_782158.1| phage protein [Clostridium tetani E88]
>gi|28211216|ref|NP_782160.1| hypothetical protein CTC01548 [Clostridium tetani E88]
>gi|28211221|ref|NP_782165.1| hypothetical protein CTC01553 [Clostridium tetani E88]
>gi|28211227|ref|NP_782171.1| hypothetical protein CTC01559 [Clostridium tetani E88]
>gi|28211231|ref|NP_782175.1| hypothetical protein CTC01564 [Clostridium tetani E88]
>gi|28211237|ref|NP_782181.1| DNA repair protein recN [Clostridium tetani E88]
>gi|28211245|ref|NP_782189.1| methenyltetrahydrofolate cyclohydrolase [Clostridium tetani E88]
>gi|28211254|ref|NP_782198.1| hypothetical protein CTC01590 [Clostridium tetani E88]
>gi|28211259|ref|NP_782203.1| hypothetical protein CTC01595 [Clostridium tetani E88]
>gi|28211260|ref|NP_782204.1| hypothetical protein CTC01596 [Clostridium tetani E88]
>gi|28211285|ref|NP_782229.1| hypothetical protein CTC01623 [Clostridium tetani E88]
>gi|28211299|ref|NP_782243.1| trk system potassium uptake protein trkA [Clostridium tetani E88]
>gi|28211303|ref|NP_782247.1| GTP-binding protein [Clostridium tetani E88]
>gi|28211309|ref|NP_782253.1| flagellar basal-body rod protein flgG [Clostridium tetani E88]
>gi|28211326|ref|NP_782270.1| putative flagellar hook-length control protein [Clostridium tetani E88]
>gi|28211329|ref|NP_782273.1| putative fliH protein [Clostridium tetani E88]
>gi|28211338|ref|NP_782282.1| hypothetical protein CTC01684 [Clostridium tetani E88]
>gi|28211340|ref|NP_782284.1| hypothetical protein CTC01686 [Clostridium tetani E88]
>gi|28211342|ref|NP_782286.1| hypothetical protein CTC01688 [Clostridium tetani E88]
>gi|28211344|ref|NP_782288.1| hypothetical protein CTC01690 [Clostridium tetani E88]
>gi|28211350|ref|NP_782294.1| transcriptional regulatory protein [Clostridium tetani E88]
>gi|28211370|ref|NP_782314.1| hypothetical protein CTC01716 [Clostridium tetani E88]
>gi|28211380|ref|NP_782324.1| putative chemotaxis protein cheW [Clostridium tetani E88]
>gi|28211391|ref|NP_782335.1| hypothetical protein CTC01740 [Clostridium tetani E88]
>gi|28211423|ref|NP_782367.1| permease [Clostridium tetani E88]
>gi|28211469|ref|NP_782413.1| murein hydrolase export regulator [Clostridium tetani E88]
>gi|28211472|ref|NP_782416.1| hypothetical protein CTC01832 [Clostridium tetani E88]
>gi|28211506|ref|NP_782450.1| hypothetical protein CTC01870 [Clostridium tetani E88]
>gi|28211507|ref|NP_782451.1| putative N-acetylmuramoyl-L-alanine amidase [Clostridium tetani E88]
>gi|28211509|ref|NP_782453.1| hypothetical protein CTC01874 [Clostridium tetani E88]
>gi|28211570|ref|NP_782514.1| hypothetical protein CTC01942 [Clostridium tetani E88]
>gi|28211595|ref|NP_782539.1| transcriptional regulatory protein [Clostridium tetani E88]
>gi|28211596|ref|NP_782540.1| membrane associated protein [Clostridium tetani E88]
>gi|28211613|ref|NP_782557.1| membrane spanning protein [Clostridium tetani E88]
>gi|28211617|ref|NP_782561.1| hypothetical protein CTC01992 [Clostridium tetani E88]
>gi|28211629|ref|NP_782573.1| hypothetical protein CTC02006 [Clostridium tetani E88]
>gi|28211654|ref|NP_782598.1| putative grpE protein [Clostridium tetani E88]
>gi|28211680|ref|NP_782624.1| LSU ribosomal protein L21P [Clostridium tetani E88]
>gi|28211695|ref|NP_782639.1| hypothetical protein CTC02077 [Clostridium tetani E88]
>gi|28211742|ref|NP_782686.1| hypothetical protein CTC02127 [Clostridium tetani E88]
>gi|28211757|ref|NP_782701.1| hypothetical protein CTC02142 [Clostridium tetani E88]
>gi|28211777|ref|NP_782721.1| putative ethanolamine utilization protein [Clostridium tetani E88]
>gi|28211818|ref|NP_782762.1| hypothetical protein CTC02214 [Clostridium tetani E88]
>gi|28211822|ref|NP_782766.1| hypothetical protein CTC02218 [Clostridium tetani E88]
>gi|28211864|ref|NP_782808.1| acetyltransferase [Clostridium tetani E88]
>gi|28211875|ref|NP_782819.1| hypothetical protein CTC02276 [Clostridium tetani E88]
>gi|28211925|ref|NP_782869.1| putative ATPase related protein [Clostridium tetani E88]
>gi|28211928|ref|NP_782872.1| hypothetical protein CTC02333 [Clostridium tetani E88]
>gi|28211942|ref|NP_782886.1| ribose transport system permease protein rbsC [Clostridium tetani E88]
>gi|28211960|ref|NP_782904.1| membrane-associated protein [Clostridium tetani E88]
>gi|28211969|ref|NP_782913.1| hypothetical protein CTC02377 [Clostridium tetani E88]
>gi|28211975|ref|NP_782919.1| aspartate carbamoyltransferase regulatory chain [Clostridium tetani E88]
>gi|28211977|ref|NP_782921.1| hypothetical protein CTC02385 [Clostridium tetani E88]
>gi|28212091|ref|NP_783035.1| transporter [Clostridium tetani E88]

>gi|28212094|ref|NP_783038.1| membrane associated protein [Clostridium tetani E88]
>gi|28212115|ref|NP_783059.1| putative DNA polymerase beta [Clostridium tetani E88]
>gi|28212133|ref|NP_783077.1| hypothetical protein CTC02554 [Clostridium tetani E88]
>gi|28212168|ref|NP_783112.1| LSU ribosomal protein L14P [Clostridium tetani E88]
>gi|28373144|ref|NP_783742.1| hypothetical protein pE88_14 [Clostridium tetani E88]
>gi|28373161|ref|NP_783760.1| hypothetical protein pE88_33 [Clostridium tetani E88]
>gi|28373169|ref|NP_783812.1| hypothetical protein pE88_41 [Clostridium tetani E88]
>gi|28373171|ref|NP_783814.1| hypothetical protein pE88_43 [Clostridium tetani E88]
>gi|28373172|ref|NP_783815.1| hypothetical protein pE88_44 [Clostridium tetani E88]
>gi|28373177|ref|NP_783820.1| conserved membrane-spanning protein [Clostridium tetani E88]
>gi|59710651|ref|YP_203427.1| RmuC family protein [Vibrio fischeri ES114]
>gi|59710685|ref|YP_203461.1| putative coproporphyrinogen III oxidase [Vibrio fischeri ES114]
>gi|59710716|ref|YP_203492.1| transporter, drug/metabolite exporter family [Vibrio fischeri ES114]
>gi|59710768|ref|YP_203544.1| hypothetical protein VF0161 [Vibrio fischeri ES114]
>gi|59710796|ref|YP_203572.1| hypothetical protein VF0189 [Vibrio fischeri ES114]
>gi|59710805|ref|YP_203581.1| UDP-glucose 6-dehydrogenase [Vibrio fischeri ES114]
>gi|59710830|ref|YP_203606.1| peptidyl-prolyl cis-trans isomerase [Vibrio fischeri ES114]
>gi|59710860|ref|YP_203636.1| LSU ribosomal protein L18P [Vibrio fischeri ES114]
>gi|59710863|ref|YP_203639.1| LSU ribosomal protein L15P [Vibrio fischeri ES114]
>gi|59710920|ref|YP_203696.1| hypothetical protein VF0313 [Vibrio fischeri ES114]
>gi|59710935|ref|YP_203711.1| hypothetical protein VF0328 [Vibrio fischeri ES114]
>gi|59710950|ref|YP_203726.1| hypothetical protein VF0343 [Vibrio fischeri ES114]
>gi|59710960|ref|YP_203736.1| single-strand DNA binding protein [Vibrio fischeri ES114]
>gi|59711071|ref|YP_203847.1| SSU ribosomal protein S20P [Vibrio fischeri ES114]
>gi|59711090|ref|YP_203866.1| protein translocase subunit SecG [Vibrio fischeri ES114]
>gi|59711093|ref|YP_203869.1| bacterial protein translation initiation factor 2 (IF-2) [Vibrio fischeri ES114]
>gi|59711110|ref|YP_203886.1| hypothetical protein VF0503 [Vibrio fischeri ES114]
>gi|59711163|ref|YP_203939.1| hypothetical protein VF0556 [Vibrio fischeri ES114]
>gi|59711184|ref|YP_203960.1| hypothetical protein VF0577 [Vibrio fischeri ES114]
>gi|59711261|ref|YP_204037.1| hypothetical protein VF0654 [Vibrio fischeri ES114]
>gi|59711454|ref|YP_204230.1| acyl carrier protein [Vibrio fischeri ES114]
>gi|59711474|ref|YP_204250.1| hypothetical protein VF0867 [Vibrio fischeri ES114]
>gi|59711537|ref|YP_204313.1| Na(+)-translocating NADH-quinone reductase subunit D [Vibrio fischeri ES114]
>gi|59711540|ref|YP_204316.1| electron transport complex protein RnfC [Vibrio fischeri ES114]
>gi|59711568|ref|YP_204344.1| TolA protein [Vibrio fischeri ES114]
>gi|59711571|ref|YP_204347.1| Tol system periplasmic component YbgF [Vibrio fischeri ES114]
>gi|59711664|ref|YP_204440.1| lysine exporter protein [Vibrio fischeri ES114]
>gi|59711715|ref|YP_204491.1| chaperone protein DnaJ [Vibrio fischeri ES114]
>gi|59711717|ref|YP_204493.1| hypothetical protein VF1110 [Vibrio fischeri ES114]
>gi|59711801|ref|YP_204577.1| hydroxyacylglutathione hydrolase [Vibrio fischeri ES114]
>gi|59711802|ref|YP_204578.1| hypothetical protein VF1195 [Vibrio fischeri ES114]
>gi|59711824|ref|YP_204600.1| LSU ribosomal protein L35P [Vibrio fischeri ES114]
>gi|59711831|ref|YP_204607.1| TolQ protein [Vibrio fischeri ES114]
>gi|59711832|ref|YP_204608.1| TonB protein [Vibrio fischeri ES114]
>gi|59711857|ref|YP_204633.1| hypothetical protein VF1250 [Vibrio fischeri ES114]
>gi|59711886|ref|YP_204662.1| ProP effector [Vibrio fischeri ES114]
>gi|59711933|ref|YP_204709.1| putative acetyltransferase in HXT11-HXT8 intergenic region [Vibrio fischeri ES114]
>gi|59712010|ref|YP_204786.1| molybdenum transport system permease protein ModB [Vibrio fischeri ES114]
>gi|59712060|ref|YP_204836.1| hypothetical protein VF1453 [Vibrio fischeri ES114]
>gi|59712123|ref|YP_204899.1| hypothetical protein VF1516 [Vibrio fischeri ES114]
>gi|59712196|ref|YP_204972.1| hypothetical protein VF1589 [Vibrio fischeri ES114]
>gi|59712238|ref|YP_205014.1| DNA-binding protein H-NS [Vibrio fischeri ES114]
>gi|59712270|ref|YP_205046.1| hypothetical protein VF1663 [Vibrio fischeri ES114]
>gi|59712291|ref|YP_205067.1| integral membrane protein (rhomboid family) [Vibrio fischeri ES114]
>gi|59712304|ref|YP_205080.1| latent nuclear antigen [Vibrio fischeri ES114]
>gi|59712318|ref|YP_205094.1| hypothetical protein VF1711 [Vibrio fischeri ES114]
>gi|59712355|ref|YP_205131.1| ribonuclease E [Vibrio fischeri ES114]
>gi|59712384|ref|YP_205160.1| hypothetical protein VF1777 [Vibrio fischeri ES114]
>gi|59712430|ref|YP_205206.1| heme exporter protein B [Vibrio fischeri ES114]
>gi|59712432|ref|YP_205208.1| hypothetical protein VF1825 [Vibrio fischeri ES114]
>gi|59712435|ref|YP_205211.1| hypothetical protein VF1828 [Vibrio fischeri ES114]
>gi|59712454|ref|YP_205230.1| flagellar hook-length control protein [Vibrio fischeri ES114]
>gi|59712457|ref|YP_205233.1| flagellar assembly protein FliH [Vibrio fischeri ES114]
>gi|59712498|ref|YP_205274.1| cell division protein ZipA [Vibrio fischeri ES114]
>gi|59712508|ref|YP_205284.1| hypothetical protein VF1901 [Vibrio fischeri ES114]
>gi|59712559|ref|YP_205335.1| UDP-3-O-[3-hydroxymyristoyl] glucosamine N-acyltransferase [Vibrio fischeri ES114]
>gi|59712584|ref|YP_205360.1| Protein YgiW precursor [Vibrio fischeri ES114]
>gi|59712600|ref|YP_205376.1| chaperone protein DnaJ [Vibrio fischeri ES114]
>gi|59712675|ref|YP_205451.1| lipoprotein NlpD [Vibrio fischeri ES114]
>gi|59712763|ref|YP_205539.1| gluconate permease [Vibrio fischeri ES114]

>gi|59712786|ref|YP_205562.1| dihydrolipoamide acetyltransferase component of pyruvate dehydrogenase complex [Vibrio fischeri ES114]
>gi|59712803|ref|YP_205579.1| cell division protein FtsZ [Vibrio fischeri ES114]
>gi|59712953|ref|YP_205729.1| serine acetyltransferase [Vibrio fischeri ES114]
>gi|59712975|ref|YP_205751.1| high-affinity zinc uptake system membrane protein ZnuB [Vibrio fischeri ES114]
>gi|59712981|ref|YP_205757.1| two-component response regulator [Vibrio fischeri ES114]
>gi|59712993|ref|YP_205769.1| biotin carboxyl carrier protein of acetyl-CoA carboxylase [Vibrio fischeri ES114]
>gi|59713008|ref|YP_205784.1| protein YhgI [Vibrio fischeri ES114]
>gi|59713016|ref|YP_205792.1| minor curlin subunit CsgB [Vibrio fischeri ES114]
>gi|59713022|ref|YP_205798.1| LSU ribosomal protein L12P (L7/L12) [Vibrio fischeri ES114]
>gi|59713029|ref|YP_205805.1| protein translocase subunit SecE [Vibrio fischeri ES114]
>gi|59713039|ref|YP_205815.1| hypothetical protein VF2432 [Vibrio fischeri ES114]
>gi|59713063|ref|YP_205839.1| multiple antibiotic resistance protein MarC [Vibrio fischeri ES114]
>gi|59713118|ref|YP_205894.1| hypothetical protein VF2511 [Vibrio fischeri ES114]
>gi|59713304|ref|YP_206079.1| hypothetical protein VFA0121 [Vibrio fischeri ES114]
>gi|59713312|ref|YP_206087.1| putative transport protein SgaT [Vibrio fischeri ES114]
>gi|59713349|ref|YP_206124.1| hypothetical protein VFA0166 [Vibrio fischeri ES114]
>gi|59713379|ref|YP_206154.1| TonB protein [Vibrio fischeri ES114]
>gi|59713401|ref|YP_206176.1| fimbrial protein precursor Flp1 [Vibrio fischeri ES114]
>gi|59713413|ref|YP_206188.1| outer membrane protein [Vibrio fischeri ES114]
>gi|59713478|ref|YP_206253.1| nucleoprotein/polynucleotide-associated enzyme [Vibrio fischeri ES114]
>gi|59713511|ref|YP_206286.1| outer membrane protein [Vibrio fischeri ES114]
>gi|59713553|ref|YP_206328.1| PTS system, fructose-like enzyme IIC component [Vibrio fischeri ES114]
>gi|59713629|ref|YP_206404.1| copper-binding protein [Vibrio fischeri ES114]
>gi|59713715|ref|YP_206490.1| hypothetical protein VFA0532 [Vibrio fischeri ES114]
>gi|59713749|ref|YP_206524.1| hypothetical protein VFA0566 [Vibrio fischeri ES114]
>gi|59713752|ref|YP_206527.1| hypothetical protein VFA0569 [Vibrio fischeri ES114]
>gi|59713761|ref|YP_206536.1| OrfU [Vibrio fischeri ES114]
>gi|59713779|ref|YP_206554.1| hypothetical protein VFA0596 [Vibrio fischeri ES114]
>gi|59713851|ref|YP_206626.1| hypothetical protein VFA0668 [Vibrio fischeri ES114]
>gi|59713865|ref|YP_206640.1| transporter [Vibrio fischeri ES114]
>gi|59713959|ref|YP_206734.1| TonB protein [Vibrio fischeri ES114]
>gi|59714033|ref|YP_206808.1| chloramphenicol-sensitive protein RarD [Vibrio fischeri ES114]
>gi|59714264|ref|YP_207039.1| hypothetical protein VFA1081 [Vibrio fischeri ES114]
>gi|59714265|ref|YP_207040.1| transcriptional regulator, AraC family [Vibrio fischeri ES114]
>gi|59714310|ref|YP_207085.1| hypothetical protein VFA1127 [Vibrio fischeri ES114]
>gi|59714320|ref|YP_207095.1| anaerobic glycerol-3-phosphate dehydrogenase subunit C [Vibrio fischeri ES114]
>gi|59714335|ref|YP_207110.1| acriflavin resistance periplasmic protein [Vibrio fischeri ES114]
>gi|59714370|ref|YP_207144.1| hypothetical protein VFB14 [Vibrio fischeri ES114]
>gi|68535069|ref|YP_249774.1| putative surface-anchored protein [Corynebacterium jeikeium K411]
>gi|68535074|ref|YP_249779.1| hypothetical protein jk0012 [Corynebacterium jeikeium K411]
>gi|68535078|ref|YP_249783.1| manganese transport protein of NRAMP family [Corynebacterium jeikeium K411]
>gi|68535081|ref|YP_249786.1| putative membrane protein [Corynebacterium jeikeium K411]
>gi|68535086|ref|YP_249791.1| hypothetical protein jk0024 [Corynebacterium jeikeium K411]
>gi|68535094|ref|YP_249799.1| hypothetical protein jk0032 [Corynebacterium jeikeium K411]
>gi|68535100|ref|YP_249805.1| serine/threonine protein kinase PknA [Corynebacterium jeikeium K411]
>gi|68535105|ref|YP_249810.1| hypothetical protein jk0043 [Corynebacterium jeikeium K411]
>gi|68535111|ref|YP_249816.1| hypothetical protein jk0049 [Corynebacterium jeikeium K411]
>gi|68535112|ref|YP_249817.1| putative membrane protein [Corynebacterium jeikeium K411]
>gi|68535113|ref|YP_249818.1| resuscitation-promoting factor RpfC [Corynebacterium jeikeium K411]
>gi|68535115|ref|YP_249820.1| putative secreted protein [Corynebacterium jeikeium K411]
>gi|68535118|ref|YP_249823.1| putative membrane protein [Corynebacterium jeikeium K411]
>gi|68535119|ref|YP_249824.1| hypothetical protein jk0057 [Corynebacterium jeikeium K411]
>gi|68535120|ref|YP_249825.1| hypothetical protein jk0058 [Corynebacterium jeikeium K411]
>gi|68535130|ref|YP_249835.1| hypothetical protein jk0068 [Corynebacterium jeikeium K411]
>gi|68535131|ref|YP_249836.1| hypothetical protein jk0069 [Corynebacterium jeikeium K411]
>gi|68535133|ref|YP_249838.1| hypothetical protein jk0071 [Corynebacterium jeikeium K411]
>gi|68535134|ref|YP_249839.1| putative polyhydroxybutyrate depolymerase [Corynebacterium jeikeium K411]
>gi|68535135|ref|YP_249840.1| putative membrane protein [Corynebacterium jeikeium K411]
>gi|68535150|ref|YP_249855.1| hypothetical protein jk0087 [Corynebacterium jeikeium K411]
>gi|68535166|ref|YP_249871.1| putative transcriptional regulator (TetR family) [Corynebacterium jeikeium K411]
>gi|68535173|ref|YP_249878.1| hypothetical protein jk0108 [Corynebacterium jeikeium K411]
>gi|68535176|ref|YP_249881.1| putative secreted protein [Corynebacterium jeikeium K411]
>gi|68535200|ref|YP_249905.1| putative secreted protein [Corynebacterium jeikeium K411]
>gi|68535205|ref|YP_249910.1| putative secreted protein [Corynebacterium jeikeium K411]
>gi|68535232|ref|YP_249937.1| hypothetical protein jk0167 [Corynebacterium jeikeium K411]
>gi|68535234|ref|YP_249939.1| putative beta-glucosidase-related glycosidase [Corynebacterium jeikeium K411]
>gi|68535241|ref|YP_249946.1| hypothetical protein jk0176 [Corynebacterium jeikeium K411]
>gi|68535242|ref|YP_249947.1| putative Fe-S oxidoreductase [Corynebacterium jeikeium K411]
>gi|68535245|ref|YP_249950.1| molecular chaperone protein [Corynebacterium jeikeium K411]
>gi|68535248|ref|YP_249953.1| molecular chaperone protein [Corynebacterium jeikeium K411]

>gi|68535269|ref|YP_249974.1| sodium/glutamate symporter [Corynebacterium jeikeium K411]
>gi|68535277|ref|YP_249982.1| putative secreted protein [Corynebacterium jeikeium K411]
>gi|68535285|ref|YP_249990.1| putative cell surface protein [Corynebacterium jeikeium K411]
>gi|68535300|ref|YP_250005.1| putative ABC transport system, permease protein [Corynebacterium jeikeium K411]
>gi|68535315|ref|YP_250020.1| phosphate acetyltransferase [Corynebacterium jeikeium K411]
>gi|68535328|ref|YP_250033.1| hypothetical protein jk0263 [Corynebacterium jeikeium K411]
>gi|68535349|ref|YP_250054.1| hypothetical protein jk0284 [Corynebacterium jeikeium K411]
>gi|68535367|ref|YP_250072.1| putative A/G-specific DNA glycosylase [Corynebacterium jeikeium K411]
>gi|68535369|ref|YP_250074.1| hypothetical protein jk0304 [Corynebacterium jeikeium K411]
>gi|68535370|ref|YP_250075.1| DNA repair protein RadA [Corynebacterium jeikeium K411]
>gi|68535380|ref|YP_250085.1| putative membrane protein [Corynebacterium jeikeium K411]
>gi|68535384|ref|YP_250089.1| putative membrane protein [Corynebacterium jeikeium K411]
>gi|68535391|ref|YP_250096.1| putative ABC transport system, permease protein [Corynebacterium jeikeium K411]
>gi|68535398|ref|YP_250103.1| hypothetical protein jk0333 [Corynebacterium jeikeium K411]
>gi|68535405|ref|YP_250110.1| putative secreted protein [Corynebacterium jeikeium K411]
>gi|68535427|ref|YP_250132.1| ABC-type transport system TetB [Corynebacterium jeikeium K411]
>gi|68535429|ref|YP_250134.1| putative membrane protein [Corynebacterium jeikeium K411]
>gi|68535436|ref|YP_250141.1| hypothetical protein jk0371 [Corynebacterium jeikeium K411]
>gi|68535472|ref|YP_250177.1| putative membrane protein [Corynebacterium jeikeium K411]
>gi|68535486|ref|YP_250191.1| resuscitation-promoting factor RpfA [Corynebacterium jeikeium K411]
>gi|68535509|ref|YP_250214.1| hypothetical protein jk0439 [Corynebacterium jeikeium K411]
>gi|68535518|ref|YP_250223.1| putative neuraminidase [Corynebacterium jeikeium K411]
>gi|68535529|ref|YP_250234.1| hypothetical protein jk0459 [Corynebacterium jeikeium K411]
>gi|68535536|ref|YP_250241.1| putative secreted protein [Corynebacterium jeikeium K411]
>gi|68535555|ref|YP_250260.1| hypothetical protein jk0485 [Corynebacterium jeikeium K411]
>gi|68535560|ref|YP_250265.1| hypothetical protein jk0490 [Corynebacterium jeikeium K411]
>gi|68535565|ref|YP_250270.1| hypothetical protein jk0495 [Corynebacterium jeikeium K411]
>gi|68535592|ref|YP_250297.1| putative membrane protein [Corynebacterium jeikeium K411]
>gi|68535605|ref|YP_250310.1| hypothetical protein jk0533 [Corynebacterium jeikeium K411]
>gi|68535609|ref|YP_250314.1| putative membrane protein [Corynebacterium jeikeium K411]
>gi|68535612|ref|YP_250317.1| hypothetical protein jk0540 [Corynebacterium jeikeium K411]
>gi|68535628|ref|YP_250333.1| ribonuclease E [Corynebacterium jeikeium K411]
>gi|68535643|ref|YP_250348.1| hypothetical protein jk0571 [Corynebacterium jeikeium K411]
>gi|68535644|ref|YP_250349.1| putative membrane protein [Corynebacterium jeikeium K411]
>gi|68535652|ref|YP_250357.1| putative secreted protein [Corynebacterium jeikeium K411]
>gi|68535656|ref|YP_250361.1| putative secreted protein [Corynebacterium jeikeium K411]
>gi|68535657|ref|YP_250362.1| hypothetical protein jk0585 [Corynebacterium jeikeium K411]
>gi|68535674|ref|YP_250379.1| hypothetical protein jk0602 [Corynebacterium jeikeium K411]
>gi|68535679|ref|YP_250384.1| hypothetical protein jk0607 [Corynebacterium jeikeium K411]
>gi|68535689|ref|YP_250394.1| hypothetical protein jk0617 [Corynebacterium jeikeium K411]
>gi|68535695|ref|YP_250400.1| putative membrane protein [Corynebacterium jeikeium K411]
>gi|68535698|ref|YP_250403.1| manganese transport protein of NRAMP family [Corynebacterium jeikeium K411]
>gi|68535712|ref|YP_250417.1| hypothetical protein jk0640 [Corynebacterium jeikeium K411]
>gi|68535713|ref|YP_250418.1| hypothetical protein jk0641 [Corynebacterium jeikeium K411]
>gi|68535737|ref|YP_250442.1| hypothetical protein jk0662 [Corynebacterium jeikeium K411]
>gi|68535739|ref|YP_250444.1| hypothetical protein jk0664 [Corynebacterium jeikeium K411]
>gi|68535740|ref|YP_250445.1| low molecular weight protein-tyrosine-phosphatase [Corynebacterium jeikeium K411]
>gi|68535743|ref|YP_250448.1| hypothetical protein jk0668 [Corynebacterium jeikeium K411]
>gi|68535748|ref|YP_250453.1| hypothetical protein jk0673 [Corynebacterium jeikeium K411]
>gi|68535755|ref|YP_250460.1| hypothetical protein jk0680 [Corynebacterium jeikeium K411]
>gi|68535762|ref|YP_250467.1| putative membrane protein [Corynebacterium jeikeium K411]
>gi|68535764|ref|YP_250469.1| putative membrane protein [Corynebacterium jeikeium K411]
>gi|68535782|ref|YP_250487.1| dihydrolipoamide succinyltransferase [Corynebacterium jeikeium K411]
>gi|68535786|ref|YP_250491.1| nicotinate-nucleotide--dimethylbenzimidazole phosphoribosyltransferase [Corynebacterium jeikeium K411]
>gi|68535800|ref|YP_250505.1| putative secreted protein [Corynebacterium jeikeium K411]
>gi|68535814|ref|YP_250519.1| hypothetical protein jk0739 [Corynebacterium jeikeium K411]
>gi|68535818|ref|YP_250523.1| hypothetical protein jk0743 [Corynebacterium jeikeium K411]
>gi|68535830|ref|YP_250535.1| hypothetical protein jk0755 [Corynebacterium jeikeium K411]
>gi|68535831|ref|YP_250536.1| hypothetical protein jk0756 [Corynebacterium jeikeium K411]
>gi|68535832|ref|YP_250537.1| hypothetical protein jk0757 [Corynebacterium jeikeium K411]
>gi|68535833|ref|YP_250538.1| hypothetical protein jk0758 [Corynebacterium jeikeium K411]
>gi|68535838|ref|YP_250543.1| putative secreted protein [Corynebacterium jeikeium K411]
>gi|68535860|ref|YP_250565.1| hypothetical protein jk0785 [Corynebacterium jeikeium K411]
>gi|68535864|ref|YP_250569.1| putative permease of the major facilitator superfamily [Corynebacterium jeikeium K411]
>gi|68535886|ref|YP_250591.1| putative membrane protein [Corynebacterium jeikeium K411]
>gi|68535899|ref|YP_250604.1| hypothetical protein jk0824 [Corynebacterium jeikeium K411]
>gi|68535905|ref|YP_250610.1| hypothetical protein jk0830 [Corynebacterium jeikeium K411]
>gi|68535908|ref|YP_250613.1| putative membrane protein [Corynebacterium jeikeium K411]
>gi|68535938|ref|YP_250643.1| hypothetical protein jk0861 [Corynebacterium jeikeium K411]
>gi|68535945|ref|YP_250650.1| hypothetical protein jk0868 [Corynebacterium jeikeium K411]

>gi|68536015|ref|YP_250720.1| putative membrane protein [Corynebacterium jeikeium K411]
>gi|68536040|ref|YP_250745.1| putative secreted protein [Corynebacterium jeikeium K411]
>gi|68536044|ref|YP_250749.1| putative cell wall-associated hydrolase [Corynebacterium jeikeium K411]
>gi|68536050|ref|YP_250755.1| hypothetical protein jk0973 [Corynebacterium jeikeium K411]
>gi|68536063|ref|YP_250768.1| putative membrane protein [Corynebacterium jeikeium K411]
>gi|68536065|ref|YP_250770.1| putative ABC transport system, permease protein [Corynebacterium jeikeium K411]
>gi|68536074|ref|YP_250779.1| protein-export membrane protein SecG [Corynebacterium jeikeium K411]
>gi|68536084|ref|YP_250789.1| riboflavin synthase beta chain [Corynebacterium jeikeium K411]
>gi|68536097|ref|YP_250802.1| putative integration host factor [Corynebacterium jeikeium K411]
>gi|68536146|ref|YP_250851.1| putative secreted protein [Corynebacterium jeikeium K411]
>gi|68536159|ref|YP_250864.1| deoxyuridine 5'-triphosphate nucleotidohydrolase [Corynebacterium jeikeium K411]
>gi|68536164|ref|YP_250869.1| RNA polymerase sigma factor A [Corynebacterium jeikeium K411]
>gi|68536165|ref|YP_250870.1| putative membrane protein [Corynebacterium jeikeium K411]
>gi|68536168|ref|YP_250873.1| hypothetical protein jk1091 [Corynebacterium jeikeium K411]
>gi|68536195|ref|YP_250900.1| putative BioY family protein [Corynebacterium jeikeium K411]
>gi|68536197|ref|YP_250902.1| putative ABC transport system, permease protein [Corynebacterium jeikeium K411]
>gi|68536198|ref|YP_250903.1| hypothetical protein jk1121 [Corynebacterium jeikeium K411]
>gi|68536199|ref|YP_250904.1| putative transcriptional regulator ClgR [Corynebacterium jeikeium K411]
>gi|68536217|ref|YP_250922.1| DNA-damage-inducible protein F [Corynebacterium jeikeium K411]
>gi|68536219|ref|YP_250924.1| translation initiation factor IF-2 [Corynebacterium jeikeium K411]
>gi|68536247|ref|YP_250952.1| hypothetical protein jk1170 [Corynebacterium jeikeium K411]
>gi|68536251|ref|YP_250956.1| elongation factor EF-Ts [Corynebacterium jeikeium K411]
>gi|68536270|ref|YP_250975.1| 30S ribosomal protein S16 [Corynebacterium jeikeium K411]
>gi|68536272|ref|YP_250977.1| signal recognition particle protein [Corynebacterium jeikeium K411]
>gi|68536275|ref|YP_250980.1| signal recognition particle receptor [Corynebacterium jeikeium K411]
>gi|68536279|ref|YP_250984.1| chromosome segregation protein [Corynebacterium jeikeium K411]
>gi|68536285|ref|YP_250990.1| hypothetical protein jk1208 [Corynebacterium jeikeium K411]
>gi|68536365|ref|YP_251070.1| putative polyhydroxybutyrate depolymerase [Corynebacterium jeikeium K411]
>gi|68536377|ref|YP_251082.1| putative iron ABC transport system, permease protein [Corynebacterium jeikeium K411]
>gi|68536378|ref|YP_251083.1| putative iron ABC transport system, permease protein [Corynebacterium jeikeium K411]
>gi|68536380|ref|YP_251085.1| cobalt-zinc-cadmium resistance protein [Corynebacterium jeikeium K411]
>gi|68536385|ref|YP_251090.1| hypothetical protein jk1301 [Corynebacterium jeikeium K411]
>gi|68536389|ref|YP_251094.1| putative membrane protein [Corynebacterium jeikeium K411]
>gi|68536391|ref|YP_251096.1| hypothetical protein jk1307 [Corynebacterium jeikeium K411]
>gi|68536407|ref|YP_251112.1| hypothetical protein jk1322 [Corynebacterium jeikeium K411]
>gi|68536416|ref|YP_251121.1| hypothetical protein jk1331 [Corynebacterium jeikeium K411]
>gi|68536425|ref|YP_251130.1| ATP synthase C chain [Corynebacterium jeikeium K411]
>gi|68536427|ref|YP_251132.1| putative membrane protein [Corynebacterium jeikeium K411]
>gi|68536432|ref|YP_251137.1| hypothetical protein jk1347 [Corynebacterium jeikeium K411]
>gi|68536443|ref|YP_251148.1| putative secreted protein [Corynebacterium jeikeium K411]
>gi|68536490|ref|YP_251195.1| chloramphenicol exporter [Corynebacterium jeikeium K411]
>gi|68536492|ref|YP_251197.1| hypothetical protein jk1406 [Corynebacterium jeikeium K411]
>gi|68536493|ref|YP_251198.1| hypothetical protein jk1407 [Corynebacterium jeikeium K411]
>gi|68536498|ref|YP_251203.1| putative membrane protein [Corynebacterium jeikeium K411]
>gi|68536510|ref|YP_251215.1| putative secreted protein [Corynebacterium jeikeium K411]
>gi|68536515|ref|YP_251220.1| copper-exporting ATPase [Corynebacterium jeikeium K411]
>gi|68536519|ref|YP_251224.1| putative secreted protein [Corynebacterium jeikeium K411]
>gi|68536534|ref|YP_251239.1| putative membrane protein [Corynebacterium jeikeium K411]
>gi|68536545|ref|YP_251250.1| putative secreted protein [Corynebacterium jeikeium K411]
>gi|68536549|ref|YP_251254.1| enoyl-CoA hydratase [Corynebacterium jeikeium K411]
>gi|68536550|ref|YP_251255.1| pyrazinamidase / nicotinamidase [Corynebacterium jeikeium K411]
>gi|68536558|ref|YP_251263.1| putative membrane protein [Corynebacterium jeikeium K411]
>gi|68536564|ref|YP_251269.1| hypothetical protein jk1478 [Corynebacterium jeikeium K411]
>gi|68536575|ref|YP_251280.1| hypothetical protein jk1489 [Corynebacterium jeikeium K411]
>gi|68536577|ref|YP_251282.1| putative membrane protein [Corynebacterium jeikeium K411]
>gi|68536583|ref|YP_251288.1| putative membrane protein [Corynebacterium jeikeium K411]
>gi|68536596|ref|YP_251301.1| 4-diphosphocytidyl-2-C-methyl-D-erythritol kinase [Corynebacterium jeikeium K411]
>gi|68536608|ref|YP_251313.1| putative membrane protein [Corynebacterium jeikeium K411]
>gi|68536623|ref|YP_251328.1| 30S ribosomal protein S14 [Corynebacterium jeikeium K411]
>gi|68536627|ref|YP_251332.1| hypothetical protein jk1541 [Corynebacterium jeikeium K411]
>gi|68536641|ref|YP_251346.1| hypothetical protein jk1555 [Corynebacterium jeikeium K411]
>gi|68536644|ref|YP_251349.1| putative membrane protein [Corynebacterium jeikeium K411]
>gi|68536645|ref|YP_251350.1| putative secreted metallopeptidase [Corynebacterium jeikeium K411]
>gi|68536652|ref|YP_251357.1| putative secreted protein [Corynebacterium jeikeium K411]
>gi|68536678|ref|YP_251383.1| putative molybdate ABC transport system, permease protein [Corynebacterium jeikeium K411]
>gi|68536760|ref|YP_251465.1| hypothetical protein jk1670 [Corynebacterium jeikeium K411]
>gi|68536771|ref|YP_251476.1| putative membrane protein [Corynebacterium jeikeium K411]
>gi|68536786|ref|YP_251491.1| putative membrane protein [Corynebacterium jeikeium K411]
>gi|68536789|ref|YP_251494.1| putative surface-anchored protein [Corynebacterium jeikeium K411]
>gi|68536797|ref|YP_251502.1| hypothetical protein jk1707 [Corynebacterium jeikeium K411]

>gi|68536823|ref|YP_251528.1| putative secreted protein [Corynebacterium jeikeium K411]
>gi|68536828|ref|YP_251533.1| hypothetical protein jk1738 [Corynebacterium jeikeium K411]
>gi|68536831|ref|YP_251536.1| putative membrane protein [Corynebacterium jeikeium K411]
>gi|68536836|ref|YP_251541.1| hypothetical protein jk1746 [Corynebacterium jeikeium K411]
>gi|68536841|ref|YP_251546.1| putative membrane protein [Corynebacterium jeikeium K411]
>gi|68536844|ref|YP_251549.1| hypothetical protein jk1754 [Corynebacterium jeikeium K411]
>gi|68536846|ref|YP_251551.1| putative Co/Zn/Cd efflux system component [Corynebacterium jeikeium K411]
>gi|68536847|ref|YP_251552.1| 50S ribosomal protein L17 [Corynebacterium jeikeium K411]
>gi|68536862|ref|YP_251567.1| putative iron ABC transport system, permease protein [Corynebacterium jeikeium K411]
>gi|68536863|ref|YP_251568.1| putative iron ABC transport system, permease protein [Corynebacterium jeikeium K411]
>gi|68536884|ref|YP_251589.1| 30S ribosomal protein S5 [Corynebacterium jeikeium K411]
>gi|68536888|ref|YP_251593.1| putative secreted protein [Corynebacterium jeikeium K411]
>gi|68536892|ref|YP_251597.1| putative membrane protein [Corynebacterium jeikeium K411]
>gi|68536897|ref|YP_251602.1| putative iron ABC transport system, permease protein [Corynebacterium jeikeium K411]
>gi|68536898|ref|YP_251603.1| putative iron ABC transport system, permease protein [Corynebacterium jeikeium K411]
>gi|68536906|ref|YP_251611.1| putative iron ABC transport system, permease protein [Corynebacterium jeikeium K411]
>gi|68536907|ref|YP_251612.1| putative iron ABC transport system, permease protein [Corynebacterium jeikeium K411]
>gi|68536940|ref|YP_251645.1| 50S ribosomal protein L7/L12 [Corynebacterium jeikeium K411]
>gi|68536943|ref|YP_251648.1| hypothetical protein jk1851 [Corynebacterium jeikeium K411]
>gi|68536946|ref|YP_251651.1| putative membrane protein [Corynebacterium jeikeium K411]
>gi|68536948|ref|YP_251653.1| putative surface-anchored protein [Corynebacterium jeikeium K411]
>gi|68536954|ref|YP_251659.1| transcription antitermination protein [Corynebacterium jeikeium K411]
>gi|68536968|ref|YP_251673.1| putative membrane protein [Corynebacterium jeikeium K411]
>gi|68536973|ref|YP_251678.1| hypothetical protein jk1879 [Corynebacterium jeikeium K411]
>gi|68536980|ref|YP_251685.1| putative iron ABC transport system, permease protein [Corynebacterium jeikeium K411]
>gi|68536991|ref|YP_251696.1| putative membrane protein [Corynebacterium jeikeium K411]
>gi|68536992|ref|YP_251697.1| putative membrane protein [Corynebacterium jeikeium K411]
>gi|68537001|ref|YP_251706.1| putative membrane protein [Corynebacterium jeikeium K411]
>gi|68537011|ref|YP_251716.1| hypothetical protein jk1917 [Corynebacterium jeikeium K411]
>gi|68537016|ref|YP_251721.1| hypothetical protein jk1922 [Corynebacterium jeikeium K411]
>gi|68537052|ref|YP_251757.1| putative secreted protein [Corynebacterium jeikeium K411]
>gi|68537056|ref|YP_251761.1| putative membrane protein [Corynebacterium jeikeium K411]
>gi|68537077|ref|YP_251782.1| multidrug and toxic compound extrusion family protein [Corynebacterium jeikeium K411]
>gi|68537098|ref|YP_251803.1| DNA polymerase III, epsilon subunit [Corynebacterium jeikeium K411]
>gi|68537103|ref|YP_251808.1| hypothetical protein jk2008 [Corynebacterium jeikeium K411]
>gi|68537107|ref|YP_251812.1| DNA polymerase III, gamma and tau subunits [Corynebacterium jeikeium K411]
>gi|68537113|ref|YP_251818.1| glutamyl-tRNA synthetase [Corynebacterium jeikeium K411]
>gi|68537117|ref|YP_251822.1| hypothetical protein jk2022 [Corynebacterium jeikeium K411]
>gi|68537120|ref|YP_251825.1| hypothetical protein jk2025 [Corynebacterium jeikeium K411]
>gi|68537121|ref|YP_251826.1| hypothetical protein jk2026 [Corynebacterium jeikeium K411]
>gi|68537122|ref|YP_251827.1| putative protease [Corynebacterium jeikeium K411]
>gi|68537123|ref|YP_251828.1| putative membrane protein [Corynebacterium jeikeium K411]
>gi|68537125|ref|YP_251830.1| phenylalanine aminotransferase [Corynebacterium jeikeium K411]
>gi|68537127|ref|YP_251832.1| putative cell surface protein [Corynebacterium jeikeium K411]
>gi|68537130|ref|YP_251835.1| putative aminotransferase [Corynebacterium jeikeium K411]
>gi|68537140|ref|YP_251845.1| putative membrane protein [Corynebacterium jeikeium K411]
>gi|68537141|ref|YP_251846.1| hypothetical protein jk2046 [Corynebacterium jeikeium K411]
>gi|68537142|ref|YP_251847.1| hypothetical protein jk2047 [Corynebacterium jeikeium K411]
>gi|68537147|ref|YP_251852.1| hypothetical protein jk2052 [Corynebacterium jeikeium K411]
>gi|68537148|ref|YP_251853.1| putative membrane protein [Corynebacterium jeikeium K411]
>gi|68537154|ref|YP_251859.1| putative membrane protein [Corynebacterium jeikeium K411]
>gi|68537156|ref|YP_251861.1| putative transcriptional regulator (MarR family) [Corynebacterium jeikeium K411]
>gi|68537160|ref|YP_251865.1| hypothetical protein jk2065 [Corynebacterium jeikeium K411]
>gi|68537164|ref|YP_251869.1| putative penicillin-binding protein 1 [Corynebacterium jeikeium K411]
>gi|68537171|ref|YP_251876.1| putative membrane protein [Corynebacterium jeikeium K411]
>gi|68537172|ref|YP_251877.1| ribokinase [Corynebacterium jeikeium K411]
>gi|68537184|ref|YP_251889.1| putative protein with NUDIX domain [Corynebacterium jeikeium K411]
>gi|68537186|ref|YP_251891.1| putative membrane protein [Corynebacterium jeikeium K411]
>gi|68537199|ref|YP_251904.1| 50S ribosomal protein L34 [Corynebacterium jeikeium K411]
>gi|16752002|ref|NP_445368.1| hypothetical protein CP0829 [Chlamydophila pneumoniae AR39]
>gi|16752012|ref|NP_445378.1| sulfate transporter family protein [Chlamydophila pneumoniae AR39]
>gi|16752019|ref|NP_445385.1| hypothetical protein CP0847 [Chlamydophila pneumoniae AR39]
>gi|16752020|ref|NP_445386.1| hypothetical protein CP0848 [Chlamydophila pneumoniae AR39]
>gi|16752021|ref|NP_445387.1| hypothetical protein CP0849 [Chlamydophila pneumoniae AR39]
>gi|16752150|ref|NP_445517.1| histone H1-like protein HC1 [Chlamydophila pneumoniae AR39]
>gi|16752156|ref|NP_445523.1| hypothetical protein CP0986 [Chlamydophila pneumoniae AR39]
>gi|16752231|ref|NP_445599.1| hypothetical protein CP1062 [Chlamydophila pneumoniae AR39]
>gi|16752232|ref|NP_445600.1| hypothetical protein CP1063 [Chlamydophila pneumoniae AR39]
>gi|16752291|ref|NP_445660.1| hypothetical protein CP1123 [Chlamydophila pneumoniae AR39]
>gi|16752312|ref|NP_444570.1| hypothetical protein CP0018 [Chlamydophila pneumoniae AR39]

>gi|16752320|ref|NP_444578.1| hypothetical protein CP0026 [Chlamydophila pneumoniae AR39]
>gi|16752334|ref|NP_444592.1| hypothetical protein CP0040 [Chlamydophila pneumoniae AR39]
>gi|16752363|ref|NP_444621.1| hypothetical protein CP0069 [Chlamydophila pneumoniae AR39]
>gi|16752410|ref|NP_444669.1| ribosomal protein L15 [Chlamydophila pneumoniae AR39]
>gi|16752439|ref|NP_444698.1| hypothetical protein CP0146 [Chlamydophila pneumoniae AR39]
>gi|16752466|ref|NP_444728.1| hypothetical protein CP0177 [Chlamydophila pneumoniae AR39]
>gi|16752519|ref|NP_444781.1| hypothetical protein CP0230 [Chlamydophila pneumoniae AR39]
>gi|16752530|ref|NP_444792.1| hypothetical protein CP0241 [Chlamydophila pneumoniae AR39]
>gi|16752558|ref|NP_444820.1| hypothetical protein CP0269 [Chlamydophila pneumoniae AR39]
>gi|16752570|ref|NP_444832.1| hypothetical protein CP0282 [Chlamydophila pneumoniae AR39]
>gi|16752598|ref|NP_444860.1| hypothetical protein CP0311 [Chlamydophila pneumoniae AR39]
>gi|16752635|ref|NP_444899.1| hypothetical protein CP0350 [Chlamydophila pneumoniae AR39]
>gi|16752645|ref|NP_444910.1| hypothetical protein CP0362 [Chlamydophila pneumoniae AR39]
>gi|16752654|ref|NP_444919.1| Hc2 nucleoprotein [Chlamydophila pneumoniae AR39]
>gi|16752657|ref|NP_444922.1| hypothetical protein CP0374 [Chlamydophila pneumoniae AR39]
>gi|16752669|ref|NP_444934.1| hypothetical protein CP0386 [Chlamydophila pneumoniae AR39]
>gi|16752688|ref|NP_444955.1| hypothetical protein CP0406 [Chlamydophila pneumoniae AR39]
>gi|16752696|ref|NP_444963.1| ABC transporter, permease protein, putative [Chlamydophila pneumoniae AR39]
>gi|16752731|ref|NP_444998.1| hypothetical protein CP0450 [Chlamydophila pneumoniae AR39]
>gi|16752737|ref|NP_445004.1| UDP-3-O-(R-3-hydroxymyristoyl)-glucosamine N-acyltransferase [Chlamydophila pneumoniae AR39]
>gi|16752747|ref|NP_445015.1| inclusion membrane protein B [Chlamydophila pneumoniae AR39]
>gi|16752760|ref|NP_445028.1| hypothetical protein CP0481 [Chlamydophila pneumoniae AR39]
>gi|16752771|ref|NP_445039.1| hypothetical protein CP0492 [Chlamydophila pneumoniae AR39]
>gi|16752774|ref|NP_445042.1| hypothetical protein CP0495 [Chlamydophila pneumoniae AR39]
>gi|16752798|ref|NP_445066.1| hypothetical protein CP0522 [Chlamydophila pneumoniae AR39]
>gi|16752814|ref|NP_445083.1| hypothetical protein CP0539 [Chlamydophila pneumoniae AR39]
>gi|16752819|ref|NP_445088.1| hypothetical protein CP0544 [Chlamydophila pneumoniae AR39]
>gi|16752824|ref|NP_445093.1| hypothetical protein CP0549 [Chlamydophila pneumoniae AR39]
>gi|16752829|ref|NP_445098.1| hypothetical protein CP0554 [Chlamydophila pneumoniae AR39]
>gi|16752868|ref|NP_445138.1| hypothetical protein CP0595 [Chlamydophila pneumoniae AR39]
>gi|16752870|ref|NP_445140.1| hypothetical protein CP0597 [Chlamydophila pneumoniae AR39]
>gi|16752877|ref|NP_445147.1| hypothetical protein CP0605 [Chlamydophila pneumoniae AR39]
>gi|16752897|ref|NP_445168.1| hypothetical protein CP0626 [Chlamydophila pneumoniae AR39]
>gi|16752979|ref|NP_445252.1| hypothetical protein CP0710 [Chlamydophila pneumoniae AR39]
>gi|16752994|ref|NP_445267.1| hypothetical protein CP0725 [Chlamydophila pneumoniae AR39]
>gi|16753014|ref|NP_445287.1| ribosomal protein S21 [Chlamydophila pneumoniae AR39]
>gi|16753017|ref|NP_445290.1| hypothetical protein CP0748 [Chlamydophila pneumoniae AR39]
>gi|16753019|ref|NP_445292.1| hypothetical protein CP0750 [Chlamydophila pneumoniae AR39]
>gi|16753029|ref|NP_445302.1| hypothetical protein CP0763 [Chlamydophila pneumoniae AR39]
>gi|16753063|ref|NP_445463.1| ribosomal protein L34 [Chlamydophila pneumoniae AR39]
>gi|16753078|ref|NP_444939.1| hypothetical protein CP0390 [Chlamydophila pneumoniae AR39]
>gi|22297553|ref|NP_680800.1| hypothetical protein tll0009 [Thermosynechococcus elongatus BP-1]
>gi|22297568|ref|NP_680815.1| hypothetical protein tll0024 [Thermosynechococcus elongatus BP-1]
>gi|22297570|ref|NP_680817.1| hypothetical protein tll0026 [Thermosynechococcus elongatus BP-1]
>gi|22297572|ref|NP_680819.1| hypothetical protein tlr0028 [Thermosynechococcus elongatus BP-1]
>gi|22297576|ref|NP_680823.1| UDP-3-O-(R-3-hydroxymyristoyl)-glucosamine N-acyltransferase [Thermosynechococcus elongatus BP-1]
>gi|22297609|ref|NP_680856.1| thiamine biosynthesis protein [Thermosynechococcus elongatus BP-1]
>gi|22297619|ref|NP_680866.1| hypothetical protein tll0075 [Thermosynechococcus elongatus BP-1]
>gi|22297641|ref|NP_680888.1| 30S ribosomal protein S5 [Thermosynechococcus elongatus BP-1]
>gi|22297647|ref|NP_680894.1| 30S ribosomal protein S13 [Thermosynechococcus elongatus BP-1]
>gi|22297650|ref|NP_680897.1| 50S ribosomal protein L17 [Thermosynechococcus elongatus BP-1]
>gi|22297670|ref|NP_680917.1| hypothetical protein tll0126 [Thermosynechococcus elongatus BP-1]
>gi|22297672|ref|NP_680919.1| hypothetical protein tll0128 [Thermosynechococcus elongatus BP-1]
>gi|22297676|ref|NP_680923.1| hypothetical protein tlr0132 [Thermosynechococcus elongatus BP-1]
>gi|22297702|ref|NP_680949.1| sodium/hydrogen antiporter [Thermosynechococcus elongatus BP-1]
>gi|22297715|ref|NP_680962.1| hypothetical protein tlr0171 [Thermosynechococcus elongatus BP-1]
>gi|22297720|ref|NP_680967.1| photosystem II protein [Thermosynechococcus elongatus BP-1]
>gi|22297733|ref|NP_680980.1| hypothetical protein tlr0189 [Thermosynechococcus elongatus BP-1]
>gi|22297740|ref|NP_680987.1| hypothetical protein tlr0196 [Thermosynechococcus elongatus BP-1]
>gi|22297747|ref|NP_680994.1| protein-export membrane protein [Thermosynechococcus elongatus BP-1]
>gi|22297754|ref|NP_681001.1| hypothetical protein tll0210 [Thermosynechococcus elongatus BP-1]
>gi|22297796|ref|NP_681043.1| hypothetical protein tlr0252 [Thermosynechococcus elongatus BP-1]
>gi|22297799|ref|NP_681046.1| amino acid permease family protein [Thermosynechococcus elongatus BP-1]
>gi|22297807|ref|NP_681054.1| hypothetical protein tlr0263 [Thermosynechococcus elongatus BP-1]
>gi|22297827|ref|NP_681074.1| lipid transfer protein M30 [Thermosynechococcus elongatus BP-1]
>gi|22297842|ref|NP_681089.1| 50S ribosomal protein L12 [Thermosynechococcus elongatus BP-1]
>gi|22297849|ref|NP_681096.1| hypothetical protein tsr0305 [Thermosynechococcus elongatus BP-1]
>gi|22297872|ref|NP_681119.1| hypothetical protein tll0329 [Thermosynechococcus elongatus BP-1]
>gi|22297884|ref|NP_681131.1| hypothetical protein tlr0341 [Thermosynechococcus elongatus BP-1]
>gi|22297885|ref|NP_681132.1| hypothetical protein tlr0342 [Thermosynechococcus elongatus BP-1]

>gi|22297906|ref|NP_681153.1| hypothetical protein tlr0363 [Thermosynechococcus elongatus BP-1]
>gi|22297915|ref|NP_681162.1| hypothetical protein tlr0372 [Thermosynechococcus elongatus BP-1]
>gi|22297929|ref|NP_681176.1| hypothetical protein tll0386 [Thermosynechococcus elongatus BP-1]
>gi|22297935|ref|NP_681182.1| hypothetical protein tll0392 [Thermosynechococcus elongatus BP-1]
>gi|22297950|ref|NP_681197.1| hypothetical protein tlr0407 [Thermosynechococcus elongatus BP-1]
>gi|22297952|ref|NP_681199.1| hypothetical protein tlr0409 [Thermosynechococcus elongatus BP-1]
>gi|22297956|ref|NP_681203.1| hypothetical protein tlr0413 [Thermosynechococcus elongatus BP-1]
>gi|22297963|ref|NP_681210.1| hypothetical protein tlr0420 [Thermosynechococcus elongatus BP-1]
>gi|22297972|ref|NP_681219.1| H+-transporting ATP synthase chain [Thermosynechococcus elongatus BP-1]
>gi|22297973|ref|NP_681220.1| H+-transporting ATP synthase chain a [Thermosynechococcus elongatus BP-1]
>gi|22297975|ref|NP_681222.1| H+-transporting ATP synthase chain b' [Thermosynechococcus elongatus BP-1]
>gi|22297976|ref|NP_681223.1| H+-transporting ATP synthase chain b [Thermosynechococcus elongatus BP-1]
>gi|22298019|ref|NP_681266.1| type 4 prepilin-like proteins leader peptide processing enzyme [Thermosynechococcus elongatus BP-1]
>gi|22298043|ref|NP_681290.1| isopentenyl monophosphate kinase [Thermosynechococcus elongatus BP-1]
>gi|22298044|ref|NP_681291.1| zinc ABC transporter, permease protein [Thermosynechococcus elongatus BP-1]
>gi|22298048|ref|NP_681295.1| hypothetical protein tlr0505 [Thermosynechococcus elongatus BP-1]
>gi|22298058|ref|NP_681305.1| hypothetical protein tll0515 [Thermosynechococcus elongatus BP-1]
>gi|22298060|ref|NP_681307.1| putative RND efflux membrane fusion protein [Thermosynechococcus elongatus BP-1]
>gi|22298063|ref|NP_681310.1| hypothetical protein tll0520 [Thermosynechococcus elongatus BP-1]
>gi|22298069|ref|NP_681316.1| ATP synthase epsilon subunit [Thermosynechococcus elongatus BP-1]
>gi|22298081|ref|NP_681328.1| putative potassium/proton antiporter [Thermosynechococcus elongatus BP-1]
>gi|22298099|ref|NP_681346.1| hypothetical protein tlr0556 [Thermosynechococcus elongatus BP-1]
>gi|22298101|ref|NP_681348.1| hypothetical protein tlr0558 [Thermosynechococcus elongatus BP-1]
>gi|22298103|ref|NP_681350.1| hypothetical protein tlr0560 [Thermosynechococcus elongatus BP-1]
>gi|22298145|ref|NP_681392.1| 50S ribosomal protein L34 [Thermosynechococcus elongatus BP-1]
>gi|22298152|ref|NP_681399.1| hypothetical protein tlr0609 [Thermosynechococcus elongatus BP-1]
>gi|22298153|ref|NP_681400.1| hypothetical protein tlr0610 [Thermosynechococcus elongatus BP-1]
>gi|22298172|ref|NP_681419.1| hypothetical protein tlr0630 [Thermosynechococcus elongatus BP-1]
>gi|22298173|ref|NP_681420.1| hypothetical protein tlr0631 [Thermosynechococcus elongatus BP-1]
>gi|22298177|ref|NP_681424.1| hypothetical protein tll0635 [Thermosynechococcus elongatus BP-1]
>gi|22298215|ref|NP_681462.1| hypothetical protein tlr0673 [Thermosynechococcus elongatus BP-1]
>gi|22298216|ref|NP_681463.1| hypothetical protein tlr0674 [Thermosynechococcus elongatus BP-1]
>gi|22298217|ref|NP_681464.1| hypothetical protein tlr0675 [Thermosynechococcus elongatus BP-1]
>gi|22298218|ref|NP_681465.1| hypothetical protein tlr0676 [Thermosynechococcus elongatus BP-1]
>gi|22298219|ref|NP_681466.1| hypothetical protein tlr0677 [Thermosynechococcus elongatus BP-1]
>gi|22298223|ref|NP_681470.1| hypothetical protein tlr0681 [Thermosynechococcus elongatus BP-1]
>gi|22298251|ref|NP_681498.1| hypothetical protein tll0709 [Thermosynechococcus elongatus BP-1]
>gi|22298256|ref|NP_681503.1| hypothetical protein tlr0714 [Thermosynechococcus elongatus BP-1]
>gi|22298275|ref|NP_681522.1| hypothetical protein tlr0733 [Thermosynechococcus elongatus BP-1]
>gi|22298279|ref|NP_681526.1| bacitracin resistance protein [Thermosynechococcus elongatus BP-1]
>gi|22298314|ref|NP_681561.1| hypothetical protein tll0772 [Thermosynechococcus elongatus BP-1]
>gi|22298318|ref|NP_681565.1| hypothetical protein tlr0776 [Thermosynechococcus elongatus BP-1]
>gi|22298337|ref|NP_681584.1| hypothetical protein tll0795 [Thermosynechococcus elongatus BP-1]
>gi|22298359|ref|NP_681606.1| hypothetical protein tsl0817 [Thermosynechococcus elongatus BP-1]
>gi|22298364|ref|NP_681611.1| hypothetical protein tlr0822 [Thermosynechococcus elongatus BP-1]
>gi|22298369|ref|NP_681616.1| hypothetical protein tsl0827 [Thermosynechococcus elongatus BP-1]
>gi|22298371|ref|NP_681618.1| hypothetical protein tlr0828 [Thermosynechococcus elongatus BP-1]
>gi|22298386|ref|NP_681633.1| ferripyochelin binding protein [Thermosynechococcus elongatus BP-1]
>gi|22298394|ref|NP_681641.1| serine acetyltransferase [Thermosynechococcus elongatus BP-1]
>gi|22298396|ref|NP_681643.1| hypothetical protein tll0853 [Thermosynechococcus elongatus BP-1]
>gi|22298427|ref|NP_681674.1| hypothetical protein tlr0884 [Thermosynechococcus elongatus BP-1]
>gi|22298444|ref|NP_681691.1| hypothetical protein tll0901 [Thermosynechococcus elongatus BP-1]
>gi|22298446|ref|NP_681693.1| hypothetical protein tlr0903 [Thermosynechococcus elongatus BP-1]
>gi|22298455|ref|NP_681702.1| hypothetical protein tll0912 [Thermosynechococcus elongatus BP-1]
>gi|22298486|ref|NP_681733.1| carbon dioxide concentrating mechanism protein [Thermosynechococcus elongatus BP-1]
>gi|22298492|ref|NP_681739.1| hypothetical protein tlr0949 [Thermosynechococcus elongatus BP-1]
>gi|22298510|ref|NP_681757.1| hypothetical protein tlr0967 [Thermosynechococcus elongatus BP-1]
>gi|22298547|ref|NP_681794.1| hypothetical protein tlr1004 [Thermosynechococcus elongatus BP-1]
>gi|22298566|ref|NP_681813.1| methyl-accepting chemotaxis protein [Thermosynechococcus elongatus BP-1]
>gi|22298567|ref|NP_681814.1| hypothetical protein tll1023 [Thermosynechococcus elongatus BP-1]
>gi|22298574|ref|NP_681821.1| hypothetical protein tll1030 [Thermosynechococcus elongatus BP-1]
>gi|22298610|ref|NP_681857.1| translation initiation factor IF-2 [Thermosynechococcus elongatus BP-1]
>gi|22298617|ref|NP_681864.1| hypothetical protein tlr1073 [Thermosynechococcus elongatus BP-1]
>gi|22298653|ref|NP_681900.1| hypothetical protein tll1109 [Thermosynechococcus elongatus BP-1]
>gi|22298654|ref|NP_681901.1| hypothetical protein tll1110 [Thermosynechococcus elongatus BP-1]
>gi|22298665|ref|NP_681912.1| ABC transporter permease protein [Thermosynechococcus elongatus BP-1]
>gi|22298672|ref|NP_681919.1| hypothetical protein tlr1129 [Thermosynechococcus elongatus BP-1]
>gi|22298674|ref|NP_681921.1| hypothetical protein tsr1131 [Thermosynechococcus elongatus BP-1]
>gi|22298675|ref|NP_681922.1| DNA polymerase III gamma and tau subunits [Thermosynechococcus elongatus BP-1]
>gi|22298692|ref|NP_681939.1| hypothetical protein tll1149 [Thermosynechococcus elongatus BP-1]

>gi|22298697|ref|NP_681944.1| hypothetical protein tlr1154 [Thermosynechococcus elongatus BP-1]
>gi|22298703|ref|NP_681950.1| hypothetical protein tsl1160 [Thermosynechococcus elongatus BP-1]
>gi|22298709|ref|NP_681956.1| hypothetical protein tlr1166 [Thermosynechococcus elongatus BP-1]
>gi|22298715|ref|NP_681962.1| hypothetical protein tsl1172 [Thermosynechococcus elongatus BP-1]
>gi|22298747|ref|NP_681994.1| ferrous iron transport protein A [Thermosynechococcus elongatus BP-1]
>gi|22298755|ref|NP_682002.1| hypothetical protein tsr1212 [Thermosynechococcus elongatus BP-1]
>gi|22298774|ref|NP_682021.1| hypothetical protein tll1231 [Thermosynechococcus elongatus BP-1]
>gi|22298822|ref|NP_682069.1| 30S ribosomal protein S6 [Thermosynechococcus elongatus BP-1]
>gi|22298838|ref|NP_682085.1| biotin carboxyl carrier protein of acetyl-CoA carboxylase [Thermosynechococcus elongatus BP-1]
>gi|22298842|ref|NP_682089.1| dihydrolipoamide S-acetyltransferase [Thermosynechococcus elongatus BP-1]
>gi|22298843|ref|NP_682090.1| hypothetical protein tll1300 [Thermosynechococcus elongatus BP-1]
>gi|22298846|ref|NP_682093.1| 50S ribosomal protein L19 [Thermosynechococcus elongatus BP-1]
>gi|22298848|ref|NP_682095.1| hypothetical protein tsr1305 [Thermosynechococcus elongatus BP-1]
>gi|22298862|ref|NP_682109.1| branched-chain amino acid ABC transporter permease protein [Thermosynechococcus elongatus BP-1]
>gi|22298880|ref|NP_682127.1| putative cobalamin [5'-phosphate] synthase [Thermosynechococcus elongatus BP-1]
>gi|22298885|ref|NP_682132.1| putative acetyl transferase [Thermosynechococcus elongatus BP-1]
>gi|22298888|ref|NP_682135.1| hypothetical protein tlr1345 [Thermosynechococcus elongatus BP-1]
>gi|22299017|ref|NP_682264.1| hypothetical protein tlr1474 [Thermosynechococcus elongatus BP-1]
>gi|22299028|ref|NP_682275.1| hypothetical protein tlr1485 [Thermosynechococcus elongatus BP-1]
>gi|22299040|ref|NP_682287.1| hypothetical protein tlr1497 [Thermosynechococcus elongatus BP-1]
>gi|22299044|ref|NP_682291.1| hypothetical protein tlr1501 [Thermosynechococcus elongatus BP-1]
>gi|22299097|ref|NP_682344.1| hypothetical protein tll1554 [Thermosynechococcus elongatus BP-1]
>gi|22299122|ref|NP_682369.1| hypothetical protein tll1579 [Thermosynechococcus elongatus BP-1]
>gi|22299142|ref|NP_682389.1| hypothetical protein tlr1599 [Thermosynechococcus elongatus BP-1]
>gi|22299144|ref|NP_682391.1| cytochrome oxidase d subunit II [Thermosynechococcus elongatus BP-1]
>gi|22299171|ref|NP_682418.1| hypothetical protein tlr1628 [Thermosynechococcus elongatus BP-1]
>gi|22299217|ref|NP_682464.1| hypothetical protein tlr1674 [Thermosynechococcus elongatus BP-1]
>gi|22299222|ref|NP_682469.1| branched-chain amino acid ABC transporter permease protein [Thermosynechococcus elongatus BP-1]
>gi|22299230|ref|NP_682477.1| elongation factor TS [Thermosynechococcus elongatus BP-1]
>gi|22299235|ref|NP_682482.1| lipid transfer protein M30 homolog [Thermosynechococcus elongatus BP-1]
>gi|22299243|ref|NP_682490.1| hypothetical protein tlr1700 [Thermosynechococcus elongatus BP-1]
>gi|22299248|ref|NP_682495.1| hypothetical protein tll1705 [Thermosynechococcus elongatus BP-1]
>gi|22299252|ref|NP_682499.1| hypothetical protein tlr1709 [Thermosynechococcus elongatus BP-1]
>gi|22299278|ref|NP_682525.1| hypothetical protein tlr1735 [Thermosynechococcus elongatus BP-1]
>gi|22299286|ref|NP_682533.1| hypothetical protein tlr1743 [Thermosynechococcus elongatus BP-1]
>gi|22299303|ref|NP_682550.1| putative prohibitin [Thermosynechococcus elongatus BP-1]
>gi|22299324|ref|NP_682571.1| hypothetical protein tll1781 [Thermosynechococcus elongatus BP-1]
>gi|22299326|ref|NP_682573.1| H+/Ca2+ exchanging protein [Thermosynechococcus elongatus BP-1]
>gi|22299327|ref|NP_682574.1| hypothetical protein tll1784 [Thermosynechococcus elongatus BP-1]
>gi|22299335|ref|NP_682582.1| acyl-[acyl-carrier-protein]-UDP-N-acetylglucosam ine o-acyltransferase [Thermosynechococcus elongatus BP-1]
>gi|22299356|ref|NP_682603.1| hypothetical protein tll1813 [Thermosynechococcus elongatus BP-1]
>gi|22299377|ref|NP_682624.1| hypothetical protein tlr1834 [Thermosynechococcus elongatus BP-1]
>gi|22299378|ref|NP_682625.1| hypothetical protein tll1835 [Thermosynechococcus elongatus BP-1]
>gi|22299390|ref|NP_682637.1| hypothetical protein tsl1847 [Thermosynechococcus elongatus BP-1]
>gi|22299392|ref|NP_682639.1| hypothetical protein tlr1849 [Thermosynechococcus elongatus BP-1]
>gi|22299406|ref|NP_682653.1| hypothetical protein tll1863 [Thermosynechococcus elongatus BP-1]
>gi|22299410|ref|NP_682657.1| hypothetical protein tll1867 [Thermosynechococcus elongatus BP-1]
>gi|22299437|ref|NP_682684.1| hypothetical protein tll1894 [Thermosynechococcus elongatus BP-1]
>gi|22299451|ref|NP_682698.1| hypothetical protein tsl1908 [Thermosynechococcus elongatus BP-1]
>gi|22299458|ref|NP_682705.1| 50S ribosomal protein L32 [Thermosynechococcus elongatus BP-1]
>gi|22299466|ref|NP_682713.1| hypothetical protein tll1923 [Thermosynechococcus elongatus BP-1]
>gi|22299468|ref|NP_682715.1| chromosome segregation SMC protein [Thermosynechococcus elongatus BP-1]
>gi|22299486|ref|NP_682733.1| hypothetical protein tll1943 [Thermosynechococcus elongatus BP-1]
>gi|22299489|ref|NP_682736.1| hypothetical protein tll1946 [Thermosynechococcus elongatus BP-1]
>gi|22299550|ref|NP_682797.1| hypothetical protein tlr2007 [Thermosynechococcus elongatus BP-1]
>gi|22299557|ref|NP_682804.1| hypothetical protein tlr2014 [Thermosynechococcus elongatus BP-1]
>gi|22299566|ref|NP_682813.1| hypothetical protein tll2023 [Thermosynechococcus elongatus BP-1]
>gi|22299577|ref|NP_682824.1| phycobilisome core component [Thermosynechococcus elongatus BP-1]
>gi|22299620|ref|NP_682867.1| hypothetical protein tll2077 [Thermosynechococcus elongatus BP-1]
>gi|22299627|ref|NP_682874.1| hypothetical protein tll2084 [Thermosynechococcus elongatus BP-1]
>gi|22299631|ref|NP_682878.1| hypothetical protein tlr2088 [Thermosynechococcus elongatus BP-1]
>gi|22299665|ref|NP_682912.1| hypothetical protein tsr2122 [Thermosynechococcus elongatus BP-1]
>gi|22299668|ref|NP_682915.1| NADH dehydrogenase subunit 4 [Thermosynechococcus elongatus BP-1]
>gi|22299675|ref|NP_682922.1| hypothetical protein tlr2132 [Thermosynechococcus elongatus BP-1]
>gi|22299686|ref|NP_682933.1| hypothetical protein tlr2143 [Thermosynechococcus elongatus BP-1]
>gi|22299718|ref|NP_682965.1| hypothetical protein tlr2175 [Thermosynechococcus elongatus BP-1]
>gi|22299727|ref|NP_682974.1| hypothetical protein tlr2184 [Thermosynechococcus elongatus BP-1]
>gi|22299746|ref|NP_682993.1| two-component response regulator [Thermosynechococcus elongatus BP-1]
>gi|22299756|ref|NP_683003.1| hypothetical protein tsl2213 [Thermosynechococcus elongatus BP-1]
>gi|22299765|ref|NP_683012.1| serine/threonine protein kinase [Thermosynechococcus elongatus BP-1]

>gi|22299778|ref|NP_683025.1| hypothetical protein tlr2235 [Thermosynechococcus elongatus BP-1]
>gi|22299801|ref|NP_683048.1| hypothetical protein tlr2258 [Thermosynechococcus elongatus BP-1]
>gi|22299806|ref|NP_683053.1| hypothetical protein tlr2263 [Thermosynechococcus elongatus BP-1]
>gi|22299814|ref|NP_683061.1| hypothetical protein tlr2271 [Thermosynechococcus elongatus BP-1]
>gi|22299834|ref|NP_683081.1| hypothetical protein tll2291 [Thermosynechococcus elongatus BP-1]
>gi|22299871|ref|NP_683118.1| hypothetical protein tlr2328 [Thermosynechococcus elongatus BP-1]
>gi|22299903|ref|NP_683150.1| hypothetical protein tlr2360 [Thermosynechococcus elongatus BP-1]
>gi|22299904|ref|NP_683151.1| hypothetical protein tll2361 [Thermosynechococcus elongatus BP-1]
>gi|22299913|ref|NP_683160.1| hypothetical protein tlr2370 [Thermosynechococcus elongatus BP-1]
>gi|22299944|ref|NP_683191.1| hypothetical protein tlr2401 [Thermosynechococcus elongatus BP-1]
>gi|22299958|ref|NP_683205.1| hypothetical protein tlr2415 [Thermosynechococcus elongatus BP-1]
>gi|22299975|ref|NP_683222.1| serine/threonine protein kinase [Thermosynechococcus elongatus BP-1]
>gi|15616634|ref|NP_239846.1| ATP synthase B chain [Buchnera aphidicola str. APS (Acyrthosiphon pisum)]
>gi|15616679|ref|NP_239891.1| serine acetyltransferase [Buchnera aphidicola str. APS (Acyrthosiphon pisum)]
>gi|15616699|ref|NP_239911.1| flagellar FliJ protein [Buchnera aphidicola str. APS (Acyrthosiphon pisum)]
>gi|15616701|ref|NP_239913.1| flagellar hook-length control protein [Buchnera aphidicola str. APS (Acyrthosiphon pisum)]
>gi|15616702|ref|NP_239914.1| flagellar motor switch protein FliM [Buchnera aphidicola str. APS (Acyrthosiphon pisum)]
>gi|15616706|ref|NP_239918.1| flagellar biosynthetic protein FliR [Buchnera aphidicola str. APS (Acyrthosiphon pisum)]
>gi|15616743|ref|NP_239955.1| hypothetical protein BU123 [Buchnera aphidicola str. APS (Acyrthosiphon pisum)]
>gi|15616782|ref|NP_239994.1| NADH dehydrogenase I chain J [Buchnera aphidicola str. APS (Acyrthosiphon pisum)]
>gi|15616783|ref|NP_239995.1| NADH dehydrogenase I chain K [Buchnera aphidicola str. APS (Acyrthosiphon pisum)]
>gi|15616800|ref|NP_240012.1| hypothetical protein BU181 [Buchnera aphidicola str. APS (Acyrthosiphon pisum)]
>gi|15616803|ref|NP_240015.1| heat shock protein GrpE2 [Buchnera aphidicola str. APS (Acyrthosiphon pisum)]
>gi|15616856|ref|NP_240069.1| flagellar biosynthetic protein FlhB [Buchnera aphidicola str. APS (Acyrthosiphon pisum)]
>gi|15616863|ref|NP_240076.1| heat shock protein GrpE1 [Buchnera aphidicola str. APS (Acyrthosiphon pisum)]
>gi|15616868|ref|NP_240081.1| GTP-binding protein Era [Buchnera aphidicola str. APS (Acyrthosiphon pisum)]
>gi|15616883|ref|NP_240096.1| DNA-binding protein H-ns [Buchnera aphidicola str. APS (Acyrthosiphon pisum)]
>gi|15616886|ref|NP_240099.1| hypothetical protein BU275 [Buchnera aphidicola str. APS (Acyrthosiphon pisum)]
>gi|15616887|ref|NP_240100.1| hypothetical protein BU276 [Buchnera aphidicola str. APS (Acyrthosiphon pisum)]
>gi|28952049|ref|NP_240107.2| possible protease SohB [Buchnera aphidicola str. APS (Acyrthosiphon pisum)]
>gi|15616906|ref|NP_240119.1| hypothetical protein BU295 [Buchnera aphidicola str. APS (Acyrthosiphon pisum)]
>gi|15616926|ref|NP_240139.1| high-affinity zinc uptake system membrane protein ZnuB [Buchnera aphidicola str. APS (Acyrthosiphon pisum)]
>gi|15616941|ref|NP_240154.1| flagella synthesis protein FlgN [Buchnera aphidicola str. APS (Acyrthosiphon pisum)]
>gi|15616943|ref|NP_240156.1| flagellar basal-body rod protein FlgB [Buchnera aphidicola str. APS (Acyrthosiphon pisum)]
>gi|15617017|ref|NP_240230.1| lipoprotein NlpD precursor [Buchnera aphidicola str. APS (Acyrthosiphon pisum)]
>gi|15617031|ref|NP_240244.1| hypothetical GTP-binding protein [Buchnera aphidicola str. APS (Acyrthosiphon pisum)]
>gi|28952054|ref|NP_240261.2| hypothetical protein BU449 [Buchnera aphidicola str. APS (Acyrthosiphon pisum)]
>gi|15617064|ref|NP_240277.1| hypothetical protein BU466 [Buchnera aphidicola str. APS (Acyrthosiphon pisum)]
>gi|15617068|ref|NP_240281.1| cytochrome O ubiquinol oxidase subunit III [Buchnera aphidicola str. APS (Acyrthosiphon pisum)]
>gi|15617072|ref|NP_240285.1| trigger factor [Buchnera aphidicola str. APS (Acyrthosiphon pisum)]
>gi|15617099|ref|NP_240312.1| 50S ribosomal protein L15 [Buchnera aphidicola str. APS (Acyrthosiphon pisum)]
>gi|15617116|ref|NP_240329.1| 50S ribosomal protein L23 [Buchnera aphidicola str. APS (Acyrthosiphon pisum)]
>gi|15617153|ref|NP_240366.1| 50S ribosomal protein L9 [Buchnera aphidicola str. APS (Acyrthosiphon pisum)]
>gi|15617189|ref|NP_240402.1| chaperone protein HscB [Buchnera aphidicola str. APS (Acyrthosiphon pisum)]
>gi|15617193|ref|NP_240406.1| hypothetical protein BU608 [Buchnera aphidicola str. APS (Acyrthosiphon pisum)]
>gi|56479581|ref|NP_705965.2| hypothetical protein SF0005 [Shigella flexneri 2a str. 301]
>gi|56479584|ref|NP_705974.2| chaperone with DnaK; heat shock protein [Shigella flexneri 2a str. 301]
>gi|24111465|ref|NP_705975.1| Na+/H+ antiporter [Shigella flexneri 2a str. 301]
>gi|56479586|ref|NP_705979.2| 30S ribosomal subunit protein S20 [Shigella flexneri 2a str. 301]
>gi|24111559|ref|NP_706069.1| pyruvate dehydrogenase (dihydrolipoyltransacetylase component) [Shigella flexneri 2a str. 301]
>gi|24111669|ref|NP_706179.1| putative DNA transfer protein [Shigella flexneri 2a str. 301]
>gi|24111775|ref|NP_706285.1| ATP-dependent dsDNA exonuclease [Shigella flexneri 2a str. 301]
>gi|24111826|ref|NP_706336.1| hypothetical protein SF0387 [Shigella flexneri 2a str. 301]
>gi|24111878|ref|NP_706388.1| putative oxidoreductase [Shigella flexneri 2a str. 301]
>gi|24111920|ref|NP_706430.1| hypothetical protein SF0487 [Shigella flexneri 2a str. 301]
>gi|24111936|ref|NP_706446.1| ferric enterobactin (enterochelin) transport protein [Shigella flexneri 2a str. 301]
>gi|24111937|ref|NP_706447.1| hypothetical membrane protein [Shigella flexneri 2a str. 301]
>gi|24111978|ref|NP_706488.1| putative homeobox protein [Shigella flexneri 2a str. 301]
>gi|24111982|ref|NP_706492.1| hypothetical protein SF0555 [Shigella flexneri 2a str. 301]
>gi|24111985|ref|NP_706495.1| membrane spanning protein [Shigella flexneri 2a str. 301]
>gi|56479699|ref|NP_706539.2| hypothetical protein SF0608 [Shigella flexneri 2a str. 301]
>gi|24112073|ref|NP_706583.1| hypothetical protein SF0654 [Shigella flexneri 2a str. 301]
>gi|56479719|ref|NP_706620.2| endopeptidase [Shigella flexneri 2a str. 301]
>gi|24112138|ref|NP_706648.1| putative tail component encoded by cryptic prophage CP-933M [Shigella flexneri 2a str. 301]
>gi|24112140|ref|NP_706650.1| invasion plasmid antigen [Shigella flexneri 2a str. 301]
>gi|24112159|ref|NP_706669.1| hypothetical protein SF0741 [Shigella flexneri 2a str. 301]
>gi|24112163|ref|NP_706673.1| putative membrane protein [Shigella flexneri 2a str. 301]
>gi|56479733|ref|NP_706691.2| putative transmembrane protein subunit [Shigella flexneri 2a str. 301]
>gi|24112212|ref|NP_706722.1| hypothetical protein SF0796 [Shigella flexneri 2a str. 301]
>gi|24112219|ref|NP_706729.1| hypothetical protein SF0803 [Shigella flexneri 2a str. 301]

>gi|56479753|ref|NP_706758.2| transmembrane water channel AqpZ protein [Shigella flexneri 2a str. 301]
>gi|24112265|ref|NP_706775.1| cell division protein [Shigella flexneri 2a str. 301]
>gi|56479789|ref|NP_706910.2| hypothetical protein SF0989 [Shigella flexneri 2a str. 301]
>gi|56479819|ref|NP_707000.2| RNase E [Shigella flexneri 2a str. 301]
>gi|24112510|ref|NP_707020.1| hypothetical protein SF1109 [Shigella flexneri 2a str. 301]
>gi|56479839|ref|NP_707069.2| hypothetical protein SF1159 [Shigella flexneri 2a str. 301]
>gi|56479842|ref|NP_707083.2| Na+/H+ antiporter [Shigella flexneri 2a str. 301]
>gi|24112583|ref|NP_707093.1| hypothetical protein SF1185 [Shigella flexneri 2a str. 301]
>gi|56479846|ref|NP_707096.2| hypothetical protein SF1188 [Shigella flexneri 2a str. 301]
>gi|24112590|ref|NP_707100.1| putative iron compound ABC transporter permease protein [Shigella flexneri 2a str. 301]
>gi|24112633|ref|NP_707143.1| DNA-binding protein HLP-II (HU, BH2, HD, NS); pleiotropic regulator [Shigella flexneri 2a str. 301]
>gi|56479866|ref|NP_707161.2| membrane protein, energy transducer [Shigella flexneri 2a str. 301]
>gi|56479867|ref|NP_707164.2| hypothetical protein SF1258 [Shigella flexneri 2a str. 301]
>gi|24112658|ref|NP_707168.1| hypothetical protein SF1262 [Shigella flexneri 2a str. 301]
>gi|24112682|ref|NP_707192.1| osmotically inducible lipoprotein [Shigella flexneri 2a str. 301]
>gi|24112703|ref|NP_707213.1| phage shock protein, inner membrane protein [Shigella flexneri 2a str. 301]
>gi|24112769|ref|NP_707279.1| invasion plasmid antigen [Shigella flexneri 2a str. 301]
>gi|24112816|ref|NP_707326.1| putative amino acid/amine transport protein [Shigella flexneri 2a str. 301]
>gi|56479905|ref|NP_707357.2| putative cytochrome oxidase [Shigella flexneri 2a str. 301]
>gi|56479920|ref|NP_707397.2| 50S ribosomal subunit protein A [Shigella flexneri 2a str. 301]
>gi|24112893|ref|NP_707403.1| Vitamin B12 transport permease protein [Shigella flexneri 2a str. 301]
>gi|24112954|ref|NP_707464.1| putative transport system permease protein [Shigella flexneri 2a str. 301]
>gi|24112984|ref|NP_707494.1| acid shock protein [Shigella flexneri 2a str. 301]
>gi|24112986|ref|NP_707496.1| possible chaperone [Shigella flexneri 2a str. 301]
>gi|24112988|ref|NP_707498.1| putative transport protein [Shigella flexneri 2a str. 301]
>gi|56479944|ref|NP_707526.2| hypothetical protein SF1651 [Shigella flexneri 2a str. 301]
>gi|24113019|ref|NP_707529.1| putative membrane protein [Shigella flexneri 2a str. 301]
>gi|24113022|ref|NP_707532.1| hypothetical protein SF1657 [Shigella flexneri 2a str. 301]
>gi|24113033|ref|NP_707543.1| putative outer membrane protein [Shigella flexneri 2a str. 301]
>gi|56479949|ref|NP_707555.2| putative lipoprotein [Shigella flexneri 2a str. 301]
>gi|24113066|ref|NP_707576.1| murein lipoprotein [Shigella flexneri 2a str. 301]
>gi|24113129|ref|NP_707639.1| putative transport system permease protein [Shigella flexneri 2a str. 301]
>gi|56479965|ref|NP_707670.2| probable enzyme [Shigella flexneri 2a str. 301]
>gi|24113198|ref|NP_707708.1| hypothetical protein SF1857 [Shigella flexneri 2a str. 301]
>gi|56479979|ref|NP_707730.2| putative tail component encoded by cryptic prophage CP-933M [Shigella flexneri 2a str. 301]
>gi|24113272|ref|NP_707782.1| positive regulator of CheA protein activity [Shigella flexneri 2a str. 301]
>gi|56480006|ref|NP_707825.2| flagellar assembly protein FliH [Shigella flexneri 2a str. 301]
>gi|24113322|ref|NP_707832.1| flagellar protein FliO [Shigella flexneri 2a str. 301]
>gi|56480008|ref|NP_707835.2| flagellar biosynthetic protein FliR [Shigella flexneri 2a str. 301]
>gi|24113332|ref|NP_707842.1| putative transmembrane subunit [Shigella flexneri 2a str. 301]
>gi|24113346|ref|NP_707856.1| hypothetical protein SF2019 [Shigella flexneri 2a str. 301]
>gi|56480019|ref|NP_707894.2| cobalamin 5-phosphate synthase [Shigella flexneri 2a str. 301]
>gi|24113418|ref|NP_707928.1| glycosyl translocase [Shigella flexneri 2a str. 301]
>gi|24113586|ref|NP_708096.1| heme exporter protein B, cytochrome c-type biogenesis protein [Shigella flexneri 2a str. 301]
>gi|56480088|ref|NP_708196.2| putative lipoprotein [Shigella flexneri 2a str. 301]
>gi|24113747|ref|NP_708257.1| hypothetical protein SF2456 [Shigella flexneri 2a str. 301]
>gi|24113753|ref|NP_708263.1| hypothetical protein SF2463 [Shigella flexneri 2a str. 301]
>gi|56480101|ref|NP_708267.2| cell division protein involved in FtsZ ring [Shigella flexneri 2a str. 301]
>gi|56480105|ref|NP_708284.2| hypothetical protein SF2486 [Shigella flexneri 2a str. 301]
>gi|24113814|ref|NP_708324.1| hydrogenase 4 membrane subunit [Shigella flexneri 2a str. 301]
>gi|24113834|ref|NP_708344.1| putative outer membrane lipoprotein [Shigella flexneri 2a str. 301]
>gi|24113835|ref|NP_708345.1| putative membrane protein [Shigella flexneri 2a str. 301]
>gi|24113842|ref|NP_708352.1| hypothetical protein SF2559 [Shigella flexneri 2a str. 301]
>gi|24113845|ref|NP_708355.1| putative membrane protein [Shigella flexneri 2a str. 301]
>gi|24113892|ref|NP_708402.1| putative tail fiber protein [Shigella flexneri 2a str. 301]
>gi|24113912|ref|NP_708422.1| sigma-E factor [Shigella flexneri 2a str. 301]
>gi|24113973|ref|NP_708483.1| DNA-binding protein; H-NS-like protein [Shigella flexneri 2a str. 301]
>gi|24114000|ref|NP_708510.1| PTS system, glucitol/sorbitol-specific IIB component and second of two IIC components [Shigella flexneri 2a str. 301]
>gi|56480172|ref|NP_708524.2| membrane-spanning protein of hydrogenase 3 (part of FHL complex) [Shigella flexneri 2a str. 301]
>gi|56480176|ref|NP_708547.2| lipoprotein [Shigella flexneri 2a str. 301]
>gi|24114058|ref|NP_708568.1| putative transport protein [Shigella flexneri 2a str. 301]
>gi|56480190|ref|NP_708614.2| prepilin peptidase dependent protein C [Shigella flexneri 2a str. 301]
>gi|24114179|ref|NP_708689.1| putative inner membrane protein [Shigella flexneri 2a str. 301]
>gi|24114188|ref|NP_708698.1| putative serine protease [Shigella flexneri 2a str. 301]
>gi|56480250|ref|NP_708868.2| putative kinase [Shigella flexneri 2a str. 301]
>gi|24114369|ref|NP_708879.1| hypothetical protein SF3112 [Shigella flexneri 2a str. 301]
>gi|24114421|ref|NP_708931.1| N-acetylgalactosameine-specific IIC component 2 [Shigella flexneri 2a str. 301]
>gi|56480276|ref|NP_708963.2| inducible ATP-independent RNA helicase [Shigella flexneri 2a str. 301]
>gi|24114459|ref|NP_708969.1| protein chain initiation factor IF-2 [Shigella flexneri 2a str. 301]

>gi|24114464|ref|NP_708974.1| protein translocase membrane component [Shigella flexneri 2a str. 301]
>gi|24114535|ref|NP_709045.1| rod shape-determining protein [Shigella flexneri 2a str. 301]
>gi|24114540|ref|NP_709050.1| acetyl CoA carboxylase, carrier of biotin, BCCP subunit [Shigella flexneri 2a str. 301]
>gi|24114579|ref|NP_709089.1| 50S ribosomal subunit protein L15 [Shigella flexneri 2a str. 301]
>gi|24114582|ref|NP_709092.1| 50S ribosomal subunit protein L18 [Shigella flexneri 2a str. 301]
>gi|24114590|ref|NP_709100.1| 50S ribosomal subunit protein L29 [Shigella flexneri 2a str. 301]
>gi|24114613|ref|NP_709123.1| FKBP-type peptidyl-prolyl cis-trans isomerase (rotamase) [Shigella flexneri 2a str. 301]
>gi|24114651|ref|NP_709161.1| putative membrane protein [Shigella flexneri 2a str. 301]
>gi|24114656|ref|NP_709166.1| hypothetical protein SF3412 [Shigella flexneri 2a str. 301]
>gi|24114708|ref|NP_709218.1| hypothetical protein SF3465 [Shigella flexneri 2a str. 301]
>gi|24114724|ref|NP_709234.1| cell division membrane protein [Shigella flexneri 2a str. 301]
>gi|24114728|ref|NP_709238.1| putative enzyme [Shigella flexneri 2a str. 301]
>gi|24114745|ref|NP_709255.1| putative membrane protein [Shigella flexneri 2a str. 301]
>gi|24114811|ref|NP_709321.1| dipeptide transport system permease protein 2 [Shigella flexneri 2a str. 301]
>gi|56480373|ref|NP_709330.2| putative outer membrane protein [Shigella flexneri 2a str. 301]
>gi|24114870|ref|NP_709380.1| hypothetical protein SF3640 [Shigella flexneri 2a str. 301]
>gi|24114871|ref|NP_709381.1| hypothetical protein SF3641 [Shigella flexneri 2a str. 301]
>gi|56480399|ref|NP_709443.2| hypothetical protein SF3704 [Shigella flexneri 2a str. 301]
>gi|24114971|ref|NP_709481.1| hypothetical protein SF3744 [Shigella flexneri 2a str. 301]
>gi|56480420|ref|NP_709526.2| sensor histidine protein kinase, phosphorylates UhpA [Shigella flexneri 2a str. 301]
>gi|24115039|ref|NP_709549.1| membrane-bound ATP synthase, F0 sector, subunit b [Shigella flexneri 2a str. 301]
>gi|24115040|ref|NP_709550.1| membrane-bound ATP synthase, F0 sector, subunit c [Shigella flexneri 2a str. 301]
>gi|24115042|ref|NP_709552.1| membrane-bound ATP synthase [Shigella flexneri 2a str. 301]
>gi|24115055|ref|NP_709565.1| D-ribose high-affinity transport protein [Shigella flexneri 2a str. 301]
>gi|24115095|ref|NP_709605.1| uroporphyrinogen III methylase [Shigella flexneri 2a str. 301]
>gi|56480443|ref|NP_709623.2| hypothetical protein SF3895 [Shigella flexneri 2a str. 301]
>gi|24115132|ref|NP_709642.1| hypothetical protein SF3914 [Shigella flexneri 2a str. 301]
>gi|24115133|ref|NP_709643.1| hypothetical protein SF3915 [Shigella flexneri 2a str. 301]
>gi|24115154|ref|NP_709664.1| hypothetical protein SF3936 [Shigella flexneri 2a str. 301]
>gi|56480477|ref|NP_709713.2| 2-keto-3-deoxy-D-gluconate transport protein [Shigella flexneri 2a str. 301]
>gi|24115227|ref|NP_709737.1| essential cell division protein [Shigella flexneri 2a str. 301]
>gi|24115271|ref|NP_709781.1| 50S ribosomal subunit protein L7/L12 [Shigella flexneri 2a str. 301]
>gi|56480523|ref|NP_709860.2| ssDNA-binding protein [Shigella flexneri 2a str. 301]
>gi|24115383|ref|NP_709893.1| hypothetical protein SF4179 [Shigella flexneri 2a str. 301]
>gi|24115461|ref|NP_709971.1| similar to D-ribose high-affinity transport protein [Shigella flexneri 2a str. 301]
>gi|56480573|ref|NP_710005.2| hypothetical protein SF4294 [Shigella flexneri 2a str. 301]
>gi|24115529|ref|NP_710039.1| protease specific for phage lambda cII repressor [Shigella flexneri 2a str. 301]
>gi|24115606|ref|NP_710116.1| hypothetical protein SF4408 [Shigella flexneri 2a str. 301]
>gi|31983583|ref|NP_858212.1| invasion plasmid antigen, probably secreted by the Mxi-Spa machinery [Shigella flexneri 2a str. 301]
>gi|31983792|ref|NP_858287.1| Spa9, component of the Mxi-Spa secretion machinery [Shigella flexneri 2a str. 301]
>gi|31983670|ref|NP_858301.1| hypothetical protein CP0168 [Shigella flexneri 2a str. 301]
>gi|31983529|ref|NP_858315.1| IcsA (VirG), outermembrane protein exposed to the bacterial surface by a C-terminal autotransporter domain and involved in the movement of intracellular bacteria by binding to N-WASP [Shigella flexneri 2a str. 301]
>gi|31983615|ref|NP_858320.1| hypothetical protein CP0187 [Shigella flexneri 2a str. 301]
>gi|56404032|ref|NP_858383.2| F pilin acetylation protein [Shigella flexneri 2a str. 301]
>gi|62388903|ref|YP_224305.1| Bacterial regulatory proteins, tetR family [Corynebacterium glutamicum ATCC 13032]
>gi|62388904|ref|YP_224306.1| Helix-turn-helix protein, copG family [Corynebacterium glutamicum ATCC 13032]
>gi|62388911|ref|YP_224313.1| HCCA ISOMERASE, secreted protein [Corynebacterium glutamicum ATCC 13032]
>gi|62388928|ref|YP_224330.1| hypothetical protein cg0047 [Corynebacterium glutamicum ATCC 13032]
>gi|62388932|ref|YP_224334.1| PUTATIVE IRON-SIDEROPHORE UPTAKE SYSTEM TRANSMEMBR [Corynebacterium glutamicum ATCC 13032]
>gi|62388937|ref|YP_224339.1| SERINE/THREONINE PROTEIN KINASE [Corynebacterium glutamicum ATCC 13032]
>gi|62388955|ref|YP_224357.1| putative secreted protein [Corynebacterium glutamicum ATCC 13032]
>gi|62388973|ref|YP_224375.1| secreted protein [Corynebacterium glutamicum ATCC 13032]
>gi|62388983|ref|YP_224385.1| UREASE ACCESSORY PROTEIN [Corynebacterium glutamicum ATCC 13032]
>gi|62388987|ref|YP_224389.1| Permease of the major facilitator superfamily [Corynebacterium glutamicum ATCC 13032]
>gi|62388993|ref|YP_224395.1| secreted protein, Signal peptide [Corynebacterium glutamicum ATCC 13032]
>gi|62389015|ref|YP_224417.1| PUTATIVE ACETYLTRANSFERASE [Corynebacterium glutamicum ATCC 13032]
>gi|62389026|ref|YP_224428.1| permease [Corynebacterium glutamicum ATCC 13032]
>gi|62389033|ref|YP_224435.1| permease [Corynebacterium glutamicum ATCC 13032]
>gi|62389058|ref|YP_224460.1| PUTATIVE OXIDOREDUCTASE MYO-INOSITOL 2-DEHYDROGENA [Corynebacterium glutamicum ATCC 13032]
>gi|62389081|ref|YP_224483.1| membrane protein [Corynebacterium glutamicum ATCC 13032]
>gi|62389107|ref|YP_224509.1| MOLYBDOPTERIN SYNTHASE, LARGE SUBUNIT [Corynebacterium glutamicum ATCC 13032]
>gi|62389125|ref|YP_224527.1| hypothetical protein cg0280 [Corynebacterium glutamicum ATCC 13032]
>gi|62389135|ref|YP_224537.1| 3,4-dioxygenase beta subunit [Corynebacterium glutamicum ATCC 13032]
>gi|62389140|ref|YP_224542.1| DNA POLYMERASE III GAMMA AND TAU SUBUNITS [Corynebacterium glutamicum ATCC 13032]
>gi|62389141|ref|YP_224543.1| Uncharacterized BCR, YbaB family COG0718 [Corynebacterium glutamicum ATCC 13032]
>gi|62389154|ref|YP_224556.1| secreted protein [Corynebacterium glutamicum ATCC 13032]
>gi|62389158|ref|YP_224560.1| secreted protein [Corynebacterium glutamicum ATCC 13032]

>gi|62389168|ref|YP_224570.1| membrane protein [Corynebacterium glutamicum ATCC 13032]
>gi|62389172|ref|YP_224574.1| secreted protein [Corynebacterium glutamicum ATCC 13032]
>gi|62389179|ref|YP_224581.1| translation initiation inhibitor [Corynebacterium glutamicum ATCC 13032]
>gi|62389195|ref|YP_224597.1| PUTATIVE MEMBRANE PROTEIN [Corynebacterium glutamicum ATCC 13032]
>gi|62389201|ref|YP_224603.1| conserved hypothetical membrane protein [Corynebacterium glutamicum ATCC 13032]
>gi|62389202|ref|YP_224604.1| conserved secreted protein [Corynebacterium glutamicum ATCC 13032]
>gi|62389239|ref|YP_224641.1| putative membrane protein [Corynebacterium glutamicum ATCC 13032]
>gi|62389262|ref|YP_224664.1| PUTATIVE ACETYL TRANSFERASE PROTEIN [Corynebacterium glutamicum ATCC 13032]
>gi|62389287|ref|YP_224689.1| conserved secreted protein [Corynebacterium glutamicum ATCC 13032]
>gi|62389291|ref|YP_224693.1| conserved secreted protein [Corynebacterium glutamicum ATCC 13032]
>gi|62389309|ref|YP_224711.1| hypothetical membrane protein [Corynebacterium glutamicum ATCC 13032]
>gi|62389315|ref|YP_224717.1| putative phosphatase [Corynebacterium glutamicum ATCC 13032]
>gi|62389347|ref|YP_224749.1| hypothetical protein cg0530 [Corynebacterium glutamicum ATCC 13032]
>gi|62389351|ref|YP_224753.1| PUTATIVE INTEGRAL MEMBRANE PROTEIN [Corynebacterium glutamicum ATCC 13032]
>gi|62389373|ref|YP_224775.1| TRANSCRIPTION ANTITERMINATION PROTEIN NUSG [Corynebacterium glutamicum ATCC 13032]
>gi|62389382|ref|YP_224784.1| hypothetical protein cg0571 [Corynebacterium glutamicum ATCC 13032]
>gi|62389384|ref|YP_224786.1| PROBABLE 50S RIBOSOMAL SUBUNIT PROTEIN L7/L12 [Corynebacterium glutamicum ATCC 13032]
>gi|62389423|ref|YP_224825.1| PUTATIVE INTEGRAL MEMBRANE PROTEIN [Corynebacterium glutamicum ATCC 13032]
>gi|62389449|ref|YP_224851.1| secreted protein [Corynebacterium glutamicum ATCC 13032]
>gi|62389455|ref|YP_224857.1| 50S RIBOSOMAL PROTEIN L17 [Corynebacterium glutamicum ATCC 13032]
>gi|62389474|ref|YP_224876.1| hypothetical protein cg0678 [Corynebacterium glutamicum ATCC 13032]
>gi|62389485|ref|YP_224887.1| hypothetical protein cg0689 [Corynebacterium glutamicum ATCC 13032]
>gi|62389492|ref|YP_224894.1| hypothetical protein cg0697 [Corynebacterium glutamicum ATCC 13032]
>gi|62389500|ref|YP_224902.1| hypothetical protein predicted by Glimmer [Corynebacterium glutamicum ATCC 13032]
>gi|62389557|ref|YP_224959.1| ABC-type cobalamin/Fe3+-siderophores transport system, permease component [Corynebacterium glutamicum ATCC 13032]
>gi|62389562|ref|YP_224964.1| hypothetical protein cg0775 [Corynebacterium glutamicum ATCC 13032]
>gi|62389565|ref|YP_224967.1| ABC-type cobalamin/Fe3+-siderophores transport system, permease component [Corynebacterium glutamicum ATCC 13032]
>gi|62389612|ref|YP_225014.1| membrane protein [Corynebacterium glutamicum ATCC 13032]
>gi|62389638|ref|YP_225040.1| secreted protein [Corynebacterium glutamicum ATCC 13032]
>gi|62389641|ref|YP_225043.1| THYMIDYLATE KINASE, PUTATIVE [Corynebacterium glutamicum ATCC 13032]
>gi|62389673|ref|YP_225075.1| conserved hypothetical protein, possibly secreted [Corynebacterium glutamicum ATCC 13032]
>gi|62389709|ref|YP_225111.1| PUTATIVE RPF PROTEIN PRECURSOR [Corynebacterium glutamicum ATCC 13032]
>gi|62389749|ref|YP_225151.1| membrane protein [Corynebacterium glutamicum ATCC 13032]
>gi|62389774|ref|YP_225176.1| putative membrane protein [Corynebacterium glutamicum ATCC 13032]
>gi|62389797|ref|YP_225199.1| Cadmium resistance transporter [Corynebacterium glutamicum ATCC 13032]
>gi|62389848|ref|YP_225250.1| putative secreted protein [Corynebacterium glutamicum ATCC 13032]
>gi|62389860|ref|YP_225262.1| hypothetical protein cg1106 [Corynebacterium glutamicum ATCC 13032]
>gi|62389908|ref|YP_225310.1| similar to arabinose efflux permease [Corynebacterium glutamicum ATCC 13032]
>gi|62389976|ref|YP_225378.1| putative membrane protein [Corynebacterium glutamicum ATCC 13032]
>gi|62390025|ref|YP_225427.1| hypothetical protein cg1286 [Corynebacterium glutamicum ATCC 13032]
>gi|62390044|ref|YP_225446.1| Superfamily II DNA and RNA helicase [Corynebacterium glutamicum ATCC 13032]
>gi|62390087|ref|YP_225489.1| Transcription termination factor Rho [Corynebacterium glutamicum ATCC 13032]
>gi|62390096|ref|YP_225498.1| ATP synthase B chain [Corynebacterium glutamicum ATCC 13032]
>gi|62390140|ref|YP_225542.1| Ribose/xylose/arabinose/galactoside ABC-type transport system, permease component [Corynebacterium glutamicum ATCC 13032]
>gi|62390153|ref|YP_225555.1| putative membrane protein [Corynebacterium glutamicum ATCC 13032]
>gi|62390157|ref|YP_225559.1| Small-conductance mechanosensitive channel [Corynebacterium glutamicum ATCC 13032]
>gi|62390194|ref|YP_225596.1| putative membrane protein [Corynebacterium glutamicum ATCC 13032]
>gi|62390195|ref|YP_225597.1| putative secreted protein [Corynebacterium glutamicum ATCC 13032]
>gi|62390234|ref|YP_225636.1| putative membrane protein [Corynebacterium glutamicum ATCC 13032]
>gi|62390244|ref|YP_225646.1| Dephospho-CoA kinase [Corynebacterium glutamicum ATCC 13032]
>gi|62390278|ref|YP_225680.1| putative secreted protein [Corynebacterium glutamicum ATCC 13032]
>gi|62390329|ref|YP_225731.1| putative secreted protein [Corynebacterium glutamicum ATCC 13032]
>gi|62390347|ref|YP_225749.1| HYDROXYETHYLTHIAZOLE KINASE [Corynebacterium glutamicum ATCC 13032]
>gi|62390357|ref|YP_225759.1| putative secreted protein [Corynebacterium glutamicum ATCC 13032]
>gi|62390373|ref|YP_225775.1| Sec-independent protein secretion pathway component [Corynebacterium glutamicum ATCC 13032]
>gi|62390415|ref|YP_225817.1| secreted protease subunit, stomatin/prohibitin homolog [Corynebacterium glutamicum ATCC 13032]
>gi|62390420|ref|YP_225822.1| secreted cell wall-associated hydrolase (invasion-associated protein) [Corynebacterium glutamicum ATCC 13032]
>gi|62390451|ref|YP_225853.1| ABC-type multidrug transport system, permease component [Corynebacterium glutamicum ATCC 13032]
>gi|62390500|ref|YP_225902.1| hypothetical protein cg1823 [Corynebacterium glutamicum ATCC 13032]
>gi|62390501|ref|YP_225903.1| Transcription termination factor [Corynebacterium glutamicum ATCC 13032]
>gi|62390507|ref|YP_225909.1| putative signal peptidase, membrane protein, cleaves prepilin-like proteins [Corynebacterium glutamicum ATCC 13032]
>gi|62390534|ref|YP_225936.1| putative membrane protein [Corynebacterium glutamicum ATCC 13032]
>gi|62390547|ref|YP_225949.1| putative membrane protein [Corynebacterium glutamicum ATCC 13032]
>gi|62390554|ref|YP_225956.1| putative secreted protein [Corynebacterium glutamicum ATCC 13032]
>gi|62390555|ref|YP_225957.1| putative membrane protein [Corynebacterium glutamicum ATCC 13032]
>gi|62390557|ref|YP_225959.1| hypothetical protein cg1891 [Corynebacterium glutamicum ATCC 13032]

>gi|62390561|ref|YP_225963.1| putative secreted protein [Corynebacterium glutamicum ATCC 13032]
>gi|62390562|ref|YP_225964.1| putative secreted protein [Corynebacterium glutamicum ATCC 13032]
>gi|62390563|ref|YP_225965.1| putative secreted protein [Corynebacterium glutamicum ATCC 13032]
>gi|62390584|ref|YP_225986.1| putative secreted protein [Corynebacterium glutamicum ATCC 13032]
>gi|62390587|ref|YP_225989.1| hypothetical protein cg1921 [Corynebacterium glutamicum ATCC 13032]
>gi|62390635|ref|YP_226037.1| hypothetical protein cg1975 [Corynebacterium glutamicum ATCC 13032]
>gi|62390669|ref|YP_226071.1| Permease of the major facilitator superfamily [Corynebacterium glutamicum ATCC 13032]
>gi|62390671|ref|YP_226073.1| putative secreted protein [Corynebacterium glutamicum ATCC 13032]
>gi|62390672|ref|YP_226074.1| hypothetical protein cg2014 [Corynebacterium glutamicum ATCC 13032]
>gi|62390690|ref|YP_226092.1| putative membrane protein [Corynebacterium glutamicum ATCC 13032]
>gi|62390691|ref|YP_226093.1| putative secreted protein [Corynebacterium glutamicum ATCC 13032]
>gi|62390698|ref|YP_226100.1| putative transcriptional regulator [Corynebacterium glutamicum ATCC 13032]
>gi|62390712|ref|YP_226114.1| putative membrane protein [Corynebacterium glutamicum ATCC 13032]
>gi|62390729|ref|YP_226131.1| putative phage integrase (C-terminal fragment) [Corynebacterium glutamicum ATCC 13032]
>gi|62390731|ref|YP_226133.1| Di-and tricarboxylate transporter [Corynebacterium glutamicum ATCC 13032]
>gi|62390750|ref|YP_226152.1| RNA POLYMERASE SIGMA 70 FACTOR [Corynebacterium glutamicum ATCC 13032]
>gi|62390753|ref|YP_226155.1| putative membrane protein [Corynebacterium glutamicum ATCC 13032]
>gi|62390766|ref|YP_226168.1| putative membrane protein [Corynebacterium glutamicum ATCC 13032]
>gi|62390798|ref|YP_226200.1| membrane protein, BioY family [Corynebacterium glutamicum ATCC 13032]
>gi|62390814|ref|YP_226216.1| putative secreted protein [Corynebacterium glutamicum ATCC 13032]
>gi|62390816|ref|YP_226218.1| RIBOSOMAL PROTEIN S15 [Corynebacterium glutamicum ATCC 13032]
>gi|62390825|ref|YP_226227.1| Translation initiation factor 2 (GTPase) [Corynebacterium glutamicum ATCC 13032]
>gi|62390846|ref|YP_226248.1| putative membrane protein [Corynebacterium glutamicum ATCC 13032]
>gi|62390864|ref|YP_226266.1| TRANSLATION ELONGATION FACTOR TS (EF-TS) [Corynebacterium glutamicum ATCC 13032]
>gi|62390888|ref|YP_226290.1| PUTATIVE SECRETED LIPOPROTEIN [Corynebacterium glutamicum ATCC 13032]
>gi|62390891|ref|YP_226293.1| 30S RIBOSOMAL PROTEIN S16 [Corynebacterium glutamicum ATCC 13032]
>gi|62390899|ref|YP_226301.1| Signal recognition particle GTPase [Corynebacterium glutamicum ATCC 13032]
>gi|62390934|ref|YP_226336.1| putative membrane protein [Corynebacterium glutamicum ATCC 13032]
>gi|62390940|ref|YP_226342.1| putative secreted protein [Corynebacterium glutamicum ATCC 13032]
>gi|62390989|ref|YP_226391.1| Cell division initiation protein-Antigen 84 homolog [Corynebacterium glutamicum ATCC 13032]
>gi|62391008|ref|YP_226410.1| putative membrane protein [Corynebacterium glutamicum ATCC 13032]
>gi|62391022|ref|YP_226424.1| putative secreted or membrane protein [Corynebacterium glutamicum ATCC 13032]
>gi|62391028|ref|YP_226430.1| NPL/P60 FAMILY SECRETED PROTEIN [Corynebacterium glutamicum ATCC 13032]
>gi|62391046|ref|YP_226448.1| DIHYDROLIPOAMIDE SUCCINYLTRANSFERASE [Corynebacterium glutamicum ATCC 13032]
>gi|62391066|ref|YP_226468.1| hypothetical protein cg2444 [Corynebacterium glutamicum ATCC 13032]
>gi|62391092|ref|YP_226494.1| weakly conserved hypothetical protein [Corynebacterium glutamicum ATCC 13032]
>gi|62391093|ref|YP_226495.1| hypothetical protein cg4003 [Corynebacterium glutamicum ATCC 13032]
>gi|62391110|ref|YP_226512.1| secreted guanine-specific ribonuclease [Corynebacterium glutamicum ATCC 13032]
>gi|62391122|ref|YP_226524.1| hypothetical protein cg2504 [Corynebacterium glutamicum ATCC 13032]
>gi|62391141|ref|YP_226543.1| putative secreted or membrane protein [Corynebacterium glutamicum ATCC 13032]
>gi|62391147|ref|YP_226549.1| hypothetical protein cg2533 [Corynebacterium glutamicum ATCC 13032]
>gi|62391167|ref|YP_226569.1| PROBABLE RIBOKINASE PROTEIN [Corynebacterium glutamicum ATCC 13032]
>gi|62391174|ref|YP_226576.1| secreted protein potentially involved into thiamin biosynthesis [Corynebacterium glutamicum ATCC 13032]
>gi|62391177|ref|YP_226579.1| hypothetical protein cg2564 [Corynebacterium glutamicum ATCC 13032]
>gi|62391186|ref|YP_226588.1| 30S RIBOSOMAL PROTEIN S20 [Corynebacterium glutamicum ATCC 13032]
>gi|62391207|ref|YP_226609.1| PROBABLE RIBONUCLEASE E (RNASE E) PROTEIN [Corynebacterium glutamicum ATCC 13032]
>gi|62391214|ref|YP_226616.1| putative secreted or membrane protein [Corynebacterium glutamicum ATCC 13032]
>gi|62391251|ref|YP_226653.1| PUTATIVE BENZOATE TRANSPORT PROTEIN [Corynebacterium glutamicum ATCC 13032]
>gi|62391262|ref|YP_226664.1| putative membrane protein-fragment [Corynebacterium glutamicum ATCC 13032]
>gi|62391283|ref|YP_226685.1| membrane protein DedA family [Corynebacterium glutamicum ATCC 13032]
>gi|62391298|ref|YP_226700.1| putative membrane protein [Corynebacterium glutamicum ATCC 13032]
>gi|62391318|ref|YP_226720.1| putative membrane protein [Corynebacterium glutamicum ATCC 13032]
>gi|62391331|ref|YP_226733.1| Permease of the major facilitator superfamily [Corynebacterium glutamicum ATCC 13032]
>gi|62391337|ref|YP_226739.1| SECRETED PEPTIDASE, M23/M37 FAMILY [Corynebacterium glutamicum ATCC 13032]
>gi|62391350|ref|YP_226752.1| putative secreted protein [Corynebacterium glutamicum ATCC 13032]
>gi|62391360|ref|YP_226762.1| putative membrane protein [Corynebacterium glutamicum ATCC 13032]
>gi|62391368|ref|YP_226770.1| putative membrane protein [Corynebacterium glutamicum ATCC 13032]
>gi|62391413|ref|YP_226815.1| ABC-type phosphate transport system, secreted component [Corynebacterium glutamicum ATCC 13032]
>gi|62391462|ref|YP_226864.1| hypothetical protein cg2906 [Corynebacterium glutamicum ATCC 13032]
>gi|62391467|ref|YP_226869.1| ABC-type Mn/Zn transport system, secreted Mn/Zn-binding (lipo)protein (surface adhesin) [Corynebacterium glutamicum ATCC 13032]
>gi|62391469|ref|YP_226871.1| ABC-type Mn2+/Zn2+ transport system, permease component [Corynebacterium glutamicum ATCC 13032]
>gi|62391494|ref|YP_226896.1| LysE type translocator [Corynebacterium glutamicum ATCC 13032]
>gi|62391499|ref|YP_226901.1| CarD-like transcriptional regulator [Corynebacterium glutamicum ATCC 13032]
>gi|62391505|ref|YP_226907.1| putative secreted protein [Corynebacterium glutamicum ATCC 13032]
>gi|62391527|ref|YP_226929.1| putative membrane protein [Corynebacterium glutamicum ATCC 13032]
>gi|62391538|ref|YP_226940.1| PUTATIVE SPERMIDINE SYNTHASE TRANSMEMBRANE PROTEIN [Corynebacterium glutamicum ATCC 13032]
>gi|62391558|ref|YP_226960.1| hypothetical protein cg3016 [Corynebacterium glutamicum ATCC 13032]
>gi|62391562|ref|YP_226964.1| putative membrane protein [Corynebacterium glutamicum ATCC 13032]

>gi|62391573|ref|YP_226975.1| putative secreted protein [Corynebacterium glutamicum ATCC 13032]
>gi|62391579|ref|YP_226981.1| Permease of the major facilitator superfamilyynebacterium glutamicum] [Corynebacterium glutamicum ATCC 13032]
>gi|62391633|ref|YP_227035.1| CHAPERONE WITH DNAK, HEAT SHOCK PROTEIN (DNAJ PROTEIN) [Corynebacterium glutamicum ATCC 13032]
>gi|62391667|ref|YP_227069.1| putative membrane protein [Corynebacterium glutamicum ATCC 13032]
>gi|62391670|ref|YP_227072.1| putative membrane protein [Corynebacterium glutamicum ATCC 13032]
>gi|62391693|ref|YP_227095.1| putative secreted protein [Corynebacterium glutamicum ATCC 13032]
>gi|62391698|ref|YP_227100.1| putative membrane protein [Corynebacterium glutamicum ATCC 13032]
>gi|62391713|ref|YP_227115.1| putative secreted protein [Corynebacterium glutamicum ATCC 13032]
>gi|62391726|ref|YP_227128.1| Putative secreted protein [Corynebacterium glutamicum ATCC 13032]
>gi|62391739|ref|YP_227141.1| cell envelope-related transcriptional regulator [Corynebacterium glutamicum ATCC 13032]
>gi|62391742|ref|YP_227144.1| putative secreted protein [Corynebacterium glutamicum ATCC 13032]
>gi|62391770|ref|YP_227172.1| hypothetical protein cg3244 [Corynebacterium glutamicum ATCC 13032]
>gi|62391780|ref|YP_227182.1| putative membrane protein [Corynebacterium glutamicum ATCC 13032]
>gi|62391787|ref|YP_227189.1| hypothetical protein cg3263 [Corynebacterium glutamicum ATCC 13032]
>gi|62391804|ref|YP_227206.1| putative secreted protein [Corynebacterium glutamicum ATCC 13032]
>gi|62391805|ref|YP_227207.1| PROBABLE CATION-TRANSPORTING ATPASE TRANSMEMBRANE PROTEIN [Corynebacterium glutamicum ATCC 13032]
>gi|62391807|ref|YP_227209.1| hypothetical protein predicted by Glimmer [Corynebacterium glutamicum ATCC 13032]
>gi|62391824|ref|YP_227226.1| Cation transport ATPase [Corynebacterium glutamicum ATCC 13032]
>gi|62391825|ref|YP_227227.1| Permease of the major facilitator superfamily [Corynebacterium glutamicum ATCC 13032]
>gi|62391826|ref|YP_227228.1| Transcriptional regulator PadR-like family [Corynebacterium glutamicum ATCC 13032]
>gi|62391829|ref|YP_227231.1| SINGLE-STRANDED DNA-BINDING PROTEIN [Corynebacterium glutamicum ATCC 13032]
>gi|62391857|ref|YP_227259.1| putative membrane protein [Corynebacterium glutamicum ATCC 13032]
>gi|62391880|ref|YP_227282.1| ANTHRANILATE PHOSPHORIBOSYLTRANSFERASE [Corynebacterium glutamicum ATCC 13032]
>gi|62391885|ref|YP_227287.1| putative ribitol-specific enzyme II of PTS system [Corynebacterium glutamicum ATCC 13032]
>gi|62391897|ref|YP_227299.1| hypothetical protein cg3378 [Corynebacterium glutamicum ATCC 13032]
>gi|62391923|ref|YP_227325.1| putative membrane protein [Corynebacterium glutamicum ATCC 13032]
>gi|62391927|ref|YP_227329.1| Copper chaperone [Corynebacterium glutamicum ATCC 13032]
>gi|62391932|ref|YP_227334.1| putative membrane protein [Corynebacterium glutamicum ATCC 13032]
>gi|62391941|ref|YP_227343.1| Predicted transcriptional regulator involved in chromosome patitioning [Corynebacterium glutamicum ATCC 13032]
>gi|62391947|ref|YP_227349.1| 50S RIBOSOMAL PROTEIN L34 [Corynebacterium glutamicum ATCC 13032]
>gi|21909550|ref|NP_663818.1| putative secreted protein [Streptococcus pyogenes MGAS315]
>gi|21909577|ref|NP_663845.1| 50S ribosomal protein L4 [Streptococcus pyogenes MGAS315]
>gi|21909584|ref|NP_663852.1| 50S ribosomal protein L29 [Streptococcus pyogenes MGAS315]
>gi|21909653|ref|NP_663921.1| putative V-type Na+ -ATPase subunit E [Streptococcus pyogenes MGAS315]
>gi|21909687|ref|NP_663955.1| putative biotin synthase [Streptococcus pyogenes MGAS315]
>gi|21909720|ref|NP_663988.1| hypothetical protein SpyM3_0184 [Streptococcus pyogenes MGAS315]
>gi|21909778|ref|NP_664046.1| putative heat shock protein [Streptococcus pyogenes MGAS315]
>gi|21909809|ref|NP_664077.1| putative phosphatase [Streptococcus pyogenes MGAS315]
>gi|21909816|ref|NP_664084.1| putative ferrichrome ABC transporter (permease) [Streptococcus pyogenes MGAS315]
>gi|21909842|ref|NP_664110.1| hypothetical protein SpyM3_0306 [Streptococcus pyogenes MGAS315]
>gi|21909863|ref|NP_664131.1| putative ribosome recycling factor [Streptococcus pyogenes MGAS315]
>gi|21909867|ref|NP_664135.1| hypothetical protein SpyM3_0331 [Streptococcus pyogenes MGAS315]
>gi|21909875|ref|NP_664143.1| putative bacteriocin [Streptococcus pyogenes MGAS315]
>gi|21909876|ref|NP_664144.1| hypothetical protein SpyM3_0340 [Streptococcus pyogenes MGAS315]
>gi|21909944|ref|NP_664212.1| hypothetical protein SpyM3_0408 [Streptococcus pyogenes MGAS315]
>gi|21909947|ref|NP_664215.1| hypothetical protein SpyM3_0411 [Streptococcus pyogenes MGAS315]
>gi|21909962|ref|NP_664230.1| hypothetical protein SpyM3_0426 [Streptococcus pyogenes MGAS315]
>gi|21910016|ref|NP_664284.1| streptolysin S associated protein [Streptococcus pyogenes MGAS315]
>gi|21910021|ref|NP_664289.1| hypothetical protein SpyM3_0485 [Streptococcus pyogenes MGAS315]
>gi|21910052|ref|NP_664320.1| 30S ribosomal protein S21 [Streptococcus pyogenes MGAS315]
>gi|21910072|ref|NP_664340.1| hypothetical protein SpyM3_0536 [Streptococcus pyogenes MGAS315]
>gi|21910084|ref|NP_664352.1| hypothetical protein SpyM3_0548 [Streptococcus pyogenes MGAS315]
>gi|21910088|ref|NP_664356.1| 50S ribosomal protein L21 [Streptococcus pyogenes MGAS315]
>gi|21910116|ref|NP_664384.1| putative fructose-specific enzyme II [Streptococcus pyogenes MGAS315]
>gi|21910159|ref|NP_664427.1| hypothetical protein SpyM3_0623 [Streptococcus pyogenes MGAS315]
>gi|21910192|ref|NP_664460.1| putative ABC transporter (ATP-binding protein) [Streptococcus pyogenes MGAS315]
>gi|21910216|ref|NP_664484.1| hypothetical protein SpyM3_0680 [Streptococcus pyogenes MGAS315]
>gi|21910238|ref|NP_664506.1| hypothetical protein SpyM3_0702 [Streptococcus pyogenes MGAS315]
>gi|21910263|ref|NP_664531.1| hypothetical protein SpyM3_0727 [Streptococcus pyogenes MGAS315]
>gi|21910266|ref|NP_664534.1| hypothetical protein SpyM3_0730 [Streptococcus pyogenes MGAS315]
>gi|21910274|ref|NP_664542.1| collagen-like protein SclB [Streptococcus pyogenes MGAS315]
>gi|21910290|ref|NP_664558.1| 50S ribosomal protein L7/L12 [Streptococcus pyogenes MGAS315]
>gi|21910310|ref|NP_664578.1| hypothetical protein SpyM3_0774 [Streptococcus pyogenes MGAS315]
>gi|21910359|ref|NP_664627.1| hypothetical protein SpyM3_0823 [Streptococcus pyogenes MGAS315]
>gi|21910366|ref|NP_664634.1| putative decarboxylase gamma chain [Streptococcus pyogenes MGAS315]
>gi|21910399|ref|NP_664667.1| hypothetical protein SpyM3_0863 [Streptococcus pyogenes MGAS315]

>gi|21910408|ref|NP_664676.1| 30S ribosomal protein S20 [Streptococcus pyogenes MGAS315]
>gi|21910432|ref|NP_664700.1| hypothetical protein SpyM3_0896 [Streptococcus pyogenes MGAS315]
>gi|21910434|ref|NP_664702.1| hypothetical protein SpyM3_0898 [Streptococcus pyogenes MGAS315]
>gi|21910436|ref|NP_664704.1| hypothetical protein SpyM3_0900 [Streptococcus pyogenes MGAS315]
>gi|21910459|ref|NP_664727.1| putative holin - phage associated [Streptococcus pyogenes MGAS315]
>gi|21910462|ref|NP_664730.1| hypothetical protein SpyM3_0926 [Streptococcus pyogenes MGAS315]
>gi|21910481|ref|NP_664749.1| hypothetical protein SpyM3_0945 [Streptococcus pyogenes MGAS315]
>gi|21910568|ref|NP_664836.1| protein G-related alpha 2M-bindingprotein [Streptococcus pyogenes MGAS315]
>gi|21910573|ref|NP_664841.1| hypothetical protein SpyM3_1037 [Streptococcus pyogenes MGAS315]
>gi|21910617|ref|NP_664885.1| hypothetical protein SpyM3_1081 [Streptococcus pyogenes MGAS315]
>gi|21910624|ref|NP_664892.1| hypothetical protein SpyM3_1088 [Streptococcus pyogenes MGAS315]
>gi|21910719|ref|NP_664987.1| hypothetical protein SpyM3_1183 [Streptococcus pyogenes MGAS315]
>gi|21910721|ref|NP_664989.1| hypothetical protein SpyM3_1185 [Streptococcus pyogenes MGAS315]
>gi|21910748|ref|NP_665016.1| hypothetical protein SpyM3_1212 [Streptococcus pyogenes MGAS315]
>gi|21910787|ref|NP_665055.1| hypothetical protein SpyM3_1251 [Streptococcus pyogenes MGAS315]
>gi|21910804|ref|NP_665072.1| hypothetical protein SpyM3_1268 [Streptococcus pyogenes MGAS315]
>gi|21910859|ref|NP_665127.1| hypothetical protein SpyM3_1323 [Streptococcus pyogenes MGAS315]
>gi|21910904|ref|NP_665172.1| hypothetical protein SpyM3_1368 [Streptococcus pyogenes MGAS315]
>gi|21910910|ref|NP_665178.1| putative DNA-directed RNA polymerase omega chain [Streptococcus pyogenes MGAS315]
>gi|21910949|ref|NP_665217.1| putative holin - phage associated [Streptococcus pyogenes MGAS315]
>gi|21910952|ref|NP_665220.1| hypothetical protein SpyM3_1416 [Streptococcus pyogenes MGAS315]
>gi|21910957|ref|NP_665225.1| putative tail protein - phage associated [Streptococcus pyogenes MGAS315]
>gi|21910982|ref|NP_665250.1| hypothetical protein SpyM3_1446 [Streptococcus pyogenes MGAS315]
>gi|21911002|ref|NP_665270.1| putative glycerol uptake facilitator [Streptococcus pyogenes MGAS315]
>gi|21911013|ref|NP_665281.1| hypothetical protein SpyM3_1477 [Streptococcus pyogenes MGAS315]
>gi|21911038|ref|NP_665306.1| putative ABC transporter (permease) [Streptococcus pyogenes MGAS315]
>gi|21911045|ref|NP_665313.1| hypothetical protein SpyM3_1509 [Streptococcus pyogenes MGAS315]
>gi|21911068|ref|NP_665336.1| putative Hsp-70 cofactor [Streptococcus pyogenes MGAS315]
>gi|21911090|ref|NP_665358.1| hypothetical protein SpyM3_1554 [Streptococcus pyogenes MGAS315]
>gi|21911095|ref|NP_665363.1| putative ferrichrome ABC transporter (permease) [Streptococcus pyogenes MGAS315]
>gi|21911125|ref|NP_665393.1| hypothetical protein SpyM3_1589 [Streptococcus pyogenes MGAS315]
>gi|21911145|ref|NP_665413.1| hypothetical protein SpyM3_1609 [Streptococcus pyogenes MGAS315]
>gi|21911169|ref|NP_665437.1| putative DNA-directed RNA polymerase delta subunit [Streptococcus pyogenes MGAS315]
>gi|21911235|ref|NP_665503.1| hypothetical protein SpyM3_1699 [Streptococcus pyogenes MGAS315]
>gi|21911238|ref|NP_665506.1| putative collagen-like protein [Streptococcus pyogenes MGAS315]
>gi|21911259|ref|NP_665527.1| hypothetical protein SpyM3_1723 [Streptococcus pyogenes MGAS315]
>gi|21911263|ref|NP_665531.1| antiphagocytic M protein, type 3 [Streptococcus pyogenes MGAS315]
>gi|21911267|ref|NP_665535.1| immunogenic secreted protein precursor [Streptococcus pyogenes MGAS315]
>gi|21911273|ref|NP_665541.1| hypothetical protein SpyM3_1737 [Streptococcus pyogenes MGAS315]
>gi|21911302|ref|NP_665570.1| putative heat shock protein [Streptococcus pyogenes MGAS315]
>gi|21911354|ref|NP_665622.1| putative cadmium resistance protein [Streptococcus pyogenes MGAS315]
>gi|21911367|ref|NP_665635.1| hypothetical protein SpyM3_1831 [Streptococcus pyogenes MGAS315]
>gi|21911372|ref|NP_665640.1| 50S ribosomal protein L9 [Streptococcus pyogenes MGAS315]
>gi|28572187|ref|NP_788967.1| conserved hypothetical membrane protein [Tropheryma whipplei TW08/27]
>gi|28572205|ref|NP_788985.1| putative integral membrane protein [Tropheryma whipplei TW08/27]
>gi|28572206|ref|NP_788986.1| putative polysaccharide biosynthesis protein [Tropheryma whipplei TW08/27]
>gi|28572214|ref|NP_788994.1| putative integral membrane protein [Tropheryma whipplei TW08/27]
>gi|28572220|ref|NP_789000.1| putative secreted protein [Tropheryma whipplei TW08/27]
>gi|28572221|ref|NP_789001.1| putative integral membrane protein [Tropheryma whipplei TW08/27]
>gi|28572280|ref|NP_789060.1| 50s ribosomal protein L9 [Tropheryma whipplei TW08/27]
>gi|28572288|ref|NP_789068.1| 50s ribosomal protein L28 [Tropheryma whipplei TW08/27]
>gi|28572306|ref|NP_789086.1| hypothetical phase-variable integral membrane protein [Tropheryma whipplei TW08/27]
>gi|28572320|ref|NP_789100.1| WiSP family protein [Tropheryma whipplei TW08/27]
>gi|28572338|ref|NP_789118.1| putative integral membrane protein [Tropheryma whipplei TW08/27]
>gi|28572343|ref|NP_789123.1| putative secreted protein [Tropheryma whipplei TW08/27]
>gi|28572349|ref|NP_789129.1| putative membrane protein [Tropheryma whipplei TW08/27]
>gi|28572352|ref|NP_789132.1| possible integral membrane protein [Tropheryma whipplei TW08/27]
>gi|28572353|ref|NP_789133.1| possible integral membrane protein [Tropheryma whipplei TW08/27]
>gi|28572361|ref|NP_789141.1| putative ABC transporter branched chain amino acid transport permease [Tropheryma whipplei TW08/27]
>gi|28572377|ref|NP_789157.1| 50s ribosomal protein L14 [Tropheryma whipplei TW08/27]
>gi|28572383|ref|NP_789163.1| 30s ribosomal protein S5 [Tropheryma whipplei TW08/27]
>gi|28572393|ref|NP_789173.1| 50S ribosomal protein L17 [Tropheryma whipplei TW08/27]
>gi|28572408|ref|NP_789188.1| putative cell division protein [Tropheryma whipplei TW08/27]
>gi|28572438|ref|NP_789218.1| putative integral membrane protein [Tropheryma whipplei TW08/27]
>gi|28572513|ref|NP_789293.1| putative integral membrane protein [Tropheryma whipplei TW08/27]
>gi|28572578|ref|NP_789358.1| putative integral membrane protein [Tropheryma whipplei TW08/27]
>gi|28572598|ref|NP_789378.1| putative integral membrane protein [Tropheryma whipplei TW08/27]
>gi|28572616|ref|NP_789396.1| putative membrane protein [Tropheryma whipplei TW08/27]
>gi|28572618|ref|NP_789398.1| protein-export membrane protein SecG [Tropheryma whipplei TW08/27]
>gi|28572649|ref|NP_789429.1| putative secreted protein [Tropheryma whipplei TW08/27]

>gi|28572679|ref|NP_789459.1| putative integral membrane protein [Tropheryma whipplei TW08/27]
>gi|28572686|ref|NP_789466.1| putative integral membrane protein [Tropheryma whipplei TW08/27]
>gi|28572700|ref|NP_789480.1| WiSP family protein [Tropheryma whipplei TW08/27]
>gi|28572707|ref|NP_789487.1| WiSP family protein [Tropheryma whipplei TW08/27]
>gi|28572708|ref|NP_789488.1| conserved putative integral membrane protein (BioY-family) [Tropheryma whipplei TW08/27]
>gi|28572709|ref|NP_789489.1| WiSP family protein [Tropheryma whipplei TW08/27]
>gi|28572718|ref|NP_789498.1| WiSP family protein [Tropheryma whipplei TW08/27]
>gi|28572723|ref|NP_789503.1| putative integral membrane transport protein [Tropheryma whipplei TW08/27]
>gi|28572735|ref|NP_789515.1| putative membrane protein [Tropheryma whipplei TW08/27]
>gi|28572741|ref|NP_789521.1| putative secreted protein [Tropheryma whipplei TW08/27]
>gi|28572748|ref|NP_789528.1| hypothetical protein TW600 [Tropheryma whipplei TW08/27]
>gi|28572765|ref|NP_789545.1| putative membrane protein [Tropheryma whipplei TW08/27]
>gi|28572767|ref|NP_789547.1| proline/alanine-rich repetetive membrane anchored protein [Tropheryma whipplei TW08/27]
>gi|28572770|ref|NP_789550.1| putative membrane protein [Tropheryma whipplei TW08/27]
>gi|28572771|ref|NP_789551.1| WiSP family protein [Tropheryma whipplei TW08/27]
>gi|28572776|ref|NP_789556.1| hypothetical protein TW630 [Tropheryma whipplei TW08/27]
>gi|28572780|ref|NP_789560.1| putative membrane protein [Tropheryma whipplei TW08/27]
>gi|28572782|ref|NP_789562.1| hypothetical protein TW637 [Tropheryma whipplei TW08/27]
>gi|28572787|ref|NP_789567.1| WiSP family protein [Tropheryma whipplei TW08/27]
>gi|28572794|ref|NP_789574.1| putative integral membrane protein [Tropheryma whipplei TW08/27]
>gi|28572838|ref|NP_789618.1| hypothetical protein TW697 [Tropheryma whipplei TW08/27]
>gi|28572869|ref|NP_789649.1| 50s ribosomal protein L7/L12 [Tropheryma whipplei TW08/27]
>gi|28572914|ref|NP_789694.1| putative membrane protein [Tropheryma whipplei TW08/27]
>gi|28572920|ref|NP_789700.1| putative membrane protein [Tropheryma whipplei TW08/27]
>gi|15081481|ref|NP_149994.1| ParB protein [Clostridium perfringens str. 13]
>gi|15081482|ref|NP_149995.1| hypothetical protein PCP03 [Clostridium perfringens str. 13]
>gi|15081498|ref|NP_150011.1| hypothetical protein PCP18 [Clostridium perfringens str. 13]
>gi|15081510|ref|NP_150023.1| hypothetical protein PCP30 [Clostridium perfringens str. 13]
>gi|15081523|ref|NP_150036.1| hypothetical protein PCP43 [Clostridium perfringens str. 13]
>gi|15081528|ref|NP_150041.1| hypothetical protein PCP48 [Clostridium perfringens str. 13]
>gi|15081530|ref|NP_150043.1| hypothetical protein PCP50 [Clostridium perfringens str. 13]
>gi|15081536|ref|NP_150049.1| hypothetical protein PCP56 [Clostridium perfringens str. 13]
>gi|15081539|ref|NP_150052.1| hypothetical protein PCP59 [Clostridium perfringens str. 13]
>gi|15081541|ref|NP_150054.1| probable transcription regulator phage-related [Clostridium perfringens str. 13]
>gi|15081542|ref|NP_150055.1| hypothetical protein PCP62 [Clostridium perfringens str. 13]
>gi|15081543|ref|NP_150056.1| hypothetical protein PCP63 [Clostridium perfringens str. 13]
>gi|18308990|ref|NP_560924.1| hypothetical protein CPE0008 [Clostridium perfringens str. 13]
>gi|18308999|ref|NP_560933.1| hypothetical protein CPE0017 [Clostridium perfringens str. 13]
>gi|18309031|ref|NP_560965.1| hypothetical protein CPE0049 [Clostridium perfringens str. 13]
>gi|18309044|ref|NP_560978.1| hypothetical protein CPE0062 [Clostridium perfringens str. 13]
>gi|18309060|ref|NP_560994.1| probable transcriptional regulator [Clostridium perfringens str. 13]
>gi|18309084|ref|NP_561018.1| hypothetical protein CPE0102 [Clostridium perfringens str. 13]
>gi|18309094|ref|NP_561028.1| hypothetical protein CPE0112 [Clostridium perfringens str. 13]
>gi|18309097|ref|NP_561031.1| probable N-acetylmuramoyl-L-alanine amidase [Clostridium perfringens str. 13]
>gi|18309106|ref|NP_561040.1| hypothetical protein CPE0124 [Clostridium perfringens str. 13]
>gi|18309108|ref|NP_561042.1| hypothetical protein CPE0126 [Clostridium perfringens str. 13]
>gi|18309112|ref|NP_561046.1| hypothetical protein CPE0130 [Clostridium perfringens str. 13]
>gi|18309116|ref|NP_561050.1| hypothetical protein CPE0134 [Clostridium perfringens str. 13]
>gi|18309125|ref|NP_561059.1| phage-related hypothetical protein [Clostridium perfringens str. 13]
>gi|18309163|ref|NP_561097.1| hypothetical protein CPE0181 [Clostridium perfringens str. 13]
>gi|18309192|ref|NP_561126.1| hypothetical protein CPE0210 [Clostridium perfringens str. 13]
>gi|18309193|ref|NP_561127.1| hypothetical protein CPE0211 [Clostridium perfringens str. 13]
>gi|18309198|ref|NP_561132.1| probable exonuclease [Clostridium perfringens str. 13]
>gi|18309204|ref|NP_561138.1| hypothetical protein CPE0222 [Clostridium perfringens str. 13]
>gi|18309220|ref|NP_561154.1| hypothetical protein CPE0238 [Clostridium perfringens str. 13]
>gi|18309226|ref|NP_561160.1| hypothetical protein CPE0244 [Clostridium perfringens str. 13]
>gi|18309231|ref|NP_561165.1| hypothetical protein CPE0249 [Clostridium perfringens str. 13]
>gi|18309313|ref|NP_561247.1| hypothetical protein CPE0331 [Clostridium perfringens str. 13]
>gi|18309314|ref|NP_561248.1| alkaline phosphatase-like protein [Clostridium perfringens str. 13]
>gi|18309383|ref|NP_561317.1| hypothetical protein CPE0401 [Clostridium perfringens str. 13]
>gi|18309384|ref|NP_561318.1| hypothetical protein CPE0402 [Clostridium perfringens str. 13]
>gi|18309411|ref|NP_561345.1| hypothetical protein CPE0429 [Clostridium perfringens str. 13]
>gi|18309424|ref|NP_561358.1| hypothetical protein CPE0442 [Clostridium perfringens str. 13]
>gi|18309434|ref|NP_561368.1| probable enterotoxin [Clostridium perfringens str. 13]
>gi|18309485|ref|NP_561419.1| hypothetical protein CPE0503 [Clostridium perfringens str. 13]
>gi|18309517|ref|NP_561451.1| hypothetical protein CPE0535 [Clostridium perfringens str. 13]
>gi|18309522|ref|NP_561456.1| hypothetical protein CPE0540 [Clostridium perfringens str. 13]
>gi|18309527|ref|NP_561461.1| hypothetical protein CPE0545 [Clostridium perfringens str. 13]
>gi|18309528|ref|NP_561462.1| hypothetical protein CPE0546 [Clostridium perfringens str. 13]
>gi|18309552|ref|NP_561486.1| hypothetical protein CPE0570 [Clostridium perfringens str. 13]

>gi|18309608|ref|NP_561542.1| hypothetical protein CPE0626 [Clostridium perfringens str. 13]
>gi|18309612|ref|NP_561546.1| hypothetical protein CPE0630 [Clostridium perfringens str. 13]
>gi|18309657|ref|NP_561591.1| hypothetical protein CPE0675 [Clostridium perfringens str. 13]
>gi|18309687|ref|NP_561621.1| hypothetical protein CPE0705 [Clostridium perfringens str. 13]
>gi|18309695|ref|NP_561629.1| hypothetical protein CPE0713 [Clostridium perfringens str. 13]
>gi|18309709|ref|NP_561643.1| hypothetical protein CPE0727 [Clostridium perfringens str. 13]
>gi|18309733|ref|NP_561667.1| hypothetical protein CPE0751 [Clostridium perfringens str. 13]
>gi|18309767|ref|NP_561701.1| probable regulatory protein [Clostridium perfringens str. 13]
>gi|18309804|ref|NP_561738.1| PTS system [Clostridium perfringens str. 13]
>gi|18309811|ref|NP_561745.1| hypothetical protein CPE0829 [Clostridium perfringens str. 13]
>gi|18309814|ref|NP_561748.1| hypothetical protein CPE0832 [Clostridium perfringens str. 13]
>gi|18309842|ref|NP_561776.1| probable gluconate permease [Clostridium perfringens str. 13]
>gi|18309847|ref|NP_561781.1| hypothetical protein CPE0865 [Clostridium perfringens str. 13]
>gi|18309852|ref|NP_561786.1| two-component sensor histidine kinase [Clostridium perfringens str. 13]
>gi|18309853|ref|NP_561787.1| hypothetical protein CPE0871 [Clostridium perfringens str. 13]
>gi|18309855|ref|NP_561789.1| hypothetical protein CPE0873 [Clostridium perfringens str. 13]
>gi|18309865|ref|NP_561799.1| hypothetical protein CPE0883 [Clostridium perfringens str. 13]
>gi|18309868|ref|NP_561802.1| hypothetical protein CPE0886 [Clostridium perfringens str. 13]
>gi|18309883|ref|NP_561817.1| propanediol utilization protein [Clostridium perfringens str. 13]
>gi|18309885|ref|NP_561819.1| propanediol utilization protein [Clostridium perfringens str. 13]
>gi|18309891|ref|NP_561825.1| ethanolamine utilization protein [Clostridium perfringens str. 13]
>gi|18309902|ref|NP_561836.1| hypothetical protein CPE0920 [Clostridium perfringens str. 13]
>gi|18309924|ref|NP_561858.1| hypothetical protein CPE0942 [Clostridium perfringens str. 13]
>gi|18309925|ref|NP_561859.1| hypothetical protein CPE0943 [Clostridium perfringens str. 13]
>gi|18309937|ref|NP_561871.1| collagen-like protein [Clostridium perfringens str. 13]
>gi|18309938|ref|NP_561872.1| hypothetical protein CPE0956 [Clostridium perfringens str. 13]
>gi|18309944|ref|NP_561878.1| hypothetical protein CPE0962 [Clostridium perfringens str. 13]
>gi|18309950|ref|NP_561884.1| hypothetical protein CPE0968 [Clostridium perfringens str. 13]
>gi|18309965|ref|NP_561899.1| hypothetical protein CPE0983 [Clostridium perfringens str. 13]
>gi|18309984|ref|NP_561918.1| hypothetical protein CPE1002 [Clostridium perfringens str. 13]
>gi|18309985|ref|NP_561919.1| hypothetical protein CPE1003 [Clostridium perfringens str. 13]
>gi|18310030|ref|NP_561964.1| hypothetical protein CPE1048 [Clostridium perfringens str. 13]
>gi|18310035|ref|NP_561969.1| hypothetical protein CPE1053 [Clostridium perfringens str. 13]
>gi|18310042|ref|NP_561976.1| hypothetical protein CPE1060 [Clostridium perfringens str. 13]
>gi|18310060|ref|NP_561994.1| hypothetical protein CPE1078 [Clostridium perfringens str. 13]
>gi|18310082|ref|NP_562016.1| hypothetical protein CPE1100 [Clostridium perfringens str. 13]
>gi|18310093|ref|NP_562027.1| hypothetical protein CPE1111 [Clostridium perfringens str. 13]
>gi|18310096|ref|NP_562030.1| hypothetical protein CPE1114 [Clostridium perfringens str. 13]
>gi|18310101|ref|NP_562035.1| hypothetical protein CPE1119 [Clostridium perfringens str. 13]
>gi|18310123|ref|NP_562057.1| hypothetical protein CPE1141 [Clostridium perfringens str. 13]
>gi|18310142|ref|NP_562076.1| hypothetical protein CPE1160 [Clostridium perfringens str. 13]
>gi|18310144|ref|NP_562078.1| hypothetical protein CPE1162 [Clostridium perfringens str. 13]
>gi|18310211|ref|NP_562145.1| hypothetical protein CPE1229 [Clostridium perfringens str. 13]
>gi|18310212|ref|NP_562146.1| hypothetical protein CPE1230 [Clostridium perfringens str. 13]
>gi|18310235|ref|NP_562169.1| hypothetical protein CPE1253 [Clostridium perfringens str. 13]
>gi|18310240|ref|NP_562174.1| probable enterotoxin [Clostridium perfringens str. 13]
>gi|18310242|ref|NP_562176.1| hypothetical protein CPE1260 [Clostridium perfringens str. 13]
>gi|18310244|ref|NP_562178.1| hypothetical protein CPE1262 [Clostridium perfringens str. 13]
>gi|18310269|ref|NP_562203.1| hypothetical protein CPE1287 [Clostridium perfringens str. 13]
>gi|18310303|ref|NP_562237.1| serine O-acetyltransferase [Clostridium perfringens str. 13]
>gi|18310331|ref|NP_562265.1| hypothetical protein CPE1349 [Clostridium perfringens str. 13]
>gi|18310359|ref|NP_562293.1| hypothetical protein CPE1377 [Clostridium perfringens str. 13]
>gi|18310367|ref|NP_562301.1| hypothetical protein CPE1385 [Clostridium perfringens str. 13]
>gi|18310368|ref|NP_562302.1| hypothetical protein CPE1386 [Clostridium perfringens str. 13]
>gi|18310369|ref|NP_562303.1| hypothetical protein CPE1387 [Clostridium perfringens str. 13]
>gi|18310370|ref|NP_562304.1| hypothetical protein CPE1388 [Clostridium perfringens str. 13]
>gi|18310376|ref|NP_562310.1| trigger factor [Clostridium perfringens str. 13]
>gi|18310379|ref|NP_562313.1| hypothetical protein CPE1397 [Clostridium perfringens str. 13]
>gi|18310446|ref|NP_562380.1| probable PTS system [Clostridium perfringens str. 13]
>gi|18310454|ref|NP_562388.1| hypothetical protein CPE1472 [Clostridium perfringens str. 13]
>gi|18310458|ref|NP_562392.1| hypothetical protein CPE1476 [Clostridium perfringens str. 13]
>gi|18310493|ref|NP_562427.1| hypothetical protein CPE1511 [Clostridium perfringens str. 13]
>gi|18310512|ref|NP_562446.1| hypothetical protein CPE1530 [Clostridium perfringens str. 13]
>gi|18310521|ref|NP_562455.1| hypothetical protein CPE1539 [Clostridium perfringens str. 13]
>gi|18310544|ref|NP_562478.1| hypothetical protein CPE1562 [Clostridium perfringens str. 13]
>gi|18310552|ref|NP_562486.1| probable phage shock protein [Clostridium perfringens str. 13]
>gi|18310553|ref|NP_562487.1| hypothetical protein CPE1571 [Clostridium perfringens str. 13]
>gi|18310557|ref|NP_562491.1| hypothetical protein CPE1575 [Clostridium perfringens str. 13]
>gi|18310567|ref|NP_562501.1| hypothetical protein CPE1585 [Clostridium perfringens str. 13]
>gi|18310585|ref|NP_562519.1| hypothetical protein CPE1603 [Clostridium perfringens str. 13]

>gi|18310591|ref|NP_562525.1| hypothetical protein CPE1609 [Clostridium perfringens str. 13]
>gi|18310594|ref|NP_562528.1| hypothetical protein CPE1612 [Clostridium perfringens str. 13]
>gi|18310596|ref|NP_562530.1| probable cell wall-binding protein [Clostridium perfringens str. 13]
>gi|18310613|ref|NP_562547.1| probable ribose ABC transporter [Clostridium perfringens str. 13]
>gi|18310630|ref|NP_562564.1| stage V sporulation protein B [Clostridium perfringens str. 13]
>gi|18310653|ref|NP_562587.1| stage V sporulation protein S [Clostridium perfringens str. 13]
>gi|18310698|ref|NP_562632.1| chromosome partition protein [Clostridium perfringens str. 13]
>gi|18310701|ref|NP_562635.1| acyl carrier protein [Clostridium perfringens str. 13]
>gi|18310759|ref|NP_562693.1| hypothetical protein CPE1777 [Clostridium perfringens str. 13]
>gi|18310763|ref|NP_562697.1| hypothetical protein CPE1781 [Clostridium perfringens str. 13]
>gi|18310803|ref|NP_562737.1| exodeoxyribonuclease VII small subunit [Clostridium perfringens str. 13]
>gi|18310810|ref|NP_562744.1| hypothetical protein CPE1828 [Clostridium perfringens str. 13]
>gi|18310816|ref|NP_562750.1| hypothetical protein CPE1834 [Clostridium perfringens str. 13]
>gi|18310835|ref|NP_562769.1| hypothetical protein CPE1853 [Clostridium perfringens str. 13]
>gi|18310846|ref|NP_562780.1| hypothetical protein CPE1864 [Clostridium perfringens str. 13]
>gi|18310866|ref|NP_562800.1| hypothetical protein CPE1884 [Clostridium perfringens str. 13]
>gi|18310875|ref|NP_562809.1| 50S ribosomal protein L35 [Clostridium perfringens str. 13]
>gi|18310891|ref|NP_562825.1| probable tetrahydrodipicolinate succinylase [Clostridium perfringens str. 13]
>gi|18310896|ref|NP_562830.1| probable PBP 5 synthesis repressor [Clostridium perfringens str. 13]
>gi|18310945|ref|NP_562879.1| hypothetical protein CPE1963 [Clostridium perfringens str. 13]
>gi|18310969|ref|NP_562903.1| two-component sensor histidine kinase [Clostridium perfringens str. 13]
>gi|18310975|ref|NP_562909.1| hypothetical protein CPE1993 [Clostridium perfringens str. 13]
>gi|18310995|ref|NP_562929.1| hypothetical protein CPE2013 [Clostridium perfringens str. 13]
>gi|18311003|ref|NP_562937.1| hypothetical protein CPE2021 [Clostridium perfringens str. 13]
>gi|18311005|ref|NP_562939.1| 30S ribosomal protein S2 [Clostridium perfringens str. 13]
>gi|18311014|ref|NP_562948.1| heat shock protein [Clostridium perfringens str. 13]
>gi|18311039|ref|NP_562973.1| hypothetical protein CPE2057 [Clostridium perfringens str. 13]
>gi|18311055|ref|NP_562989.1| hypothetical protein CPE2073 [Clostridium perfringens str. 13]
>gi|18311061|ref|NP_562995.1| hypothetical protein CPE2079 [Clostridium perfringens str. 13]
>gi|18311089|ref|NP_563023.1| hypothetical protein CPE2107 [Clostridium perfringens str. 13]
>gi|18311090|ref|NP_563024.1| hypothetical protein CPE2108 [Clostridium perfringens str. 13]
>gi|18311102|ref|NP_563036.1| ComE operon protein [Clostridium perfringens str. 13]
>gi|18311112|ref|NP_563046.1| 50S ribosomal protein L21 [Clostridium perfringens str. 13]
>gi|18311133|ref|NP_563067.1| probable mercuric ion-binding protein [Clostridium perfringens str. 13]
>gi|18311141|ref|NP_563075.1| hypothetical protein CPE2159 [Clostridium perfringens str. 13]
>gi|18311144|ref|NP_563078.1| 2`,3`-cyclic-nucleotide 2`-phosphodiesterase [Clostridium perfringens str. 13]
>gi|18311160|ref|NP_563094.1| hypothetical protein CPE2178 [Clostridium perfringens str. 13]
>gi|18311173|ref|NP_563107.1| ATP synthase B chain [Clostridium perfringens str. 13]
>gi|18311174|ref|NP_563108.1| ATP synthase C chain [Clostridium perfringens str. 13]
>gi|18311206|ref|NP_563140.1| hypothetical protein CPE2224 [Clostridium perfringens str. 13]
>gi|18311232|ref|NP_563166.1| hypothetical protein CPE2250 [Clostridium perfringens str. 13]
>gi|18311238|ref|NP_563172.1| probable oligopeptide-binding protein [Clostridium perfringens str. 13]
>gi|18311239|ref|NP_563173.1| hypothetical protein CPE2257 [Clostridium perfringens str. 13]
>gi|18311243|ref|NP_563177.1| hypothetical protein CPE2261 [Clostridium perfringens str. 13]
>gi|18311269|ref|NP_563203.1| probable prepilin peptidase [Clostridium perfringens str. 13]
>gi|18311273|ref|NP_563207.1| hypothetical protein CPE2291 [Clostridium perfringens str. 13]
>gi|18311293|ref|NP_563227.1| hypothetical protein CPE2311 [Clostridium perfringens str. 13]
>gi|18311297|ref|NP_563231.1| hypothetical protein CPE2315 [Clostridium perfringens str. 13]
>gi|18311299|ref|NP_563233.1| probable Na+/H+ antiporter [Clostridium perfringens str. 13]
>gi|18311348|ref|NP_563282.1| hypothetical protein CPE2366 [Clostridium perfringens str. 13]
>gi|18311376|ref|NP_563310.1| 50S ribosomal protein L24 [Clostridium perfringens str. 13]
>gi|18311396|ref|NP_563330.1| 50S ribosomal protein L7/L12 [Clostridium perfringens str. 13]
>gi|18311448|ref|NP_563382.1| transcriptional elongation factor [Clostridium perfringens str. 13]
>gi|18311498|ref|NP_563432.1| hypothetical protein CPE2516 [Clostridium perfringens str. 13]
>gi|18311516|ref|NP_563450.1| hypothetical protein CPE2534 [Clostridium perfringens str. 13]
>gi|18311518|ref|NP_563452.1| hypothetical protein CPE2536 [Clostridium perfringens str. 13]
>gi|18311524|ref|NP_563458.1| hypothetical protein CPE2542 [Clostridium perfringens str. 13]
>gi|18311569|ref|NP_563503.1| maltose transacetylase [Clostridium perfringens str. 13]
>gi|18311595|ref|NP_563529.1| probable chromosome replication initiation protein [Clostridium perfringens str. 13]
>gi|18311623|ref|NP_563557.1| probable phage-related single-strand DNA-binding protein [Clostridium perfringens str. 13]
>gi|18311630|ref|NP_563564.1| hypothetical protein CPE2648 [Clostridium perfringens str. 13]
>gi|27375122|ref|NP_766651.1| hypothetical protein bll0011 [Bradyrhizobium japonicum USDA 110]
>gi|27375124|ref|NP_766653.1| putative transposase [Bradyrhizobium japonicum USDA 110]
>gi|27375133|ref|NP_766662.1| hypothetical protein bsr0022 [Bradyrhizobium japonicum USDA 110]
>gi|27375153|ref|NP_766682.1| hypothetical protein bll0042 [Bradyrhizobium japonicum USDA 110]
>gi|27375172|ref|NP_766701.1| hypothetical protein bll0061 [Bradyrhizobium japonicum USDA 110]
>gi|27375193|ref|NP_766722.1| hypothetical protein bll0082 [Bradyrhizobium japonicum USDA 110]
>gi|27375207|ref|NP_766736.1| putative methyl accepting chemotaxis protein [Bradyrhizobium japonicum USDA 110]
>gi|27375216|ref|NP_766745.1| hypothetical protein blr0105 [Bradyrhizobium japonicum USDA 110]
>gi|27375238|ref|NP_766767.1| hypothetical protein bsr0127 [Bradyrhizobium japonicum USDA 110]

>gi|27375244|ref|NP_766773.1| hypothetical protein bll0133 [Bradyrhizobium japonicum USDA 110]
>gi|27375246|ref|NP_766775.1| hypothetical protein blr0135 [Bradyrhizobium japonicum USDA 110]
>gi|27375247|ref|NP_766776.1| hypothetical protein bsr0136 [Bradyrhizobium japonicum USDA 110]
>gi|27375257|ref|NP_766786.1| hypothetical protein blr0146 [Bradyrhizobium japonicum USDA 110]
>gi|27375259|ref|NP_766788.1| MFS permease [Bradyrhizobium japonicum USDA 110]
>gi|27375267|ref|NP_766796.1| probable transcriptional regulator [Bradyrhizobium japonicum USDA 110]
>gi|27375270|ref|NP_766799.1| ATP-dependent helicase [Bradyrhizobium japonicum USDA 110]
>gi|27375276|ref|NP_766805.1| hypothetical protein blr0165 [Bradyrhizobium japonicum USDA 110]
>gi|27375303|ref|NP_766832.1| hypothetical protein blr0192 [Bradyrhizobium japonicum USDA 110]
>gi|27375306|ref|NP_766835.1| ABC transporter permease protein [Bradyrhizobium japonicum USDA 110]
>gi|27375331|ref|NP_766860.1| hypothetical protein blr0220 [Bradyrhizobium japonicum USDA 110]
>gi|27375335|ref|NP_766864.1| hypothetical protein bll0224 [Bradyrhizobium japonicum USDA 110]
>gi|27375342|ref|NP_766871.1| hypothetical protein bsl0231 [Bradyrhizobium japonicum USDA 110]
>gi|27375348|ref|NP_766877.1| transcriptional regulatory protein [Bradyrhizobium japonicum USDA 110]
>gi|27375358|ref|NP_766887.1| hypothetical protein bsr0247 [Bradyrhizobium japonicum USDA 110]
>gi|27375362|ref|NP_766891.1| putative efflux protein [Bradyrhizobium japonicum USDA 110]
>gi|27375371|ref|NP_766900.1| hypothetical protein blr0260 [Bradyrhizobium japonicum USDA 110]
>gi|27375411|ref|NP_766940.1| RagD protein [Bradyrhizobium japonicum USDA 110]
>gi|27375417|ref|NP_766946.1| hypothetical protein blr0306 [Bradyrhizobium japonicum USDA 110]
>gi|27375429|ref|NP_766958.1| nitrous oxide metabolic protein [Bradyrhizobium japonicum USDA 110]
>gi|27375456|ref|NP_766985.1| hypothetical protein bsl0345 [Bradyrhizobium japonicum USDA 110]
>gi|27375463|ref|NP_766992.1| hypothetical protein blr0352 [Bradyrhizobium japonicum USDA 110]
>gi|27375473|ref|NP_767002.1| hypothetical protein bll0362 [Bradyrhizobium japonicum USDA 110]
>gi|27375474|ref|NP_767003.1| hypothetical protein bll0363 [Bradyrhizobium japonicum USDA 110]
>gi|27375476|ref|NP_767005.1| 30S ribosomal protein S21 [Bradyrhizobium japonicum USDA 110]
>gi|27375508|ref|NP_767037.1| two-component sensor histidine kinase [Bradyrhizobium japonicum USDA 110]
>gi|27375517|ref|NP_767046.1| hypothetical protein bll0406 [Bradyrhizobium japonicum USDA 110]
>gi|27375544|ref|NP_767073.1| hypothetical protein blr0433 [Bradyrhizobium japonicum USDA 110]
>gi|27375549|ref|NP_767078.1| hypothetical protein bll0438 [Bradyrhizobium japonicum USDA 110]
>gi|27375554|ref|NP_767083.1| ATP synthase delta chain [Bradyrhizobium japonicum USDA 110]
>gi|27375555|ref|NP_767084.1| hypothetical protein blr044 [Bradyrhizobium japonicum USDA 110]
>gi|27375562|ref|NP_767091.1| dihydrolipoamide S-succinyltransferase [Bradyrhizobium japonicum USDA 110]
>gi|27375579|ref|NP_767108.1| heme exporter protein B [Bradyrhizobium japonicum USDA 110]
>gi|27375589|ref|NP_767118.1| hypothetical protein blr0478 [Bradyrhizobium japonicum USDA 110]
>gi|27375600|ref|NP_767129.1| putative glicine-rich protein [Bradyrhizobium japonicum USDA 110]
>gi|27375602|ref|NP_767131.1| hypothetical protein bll0491 [Bradyrhizobium japonicum USDA 110]
>gi|27375603|ref|NP_767132.1| hypothetical protein blr0492 [Bradyrhizobium japonicum USDA 110]
>gi|27375615|ref|NP_767144.1| hypothetical protein blr0503 [Bradyrhizobium japonicum USDA 110]
>gi|27375631|ref|NP_767160.1| hypothetical protein bsr0520 [Bradyrhizobium japonicum USDA 110]
>gi|27375632|ref|NP_767161.1| hypothetical protein blr0521 [Bradyrhizobium japonicum USDA 110]
>gi|27375637|ref|NP_767166.1| hypothetical protein bll0526 [Bradyrhizobium japonicum USDA 110]
>gi|27375640|ref|NP_767169.1| hypothetical protein blr0529 [Bradyrhizobium japonicum USDA 110]
>gi|27375650|ref|NP_767179.1| hypothetical protein bsr0539 [Bradyrhizobium japonicum USDA 110]
>gi|27375661|ref|NP_767190.1| hypothetical protein bsl0550 [Bradyrhizobium japonicum USDA 110]
>gi|27375673|ref|NP_767202.1| putative polysaccharide deacetylase [Bradyrhizobium japonicum USDA 110]
>gi|27375674|ref|NP_767203.1| hypothetical protein bll0563 [Bradyrhizobium japonicum USDA 110]
>gi|27375675|ref|NP_767204.1| hypothetical protein bll0564 [Bradyrhizobium japonicum USDA 110]
>gi|27375676|ref|NP_767205.1| hypothetical protein bll0565 [Bradyrhizobium japonicum USDA 110]
>gi|27375681|ref|NP_767210.1| adenylate cyclase [Bradyrhizobium japonicum USDA 110]
>gi|27375687|ref|NP_767216.1| putative methyl-accepting chemotaxis protein [Bradyrhizobium japonicum USDA 110]
>gi|27375698|ref|NP_767227.1| putative transmembrane protein [Bradyrhizobium japonicum USDA 110]
>gi|27375712|ref|NP_767241.1| transcriptional regulatory protein [Bradyrhizobium japonicum USDA 110]
>gi|27375718|ref|NP_767247.1| similar to ammonium transporter [Bradyrhizobium japonicum USDA 110]
>gi|27375755|ref|NP_767284.1| hypothetical protein blr0644 [Bradyrhizobium japonicum USDA 110]
>gi|27375768|ref|NP_767297.1| hypothetical protein bll0657 [Bradyrhizobium japonicum USDA 110]
>gi|27375786|ref|NP_767315.1| heat-inducible transcription repressor [Bradyrhizobium japonicum USDA 110]
>gi|27375803|ref|NP_767332.1| hypothetical protein bll0692 [Bradyrhizobium japonicum USDA 110]
>gi|27375815|ref|NP_767344.1| hypothetical protein bll0704 [Bradyrhizobium japonicum USDA 110]
>gi|27375820|ref|NP_767349.1| hypothetical protein blr0709 [Bradyrhizobium japonicum USDA 110]
>gi|27375846|ref|NP_767375.1| organic hydroperoxide resistance protein [Bradyrhizobium japonicum USDA 110]
>gi|27375848|ref|NP_767377.1| hypothetical protein bll0737 [Bradyrhizobium japonicum USDA 110]
>gi|27375854|ref|NP_767383.1| hypothetical protein blr0743 [Bradyrhizobium japonicum USDA 110]
>gi|27375886|ref|NP_767415.1| hypothetical protein bll0775 [Bradyrhizobium japonicum USDA 110]
>gi|27375894|ref|NP_767423.1| translation initiation factor IF-2 [Bradyrhizobium japonicum USDA 110]
>gi|27375898|ref|NP_767427.1| hypothetical protein blr0787 [Bradyrhizobium japonicum USDA 110]
>gi|27375904|ref|NP_767433.1| hypothetical protein bll0793 [Bradyrhizobium japonicum USDA 110]
>gi|27375912|ref|NP_767441.1| hypothetical protein bll0801 [Bradyrhizobium japonicum USDA 110]
>gi|27375914|ref|NP_767443.1| putative virulence factor [Bradyrhizobium japonicum USDA 110]
>gi|27375919|ref|NP_767448.1| hypothetical protein bsl0808 [Bradyrhizobium japonicum USDA 110]
>gi|27375920|ref|NP_767449.1| hypothetical protein bll0809 [Bradyrhizobium japonicum USDA 110]

>gi|27375928|ref|NP_767457.1| hypothetical protein blr0817 [Bradyrhizobium japonicum USDA 110]
>gi|27375932|ref|NP_767461.1| multidrug efflux protein [Bradyrhizobium japonicum USDA 110]
>gi|27375947|ref|NP_767476.1| hypothetical protein bll0836 [Bradyrhizobium japonicum USDA 110]
>gi|27375970|ref|NP_767499.1| hypothetical protein bsr0859 [Bradyrhizobium japonicum USDA 110]
>gi|27375995|ref|NP_767524.1| ABC transporter permease protein [Bradyrhizobium japonicum USDA 110]
>gi|27376051|ref|NP_767580.1| putative Na(+)/H(+) exchanger [Bradyrhizobium japonicum USDA 110]
>gi|27376055|ref|NP_767584.1| hypothetical protein blr0944 [Bradyrhizobium japonicum USDA 110]
>gi|27376060|ref|NP_767589.1| hypothetical protein blr0949 [Bradyrhizobium japonicum USDA 110]
>gi|27376099|ref|NP_767628.1| hypothetical protein blr0988 [Bradyrhizobium japonicum USDA 110]
>gi|27376116|ref|NP_767645.1| hypothetical protein bll1005 [Bradyrhizobium japonicum USDA 110]
>gi|27376121|ref|NP_767650.1| Hypothetical protein between soxB and soxA [Chlorobium tepidum TLS] [Bradyrhizobium japonicum USDA 110]
>gi|27376138|ref|NP_767667.1| putative cytochrome C biogenesis protein [Bradyrhizobium japonicum USDA 110]
>gi|27376152|ref|NP_767681.1| glutamyl-tRNA(Gln) amidotransferase [Bradyrhizobium japonicum USDA 110]
>gi|27376175|ref|NP_767704.1| ABC transporter permease protein [Bradyrhizobium japonicum USDA 110]
>gi|27376179|ref|NP_767708.1| hypothetical protein bll1068 [Bradyrhizobium japonicum USDA 110]
>gi|27376184|ref|NP_767713.1| probable glyoxalase [Bradyrhizobium japonicum USDA 110]
>gi|27376220|ref|NP_767749.1| hypothetical protein bll1109 [Bradyrhizobium japonicum USDA 110]
>gi|27376243|ref|NP_767772.1| putative amino acid binding protein [Bradyrhizobium japonicum USDA 110]
>gi|27376266|ref|NP_767795.1| hypothetical protein bll1155 [Bradyrhizobium japonicum USDA 110]
>gi|27376296|ref|NP_767825.1| FoF1 ATP synthase B chain [Bradyrhizobium japonicum USDA 110]
>gi|27376298|ref|NP_767827.1| FoF1 ATP synthase C chain [Bradyrhizobium japonicum USDA 110]
>gi|27376300|ref|NP_767829.1| FoF1 ATP synthase subunit I [Bradyrhizobium japonicum USDA 110]
>gi|27376314|ref|NP_767843.1| probable methyl-accepting chemotaxis protein [Bradyrhizobium japonicum USDA 110]
>gi|27376317|ref|NP_767846.1| hypothetical protein blr1206 [Bradyrhizobium japonicum USDA 110]
>gi|27376324|ref|NP_767853.1| transcriptional regulatory protein [Bradyrhizobium japonicum USDA 110]
>gi|27376343|ref|NP_767872.1| hypothetical protein bsr1232 [Bradyrhizobium japonicum USDA 110]
>gi|27376361|ref|NP_767890.1| hypothetical protein blr1250 [Bradyrhizobium japonicum USDA 110]
>gi|27376363|ref|NP_767892.1| transcriptional regulatory protein [Bradyrhizobium japonicum USDA 110]
>gi|27376384|ref|NP_767913.1| probable malonate decarboxylase [Bradyrhizobium japonicum USDA 110]
>gi|27376385|ref|NP_767914.1| malonate decarboxylase gamma subunit [Bradyrhizobium japonicum USDA 110]
>gi|27376388|ref|NP_767917.1| malonate carrier protein [Bradyrhizobium japonicum USDA 110]
>gi|27376392|ref|NP_767921.1| hypothetical protein bll1281 [Bradyrhizobium japonicum USDA 110]
>gi|27376396|ref|NP_767925.1| hypothetical protein bll1285 [Bradyrhizobium japonicum USDA 110]
>gi|27376421|ref|NP_767950.1| hypothetical protein bsr1310 [Bradyrhizobium japonicum USDA 110]
>gi|27376431|ref|NP_767960.1| probable penicillin-binding protein [Bradyrhizobium japonicum USDA 110]
>gi|27376436|ref|NP_767965.1| hypothetical protein blr1325 [Bradyrhizobium japonicum USDA 110]
>gi|27376443|ref|NP_767972.1| hypothetical protein blr1332 [Bradyrhizobium japonicum USDA 110]
>gi|27376451|ref|NP_767980.1| hypothetical protein blr1340 [Bradyrhizobium japonicum USDA 110]
>gi|27376452|ref|NP_767981.1| hypothetical protein bll1341 [Bradyrhizobium japonicum USDA 110]
>gi|27376453|ref|NP_767982.1| hypothetical protein bll1342 [Bradyrhizobium japonicum USDA 110]
>gi|27376454|ref|NP_767983.1| hypothetical protein blr1343 [Bradyrhizobium japonicum USDA 110]
>gi|27376458|ref|NP_767987.1| hypothetical protein blr1347 [Bradyrhizobium japonicum USDA 110]
>gi|27376459|ref|NP_767988.1| hypothetical protein blr1348 [Bradyrhizobium japonicum USDA 110]
>gi|27376471|ref|NP_768000.1| hypothetical protein blr1360 [Bradyrhizobium japonicum USDA 110]
>gi|27376489|ref|NP_768018.1| electron transfer flavoprotein large subunit [Bradyrhizobium japonicum USDA 110]
>gi|27376493|ref|NP_768022.1| hypothetical protein bsr1382 [Bradyrhizobium japonicum USDA 110]
>gi|27376498|ref|NP_768027.1| hypothetical protein blr1387 [Bradyrhizobium japonicum USDA 110]
>gi|27376513|ref|NP_768042.1| hypothetical protein blr1402 [Bradyrhizobium japonicum USDA 110]
>gi|27376519|ref|NP_768048.1| hypothetical protein blr1408 [Bradyrhizobium japonicum USDA 110]
>gi|27376534|ref|NP_768063.1| cytochrome c [Bradyrhizobium japonicum USDA 110]
>gi|27376540|ref|NP_768069.1| hypothetical protein blr1429 [Bradyrhizobium japonicum USDA 110]
>gi|27376552|ref|NP_768081.1| pilus assembly protein prepilin peptidase subunit [Bradyrhizobium japonicum USDA 110]
>gi|27376558|ref|NP_768087.1| dead-box ATP-dependent RNA helicase [Bradyrhizobium japonicum USDA 110]
>gi|27376564|ref|NP_768093.1| urease accessory protein [Bradyrhizobium japonicum USDA 110]
>gi|27376576|ref|NP_768105.1| hypothetical protein bll1465 [Bradyrhizobium japonicum USDA 110]
>gi|27376583|ref|NP_768112.1| hypothetical protein bsr1472 [Bradyrhizobium japonicum USDA 110]
>gi|27376608|ref|NP_768137.1| hypothetical protein bll1497 [Bradyrhizobium japonicum USDA 110]
>gi|27376636|ref|NP_768165.1| hypothetical protein bsr1525 [Bradyrhizobium japonicum USDA 110]
>gi|27376638|ref|NP_768167.1| putative 5-formyltetrahydrofolate cyclo-ligase [Bradyrhizobium japonicum USDA 110]
>gi|27376643|ref|NP_768172.1| putative chemotaxis protein [Bradyrhizobium japonicum USDA 110]
>gi|27376727|ref|NP_768256.1| hypothetical protein blr1616 [Bradyrhizobium japonicum USDA 110]
>gi|27376728|ref|NP_768257.1| conjugal transfer protein [Bradyrhizobium japonicum USDA 110]
>gi|27376750|ref|NP_768279.1| hypothetical protein bsl1639 [Bradyrhizobium japonicum USDA 110]
>gi|27376760|ref|NP_768289.1| hypothetical protein blr1649 [Bradyrhizobium japonicum USDA 110]
>gi|27376787|ref|NP_768316.1| hypothetical protein blr1676 [Bradyrhizobium japonicum USDA 110]
>gi|27376808|ref|NP_768337.1| hypothetical protein bll1697 [Bradyrhizobium japonicum USDA 110]
>gi|27376816|ref|NP_768345.1| hypothetical protein blr1705 [Bradyrhizobium japonicum USDA 110]
>gi|27376820|ref|NP_768349.1| hypothetical protein blr1709 [Bradyrhizobium japonicum USDA 110]
>gi|27376830|ref|NP_768359.1| molybdenum transport system permease protein [Bradyrhizobium japonicum USDA 110]
>gi|27376887|ref|NP_768416.1| alkyl hydroperoxide reductase [Bradyrhizobium japonicum USDA 110]

>gi|27376912|ref|NP_768441.1| hypothetical protein bll1801 [Bradyrhizobium japonicum USDA 110]
>gi|27376915|ref|NP_768444.1| hypothetical protein bll1804 [Bradyrhizobium japonicum USDA 110]
>gi|27376921|ref|NP_768450.1| hypothetical protein bll1810 [Bradyrhizobium japonicum USDA 110]
>gi|27376925|ref|NP_768454.1| nodulation protein [Bradyrhizobium japonicum USDA 110]
>gi|27376926|ref|NP_768455.1| nodulation protein [Bradyrhizobium japonicum USDA 110]
>gi|27376928|ref|NP_768457.1| hypothetical protein blr1817 [Bradyrhizobium japonicum USDA 110]
>gi|27376931|ref|NP_768460.1| RhcS protein [Bradyrhizobium japonicum USDA 110]
>gi|27376937|ref|NP_768466.1| putative transposase [Bradyrhizobium japonicum USDA 110]
>gi|27376939|ref|NP_768468.1| hypothetical protein bsl1828 [Bradyrhizobium japonicum USDA 110]
>gi|27376941|ref|NP_768470.1| hypothetical protein blr1830 [Bradyrhizobium japonicum USDA 110]
>gi|27376971|ref|NP_768500.1| hypothetical protein bll1860 [Bradyrhizobium japonicum USDA 110]
>gi|27376974|ref|NP_768503.1| hypothetical protein bsr1863 [Bradyrhizobium japonicum USDA 110]
>gi|27377015|ref|NP_768544.1| hypothetical protein blr1904 [Bradyrhizobium japonicum USDA 110]
>gi|27377030|ref|NP_768559.1| exonuclease subunit SbcC homolog [Bradyrhizobium japonicum USDA 110]
>gi|27377049|ref|NP_768578.1| hypothetical protein bll1938 [Bradyrhizobium japonicum USDA 110]
>gi|27377056|ref|NP_768585.1| hypothetical protein blr1945 [Bradyrhizobium japonicum USDA 110]
>gi|27377078|ref|NP_768607.1| hypothetical protein bsl1967 [Bradyrhizobium japonicum USDA 110]
>gi|27377092|ref|NP_768621.1| hypothetical protein bll1981 [Bradyrhizobium japonicum USDA 110]
>gi|27377133|ref|NP_768662.1| hypothetical protein blr2022 [Bradyrhizobium japonicum USDA 110]
>gi|27377143|ref|NP_768672.1| NolM protein [Bradyrhizobium japonicum USDA 110]
>gi|27377201|ref|NP_768730.1| hypothetical protein blr2090 [Bradyrhizobium japonicum USDA 110]
>gi|27377233|ref|NP_768762.1| hypothetical protein blr2122 [Bradyrhizobium japonicum USDA 110]
>gi|27377243|ref|NP_768772.1| hypothetical protein blr2132 [Bradyrhizobium japonicum USDA 110]
>gi|27377257|ref|NP_768786.1| dehydrogenase [Bradyrhizobium japonicum USDA 110]
>gi|27377267|ref|NP_768796.1| hypothetical protein blr2156 [Bradyrhizobium japonicum USDA 110]
>gi|27377313|ref|NP_768842.1| hypothetical protein blr2202 [Bradyrhizobium japonicum USDA 110]
>gi|27377318|ref|NP_768847.1| flagellar biosynthesis protein [Bradyrhizobium japonicum USDA 110]
>gi|27377319|ref|NP_768848.1| hypothetical protein bll2208 [Bradyrhizobium japonicum USDA 110]
>gi|27377329|ref|NP_768858.1| putative xanthine dehydrogenase [Bradyrhizobium japonicum USDA 110]
>gi|27377339|ref|NP_768868.1| hypothetical protein bll2228 [Bradyrhizobium japonicum USDA 110]
>gi|27377342|ref|NP_768871.1| hypothetical protein blr2231 [Bradyrhizobium japonicum USDA 110]
>gi|27377353|ref|NP_768882.1| hypothetical protein blr2242 [Bradyrhizobium japonicum USDA 110]
>gi|27377357|ref|NP_768886.1| hypothetical protein blr2246 [Bradyrhizobium japonicum USDA 110]
>gi|27377359|ref|NP_768888.1| hypothetical protein blr2248 [Bradyrhizobium japonicum USDA 110]
>gi|27377382|ref|NP_768911.1| sugar ABC transporter permease protein [Bradyrhizobium japonicum USDA 110]
>gi|27377418|ref|NP_768947.1| hypothetical protein blr2307 [Bradyrhizobium japonicum USDA 110]
>gi|27377426|ref|NP_768955.1| hypothetical protein bsr2315 [Bradyrhizobium japonicum USDA 110]
>gi|27377456|ref|NP_768985.1| probable methyl-accepting chemotaxis protein [Bradyrhizobium japonicum USDA 110]
>gi|27377464|ref|NP_768993.1| hypothetical protein bll2353 [Bradyrhizobium japonicum USDA 110]
>gi|27377476|ref|NP_769005.1| hypothetical protein bll2365 [Bradyrhizobium japonicum USDA 110]
>gi|27377477|ref|NP_769006.1| hypothetical protein bll2366 [Bradyrhizobium japonicum USDA 110]
>gi|27377482|ref|NP_769011.1| serine acetyltransferase [Bradyrhizobium japonicum USDA 110]
>gi|27377486|ref|NP_769015.1| hypothetical protein bll2375 [Bradyrhizobium japonicum USDA 110]
>gi|27377517|ref|NP_769046.1| hypothetical protein bsl2406 [Bradyrhizobium japonicum USDA 110]
>gi|27377521|ref|NP_769050.1| hypothetical protein bll2410 [Bradyrhizobium japonicum USDA 110]
>gi|27377542|ref|NP_769071.1| hypothetical protein bll2431 [Bradyrhizobium japonicum USDA 110]
>gi|27377562|ref|NP_769091.1| hypothetical protein blr2451 [Bradyrhizobium japonicum USDA 110]
>gi|27377564|ref|NP_769093.1| hypothetical protein bll2453 [Bradyrhizobium japonicum USDA 110]
>gi|27377581|ref|NP_769110.1| hypothetical protein blr2470 [Bradyrhizobium japonicum USDA 110]
>gi|27377590|ref|NP_769119.1| hypothetical protein bsl2479 [Bradyrhizobium japonicum USDA 110]
>gi|27377604|ref|NP_769133.1| hypothetical protein bll2493 [Bradyrhizobium japonicum USDA 110]
>gi|27377625|ref|NP_769154.1| hypothetical protein bll2514 [Bradyrhizobium japonicum USDA 110]
>gi|27377633|ref|NP_769162.1| hypothetical protein blr2522 [Bradyrhizobium japonicum USDA 110]
>gi|27377634|ref|NP_769163.1| hypothetical protein bll2523 [Bradyrhizobium japonicum USDA 110]
>gi|27377658|ref|NP_769187.1| putative methyl accepting chemotaxis protein [Bradyrhizobium japonicum USDA 110]
>gi|27377659|ref|NP_769188.1| putative chemotaxis protein [Bradyrhizobium japonicum USDA 110]
>gi|27377660|ref|NP_769189.1| hypothetical protein bll2549 [Bradyrhizobium japonicum USDA 110]
>gi|27377669|ref|NP_769198.1| hypothetical protein blr2558 [Bradyrhizobium japonicum USDA 110]
>gi|27377689|ref|NP_769218.1| MFS permease [Bradyrhizobium japonicum USDA 110]
>gi|27377690|ref|NP_769219.1| hypothetical protein blr2579 [Bradyrhizobium japonicum USDA 110]
>gi|27377701|ref|NP_769230.1| hypothetical protein bll2590 [Bradyrhizobium japonicum USDA 110]
>gi|27377704|ref|NP_769233.1| hypothetical protein bsl2593 [Bradyrhizobium japonicum USDA 110]
>gi|27377707|ref|NP_769236.1| hypothetical protein bsl2596 [Bradyrhizobium japonicum USDA 110]
>gi|27377718|ref|NP_769247.1| hypothetical protein blr2607 [Bradyrhizobium japonicum USDA 110]
>gi|27377731|ref|NP_769260.1| hypothetical protein blr2620 [Bradyrhizobium japonicum USDA 110]
>gi|27377737|ref|NP_769266.1| chaperone protein [Bradyrhizobium japonicum USDA 110]
>gi|27377751|ref|NP_769280.1| major facilitator superfamily transporter [Bradyrhizobium japonicum USDA 110]
>gi|27377774|ref|NP_769303.1| hypothetical protein bll2663 [Bradyrhizobium japonicum USDA 110]
>gi|27377783|ref|NP_769312.1| hypothetical protein bsr2672 [Bradyrhizobium japonicum USDA 110]
>gi|27377787|ref|NP_769316.1| sugar ABC transporter permease protein [Bradyrhizobium japonicum USDA 110]

>gi|27377839|ref|NP_769368.1| hypothetical protein blr2728 [Bradyrhizobium japonicum USDA 110]
>gi|27377851|ref|NP_769380.1| hypothetical transport protein [Bradyrhizobium japonicum USDA 110]
>gi|27377872|ref|NP_769401.1| hypothetical protein blr2761 [Bradyrhizobium japonicum USDA 110]
>gi|27377960|ref|NP_769489.1| hypothetical protein bll2849 [Bradyrhizobium japonicum USDA 110]
>gi|27377977|ref|NP_769506.1| ABC transporter permease protein [Bradyrhizobium japonicum USDA 110]
>gi|27377998|ref|NP_769527.1| hypothetical protein blr2887 [Bradyrhizobium japonicum USDA 110]
>gi|27378034|ref|NP_769563.1| amino acid ABC transporter permease protein [Bradyrhizobium japonicum USDA 110]
>gi|27378039|ref|NP_769568.1| oxidoreductase [Bradyrhizobium japonicum USDA 110]
>gi|27378042|ref|NP_769571.1| putative methyl-accepting chemotaxis protein [Bradyrhizobium japonicum USDA 110]
>gi|27378044|ref|NP_769573.1| probable aerotaxis receptor [Bradyrhizobium japonicum USDA 110]
>gi|27378075|ref|NP_769604.1| hypothetical protein blr2964 [Bradyrhizobium japonicum USDA 110]
>gi|27378087|ref|NP_769616.1| putative methyl accepting chemotaxis protein [Bradyrhizobium japonicum USDA 110]
>gi|27378098|ref|NP_769627.1| hypothetical protein blr2987 [Bradyrhizobium japonicum USDA 110]
>gi|27378107|ref|NP_769636.1| ethanolamine ammonia-lyase light chain [Bradyrhizobium japonicum USDA 110]
>gi|27378111|ref|NP_769640.1| hypothetical protein bll3000 [Bradyrhizobium japonicum USDA 110]
>gi|27378120|ref|NP_769649.1| hypothetical protein bll3009 [Bradyrhizobium japonicum USDA 110]
>gi|27378148|ref|NP_769677.1| hypothetical protein bll3037 [Bradyrhizobium japonicum USDA 110]
>gi|27378150|ref|NP_769679.1| hypothetical protein blr3039 [Bradyrhizobium japonicum USDA 110]
>gi|27378156|ref|NP_769685.1| hypothetical protein blr3045 [Bradyrhizobium japonicum USDA 110]
>gi|27378172|ref|NP_769701.1| hypothetical protein bll3061 [Bradyrhizobium japonicum USDA 110]
>gi|27378178|ref|NP_769707.1| hypothetical protein blr3067 [Bradyrhizobium japonicum USDA 110]
>gi|27378183|ref|NP_769712.1| hypothetical protein blr3072 [Bradyrhizobium japonicum USDA 110]
>gi|27378193|ref|NP_769722.1| hypothetical protein bsr3082 [Bradyrhizobium japonicum USDA 110]
>gi|27378200|ref|NP_769729.1| hypothetical protein bll3089 [Bradyrhizobium japonicum USDA 110]
>gi|27378204|ref|NP_769733.1| hypothetical protein blr3093 [Bradyrhizobium japonicum USDA 110]
>gi|27378219|ref|NP_769748.1| hypothetical protein bll3108 [Bradyrhizobium japonicum USDA 110]
>gi|27378220|ref|NP_769749.1| putative Ca binding protein [Bradyrhizobium japonicum USDA 110]
>gi|27378221|ref|NP_769750.1| hypothetical protein blr3110 [Bradyrhizobium japonicum USDA 110]
>gi|27378225|ref|NP_769754.1| hypothetical protein blr3114 [Bradyrhizobium japonicum USDA 110]
>gi|27378239|ref|NP_769768.1| cytochrome C-type biogenesis protein [Bradyrhizobium japonicum USDA 110]
>gi|27378260|ref|NP_769789.1| putative Oxalate:formate antiporter [Bradyrhizobium japonicum USDA 110]
>gi|27378273|ref|NP_769802.1| hypothetical protein blr3162 [Bradyrhizobium japonicum USDA 110]
>gi|27378280|ref|NP_769809.1| hypothetical protein blr3169 [Bradyrhizobium japonicum USDA 110]
>gi|27378285|ref|NP_769814.1| flagellar basal-body rod protein [Bradyrhizobium japonicum USDA 110]
>gi|27378295|ref|NP_769824.1| ABC transporter permease protein [Bradyrhizobium japonicum USDA 110]
>gi|27378300|ref|NP_769829.1| hypothetical protein blr3189 [Bradyrhizobium japonicum USDA 110]
>gi|27378323|ref|NP_769852.1| nitric oxide reductase subunit E [Bradyrhizobium japonicum USDA 110]
>gi|27378324|ref|NP_769853.1| hypothetical protein bsr3213 [Bradyrhizobium japonicum USDA 110]
>gi|27378362|ref|NP_769891.1| hypothetical protein blr3251 [Bradyrhizobium japonicum USDA 110]
>gi|27378363|ref|NP_769892.1| hypothetical protein blr3252 [Bradyrhizobium japonicum USDA 110]
>gi|27378366|ref|NP_769895.1| cobalamin (5'-phosphate) synthase [Bradyrhizobium japonicum USDA 110]
>gi|27378368|ref|NP_769897.1| cobalamin biosynthesis protein [Bradyrhizobium japonicum USDA 110]
>gi|27378375|ref|NP_769904.1| hypothetical protein blr3264 [Bradyrhizobium japonicum USDA 110]
>gi|27378381|ref|NP_769910.1| precorrin methylase [Bradyrhizobium japonicum USDA 110]
>gi|27378392|ref|NP_769921.1| putative glutamyl-tRNA(Gln) amidotransferase subunit (EC 6.3.5.-) [Bradyrhizobium japonicum USDA 110]
>gi|27378393|ref|NP_769922.1| hypothetical protein bll3282 [Bradyrhizobium japonicum USDA 110]
>gi|27378399|ref|NP_769928.1| putative glutamyl-tRNA(Gln) amidotransferase subunit (EC 6.3.5.-) [Bradyrhizobium japonicum USDA 110]
>gi|27378426|ref|NP_769955.1| ABC transporter permease protein [Bradyrhizobium japonicum USDA 110]
>gi|27378483|ref|NP_770012.1| hypothetical protein bll3372 [Bradyrhizobium japonicum USDA 110]
>gi|27378484|ref|NP_770013.1| hypothetical protein bll3373 [Bradyrhizobium japonicum USDA 110]
>gi|27378491|ref|NP_770020.1| hypothetical protein bll3380 [Bradyrhizobium japonicum USDA 110]
>gi|27378520|ref|NP_770049.1| transcriptional regulatory protein [Bradyrhizobium japonicum USDA 110]
>gi|27378536|ref|NP_770065.1| ABC transporter permease protein [Bradyrhizobium japonicum USDA 110]
>gi|27378548|ref|NP_770077.1| putative acyl-CoA dehydrogenase (EC 1.3.99.2) [Bradyrhizobium japonicum USDA 110]
>gi|27378561|ref|NP_770090.1| hypothetical protein blr3450 [Bradyrhizobium japonicum USDA 110]
>gi|27378566|ref|NP_770095.1| hypothetical protein blr3455 [Bradyrhizobium japonicum USDA 110]
>gi|27378582|ref|NP_770111.1| hypothetical membrane protein [Bradyrhizobium japonicum USDA 110]
>gi|27378584|ref|NP_770113.1| probable sulfate transporter [Bradyrhizobium japonicum USDA 110]
>gi|27378589|ref|NP_770118.1| hypothetical protein blr3478 [Bradyrhizobium japonicum USDA 110]
>gi|27378590|ref|NP_770119.1| hypothetical protein blr3479 [Bradyrhizobium japonicum USDA 110]
>gi|27378595|ref|NP_770124.1| hypothetical protein bsr3484 [Bradyrhizobium japonicum USDA 110]
>gi|27378597|ref|NP_770126.1| hypothetical protein blr3486 [Bradyrhizobium japonicum USDA 110]
>gi|27378602|ref|NP_770131.1| hypothetical protein blr3491 [Bradyrhizobium japonicum USDA 110]
>gi|27378620|ref|NP_770149.1| hypothetical protein bll3509 [Bradyrhizobium japonicum USDA 110]
>gi|27378664|ref|NP_770193.1| hypothetical protein blr3553 [Bradyrhizobium japonicum USDA 110]
>gi|27378674|ref|NP_770203.1| hypothetical protein bll3563 [Bradyrhizobium japonicum USDA 110]
>gi|27378711|ref|NP_770240.1| hypothetical protein blr3600 [Bradyrhizobium japonicum USDA 110]
>gi|27378750|ref|NP_770279.1| putative Biotin carboxyl carrier protein of acetyl-CoA carboxylase [Bradyrhizobium japonicum USDA 110]
>gi|27378783|ref|NP_770312.1| oxidoreductase [Bradyrhizobium japonicum USDA 110]
>gi|27378806|ref|NP_770335.1| probable Flagellin [Bradyrhizobium japonicum USDA 110]

>gi|27378807|ref|NP_770336.1| hypothetical protein blr3696 [Bradyrhizobium japonicum USDA 110]
>gi|27378809|ref|NP_770338.1| hypothetical protein blr3698 [Bradyrhizobium japonicum USDA 110]
>gi|27378810|ref|NP_770339.1| hypothetical protein blr3699 [Bradyrhizobium japonicum USDA 110]
>gi|27378811|ref|NP_770340.1| probable flagellar hook protein [Bradyrhizobium japonicum USDA 110]
>gi|27378812|ref|NP_770341.1| hypothetical protein bsr3701 [Bradyrhizobium japonicum USDA 110]
>gi|27378814|ref|NP_770343.1| probable flagellar hook-associated protein [Bradyrhizobium japonicum USDA 110]
>gi|27378819|ref|NP_770348.1| efflux protein [Bradyrhizobium japonicum USDA 110]
>gi|27378829|ref|NP_770358.1| hypothetical protein bsr3718 [Bradyrhizobium japonicum USDA 110]
>gi|27378832|ref|NP_770361.1| dihydrolipoamide S-acetyltransferase [Bradyrhizobium japonicum USDA 110]
>gi|27378864|ref|NP_770393.1| hypothetical protein bll3753 [Bradyrhizobium japonicum USDA 110]
>gi|27378867|ref|NP_770396.1| hypothetical protein blr3756 [Bradyrhizobium japonicum USDA 110]
>gi|27378873|ref|NP_770402.1| hypothetical protein blr3762 [Bradyrhizobium japonicum USDA 110]
>gi|27378879|ref|NP_770408.1| hypothetical protein bll3768 [Bradyrhizobium japonicum USDA 110]
>gi|27378881|ref|NP_770410.1| hypothetical protein blr3770 [Bradyrhizobium japonicum USDA 110]
>gi|27378892|ref|NP_770421.1| hypothetical transmembrane protein [Bradyrhizobium japonicum USDA 110]
>gi|27378898|ref|NP_770427.1| hypothetical protein blr3787 [Bradyrhizobium japonicum USDA 110]
>gi|27378899|ref|NP_770428.1| hypothetical protein bll3788 [Bradyrhizobium japonicum USDA 110]
>gi|27378924|ref|NP_770453.1| hypothetical protein bsl3813 [Bradyrhizobium japonicum USDA 110]
>gi|27378969|ref|NP_770498.1| putative shikimate 5-dehydrogenase (EC 1.1.1.25) [Bradyrhizobium japonicum USDA 110]
>gi|27378990|ref|NP_770519.1| major facilitator superfamily transporter [Bradyrhizobium japonicum USDA 110]
>gi|27379008|ref|NP_770537.1| hypothetical protein bsr3897 [Bradyrhizobium japonicum USDA 110]
>gi|27379012|ref|NP_770541.1| hypothetical protein bll3901 [Bradyrhizobium japonicum USDA 110]
>gi|27379017|ref|NP_770546.1| biopolymer transport protein [Bradyrhizobium japonicum USDA 110]
>gi|27379021|ref|NP_770550.1| hypothetical protein bll3910 [Bradyrhizobium japonicum USDA 110]
>gi|27379025|ref|NP_770554.1| oxidoreductase [Bradyrhizobium japonicum USDA 110]
>gi|27379036|ref|NP_770565.1| hypothetical protein bsr3925 [Bradyrhizobium japonicum USDA 110]
>gi|27379073|ref|NP_770602.1| MFS permease [Bradyrhizobium japonicum USDA 110]
>gi|27379092|ref|NP_770621.1| D-serine deaminase [Bradyrhizobium japonicum USDA 110]
>gi|27379094|ref|NP_770623.1| hypothetical protein blr3983 [Bradyrhizobium japonicum USDA 110]
>gi|27379131|ref|NP_770660.1| short chain dehydrogenase [Bradyrhizobium japonicum USDA 110]
>gi|27379138|ref|NP_770667.1| hypothetical protein bsr4027 [Bradyrhizobium japonicum USDA 110]
>gi|27379139|ref|NP_770668.1| putative RNA polymerase [Bradyrhizobium japonicum USDA 110]
>gi|27379161|ref|NP_770690.1| hypothetical protein blr4050 [Bradyrhizobium japonicum USDA 110]
>gi|27379162|ref|NP_770691.1| hypothetical protein blr4051 [Bradyrhizobium japonicum USDA 110]
>gi|27379166|ref|NP_770695.1| hypothetical protein blr4055 [Bradyrhizobium japonicum USDA 110]
>gi|27379167|ref|NP_770696.1| hypothetical protein blr4056 [Bradyrhizobium japonicum USDA 110]
>gi|27379177|ref|NP_770706.1| hypothetical protein blr4066 [Bradyrhizobium japonicum USDA 110]
>gi|27379184|ref|NP_770713.1| hypothetical protein blr4073 [Bradyrhizobium japonicum USDA 110]
>gi|27379188|ref|NP_770717.1| hypothetical protein bll4077 [Bradyrhizobium japonicum USDA 110]
>gi|27379190|ref|NP_770719.1| 30S ribosomal protein S6 [Bradyrhizobium japonicum USDA 110]
>gi|27379193|ref|NP_770722.1| malonyl-CoA:acyl carrier protein transacylase [Bradyrhizobium japonicum USDA 110]
>gi|27379200|ref|NP_770729.1| hypothetical protein bll4089 [Bradyrhizobium japonicum USDA 110]
>gi|27379201|ref|NP_770730.1| hypothetical protein bll4090 [Bradyrhizobium japonicum USDA 110]
>gi|27379204|ref|NP_770733.1| hypothetical protein blr4093 [Bradyrhizobium japonicum USDA 110]
>gi|27379211|ref|NP_770740.1| hypothetical protein blr4100 [Bradyrhizobium japonicum USDA 110]
>gi|27379224|ref|NP_770753.1| hypothetical protein blr4113 [Bradyrhizobium japonicum USDA 110]
>gi|27379231|ref|NP_770760.1| hypothetical protein blr4120 [Bradyrhizobium japonicum USDA 110]
>gi|27379240|ref|NP_770769.1| hypothetical protein blr4129 [Bradyrhizobium japonicum USDA 110]
>gi|27379251|ref|NP_770780.1| probable ABC transporter permease protein [Bradyrhizobium japonicum USDA 110]
>gi|27379259|ref|NP_770788.1| putative oligosaccharide deacetylase [Bradyrhizobium japonicum USDA 110]
>gi|27379261|ref|NP_770790.1| hypothetical protein bll4150 [Bradyrhizobium japonicum USDA 110]
>gi|27379283|ref|NP_770812.1| hypothetical protein blr4172 [Bradyrhizobium japonicum USDA 110]
>gi|27379288|ref|NP_770817.1| hypothetical protein bll4177 [Bradyrhizobium japonicum USDA 110]
>gi|27379326|ref|NP_770855.1| hypothetical protein blr4215 [Bradyrhizobium japonicum USDA 110]
>gi|27379329|ref|NP_770858.1| hypothetical protein bll4218 [Bradyrhizobium japonicum USDA 110]
>gi|27379334|ref|NP_770863.1| hypothetical protein blr4223 [Bradyrhizobium japonicum USDA 110]
>gi|27379354|ref|NP_770883.1| hypothetical protein bll4243 [Bradyrhizobium japonicum USDA 110]
>gi|27379358|ref|NP_770887.1| hypothetical protein bll4247 [Bradyrhizobium japonicum USDA 110]
>gi|27379359|ref|NP_770888.1| hypothetical protein bsr4248 [Bradyrhizobium japonicum USDA 110]
>gi|27379389|ref|NP_770918.1| hypothetical protein bll4278 [Bradyrhizobium japonicum USDA 110]
>gi|27379394|ref|NP_770923.1| hypothetical protein bll4283 [Bradyrhizobium japonicum USDA 110]
>gi|27379396|ref|NP_770925.1| hypothetical protein blr4285 [Bradyrhizobium japonicum USDA 110]
>gi|27379402|ref|NP_770931.1| biotin carboxyl carrier protein subunit of acetyl-CoA carboxylasen [Bradyrhizobium japonicum USDA 110]
>gi|27379404|ref|NP_770933.1| hypothetical protein bll4293 [Bradyrhizobium japonicum USDA 110]
>gi|27379416|ref|NP_770945.1| ribonuclease E [Bradyrhizobium japonicum USDA 110]
>gi|27379417|ref|NP_770946.1| putative N-acetylmuramoyl-L-alanine amidase precursor (EC 3.5.1.28) [Bradyrhizobium japonicum USDA 110]
>gi|27379429|ref|NP_770958.1| hypothetical protein blr4318 [Bradyrhizobium japonicum USDA 110]
>gi|27379435|ref|NP_770964.1| probable bacterioferritin [Bradyrhizobium japonicum USDA 110]
>gi|27379455|ref|NP_770984.1| hypothetical protein blr4344 [Bradyrhizobium japonicum USDA 110]
>gi|27379456|ref|NP_770985.1| hypothetical protein blr4345 [Bradyrhizobium japonicum USDA 110]

>gi|27379459|ref|NP_770988.1| ATP-dependent RNA helicase [Bradyrhizobium japonicum USDA 110]
>gi|27379467|ref|NP_770996.1| hypothetical protein blr4356 [Bradyrhizobium japonicum USDA 110]
>gi|27379483|ref|NP_771012.1| hypothetical protein blr4372 [Bradyrhizobium japonicum USDA 110]
>gi|27379503|ref|NP_771032.1| hypothetical protein blr4392 [Bradyrhizobium japonicum USDA 110]
>gi|27379507|ref|NP_771036.1| hypothetical protein bsr4396 [Bradyrhizobium japonicum USDA 110]
>gi|27379516|ref|NP_771045.1| hypothetical protein bsl4405 [Bradyrhizobium japonicum USDA 110]
>gi|27379519|ref|NP_771048.1| hypothetical protein bsr4408 [Bradyrhizobium japonicum USDA 110]
>gi|27379540|ref|NP_771069.1| hypothetical protein bll4429 [Bradyrhizobium japonicum USDA 110]
>gi|27379542|ref|NP_771071.1| hypothetical protein bsr4431 [Bradyrhizobium japonicum USDA 110]
>gi|27379543|ref|NP_771072.1| hypothetical protein bll4432 [Bradyrhizobium japonicum USDA 110]
>gi|27379546|ref|NP_771075.1| hypothetical protein blr4435 [Bradyrhizobium japonicum USDA 110]
>gi|27379552|ref|NP_771081.1| hypothetical protein blr4441 [Bradyrhizobium japonicum USDA 110]
>gi|27379581|ref|NP_771110.1| hypothetical protein blr4470 [Bradyrhizobium japonicum USDA 110]
>gi|27379585|ref|NP_771114.1| hypothetical protein blr4474 [Bradyrhizobium japonicum USDA 110]
>gi|27379595|ref|NP_771124.1| hypothetical protein bll4484 [Bradyrhizobium japonicum USDA 110]
>gi|27379608|ref|NP_771137.1| nitrile hydratase subunit beta [Bradyrhizobium japonicum USDA 110]
>gi|27379613|ref|NP_771142.1| probable ABC transporter permease protein [Bradyrhizobium japonicum USDA 110]
>gi|27379627|ref|NP_771156.1| methionyl-tRNA synthetase [Bradyrhizobium japonicum USDA 110]
>gi|27379638|ref|NP_771167.1| hypothetical protein bsl4527 [Bradyrhizobium japonicum USDA 110]
>gi|27379646|ref|NP_771175.1| hypothetical protein bll4535 [Bradyrhizobium japonicum USDA 110]
>gi|27379663|ref|NP_771192.1| hypothetical protein bll4552 [Bradyrhizobium japonicum USDA 110]
>gi|27379673|ref|NP_771202.1| hypothetical protein blr4562 [Bradyrhizobium japonicum USDA 110]
>gi|27379674|ref|NP_771203.1| hypothetical protein blr4563 [Bradyrhizobium japonicum USDA 110]
>gi|27379691|ref|NP_771220.1| hypothetical protein blr4580 [Bradyrhizobium japonicum USDA 110]
>gi|27379700|ref|NP_771229.1| hypothetical protein bll4589 [Bradyrhizobium japonicum USDA 110]
>gi|27379720|ref|NP_771249.1| hypothetical protein blr4609 [Bradyrhizobium japonicum USDA 110]
>gi|27379722|ref|NP_771251.1| hypothetical protein blr4611 [Bradyrhizobium japonicum USDA 110]
>gi|27379724|ref|NP_771253.1| hypothetical protein blr4613 [Bradyrhizobium japonicum USDA 110]
>gi|27379736|ref|NP_771265.1| hypothetical protein bsl4625 [Bradyrhizobium japonicum USDA 110]
>gi|27379742|ref|NP_771271.1| hypothetical protein bsr4631 [Bradyrhizobium japonicum USDA 110]
>gi|27379745|ref|NP_771274.1| hypothetical protein bll4634 [Bradyrhizobium japonicum USDA 110]
>gi|27379775|ref|NP_771304.1| hypothetical protein bll4664 [Bradyrhizobium japonicum USDA 110]
>gi|27379781|ref|NP_771310.1| hypothetical protein bll4670 [Bradyrhizobium japonicum USDA 110]
>gi|27379782|ref|NP_771311.1| putative outer-membrane immunogenic protein precursor [Bradyrhizobium japonicum USDA 110]
>gi|27379786|ref|NP_771315.1| hypothetical protein blr4675 [Bradyrhizobium japonicum USDA 110]
>gi|27379809|ref|NP_771338.1| single-strand DNA binding protein [Bradyrhizobium japonicum USDA 110]
>gi|27379824|ref|NP_771353.1| hypothetical protein bll4713 [Bradyrhizobium japonicum USDA 110]
>gi|27379826|ref|NP_771355.1| putative outer-membrane immunogenic protein precursor [Bradyrhizobium japonicum USDA 110]
>gi|27379831|ref|NP_771360.1| hypothetical protein bll4720 [Bradyrhizobium japonicum USDA 110]
>gi|27379833|ref|NP_771362.1| hypothetical protein bll4722 [Bradyrhizobium japonicum USDA 110]
>gi|27379854|ref|NP_771383.1| putative lipoprotein [Bradyrhizobium japonicum USDA 110]
>gi|27379861|ref|NP_771390.1| putative sec-independent protein translocase protein [Bradyrhizobium japonicum USDA 110]
>gi|27379866|ref|NP_771395.1| hypothetical protein bll4755 [Bradyrhizobium japonicum USDA 110]
>gi|27379875|ref|NP_771404.1| hypothetical protein blr4764 [Bradyrhizobium japonicum USDA 110]
>gi|27379876|ref|NP_771405.1| hypothetical protein bll4765 [Bradyrhizobium japonicum USDA 110]
>gi|27379879|ref|NP_771408.1| hypothetical protein blr4768 [Bradyrhizobium japonicum USDA 110]
>gi|27379890|ref|NP_771419.1| dihydrolipoamide acetyltransferase [Bradyrhizobium japonicum USDA 110]
>gi|27379926|ref|NP_771455.1| hypothetical protein bll4815 [Bradyrhizobium japonicum USDA 110]
>gi|27379928|ref|NP_771457.1| hypothetical protein bll4817 [Bradyrhizobium japonicum USDA 110]
>gi|27379931|ref|NP_771460.1| hypothetical protein bll4820 [Bradyrhizobium japonicum USDA 110]
>gi|27379941|ref|NP_771470.1| hypothetical protein bll4830 [Bradyrhizobium japonicum USDA 110]
>gi|27379944|ref|NP_771473.1| hypothetical protein bll4833 [Bradyrhizobium japonicum USDA 110]
>gi|27379963|ref|NP_771492.1| UDP glucosamine N-acyltransferase [Bradyrhizobium japonicum USDA 110]
>gi|27379967|ref|NP_771496.1| phosphatidate cytidylyltransferase [Bradyrhizobium japonicum USDA 110]
>gi|27379973|ref|NP_771502.1| hypothetical protein bll4862 [Bradyrhizobium japonicum USDA 110]
>gi|27379989|ref|NP_771518.1| possible Copper export protein [Bradyrhizobium japonicum USDA 110]
>gi|27380002|ref|NP_771531.1| hypothetical protein blr4891 [Bradyrhizobium japonicum USDA 110]
>gi|27380091|ref|NP_771620.1| hypothetical protein bll4980 [Bradyrhizobium japonicum USDA 110]
>gi|27380105|ref|NP_771634.1| hypothetical protein blr4994 [Bradyrhizobium japonicum USDA 110]
>gi|27380112|ref|NP_771641.1| hypothetical protein bll5001 [Bradyrhizobium japonicum USDA 110]
>gi|27380121|ref|NP_771650.1| putative resolvase [Bradyrhizobium japonicum USDA 110]
>gi|27380129|ref|NP_771658.1| hypothetical protein bll5018 [Bradyrhizobium japonicum USDA 110]
>gi|27380138|ref|NP_771667.1| putative thiamine-monophosphate kinase (EC 2.7.4.16) [Bradyrhizobium japonicum USDA 110]
>gi|27380140|ref|NP_771669.1| riboflavin synthase beta chain [Bradyrhizobium japonicum USDA 110]
>gi|27380155|ref|NP_771684.1| manganese transport protein [Bradyrhizobium japonicum USDA 110]
>gi|27380156|ref|NP_771685.1| putative inner membrane protein [Bradyrhizobium japonicum USDA 110]
>gi|27380159|ref|NP_771688.1| hypothetical protein bll5048 [Bradyrhizobium japonicum USDA 110]
>gi|27380161|ref|NP_771690.1| cation efflux system protein [Bradyrhizobium japonicum USDA 110]
>gi|27380184|ref|NP_771713.1| putative soluble lytic transglycosylase precursor [Bradyrhizobium japonicum USDA 110]
>gi|27380196|ref|NP_771725.1| hypothetical protein bll5085 [Bradyrhizobium japonicum USDA 110]

>gi|27380197|ref|NP_771726.1| hypothetical protein bsl5086 [Bradyrhizobium japonicum USDA 110]
>gi|27380217|ref|NP_771746.1| acyl-carrier-protein phosphodiesterase [Bradyrhizobium japonicum USDA 110]
>gi|27380262|ref|NP_771791.1| hypothetical transport protein [Bradyrhizobium japonicum USDA 110]
>gi|27380267|ref|NP_771796.1| putative D-alanyl-D-alanine carboxypeptidase [Bradyrhizobium japonicum USDA 110]
>gi|27380268|ref|NP_771797.1| hypothetical protein bll5157 [Bradyrhizobium japonicum USDA 110]
>gi|27380275|ref|NP_771804.1| hypothetical protein bll5164 [Bradyrhizobium japonicum USDA 110]
>gi|27380277|ref|NP_771806.1| hypothetical protein bll5166 [Bradyrhizobium japonicum USDA 110]
>gi|27380286|ref|NP_771815.1| hypothetical protein bll5175 [Bradyrhizobium japonicum USDA 110]
>gi|27380312|ref|NP_771841.1| hypothetical protein bll5201 [Bradyrhizobium japonicum USDA 110]
>gi|27380316|ref|NP_771845.1| hypothetical protein bll5205 [Bradyrhizobium japonicum USDA 110]
>gi|27380329|ref|NP_771858.1| hypothetical protein bll5218 [Bradyrhizobium japonicum USDA 110]
>gi|27380348|ref|NP_771877.1| hypothetical protein bll5237 [Bradyrhizobium japonicum USDA 110]
>gi|27380349|ref|NP_771878.1| hypothetical protein bll5238 [Bradyrhizobium japonicum USDA 110]
>gi|27380359|ref|NP_771888.1| hypothetical protein bll5248 [Bradyrhizobium japonicum USDA 110]
>gi|27380371|ref|NP_771900.1| transcriptional regulatory protein [Bradyrhizobium japonicum USDA 110]
>gi|27380409|ref|NP_771938.1| hypothetical protein bll5298 [Bradyrhizobium japonicum USDA 110]
>gi|27380410|ref|NP_771939.1| hypothetical protein bll5299 [Bradyrhizobium japonicum USDA 110]
>gi|27380422|ref|NP_771951.1| probable histon H1 [Bradyrhizobium japonicum USDA 110]
>gi|27380424|ref|NP_771953.1| hypothetical protein bll5313 [Bradyrhizobium japonicum USDA 110]
>gi|27380431|ref|NP_771960.1| hypothetical protein bll5320 [Bradyrhizobium japonicum USDA 110]
>gi|27380452|ref|NP_771981.1| hypothetical protein blr5341 [Bradyrhizobium japonicum USDA 110]
>gi|27380458|ref|NP_771987.1| hypothetical protein bsr5347 [Bradyrhizobium japonicum USDA 110]
>gi|27380459|ref|NP_771988.1| hypothetical protein blr5348 [Bradyrhizobium japonicum USDA 110]
>gi|27380466|ref|NP_771995.1| hypothetical protein bll5355 [Bradyrhizobium japonicum USDA 110]
>gi|27380468|ref|NP_771997.1| hypothetical protein blr5357 [Bradyrhizobium japonicum USDA 110]
>gi|27380474|ref|NP_772003.1| hypothetical protein bll5363 [Bradyrhizobium japonicum USDA 110]
>gi|27380475|ref|NP_772004.1| hypothetical protein bll5364 [Bradyrhizobium japonicum USDA 110]
>gi|27380490|ref|NP_772019.1| probable adenylate kinase [Bradyrhizobium japonicum USDA 110]
>gi|27380492|ref|NP_772021.1| 50S ribosomal protein L15 [Bradyrhizobium japonicum USDA 110]
>gi|27380510|ref|NP_772039.1| 50S ribosomal protein L4 [Bradyrhizobium japonicum USDA 110]
>gi|27380522|ref|NP_772051.1| 50S ribosomal protein L7/L12 [Bradyrhizobium japonicum USDA 110]
>gi|27380550|ref|NP_772079.1| hypothetical protein blr5439 [Bradyrhizobium japonicum USDA 110]
>gi|27380557|ref|NP_772086.1| hypothetical protein blr5446 [Bradyrhizobium japonicum USDA 110]
>gi|27380558|ref|NP_772087.1| hypothetical protein blr5447 [Bradyrhizobium japonicum USDA 110]
>gi|27380562|ref|NP_772091.1| RhtB family transporter [Bradyrhizobium japonicum USDA 110]
>gi|27380611|ref|NP_772140.1| hypothetical protein bll5500 [Bradyrhizobium japonicum USDA 110]
>gi|27380618|ref|NP_772147.1| hypothetical protein bll5507 [Bradyrhizobium japonicum USDA 110]
>gi|27380622|ref|NP_772151.1| hypothetical protein bll5511 [Bradyrhizobium japonicum USDA 110]
>gi|27380630|ref|NP_772159.1| hypothetical protein bll5519 [Bradyrhizobium japonicum USDA 110]
>gi|27380640|ref|NP_772169.1| hypothetical metabolite transport protein [Bradyrhizobium japonicum USDA 110]
>gi|27380646|ref|NP_772175.1| hypothetical protein bll5535 [Bradyrhizobium japonicum USDA 110]
>gi|27380649|ref|NP_772178.1| hypothetical protein blr5538 [Bradyrhizobium japonicum USDA 110]
>gi|27380660|ref|NP_772189.1| hypothetical protein bll5549 [Bradyrhizobium japonicum USDA 110]
>gi|27380682|ref|NP_772211.1| hypothetical protein bsr5571 [Bradyrhizobium japonicum USDA 110]
>gi|27380690|ref|NP_772219.1| hypothetical protein bll5579 [Bradyrhizobium japonicum USDA 110]
>gi|27380693|ref|NP_772222.1| hypothetical protein bll5582 [Bradyrhizobium japonicum USDA 110]
>gi|27380703|ref|NP_772232.1| hypothetical protein bll5592 [Bradyrhizobium japonicum USDA 110]
>gi|27380710|ref|NP_772239.1| ABC transporter permease protein [Bradyrhizobium japonicum USDA 110]
>gi|27380733|ref|NP_772262.1| hypothetical protein bll5622 [Bradyrhizobium japonicum USDA 110]
>gi|27380742|ref|NP_772271.1| putative isochorismatase [Bradyrhizobium japonicum USDA 110]
>gi|27380746|ref|NP_772275.1| hypothetical protein bll5635 [Bradyrhizobium japonicum USDA 110]
>gi|27380762|ref|NP_772291.1| transcriptional regulatory protein [Bradyrhizobium japonicum USDA 110]
>gi|27380768|ref|NP_772297.1| hypothetical protein bll5657 [Bradyrhizobium japonicum USDA 110]
>gi|27380778|ref|NP_772307.1| 3-carboxy-cis,cis-muconate cycloisomerase [Bradyrhizobium japonicum USDA 110]
>gi|27380781|ref|NP_772310.1| hypothetical protein bsr5670 [Bradyrhizobium japonicum USDA 110]
>gi|27380790|ref|NP_772319.1| hypothetical protein bll5679 [Bradyrhizobium japonicum USDA 110]
>gi|27380798|ref|NP_772327.1| two-component sensor histidine kinase [Bradyrhizobium japonicum USDA 110]
>gi|27380806|ref|NP_772335.1| hypothetical protein bll5695 [Bradyrhizobium japonicum USDA 110]
>gi|27380807|ref|NP_772336.1| hypothetical protein bll5696 [Bradyrhizobium japonicum USDA 110]
>gi|27380841|ref|NP_772370.1| hypothetical protein blr5730 [Bradyrhizobium japonicum USDA 110]
>gi|27380898|ref|NP_772427.1| hypothetical protein bll5787 [Bradyrhizobium japonicum USDA 110]
>gi|27380900|ref|NP_772429.1| transcriptional regulatory protein [Bradyrhizobium japonicum USDA 110]
>gi|27380903|ref|NP_772432.1| hypothetical protein blr5792 [Bradyrhizobium japonicum USDA 110]
>gi|27380914|ref|NP_772443.1| ABC transporter permease protein [Bradyrhizobium japonicum USDA 110]
>gi|27380918|ref|NP_772447.1| hypothetical protein bll5807 [Bradyrhizobium japonicum USDA 110]
>gi|27380926|ref|NP_772455.1| hypothetical protein blr5815 [Bradyrhizobium japonicum USDA 110]
>gi|27380928|ref|NP_772457.1| hypothetical protein bll5817 [Bradyrhizobium japonicum USDA 110]
>gi|27380935|ref|NP_772464.1| hypothetical protein bll5824 [Bradyrhizobium japonicum USDA 110]
>gi|27380954|ref|NP_772483.1| hypothetical protein bll5843 [Bradyrhizobium japonicum USDA 110]
>gi|27380955|ref|NP_772484.1| hypothetical protein bll5844 [Bradyrhizobium japonicum USDA 110]

>gi|27380956|ref|NP_772485.1| hypothetical protein bll5845 [Bradyrhizobium japonicum USDA 110]
>gi|27380957|ref|NP_772486.1| hypothetical protein bll5846 [Bradyrhizobium japonicum USDA 110]
>gi|27380965|ref|NP_772494.1| hypothetical protein bll5854 [Bradyrhizobium japonicum USDA 110]
>gi|27380990|ref|NP_772519.1| hypothetical protein blr5879 [Bradyrhizobium japonicum USDA 110]
>gi|27380997|ref|NP_772526.1| two-component hybrid sensor and regulator [Bradyrhizobium japonicum USDA 110]
>gi|27380998|ref|NP_772527.1| hypothetical protein blr5887 [Bradyrhizobium japonicum USDA 110]
>gi|27381001|ref|NP_772530.1| monocarboxylic acid permease [Bradyrhizobium japonicum USDA 110]
>gi|27381002|ref|NP_772531.1| hypothetical protein bsl5891 [Bradyrhizobium japonicum USDA 110]
>gi|27381005|ref|NP_772534.1| ABC transporter permease protein [Bradyrhizobium japonicum USDA 110]
>gi|27381008|ref|NP_772537.1| hypothetical protein blr5897 [Bradyrhizobium japonicum USDA 110]
>gi|27381011|ref|NP_772540.1| hypothetical protein bll5900 [Bradyrhizobium japonicum USDA 110]
>gi|27381012|ref|NP_772541.1| hypothetical protein blr5901 [Bradyrhizobium japonicum USDA 110]
>gi|27381033|ref|NP_772562.1| probable glycosyl transferase [Bradyrhizobium japonicum USDA 110]
>gi|27381050|ref|NP_772579.1| hypothetical protein blr5939 [Bradyrhizobium japonicum USDA 110]
>gi|27381086|ref|NP_772615.1| putative oxidoreductase [Bradyrhizobium japonicum USDA 110]
>gi|27381116|ref|NP_772645.1| hypothetical protein blr6005 [Bradyrhizobium japonicum USDA 110]
>gi|27381123|ref|NP_772652.1| hypothetical protein bll6012 [Bradyrhizobium japonicum USDA 110]
>gi|27381124|ref|NP_772653.1| hypothetical protein blr6013 [Bradyrhizobium japonicum USDA 110]
>gi|27381134|ref|NP_772663.1| probable prepilin peptidase [Bradyrhizobium japonicum USDA 110]
>gi|27381166|ref|NP_772695.1| putative oxidoreductase [Bradyrhizobium japonicum USDA 110]
>gi|27381178|ref|NP_772707.1| hypothetical protein blr6067 [Bradyrhizobium japonicum USDA 110]
>gi|27381183|ref|NP_772712.1| hypothetical protein blr6072 [Bradyrhizobium japonicum USDA 110]
>gi|27381193|ref|NP_772722.1| putative oxidoreductase [Bradyrhizobium japonicum USDA 110]
>gi|27381215|ref|NP_772744.1| hypothetical protein bll6104 [Bradyrhizobium japonicum USDA 110]
>gi|27381226|ref|NP_772755.1| hypothetical protein bsr6115 [Bradyrhizobium japonicum USDA 110]
>gi|27381234|ref|NP_772763.1| hypothetical protein blr6123 [Bradyrhizobium japonicum USDA 110]
>gi|27381236|ref|NP_772765.1| hypothetical protein bll6125 [Bradyrhizobium japonicum USDA 110]
>gi|27381239|ref|NP_772768.1| cytochrome c552 [Bradyrhizobium japonicum USDA 110]
>gi|27381241|ref|NP_772770.1| hypothetical protein bsr6130 [Bradyrhizobium japonicum USDA 110]
>gi|27381250|ref|NP_772779.1| hypothetical protein blr6139 [Bradyrhizobium japonicum USDA 110]
>gi|27381260|ref|NP_772789.1| ABC transporter permease protein [Bradyrhizobium japonicum USDA 110]
>gi|27381277|ref|NP_772806.1| hypothetical protein bll6166 [Bradyrhizobium japonicum USDA 110]
>gi|27381279|ref|NP_772808.1| hypothetical protein bll6168 [Bradyrhizobium japonicum USDA 110]
>gi|27381293|ref|NP_772822.1| hypothetical protein blr6182 [Bradyrhizobium japonicum USDA 110]
>gi|27381312|ref|NP_772841.1| ABC transporter ATP-binding protein [Bradyrhizobium japonicum USDA 110]
>gi|27381313|ref|NP_772842.1| hypothetical protein blr6202 [Bradyrhizobium japonicum USDA 110]
>gi|27381317|ref|NP_772846.1| hypothetical protein bll6206 [Bradyrhizobium japonicum USDA 110]
>gi|27381322|ref|NP_772851.1| hypothetical protein blr6211 [Bradyrhizobium japonicum USDA 110]
>gi|27381339|ref|NP_772868.1| hypothetical protein blr6228 [Bradyrhizobium japonicum USDA 110]
>gi|27381345|ref|NP_772874.1| ABC transporter permease protein [Bradyrhizobium japonicum USDA 110]
>gi|27381364|ref|NP_772893.1| hypothetical protein blr6253 [Bradyrhizobium japonicum USDA 110]
>gi|27381405|ref|NP_772934.1| hypothetical protein bll6294 [Bradyrhizobium japonicum USDA 110]
>gi|27381481|ref|NP_773010.1| ABC transporter permease protein [Bradyrhizobium japonicum USDA 110]
>gi|27381515|ref|NP_773044.1| ABC transporter permease protein [Bradyrhizobium japonicum USDA 110]
>gi|27381521|ref|NP_773050.1| ABC transporter ATP-binding/permease protein [Bradyrhizobium japonicum USDA 110]
>gi|27381523|ref|NP_773052.1| putative nitrile hydratase regulator [Bradyrhizobium japonicum USDA 110]
>gi|27381535|ref|NP_773064.1| hypothetical protein bll6424 [Bradyrhizobium japonicum USDA 110]
>gi|27381554|ref|NP_773083.1| ABC transporter permease protein [Bradyrhizobium japonicum USDA 110]
>gi|27381568|ref|NP_773097.1| hypothetical protein bll6457 [Bradyrhizobium japonicum USDA 110]
>gi|27381601|ref|NP_773130.1| ABC transporter permease protein [Bradyrhizobium japonicum USDA 110]
>gi|27381625|ref|NP_773154.1| hypothetical protein bll6514 [Bradyrhizobium japonicum USDA 110]
>gi|27381627|ref|NP_773156.1| putative transmembrane protein [Bradyrhizobium japonicum USDA 110]
>gi|27381631|ref|NP_773160.1| hypothetical protein bsr6520 [Bradyrhizobium japonicum USDA 110]
>gi|27381638|ref|NP_773167.1| hypothetical protein bll6527 [Bradyrhizobium japonicum USDA 110]
>gi|27381665|ref|NP_773194.1| hypothetical protein blr6554 [Bradyrhizobium japonicum USDA 110]
>gi|27381666|ref|NP_773195.1| putative sulfate transporter [Bradyrhizobium japonicum USDA 110]
>gi|27381667|ref|NP_773196.1| hypothetical protein bll6556 [Bradyrhizobium japonicum USDA 110]
>gi|27381668|ref|NP_773197.1| hypothetical protein bll6557 [Bradyrhizobium japonicum USDA 110]
>gi|27381671|ref|NP_773200.1| hypothetical protein bsl6560 [Bradyrhizobium japonicum USDA 110]
>gi|27381673|ref|NP_773202.1| hypothetical protein blr6562 [Bradyrhizobium japonicum USDA 110]
>gi|27381691|ref|NP_773220.1| hypothetical protein bll6580 [Bradyrhizobium japonicum USDA 110]
>gi|27381707|ref|NP_773236.1| cell division protein [Bradyrhizobium japonicum USDA 110]
>gi|27381716|ref|NP_773245.1| phospho-N-acetylmuramoyl-pentapeptide-transferas e [Bradyrhizobium japonicum USDA 110]
>gi|27381726|ref|NP_773255.1| hypothetical protein bll6615 [Bradyrhizobium japonicum USDA 110]
>gi|27381728|ref|NP_773257.1| hypothetical protein bsl6617 [Bradyrhizobium japonicum USDA 110]
>gi|27381729|ref|NP_773258.1| hypothetical protein blr6618 [Bradyrhizobium japonicum USDA 110]
>gi|27381730|ref|NP_773259.1| hypothetical protein bll6619 [Bradyrhizobium japonicum USDA 110]
>gi|27381733|ref|NP_773262.1| multidrug resistance efflux pump [Bradyrhizobium japonicum USDA 110]
>gi|27381738|ref|NP_773267.1| hypothetical protein bll6627 [Bradyrhizobium japonicum USDA 110]
>gi|27381739|ref|NP_773268.1| hypothetical protein blr6628 [Bradyrhizobium japonicum USDA 110]

>gi|27381740|ref|NP_773269.1| hypothetical protein blr6629 [Bradyrhizobium japonicum USDA 110]
>gi|27381750|ref|NP_773279.1| hypothetical protein bsl6639 [Bradyrhizobium japonicum USDA 110]
>gi|27381752|ref|NP_773281.1| hypothetical protein blr6641 [Bradyrhizobium japonicum USDA 110]
>gi|27381758|ref|NP_773287.1| ABC transporter permease protein [Bradyrhizobium japonicum USDA 110]
>gi|27381760|ref|NP_773289.1| hypothetical protein bll6649 [Bradyrhizobium japonicum USDA 110]
>gi|27381761|ref|NP_773290.1| hypothetical protein bll6650 [Bradyrhizobium japonicum USDA 110]
>gi|27381777|ref|NP_773306.1| hypothetical protein bll6666 [Bradyrhizobium japonicum USDA 110]
>gi|27381785|ref|NP_773314.1| hypothetical protein bll6674 [Bradyrhizobium japonicum USDA 110]
>gi|27381793|ref|NP_773322.1| hypothetical protein blr6682 [Bradyrhizobium japonicum USDA 110]
>gi|27381794|ref|NP_773323.1| hypothetical protein bsr6683 [Bradyrhizobium japonicum USDA 110]
>gi|27381799|ref|NP_773328.1| hypothetical protein bll6688 [Bradyrhizobium japonicum USDA 110]
>gi|27381800|ref|NP_773329.1| hypothetical protein blr6689 [Bradyrhizobium japonicum USDA 110]
>gi|27381804|ref|NP_773333.1| hypothetical protein bsr6693 [Bradyrhizobium japonicum USDA 110]
>gi|27381883|ref|NP_773412.1| hypothetical protein blr6772 [Bradyrhizobium japonicum USDA 110]
>gi|27381892|ref|NP_773421.1| hypothetical protein blr6781 [Bradyrhizobium japonicum USDA 110]
>gi|27381910|ref|NP_773439.1| hypothetical protein bll6799 [Bradyrhizobium japonicum USDA 110]
>gi|27381919|ref|NP_773448.1| hypothetical protein bll6808 [Bradyrhizobium japonicum USDA 110]
>gi|27381955|ref|NP_773484.1| hypothetical protein bll6844 [Bradyrhizobium japonicum USDA 110]
>gi|27381960|ref|NP_773489.1| hypothetical protein bll6849 [Bradyrhizobium japonicum USDA 110]
>gi|27381971|ref|NP_773500.1| hypothetical protein bll6860 [Bradyrhizobium japonicum USDA 110]
>gi|27381974|ref|NP_773503.1| hypothetical protein bll6863 [Bradyrhizobium japonicum USDA 110]
>gi|27381976|ref|NP_773505.1| flagellin [Bradyrhizobium japonicum USDA 110]
>gi|27381977|ref|NP_773506.1| flagellin [Bradyrhizobium japonicum USDA 110]
>gi|27381981|ref|NP_773510.1| hypothetical protein bll6870 [Bradyrhizobium japonicum USDA 110]
>gi|27382004|ref|NP_773533.1| hypothetical protein bll6893 [Bradyrhizobium japonicum USDA 110]
>gi|27382015|ref|NP_773544.1| probable cation efflux system protein [Bradyrhizobium japonicum USDA 110]
>gi|27382021|ref|NP_773550.1| hydrogenase nickel incorporation protein [Bradyrhizobium japonicum USDA 110]
>gi|27382032|ref|NP_773561.1| putative multidrag resistance protein [Bradyrhizobium japonicum USDA 110]
>gi|27382042|ref|NP_773571.1| HypB protein [Bradyrhizobium japonicum USDA 110]
>gi|27382057|ref|NP_773586.1| putative methyl-accepting chemotaxis protein [Bradyrhizobium japonicum USDA 110]
>gi|27382069|ref|NP_773598.1| hypothetical protein bsl6958 [Bradyrhizobium japonicum USDA 110]
>gi|27382079|ref|NP_773608.1| hypothetical protein blr6968 [Bradyrhizobium japonicum USDA 110]
>gi|27382093|ref|NP_773622.1| HlyD family secretion protein [Bradyrhizobium japonicum USDA 110]
>gi|27382104|ref|NP_773633.1| hypothetical protein bll6993 [Bradyrhizobium japonicum USDA 110]
>gi|27382107|ref|NP_773636.1| hypothetical protein blr6996 [Bradyrhizobium japonicum USDA 110]
>gi|27382112|ref|NP_773641.1| hypothetical protein blr7001 [Bradyrhizobium japonicum USDA 110]
>gi|27382129|ref|NP_773658.1| hypothetical protein bll7018 [Bradyrhizobium japonicum USDA 110]
>gi|27382132|ref|NP_773661.1| HlyD family secretion protein [Bradyrhizobium japonicum USDA 110]
>gi|27382146|ref|NP_773675.1| transcriptional regulatory protein [Bradyrhizobium japonicum USDA 110]
>gi|27382156|ref|NP_773685.1| hypothetical protein bsr7045 [Bradyrhizobium japonicum USDA 110]
>gi|27382162|ref|NP_773691.1| hypothetical protein bll7051 [Bradyrhizobium japonicum USDA 110]
>gi|27382182|ref|NP_773711.1| TonB protein [Bradyrhizobium japonicum USDA 110]
>gi|27382225|ref|NP_773754.1| phosphoribosylaminoimidazole carboxylase catalytic subunit [Bradyrhizobium japonicum USDA 110]
>gi|27382228|ref|NP_773757.1| 30S ribosomal protein S21 [Bradyrhizobium japonicum USDA 110]
>gi|27382230|ref|NP_773759.1| hypothetical protein bll7119 [Bradyrhizobium japonicum USDA 110]
>gi|27382232|ref|NP_773761.1| putative ionic transporter [Bradyrhizobium japonicum USDA 110]
>gi|27382242|ref|NP_773771.1| hypothetical protein blr7131 [Bradyrhizobium japonicum USDA 110]
>gi|27382243|ref|NP_773772.1| hypothetical protein blr7132 [Bradyrhizobium japonicum USDA 110]
>gi|27382244|ref|NP_773773.1| hypothetical protein blr7133 [Bradyrhizobium japonicum USDA 110]
>gi|27382250|ref|NP_773779.1| transcriptional regulatory protein [Bradyrhizobium japonicum USDA 110]
>gi|27382258|ref|NP_773787.1| hypothetical protein bll7147 [Bradyrhizobium japonicum USDA 110]
>gi|27382259|ref|NP_773788.1| hypothetical protein bll7148 [Bradyrhizobium japonicum USDA 110]
>gi|27382264|ref|NP_773793.1| hypothetical protein bll7153 [Bradyrhizobium japonicum USDA 110]
>gi|27382268|ref|NP_773797.1| putative glucose 1-dehydrogenase (EC 1.1.1.47) [Bradyrhizobium japonicum USDA 110]
>gi|27382271|ref|NP_773800.1| hypothetical protein bll7160 [Bradyrhizobium japonicum USDA 110]
>gi|27382284|ref|NP_773813.1| putative methyl accepting chemotaxis protein [Bradyrhizobium japonicum USDA 110]
>gi|27382287|ref|NP_773816.1| hypothetical protein bll7176 [Bradyrhizobium japonicum USDA 110]
>gi|27382324|ref|NP_773853.1| hypothetical protein bll7213 [Bradyrhizobium japonicum USDA 110]
>gi|27382336|ref|NP_773865.1| hypothetical protein bll7225 [Bradyrhizobium japonicum USDA 110]
>gi|27382337|ref|NP_773866.1| hypothetical protein bll7226 [Bradyrhizobium japonicum USDA 110]
>gi|27382340|ref|NP_773869.1| hypothetical protein bll7229 [Bradyrhizobium japonicum USDA 110]
>gi|27382365|ref|NP_773894.1| hypothetical protein blr7254 [Bradyrhizobium japonicum USDA 110]
>gi|27382371|ref|NP_773900.1| leucine-responsive transcriptional regulatory protein [Bradyrhizobium japonicum USDA 110]
>gi|27382375|ref|NP_773904.1| hypothetical protein bll7264 [Bradyrhizobium japonicum USDA 110]
>gi|27382386|ref|NP_773915.1| transcriptional regulatory protein [Bradyrhizobium japonicum USDA 110]
>gi|27382390|ref|NP_773919.1| hypothetical protein bll7279 [Bradyrhizobium japonicum USDA 110]
>gi|27382396|ref|NP_773925.1| hypothetical protein blr7285 [Bradyrhizobium japonicum USDA 110]
>gi|27382405|ref|NP_773934.1| hypothetical protein bll7294 [Bradyrhizobium japonicum USDA 110]
>gi|27382407|ref|NP_773936.1| hypothetical protein blr7296 [Bradyrhizobium japonicum USDA 110]
>gi|27382428|ref|NP_773957.1| hypothetical protein bsr7317 [Bradyrhizobium japonicum USDA 110]

>gi|27382429|ref|NP_773958.1| hypothetical protein blr7318 [Bradyrhizobium japonicum USDA 110]
>gi|27382431|ref|NP_773960.1| hypothetical protein blr7320 [Bradyrhizobium japonicum USDA 110]
>gi|27382433|ref|NP_773962.1| hypothetical protein bll7322 [Bradyrhizobium japonicum USDA 110]
>gi|27382436|ref|NP_773965.1| hypothetical protein blr7325 [Bradyrhizobium japonicum USDA 110]
>gi|27382438|ref|NP_773967.1| hypothetical protein blr7327 [Bradyrhizobium japonicum USDA 110]
>gi|27382450|ref|NP_773979.1| hypothetical protein blr7339 [Bradyrhizobium japonicum USDA 110]
>gi|27382452|ref|NP_773981.1| RhtB family transporter [Bradyrhizobium japonicum USDA 110]
>gi|27382477|ref|NP_774006.1| transcriptional regulatory protein [Bradyrhizobium japonicum USDA 110]
>gi|27382487|ref|NP_774016.1| putative integral membrane transporter protein [Bradyrhizobium japonicum USDA 110]
>gi|27382503|ref|NP_774032.1| hypothetical protein bsr7392 [Bradyrhizobium japonicum USDA 110]
>gi|27382507|ref|NP_774036.1| two-component hybrid sensor and regulator [Bradyrhizobium japonicum USDA 110]
>gi|27382518|ref|NP_774047.1| fosmidomycin resistance protein [Bradyrhizobium japonicum USDA 110]
>gi|27382526|ref|NP_774055.1| hypothetical protein bll7415 [Bradyrhizobium japonicum USDA 110]
>gi|27382543|ref|NP_774072.1| hypothetical protein bsl7432 [Bradyrhizobium japonicum USDA 110]
>gi|27382552|ref|NP_774081.1| 50S ribosomal protein L25 [Bradyrhizobium japonicum USDA 110]
>gi|27382553|ref|NP_774082.1| hypothetical protein bsl7442 [Bradyrhizobium japonicum USDA 110]
>gi|27382586|ref|NP_774115.1| hypothetical protein blr7475 [Bradyrhizobium japonicum USDA 110]
>gi|27382606|ref|NP_774135.1| hypothetical protein bll7495 [Bradyrhizobium japonicum USDA 110]
>gi|27382616|ref|NP_774145.1| hypothetical protein bll7505 [Bradyrhizobium japonicum USDA 110]
>gi|27382633|ref|NP_774162.1| hypothetical protein bll7522 [Bradyrhizobium japonicum USDA 110]
>gi|27382639|ref|NP_774168.1| hypothetical protein blr7528 [Bradyrhizobium japonicum USDA 110]
>gi|27382650|ref|NP_774179.1| probable histone H1 [Bradyrhizobium japonicum USDA 110]
>gi|27382698|ref|NP_774227.1| hypothetical protein blr7587 [Bradyrhizobium japonicum USDA 110]
>gi|27382724|ref|NP_774253.1| hypothetical protein bll7613 [Bradyrhizobium japonicum USDA 110]
>gi|27382732|ref|NP_774261.1| hypothetical protein blr7621 [Bradyrhizobium japonicum USDA 110]
>gi|27382744|ref|NP_774273.1| hypothetical protein bsr7633 [Bradyrhizobium japonicum USDA 110]
>gi|27382751|ref|NP_774280.1| hypothetical protein bll7640 [Bradyrhizobium japonicum USDA 110]
>gi|27382753|ref|NP_774282.1| hypothetical exported glutamine-rich protein [Bradyrhizobium japonicum USDA 110]
>gi|27382754|ref|NP_774283.1| hypothetical protein bsr7643 [Bradyrhizobium japonicum USDA 110]
>gi|27382755|ref|NP_774284.1| hypothetical protein bll7644 [Bradyrhizobium japonicum USDA 110]
>gi|27382763|ref|NP_774292.1| hypothetical protein blr7652 [Bradyrhizobium japonicum USDA 110]
>gi|27382773|ref|NP_774302.1| hypothetical protein bsr7662 [Bradyrhizobium japonicum USDA 110]
>gi|27382778|ref|NP_774307.1| hypothetical protein bsl7667 [Bradyrhizobium japonicum USDA 110]
>gi|27382784|ref|NP_774313.1| hypothetical protein bll7673 [Bradyrhizobium japonicum USDA 110]
>gi|27382788|ref|NP_774317.1| hypothetical protein blr7677 [Bradyrhizobium japonicum USDA 110]
>gi|27382810|ref|NP_774339.1| hypothetical protein bsr7699 [Bradyrhizobium japonicum USDA 110]
>gi|27382813|ref|NP_774342.1| hypothetical protein bll7702 [Bradyrhizobium japonicum USDA 110]
>gi|27382820|ref|NP_774349.1| hypothetical protein bll7709 [Bradyrhizobium japonicum USDA 110]
>gi|27382824|ref|NP_774353.1| hypothetical protein blr7713 [Bradyrhizobium japonicum USDA 110]
>gi|27382833|ref|NP_774362.1| hypothetical protein blr7722 [Bradyrhizobium japonicum USDA 110]
>gi|27382835|ref|NP_774364.1| hypothetical protein blr7724 [Bradyrhizobium japonicum USDA 110]
>gi|27382844|ref|NP_774373.1| transcriptional regulatory protein [Bradyrhizobium japonicum USDA 110]
>gi|27382853|ref|NP_774382.1| hypothetical protein bll7742 [Bradyrhizobium japonicum USDA 110]
>gi|27382861|ref|NP_774390.1| hypothetical protein bll7750 [Bradyrhizobium japonicum USDA 110]
>gi|27382865|ref|NP_774394.1| hypothetical protein bll7754 [Bradyrhizobium japonicum USDA 110]
>gi|27382875|ref|NP_774404.1| hypothetical protein bsr7764 [Bradyrhizobium japonicum USDA 110]
>gi|27382879|ref|NP_774408.1| hypothetical protein bll7768 [Bradyrhizobium japonicum USDA 110]
>gi|27382883|ref|NP_774412.1| hypothetical protein bll7772 [Bradyrhizobium japonicum USDA 110]
>gi|27382899|ref|NP_774428.1| hypothetical protein blr7788 [Bradyrhizobium japonicum USDA 110]
>gi|27382903|ref|NP_774432.1| hypothetical protein bll7792 [Bradyrhizobium japonicum USDA 110]
>gi|27382924|ref|NP_774453.1| transcriptional regulatory protein [Bradyrhizobium japonicum USDA 110]
>gi|27382941|ref|NP_774470.1| hydroxypyruvate reductase [Bradyrhizobium japonicum USDA 110]
>gi|27382964|ref|NP_774493.1| hypothetical protein blr7853 [Bradyrhizobium japonicum USDA 110]
>gi|27382991|ref|NP_774520.1| putative oxidoreductase protein [Bradyrhizobium japonicum USDA 110]
>gi|27382993|ref|NP_774522.1| hypothetical protein bll7882 [Bradyrhizobium japonicum USDA 110]
>gi|27382994|ref|NP_774523.1| hypothetical protein blr7883 [Bradyrhizobium japonicum USDA 110]
>gi|27383022|ref|NP_774551.1| hypothetical protein bll7911 [Bradyrhizobium japonicum USDA 110]
>gi|27383026|ref|NP_774555.1| hypothetical protein bsl7915 [Bradyrhizobium japonicum USDA 110]
>gi|27383035|ref|NP_774564.1| hypothetical protein bll7924 [Bradyrhizobium japonicum USDA 110]
>gi|27383036|ref|NP_774565.1| hypothetical protein blr7925 [Bradyrhizobium japonicum USDA 110]
>gi|27383062|ref|NP_774591.1| hypothetical protein bll7951 [Bradyrhizobium japonicum USDA 110]
>gi|27383074|ref|NP_774603.1| hypothetical protein bll7963 [Bradyrhizobium japonicum USDA 110]
>gi|27383103|ref|NP_774632.1| hypothetical protein bsl7992 [Bradyrhizobium japonicum USDA 110]
>gi|27383119|ref|NP_774648.1| similar to recombinase [Bradyrhizobium japonicum USDA 110]
>gi|27383128|ref|NP_774657.1| hypothetical protein blr8017 [Bradyrhizobium japonicum USDA 110]
>gi|27383132|ref|NP_774661.1| hypothetical protein bsl8021 [Bradyrhizobium japonicum USDA 110]
>gi|27383134|ref|NP_774663.1| hypothetical protein bsl8023 [Bradyrhizobium japonicum USDA 110]
>gi|27383136|ref|NP_774665.1| hypothetical protein bll8025 [Bradyrhizobium japonicum USDA 110]
>gi|27383143|ref|NP_774672.1| hypothetical protein bll8032 [Bradyrhizobium japonicum USDA 110]
>gi|27383145|ref|NP_774674.1| hypothetical protein bll8034 [Bradyrhizobium japonicum USDA 110]

>gi|27383146|ref|NP_774675.1| hypothetical protein bll8035 [Bradyrhizobium japonicum USDA 110]
>gi|27383154|ref|NP_774683.1| hypothetical protein bll8043 [Bradyrhizobium japonicum USDA 110]
>gi|27383159|ref|NP_774688.1| hypothetical protein bll8048 [Bradyrhizobium japonicum USDA 110]
>gi|27383167|ref|NP_774696.1| hypothetical protein bll8056 [Bradyrhizobium japonicum USDA 110]
>gi|27383168|ref|NP_774697.1| hypothetical protein bll8057 [Bradyrhizobium japonicum USDA 110]
>gi|27383169|ref|NP_774698.1| hypothetical protein bsl8058 [Bradyrhizobium japonicum USDA 110]
>gi|27383175|ref|NP_774704.1| hypothetical protein blr8064 [Bradyrhizobium japonicum USDA 110]
>gi|27383191|ref|NP_774720.1| hypothetical protein blr8080 [Bradyrhizobium japonicum USDA 110]
>gi|27383196|ref|NP_774725.1| hypothetical protein bll8085 [Bradyrhizobium japonicum USDA 110]
>gi|27383211|ref|NP_774740.1| hypothetical protein blr8100 [Bradyrhizobium japonicum USDA 110]
>gi|27383231|ref|NP_774760.1| putative dehdyrogenase [Bradyrhizobium japonicum USDA 110]
>gi|27383259|ref|NP_774788.1| phosphocarrier protein HPr [Bradyrhizobium japonicum USDA 110]
>gi|27383262|ref|NP_774791.1| hypothetical protein blr8151 [Bradyrhizobium japonicum USDA 110]
>gi|27383270|ref|NP_774799.1| hypothetical protein blr8159 [Bradyrhizobium japonicum USDA 110]
>gi|27383273|ref|NP_774802.1| molybdenum ABC transporter ATP-binding protein [Bradyrhizobium japonicum USDA 110]
>gi|27383283|ref|NP_774812.1| hypothetical protein blr8172 [Bradyrhizobium japonicum USDA 110]
>gi|27383295|ref|NP_774824.1| hypothetical protein bll8184 [Bradyrhizobium japonicum USDA 110]
>gi|27383315|ref|NP_774844.1| putative transposase [Bradyrhizobium japonicum USDA 110]
>gi|27383320|ref|NP_774849.1| hypothetical protein bll8209 [Bradyrhizobium japonicum USDA 110]
>gi|27383328|ref|NP_774857.1| putative transposase [Bradyrhizobium japonicum USDA 110]
>gi|27383337|ref|NP_774866.1| hypothetical protein blr8226 [Bradyrhizobium japonicum USDA 110]
>gi|27383348|ref|NP_774877.1| hypothetical protein bll8237 [Bradyrhizobium japonicum USDA 110]
>gi|27383351|ref|NP_774880.1| hypothetical protein blr8240 [Bradyrhizobium japonicum USDA 110]
>gi|27383379|ref|NP_774908.1| hypothetical protein bsl8268 [Bradyrhizobium japonicum USDA 110]
>gi|27383386|ref|NP_774915.1| hypothetical protein bll8275 [Bradyrhizobium japonicum USDA 110]
>gi|27383393|ref|NP_774922.1| probable conjugal transfer protein [Bradyrhizobium japonicum USDA 110]
>gi|27383394|ref|NP_774923.1| hypothetical protein bll8283 [Bradyrhizobium japonicum USDA 110]
>gi|27383397|ref|NP_774926.1| probable conjugal transfer protein [Bradyrhizobium japonicum USDA 110]
>gi|27383401|ref|NP_774930.1| putative transposase [Bradyrhizobium japonicum USDA 110]
>gi|27383409|ref|NP_774938.1| hypothetical protein blr8298 [Bradyrhizobium japonicum USDA 110]
>gi|27383421|ref|NP_774950.1| hypothetical protein bll8310 [Bradyrhizobium japonicum USDA 110]
>gi|33241346|ref|NP_876287.1| HB2 protein [Chlamydophila pneumoniae TW-183]
>gi|33241348|ref|NP_876289.1| HB6 protein [Chlamydophila pneumoniae TW-183]
>gi|33241365|ref|NP_876306.1| hypothetical protein CpB0030 [Chlamydophila pneumoniae TW-183]
>gi|33241367|ref|NP_876308.1| hypothetical protein CpB0032 [Chlamydophila pneumoniae TW-183]
>gi|33241370|ref|NP_876311.1| ribosomal protein S21 [Chlamydophila pneumoniae TW-183]
>gi|33241385|ref|NP_876326.1| hypothetical protein CpB0050 [Chlamydophila pneumoniae TW-183]
>gi|33241386|ref|NP_876327.1| hypothetical protein CpB0051 [Chlamydophila pneumoniae TW-183]
>gi|33241399|ref|NP_876340.1| hypothetical protein CpB0064 [Chlamydophila pneumoniae TW-183]
>gi|33241400|ref|NP_876341.1| hypothetical protein CpB0065 [Chlamydophila pneumoniae TW-183]
>gi|33241483|ref|NP_876424.1| hypothetical protein CpB0148 [Chlamydophila pneumoniae TW-183]
>gi|33241502|ref|NP_876443.1| hypothetical protein CpB0167 [Chlamydophila pneumoniae TW-183]
>gi|33241511|ref|NP_876452.1| hypothetical protein CpB0176 [Chlamydophila pneumoniae TW-183]
>gi|33241512|ref|NP_876453.1| hypothetical protein CpB0177 [Chlamydophila pneumoniae TW-183]
>gi|33241550|ref|NP_876491.1| hypothetical protein CpB0215 [Chlamydophila pneumoniae TW-183]
>gi|33241555|ref|NP_876496.1| hypothetical protein CpB0220 [Chlamydophila pneumoniae TW-183]
>gi|33241560|ref|NP_876501.1| hypothetical protein CpB0225 [Chlamydophila pneumoniae TW-183]
>gi|33241566|ref|NP_876507.1| hypothetical protein CpB0231 [Chlamydophila pneumoniae TW-183]
>gi|33241581|ref|NP_876522.1| hypothetical protein CpB0246 [Chlamydophila pneumoniae TW-183]
>gi|33241608|ref|NP_876549.1| HB6 protein [Chlamydophila pneumoniae TW-183]
>gi|33241610|ref|NP_876551.1| hypothetical protein CpB0275 [Chlamydophila pneumoniae TW-183]
>gi|33241620|ref|NP_876561.1| hypothetical protein CpB0285 [Chlamydophila pneumoniae TW-183]
>gi|33241635|ref|NP_876576.1| inclusion membrane protein B [Chlamydophila pneumoniae TW-183]
>gi|33241646|ref|NP_876587.1| UDP glucosamine N-acyltransferase [Chlamydophila pneumoniae TW-183]
>gi|33241652|ref|NP_876593.1| hypothetical protein CpB0318 [Chlamydophila pneumoniae TW-183]
>gi|33241686|ref|NP_876627.1| putative integral membrane protein [Chlamydophila pneumoniae TW-183]
>gi|33241694|ref|NP_876635.1| hypothetical protein CpB0361 [Chlamydophila pneumoniae TW-183]
>gi|33241712|ref|NP_876653.1| hypothetical protein CpB0379 [Chlamydophila pneumoniae TW-183]
>gi|33241716|ref|NP_876657.1| hypothetical protein CpB0383 [Chlamydophila pneumoniae TW-183]
>gi|33241729|ref|NP_876670.1| putative histone-like protein 2 [Chlamydophila pneumoniae TW-183]
>gi|33241738|ref|NP_876679.1| hypothetical protein CpB0405 [Chlamydophila pneumoniae TW-183]
>gi|33241751|ref|NP_876692.1| hypothetical protein CpB0418 [Chlamydophila pneumoniae TW-183]
>gi|33241791|ref|NP_876732.1| hypothetical protein CpB0458 [Chlamydophila pneumoniae TW-183]
>gi|33241823|ref|NP_876764.1| hypothetical protein CpB0490 [Chlamydophila pneumoniae TW-183]
>gi|33241825|ref|NP_876766.1| hypothetical protein CpB0492 [Chlamydophila pneumoniae TW-183]
>gi|33241836|ref|NP_876777.1| hypothetical protein CpB0505 [Chlamydophila pneumoniae TW-183]
>gi|33241875|ref|NP_876816.1| hypothetical protein CpB0544 [Chlamydophila pneumoniae TW-183]
>gi|33241925|ref|NP_876866.1| hypothetical protein CpB0594 [Chlamydophila pneumoniae TW-183]
>gi|33241987|ref|NP_876928.1| ribosomal protein L15 [Chlamydophila pneumoniae TW-183]
>gi|33242036|ref|NP_876977.1| hypothetical protein CpB0705 [Chlamydophila pneumoniae TW-183]

>gi|33242064|ref|NP_877005.1| hypothetical protein CpB0733 [Chlamydophila pneumoniae TW-183]
>gi|33242079|ref|NP_877020.1| hypothetical protein CpB0748 [Chlamydophila pneumoniae TW-183]
>gi|33242087|ref|NP_877028.1| hypothetical protein CpB0756 [Chlamydophila pneumoniae TW-183]
>gi|33242168|ref|NP_877109.1| hypothetical protein CpB0837 [Chlamydophila pneumoniae TW-183]
>gi|33242169|ref|NP_877110.1| hypothetical protein CpB0838 [Chlamydophila pneumoniae TW-183]
>gi|33242247|ref|NP_877188.1| histone H1-like protein [Chlamydophila pneumoniae TW-183]
>gi|33242261|ref|NP_877202.1| hypothetical protein CpB0930 [Chlamydophila pneumoniae TW-183]
>gi|33242300|ref|NP_877241.1| ribosomal protein L34 [Chlamydophila pneumoniae TW-183]
>gi|33242373|ref|NP_877314.1| hypothetical protein CpB1042 [Chlamydophila pneumoniae TW-183]
>gi|33242374|ref|NP_877315.1| hypothetical protein CpB1043 [Chlamydophila pneumoniae TW-183]
>gi|33242375|ref|NP_877316.1| hypothetical protein CpB1044 [Chlamydophila pneumoniae TW-183]
>gi|33242379|ref|NP_877320.1| hypothetical protein CpB1048 [Chlamydophila pneumoniae TW-183]
>gi|33242383|ref|NP_877324.1| sulfate transporter [Chlamydophila pneumoniae TW-183]
>gi|33242393|ref|NP_877334.1| hypothetical protein CpB1062 [Chlamydophila pneumoniae TW-183]
>gi|33242443|ref|NP_877384.1| hypothetical protein CpB1113 [Chlamydophila pneumoniae TW-183]
>gi|16081229|ref|NP_393531.1| apoptosis-related Tfar19 related protein [Thermoplasma acidophilum DSM 1728]
>gi|16081396|ref|NP_393733.1| hypothetical membrane protein [Thermoplasma acidophilum DSM 1728]
>gi|16081487|ref|NP_393837.1| probable 50S ribosomal protein L12 [Thermoplasma acidophilum DSM 1728]
>gi|16081518|ref|NP_393869.1| DNA-DIRECTED RNA POLYMERASE CHAIN H related protein [Thermoplasma acidophilum DSM 1728]
>gi|16081540|ref|NP_393896.1| hypothetical membrane protein [Thermoplasma acidophilum DSM 1728]
>gi|16081542|ref|NP_393898.1| conserved hypothetical membrane protein [Thermoplasma acidophilum DSM 1728]
>gi|16081622|ref|NP_393985.1| hypothetical membrane protein [Thermoplasma acidophilum DSM 1728]
>gi|16081639|ref|NP_394005.1| homoserine kinase related protein [Thermoplasma acidophilum DSM 1728]
>gi|16081729|ref|NP_394113.1| hypothetical membrane protein [Thermoplasma acidophilum DSM 1728]
>gi|16081828|ref|NP_394221.1| hypothetical membrane protein [Thermoplasma acidophilum DSM 1728]
>gi|16081859|ref|NP_394258.1| conserved hypothetical membrane protein [Thermoplasma acidophilum DSM 1728]
>gi|16081885|ref|NP_394288.1| hypothetical protein Ta0827 [Thermoplasma acidophilum DSM 1728]
>gi|16081962|ref|NP_394372.1| conserved hypothetical membrane protein [Thermoplasma acidophilum DSM 1728]
>gi|16082007|ref|NP_394424.1| NADH dehydrogenase, chain J related protein [Thermoplasma acidophilum DSM 1728]
>gi|16082031|ref|NP_394453.1| hypothetical membrane protein [Thermoplasma acidophilum DSM 1728]
>gi|16082067|ref|NP_394493.1| probable ribosomal protein S13 [Thermoplasma acidophilum DSM 1728]
>gi|16082146|ref|NP_394584.1| hypothetical protein Ta1125 [Thermoplasma acidophilum DSM 1728]
>gi|16082369|ref|NP_394846.1| hypothetical protein Ta1392 [Thermoplasma acidophilum DSM 1728]
>gi|16082597|ref|NP_394641.1| Kef-type K+ transport system, membrane component [Thermoplasma acidophilum DSM 1728]
>gi|16082619|ref|NP_394860.1| Permease (major facilitator superfamily) [Thermoplasma acidophilum DSM 1728]
>gi|16082633|ref|NP_393485.1| Archaeal-type H+-ATPase subunit H [Thermoplasma acidophilum DSM 1728]
>gi|16082636|ref|NP_393589.1| hypothetical protein Ta0110a [Thermoplasma acidophilum DSM 1728]
>gi|16082641|ref|NP_393713.1| hypothetical protein Ta0233a [Thermoplasma acidophilum DSM 1728]
>gi|16082661|ref|NP_394716.1| 50S ribosomal protein L24 [Thermoplasma acidophilum DSM 1728]
>gi|51038607|ref|YP_063253.1| hypothetical protein BGB10 [Borrelia garinii PBi]
>gi|51038608|ref|YP_063254.1| hypothetical protein BGB11 [Borrelia garinii PBi]
>gi|51038610|ref|YP_063256.1| hypothetical protein BGB13 [Borrelia garinii PBi]
>gi|51038637|ref|YP_063282.1| hypothetical protein BGA13 [Borrelia garinii PBi]
>gi|51038640|ref|YP_063285.1| hypothetical protein BGA16 [Borrelia garinii PBi]
>gi|51038650|ref|YP_063295.1| hypothetical protein BGA26 [Borrelia garinii PBi]
>gi|51038653|ref|YP_063298.1| hypothetical protein BGA29 [Borrelia garinii PBi]
>gi|51038655|ref|YP_063300.1| hypothetical protein BGA31 [Borrelia garinii PBi]
>gi|51038662|ref|YP_063307.1| hypothetical protein BGA38 [Borrelia garinii PBi]
>gi|51038671|ref|YP_063316.1| hypothetical protein BGA47 [Borrelia garinii PBi]
>gi|51038675|ref|YP_063320.1| outer membrane protein [Borrelia garinii PBi]
>gi|51038682|ref|YP_063327.1| hypothetical protein BGA58 [Borrelia garinii PBi]
>gi|51038684|ref|YP_063329.1| surface lipoprotein P27 [Borrelia garinii PBi]
>gi|51038689|ref|YP_063334.1| antigen, P35, putative [Borrelia garinii PBi]
>gi|51038691|ref|YP_063336.1| hypothetical protein BGA67 [Borrelia garinii PBi]
>gi|51598272|ref|YP_072460.1| hypothetical protein BG0009 [Borrelia garinii PBi]
>gi|51598280|ref|YP_072468.1| conserved hypothetical integral membrane protein [Borrelia garinii PBi]
>gi|51598315|ref|YP_072503.1| hypothetical protein BG0053 [Borrelia garinii PBi]
>gi|51598323|ref|YP_072511.1| hypothetical protein BG0061 [Borrelia garinii PBi]
>gi|51598332|ref|YP_072520.1| hypothetical protein BG0070 [Borrelia garinii PBi]
>gi|51598334|ref|YP_072522.1| hypothetical protein BG0072 [Borrelia garinii PBi]
>gi|51598336|ref|YP_072524.1| hypothetical protein BG0074 [Borrelia garinii PBi]
>gi|51598337|ref|YP_072525.1| signal recognition particle-docking protein FtsY [Borrelia garinii PBi]
>gi|51598339|ref|YP_072527.1| hypothetical protein BG0077 [Borrelia garinii PBi]
>gi|51598341|ref|YP_072529.1| hypothetical protein BG0079 [Borrelia garinii PBi]
>gi|51598345|ref|YP_072533.1| hypothetical protein BG0083 [Borrelia garinii PBi]
>gi|51598347|ref|YP_072535.1| hypothetical protein BG0085 [Borrelia garinii PBi]
>gi|51598353|ref|YP_072541.1| V-type ATPase, subunit K, putative [Borrelia garinii PBi]
>gi|51598365|ref|YP_072553.1| hypothetical protein BG0103 [Borrelia garinii PBi]
>gi|51598382|ref|YP_072570.1| phosphatidate cytidylyltransferase [Borrelia garinii PBi]
>gi|51598387|ref|YP_072575.1| hypothetical protein BG0125 [Borrelia garinii PBi]

>gi|51598390|ref|YP_072578.1| hypothetical protein BG0128 [Borrelia garinii PBi]
>gi|51598394|ref|YP_072582.1| hypothetical protein BG0132 [Borrelia garinii PBi]
>gi|51598424|ref|YP_072612.1| Na+/Ca+ exchange protein, putative [Borrelia garinii PBi]
>gi|51598438|ref|YP_072626.1| glucose inhibited division protein B [Borrelia garinii PBi]
>gi|51598441|ref|YP_072629.1| flagellar protein, putative [Borrelia garinii PBi]
>gi|51598445|ref|YP_072633.1| carbon storage regulator [Borrelia garinii PBi]
>gi|51598450|ref|YP_072638.1| ribosomal protein L35 [Borrelia garinii PBi]
>gi|51598453|ref|YP_072641.1| lipoprotein, putative [Borrelia garinii PBi]
>gi|51598456|ref|YP_072644.1| peptide chain release factor 1 [Borrelia garinii PBi]
>gi|51598459|ref|YP_072647.1| hypothetical protein BG0197 [Borrelia garinii PBi]
>gi|51598463|ref|YP_072651.1| hemolysin, putative [Borrelia garinii PBi]
>gi|51598469|ref|YP_072657.1| hypothetical protein BG0210 [Borrelia garinii PBi]
>gi|51598473|ref|YP_072661.1| hypothetical protein BG0214 [Borrelia garinii PBi]
>gi|51598495|ref|YP_072683.1| conserved hypothetical integral membrane protein [Borrelia garinii PBi]
>gi|51598509|ref|YP_072697.1| phosphatidyltransferase [Borrelia garinii PBi]
>gi|51598510|ref|YP_072698.1| dedA protein [Borrelia garinii PBi]
>gi|51598518|ref|YP_072706.1| bacitracin resistance protein [Borrelia garinii PBi]
>gi|51598520|ref|YP_072708.1| hypothetical protein BG0263 [Borrelia garinii PBi]
>gi|51598522|ref|YP_072710.1| hypothetical protein BG0265 [Borrelia garinii PBi]
>gi|51598525|ref|YP_072713.1| hypothetical protein BG0268 [Borrelia garinii PBi]
>gi|51598533|ref|YP_072721.1| flagellar biosynthesis protein [Borrelia garinii PBi]
>gi|51598545|ref|YP_072733.1| flagellar protein [Borrelia garinii PBi]
>gi|51598546|ref|YP_072734.1| flagellar protein [Borrelia garinii PBi]
>gi|51598549|ref|YP_072737.1| flagellar assembly protein [Borrelia garinii PBi]
>gi|51598563|ref|YP_072751.1| cell division protein [Borrelia garinii PBi]
>gi|51598576|ref|YP_072764.1| conserved hypothetical integral membrane protein [Borrelia garinii PBi]
>gi|51598577|ref|YP_072765.1| conserved hypothetical integral membrane protein [Borrelia garinii PBi]
>gi|51598589|ref|YP_072777.1| oligopeptide ABC transporter, permease protein [Borrelia garinii PBi]
>gi|51598601|ref|YP_072789.1| Glu-tRNA(Gln) amidotransferase, subunit C [Borrelia garinii PBi]
>gi|51598607|ref|YP_072795.1| hypothetical protein BG0350 [Borrelia garinii PBi]
>gi|51598611|ref|YP_072799.1| hypothetical protein BG0354 [Borrelia garinii PBi]
>gi|51598612|ref|YP_072800.1| hypothetical protein BG0355 [Borrelia garinii PBi]
>gi|51598656|ref|YP_072844.1| hypothetical protein BG0402 [Borrelia garinii PBi]
>gi|51598703|ref|YP_072891.1| phosphocarrier protein HPr [Borrelia garinii PBi]
>gi|51598706|ref|YP_072894.1| chromate transport protein, putative [Borrelia garinii PBi]
>gi|51598709|ref|YP_072897.1| hypothetical protein BG0462 [Borrelia garinii PBi]
>gi|51598742|ref|YP_072930.1| ribosomal protein S17 [Borrelia garinii PBi]
>gi|51598751|ref|YP_072939.1| ribosomal protein L30 [Borrelia garinii PBi]
>gi|51598762|ref|YP_072950.1| hypothetical protein BG0519 [Borrelia garinii PBi]
>gi|51598788|ref|YP_072976.1| hypothetical protein BG0547 [Borrelia garinii PBi]
>gi|51598789|ref|YP_072977.1| hypothetical protein BG0548 [Borrelia garinii PBi]
>gi|51598797|ref|YP_072985.1| hypothetical protein BG0556 [Borrelia garinii PBi]
>gi|51598798|ref|YP_072986.1| hypothetical protein BG0557 [Borrelia garinii PBi]
>gi|51598804|ref|YP_072992.1| hypothetical protein BG0563 [Borrelia garinii PBi]
>gi|51598825|ref|YP_073013.1| conserved hypothetical integral membrane protein [Borrelia garinii PBi]
>gi|51598827|ref|YP_073015.1| hypothetical protein BG0587 [Borrelia garinii PBi]
>gi|51598832|ref|YP_073020.1| conserved hypothetical integral membrane protein [Borrelia garinii PBi]
>gi|51598844|ref|YP_073032.1| competence locus E, putative [Borrelia garinii PBi]
>gi|51598845|ref|YP_073033.1| hypothetical protein BG0605 [Borrelia garinii PBi]
>gi|51598896|ref|YP_073084.1| spermidine/putrescine ABC transporter, permease protein [Borrelia garinii PBi]
>gi|51598919|ref|YP_073107.1| hypothetical protein BG0687 [Borrelia garinii PBi]
>gi|51598922|ref|YP_073110.1| hypothetical protein BG0690 [Borrelia garinii PBi]
>gi|51598929|ref|YP_073117.1| hypothetical protein BG0697 [Borrelia garinii PBi]
>gi|51598961|ref|YP_073149.1| hypothetical protein BG0730 [Borrelia garinii PBi]
>gi|51598969|ref|YP_073157.1| conserved hypothetical integral membrane protein [Borrelia garinii PBi]
>gi|51598975|ref|YP_073163.1| adenylyl cyclase, CyaB-type, putative [Borrelia garinii PBi]
>gi|51599003|ref|YP_073191.1| membrane spanning protein, putative [Borrelia garinii PBi]
>gi|51599006|ref|YP_073194.1| hypothetical protein BG0778 [Borrelia garinii PBi]
>gi|51599010|ref|YP_073198.1| hypothetical protein BG0783 [Borrelia garinii PBi]
>gi|51599014|ref|YP_073202.1| sensory transduction histidine kinase, putative [Borrelia garinii PBi]
>gi|51599015|ref|YP_073203.1| hypothetical protein BG0788 [Borrelia garinii PBi]
>gi|51599016|ref|YP_073204.1| colicin V production protein, putative [Borrelia garinii PBi]
>gi|51599023|ref|YP_073211.1| flagellar P-ring protein [Borrelia garinii PBi]
>gi|51599053|ref|YP_073241.1| translation initiation factor 2 [Borrelia garinii PBi]
>gi|51599059|ref|YP_073247.1| conserved hypothetical integral membrane protein [Borrelia garinii PBi]
>gi|51599077|ref|YP_073265.1| hypothetical protein BG0851 [Borrelia garinii PBi]
>gi|51599081|ref|YP_073269.1| exonuclease SbcC [Borrelia garinii PBi]
>gi|51599092|ref|YP_073280.1| hypothetical protein BG0866 [Borrelia garinii PBi]
>gi|20088916|ref|NP_614991.1| dynein heavy chain [Methanosarcina acetivorans C2A]
>gi|20088922|ref|NP_614997.1| zinc ABC transporter, solute-binding lipoprotein [Methanosarcina acetivorans C2A]

>gi|20088927|ref|NP_615002.1| hypothetical protein MA0028 [Methanosarcina acetivorans C2A]
>gi|20088928|ref|NP_615003.1| hypothetical protein MA0029 [Methanosarcina acetivorans C2A]
>gi|20088943|ref|NP_615018.1| hypothetical protein MA0044 [Methanosarcina acetivorans C2A]
>gi|20088951|ref|NP_615026.1| hypothetical protein MA0052 [Methanosarcina acetivorans C2A]
>gi|20088981|ref|NP_615056.1| hypothetical protein MA0082 [Methanosarcina acetivorans C2A]
>gi|20088991|ref|NP_615066.1| hypothetical protein MA0092 [Methanosarcina acetivorans C2A]
>gi|20088992|ref|NP_615067.1| hypothetical protein MA0093 [Methanosarcina acetivorans C2A]
>gi|20089002|ref|NP_615077.1| GPR1/FUN34/yaaH family protein [Methanosarcina acetivorans C2A]
>gi|20089053|ref|NP_615128.1| hypothetical protein MA0155 [Methanosarcina acetivorans C2A]
>gi|20089055|ref|NP_615130.1| hypothetical protein MA0157 [Methanosarcina acetivorans C2A]
>gi|20089056|ref|NP_615131.1| hypothetical protein MA0158 [Methanosarcina acetivorans C2A]
>gi|20089057|ref|NP_615132.1| hypothetical protein MA0159 [Methanosarcina acetivorans C2A]
>gi|20089065|ref|NP_615140.1| hypothetical protein MA0167 [Methanosarcina acetivorans C2A]
>gi|20089094|ref|NP_615169.1| hypothetical protein MA0196 [Methanosarcina acetivorans C2A]
>gi|20089172|ref|NP_615247.1| tetrahydromethanopterin S-methyltransferase, subunit C [Methanosarcina acetivorans C2A]
>gi|20089173|ref|NP_615248.1| tetrahydromethanopterin S-methyltransferase, subunit D [Methanosarcina acetivorans C2A]
>gi|20089194|ref|NP_615269.1| hypothetical protein MA0296 [Methanosarcina acetivorans C2A]
>gi|20089210|ref|NP_615285.1| hypothetical protein MA0312 [Methanosarcina acetivorans C2A]
>gi|20089235|ref|NP_615310.1| hypothetical protein MA0337 [Methanosarcina acetivorans C2A]
>gi|20089236|ref|NP_615311.1| hypothetical protein MA0338 [Methanosarcina acetivorans C2A]
>gi|20089287|ref|NP_615362.1| hypothetical protein MA0390 [Methanosarcina acetivorans C2A]
>gi|20089323|ref|NP_615398.1| ferredoxin [Methanosarcina acetivorans C2A]
>gi|20089327|ref|NP_615402.1| hypothetical protein MA0435 [Methanosarcina acetivorans C2A]
>gi|20089334|ref|NP_615409.1| hypothetical protein MA0443 [Methanosarcina acetivorans C2A]
>gi|20089349|ref|NP_615424.1| hypothetical protein MA0458 [Methanosarcina acetivorans C2A]
>gi|20089354|ref|NP_615429.1| ferredoxin [Methanosarcina acetivorans C2A]
>gi|20089398|ref|NP_615473.1| surface antigen gene [Methanosarcina acetivorans C2A]
>gi|20089413|ref|NP_615488.1| hypothetical protein MA0524 [Methanosarcina acetivorans C2A]
>gi|20089422|ref|NP_615497.1| hypothetical protein MA0533 [Methanosarcina acetivorans C2A]
>gi|20089425|ref|NP_615500.1| hypothetical protein MA0536 [Methanosarcina acetivorans C2A]
>gi|20089429|ref|NP_615504.1| hypothetical protein MA0540 [Methanosarcina acetivorans C2A]
>gi|20089469|ref|NP_615544.1| hypothetical protein MA0580 [Methanosarcina acetivorans C2A]
>gi|20089477|ref|NP_615552.1| cell surface protein [Methanosarcina acetivorans C2A]
>gi|20089480|ref|NP_615555.1| hypothetical protein MA0591 [Methanosarcina acetivorans C2A]
>gi|20089496|ref|NP_615571.1| hypothetical protein MA0607 [Methanosarcina acetivorans C2A]
>gi|20089515|ref|NP_615590.1| antigen [Methanosarcina acetivorans C2A]
>gi|20089524|ref|NP_615599.1| S-layer-like protein [Methanosarcina acetivorans C2A]
>gi|20089536|ref|NP_615611.1| hypothetical protein MA0649 [Methanosarcina acetivorans C2A]
>gi|20089541|ref|NP_615616.1| hypothetical protein MA0654 [Methanosarcina acetivorans C2A]
>gi|20089587|ref|NP_615662.1| hypothetical protein MA0702 [Methanosarcina acetivorans C2A]
>gi|20089605|ref|NP_615680.1| hypothetical protein MA0720 [Methanosarcina acetivorans C2A]
>gi|20089622|ref|NP_615697.1| hypothetical protein MA0737 [Methanosarcina acetivorans C2A]
>gi|20089627|ref|NP_615702.1| hypothetical protein MA0742 [Methanosarcina acetivorans C2A]
>gi|20089641|ref|NP_615716.1| hypothetical protein MA0756 [Methanosarcina acetivorans C2A]
>gi|20089665|ref|NP_615740.1| hypothetical protein MA0781 [Methanosarcina acetivorans C2A]
>gi|20089669|ref|NP_615744.1| proteophosphoglycan [Methanosarcina acetivorans C2A]
>gi|20089696|ref|NP_615771.1| hypothetical protein MA0812 [Methanosarcina acetivorans C2A]
>gi|20089709|ref|NP_615784.1| hypothetical protein MA0825 [Methanosarcina acetivorans C2A]
>gi|20089732|ref|NP_615807.1| hypothetical protein MA0848 [Methanosarcina acetivorans C2A]
>gi|20089770|ref|NP_615845.1| hypothetical protein MA0886 [Methanosarcina acetivorans C2A]
>gi|20089781|ref|NP_615856.1| hypothetical protein MA0902 [Methanosarcina acetivorans C2A]
>gi|20089788|ref|NP_615863.1| hypothetical protein MA0910 [Methanosarcina acetivorans C2A]
>gi|20089838|ref|NP_615913.1| hypothetical protein MA0960 [Methanosarcina acetivorans C2A]
>gi|20089839|ref|NP_615914.1| 4-hydroxybenzoate octaprenyltransferase [Methanosarcina acetivorans C2A]
>gi|20089862|ref|NP_615937.1| hypothetical protein MA0985 [Methanosarcina acetivorans C2A]
>gi|20089939|ref|NP_616014.1| hypothetical protein MA1069 [Methanosarcina acetivorans C2A]
>gi|20089948|ref|NP_616023.1| ribosomal protein S3p [Methanosarcina acetivorans C2A]
>gi|20089960|ref|NP_616035.1| ribosomal protein L19e [Methanosarcina acetivorans C2A]
>gi|20090011|ref|NP_616086.1| hypothetical protein MA1145 [Methanosarcina acetivorans C2A]
>gi|20090020|ref|NP_616095.1| hypothetical protein MA1154 [Methanosarcina acetivorans C2A]
>gi|20090030|ref|NP_616105.1| purine NTPase [Methanosarcina acetivorans C2A]
>gi|20090032|ref|NP_616107.1| hypothetical protein MA1166 [Methanosarcina acetivorans C2A]
>gi|20090042|ref|NP_616117.1| polysaccharide ABC transporter, permease protein [Methanosarcina acetivorans C2A]
>gi|20090150|ref|NP_616225.1| cell surface glycoprotein (S-layer protein) [Methanosarcina acetivorans C2A]
>gi|20090164|ref|NP_616239.1| hypothetical protein MA1300 [Methanosarcina acetivorans C2A]
>gi|20090165|ref|NP_616240.1| hypothetical protein MA1301 [Methanosarcina acetivorans C2A]
>gi|20090196|ref|NP_616271.1| hypothetical protein MA1335 [Methanosarcina acetivorans C2A]
>gi|20090210|ref|NP_616285.1| hypothetical protein MA1349 [Methanosarcina acetivorans C2A]
>gi|20090217|ref|NP_616292.1| acidic integral membrane protein [Methanosarcina acetivorans C2A]
>gi|20090219|ref|NP_616294.1| copper binding protein, plastocyanin/azurin family [Methanosarcina acetivorans C2A]

>gi|20090262|ref|NP_616337.1| hypothetical protein MA1402 [Methanosarcina acetivorans C2A]
>gi|20090267|ref|NP_616342.1| hypothetical protein MA1407 [Methanosarcina acetivorans C2A]
>gi|20090270|ref|NP_616345.1| hypothetical protein MA1410 [Methanosarcina acetivorans C2A]
>gi|20090275|ref|NP_616350.1| hypothetical protein MA1415 [Methanosarcina acetivorans C2A]
>gi|20090277|ref|NP_616352.1| hypothetical protein MA1417 [Methanosarcina acetivorans C2A]
>gi|20090289|ref|NP_616364.1| heme exporter protein B [Methanosarcina acetivorans C2A]
>gi|20090302|ref|NP_616377.1| hypothetical protein MA1443 [Methanosarcina acetivorans C2A]
>gi|20090313|ref|NP_616388.1| cell surface lipoprotein [Methanosarcina acetivorans C2A]
>gi|20090315|ref|NP_616390.1| hypothetical protein MA1456 [Methanosarcina acetivorans C2A]
>gi|20090318|ref|NP_616393.1| hypothetical protein MA1459 [Methanosarcina acetivorans C2A]
>gi|20090367|ref|NP_616442.1| cell surface protein [Methanosarcina acetivorans C2A]
>gi|20090411|ref|NP_616486.1| hypothetical protein MA1553 [Methanosarcina acetivorans C2A]
>gi|20090443|ref|NP_616518.1| hypothetical protein MA1585 [Methanosarcina acetivorans C2A]
>gi|20090448|ref|NP_616523.1| surface antigen gene [Methanosarcina acetivorans C2A]
>gi|20090467|ref|NP_616542.1| hypothetical protein MA1609 [Methanosarcina acetivorans C2A]
>gi|20090515|ref|NP_616590.1| hypothetical protein MA1662 [Methanosarcina acetivorans C2A]
>gi|20090533|ref|NP_616608.1| hypothetical protein MA1681 [Methanosarcina acetivorans C2A]
>gi|20090541|ref|NP_616616.1| hypothetical protein MA1689 [Methanosarcina acetivorans C2A]
>gi|20090569|ref|NP_616644.1| cell surface protein [Methanosarcina acetivorans C2A]
>gi|20090574|ref|NP_616649.1| hypothetical protein MA1722 [Methanosarcina acetivorans C2A]
>gi|20090575|ref|NP_616650.1| hypothetical protein MA1723 [Methanosarcina acetivorans C2A]
>gi|20090580|ref|NP_616655.1| STARP antigen [Methanosarcina acetivorans C2A]
>gi|20090584|ref|NP_616659.1| hypothetical protein MA1732 [Methanosarcina acetivorans C2A]
>gi|20090586|ref|NP_616661.1| hypothetical protein MA1734 [Methanosarcina acetivorans C2A]
>gi|20090616|ref|NP_616691.1| hypothetical protein MA1765 [Methanosarcina acetivorans C2A]
>gi|20090646|ref|NP_616721.1| hypothetical protein MA1795 [Methanosarcina acetivorans C2A]
>gi|20090674|ref|NP_616749.1| hypothetical protein MA1823 [Methanosarcina acetivorans C2A]
>gi|20090695|ref|NP_616770.1| hypothetical protein MA1845 [Methanosarcina acetivorans C2A]
>gi|20090705|ref|NP_616780.1| hypothetical protein MA1855 [Methanosarcina acetivorans C2A]
>gi|20090726|ref|NP_616801.1| hypothetical protein MA1876 [Methanosarcina acetivorans C2A]
>gi|20090742|ref|NP_616817.1| hypothetical protein MA1892 [Methanosarcina acetivorans C2A]
>gi|20090744|ref|NP_616819.1| hypothetical protein MA1894 [Methanosarcina acetivorans C2A]
>gi|20090776|ref|NP_616851.1| hypothetical protein MA1927 [Methanosarcina acetivorans C2A]
>gi|20090792|ref|NP_616867.1| hypothetical protein MA1943 [Methanosarcina acetivorans C2A]
>gi|20090800|ref|NP_616875.1| hypothetical protein MA1952 [Methanosarcina acetivorans C2A]
>gi|20090808|ref|NP_616883.1| hypothetical protein MA1960 [Methanosarcina acetivorans C2A]
>gi|20090831|ref|NP_616906.1| hypothetical protein MA1983 [Methanosarcina acetivorans C2A]
>gi|20090844|ref|NP_616919.1| hypothetical protein MA1996 [Methanosarcina acetivorans C2A]
>gi|20090845|ref|NP_616920.1| hypothetical protein MA1997 [Methanosarcina acetivorans C2A]
>gi|20090856|ref|NP_616931.1| sodium/calcium exchanger protein [Methanosarcina acetivorans C2A]
>gi|20090867|ref|NP_616942.1| pneumococcal surface protein [Methanosarcina acetivorans C2A]
>gi|20090945|ref|NP_617020.1| hypothetical protein MA2101 [Methanosarcina acetivorans C2A]
>gi|20090960|ref|NP_617035.1| hypothetical protein MA2117 [Methanosarcina acetivorans C2A]
>gi|20091016|ref|NP_617091.1| galactoside O-acetyltransferase [Methanosarcina acetivorans C2A]
>gi|20091044|ref|NP_617119.1| cellulosomal protein [Methanosarcina acetivorans C2A]
>gi|20091058|ref|NP_617133.1| hypothetical protein MA2217 [Methanosarcina acetivorans C2A]
>gi|20091069|ref|NP_617144.1| hypothetical protein MA2229 [Methanosarcina acetivorans C2A]
>gi|20091073|ref|NP_617148.1| hypothetical protein MA2233 [Methanosarcina acetivorans C2A]
>gi|20091074|ref|NP_617149.1| hypothetical protein MA2234 [Methanosarcina acetivorans C2A]
>gi|20091141|ref|NP_617216.1| hypothetical protein MA2303 [Methanosarcina acetivorans C2A]
>gi|20091187|ref|NP_617262.1| hypothetical protein MA2353 [Methanosarcina acetivorans C2A]
>gi|20091188|ref|NP_617263.1| hypothetical protein MA2354 [Methanosarcina acetivorans C2A]
>gi|20091206|ref|NP_617281.1| hypothetical protein MA2374 [Methanosarcina acetivorans C2A]
>gi|20091236|ref|NP_617311.1| hypothetical protein MA2405 [Methanosarcina acetivorans C2A]
>gi|20091242|ref|NP_617317.1| hypothetical protein MA2411 [Methanosarcina acetivorans C2A]
>gi|20091263|ref|NP_617338.1| hypothetical protein MA2432 [Methanosarcina acetivorans C2A]
>gi|20091266|ref|NP_617341.1| H(+)-transporting ATP synthase, subunit B [Methanosarcina acetivorans C2A]
>gi|20091331|ref|NP_617406.1| hypothetical protein MA2500 [Methanosarcina acetivorans C2A]
>gi|20091337|ref|NP_617412.1| hypothetical protein MA2506 [Methanosarcina acetivorans C2A]
>gi|20091355|ref|NP_617430.1| hypothetical protein MA2527 [Methanosarcina acetivorans C2A]
>gi|20091362|ref|NP_617437.1| hypothetical protein MA2534 [Methanosarcina acetivorans C2A]
>gi|20091369|ref|NP_617444.1| hypothetical protein MA2542 [Methanosarcina acetivorans C2A]
>gi|20091373|ref|NP_617448.1| hypothetical protein MA2546 [Methanosarcina acetivorans C2A]
>gi|20091400|ref|NP_617475.1| hypothetical protein MA2574 [Methanosarcina acetivorans C2A]
>gi|20091410|ref|NP_617485.1| hypothetical protein MA2584 [Methanosarcina acetivorans C2A]
>gi|20091455|ref|NP_617530.1| transmembrane transport protein [Methanosarcina acetivorans C2A]
>gi|20091459|ref|NP_617534.1| hypothetical protein MA2636 [Methanosarcina acetivorans C2A]
>gi|20091462|ref|NP_617537.1| hypothetical protein MA2639 [Methanosarcina acetivorans C2A]
>gi|20091464|ref|NP_617539.1| hypothetical protein MA2641 [Methanosarcina acetivorans C2A]
>gi|20091469|ref|NP_617544.1| transmembrane protein [Methanosarcina acetivorans C2A]

>gi|20091475|ref|NP_617550.1| hypothetical protein MA2652 [Methanosarcina acetivorans C2A]
>gi|20091516|ref|NP_617591.1| transposase [Methanosarcina acetivorans C2A]
>gi|20091582|ref|NP_617657.1| hypothetical protein MA2759 [Methanosarcina acetivorans C2A]
>gi|20091590|ref|NP_617665.1| hypothetical protein MA2767 [Methanosarcina acetivorans C2A]
>gi|20091597|ref|NP_617672.1| iron ABC transporter, permease [Methanosarcina acetivorans C2A]
>gi|20091598|ref|NP_617673.1| hypothetical protein MA2775 [Methanosarcina acetivorans C2A]
>gi|20091612|ref|NP_617687.1| hypothetical protein MA2789 [Methanosarcina acetivorans C2A]
>gi|20091634|ref|NP_617709.1| hypothetical protein MA2810 [Methanosarcina acetivorans C2A]
>gi|20091641|ref|NP_617716.1| sodium/calcium exchanger protein [Methanosarcina acetivorans C2A]
>gi|20091670|ref|NP_617745.1| hypothetical protein MA4676 [Methanosarcina acetivorans C2A]
>gi|20091680|ref|NP_617755.1| hypothetical protein MA2856 [Methanosarcina acetivorans C2A]
>gi|20091693|ref|NP_617768.1| albumin-binding protein [Methanosarcina acetivorans C2A]
>gi|20091707|ref|NP_617782.1| cell surface protein [Methanosarcina acetivorans C2A]
>gi|20091708|ref|NP_617783.1| cell surface protein [Methanosarcina acetivorans C2A]
>gi|20091709|ref|NP_617784.1| hypothetical protein MA2887 [Methanosarcina acetivorans C2A]
>gi|20091719|ref|NP_617794.1| hypothetical protein MA2898 [Methanosarcina acetivorans C2A]
>gi|20091734|ref|NP_617809.1| hypothetical protein MA2913 [Methanosarcina acetivorans C2A]
>gi|20091741|ref|NP_617816.1| transcriptional regulator, ArsR family [Methanosarcina acetivorans C2A]
>gi|20091745|ref|NP_617820.1| cell surface protein [Methanosarcina acetivorans C2A]
>gi|20091748|ref|NP_617823.1| hypothetical protein MA2929 [Methanosarcina acetivorans C2A]
>gi|20091792|ref|NP_617867.1| hypothetical protein MA2974 [Methanosarcina acetivorans C2A]
>gi|20091819|ref|NP_617894.1| hypothetical protein MA3001 [Methanosarcina acetivorans C2A]
>gi|20091829|ref|NP_617904.1| hypothetical protein MA3011 [Methanosarcina acetivorans C2A]
>gi|20091854|ref|NP_617929.1| hypothetical protein MA3036 [Methanosarcina acetivorans C2A]
>gi|20091855|ref|NP_617930.1| prion protein precursor [Methanosarcina acetivorans C2A]
>gi|20091872|ref|NP_617947.1| hypothetical protein MA3054 [Methanosarcina acetivorans C2A]
>gi|20091892|ref|NP_617967.1| hypothetical protein MA3074 [Methanosarcina acetivorans C2A]
>gi|20091924|ref|NP_617999.1| hypothetical protein MA3106 [Methanosarcina acetivorans C2A]
>gi|20091937|ref|NP_618012.1| hypothetical protein MA3119 [Methanosarcina acetivorans C2A]
>gi|20091940|ref|NP_618015.1| surface antigen gene [Methanosarcina acetivorans C2A]
>gi|20091952|ref|NP_618027.1| hypothetical protein MA3134 [Methanosarcina acetivorans C2A]
>gi|20092048|ref|NP_618123.1| hypothetical protein MA3232 [Methanosarcina acetivorans C2A]
>gi|20092056|ref|NP_618131.1| hypothetical protein MA3240 [Methanosarcina acetivorans C2A]
>gi|20092095|ref|NP_618170.1| hypothetical protein MA3280 [Methanosarcina acetivorans C2A]
>gi|20092118|ref|NP_618193.1| hypothetical protein MA3303 [Methanosarcina acetivorans C2A]
>gi|20092120|ref|NP_618195.1| hypothetical protein MA3305 [Methanosarcina acetivorans C2A]
>gi|20092132|ref|NP_618207.1| hypothetical protein MA3318 [Methanosarcina acetivorans C2A]
>gi|20092142|ref|NP_618217.1| hypothetical protein MA3328 [Methanosarcina acetivorans C2A]
>gi|20092151|ref|NP_618226.1| hypothetical protein MA3337 [Methanosarcina acetivorans C2A]
>gi|20092164|ref|NP_618239.1| hypothetical protein MA3350 [Methanosarcina acetivorans C2A]
>gi|20092185|ref|NP_618260.1| hypothetical protein MA3371 [Methanosarcina acetivorans C2A]
>gi|20092194|ref|NP_618269.1| hypothetical protein MA3380 [Methanosarcina acetivorans C2A]
>gi|20092207|ref|NP_618282.1| hypothetical protein MA3395 [Methanosarcina acetivorans C2A]
>gi|20092215|ref|NP_618290.1| hypothetical protein MA3403 [Methanosarcina acetivorans C2A]
>gi|20092249|ref|NP_618324.1| branched chain amino acid transport protein AzlC [Methanosarcina acetivorans C2A]
>gi|20092252|ref|NP_618327.1| hypothetical protein MA3440 [Methanosarcina acetivorans C2A]
>gi|20092287|ref|NP_618362.1| hypothetical protein MA3476 [Methanosarcina acetivorans C2A]
>gi|20092297|ref|NP_618372.1| mttA/Hcf106 family protein [Methanosarcina acetivorans C2A]
>gi|20092313|ref|NP_618388.1| hypothetical protein MA3503 [Methanosarcina acetivorans C2A]
>gi|20092357|ref|NP_618432.1| hypothetical protein MA3550 [Methanosarcina acetivorans C2A]
>gi|20092377|ref|NP_618452.1| hypothetical protein MA3571 [Methanosarcina acetivorans C2A]
>gi|20092383|ref|NP_618458.1| hypothetical protein MA3579 [Methanosarcina acetivorans C2A]
>gi|20092418|ref|NP_618493.1| hypothetical protein MA3618 [Methanosarcina acetivorans C2A]
>gi|20092449|ref|NP_618524.1| hypothetical protein MA3649 [Methanosarcina acetivorans C2A]
>gi|20092497|ref|NP_618572.1| hypothetical protein MA3697 [Methanosarcina acetivorans C2A]
>gi|20092581|ref|NP_618656.1| hypothetical protein MA3785 [Methanosarcina acetivorans C2A]
>gi|20092601|ref|NP_618676.1| hypothetical protein MA3805 [Methanosarcina acetivorans C2A]
>gi|20092607|ref|NP_618682.1| hypothetical protein MA3811 [Methanosarcina acetivorans C2A]
>gi|20092626|ref|NP_618701.1| hypothetical protein MA3830 [Methanosarcina acetivorans C2A]
>gi|20092646|ref|NP_618721.1| prefoldin, subunit beta [Methanosarcina acetivorans C2A]
>gi|20092776|ref|NP_618851.1| hypothetical protein MA3982 [Methanosarcina acetivorans C2A]
>gi|20092805|ref|NP_618880.1| UbiA prenyltransferase family protein [Methanosarcina acetivorans C2A]
>gi|20092813|ref|NP_618888.1| cobalt transport protein [Methanosarcina acetivorans C2A]
>gi|20092818|ref|NP_618893.1| hypothetical protein MA4024 [Methanosarcina acetivorans C2A]
>gi|20092823|ref|NP_618898.1| hypothetical protein MA4030 [Methanosarcina acetivorans C2A]
>gi|20092826|ref|NP_618901.1| surface antigen gene [Methanosarcina acetivorans C2A]
>gi|20092831|ref|NP_618906.1| hypothetical protein MA4038 [Methanosarcina acetivorans C2A]
>gi|20092849|ref|NP_618924.1| hypothetical protein MA4056 [Methanosarcina acetivorans C2A]
>gi|20092860|ref|NP_618935.1| hypothetical protein MA4067 [Methanosarcina acetivorans C2A]
>gi|20092877|ref|NP_618952.1| hypothetical protein MA4084 [Methanosarcina acetivorans C2A]

>gi|20092902|ref|NP_618977.1| signal recognition particle receptor [Methanosarcina acetivorans C2A]
>gi|20092909|ref|NP_618984.1| hypothetical protein MA4116 [Methanosarcina acetivorans C2A]
>gi|20092913|ref|NP_618988.1| hypothetical protein MA4120 [Methanosarcina acetivorans C2A]
>gi|20092939|ref|NP_619014.1| hypothetical protein MA4146 [Methanosarcina acetivorans C2A]
>gi|20092956|ref|NP_619031.1| hypothetical protein MA4163 [Methanosarcina acetivorans C2A]
>gi|20092985|ref|NP_619060.1| ferredoxin [Methanosarcina acetivorans C2A]
>gi|20093004|ref|NP_619079.1| benzodiazepine receptor TspO [Methanosarcina acetivorans C2A]
>gi|20093012|ref|NP_619087.1| cell surface protein [Methanosarcina acetivorans C2A]
>gi|20093045|ref|NP_619120.1| c-type cytochrome biogenesis protein [Methanosarcina acetivorans C2A]
>gi|20093065|ref|NP_619140.1| acidic ribosomal protein P0 homolog [Methanosarcina acetivorans C2A]
>gi|20093066|ref|NP_619141.1| ribosomal protein L12p [Methanosarcina acetivorans C2A]
>gi|20093087|ref|NP_619162.1| hypothetical protein MA4298 [Methanosarcina acetivorans C2A]
>gi|20093128|ref|NP_619203.1| biotin synthesis BioY protein [Methanosarcina acetivorans C2A]
>gi|20093134|ref|NP_619209.1| hypothetical protein MA4346 [Methanosarcina acetivorans C2A]
>gi|20093151|ref|NP_619226.1| corrinoid protein [Methanosarcina acetivorans C2A]
>gi|20093168|ref|NP_619243.1| hypothetical protein MA4381 [Methanosarcina acetivorans C2A]
>gi|20093199|ref|NP_619274.1| hypothetical protein MA4412 [Methanosarcina acetivorans C2A]
>gi|20093233|ref|NP_619308.1| hypothetical protein MA4447 [Methanosarcina acetivorans C2A]
>gi|20093244|ref|NP_619319.1| hypothetical protein MA4458 [Methanosarcina acetivorans C2A]
>gi|20093251|ref|NP_619326.1| hypothetical protein MA4465 [Methanosarcina acetivorans C2A]
>gi|20093266|ref|NP_619341.1| cell surface protein [Methanosarcina acetivorans C2A]
>gi|20093276|ref|NP_619351.1| hypothetical protein MA4491 [Methanosarcina acetivorans C2A]
>gi|20093285|ref|NP_619360.1| hypothetical protein MA4500 [Methanosarcina acetivorans C2A]
>gi|20093313|ref|NP_619388.1| hypothetical protein MA4528 [Methanosarcina acetivorans C2A]
>gi|20093316|ref|NP_619391.1| hypothetical protein MA4531 [Methanosarcina acetivorans C2A]
>gi|20093352|ref|NP_619427.1| multiple resistance/pH regulation related protein E (Na+/H+ antiporter) [Methanosarcina acetivorans C2A]
>gi|20093361|ref|NP_619436.1| cell surface protein [Methanosarcina acetivorans C2A]
>gi|20093412|ref|NP_619487.1| acetyltransferase (GNAT) family protein [Methanosarcina acetivorans C2A]
>gi|20093414|ref|NP_619489.1| hypothetical protein MA4635 [Methanosarcina acetivorans C2A]
>gi|20093436|ref|NP_619511.1| hypothetical protein MA4657 [Methanosarcina acetivorans C2A]
>gi|56694933|ref|YP_165278.1| hypothetical protein SPO0005 [Silicibacter pomeroyi DSS-3]
>gi|56694938|ref|YP_165283.1| co-chaperone GrpE [Silicibacter pomeroyi DSS-3]
>gi|56694947|ref|YP_165292.1| membrane protein, putative [Silicibacter pomeroyi DSS-3]
>gi|56694954|ref|YP_165299.1| polyA polymerase family protein [Silicibacter pomeroyi DSS-3]
>gi|56694975|ref|YP_165321.1| thiazole biosynthesis protein ThiG [Silicibacter pomeroyi DSS-3]
>gi|56694989|ref|YP_165335.1| translation initiation factor IF-2 [Silicibacter pomeroyi DSS-3]
>gi|56694993|ref|YP_165339.1| peptide/opine/nickel uptake family ABC transporter, periplasmic substrate-binding protein [Silicibacter pomeroyi DSS-3]
>gi|56695017|ref|YP_165363.1| hypothetical protein SPO0089 [Silicibacter pomeroyi DSS-3]
>gi|56695021|ref|YP_165366.1| heat shock protein, Hsp70 family [Silicibacter pomeroyi DSS-3]
>gi|56695024|ref|YP_165370.1| membrane protein, putative [Silicibacter pomeroyi DSS-3]
>gi|56695054|ref|YP_165401.1| peptidase, T4 family [Silicibacter pomeroyi DSS-3]
>gi|56695096|ref|YP_165443.1| hypothetical protein SPO0173 [Silicibacter pomeroyi DSS-3]
>gi|56695097|ref|YP_165444.1| hypothetical protein SPO0174 [Silicibacter pomeroyi DSS-3]
>gi|56695102|ref|YP_165449.1| flagellar biosynthetic protein FliQ [Silicibacter pomeroyi DSS-3]
>gi|56695108|ref|YP_165455.1| membrane protein, putative [Silicibacter pomeroyi DSS-3]
>gi|56695125|ref|YP_165472.1| hypothetical protein SPO0202 [Silicibacter pomeroyi DSS-3]
>gi|56695131|ref|YP_165478.1| Na/Pi-cotransporter family protein [Silicibacter pomeroyi DSS-3]
>gi|56695144|ref|YP_165491.1| alanine dehydrogenase [Silicibacter pomeroyi DSS-3]
>gi|56695149|ref|YP_165496.1| PaxA, putative [Silicibacter pomeroyi DSS-3]
>gi|56695151|ref|YP_165498.1| ribosomal protein S21 [Silicibacter pomeroyi DSS-3]
>gi|56695152|ref|YP_165499.1| hypothetical protein SPO0230 [Silicibacter pomeroyi DSS-3]
>gi|56695165|ref|YP_165512.1| farnesyl diphosphate synthase [Silicibacter pomeroyi DSS-3]
>gi|56695178|ref|YP_165525.1| membrane protein, putative [Silicibacter pomeroyi DSS-3]
>gi|56695181|ref|YP_165528.1| CaiB/BaiF family protein [Silicibacter pomeroyi DSS-3]
>gi|56695182|ref|YP_165529.1| auxin efflux carrier family protein [Silicibacter pomeroyi DSS-3]
>gi|56695191|ref|YP_165538.1| hypothetical protein SPO0274 [Silicibacter pomeroyi DSS-3]
>gi|56695198|ref|YP_165546.1| conserved hypothetical protein TIGR00023 [Silicibacter pomeroyi DSS-3]
>gi|56695200|ref|YP_165548.1| hypothetical protein SPO0285 [Silicibacter pomeroyi DSS-3]
>gi|56695203|ref|YP_165551.1| uracil-DNA glycosylase [Silicibacter pomeroyi DSS-3]
>gi|56695205|ref|YP_165553.1| transmembrane amino acid efflux protein [Silicibacter pomeroyi DSS-3]
>gi|56695207|ref|YP_165555.1| efflux transporter, RND family, MFP subunit [Silicibacter pomeroyi DSS-3]
>gi|56695216|ref|YP_165564.1| glycerophosphoryl diester phosphodiesterase family protein [Silicibacter pomeroyi DSS-3]
>gi|56695222|ref|YP_165570.1| molybdopterin-guanine dinucleotide biosynthesis protein A [Silicibacter pomeroyi DSS-3]
>gi|56695226|ref|YP_165574.1| hypothetical protein SPO0311 [Silicibacter pomeroyi DSS-3]
>gi|56695238|ref|YP_165586.1| membrane protein, putative [Silicibacter pomeroyi DSS-3]
>gi|56695251|ref|YP_165599.1| MJ0042 family finger-like domain protein [Silicibacter pomeroyi DSS-3]
>gi|56695253|ref|YP_165601.1| cell division permease protein FtsX, putative [Silicibacter pomeroyi DSS-3]
>gi|56695258|ref|YP_165606.1| 2-oxoglutarate dehydrogenase, E2 component, dihydrolipoamide succinyltransferase [Silicibacter pomeroyi DSS-3]
>gi|56695260|ref|YP_165608.1| hypothetical protein SPO0345 [Silicibacter pomeroyi DSS-3]

>gi|56695265|ref|YP_165613.1| membrane protein, putative [Silicibacter pomeroyi DSS-3]
>gi|56695282|ref|YP_165630.1| DNA-binding protein, H-NS family [Silicibacter pomeroyi DSS-3]
>gi|56695296|ref|YP_165644.1| protease, putative [Silicibacter pomeroyi DSS-3]
>gi|56695302|ref|YP_165650.1| membrane protein, putative [Silicibacter pomeroyi DSS-3]
>gi|56695308|ref|YP_165656.1| rhomboid family protein [Silicibacter pomeroyi DSS-3]
>gi|56695309|ref|YP_165657.1| hypothetical protein SPO0394 [Silicibacter pomeroyi DSS-3]
>gi|56695317|ref|YP_165665.1| competence protein ComM [Silicibacter pomeroyi DSS-3]
>gi|56695322|ref|YP_165670.1| hypothetical protein SPO0407 [Silicibacter pomeroyi DSS-3]
>gi|56695333|ref|YP_165681.1| membrane protein, putative [Silicibacter pomeroyi DSS-3]
>gi|56695348|ref|YP_165696.1| ABC transporter, permease protein [Silicibacter pomeroyi DSS-3]
>gi|56695349|ref|YP_165698.1| acyl-CoA thioesterase, putative [Silicibacter pomeroyi DSS-3]
>gi|56695365|ref|YP_165713.1| membrane protein, putative [Silicibacter pomeroyi DSS-3]
>gi|56695379|ref|YP_165727.1| hypothetical protein SPO0464 [Silicibacter pomeroyi DSS-3]
>gi|56695381|ref|YP_165729.1| transporter, formate/nitrate family [Silicibacter pomeroyi DSS-3]
>gi|56695383|ref|YP_165731.1| alkylphosphonate utilization protein PhnG [Silicibacter pomeroyi DSS-3]
>gi|56695389|ref|YP_165737.1| alkylphosphonate utilization protein PhnN [Silicibacter pomeroyi DSS-3]
>gi|56695392|ref|YP_165740.1| hypothetical protein SPO0477 [Silicibacter pomeroyi DSS-3]
>gi|56695439|ref|YP_165787.1| sterol desaturase, homolog [Silicibacter pomeroyi DSS-3]
>gi|56695449|ref|YP_165797.1| K+-dependent Na+/Ca+ exchanger related-protein [Silicibacter pomeroyi DSS-3]
>gi|56695491|ref|YP_165839.1| acetyl-CoA carboxylase, biotin carboxylase, putative [Silicibacter pomeroyi DSS-3]
>gi|56695492|ref|YP_165840.1| oxidoreductase, 2-nitropropane dioxygenase family [Silicibacter pomeroyi DSS-3]
>gi|56695509|ref|YP_165857.1| universal stress family protein [Silicibacter pomeroyi DSS-3]
>gi|56695525|ref|YP_165873.1| hypothetical protein SPO0613 [Silicibacter pomeroyi DSS-3]
>gi|56695527|ref|YP_165876.1| oxidoreductase, zinc-binding dehydrogenase family [Silicibacter pomeroyi DSS-3]
>gi|56695528|ref|YP_165877.1| transcriptional regulator, AraC family [Silicibacter pomeroyi DSS-3]
>gi|56695554|ref|YP_165904.1| sugar ABC transporter, permease protein [Silicibacter pomeroyi DSS-3]
>gi|56695560|ref|YP_165910.1| membrane protein, putative [Silicibacter pomeroyi DSS-3]
>gi|56695586|ref|YP_165936.1| hypothetical protein SPO0681 [Silicibacter pomeroyi DSS-3]
>gi|56695598|ref|YP_165949.1| hypothetical protein SPO0694 [Silicibacter pomeroyi DSS-3]
>gi|56695600|ref|YP_165952.1| molybdate ABC transporter, permease protein [Silicibacter pomeroyi DSS-3]
>gi|56695622|ref|YP_165973.1| electron transfer flavoprotein, alpha subunit [Silicibacter pomeroyi DSS-3]
>gi|56695648|ref|YP_165999.1| hypothetical protein SPO0746 [Silicibacter pomeroyi DSS-3]
>gi|56695679|ref|YP_166030.1| enoyl-CoA hydratase/isomerase family protein [Silicibacter pomeroyi DSS-3]
>gi|56695692|ref|YP_166043.1| hypothetical protein SPO0790 [Silicibacter pomeroyi DSS-3]
>gi|56695707|ref|YP_166058.1| hypothetical protein SPO0805 [Silicibacter pomeroyi DSS-3]
>gi|56695720|ref|YP_166071.1| MAPEG family protein [Silicibacter pomeroyi DSS-3]
>gi|56695726|ref|YP_166077.1| branched-chain amino acid ABC transporter, permease protein [Silicibacter pomeroyi DSS-3]
>gi|56695730|ref|YP_166081.1| hypothetical protein SPO0828 [Silicibacter pomeroyi DSS-3]
>gi|56695743|ref|YP_166094.1| polysaccharide biosynthesis protein [Silicibacter pomeroyi DSS-3]
>gi|56695745|ref|YP_166096.1| glycosyl transferase, group 2 family protein [Silicibacter pomeroyi DSS-3]
>gi|56695748|ref|YP_166099.1| phosphopantetheinyl transferase PptA, putative [Silicibacter pomeroyi DSS-3]
>gi|56695769|ref|YP_166120.1| hypothetical protein SPO0867 [Silicibacter pomeroyi DSS-3]
>gi|56695786|ref|YP_166137.1| membrane protein, putative [Silicibacter pomeroyi DSS-3]
>gi|56695791|ref|YP_166142.1| membrane protein, putative [Silicibacter pomeroyi DSS-3]
>gi|56695844|ref|YP_166195.1| hypothetical protein SPO0942 [Silicibacter pomeroyi DSS-3]
>gi|56695847|ref|YP_166198.1| AsmA family protein [Silicibacter pomeroyi DSS-3]
>gi|56695853|ref|YP_166204.1| hypothetical protein SPO0951 [Silicibacter pomeroyi DSS-3]
>gi|56695854|ref|YP_166205.1| hypothetical protein SPO0952 [Silicibacter pomeroyi DSS-3]
>gi|56695858|ref|YP_166209.1| hypothetical protein SPO0956 [Silicibacter pomeroyi DSS-3]
>gi|56695859|ref|YP_166210.1| ABC transporter, permease protein [Silicibacter pomeroyi DSS-3]
>gi|56695861|ref|YP_166212.1| membrane protein, putative [Silicibacter pomeroyi DSS-3]
>gi|56695869|ref|YP_166220.1| phosphate transporter family protein [Silicibacter pomeroyi DSS-3]
>gi|56695873|ref|YP_166224.1| ATP-dependent helicase HrpB [Silicibacter pomeroyi DSS-3]
>gi|56695876|ref|YP_166227.1| hypothetical protein SPO0975 [Silicibacter pomeroyi DSS-3]
>gi|56695883|ref|YP_166234.1| hypothetical protein SPO0982 [Silicibacter pomeroyi DSS-3]
>gi|56695885|ref|YP_166236.1| zinc ABC transporter, permease protein [Silicibacter pomeroyi DSS-3]
>gi|56695888|ref|YP_166239.1| zinc ABC transporter, periplasmic zinc-binding protein [Silicibacter pomeroyi DSS-3]
>gi|56695912|ref|YP_166263.1| acetyl-CoA carboxylase, biotin carboxyl carrier protein [Silicibacter pomeroyi DSS-3]
>gi|56695916|ref|YP_166267.1| 3-hydroxybutyrate dehydrogenase, putative [Silicibacter pomeroyi DSS-3]
>gi|56695927|ref|YP_166278.1| hypothetical protein SPO1026 [Silicibacter pomeroyi DSS-3]
>gi|56695952|ref|YP_166306.1| membrane protein, putative [Silicibacter pomeroyi DSS-3]
>gi|56695956|ref|YP_166310.1| serine/threonine protein kinase [Silicibacter pomeroyi DSS-3]
>gi|56695957|ref|YP_166311.1| hypothetical protein SPO1060 [Silicibacter pomeroyi DSS-3]
>gi|56695960|ref|YP_166314.1| hypothetical protein SPO1063 [Silicibacter pomeroyi DSS-3]
>gi|56695962|ref|YP_166316.1| hypothetical protein SPO1065 [Silicibacter pomeroyi DSS-3]
>gi|56695971|ref|YP_166325.1| hypothetical protein SPO1074 [Silicibacter pomeroyi DSS-3]
>gi|56695973|ref|YP_166327.1| trkA-C domain protein [Silicibacter pomeroyi DSS-3]
>gi|56695997|ref|YP_166351.1| lipoprotein, putative [Silicibacter pomeroyi DSS-3]
>gi|56696010|ref|YP_166364.1| TRAP transporter, transmembrane protein, putative [Silicibacter pomeroyi DSS-3]
>gi|56696011|ref|YP_166365.1| TRAP dicarboxylate transporter, DctM subunit [Silicibacter pomeroyi DSS-3]

>gi|56696016|ref|YP_166370.1| membrane protein [Silicibacter pomeroyi DSS-3]
>gi|56696031|ref|YP_166385.1| NnrU family protein [Silicibacter pomeroyi DSS-3]
>gi|56696047|ref|YP_166401.1| hypothetical protein SPO1151 [Silicibacter pomeroyi DSS-3]
>gi|56696068|ref|YP_166422.1| membrane protein, putative [Silicibacter pomeroyi DSS-3]
>gi|56696082|ref|YP_166436.1| membrane protein, putative [Silicibacter pomeroyi DSS-3]
>gi|56696088|ref|YP_166442.1| UDP-N-acetylglucosamine--N-acetylmuramyl-(pentapeptide) pyrophosphoryl-undecaprenol N-acetylglucosamine transferase, putative [Silicibacter pomeroyi DSS-3]
>gi|56696090|ref|YP_166444.1| amino acid permease [Silicibacter pomeroyi DSS-3]
>gi|56696092|ref|YP_166446.1| hypothetical protein SPO1199 [Silicibacter pomeroyi DSS-3]
>gi|56696100|ref|YP_166454.1| DNA repair protein RecN [Silicibacter pomeroyi DSS-3]
>gi|56696112|ref|YP_166467.1| hypothetical protein SPO1220 [Silicibacter pomeroyi DSS-3]
>gi|56696124|ref|YP_166479.1| DNA-binding protein, H-NS family [Silicibacter pomeroyi DSS-3]
>gi|56696129|ref|YP_166485.1| protein-L-isoaspartate O-methyltransferase [Silicibacter pomeroyi DSS-3]
>gi|56696131|ref|YP_166487.1| hypothetical protein SPO1241 [Silicibacter pomeroyi DSS-3]
>gi|56696150|ref|YP_166506.1| transcriptional regulator, MarR family [Silicibacter pomeroyi DSS-3]
>gi|56696151|ref|YP_166507.1| membrane protein, putative [Silicibacter pomeroyi DSS-3]
>gi|56696158|ref|YP_166514.1| hypothetical protein SPO1269 [Silicibacter pomeroyi DSS-3]
>gi|56696170|ref|YP_166526.1| hypothetical protein SPO1281 [Silicibacter pomeroyi DSS-3]
>gi|56696182|ref|YP_166538.1| phasin, PhaP [Silicibacter pomeroyi DSS-3]
>gi|56696194|ref|YP_166551.1| hypothetical protein SPO1308 [Silicibacter pomeroyi DSS-3]
>gi|56696195|ref|YP_166552.1| hypothetical protein SPO1309 [Silicibacter pomeroyi DSS-3]
>gi|56696199|ref|YP_166556.1| preprotein translocase, SecG subunit [Silicibacter pomeroyi DSS-3]
>gi|56696206|ref|YP_166563.1| lipoprotein, putative [Silicibacter pomeroyi DSS-3]
>gi|56696208|ref|YP_166565.1| membrane protein, putative [Silicibacter pomeroyi DSS-3]
>gi|56696212|ref|YP_166569.1| transcriptional regulator, MarR family [Silicibacter pomeroyi DSS-3]
>gi|56696215|ref|YP_166572.1| HflK protein [Silicibacter pomeroyi DSS-3]
>gi|56696218|ref|YP_166575.1| hypothetical protein SPO1332 [Silicibacter pomeroyi DSS-3]
>gi|56696225|ref|YP_166582.1| membrane protein, putative [Silicibacter pomeroyi DSS-3]
>gi|56696226|ref|YP_166583.1| alkane-1 monooxygenase, putative [Silicibacter pomeroyi DSS-3]
>gi|56696229|ref|YP_166586.1| exodeoxyribonuclease VII, large subunit [Silicibacter pomeroyi DSS-3]
>gi|56696242|ref|YP_166599.1| sodium/glutamate symporter [Silicibacter pomeroyi DSS-3]
>gi|56696253|ref|YP_166610.1| transcriptional regulator, AsnC family [Silicibacter pomeroyi DSS-3]
>gi|56696266|ref|YP_166623.1| hypothetical protein SPO1381 [Silicibacter pomeroyi DSS-3]
>gi|56696272|ref|YP_166629.1| cation efflux system protein [Silicibacter pomeroyi DSS-3]
>gi|56696278|ref|YP_166635.1| beta-lactamase, putative [Silicibacter pomeroyi DSS-3]
>gi|56696280|ref|YP_166637.1| transporter, putative [Silicibacter pomeroyi DSS-3]
>gi|56696284|ref|YP_166641.1| hypothetical protein SPO1400 [Silicibacter pomeroyi DSS-3]
>gi|56696290|ref|YP_166647.1| membrane protein, putative [Silicibacter pomeroyi DSS-3]
>gi|56696300|ref|YP_166657.1| TPR domain protein [Silicibacter pomeroyi DSS-3]
>gi|56696306|ref|YP_166663.1| cobalamin 5'-phosphate synthase [Silicibacter pomeroyi DSS-3]
>gi|56696307|ref|YP_166664.1| nicotinate-nucleotide--dimethylbenzimidazole phosphoribosyltransferase [Silicibacter pomeroyi DSS-3]
>gi|56696311|ref|YP_166668.1| hypothetical protein SPO1427 [Silicibacter pomeroyi DSS-3]
>gi|56696312|ref|YP_166669.1| hypothetical protein SPO1428 [Silicibacter pomeroyi DSS-3]
>gi|56696314|ref|YP_166671.1| antibiotic efflux protein [Silicibacter pomeroyi DSS-3]
>gi|56696322|ref|YP_166679.1| hypothetical protein SPO1438 [Silicibacter pomeroyi DSS-3]
>gi|56696324|ref|YP_166681.1| hypothetical protein SPO1440 [Silicibacter pomeroyi DSS-3]
>gi|56696327|ref|YP_166684.1| ATP-dependent RNA helicase RhlE [Silicibacter pomeroyi DSS-3]
>gi|56696333|ref|YP_166690.1| AMP-binding protein [Silicibacter pomeroyi DSS-3]
>gi|56696344|ref|YP_166701.1| indolepyruvate oxidoreductase, IorB subunit, putative [Silicibacter pomeroyi DSS-3]
>gi|56696399|ref|YP_166756.1| cytochrome c550, putative [Silicibacter pomeroyi DSS-3]
>gi|56696413|ref|YP_166770.1| hypothetical protein SPO1529 [Silicibacter pomeroyi DSS-3]
>gi|56696414|ref|YP_166771.1| integral membrane protein MviN [Silicibacter pomeroyi DSS-3]
>gi|56696421|ref|YP_166778.1| twin-arginine translocation pathway signal sequence domain protein, putative [Silicibacter pomeroyi DSS-3]
>gi|56696423|ref|YP_166780.1| O-antigen polymerase, putative [Silicibacter pomeroyi DSS-3]
>gi|56696445|ref|YP_166802.1| membrane protein, putative [Silicibacter pomeroyi DSS-3]
>gi|56696483|ref|YP_166840.1| MmgE/PrpD family protein [Silicibacter pomeroyi DSS-3]
>gi|56696486|ref|YP_166843.1| trimethylamine methyltransferase family protein [Silicibacter pomeroyi DSS-3]
>gi|56696501|ref|YP_166858.1| type I secretion target repeat protein [Silicibacter pomeroyi DSS-3]
>gi|56696502|ref|YP_166859.1| hypothetical protein SPO1618 [Silicibacter pomeroyi DSS-3]
>gi|56696504|ref|YP_166861.1| glyoxalase family protein [Silicibacter pomeroyi DSS-3]
>gi|56696507|ref|YP_166864.1| sensor histidine kinase [Silicibacter pomeroyi DSS-3]
>gi|56696510|ref|YP_166867.1| type I secretion target repeat protein [Silicibacter pomeroyi DSS-3]
>gi|56696513|ref|YP_166870.1| hypothetical protein SPO1629 [Silicibacter pomeroyi DSS-3]
>gi|56696517|ref|YP_166874.1| hypothetical protein SPO1633 [Silicibacter pomeroyi DSS-3]
>gi|56696521|ref|YP_166878.1| single-strand binding protein [Silicibacter pomeroyi DSS-3]
>gi|56696549|ref|YP_166906.1| 1-deoxy-D-xylulose 5-phosphate reductoisomerase [Silicibacter pomeroyi DSS-3]
>gi|56696557|ref|YP_166914.1| lipid-A-disaccharide synthase [Silicibacter pomeroyi DSS-3]
>gi|56696612|ref|YP_166973.1| hypothetical protein SPO1735 [Silicibacter pomeroyi DSS-3]
>gi|56696635|ref|YP_166996.1| hypothetical protein SPO1758 [Silicibacter pomeroyi DSS-3]
>gi|56696642|ref|YP_167003.1| hypothetical protein SPO1765 [Silicibacter pomeroyi DSS-3]

>gi|56696646|ref|YP_167007.1| hypothetical protein SPO1770 [Silicibacter pomeroyi DSS-3]
>gi|56696665|ref|YP_167026.1| sulfate/tungstate uptake family ABC transporter, permease protein [Silicibacter pomeroyi DSS-3]
>gi|56696666|ref|YP_167027.1| sulfate/tungstate uptake family ABC transporter, ATP-binding protein [Silicibacter pomeroyi DSS-3]
>gi|56696673|ref|YP_167034.1| twin-arginine translocation pathway signal sequence domain protein [Silicibacter pomeroyi DSS-3]
>gi|56696675|ref|YP_167036.1| hypothetical protein SPO1799 [Silicibacter pomeroyi DSS-3]
>gi|56696680|ref|YP_167041.1| hypothetical protein SPO1804 [Silicibacter pomeroyi DSS-3]
>gi|56696683|ref|YP_167044.1| exonuclease, DNA polymerase III, epsilon subunit family [Silicibacter pomeroyi DSS-3]
>gi|56696731|ref|YP_167092.1| hypothetical protein SPO1855 [Silicibacter pomeroyi DSS-3]
>gi|56696733|ref|YP_167094.1| hypothetical protein SPO1857 [Silicibacter pomeroyi DSS-3]
>gi|56696742|ref|YP_167103.1| hypothetical protein SPO1866 [Silicibacter pomeroyi DSS-3]
>gi|56696743|ref|YP_167104.1| hypothetical protein SPO1867 [Silicibacter pomeroyi DSS-3]
>gi|56696759|ref|YP_167120.1| hypothetical protein SPO1883 [Silicibacter pomeroyi DSS-3]
>gi|56696761|ref|YP_167122.1| membrane protein, putative [Silicibacter pomeroyi DSS-3]
>gi|56696762|ref|YP_167123.1| hypothetical protein SPO1886 [Silicibacter pomeroyi DSS-3]
>gi|56696765|ref|YP_167126.1| alcohol dehydrogenase, zinc-containing [Silicibacter pomeroyi DSS-3]
>gi|56696769|ref|YP_167130.1| ribonuclease, Rne/Rng family [Silicibacter pomeroyi DSS-3]
>gi|56696779|ref|YP_167140.1| hypothetical protein SPO1904 [Silicibacter pomeroyi DSS-3]
>gi|56696783|ref|YP_167144.1| chromate transporter [Silicibacter pomeroyi DSS-3]
>gi|56696803|ref|YP_167165.1| UDP-3-O-3-hydroxymyristoyl glucosamine N-acyltransferase [Silicibacter pomeroyi DSS-3]
>gi|56696817|ref|YP_167179.1| yicC family protein [Silicibacter pomeroyi DSS-3]
>gi|56696819|ref|YP_167181.1| bacterial transferase family protein [Silicibacter pomeroyi DSS-3]
>gi|56696840|ref|YP_167202.1| acyl-CoA dehydrogenase, putative [Silicibacter pomeroyi DSS-3]
>gi|56696843|ref|YP_167205.1| phosphoglycerate mutase family protein [Silicibacter pomeroyi DSS-3]
>gi|56696844|ref|YP_167206.1| enoyl-CoA hydratase/isomerase family protein [Silicibacter pomeroyi DSS-3]
>gi|56696848|ref|YP_167210.1| translation elongation factor Ts [Silicibacter pomeroyi DSS-3]
>gi|56696863|ref|YP_167225.1| ribosomal protein L21 [Silicibacter pomeroyi DSS-3]
>gi|56696871|ref|YP_167233.1| membrane protein, putative [Silicibacter pomeroyi DSS-3]
>gi|56696883|ref|YP_167245.1| membrane protein, putative [Silicibacter pomeroyi DSS-3]
>gi|56696885|ref|YP_167247.1| hypothetical protein SPO2015 [Silicibacter pomeroyi DSS-3]
>gi|56696886|ref|YP_167248.1| hypothetical protein SPO2016 [Silicibacter pomeroyi DSS-3]
>gi|56696889|ref|YP_167251.1| exoV domain protein [Silicibacter pomeroyi DSS-3]
>gi|56696929|ref|YP_167291.1| indigoidine synthase A family protein [Silicibacter pomeroyi DSS-3]
>gi|56696941|ref|YP_167303.1| hypothetical protein SPO2073 [Silicibacter pomeroyi DSS-3]
>gi|56696947|ref|YP_167309.1| Na(+)/H(+) antiporter, homolog [Silicibacter pomeroyi DSS-3]
>gi|56696967|ref|YP_167329.1| cytochrome c-554 [Silicibacter pomeroyi DSS-3]
>gi|56696983|ref|YP_167345.1| disulfide bond formation protein, DsbB family [Silicibacter pomeroyi DSS-3]
>gi|56696994|ref|YP_167356.1| transporter, putative [Silicibacter pomeroyi DSS-3]
>gi|56696999|ref|YP_167361.1| outer membrane protein, putative [Silicibacter pomeroyi DSS-3]
>gi|56697001|ref|YP_167363.1| hypothetical protein SPO2135 [Silicibacter pomeroyi DSS-3]
>gi|56697013|ref|YP_167375.1| hypothetical protein SPO2148 [Silicibacter pomeroyi DSS-3]
>gi|56697017|ref|YP_167379.1| molybdenum cofactor biosynthesis protein C [Silicibacter pomeroyi DSS-3]
>gi|56697020|ref|YP_167382.1| competence protein [Silicibacter pomeroyi DSS-3]
>gi|56697043|ref|YP_167406.1| ABC transporter, ATP-binding protein [Silicibacter pomeroyi DSS-3]
>gi|56697045|ref|YP_167407.1| permease, putative [Silicibacter pomeroyi DSS-3]
>gi|56697076|ref|YP_167439.1| 3-hydroxyisobutyrate dehydrogenase [Silicibacter pomeroyi DSS-3]
>gi|56697082|ref|YP_167445.1| rhodanese-like domain protein [Silicibacter pomeroyi DSS-3]
>gi|56697086|ref|YP_167449.1| hypothetical protein SPO2223 [Silicibacter pomeroyi DSS-3]
>gi|56697090|ref|YP_167453.1| hypothetical protein SPO2227 [Silicibacter pomeroyi DSS-3]
>gi|56697094|ref|YP_167457.1| hypothetical protein SPO2231 [Silicibacter pomeroyi DSS-3]
>gi|56697100|ref|YP_167463.1| hypothetical protein SPO2237 [Silicibacter pomeroyi DSS-3]
>gi|56697105|ref|YP_167468.1| pyruvate dehydrogenase complex, E2 component, dihydrolipoamide acetyltransferase [Silicibacter pomeroyi DSS-3]
>gi|56697117|ref|YP_167480.1| hypothetical protein SPO2255 [Silicibacter pomeroyi DSS-3]
>gi|56697118|ref|YP_167481.1| hypothetical protein SPO2256 [Silicibacter pomeroyi DSS-3]
>gi|56697120|ref|YP_167483.1| hypothetical protein SPO2258 [Silicibacter pomeroyi DSS-3]
>gi|56697122|ref|YP_167485.1| hypothetical protein SPO2260 [Silicibacter pomeroyi DSS-3]
>gi|56697137|ref|YP_167500.1| malonyl CoA-acyl carrier protein transacylase [Silicibacter pomeroyi DSS-3]
>gi|56697144|ref|YP_167507.1| ribosomal protein L9 [Silicibacter pomeroyi DSS-3]
>gi|56697159|ref|YP_167523.1| hypothetical protein SPO2300 [Silicibacter pomeroyi DSS-3]
>gi|56697161|ref|YP_167525.1| bioY family protein [Silicibacter pomeroyi DSS-3]
>gi|56697168|ref|YP_167532.1| membrane protein, putative [Silicibacter pomeroyi DSS-3]
>gi|56697175|ref|YP_167539.1| heme exporter protein CcmB [Silicibacter pomeroyi DSS-3]
>gi|56697177|ref|YP_167541.1| hypothetical protein SPO2318 [Silicibacter pomeroyi DSS-3]
>gi|56697178|ref|YP_167542.1| hypothetical protein SPO2319 [Silicibacter pomeroyi DSS-3]
>gi|56697185|ref|YP_167549.1| hypothetical protein SPO2326 [Silicibacter pomeroyi DSS-3]
>gi|56697189|ref|YP_167553.1| hypothetical protein SPO2330 [Silicibacter pomeroyi DSS-3]
>gi|56697192|ref|YP_167556.1| hypothetical protein SPO2333 [Silicibacter pomeroyi DSS-3]
>gi|56697193|ref|YP_167557.1| CAAX amino terminal protease family protein [Silicibacter pomeroyi DSS-3]
>gi|56697195|ref|YP_167559.1| lysM domain protein [Silicibacter pomeroyi DSS-3]
>gi|56697198|ref|YP_167562.1| enoyl-CoA hydratase/isomerase family protein [Silicibacter pomeroyi DSS-3]
>gi|56697211|ref|YP_167575.1| type I secretion target repeat protein [Silicibacter pomeroyi DSS-3]

>gi|56697238|ref|YP_167603.1| membrane protein, putative [Silicibacter pomeroyi DSS-3]
>gi|56697255|ref|YP_167620.1| type I secretion target repeat protein [Silicibacter pomeroyi DSS-3]
>gi|56697264|ref|YP_167630.1| oxidoreductase, short chain dehydrogenase/reductase family [Silicibacter pomeroyi DSS-3]
>gi|56697269|ref|YP_167635.1| transporter, LysE family [Silicibacter pomeroyi DSS-3]
>gi|56697296|ref|YP_167662.1| drug resistance transporter, Bcr/CflA subfamily [Silicibacter pomeroyi DSS-3]
>gi|56697299|ref|YP_167665.1| membrane protein, MarC family [Silicibacter pomeroyi DSS-3]
>gi|56697310|ref|YP_167676.1| hypothetical protein SPO2459 [Silicibacter pomeroyi DSS-3]
>gi|56697314|ref|YP_167680.1| hypothetical protein SPO2463 [Silicibacter pomeroyi DSS-3]
>gi|56697331|ref|YP_167697.1| drug/metabolite exporter family protein [Silicibacter pomeroyi DSS-3]
>gi|56697344|ref|YP_167712.1| transcriptional regulator, MerR family [Silicibacter pomeroyi DSS-3]
>gi|56697349|ref|YP_167717.1| beta-N-acetylhexosaminidase, putative [Silicibacter pomeroyi DSS-3]
>gi|56697354|ref|YP_167722.1| membrane protein, putative [Silicibacter pomeroyi DSS-3]
>gi|56697355|ref|YP_167723.1| hypothetical protein SPO2508 [Silicibacter pomeroyi DSS-3]
>gi|56697360|ref|YP_167728.1| hypothetical protein SPO2513 [Silicibacter pomeroyi DSS-3]
>gi|56697361|ref|YP_167729.1| hypothetical protein SPO2514 [Silicibacter pomeroyi DSS-3]
>gi|56697405|ref|YP_167773.1| 2-hydroxy-3-oxopropionate reductase [Silicibacter pomeroyi DSS-3]
>gi|56697422|ref|YP_167790.1| membrane protein, putative [Silicibacter pomeroyi DSS-3]
>gi|56697427|ref|YP_167795.1| membrane protein, putative [Silicibacter pomeroyi DSS-3]
>gi|56697431|ref|YP_167799.1| type I secretion target repeat protein [Silicibacter pomeroyi DSS-3]
>gi|56697435|ref|YP_167803.1| peptidase, M48 family [Silicibacter pomeroyi DSS-3]
>gi|56697459|ref|YP_167827.1| SPFH domain/band 7 family protein [Silicibacter pomeroyi DSS-3]
>gi|56697460|ref|YP_167828.1| hypothetical protein SPO2618 [Silicibacter pomeroyi DSS-3]
>gi|56697465|ref|YP_167833.1| transporter, LysE family [Silicibacter pomeroyi DSS-3]
>gi|56697483|ref|YP_167851.1| membrane protein, putative [Silicibacter pomeroyi DSS-3]
>gi|56697502|ref|YP_167870.1| glutamate/aspartate ABC transporter, permease protein [Silicibacter pomeroyi DSS-3]
>gi|56697526|ref|YP_167894.1| hypothetical protein SPO2684 [Silicibacter pomeroyi DSS-3]
>gi|56697528|ref|YP_167896.1| LysM domain/M23/M37 peptidase [Silicibacter pomeroyi DSS-3]
>gi|56697536|ref|YP_167904.1| cytochrome c' [Silicibacter pomeroyi DSS-3]
>gi|56697568|ref|YP_167936.1| hypothetical protein SPO2726 [Silicibacter pomeroyi DSS-3]
>gi|56697576|ref|YP_167945.1| lipoprotein, putative [Silicibacter pomeroyi DSS-3]
>gi|56697583|ref|YP_167952.1| hypothetical protein SPO2745 [Silicibacter pomeroyi DSS-3]
>gi|56697587|ref|YP_167956.1| membrane protein, putative [Silicibacter pomeroyi DSS-3]
>gi|56697592|ref|YP_167961.1| hypothetical protein SPO2754 [Silicibacter pomeroyi DSS-3]
>gi|56697593|ref|YP_167962.1| hypothetical protein SPO2755 [Silicibacter pomeroyi DSS-3]
>gi|56697594|ref|YP_167963.1| RNA polymerase sigma-70 factor, ECF family [Silicibacter pomeroyi DSS-3]
>gi|56697605|ref|YP_167974.1| NADH dehydrogenase I, J subunit [Silicibacter pomeroyi DSS-3]
>gi|56697613|ref|YP_167982.1| hypothetical protein SPO2775 [Silicibacter pomeroyi DSS-3]
>gi|56697617|ref|YP_167986.1| hypothetical protein SPO2779 [Silicibacter pomeroyi DSS-3]
>gi|56697621|ref|YP_167990.1| hypothetical protein SPO2783 [Silicibacter pomeroyi DSS-3]
>gi|56697625|ref|YP_167994.1| enoyl-CoA hydratase/isomerase family protein [Silicibacter pomeroyi DSS-3]
>gi|56697640|ref|YP_168010.1| bmp family protein [Silicibacter pomeroyi DSS-3]
>gi|56697655|ref|YP_168025.1| YeeE/YedE family protein [Silicibacter pomeroyi DSS-3]
>gi|56697666|ref|YP_168036.1| type I secretion target repeat protein [Silicibacter pomeroyi DSS-3]
>gi|56697676|ref|YP_168046.1| hypothetical protein SPO2838 [Silicibacter pomeroyi DSS-3]
>gi|56697682|ref|YP_168052.1| hypothetical protein SPO2844 [Silicibacter pomeroyi DSS-3]
>gi|56697684|ref|YP_168054.1| hypothetical protein SPO2846 [Silicibacter pomeroyi DSS-3]
>gi|56697686|ref|YP_168056.1| hypothetical protein SPO2848 [Silicibacter pomeroyi DSS-3]
>gi|56697690|ref|YP_168060.1| czcN domain protein [Silicibacter pomeroyi DSS-3]
>gi|56697693|ref|YP_168063.1| cobalt chelatase, pCobT subunit [Silicibacter pomeroyi DSS-3]
>gi|56697708|ref|YP_168078.1| cobalamin biosynthesis domain protein [Silicibacter pomeroyi DSS-3]
>gi|56697711|ref|YP_168081.1| uroporphyrin-III C-methyltransferase [Silicibacter pomeroyi DSS-3]
>gi|56697714|ref|YP_168084.1| membrane protein, putative [Silicibacter pomeroyi DSS-3]
>gi|56697715|ref|YP_168085.1| hypothetical protein SPO2877 [Silicibacter pomeroyi DSS-3]
>gi|56697718|ref|YP_168088.1| hypothetical protein SPO2880 [Silicibacter pomeroyi DSS-3]
>gi|56697730|ref|YP_168100.1| hypothetical protein SPO2892 [Silicibacter pomeroyi DSS-3]
>gi|56697749|ref|YP_168119.1| thioesterase family protein [Silicibacter pomeroyi DSS-3]
>gi|56697763|ref|YP_168133.1| hypothetical protein SPO2925 [Silicibacter pomeroyi DSS-3]
>gi|56697774|ref|YP_168143.1| hypothetical protein SPO2936 [Silicibacter pomeroyi DSS-3]
>gi|56697780|ref|YP_168150.1| hypothetical protein SPO2942 [Silicibacter pomeroyi DSS-3]
>gi|56697782|ref|YP_168152.1| hypothetical protein SPO2944 [Silicibacter pomeroyi DSS-3]
>gi|56697810|ref|YP_168181.1| membrane protein, putative [Silicibacter pomeroyi DSS-3]
>gi|56697814|ref|YP_168185.1| hypothetical protein SPO2979 [Silicibacter pomeroyi DSS-3]
>gi|56697818|ref|YP_168189.1| cytidine and deoxycytidylate deaminase family protein [Silicibacter pomeroyi DSS-3]
>gi|56697819|ref|YP_168190.1| RNA pseudouridylate synthase family protein [Silicibacter pomeroyi DSS-3]
>gi|56697820|ref|YP_168191.1| hypothetical protein SPO2985 [Silicibacter pomeroyi DSS-3]
>gi|56697828|ref|YP_168199.1| MOFRL family protein [Silicibacter pomeroyi DSS-3]
>gi|56697861|ref|YP_168232.1| hypothetical protein SPO3028 [Silicibacter pomeroyi DSS-3]
>gi|56697890|ref|YP_168261.1| streptogramin acetyltransferase, putative [Silicibacter pomeroyi DSS-3]
>gi|56697895|ref|YP_168266.1| transmembrane efflux protein, homoserine/threonine (RhtB) family [Silicibacter pomeroyi DSS-3]
>gi|56697912|ref|YP_168283.1| transcriptional regulator, TetR family [Silicibacter pomeroyi DSS-3]

>gi|56697917|ref|YP_168288.1| hypothetical protein SPO3085 [Silicibacter pomeroyi DSS-3]
>gi|56697927|ref|YP_168298.1| membrane protein, putative [Silicibacter pomeroyi DSS-3]
>gi|56697942|ref|YP_168313.1| tonB domain protein, putative [Silicibacter pomeroyi DSS-3]
>gi|56697949|ref|YP_168319.1| Holliday junction DNA helicase RuvA [Silicibacter pomeroyi DSS-3]
>gi|56697955|ref|YP_168326.1| YeeE/YedE family protein [Silicibacter pomeroyi DSS-3]
>gi|56697964|ref|YP_168335.1| hypothetical protein SPO3132 [Silicibacter pomeroyi DSS-3]
>gi|56697972|ref|YP_168343.1| conserved hypothetical protein TIGR01620 [Silicibacter pomeroyi DSS-3]
>gi|56697982|ref|YP_168353.1| transcriptional regulator, AraC family [Silicibacter pomeroyi DSS-3]
>gi|56697997|ref|YP_168368.1| ATP synthase delta chain [Silicibacter pomeroyi DSS-3]
>gi|56698003|ref|YP_168374.1| OmpA domain protein [Silicibacter pomeroyi DSS-3]
>gi|56698007|ref|YP_168378.1| hypothetical protein SPO3175 [Silicibacter pomeroyi DSS-3]
>gi|56698041|ref|YP_168412.1| transporter, LysE family [Silicibacter pomeroyi DSS-3]
>gi|56698045|ref|YP_168416.1| membrane protein, putative [Silicibacter pomeroyi DSS-3]
>gi|56698046|ref|YP_168417.1| hypothetical protein SPO3214 [Silicibacter pomeroyi DSS-3]
>gi|56698060|ref|YP_168431.1| SMC protein [Silicibacter pomeroyi DSS-3]
>gi|56698065|ref|YP_168436.1| ATP synthase F0, B subunit [Silicibacter pomeroyi DSS-3]
>gi|56698066|ref|YP_168437.1| ATP synthase F0, B' subunit [Silicibacter pomeroyi DSS-3]
>gi|56698067|ref|YP_168438.1| ATP synthase F0, C subunit [Silicibacter pomeroyi DSS-3]
>gi|56698069|ref|YP_168440.1| ATP synthase F0, subunit I [Silicibacter pomeroyi DSS-3]
>gi|56698071|ref|YP_168442.1| integral membrane protein [Silicibacter pomeroyi DSS-3]
>gi|56698078|ref|YP_168449.1| signal recognition particle protein [Silicibacter pomeroyi DSS-3]
>gi|56698112|ref|YP_168484.1| ferric iron ABC transporter, permease protein [Silicibacter pomeroyi DSS-3]
>gi|56698126|ref|YP_168498.1| hypothetical protein SPO3302 [Silicibacter pomeroyi DSS-3]
>gi|56698140|ref|YP_168512.1| membrane protein [Silicibacter pomeroyi DSS-3]
>gi|56698163|ref|YP_168535.1| bioY family protein [Silicibacter pomeroyi DSS-3]
>gi|56698165|ref|YP_168537.1| pyridoxal-phosphate dependent enzyme family protein [Silicibacter pomeroyi DSS-3]
>gi|56698169|ref|YP_168541.1| hypothetical protein SPO3345 [Silicibacter pomeroyi DSS-3]
>gi|56698171|ref|YP_168543.1| hypothetical protein SPO3347 [Silicibacter pomeroyi DSS-3]
>gi|56698180|ref|YP_168552.1| hypothetical protein SPO3356 [Silicibacter pomeroyi DSS-3]
>gi|56698193|ref|YP_168565.1| oxidoreductase, short-chain dehydrogenase/reductase family [Silicibacter pomeroyi DSS-3]
>gi|56698202|ref|YP_168574.1| benzoate transporter [Silicibacter pomeroyi DSS-3]
>gi|56698209|ref|YP_168581.1| glycosyl transferase, family 25 [Silicibacter pomeroyi DSS-3]
>gi|56698230|ref|YP_168602.1| transcriptional regulator, TetR family [Silicibacter pomeroyi DSS-3]
>gi|56698231|ref|YP_168603.1| hypothetical protein SPO3407 [Silicibacter pomeroyi DSS-3]
>gi|56698232|ref|YP_168604.1| acetyl-CoA C-acetyltransferase [Silicibacter pomeroyi DSS-3]
>gi|56698246|ref|YP_168619.1| thioredoxin [Silicibacter pomeroyi DSS-3]
>gi|56698247|ref|YP_168620.1| leucine rich repeat protein [Silicibacter pomeroyi DSS-3]
>gi|56698263|ref|YP_168636.1| 20-beta-hydroxysteroid dehydrogenase, putative [Silicibacter pomeroyi DSS-3]
>gi|56698265|ref|YP_168638.1| PmbA [Silicibacter pomeroyi DSS-3]
>gi|56698283|ref|YP_168656.1| hypothetical protein SPO3460 [Silicibacter pomeroyi DSS-3]
>gi|56698297|ref|YP_168670.1| polyamine ABC transporter, permease protein [Silicibacter pomeroyi DSS-3]
>gi|56698316|ref|YP_168689.1| transporter, putative [Silicibacter pomeroyi DSS-3]
>gi|56698318|ref|YP_168691.1| large conductance mechanosensitive channel protein [Silicibacter pomeroyi DSS-3]
>gi|56698327|ref|YP_168700.1| membrane protein, putative [Silicibacter pomeroyi DSS-3]
>gi|56698331|ref|YP_168704.1| ribosomal protein L7/L12 [Silicibacter pomeroyi DSS-3]
>gi|56698350|ref|YP_168723.1| membrane protein, putative [Silicibacter pomeroyi DSS-3]
>gi|56698379|ref|YP_168752.1| dimethyl sulfoxide reductase, C subunit, putative [Silicibacter pomeroyi DSS-3]
>gi|56698386|ref|YP_168759.1| permease, putative [Silicibacter pomeroyi DSS-3]
>gi|56698387|ref|YP_168760.1| hypothetical protein SPO3565 [Silicibacter pomeroyi DSS-3]
>gi|56698394|ref|YP_168767.1| isochorismatase family protein [Silicibacter pomeroyi DSS-3]
>gi|56698398|ref|YP_168771.1| OmpA domain protein [Silicibacter pomeroyi DSS-3]
>gi|56698406|ref|YP_168779.1| membrane protein, drug/metabolite transporter (DMT) family [Silicibacter pomeroyi DSS-3]
>gi|56698413|ref|YP_168786.1| auxin efflux carrier family protein [Silicibacter pomeroyi DSS-3]
>gi|56698439|ref|YP_168813.1| sulfonate ABC transporter, permease protein, putative [Silicibacter pomeroyi DSS-3]
>gi|56698444|ref|YP_168818.1| hypothetical protein SPO3623 [Silicibacter pomeroyi DSS-3]
>gi|56698447|ref|YP_168821.1| hypothetical protein SPO3627 [Silicibacter pomeroyi DSS-3]
>gi|56698464|ref|YP_168839.1| hypothetical protein SPO3644 [Silicibacter pomeroyi DSS-3]
>gi|56698482|ref|YP_168857.1| membrane protein, putative [Silicibacter pomeroyi DSS-3]
>gi|56698492|ref|YP_168868.1| type I secretion target repeat protein [Silicibacter pomeroyi DSS-3]
>gi|56698526|ref|YP_168902.1| branched-chain amino acid ABC transporter, permease protein [Silicibacter pomeroyi DSS-3]
>gi|56698527|ref|YP_168903.1| branched-chain amino acid ABC transporter, permease protein [Silicibacter pomeroyi DSS-3]
>gi|56698529|ref|YP_168905.1| membrane protein, putative [Silicibacter pomeroyi DSS-3]
>gi|56698553|ref|YP_168929.1| major facilitator family protein [Silicibacter pomeroyi DSS-3]
>gi|56698561|ref|YP_168937.1| membrane protein, putative [Silicibacter pomeroyi DSS-3]
>gi|56698603|ref|YP_168980.1| sugar ABC transporter, permease protein [Silicibacter pomeroyi DSS-3]
>gi|56698634|ref|YP_169011.1| ribosomal protein L23 [Silicibacter pomeroyi DSS-3]
>gi|56698644|ref|YP_169021.1| ATP-dependent RNA helicase, DEAD/DEAH box family [Silicibacter pomeroyi DSS-3]
>gi|56698645|ref|YP_169022.1| dihydrodipicolinate reductase [Silicibacter pomeroyi DSS-3]
>gi|56698650|ref|YP_169027.1| type I secretion target repeat protein [Silicibacter pomeroyi DSS-3]
>gi|56698653|ref|YP_169030.1| hypothetical protein SPO3842 [Silicibacter pomeroyi DSS-3]

>gi|56698663|ref|YP_169040.1| hypothetical protein SPO3852 [Silicibacter pomeroyi DSS-3]
>gi|56698665|ref|YP_169042.1| O-sialoglycoprotein endopeptidase, putative [Silicibacter pomeroyi DSS-3]
>gi|56698690|ref|YP_169067.1| membrane protein, putative [Silicibacter pomeroyi DSS-3]
>gi|56698722|ref|YP_168837.1| carbon monoxide dehydrogenase G protein, putative [Silicibacter pomeroyi DSS-3]
>gi|56698733|ref|YP_167702.1| hypothetical protein SPO2486 [Silicibacter pomeroyi DSS-3]
>gi|56708798|ref|YP_164839.1| hypothetical protein SPOA0007 [Silicibacter pomeroyi DSS-3]
>gi|56708801|ref|YP_164842.1| apolipoprotein N-acyltransferase [Silicibacter pomeroyi DSS-3]
>gi|56708843|ref|YP_164884.1| nitrous oxide maturation protein NosY [Silicibacter pomeroyi DSS-3]
>gi|56708845|ref|YP_164886.1| cytochrome c family protein [Silicibacter pomeroyi DSS-3]
>gi|56708855|ref|YP_164896.1| thiamine pyrophosphate-dependent enzyme [Silicibacter pomeroyi DSS-3]
>gi|56708861|ref|YP_164902.1| polar amino acid uptake family ABC transporter, permease protein [Silicibacter pomeroyi DSS-3]
>gi|56708881|ref|YP_164924.1| hypothetical protein SPOA0094 [Silicibacter pomeroyi DSS-3]
>gi|56708885|ref|YP_164928.1| branched-chain amino acid ABC transporter, permease protein [Silicibacter pomeroyi DSS-3]
>gi|56708922|ref|YP_164967.1| hypothetical protein SPOA0136 [Silicibacter pomeroyi DSS-3]
>gi|56708934|ref|YP_164979.1| membrane protein, major facilitator transporter family [Silicibacter pomeroyi DSS-3]
>gi|56708941|ref|YP_164986.1| type I secretion target repeat protein [Silicibacter pomeroyi DSS-3]
>gi|56708988|ref|YP_165033.1| hypothetical protein SPOA0204 [Silicibacter pomeroyi DSS-3]
>gi|56708996|ref|YP_165041.1| nitric oxide reductase F protein, putative [Silicibacter pomeroyi DSS-3]
>gi|56708997|ref|YP_165042.1| nitric oxide reductase E protein, putative [Silicibacter pomeroyi DSS-3]
>gi|56709003|ref|YP_165048.1| NnrS, putative [Silicibacter pomeroyi DSS-3]
>gi|56709010|ref|YP_165055.1| nitrite reductase heme biosynthesis H protein [Silicibacter pomeroyi DSS-3]
>gi|56709013|ref|YP_165058.1| membrane protein, putative [Silicibacter pomeroyi DSS-3]
>gi|56709017|ref|YP_165062.1| glycine betaine/proline ABC transporter, permease protein, putative [Silicibacter pomeroyi DSS-3]
>gi|56709032|ref|YP_165077.1| hypothetical protein SPOA0248 [Silicibacter pomeroyi DSS-3]
>gi|56709041|ref|YP_165086.1| ribose ABC transporter, permease protein [Silicibacter pomeroyi DSS-3]
>gi|56709074|ref|YP_165119.1| hypothetical protein SPOA0290 [Silicibacter pomeroyi DSS-3]
>gi|56709129|ref|YP_165174.1| membrane protein, putative [Silicibacter pomeroyi DSS-3]
>gi|56709145|ref|YP_165190.1| membrane protein, putative [Silicibacter pomeroyi DSS-3]
>gi|56709149|ref|YP_165194.1| ABC transporter, permease protein [Silicibacter pomeroyi DSS-3]
>gi|56709175|ref|YP_165220.1| R body protein RebB homolog [Silicibacter pomeroyi DSS-3]
>gi|56709176|ref|YP_165221.1| hypothetical protein SPOA0394 [Silicibacter pomeroyi DSS-3]
>gi|56709178|ref|YP_165223.1| hypothetical protein SPOA0396 [Silicibacter pomeroyi DSS-3]
>gi|56709189|ref|YP_165235.1| hypothetical protein SPOA0408 [Silicibacter pomeroyi DSS-3]
>gi|56709191|ref|YP_165237.1| hypothetical protein SPOA0410 [Silicibacter pomeroyi DSS-3]
>gi|56709192|ref|YP_165238.1| hypothetical protein SPOA0411 [Silicibacter pomeroyi DSS-3]
>gi|56709193|ref|YP_165239.1| xanthine dehydrogenase family protein, medium subunit [Silicibacter pomeroyi DSS-3]
>gi|34556463|ref|NP_906278.1| PUTATIVE INTEGRAL MEMBRANE PROTEIN [Wolinella succinogenes DSM 1740]
>gi|34556474|ref|NP_906289.1| hypothetical protein WS0015 [Wolinella succinogenes DSM 1740]
>gi|34556530|ref|NP_906345.1| hypothetical protein WS0076 [Wolinella succinogenes DSM 1740]
>gi|34556534|ref|NP_906349.1| APOLIPOPROTEIN N-ACYLTRANSFERASE [Wolinella succinogenes DSM 1740]
>gi|34556540|ref|NP_906355.1| hypothetical protein WS0087 [Wolinella succinogenes DSM 1740]
>gi|34556544|ref|NP_906359.1| hypothetical protein WS0091 [Wolinella succinogenes DSM 1740]
>gi|34556584|ref|NP_906399.1| hypothetical protein WS0136 [Wolinella succinogenes DSM 1740]
>gi|34556635|ref|NP_906450.1| hypothetical protein WS0189 [Wolinella succinogenes DSM 1740]
>gi|34556642|ref|NP_906457.1| CONSERED HYPOTHETICAL PROTEIN [Wolinella succinogenes DSM 1740]
>gi|34556666|ref|NP_906481.1| PUTATIVE ZINC-METALLO PROTEASE [Wolinella succinogenes DSM 1740]
>gi|34556687|ref|NP_906502.1| hypothetical protein WS0247 [Wolinella succinogenes DSM 1740]
>gi|34556757|ref|NP_906572.1| COMPETENCE PROETIN 1 [Wolinella succinogenes DSM 1740]
>gi|34556763|ref|NP_906578.1| 50S RIBOSOMAL PROTEIN L28 [Wolinella succinogenes DSM 1740]
>gi|34556835|ref|NP_906650.1| hypothetical protein WS0406 [Wolinella succinogenes DSM 1740]
>gi|34556837|ref|NP_906652.1| hypothetical protein WS0408 [Wolinella succinogenes DSM 1740]
>gi|34556839|ref|NP_906654.1| AMMONIUM TRANSPORTER, AMTB [Wolinella succinogenes DSM 1740]
>gi|34556854|ref|NP_906669.1| hypothetical protein WS0426 [Wolinella succinogenes DSM 1740]
>gi|34556855|ref|NP_906670.1| hypothetical protein WS0427 [Wolinella succinogenes DSM 1740]
>gi|34556891|ref|NP_906706.1| PUTATIVE 50S RIBOSOMAL SUBUNIT PROTEIN L7/L12 [Wolinella succinogenes DSM 1740]
>gi|34556934|ref|NP_906749.1| ATP SYNTHASE F0 SECTOR B SUBUNIT [Wolinella succinogenes DSM 1740]
>gi|34556953|ref|NP_906768.1| HYPOTHETICAL PROTEIN , WS0532 [Wolinella succinogenes DSM 1740]
>gi|34556961|ref|NP_906776.1| PUTATIVE TYPE II SECRETION SYSTEM D [Wolinella succinogenes DSM 1740]
>gi|34556981|ref|NP_906796.1| hypothetical protein WS0562 [Wolinella succinogenes DSM 1740]
>gi|34557018|ref|NP_906833.1| hypothetical protein WS0602 [Wolinella succinogenes DSM 1740]
>gi|34557048|ref|NP_906863.1| 30S ribosomal protein S21 [Wolinella succinogenes DSM 1740]
>gi|34557054|ref|NP_906869.1| ABC TRANSPORTER (ATP-BINDING PROTEIN) [Wolinella succinogenes DSM 1740]
>gi|34557062|ref|NP_906877.1| CBIM PROTEIN [Wolinella succinogenes DSM 1740]
>gi|34557064|ref|NP_906879.1| hypothetical protein WS0648 [Wolinella succinogenes DSM 1740]
>gi|34557085|ref|NP_906900.1| hypothetical protein WS0672 [Wolinella succinogenes DSM 1740]
>gi|34557133|ref|NP_906948.1| PUTATIVE INTEGRAL MEMBRANE PROTEIN [Wolinella succinogenes DSM 1740]
>gi|34557179|ref|NP_906994.1| hypothetical protein WS0780 [Wolinella succinogenes DSM 1740]
>gi|34557195|ref|NP_907010.1| hypothetical protein WS0797 [Wolinella succinogenes DSM 1740]
>gi|34557242|ref|NP_907057.1| hypothetical protein WS0846 [Wolinella succinogenes DSM 1740]
>gi|34557254|ref|NP_907069.1| hypothetical protein WS0860 [Wolinella succinogenes DSM 1740]

>gi|34557256|ref|NP_907071.1| hypothetical protein WS0862 [Wolinella succinogenes DSM 1740]
>gi|34557298|ref|NP_907113.1| ABCB PROTEIN [Wolinella succinogenes DSM 1740]
>gi|34557331|ref|NP_907146.1| PUTATIVE INTEGRAL MEMBRANE PROTEIN [Wolinella succinogenes DSM 1740]
>gi|34557379|ref|NP_907194.1| hypothetical protein WS0991 [Wolinella succinogenes DSM 1740]
>gi|34557406|ref|NP_907221.1| hypothetical protein WS1019 [Wolinella succinogenes DSM 1740]
>gi|34557427|ref|NP_907242.1| hypothetical protein WS1042 [Wolinella succinogenes DSM 1740]
>gi|34557493|ref|NP_907308.1| PROBABLE TRANSMEMBRANE PROTEIN [Wolinella succinogenes DSM 1740]
>gi|34557503|ref|NP_907318.1| hypothetical protein WS1128 [Wolinella succinogenes DSM 1740]
>gi|34557511|ref|NP_907326.1| hypothetical protein WS1138 [Wolinella succinogenes DSM 1740]
>gi|34557516|ref|NP_907331.1| hypothetical protein WS1144 [Wolinella succinogenes DSM 1740]
>gi|34557530|ref|NP_907345.1| hypothetical protein WS1158 [Wolinella succinogenes DSM 1740]
>gi|34557531|ref|NP_907346.1| PUTATIVE ABC TRANSPORTER, PERMEASE PROTEIN [Wolinella succinogenes DSM 1740]
>gi|34557533|ref|NP_907348.1| hypothetical protein WS1161 [Wolinella succinogenes DSM 1740]
>gi|34557536|ref|NP_907351.1| hypothetical protein WS1164 [Wolinella succinogenes DSM 1740]
>gi|34557546|ref|NP_907361.1| PUTATIVE FERREDOXIN [Wolinella succinogenes DSM 1740]
>gi|34557555|ref|NP_907370.1| INTEGRAL MEMBRANE PROTEIN [Wolinella succinogenes DSM 1740]
>gi|34557561|ref|NP_907376.1| hypothetical protein WS1192 [Wolinella succinogenes DSM 1740]
>gi|34557565|ref|NP_907380.1| hypothetical protein WS1196 [Wolinella succinogenes DSM 1740]
>gi|34557594|ref|NP_907409.1| hypothetical protein WS1228 [Wolinella succinogenes DSM 1740]
>gi|34557597|ref|NP_907412.1| hypothetical protein WS1231 [Wolinella succinogenes DSM 1740]
>gi|34557611|ref|NP_907426.1| hypothetical protein WS1246 [Wolinella succinogenes DSM 1740]
>gi|34557617|ref|NP_907432.1| CO-CHAPERONE-CURVED DNA BINDING PROTEIN A (CBPA) [Wolinella succinogenes DSM 1740]
>gi|34557632|ref|NP_907447.1| PUTATIVE INTEGRAL MEMBRANE PROTEIN [Wolinella succinogenes DSM 1740]
>gi|34557676|ref|NP_907491.1| ABC TRANSPORTER INTEGRAL MEMBRANE PROTEIN [Wolinella succinogenes DSM 1740]
>gi|34557690|ref|NP_907505.1| hypothetical protein WS1330 [Wolinella succinogenes DSM 1740]
>gi|34557712|ref|NP_907527.1| hypothetical protein WS1356 [Wolinella succinogenes DSM 1740]
>gi|34557765|ref|NP_907580.1| PROBABLE OUTER MEMBRANE PROTEIN [Wolinella succinogenes DSM 1740]
>gi|34557773|ref|NP_907588.1| hypothetical protein WS1426 [Wolinella succinogenes DSM 1740]
>gi|34557795|ref|NP_907610.1| hypothetical protein WS1451 [Wolinella succinogenes DSM 1740]
>gi|34557800|ref|NP_907615.1| hypothetical protein WS1456 [Wolinella succinogenes DSM 1740]
>gi|34557808|ref|NP_907623.1| hypothetical protein WS1465 [Wolinella succinogenes DSM 1740]
>gi|34557818|ref|NP_907633.1| hypothetical protein WS1478 [Wolinella succinogenes DSM 1740]
>gi|34557854|ref|NP_907669.1| hypothetical protein WS1521 [Wolinella succinogenes DSM 1740]
>gi|34557857|ref|NP_907672.1| hypothetical protein WS1525 [Wolinella succinogenes DSM 1740]
>gi|34557870|ref|NP_907685.1| hypothetical protein WS1542 [Wolinella succinogenes DSM 1740]
>gi|34557879|ref|NP_907694.1| hypothetical protein WS1551 [Wolinella succinogenes DSM 1740]
>gi|34557880|ref|NP_907695.1| hypothetical protein WS1552 [Wolinella succinogenes DSM 1740]
>gi|34557890|ref|NP_907705.1| INTEGRAL MEMBRANE PROTEIN [Wolinella succinogenes DSM 1740]
>gi|34557899|ref|NP_907714.1| hypothetical protein WS1574 [Wolinella succinogenes DSM 1740]
>gi|34557909|ref|NP_907724.1| hypothetical protein WS1584 [Wolinella succinogenes DSM 1740]
>gi|34557937|ref|NP_907752.1| hypothetical protein WS1620 [Wolinella succinogenes DSM 1740]
>gi|34557939|ref|NP_907754.1| DNA REPAIR PROTEIN-ATPASE [Wolinella succinogenes DSM 1740]
>gi|34558013|ref|NP_907828.1| 50S RIBOSOMAL PROTEIN L18 [Wolinella succinogenes DSM 1740]
>gi|34558017|ref|NP_907832.1| 50S RIBOSOMAL PROTEIN L24 [Wolinella succinogenes DSM 1740]
>gi|34558050|ref|NP_907865.1| PREDICTED PEMEASE [Wolinella succinogenes DSM 1740]
>gi|34558068|ref|NP_907883.1| hypothetical protein WS1761 [Wolinella succinogenes DSM 1740]
>gi|34558069|ref|NP_907884.1| hypothetical protein WS1762 [Wolinella succinogenes DSM 1740]
>gi|34558099|ref|NP_907914.1| HYPOTHETICAL PROTEIN-Signal transduction histidine kinase [Wolinella succinogenes DSM 1740]
>gi|34558108|ref|NP_907923.1| MOLYBDENUM TRANSPORT SYSTEM PROTEIN [Wolinella succinogenes DSM 1740]
>gi|34558109|ref|NP_907924.1| PUTATIVE TRANSMEMBRANE PROTEIN [Wolinella succinogenes DSM 1740]
>gi|34558124|ref|NP_907939.1| MEMBRANE PROTEIN-Predicted permease [Wolinella succinogenes DSM 1740]
>gi|34558126|ref|NP_907941.1| 3-METHYL-2-OXOBUTANOATE HYDROXYMETHYLTRANSFERASE [Wolinella succinogenes DSM 1740]
>gi|34558152|ref|NP_907967.1| hypothetical protein WS1854 [Wolinella succinogenes DSM 1740]
>gi|34558153|ref|NP_907968.1| hypothetical protein WS1855 [Wolinella succinogenes DSM 1740]
>gi|34558154|ref|NP_907969.1| hypothetical protein WS1857 [Wolinella succinogenes DSM 1740]
>gi|34558161|ref|NP_907976.1| hypothetical protein WS1866 [Wolinella succinogenes DSM 1740]
>gi|34558163|ref|NP_907978.1| TRANSPORT TRANSMEMBRANE PROTEIN-Phosphate/sulphate permeases [Wolinella succinogenes DSM 1740]
>gi|34558176|ref|NP_907991.1| PUTATIVE PERIPLASMIC PROTEIN [Wolinella succinogenes DSM 1740]
>gi|34558182|ref|NP_907997.1| hypothetical protein WS1887 [Wolinella succinogenes DSM 1740]
>gi|34558183|ref|NP_907998.1| hypothetical protein WS1888 [Wolinella succinogenes DSM 1740]
>gi|34558187|ref|NP_908002.1| hypothetical protein WS1892 [Wolinella succinogenes DSM 1740]
>gi|34558198|ref|NP_908013.1| hypothetical protein WS1904 [Wolinella succinogenes DSM 1740]
>gi|34558226|ref|NP_908041.1| hypothetical protein WS1934 [Wolinella succinogenes DSM 1740]
>gi|34558228|ref|NP_908043.1| PHOSPHATIDYLGLYCEROPHOSPHATASE A [Wolinella succinogenes DSM 1740]
>gi|34558273|ref|NP_908088.1| hypothetical protein WS1985 [Wolinella succinogenes DSM 1740]
>gi|34558284|ref|NP_908099.1| PUTATIVE FLAGELLAR EXPORT APPARATUS [Wolinella succinogenes DSM 1740]
>gi|34558303|ref|NP_908118.1| TRANSLATION INITIATION FACTOR IF-2 [Wolinella succinogenes DSM 1740]
>gi|34558312|ref|NP_908127.1| hypothetical protein WS2026 [Wolinella succinogenes DSM 1740]
>gi|34558349|ref|NP_908164.1| conserved hypothetical protein-ACETYLTRANSFERASE ARGA [Wolinella succinogenes DSM 1740]

>gi|34558400|ref|NP_908215.1| hypothetical protein WS2117 [Wolinella succinogenes DSM 1740]
>gi|34558463|ref|NP_908278.1| PUTATIVE ACETYLTRANSFERASE [Wolinella succinogenes DSM 1740]
>gi|34558473|ref|NP_908288.1| FLAGELLIN B [Wolinella succinogenes DSM 1740]
>gi|34558479|ref|NP_908294.1| hypothetical protein WS2204 [Wolinella succinogenes DSM 1740]
>gi|15896982|ref|NP_341587.1| hypothetical protein SSO5023 [Sulfolobus solfataricus P2]
>gi|15896997|ref|NP_341602.1| hypothetical protein SSO0025 [Sulfolobus solfataricus P2]
>gi|15897005|ref|NP_341610.1| hypothetical protein SSO0035 [Sulfolobus solfataricus P2]
>gi|15897025|ref|NP_341630.1| hypothetical protein SSO0055 [Sulfolobus solfataricus P2]
>gi|15897076|ref|NP_341681.1| hypothetical protein SSO5209 [Sulfolobus solfataricus P2]
>gi|15897089|ref|NP_341694.1| hypothetical protein SSO0131 [Sulfolobus solfataricus P2]
>gi|15897178|ref|NP_341783.1| SSU ribosomal protein S28E (rps28E) [Sulfolobus solfataricus P2]
>gi|15897195|ref|NP_341800.1| hypothetical protein SSO0251 [Sulfolobus solfataricus P2]
>gi|15897229|ref|NP_341834.1| SSU ribosomal protein S17E (rps17E) [Sulfolobus solfataricus P2]
>gi|15897256|ref|NP_341861.1| hypothetical protein SSO0315 [Sulfolobus solfataricus P2]
>gi|15897262|ref|NP_341867.1| NADH dehydrogenase subunit A (NuoA) [Sulfolobus solfataricus P2]
>gi|15897277|ref|NP_341882.1| LSU ribosomal protein L12AB (rpl12AB) [Sulfolobus solfataricus P2]
>gi|15897289|ref|NP_341894.1| Apoptosis-related Tfar19 related protein (pdcd5) [Sulfolobus solfataricus P2]
>gi|15897313|ref|NP_341918.1| hypothetical protein SSO0379 [Sulfolobus solfataricus P2]
>gi|15897322|ref|NP_341927.1| hypothetical protein SSO0390 [Sulfolobus solfataricus P2]
>gi|15897330|ref|NP_341935.1| hypothetical protein SSO0396 [Sulfolobus solfataricus P2]
>gi|15897354|ref|NP_341959.1| SSU ribosomal protein S25E (rps25E) [Sulfolobus solfataricus P2]
>gi|15897386|ref|NP_341991.1| hypothetical protein SSO0455 [Sulfolobus solfataricus P2]
>gi|15897399|ref|NP_342004.1| hypothetical protein SSO5909 [Sulfolobus solfataricus P2]
>gi|15897408|ref|NP_342013.1| hypothetical protein SSO0482 [Sulfolobus solfataricus P2]
>gi|15897410|ref|NP_342015.1| tatC protein (tatC) [Sulfolobus solfataricus P2]
>gi|15897421|ref|NP_342026.1| hypothetical protein SSO0496 [Sulfolobus solfataricus P2]
>gi|15897446|ref|NP_342051.1| hypothetical protein SSO0523 [Sulfolobus solfataricus P2]
>gi|15897466|ref|NP_342071.1| hypothetical protein SSO0543 [Sulfolobus solfataricus P2]
>gi|15897482|ref|NP_342087.1| ATP synthase subunit F (atpF) [Sulfolobus solfataricus P2]
>gi|15897488|ref|NP_342093.1| ATP synthase subunit K (atpK) [Sulfolobus solfataricus P2]
>gi|15897501|ref|NP_342106.1| 4-hydroxybenzoate octaprenyltransferase , putative (ubiA-2) [Sulfolobus solfataricus P2]
>gi|15897521|ref|NP_342126.1| Zn finger protein, hypothetical [Sulfolobus solfataricus P2]
>gi|15897523|ref|NP_342128.1| hypothetical protein SSO0607 [Sulfolobus solfataricus P2]
>gi|15897524|ref|NP_342129.1| hypothetical protein SSO0608 [Sulfolobus solfataricus P2]
>gi|15897543|ref|NP_342148.1| hypothetical protein SSO6264 [Sulfolobus solfataricus P2]
>gi|15897559|ref|NP_342164.1| hypothetical protein SSO0649 [Sulfolobus solfataricus P2]
>gi|15897578|ref|NP_342183.1| hypothetical protein SSO0670 [Sulfolobus solfataricus P2]
>gi|15897579|ref|NP_342184.1| hypothetical protein SSO0671 [Sulfolobus solfataricus P2]
>gi|15897591|ref|NP_342196.1| hypothetical protein SSO0686 [Sulfolobus solfataricus P2]
>gi|15897663|ref|NP_342268.1| hypothetical protein SSO6469 [Sulfolobus solfataricus P2]
>gi|15897677|ref|NP_342282.1| hypothetical protein SSO0775 [Sulfolobus solfataricus P2]
>gi|15897717|ref|NP_342322.1| hypothetical protein SSO0816 [Sulfolobus solfataricus P2]
>gi|15897734|ref|NP_342339.1| hypothetical protein SSO0834 [Sulfolobus solfataricus P2]
>gi|15897746|ref|NP_342351.1| hypothetical protein SSO0847 [Sulfolobus solfataricus P2]
>gi|15897748|ref|NP_342353.1| hypothetical protein SSO0849 [Sulfolobus solfataricus P2]
>gi|15897786|ref|NP_342391.1| hypothetical protein SSO0900 [Sulfolobus solfataricus P2]
>gi|15897799|ref|NP_342404.1| hypothetical protein SSO6778 [Sulfolobus solfataricus P2]
>gi|15897807|ref|NP_342412.1| Glycine cleavage system protein H [Sulfolobus solfataricus P2]
>gi|15897825|ref|NP_342430.1| hypothetical protein SSO0944 [Sulfolobus solfataricus P2]
>gi|15897827|ref|NP_342432.1| hypothetical protein SSO0947 [Sulfolobus solfataricus P2]
>gi|15897870|ref|NP_342475.1| hypothetical protein SSO0994 [Sulfolobus solfataricus P2]
>gi|15897886|ref|NP_342491.1| High-affinity nickel-transport protein (hoxN) [Sulfolobus solfataricus P2]
>gi|15897888|ref|NP_342493.1| hypothetical protein SSO1020 [Sulfolobus solfataricus P2]
>gi|15897893|ref|NP_342498.1| hypothetical protein SSO1025 [Sulfolobus solfataricus P2]
>gi|15897899|ref|NP_342504.1| hypothetical protein SSO1031 [Sulfolobus solfataricus P2]
>gi|15897951|ref|NP_342556.1| hypothetical protein SSO1086 [Sulfolobus solfataricus P2]
>gi|15897954|ref|NP_342559.1| hypothetical protein SSO1088 [Sulfolobus solfataricus P2]
>gi|15897995|ref|NP_342600.1| Na(+)/H(+) antiporter [Sulfolobus solfataricus P2]
>gi|15898002|ref|NP_342607.1| hypothetical protein SSO1146 [Sulfolobus solfataricus P2]
>gi|15898026|ref|NP_342631.1| Amino acid permease related protein [Sulfolobus solfataricus P2]
>gi|15898039|ref|NP_342644.1| hypothetical protein SSO1185 [Sulfolobus solfataricus P2]
>gi|15898054|ref|NP_342659.1| Transposase ISC1048 [Sulfolobus solfataricus P2]
>gi|15898063|ref|NP_342668.1| hypothetical protein SSO1210 [Sulfolobus solfataricus P2]
>gi|15898086|ref|NP_342691.1| First ORF in transposon ISC1491 [Sulfolobus solfataricus P2]
>gi|15898091|ref|NP_342696.1| Third ORF in transposon ISC1217 [Sulfolobus solfataricus P2]
>gi|15898100|ref|NP_342705.1| Microtubule binding protein, putative [Sulfolobus solfataricus P2]
>gi|15898103|ref|NP_342708.1| hypothetical protein SSO1260 [Sulfolobus solfataricus P2]
>gi|15898130|ref|NP_342735.1| hypothetical protein SSO1287 [Sulfolobus solfataricus P2]
>gi|15898187|ref|NP_342792.1| hypothetical protein SSO1346 [Sulfolobus solfataricus P2]
>gi|15898195|ref|NP_342800.1| Endoglucanase precursor [Sulfolobus solfataricus P2]

>gi|15898273|ref|NP_342878.1| Transposase ISC1173 [Sulfolobus solfataricus P2]
>gi|15898301|ref|NP_342906.1| hypothetical protein SSO1469 [Sulfolobus solfataricus P2]
>gi|15898324|ref|NP_342929.1| hypothetical protein SSO1494 [Sulfolobus solfataricus P2]
>gi|15898398|ref|NP_343003.1| hypothetical protein SSO1583 [Sulfolobus solfataricus P2]
>gi|15898422|ref|NP_343027.1| hypothetical protein SSO1606 [Sulfolobus solfataricus P2]
>gi|15898426|ref|NP_343031.1| hypothetical protein SSO1608 [Sulfolobus solfataricus P2]
>gi|15898435|ref|NP_343040.1| hypothetical protein SSO1615 [Sulfolobus solfataricus P2]
>gi|15898455|ref|NP_343060.1| hypothetical protein SSO1637 [Sulfolobus solfataricus P2]
>gi|15898511|ref|NP_343116.1| Second ORF in transposon ISC1491 [Sulfolobus solfataricus P2]
>gi|15898527|ref|NP_343132.1| Second ORF in transposon ISC1491 [Sulfolobus solfataricus P2]
>gi|15898542|ref|NP_343147.1| hypothetical protein SSO1740 [Sulfolobus solfataricus P2]
>gi|15898553|ref|NP_343158.1| hypothetical protein SSO1752 [Sulfolobus solfataricus P2]
>gi|15898571|ref|NP_343176.1| Multidrug-efflux transporter [Sulfolobus solfataricus P2]
>gi|15898581|ref|NP_343186.1| hypothetical protein SSO1784 [Sulfolobus solfataricus P2]
>gi|15898599|ref|NP_343204.1| hypothetical protein SSO8938 [Sulfolobus solfataricus P2]
>gi|15898615|ref|NP_343220.1| hypothetical protein SSO1813 [Sulfolobus solfataricus P2]
>gi|15898621|ref|NP_343226.1| hypothetical protein SSO1818 [Sulfolobus solfataricus P2]
>gi|15898623|ref|NP_343228.1| hypothetical protein SSO1819 [Sulfolobus solfataricus P2]
>gi|15898629|ref|NP_343234.1| hypothetical protein SSO1827 [Sulfolobus solfataricus P2]
>gi|15898663|ref|NP_343268.1| hypothetical protein SSO1870 [Sulfolobus solfataricus P2]
>gi|15898665|ref|NP_343270.1| hypothetical protein SSO1872 [Sulfolobus solfataricus P2]
>gi|15898667|ref|NP_343272.1| hypothetical protein SSO1874 [Sulfolobus solfataricus P2]
>gi|15898674|ref|NP_343279.1| hypothetical protein SSO1880 [Sulfolobus solfataricus P2]
>gi|15898719|ref|NP_343324.1| hypothetical protein SSO1920 [Sulfolobus solfataricus P2]
>gi|15898752|ref|NP_343357.1| hypothetical protein SSO1956 [Sulfolobus solfataricus P2]
>gi|15898753|ref|NP_343358.1| hypothetical protein SSO1957 [Sulfolobus solfataricus P2]
>gi|15898754|ref|NP_343359.1| Transport membrane protein (permease) [Sulfolobus solfataricus P2]
>gi|15898765|ref|NP_343370.1| hypothetical protein SSO9378 [Sulfolobus solfataricus P2]
>gi|15898805|ref|NP_343410.1| hypothetical protein SSO2014 [Sulfolobus solfataricus P2]
>gi|15898846|ref|NP_343451.1| Sugar transport related protein [Sulfolobus solfataricus P2]
>gi|15898895|ref|NP_343500.1| hypothetical protein SSO2111 [Sulfolobus solfataricus P2]
>gi|15898902|ref|NP_343507.1| hypothetical protein SSO2118 [Sulfolobus solfataricus P2]
>gi|15898929|ref|NP_343534.1| hypothetical protein SSO2146 [Sulfolobus solfataricus P2]
>gi|15898947|ref|NP_343552.1| hypothetical protein SSO2166 [Sulfolobus solfataricus P2]
>gi|15898956|ref|NP_343561.1| hypothetical protein SSO2176 [Sulfolobus solfataricus P2]
>gi|15898962|ref|NP_343567.1| hypothetical protein SSO2183 [Sulfolobus solfataricus P2]
>gi|15898964|ref|NP_343569.1| hypothetical protein SSO2186 [Sulfolobus solfataricus P2]
>gi|15898994|ref|NP_343599.1| hypothetical protein SSO2220 [Sulfolobus solfataricus P2]
>gi|15899013|ref|NP_343618.1| BPS2 protein homolog (bps2) [Sulfolobus solfataricus P2]
>gi|15899022|ref|NP_343627.1| Purine NTPase [Sulfolobus solfataricus P2]
>gi|15899025|ref|NP_343630.1| hypothetical protein SSO2253 [Sulfolobus solfataricus P2]
>gi|15899083|ref|NP_343688.1| hypothetical protein SSO2325 [Sulfolobus solfataricus P2]
>gi|15899096|ref|NP_343701.1| Transport protein, putative [Sulfolobus solfataricus P2]
>gi|15899154|ref|NP_343759.1| hypothetical protein SSO2404 [Sulfolobus solfataricus P2]
>gi|15899155|ref|NP_343760.1| Transcription regulator, putative [Sulfolobus solfataricus P2]
>gi|15899229|ref|NP_343834.1| Quinol oxidase-2, sulfocyanin (blue copper protein), putative (soxE-like) [Sulfolobus solfataricus P2]
>gi|15899230|ref|NP_343835.1| hypothetical protein SSO2489 [Sulfolobus solfataricus P2]
>gi|15899283|ref|NP_343888.1| hypothetical protein SSO2550 [Sulfolobus solfataricus P2]
>gi|15899295|ref|NP_343900.1| hypothetical protein SSO2561 [Sulfolobus solfataricus P2]
>gi|15899299|ref|NP_343904.1| Second ORF in transposon ISC1359 [Sulfolobus solfataricus P2]
>gi|15899319|ref|NP_343924.1| hypothetical protein SSO2590 [Sulfolobus solfataricus P2]
>gi|15899384|ref|NP_343989.1| hypothetical protein SSO2661 [Sulfolobus solfataricus P2]
>gi|15899393|ref|NP_343998.1| ABC transporter, permease protein [Sulfolobus solfataricus P2]
>gi|15899400|ref|NP_344005.1| hypothetical protein SSO2676 [Sulfolobus solfataricus P2]
>gi|15899407|ref|NP_344012.1| hypothetical protein SSO2686 [Sulfolobus solfataricus P2]
>gi|15899486|ref|NP_344091.1| hypothetical protein SSO2772 [Sulfolobus solfataricus P2]
>gi|15899504|ref|NP_344109.1| hypothetical protein SSO2786 [Sulfolobus solfataricus P2]
>gi|15899505|ref|NP_344110.1| Membrane protein, putative [Sulfolobus solfataricus P2]
>gi|15899520|ref|NP_344125.1| Cytochrome b558/566, subunit B (cbsB) [Sulfolobus solfataricus P2]
>gi|15899553|ref|NP_344158.1| ABC transporter, permease (branched chain amino acid) [Sulfolobus solfataricus P2]
>gi|15899554|ref|NP_344159.1| ABC transporter, permease (branched chain amino acid) [Sulfolobus solfataricus P2]
>gi|15899561|ref|NP_344166.1| hypothetical protein SSO2846 [Sulfolobus solfataricus P2]
>gi|15899578|ref|NP_344183.1| hypothetical protein SSO2866 [Sulfolobus solfataricus P2]
>gi|15899597|ref|NP_344202.1| hypothetical protein SSO2886 [Sulfolobus solfataricus P2]
>gi|15899628|ref|NP_344233.1| hypothetical protein SSO2915 [Sulfolobus solfataricus P2]
>gi|15899631|ref|NP_344236.1| Second ORF in transposon ISC1058 [Sulfolobus solfataricus P2]
>gi|15899654|ref|NP_344259.1| hypothetical protein SSO2946 [Sulfolobus solfataricus P2]
>gi|15899675|ref|NP_344280.1| Na(+)/H(+) antiporter, putative [Sulfolobus solfataricus P2]
>gi|15899682|ref|NP_344287.1| Quinol oxidase-2, sulfocyanin (blue copper protein) (soxE) [Sulfolobus solfataricus P2]
>gi|15899694|ref|NP_344299.1| hypothetical protein SSO2984 [Sulfolobus solfataricus P2]

>gi|15899752|ref|NP_344357.1| ABC transporter, permease [Sulfolobus solfataricus P2]
>gi|15899778|ref|NP_344383.1| hypothetical protein SSO3074 [Sulfolobus solfataricus P2]
>gi|15899791|ref|NP_344396.1| hypothetical protein SSO3082 [Sulfolobus solfataricus P2]
>gi|15899808|ref|NP_344413.1| hypothetical protein SSO3099 [Sulfolobus solfataricus P2]
>gi|15899809|ref|NP_344414.1| hypothetical protein SSO3100 [Sulfolobus solfataricus P2]
>gi|15899872|ref|NP_344477.1| hypothetical protein SSO3168 [Sulfolobus solfataricus P2]
>gi|15899879|ref|NP_344484.1| hypothetical protein SSO3176 [Sulfolobus solfataricus P2]
>gi|15899916|ref|NP_344521.1| hypothetical protein SSO12199 [Sulfolobus solfataricus P2]
>gi|15899933|ref|NP_344538.1| Cobalamin synthase related protein [Sulfolobus solfataricus P2]
>gi|27467001|ref|NP_763638.1| hypothetical protein SE0083 [Staphylococcus epidermidis ATCC 12228]
>gi|27467002|ref|NP_763639.1| MERCURIC TRANSPORT PROTEIN [Staphylococcus epidermidis ATCC 12228]
>gi|27467034|ref|NP_763671.1| hypothetical protein SE0116 [Staphylococcus epidermidis ATCC 12228]
>gi|27467037|ref|NP_763674.1| hypothetical protein SE0119 [Staphylococcus epidermidis ATCC 12228]
>gi|27467051|ref|NP_763688.1| hypothetical protein SE0133 [Staphylococcus epidermidis ATCC 12228]
>gi|27467093|ref|NP_763730.1| accumulation-associated protein [Staphylococcus epidermidis ATCC 12228]
>gi|27467111|ref|NP_763748.1| Zn-binding lipoprotein adcA [Staphylococcus epidermidis ATCC 12228]
>gi|27467114|ref|NP_763751.1| transmembrane efflux pump protein [Staphylococcus epidermidis ATCC 12228]
>gi|27467117|ref|NP_763754.1| hypothetical protein SE0199 [Staphylococcus epidermidis ATCC 12228]
>gi|27467131|ref|NP_763768.1| membrane spanning protein [Staphylococcus epidermidis ATCC 12228]
>gi|27467170|ref|NP_763807.1| 2-oxoglutarate/malate translocator-like protein [Staphylococcus epidermidis ATCC 12228]
>gi|27467222|ref|NP_763859.1| 50S ribosomal protein L7/L12 [Staphylococcus epidermidis ATCC 12228]
>gi|27467234|ref|NP_763871.1| hypothetical protein SE0316 [Staphylococcus epidermidis ATCC 12228]
>gi|27467249|ref|NP_763886.1| Ser-Asp rich fibrinogen-binding, bone sialoprotein-binding protein [Staphylococcus epidermidis ATCC 12228]
>gi|27467282|ref|NP_763919.1| hypothetical protein SE0364 [Staphylococcus epidermidis ATCC 12228]
>gi|27467302|ref|NP_763939.1| iron(III) ABC transporter permease protein [Staphylococcus epidermidis ATCC 12228]
>gi|27467320|ref|NP_763957.1| Na+/H+ antiporter [Staphylococcus epidermidis ATCC 12228]
>gi|27467365|ref|NP_764002.1| hypothetical protein SE0447 [Staphylococcus epidermidis ATCC 12228]
>gi|27467370|ref|NP_764007.1| hypothetical protein SE0452 [Staphylococcus epidermidis ATCC 12228]
>gi|27467378|ref|NP_764015.1| hypothetical protein SE0460 [Staphylococcus epidermidis ATCC 12228]
>gi|27467409|ref|NP_764046.1| hypothetical protein SE0491 [Staphylococcus epidermidis ATCC 12228]
>gi|27467417|ref|NP_764054.1| conserved membrane protein [Staphylococcus epidermidis ATCC 12228]
>gi|27467470|ref|NP_764107.1| hypothetical protein SE0552 [Staphylococcus epidermidis ATCC 12228]
>gi|27467472|ref|NP_764109.1| hypothetical protein SE0554 [Staphylococcus epidermidis ATCC 12228]
>gi|27467487|ref|NP_764124.1| hypothetical protein SE0569 [Staphylococcus epidermidis ATCC 12228]
>gi|27467519|ref|NP_764156.1| hypothetical protein SE0601 [Staphylococcus epidermidis ATCC 12228]
>gi|27467523|ref|NP_764160.1| hypothetical protein SE0605 [Staphylococcus epidermidis ATCC 12228]
>gi|27467589|ref|NP_764226.1| hypothetical protein SE0671 [Staphylococcus epidermidis ATCC 12228]
>gi|27467708|ref|NP_764345.1| hypothetical protein SE0790 [Staphylococcus epidermidis ATCC 12228]
>gi|27467719|ref|NP_764356.1| hypothetical protein SE0801 [Staphylococcus epidermidis ATCC 12228]
>gi|27467746|ref|NP_764383.1| lipoprotein VsaC [Staphylococcus epidermidis ATCC 12228]
>gi|27467747|ref|NP_764384.1| hypothetical protein SE0829 [Staphylococcus epidermidis ATCC 12228]
>gi|27467753|ref|NP_764390.1| hypothetical protein SE0835 [Staphylococcus epidermidis ATCC 12228]
>gi|27467855|ref|NP_764492.1| phosphatidate cytidylyltransferase [Staphylococcus epidermidis ATCC 12228]
>gi|27467864|ref|NP_764501.1| ribosome-binding factor A [Staphylococcus epidermidis ATCC 12228]
>gi|27467921|ref|NP_764558.1| hypothetical protein SE1003 [Staphylococcus epidermidis ATCC 12228]
>gi|27467944|ref|NP_764581.1| hypothetical protein SE1026 [Staphylococcus epidermidis ATCC 12228]
>gi|27467987|ref|NP_764624.1| phosphate ABC transporter [Staphylococcus epidermidis ATCC 12228]
>gi|27467995|ref|NP_764632.1| tetrahydrodipicolinate acetyltransferase [Staphylococcus epidermidis ATCC 12228]
>gi|27468001|ref|NP_764638.1| hypothetical protein SE1083 [Staphylococcus epidermidis ATCC 12228]
>gi|27468003|ref|NP_764640.1| hypothetical protein SE1085 [Staphylococcus epidermidis ATCC 12228]
>gi|27468087|ref|NP_764724.1| elastin binding protein [Staphylococcus epidermidis ATCC 12228]
>gi|27468134|ref|NP_764771.1| hypothetical protein SE1216 [Staphylococcus epidermidis ATCC 12228]
>gi|27468146|ref|NP_764783.1| exogenous DNA-binding protein comGC [Staphylococcus epidermidis ATCC 12228]
>gi|27468174|ref|NP_764811.1| diacylglycerol kinase [Staphylococcus epidermidis ATCC 12228]
>gi|27468177|ref|NP_764814.1| hypothetical protein SE1259 [Staphylococcus epidermidis ATCC 12228]
>gi|27468179|ref|NP_764816.1| hypothetical protein SE1261 [Staphylococcus epidermidis ATCC 12228]
>gi|27468180|ref|NP_764817.1| 30S ribosomal protein S21 [Staphylococcus epidermidis ATCC 12228]
>gi|27468186|ref|NP_764823.1| GrpE protein [Staphylococcus epidermidis ATCC 12228]
>gi|27468207|ref|NP_764844.1| acetyl-CoA carboxylase (biotin carboxyl carrier subunit) [Staphylococcus epidermidis ATCC 12228]
>gi|27468216|ref|NP_764853.1| hypothetical protein SE1298 [Staphylococcus epidermidis ATCC 12228]
>gi|27468225|ref|NP_764862.1| hypothetical protein SE1307 [Staphylococcus epidermidis ATCC 12228]
>gi|27468253|ref|NP_764890.1| hypothetical protein SE1335 [Staphylococcus epidermidis ATCC 12228]
>gi|27468255|ref|NP_764892.1| type IV prepilin peptidase [Staphylococcus epidermidis ATCC 12228]
>gi|27468329|ref|NP_764966.1| smooth muscle caldesmon [Staphylococcus epidermidis ATCC 12228]
>gi|27468368|ref|NP_765005.1| hypothetical protein SE1450 [Staphylococcus epidermidis ATCC 12228]
>gi|27468369|ref|NP_765006.1| hypothetical protein SE1451 [Staphylococcus epidermidis ATCC 12228]
>gi|27468415|ref|NP_765052.1| hypothetical protein SE1497 [Staphylococcus epidermidis ATCC 12228]
>gi|27468417|ref|NP_765054.1| hypothetical protein SE1499 [Staphylococcus epidermidis ATCC 12228]
>gi|27468419|ref|NP_765056.1| hypothetical protein SE1501 [Staphylococcus epidermidis ATCC 12228]
>gi|27468420|ref|NP_765057.1| hypothetical protein SE1502 [Staphylococcus epidermidis ATCC 12228]

>gi|27468439|ref|NP_765076.1| prsA [Staphylococcus epidermidis ATCC 12228]
>gi|27468529|ref|NP_765166.1| transposition regulatory protein tnpC [Staphylococcus epidermidis ATCC 12228]
>gi|27468536|ref|NP_765173.1| hypothetical protein SE1618 [Staphylococcus epidermidis ATCC 12228]
>gi|27468537|ref|NP_765174.1| hypothetical protein SE1619 [Staphylococcus epidermidis ATCC 12228]
>gi|27468539|ref|NP_765176.1| hypothetical protein SE1621 [Staphylococcus epidermidis ATCC 12228]
>gi|27468541|ref|NP_765178.1| hypothetical protein SE1623 [Staphylococcus epidermidis ATCC 12228]
>gi|27468546|ref|NP_765183.1| hypothetical protein SE1628 [Staphylococcus epidermidis ATCC 12228]
>gi|27468550|ref|NP_765187.1| Ser-Asp rich fibrinogen-binding protein [Staphylococcus epidermidis ATCC 12228]
>gi|27468596|ref|NP_765233.1| hypothetical protein SE1678 [Staphylococcus epidermidis ATCC 12228]
>gi|27468612|ref|NP_765249.1| SceD precursor [Staphylococcus epidermidis ATCC 12228]
>gi|27468644|ref|NP_765281.1| DNA-directed RNA polymerase subunit delta [Staphylococcus epidermidis ATCC 12228]
>gi|27468691|ref|NP_765328.1| alkaline shock protein 23 [Staphylococcus epidermidis ATCC 12228]
>gi|27468693|ref|NP_765330.1| hypothetical protein SE1775 [Staphylococcus epidermidis ATCC 12228]
>gi|27468711|ref|NP_765348.1| hypothetical protein SE1793 [Staphylococcus epidermidis ATCC 12228]
>gi|27468716|ref|NP_765353.1| 30S ribosomal protein S11 [Staphylococcus epidermidis ATCC 12228]
>gi|27468733|ref|NP_765370.1| 30S ribosomal protein S17 [Staphylococcus epidermidis ATCC 12228]
>gi|27468790|ref|NP_765427.1| ssaA protein [Staphylococcus epidermidis ATCC 12228]
>gi|27468811|ref|NP_765448.1| hypothetical protein SE1893 [Staphylococcus epidermidis ATCC 12228]
>gi|27468833|ref|NP_765470.1| hypothetical protein SE1915 [Staphylococcus epidermidis ATCC 12228]
>gi|27468865|ref|NP_765502.1| TpgX protein [Staphylococcus epidermidis ATCC 12228]
>gi|27468874|ref|NP_765511.1| hypothetical protein SE1956 [Staphylococcus epidermidis ATCC 12228]
>gi|27468905|ref|NP_765542.1| hypothetical protein SE1987 [Staphylococcus epidermidis ATCC 12228]
>gi|27468931|ref|NP_765568.1| holin-like protein LrgA [Staphylococcus epidermidis ATCC 12228]
>gi|27468984|ref|NP_765621.1| hypothetical protein SE2066 [Staphylococcus epidermidis ATCC 12228]
>gi|27468998|ref|NP_765635.1| putative L-serine dehydratase [Staphylococcus epidermidis ATCC 12228]
>gi|27469012|ref|NP_765649.1| hypothetical protein SE2094 [Staphylococcus epidermidis ATCC 12228]
>gi|27469036|ref|NP_765673.1| hypothetical protein SE2118 [Staphylococcus epidermidis ATCC 12228]
>gi|27469042|ref|NP_765679.1| ssaA protein [Staphylococcus epidermidis ATCC 12228]
>gi|27469044|ref|NP_765681.1| immunodominant antigen A [Staphylococcus epidermidis ATCC 12228]
>gi|27469047|ref|NP_765684.1| regulatory protein [Staphylococcus epidermidis ATCC 12228]
>gi|27469067|ref|NP_765704.1| hypothetical protein SE2149 [Staphylococcus epidermidis ATCC 12228]
>gi|27469119|ref|NP_765756.1| hypothetical protein SE2201 [Staphylococcus epidermidis ATCC 12228]
>gi|27469139|ref|NP_765776.1| hypothetical protein SE2221 [Staphylococcus epidermidis ATCC 12228]
>gi|27469167|ref|NP_765804.1| streptococcal hemagglutinin protein [Staphylococcus epidermidis ATCC 12228]
>gi|27469168|ref|NP_765805.1| hypothetical protein SE2250 [Staphylococcus epidermidis ATCC 12228]
>gi|27469169|ref|NP_765806.1| hypothetical protein SE2251 [Staphylococcus epidermidis ATCC 12228]
>gi|27469224|ref|NP_765861.1| hypothetical protein SE2306 [Staphylococcus epidermidis ATCC 12228]
>gi|27469227|ref|NP_765864.1| hypothetical protein SE2309 [Staphylococcus epidermidis ATCC 12228]
>gi|27469239|ref|NP_765876.1| ABC transporter permease protein [Staphylococcus epidermidis ATCC 12228]
>gi|27469252|ref|NP_765889.1| hypothetical protein SE2334 [Staphylococcus epidermidis ATCC 12228]
>gi|27469255|ref|NP_765892.1| regulatory protein PfoR [Staphylococcus epidermidis ATCC 12228]
>gi|27469260|ref|NP_765897.1| hypothetical protein SE2342 [Staphylococcus epidermidis ATCC 12228]
>gi|27469271|ref|NP_765908.1| hypothetical protein SE2353 [Staphylococcus epidermidis ATCC 12228]
>gi|27469280|ref|NP_765917.1| hypothetical protein SE2362 [Staphylococcus epidermidis ATCC 12228]
>gi|27469285|ref|NP_765922.1| hypothetical protein SE2367 [Staphylococcus epidermidis ATCC 12228]
>gi|27469289|ref|NP_765926.1| single-strand DNA-binding protein of phage phi PVL [Staphylococcus epidermidis ATCC 12228]
>gi|27469305|ref|NP_765942.1| hypothetical protein SE2387 [Staphylococcus epidermidis ATCC 12228]
>gi|27469313|ref|NP_765950.1| Ser-Asp rich fibrinogen-binding,bone sialoprotein-binding protein [Staphylococcus epidermidis ATCC 12228]
>gi|27469316|ref|NP_765953.1| hypothetical protein SE2398 [Staphylococcus epidermidis ATCC 12228]
>gi|27469330|ref|NP_765967.1| hypothetical protein SE2412 [Staphylococcus epidermidis ATCC 12228]
>gi|27469337|ref|NP_765974.1| 50S ribosomal protein L34 [Staphylococcus epidermidis ATCC 12228]
>gi|32470522|ref|NP_863194.1| teichoic acid translocation permease protein [Staphylococcus epidermidis ATCC 12228]
>gi|32470537|ref|NP_863208.1| hypothetical protein p505 [Staphylococcus epidermidis ATCC 12228]
>gi|32470550|ref|NP_863221.1| hypothetical protein p518 [Staphylococcus epidermidis ATCC 12228]
>gi|32470561|ref|NP_863231.1| replication-associated protein [Staphylococcus epidermidis ATCC 12228]
>gi|26553457|ref|NP_757391.1| transposase [Mycoplasma penetrans HF-2]
>gi|26553459|ref|NP_757393.1| transposase [Mycoplasma penetrans HF-2]
>gi|26553464|ref|NP_757398.1| hypothetical protein MYPE120 [Mycoplasma penetrans HF-2]
>gi|26553478|ref|NP_757412.1| ribosomal protein L1 [Mycoplasma penetrans HF-2]
>gi|26553485|ref|NP_757419.1| hypothetical protein MYPE330 [Mycoplasma penetrans HF-2]
>gi|26553497|ref|NP_757431.1| hypothetical protein MYPE450 [Mycoplasma penetrans HF-2]
>gi|26553498|ref|NP_757432.1| putative integral membrane protein [Mycoplasma penetrans HF-2]
>gi|26553501|ref|NP_757435.1| hypothetical protein MYPE490 [Mycoplasma penetrans HF-2]
>gi|26553507|ref|NP_757441.1| hypothetical protein MYPE550 [Mycoplasma penetrans HF-2]
>gi|26553510|ref|NP_757444.1| ATP synthase subunit B [Mycoplasma penetrans HF-2]
>gi|26553554|ref|NP_757488.1| ribosomal protein S16 [Mycoplasma penetrans HF-2]
>gi|26553567|ref|NP_757501.1| hypothetical protein MYPE1130 [Mycoplasma penetrans HF-2]
>gi|26553570|ref|NP_757504.1| ribosomal protein S18 [Mycoplasma penetrans HF-2]
>gi|26553591|ref|NP_757525.1| hypothetical protein MYPE1380 [Mycoplasma penetrans HF-2]
>gi|26553597|ref|NP_757531.1| hypothetical protein MYPE1440 [Mycoplasma penetrans HF-2]

>gi|26553605|ref|NP_757539.1| predicted coiled-coil structure containing protein [Mycoplasma penetrans HF-2]
>gi|26553621|ref|NP_757555.1| hypothetical protein MYPE1680 [Mycoplasma penetrans HF-2]
>gi|26553623|ref|NP_757557.1| acetyl transferase [Mycoplasma penetrans HF-2]
>gi|26553641|ref|NP_757575.1| ABC transporter ATP-binding protein [Mycoplasma penetrans HF-2]
>gi|26553659|ref|NP_757593.1| hypothetical protein MYPE2060 [Mycoplasma penetrans HF-2]
>gi|26553668|ref|NP_757602.1| hypothetical protein MYPE2150 [Mycoplasma penetrans HF-2]
>gi|26553687|ref|NP_757621.1| predicted integral membrane protein [Mycoplasma penetrans HF-2]
>gi|26553688|ref|NP_757622.1| predicted integral membrane protein [Mycoplasma penetrans HF-2]
>gi|26553689|ref|NP_757623.1| predicted integral membrane protein [Mycoplasma penetrans HF-2]
>gi|26553691|ref|NP_757625.1| hypothetical protein MYPE2390 [Mycoplasma penetrans HF-2]
>gi|26553701|ref|NP_757635.1| DNA-binding histone-like protein HU-alpha [Mycoplasma penetrans HF-2]
>gi|26553702|ref|NP_757636.1| predicted integral membrane protein [Mycoplasma penetrans HF-2]
>gi|26553752|ref|NP_757686.1| hypothetical protein MYPE2980 [Mycoplasma penetrans HF-2]
>gi|26553757|ref|NP_757691.1| ABC transporter ATP-binding protein [Mycoplasma penetrans HF-2]
>gi|26553770|ref|NP_757704.1| hypothetical protein MYPE3155 [Mycoplasma penetrans HF-2]
>gi|26553782|ref|NP_757716.1| large-conductance mechanosensitive channel [Mycoplasma penetrans HF-2]
>gi|26553792|ref|NP_757726.1| GTP-binding protein Era homolog [Mycoplasma penetrans HF-2]
>gi|26553796|ref|NP_757730.1| hypothetical protein MYPE3410 [Mycoplasma penetrans HF-2]
>gi|26553797|ref|NP_757731.1| hypothetical protein MYPE3420 [Mycoplasma penetrans HF-2]
>gi|26553798|ref|NP_757732.1| hypothetical protein MYPE3430 [Mycoplasma penetrans HF-2]
>gi|26553802|ref|NP_757736.1| ABC transporter ATP-binding protein [Mycoplasma penetrans HF-2]
>gi|26553805|ref|NP_757739.1| hypothetical protein MYPE3520 [Mycoplasma penetrans HF-2]
>gi|26553831|ref|NP_757765.1| hypothetical protein MYPE3800 [Mycoplasma penetrans HF-2]
>gi|26553832|ref|NP_757766.1| hypothetical protein MYPE3810 [Mycoplasma penetrans HF-2]
>gi|26553877|ref|NP_757811.1| hypothetical protein MYPE4250 [Mycoplasma penetrans HF-2]
>gi|26553884|ref|NP_757818.1| putative integral membrane protein [Mycoplasma penetrans HF-2]
>gi|26553925|ref|NP_757859.1| hypothetical protein MYPE4740 [Mycoplasma penetrans HF-2]
>gi|26553926|ref|NP_757860.1| hypothetical protein MYPE4750 [Mycoplasma penetrans HF-2]
>gi|26553927|ref|NP_757861.1| hypothetical protein MYPE4760 [Mycoplasma penetrans HF-2]
>gi|26553928|ref|NP_757862.1| hypothetical protein MYPE4765 [Mycoplasma penetrans HF-2]
>gi|26553938|ref|NP_757872.1| hypothetical protein MYPE4860 [Mycoplasma penetrans HF-2]
>gi|26553944|ref|NP_757878.1| hypothetical protein MYPE4920 [Mycoplasma penetrans HF-2]
>gi|26553950|ref|NP_757884.1| hypothetical protein MYPE4980 [Mycoplasma penetrans HF-2]
>gi|26553952|ref|NP_757886.1| hypothetical protein MYPE5000 [Mycoplasma penetrans HF-2]
>gi|26553970|ref|NP_757904.1| hypothetical protein MYPE5180 [Mycoplasma penetrans HF-2]
>gi|26553977|ref|NP_757911.1| hypothetical protein MYPE5250 [Mycoplasma penetrans HF-2]
>gi|26553979|ref|NP_757913.1| hypothetical protein MYPE5270 [Mycoplasma penetrans HF-2]
>gi|26553982|ref|NP_757916.1| hypothetical protein MYPE5300 [Mycoplasma penetrans HF-2]
>gi|26553985|ref|NP_757919.1| hypothetical protein MYPE5340 [Mycoplasma penetrans HF-2]
>gi|26553988|ref|NP_757922.1| hypothetical protein MYPE5370 [Mycoplasma penetrans HF-2]
>gi|26553990|ref|NP_757924.1| hypothetical protein MYPE5390 [Mycoplasma penetrans HF-2]
>gi|26553994|ref|NP_757928.1| hypothetical protein MYPE5430 [Mycoplasma penetrans HF-2]
>gi|26554019|ref|NP_757953.1| hypothetical protein MYPE5670 [Mycoplasma penetrans HF-2]
>gi|26554020|ref|NP_757954.1| hypothetical protein MYPE5680 [Mycoplasma penetrans HF-2]
>gi|26554025|ref|NP_757959.1| ribosomal protein L7/L12 [Mycoplasma penetrans HF-2]
>gi|26554030|ref|NP_757964.1| hypothetical protein MYPE5780 [Mycoplasma penetrans HF-2]
>gi|26554036|ref|NP_757970.1| hypothetical protein MYPE5840 [Mycoplasma penetrans HF-2]
>gi|26554056|ref|NP_757990.1| hypothetical protein MYPE6040 [Mycoplasma penetrans HF-2]
>gi|26554066|ref|NP_758000.1| hypothetical protein MYPE6140 [Mycoplasma penetrans HF-2]
>gi|26554074|ref|NP_758008.1| hypothetical protein MYPE6220 [Mycoplasma penetrans HF-2]
>gi|26554093|ref|NP_758027.1| hypothetical protein MYPE6410 [Mycoplasma penetrans HF-2]
>gi|26554099|ref|NP_758033.1| DNA topoisomerase IV subunit A [Mycoplasma penetrans HF-2]
>gi|26554114|ref|NP_758048.1| transport system permease [Mycoplasma penetrans HF-2]
>gi|26554133|ref|NP_758067.1| P35 lipoprotein homolog [Mycoplasma penetrans HF-2]
>gi|26554136|ref|NP_758070.1| P35 lipoprotein homolog reported as IMP14 [Mycoplasma penetrans HF-2]
>gi|26554139|ref|NP_758073.1| putative lipoprotein [Mycoplasma penetrans HF-2]
>gi|26554149|ref|NP_758083.1| hypothetical protein MYPE6950 [Mycoplasma penetrans HF-2]
>gi|26554152|ref|NP_758086.1| hypothetical protein MYPE6980 [Mycoplasma penetrans HF-2]
>gi|26554191|ref|NP_758125.1| hypothetical protein MYPE7350 [Mycoplasma penetrans HF-2]
>gi|26554195|ref|NP_758129.1| P35 lipoprotein homolog fragment [Mycoplasma penetrans HF-2]
>gi|26554204|ref|NP_758138.1| hypothetical protein MYPE7480 [Mycoplasma penetrans HF-2]
>gi|26554212|ref|NP_758146.1| hypothetical protein MYPE7560 [Mycoplasma penetrans HF-2]
>gi|26554225|ref|NP_758159.1| putative regulatory protein [Mycoplasma penetrans HF-2]
>gi|26554253|ref|NP_758187.1| hypothetical protein MYPE7980 [Mycoplasma penetrans HF-2]
>gi|26554255|ref|NP_758189.1| hypothetical protein MYPE8000 [Mycoplasma penetrans HF-2]
>gi|26554262|ref|NP_758196.1| hypothetical protein MYPE8070 [Mycoplasma penetrans HF-2]
>gi|26554263|ref|NP_758197.1| DNA polymerase III gamma-tau subunits [Mycoplasma penetrans HF-2]
>gi|26554267|ref|NP_758201.1| thiophene and furan oxidation protein-related GTPase [Mycoplasma penetrans HF-2]
>gi|26554281|ref|NP_758215.1| hypothetical protein MYPE8270 [Mycoplasma penetrans HF-2]
>gi|26554282|ref|NP_758216.1| hypothetical protein MYPE8280 [Mycoplasma penetrans HF-2]

>gi|26554283|ref|NP_758217.1| hypothetical protein MYPE8290 [Mycoplasma penetrans HF-2]
>gi|26554289|ref|NP_758223.1| hypothetical protein MYPE8350 [Mycoplasma penetrans HF-2]
>gi|26554293|ref|NP_758227.1| hypothetical protein MYPE8390 [Mycoplasma penetrans HF-2]
>gi|26554301|ref|NP_758235.1| hypothetical protein MYPE8470 [Mycoplasma penetrans HF-2]
>gi|26554302|ref|NP_758236.1| hypothetical protein MYPE8480 [Mycoplasma penetrans HF-2]
>gi|26554317|ref|NP_758251.1| hypothetical protein MYPE8630 [Mycoplasma penetrans HF-2]
>gi|26554321|ref|NP_758255.1| hypothetical protein MYPE8670 [Mycoplasma penetrans HF-2]
>gi|26554332|ref|NP_758266.1| hypothetical protein MYPE8790 [Mycoplasma penetrans HF-2]
>gi|26554337|ref|NP_758271.1| transcription antitermination factor [Mycoplasma penetrans HF-2]
>gi|26554338|ref|NP_758272.1| predicted preprotein translocase subunit SecE [Mycoplasma penetrans HF-2]
>gi|26554368|ref|NP_758302.1| translation initiation factor IF3 [Mycoplasma penetrans HF-2]
>gi|26554370|ref|NP_758304.1| ribosomal protein S21 [Mycoplasma penetrans HF-2]
>gi|26554373|ref|NP_758307.1| hypothetical protein MYPE9240 [Mycoplasma penetrans HF-2]
>gi|26554381|ref|NP_758315.1| hypothetical protein MYPE9320 [Mycoplasma penetrans HF-2]
>gi|26554401|ref|NP_758335.1| hypothetical protein MYPE9520 [Mycoplasma penetrans HF-2]
>gi|26554408|ref|NP_758342.1| hypothetical protein MYPE9600 [Mycoplasma penetrans HF-2]
>gi|26554409|ref|NP_758343.1| hypothetical protein MYPE9610 [Mycoplasma penetrans HF-2]
>gi|26554420|ref|NP_758354.1| hypothetical protein MYPE9720 [Mycoplasma penetrans HF-2]
>gi|26554447|ref|NP_758381.1| ribosomal protein L15 [Mycoplasma penetrans HF-2]
>gi|26554448|ref|NP_758382.1| ribosomal protein S5 [Mycoplasma penetrans HF-2]
>gi|26554458|ref|NP_758392.1| ribosomal protein L29 [Mycoplasma penetrans HF-2]
>gi|26554460|ref|NP_758394.1| ribosomal protein L22 [Mycoplasma penetrans HF-2]
>gi|26554463|ref|NP_758397.1| ribosomal protein L23 [Mycoplasma penetrans HF-2]
>gi|26554465|ref|NP_758399.1| ribosomal protein L3 [Mycoplasma penetrans HF-2]
>gi|26554471|ref|NP_758405.1| predicted protein-export membrane protein SecD [Mycoplasma penetrans HF-2]
>gi|26554482|ref|NP_758416.1| DNA-directed RNA polymerase subunit delta [Mycoplasma penetrans HF-2]
>gi|26554485|ref|NP_758419.1| putative GHMP kinases ATP-binding protein [Mycoplasma penetrans HF-2]
>gi|16082715|ref|NP_395161.1| secreted effector protein [Yersinia pestis CO92]
>gi|16082723|ref|NP_395169.1| hypothetical protein YPCD1.35c [Yersinia pestis CO92]
>gi|16082775|ref|NP_395221.1| hypothetical protein YPCD1.92 [Yersinia pestis CO92]
>gi|40787862|ref|NP_395340.2| putative phage tail protein [Yersinia pestis CO92]
>gi|16082804|ref|NP_395358.1| hypothetical protein YPMT1.22c [Yersinia pestis CO92]
>gi|16082843|ref|NP_395397.1| hypothetical protein YPMT1.52c [Yersinia pestis CO92]
>gi|16082876|ref|NP_395430.1| F1 capsule antigen [Yersinia pestis CO92]
>gi|16082880|ref|NP_395434.1| porphyrin biosynthetic protein [Yersinia pestis CO92]
>gi|16120414|ref|NP_403727.1| putative membrane protein [Yersinia pestis CO92]
>gi|16120447|ref|NP_403760.1| hypothetical protein YPO0100 [Yersinia pestis CO92]
>gi|16120454|ref|NP_403767.1| cell division protein [Yersinia pestis CO92]
>gi|16120490|ref|NP_403803.1| putative membrane protein [Yersinia pestis CO92]
>gi|16120532|ref|NP_403845.1| peptidyl-prolyl cis-trans isomerase [Yersinia pestis CO92]
>gi|16120538|ref|NP_403851.1| hypothetical protein YPO0199 [Yersinia pestis CO92]
>gi|16120547|ref|NP_403860.1| 50S ribosomal protein L3 [Yersinia pestis CO92]
>gi|16120555|ref|NP_403868.1| 50S ribosomal protein L29 [Yersinia pestis CO92]
>gi|16120602|ref|NP_403915.1| hypothetical protein YPO0264 [Yersinia pestis CO92]
>gi|16120603|ref|NP_403916.1| putative type III secretion system apparatus protein [Yersinia pestis CO92]
>gi|16120606|ref|NP_403919.1| type III secretion system apparatus protein [Yersinia pestis CO92]
>gi|16120619|ref|NP_403932.1| hemin transport system permease protein HmuU [Yersinia pestis CO92]
>gi|16120655|ref|NP_403968.1| hypothetical protein YPO0318 [Yersinia pestis CO92]
>gi|16120662|ref|NP_403975.1| single-strand binding protein [Yersinia pestis CO92]
>gi|16120672|ref|NP_403985.1| hypothetical protein YPO0335 [Yersinia pestis CO92]
>gi|16120684|ref|NP_403997.1| putative membrane protein [Yersinia pestis CO92]
>gi|16120690|ref|NP_404003.1| putative chaperone [Yersinia pestis CO92]
>gi|16120710|ref|NP_404023.1| putative membrane protein [Yersinia pestis CO92]
>gi|16120745|ref|NP_404058.1| putative ABC transporter permease protein [Yersinia pestis CO92]
>gi|16120747|ref|NP_404060.1| putative membrane protein [Yersinia pestis CO92]
>gi|16120759|ref|NP_404072.1| putative membrane protein [Yersinia pestis CO92]
>gi|16120768|ref|NP_404081.1| putative Na+ dependent nucleoside transporter-family protein [Yersinia pestis CO92]
>gi|16120798|ref|NP_404111.1| chaperone protein DnaJ [Yersinia pestis CO92]
>gi|16120837|ref|NP_404150.1| hypothetical protein YPO0507 [Yersinia pestis CO92]
>gi|16120896|ref|NP_404209.1| putative membrane protein [Yersinia pestis CO92]
>gi|16120898|ref|NP_404211.1| hypothetical protein YPO0569a [Yersinia pestis CO92]
>gi|16120901|ref|NP_404214.1| hypothetical protein YPO0572 [Yersinia pestis CO92]
>gi|16120919|ref|NP_404232.1| putative membrane protein [Yersinia pestis CO92]
>gi|16121003|ref|NP_404316.1| putative DedA-family membrane protein [Yersinia pestis CO92]
>gi|16121041|ref|NP_404354.1| putative flagellar basal-body rod protein [Yersinia pestis CO92]
>gi|16121061|ref|NP_404374.1| putative flagellar hook-length control protein [Yersinia pestis CO92]
>gi|16121199|ref|NP_404512.1| putative membrane protein [Yersinia pestis CO92]
>gi|16121207|ref|NP_404520.1| hypothetical protein YPO0901 [Yersinia pestis CO92]
>gi|16121208|ref|NP_404521.1| putative surface protein (partial) [Yersinia pestis CO92]
>gi|16121247|ref|NP_404560.1| putative membrane protein [Yersinia pestis CO92]

>gi|16121253|ref|NP_404566.1| hypothetical protein YPO0950 [Yersinia pestis CO92]
>gi|16121292|ref|NP_404605.1| putative membrane protein [Yersinia pestis CO92]
>gi|16121306|ref|NP_404619.1| putative autotransporter protein [Yersinia pestis CO92]
>gi|16121308|ref|NP_404621.1| putative antigenic leucine-rich repeat protein [Yersinia pestis CO92]
>gi|16121318|ref|NP_404631.1| putative prepilin peptidase dependent protein [Yersinia pestis CO92]
>gi|16121330|ref|NP_404643.1| hypothetical protein YPO1030 [Yersinia pestis CO92]
>gi|16121354|ref|NP_404667.1| UDP-3-o-[3-hydroxymyristoyl] glucosamine N-acyltransferase [Yersinia pestis CO92]
>gi|16121423|ref|NP_404736.1| TolA colicin import membrane protein [Yersinia pestis CO92]
>gi|16121426|ref|NP_404739.1| hypothetical protein YPO1126 [Yersinia pestis CO92]
>gi|16121437|ref|NP_404750.1| putative membrane protein [Yersinia pestis CO92]
>gi|16121469|ref|NP_404782.1| hypothetical protein YPO1174 [Yersinia pestis CO92]
>gi|16121476|ref|NP_404789.1| putative membrane protein [Yersinia pestis CO92]
>gi|16121483|ref|NP_404796.1| putative Branched-chain amino acid transport system, permease component [Yersinia pestis CO92]
>gi|16121484|ref|NP_404797.1| putative Branched-chain amino acid transport system, permease component [Yersinia pestis CO92]
>gi|16121490|ref|NP_404803.1| putative ABC transport integral membrane subunit [Yersinia pestis CO92]
>gi|16121667|ref|NP_404980.1| hypothetical protein YPO1387 [Yersinia pestis CO92]
>gi|16121668|ref|NP_404981.1| hypothetical protein YPO1388 [Yersinia pestis CO92]
>gi|16121702|ref|NP_405015.1| putative lipoprotein protein [Yersinia pestis CO92]
>gi|16121714|ref|NP_405027.1| putative cell division inhibitor [Yersinia pestis CO92]
>gi|16121761|ref|NP_405074.1| hypothetical protein YPO1488 [Yersinia pestis CO92]
>gi|16121808|ref|NP_405121.1| putative iron-siderophore transport system, transmembrane component [Yersinia pestis CO92]
>gi|16121838|ref|NP_405151.1| hypothetical protein YPO1568 [Yersinia pestis CO92]
>gi|16121858|ref|NP_405171.1| ribonuclease E [Yersinia pestis CO92]
>gi|16121874|ref|NP_405187.1| DNA polymerase III, delta' subunit [Yersinia pestis CO92]
>gi|16121881|ref|NP_405194.1| putative lipoprotein [Yersinia pestis CO92]
>gi|16121892|ref|NP_405205.1| hypothetical protein YPO1624 [Yersinia pestis CO92]
>gi|16121929|ref|NP_405242.1| chemotaxis MotB protein [Yersinia pestis CO92]
>gi|16121960|ref|NP_405273.1| hypothetical protein YPO1700 [Yersinia pestis CO92]
>gi|16121964|ref|NP_405277.1| ProP effector homologue [Yersinia pestis CO92]
>gi|16121967|ref|NP_405280.1| putative fimbrial protein [Yersinia pestis CO92]
>gi|16121997|ref|NP_405310.1| hypothetical protein YPO1740 [Yersinia pestis CO92]
>gi|16122001|ref|NP_405314.1| hypothetical protein YPO1744 [Yersinia pestis CO92]
>gi|16122072|ref|NP_405385.1| flagellar biosynthetic protein FliQ [Yersinia pestis CO92]
>gi|16122078|ref|NP_405391.1| flagellar hook-length control protein FliK [Yersinia pestis CO92]
>gi|16122163|ref|NP_405476.1| putative signal transducer [Yersinia pestis CO92]
>gi|16122279|ref|NP_405592.1| putative membrane protein [Yersinia pestis CO92]
>gi|16122364|ref|NP_405677.1| putative phage tail assembly protein [Yersinia pestis CO92]
>gi|16122384|ref|NP_405697.1| putative membrane protein [Yersinia pestis CO92]
>gi|16122406|ref|NP_405719.1| DNA-binding protein Hns [Yersinia pestis CO92]
>gi|16122423|ref|NP_405736.1| TonB protein [Yersinia pestis CO92]
>gi|16122458|ref|NP_405771.1| osmotically inducible lipoprotein B precursor [Yersinia pestis CO92]
>gi|16122468|ref|NP_405781.1| putative membrane protein [Yersinia pestis CO92]
>gi|16122472|ref|NP_405785.1| putative iron-sulfur binding NADH dehydrogenase [Yersinia pestis CO92]
>gi|16122475|ref|NP_405788.1| putative membrane protein [Yersinia pestis CO92]
>gi|16122502|ref|NP_405815.1| putative phage-related membrane protein [Yersinia pestis CO92]
>gi|16122542|ref|NP_405855.1| putative acid shock protein [Yersinia pestis CO92]
>gi|16122555|ref|NP_405868.1| putative lipoprotein [Yersinia pestis CO92]
>gi|16122578|ref|NP_405891.1| hypothetical protein YPO2354 [Yersinia pestis CO92]
>gi|16122596|ref|NP_405909.1| putative lipoprotein [Yersinia pestis CO92]
>gi|16122608|ref|NP_405921.1| hypothetical protein YPO2385 [Yersinia pestis CO92]
>gi|16122617|ref|NP_405930.1| major outer membrane lipoprotein [Yersinia pestis CO92]
>gi|16122636|ref|NP_405949.1| putative membrane protein [Yersinia pestis CO92]
>gi|16122646|ref|NP_405959.1| hypothetical protein YPO2426 [Yersinia pestis CO92]
>gi|16122652|ref|NP_405965.1| 50S ribosomal protein L35 [Yersinia pestis CO92]
>gi|16122670|ref|NP_405983.1| putative LuxR-family regulatory protein [Yersinia pestis CO92]
>gi|16122690|ref|NP_406003.1| hypothetical protein YPO2469 [Yersinia pestis CO92]
>gi|16122764|ref|NP_406077.1| NADH dehydrogenase i chain k [Yersinia pestis CO92]
>gi|16122796|ref|NP_406109.1| sugar transport system permease protein [Yersinia pestis CO92]
>gi|16122810|ref|NP_406123.1| sec-independent protein translocase protein [Yersinia pestis CO92]
>gi|16122861|ref|NP_406174.1| putative acid shock protein [Yersinia pestis CO92]
>gi|16122892|ref|NP_406205.1| putative membrane protein [Yersinia pestis CO92]
>gi|16122904|ref|NP_406217.1| hypothetical protein YPO2700 [Yersinia pestis CO92]
>gi|16122918|ref|NP_406231.1| sigma E factor regulatory protein [Yersinia pestis CO92]
>gi|16122929|ref|NP_406242.1| hypothetical protein YPO2725 [Yersinia pestis CO92]
>gi|16122935|ref|NP_406248.1| putative membrane protein [Yersinia pestis CO92]
>gi|16122939|ref|NP_406252.1| heme exporter protein B [Yersinia pestis CO92]
>gi|16122941|ref|NP_406254.1| putative heme exporter protein D [Yersinia pestis CO92]
>gi|16122957|ref|NP_406270.1| putative membrane protein [Yersinia pestis CO92]
>gi|16122974|ref|NP_406287.1| putative membrane protein [Yersinia pestis CO92]
>gi|16122984|ref|NP_406297.1| hypothetical protein YPO2780 [Yersinia pestis CO92]

>gi|16123056|ref|NP_406369.1| putative membrane protein [Yersinia pestis CO92]
>gi|16123059|ref|NP_406372.1| putative membrane protein [Yersinia pestis CO92]
>gi|16123064|ref|NP_406377.1| exodeoxyribonuclease VII large subunit [Yersinia pestis CO92]
>gi|16123078|ref|NP_406391.1| putative autotransporter protein [Yersinia pestis CO92]
>gi|16123103|ref|NP_406416.1| putative lipoprotein [Yersinia pestis CO92]
>gi|16123111|ref|NP_406424.1| putative membrane protein [Yersinia pestis CO92]
>gi|16123144|ref|NP_406457.1| hypothetical protein YPO2962 [Yersinia pestis CO92]
>gi|16123162|ref|NP_406475.1| hypothetical protein YPO2981 [Yersinia pestis CO92]
>gi|16123195|ref|NP_406508.1| putative sugar transporter [Yersinia pestis CO92]
>gi|16123204|ref|NP_406517.1| hypothetical protein YPO3027 [Yersinia pestis CO92]
>gi|16123222|ref|NP_406535.1| putative membrane protein [Yersinia pestis CO92]
>gi|16123234|ref|NP_406547.1| putative membrane protein [Yersinia pestis CO92]
>gi|16123255|ref|NP_406568.1| putative permease [Yersinia pestis CO92]
>gi|16123259|ref|NP_406572.1| putative thioredoxin [Yersinia pestis CO92]
>gi|16123260|ref|NP_406573.1| hypothetical protein YPO3083 [Yersinia pestis CO92]
>gi|16123261|ref|NP_406574.1| putative membrane protein [Yersinia pestis CO92]
>gi|16123426|ref|NP_406739.1| putative tRNA/rRNA methyltransferase [Yersinia pestis CO92]
>gi|16123455|ref|NP_406768.1| putative membrane protein [Yersinia pestis CO92]
>gi|16123459|ref|NP_406772.1| putative regulatory protein [Yersinia pestis CO92]
>gi|16123484|ref|NP_406797.1| putative sugar ABC transporter, permease protein [Yersinia pestis CO92]
>gi|16123524|ref|NP_406837.1| superoxide dismutase [Cu-Zn] precursor [Yersinia pestis CO92]
>gi|16123537|ref|NP_406850.1| putative chloride channel protein [Yersinia pestis CO92]
>gi|16123539|ref|NP_406852.1| ferrichrome transport system permease protein FhuB [Yersinia pestis CO92]
>gi|16123567|ref|NP_406880.1| dihydrolipoamide acetyltransferase component of pyruvate dehydrogenase complex [Yersinia pestis CO92]
>gi|16123599|ref|NP_406912.1| putative ABC transporter, ATP-binding protein [Yersinia pestis CO92]
>gi|16123634|ref|NP_406947.1| cold-shock dead-box protein A [Yersinia pestis CO92]
>gi|16123645|ref|NP_406958.1| protein-export membrane protein [Yersinia pestis CO92]
>gi|16123788|ref|NP_407101.1| outer membrane lipoprotein [Yersinia pestis CO92]
>gi|16123802|ref|NP_407115.1| biotin carboxyl carrier protein of acetyl-CoA carboxylase [Yersinia pestis CO92]
>gi|16123804|ref|NP_407117.1| putative membrane protein [Yersinia pestis CO92]
>gi|16123818|ref|NP_407131.1| hypothetical protein YPO3675 [Yersinia pestis CO92]
>gi|16123885|ref|NP_407198.1| 50S ribosomal protein L7/L12 [Yersinia pestis CO92]
>gi|16123886|ref|NP_407199.1| 50S ribosomal protein L10 [Yersinia pestis CO92]
>gi|16123890|ref|NP_407203.1| preprotein translocase SecE subunit [Yersinia pestis CO92]
>gi|16123911|ref|NP_407224.1| Sec-independent protein translocase protein TatB [Yersinia pestis CO92]
>gi|16123916|ref|NP_407229.1| putative DNA recombination protein [Yersinia pestis CO92]
>gi|16123917|ref|NP_407230.1| putative membrane protein [Yersinia pestis CO92]
>gi|16123940|ref|NP_407253.1| high-affinity branched-chain amino acid transport system, permease protein [Yersinia pestis CO92]
>gi|16123948|ref|NP_407261.1| cell division protein [Yersinia pestis CO92]
>gi|16123952|ref|NP_407265.1| putative membrane protein [Yersinia pestis CO92]
>gi|16123954|ref|NP_407267.1| putative membrane protein [Yersinia pestis CO92]
>gi|16123986|ref|NP_407299.1| putative uroporphyrin-III C-methyltransferase [Yersinia pestis CO92]
>gi|16124037|ref|NP_407350.1| putative sugar transport system permease [Yersinia pestis CO92]
>gi|16124078|ref|NP_407391.1| putative membrane protein [Yersinia pestis CO92]
>gi|16124106|ref|NP_407419.1| GntP family permease [Yersinia pestis CO92]
>gi|16124126|ref|NP_407439.1| dipeptide transport system permease protein [Yersinia pestis CO92]
>gi|16124143|ref|NP_407456.1| putative membrane protein [Yersinia pestis CO92]
>gi|16124167|ref|NP_407480.1| putative membrane protein [Yersinia pestis CO92]
>gi|16124188|ref|NP_407501.1| putative lipoprotein [Yersinia pestis CO92]
>gi|16124213|ref|NP_407526.1| hypothetical protein YPO4105 [Yersinia pestis CO92]
>gi|16124233|ref|NP_407546.1| ATP synthase subunit B protein [Yersinia pestis CO92]
>gi|16124234|ref|NP_407547.1| ATP synthase subunit C protein [Yersinia pestis CO92]
>gi|52421253|ref|YP_087165.1| hypothetical protein BFp0039 [Bacteroides fragilis YCH46]
>gi|52421260|ref|YP_087172.1| hypothetical protein BFp0046 [Bacteroides fragilis YCH46]
>gi|53711448|ref|YP_097440.1| hypothetical protein BF0157 [Bacteroides fragilis YCH46]
>gi|53711493|ref|YP_097485.1| hypothetical protein BF0202 [Bacteroides fragilis YCH46]
>gi|53711535|ref|YP_097527.1| hypothetical protein BF0244 [Bacteroides fragilis YCH46]
>gi|53711554|ref|YP_097546.1| translation initiation factor IF-2 [Bacteroides fragilis YCH46]
>gi|53711836|ref|YP_097828.1| hypothetical protein BF0545 [Bacteroides fragilis YCH46]
>gi|53711888|ref|YP_097880.1| ferredoxin [Bacteroides fragilis YCH46]
>gi|53711950|ref|YP_097942.1| hypothetical protein BF0660 [Bacteroides fragilis YCH46]
>gi|53711975|ref|YP_097967.1| hypothetical protein BF0686 [Bacteroides fragilis YCH46]
>gi|53711979|ref|YP_097971.1| hypothetical protein BF0690 [Bacteroides fragilis YCH46]
>gi|53712008|ref|YP_098000.1| hypothetical protein BF0719 [Bacteroides fragilis YCH46]
>gi|53712014|ref|YP_098006.1| zinc ABC transporter permease [Bacteroides fragilis YCH46]
>gi|53712024|ref|YP_098016.1| hypothetical protein BF0731.4 [Bacteroides fragilis YCH46]
>gi|53712095|ref|YP_098087.1| hypothetical protein BF0802 [Bacteroides fragilis YCH46]
>gi|53712214|ref|YP_098206.1| flotillin-like protein [Bacteroides fragilis YCH46]
>gi|53712260|ref|YP_098252.1| putative ion channel [Bacteroides fragilis YCH46]
>gi|53712277|ref|YP_098269.1| hypothetical protein BF0985 [Bacteroides fragilis YCH46]

>gi|53712303|ref|YP_098295.1| hypothetical protein BF1011 [Bacteroides fragilis YCH46]
>gi|53712320|ref|YP_098312.1| hypothetical protein BF1028 [Bacteroides fragilis YCH46]
>gi|53712358|ref|YP_098350.1| hypothetical protein BF1066 [Bacteroides fragilis YCH46]
>gi|53712363|ref|YP_098355.1| hypothetical protein BF1071 [Bacteroides fragilis YCH46]
>gi|53712482|ref|YP_098474.1| hypothetical protein BF1190 [Bacteroides fragilis YCH46]
>gi|53712485|ref|YP_098477.1| hypothetical protein BF1193 [Bacteroides fragilis YCH46]
>gi|53712657|ref|YP_098649.1| hypothetical protein BF1364.4 [Bacteroides fragilis YCH46]
>gi|53712712|ref|YP_098704.1| hypothetical protein BF1419 [Bacteroides fragilis YCH46]
>gi|53712806|ref|YP_098798.1| hypothetical protein BF1513 [Bacteroides fragilis YCH46]
>gi|53712837|ref|YP_098829.1| probable O-antigen polymerase [Bacteroides fragilis YCH46]
>gi|53712922|ref|YP_098914.1| hypothetical protein BF1632 [Bacteroides fragilis YCH46]
>gi|53712948|ref|YP_098940.1| hypothetical protein BF1658 [Bacteroides fragilis YCH46]
>gi|53712952|ref|YP_098944.1| hypothetical protein BF1662 [Bacteroides fragilis YCH46]
>gi|53713031|ref|YP_099023.1| chaperone protein DnaJ [Bacteroides fragilis YCH46]
>gi|53713109|ref|YP_099101.1| hypothetical protein BF1820 [Bacteroides fragilis YCH46]
>gi|53713131|ref|YP_099123.1| putative polymerase [Bacteroides fragilis YCH46]
>gi|53713187|ref|YP_099179.1| OmpA family outer membrane protein [Bacteroides fragilis YCH46]
>gi|53713208|ref|YP_099200.1| hypothetical protein BF1918 [Bacteroides fragilis YCH46]
>gi|53713253|ref|YP_099245.1| hypothetical protein BF1963 [Bacteroides fragilis YCH46]
>gi|53713464|ref|YP_099456.1| ATP synthase C subunit [Bacteroides fragilis YCH46]
>gi|53713498|ref|YP_099490.1| hypothetical protein BF2209 [Bacteroides fragilis YCH46]
>gi|53713536|ref|YP_099528.1| hypothetical protein BF2247 [Bacteroides fragilis YCH46]
>gi|53713573|ref|YP_099565.1| hypothetical protein BF2284 [Bacteroides fragilis YCH46]
>gi|53713577|ref|YP_099569.1| hypothetical protein BF2288 [Bacteroides fragilis YCH46]
>gi|53713603|ref|YP_099595.1| hypothetical protein BF2314 [Bacteroides fragilis YCH46]
>gi|53713635|ref|YP_099627.1| hypothetical protein BF2345.1 [Bacteroides fragilis YCH46]
>gi|53713681|ref|YP_099673.1| hypothetical protein BF2390 [Bacteroides fragilis YCH46]
>gi|53713708|ref|YP_099700.1| conserved hypothetical protein BatC [Bacteroides fragilis YCH46]
>gi|53713714|ref|YP_099706.1| putative integration host factor IHF alpha subunit [Bacteroides fragilis YCH46]
>gi|53713735|ref|YP_099727.1| hypothetical protein BF2444 [Bacteroides fragilis YCH46]
>gi|53713772|ref|YP_099764.1| hypothetical protein BF2481 [Bacteroides fragilis YCH46]
>gi|53713941|ref|YP_099933.1| hypothetical protein BF2649 [Bacteroides fragilis YCH46]
>gi|53714008|ref|YP_100000.1| 30S ribosomal protein S16 [Bacteroides fragilis YCH46]
>gi|53714011|ref|YP_100003.1| putative RNA-binding protein [Bacteroides fragilis YCH46]
>gi|53714040|ref|YP_100032.1| hypothetical protein BF2748 [Bacteroides fragilis YCH46]
>gi|53714054|ref|YP_100046.1| hypothetical protein BF2762 [Bacteroides fragilis YCH46]
>gi|53714056|ref|YP_100048.1| hypothetical protein BF2764 [Bacteroides fragilis YCH46]
>gi|53714152|ref|YP_100144.1| hypothetical protein BF2861 [Bacteroides fragilis YCH46]
>gi|53714172|ref|YP_100164.1| hypothetical protein BF2881 [Bacteroides fragilis YCH46]
>gi|53714178|ref|YP_100170.1| hypothetical protein BF2887 [Bacteroides fragilis YCH46]
>gi|53714247|ref|YP_100239.1| hypothetical protein BF2957 [Bacteroides fragilis YCH46]
>gi|53714279|ref|YP_100271.1| hypothetical protein BF2989 [Bacteroides fragilis YCH46]
>gi|53714335|ref|YP_100327.1| hypothetical protein BF3047 [Bacteroides fragilis YCH46]
>gi|53714393|ref|YP_100385.1| hypothetical protein BF3106 [Bacteroides fragilis YCH46]
>gi|53714500|ref|YP_100492.1| transcription termination factor rho [Bacteroides fragilis YCH46]
>gi|53714517|ref|YP_100509.1| hypothetical protein BF3230 [Bacteroides fragilis YCH46]
>gi|53714570|ref|YP_100562.1| hypothetical protein BF3284 [Bacteroides fragilis YCH46]
>gi|53714608|ref|YP_100600.1| hypothetical protein BF3322 [Bacteroides fragilis YCH46]
>gi|53714617|ref|YP_100609.1| hypothetical protein BF3331 [Bacteroides fragilis YCH46]
>gi|53714630|ref|YP_100622.1| hypothetical protein BF3344 [Bacteroides fragilis YCH46]
>gi|53714663|ref|YP_100655.1| putative chaperone DnaJ [Bacteroides fragilis YCH46]
>gi|53714734|ref|YP_100726.1| hypothetical protein BF3449 [Bacteroides fragilis YCH46]
>gi|53714770|ref|YP_100762.1| hypothetical protein BF3485 [Bacteroides fragilis YCH46]
>gi|53714773|ref|YP_100765.1| galactoside O-acetyltransferase [Bacteroides fragilis YCH46]
>gi|53714847|ref|YP_100839.1| hypothetical protein BF3562 [Bacteroides fragilis YCH46]
>gi|53714854|ref|YP_100846.1| hypothetical protein BF3569 [Bacteroides fragilis YCH46]
>gi|53714938|ref|YP_100930.1| putative O-acetyltransferase [Bacteroides fragilis YCH46]
>gi|53714963|ref|YP_100955.1| hypothetical protein BF3678 [Bacteroides fragilis YCH46]
>gi|53714974|ref|YP_100966.1| Holliday junction DNA helicase RuvA [Bacteroides fragilis YCH46]
>gi|53715003|ref|YP_100995.1| hypothetical protein BF3718 [Bacteroides fragilis YCH46]
>gi|53715049|ref|YP_101041.1| hypothetical protein BF3765 [Bacteroides fragilis YCH46]
>gi|53715120|ref|YP_101112.1| hypothetical protein BF3836 [Bacteroides fragilis YCH46]
>gi|53715133|ref|YP_101125.1| hypothetical protein BF3849 [Bacteroides fragilis YCH46]
>gi|53715134|ref|YP_101126.1| hypothetical protein BF3850 [Bacteroides fragilis YCH46]
>gi|53715142|ref|YP_101134.1| hypothetical protein BF3858 [Bacteroides fragilis YCH46]
>gi|53715159|ref|YP_101151.1| hypothetical protein BF3875 [Bacteroides fragilis YCH46]
>gi|53715248|ref|YP_101240.1| TonB-like protein [Bacteroides fragilis YCH46]
>gi|53715269|ref|YP_101261.1| hypothetical protein BF3985 [Bacteroides fragilis YCH46]
>gi|53715301|ref|YP_101293.1| ribosomal large subunit pseudouridine synthase B [Bacteroides fragilis YCH46]
>gi|53715304|ref|YP_101296.1| putative maltose O-acetyltransferase [Bacteroides fragilis YCH46]

>gi|53715400|ref|YP_101392.1| hypothetical protein BF4116 [Bacteroides fragilis YCH46]
>gi|53715473|ref|YP_101465.1| hypothetical protein BF4188 [Bacteroides fragilis YCH46]
>gi|53715478|ref|YP_101470.1| 50S ribosomal protein L7/L12 [Bacteroides fragilis YCH46]
>gi|53715523|ref|YP_101515.1| hypothetical protein BF4239 [Bacteroides fragilis YCH46]
>gi|53715625|ref|YP_101617.1| hypothetical protein BF4345 [Bacteroides fragilis YCH46]
>gi|56550920|ref|YP_161759.1| integral membrane protein [Zymomonas mobilis subsp. mobilis ZM4]
>gi|56550943|ref|YP_161781.1| hypothetical protein ZMO0047 [Zymomonas mobilis subsp. mobilis ZM4]
>gi|56550948|ref|YP_161787.1| cyanate permease [Zymomonas mobilis subsp. mobilis ZM4]
>gi|56550992|ref|YP_161831.1| phosphatidylglycerophosphate synthase [Zymomonas mobilis subsp. mobilis ZM4]
>gi|56550994|ref|YP_161833.1| hypothetical protein ZMO0098 [Zymomonas mobilis subsp. mobilis ZM4]
>gi|56551033|ref|YP_161872.1| hypothetical protein ZMO0137 [Zymomonas mobilis subsp. mobilis ZM4]
>gi|56551047|ref|YP_161886.1| hypothetical protein ZMO0151 [Zymomonas mobilis subsp. mobilis ZM4]
>gi|56551059|ref|YP_161898.1| hypothetical protein ZMO0163 [Zymomonas mobilis subsp. mobilis ZM4]
>gi|56551060|ref|YP_161899.1| TonB protein [Zymomonas mobilis subsp. mobilis ZM4]
>gi|56551080|ref|YP_161920.1| hypothetical protein ZMO0184 [Zymomonas mobilis subsp. mobilis ZM4]
>gi|56551089|ref|YP_161928.1| ribonuclease [Zymomonas mobilis subsp. mobilis ZM4]
>gi|56551125|ref|YP_161964.1| ABC-type Fe3+ transport system [Zymomonas mobilis subsp. mobilis ZM4]
>gi|56551158|ref|YP_161997.1| ABC-type transport system permease component [Zymomonas mobilis subsp. mobilis ZM4]
>gi|56551226|ref|YP_162065.1| hypothetical protein ZMO0330 [Zymomonas mobilis subsp. mobilis ZM4]
>gi|56551239|ref|YP_162078.1| anthranilate/para-aminobenzoate synthases component I [Zymomonas mobilis subsp. mobilis ZM4]
>gi|56551289|ref|YP_162128.1| hypothetical protein ZMO0393 [Zymomonas mobilis subsp. mobilis ZM4]
>gi|56551313|ref|YP_162152.1| cell division protein [Zymomonas mobilis subsp. mobilis ZM4]
>gi|56551331|ref|YP_162170.1| hypothetical protein ZMO0435 [Zymomonas mobilis subsp. mobilis ZM4]
>gi|56551333|ref|YP_162172.1| hypothetical protein ZMO0437 [Zymomonas mobilis subsp. mobilis ZM4]
>gi|56551343|ref|YP_162182.1| hypothetical protein ZMO0447 [Zymomonas mobilis subsp. mobilis ZM4]
>gi|56551400|ref|YP_162239.1| hypothetical protein ZMO0504 [Zymomonas mobilis subsp. mobilis ZM4]
>gi|56551405|ref|YP_162244.1| hypothetical protein ZMO0509 [Zymomonas mobilis subsp. mobilis ZM4]
>gi|56551429|ref|YP_162268.1| ribosomal protein S5 [Zymomonas mobilis subsp. mobilis ZM4]
>gi|56551431|ref|YP_162270.1| hypothetical protein ZMO0535 [Zymomonas mobilis subsp. mobilis ZM4]
>gi|56551432|ref|YP_162271.1| ribosomal protein L15 [Zymomonas mobilis subsp. mobilis ZM4]
>gi|56551450|ref|YP_162289.1| translation initiation factor 2 [Zymomonas mobilis subsp. mobilis ZM4]
>gi|56551472|ref|YP_162311.1| pseudouridylate synthase [Zymomonas mobilis subsp. mobilis ZM4]
>gi|56551488|ref|YP_162327.1| hypothetical protein ZMO0592 [Zymomonas mobilis subsp. mobilis ZM4]
>gi|56551501|ref|YP_162340.1| flagellar hook-associated protein 1 [Zymomonas mobilis subsp. mobilis ZM4]
>gi|56551502|ref|YP_162341.1| peptidoglycan hydrolase [Zymomonas mobilis subsp. mobilis ZM4]
>gi|56551507|ref|YP_162346.1| flagellar hook protein [Zymomonas mobilis subsp. mobilis ZM4]
>gi|56551508|ref|YP_162347.1| flagellar scaffolding protein [Zymomonas mobilis subsp. mobilis ZM4]
>gi|56551516|ref|YP_162355.1| hypothetical protein ZMO0620 [Zymomonas mobilis subsp. mobilis ZM4]
>gi|56551517|ref|YP_162356.1| platelet binding protein [Zymomonas mobilis subsp. mobilis ZM4]
>gi|56551532|ref|YP_162371.1| flagellar biosynthetic protein [Zymomonas mobilis subsp. mobilis ZM4]
>gi|56551540|ref|YP_162379.1| flagellar motor switch [Zymomonas mobilis subsp. mobilis ZM4]
>gi|56551564|ref|YP_162403.1| ATP synthase C chain [Zymomonas mobilis subsp. mobilis ZM4]
>gi|56551565|ref|YP_162404.1| ATP synthase B chain [Zymomonas mobilis subsp. mobilis ZM4]
>gi|56551566|ref|YP_162405.1| hypothetical protein ZMO0670 [Zymomonas mobilis subsp. mobilis ZM4]
>gi|56551567|ref|YP_162406.1| ATP synthase subunit b [Zymomonas mobilis subsp. mobilis ZM4]
>gi|56551624|ref|YP_162464.1| ribosomal protein L7/L12 [Zymomonas mobilis subsp. mobilis ZM4]
>gi|56551625|ref|YP_162463.1| ribosomal protein L7/L12 [Zymomonas mobilis subsp. mobilis ZM4]
>gi|56551632|ref|YP_162471.1| acetyl-CoA carboxylase [Zymomonas mobilis subsp. mobilis ZM4]
>gi|56551692|ref|YP_162531.1| hypothetical protein ZMO0796 [Zymomonas mobilis subsp. mobilis ZM4]
>gi|56551699|ref|YP_162538.1| hypothetical protein ZMO0803 [Zymomonas mobilis subsp. mobilis ZM4]
>gi|56551711|ref|YP_162550.1| hypothetical protein ZMO0815 [Zymomonas mobilis subsp. mobilis ZM4]
>gi|56551735|ref|YP_162574.1| hypothetical protein ZMO0839 [Zymomonas mobilis subsp. mobilis ZM4]
>gi|56551744|ref|YP_162583.1| hypothetical protein ZMO0848 [Zymomonas mobilis subsp. mobilis ZM4]
>gi|56551781|ref|YP_162620.1| hypothetical protein ZMO0885 [Zymomonas mobilis subsp. mobilis ZM4]
>gi|56551890|ref|YP_162729.1| hypothetical protein ZMO0994 [Zymomonas mobilis subsp. mobilis ZM4]
>gi|56551930|ref|YP_162769.1| hypothetical protein ZMO1034 [Zymomonas mobilis subsp. mobilis ZM4]
>gi|56551972|ref|YP_162811.1| ribosomal protein S16 [Zymomonas mobilis subsp. mobilis ZM4]
>gi|56552007|ref|YP_162846.1| ABC transporter [Zymomonas mobilis subsp. mobilis ZM4]
>gi|56552109|ref|YP_162948.1| hypothetical protein ZMO1213 [Zymomonas mobilis subsp. mobilis ZM4]
>gi|56552114|ref|YP_162953.1| Sec-independent protein secretion pathway component [Zymomonas mobilis subsp. mobilis ZM4]
>gi|56552139|ref|YP_162978.1| hypothetical protein ZMO1243 [Zymomonas mobilis subsp. mobilis ZM4]
>gi|56552165|ref|YP_163004.1| hypothetical protein ZMO1269 [Zymomonas mobilis subsp. mobilis ZM4]
>gi|56552185|ref|YP_163024.1| transglycosylase associated protein [Zymomonas mobilis subsp. mobilis ZM4]
>gi|56552192|ref|YP_163031.1| hypothetical protein ZMO1296 [Zymomonas mobilis subsp. mobilis ZM4]
>gi|56552237|ref|YP_163076.1| metalloprotease [Zymomonas mobilis subsp. mobilis ZM4]
>gi|56552250|ref|YP_163089.1| peptidoglycan-associated protein [Zymomonas mobilis subsp. mobilis ZM4]
>gi|56552263|ref|YP_163102.1| hypothetical protein ZMO1367 [Zymomonas mobilis subsp. mobilis ZM4]
>gi|56552282|ref|YP_163121.1| hypothetical protein ZMO1386 [Zymomonas mobilis subsp. mobilis ZM4]
>gi|56552299|ref|YP_163138.1| membrane protein [Zymomonas mobilis subsp. mobilis ZM4]
>gi|56552343|ref|YP_163182.1| hypothetical protein ZMO1447 [Zymomonas mobilis subsp. mobilis ZM4]

>gi|56552355|ref|YP_163194.1| putative transport protein [Zymomonas mobilis subsp. mobilis ZM4]
>gi|56552386|ref|YP_163225.1| putative outer membrane protein [Zymomonas mobilis subsp. mobilis ZM4]
>gi|56552388|ref|YP_163227.1| hypothetical protein ZMO1492 [Zymomonas mobilis subsp. mobilis ZM4]
>gi|56552400|ref|YP_163239.1| hypothetical protein ZMO1504 [Zymomonas mobilis subsp. mobilis ZM4]
>gi|56552407|ref|YP_163246.1| hypothetical protein ZMO1511 [Zymomonas mobilis subsp. mobilis ZM4]
>gi|56552421|ref|YP_163260.1| outer membrane protein [Zymomonas mobilis subsp. mobilis ZM4]
>gi|56552438|ref|YP_163277.1| single-strand DNA binding protein [Zymomonas mobilis subsp. mobilis ZM4]
>gi|56552460|ref|YP_163299.1| putative nicotinamide mononucleotide transporter [Zymomonas mobilis subsp. mobilis ZM4]
>gi|56552483|ref|YP_163322.1| hypothetical protein ZMO1587 [Zymomonas mobilis subsp. mobilis ZM4]
>gi|56552491|ref|YP_163330.1| hypothetical protein ZMO1595 [Zymomonas mobilis subsp. mobilis ZM4]
>gi|56552506|ref|YP_163345.1| hypothetical protein ZMO1610 [Zymomonas mobilis subsp. mobilis ZM4]
>gi|56552515|ref|YP_163354.1| hypothetical protein ZMO1619 [Zymomonas mobilis subsp. mobilis ZM4]
>gi|56552526|ref|YP_163365.1| hypothetical protein ZMO1630 [Zymomonas mobilis subsp. mobilis ZM4]
>gi|56552537|ref|YP_163376.1| hypothetical protein ZMO1641 [Zymomonas mobilis subsp. mobilis ZM4]
>gi|56552567|ref|YP_163406.1| hypothetical protein ZMO1671 [Zymomonas mobilis subsp. mobilis ZM4]
>gi|56552586|ref|YP_163425.1| DnaJ-class molecular chaperone [Zymomonas mobilis subsp. mobilis ZM4]
>gi|56552609|ref|YP_163448.1| ribosomal protein S21 [Zymomonas mobilis subsp. mobilis ZM4]
>gi|56552613|ref|YP_163452.1| hypothetical protein ZMO1717 [Zymomonas mobilis subsp. mobilis ZM4]
>gi|56552617|ref|YP_163456.1| lactoylglutathione lyase [Zymomonas mobilis subsp. mobilis ZM4]
>gi|56552645|ref|YP_163484.1| possible lipoprotein precursor [Zymomonas mobilis subsp. mobilis ZM4]
>gi|56552659|ref|YP_163498.1| hypothetical protein ZMO1763 [Zymomonas mobilis subsp. mobilis ZM4]
>gi|56552690|ref|YP_163529.1| hypothetical protein ZMO1794 [Zymomonas mobilis subsp. mobilis ZM4]
>gi|56552700|ref|YP_163539.1| amino acid transporter [Zymomonas mobilis subsp. mobilis ZM4]
>gi|56552748|ref|YP_163587.1| hypothetical protein ZMO1852 [Zymomonas mobilis subsp. mobilis ZM4]
>gi|56552816|ref|YP_163655.1| hypothetical protein ZMO1920 [Zymomonas mobilis subsp. mobilis ZM4]
>gi|56552832|ref|YP_163671.1| hypothetical protein ZMO1936 [Zymomonas mobilis subsp. mobilis ZM4]
>gi|56552834|ref|YP_163673.1| hypothetical protein ZMO1938 [Zymomonas mobilis subsp. mobilis ZM4]
>gi|56552836|ref|YP_163675.1| hypothetical protein ZMO1940 [Zymomonas mobilis subsp. mobilis ZM4]
>gi|56552837|ref|YP_163676.1| conjugal transfer protein [Zymomonas mobilis subsp. mobilis ZM4]
>gi|56552838|ref|YP_163677.1| VirD2 components relaxase [Zymomonas mobilis subsp. mobilis ZM4]
>gi|56552850|ref|YP_163689.1| conjugal transfer protein [Zymomonas mobilis subsp. mobilis ZM4]
>gi|56552861|ref|YP_163700.1| predicted membrane metal-binding protein [Zymomonas mobilis subsp. mobilis ZM4]
>gi|57865353|ref|YP_189555.1| glutaredoxin, putative [Staphylococcus epidermidis RP62A]
>gi|57865398|ref|YP_189582.1| holin-like protein LrgA [Staphylococcus epidermidis RP62A]
>gi|57865404|ref|YP_189517.1| lipoprotein, putative [Staphylococcus epidermidis RP62A]
>gi|57865503|ref|YP_189635.1| hypothetical protein SERP2079 [Staphylococcus epidermidis RP62A]
>gi|57865510|ref|YP_189650.1| L-serine dehydratase, iron-sulfur-dependent, alpha subunit [Staphylococcus epidermidis RP62A]
>gi|57865532|ref|YP_189664.1| hypothetical protein SERP2108 [Staphylococcus epidermidis RP62A]
>gi|57865590|ref|YP_189692.1| secretory antigen precursor SsaA [Staphylococcus epidermidis RP62A]
>gi|57865592|ref|YP_189694.1| immunodominant antigen A, putative [Staphylococcus epidermidis RP62A]
>gi|57865599|ref|YP_189768.1| hypothetical protein SERP2212 [Staphylococcus epidermidis RP62A]
>gi|57865604|ref|YP_189697.1| regulatory protein, putative [Staphylococcus epidermidis RP62A]
>gi|57865616|ref|YP_189777.1| transcriptional regulator CadC [Staphylococcus epidermidis RP62A]
>gi|57865618|ref|YP_189705.1| hypothetical protein SERP2149 [Staphylococcus epidermidis RP62A]
>gi|57865652|ref|YP_189805.1| hypothetical protein SERP2254 [Staphylococcus epidermidis RP62A]
>gi|57865710|ref|YP_189831.1| serine threonine rich antigen [Staphylococcus epidermidis RP62A]
>gi|57865751|ref|YP_189925.1| hypothetical protein SERP2377 [Staphylococcus epidermidis RP62A]
>gi|57865761|ref|YP_189928.1| drug transporter, putative [Staphylococcus epidermidis RP62A]
>gi|57865764|ref|YP_189931.1| ABC transporter, substrate-binding protein [Staphylococcus epidermidis RP62A]
>gi|57865779|ref|YP_189939.1| cell wall associated biofilm protein [Staphylococcus epidermidis RP62A]
>gi|57865793|ref|YP_189945.1| accumulation associated protein [Staphylococcus epidermidis RP62A]
>gi|57865800|ref|YP_189877.1| anion transporter family protein [Staphylococcus epidermidis RP62A]
>gi|57865843|ref|YP_189977.1| hypothetical protein SERP2432 [Staphylococcus epidermidis RP62A]
>gi|57865940|ref|YP_187601.1| ribosomal protein L34 [Staphylococcus epidermidis RP62A]
>gi|57865956|ref|YP_187609.1| hypothetical protein SERP0009 [Staphylococcus epidermidis RP62A]
>gi|57865976|ref|YP_187623.1| hypothetical protein SERP0023 [Staphylococcus epidermidis RP62A]
>gi|57865984|ref|YP_187645.1| single-stranded DNA-binding protein [Staphylococcus epidermidis RP62A]
>gi|57865995|ref|YP_187648.1| hypothetical protein SERP0049 [Staphylococcus epidermidis RP62A]
>gi|57865999|ref|YP_187652.1| hypothetical protein SERP0054 [Staphylococcus epidermidis RP62A]
>gi|57866017|ref|YP_187662.1| hypothetical protein SERP0064 [Staphylococcus epidermidis RP62A]
>gi|57866033|ref|YP_187670.1| hypothetical protein SERP0073 [Staphylococcus epidermidis RP62A]
>gi|57866038|ref|YP_187675.1| perfringolysin O regulator protein [Staphylococcus epidermidis RP62A]
>gi|57866049|ref|YP_187678.1| hypothetical protein SERP0081 [Staphylococcus epidermidis RP62A]
>gi|57866080|ref|YP_187695.1| ABC transporter, permease protein [Staphylococcus epidermidis RP62A]
>gi|57866099|ref|YP_187707.1| membrane protein, putative [Staphylococcus epidermidis RP62A]
>gi|57866111|ref|YP_187711.1| hypothetical protein SERP0115 [Staphylococcus epidermidis RP62A]
>gi|57866132|ref|YP_187777.1| ribosomal protein L7/L12 [Staphylococcus epidermidis RP62A]
>gi|57866152|ref|YP_187789.1| hypothetical protein SERP0193 [Staphylococcus epidermidis RP62A]
>gi|57866190|ref|YP_187837.1| hypothetical protein SERP0241 [Staphylococcus epidermidis RP62A]
>gi|57866247|ref|YP_187863.1| iron compound ABC transporter, permease protein [Staphylococcus epidermidis RP62A]

>gi|57866256|ref|YP_187928.1| lipoprotein, putative [Staphylococcus epidermidis RP62A]
>gi|57866269|ref|YP_187933.1| hypothetical protein SERP0337 [Staphylococcus epidermidis RP62A]
>gi|57866285|ref|YP_187941.1| hypothetical protein SERP0345 [Staphylococcus epidermidis RP62A]
>gi|57866291|ref|YP_187882.1| Na+/H+ antiporter, MnhF component, putative [Staphylococcus epidermidis RP62A]
>gi|57866305|ref|YP_187971.1| hypothetical protein SERP0376 [Staphylococcus epidermidis RP62A]
>gi|57866310|ref|YP_187976.1| gufA protein, putative [Staphylococcus epidermidis RP62A]
>gi|57866342|ref|YP_187992.1| hypothetical protein SERP0399 [Staphylococcus epidermidis RP62A]
>gi|57866351|ref|YP_188054.1| hypothetical protein SERP0462 [Staphylococcus epidermidis RP62A]
>gi|57866403|ref|YP_188082.1| ABC transporter, permease protein [Staphylococcus epidermidis RP62A]
>gi|57866416|ref|YP_188087.1| hypothetical protein SERP0495 [Staphylococcus epidermidis RP62A]
>gi|57866422|ref|YP_188030.1| hypothetical protein SERP0437 [Staphylococcus epidermidis RP62A]
>gi|57866424|ref|YP_188032.1| hypothetical protein SERP0439 [Staphylococcus epidermidis RP62A]
>gi|57866435|ref|YP_188098.1| hypothetical protein SERP0506 [Staphylococcus epidermidis RP62A]
>gi|57866456|ref|YP_188047.1| lipoprotein, putative [Staphylococcus epidermidis RP62A]
>gi|57866517|ref|YP_188160.1| hypothetical protein SERP0569 [Staphylococcus epidermidis RP62A]
>gi|57866605|ref|YP_188263.1| hypothetical protein SERP0679 [Staphylococcus epidermidis RP62A]
>gi|57866631|ref|YP_188274.1| hypothetical protein SERP0690 [Staphylococcus epidermidis RP62A]
>gi|57866639|ref|YP_188302.1| cell wall surface anchor family protein [Staphylococcus epidermidis RP62A]
>gi|57866735|ref|YP_188410.1| phosphatidate cytidylyltransferase [Staphylococcus epidermidis RP62A]
>gi|57866799|ref|YP_188470.1| hypothetical protein SERP0890 [Staphylococcus epidermidis RP62A]
>gi|57866843|ref|YP_188493.1| hypothetical protein SERP0914 [Staphylococcus epidermidis RP62A]
>gi|57866853|ref|YP_188545.1| 2,3,4,5-tetrahydropyridine-2,6-dicarboxylate N-succinyltransferase [Staphylococcus epidermidis RP62A]
>gi|57866884|ref|YP_188561.1| hypothetical protein SERP0983 [Staphylococcus epidermidis RP62A]
>gi|57866924|ref|YP_188537.1| phosphate ABC transporter, permease protein [Staphylococcus epidermidis RP62A]
>gi|57866944|ref|YP_188625.1| elastin binding protein, putative [Staphylococcus epidermidis RP62A]
>gi|57867018|ref|YP_188712.1| diacylglycerol kinase [Staphylococcus epidermidis RP62A]
>gi|57867021|ref|YP_188715.1| hypothetical protein SERP1139 [Staphylococcus epidermidis RP62A]
>gi|57867023|ref|YP_188717.1| hypothetical protein SERP1141 [Staphylococcus epidermidis RP62A]
>gi|57867025|ref|YP_188719.1| ribosomal protein S21 [Staphylococcus epidermidis RP62A]
>gi|57867038|ref|YP_188725.1| heat shock protein GrpE [Staphylococcus epidermidis RP62A]
>gi|57867042|ref|YP_188673.1| hypothetical protein SERP1096 [Staphylococcus epidermidis RP62A]
>gi|57867081|ref|YP_188746.1| acetyl-CoA carboxylase, biotin carboxyl carrier protein, putative [Staphylococcus epidermidis RP62A]
>gi|57867098|ref|YP_188755.1| hypothetical protein SERP1179 [Staphylococcus epidermidis RP62A]
>gi|57867107|ref|YP_188791.1| hypothetical protein SERP1216 [Staphylococcus epidermidis RP62A]
>gi|57867115|ref|YP_188764.1| hypothetical protein SERP1188 [Staphylococcus epidermidis RP62A]
>gi|57867123|ref|YP_188800.1| type III leader peptidase family protein [Staphylococcus epidermidis RP62A]
>gi|57867234|ref|YP_188909.1| hypothetical protein SERP1337 [Staphylococcus epidermidis RP62A]
>gi|57867235|ref|YP_188910.1| CrcB family protein [Staphylococcus epidermidis RP62A]
>gi|57867265|ref|YP_188870.1| hypothetical protein SERP1298 [Staphylococcus epidermidis RP62A]
>gi|57867315|ref|YP_188945.1| protein export protein PrsA, putative [Staphylococcus epidermidis RP62A]
>gi|57867334|ref|YP_189038.1| hypothetical protein SERP1471 [Staphylococcus epidermidis RP62A]
>gi|57867335|ref|YP_189039.1| hypothetical protein SERP1472 [Staphylococcus epidermidis RP62A]
>gi|57867336|ref|YP_189040.1| hypothetical protein SERP1474 [Staphylococcus epidermidis RP62A]
>gi|57867346|ref|YP_189042.1| hypothetical protein SERP1476 [Staphylococcus epidermidis RP62A]
>gi|57867362|ref|YP_189049.1| cell wall surface anchor family protein [Staphylococcus epidermidis RP62A]
>gi|57867366|ref|YP_189053.1| sdrH protein [Staphylococcus epidermidis RP62A]
>gi|57867417|ref|YP_189032.1| Tn554-related, transposase C [Staphylococcus epidermidis RP62A]
>gi|57867425|ref|YP_189083.1| hypothetical protein SERP1518 [Staphylococcus epidermidis RP62A]
>gi|57867443|ref|YP_189094.1| hypothetical protein SERP1529 [Staphylococcus epidermidis RP62A]
>gi|57867453|ref|YP_189095.1| hypothetical protein SERP1530 [Staphylococcus epidermidis RP62A]
>gi|57867472|ref|YP_189105.1| hypothetical protein SERP1540 [Staphylococcus epidermidis RP62A]
>gi|57867475|ref|YP_189108.1| hypothetical protein SERP1543 [Staphylococcus epidermidis RP62A]
>gi|57867495|ref|YP_189158.1| hypothetical protein SERP1593 [Staphylococcus epidermidis RP62A]
>gi|57867524|ref|YP_189169.1| hypothetical protein SERP1604 [Staphylococcus epidermidis RP62A]
>gi|57867536|ref|YP_189219.1| cell wall surface anchor family protein [Staphylococcus epidermidis RP62A]
>gi|57867604|ref|YP_189252.1| hypothetical protein SERP1687 [Staphylococcus epidermidis RP62A]
>gi|57867611|ref|YP_189299.1| DNA-directed RNA polymerase, delta subunit [Staphylococcus epidermidis RP62A]
>gi|57867634|ref|YP_189267.1| sceD protein [Staphylococcus epidermidis RP62A]
>gi|57867676|ref|YP_189369.1| ribosomal protein S11 [Staphylococcus epidermidis RP62A]
>gi|57867705|ref|YP_189347.1| hypothetical protein SERP1783 [Staphylococcus epidermidis RP62A]
>gi|57867708|ref|YP_189385.1| ribosomal protein S17 [Staphylococcus epidermidis RP62A]
>gi|57867748|ref|YP_189364.1| cobalt transport family protein [Staphylococcus epidermidis RP62A]
>gi|57867779|ref|YP_189465.1| hypothetical protein SERP1904 [Staphylococcus epidermidis RP62A]
>gi|57867780|ref|YP_189466.1| hypothetical protein SERP1905 [Staphylococcus epidermidis RP62A]
>gi|57867814|ref|YP_189441.1| secretory antigen precursor SsaA [Staphylococcus epidermidis RP62A]
>gi|57867826|ref|YP_189444.1| hypothetical protein SERP1883 [Staphylococcus epidermidis RP62A]
>gi|49484925|ref|YP_042146.1| putative membrane protein [Staphylococcus aureus subsp. aureus MSSA476]
>gi|49484959|ref|YP_042180.1| putative membrane protein [Staphylococcus aureus subsp. aureus MSSA476]
>gi|49484988|ref|YP_042209.1| immunoglobulin G binding protein A precursor [Staphylococcus aureus subsp. aureus MSSA476]
>gi|49484991|ref|YP_042212.1| putative siderophore transport system permease [Staphylococcus aureus subsp. aureus MSSA476]

>gi|49485003|ref|YP_042224.1| putative membrane protein [Staphylococcus aureus subsp. aureus MSSA476]
>gi|49485012|ref|YP_042233.1| putative surface anchored protein [Staphylococcus aureus subsp. aureus MSSA476]
>gi|49485035|ref|YP_042256.1| capsular polysaccharide synthesis enzyme [Staphylococcus aureus subsp. aureus MSSA476]
>gi|49485158|ref|YP_042379.1| putative membrane protein [Staphylococcus aureus subsp. aureus MSSA476]
>gi|49485162|ref|YP_042383.1| hypothetical protein SAS0258 [Staphylococcus aureus subsp. aureus MSSA476]
>gi|49485175|ref|YP_042396.1| putative membrane protein [Staphylococcus aureus subsp. aureus MSSA476]
>gi|49485185|ref|YP_042406.1| putative membrane protein [Staphylococcus aureus subsp. aureus MSSA476]
>gi|49485230|ref|YP_042451.1| putative membrane protein [Staphylococcus aureus subsp. aureus MSSA476]
>gi|49485253|ref|YP_042474.1| putative membrane protein [Staphylococcus aureus subsp. aureus MSSA476]
>gi|49485256|ref|YP_042477.1| putative membrane protein [Staphylococcus aureus subsp. aureus MSSA476]
>gi|49485277|ref|YP_042498.1| putative membrane protein [Staphylococcus aureus subsp. aureus MSSA476]
>gi|49485282|ref|YP_042503.1| hypothetical protein SAS0379 [Staphylococcus aureus subsp. aureus MSSA476]
>gi|49485290|ref|YP_042511.1| exotoxin [Staphylococcus aureus subsp. aureus MSSA476]
>gi|49485291|ref|YP_042512.1| exotoxin [Staphylococcus aureus subsp. aureus MSSA476]
>gi|49485393|ref|YP_042614.1| serine acetyltransferase [Staphylococcus aureus subsp. aureus MSSA476]
>gi|49485405|ref|YP_042626.1| 50S ribosomal protein L7/L12 [Staphylococcus aureus subsp. aureus MSSA476]
>gi|49485426|ref|YP_042647.1| putative surface anchored protein [Staphylococcus aureus subsp. aureus MSSA476]
>gi|49485427|ref|YP_042648.1| putative surface anchored protein [Staphylococcus aureus subsp. aureus MSSA476]
>gi|49485428|ref|YP_042649.1| bone sialoprotein-binding protein [Staphylococcus aureus subsp. aureus MSSA476]
>gi|49485459|ref|YP_042680.1| hypothetical protein SAS0552 [Staphylococcus aureus subsp. aureus MSSA476]
>gi|49485484|ref|YP_042705.1| FecCD transport family protein [Staphylococcus aureus subsp. aureus MSSA476]
>gi|49485491|ref|YP_042712.1| putative membrane protein [Staphylococcus aureus subsp. aureus MSSA476]
>gi|49485549|ref|YP_042770.1| putative lipoprotein [Staphylococcus aureus subsp. aureus MSSA476]
>gi|49485554|ref|YP_042775.1| hypothetical protein SAS0647 [Staphylococcus aureus subsp. aureus MSSA476]
>gi|49485581|ref|YP_042802.1| putative membrane protein [Staphylococcus aureus subsp. aureus MSSA476]
>gi|49485588|ref|YP_042809.1| putative membrane protein [Staphylococcus aureus subsp. aureus MSSA476]
>gi|49485606|ref|YP_042827.1| FecCD transport family protein [Staphylococcus aureus subsp. aureus MSSA476]
>gi|49485619|ref|YP_042840.1| putative glycosyl transferase [Staphylococcus aureus subsp. aureus MSSA476]
>gi|49485627|ref|YP_042848.1| hypothetical protein SAS0720 [Staphylococcus aureus subsp. aureus MSSA476]
>gi|49485642|ref|YP_042863.1| hypothetical protein SAS0736 [Staphylococcus aureus subsp. aureus MSSA476]
>gi|49485654|ref|YP_042875.1| putative lipoprotein [Staphylococcus aureus subsp. aureus MSSA476]
>gi|49485657|ref|YP_042878.1| clumping factor [Staphylococcus aureus subsp. aureus MSSA476]
>gi|49485671|ref|YP_042892.1| putative LysE type translocator protein [Staphylococcus aureus subsp. aureus MSSA476]
>gi|49485685|ref|YP_042906.1| ABC transporter permease protein [Staphylococcus aureus subsp. aureus MSSA476]
>gi|49485723|ref|YP_042944.1| Na+/H+ antiporter subunit [Staphylococcus aureus subsp. aureus MSSA476]
>gi|49485941|ref|YP_043162.1| putative membrane protein [Staphylococcus aureus subsp. aureus MSSA476]
>gi|49485943|ref|YP_043164.1| putative manganese transport protein [Staphylococcus aureus subsp. aureus MSSA476]
>gi|49485966|ref|YP_043187.1| iron-regulated heme-iron binding protein [Staphylococcus aureus subsp. aureus MSSA476]
>gi|49485967|ref|YP_043188.1| iron-regulated heme-iron binding protein [Staphylococcus aureus subsp. aureus MSSA476]
>gi|49485994|ref|YP_043215.1| putative membrane protein [Staphylococcus aureus subsp. aureus MSSA476]
>gi|49485999|ref|YP_043220.1| putative membrane protein [Staphylococcus aureus subsp. aureus MSSA476]
>gi|49486154|ref|YP_043375.1| hypothetical protein SAS1249 [Staphylococcus aureus subsp. aureus MSSA476]
>gi|49486155|ref|YP_043376.1| hypothetical protein SAS1250 [Staphylococcus aureus subsp. aureus MSSA476]
>gi|49486167|ref|YP_043388.1| putative membrane protein [Staphylococcus aureus subsp. aureus MSSA476]
>gi|49486187|ref|YP_043408.1| hypothetical protein SAS1283 [Staphylococcus aureus subsp. aureus MSSA476]
>gi|49486238|ref|YP_043459.1| putative tetrahydrodipicolinate acetyltransferase [Staphylococcus aureus subsp. aureus MSSA476]
>gi|49486319|ref|YP_043540.1| cell surface elastin binding protein [Staphylococcus aureus subsp. aureus MSSA476]
>gi|49486365|ref|YP_043586.1| putative elongation factor P [Staphylococcus aureus subsp. aureus MSSA476]
>gi|49486368|ref|YP_043589.1| hypothetical protein SAS1470 [Staphylococcus aureus subsp. aureus MSSA476]
>gi|49486404|ref|YP_043625.1| putative diacylglycerol kinase [Staphylococcus aureus subsp. aureus MSSA476]
>gi|49486410|ref|YP_043631.1| 30S ribosomal protein S21 [Staphylococcus aureus subsp. aureus MSSA476]
>gi|49486420|ref|YP_043641.1| putative 30S ribosomal protein S20 [Staphylococcus aureus subsp. aureus MSSA476]
>gi|49486437|ref|YP_043658.1| putative membrane protein [Staphylococcus aureus subsp. aureus MSSA476]
>gi|49486448|ref|YP_043669.1| hypothetical protein SAS1551 [Staphylococcus aureus subsp. aureus MSSA476]
>gi|49486457|ref|YP_043678.1| hypothetical protein SAS1560 [Staphylococcus aureus subsp. aureus MSSA476]
>gi|49486458|ref|YP_043679.1| hypothetical protein SAS1561 [Staphylococcus aureus subsp. aureus MSSA476]
>gi|49486485|ref|YP_043706.1| putative membrane protein [Staphylococcus aureus subsp. aureus MSSA476]
>gi|49486555|ref|YP_043776.1| haptoglobin-binding surface anchored protein [Staphylococcus aureus subsp. aureus MSSA476]
>gi|49486563|ref|YP_043784.1| putative membrane protein [Staphylococcus aureus subsp. aureus MSSA476]
>gi|49486603|ref|YP_043824.1| putative membrane protein [Staphylococcus aureus subsp. aureus MSSA476]
>gi|49486618|ref|YP_043839.1| putative lipoprotein [Staphylococcus aureus subsp. aureus MSSA476]
>gi|49486619|ref|YP_043840.1| putative membrane protein [Staphylococcus aureus subsp. aureus MSSA476]
>gi|49486625|ref|YP_043846.1| putative membrane protein [Staphylococcus aureus subsp. aureus MSSA476]
>gi|49486658|ref|YP_043879.1| putative peptidyl-prolyl cis-isomerase [Staphylococcus aureus subsp. aureus MSSA476]
>gi|49486662|ref|YP_043883.1| hypothetical protein SAS1766 [Staphylococcus aureus subsp. aureus MSSA476]
>gi|49486746|ref|YP_043967.1| putative membrane protein [Staphylococcus aureus subsp. aureus MSSA476]
>gi|49486749|ref|YP_043970.1| putative membrane protein [Staphylococcus aureus subsp. aureus MSSA476]
>gi|49486759|ref|YP_043980.1| putative membrane protein [Staphylococcus aureus subsp. aureus MSSA476]
>gi|49486760|ref|YP_043981.1| hypothetical protein SAS1866 [Staphylococcus aureus subsp. aureus MSSA476]
>gi|49486774|ref|YP_043995.1| hypothetical protein SAS1881 [Staphylococcus aureus subsp. aureus MSSA476]

>gi|49486791|ref|YP_044012.1| hypothetical protein SAS1898 [Staphylococcus aureus subsp. aureus MSSA476]
>gi|49486821|ref|YP_044042.1| putative membrane protein [Staphylococcus aureus subsp. aureus MSSA476]
>gi|49486827|ref|YP_044048.1| membrane anchored protein [Staphylococcus aureus subsp. aureus MSSA476]
>gi|49486833|ref|YP_044054.1| autoinducer sensor protein [Staphylococcus aureus subsp. aureus MSSA476]
>gi|49486921|ref|YP_044142.1| DNA-directed RNA polymerase delta subunit [Staphylococcus aureus subsp. aureus MSSA476]
>gi|49486964|ref|YP_044185.1| FecCD transport family protein [Staphylococcus aureus subsp. aureus MSSA476]
>gi|49487044|ref|YP_044265.1| putative membrane protein [Staphylococcus aureus subsp. aureus MSSA476]
>gi|49487063|ref|YP_044284.1| BioY family protein [Staphylococcus aureus subsp. aureus MSSA476]
>gi|49487080|ref|YP_044301.1| hypothetical protein SAS2189 [Staphylococcus aureus subsp. aureus MSSA476]
>gi|49487084|ref|YP_044305.1| putative membrane protein [Staphylococcus aureus subsp. aureus MSSA476]
>gi|49487085|ref|YP_044306.1| hypothetical protein SAS2194 [Staphylococcus aureus subsp. aureus MSSA476]
>gi|49487100|ref|YP_044321.1| putative membrane protein [Staphylococcus aureus subsp. aureus MSSA476]
>gi|49487101|ref|YP_044322.1| putative membrane protein [Staphylococcus aureus subsp. aureus MSSA476]
>gi|49487110|ref|YP_044331.1| putative membrane protein [Staphylococcus aureus subsp. aureus MSSA476]
>gi|49487136|ref|YP_044357.1| hypothetical protein SAS2244 [Staphylococcus aureus subsp. aureus MSSA476]
>gi|49487151|ref|YP_044372.1| putative lipoprotein [Staphylococcus aureus subsp. aureus MSSA476]
>gi|49487154|ref|YP_044375.1| putative membrane protein [Staphylococcus aureus subsp. aureus MSSA476]
>gi|49487165|ref|YP_044386.1| hypothetical protein SAS2273 [Staphylococcus aureus subsp. aureus MSSA476]
>gi|49487200|ref|YP_044421.1| IgG-binding protein [Staphylococcus aureus subsp. aureus MSSA476]
>gi|49487204|ref|YP_044425.1| putative membrane protein [Staphylococcus aureus subsp. aureus MSSA476]
>gi|49487216|ref|YP_044437.1| putative membrane protein [Staphylococcus aureus subsp. aureus MSSA476]
>gi|49487273|ref|YP_044494.1| putative membrane protein [Staphylococcus aureus subsp. aureus MSSA476]
>gi|49487274|ref|YP_044495.1| putative membrane protein [Staphylococcus aureus subsp. aureus MSSA476]
>gi|49487280|ref|YP_044501.1| fibronectin-binding protein precursor [Staphylococcus aureus subsp. aureus MSSA476]
>gi|49487289|ref|YP_044510.1| hypothetical protein SAS2396a [Staphylococcus aureus subsp. aureus MSSA476]
>gi|49487293|ref|YP_044514.1| putative membrane protein [Staphylococcus aureus subsp. aureus MSSA476]
>gi|49487305|ref|YP_044526.1| putative membrane protein [Staphylococcus aureus subsp. aureus MSSA476]
>gi|49487306|ref|YP_044527.1| sortase A [Staphylococcus aureus subsp. aureus MSSA476]
>gi|49487309|ref|YP_044530.1| putative L-serine dehydratase, alpha chain [Staphylococcus aureus subsp. aureus MSSA476]
>gi|49487345|ref|YP_044566.1| secretory antigen precursor [Staphylococcus aureus subsp. aureus MSSA476]
>gi|49487348|ref|YP_044569.1| immunodominant antigen A [Staphylococcus aureus subsp. aureus MSSA476]
>gi|49487409|ref|YP_044630.1| fibrinogen and keratin-10 binding surface anchored protein [Staphylococcus aureus subsp. aureus MSSA476]
>gi|49487418|ref|YP_044639.1| hypothetical protein SAS2525 [Staphylococcus aureus subsp. aureus MSSA476]
>gi|49487423|ref|YP_044644.1| hypothetical protein SAS2530 [Staphylococcus aureus subsp. aureus MSSA476]
>gi|49487433|ref|YP_044654.1| putative cell wall-anchored protein [Staphylococcus aureus subsp. aureus MSSA476]
>gi|49487466|ref|YP_044687.1| putative membrane protein [Staphylococcus aureus subsp. aureus MSSA476]
>gi|49487478|ref|YP_044699.1| putative membrane protein [Staphylococcus aureus subsp. aureus MSSA476]
>gi|49487485|ref|YP_044706.1| putative membrane protein [Staphylococcus aureus subsp. aureus MSSA476]
>gi|49487491|ref|YP_044712.1| 50S ribosomal protein L34 [Staphylococcus aureus subsp. aureus MSSA476]
>gi|49398100|ref|YP_031699.1| hypothetical protein pSAS03 [Staphylococcus aureus subsp. aureus MSSA476]
>gi|58579628|ref|YP_198644.1| hypothetical protein XOO0005 [Xanthomonas oryzae pv. oryzae KACC10331]
>gi|58579630|ref|YP_198646.1| hypothetical protein XOO0007 [Xanthomonas oryzae pv. oryzae KACC10331]
>gi|58579631|ref|YP_198647.1| TonB protein [Xanthomonas oryzae pv. oryzae KACC10331]
>gi|58579634|ref|YP_198650.1| biopolymer transport ExbD2 protein [Xanthomonas oryzae pv. oryzae KACC10331]
>gi|58579636|ref|YP_198652.1| hypothetical protein XOO0013 [Xanthomonas oryzae pv. oryzae KACC10331]
>gi|58579641|ref|YP_198657.1| hypothetical protein XOO0018 [Xanthomonas oryzae pv. oryzae KACC10331]
>gi|58579681|ref|YP_198697.1| histone H1 homolog [Xanthomonas oryzae pv. oryzae KACC10331]
>gi|58579683|ref|YP_198699.1| hypothetical protein XOO0060 [Xanthomonas oryzae pv. oryzae KACC10331]
>gi|58579688|ref|YP_198704.1| HpaF [Xanthomonas oryzae pv. oryzae KACC10331]
>gi|58579705|ref|YP_198721.1| hrpD1 [Xanthomonas oryzae pv. oryzae KACC10331]
>gi|58579709|ref|YP_198725.1| HrpB1 [Xanthomonas oryzae pv. oryzae KACC10331]
>gi|58579713|ref|YP_198729.1| HrpB5 [Xanthomonas oryzae pv. oryzae KACC10331]
>gi|58579715|ref|YP_198731.1| HrpB7 [Xanthomonas oryzae pv. oryzae KACC10331]
>gi|58579718|ref|YP_198734.1| Hpa1 [Xanthomonas oryzae pv. oryzae KACC10331]
>gi|58579734|ref|YP_198750.1| hypothetical protein XOO0111 [Xanthomonas oryzae pv. oryzae KACC10331]
>gi|58579762|ref|YP_198778.1| NagN [Xanthomonas oryzae pv. oryzae KACC10331]
>gi|58579765|ref|YP_198781.1| IS1478 transposase [Xanthomonas oryzae pv. oryzae KACC10331]
>gi|58579767|ref|YP_198783.1| IS1478 transposase [Xanthomonas oryzae pv. oryzae KACC10331]
>gi|58579781|ref|YP_198797.1| hypothetical protein XOO0158 [Xanthomonas oryzae pv. oryzae KACC10331]
>gi|58579790|ref|YP_198806.1| hypothetical protein XOO0167 [Xanthomonas oryzae pv. oryzae KACC10331]
>gi|58579793|ref|YP_198809.1| TonB-dependent receptor [Xanthomonas oryzae pv. oryzae KACC10331]
>gi|58579799|ref|YP_198815.1| hypothetical protein XOO0176 [Xanthomonas oryzae pv. oryzae KACC10331]
>gi|58579829|ref|YP_198845.1| NagN [Xanthomonas oryzae pv. oryzae KACC10331]
>gi|58579840|ref|YP_198856.1| hypothetical protein XOO0217 [Xanthomonas oryzae pv. oryzae KACC10331]
>gi|58579851|ref|YP_198867.1| threonine dehydratase [Xanthomonas oryzae pv. oryzae KACC10331]
>gi|58579858|ref|YP_198874.1| biotin biosynthesis protein [Xanthomonas oryzae pv. oryzae KACC10331]
>gi|58579869|ref|YP_198885.1| acid phosphatase [Xanthomonas oryzae pv. oryzae KACC10331]
>gi|58579874|ref|YP_198890.1| hypothetical protein XOO0251 [Xanthomonas oryzae pv. oryzae KACC10331]
>gi|58579880|ref|YP_198896.1| hypothetical protein XOO0257 [Xanthomonas oryzae pv. oryzae KACC10331]
>gi|58579882|ref|YP_198898.1| hypothetical protein XOO0260 [Xanthomonas oryzae pv. oryzae KACC10331]

>gi|58579884|ref|YP_198900.1| hypothetical protein XOO0261 [Xanthomonas oryzae pv. oryzae KACC10331]
>gi|58579885|ref|YP_198901.1| IS1404 transposase [Xanthomonas oryzae pv. oryzae KACC10331]
>gi|58579893|ref|YP_198909.1| ferripyoverdine receptor [Xanthomonas oryzae pv. oryzae KACC10331]
>gi|58579912|ref|YP_198928.1| tetracycline-efflux transporter [Xanthomonas oryzae pv. oryzae KACC10331]
>gi|58579913|ref|YP_198929.1| tetracycline-efflux transporter [Xanthomonas oryzae pv. oryzae KACC10331]
>gi|58579914|ref|YP_198930.1| hypothetical protein XOO0291 [Xanthomonas oryzae pv. oryzae KACC10331]
>gi|58579916|ref|YP_198932.1| benzoate transporter [Xanthomonas oryzae pv. oryzae KACC10331]
>gi|58579919|ref|YP_198935.1| IS1404 transposase [Xanthomonas oryzae pv. oryzae KACC10331]
>gi|58579928|ref|YP_198944.1| lipoprotein [Xanthomonas oryzae pv. oryzae KACC10331]
>gi|58579929|ref|YP_198945.1| hypothetical protein XOO0306 [Xanthomonas oryzae pv. oryzae KACC10331]
>gi|58579938|ref|YP_198954.1| hypothetical protein XOO0315 [Xanthomonas oryzae pv. oryzae KACC10331]
>gi|58579970|ref|YP_198986.1| hypothetical protein XOO0347 [Xanthomonas oryzae pv. oryzae KACC10331]
>gi|58579974|ref|YP_198990.1| hypothetical protein XOO0351 [Xanthomonas oryzae pv. oryzae KACC10331]
>gi|58579996|ref|YP_199012.1| transposase [Xanthomonas oryzae pv. oryzae KACC10331]
>gi|58580006|ref|YP_199022.1| heavy metal transporter [Xanthomonas oryzae pv. oryzae KACC10331]
>gi|58580008|ref|YP_199024.1| hypothetical protein XOO0385 [Xanthomonas oryzae pv. oryzae KACC10331]
>gi|58580013|ref|YP_199029.1| TonB-like protein [Xanthomonas oryzae pv. oryzae KACC10331]
>gi|58580023|ref|YP_199039.1| hypothetical protein XOO0400 [Xanthomonas oryzae pv. oryzae KACC10331]
>gi|58580033|ref|YP_199049.1| hypothetical protein XOO0410 [Xanthomonas oryzae pv. oryzae KACC10331]
>gi|58580043|ref|YP_199059.1| hypothetical protein XOO0420 [Xanthomonas oryzae pv. oryzae KACC10331]
>gi|58580051|ref|YP_199067.1| biotin acetyl-CoA-carboxylase synthetase [Xanthomonas oryzae pv. oryzae KACC10331]
>gi|58580068|ref|YP_199084.1| hypothetical protein XOO0445 [Xanthomonas oryzae pv. oryzae KACC10331]
>gi|58580069|ref|YP_199085.1| cytochrome B561 [Xanthomonas oryzae pv. oryzae KACC10331]
>gi|58580072|ref|YP_199088.1| transmembrane regulator protein prtR [Xanthomonas oryzae pv. oryzae KACC10331]
>gi|58580088|ref|YP_199104.1| ankyrin-like protein [Xanthomonas oryzae pv. oryzae KACC10331]
>gi|58580107|ref|YP_199123.1| 3-carboxy-cis,cis-muconate cycloisomerase [Xanthomonas oryzae pv. oryzae KACC10331]
>gi|58580116|ref|YP_199132.1| hypothetical protein XOO0493 [Xanthomonas oryzae pv. oryzae KACC10331]
>gi|58580129|ref|YP_199145.1| hypothetical protein XOO0506 [Xanthomonas oryzae pv. oryzae KACC10331]
>gi|58580135|ref|YP_199151.1| hypothetical protein XOO0512 [Xanthomonas oryzae pv. oryzae KACC10331]
>gi|58580140|ref|YP_199156.1| IS1404 transposase [Xanthomonas oryzae pv. oryzae KACC10331]
>gi|58580141|ref|YP_199157.1| hypothetical protein XOO0518 [Xanthomonas oryzae pv. oryzae KACC10331]
>gi|58580142|ref|YP_199158.1| two-component system sensor protein [Xanthomonas oryzae pv. oryzae KACC10331]
>gi|58580144|ref|YP_199160.1| hypothetical protein XOO0521 [Xanthomonas oryzae pv. oryzae KACC10331]
>gi|58580147|ref|YP_199163.1| hypothetical protein XOO0524 [Xanthomonas oryzae pv. oryzae KACC10331]
>gi|58580151|ref|YP_199167.1| transmembrane protein [Xanthomonas oryzae pv. oryzae KACC10331]
>gi|58580154|ref|YP_199170.1| hypothetical protein XOO0531 [Xanthomonas oryzae pv. oryzae KACC10331]
>gi|58580156|ref|YP_199172.1| MFS transporter [Xanthomonas oryzae pv. oryzae KACC10331]
>gi|58580163|ref|YP_199179.1| CDP-alcohol phosphatidyltransferase [Xanthomonas oryzae pv. oryzae KACC10331]
>gi|58580172|ref|YP_199188.1| hypothetical protein XOO0549 [Xanthomonas oryzae pv. oryzae KACC10331]
>gi|58580174|ref|YP_199190.1| hypothetical protein XOO0551 [Xanthomonas oryzae pv. oryzae KACC10331]
>gi|58580176|ref|YP_199192.1| hypothetical protein XOO0553 [Xanthomonas oryzae pv. oryzae KACC10331]
>gi|58580181|ref|YP_199197.1| biotin carboxyl carrier protein of acetyl-CoA carboxilase [Xanthomonas oryzae pv. oryzae KACC10331]
>gi|58580183|ref|YP_199199.1| catabolic dehydroquinase [Xanthomonas oryzae pv. oryzae KACC10331]
>gi|58580184|ref|YP_199200.1| C-type cytochrome biogenesis protein (copper tolerance) [Xanthomonas oryzae pv. oryzae KACC10331]
>gi|58580191|ref|YP_199207.1| hypothetical protein XOO0568 [Xanthomonas oryzae pv. oryzae KACC10331]
>gi|58580192|ref|YP_199208.1| hypothetical protein XOO0569 [Xanthomonas oryzae pv. oryzae KACC10331]
>gi|58580194|ref|YP_199210.1| hypothetical protein XOO0571 [Xanthomonas oryzae pv. oryzae KACC10331]
>gi|58580195|ref|YP_199211.1| multidrug efflux protein [Xanthomonas oryzae pv. oryzae KACC10331]
>gi|58580199|ref|YP_199215.1| hypothetical protein XOO0576 [Xanthomonas oryzae pv. oryzae KACC10331]
>gi|58580200|ref|YP_199216.1| hypothetical protein XOO0577 [Xanthomonas oryzae pv. oryzae KACC10331]
>gi|58580203|ref|YP_199219.1| hypothetical protein XOO0580 [Xanthomonas oryzae pv. oryzae KACC10331]
>gi|58580204|ref|YP_199220.1| fimbrial protein [Xanthomonas oryzae pv. oryzae KACC10331]
>gi|58580247|ref|YP_199263.1| hypothetical protein XOO0624 [Xanthomonas oryzae pv. oryzae KACC10331]
>gi|58580248|ref|YP_199264.1| hypothetical protein XOO0625 [Xanthomonas oryzae pv. oryzae KACC10331]
>gi|58580252|ref|YP_199268.1| hypothetical protein XOO0629 [Xanthomonas oryzae pv. oryzae KACC10331]
>gi|58580253|ref|YP_199269.1| hypothetical protein XOO0630 [Xanthomonas oryzae pv. oryzae KACC10331]
>gi|58580257|ref|YP_199273.1| hypothetical protein XOO0634 [Xanthomonas oryzae pv. oryzae KACC10331]
>gi|58580263|ref|YP_199279.1| hypothetical protein XOO0640 [Xanthomonas oryzae pv. oryzae KACC10331]
>gi|58580274|ref|YP_199290.1| transcriptional regulator protein [Xanthomonas oryzae pv. oryzae KACC10331]
>gi|58580289|ref|YP_199305.1| hypothetical protein XOO0666 [Xanthomonas oryzae pv. oryzae KACC10331]
>gi|58580291|ref|YP_199307.1| hypothetical protein XOO0668 [Xanthomonas oryzae pv. oryzae KACC10331]
>gi|58580298|ref|YP_199314.1| Na+:H+ antiporter [Xanthomonas oryzae pv. oryzae KACC10331]
>gi|58580304|ref|YP_199320.1| outer membrane protein [Xanthomonas oryzae pv. oryzae KACC10331]
>gi|58580305|ref|YP_199321.1| histidine kinase/response regulator hybrid protein [Xanthomonas oryzae pv. oryzae KACC10331]
>gi|58580307|ref|YP_199323.1| hypothetical protein XOO0684 [Xanthomonas oryzae pv. oryzae KACC10331]
>gi|58580308|ref|YP_199324.1| hypothetical protein XOO0685 [Xanthomonas oryzae pv. oryzae KACC10331]
>gi|58580309|ref|YP_199325.1| hypothetical protein XOO0686 [Xanthomonas oryzae pv. oryzae KACC10331]
>gi|58580314|ref|YP_199330.1| hypothetical protein XOO0691 [Xanthomonas oryzae pv. oryzae KACC10331]
>gi|58580330|ref|YP_199346.1| hypothetical protein XOO0707 [Xanthomonas oryzae pv. oryzae KACC10331]
>gi|58580331|ref|YP_199347.1| hypothetical protein XOO0708 [Xanthomonas oryzae pv. oryzae KACC10331]

>gi|58580337|ref|YP_199353.1| ABC transporter permease [Xanthomonas oryzae pv. oryzae KACC10331]
>gi|58580348|ref|YP_199364.1| hypothetical protein XOO0725 [Xanthomonas oryzae pv. oryzae KACC10331]
>gi|58580371|ref|YP_199387.1| hypothetical protein XOO0748 [Xanthomonas oryzae pv. oryzae KACC10331]
>gi|58580380|ref|YP_199396.1| hypothetical protein XOO0757 [Xanthomonas oryzae pv. oryzae KACC10331]
>gi|58580383|ref|YP_199399.1| hypothetical protein XOO0760 [Xanthomonas oryzae pv. oryzae KACC10331]
>gi|58580390|ref|YP_199406.1| dethiobiotin synthetase [Xanthomonas oryzae pv. oryzae KACC10331]
>gi|58580398|ref|YP_199414.1| outer membrane protein [Xanthomonas oryzae pv. oryzae KACC10331]
>gi|58580413|ref|YP_199429.1| electron transfer flavoprotein alpha subunit [Xanthomonas oryzae pv. oryzae KACC10331]
>gi|58580428|ref|YP_199444.1| ABC transporter ATP-binding protein [Xanthomonas oryzae pv. oryzae KACC10331]
>gi|58580433|ref|YP_199449.1| hypothetical protein XOO0810 [Xanthomonas oryzae pv. oryzae KACC10331]
>gi|58580435|ref|YP_199451.1| ribosomal-protein-alanine acetyltransferase [Xanthomonas oryzae pv. oryzae KACC10331]
>gi|58580460|ref|YP_199476.1| hypothetical protein XOO0837 [Xanthomonas oryzae pv. oryzae KACC10331]
>gi|58580465|ref|YP_199481.1| outer membrane protein [Xanthomonas oryzae pv. oryzae KACC10331]
>gi|58580468|ref|YP_199484.1| protease [Xanthomonas oryzae pv. oryzae KACC10331]
>gi|58580477|ref|YP_199493.1| general secretion pathway protein L [Xanthomonas oryzae pv. oryzae KACC10331]
>gi|58580479|ref|YP_199495.1| general secretion pathway protein N [Xanthomonas oryzae pv. oryzae KACC10331]
>gi|58580480|ref|YP_199496.1| general secretion pathway protein D [Xanthomonas oryzae pv. oryzae KACC10331]
>gi|58580526|ref|YP_199542.1| hypothetical protein XOO0903 [Xanthomonas oryzae pv. oryzae KACC10331]
>gi|58580533|ref|YP_199549.1| curved DNA binding protein [Xanthomonas oryzae pv. oryzae KACC10331]
>gi|58580537|ref|YP_199553.1| hypothetical protein XOO0914 [Xanthomonas oryzae pv. oryzae KACC10331]
>gi|58580556|ref|YP_199572.1| hypothetical protein XOO0933 [Xanthomonas oryzae pv. oryzae KACC10331]
>gi|58580565|ref|YP_199581.1| threonine dehydratase catabolic [Xanthomonas oryzae pv. oryzae KACC10331]
>gi|58580566|ref|YP_199582.1| threonine dehydratase catabolic [Xanthomonas oryzae pv. oryzae KACC10331]
>gi|58580605|ref|YP_199621.1| IS1478 transposase [Xanthomonas oryzae pv. oryzae KACC10331]
>gi|58580623|ref|YP_199639.1| hypothetical protein XOO1000 [Xanthomonas oryzae pv. oryzae KACC10331]
>gi|58580687|ref|YP_199703.1| DNA polymerase III tau and gamma subunits [Xanthomonas oryzae pv. oryzae KACC10331]
>gi|58580693|ref|YP_199709.1| hypothetical protein XOO1070 [Xanthomonas oryzae pv. oryzae KACC10331]
>gi|58580714|ref|YP_199730.1| acetylxylan esterase [Xanthomonas oryzae pv. oryzae KACC10331]
>gi|58580744|ref|YP_199760.1| hypothetical protein XOO1121 [Xanthomonas oryzae pv. oryzae KACC10331]
>gi|58580746|ref|YP_199762.1| thiamin-phosphate pyrophosphorylase [Xanthomonas oryzae pv. oryzae KACC10331]
>gi|58580754|ref|YP_199770.1| hypothetical protein XOO1131 [Xanthomonas oryzae pv. oryzae KACC10331]
>gi|58580757|ref|YP_199773.1| hypothetical protein XOO1134 [Xanthomonas oryzae pv. oryzae KACC10331]
>gi|58580762|ref|YP_199778.1| hypothetical protein XOO1139 [Xanthomonas oryzae pv. oryzae KACC10331]
>gi|58580767|ref|YP_199783.1| hypothetical protein XOO1144 [Xanthomonas oryzae pv. oryzae KACC10331]
>gi|58580771|ref|YP_199787.1| RNA polymerase omega subunit [Xanthomonas oryzae pv. oryzae KACC10331]
>gi|58580781|ref|YP_199797.1| fimbrial assembly membrane protein [Xanthomonas oryzae pv. oryzae KACC10331]
>gi|58580789|ref|YP_199805.1| transposase [Xanthomonas oryzae pv. oryzae KACC10331]
>gi|58580794|ref|YP_199810.1| hypothetical protein XOO1171 [Xanthomonas oryzae pv. oryzae KACC10331]
>gi|58580797|ref|YP_199813.1| hypothetical protein XOO1174 [Xanthomonas oryzae pv. oryzae KACC10331]
>gi|58580801|ref|YP_199817.1| hypothetical protein XOO1178 [Xanthomonas oryzae pv. oryzae KACC10331]
>gi|58580802|ref|YP_199818.1| hypothetical protein XOO1179 [Xanthomonas oryzae pv. oryzae KACC10331]
>gi|58580809|ref|YP_199825.1| Dehydrogenases with different specificities (related to short-chain alcohol dehydrogenases) [Xanthomonas oryzae pv. oryzae KACC10331]
>gi|58580811|ref|YP_199827.1| Response regulators consisting of a CheY-like receiver domain and a winged-helix DNA-binding domain [Xanthomonas oryzae pv. oryzae KACC10331]
>gi|58580814|ref|YP_199830.1| TonB-like protein [Xanthomonas oryzae pv. oryzae KACC10331]
>gi|58580819|ref|YP_199835.1| beta-lactamase [Xanthomonas oryzae pv. oryzae KACC10331]
>gi|58580824|ref|YP_199840.1| outer membrane protein [Xanthomonas oryzae pv. oryzae KACC10331]
>gi|58580851|ref|YP_199867.1| histidinol-phosphate aminotransferase [Xanthomonas oryzae pv. oryzae KACC10331]
>gi|58580854|ref|YP_199870.1| transcriptional regulator [Xanthomonas oryzae pv. oryzae KACC10331]
>gi|58580860|ref|YP_199876.1| avirulence protein [Xanthomonas oryzae pv. oryzae KACC10331]
>gi|58580861|ref|YP_199877.1| avirulence protein [Xanthomonas oryzae pv. oryzae KACC10331]
>gi|58580874|ref|YP_199890.1| hypothetical protein XOO1251 [Xanthomonas oryzae pv. oryzae KACC10331]
>gi|58580876|ref|YP_199892.1| MFS transporter [Xanthomonas oryzae pv. oryzae KACC10331]
>gi|58580878|ref|YP_199894.1| MFS transporter [Xanthomonas oryzae pv. oryzae KACC10331]
>gi|58580879|ref|YP_199895.1| MFS transporter [Xanthomonas oryzae pv. oryzae KACC10331]
>gi|58580887|ref|YP_199903.1| hydrolase [Xanthomonas oryzae pv. oryzae KACC10331]
>gi|58580916|ref|YP_199932.1| hypothetical protein XOO1293 [Xanthomonas oryzae pv. oryzae KACC10331]
>gi|58580924|ref|YP_199940.1| hypothetical protein XOO1301 [Xanthomonas oryzae pv. oryzae KACC10331]
>gi|58580927|ref|YP_199943.1| hypothetical protein XOO1304 [Xanthomonas oryzae pv. oryzae KACC10331]
>gi|58580933|ref|YP_199949.1| hypothetical protein XOO1310 [Xanthomonas oryzae pv. oryzae KACC10331]
>gi|58580942|ref|YP_199958.1| hypothetical protein XOO1319 [Xanthomonas oryzae pv. oryzae KACC10331]
>gi|58580946|ref|YP_199962.1| 2-nitropropane dioxygenase [Xanthomonas oryzae pv. oryzae KACC10331]
>gi|58580959|ref|YP_199975.1| hypothetical protein XOO1336 [Xanthomonas oryzae pv. oryzae KACC10331]
>gi|58580963|ref|YP_199979.1| hypothetical protein XOO1340 [Xanthomonas oryzae pv. oryzae KACC10331]
>gi|58580976|ref|YP_199992.1| cobalamin synthase [Xanthomonas oryzae pv. oryzae KACC10331]
>gi|58580979|ref|YP_199995.1| transport protein [Xanthomonas oryzae pv. oryzae KACC10331]
>gi|58581019|ref|YP_200035.1| IS1404 transposase [Xanthomonas oryzae pv. oryzae KACC10331]
>gi|58581024|ref|YP_200040.1| acetyltransferase [Xanthomonas oryzae pv. oryzae KACC10331]
>gi|58581034|ref|YP_200050.1| hypothetical protein XOO1411 [Xanthomonas oryzae pv. oryzae KACC10331]

>gi|58581037|ref|YP_200053.1| histone-like protein [Xanthomonas oryzae pv. oryzae KACC10331]
>gi|58581039|ref|YP_200055.1| pyrroline-5-carboxylate reductase [Xanthomonas oryzae pv. oryzae KACC10331]
>gi|58581044|ref|YP_200060.1| hypothetical protein XOO1421 [Xanthomonas oryzae pv. oryzae KACC10331]
>gi|58581045|ref|YP_200061.1| hypothetical protein XOO1422 [Xanthomonas oryzae pv. oryzae KACC10331]
>gi|58581052|ref|YP_200068.1| hypothetical protein XOO1429 [Xanthomonas oryzae pv. oryzae KACC10331]
>gi|58581057|ref|YP_200073.1| hypothetical protein XOO1434 [Xanthomonas oryzae pv. oryzae KACC10331]
>gi|58581062|ref|YP_200078.1| single-stranded DNA binding protein [Xanthomonas oryzae pv. oryzae KACC10331]
>gi|58581084|ref|YP_200100.1| hypothetical protein XOO1461 [Xanthomonas oryzae pv. oryzae KACC10331]
>gi|58581104|ref|YP_200120.1| inner membrane protein [Xanthomonas oryzae pv. oryzae KACC10331]
>gi|58581105|ref|YP_200121.1| hypothetical protein o263 [Xanthomonas oryzae pv. oryzae KACC10331]
>gi|58581112|ref|YP_200128.1| hypothetical protein XOO1489 [Xanthomonas oryzae pv. oryzae KACC10331]
>gi|58581113|ref|YP_200129.1| hypothetical protein XOO1490 [Xanthomonas oryzae pv. oryzae KACC10331]
>gi|58581132|ref|YP_200148.1| hypothetical protein XOO1509 [Xanthomonas oryzae pv. oryzae KACC10331]
>gi|58581134|ref|YP_200150.1| peptidoglycan-associated outer membrane lipoprotein [Xanthomonas oryzae pv. oryzae KACC10331]
>gi|58581138|ref|YP_200154.1| hypothetical protein XOO1515 [Xanthomonas oryzae pv. oryzae KACC10331]
>gi|58581139|ref|YP_200155.1| hypothetical protein XOO1516 [Xanthomonas oryzae pv. oryzae KACC10331]
>gi|58581143|ref|YP_200159.1| hypothetical protein XOO1520 [Xanthomonas oryzae pv. oryzae KACC10331]
>gi|58581144|ref|YP_200160.1| glutathione S-transferase [Xanthomonas oryzae pv. oryzae KACC10331]
>gi|58581145|ref|YP_200161.1| hypothetical protein XOO1522 [Xanthomonas oryzae pv. oryzae KACC10331]
>gi|58581150|ref|YP_200166.1| OmpA family protein [Xanthomonas oryzae pv. oryzae KACC10331]
>gi|58581156|ref|YP_200172.1| hypothetical protein XOO1533 [Xanthomonas oryzae pv. oryzae KACC10331]
>gi|58581157|ref|YP_200173.1| hypothetical protein XOO1534 [Xanthomonas oryzae pv. oryzae KACC10331]
>gi|58581164|ref|YP_200180.1| hypothetical protein XOO1541 [Xanthomonas oryzae pv. oryzae KACC10331]
>gi|58581165|ref|YP_200181.1| hypothetical protein XOO1542 [Xanthomonas oryzae pv. oryzae KACC10331]
>gi|58581166|ref|YP_200182.1| hypothetical protein XOO1543 [Xanthomonas oryzae pv. oryzae KACC10331]
>gi|58581176|ref|YP_200192.1| hypothetical protein XOO1553 [Xanthomonas oryzae pv. oryzae KACC10331]
>gi|58581185|ref|YP_200201.1| RND efflux membrane fusion protein [Xanthomonas oryzae pv. oryzae KACC10331]
>gi|58581230|ref|YP_200246.1| hypothetical protein XOO1607 [Xanthomonas oryzae pv. oryzae KACC10331]
>gi|58581263|ref|YP_200279.1| IS1404 transposase [Xanthomonas oryzae pv. oryzae KACC10331]
>gi|58581276|ref|YP_200292.1| transmembrane transport protein [Xanthomonas oryzae pv. oryzae KACC10331]
>gi|58581278|ref|YP_200294.1| hypothetical protein XOO1655 [Xanthomonas oryzae pv. oryzae KACC10331]
>gi|58581290|ref|YP_200306.1| TolA [Xanthomonas oryzae pv. oryzae KACC10331]
>gi|58581293|ref|YP_200309.1| hypothetical protein XOO1670 [Xanthomonas oryzae pv. oryzae KACC10331]
>gi|58581311|ref|YP_200327.1| hypothetical protein XOO1688 [Xanthomonas oryzae pv. oryzae KACC10331]
>gi|58581314|ref|YP_200330.1| phage-related tail protein [Xanthomonas oryzae pv. oryzae KACC10331]
>gi|58581320|ref|YP_200336.1| phage-related baseplate protein [Xanthomonas oryzae pv. oryzae KACC10331]
>gi|58581321|ref|YP_200337.1| hypothetical protein XOO1698 [Xanthomonas oryzae pv. oryzae KACC10331]
>gi|58581334|ref|YP_200350.1| hypothetical protein XOO1711 [Xanthomonas oryzae pv. oryzae KACC10331]
>gi|58581352|ref|YP_200368.1| glucose kinase [Xanthomonas oryzae pv. oryzae KACC10331]
>gi|58581360|ref|YP_200376.1| hypothetical protein XOO1737 [Xanthomonas oryzae pv. oryzae KACC10331]
>gi|58581363|ref|YP_200379.1| hypothetical protein XOO1740 [Xanthomonas oryzae pv. oryzae KACC10331]
>gi|58581365|ref|YP_200381.1| hypothetical protein XOO1742 [Xanthomonas oryzae pv. oryzae KACC10331]
>gi|58581373|ref|YP_200389.1| PilL [Xanthomonas oryzae pv. oryzae KACC10331]
>gi|58581391|ref|YP_200407.1| hypothetical protein XOO1768 [Xanthomonas oryzae pv. oryzae KACC10331]
>gi|58581411|ref|YP_200427.1| sulfite synthesis pathway protein [Xanthomonas oryzae pv. oryzae KACC10331]
>gi|58581414|ref|YP_200430.1| hypothetical protein XOO1791 [Xanthomonas oryzae pv. oryzae KACC10331]
>gi|58581415|ref|YP_200431.1| hypothetical protein XOO1792 [Xanthomonas oryzae pv. oryzae KACC10331]
>gi|58581416|ref|YP_200432.1| hypothetical protein XOO1793 [Xanthomonas oryzae pv. oryzae KACC10331]
>gi|58581418|ref|YP_200434.1| glycine cleavage H protein [Xanthomonas oryzae pv. oryzae KACC10331]
>gi|58581419|ref|YP_200435.1| hypothetical protein XOO1796 [Xanthomonas oryzae pv. oryzae KACC10331]
>gi|58581442|ref|YP_200458.1| homoserine dehydrogenase [Xanthomonas oryzae pv. oryzae KACC10331]
>gi|58581446|ref|YP_200462.1| cold shock domain protein [Xanthomonas oryzae pv. oryzae KACC10331]
>gi|58581473|ref|YP_200489.1| regulatory protein [Xanthomonas oryzae pv. oryzae KACC10331]
>gi|58581500|ref|YP_200516.1| hypothetical protein XOO1877 [Xanthomonas oryzae pv. oryzae KACC10331]
>gi|58581501|ref|YP_200517.1| hypothetical protein XOO1878 [Xanthomonas oryzae pv. oryzae KACC10331]
>gi|58581502|ref|YP_200518.1| sensor histidine kinase [Xanthomonas oryzae pv. oryzae KACC10331]
>gi|58581503|ref|YP_200519.1| hypothetical protein XOO1880 [Xanthomonas oryzae pv. oryzae KACC10331]
>gi|58581508|ref|YP_200524.1| hypothetical protein XOO1885 [Xanthomonas oryzae pv. oryzae KACC10331]
>gi|58581536|ref|YP_200552.1| hypothetical protein XOO1913 [Xanthomonas oryzae pv. oryzae KACC10331]
>gi|58581537|ref|YP_200553.1| cyclopropane-fatty-acyl-phospholipid synthase [Xanthomonas oryzae pv. oryzae KACC10331]
>gi|58581542|ref|YP_200558.1| RNA polymerase sigma factor [Xanthomonas oryzae pv. oryzae KACC10331]
>gi|58581558|ref|YP_200574.1| hypothetical protein XOO1935 [Xanthomonas oryzae pv. oryzae KACC10331]
>gi|58581562|ref|YP_200578.1| NagN [Xanthomonas oryzae pv. oryzae KACC10331]
>gi|58581568|ref|YP_200584.1| NagN [Xanthomonas oryzae pv. oryzae KACC10331]
>gi|58581579|ref|YP_200595.1| hypothetical protein XOO1956 [Xanthomonas oryzae pv. oryzae KACC10331]
>gi|58581580|ref|YP_200596.1| hypothetical protein XOO1957 [Xanthomonas oryzae pv. oryzae KACC10331]
>gi|58581590|ref|YP_200606.1| UDP-3-O-(R-3-hydroxymyristoyl)-glucosamine N-acyltransferase [Xanthomonas oryzae pv. oryzae KACC10331]
>gi|58581593|ref|YP_200609.1| 1-deoxy-D-xylulose 5-phosphate reductoisomerase [Xanthomonas oryzae pv. oryzae KACC10331]
>gi|58581594|ref|YP_200610.1| phosphatidate cytidylyltransferase [Xanthomonas oryzae pv. oryzae KACC10331]
>gi|58581605|ref|YP_200621.1| protein U [Xanthomonas oryzae pv. oryzae KACC10331]

>gi|58581608|ref|YP_200624.1| 2,3,4,5-tetrahydropyridine-2-carboxylate N-succin [Xanthomonas oryzae pv. oryzae KACC10331]
>gi|58581620|ref|YP_200636.1| transcriptional regulator [Xanthomonas oryzae pv. oryzae KACC10331]
>gi|58581637|ref|YP_200653.1| glycosyltransferase [Xanthomonas oryzae pv. oryzae KACC10331]
>gi|58581655|ref|YP_200671.1| DnaJ [Xanthomonas oryzae pv. oryzae KACC10331]
>gi|58581683|ref|YP_200699.1| hypothetical protein XOO2060 [Xanthomonas oryzae pv. oryzae KACC10331]
>gi|58581690|ref|YP_200706.1| hypothetical protein XOO2067 [Xanthomonas oryzae pv. oryzae KACC10331]
>gi|58581738|ref|YP_200754.1| hypothetical protein XOO2115 [Xanthomonas oryzae pv. oryzae KACC10331]
>gi|58581750|ref|YP_200766.1| hypothetical 125K protein [Xanthomonas oryzae pv. oryzae KACC10331]
>gi|58581751|ref|YP_200767.1| hypothetical 125K protein [Xanthomonas oryzae pv. oryzae KACC10331]
>gi|58581754|ref|YP_200770.1| avirulence protein [Xanthomonas oryzae pv. oryzae KACC10331]
>gi|58581757|ref|YP_200773.1| hypothetical protein XOO2134 [Xanthomonas oryzae pv. oryzae KACC10331]
>gi|58581758|ref|YP_200774.1| hypothetical protein XOO2135 [Xanthomonas oryzae pv. oryzae KACC10331]
>gi|58581773|ref|YP_200789.1| C-type cytochrome biogenesis membrane protein [Xanthomonas oryzae pv. oryzae KACC10331]
>gi|58581775|ref|YP_200791.1| hypothetical protein XOO2152 [Xanthomonas oryzae pv. oryzae KACC10331]
>gi|58581777|ref|YP_200793.1| ABC transporter heme permease [Xanthomonas oryzae pv. oryzae KACC10331]
>gi|58581784|ref|YP_200800.1| pseudouridylate synthase [Xanthomonas oryzae pv. oryzae KACC10331]
>gi|58581785|ref|YP_200801.1| hypothetical protein XOO2162 [Xanthomonas oryzae pv. oryzae KACC10331]
>gi|58581787|ref|YP_200803.1| hypothetical protein XOO2164 [Xanthomonas oryzae pv. oryzae KACC10331]
>gi|58581810|ref|YP_200826.1| transport protein [Xanthomonas oryzae pv. oryzae KACC10331]
>gi|58581811|ref|YP_200827.1| transport protein [Xanthomonas oryzae pv. oryzae KACC10331]
>gi|58581820|ref|YP_200836.1| NADH dehydrogenase [Xanthomonas oryzae pv. oryzae KACC10331]
>gi|58581823|ref|YP_200839.1| hypothetical protein XOO2200 [Xanthomonas oryzae pv. oryzae KACC10331]
>gi|58581843|ref|YP_200859.1| PHA synthase subunit [Xanthomonas oryzae pv. oryzae KACC10331]
>gi|58581855|ref|YP_200871.1| hypothetical protein XOO2232 [Xanthomonas oryzae pv. oryzae KACC10331]
>gi|58581866|ref|YP_200882.1| homoserine kinase [Xanthomonas oryzae pv. oryzae KACC10331]
>gi|58581871|ref|YP_200887.1| hypothetical protein XOO2248 [Xanthomonas oryzae pv. oryzae KACC10331]
>gi|58581872|ref|YP_200888.1| fumarate and nitrate reduction regulatory protein [Xanthomonas oryzae pv. oryzae KACC10331]
>gi|58581879|ref|YP_200895.1| histidinol dehydrogenase [Xanthomonas oryzae pv. oryzae KACC10331]
>gi|58581887|ref|YP_200903.1| hypothetical 125K protein [Xanthomonas oryzae pv. oryzae KACC10331]
>gi|58581898|ref|YP_200914.1| avirulence protein [Xanthomonas oryzae pv. oryzae KACC10331]
>gi|58581899|ref|YP_200915.1| hypothetical 125K protein [Xanthomonas oryzae pv. oryzae KACC10331]
>gi|58581902|ref|YP_200918.1| avirulence protein [Xanthomonas oryzae pv. oryzae KACC10331]
>gi|58581905|ref|YP_200921.1| transposase [Xanthomonas oryzae pv. oryzae KACC10331]
>gi|58581923|ref|YP_200939.1| competence protein [Xanthomonas oryzae pv. oryzae KACC10331]
>gi|58581965|ref|YP_200981.1| extracellular protease [Xanthomonas oryzae pv. oryzae KACC10331]
>gi|58581976|ref|YP_200992.1| hypothetical protein XOO2353 [Xanthomonas oryzae pv. oryzae KACC10331]
>gi|58581982|ref|YP_200998.1| hypothetical protein XOO2359 [Xanthomonas oryzae pv. oryzae KACC10331]
>gi|58581988|ref|YP_201004.1| 2-amino-4-hydroxy-6-hydroxymethyldihydropteridine [Xanthomonas oryzae pv. oryzae KACC10331]
>gi|58581991|ref|YP_201007.1| N-acetylmuramoyl-L-alanine amidase [Xanthomonas oryzae pv. oryzae KACC10331]
>gi|58581993|ref|YP_201009.1| NagN [Xanthomonas oryzae pv. oryzae KACC10331]
>gi|58581995|ref|YP_201011.1| IS1404 transposase [Xanthomonas oryzae pv. oryzae KACC10331]
>gi|58581996|ref|YP_201012.1| hypothetical protein XOO2373 [Xanthomonas oryzae pv. oryzae KACC10331]
>gi|58581999|ref|YP_201015.1| cation efflux system protein [Xanthomonas oryzae pv. oryzae KACC10331]
>gi|58582000|ref|YP_201016.1| cation efflux system protein [Xanthomonas oryzae pv. oryzae KACC10331]
>gi|58582003|ref|YP_201019.1| YapH protein [Xanthomonas oryzae pv. oryzae KACC10331]
>gi|58582017|ref|YP_201033.1| hypothetical protein XOO2394 [Xanthomonas oryzae pv. oryzae KACC10331]
>gi|58582025|ref|YP_201041.1| hypothetical protein XOO2402 [Xanthomonas oryzae pv. oryzae KACC10331]
>gi|58582030|ref|YP_201046.1| initiation factor eIF-2B [Xanthomonas oryzae pv. oryzae KACC10331]
>gi|58582048|ref|YP_201064.1| hypothetical protein XOO2425 [Xanthomonas oryzae pv. oryzae KACC10331]
>gi|58582049|ref|YP_201065.1| hypothetical protein XOO2426 [Xanthomonas oryzae pv. oryzae KACC10331]
>gi|58582063|ref|YP_201079.1| ABC transporter ATP-binding protein [Xanthomonas oryzae pv. oryzae KACC10331]
>gi|58582070|ref|YP_201086.1| thiosulfate sulfurtransferase [Xanthomonas oryzae pv. oryzae KACC10331]
>gi|58582075|ref|YP_201091.1| 3-hydroxybutyryl-CoA dehydratase [Xanthomonas oryzae pv. oryzae KACC10331]
>gi|58582076|ref|YP_201092.1| hypothetical protein XOO2453 [Xanthomonas oryzae pv. oryzae KACC10331]
>gi|58582090|ref|YP_201106.1| hypothetical protein XOO2467 [Xanthomonas oryzae pv. oryzae KACC10331]
>gi|58582103|ref|YP_201119.1| hypothetical protein XOO2480 [Xanthomonas oryzae pv. oryzae KACC10331]
>gi|58582130|ref|YP_201146.1| hypothetical protein XOO2507 [Xanthomonas oryzae pv. oryzae KACC10331]
>gi|58582132|ref|YP_201148.1| hypothetical protein XOO2509 [Xanthomonas oryzae pv. oryzae KACC10331]
>gi|58582163|ref|YP_201179.1| hypothetical protein XOO2540 [Xanthomonas oryzae pv. oryzae KACC10331]
>gi|58582182|ref|YP_201198.1| hypothetical protein XOO2559 [Xanthomonas oryzae pv. oryzae KACC10331]
>gi|58582201|ref|YP_201217.1| flagellar protein [Xanthomonas oryzae pv. oryzae KACC10331]
>gi|58582204|ref|YP_201220.1| flagellar protein [Xanthomonas oryzae pv. oryzae KACC10331]
>gi|58582218|ref|YP_201234.1| acetyltransferase [Xanthomonas oryzae pv. oryzae KACC10331]
>gi|58582229|ref|YP_201245.1| flagellar protein [Xanthomonas oryzae pv. oryzae KACC10331]
>gi|58582233|ref|YP_201249.1| flagellar protein [Xanthomonas oryzae pv. oryzae KACC10331]
>gi|58582234|ref|YP_201250.1| flagellar biosynthetic protein [Xanthomonas oryzae pv. oryzae KACC10331]
>gi|58582242|ref|YP_201258.1| flagellar biosynthetic protein FlhF [Xanthomonas oryzae pv. oryzae KACC10331]
>gi|58582255|ref|YP_201271.1| Glutamate synthase domain 2 [Xanthomonas oryzae pv. oryzae KACC10331]
>gi|58582258|ref|YP_201274.1| Glutamate synthase domain 2 [Xanthomonas oryzae pv. oryzae KACC10331]
>gi|58582268|ref|YP_201284.1| cytochrome C4 [Xanthomonas oryzae pv. oryzae KACC10331]

>gi|58582276|ref|YP_201292.1| ABC transporter ATP-binding protein [Xanthomonas oryzae pv. oryzae KACC10331]
>gi|58582277|ref|YP_201293.1| transport protein [Xanthomonas oryzae pv. oryzae KACC10331]
>gi|58582278|ref|YP_201294.1| hypothetical protein XOO2655 [Xanthomonas oryzae pv. oryzae KACC10331]
>gi|58582286|ref|YP_201302.1| MFS transporter [Xanthomonas oryzae pv. oryzae KACC10331]
>gi|58582288|ref|YP_201304.1| gamma-glutamyl phosphate reductase [Xanthomonas oryzae pv. oryzae KACC10331]
>gi|58582305|ref|YP_201321.1| DnaK supressor [Xanthomonas oryzae pv. oryzae KACC10331]
>gi|58582323|ref|YP_201339.1| hypothetical protein XOO2700 [Xanthomonas oryzae pv. oryzae KACC10331]
>gi|58582327|ref|YP_201343.1| hypothetical protein XOO2704 [Xanthomonas oryzae pv. oryzae KACC10331]
>gi|58582329|ref|YP_201345.1| hypothetical protein XOO2706 [Xanthomonas oryzae pv. oryzae KACC10331]
>gi|58582338|ref|YP_201354.1| ABC transporter ATP-binding protein [Xanthomonas oryzae pv. oryzae KACC10331]
>gi|58582340|ref|YP_201356.1| adenine phosphoribosyltransferase [Xanthomonas oryzae pv. oryzae KACC10331]
>gi|58582356|ref|YP_201372.1| N-acetylmuramoyl-L-alanine amidase [Xanthomonas oryzae pv. oryzae KACC10331]
>gi|58582358|ref|YP_201374.1| hypothetical protein XOO2735 [Xanthomonas oryzae pv. oryzae KACC10331]
>gi|58582386|ref|YP_201402.1| soluble lytic murein transglycosylase [Xanthomonas oryzae pv. oryzae KACC10331]
>gi|58582387|ref|YP_201403.1| repressor [Xanthomonas oryzae pv. oryzae KACC10331]
>gi|58582403|ref|YP_201419.1| transport protein [Xanthomonas oryzae pv. oryzae KACC10331]
>gi|58582418|ref|YP_201434.1| hypothetical protein XOO2795 [Xanthomonas oryzae pv. oryzae KACC10331]
>gi|58582427|ref|YP_201443.1| multidrug resistance protein [Xanthomonas oryzae pv. oryzae KACC10331]
>gi|58582435|ref|YP_201451.1| PTS system, fructose-specific IIBC component [Xanthomonas oryzae pv. oryzae KACC10331]
>gi|58582444|ref|YP_201460.1| hypothetical protein XOO2821 [Xanthomonas oryzae pv. oryzae KACC10331]
>gi|58582456|ref|YP_201472.1| chemotaxis protein [Xanthomonas oryzae pv. oryzae KACC10331]
>gi|58582460|ref|YP_201476.1| transposase [Xanthomonas oryzae pv. oryzae KACC10331]
>gi|58582461|ref|YP_201477.1| chemotaxis protein [Xanthomonas oryzae pv. oryzae KACC10331]
>gi|58582479|ref|YP_201495.1| chemotaxis protein [Xanthomonas oryzae pv. oryzae KACC10331]
>gi|58582501|ref|YP_201517.1| regulatory protein [Xanthomonas oryzae pv. oryzae KACC10331]
>gi|58582527|ref|YP_201543.1| 3-hydroxyacyl-CoA dehydrogenase type II [Xanthomonas oryzae pv. oryzae KACC10331]
>gi|58582538|ref|YP_201554.1| relaxation protein [Xanthomonas oryzae pv. oryzae KACC10331]
>gi|58582541|ref|YP_201557.1| regucalcin [Xanthomonas oryzae pv. oryzae KACC10331]
>gi|58582554|ref|YP_201570.1| hypothetical protein XOO2931 [Xanthomonas oryzae pv. oryzae KACC10331]
>gi|58582574|ref|YP_201590.1| hypothetical protein XOO2951 [Xanthomonas oryzae pv. oryzae KACC10331]
>gi|58582581|ref|YP_201597.1| salivary glue protein [Xanthomonas oryzae pv. oryzae KACC10331]
>gi|58582587|ref|YP_201603.1| hypothetical protein XOO2964 [Xanthomonas oryzae pv. oryzae KACC10331]
>gi|58582588|ref|YP_201604.1| hypothetical protein XOO2965 [Xanthomonas oryzae pv. oryzae KACC10331]
>gi|58582591|ref|YP_201607.1| hypothetical protein XOO2968 [Xanthomonas oryzae pv. oryzae KACC10331]
>gi|58582600|ref|YP_201616.1| ExoD protein [Xanthomonas oryzae pv. oryzae KACC10331]
>gi|58582613|ref|YP_201629.1| hypothetical protein XOO2990 [Xanthomonas oryzae pv. oryzae KACC10331]
>gi|58582622|ref|YP_201638.1| cytochrome like B561 [Xanthomonas oryzae pv. oryzae KACC10331]
>gi|58582627|ref|YP_201643.1| serine protease [Xanthomonas oryzae pv. oryzae KACC10331]
>gi|58582630|ref|YP_201646.1| C-type cytochrome biogenesis protein [Xanthomonas oryzae pv. oryzae KACC10331]
>gi|58582631|ref|YP_201647.1| hypothetical protein XOO3008 [Xanthomonas oryzae pv. oryzae KACC10331]
>gi|58582635|ref|YP_201651.1| hypothetical protein XOO3012 [Xanthomonas oryzae pv. oryzae KACC10331]
>gi|58582636|ref|YP_201652.1| avirulence protein [Xanthomonas oryzae pv. oryzae KACC10331]
>gi|58582637|ref|YP_201653.1| avirulence protein [Xanthomonas oryzae pv. oryzae KACC10331]
>gi|58582638|ref|YP_201654.1| avirulence protein [Xanthomonas oryzae pv. oryzae KACC10331]
>gi|58582660|ref|YP_201676.1| hypothetical protein XOO3037 [Xanthomonas oryzae pv. oryzae KACC10331]
>gi|58582662|ref|YP_201678.1| hypothetical protein XOO3039 [Xanthomonas oryzae pv. oryzae KACC10331]
>gi|58582663|ref|YP_201679.1| hypothetical protein XOO3040 [Xanthomonas oryzae pv. oryzae KACC10331]
>gi|58582665|ref|YP_201681.1| hypothetical protein XOO3042 [Xanthomonas oryzae pv. oryzae KACC10331]
>gi|58582678|ref|YP_201694.1| Orf2/integrase/recombinase fusion protein [Xanthomonas oryzae pv. oryzae KACC10331]
>gi|58582688|ref|YP_201704.1| hypothetical protein XOO3065 [Xanthomonas oryzae pv. oryzae KACC10331]
>gi|58582692|ref|YP_201708.1| hypothetical protein XOO3069 [Xanthomonas oryzae pv. oryzae KACC10331]
>gi|58582693|ref|YP_201709.1| hypothetical protein XOO3070 [Xanthomonas oryzae pv. oryzae KACC10331]
>gi|58582694|ref|YP_201710.1| hypothetical protein XOO3071 [Xanthomonas oryzae pv. oryzae KACC10331]
>gi|58582696|ref|YP_201712.1| ribonuclease E [Xanthomonas oryzae pv. oryzae KACC10331]
>gi|58582697|ref|YP_201713.1| ribonuclease E [Xanthomonas oryzae pv. oryzae KACC10331]
>gi|58582721|ref|YP_201737.1| nitrate transport ATP-binding protein [Xanthomonas oryzae pv. oryzae KACC10331]
>gi|58582731|ref|YP_201747.1| exodeoxyribonuclease III [Xanthomonas oryzae pv. oryzae KACC10331]
>gi|58582749|ref|YP_201765.1| hypothetical protein XOO3126 [Xanthomonas oryzae pv. oryzae KACC10331]
>gi|58582759|ref|YP_201775.1| ketoglutarate semialdehyde dehydrogenase [Xanthomonas oryzae pv. oryzae KACC10331]
>gi|58582761|ref|YP_201777.1| oxidoreductase [Xanthomonas oryzae pv. oryzae KACC10331]
>gi|58582766|ref|YP_201782.1| cell division protein [Xanthomonas oryzae pv. oryzae KACC10331]
>gi|58582779|ref|YP_201795.1| hypothetical protein XOO3156 [Xanthomonas oryzae pv. oryzae KACC10331]
>gi|58582797|ref|YP_201813.1| GumG [Xanthomonas oryzae pv. oryzae KACC10331]
>gi|58582812|ref|YP_201828.1| IS1404 transposase [Xanthomonas oryzae pv. oryzae KACC10331]
>gi|58582829|ref|YP_201845.1| short chain dehydrogenase [Xanthomonas oryzae pv. oryzae KACC10331]
>gi|58582849|ref|YP_201865.1| NADH-ubiquinone oxidoreductase NQO10 subunit [Xanthomonas oryzae pv. oryzae KACC10331]
>gi|58582860|ref|YP_201876.1| protein-export membrane protein [Xanthomonas oryzae pv. oryzae KACC10331]
>gi|58582881|ref|YP_201897.1| FimV [Xanthomonas oryzae pv. oryzae KACC10331]
>gi|58582891|ref|YP_201907.1| hypothetical protein XOO3268 [Xanthomonas oryzae pv. oryzae KACC10331]
>gi|58582892|ref|YP_201908.1| integral membrane protein [Xanthomonas oryzae pv. oryzae KACC10331]

>gi|58582903|ref|YP_201919.1| transposase [Xanthomonas oryzae pv. oryzae KACC10331]
>gi|58582912|ref|YP_201928.1| transport protein [Xanthomonas oryzae pv. oryzae KACC10331]
>gi|58582914|ref|YP_201930.1| lipoprotein [Xanthomonas oryzae pv. oryzae KACC10331]
>gi|58582915|ref|YP_201931.1| hypothetical protein XOO3292 [Xanthomonas oryzae pv. oryzae KACC10331]
>gi|58582920|ref|YP_201936.1| cell cycle protein [Xanthomonas oryzae pv. oryzae KACC10331]
>gi|58582925|ref|YP_201941.1| hypothetical protein XOO3302 [Xanthomonas oryzae pv. oryzae KACC10331]
>gi|58582935|ref|YP_201951.1| hypothetical protein XOO3312 [Xanthomonas oryzae pv. oryzae KACC10331]
>gi|58582937|ref|YP_201953.1| hypothetical protein XOO3314 [Xanthomonas oryzae pv. oryzae KACC10331]
>gi|58582953|ref|YP_201969.1| hypothetical protein XOO3330 [Xanthomonas oryzae pv. oryzae KACC10331]
>gi|58582954|ref|YP_201970.1| hypothetical protein XOO3331 [Xanthomonas oryzae pv. oryzae KACC10331]
>gi|58582955|ref|YP_201971.1| thioredoxin [Xanthomonas oryzae pv. oryzae KACC10331]
>gi|58582963|ref|YP_201979.1| peptidyl-prolyl cis-trans isomerase [Xanthomonas oryzae pv. oryzae KACC10331]
>gi|58582991|ref|YP_202007.1| hypothetical protein XOO3368 [Xanthomonas oryzae pv. oryzae KACC10331]
>gi|58583011|ref|YP_202027.1| tRNA/rRNA methyltransferase [Xanthomonas oryzae pv. oryzae KACC10331]
>gi|58583031|ref|YP_202047.1| siroheme synthase [Xanthomonas oryzae pv. oryzae KACC10331]
>gi|58583033|ref|YP_202049.1| hypothetical protein XOO3410 [Xanthomonas oryzae pv. oryzae KACC10331]
>gi|58583039|ref|YP_202055.1| hypothetical protein XOO3416 [Xanthomonas oryzae pv. oryzae KACC10331]
>gi|58583040|ref|YP_202056.1| hypothetical protein XOO3417 [Xanthomonas oryzae pv. oryzae KACC10331]
>gi|58583051|ref|YP_202067.1| hypothetical protein XOO3428 [Xanthomonas oryzae pv. oryzae KACC10331]
>gi|58583064|ref|YP_202080.1| hypothetical protein XOO3441 [Xanthomonas oryzae pv. oryzae KACC10331]
>gi|58583070|ref|YP_202086.1| hypothetical protein XOO3447 [Xanthomonas oryzae pv. oryzae KACC10331]
>gi|58583094|ref|YP_202110.1| hypothetical protein XOO3471 [Xanthomonas oryzae pv. oryzae KACC10331]
>gi|58583096|ref|YP_202112.1| Uncharacterized protein conserved in bacteria [Xanthomonas oryzae pv. oryzae KACC10331]
>gi|58583119|ref|YP_202135.1| Uncharacterized protein conserved in bacteria [Xanthomonas oryzae pv. oryzae KACC10331]
>gi|58583120|ref|YP_202136.1| hypothetical protein XOO3497 [Xanthomonas oryzae pv. oryzae KACC10331]
>gi|58583125|ref|YP_202141.1| putative vgr-related protein [Xanthomonas oryzae pv. oryzae KACC10331]
>gi|58583135|ref|YP_202151.1| putative vgr-related protein [Xanthomonas oryzae pv. oryzae KACC10331]
>gi|58583136|ref|YP_202152.1| membrane protein [Xanthomonas oryzae pv. oryzae KACC10331]
>gi|58583141|ref|YP_202157.1| hypothetical protein XOO3518 [Xanthomonas oryzae pv. oryzae KACC10331]
>gi|58583144|ref|YP_202160.1| hypothetical protein XOO3521 [Xanthomonas oryzae pv. oryzae KACC10331]
>gi|58583148|ref|YP_202164.1| hypothetical protein XOO3525 [Xanthomonas oryzae pv. oryzae KACC10331]
>gi|58583165|ref|YP_202181.1| MFS transporter [Xanthomonas oryzae pv. oryzae KACC10331]
>gi|58583167|ref|YP_202183.1| putative membrane fusion protein RaxA [Xanthomonas oryzae pv. oryzae KACC10331]
>gi|58583169|ref|YP_202185.1| putative sulfotransferase RaxST [Xanthomonas oryzae pv. oryzae KACC10331]
>gi|58583171|ref|YP_202187.1| hypothetical protein XOO3548 [Xanthomonas oryzae pv. oryzae KACC10331]
>gi|58583184|ref|YP_202200.1| 30S ribosomal protein S13 [Xanthomonas oryzae pv. oryzae KACC10331]
>gi|58583186|ref|YP_202202.1| 50S ribosomal protein L15 [Xanthomonas oryzae pv. oryzae KACC10331]
>gi|58583204|ref|YP_202220.1| 50S ribosomal protein L23 [Xanthomonas oryzae pv. oryzae KACC10331]
>gi|58583215|ref|YP_202231.1| 50S ribosomal protein L7/L12 [Xanthomonas oryzae pv. oryzae KACC10331]
>gi|58583229|ref|YP_202245.1| hypothetical protein XOO3606 [Xanthomonas oryzae pv. oryzae KACC10331]
>gi|58583237|ref|YP_202253.1| thioredoxin [Xanthomonas oryzae pv. oryzae KACC10331]
>gi|58583239|ref|YP_202255.1| tryptophan repressor binding protein [Xanthomonas oryzae pv. oryzae KACC10331]
>gi|58583244|ref|YP_202260.1| extracellular protease [Xanthomonas oryzae pv. oryzae KACC10331]
>gi|58583249|ref|YP_202265.1| hypothetical protein XOO3626 [Xanthomonas oryzae pv. oryzae KACC10331]
>gi|58583264|ref|YP_202280.1| hypothetical protein XOO3641 [Xanthomonas oryzae pv. oryzae KACC10331]
>gi|58583282|ref|YP_202298.1| two-component system regulatory protein [Xanthomonas oryzae pv. oryzae KACC10331]
>gi|58583300|ref|YP_202316.1| colicin V production protein [Xanthomonas oryzae pv. oryzae KACC10331]
>gi|58583301|ref|YP_202317.1| hypothetical protein XOO3678 [Xanthomonas oryzae pv. oryzae KACC10331]
>gi|58583302|ref|YP_202318.1| folylpolyglutamate synthase; dihydrofolate synthase [Xanthomonas oryzae pv. oryzae KACC10331]
>gi|58583309|ref|YP_202325.1| sulfate ABC transporter ATP-binding protein [Xanthomonas oryzae pv. oryzae KACC10331]
>gi|58583310|ref|YP_202326.1| phenol hydroxylase [Xanthomonas oryzae pv. oryzae KACC10331]
>gi|58583317|ref|YP_202333.1| hypothetical protein XOO3694 [Xanthomonas oryzae pv. oryzae KACC10331]
>gi|58583322|ref|YP_202338.1| hypothetical protein XOO3699 [Xanthomonas oryzae pv. oryzae KACC10331]
>gi|58583323|ref|YP_202339.1| IS1404 transposase [Xanthomonas oryzae pv. oryzae KACC10331]
>gi|58583342|ref|YP_202358.1| integral membrane protein [Xanthomonas oryzae pv. oryzae KACC10331]
>gi|58583378|ref|YP_202394.1| glycine rich protein [Xanthomonas oryzae pv. oryzae KACC10331]
>gi|58583389|ref|YP_202405.1| oxidoreductase [Xanthomonas oryzae pv. oryzae KACC10331]
>gi|58583391|ref|YP_202407.1| ABC transporter substrate binding protein [Xanthomonas oryzae pv. oryzae KACC10331]
>gi|58583393|ref|YP_202409.1| ABC transporter ATP-binding component [Xanthomonas oryzae pv. oryzae KACC10331]
>gi|58583395|ref|YP_202411.1| permease [Xanthomonas oryzae pv. oryzae KACC10331]
>gi|58583398|ref|YP_202414.1| hypothetical protein XOO3775 [Xanthomonas oryzae pv. oryzae KACC10331]
>gi|58583411|ref|YP_202427.1| methyltransferase [Xanthomonas oryzae pv. oryzae KACC10331]
>gi|58583413|ref|YP_202429.1| hypothetical protein XOO3790 [Xanthomonas oryzae pv. oryzae KACC10331]
>gi|58583415|ref|YP_202431.1| phosphoanhydride phosphohydrolase [Xanthomonas oryzae pv. oryzae KACC10331]
>gi|58583450|ref|YP_202466.1| cell division protein [Xanthomonas oryzae pv. oryzae KACC10331]
>gi|58583452|ref|YP_202468.1| UDP-N-acetylmuramoylalanyl-D-glutamyl-2,6-diaminopimelate-D-alanyl-D-alanyl ligase [Xanthomonas oryzae pv. oryzae KACC10331]
>gi|58583461|ref|YP_202477.1| hypothetical protein XOO3838 [Xanthomonas oryzae pv. oryzae KACC10331]
>gi|58583463|ref|YP_202479.1| hypothetical protein XOO3840 [Xanthomonas oryzae pv. oryzae KACC10331]
>gi|58583467|ref|YP_202483.1| potassium-transporting ATPase C chain [Xanthomonas oryzae pv. oryzae KACC10331]

>gi|58583468|ref|YP_202484.1| potassium-transporting ATPase B chain [Xanthomonas oryzae pv. oryzae KACC10331]
>gi|58583471|ref|YP_202487.1| hypothetical protein XOO3848 [Xanthomonas oryzae pv. oryzae KACC10331]
>gi|58583485|ref|YP_202501.1| hypothetical protein XOO3862 [Xanthomonas oryzae pv. oryzae KACC10331]
>gi|58583495|ref|YP_202511.1| hypothetical protein XOO3872 [Xanthomonas oryzae pv. oryzae KACC10331]
>gi|58583501|ref|YP_202517.1| nodulin 21-related protein [Xanthomonas oryzae pv. oryzae KACC10331]
>gi|58583507|ref|YP_202523.1| thiol:disulfide interchange protein [Xanthomonas oryzae pv. oryzae KACC10331]
>gi|58583519|ref|YP_202535.1| ISRSO17-transposase protein [Xanthomonas oryzae pv. oryzae KACC10331]
>gi|58583524|ref|YP_202540.1| IS1595 transposase [Xanthomonas oryzae pv. oryzae KACC10331]
>gi|58583526|ref|YP_202542.1| hypothetical protein XOO3903 [Xanthomonas oryzae pv. oryzae KACC10331]
>gi|58583540|ref|YP_202556.1| glucosamine-fructose-6-phosphate aminotransferase [Xanthomonas oryzae pv. oryzae KACC10331]
>gi|58583560|ref|YP_202576.1| hypothetical protein XOO3937 [Xanthomonas oryzae pv. oryzae KACC10331]
>gi|58583564|ref|YP_202580.1| hypothetical protein XOO3941 [Xanthomonas oryzae pv. oryzae KACC10331]
>gi|58583565|ref|YP_202581.1| hypothetical protein XOO3942 [Xanthomonas oryzae pv. oryzae KACC10331]
>gi|58583580|ref|YP_202596.1| rare lipoprotein A [Xanthomonas oryzae pv. oryzae KACC10331]
>gi|58583592|ref|YP_202608.1| rod shape-determining protein [Xanthomonas oryzae pv. oryzae KACC10331]
>gi|58583606|ref|YP_202622.1| multidrug resistance efflux pump [Xanthomonas oryzae pv. oryzae KACC10331]
>gi|58583607|ref|YP_202623.1| hypothetical protein XOO3984 [Xanthomonas oryzae pv. oryzae KACC10331]
>gi|58583608|ref|YP_202624.1| MFS transporter [Xanthomonas oryzae pv. oryzae KACC10331]
>gi|58583613|ref|YP_202629.1| hypothetical protein XOO3990 [Xanthomonas oryzae pv. oryzae KACC10331]
>gi|58583614|ref|YP_202630.1| hypothetical protein XOO3991 [Xanthomonas oryzae pv. oryzae KACC10331]
>gi|58583626|ref|YP_202642.1| hypothetical protein XOO4003 [Xanthomonas oryzae pv. oryzae KACC10331]
>gi|58583632|ref|YP_202648.1| two-component system sensor protein [Xanthomonas oryzae pv. oryzae KACC10331]
>gi|58583640|ref|YP_202656.1| GGDEF family protein [Xanthomonas oryzae pv. oryzae KACC10331]
>gi|58583642|ref|YP_202658.1| cellulase [Xanthomonas oryzae pv. oryzae KACC10331]
>gi|58583643|ref|YP_202659.1| chemotaxis protein [Xanthomonas oryzae pv. oryzae KACC10331]
>gi|58583647|ref|YP_202663.1| hypothetical protein XOO4024 [Xanthomonas oryzae pv. oryzae KACC10331]
>gi|58583664|ref|YP_202680.1| Permeases of the major facilitator superfamily [Xanthomonas oryzae pv. oryzae KACC10331]
>gi|58583676|ref|YP_202692.1| hypothetical protein XOO4053 [Xanthomonas oryzae pv. oryzae KACC10331]
>gi|58583684|ref|YP_202700.1| metalloproteinase [Xanthomonas oryzae pv. oryzae KACC10331]
>gi|58583686|ref|YP_202702.1| hypothetical protein XOO4063 [Xanthomonas oryzae pv. oryzae KACC10331]
>gi|58583696|ref|YP_202712.1| hypothetical protein XOO4073 [Xanthomonas oryzae pv. oryzae KACC10331]
>gi|58583699|ref|YP_202715.1| nuclease [Xanthomonas oryzae pv. oryzae KACC10331]
>gi|58583710|ref|YP_202726.1| hypothetical protein XOO4087 [Xanthomonas oryzae pv. oryzae KACC10331]
>gi|58583711|ref|YP_202727.1| uroporphyrinogen-III synthase [Xanthomonas oryzae pv. oryzae KACC10331]
>gi|58583716|ref|YP_202732.1| hypothetical protein XOO4093 [Xanthomonas oryzae pv. oryzae KACC10331]
>gi|58583718|ref|YP_202734.1| hypothetical protein XOO4095 [Xanthomonas oryzae pv. oryzae KACC10331]
>gi|58583719|ref|YP_202735.1| hypothetical protein XOO4096 [Xanthomonas oryzae pv. oryzae KACC10331]
>gi|58583735|ref|YP_202751.1| hypothetical protein XOO4112 [Xanthomonas oryzae pv. oryzae KACC10331]
>gi|58583756|ref|YP_202772.1| hypothetical protein XOO4133 [Xanthomonas oryzae pv. oryzae KACC10331]
>gi|58583778|ref|YP_202794.1| SugE [Xanthomonas oryzae pv. oryzae KACC10331]
>gi|58583779|ref|YP_202795.1| Hypothetical protein in CLP 5'region [Xanthomonas oryzae pv. oryzae KACC10331]
>gi|58583817|ref|YP_202833.1| hypothetical protein XOO4194 [Xanthomonas oryzae pv. oryzae KACC10331]
>gi|58583826|ref|YP_202842.1| hypothetical protein XOO4203 [Xanthomonas oryzae pv. oryzae KACC10331]
>gi|58583827|ref|YP_202843.1| hypothetical protein XOO4204 [Xanthomonas oryzae pv. oryzae KACC10331]
>gi|58583828|ref|YP_202844.1| MFS transporter [Xanthomonas oryzae pv. oryzae KACC10331]
>gi|58583830|ref|YP_202846.1| hypothetical protein XOO4207 [Xanthomonas oryzae pv. oryzae KACC10331]
>gi|58583842|ref|YP_202858.1| transcriptional regulator MarR family [Xanthomonas oryzae pv. oryzae KACC10331]
>gi|58583844|ref|YP_202860.1| MFS transporter [Xanthomonas oryzae pv. oryzae KACC10331]
>gi|58583866|ref|YP_202882.1| hypothetical protein XOO4243 [Xanthomonas oryzae pv. oryzae KACC10331]
>gi|58583878|ref|YP_202894.1| avirulence/virulence protein [Xanthomonas oryzae pv. oryzae KACC10331]
>gi|58583879|ref|YP_202895.1| avirulence protein [Xanthomonas oryzae pv. oryzae KACC10331]
>gi|58583887|ref|YP_202903.1| anti-sigma F factor antagonist [Xanthomonas oryzae pv. oryzae KACC10331]
>gi|58583889|ref|YP_202905.1| hypothetical protein XOO4266 [Xanthomonas oryzae pv. oryzae KACC10331]
>gi|58583902|ref|YP_202918.1| hypothetical protein XOO4279 [Xanthomonas oryzae pv. oryzae KACC10331]
>gi|58583916|ref|YP_202932.1| hypothetical protein XOO4293 [Xanthomonas oryzae pv. oryzae KACC10331]
>gi|58583922|ref|YP_202938.1| cell division protein [Xanthomonas oryzae pv. oryzae KACC10331]
>gi|58583924|ref|YP_202940.1| ATP-dependent RNA helicase [Xanthomonas oryzae pv. oryzae KACC10331]
>gi|58583926|ref|YP_202942.1| transcription termination factor Rho [Xanthomonas oryzae pv. oryzae KACC10331]
>gi|58583936|ref|YP_202952.1| putative inner membrane protein [Xanthomonas oryzae pv. oryzae KACC10331]
>gi|58583940|ref|YP_202956.1| putative dehydratase [Xanthomonas oryzae pv. oryzae KACC10331]
>gi|58583942|ref|YP_202958.1| ketosynthase [Xanthomonas oryzae pv. oryzae KACC10331]
>gi|58583949|ref|YP_202965.1| hypothetical protein XOO4326 [Xanthomonas oryzae pv. oryzae KACC10331]
>gi|58583952|ref|YP_202968.1| oxidoreductase [Xanthomonas oryzae pv. oryzae KACC10331]
>gi|58583953|ref|YP_202969.1| hypothetical protein XOO4330 [Xanthomonas oryzae pv. oryzae KACC10331]
>gi|58583955|ref|YP_202971.1| 2-keto-3-deoxy-D-gluconate transport system [Xanthomonas oryzae pv. oryzae KACC10331]
>gi|58583972|ref|YP_202988.1| MFS transporter [Xanthomonas oryzae pv. oryzae KACC10331]
>gi|58584005|ref|YP_203021.1| hypothetical protein XOO4382 [Xanthomonas oryzae pv. oryzae KACC10331]
>gi|58584010|ref|YP_203026.1| cytochrome C5 [Xanthomonas oryzae pv. oryzae KACC10331]
>gi|58584020|ref|YP_203036.1| hypothetical protein XOO4397 [Xanthomonas oryzae pv. oryzae KACC10331]
>gi|58584028|ref|YP_203044.1| hypothetical protein XOO4405 [Xanthomonas oryzae pv. oryzae KACC10331]

>gi|58584030|ref|YP_203046.1| sec-independent protein translocase [Xanthomonas oryzae pv. oryzae KACC10331]
>gi|58584032|ref|YP_203048.1| hypothetical protein XOO4409 [Xanthomonas oryzae pv. oryzae KACC10331]
>gi|58584092|ref|YP_203108.1| exodeoxyribonuclease V alpha chain [Xanthomonas oryzae pv. oryzae KACC10331]
>gi|58584097|ref|YP_203113.1| hemagglutinin [Xanthomonas oryzae pv. oryzae KACC10331]
>gi|58584098|ref|YP_203114.1| hemagglutinin [Xanthomonas oryzae pv. oryzae KACC10331]
>gi|58584099|ref|YP_203115.1| microcystin dependent protein [Xanthomonas oryzae pv. oryzae KACC10331]
>gi|58584104|ref|YP_203120.1| superoxide dismutase [Xanthomonas oryzae pv. oryzae KACC10331]
>gi|58584105|ref|YP_203121.1| superoxide dismutase like protein [Xanthomonas oryzae pv. oryzae KACC10331]
>gi|58584107|ref|YP_203123.1| two-component system sensor protein [Xanthomonas oryzae pv. oryzae KACC10331]
>gi|58584108|ref|YP_203124.1| PilL [Xanthomonas oryzae pv. oryzae KACC10331]
>gi|58584109|ref|YP_203125.1| ammonium transporter [Xanthomonas oryzae pv. oryzae KACC10331]
>gi|58584133|ref|YP_203149.1| hypothetical protein XOO4510 [Xanthomonas oryzae pv. oryzae KACC10331]
>gi|58584139|ref|YP_203155.1| Transcriptional regulator [Xanthomonas oryzae pv. oryzae KACC10331]
>gi|58584149|ref|YP_203165.1| hypothetical protein XOO4526 [Xanthomonas oryzae pv. oryzae KACC10331]
>gi|58584155|ref|YP_203171.1| hypothetical protein XOO4532 [Xanthomonas oryzae pv. oryzae KACC10331]
>gi|58584160|ref|YP_203176.1| oxidoreductase [Xanthomonas oryzae pv. oryzae KACC10331]
>gi|58584161|ref|YP_203177.1| hypothetical protein XOO4538 [Xanthomonas oryzae pv. oryzae KACC10331]
>gi|58584165|ref|YP_203181.1| hypothetical protein XOO4542 [Xanthomonas oryzae pv. oryzae KACC10331]
>gi|58584175|ref|YP_203191.1| transglycosylase associated protein [Xanthomonas oryzae pv. oryzae KACC10331]
>gi|58584180|ref|YP_203196.1| hypothetical protein XOO4557 [Xanthomonas oryzae pv. oryzae KACC10331]
>gi|58584187|ref|YP_203203.1| capsid protein VPI [Xanthomonas oryzae pv. oryzae KACC10331]
>gi|58584206|ref|YP_203222.1| hypothetical protein XOO4583 [Xanthomonas oryzae pv. oryzae KACC10331]
>gi|58584208|ref|YP_203224.1| D-3-phosphoglycerate dehydrogenase [Xanthomonas oryzae pv. oryzae KACC10331]
>gi|58584209|ref|YP_203225.1| carboxyl-terminal protease [Xanthomonas oryzae pv. oryzae KACC10331]
>gi|58584210|ref|YP_203226.1| hypothetical protein XOO4587 [Xanthomonas oryzae pv. oryzae KACC10331]
>gi|58584211|ref|YP_203227.1| hypothetical protein XOO4588 [Xanthomonas oryzae pv. oryzae KACC10331]
>gi|58584230|ref|YP_203246.1| cardiolipin synthetase [Xanthomonas oryzae pv. oryzae KACC10331]
>gi|58584233|ref|YP_203249.1| hypothetical protein XOO4610 [Xanthomonas oryzae pv. oryzae KACC10331]
>gi|58584241|ref|YP_203257.1| sugar diacide regulator [Xanthomonas oryzae pv. oryzae KACC10331]
>gi|58584243|ref|YP_203259.1| glycerate kinase [Xanthomonas oryzae pv. oryzae KACC10331]
>gi|58584246|ref|YP_203262.1| dipeptidase [Xanthomonas oryzae pv. oryzae KACC10331]
>gi|58584250|ref|YP_203266.1| hypothetical protein XOO4627 [Xanthomonas oryzae pv. oryzae KACC10331]
>gi|58584257|ref|YP_203273.1| thiophene and furan oxidation protein [Xanthomonas oryzae pv. oryzae KACC10331]
>gi|16262461|ref|NP_435254.1| hypothetical protein SMa0017 [Sinorhizobium meliloti 1021]
>gi|16262516|ref|NP_435309.1| hypothetical protein SMa0123 [Sinorhizobium meliloti 1021]
>gi|16262520|ref|NP_435313.1| hypothetical protein SMa0128 [Sinorhizobium meliloti 1021]
>gi|16262523|ref|NP_435316.1| hypothetical protein SMa0134 [Sinorhizobium meliloti 1021]
>gi|16262524|ref|NP_435317.1| hypothetical protein SMa0136 [Sinorhizobium meliloti 1021]
>gi|16262527|ref|NP_435320.1| possible protease [Sinorhizobium meliloti 1021]
>gi|16262530|ref|NP_435323.1| hypothetical protein SMa0146 [Sinorhizobium meliloti 1021]
>gi|16262573|ref|NP_435366.1| putative transmembrane-transport protein [Sinorhizobium meliloti 1021]
>gi|16262586|ref|NP_435379.1| conserved hypothetical dedA-like protein [Sinorhizobium meliloti 1021]
>gi|16262604|ref|NP_435397.1| putative regulator, MerR family [Sinorhizobium meliloti 1021]
>gi|16262614|ref|NP_435407.1| ABC transporter, permease [Sinorhizobium meliloti 1021]
>gi|16262638|ref|NP_435431.1| hypothetical protein SMa0343 [Sinorhizobium meliloti 1021]
>gi|16262649|ref|NP_435442.1| hypothetical protein SMa0364 [Sinorhizobium meliloti 1021]
>gi|16262650|ref|NP_435443.1| hypothetical protein SMa0367 [Sinorhizobium meliloti 1021]
>gi|16262687|ref|NP_435480.1| hypothetical protein SMa0447 [Sinorhizobium meliloti 1021]
>gi|16262688|ref|NP_435481.1| hypothetical protein SMa0448 [Sinorhizobium meliloti 1021]
>gi|16262695|ref|NP_435488.1| putative adenylate cyclase [Sinorhizobium meliloti 1021]
>gi|16262700|ref|NP_435493.1| hypothetical protein SMa0471 [Sinorhizobium meliloti 1021]
>gi|16262718|ref|NP_435511.1| putative ABC transporter permease [Sinorhizobium meliloti 1021]
>gi|16262723|ref|NP_435516.1| IdnO1 gluconate 5-dehydrogenase [Sinorhizobium meliloti 1021]
>gi|16262730|ref|NP_435523.1| putative ABC-type iron transport system protein [Sinorhizobium meliloti 1021]
>gi|16262736|ref|NP_435529.1| hypothetical protein SMa0541 [Sinorhizobium meliloti 1021]
>gi|16262737|ref|NP_435530.1| hypothetical protein SMa0543 [Sinorhizobium meliloti 1021]
>gi|16262769|ref|NP_435562.1| hypothetical protein SMa0606 [Sinorhizobium meliloti 1021]
>gi|16262783|ref|NP_435576.1| hypothetical protein SMa0629 [Sinorhizobium meliloti 1021]
>gi|16262788|ref|NP_435581.1| hypothetical protein SMa0637 [Sinorhizobium meliloti 1021]
>gi|16262796|ref|NP_435589.1| hypothetical protein SMa0653 [Sinorhizobium meliloti 1021]
>gi|16262804|ref|NP_435597.1| hypothetical protein SMa0665 [Sinorhizobium meliloti 1021]
>gi|16262819|ref|NP_435612.1| hypothetical protein SMa0690 [Sinorhizobium meliloti 1021]
>gi|16262867|ref|NP_435660.1| hypothetical protein with local similarity [Sinorhizobium meliloti 1021]
>gi|16262907|ref|NP_435700.1| hypothetical protein SMa0833 [Sinorhizobium meliloti 1021]
>gi|16262910|ref|NP_435703.1| SyrA protein involved in EPS production [Sinorhizobium meliloti 1021]
>gi|16262964|ref|NP_435757.1| hypothetical protein SMa0945 [Sinorhizobium meliloti 1021]
>gi|16263009|ref|NP_435802.1| hypothetical protein SMa1028 [Sinorhizobium meliloti 1021]
>gi|16263012|ref|NP_435805.1| Partial conserved hypothetical hypothetical protein inactivated by IS Rm1 [Sinorhizobium meliloti 1021]
>gi|16263015|ref|NP_435808.1| hypothetical protein SMa1037 [Sinorhizobium meliloti 1021]
>gi|16263023|ref|NP_435816.1| hypothetical protein SMa1053 [Sinorhizobium meliloti 1021]

>gi|16263036|ref|NP_435829.1| hypothetical protein SMa1078 [Sinorhizobium meliloti 1021]
>gi|16263039|ref|NP_435832.1| hypothetical protein SMa1082 [Sinorhizobium meliloti 1021]
>gi|16263051|ref|NP_435844.1| hypothetical protein SMa1100 [Sinorhizobium meliloti 1021]
>gi|16263081|ref|NP_435874.1| hypothetical protein SMa1159 [Sinorhizobium meliloti 1021]
>gi|16263082|ref|NP_435875.1| hypothetical protein membrane domain [Sinorhizobium meliloti 1021]
>gi|16263083|ref|NP_435876.1| hypothetical protein SMa1161 [Sinorhizobium meliloti 1021]
>gi|16263091|ref|NP_435884.1| hypothetical protein SMa1172 [Sinorhizobium meliloti 1021]
>gi|16263099|ref|NP_435892.1| NosY nitrous oxide metabolic protein [Sinorhizobium meliloti 1021]
>gi|16263107|ref|NP_435900.1| possible Copper export protein [Sinorhizobium meliloti 1021]
>gi|16263111|ref|NP_435904.1| FixS1 nitrogen fixation protein [Sinorhizobium meliloti 1021]
>gi|16263140|ref|NP_435933.1| hypothetical protein SMa1259 [Sinorhizobium meliloti 1021]
>gi|16263149|ref|NP_435942.1| NorE protein involved in nitric oxide reduction [Sinorhizobium meliloti 1021]
>gi|16263153|ref|NP_435946.1| hypothetical protein SMa1289 [Sinorhizobium meliloti 1021]
>gi|16263161|ref|NP_435954.1| putative transmembrane transport protein [Sinorhizobium meliloti 1021]
>gi|16263167|ref|NP_435960.1| VirB6 type IV secretion protein [Sinorhizobium meliloti 1021]
>gi|16263197|ref|NP_435990.1| hypothetical protein SMa1361 [Sinorhizobium meliloti 1021]
>gi|16263203|ref|NP_435996.1| hypothetical protein SMa1368 [Sinorhizobium meliloti 1021]
>gi|16263209|ref|NP_436002.1| Putative amidase [Sinorhizobium meliloti 1021]
>gi|16263218|ref|NP_436011.1| hypothetical protein SMa1398 [Sinorhizobium meliloti 1021]
>gi|16263229|ref|NP_436022.1| Putative ABC transporter permease protein [Sinorhizobium meliloti 1021]
>gi|16263231|ref|NP_436024.1| Putative ABC transporter permease protein [Sinorhizobium meliloti 1021]
>gi|16263261|ref|NP_436054.1| hypothetical protein SMa1485 [Sinorhizobium meliloti 1021]
>gi|16263275|ref|NP_436068.1| Putative ABC transporter permease [Sinorhizobium meliloti 1021]
>gi|16263276|ref|NP_436069.1| Putative ABC transporter permease [Sinorhizobium meliloti 1021]
>gi|16263294|ref|NP_436087.1| putative oxidoreductase [Sinorhizobium meliloti 1021]
>gi|16263300|ref|NP_436093.1| putative methyl-accepting chemotaxis protein [Sinorhizobium meliloti 1021]
>gi|16263306|ref|NP_436099.1| probable PilA2 pilus assembly protein [Sinorhizobium meliloti 1021]
>gi|16263382|ref|NP_436175.1| hypothetical protein SMa1693 [Sinorhizobium meliloti 1021]
>gi|16263428|ref|NP_436221.1| hypothetical protein SMa1766 [Sinorhizobium meliloti 1021]
>gi|16263432|ref|NP_436225.1| hypothetical protein SMa1773 [Sinorhizobium meliloti 1021]
>gi|16263448|ref|NP_436241.1| hypothetical protein SMa1806 [Sinorhizobium meliloti 1021]
>gi|16263449|ref|NP_436242.1| hypothetical protein SMa1808 [Sinorhizobium meliloti 1021]
>gi|16263452|ref|NP_436245.1| Putative Dioxygenase [Sinorhizobium meliloti 1021]
>gi|16263455|ref|NP_436248.1| hypothetical protein SMa1820 [Sinorhizobium meliloti 1021]
>gi|16263490|ref|NP_436283.1| Putative membrane efflux protein [Sinorhizobium meliloti 1021]
>gi|16263505|ref|NP_436298.1| hypothetical protein SMa1916 [Sinorhizobium meliloti 1021]
>gi|16263513|ref|NP_436306.1| Putative transcriptional activator [Sinorhizobium meliloti 1021]
>gi|16263515|ref|NP_436308.1| putative transmembrane transport protein [Sinorhizobium meliloti 1021]
>gi|16263524|ref|NP_436317.1| Putative LysR-family transcriptional regulator [Sinorhizobium meliloti 1021]
>gi|16263542|ref|NP_436335.1| Putative LysR-family transcriptional regulator [Sinorhizobium meliloti 1021]
>gi|16263553|ref|NP_436346.1| hypothetical protein SMa2009 [Sinorhizobium meliloti 1021]
>gi|16263584|ref|NP_436377.1| hypothetical protein SMa2071 [Sinorhizobium meliloti 1021]
>gi|16263596|ref|NP_436389.1| hypothetical protein SMa2095 [Sinorhizobium meliloti 1021]
>gi|16263604|ref|NP_436397.1| hypothetical protein SMa2111 [Sinorhizobium meliloti 1021]
>gi|16263611|ref|NP_436404.1| probable ABC transporter, permease protein [Sinorhizobium meliloti 1021]
>gi|16263670|ref|NP_436463.1| hypothetical protein SMa2259 [Sinorhizobium meliloti 1021]
>gi|16263677|ref|NP_436470.1| hypothetical protein SMa2273 [Sinorhizobium meliloti 1021]
>gi|16263690|ref|NP_436483.1| hypothetical protein SMa2297 [Sinorhizobium meliloti 1021]
>gi|16263691|ref|NP_436484.1| hypothetical protein SMa2299 [Sinorhizobium meliloti 1021]
>gi|16263733|ref|NP_436526.1| putative ABC transporter, permease [Sinorhizobium meliloti 1021]
>gi|15963755|ref|NP_384108.1| hypothetical protein SMc02792 [Sinorhizobium meliloti 1021]
>gi|15963760|ref|NP_384113.1| HYPOTHETICAL TRANSMEMBRANE PROTEIN [Sinorhizobium meliloti 1021]
>gi|15963778|ref|NP_384131.1| CONSERVED HYPOTHETICAL TRANSMEMBRANE PROTEIN [Sinorhizobium meliloti 1021]
>gi|15963807|ref|NP_384160.1| HYPOTHETICAL TRANSMEMBRANE PROTEIN [Sinorhizobium meliloti 1021]
>gi|15963822|ref|NP_384175.1| PUTATIVE PERMEASE ABC TRANSPORTER PROTEIN [Sinorhizobium meliloti 1021]
>gi|15963827|ref|NP_384180.1| HYPOTHETICAL TRANSMEMBRANE PROTEIN [Sinorhizobium meliloti 1021]
>gi|15963833|ref|NP_384186.1| HYPOTHETICAL TRANSMEMBRANE PROTEIN [Sinorhizobium meliloti 1021]
>gi|15963848|ref|NP_384201.1| CONSERVED HYPOTHETICAL TRANSMEMBRANE PROTEIN [Sinorhizobium meliloti 1021]
>gi|15963880|ref|NP_384233.1| PUTATIVE TRANSPORT SYSTEM PERMEASE ABC TRANSPORTER PROTEIN [Sinorhizobium meliloti 1021]
>gi|15963910|ref|NP_384263.1| PUTATIVE MUREIN ENDOPEPTIDASE TRANSMEMBRANE PROTEIN [Sinorhizobium meliloti 1021]
>gi|15963922|ref|NP_384275.1| PUTATIVE TRANSCRIPTION REGULATOR PROTEIN [Sinorhizobium meliloti 1021]
>gi|15963952|ref|NP_384305.1| hypothetical protein SMc02874 [Sinorhizobium meliloti 1021]
>gi|15963953|ref|NP_384306.1| hypothetical protein SMc02875 [Sinorhizobium meliloti 1021]
>gi|15963960|ref|NP_384313.1| PUTATIVE TRANSMEMBRANE PROTEIN [Sinorhizobium meliloti 1021]
>gi|15963965|ref|NP_384318.1| hypothetical protein SMc02887 [Sinorhizobium meliloti 1021]
>gi|15963967|ref|NP_384320.1| PUTATIVE TRANSPORT PROTEIN [Sinorhizobium meliloti 1021]
>gi|15963970|ref|NP_384323.1| PUTATIVE TRANSPORT PROTEIN [Sinorhizobium meliloti 1021]
>gi|15963975|ref|NP_384328.1| PUTATIVE CYTOCHROME C TRANSMEMBRANE PROTEIN [Sinorhizobium meliloti 1021]
>gi|15963992|ref|NP_384345.1| PROBABLE TRANSLATION INITIATION FACTOR IF-2 PROTEIN [Sinorhizobium meliloti 1021]

>gi|15964005|ref|NP_384358.1| hypothetical protein SMc00332 [Sinorhizobium meliloti 1021]
>gi|15964009|ref|NP_384362.1| hypothetical protein SMc00336 [Sinorhizobium meliloti 1021]
>gi|15964011|ref|NP_384364.1| hypothetical protein SMc00338 [Sinorhizobium meliloti 1021]
>gi|15964021|ref|NP_384374.1| hypothetical protein SMc00348 [Sinorhizobium meliloti 1021]
>gi|15964025|ref|NP_384378.1| PUTATIVE ACETYLTRANSFERASE PROTEIN [Sinorhizobium meliloti 1021]
>gi|15964027|ref|NP_384380.1| HYPOTHETICAL TRANSMEMBRANE PROTEIN [Sinorhizobium meliloti 1021]
>gi|15964045|ref|NP_384398.1| PUTATIVE OXIDOREDUCTASE PROTEIN [Sinorhizobium meliloti 1021]
>gi|15964049|ref|NP_384402.1| HYPOTHETICAL TRANSMEMBRANE PROTEIN [Sinorhizobium meliloti 1021]
>gi|15964054|ref|NP_384407.1| HYPOTHETICAL TRANSMEMBRANE PROTEIN [Sinorhizobium meliloti 1021]
>gi|15964094|ref|NP_384447.1| PUTATIVE AMINO ACID EFFLUX PROTEIN [Sinorhizobium meliloti 1021]
>gi|15964100|ref|NP_384453.1| PUTATIVE TRANSMEMBRANE PROTEIN [Sinorhizobium meliloti 1021]
>gi|15964110|ref|NP_384463.1| hypothetical protein SMc01162 [Sinorhizobium meliloti 1021]
>gi|15964120|ref|NP_384473.1| 30S RIBOSOMAL PROTEIN S20 [Sinorhizobium meliloti 1021]
>gi|15964153|ref|NP_384506.1| hypothetical protein SMc01120 [Sinorhizobium meliloti 1021]
>gi|15964170|ref|NP_384523.1| PROBABLE RIBOKINASE PROTEIN [Sinorhizobium meliloti 1021]
>gi|15964176|ref|NP_384529.1| NTERM FRAGMENT OF A PUTATIVE TRANSPOSASE PROTEIN [Sinorhizobium meliloti 1021]
>gi|15964177|ref|NP_384530.1| HYPOTHETICAL/UNKNOWN TRANSMEMBRANE PROTEIN [Sinorhizobium meliloti 1021]
>gi|15964189|ref|NP_384542.1| HYPOTHETICAL TRANSMEMBRANE PROTEIN [Sinorhizobium meliloti 1021]
>gi|15964195|ref|NP_384548.1| HYPOTHETICAL TRANSMEMBRANE PROTEIN [Sinorhizobium meliloti 1021]
>gi|15964201|ref|NP_384554.1| HYPOTHETICAL TRANSMEMBRANE PROTEIN [Sinorhizobium meliloti 1021]
>gi|15964211|ref|NP_384564.1| hypothetical protein SMc01708 [Sinorhizobium meliloti 1021]
>gi|15964215|ref|NP_384568.1| HYPOTHETICAL TRANSMEMBRANE PROTEIN [Sinorhizobium meliloti 1021]
>gi|15964238|ref|NP_384591.1| PUTATIVE TRANSPORT TRANSMEMBRANE PROTEIN [Sinorhizobium meliloti 1021]
>gi|15964240|ref|NP_384593.1| HYPOTHETICAL TRANSMEMBRANE PROTEIN [Sinorhizobium meliloti 1021]
>gi|15964269|ref|NP_384622.1| hypothetical protein SMc02139 [Sinorhizobium meliloti 1021]
>gi|15964274|ref|NP_384627.1| HYPOTHETICAL TRANSMEMBRANE PROTEIN [Sinorhizobium meliloti 1021]
>gi|15964288|ref|NP_384641.1| HYPOTHETICAL TRANSMEMBRANE PROTEIN [Sinorhizobium meliloti 1021]
>gi|15964289|ref|NP_384642.1| hypothetical protein SMc02231 [Sinorhizobium meliloti 1021]
>gi|15964297|ref|NP_384650.1| HYPOTHETICAL TRANSMEMBRANE PROTEIN [Sinorhizobium meliloti 1021]
>gi|15964301|ref|NP_384654.1| PUTATIVE TRANSMEMBRANE PROTEIN [Sinorhizobium meliloti 1021]
>gi|15964321|ref|NP_384674.1| HYPOTHETICAL TRANSMEMBRANE PROTEIN [Sinorhizobium meliloti 1021]
>gi|15964375|ref|NP_384728.1| hypothetical protein SMc02316 [Sinorhizobium meliloti 1021]
>gi|15964379|ref|NP_384732.1| HYPOTHETICAL TRANSMEMBRANE PROTEIN [Sinorhizobium meliloti 1021]
>gi|15964386|ref|NP_384739.1| PUTATIVE PERMEASE ABC TRANSPORTER PROTEIN [Sinorhizobium meliloti 1021]
>gi|15964405|ref|NP_384758.1| FLAGELLAR MOTOR SWITCH PROTEIN [Sinorhizobium meliloti 1021]
>gi|15964426|ref|NP_384779.1| hypothetical protein SMc03041 [Sinorhizobium meliloti 1021]
>gi|15964428|ref|NP_384781.1| CHEMOTAXIS PRECURSOR (MOTILITY PROTEIN C) TRANSMEMBRANE [Sinorhizobium meliloti 1021]
>gi|15964429|ref|NP_384782.1| CHEMOTAXIS PROTEIN (MOTILITY PROTEIN D) [Sinorhizobium meliloti 1021]
>gi|15964456|ref|NP_384809.1| 6-PHOSPHOGLUCONOLACTONASE PROTEIN [Sinorhizobium meliloti 1021]
>gi|15964474|ref|NP_384827.1| PUTATIVE SENSOR HISTIDINE KINASE PROTEIN [Sinorhizobium meliloti 1021]
>gi|15964475|ref|NP_384828.1| hypothetical protein SMc00777 [Sinorhizobium meliloti 1021]
>gi|15964490|ref|NP_384843.1| HYPOTHETICAL TRANSMEMBRANE PROTEIN [Sinorhizobium meliloti 1021]
>gi|15964493|ref|NP_384846.1| hypothetical protein SMc00795 [Sinorhizobium meliloti 1021]
>gi|15964494|ref|NP_384847.1| HYPOTHETICAL TRANSMEMBRANE PROTEIN [Sinorhizobium meliloti 1021]
>gi|15964498|ref|NP_384851.1| HYPOTHETICAL TRANSMEMBRANE PROTEIN [Sinorhizobium meliloti 1021]
>gi|15964502|ref|NP_384855.1| hypothetical protein SMc00804 [Sinorhizobium meliloti 1021]
>gi|15964505|ref|NP_384858.1| PUTATIVE TRANSPORT TRANSMEMBRANE PROTEIN [Sinorhizobium meliloti 1021]
>gi|15964507|ref|NP_384860.1| PUTATIVE SIGNAL PEPTIDE PROTEIN [Sinorhizobium meliloti 1021]
>gi|15964525|ref|NP_384878.1| PUTATIVE TRANSPORT TRANSMEMBRANE PROTEIN [Sinorhizobium meliloti 1021]
>gi|15964554|ref|NP_384907.1| hypothetical protein SMc00904 [Sinorhizobium meliloti 1021]
>gi|15964558|ref|NP_384911.1| hypothetical protein SMc00899 [Sinorhizobium meliloti 1021]
>gi|15964574|ref|NP_384927.1| HYPOTHETICAL TRANSMEMBRANE SIGNAL PEPTIDE PROTEIN [Sinorhizobium meliloti 1021]
>gi|15964583|ref|NP_384936.1| PUTATIVE MRP PROTEIN HOMOLOG ATP-BINDING [Sinorhizobium meliloti 1021]
>gi|15964590|ref|NP_384943.1| PROBABLE ATP SYNTHASE SUBUNIT B' TRANSMEMBRANE PROTEIN [Sinorhizobium meliloti 1021]
>gi|15964591|ref|NP_384944.1| PROBABLE ATP SYNTHASE B CHAIN TRANSMEMBRANE PROTEIN [Sinorhizobium meliloti 1021]
>gi|15964601|ref|NP_384954.1| hypothetical protein SMc00858 [Sinorhizobium meliloti 1021]
>gi|15964626|ref|NP_384979.1| HYPOTHETICAL TRANSMEMBRANE PROTEIN [Sinorhizobium meliloti 1021]
>gi|15964635|ref|NP_384988.1| PUTATIVE EXODEOXYRIBONUCLEASE PROTEIN [Sinorhizobium meliloti 1021]
>gi|15964641|ref|NP_384994.1| HYPOTHETICAL TRANSMEMBRANE PROTEIN [Sinorhizobium meliloti 1021]
>gi|15964642|ref|NP_384995.1| HYPOTHETICAL TRANSMEMBRANE PROTEIN [Sinorhizobium meliloti 1021]
>gi|15964650|ref|NP_385003.1| PUTATIVE CHAPERONE PROTEIN [Sinorhizobium meliloti 1021]
>gi|15964683|ref|NP_385036.1| hypothetical protein SMc00023 [Sinorhizobium meliloti 1021]
>gi|15964684|ref|NP_385037.1| PUTATIVE CHROMOSOME PARTITION PROTEIN [Sinorhizobium meliloti 1021]
>gi|15964704|ref|NP_385057.1| HYPOTHETICAL TRANSMEMBRANE PROTEIN [Sinorhizobium meliloti 1021]
>gi|15964718|ref|NP_385071.1| HYPOTHETICAL TRANSMEMBRANE PROTEIN [Sinorhizobium meliloti 1021]
>gi|15964748|ref|NP_385101.1| PUTATIVE CATION EFFLUX SYSTEM PROTEIN [Sinorhizobium meliloti 1021]
>gi|15964750|ref|NP_385103.1| PUTATIVE CATION EFFLUX SYSTEM PROTEIN [Sinorhizobium meliloti 1021]
>gi|15964759|ref|NP_385112.1| hypothetical protein SMc00062 [Sinorhizobium meliloti 1021]
>gi|15964760|ref|NP_385113.1| HYPOTHETICAL TRANSMEMBRANE PROTEIN [Sinorhizobium meliloti 1021]
>gi|15964766|ref|NP_385119.1| CONSERVED HYPOTHETICAL SIGNAL PEPTIDE PROTEIN [Sinorhizobium meliloti 1021]

>gi|15964770|ref|NP_385123.1| CYTOCHROME C-TYPE BIOGENESIS TRANSMEMBRANE PROTEIN [Sinorhizobium meliloti 1021]
>gi|15964800|ref|NP_385153.1| CONSERVED HYPOTHETICAL TRANSMEMBRANE PROTEIN [Sinorhizobium meliloti 1021]
>gi|15964801|ref|NP_385154.1| hypothetical protein SMc02392 [Sinorhizobium meliloti 1021]
>gi|15964810|ref|NP_385163.1| HYPOTHETICAL TRANSMEMBRANE PROTEIN [Sinorhizobium meliloti 1021]
>gi|15964814|ref|NP_385167.1| hypothetical protein SMc02406 [Sinorhizobium meliloti 1021]
>gi|15964815|ref|NP_385168.1| hypothetical protein SMc02422 [Sinorhizobium meliloti 1021]
>gi|15964843|ref|NP_385196.1| HYPOTHETICAL TRANSMEMBRANE PROTEIN [Sinorhizobium meliloti 1021]
>gi|15964857|ref|NP_385210.1| hypothetical protein SMc02558 [Sinorhizobium meliloti 1021]
>gi|15964863|ref|NP_385216.1| HYPOTHETICAL UNKNOWN PROTEIN [Sinorhizobium meliloti 1021]
>gi|15964872|ref|NP_385225.1| HYPOTHETICAL TRANSMEMBRANE PROTEIN [Sinorhizobium meliloti 1021]
>gi|15964882|ref|NP_385235.1| HYPOTHETICAL TRANSMEMBRANE PROTEIN [Sinorhizobium meliloti 1021]
>gi|15964883|ref|NP_385236.1| HYPOTHETICAL TRANSMEMBRANE PROTEIN [Sinorhizobium meliloti 1021]
>gi|15964889|ref|NP_385242.1| HYPOTHETICAL TRANSMEMBRANE PROTEIN [Sinorhizobium meliloti 1021]
>gi|15964891|ref|NP_385244.1| PUTATIVE 30S RIBOSOMAL PROTEIN S6 [Sinorhizobium meliloti 1021]
>gi|15964920|ref|NP_385273.1| PUTATIVE BRANCHED-CHAIN AMINO ACID TRANSPORT PERMEASE PROTEIN [Sinorhizobium meliloti 1021]
>gi|15964925|ref|NP_385278.1| PUTATIVE OXIDOREDUCTASE PROTEIN [Sinorhizobium meliloti 1021]
>gi|15964933|ref|NP_385286.1| HYPOTHETICAL TRANSMEMBRANE PROTEIN [Sinorhizobium meliloti 1021]
>gi|15964953|ref|NP_385306.1| PUTATIVE TRANSCRIPTION REGULATOR PROTEIN [Sinorhizobium meliloti 1021]
>gi|15964968|ref|NP_385321.1| PROBABLE 6,7-DIMETHYL-8-RIBITYLLUMAZINE SYNTHASE PROTEIN [Sinorhizobium meliloti 1021]
>gi|15964979|ref|NP_385332.1| hypothetical protein SMc01788 [Sinorhizobium meliloti 1021]
>gi|15964980|ref|NP_385333.1| HYPOTHETICAL SIGNAL PEPTIDE PROTEIN [Sinorhizobium meliloti 1021]
>gi|15964981|ref|NP_385334.1| hypothetical protein SMc01902 [Sinorhizobium meliloti 1021]
>gi|15965015|ref|NP_385368.1| hypothetical protein SMc01910 [Sinorhizobium meliloti 1021]
>gi|15965021|ref|NP_385374.1| HYPOTHETICAL SIGNAL PEPTIDE PROTEIN [Sinorhizobium meliloti 1021]
>gi|15965024|ref|NP_385377.1| HYPOTHETICAL TRANSMEMBRANE PROTEIN [Sinorhizobium meliloti 1021]
>gi|15965050|ref|NP_385403.1| hypothetical protein SMc01367 [Sinorhizobium meliloti 1021]
>gi|15965055|ref|NP_385408.1| HYPOTHETICAL TRANSMEMBRANE PROTEIN [Sinorhizobium meliloti 1021]
>gi|15965064|ref|NP_385417.1| PUTATIVE GLUTAMYL-TRNA(GLN) AMIDOTRANSFERASE SUBUNIT C PROTEIN [Sinorhizobium meliloti 1021]
>gi|15965081|ref|NP_385434.1| PROBABLE RIBONUCLEASE E PROTEIN [Sinorhizobium meliloti 1021]
>gi|15965097|ref|NP_385450.1| PROBABLE 50S RIBOSOMAL PROTEIN L11 [Sinorhizobium meliloti 1021]
>gi|15965100|ref|NP_385453.1| PROBABLE 50S RIBOSOMAL PROTEIN L7/L12 (L8) [Sinorhizobium meliloti 1021]
>gi|15965127|ref|NP_385480.1| PROBABLE 50S RIBOSOMAL PROTEIN L30 [Sinorhizobium meliloti 1021]
>gi|15965139|ref|NP_385492.1| PROBABLE CHAPERONE HOMOLOGUE TRANSMEMBRANE PROTEIN [Sinorhizobium meliloti 1021]
>gi|15965159|ref|NP_385512.1| HYPOTHETICAL TRANSMEMBRANE PROTEIN [Sinorhizobium meliloti 1021]
>gi|15965177|ref|NP_385530.1| hypothetical protein SMc01009 [Sinorhizobium meliloti 1021]
>gi|15965189|ref|NP_385542.1| HYPOTHETICAL TRANSMEMBRANE PROTEIN [Sinorhizobium meliloti 1021]
>gi|15965192|ref|NP_385545.1| HYPOTHETICAL TRANSMEMBRANE PROTEIN [Sinorhizobium meliloti 1021]
>gi|15965200|ref|NP_385553.1| DIHYDROLIPOAMIDE S-ACETYLTRANSFERASE PROTEIN [Sinorhizobium meliloti 1021]
>gi|15965204|ref|NP_385557.1| HYPOTHETICAL TRANSMEMBRANE PROTEIN [Sinorhizobium meliloti 1021]
>gi|15965256|ref|NP_385609.1| PROBABLE UDP-3-O-3-HYDROXYMYRISTOYL GLUCOSAMINE N-ACYLTRANSFERASE PROTEIN [Sinorhizobium meliloti 1021]
>gi|15965268|ref|NP_385621.1| hypothetical protein SMc02081 [Sinorhizobium meliloti 1021]
>gi|15965277|ref|NP_385630.1| HYPOTHETICAL TRANSMEMBRANE PROTEIN [Sinorhizobium meliloti 1021]
>gi|15965283|ref|NP_385636.1| PUTATIVE SEC-INDEPENDENT TRANSLOCASE TRANSMEMBRANE PROTEIN [Sinorhizobium meliloti 1021]
>gi|15965304|ref|NP_385657.1| hypothetical protein SMc01241 [Sinorhizobium meliloti 1021]
>gi|15965311|ref|NP_385664.1| PROBABLE SINGLE-STRAND BINDING PROTEIN [Sinorhizobium meliloti 1021]
>gi|15965312|ref|NP_385665.1| HYPOTHETICAL TRANSMEMBRANE PROTEIN [Sinorhizobium meliloti 1021]
>gi|15965341|ref|NP_385694.1| PUTATIVE TRANSMEMBRANE OXIDOREDUCTASE PROTEIN [Sinorhizobium meliloti 1021]
>gi|15965346|ref|NP_385699.1| PUTATIVE TRANSPORT TRANSMEMBRANE PROTEIN [Sinorhizobium meliloti 1021]
>gi|15965363|ref|NP_385716.1| PUTATIVE LEXA REPRESSOR TRANSCRIPTION REGULATOR PROTEIN [Sinorhizobium meliloti 1021]
>gi|15965364|ref|NP_385717.1| hypothetical protein SMc01182 [Sinorhizobium meliloti 1021]
>gi|15965366|ref|NP_385719.1| CONSERVED HYPOTHETICAL TRANSMEMBRANE PROTEIN [Sinorhizobium meliloti 1021]
>gi|15965368|ref|NP_385721.1| PUTATIVE MULTIDRUG TRANSMEMBRANE RESISTANCE SIGNAL PEPTIDE PROTEIN [Sinorhizobium meliloti 1021]
>gi|15965369|ref|NP_385722.1| hypothetical protein SMc01177 [Sinorhizobium meliloti 1021]
>gi|15965387|ref|NP_385740.1| hypothetical protein SMc00942 [Sinorhizobium meliloti 1021]
>gi|15965391|ref|NP_385744.1| hypothetical protein SMc00946 [Sinorhizobium meliloti 1021]
>gi|15965417|ref|NP_385770.1| HYPOTHETICAL TRANSMEMBRANE PROTEIN [Sinorhizobium meliloti 1021]
>gi|15965421|ref|NP_385774.1| PUTATIVE TRANSPORT SYSTEM PERMEASE ABC TRANSPORTER PROTEIN [Sinorhizobium meliloti 1021]
>gi|15965428|ref|NP_385781.1| PUTATIVE CYTOCHROME C-TYPE BIOGENESIS PROTEIN [Sinorhizobium meliloti 1021]
>gi|15965433|ref|NP_385786.1| HYPOTHETICAL SIGNAL PEPTIDE PROTEIN [Sinorhizobium meliloti 1021]
>gi|15965434|ref|NP_385787.1| hypothetical protein SMc00254 [Sinorhizobium meliloti 1021]
>gi|15965439|ref|NP_385792.1| HYPOTHETICAL SIGNAL PEPTIDE PROTEIN [Sinorhizobium meliloti 1021]
>gi|15965445|ref|NP_385798.1| PUTATIVE OXIDOREDUCTASE PROTEIN [Sinorhizobium meliloti 1021]
>gi|15965468|ref|NP_385821.1| HYPOTHETICAL HEMOLYSIN-TYPE CALCIUM-BINDING PROTEIN [Sinorhizobium meliloti 1021]
>gi|15965473|ref|NP_385826.1| HYPOTHETICAL LIPOPROTEIN [Sinorhizobium meliloti 1021]

>gi|15965474|ref|NP_385827.1| PROBABLE SINGLE-STRANDED-DNA-SPECIFIC EXONUCLEASE PROTEIN [Sinorhizobium meliloti 1021]
>gi|15965507|ref|NP_385860.1| PUTATIVE AMINO ACID-BINDING PERIPLASMIC PROTEIN [Sinorhizobium meliloti 1021]
>gi|15965520|ref|NP_385873.1| HYPOTHETICAL TRANSMEMBRANE PROTEIN [Sinorhizobium meliloti 1021]
>gi|15965528|ref|NP_385881.1| CONSERVED HYPOTHETICAL TRANSMEMBRANE PROTEIN [Sinorhizobium meliloti 1021]
>gi|15965553|ref|NP_385906.1| PUTATIVE CARBOHYDRATE KINASE PROTEIN [Sinorhizobium meliloti 1021]
>gi|15965556|ref|NP_385909.1| HYPOTHETICAL TRANSMEMBRANE PROTEIN [Sinorhizobium meliloti 1021]
>gi|15965568|ref|NP_385921.1| hypothetical protein SMc00191 [Sinorhizobium meliloti 1021]
>gi|15965569|ref|NP_385922.1| HYPOTHETICAL TRANSMEMBRANE PROTEIN [Sinorhizobium meliloti 1021]
>gi|15965578|ref|NP_385931.1| PUTATIVE TRANSPORT PROTEIN TRANSMEMBRANE [Sinorhizobium meliloti 1021]
>gi|15965579|ref|NP_385932.1| hypothetical protein SMc00181 [Sinorhizobium meliloti 1021]
>gi|15965581|ref|NP_385934.1| HYPOTHETICAL/UNKNOWN PROTEIN [Sinorhizobium meliloti 1021]
>gi|15965595|ref|NP_385948.1| PUTATIVE OXIDOREDUCTASE PROTEIN [Sinorhizobium meliloti 1021]
>gi|15965621|ref|NP_385974.1| hypothetical protein SMc00141 [Sinorhizobium meliloti 1021]
>gi|15965644|ref|NP_385997.1| hypothetical protein SMc04221 [Sinorhizobium meliloti 1021]
>gi|15965645|ref|NP_385998.1| hypothetical protein SMc04222 [Sinorhizobium meliloti 1021]
>gi|15965655|ref|NP_386008.1| HYPOTHETICAL GLYCINE RICH TRANSMEMBRANE PROTEIN [Sinorhizobium meliloti 1021]
>gi|15965659|ref|NP_386012.1| PUTATIVE GLYCINE-RICH CELL WALL STRUCTURAL TRANSMEMBRANE PROTEIN [Sinorhizobium meliloti 1021]
>gi|15965668|ref|NP_386021.1| PROBABLE HIGH-AFFINITY ZINC UPTAKE SYSTEM ABC TRANSPORTER PROTEIN [Sinorhizobium meliloti 1021]
>gi|15965669|ref|NP_386022.1| HYPOTHETICAL TRANSMEMBRANE SIGNAL PEPTIDE PROTEIN [Sinorhizobium meliloti 1021]
>gi|15965713|ref|NP_386066.1| hypothetical protein SMc04306 [Sinorhizobium meliloti 1021]
>gi|15965720|ref|NP_386073.1| hypothetical protein SMc04312 [Sinorhizobium meliloti 1021]
>gi|15965731|ref|NP_386084.1| PUTATIVE ENTERICIDIN B SIGNAL PEPTIDE PROTEIN [Sinorhizobium meliloti 1021]
>gi|15965734|ref|NP_386087.1| HYPOTHETICAL LIPOPROTEIN TRANSMEMBRANE [Sinorhizobium meliloti 1021]
>gi|15965735|ref|NP_386088.1| HYPOTHETICAL TRANSMEMBRANE SIGNAL PEPTIDE PROTEIN [Sinorhizobium meliloti 1021]
>gi|15965736|ref|NP_386089.1| hypothetical protein SMc04337 [Sinorhizobium meliloti 1021]
>gi|15965740|ref|NP_386093.1| hypothetical protein SMc04345 [Sinorhizobium meliloti 1021]
>gi|15965749|ref|NP_386102.1| PUTATIVE HISTIDINE-RICH TRANSPORTER TRANSMEMBRANE PROTEIN [Sinorhizobium meliloti 1021]
>gi|15965753|ref|NP_386106.1| PUTATIVE HEMOLYSIN-TYPE CALCIUM-BINDING PROTEIN [Sinorhizobium meliloti 1021]
>gi|15965757|ref|NP_386110.1| PUTATIVE TRANSMEMBRANE PROTEIN [Sinorhizobium meliloti 1021]
>gi|15965764|ref|NP_386117.1| HYPOTHETICAL TRANSMEMBRANE PROTEIN [Sinorhizobium meliloti 1021]
>gi|15965771|ref|NP_386124.1| HYPOTHETICAL TRANSMEMBRANE PROTEIN [Sinorhizobium meliloti 1021]
>gi|15965772|ref|NP_386125.1| CONSERVED HYPOTHETICAL SIGNAL PEPTIDE PROTEIN [Sinorhizobium meliloti 1021]
>gi|15965778|ref|NP_386131.1| hypothetical protein SMc04197 [Sinorhizobium meliloti 1021]
>gi|15965783|ref|NP_386136.1| PUTATIVE TRANSMEMBRANE PROTEIN [Sinorhizobium meliloti 1021]
>gi|15965805|ref|NP_386158.1| hypothetical protein SMc04298 [Sinorhizobium meliloti 1021]
>gi|15965808|ref|NP_386161.1| PROBABLE FERRIC IRON TRANSPORT SYSTEM PERMEASE PROTEIN [Sinorhizobium meliloti 1021]
>gi|15965826|ref|NP_386179.1| PUTATIVE GLYCINE-RICH PROTEIN [Sinorhizobium meliloti 1021]
>gi|15965827|ref|NP_386180.1| PUTATIVE TRANSMEMBRANE PROTEIN [Sinorhizobium meliloti 1021]
>gi|15965836|ref|NP_386189.1| hypothetical protein SMc01405 [Sinorhizobium meliloti 1021]
>gi|15965842|ref|NP_386195.1| PUTATIVE LIPOPROTEIN TRANSMEMBRANE [Sinorhizobium meliloti 1021]
>gi|15965856|ref|NP_386209.1| HYPOTHETICAL TRANSMEMBRANE PROTEIN [Sinorhizobium meliloti 1021]
>gi|15965863|ref|NP_386216.1| hypothetical protein SMc01427 [Sinorhizobium meliloti 1021]
>gi|15965882|ref|NP_386235.1| hypothetical protein SMc01446 [Sinorhizobium meliloti 1021]
>gi|15965900|ref|NP_386253.1| HYPOTHETICAL TRANSMEMBRANE PROTEIN [Sinorhizobium meliloti 1021]
>gi|15965903|ref|NP_386256.1| HYPOTHETICAL SIGNAL PEPTIDE PROTEIN [Sinorhizobium meliloti 1021]
>gi|15965905|ref|NP_386258.1| PROBABLE METHYL ACCEPTING CHEMOTAXIS TRANSMEMBRANE PROTEIN [Sinorhizobium meliloti 1021]
>gi|15965906|ref|NP_386259.1| HYPOTHETICAL SIGNAL PEPTIDE PROTEIN [Sinorhizobium meliloti 1021]
>gi|15965921|ref|NP_386274.1| CELL DIVISION PROTEIN [Sinorhizobium meliloti 1021]
>gi|15965925|ref|NP_386278.1| PROBABLE AQUAPORIN Z (BACTERIAL NODULIN-LIKE INTRINSIC) TRANSMEMBRANE PROTEIN [Sinorhizobium meliloti 1021]
>gi|15965926|ref|NP_386279.1| PUTATIVE TRANSPORT TRANSMEMBRANE PROTEIN [Sinorhizobium meliloti 1021]
>gi|15965945|ref|NP_386298.1| hypothetical protein SMc01850 [Sinorhizobium meliloti 1021]
>gi|15966000|ref|NP_386353.1| PUTATIVE OXIDOREDUCTASE PROTEIN [Sinorhizobium meliloti 1021]
>gi|15966025|ref|NP_386378.1| HYPOTHETICAL TRANSMEMBRANE PROTEIN [Sinorhizobium meliloti 1021]
>gi|15966035|ref|NP_386388.1| HYPOTHETICAL TRANSMEMBRANE PROTEIN [Sinorhizobium meliloti 1021]
>gi|15966061|ref|NP_386414.1| HYPOTHETICAL SIGNAL PEPTIDE PROTEIN [Sinorhizobium meliloti 1021]
>gi|15966076|ref|NP_386429.1| PUTATIVE OXIDOREDUCTASE PROTEIN [Sinorhizobium meliloti 1021]
>gi|15966084|ref|NP_386437.1| HYPOTHETICAL TRANSMEMBRANE PROTEIN [Sinorhizobium meliloti 1021]
>gi|15966088|ref|NP_386441.1| HYPOTHETICAL TRANSMEMBRANE PROTEIN [Sinorhizobium meliloti 1021]
>gi|15966095|ref|NP_386448.1| hypothetical protein SMc03744 [Sinorhizobium meliloti 1021]
>gi|15966100|ref|NP_386453.1| HYPOTHETICAL TRANSMEMBRANE PROTEIN [Sinorhizobium meliloti 1021]
>gi|15966173|ref|NP_386526.1| HYPOTHETICAL TRANSMEMBRANE PROTEIN [Sinorhizobium meliloti 1021]
>gi|15966178|ref|NP_386531.1| hypothetical protein SMc01515 [Sinorhizobium meliloti 1021]
>gi|15966183|ref|NP_386536.1| PUTATIVE HEMIN TRANSPORT SYSTEM PERMEASE TRANSMEMBRANE PROTEIN [Sinorhizobium meliloti 1021]
>gi|15966186|ref|NP_386539.1| HYPOTHETICAL TRANSMEMBRANE PROTEIN [Sinorhizobium meliloti 1021]

>gi|15966250|ref|NP_386603.1| PUTATIVE SPERMIDINE/PUTRESCINE TRANSPORT SYSTEM PERMEASE ABC TRANSPORTER PROTEIN [Sinorhizobium meliloti 1021]
>gi|15966257|ref|NP_386610.1| HYPOTHETICAL TRANSMEMBRANE PROTEIN [Sinorhizobium meliloti 1021]
>gi|15966271|ref|NP_386624.1| PROBABLE CYTOCHROME-C OXIDASE TRANSMEMBRANE PROTEIN [Sinorhizobium meliloti 1021]
>gi|15966283|ref|NP_386636.1| hypothetical protein SMc02018 [Sinorhizobium meliloti 1021]
>gi|15966284|ref|NP_386637.1| PUTATIVE PERMEASE ABC TRANSPORTER PROTEIN [Sinorhizobium meliloti 1021]
>gi|15966294|ref|NP_386647.1| PUTATIVE DIPEPTIDE TRANSPORT SYSTEM PERMEASE ABC TRANSPORTER PROTEIN [Sinorhizobium meliloti 1021]
>gi|15966340|ref|NP_386693.1| PUTATIVE BRANCHED CHAIN AMINO ACID TRANSPORT SYSTEM PERMEASE ABC TRANSPORTER PROTEIN [Sinorhizobium meliloti 1021]
>gi|15966362|ref|NP_386715.1| hypothetical protein SMc02434 [Sinorhizobium meliloti 1021]
>gi|15966374|ref|NP_386727.1| hypothetical protein SMc02446 [Sinorhizobium meliloti 1021]
>gi|15966382|ref|NP_386735.1| HYPOTHETICAL TRANSMEMBRANE PROTEIN [Sinorhizobium meliloti 1021]
>gi|15966391|ref|NP_386744.1| PUTATIVE RIBONUCLEASE RNASE BN (RBN) TRANSMEMBRANE PROTEIN [Sinorhizobium meliloti 1021]
>gi|15966403|ref|NP_386756.1| HYPOTHETICAL TRANSMEMBRANE PROTEIN [Sinorhizobium meliloti 1021]
>gi|15966407|ref|NP_386760.1| HYPOTHETICAL TRANSMEMBRANE PROTEIN [Sinorhizobium meliloti 1021]
>gi|15966436|ref|NP_386789.1| HYPOTHETICAL LIPOPROTEIN [Sinorhizobium meliloti 1021]
>gi|15966465|ref|NP_386818.1| HYPOTHETICAL SIGNAL PEPTIDE PROTEIN [Sinorhizobium meliloti 1021]
>gi|15966480|ref|NP_386833.1| hypothetical protein SMc00644 [Sinorhizobium meliloti 1021]
>gi|15966490|ref|NP_386843.1| HYPOTHETICAL TRANSMEMBRANE PROTEIN [Sinorhizobium meliloti 1021]
>gi|15966493|ref|NP_386846.1| HYPOTHETICAL SIGNAL PEPTIDE PROTEIN [Sinorhizobium meliloti 1021]
>gi|15966514|ref|NP_386867.1| hypothetical protein SMc03977 [Sinorhizobium meliloti 1021]
>gi|15966541|ref|NP_386894.1| HYPOTHETICAL SIGNAL PEPTIDE PROTEIN [Sinorhizobium meliloti 1021]
>gi|15966547|ref|NP_386900.1| hypothetical protein SMc04010 [Sinorhizobium meliloti 1021]
>gi|15966551|ref|NP_386904.1| hypothetical protein SMc04014 [Sinorhizobium meliloti 1021]
>gi|15966557|ref|NP_386910.1| HYPOTHETICAL TRANSMEMBRANE PROTEIN [Sinorhizobium meliloti 1021]
>gi|15966558|ref|NP_386911.1| HYPOTHETICAL TRANSMEMBRANE PROTEIN [Sinorhizobium meliloti 1021]
>gi|15966586|ref|NP_386939.1| HYPOTHETICAL TRANSMEMBRANE PROTEIN [Sinorhizobium meliloti 1021]
>gi|15966611|ref|NP_386964.1| CONSERVED HYPOTHETICAL SIGNAL PEPTIDE PROTEIN [Sinorhizobium meliloti 1021]
>gi|15966614|ref|NP_386967.1| PROBABLE NAD(P) TRANSHYDROGENASE SUBUNIT ALPHA PART 2 TRANSMEMBRANE PROTEIN [Sinorhizobium meliloti 1021]
>gi|15966623|ref|NP_386976.1| hypothetical protein SMc02978 [Sinorhizobium meliloti 1021]
>gi|15966631|ref|NP_386984.1| HYPOTHETICAL TRANSMEMBRANE PROTEIN [Sinorhizobium meliloti 1021]
>gi|15966636|ref|NP_386989.1| HYPOTHETICAL TRANSMEMBRANE PROTEIN [Sinorhizobium meliloti 1021]
>gi|15966637|ref|NP_386990.1| PROBABLE KHG/KDPG ALDOLASE (INCLUDES: 4-HYDROXY-2-OXOGLUTARATE ALDOLASE, 2-DEHYDRO-3-DEOXYPHOSPHOGLUCONATE ALDOLASE) PROTEIN [Sinorhizobium meliloti 1021]
>gi|15966639|ref|NP_386992.1| hypothetical protein SMc03155 [Sinorhizobium meliloti 1021]
>gi|15966697|ref|NP_387050.1| hypothetical protein SMc03149 [Sinorhizobium meliloti 1021]
>gi|15966708|ref|NP_387061.1| PUTATIVE SUGAR KINASE PROTEIN [Sinorhizobium meliloti 1021]
>gi|15966712|ref|NP_387065.1| HYPOTHETICAL TRANSMEMBRANE PROTEIN [Sinorhizobium meliloti 1021]
>gi|15966738|ref|NP_387091.1| HYPOTHETICAL CALCIUM-BINDING PROTEIN [Sinorhizobium meliloti 1021]
>gi|15966746|ref|NP_387099.1| hypothetical protein SMc03100 [Sinorhizobium meliloti 1021]
>gi|15966766|ref|NP_387119.1| PUTATIVE OXIDOREDUCTASE PROTEIN [Sinorhizobium meliloti 1021]
>gi|15966777|ref|NP_387130.1| HYPOTHETICAL TRANSMEMBRANE PROTEIN [Sinorhizobium meliloti 1021]
>gi|15966782|ref|NP_387135.1| PUTATIVE IRON TRANSPORT SYSTEM MEMBRANE ABC TRANSPORTER PROTEIN [Sinorhizobium meliloti 1021]
>gi|15966791|ref|NP_387144.1| HYPOTHETICAL TRANSMEMBRANE PROTEIN [Sinorhizobium meliloti 1021]
>gi|15966805|ref|NP_387158.1| PROBABLE DIHYDROLIPOAMIDE SUCCINYL TRANSFERASE COMPONENT OF 2-OXOGLUTARATE DEHYDROGENASE COMPLEX (E2) PROTEIN [Sinorhizobium meliloti 1021]
>gi|15966813|ref|NP_387166.1| PUTATIVE OUTER MEMBRANE LIPOPROTEIN PRECURSOR [Sinorhizobium meliloti 1021]
>gi|15966834|ref|NP_387187.1| HYPOTHETICAL TRANSMEMBRANE PROTEIN [Sinorhizobium meliloti 1021]
>gi|15966868|ref|NP_387221.1| HYPOTHETICAL/UNKNOWN PROTEIN [Sinorhizobium meliloti 1021]
>gi|15966918|ref|NP_387271.1| HYPOTHETICAL SIGNAL PEPTIDE PROTEIN [Sinorhizobium meliloti 1021]
>gi|15966930|ref|NP_387283.1| HYPOTHETICAL/UNKNOWN TRANSMEMBRANE PROTEIN [Sinorhizobium meliloti 1021]
>gi|15966965|ref|NP_387318.1| PUTATIVE TRANSPORT SYSTEM PERMEASE ABC TRANSPORTER PROTEIN [Sinorhizobium meliloti 1021]
>gi|15966984|ref|NP_387337.1| PUTATIVE HEME EXPORTER B (CYTOCHROME C-TYPE BIOGENESIS PROTEIN) TRANSMEMBRANE [Sinorhizobium meliloti 1021]
>gi|15966986|ref|NP_387339.1| PUTATIVE HEME EXPORTER D (CYTOCHROME C-TYPE BIOGENESIS PROTEIN) TRANSMEMBRANE [Sinorhizobium meliloti 1021]
>gi|15966993|ref|NP_387346.1| PROBABLE SIGNAL RECOGNITION PARTICLE PROTEIN (FIFTY-FOUR HOMOLOG) [Sinorhizobium meliloti 1021]
>gi|15966998|ref|NP_387351.1| HYPOTHETICAL TRANSMEMBRANE PROTEIN [Sinorhizobium meliloti 1021]
>gi|15966999|ref|NP_387352.1| PROBABLE 50S RIBOSOMAL PROTEIN L19 [Sinorhizobium meliloti 1021]
>gi|15967006|ref|NP_387359.1| PUTATIVE TRANSPORT SYSTEM PERMEASE ABC TRANSPORTER PROTEIN [Sinorhizobium meliloti 1021]
>gi|15967025|ref|NP_387378.1| PUTATIVE TRANSPORT PROTEIN [Sinorhizobium meliloti 1021]
>gi|15967037|ref|NP_387390.1| hypothetical protein SMc03901 [Sinorhizobium meliloti 1021]
>gi|15967047|ref|NP_387400.1| PUTATIVE HYDROXYPYRUVATE REDUCTASE PROTEIN [Sinorhizobium meliloti 1021]

>gi|15967062|ref|NP_387415.1| PUTATIVE AMINO ACID EFFLUX TRANSMEMBRANE PROTEIN [Sinorhizobium meliloti 1021]
>gi|15967079|ref|NP_387432.1| hypothetical protein SMc04092 [Sinorhizobium meliloti 1021]
>gi|16263766|ref|NP_436558.1| putative sugar ABC transporter permease protein ABC TRANSPORTER [Sinorhizobium meliloti 1021]
>gi|16263767|ref|NP_436559.1| putative sugar ABC transporter permease protein ABC TRANSPORTER [Sinorhizobium meliloti 1021]
>gi|16263777|ref|NP_436569.1| hypothetical protein SMb20026 [Sinorhizobium meliloti 1021]
>gi|16263805|ref|NP_436597.1| putative ABC transporter permease protein [Sinorhizobium meliloti 1021]
>gi|16263810|ref|NP_436602.1| hypothetical protein SMb20062 [Sinorhizobium meliloti 1021]
>gi|16263827|ref|NP_436619.1| putative hemolysin-adenlyate cyclase protein [Sinorhizobium meliloti 1021]
>gi|16263833|ref|NP_436625.1| hypothetical protein SMb20085 [Sinorhizobium meliloti 1021]
>gi|16263835|ref|NP_436627.1| hypothetical protein SMb20087 [Sinorhizobium meliloti 1021]
>gi|16263852|ref|NP_436644.1| hypothetical membrane protein [Sinorhizobium meliloti 1021]
>gi|16263870|ref|NP_436662.1| hypothetical protein SMb20122 [Sinorhizobium meliloti 1021]
>gi|16263873|ref|NP_436665.1| putative sugar ABC transporter permease protein [Sinorhizobium meliloti 1021]
>gi|16263881|ref|NP_436673.1| hypothetical protein SMb20133 [Sinorhizobium meliloti 1021]
>gi|16263896|ref|NP_436688.1| putative transcriptional regulator protein [Sinorhizobium meliloti 1021]
>gi|16263901|ref|NP_436693.1| hypothetical protein TRANSMEMBRANE [Sinorhizobium meliloti 1021]
>gi|16263964|ref|NP_436756.1| probable osmotically inducible sensory protein [Sinorhizobium meliloti 1021]
>gi|16263968|ref|NP_436760.1| probable nutrient deprivation-induced protein [Sinorhizobium meliloti 1021]
>gi|16263998|ref|NP_436790.1| hypothetical protein SMb20260 [Sinorhizobium meliloti 1021]
>gi|16264025|ref|NP_436817.1| hypothetical protein SMb20287 [Sinorhizobium meliloti 1021]
>gi|16264028|ref|NP_436820.1| putative transcriptional regulator protein [Sinorhizobium meliloti 1021]
>gi|16264040|ref|NP_436832.1| hypothetical protein SMb20302 [Sinorhizobium meliloti 1021]
>gi|16264045|ref|NP_436837.1| putative dihydroxyacetone kinase protein [Sinorhizobium meliloti 1021]
>gi|16264053|ref|NP_436845.1| hypothetical protein SMb20319 [Sinorhizobium meliloti 1021]
>gi|16264167|ref|NP_436959.1| putative cyclodeaminase protein [Sinorhizobium meliloti 1021]
>gi|16264181|ref|NP_436973.1| hypothetical protein SMb20451 [Sinorhizobium meliloti 1021]
>gi|16264184|ref|NP_436976.1| hypothetical protein SMb20454 [Sinorhizobium meliloti 1021]
>gi|16264186|ref|NP_436978.1| putative dehydrogenasereductase protein [Sinorhizobium meliloti 1021]
>gi|16264200|ref|NP_436992.1| hypothetical protein SMb20470 [Sinorhizobium meliloti 1021]
>gi|16264216|ref|NP_437008.1| putative sugar ABC transporter permease protein [Sinorhizobium meliloti 1021]
>gi|16264223|ref|NP_437015.1| putative short chain dehydrogenasereductase protein [Sinorhizobium meliloti 1021]
>gi|16264232|ref|NP_437024.1| putative sugar ABC transporter permease protein [Sinorhizobium meliloti 1021]
>gi|16264245|ref|NP_437037.1| putative response regulator protein [Sinorhizobium meliloti 1021]
>gi|16264254|ref|NP_437046.1| hypothetical protein SMb20527 [Sinorhizobium meliloti 1021]
>gi|16264259|ref|NP_437051.1| hypothetical protein SMb20532 [Sinorhizobium meliloti 1021]
>gi|16264272|ref|NP_437064.1| hypothetical protein SMb20545 [Sinorhizobium meliloti 1021]
>gi|16264286|ref|NP_437078.1| hypothetical protein SMb20559 [Sinorhizobium meliloti 1021]
>gi|16264329|ref|NP_437121.1| putative secreted calcium-binding protein [Sinorhizobium meliloti 1021]
>gi|16264332|ref|NP_437124.1| hypothetical membrane-anchored protein [Sinorhizobium meliloti 1021]
>gi|16264343|ref|NP_437135.1| probable sugar kinase, probably EGGY family protein [Sinorhizobium meliloti 1021]
>gi|16264351|ref|NP_437143.1| hypothetical protein SMb21030 [Sinorhizobium meliloti 1021]
>gi|16264352|ref|NP_437144.1| hypothetical membrane-anchored protein [Sinorhizobium meliloti 1021]
>gi|16264353|ref|NP_437145.1| hypothetical proline-rich protein [Sinorhizobium meliloti 1021]
>gi|16264375|ref|NP_437167.1| hypothetical membrane protein [Sinorhizobium meliloti 1021]
>gi|16264425|ref|NP_437217.1| hypothetical membrane-anchored protein [Sinorhizobium meliloti 1021]
>gi|16264426|ref|NP_437218.1| hypothetical protein SMb21099 [Sinorhizobium meliloti 1021]
>gi|16264470|ref|NP_437262.1| putative sugar-processing enzyme, possibly aminotransferase protein [Sinorhizobium meliloti 1021]
>gi|16264471|ref|NP_437263.1| putative sugar uptake ABC transporter permease protein [Sinorhizobium meliloti 1021]
>gi|16264481|ref|NP_437273.1| putative calcium-binding exported protein [Sinorhizobium meliloti 1021]
>gi|16264520|ref|NP_437312.1| hypothetical membrane-anchored protein [Sinorhizobium meliloti 1021]
>gi|16264526|ref|NP_437318.1| putative spermidineputrescine ABC transporter permeasee protein [Sinorhizobium meliloti 1021]
>gi|16264533|ref|NP_437325.1| putative integral membrane transporter protein, xanthineuracil permeases family [Sinorhizobium meliloti 1021]
>gi|16264559|ref|NP_437351.1| putative choline uptake ABC transporter permease protein [Sinorhizobium meliloti 1021]
>gi|16264576|ref|NP_437368.1| putative integral membrane transporter protein [Sinorhizobium meliloti 1021]
>gi|16264580|ref|NP_437372.1| putative imidazolonepropionase protein [Sinorhizobium meliloti 1021]
>gi|16264621|ref|NP_437413.1| putative protein secretion protein, HlyD family [Sinorhizobium meliloti 1021]
>gi|16264638|ref|NP_437430.1| putative secreted calcium-binding protein [Sinorhizobium meliloti 1021]
>gi|16264658|ref|NP_437450.1| hypothetical exported glutamine-rich protein [Sinorhizobium meliloti 1021]
>gi|16264659|ref|NP_437451.1| conserved putative membrane protein, possibly a permease [Sinorhizobium meliloti 1021]
>gi|16264661|ref|NP_437453.1| putative iron-sulfur-binding protein, probably subunit of an oxidoreductase like aldehyde oxidase or xanthine dehydrogenase [Sinorhizobium meliloti 1021]
>gi|16264666|ref|NP_437458.1| putative sugar uptake ABC transporter permease protein [Sinorhizobium meliloti 1021]
>gi|16264697|ref|NP_437489.1| hypothetical membrane protein [Sinorhizobium meliloti 1021]
>gi|16264701|ref|NP_437493.1| putative sugar uptake ABC transporter permease protein [Sinorhizobium meliloti 1021]
>gi|16264719|ref|NP_437511.1| conserved hypothetical membrane protein [Sinorhizobium meliloti 1021]
>gi|16264723|ref|NP_437515.1| putative transcriptional regulator, lysR family protein [Sinorhizobium meliloti 1021]
>gi|16264724|ref|NP_437516.1| conserved hypothetical membrane protein [Sinorhizobium meliloti 1021]
>gi|16264732|ref|NP_437524.1| putative outer membrane secretion protein [Sinorhizobium meliloti 1021]
>gi|16264733|ref|NP_437525.1| hypothetical protein SMb21544 [Sinorhizobium meliloti 1021]
>gi|16264737|ref|NP_437529.1| hypothetical glycine-rich protein [Sinorhizobium meliloti 1021]

>gi|16264739|ref|NP_437531.1| hypothetical protein SMb21550 [Sinorhizobium meliloti 1021]
>gi|16264740|ref|NP_437532.1| hypothetical protein SMb21551 [Sinorhizobium meliloti 1021]
>gi|16264750|ref|NP_437542.1| hypothetical membrane-anchored protein [Sinorhizobium meliloti 1021]
>gi|16264755|ref|NP_437547.1| hypothetical protein SMb21567 [Sinorhizobium meliloti 1021]
>gi|16264770|ref|NP_437562.1| putative exoD-like membrane protein [Sinorhizobium meliloti 1021]
>gi|16264798|ref|NP_437590.1| hypothetical protein SMb20927 [Sinorhizobium meliloti 1021]
>gi|16264823|ref|NP_437615.1| hypothetical protein SMb21673 [Sinorhizobium meliloti 1021]
>gi|16264848|ref|NP_437640.1| putative amino acid uptake ABC transporter permease protein [Sinorhizobium meliloti 1021]
>gi|16264899|ref|NP_437691.1| hypothetical protein, similar to C-terminal part of L-lactate dehydrogenase (cytochrome) LldD [Sinorhizobium meliloti 1021]
>gi|16264931|ref|NP_437723.1| hypothetical glycine-rich protein [Sinorhizobium meliloti 1021]
>gi|16264944|ref|NP_437736.1| hypothetical protein SMb21679 [Sinorhizobium meliloti 1021]
>gi|16264956|ref|NP_437748.1| hypothetical protein SMb20910 [Sinorhizobium meliloti 1021]
>gi|16264968|ref|NP_437760.1| hypothetical protein SMb20922 [Sinorhizobium meliloti 1021]
>gi|16264969|ref|NP_437761.1| hypothetical protein SMb21395 [Sinorhizobium meliloti 1021]
>gi|16264977|ref|NP_437769.1| hypothetical calcium binding protein [Sinorhizobium meliloti 1021]
>gi|16264988|ref|NP_437780.1| hypothetical protein SMb21413 [Sinorhizobium meliloti 1021]
>gi|16264999|ref|NP_437791.1| putative sugar uptake ABC transporter permease protein [Sinorhizobium meliloti 1021]
>gi|16265018|ref|NP_437810.1| putative inosine-5'-monophosphate dehydrogenase protein [Sinorhizobium meliloti 1021]
>gi|16265019|ref|NP_437811.1| hypothetical protein SMb21442 [Sinorhizobium meliloti 1021]
>gi|16265020|ref|NP_437812.1| hypothetical protein SMb21443 [Sinorhizobium meliloti 1021]
>gi|16265021|ref|NP_437813.1| conserved hypothetical protein, homolog to osmotically inducible sensory protein SMC22-1 (AF178441) [Sinorhizobium meliloti 1021]
>gi|16265061|ref|NP_437853.1| hypothetical protein SMb21485 [Sinorhizobium meliloti 1021]
>gi|16265062|ref|NP_437854.1| putative metabolite transport protein [Sinorhizobium meliloti 1021]
>gi|16265067|ref|NP_437859.1| putative SUR1-like protein, similar to Bradyrhizobium japonicum shb1 gene [Sinorhizobium meliloti 1021]
>gi|16265068|ref|NP_437860.1| hypothetical protein SMb21491 [Sinorhizobium meliloti 1021]
>gi|16265074|ref|NP_437866.1| putative protease protein [Sinorhizobium meliloti 1021]
>gi|16265078|ref|NP_437870.1| putative glycosyltransferase protein [Sinorhizobium meliloti 1021]
>gi|16265079|ref|NP_437871.1| hypothetical membrane protein [Sinorhizobium meliloti 1021]
>gi|16265096|ref|NP_437888.1| hypothetical protein SMb21518 [Sinorhizobium meliloti 1021]
>gi|16265099|ref|NP_437891.1| hypothetical exported protein, glycine-rich [Sinorhizobium meliloti 1021]
>gi|16265125|ref|NP_437917.1| hypothetical protein SMb20670 [Sinorhizobium meliloti 1021]
>gi|16265131|ref|NP_437923.1| hypothetical protein SMb20676 [Sinorhizobium meliloti 1021]
>gi|16265133|ref|NP_437925.1| putative hydroxypyruvate reductase protein [Sinorhizobium meliloti 1021]
>gi|16265148|ref|NP_437940.1| hypothetical protein SMb20693 [Sinorhizobium meliloti 1021]
>gi|16265153|ref|NP_437945.1| putative transport protein, similar to E. coli multidrug resistance protein EmrB [Sinorhizobium meliloti 1021]
>gi|16265154|ref|NP_437946.1| putative protein secretion protein, HlyD family, similar to E. coli EmrK [Sinorhizobium meliloti 1021]
>gi|16265176|ref|NP_437968.1| putative two-component sensor histidine kinase protein [Sinorhizobium meliloti 1021]
>gi|16265182|ref|NP_437974.1| hypothetical protein SMb20727 [Sinorhizobium meliloti 1021]
>gi|16265205|ref|NP_437997.1| putative acetyltransferase protein [Sinorhizobium meliloti 1021]
>gi|16265226|ref|NP_438018.1| putative branched-chain amino acid uptake ABC transporter permease protein [Sinorhizobium meliloti 1021]
>gi|16265227|ref|NP_438019.1| putative branched-chain amino acid uptake ABC transporter permease protein [Sinorhizobium meliloti 1021]
>gi|16265256|ref|NP_438048.1| hypothetical protein SMb20596 [Sinorhizobium meliloti 1021]
>gi|16265278|ref|NP_438070.1| putative thiamine-phosphate pyrophosphorylase protein [Sinorhizobium meliloti 1021]
>gi|16265289|ref|NP_438081.1| hypothetical protein SMb20629 [Sinorhizobium meliloti 1021]
>gi|16265309|ref|NP_438101.1| hypothetical protein SMb21642 [Sinorhizobium meliloti 1021]
>gi|15791435|ref|NP_281258.1| hypothetical protein Cj0036 [Campylobacter jejuni subsp. jejuni NCTC 11168]
>gi|15791437|ref|NP_281260.1| putative membrane protein [Campylobacter jejuni subsp. jejuni NCTC 11168]
>gi|15791439|ref|NP_281262.1| hypothetical protein Cj0040 [Campylobacter jejuni subsp. jejuni NCTC 11168]
>gi|15791440|ref|NP_281263.1| hypothetical protein Cj0041 [Campylobacter jejuni subsp. jejuni NCTC 11168]
>gi|15791441|ref|NP_281264.1| putative flagellar hook assembly protein [Campylobacter jejuni subsp. jejuni NCTC 11168]
>gi|15791457|ref|NP_281280.1| putative 2-amino-4-hydroxy-6-hydroxymethyldihydropteridine pyrophosphokinase [Campylobacter jejuni subsp. jejuni NCTC 11168]
>gi|15791491|ref|NP_281314.1| ATP synthase F0 sector B subunit [Campylobacter jejuni subsp. jejuni NCTC 11168]
>gi|15791529|ref|NP_281352.1| ABC transporter integral membrane protein [Campylobacter jejuni subsp. jejuni NCTC 11168]
>gi|15791554|ref|NP_281377.1| putative integral membrane protein [Campylobacter jejuni subsp. jejuni NCTC 11168]
>gi|15791555|ref|NP_281378.1| putative periplasmic protein [Campylobacter jejuni subsp. jejuni NCTC 11168]
>gi|15791573|ref|NP_281396.1| putative integral membrane protein [Campylobacter jejuni subsp. jejuni NCTC 11168]
>gi|15791590|ref|NP_281413.1| putative transmembrane transport protein [Campylobacter jejuni subsp. jejuni NCTC 11168]
>gi|15791604|ref|NP_281427.1| putative integral membrane protein [Campylobacter jejuni subsp. jejuni NCTC 11168]
>gi|15791622|ref|NP_281445.1| highly acidic protein [Campylobacter jejuni subsp. jejuni NCTC 11168]
>gi|15791628|ref|NP_281451.1| diacylglycerol kinase [Campylobacter jejuni subsp. jejuni NCTC 11168]
>gi|15791637|ref|NP_281460.1| putative integral membrane protein [Campylobacter jejuni subsp. jejuni NCTC 11168]
>gi|15791711|ref|NP_281534.1| putative integral membrane protein [Campylobacter jejuni subsp. jejuni NCTC 11168]
>gi|15791737|ref|NP_281560.1| 30S ribosomal protein S21 [Campylobacter jejuni subsp. jejuni NCTC 11168]
>gi|15791745|ref|NP_281568.1| putative integral membrane protein [Campylobacter jejuni subsp. jejuni NCTC 11168]
>gi|15791766|ref|NP_281589.1| putative integral membrane protein [Campylobacter jejuni subsp. jejuni NCTC 11168]
>gi|15791771|ref|NP_281594.1| putative transmembrane protein [Campylobacter jejuni subsp. jejuni NCTC 11168]
>gi|15791785|ref|NP_281608.1| hypothetical protein Cj0418c [Campylobacter jejuni subsp. jejuni NCTC 11168]

>gi|15791795|ref|NP_281618.1| hypothetical protein Cj0428 [Campylobacter jejuni subsp. jejuni NCTC 11168]
>gi|15791797|ref|NP_281620.1| putative integral membrane protein [Campylobacter jejuni subsp. jejuni NCTC 11168]
>gi|15791841|ref|NP_281664.1| 50S ribosomal protein L7 /L12 [Campylobacter jejuni subsp. jejuni NCTC 11168]
>gi|15791844|ref|NP_281667.1| putative transcriptional regulator [Campylobacter jejuni subsp. jejuni NCTC 11168]
>gi|15791883|ref|NP_281706.1| putative membrane protein [Campylobacter jejuni subsp. jejuni NCTC 11168]
>gi|15791905|ref|NP_281728.1| putative integral memnbrane protein [Campylobacter jejuni subsp. jejuni NCTC 11168]
>gi|15791947|ref|NP_281770.1| putative integral membrane protein [Campylobacter jejuni subsp. jejuni NCTC 11168]
>gi|15791963|ref|NP_281786.1| putative thiol:disulfide interchange protein [Campylobacter jejuni subsp. jejuni NCTC 11168]
>gi|15791980|ref|NP_281803.1| hypothetical protein Cj0620 [Campylobacter jejuni subsp. jejuni NCTC 11168]
>gi|15791989|ref|NP_281812.1| possible lipoprotein [Campylobacter jejuni subsp. jejuni NCTC 11168]
>gi|15792014|ref|NP_281837.1| putative periplasmic protein [Campylobacter jejuni subsp. jejuni NCTC 11168]
>gi|15792015|ref|NP_281838.1| putative transmembrane protein [Campylobacter jejuni subsp. jejuni NCTC 11168]
>gi|15792026|ref|NP_281849.1| putative periplasmic protein [Campylobacter jejuni subsp. jejuni NCTC 11168]
>gi|15792032|ref|NP_281855.1| putative periplasmic protein [Campylobacter jejuni subsp. jejuni NCTC 11168]
>gi|15792041|ref|NP_281864.1| putative membrane protein [Campylobacter jejuni subsp. jejuni NCTC 11168]
>gi|15792070|ref|NP_281893.1| putative integral membrane protein [Campylobacter jejuni subsp. jejuni NCTC 11168]
>gi|15792101|ref|NP_281924.1| serine acetyltransferase [Campylobacter jejuni subsp. jejuni NCTC 11168]
>gi|15792114|ref|NP_281937.1| putative periplasmic protein [Campylobacter jejuni subsp. jejuni NCTC 11168]
>gi|15792120|ref|NP_281943.1| putative ferredoxin [Campylobacter jejuni subsp. jejuni NCTC 11168]
>gi|15792124|ref|NP_281947.1| small hydrophobic protein [Campylobacter jejuni subsp. jejuni NCTC 11168]
>gi|15792157|ref|NP_281980.1| small hydrophobic protein [Campylobacter jejuni subsp. jejuni NCTC 11168]
>gi|15792163|ref|NP_281986.1| putative processing peptidase [Campylobacter jejuni subsp. jejuni NCTC 11168]
>gi|15792187|ref|NP_282010.1| hypothetical protein Cj0849c [Campylobacter jejuni subsp. jejuni NCTC 11168]
>gi|15792188|ref|NP_282011.1| transmembrane transport protein [Campylobacter jejuni subsp. jejuni NCTC 11168]
>gi|15792265|ref|NP_282088.1| ATP synthase F0 sector C subunit [Campylobacter jejuni subsp. jejuni NCTC 11168]
>gi|15792315|ref|NP_282138.1| hypothetical protein Cj0988c [Campylobacter jejuni subsp. jejuni NCTC 11168]
>gi|15792339|ref|NP_282162.1| putative membrane protein [Campylobacter jejuni subsp. jejuni NCTC 11168]
>gi|15792343|ref|NP_282166.1| putative branched-chain amino-acid ABC transport system permease protein [Campylobacter jejuni subsp. jejuni NCTC 11168]
>gi|15792374|ref|NP_282197.1| hypothetical protein Cj1047c [Campylobacter jejuni subsp. jejuni NCTC 11168]
>gi|15792496|ref|NP_282319.1| hypothetical protein Cj1172c [Campylobacter jejuni subsp. jejuni NCTC 11168]
>gi|15792497|ref|NP_282320.1| putative efflux protein [Campylobacter jejuni subsp. jejuni NCTC 11168]
>gi|15792498|ref|NP_282321.1| putative efflux protein [Campylobacter jejuni subsp. jejuni NCTC 11168]
>gi|15792502|ref|NP_282325.1| highly acidic protein [Campylobacter jejuni subsp. jejuni NCTC 11168]
>gi|15792511|ref|NP_282334.1| putative arsenical pump membrane protein [Campylobacter jejuni subsp. jejuni NCTC 11168]
>gi|15792527|ref|NP_282350.1| putative integral membrane protein [Campylobacter jejuni subsp. jejuni NCTC 11168]
>gi|15792533|ref|NP_282356.1| hypothetical protein Cj1209 [Campylobacter jejuni subsp. jejuni NCTC 11168]
>gi|15792543|ref|NP_282366.1| putative periplasmic protein [Campylobacter jejuni subsp. jejuni NCTC 11168]
>gi|15792553|ref|NP_282376.1| putative curved-DNA binding protein [Campylobacter jejuni subsp. jejuni NCTC 11168]
>gi|15792556|ref|NP_282379.1| hypothetical protein Cj1232 [Campylobacter jejuni subsp. jejuni NCTC 11168]
>gi|15792565|ref|NP_282388.1| putative transmembrane transport protein [Campylobacter jejuni subsp. jejuni NCTC 11168]
>gi|15792571|ref|NP_282394.1| hypothetical protein Cj1247c [Campylobacter jejuni subsp. jejuni NCTC 11168]
>gi|15792658|ref|NP_282481.1| hypothetical protein Cj1335 [Campylobacter jejuni subsp. jejuni NCTC 11168]
>gi|15792661|ref|NP_282484.1| flagellin [Campylobacter jejuni subsp. jejuni NCTC 11168]
>gi|15792662|ref|NP_282485.1| flagellin [Campylobacter jejuni subsp. jejuni NCTC 11168]
>gi|15792671|ref|NP_282494.1| putative coiled-coil protein [Campylobacter jejuni subsp. jejuni NCTC 11168]
>gi|15792672|ref|NP_282495.1| possible fibronectin/fibrinogen-binding protein [Campylobacter jejuni subsp. jejuni NCTC 11168]
>gi|15792675|ref|NP_282498.1| enterochelin uptake permease [Campylobacter jejuni subsp. jejuni NCTC 11168]
>gi|15792724|ref|NP_282547.1| putative periplasmic protein [Campylobacter jejuni subsp. jejuni NCTC 11168]
>gi|15792751|ref|NP_282574.1| hypothetical protein Cj1433c [Campylobacter jejuni subsp. jejuni NCTC 11168]
>gi|15792812|ref|NP_282635.1| hypothetical protein Cj1497c [Campylobacter jejuni subsp. jejuni NCTC 11168]
>gi|15792830|ref|NP_282653.1| possible molybdopterin converting factor, subunit 1 [Campylobacter jejuni subsp. jejuni NCTC 11168]
>gi|15792833|ref|NP_282656.1| hypotetical protein Cj1520 [Campylobacter jejuni subsp. jejuni NCTC 11168]
>gi|15792838|ref|NP_282661.1| putative ATP/GTP-binding protein [Campylobacter jejuni subsp. jejuni NCTC 11168]
>gi|15792847|ref|NP_282670.1| putative anion-uptake ABC-transport system permease protein [Campylobacter jejuni subsp. jejuni NCTC 11168]
>gi|15792875|ref|NP_282698.1| NADH dehydrogenase I chain J [Campylobacter jejuni subsp. jejuni NCTC 11168]
>gi|15792887|ref|NP_282710.1| putative peptide ABC-transport system permease protein [Campylobacter jejuni subsp. jejuni NCTC 11168]
>gi|15792928|ref|NP_282751.1| putative membrane protein [Campylobacter jejuni subsp. jejuni NCTC 11168]
>gi|15792935|ref|NP_282758.1| putative tonB transport protein [Campylobacter jejuni subsp. jejuni NCTC 11168]
>gi|15792953|ref|NP_282776.1| possible ABC transport system periplasmic substrate-binding protein [Campylobacter jejuni subsp. jejuni NCTC 11168]
>gi|15792981|ref|NP_282804.1| putative lipoprotein [Campylobacter jejuni subsp. jejuni NCTC 11168]
>gi|15792982|ref|NP_282805.1| possible lipoprotein [Campylobacter jejuni subsp. jejuni NCTC 11168]
>gi|15792999|ref|NP_282822.1| 50S ribosomal protein L24 [Campylobacter jejuni subsp. jejuni NCTC 11168]
>gi|15793002|ref|NP_282825.1| 50S ribosomal protein L29 [Campylobacter jejuni subsp. jejuni NCTC 11168]
>gi|15793026|ref|NP_282849.1| putative periplasmic protein [Campylobacter jejuni subsp. jejuni NCTC 11168]
>gi|29826541|ref|NP_821175.1| hypothetical protein SAV1 [Streptomyces avermitilis MA-4680]
>gi|29826552|ref|NP_821186.1| hypothetical protein SAV12 [Streptomyces avermitilis MA-4680]
>gi|29826556|ref|NP_821190.1| hypothetical protein SAV16 [Streptomyces avermitilis MA-4680]
>gi|29826570|ref|NP_821204.1| hypothetical protein SAV30 [Streptomyces avermitilis MA-4680]

>gi|29826582|ref|NP_821216.1| hypothetical protein SAV42 [Streptomyces avermitilis MA-4680]
>gi|29826586|ref|NP_821220.1| putative IS1647-like transposase [Streptomyces avermitilis MA-4680]
>gi|29826590|ref|NP_821224.1| hypothetical protein SAV50 [Streptomyces avermitilis MA-4680]
>gi|29826592|ref|NP_821226.1| hypothetical protein SAV52 [Streptomyces avermitilis MA-4680]
>gi|29826597|ref|NP_821231.1| hypothetical protein SAV57 [Streptomyces avermitilis MA-4680]
>gi|29826598|ref|NP_821232.1| hypothetical protein SAV58 [Streptomyces avermitilis MA-4680]
>gi|29826602|ref|NP_821236.1| putative transmembrane transport protein [Streptomyces avermitilis MA-4680]
>gi|29826608|ref|NP_821242.1| hypothetical protein SAV68 [Streptomyces avermitilis MA-4680]
>gi|29826612|ref|NP_821246.1| putative two-component system sensor kinase [Streptomyces avermitilis MA-4680]
>gi|29826623|ref|NP_821257.1| hypothetical protein SAV83 [Streptomyces avermitilis MA-4680]
>gi|29826625|ref|NP_821259.1| putative MerR-family transcriptional regulator [Streptomyces avermitilis MA-4680]
>gi|29826627|ref|NP_821261.1| hypothetical protein SAV87 [Streptomyces avermitilis MA-4680]
>gi|29826634|ref|NP_821268.1| hypothetical protein SAV94 [Streptomyces avermitilis MA-4680]
>gi|29826636|ref|NP_821270.1| hypothetical protein SAV96 [Streptomyces avermitilis MA-4680]
>gi|29826637|ref|NP_821271.1| hypothetical protein SAV97 [Streptomyces avermitilis MA-4680]
>gi|29826639|ref|NP_821273.1| putative integral membrane protein [Streptomyces avermitilis MA-4680]
>gi|29826641|ref|NP_821275.1| putative polyketide synthase [Streptomyces avermitilis MA-4680]
>gi|29826646|ref|NP_821280.1| hypothetical protein SAV106 [Streptomyces avermitilis MA-4680]
>gi|29826650|ref|NP_821284.1| putative transposase [Streptomyces avermitilis MA-4680]
>gi|29826651|ref|NP_821285.1| putative LuxR-family transcriptional regulator [Streptomyces avermitilis MA-4680]
>gi|29826652|ref|NP_821286.1| hypothetical protein SAV112 [Streptomyces avermitilis MA-4680]
>gi|57833848|ref|NP_821293.2| putative IS1647-like transposase [Streptomyces avermitilis MA-4680]
>gi|29826664|ref|NP_821298.1| putative membrane protein [Streptomyces avermitilis MA-4680]
>gi|29826665|ref|NP_821299.1| putative membrane protein [Streptomyces avermitilis MA-4680]
>gi|29826666|ref|NP_821300.1| putative integral membrane protein [Streptomyces avermitilis MA-4680]
>gi|29826671|ref|NP_821305.1| putative integral membrane protein [Streptomyces avermitilis MA-4680]
>gi|29826672|ref|NP_821306.1| hypothetical protein SAV132 [Streptomyces avermitilis MA-4680]
>gi|29826673|ref|NP_821307.1| putative transporter [Streptomyces avermitilis MA-4680]
>gi|29826674|ref|NP_821308.1| putative two-component system sensor kinase [Streptomyces avermitilis MA-4680]
>gi|29826675|ref|NP_821309.1| putative lipoprotein [Streptomyces avermitilis MA-4680]
>gi|29826677|ref|NP_821311.1| putative ABC transporter ATP-binding protein [Streptomyces avermitilis MA-4680]
>gi|29826678|ref|NP_821312.1| putative ABC transporter integral membrane protein [Streptomyces avermitilis MA-4680]
>gi|29826692|ref|NP_821326.1| putative dehydrogenase [Streptomyces avermitilis MA-4680]
>gi|29826712|ref|NP_821346.1| hypothetical protein SAV172 [Streptomyces avermitilis MA-4680]
>gi|29826713|ref|NP_821347.1| hypothetical protein SAV173 [Streptomyces avermitilis MA-4680]
>gi|29826714|ref|NP_821348.1| hypothetical protein SAV174 [Streptomyces avermitilis MA-4680]
>gi|29826716|ref|NP_821350.1| hypothetical protein SAV176 [Streptomyces avermitilis MA-4680]
>gi|29826717|ref|NP_821351.1| putative membrane protein [Streptomyces avermitilis MA-4680]
>gi|29826719|ref|NP_821353.1| hypothetical protein SAV179 [Streptomyces avermitilis MA-4680]
>gi|29826723|ref|NP_821357.1| hypothetical protein SAV183 [Streptomyces avermitilis MA-4680]
>gi|29826730|ref|NP_821364.1| putative membrane protein [Streptomyces avermitilis MA-4680]
>gi|29826731|ref|NP_821365.1| putative membrane protein [Streptomyces avermitilis MA-4680]
>gi|29826732|ref|NP_821366.1| putative membrane protein [Streptomyces avermitilis MA-4680]
>gi|29826743|ref|NP_821377.1| hypothetical protein SAV203 [Streptomyces avermitilis MA-4680]
>gi|29826745|ref|NP_821379.1| putative TetR-family transcriptional regulator [Streptomyces avermitilis MA-4680]
>gi|29826751|ref|NP_821385.1| hypothetical protein SAV211 [Streptomyces avermitilis MA-4680]
>gi|29826752|ref|NP_821386.1| hypothetical protein SAV212 [Streptomyces avermitilis MA-4680]
>gi|29826762|ref|NP_821396.1| hypothetical protein SAV222 [Streptomyces avermitilis MA-4680]
>gi|29826776|ref|NP_821410.1| hypothetical protein SAV236 [Streptomyces avermitilis MA-4680]
>gi|29826780|ref|NP_821414.1| hypothetical protein SAV240 [Streptomyces avermitilis MA-4680]
>gi|29826781|ref|NP_821415.1| hypothetical protein SAV241 [Streptomyces avermitilis MA-4680]
>gi|29826785|ref|NP_821419.1| putative LysE-family efflux protein [Streptomyces avermitilis MA-4680]
>gi|29826787|ref|NP_821421.1| hypothetical protein SAV247 [Streptomyces avermitilis MA-4680]
>gi|29826789|ref|NP_821423.1| hypothetical protein SAV249 [Streptomyces avermitilis MA-4680]
>gi|29826790|ref|NP_821424.1| hypothetical protein SAV250 [Streptomyces avermitilis MA-4680]
>gi|29826800|ref|NP_821434.1| putative transposase [Streptomyces avermitilis MA-4680]
>gi|29826802|ref|NP_821436.1| hypothetical protein SAV262 [Streptomyces avermitilis MA-4680]
>gi|29826804|ref|NP_821438.1| hypothetical protein SAV264 [Streptomyces avermitilis MA-4680]
>gi|29826818|ref|NP_821452.1| hypothetical protein SAV278 [Streptomyces avermitilis MA-4680]
>gi|29826820|ref|NP_821454.1| hypothetical protein SAV280 [Streptomyces avermitilis MA-4680]
>gi|29826823|ref|NP_821457.1| hypothetical protein SAV283 [Streptomyces avermitilis MA-4680]
>gi|29826824|ref|NP_821458.1| hypothetical protein SAV284 [Streptomyces avermitilis MA-4680]
>gi|29826834|ref|NP_821468.1| putative invasion protein [Streptomyces avermitilis MA-4680]
>gi|29826844|ref|NP_821478.1| hypothetical protein SAV304 [Streptomyces avermitilis MA-4680]
>gi|29826860|ref|NP_821494.1| hypothetical protein SAV320 [Streptomyces avermitilis MA-4680]
>gi|29826862|ref|NP_821496.1| putative membrane protein [Streptomyces avermitilis MA-4680]
>gi|29826863|ref|NP_821497.1| putative MerR-family transcriptional regulator [Streptomyces avermitilis MA-4680]
>gi|29826870|ref|NP_821504.1| hypothetical protein SAV330 [Streptomyces avermitilis MA-4680]
>gi|29826873|ref|NP_821507.1| hypothetical protein SAV332 [Streptomyces avermitilis MA-4680]
>gi|29826882|ref|NP_821516.1| hypothetical protein SAV342 [Streptomyces avermitilis MA-4680]

>gi|29826885|ref|NP_821519.1| hypothetical protein SAV345 [Streptomyces avermitilis MA-4680]
>gi|29826896|ref|NP_821530.1| putative membrane protein [Streptomyces avermitilis MA-4680]
>gi|29826908|ref|NP_821542.1| hypothetical protein SAV368 [Streptomyces avermitilis MA-4680]
>gi|29826916|ref|NP_821550.1| hypothetical protein SAV376 [Streptomyces avermitilis MA-4680]
>gi|29826919|ref|NP_821553.1| hypothetical protein SAV379 [Streptomyces avermitilis MA-4680]
>gi|29826920|ref|NP_821554.1| hypothetical protein SAV380 [Streptomyces avermitilis MA-4680]
>gi|29826923|ref|NP_821557.1| hypothetical protein SAV383 [Streptomyces avermitilis MA-4680]
>gi|29826924|ref|NP_821558.1| hypothetical protein SAV384 [Streptomyces avermitilis MA-4680]
>gi|29826925|ref|NP_821559.1| hypothetical protein SAV385 [Streptomyces avermitilis MA-4680]
>gi|29826926|ref|NP_821560.1| hypothetical protein SAV386 [Streptomyces avermitilis MA-4680]
>gi|29826927|ref|NP_821561.1| putative AAA family ATPase [Streptomyces avermitilis MA-4680]
>gi|29826929|ref|NP_821563.1| hypothetical protein SAV389 [Streptomyces avermitilis MA-4680]
>gi|29826934|ref|NP_821568.1| hypothetical protein SAV394 [Streptomyces avermitilis MA-4680]
>gi|29826940|ref|NP_821574.1| putative transcriptional regulator [Streptomyces avermitilis MA-4680]
>gi|29826941|ref|NP_821575.1| putative LuxR-family transcriptional regulator [Streptomyces avermitilis MA-4680]
>gi|29826942|ref|NP_821576.1| hypothetical protein SAV402 [Streptomyces avermitilis MA-4680]
>gi|29826944|ref|NP_821578.1| putative pyridine nucleotide transhydrogenase, alpha subunit [Streptomyces avermitilis MA-4680]
>gi|29826945|ref|NP_821579.1| putative acetyltransferase [Streptomyces avermitilis MA-4680]
>gi|29826947|ref|NP_821581.1| putative dipeptidase [Streptomyces avermitilis MA-4680]
>gi|29826971|ref|NP_821605.1| putative dehydrogenase [Streptomyces avermitilis MA-4680]
>gi|29826975|ref|NP_821609.1| hypothetical protein SAV434 [Streptomyces avermitilis MA-4680]
>gi|29826992|ref|NP_821626.1| putative anti-sigma factor antagonist [Streptomyces avermitilis MA-4680]
>gi|29826998|ref|NP_821632.1| hypothetical protein SAV457 [Streptomyces avermitilis MA-4680]
>gi|29826999|ref|NP_821633.1| putative hsp18 transcriptional regulator [Streptomyces avermitilis MA-4680]
>gi|29827005|ref|NP_821639.1| hypothetical protein SAV464 [Streptomyces avermitilis MA-4680]
>gi|29827011|ref|NP_821645.1| putative transposase [Streptomyces avermitilis MA-4680]
>gi|29827013|ref|NP_821647.1| putative regulatory protein [Streptomyces avermitilis MA-4680]
>gi|29827030|ref|NP_821664.1| hypothetical protein SAV489 [Streptomyces avermitilis MA-4680]
>gi|57833854|ref|YP_187528.1| putative IS402-like transposase [Streptomyces avermitilis MA-4680]
>gi|29827047|ref|NP_821681.1| hypothetical protein SAV506 [Streptomyces avermitilis MA-4680]
>gi|29827062|ref|NP_821696.1| hypothetical protein SAV521 [Streptomyces avermitilis MA-4680]
>gi|29827068|ref|NP_821702.1| putative RNA polymerase ECF-subfamily sigma factor [Streptomyces avermitilis MA-4680]
>gi|29827069|ref|NP_821703.1| putative integral membrane protein [Streptomyces avermitilis MA-4680]
>gi|29827071|ref|NP_821705.1| hypothetical protein SAV530 [Streptomyces avermitilis MA-4680]
>gi|29827072|ref|NP_821706.1| hypothetical protein SAV531 [Streptomyces avermitilis MA-4680]
>gi|29827073|ref|NP_821707.1| hypothetical protein SAV532 [Streptomyces avermitilis MA-4680]
>gi|29827082|ref|NP_821716.1| putative 3-oxoadipate enol-lactone hydrolase/4-carboxymuconolactone decarboxylase [Streptomyces avermitilis MA-4680]
>gi|29827093|ref|NP_821727.1| putative transmembrane efflux protein [Streptomyces avermitilis MA-4680]
>gi|29827100|ref|NP_821734.1| hypothetical protein SAV559 [Streptomyces avermitilis MA-4680]
>gi|29827108|ref|NP_821742.1| hypothetical protein SAV567 [Streptomyces avermitilis MA-4680]
>gi|29827115|ref|NP_821749.1| putative dehydrogenase [Streptomyces avermitilis MA-4680]
>gi|29827122|ref|NP_821756.1| hypothetical protein SAV581 [Streptomyces avermitilis MA-4680]
>gi|29827132|ref|NP_821766.1| putative gas vesicle synthesis protein [Streptomyces avermitilis MA-4680]
>gi|29827133|ref|NP_821767.1| putative gas vesicle synthesis protein [Streptomyces avermitilis MA-4680]
>gi|29827139|ref|NP_821773.1| putative gas vesicle synthesis protein [Streptomyces avermitilis MA-4680]
>gi|29827140|ref|NP_821774.1| putative gas vesicle synthesis protein [Streptomyces avermitilis MA-4680]
>gi|29827141|ref|NP_821775.1| putative ABC transporter iron(III)/siderophore transport system ATP-binding protein [Streptomyces avermitilis MA-4680]
>gi|29827142|ref|NP_821776.1| putative ABC transporter iron(III)/siderophore permease protein [Streptomyces avermitilis MA-4680]
>gi|29827150|ref|NP_821784.1| putative 3-oxoacyl-ACP synthase III [Streptomyces avermitilis MA-4680]
>gi|29827151|ref|NP_821785.1| putative MFS transporter protein [Streptomyces avermitilis MA-4680]
>gi|29827154|ref|NP_821788.1| hypothetical protein SAV613 [Streptomyces avermitilis MA-4680]
>gi|29827155|ref|NP_821789.1| putative RNA polymerase ECF-subfamily sigma factor [Streptomyces avermitilis MA-4680]
>gi|29827156|ref|NP_821790.1| hypothetical protein SAV615 [Streptomyces avermitilis MA-4680]
>gi|29827158|ref|NP_821792.1| putative cation-transporting P-type ATPase [Streptomyces avermitilis MA-4680]
>gi|29827159|ref|NP_821793.1| hypothetical protein SAV618 [Streptomyces avermitilis MA-4680]
>gi|29827172|ref|NP_821806.1| hypothetical protein SAV632 [Streptomyces avermitilis MA-4680]
>gi|29827181|ref|NP_821815.1| hypothetical protein SAV640 [Streptomyces avermitilis MA-4680]
>gi|29827182|ref|NP_821816.1| hypothetical protein SAV641 [Streptomyces avermitilis MA-4680]
>gi|29827189|ref|NP_821823.1| putative two-component system response regulator [Streptomyces avermitilis MA-4680]
>gi|29827193|ref|NP_821827.1| putative fosmidmycin resistance protein [Streptomyces avermitilis MA-4680]
>gi|29827213|ref|NP_821847.1| hypothetical protein SAV672 [Streptomyces avermitilis MA-4680]
>gi|29827216|ref|NP_821850.1| putative TetR-family transcriptional regulator [Streptomyces avermitilis MA-4680]
>gi|29827218|ref|NP_821852.1| putative dehydrogenase [Streptomyces avermitilis MA-4680]
>gi|29827232|ref|NP_821866.1| putative hsp18 transcriptional regulator [Streptomyces avermitilis MA-4680]
>gi|29827234|ref|NP_821868.1| hypothetical protein SAV693 [Streptomyces avermitilis MA-4680]
>gi|29827239|ref|NP_821873.1| putative magnesium or manganese-dependent protein phosphatase [Streptomyces avermitilis MA-4680]
>gi|29827241|ref|NP_821875.1| putative RNA polymerase ECF-subfamily sigma factor [Streptomyces avermitilis MA-4680]
>gi|29827242|ref|NP_821876.1| hypothetical protein SAV701 [Streptomyces avermitilis MA-4680]

>gi|29827243|ref|NP_821877.1| putative acyltransferase [Streptomyces avermitilis MA-4680]
>gi|29827247|ref|NP_821881.1| putative dehydrogenase [Streptomyces avermitilis MA-4680]
>gi|29827258|ref|NP_821892.1| putative enoyl-CoA hydratase [Streptomyces avermitilis MA-4680]
>gi|29827266|ref|NP_821900.1| hypothetical protein SAV725 [Streptomyces avermitilis MA-4680]
>gi|29827280|ref|NP_821914.1| putative membrane protein [Streptomyces avermitilis MA-4680]
>gi|29827291|ref|NP_821925.1| putative TetR-family transcriptional regulator [Streptomyces avermitilis MA-4680]
>gi|29827294|ref|NP_821928.1| hypothetical protein SAV753 [Streptomyces avermitilis MA-4680]
>gi|29827296|ref|NP_821930.1| putative dehydrogenase [Streptomyces avermitilis MA-4680]
>gi|29827297|ref|NP_821931.1| hypothetical protein SAV756 [Streptomyces avermitilis MA-4680]
>gi|29827302|ref|NP_821936.1| hypothetical protein SAV761 [Streptomyces avermitilis MA-4680]
>gi|29827305|ref|NP_821939.1| hypothetical protein SAV764 [Streptomyces avermitilis MA-4680]
>gi|29827306|ref|NP_821940.1| hypothetical protein SAV765 [Streptomyces avermitilis MA-4680]
>gi|29827309|ref|NP_821943.1| hypothetical protein SAV768 [Streptomyces avermitilis MA-4680]
>gi|29827322|ref|NP_821956.1| hypothetical protein SAV781 [Streptomyces avermitilis MA-4680]
>gi|29827324|ref|NP_821958.1| hypothetical protein SAV783 [Streptomyces avermitilis MA-4680]
>gi|29827325|ref|NP_821959.1| hypothetical protein SAV784 [Streptomyces avermitilis MA-4680]
>gi|29827332|ref|NP_821966.1| putative transmembrane transport protein [Streptomyces avermitilis MA-4680]
>gi|29827333|ref|NP_821967.1| putative transcriptional regulator [Streptomyces avermitilis MA-4680]
>gi|29827334|ref|NP_821968.1| putative membrane protein [Streptomyces avermitilis MA-4680]
>gi|29827337|ref|NP_821971.1| hypothetical protein SAV796 [Streptomyces avermitilis MA-4680]
>gi|29827339|ref|NP_821973.1| hypothetical protein SAV798 [Streptomyces avermitilis MA-4680]
>gi|29827341|ref|NP_821975.1| hypothetical protein SAV800 [Streptomyces avermitilis MA-4680]
>gi|29827348|ref|NP_821982.1| hypothetical protein SAV807 [Streptomyces avermitilis MA-4680]
>gi|29827351|ref|NP_821985.1| putative dehydrogenase [Streptomyces avermitilis MA-4680]
>gi|29827355|ref|NP_821989.1| hypothetical protein SAV814 [Streptomyces avermitilis MA-4680]
>gi|29827356|ref|NP_821990.1| hypothetical protein SAV815 [Streptomyces avermitilis MA-4680]
>gi|29827361|ref|NP_821995.1| hypothetical protein SAV820 [Streptomyces avermitilis MA-4680]
>gi|29827365|ref|NP_821999.1| hypothetical protein SAV824 [Streptomyces avermitilis MA-4680]
>gi|29827408|ref|NP_822042.1| putative ABC transporter ATP-binding protein [Streptomyces avermitilis MA-4680]
>gi|29827411|ref|NP_822045.1| hypothetical protein SAV870 [Streptomyces avermitilis MA-4680]
>gi|29827414|ref|NP_822048.1| hypothetical protein SAV873 [Streptomyces avermitilis MA-4680]
>gi|29827423|ref|NP_822057.1| putative TetR-family transcriptional regulator [Streptomyces avermitilis MA-4680]
>gi|29827440|ref|NP_822074.1| hypothetical protein SAV899 [Streptomyces avermitilis MA-4680]
>gi|29827452|ref|NP_822086.1| putative dehydrogenase [Streptomyces avermitilis MA-4680]
>gi|29827454|ref|NP_822088.1| putative TetR-family transcriptional regulator [Streptomyces avermitilis MA-4680]
>gi|29827467|ref|NP_822101.1| putative transcriptional activator SRCAP homolog [Streptomyces avermitilis MA-4680]
>gi|29827473|ref|NP_822107.1| putative two-component system sensor kinase [Streptomyces avermitilis MA-4680]
>gi|29827475|ref|NP_822109.1| putative ABC transporter integral membrane protein [Streptomyces avermitilis MA-4680]
>gi|29827476|ref|NP_822110.1| LuxR-family transcriptional regulator [Streptomyces avermitilis MA-4680]
>gi|29827479|ref|NP_822113.1| type I polyketide synthase AVES 1 [Streptomyces avermitilis MA-4680]
>gi|29827480|ref|NP_822114.1| type I polyketide synthase AVES 2 [Streptomyces avermitilis MA-4680]
>gi|29827483|ref|NP_822117.1| type I polyketide synthase AVES 3 [Streptomyces avermitilis MA-4680]
>gi|29827484|ref|NP_822118.1| type I polyketide synthase AVES 4 [Streptomyces avermitilis MA-4680]
>gi|29827485|ref|NP_822119.1| reductase [Streptomyces avermitilis MA-4680]
>gi|29827498|ref|NP_822132.1| hypothetical protein SAV957 [Streptomyces avermitilis MA-4680]
>gi|29827499|ref|NP_822133.1| hypothetical protein SAV958 [Streptomyces avermitilis MA-4680]
>gi|29827502|ref|NP_822136.1| putative RNA polymerase ECF-subfamily sigma factor [Streptomyces avermitilis MA-4680]
>gi|29827505|ref|NP_822139.1| hypothetical protein SAV964 [Streptomyces avermitilis MA-4680]
>gi|29827509|ref|NP_822143.1| putative ribose/xylose/arabinose/galactoside ABC transporter permease protein [Streptomyces avermitilis MA-4680]
>gi|29827510|ref|NP_822144.1| putative sugar uptake ABC transporter permease protein [Streptomyces avermitilis MA-4680]
>gi|29827544|ref|NP_822178.1| putative dehydrogenase [Streptomyces avermitilis MA-4680]
>gi|29827546|ref|NP_822180.1| putative oxidoreductase [Streptomyces avermitilis MA-4680]
>gi|29827547|ref|NP_822181.1| hypothetical protein SAV1006 [Streptomyces avermitilis MA-4680]
>gi|29827551|ref|NP_822185.1| hypothetical protein SAV1010 [Streptomyces avermitilis MA-4680]
>gi|29827555|ref|NP_822189.1| putative NDP-hexose 4-ketoreductase [Streptomyces avermitilis MA-4680]
>gi|29827556|ref|NP_822190.1| hypothetical protein SAV1015 [Streptomyces avermitilis MA-4680]
>gi|29827559|ref|NP_822193.1| putative integral membrane protein [Streptomyces avermitilis MA-4680]
>gi|57833856|ref|NP_822197.2| polyprenyl diphosphate synthase [Streptomyces avermitilis MA-4680]
>gi|29827565|ref|NP_822199.1| phytoene synthase [Streptomyces avermitilis MA-4680]
>gi|29827578|ref|NP_822212.1| putative neutral zinc metalloprotease [Streptomyces avermitilis MA-4680]
>gi|29827583|ref|NP_822217.1| putative membrane protein [Streptomyces avermitilis MA-4680]
>gi|29827592|ref|NP_822226.1| putative oxidoreductase [Streptomyces avermitilis MA-4680]
>gi|29827599|ref|NP_822233.1| hypothetical protein SAV1058 [Streptomyces avermitilis MA-4680]
>gi|29827600|ref|NP_822234.1| putative membrane protein [Streptomyces avermitilis MA-4680]
>gi|29827603|ref|NP_822237.1| hypothetical protein SAV1062 [Streptomyces avermitilis MA-4680]
>gi|29827604|ref|NP_822238.1| putative membrane protein [Streptomyces avermitilis MA-4680]
>gi|29827614|ref|NP_822248.1| hypothetical protein SAV1073 [Streptomyces avermitilis MA-4680]
>gi|29827629|ref|NP_822263.1| hypothetical protein SAV1088 [Streptomyces avermitilis MA-4680]
>gi|29827637|ref|NP_822271.1| putative anti-sigma factor [Streptomyces avermitilis MA-4680]
>gi|29827638|ref|NP_822272.1| putative magnesium or manganese-dependent protein phosphatase [Streptomyces avermitilis MA-4680]

>gi|29827650|ref|NP_822284.1| hypothetical protein SAV1109 [Streptomyces avermitilis MA-4680]
>gi|29827651|ref|NP_822285.1| hypothetical protein SAV1110 [Streptomyces avermitilis MA-4680]
>gi|29827655|ref|NP_822289.1| hypothetical protein SAV1114 [Streptomyces avermitilis MA-4680]
>gi|29827657|ref|NP_822291.1| hypothetical protein SAV1116 [Streptomyces avermitilis MA-4680]
>gi|29827661|ref|NP_822295.1| putative membrane protein [Streptomyces avermitilis MA-4680]
>gi|29827670|ref|NP_822304.1| putative polyprenyl diphosphate synthase [Streptomyces avermitilis MA-4680]
>gi|29827671|ref|NP_822305.1| hypothetical protein SAV1130 [Streptomyces avermitilis MA-4680]
>gi|29827673|ref|NP_822307.1| putative TetR-family transcriptional regulator [Streptomyces avermitilis MA-4680]
>gi|29827677|ref|NP_822311.1| putative tyrosinase co-factor protein [Streptomyces avermitilis MA-4680]
>gi|29827679|ref|NP_822313.1| putative ribonuclease H [Streptomyces avermitilis MA-4680]
>gi|29827689|ref|NP_822323.1| putative DNA-binding protein [Streptomyces avermitilis MA-4680]
>gi|29827693|ref|NP_822327.1| hypothetical protein SAV1152 [Streptomyces avermitilis MA-4680]
>gi|29827695|ref|NP_822329.1| hypothetical protein SAV1154 [Streptomyces avermitilis MA-4680]
>gi|29827696|ref|NP_822330.1| putative membrane protein [Streptomyces avermitilis MA-4680]
>gi|29827708|ref|NP_822342.1| hypothetical protein SAV1167 [Streptomyces avermitilis MA-4680]
>gi|29827710|ref|NP_822344.1| hypothetical protein SAV1169 [Streptomyces avermitilis MA-4680]
>gi|29827711|ref|NP_822345.1| putative sodium-dependent transporter [Streptomyces avermitilis MA-4680]
>gi|29827715|ref|NP_822349.1| hypothetical protein SAV1174 [Streptomyces avermitilis MA-4680]
>gi|29827717|ref|NP_822351.1| hypothetical protein SAV1176 [Streptomyces avermitilis MA-4680]
>gi|29827736|ref|NP_822370.1| putative RNA polymerase ECF-subfamily sigma factor [Streptomyces avermitilis MA-4680]
>gi|29827741|ref|NP_822375.1| putative dehydrogenase [Streptomyces avermitilis MA-4680]
>gi|29827747|ref|NP_822381.1| putative acyl-CoA dehydrogenase [Streptomyces avermitilis MA-4680]
>gi|29827751|ref|NP_822385.1| hypothetical protein SAV1210 [Streptomyces avermitilis MA-4680]
>gi|29827757|ref|NP_822391.1| putative membrane protein, ribonuclease BN-like family [Streptomyces avermitilis MA-4680]
>gi|29827763|ref|NP_822397.1| putative ROK-family transcriptional regulator [Streptomyces avermitilis MA-4680]
>gi|29827766|ref|NP_822400.1| putative transglycosylase associated protein [Streptomyces avermitilis MA-4680]
>gi|29827769|ref|NP_822403.1| hypothetical protein SAV1228 [Streptomyces avermitilis MA-4680]
>gi|29827771|ref|NP_822405.1| putative secreted protein [Streptomyces avermitilis MA-4680]
>gi|29827793|ref|NP_822427.1| putative sensor-like histidine kinase [Streptomyces avermitilis MA-4680]
>gi|29827816|ref|NP_822450.1| putative stress-inducible protein [Streptomyces avermitilis MA-4680]
>gi|29827828|ref|NP_822462.1| putative dihydroxyacetone kinase phosphotransfer protein [Streptomyces avermitilis MA-4680]
>gi|29827829|ref|NP_822463.1| putative secreted protein [Streptomyces avermitilis MA-4680]
>gi|29827835|ref|NP_822469.1| hypothetical protein SAV1294 [Streptomyces avermitilis MA-4680]
>gi|29827838|ref|NP_822472.1| putative dehydrogenase [Streptomyces avermitilis MA-4680]
>gi|29827841|ref|NP_822475.1| putative stress-inducible protein [Streptomyces avermitilis MA-4680]
>gi|29827853|ref|NP_822487.1| putative membrane protein [Streptomyces avermitilis MA-4680]
>gi|29827854|ref|NP_822488.1| hypothetical protein SAV1313 [Streptomyces avermitilis MA-4680]
>gi|29827857|ref|NP_822491.1| putative L-asparaginase II [Streptomyces avermitilis MA-4680]
>gi|29827861|ref|NP_822495.1| hypothetical protein SAV1320 [Streptomyces avermitilis MA-4680]
>gi|29827864|ref|NP_822498.1| hypothetical protein SAV1323 [Streptomyces avermitilis MA-4680]
>gi|29827868|ref|NP_822502.1| putative ABC transporter permease protein [Streptomyces avermitilis MA-4680]
>gi|29827873|ref|NP_822507.1| putative oxidoreductase [Streptomyces avermitilis MA-4680]
>gi|29827876|ref|NP_822510.1| hypothetical protein SAV1335 [Streptomyces avermitilis MA-4680]
>gi|29827883|ref|NP_822517.1| putative transmembrane efflux protein [Streptomyces avermitilis MA-4680]
>gi|29827884|ref|NP_822518.1| hypothetical protein SAV1343 [Streptomyces avermitilis MA-4680]
>gi|29827903|ref|NP_822537.1| putative transcriptional regulator [Streptomyces avermitilis MA-4680]
>gi|29827910|ref|NP_822544.1| hypothetical protein SAV1369 [Streptomyces avermitilis MA-4680]
>gi|29827919|ref|NP_822553.1| putative membrane transport protein [Streptomyces avermitilis MA-4680]
>gi|29827921|ref|NP_822555.1| putative 3-oxoacyl-ACP reductase [Streptomyces avermitilis MA-4680]
>gi|29827942|ref|NP_822576.1| hypothetical protein SAV1401 [Streptomyces avermitilis MA-4680]
>gi|29827945|ref|NP_822579.1| putative oxidoreductase [Streptomyces avermitilis MA-4680]
>gi|29827952|ref|NP_822586.1| putative ABC transporter permease protein [Streptomyces avermitilis MA-4680]
>gi|29827960|ref|NP_822594.1| hypothetical protein SAV1419 [Streptomyces avermitilis MA-4680]
>gi|29827963|ref|NP_822597.1| putative polyprenol-phosphate-mannosyl transferase [Streptomyces avermitilis MA-4680]
>gi|29827964|ref|NP_822598.1| hypothetical protein SAV1423 [Streptomyces avermitilis MA-4680]
>gi|29827969|ref|NP_822603.1| hypothetical protein SAV1428 [Streptomyces avermitilis MA-4680]
>gi|29827973|ref|NP_822607.1| putative membrane protein [Streptomyces avermitilis MA-4680]
>gi|29827979|ref|NP_822613.1| putative membrane protein [Streptomyces avermitilis MA-4680]
>gi|29827986|ref|NP_822620.1| putative tagatose-bisphosphate aldolase [Streptomyces avermitilis MA-4680]
>gi|29827988|ref|NP_822622.1| putative DeoR-family transcriptional regulator [Streptomyces avermitilis MA-4680]
>gi|29827990|ref|NP_822624.1| hypothetical protein SAV1449 [Streptomyces avermitilis MA-4680]
>gi|29827993|ref|NP_822627.1| hypothetical protein SAV1452 [Streptomyces avermitilis MA-4680]
>gi|29828002|ref|NP_822636.1| putative integral membrane transport protein [Streptomyces avermitilis MA-4680]
>gi|29828006|ref|NP_822640.1| putative two-component system sensor kinase [Streptomyces avermitilis MA-4680]
>gi|29828017|ref|NP_822651.1| putative ROK-family transcriptional regulator [Streptomyces avermitilis MA-4680]
>gi|29828020|ref|NP_822654.1| hypothetical protein SAV1479 [Streptomyces avermitilis MA-4680]
>gi|29828022|ref|NP_822656.1| putative electron transfer flavoprotein, beta subunit [Streptomyces avermitilis MA-4680]
>gi|29828025|ref|NP_822659.1| hypothetical protein SAV1484 [Streptomyces avermitilis MA-4680]
>gi|29828030|ref|NP_822664.1| putative dehydrogenase [Streptomyces avermitilis MA-4680]
>gi|29828032|ref|NP_822666.1| hypothetical protein SAV1491 [Streptomyces avermitilis MA-4680]

>gi|29828039|ref|NP_822673.1| putative integral membrane protein [Streptomyces avermitilis MA-4680]
>gi|29828040|ref|NP_822674.1| putative integral membrane protein [Streptomyces avermitilis MA-4680]
>gi|29828041|ref|NP_822675.1| putative integral membrane protein [Streptomyces avermitilis MA-4680]
>gi|29828042|ref|NP_822676.1| hypothetical protein SAV1501 [Streptomyces avermitilis MA-4680]
>gi|29828053|ref|NP_822687.1| hypothetical protein SAV1512 [Streptomyces avermitilis MA-4680]
>gi|29828056|ref|NP_822690.1| putative transport integral membrane protein [Streptomyces avermitilis MA-4680]
>gi|29828057|ref|NP_822691.1| putative integral membrane protein [Streptomyces avermitilis MA-4680]
>gi|29828058|ref|NP_822692.1| putative LysR-family transcriptional regulator [Streptomyces avermitilis MA-4680]
>gi|29828069|ref|NP_822703.1| hypothetical protein SAV1528 [Streptomyces avermitilis MA-4680]
>gi|29828070|ref|NP_822704.1| hypothetical protein SAV1529 [Streptomyces avermitilis MA-4680]
>gi|29828073|ref|NP_822707.1| putative membrane protein [Streptomyces avermitilis MA-4680]
>gi|29828077|ref|NP_822711.1| putative hydrolase [Streptomyces avermitilis MA-4680]
>gi|29828079|ref|NP_822713.1| putative oxidoreductase [Streptomyces avermitilis MA-4680]
>gi|29828087|ref|NP_822721.1| hypothetical protein SAV1545 [Streptomyces avermitilis MA-4680]
>gi|29828089|ref|NP_822723.1| putative ribose-5-phosphate isomerase [Streptomyces avermitilis MA-4680]
>gi|29828092|ref|NP_822726.1| putative modular polyketide synthase [Streptomyces avermitilis MA-4680]
>gi|29828110|ref|NP_822744.1| putative lipoprotein [Streptomyces avermitilis MA-4680]
>gi|29828111|ref|NP_822745.1| putative ABC transporter transmembrane protein [Streptomyces avermitilis MA-4680]
>gi|29828112|ref|NP_822746.1| putative ABC transporter ATP-binding protein [Streptomyces avermitilis MA-4680]
>gi|29828113|ref|NP_822747.1| hypothetical protein SAV1571 [Streptomyces avermitilis MA-4680]
>gi|29828115|ref|NP_822749.1| putative integral membrane protein [Streptomyces avermitilis MA-4680]
>gi|29828125|ref|NP_822759.1| putative integral membrane protein [Streptomyces avermitilis MA-4680]
>gi|29828135|ref|NP_822769.1| hypothetical protein SAV1593 [Streptomyces avermitilis MA-4680]
>gi|29828136|ref|NP_822770.1| hypothetical protein SAV1594 [Streptomyces avermitilis MA-4680]
>gi|29828141|ref|NP_822775.1| hypothetical protein SAV1599 [Streptomyces avermitilis MA-4680]
>gi|29828143|ref|NP_822777.1| putative precorrin-6X reductase [Streptomyces avermitilis MA-4680]
>gi|29828149|ref|NP_822783.1| putative integral membrane protein [Streptomyces avermitilis MA-4680]
>gi|29828152|ref|NP_822786.1| putative ferredoxin [Streptomyces avermitilis MA-4680]
>gi|29828175|ref|NP_822809.1| putative sensor-like histidine kinase [Streptomyces avermitilis MA-4680]
>gi|29828179|ref|NP_822813.1| putative TetR-family transcriptional regulator [Streptomyces avermitilis MA-4680]
>gi|29828182|ref|NP_822816.1| putative secreted peptidase [Streptomyces avermitilis MA-4680]
>gi|29828203|ref|NP_822837.1| putative transferase [Streptomyces avermitilis MA-4680]
>gi|29828210|ref|NP_822844.1| hypothetical alanine-rich protein [Streptomyces avermitilis MA-4680]
>gi|29828211|ref|NP_822845.1| putative transcription accessory protein [Streptomyces avermitilis MA-4680]
>gi|29828226|ref|NP_822860.1| putative endonuclease V [Streptomyces avermitilis MA-4680]
>gi|29828230|ref|NP_822864.1| hypothetical protein SAV1688 [Streptomyces avermitilis MA-4680]
>gi|29828231|ref|NP_822865.1| putative ABC transporter ATP-binding protein [Streptomyces avermitilis MA-4680]
>gi|29828234|ref|NP_822868.1| hypothetical protein SAV1692 [Streptomyces avermitilis MA-4680]
>gi|29828247|ref|NP_822881.1| putative 3-carboxy-cis,cis-muconate cycloisomerase [Streptomyces avermitilis MA-4680]
>gi|29828252|ref|NP_822886.1| putative membrane protein [Streptomyces avermitilis MA-4680]
>gi|29828254|ref|NP_822888.1| hypothetical protein SAV1712 [Streptomyces avermitilis MA-4680]
>gi|29828255|ref|NP_822889.1| putative integral membrane protein [Streptomyces avermitilis MA-4680]
>gi|29828256|ref|NP_822890.1| hypothetical protein SAV1714 [Streptomyces avermitilis MA-4680]
>gi|29828261|ref|NP_822895.1| putative ABC transporter substrate-binding periplasmic transport [Streptomyces avermitilis MA-4680]
>gi|29828262|ref|NP_822896.1| putative ABC transporter permease protein [Streptomyces avermitilis MA-4680]
>gi|29828263|ref|NP_822897.1| putative ABC transporter ATP-binding protein [Streptomyces avermitilis MA-4680]
>gi|29828274|ref|NP_822908.1| putative oxidase [Streptomyces avermitilis MA-4680]
>gi|29828282|ref|NP_822916.1| hypothetical protein SAV1740 [Streptomyces avermitilis MA-4680]
>gi|29828304|ref|NP_822938.1| hypothetical protein SAV1762 [Streptomyces avermitilis MA-4680]
>gi|29828319|ref|NP_822953.1| putative shikimate 5-dehydrogenase [Streptomyces avermitilis MA-4680]
>gi|29828323|ref|NP_822957.1| putative TetR-family transcriptional regulator [Streptomyces avermitilis MA-4680]
>gi|29828324|ref|NP_822958.1| putative transmembrane efflux protein [Streptomyces avermitilis MA-4680]
>gi|29828327|ref|NP_822961.1| hypothetical protein SAV1785 [Streptomyces avermitilis MA-4680]
>gi|29828328|ref|NP_822962.1| putative MerR-family transcriptional regulator [Streptomyces avermitilis MA-4680]
>gi|29828332|ref|NP_822966.1| putative ornithine cyclodeaminase [Streptomyces avermitilis MA-4680]
>gi|29828335|ref|NP_822969.1| hypothetical protein SAV1793 [Streptomyces avermitilis MA-4680]
>gi|29828336|ref|NP_822970.1| hypothetical protein SAV1794 [Streptomyces avermitilis MA-4680]
>gi|29828352|ref|NP_822986.1| putative regulatory protein [Streptomyces avermitilis MA-4680]
>gi|29828353|ref|NP_822987.1| putative regulatory protein [Streptomyces avermitilis MA-4680]
>gi|29828360|ref|NP_822994.1| putative succinyl-CoA synthetase beta subunit [Streptomyces avermitilis MA-4680]
>gi|29828371|ref|NP_823005.1| putative ROK-family transcriptional regulator [Streptomyces avermitilis MA-4680]
>gi|29828382|ref|NP_823016.1| putative dehydrogenase [Streptomyces avermitilis MA-4680]
>gi|29828389|ref|NP_823023.1| putative integral membrane efflux protein [Streptomyces avermitilis MA-4680]
>gi|29828411|ref|NP_823045.1| putative integral membrane protein [Streptomyces avermitilis MA-4680]
>gi|29828412|ref|NP_823046.1| hypothetical protein SAV1870 [Streptomyces avermitilis MA-4680]
>gi|29828413|ref|NP_823047.1| hypothetical protein SAV1871 [Streptomyces avermitilis MA-4680]
>gi|29828414|ref|NP_823048.1| hypothetical protein SAV1872 [Streptomyces avermitilis MA-4680]
>gi|29828421|ref|NP_823055.1| hypothetical protein SAV1879 [Streptomyces avermitilis MA-4680]
>gi|29828424|ref|NP_823058.1| putative gas vesicle synthesis protein [Streptomyces avermitilis MA-4680]
>gi|29828427|ref|NP_823061.1| hypothetical protein SAV1885 [Streptomyces avermitilis MA-4680]

>gi|29828428|ref|NP_823062.1| hypothetical protein SAV1886 [Streptomyces avermitilis MA-4680]
>gi|29828431|ref|NP_823065.1| putative gas vesicle synthesis protein [Streptomyces avermitilis MA-4680]
>gi|29828435|ref|NP_823069.1| putative membrane protein [Streptomyces avermitilis MA-4680]
>gi|29828450|ref|NP_823084.1| putative magnesium or manganese-dependent protein phosphatase [Streptomyces avermitilis MA-4680]
>gi|29828460|ref|NP_823094.1| putative glycerate kinase [Streptomyces avermitilis MA-4680]
>gi|29828469|ref|NP_823103.1| putative integral membrane protein [Streptomyces avermitilis MA-4680]
>gi|29828481|ref|NP_823115.1| hypothetical protein SAV1939 [Streptomyces avermitilis MA-4680]
>gi|29828484|ref|NP_823118.1| hypothetical protein SAV1942 [Streptomyces avermitilis MA-4680]
>gi|29828488|ref|NP_823122.1| hypothetical protein SAV1946 [Streptomyces avermitilis MA-4680]
>gi|29828496|ref|NP_823130.1| hypothetical protein SAV1954 [Streptomyces avermitilis MA-4680]
>gi|29828497|ref|NP_823131.1| putative DNA-binding protein [Streptomyces avermitilis MA-4680]
>gi|29828519|ref|NP_823153.1| hypothetical protein SAV1977 [Streptomyces avermitilis MA-4680]
>gi|29828520|ref|NP_823154.1| putative SNF2/RAD54 family helicase [Streptomyces avermitilis MA-4680]
>gi|29828524|ref|NP_823158.1| putative ABC transporter sugar permease [Streptomyces avermitilis MA-4680]
>gi|29828532|ref|NP_823166.1| putative two-component system sensor kinase [Streptomyces avermitilis MA-4680]
>gi|29828534|ref|NP_823168.1| hypothetical protein SAV1992 [Streptomyces avermitilis MA-4680]
>gi|29828540|ref|NP_823174.1| hypothetical protein SAV1998 [Streptomyces avermitilis MA-4680]
>gi|29828541|ref|NP_823175.1| hypothetical protein SAV1999 [Streptomyces avermitilis MA-4680]
>gi|29828546|ref|NP_823180.1| putative iron transport protein [Streptomyces avermitilis MA-4680]
>gi|29828548|ref|NP_823182.1| hypothetical protein SAV2006 [Streptomyces avermitilis MA-4680]
>gi|29828549|ref|NP_823183.1| putative transcriptional regulator [Streptomyces avermitilis MA-4680]
>gi|29828552|ref|NP_823186.1| hypothetical protein SAV2010 [Streptomyces avermitilis MA-4680]
>gi|29828555|ref|NP_823189.1| putative transport integral membrane protein [Streptomyces avermitilis MA-4680]
>gi|29828562|ref|NP_823196.1| hypothetical protein SAV2020 [Streptomyces avermitilis MA-4680]
>gi|29828564|ref|NP_823198.1| hypothetical protein SAV2022 [Streptomyces avermitilis MA-4680]
>gi|29828571|ref|NP_823205.1| hypothetical protein SAV2029 [Streptomyces avermitilis MA-4680]
>gi|29828574|ref|NP_823208.1| putative LuxR-family transcriptional regulator [Streptomyces avermitilis MA-4680]
>gi|29828575|ref|NP_823209.1| putative LuxR-family transcriptional regulator [Streptomyces avermitilis MA-4680]
>gi|29828577|ref|NP_823211.1| hypothetical protein SAV2035 [Streptomyces avermitilis MA-4680]
>gi|29828590|ref|NP_823224.1| hypothetical protein SAV2048 [Streptomyces avermitilis MA-4680]
>gi|29828593|ref|NP_823227.1| putative hydrolase [Streptomyces avermitilis MA-4680]
>gi|29828594|ref|NP_823228.1| hypothetical protein SAV2052 [Streptomyces avermitilis MA-4680]
>gi|29828601|ref|NP_823235.1| putative two-component system sensor kinase [Streptomyces avermitilis MA-4680]
>gi|29828607|ref|NP_823241.1| hypothetical protein SAV2065 [Streptomyces avermitilis MA-4680]
>gi|29828613|ref|NP_823247.1| hypothetical protein SAV2071 [Streptomyces avermitilis MA-4680]
>gi|29828614|ref|NP_823248.1| hypothetical protein SAV2072 [Streptomyces avermitilis MA-4680]
>gi|29828615|ref|NP_823249.1| putative GntR-family transcriptional regulator [Streptomyces avermitilis MA-4680]
>gi|29828618|ref|NP_823252.1| putative regulatory protein [Streptomyces avermitilis MA-4680]
>gi|29828622|ref|NP_823256.1| hypothetical protein SAV2080 [Streptomyces avermitilis MA-4680]
>gi|29828624|ref|NP_823258.1| hypothetical protein SAV2082 [Streptomyces avermitilis MA-4680]
>gi|29828627|ref|NP_823261.1| putative ADA-like regulatory protein [Streptomyces avermitilis MA-4680]
>gi|29828629|ref|NP_823263.1| putative cellulose-binding protein [Streptomyces avermitilis MA-4680]
>gi|29828642|ref|NP_823276.1| hypothetical protein SAV2100 [Streptomyces avermitilis MA-4680]
>gi|29828645|ref|NP_823279.1| hypothetical protein SAV2103 [Streptomyces avermitilis MA-4680]
>gi|29828652|ref|NP_823286.1| hypothetical protein SAV2110 [Streptomyces avermitilis MA-4680]
>gi|29828654|ref|NP_823288.1| putative hydrolase [Streptomyces avermitilis MA-4680]
>gi|29828657|ref|NP_823291.1| putative membrane protein [Streptomyces avermitilis MA-4680]
>gi|29828659|ref|NP_823293.1| hypothetical protein SAV2117 [Streptomyces avermitilis MA-4680]
>gi|29828661|ref|NP_823295.1| putative AraC-family transcriptional regulator [Streptomyces avermitilis MA-4680]
>gi|29828663|ref|NP_823297.1| hypothetical protein SAV2121 [Streptomyces avermitilis MA-4680]
>gi|29828664|ref|NP_823298.1| putative integral membrane protein, ribonuclease BN-like family [Streptomyces avermitilis MA-4680]
>gi|29828666|ref|NP_823300.1| putative regulatory protein [Streptomyces avermitilis MA-4680]
>gi|29828674|ref|NP_823308.1| putative sulfate adenylyltransferase large subunit [Streptomyces avermitilis MA-4680]
>gi|29828688|ref|NP_823322.1| hypothetical protein SAV2146 [Streptomyces avermitilis MA-4680]
>gi|29828689|ref|NP_823323.1| putative DNA polymerase III epsilon subunit [Streptomyces avermitilis MA-4680]
>gi|29828692|ref|NP_823326.1| hypothetical protein SAV2150 [Streptomyces avermitilis MA-4680]
>gi|29828702|ref|NP_823336.1| putative transferase [Streptomyces avermitilis MA-4680]
>gi|29828706|ref|NP_823340.1| putative hydrolase [Streptomyces avermitilis MA-4680]
>gi|29828710|ref|NP_823344.1| putative secreted cellulose-binding protein [Streptomyces avermitilis MA-4680]
>gi|29828711|ref|NP_823345.1| hypothetical protein SAV2169 [Streptomyces avermitilis MA-4680]
>gi|29828720|ref|NP_823354.1| putative ABC transporter substrate-binding protein [Streptomyces avermitilis MA-4680]
>gi|29828721|ref|NP_823355.1| putative sensor-like histidine kinase [Streptomyces avermitilis MA-4680]
>gi|29828725|ref|NP_823359.1| putative ABC transporter permease [Streptomyces avermitilis MA-4680]
>gi|29828732|ref|NP_823366.1| hypothetical protein SAV2190 [Streptomyces avermitilis MA-4680]
>gi|29828735|ref|NP_823369.1| hypothetical protein SAV2193 [Streptomyces avermitilis MA-4680]
>gi|29828738|ref|NP_823372.1| putative two-component system sensor kinase [Streptomyces avermitilis MA-4680]
>gi|29828741|ref|NP_823375.1| hypothetical protein SAV2199 [Streptomyces avermitilis MA-4680]
>gi|29828743|ref|NP_823377.1| hypothetical protein SAV2201 [Streptomyces avermitilis MA-4680]
>gi|29828749|ref|NP_823383.1| putative hydrolase [Streptomyces avermitilis MA-4680]
>gi|29828751|ref|NP_823385.1| hypothetical protein SAV2209 [Streptomyces avermitilis MA-4680]

>gi|29828753|ref|NP_823387.1| putative sulfate transporter [Streptomyces avermitilis MA-4680]
>gi|29828760|ref|NP_823394.1| hypothetical protein SAV2218 [Streptomyces avermitilis MA-4680]
>gi|29828762|ref|NP_823396.1| putative integral membrane protein [Streptomyces avermitilis MA-4680]
>gi|29828765|ref|NP_823399.1| putative protoporphyrinogen oxidase [Streptomyces avermitilis MA-4680]
>gi|29828766|ref|NP_823400.1| putative lipoprotein [Streptomyces avermitilis MA-4680]
>gi|29828779|ref|NP_823413.1| hypothetical protein SAV2237 [Streptomyces avermitilis MA-4680]
>gi|29828783|ref|NP_823417.1| hypothetical protein SAV2241 [Streptomyces avermitilis MA-4680]
>gi|29828797|ref|NP_823431.1| hypothetical protein SAV2255 [Streptomyces avermitilis MA-4680]
>gi|29828804|ref|NP_823438.1| putative serine/threonine protein kinase [Streptomyces avermitilis MA-4680]
>gi|29828810|ref|NP_823444.1| putative TetR-family transcriptional regulator [Streptomyces avermitilis MA-4680]
>gi|29828822|ref|NP_823456.1| putative modular polyketide synthase [Streptomyces avermitilis MA-4680]
>gi|29828823|ref|NP_823457.1| putative modular polyketide synthase [Streptomyces avermitilis MA-4680]
>gi|29828833|ref|NP_823467.1| putative acyl carrier protein [Streptomyces avermitilis MA-4680]
>gi|29828844|ref|NP_823478.1| putative membrane protein [Streptomyces avermitilis MA-4680]
>gi|29828848|ref|NP_823482.1| putative acyl-CoA dehydrogenase [Streptomyces avermitilis MA-4680]
>gi|29828858|ref|NP_823492.1| putative enoyl-CoA hydratase [Streptomyces avermitilis MA-4680]
>gi|29828859|ref|NP_823493.1| putative regulatory protein [Streptomyces avermitilis MA-4680]
>gi|29828863|ref|NP_823497.1| hypothetical protein SAV2321 [Streptomyces avermitilis MA-4680]
>gi|29828868|ref|NP_823502.1| hypothetical protein SAV2326 [Streptomyces avermitilis MA-4680]
>gi|29828874|ref|NP_823508.1| putative ArsR-family transcriptional regulator [Streptomyces avermitilis MA-4680]
>gi|29828876|ref|NP_823510.1| hypothetical protein SAV2334 [Streptomyces avermitilis MA-4680]
>gi|29828877|ref|NP_823511.1| hypothetical protein SAV2335 [Streptomyces avermitilis MA-4680]
>gi|29828883|ref|NP_823517.1| putative molibdenum binding protein [Streptomyces avermitilis MA-4680]
>gi|29828884|ref|NP_823518.1| putative membrane protein [Streptomyces avermitilis MA-4680]
>gi|29828885|ref|NP_823519.1| putative membrane protein [Streptomyces avermitilis MA-4680]
>gi|29828886|ref|NP_823520.1| putative membrane protein [Streptomyces avermitilis MA-4680]
>gi|29828887|ref|NP_823521.1| putative dehydrogenase [Streptomyces avermitilis MA-4680]
>gi|29828888|ref|NP_823522.1| hypothetical protein SAV2346 [Streptomyces avermitilis MA-4680]
>gi|29828890|ref|NP_823524.1| putative gas vesicle synthesis protein [Streptomyces avermitilis MA-4680]
>gi|29828893|ref|NP_823527.1| hypothetical protein SAV2351 [Streptomyces avermitilis MA-4680]
>gi|29828894|ref|NP_823528.1| hypothetical protein SAV2352 [Streptomyces avermitilis MA-4680]
>gi|29828897|ref|NP_823531.1| putative gas vesicle synthesis protein [Streptomyces avermitilis MA-4680]
>gi|29828899|ref|NP_823533.1| putative membrane protein [Streptomyces avermitilis MA-4680]
>gi|29828901|ref|NP_823535.1| hypothetical protein SAV2359 [Streptomyces avermitilis MA-4680]
>gi|29828911|ref|NP_823545.1| putative regulatory protein [Streptomyces avermitilis MA-4680]
>gi|29828944|ref|NP_823578.1| hypothetical protein SAV2402 [Streptomyces avermitilis MA-4680]
>gi|29828950|ref|NP_823584.1| putative transmembrane efflux protein [Streptomyces avermitilis MA-4680]
>gi|29828952|ref|NP_823586.1| hypothetical protein SAV2410 [Streptomyces avermitilis MA-4680]
>gi|29828953|ref|NP_823587.1| putative DNA-binding protein [Streptomyces avermitilis MA-4680]
>gi|29828962|ref|NP_823596.1| putative peptidase [Streptomyces avermitilis MA-4680]
>gi|29828970|ref|NP_823604.1| putative citrate synthase-like protein [Streptomyces avermitilis MA-4680]
>gi|29828978|ref|NP_823612.1| putative sodium:solute symporter [Streptomyces avermitilis MA-4680]
>gi|29828981|ref|NP_823615.1| putative two-component system sensor kinase [Streptomyces avermitilis MA-4680]
>gi|29828982|ref|NP_823616.1| putative integral membrane protein [Streptomyces avermitilis MA-4680]
>gi|29828986|ref|NP_823620.1| putative RNA polymerase major sigma factor, sigma-70 family [Streptomyces avermitilis MA-4680]
>gi|29828990|ref|NP_823624.1| putative glycogen debranching enzyme [Streptomyces avermitilis MA-4680]
>gi|29828991|ref|NP_823625.1| hypothetical protein SAV2449 [Streptomyces avermitilis MA-4680]
>gi|29828994|ref|NP_823628.1| hypothetical protein SAV2452 [Streptomyces avermitilis MA-4680]
>gi|29828998|ref|NP_823632.1| hypothetical protein SAV2456 [Streptomyces avermitilis MA-4680]
>gi|29828999|ref|NP_823633.1| putative hydrolase [Streptomyces avermitilis MA-4680]
>gi|29829007|ref|NP_823641.1| hypothetical protein SAV2465 [Streptomyces avermitilis MA-4680]
>gi|29829010|ref|NP_823644.1| putative secreted protein [Streptomyces avermitilis MA-4680]
>gi|29829016|ref|NP_823650.1| putative membrane protein [Streptomyces avermitilis MA-4680]
>gi|29829018|ref|NP_823652.1| hypothetical protein SAV2476 [Streptomyces avermitilis MA-4680]
>gi|29829020|ref|NP_823654.1| hypothetical protein SAV2478 [Streptomyces avermitilis MA-4680]
>gi|29829022|ref|NP_823656.1| hypothetical protein SAV2480 [Streptomyces avermitilis MA-4680]
>gi|29829030|ref|NP_823664.1| putative monooxygenase [Streptomyces avermitilis MA-4680]
>gi|29829032|ref|NP_823666.1| hypothetical protein SAV2490 [Streptomyces avermitilis MA-4680]
>gi|29829033|ref|NP_823667.1| putative regulatory protein RecX [Streptomyces avermitilis MA-4680]
>gi|29829039|ref|NP_823673.1| putative membrane protein [Streptomyces avermitilis MA-4680]
>gi|29829040|ref|NP_823674.1| putative membrane protein [Streptomyces avermitilis MA-4680]
>gi|29829045|ref|NP_823679.1| hypothetical protein SAV2503 [Streptomyces avermitilis MA-4680]
>gi|29829047|ref|NP_823681.1| putative DNA-binding protein [Streptomyces avermitilis MA-4680]
>gi|29829049|ref|NP_823683.1| putative phosphatidylglycerophosphate synthase [Streptomyces avermitilis MA-4680]
>gi|29829061|ref|NP_823695.1| putative membrane protein [Streptomyces avermitilis MA-4680]
>gi|29829062|ref|NP_823696.1| putative membrane protein [Streptomyces avermitilis MA-4680]
>gi|29829069|ref|NP_823703.1| putative membrane protein [Streptomyces avermitilis MA-4680]
>gi|29829077|ref|NP_823711.1| hypothetical protein SAV2535 [Streptomyces avermitilis MA-4680]
>gi|29829086|ref|NP_823720.1| hypothetical protein SAV2544 [Streptomyces avermitilis MA-4680]
>gi|29829089|ref|NP_823723.1| hypothetical protein SAV2547 [Streptomyces avermitilis MA-4680]

>gi|29829093|ref|NP_823727.1| putative translation initiation factor IF-2 [Streptomyces avermitilis MA-4680]
>gi|29829097|ref|NP_823731.1| hypothetical protein SAV2555 [Streptomyces avermitilis MA-4680]
>gi|29829098|ref|NP_823732.1| hypothetical protein SAV2556 [Streptomyces avermitilis MA-4680]
>gi|29829118|ref|NP_823752.1| putative ABC transporter permease protein [Streptomyces avermitilis MA-4680]
>gi|29829122|ref|NP_823756.1| putative regulatory protein [Streptomyces avermitilis MA-4680]
>gi|29829123|ref|NP_823757.1| putative regulatory protein [Streptomyces avermitilis MA-4680]
>gi|29829124|ref|NP_823758.1| putative ATP/GTP-binding protein [Streptomyces avermitilis MA-4680]
>gi|29829126|ref|NP_823760.1| putative membrane protein [Streptomyces avermitilis MA-4680]
>gi|29829128|ref|NP_823762.1| hypothetical protein SAV2587 [Streptomyces avermitilis MA-4680]
>gi|29829139|ref|NP_823773.1| putative RNA polymerase ECF-subfamily sigma factor [Streptomyces avermitilis MA-4680]
>gi|29829141|ref|NP_823775.1| hypothetical protein SAV2599 [Streptomyces avermitilis MA-4680]
>gi|29829145|ref|NP_823779.1| putative multi-drug efflux transporter [Streptomyces avermitilis MA-4680]
>gi|29829147|ref|NP_823781.1| putative dioxygenase [Streptomyces avermitilis MA-4680]
>gi|29829149|ref|NP_823783.1| putative transposase [Streptomyces avermitilis MA-4680]
>gi|29829156|ref|NP_823790.1| hypothetical protein SAV2614 [Streptomyces avermitilis MA-4680]
>gi|29829159|ref|NP_823793.1| hypothetical protein SAV2617 [Streptomyces avermitilis MA-4680]
>gi|29829165|ref|NP_823799.1| putative phosphatidate cytidylyltransferase [Streptomyces avermitilis MA-4680]
>gi|29829169|ref|NP_823803.1| putative ribosomal protein S2 [Streptomyces avermitilis MA-4680]
>gi|29829170|ref|NP_823804.1| putative peptidase [Streptomyces avermitilis MA-4680]
>gi|29829175|ref|NP_823809.1| hypothetical protein SAV2633 [Streptomyces avermitilis MA-4680]
>gi|29829180|ref|NP_823814.1| putative signal peptidase [Streptomyces avermitilis MA-4680]
>gi|57833860|ref|NP_823825.2| putative protein P-II uridylyltransferase [Streptomyces avermitilis MA-4680]
>gi|29829195|ref|NP_823829.1| hypothetical protein SAV2653 [Streptomyces avermitilis MA-4680]
>gi|29829199|ref|NP_823833.1| putative L-arabinose permease [Streptomyces avermitilis MA-4680]
>gi|29829200|ref|NP_823834.1| putative chromosome segregation protein [Streptomyces avermitilis MA-4680]
>gi|29829202|ref|NP_823836.1| hypothetical protein SAV2660 [Streptomyces avermitilis MA-4680]
>gi|29829210|ref|NP_823844.1| hypothetical protein SAV2668 [Streptomyces avermitilis MA-4680]
>gi|29829215|ref|NP_823849.1| hypothetical protein SAV2673 [Streptomyces avermitilis MA-4680]
>gi|29829216|ref|NP_823850.1| putative dihydroxyacetone kinase [Streptomyces avermitilis MA-4680]
>gi|29829224|ref|NP_823858.1| hypothetical protein SAV2682 [Streptomyces avermitilis MA-4680]
>gi|29829226|ref|NP_823860.1| putative histone-like DNA-binding protein [Streptomyces avermitilis MA-4680]
>gi|29829229|ref|NP_823863.1| putative IclR-family transcriptional regulator [Streptomyces avermitilis MA-4680]
>gi|29829236|ref|NP_823870.1| putative sensor-like histidine kinase [Streptomyces avermitilis MA-4680]
>gi|29829242|ref|NP_823876.1| hypothetical protein SAV2700 [Streptomyces avermitilis MA-4680]
>gi|29829249|ref|NP_823883.1| hypothetical protein SAV2707 [Streptomyces avermitilis MA-4680]
>gi|29829267|ref|NP_823901.1| putative transcriptional regulator [Streptomyces avermitilis MA-4680]
>gi|29829270|ref|NP_823904.1| putative transmembrane efflux protein [Streptomyces avermitilis MA-4680]
>gi|29829271|ref|NP_823905.1| putative LuxR-family transcriptional regulator [Streptomyces avermitilis MA-4680]
>gi|29829280|ref|NP_823914.1| hypothetical protein SAV2738 [Streptomyces avermitilis MA-4680]
>gi|29829281|ref|NP_823915.1| putative LuxR-family transcriptional regulator [Streptomyces avermitilis MA-4680]
>gi|29829283|ref|NP_823917.1| putative membrane protein [Streptomyces avermitilis MA-4680]
>gi|29829289|ref|NP_823923.1| putative phosphodiesterase [Streptomyces avermitilis MA-4680]
>gi|29829299|ref|NP_823933.1| putative membrane protein [Streptomyces avermitilis MA-4680]
>gi|29829300|ref|NP_823934.1| putative membrane protein [Streptomyces avermitilis MA-4680]
>gi|29829302|ref|NP_823936.1| putative DeoR-family transcriptional regulator [Streptomyces avermitilis MA-4680]
>gi|29829307|ref|NP_823941.1| putative ABC transporter ATP-binding protein [Streptomyces avermitilis MA-4680]
>gi|29829313|ref|NP_823947.1| hypothetical protein SAV2771 [Streptomyces avermitilis MA-4680]
>gi|29829321|ref|NP_823955.1| hypothetical protein SAV2779 [Streptomyces avermitilis MA-4680]
>gi|29829324|ref|NP_823958.1| hypothetical protein SAV2782 [Streptomyces avermitilis MA-4680]
>gi|29829326|ref|NP_823960.1| putative polysaccharide deacetylase [Streptomyces avermitilis MA-4680]
>gi|29829334|ref|NP_823968.1| putative ABC transporter transmembrane protein [Streptomyces avermitilis MA-4680]
>gi|29829335|ref|NP_823969.1| putative ABC transporter ATP-binding protein [Streptomyces avermitilis MA-4680]
>gi|29829337|ref|NP_823971.1| putative neutral zinc metalloprotease [Streptomyces avermitilis MA-4680]
>gi|29829355|ref|NP_823989.1| putative transmembrane efflux protein [Streptomyces avermitilis MA-4680]
>gi|29829368|ref|NP_824002.1| hypothetical protein SAV2826 [Streptomyces avermitilis MA-4680]
>gi|29829369|ref|NP_824003.1| putative membrane protein [Streptomyces avermitilis MA-4680]
>gi|29829372|ref|NP_824006.1| putative thioredoxin [Streptomyces avermitilis MA-4680]
>gi|29829387|ref|NP_824021.1| putative TetR-family transcriptional regulator [Streptomyces avermitilis MA-4680]
>gi|29829396|ref|NP_824030.1| hypothetical protein SAV2854 [Streptomyces avermitilis MA-4680]
>gi|29829400|ref|NP_824034.1| putative M protein [Streptomyces avermitilis MA-4680]
>gi|29829401|ref|NP_824035.1| putative cellulose-binding protein [Streptomyces avermitilis MA-4680]
>gi|29829404|ref|NP_824038.1| putative ABC transporter ATP-binding protein [Streptomyces avermitilis MA-4680]
>gi|29829410|ref|NP_824044.1| hypothetical protein SAV2868 [Streptomyces avermitilis MA-4680]
>gi|29829415|ref|NP_824049.1| putative ABC transporter transmembrane protein [Streptomyces avermitilis MA-4680]
>gi|29829418|ref|NP_824052.1| putative two-component system sensor kinase [Streptomyces avermitilis MA-4680]
>gi|29829422|ref|NP_824056.1| putative F-type proton-transporting ATPase epsilon chain [Streptomyces avermitilis MA-4680]
>gi|29829426|ref|NP_824060.1| putative F-type proton-transporting ATPase delta chain [Streptomyces avermitilis MA-4680]
>gi|29829427|ref|NP_824061.1| putative F-type proton-transporting ATPase b chain [Streptomyces avermitilis MA-4680]
>gi|29829430|ref|NP_824064.1| putative ATP synthase protein I [Streptomyces avermitilis MA-4680]
>gi|29829434|ref|NP_824068.1| modular polyketide synthase [Streptomyces avermitilis MA-4680]

>gi|29829449|ref|NP_824083.1| putative teichoic acid linkage unit synthesis (synthesis of undecaprenylpyrophosphate-N- aetylglucosamine) [Streptomyces avermitilis MA-4680]
>gi|29829457|ref|NP_824091.1| putative transcription termination factor Rho [Streptomyces avermitilis MA-4680]
>gi|29829463|ref|NP_824097.1| putative two-component system response regulator [Streptomyces avermitilis MA-4680]
>gi|29829481|ref|NP_824115.1| putative secreted metalloprotease [Streptomyces avermitilis MA-4680]
>gi|29829487|ref|NP_824121.1| putative Ku70/Ku80 protein [Streptomyces avermitilis MA-4680]
>gi|29829491|ref|NP_824125.1| putative lipoprotein [Streptomyces avermitilis MA-4680]
>gi|29829492|ref|NP_824126.1| putative two-component system sensor kinase [Streptomyces avermitilis MA-4680]
>gi|29829493|ref|NP_824127.1| putative cell division membrane protein [Streptomyces avermitilis MA-4680]
>gi|29829498|ref|NP_824132.1| hypothetical protein SAV2956 [Streptomyces avermitilis MA-4680]
>gi|29829499|ref|NP_824133.1| putative NLP/P60-family secreted protein [Streptomyces avermitilis MA-4680]
>gi|29829504|ref|NP_824138.1| putative sensor-like histidine kinase [Streptomyces avermitilis MA-4680]
>gi|29829510|ref|NP_824144.1| hypothetical protein SAV2968 [Streptomyces avermitilis MA-4680]
>gi|29829518|ref|NP_824152.1| hypothetical protein SAV2976 [Streptomyces avermitilis MA-4680]
>gi|29829519|ref|NP_824153.1| putative PadR-like family transcriptional regulator [Streptomyces avermitilis MA-4680]
>gi|29829520|ref|NP_824154.1| hypothetical protein SAV2978 [Streptomyces avermitilis MA-4680]
>gi|29829534|ref|NP_824168.1| putative transport protein [Streptomyces avermitilis MA-4680]
>gi|29829539|ref|NP_824173.1| polyprenyl diphosphate synthase [Streptomyces avermitilis MA-4680]
>gi|29829546|ref|NP_824180.1| hypothetical protein SAV3004 [Streptomyces avermitilis MA-4680]
>gi|29829548|ref|NP_824182.1| putative polyprenyl diphosphate synthase [Streptomyces avermitilis MA-4680]
>gi|29829552|ref|NP_824186.1| putative sodium/proton antiporter [Streptomyces avermitilis MA-4680]
>gi|29829556|ref|NP_824190.1| putative integral membrane protein [Streptomyces avermitilis MA-4680]
>gi|29829561|ref|NP_824195.1| putative beta-N-acetylhexosaminidase [Streptomyces avermitilis MA-4680]
>gi|29829577|ref|NP_824211.1| putative lipoprotein [Streptomyces avermitilis MA-4680]
>gi|29829578|ref|NP_824212.1| hypothetical protein SAV3036 [Streptomyces avermitilis MA-4680]
>gi|29829583|ref|NP_824217.1| putative integral membrane protein [Streptomyces avermitilis MA-4680]
>gi|29829591|ref|NP_824225.1| putative TetR-family transcriptional regulator [Streptomyces avermitilis MA-4680]
>gi|29829596|ref|NP_824230.1| hypothetical protein SAV3054 [Streptomyces avermitilis MA-4680]
>gi|29829602|ref|NP_824236.1| hypothetical protein SAV3060 [Streptomyces avermitilis MA-4680]
>gi|29829607|ref|NP_824241.1| hypothetical protein SAV3065 [Streptomyces avermitilis MA-4680]
>gi|29829609|ref|NP_824243.1| hypothetical protein SAV3067 [Streptomyces avermitilis MA-4680]
>gi|29829611|ref|NP_824245.1| hypothetical protein SAV3069 [Streptomyces avermitilis MA-4680]
>gi|29829621|ref|NP_824255.1| putative integral membrane protein [Streptomyces avermitilis MA-4680]
>gi|29829627|ref|NP_824261.1| putative membrane protein [Streptomyces avermitilis MA-4680]
>gi|29829628|ref|NP_824262.1| putative glycosyltransferase [Streptomyces avermitilis MA-4680]
>gi|29829629|ref|NP_824263.1| putative ABC transporter ATP-binding protein [Streptomyces avermitilis MA-4680]
>gi|29829631|ref|NP_824265.1| putative ABC transporter system integral membrane protein [Streptomyces avermitilis MA-4680]
>gi|29829633|ref|NP_824267.1| hypothetical protein SAV3091 [Streptomyces avermitilis MA-4680]
>gi|29829639|ref|NP_824273.1| hypothetical protein SAV3097 [Streptomyces avermitilis MA-4680]
>gi|29829640|ref|NP_824274.1| putative ATP-dependent RNA helicase [Streptomyces avermitilis MA-4680]
>gi|29829644|ref|NP_824278.1| putative integral membrane protein [Streptomyces avermitilis MA-4680]
>gi|29829657|ref|NP_824291.1| putative serine protease [Streptomyces avermitilis MA-4680]
>gi|29829658|ref|NP_824292.1| hypothetical protein SAV3116 [Streptomyces avermitilis MA-4680]
>gi|29829662|ref|NP_824296.1| putative enoyl-CoA hydratase/isomerase [Streptomyces avermitilis MA-4680]
>gi|29829664|ref|NP_824298.1| putative secreted protein [Streptomyces avermitilis MA-4680]
>gi|29829668|ref|NP_824302.1| hypothetical protein SAV3126 [Streptomyces avermitilis MA-4680]
>gi|29829669|ref|NP_824303.1| putative ATP-binding protein [Streptomyces avermitilis MA-4680]
>gi|29829672|ref|NP_824306.1| putative acetyltransferase [Streptomyces avermitilis MA-4680]
>gi|29829674|ref|NP_824308.1| putative two-component system response regulator [Streptomyces avermitilis MA-4680]
>gi|29829678|ref|NP_824312.1| hypothetical protein SAV3136 [Streptomyces avermitilis MA-4680]
>gi|29829679|ref|NP_824313.1| putative integral membrane protein [Streptomyces avermitilis MA-4680]
>gi|29829681|ref|NP_824315.1| hypothetical protein SAV3139 [Streptomyces avermitilis MA-4680]
>gi|29829725|ref|NP_824359.1| putative membrane protein [Streptomyces avermitilis MA-4680]
>gi|29829726|ref|NP_824360.1| hypothetical protein SAV3184 [Streptomyces avermitilis MA-4680]
>gi|29829729|ref|NP_824363.1| putative mutT-like protein [Streptomyces avermitilis MA-4680]
>gi|29829738|ref|NP_824372.1| hypothetical protein SAV3196 [Streptomyces avermitilis MA-4680]
>gi|29829750|ref|NP_824384.1| hypothetical protein SAV3208 [Streptomyces avermitilis MA-4680]
>gi|29829755|ref|NP_824389.1| putative NADH dehydrogenase/NAD(P)H nitroreductase [Streptomyces avermitilis MA-4680]
>gi|29829761|ref|NP_824395.1| hypothetical protein SAV3219 [Streptomyces avermitilis MA-4680]
>gi|29829765|ref|NP_824399.1| putative magnesium or manganese-dependent protein phosphatase [Streptomyces avermitilis MA-4680]
>gi|29829768|ref|NP_824402.1| putative integral membrane protein [Streptomyces avermitilis MA-4680]
>gi|29829773|ref|NP_824407.1| putative hydrogen peroxide sensing regulator, LysR-family transcriptional regulator [Streptomyces avermitilis MA-4680]
>gi|29829776|ref|NP_824410.1| putative integral membrane protein [Streptomyces avermitilis MA-4680]
>gi|29829777|ref|NP_824411.1| hypothetical protein SAV3235 [Streptomyces avermitilis MA-4680]
>gi|29829789|ref|NP_824423.1| hypothetical protein SAV3247 [Streptomyces avermitilis MA-4680]
>gi|29829790|ref|NP_824424.1| hypothetical protein SAV3248 [Streptomyces avermitilis MA-4680]
>gi|29829794|ref|NP_824428.1| hypothetical protein SAV3252 [Streptomyces avermitilis MA-4680]
>gi|29829797|ref|NP_824431.1| putative septum site-determining protein [Streptomyces avermitilis MA-4680]
>gi|29829800|ref|NP_824434.1| putative chitinase A [Streptomyces avermitilis MA-4680]

>gi|29829804|ref|NP_824438.1| putative regulatory protein [Streptomyces avermitilis MA-4680]
>gi|29829807|ref|NP_824441.1| hypothetical protein SAV3265 [Streptomyces avermitilis MA-4680]
>gi|29829809|ref|NP_824443.1| hypothetical protein SAV3267 [Streptomyces avermitilis MA-4680]
>gi|29829811|ref|NP_824445.1| hypothetical protein SAV3269 [Streptomyces avermitilis MA-4680]
>gi|29829814|ref|NP_824448.1| hypothetical protein SAV3272 [Streptomyces avermitilis MA-4680]
>gi|29829817|ref|NP_824451.1| hypothetical protein SAV3275 [Streptomyces avermitilis MA-4680]
>gi|29829826|ref|NP_824460.1| hypothetical protein SAV3284 [Streptomyces avermitilis MA-4680]
>gi|29829828|ref|NP_824462.1| hypothetical protein SAV3286 [Streptomyces avermitilis MA-4680]
>gi|29829831|ref|NP_824465.1| putative integral membrane efflux protein [Streptomyces avermitilis MA-4680]
>gi|29829840|ref|NP_824474.1| putative membrane protein [Streptomyces avermitilis MA-4680]
>gi|29829853|ref|NP_824487.1| hypothetical protein SAV3311 [Streptomyces avermitilis MA-4680]
>gi|29829854|ref|NP_824488.1| hypothetical protein SAV3312 [Streptomyces avermitilis MA-4680]
>gi|29829855|ref|NP_824489.1| putative iron-siderophore uptake system transmembrane component [Streptomyces avermitilis MA-4680]
>gi|29829863|ref|NP_824497.1| putative integral membrane protein [Streptomyces avermitilis MA-4680]
>gi|29829866|ref|NP_824500.1| hypothetical protein SAV3324 [Streptomyces avermitilis MA-4680]
>gi|29829870|ref|NP_824504.1| hypothetical protein SAV3328 [Streptomyces avermitilis MA-4680]
>gi|29829874|ref|NP_824508.1| hypothetical protein SAV3332 [Streptomyces avermitilis MA-4680]
>gi|29829878|ref|NP_824512.1| putative integral membrane protein [Streptomyces avermitilis MA-4680]
>gi|29829880|ref|NP_824514.1| hypothetical protein SAV3338 [Streptomyces avermitilis MA-4680]
>gi|29829886|ref|NP_824520.1| hypothetical protein SAV3344 [Streptomyces avermitilis MA-4680]
>gi|29829891|ref|NP_824525.1| hypothetical protein SAV3349 [Streptomyces avermitilis MA-4680]
>gi|29829897|ref|NP_824531.1| putative integral membrane protein [Streptomyces avermitilis MA-4680]
>gi|29829899|ref|NP_824533.1| putative secreted protein [Streptomyces avermitilis MA-4680]
>gi|29829903|ref|NP_824537.1| putative transport integral membrane protein [Streptomyces avermitilis MA-4680]
>gi|29829906|ref|NP_824540.1| hypothetical protein SAV3364 [Streptomyces avermitilis MA-4680]
>gi|29829912|ref|NP_824546.1| putative lipoprotein [Streptomyces avermitilis MA-4680]
>gi|29829924|ref|NP_824558.1| hypothetical protein SAV3381 [Streptomyces avermitilis MA-4680]
>gi|29829927|ref|NP_824561.1| hypothetical protein SAV3384 [Streptomyces avermitilis MA-4680]
>gi|29829928|ref|NP_824562.1| putative RNA polymerase ECF-subfamily sigma factor [Streptomyces avermitilis MA-4680]
>gi|29829929|ref|NP_824563.1| hypothetical protein SAV3386 [Streptomyces avermitilis MA-4680]
>gi|29829933|ref|NP_824567.1| hypothetical protein SAV3390 [Streptomyces avermitilis MA-4680]
>gi|29829935|ref|NP_824569.1| hypothetical protein SAV3392 [Streptomyces avermitilis MA-4680]
>gi|29829937|ref|NP_824571.1| hypothetical protein SAV3394 [Streptomyces avermitilis MA-4680]
>gi|29829942|ref|NP_824576.1| hypothetical protein SAV3399 [Streptomyces avermitilis MA-4680]
>gi|29829944|ref|NP_824578.1| hypothetical protein SAV3401 [Streptomyces avermitilis MA-4680]
>gi|29829954|ref|NP_824588.1| hypothetical protein SAV3411 [Streptomyces avermitilis MA-4680]
>gi|29829956|ref|NP_824590.1| putative D-alanyl-D-alanine carboxypeptidase [Streptomyces avermitilis MA-4680]
>gi|29829965|ref|NP_824599.1| hypothetical protein SAV3422 [Streptomyces avermitilis MA-4680]
>gi|29829966|ref|NP_824600.1| putative integral membrane protein [Streptomyces avermitilis MA-4680]
>gi|29829969|ref|NP_824603.1| hypothetical protein SAV3426 [Streptomyces avermitilis MA-4680]
>gi|29829974|ref|NP_824608.1| putative glycine betaine ABC transporter integral membrane protein [Streptomyces avermitilis MA-4680]
>gi|29829978|ref|NP_824612.1| hypothetical protein SAV3435 [Streptomyces avermitilis MA-4680]
>gi|29829980|ref|NP_824614.1| putative membrane protein [Streptomyces avermitilis MA-4680]
>gi|29829981|ref|NP_824615.1| putative membrane protein [Streptomyces avermitilis MA-4680]
>gi|29829983|ref|NP_824617.1| putative membrane protein [Streptomyces avermitilis MA-4680]
>gi|29829984|ref|NP_824618.1| hypothetical protein SAV3441 [Streptomyces avermitilis MA-4680]
>gi|29829986|ref|NP_824620.1| hypothetical protein SAV3443 [Streptomyces avermitilis MA-4680]
>gi|29829989|ref|NP_824623.1| hypothetical protein SAV3446 [Streptomyces avermitilis MA-4680]
>gi|29829990|ref|NP_824624.1| hypothetical protein SAV3447 [Streptomyces avermitilis MA-4680]
>gi|29829991|ref|NP_824625.1| hypothetical protein SAV3448 [Streptomyces avermitilis MA-4680]
>gi|29829992|ref|NP_824626.1| putative integral membrane protein [Streptomyces avermitilis MA-4680]
>gi|29829993|ref|NP_824627.1| hypothetical protein SAV3450 [Streptomyces avermitilis MA-4680]
>gi|29829995|ref|NP_824629.1| putative succinyl-CoA synthetase beta subunit [Streptomyces avermitilis MA-4680]
>gi|29829996|ref|NP_824630.1| hypothetical protein SAV3453 [Streptomyces avermitilis MA-4680]
>gi|29829997|ref|NP_824631.1| hypothetical protein SAV3454 [Streptomyces avermitilis MA-4680]
>gi|29829999|ref|NP_824633.1| hypothetical protein SAV3456 [Streptomyces avermitilis MA-4680]
>gi|29830001|ref|NP_824635.1| hypothetical protein SAV3458 [Streptomyces avermitilis MA-4680]
>gi|29830002|ref|NP_824636.1| hypothetical protein SAV3459 [Streptomyces avermitilis MA-4680]
>gi|29830005|ref|NP_824639.1| putative peptidase [Streptomyces avermitilis MA-4680]
>gi|29830007|ref|NP_824641.1| putative NLP/P60-family protein [Streptomyces avermitilis MA-4680]
>gi|29830008|ref|NP_824642.1| putative integral membrane protein [Streptomyces avermitilis MA-4680]
>gi|29830010|ref|NP_824644.1| putative NLP/P60-family secreted protein [Streptomyces avermitilis MA-4680]
>gi|29830011|ref|NP_824645.1| hypothetical protein SAV3468 [Streptomyces avermitilis MA-4680]
>gi|29830012|ref|NP_824646.1| hypothetical protein SAV3469 [Streptomyces avermitilis MA-4680]
>gi|29830015|ref|NP_824649.1| hypothetical protein SAV3472 [Streptomyces avermitilis MA-4680]
>gi|29830017|ref|NP_824651.1| hypothetical protein SAV3474 [Streptomyces avermitilis MA-4680]
>gi|29830018|ref|NP_824652.1| putative membrane protein [Streptomyces avermitilis MA-4680]
>gi|29830027|ref|NP_824661.1| hypothetical protein SAV3484 [Streptomyces avermitilis MA-4680]
>gi|29830028|ref|NP_824662.1| hypothetical protein SAV3485 [Streptomyces avermitilis MA-4680]
>gi|29830029|ref|NP_824663.1| hypothetical protein SAV3486 [Streptomyces avermitilis MA-4680]

>gi|29830030|ref|NP_824664.1| hypothetical protein SAV3487 [Streptomyces avermitilis MA-4680]
>gi|29830031|ref|NP_824665.1| putative regulator protein containing histidine kinase domain [Streptomyces avermitilis MA-4680]
>gi|29830034|ref|NP_824668.1| hypothetical protein SAV3491 [Streptomyces avermitilis MA-4680]
>gi|29830040|ref|NP_824674.1| putative lysine/ornithine decarboxylase [Streptomyces avermitilis MA-4680]
>gi|29830042|ref|NP_824676.1| hypothetical protein SAV3499 [Streptomyces avermitilis MA-4680]
>gi|29830043|ref|NP_824677.1| hypothetical protein SAV3500 [Streptomyces avermitilis MA-4680]
>gi|29830045|ref|NP_824679.1| putative integral membrane protein [Streptomyces avermitilis MA-4680]
>gi|29830050|ref|NP_824684.1| putative ABC transporter permease [Streptomyces avermitilis MA-4680]
>gi|29830056|ref|NP_824690.1| putative secreted protein [Streptomyces avermitilis MA-4680]
>gi|29830057|ref|NP_824691.1| hypothetical protein SAV3514 [Streptomyces avermitilis MA-4680]
>gi|29830059|ref|NP_824693.1| putative integral membrane protein [Streptomyces avermitilis MA-4680]
>gi|29830061|ref|NP_824695.1| hypothetical protein SAV3518 [Streptomyces avermitilis MA-4680]
>gi|29830065|ref|NP_824699.1| putative phosphoenolpyruvate synthase [Streptomyces avermitilis MA-4680]
>gi|29830075|ref|NP_824709.1| putative membrane protein [Streptomyces avermitilis MA-4680]
>gi|29830078|ref|NP_824712.1| putative secreted protein [Streptomyces avermitilis MA-4680]
>gi|29830084|ref|NP_824718.1| putative integral membrane protein [Streptomyces avermitilis MA-4680]
>gi|29830085|ref|NP_824719.1| putative type II restriction-modification system DNA adenine-specific methylase [Streptomyces avermitilis MA-4680]
>gi|29830088|ref|NP_824722.1| hypothetical protein SAV3545 [Streptomyces avermitilis MA-4680]
>gi|29830091|ref|NP_824725.1| putative MerR-family transcriptional regulator [Streptomyces avermitilis MA-4680]
>gi|29830096|ref|NP_824730.1| hypothetical protein SAV3553 [Streptomyces avermitilis MA-4680]
>gi|29830098|ref|NP_824732.1| putative cellulose-binding protein [Streptomyces avermitilis MA-4680]
>gi|29830099|ref|NP_824733.1| hypothetical protein SAV3556 [Streptomyces avermitilis MA-4680]
>gi|29830102|ref|NP_824736.1| hypothetical protein SAV3559 [Streptomyces avermitilis MA-4680]
>gi|29830103|ref|NP_824737.1| putative two-component system sensor kinase [Streptomyces avermitilis MA-4680]
>gi|29830108|ref|NP_824742.1| hypothetical protein SAV3565 [Streptomyces avermitilis MA-4680]
>gi|29830114|ref|NP_824748.1| putative MarR-family transcriptional regulator [Streptomyces avermitilis MA-4680]
>gi|29830115|ref|NP_824749.1| putative two-component system response regulator [Streptomyces avermitilis MA-4680]
>gi|29830122|ref|NP_824756.1| hypothetical protein SAV3579 [Streptomyces avermitilis MA-4680]
>gi|29830123|ref|NP_824757.1| hypothetical protein SAV3580 [Streptomyces avermitilis MA-4680]
>gi|29830126|ref|NP_824760.1| putative membrane protein [Streptomyces avermitilis MA-4680]
>gi|29830129|ref|NP_824763.1| putative 4-(cytidine-5'-diphospho)-2-C-methyl-D- erythritol kinase [Streptomyces avermitilis MA-4680]
>gi|29830130|ref|NP_824764.1| putative dimethyladenosine transferase [Streptomyces avermitilis MA-4680]
>gi|29830140|ref|NP_824774.1| hypothetical protein SAV3597 [Streptomyces avermitilis MA-4680]
>gi|29830145|ref|NP_824779.1| hypothetical protein SAV3602 [Streptomyces avermitilis MA-4680]
>gi|29830150|ref|NP_824784.1| putative ABC-type cobalt transport system, permease component [Streptomyces avermitilis MA-4680]
>gi|29830156|ref|NP_824790.1| putative integral membrane protein [Streptomyces avermitilis MA-4680]
>gi|29830158|ref|NP_824792.1| hypothetical protein SAV3615 [Streptomyces avermitilis MA-4680]
>gi|29830165|ref|NP_824799.1| hypothetical protein SAV3622 [Streptomyces avermitilis MA-4680]
>gi|29830169|ref|NP_824803.1| putative MerR-family transcriptional regulator [Streptomyces avermitilis MA-4680]
>gi|29830174|ref|NP_824808.1| putative two-component system sensor kinase [Streptomyces avermitilis MA-4680]
>gi|29830175|ref|NP_824809.1| putative regulatory protein [Streptomyces avermitilis MA-4680]
>gi|29830176|ref|NP_824810.1| hypothetical protein SAV3633 [Streptomyces avermitilis MA-4680]
>gi|29830178|ref|NP_824812.1| putative ABC transporter integral membrane protein [Streptomyces avermitilis MA-4680]
>gi|29830183|ref|NP_824817.1| putative multidrug resistance protein [Streptomyces avermitilis MA-4680]
>gi|29830187|ref|NP_824821.1| putative MbtH-like protein [Streptomyces avermitilis MA-4680]
>gi|29830188|ref|NP_824822.1| putative ornithine cyclodeaminase [Streptomyces avermitilis MA-4680]
>gi|29830193|ref|NP_824827.1| putative acyl-CoA dehydrogenase [Streptomyces avermitilis MA-4680]
>gi|29830194|ref|NP_824828.1| putative non-ribosomal peptide synthetase/acyl-CoA dehydrogenase fusion protein [Streptomyces avermitilis MA-4680]
>gi|29830196|ref|NP_824830.1| putative 3-oxoacyl-ACP reductase [Streptomyces avermitilis MA-4680]
>gi|29830198|ref|NP_824832.1| putative dehydratase [Streptomyces avermitilis MA-4680]
>gi|29830199|ref|NP_824833.1| putative acyl carrier protein [Streptomyces avermitilis MA-4680]
>gi|29830200|ref|NP_824834.1| putative 3-oxoacyl-ACP synthase I [Streptomyces avermitilis MA-4680]
>gi|29830202|ref|NP_824836.1| putative 3-oxoacyl-ACP synthase II [Streptomyces avermitilis MA-4680]
>gi|29830203|ref|NP_824837.1| putative 3-oxoacyl-ACP synthase I [Streptomyces avermitilis MA-4680]
>gi|29830206|ref|NP_824840.1| putative 3-oxoacyl-ACP synthase I [Streptomyces avermitilis MA-4680]
>gi|29830211|ref|NP_824845.1| hypothetical protein SAV3668 [Streptomyces avermitilis MA-4680]
>gi|29830217|ref|NP_824851.1| putative ligase [Streptomyces avermitilis MA-4680]
>gi|29830220|ref|NP_824854.1| putative ABC transporter, permease protein [Streptomyces avermitilis MA-4680]
>gi|29830221|ref|NP_824855.1| hypothetical protein SAV3678 [Streptomyces avermitilis MA-4680]
>gi|29830223|ref|NP_824857.1| hypothetical protein SAV3680 [Streptomyces avermitilis MA-4680]
>gi|29830227|ref|NP_824861.1| hypothetical protein SAV3684 [Streptomyces avermitilis MA-4680]
>gi|29830230|ref|NP_824864.1| hypothetical protein SAV3687 [Streptomyces avermitilis MA-4680]
>gi|29830231|ref|NP_824865.1| putative fructose-specific permease [Streptomyces avermitilis MA-4680]
>gi|29830232|ref|NP_824866.1| putative 1-phosphofructokinase [Streptomyces avermitilis MA-4680]
>gi|29830236|ref|NP_824870.1| putative TetR-family transcriptional regulator [Streptomyces avermitilis MA-4680]
>gi|29830241|ref|NP_824875.1| putative transmembrane efflux protein [Streptomyces avermitilis MA-4680]
>gi|29830246|ref|NP_824880.1| putative TetR-family transcriptional regulator [Streptomyces avermitilis MA-4680]
>gi|29830249|ref|NP_824883.1| putative acyl-CoA oxidase [Streptomyces avermitilis MA-4680]

>gi|29830257|ref|NP_824891.1| hypothetical protein SAV3714 [Streptomyces avermitilis MA-4680]
>gi|29830260|ref|NP_824894.1| hypothetical protein SAV3717 [Streptomyces avermitilis MA-4680]
>gi|29830262|ref|NP_824896.1| hypothetical protein SAV3719 [Streptomyces avermitilis MA-4680]
>gi|29830273|ref|NP_824907.1| putative SpdD protein [Streptomyces avermitilis MA-4680]
>gi|29830276|ref|NP_824910.1| putative mobile element transfer protein SpdA [Streptomyces avermitilis MA-4680]
>gi|29830291|ref|NP_824925.1| hypothetical protein SAV3748 [Streptomyces avermitilis MA-4680]
>gi|29830296|ref|NP_824930.1| hypothetical protein SAV3753 [Streptomyces avermitilis MA-4680]
>gi|29830298|ref|NP_824932.1| putative low molecular weight protein tyrosine phosphatase [Streptomyces avermitilis MA-4680]
>gi|29830302|ref|NP_824936.1| hypothetical protein SAV3759 [Streptomyces avermitilis MA-4680]
>gi|29830306|ref|NP_824940.1| putative LysE-family efflux protein [Streptomyces avermitilis MA-4680]
>gi|29830314|ref|NP_824948.1| hypothetical protein SAV3771 [Streptomyces avermitilis MA-4680]
>gi|29830315|ref|NP_824949.1| hypothetical protein SAV3772 [Streptomyces avermitilis MA-4680]
>gi|29830317|ref|NP_824951.1| hypothetical protein SAV3774 [Streptomyces avermitilis MA-4680]
>gi|29830319|ref|NP_824953.1| putative integral membrane protein [Streptomyces avermitilis MA-4680]
>gi|29830324|ref|NP_824958.1| putative D-alanyl-D-alanine carboxypeptidase [Streptomyces avermitilis MA-4680]
>gi|29830325|ref|NP_824959.1| hypothetical protein SAV3782 [Streptomyces avermitilis MA-4680]
>gi|29830336|ref|NP_824970.1| hypothetical protein SAV3793 [Streptomyces avermitilis MA-4680]
>gi|29830340|ref|NP_824974.1| hypothetical protein SAV3797 [Streptomyces avermitilis MA-4680]
>gi|29830341|ref|NP_824975.1| putative aldehyde dehydrogenase [Streptomyces avermitilis MA-4680]
>gi|29830347|ref|NP_824981.1| putative transcriptional regulator [Streptomyces avermitilis MA-4680]
>gi|29830348|ref|NP_824982.1| hypothetical protein SAV3805 [Streptomyces avermitilis MA-4680]
>gi|29830354|ref|NP_824988.1| hypothetical protein SAV3811 [Streptomyces avermitilis MA-4680]
>gi|29830355|ref|NP_824989.1| hypothetical protein SAV3812 [Streptomyces avermitilis MA-4680]
>gi|29830364|ref|NP_824998.1| putative acyl-CoA dehydrogenase [Streptomyces avermitilis MA-4680]
>gi|29830369|ref|NP_825003.1| hypothetical protein SAV3826 [Streptomyces avermitilis MA-4680]
>gi|29830378|ref|NP_825012.1| putative dehydrogenase [Streptomyces avermitilis MA-4680]
>gi|29830381|ref|NP_825015.1| putative acyl-CoA dehydrogenase [Streptomyces avermitilis MA-4680]
>gi|29830385|ref|NP_825019.1| putative calcium binding protein [Streptomyces avermitilis MA-4680]
>gi|29830386|ref|NP_825020.1| putative anti-sigma factor antagonist [Streptomyces avermitilis MA-4680]
>gi|29830388|ref|NP_825022.1| hypothetical protein SAV3845 [Streptomyces avermitilis MA-4680]
>gi|29830392|ref|NP_825026.1| hypothetical protein SAV3849 [Streptomyces avermitilis MA-4680]
>gi|29830396|ref|NP_825030.1| putative integral membrane transport protein [Streptomyces avermitilis MA-4680]
>gi|29830404|ref|NP_825038.1| hypothetical protein SAV3861 [Streptomyces avermitilis MA-4680]
>gi|29830414|ref|NP_825048.1| putative integral membrane protein [Streptomyces avermitilis MA-4680]
>gi|29830415|ref|NP_825049.1| putative ABC transporter ATP-binding protein [Streptomyces avermitilis MA-4680]
>gi|29830416|ref|NP_825050.1| hypothetical protein SAV3873 [Streptomyces avermitilis MA-4680]
>gi|29830420|ref|NP_825054.1| putative sensor-like histidine kinase [Streptomyces avermitilis MA-4680]
>gi|29830422|ref|NP_825056.1| hypothetical protein SAV3879 [Streptomyces avermitilis MA-4680]
>gi|29830427|ref|NP_825061.1| hypothetical protein SAV3884 [Streptomyces avermitilis MA-4680]
>gi|29830429|ref|NP_825063.1| hypothetical protein SAV3886 [Streptomyces avermitilis MA-4680]
>gi|29830430|ref|NP_825064.1| putative transmembrane efflux protein [Streptomyces avermitilis MA-4680]
>gi|29830431|ref|NP_825065.1| putative RNA polymerase ECF-subfamily sigma factor [Streptomyces avermitilis MA-4680]
>gi|29830434|ref|NP_825068.1| putative quinone oxidoreductase [Streptomyces avermitilis MA-4680]
>gi|29830444|ref|NP_825078.1| hypothetical protein SAV3901 [Streptomyces avermitilis MA-4680]
>gi|29830446|ref|NP_825080.1| putative integral membrane protein [Streptomyces avermitilis MA-4680]
>gi|29830448|ref|NP_825082.1| hypothetical protein SAV3905 [Streptomyces avermitilis MA-4680]
>gi|29830451|ref|NP_825085.1| putative hydrolase [Streptomyces avermitilis MA-4680]
>gi|29830452|ref|NP_825086.1| hypothetical protein SAV3909 [Streptomyces avermitilis MA-4680]
>gi|29830454|ref|NP_825088.1| hypothetical protein SAV3911 [Streptomyces avermitilis MA-4680]
>gi|29830456|ref|NP_825090.1| hypothetical protein SAV3913 [Streptomyces avermitilis MA-4680]
>gi|29830460|ref|NP_825094.1| putative homeostasis protein [Streptomyces avermitilis MA-4680]
>gi|29830463|ref|NP_825097.1| putative phytoene synthase [Streptomyces avermitilis MA-4680]
>gi|29830465|ref|NP_825099.1| hypothetical protein SAV3922 [Streptomyces avermitilis MA-4680]
>gi|29830466|ref|NP_825100.1| putative RpiR-family transcriptional regulator [Streptomyces avermitilis MA-4680]
>gi|29830468|ref|NP_825102.1| putative PTS sucrose-specific enzyme IIBC component [Streptomyces avermitilis MA-4680]
>gi|29830480|ref|NP_825114.1| putative trehalose-6-phosphatase [Streptomyces avermitilis MA-4680]
>gi|29830483|ref|NP_825117.1| putative ROK-family transcriptional regulator [Streptomyces avermitilis MA-4680]
>gi|29830486|ref|NP_825120.1| putative dimeric protein [Streptomyces avermitilis MA-4680]
>gi|29830497|ref|NP_825131.1| hypothetical protein SAV3954 [Streptomyces avermitilis MA-4680]
>gi|29830508|ref|NP_825142.1| putative integral membrane protein [Streptomyces avermitilis MA-4680]
>gi|29830514|ref|NP_825148.1| putative lipoprotein [Streptomyces avermitilis MA-4680]
>gi|29830518|ref|NP_825152.1| hypothetical protein SAV3975 [Streptomyces avermitilis MA-4680]
>gi|29830526|ref|NP_825160.1| putative SpdD protein [Streptomyces avermitilis MA-4680]
>gi|29830529|ref|NP_825163.1| putative mobile element transfer protein SpdA [Streptomyces avermitilis MA-4680]
>gi|29830530|ref|NP_825164.1| putative plasmid transfer protein [Streptomyces avermitilis MA-4680]
>gi|29830541|ref|NP_825175.1| hypothetical protein SAV3998 [Streptomyces avermitilis MA-4680]
>gi|29830542|ref|NP_825176.1| hypothetical protein SAV3999 [Streptomyces avermitilis MA-4680]
>gi|29830545|ref|NP_825179.1| hypothetical protein SAV4002 [Streptomyces avermitilis MA-4680]
>gi|29830546|ref|NP_825180.1| putative AraC-family transcriptional regulator [Streptomyces avermitilis MA-4680]
>gi|29830551|ref|NP_825185.1| putative NLP/P60-family secreted protein [Streptomyces avermitilis MA-4680]

>gi|29830554|ref|NP_825188.1| hypothetical protein SAV4011 [Streptomyces avermitilis MA-4680]
>gi|29830556|ref|NP_825190.1| putative integral membrane protein [Streptomyces avermitilis MA-4680]
>gi|29830559|ref|NP_825193.1| putative ATP/GTP-binding protein [Streptomyces avermitilis MA-4680]
>gi|29830560|ref|NP_825194.1| putative TetR-family transcriptional regulator [Streptomyces avermitilis MA-4680]
>gi|29830561|ref|NP_825195.1| putative dehydrogenase [Streptomyces avermitilis MA-4680]
>gi|29830569|ref|NP_825203.1| putative AmfC protein [Streptomyces avermitilis MA-4680]
>gi|29830576|ref|NP_825210.1| hypothetical protein SAV4033 [Streptomyces avermitilis MA-4680]
>gi|29830582|ref|NP_825216.1| putative integral membrane protein [Streptomyces avermitilis MA-4680]
>gi|29830583|ref|NP_825217.1| putative molybdopterin converting factor [Streptomyces avermitilis MA-4680]
>gi|29830588|ref|NP_825222.1| putative serine protease [Streptomyces avermitilis MA-4680]
>gi|29830589|ref|NP_825223.1| hypothetical protein SAV4046 [Streptomyces avermitilis MA-4680]
>gi|29830595|ref|NP_825229.1| putative transmembrane protein [Streptomyces avermitilis MA-4680]
>gi|29830597|ref|NP_825231.1| hypothetical protein SAV4054 [Streptomyces avermitilis MA-4680]
>gi|29830602|ref|NP_825236.1| putative ABC transporter transmembrane protein [Streptomyces avermitilis MA-4680]
>gi|29830603|ref|NP_825237.1| putative ABC transporter transmembrane protein [Streptomyces avermitilis MA-4680]
>gi|29830605|ref|NP_825239.1| hypothetical protein SAV4062 [Streptomyces avermitilis MA-4680]
>gi|29830606|ref|NP_825240.1| putative integral membrane protein [Streptomyces avermitilis MA-4680]
>gi|29830607|ref|NP_825241.1| putative RNA polymerase ECF-subfamily sigma factor [Streptomyces avermitilis MA-4680]
>gi|29830610|ref|NP_825244.1| putative membrane protein [Streptomyces avermitilis MA-4680]
>gi|29830613|ref|NP_825247.1| hypothetical protein SAV4070 [Streptomyces avermitilis MA-4680]
>gi|29830623|ref|NP_825257.1| putative lipoprotein [Streptomyces avermitilis MA-4680]
>gi|29830631|ref|NP_825265.1| hypothetical protein SAV4088 [Streptomyces avermitilis MA-4680]
>gi|29830637|ref|NP_825271.1| hypothetical protein SAV4094 [Streptomyces avermitilis MA-4680]
>gi|29830638|ref|NP_825272.1| hypothetical protein SAV4095 [Streptomyces avermitilis MA-4680]
>gi|29830643|ref|NP_825277.1| putative integral membrane protein [Streptomyces avermitilis MA-4680]
>gi|29830646|ref|NP_825280.1| putative membrane protein [Streptomyces avermitilis MA-4680]
>gi|29830649|ref|NP_825283.1| putative membrane protein [Streptomyces avermitilis MA-4680]
>gi|29830655|ref|NP_825289.1| hypothetical protein SAV4112 [Streptomyces avermitilis MA-4680]
>gi|29830661|ref|NP_825295.1| putative peptidase [Streptomyces avermitilis MA-4680]
>gi|29830666|ref|NP_825300.1| putative ATP-dependent RNA helicase [Streptomyces avermitilis MA-4680]
>gi|29830667|ref|NP_825301.1| hypothetical protein SAV4124 [Streptomyces avermitilis MA-4680]
>gi|29830669|ref|NP_825303.1| putative membrane protein [Streptomyces avermitilis MA-4680]
>gi|29830672|ref|NP_825306.1| hypothetical protein SAV4129 [Streptomyces avermitilis MA-4680]
>gi|29830676|ref|NP_825310.1| hypothetical protein SAV4133 [Streptomyces avermitilis MA-4680]
>gi|29830687|ref|NP_825321.1| putative two-component system sensor kinase [Streptomyces avermitilis MA-4680]
>gi|29830691|ref|NP_825325.1| putative membrane protein [Streptomyces avermitilis MA-4680]
>gi|29830693|ref|NP_825327.1| putative DNA polymerase III gamma subunit [Streptomyces avermitilis MA-4680]
>gi|29830701|ref|NP_825335.1| putative two-component system sensor kinase [Streptomyces avermitilis MA-4680]
>gi|29830702|ref|NP_825336.1| putative two-component system response regulator [Streptomyces avermitilis MA-4680]
>gi|29830703|ref|NP_825337.1| hypothetical protein SAV4160 [Streptomyces avermitilis MA-4680]
>gi|29830706|ref|NP_825340.1| putative membrane protein [Streptomyces avermitilis MA-4680]
>gi|29830707|ref|NP_825341.1| putative membrane protein [Streptomyces avermitilis MA-4680]
>gi|29830709|ref|NP_825343.1| putative membrane protein [Streptomyces avermitilis MA-4680]
>gi|29830710|ref|NP_825344.1| putative dehydrogenase [Streptomyces avermitilis MA-4680]
>gi|29830714|ref|NP_825348.1| putative penicillin acylase [Streptomyces avermitilis MA-4680]
>gi|29830716|ref|NP_825350.1| putative membrane protein [Streptomyces avermitilis MA-4680]
>gi|29830717|ref|NP_825351.1| hypothetical protein SAV4174 [Streptomyces avermitilis MA-4680]
>gi|29830722|ref|NP_825356.1| hypothetical protein SAV4179 [Streptomyces avermitilis MA-4680]
>gi|29830730|ref|NP_825364.1| hypothetical protein SAV4187 [Streptomyces avermitilis MA-4680]
>gi|29830731|ref|NP_825365.1| putative membrane protein [Streptomyces avermitilis MA-4680]
>gi|29830737|ref|NP_825371.1| putative transmembrane efflux protein [Streptomyces avermitilis MA-4680]
>gi|29830738|ref|NP_825372.1| putative membrane protein [Streptomyces avermitilis MA-4680]
>gi|29830739|ref|NP_825373.1| putative glycosyl transferase [Streptomyces avermitilis MA-4680]
>gi|29830743|ref|NP_825377.1| hypothetical protein SAV4200 [Streptomyces avermitilis MA-4680]
>gi|29830744|ref|NP_825378.1| hypothetical protein SAV4201 [Streptomyces avermitilis MA-4680]
>gi|29830745|ref|NP_825379.1| hypothetical protein SAV4202 [Streptomyces avermitilis MA-4680]
>gi|29830748|ref|NP_825382.1| putative two-component system sensor kinase/response regulator, bifunctional protein [Streptomyces avermitilis MA-4680]
>gi|29830753|ref|NP_825387.1| putative acyl-CoA dehydrogenase [Streptomyces avermitilis MA-4680]
>gi|29830755|ref|NP_825389.1| putative acyl-CoA dehydrogenase [Streptomyces avermitilis MA-4680]
>gi|29830756|ref|NP_825390.1| hypothetical protein SAV4213 [Streptomyces avermitilis MA-4680]
>gi|29830760|ref|NP_825394.1| hypothetical protein SAV4217 [Streptomyces avermitilis MA-4680]
>gi|29830761|ref|NP_825395.1| hypothetical protein SAV4218 [Streptomyces avermitilis MA-4680]
>gi|29830766|ref|NP_825400.1| putative serine protease [Streptomyces avermitilis MA-4680]
>gi|29830770|ref|NP_825404.1| hypothetical protein SAV4227 [Streptomyces avermitilis MA-4680]
>gi|29830775|ref|NP_825409.1| putative membrane protein [Streptomyces avermitilis MA-4680]
>gi|29830776|ref|NP_825410.1| putative 2-(5''-triphosphoribosyl)-3'-dephospho- CoA synthase [Streptomyces avermitilis MA-4680]
>gi|29830777|ref|NP_825411.1| putative protocatechuate dioxygenase [Streptomyces avermitilis MA-4680]
>gi|29830780|ref|NP_825414.1| hypothetical protein SAV4237 [Streptomyces avermitilis MA-4680]
>gi|29830781|ref|NP_825415.1| hypothetical protein SAV4238 [Streptomyces avermitilis MA-4680]

>gi|29830784|ref|NP_825418.1| putative integral membrane protein [Streptomyces avermitilis MA-4680]
>gi|29830788|ref|NP_825422.1| putative hydrolase [Streptomyces avermitilis MA-4680]
>gi|29830801|ref|NP_825435.1| putative ABC transporter ATP-binding protein [Streptomyces avermitilis MA-4680]
>gi|29830807|ref|NP_825441.1| putative transmembrane protein [Streptomyces avermitilis MA-4680]
>gi|29830809|ref|NP_825443.1| putative membrane protein [Streptomyces avermitilis MA-4680]
>gi|29830812|ref|NP_825446.1| putative membrane spanning protein [Streptomyces avermitilis MA-4680]
>gi|29830814|ref|NP_825448.1| putative membrane protein [Streptomyces avermitilis MA-4680]
>gi|29830815|ref|NP_825449.1| putative low molecular weight protein tyrosine phosphatase [Streptomyces avermitilis MA-4680]
>gi|29830819|ref|NP_825453.1| putative flavohemoprotein [Streptomyces avermitilis MA-4680]
>gi|29830823|ref|NP_825457.1| putative transmembrane efflux protein [Streptomyces avermitilis MA-4680]
>gi|29830828|ref|NP_825462.1| putative DNA-damage-inducible protein F [Streptomyces avermitilis MA-4680]
>gi|29830831|ref|NP_825465.1| putative single-stranded DNA-binding protein [Streptomyces avermitilis MA-4680]
>gi|29830837|ref|NP_825471.1| putative penicillin-binding protein [Streptomyces avermitilis MA-4680]
>gi|29830838|ref|NP_825472.1| putative transcriptional regulator PadR-like family [Streptomyces avermitilis MA-4680]
>gi|29830841|ref|NP_825475.1| hypothetical protein SAV4298 [Streptomyces avermitilis MA-4680]
>gi|29830844|ref|NP_825478.1| putative transmembrane protein [Streptomyces avermitilis MA-4680]
>gi|29830847|ref|NP_825481.1| hypothetical protein SAV4304 [Streptomyces avermitilis MA-4680]
>gi|29830863|ref|NP_825497.1| hypothetical protein SAV4320 [Streptomyces avermitilis MA-4680]
>gi|29830866|ref|NP_825500.1| putative membrane protein [Streptomyces avermitilis MA-4680]
>gi|29830868|ref|NP_825502.1| hypothetical protein SAV4325 [Streptomyces avermitilis MA-4680]
>gi|29830869|ref|NP_825503.1| putative serine/threonine protein kinase [Streptomyces avermitilis MA-4680]
>gi|29830871|ref|NP_825505.1| putative regulatory protein [Streptomyces avermitilis MA-4680]
>gi|29830879|ref|NP_825513.1| hypothetical protein SAV4336 [Streptomyces avermitilis MA-4680]
>gi|29830883|ref|NP_825517.1| putative cell division membrane protein [Streptomyces avermitilis MA-4680]
>gi|29830886|ref|NP_825520.1| hypothetical protein SAV4343 [Streptomyces avermitilis MA-4680]
>gi|29830898|ref|NP_825532.1| putative membrane protein [Streptomyces avermitilis MA-4680]
>gi|29830900|ref|NP_825534.1| putative rRNA methyltransferase [Streptomyces avermitilis MA-4680]
>gi|29830907|ref|NP_825541.1| putative dihydrolipoamide acyltransferase [Streptomyces avermitilis MA-4680]
>gi|29830908|ref|NP_825542.1| putative molybdopterin-guanine dinucleotide biosynthesis protein [Streptomyces avermitilis MA-4680]
>gi|29830912|ref|NP_825546.1| hypothetical protein SAV4369 [Streptomyces avermitilis MA-4680]
>gi|29830914|ref|NP_825548.1| putative serine/threonine protein kinase [Streptomyces avermitilis MA-4680]
>gi|29830915|ref|NP_825549.1| putative serine/threonine protein kinase [Streptomyces avermitilis MA-4680]
>gi|29830924|ref|NP_825558.1| putative transmembrane transport protein [Streptomyces avermitilis MA-4680]
>gi|29830928|ref|NP_825562.1| hypothetical protein SAV4385 [Streptomyces avermitilis MA-4680]
>gi|29830936|ref|NP_825570.1| putative integral membrane protein [Streptomyces avermitilis MA-4680]
>gi|29830939|ref|NP_825573.1| putative dioxygenase [Streptomyces avermitilis MA-4680]
>gi|29830940|ref|NP_825574.1| hypothetical protein SAV4397 [Streptomyces avermitilis MA-4680]
>gi|29830943|ref|NP_825577.1| hypothetical protein SAV4400 [Streptomyces avermitilis MA-4680]
>gi|29830947|ref|NP_825581.1| hypothetical protein SAV4404 [Streptomyces avermitilis MA-4680]
>gi|29830950|ref|NP_825584.1| putative secreted metalloprotease [Streptomyces avermitilis MA-4680]
>gi|29830951|ref|NP_825585.1| putative integral membrane protein [Streptomyces avermitilis MA-4680]
>gi|29830952|ref|NP_825586.1| hypothetical protein SAV4409 [Streptomyces avermitilis MA-4680]
>gi|29830954|ref|NP_825588.1| putative GntR-family transcriptional regulator [Streptomyces avermitilis MA-4680]
>gi|29830955|ref|NP_825589.1| putative transcriptional regulator [Streptomyces avermitilis MA-4680]
>gi|29830956|ref|NP_825590.1| hypothetical protein SAV4413 [Streptomyces avermitilis MA-4680]
>gi|29830958|ref|NP_825592.1| putative magnesium or manganese-dependent protein phosphatase [Streptomyces avermitilis MA-4680]
>gi|29830960|ref|NP_825594.1| putative two-component system sensor kinase [Streptomyces avermitilis MA-4680]
>gi|29830961|ref|NP_825595.1| hypothetical protein SAV4418 [Streptomyces avermitilis MA-4680]
>gi|29830963|ref|NP_825597.1| hypothetical protein SAV4420 [Streptomyces avermitilis MA-4680]
>gi|29830964|ref|NP_825598.1| putative regulatory protein [Streptomyces avermitilis MA-4680]
>gi|29830968|ref|NP_825602.1| putative RNA polymerase ECF-subfamily sigma factor [Streptomyces avermitilis MA-4680]
>gi|29830969|ref|NP_825603.1| hypothetical protein SAV4426 [Streptomyces avermitilis MA-4680]
>gi|29830970|ref|NP_825604.1| putative RNA polymerase ECF-subfamily sigma factor [Streptomyces avermitilis MA-4680]
>gi|29830978|ref|NP_825612.1| hypothetical protein SAV4435 [Streptomyces avermitilis MA-4680]
>gi|29830980|ref|NP_825614.1| hypothetical protein SAV4437 [Streptomyces avermitilis MA-4680]
>gi|29830981|ref|NP_825615.1| hypothetical protein SAV4438 [Streptomyces avermitilis MA-4680]
>gi|29830982|ref|NP_825616.1| putative permease [Streptomyces avermitilis MA-4680]
>gi|29830989|ref|NP_825623.1| putative ATP-dependent RNA helicase [Streptomyces avermitilis MA-4680]
>gi|29830992|ref|NP_825626.1| hypothetical protein SAV4449 [Streptomyces avermitilis MA-4680]
>gi|29830993|ref|NP_825627.1| hypothetical protein SAV4450 [Streptomyces avermitilis MA-4680]
>gi|29830995|ref|NP_825629.1| putative beta-lactamase [Streptomyces avermitilis MA-4680]
>gi|29831002|ref|NP_825636.1| hypothetical protein SAV4459 [Streptomyces avermitilis MA-4680]
>gi|29831012|ref|NP_825646.1| putative membrane protein [Streptomyces avermitilis MA-4680]
>gi|29831016|ref|NP_825650.1| putative integrin-like protein [Streptomyces avermitilis MA-4680]
>gi|29831017|ref|NP_825651.1| putative integrin-like protein [Streptomyces avermitilis MA-4680]
>gi|29831018|ref|NP_825652.1| putative integrin-like protein [Streptomyces avermitilis MA-4680]
>gi|29831019|ref|NP_825653.1| putative integrin-like protein [Streptomyces avermitilis MA-4680]
>gi|29831024|ref|NP_825658.1| hypothetical protein SAV4481 [Streptomyces avermitilis MA-4680]
>gi|29831026|ref|NP_825660.1| putative glycosyl transferase [Streptomyces avermitilis MA-4680]
>gi|29831028|ref|NP_825662.1| putative heat shock protein GrpE [Streptomyces avermitilis MA-4680]

>gi|29831029|ref|NP_825663.1| putative DnaJ protein [Streptomyces avermitilis MA-4680]
>gi|29831034|ref|NP_825668.1| hypothetical protein SAV4491 [Streptomyces avermitilis MA-4680]
>gi|29831035|ref|NP_825669.1| hypothetical protein SAV4492 [Streptomyces avermitilis MA-4680]
>gi|29831038|ref|NP_825672.1| hypothetical protein SAV4495 [Streptomyces avermitilis MA-4680]
>gi|29831045|ref|NP_825679.1| hypothetical protein SAV4501 [Streptomyces avermitilis MA-4680]
>gi|29831049|ref|NP_825683.1| hypothetical protein SAV4506 [Streptomyces avermitilis MA-4680]
>gi|29831059|ref|NP_825693.1| putative membrane protein [Streptomyces avermitilis MA-4680]
>gi|29831062|ref|NP_825696.1| putative spermidine synthase [Streptomyces avermitilis MA-4680]
>gi|29831063|ref|NP_825697.1| hypothetical protein SAV4520 [Streptomyces avermitilis MA-4680]
>gi|29831065|ref|NP_825699.1| putative orotate phosphoribosyltransferase [Streptomyces avermitilis MA-4680]
>gi|29831067|ref|NP_825701.1| putative transmembrane efflux protein [Streptomyces avermitilis MA-4680]
>gi|29831071|ref|NP_825705.1| hypothetical protein SAV4528 [Streptomyces avermitilis MA-4680]
>gi|29831072|ref|NP_825706.1| hypothetical protein SAV4529 [Streptomyces avermitilis MA-4680]
>gi|29831074|ref|NP_825708.1| putative lipase/esterase [Streptomyces avermitilis MA-4680]
>gi|29831075|ref|NP_825709.1| putative membrane protein [Streptomyces avermitilis MA-4680]
>gi|29831076|ref|NP_825710.1| putative membrane protein [Streptomyces avermitilis MA-4680]
>gi|29831083|ref|NP_825717.1| putative ABC transporter membrane protein [Streptomyces avermitilis MA-4680]
>gi|29831089|ref|NP_825723.1| hypothetical protein SAV4546 [Streptomyces avermitilis MA-4680]
>gi|29831097|ref|NP_825731.1| putative serine/threonine protein kinase [Streptomyces avermitilis MA-4680]
>gi|29831098|ref|NP_825732.1| hypothetical protein SAV4555 [Streptomyces avermitilis MA-4680]
>gi|29831105|ref|NP_825739.1| hypothetical protein SAV4562 [Streptomyces avermitilis MA-4680]
>gi|29831108|ref|NP_825742.1| putative membrane protein [Streptomyces avermitilis MA-4680]
>gi|29831110|ref|NP_825744.1| hypothetical protein SAV4567 [Streptomyces avermitilis MA-4680]
>gi|29831111|ref|NP_825745.1| putative permease [Streptomyces avermitilis MA-4680]
>gi|29831112|ref|NP_825746.1| putative membrane protein [Streptomyces avermitilis MA-4680]
>gi|29831116|ref|NP_825750.1| putative glycosyltransferase [Streptomyces avermitilis MA-4680]
>gi|29831119|ref|NP_825753.1| hypothetical protein SAV4576 [Streptomyces avermitilis MA-4680]
>gi|29831123|ref|NP_825757.1| putative membrane protein [Streptomyces avermitilis MA-4680]
>gi|29831135|ref|NP_825769.1| putative transcriptional regulator with cyclic nucleotide-binding domain [Streptomyces avermitilis MA-4680]
>gi|29831143|ref|NP_825777.1| putative membrane protein [Streptomyces avermitilis MA-4680]
>gi|29831148|ref|NP_825782.1| putative septum site determining protein [Streptomyces avermitilis MA-4680]
>gi|29831149|ref|NP_825783.1| hypothetical protein SAV4606 [Streptomyces avermitilis MA-4680]
>gi|29831150|ref|NP_825784.1| hypothetical protein SAV4607 [Streptomyces avermitilis MA-4680]
>gi|29831151|ref|NP_825785.1| hypothetical protein SAV4608 [Streptomyces avermitilis MA-4680]
>gi|29831152|ref|NP_825786.1| putative membrane protein [Streptomyces avermitilis MA-4680]
>gi|29831153|ref|NP_825787.1| hypothetical protein SAV4610 [Streptomyces avermitilis MA-4680]
>gi|29831154|ref|NP_825788.1| hypothetical protein SAV4611 [Streptomyces avermitilis MA-4680]
>gi|29831155|ref|NP_825789.1| putative membrane protein [Streptomyces avermitilis MA-4680]
>gi|29831158|ref|NP_825792.1| putative anti-sigma factor [Streptomyces avermitilis MA-4680]
>gi|29831159|ref|NP_825793.1| putative inorganic proton pyrophosphatase [Streptomyces avermitilis MA-4680]
>gi|29831162|ref|NP_825796.1| putative serine/threonine protein kinase [Streptomyces avermitilis MA-4680]
>gi|29831165|ref|NP_825799.1| putative thymidylate kinase [Streptomyces avermitilis MA-4680]
>gi|29831166|ref|NP_825800.1| putative DNA polymerase III delta' subunit [Streptomyces avermitilis MA-4680]
>gi|29831168|ref|NP_825802.1| putative integrase/recombinase [Streptomyces avermitilis MA-4680]
>gi|29831169|ref|NP_825803.1| hypothetical protein SAV4625 [Streptomyces avermitilis MA-4680]
>gi|29831170|ref|NP_825804.1| hypothetical protein SAV4627 [Streptomyces avermitilis MA-4680]
>gi|29831172|ref|NP_825806.1| hypothetical protein SAV4629 [Streptomyces avermitilis MA-4680]
>gi|29831175|ref|NP_825809.1| hypothetical protein SAV4632 [Streptomyces avermitilis MA-4680]
>gi|29831178|ref|NP_825812.1| putative transcriptional regulator [Streptomyces avermitilis MA-4680]
>gi|29831180|ref|NP_825814.1| putative ClpX protein [Streptomyces avermitilis MA-4680]
>gi|29831182|ref|NP_825816.1| hypothetical protein SAV4639 [Streptomyces avermitilis MA-4680]
>gi|29831183|ref|NP_825817.1| hypothetical protein SAV4640 [Streptomyces avermitilis MA-4680]
>gi|29831184|ref|NP_825818.1| putative ribosomal protein S14 [Streptomyces avermitilis MA-4680]
>gi|29831188|ref|NP_825822.1| hypothetical protein SAV4645 [Streptomyces avermitilis MA-4680]
>gi|29831199|ref|NP_825833.1| hypothetical protein SAV4656 [Streptomyces avermitilis MA-4680]
>gi|29831202|ref|NP_825836.1| putative membrane protein [Streptomyces avermitilis MA-4680]
>gi|29831211|ref|NP_825845.1| hypothetical protein SAV4668 [Streptomyces avermitilis MA-4680]
>gi|29831215|ref|NP_825849.1| putative dihydropteroate synthase [Streptomyces avermitilis MA-4680]
>gi|29831218|ref|NP_825852.1| hypothetical protein SAV4675 [Streptomyces avermitilis MA-4680]
>gi|29831220|ref|NP_825854.1| putative integral membrane protein [Streptomyces avermitilis MA-4680]
>gi|29831230|ref|NP_825864.1| putative pantoate--beta-alanine ligase [Streptomyces avermitilis MA-4680]
>gi|29831234|ref|NP_825868.1| hypothetical protein SAV4691 [Streptomyces avermitilis MA-4680]
>gi|29831236|ref|NP_825870.1| hypothetical protein SAV4693 [Streptomyces avermitilis MA-4680]
>gi|29831238|ref|NP_825872.1| putative Lsr2-like protein [Streptomyces avermitilis MA-4680]
>gi|29831239|ref|NP_825873.1| putative proline-rich protein [Streptomyces avermitilis MA-4680]
>gi|29831243|ref|NP_825877.1| putative peptidase [Streptomyces avermitilis MA-4680]
>gi|29831246|ref|NP_825880.1| putative two-component system sensor kinase [Streptomyces avermitilis MA-4680]
>gi|29831254|ref|NP_825888.1| putative alanine-rich protein [Streptomyces avermitilis MA-4680]
>gi|29831258|ref|NP_825892.1| putative TetR-family transcriptional regulator [Streptomyces avermitilis MA-4680]
>gi|29831264|ref|NP_825898.1| putative sulfate transporter [Streptomyces avermitilis MA-4680]

>gi|29831266|ref|NP_825900.1| putative ABC transporter permease protein [Streptomyces avermitilis MA-4680]
>gi|29831271|ref|NP_825905.1| putative integral membrane transport protein [Streptomyces avermitilis MA-4680]
>gi|29831277|ref|NP_825911.1| hypothetical protein SAV4734 [Streptomyces avermitilis MA-4680]
>gi|29831278|ref|NP_825912.1| putative RNA polymerase ECF-subfamily sigma factor [Streptomyces avermitilis MA-4680]
>gi|29831282|ref|NP_825916.1| putative glutamyl-tRNA reductase [Streptomyces avermitilis MA-4680]
>gi|29831286|ref|NP_825920.1| hypothetical protein SAV4743 [Streptomyces avermitilis MA-4680]
>gi|29831287|ref|NP_825921.1| putative DNA-binding protein [Streptomyces avermitilis MA-4680]
>gi|29831290|ref|NP_825924.1| hypothetical protein SAV4747 [Streptomyces avermitilis MA-4680]
>gi|29831293|ref|NP_825927.1| putative membrane protein [Streptomyces avermitilis MA-4680]
>gi|29831294|ref|NP_825928.1| hypothetical protein SAV4751 [Streptomyces avermitilis MA-4680]
>gi|29831295|ref|NP_825929.1| hypothetical protein SAV4752 [Streptomyces avermitilis MA-4680]
>gi|29831302|ref|NP_825936.1| putative dehydrogenase [Streptomyces avermitilis MA-4680]
>gi|29831303|ref|NP_825937.1| putative transmembrane efflux protein [Streptomyces avermitilis MA-4680]
>gi|29831307|ref|NP_825941.1| putative NAD(P)H-dependent FMN reductase [Streptomyces avermitilis MA-4680]
>gi|29831308|ref|NP_825942.1| putative membrane protein [Streptomyces avermitilis MA-4680]
>gi|29831311|ref|NP_825945.1| putative two-component system sensor kinase [Streptomyces avermitilis MA-4680]
>gi|29831314|ref|NP_825948.1| hypothetical protein SAV4771 [Streptomyces avermitilis MA-4680]
>gi|29831320|ref|NP_825954.1| hypothetical protein SAV4777 [Streptomyces avermitilis MA-4680]
>gi|29831321|ref|NP_825955.1| hypothetical protein SAV4778 [Streptomyces avermitilis MA-4680]
>gi|29831326|ref|NP_825960.1| putative regulatory protein [Streptomyces avermitilis MA-4680]
>gi|29831331|ref|NP_825965.1| hypothetical protein SAV4788 [Streptomyces avermitilis MA-4680]
>gi|29831334|ref|NP_825968.1| hypothetical protein SAV4791 [Streptomyces avermitilis MA-4680]
>gi|29831337|ref|NP_825971.1| hypothetical protein SAV4794 [Streptomyces avermitilis MA-4680]
>gi|29831360|ref|NP_825994.1| putative dehydratase [Streptomyces avermitilis MA-4680]
>gi|29831365|ref|NP_825999.1| putative serine/threonine protein kinase [Streptomyces avermitilis MA-4680]
>gi|29831366|ref|NP_826000.1| hypothetical protein SAV4823 [Streptomyces avermitilis MA-4680]
>gi|29831367|ref|NP_826001.1| putative type IV peptidase [Streptomyces avermitilis MA-4680]
>gi|29831370|ref|NP_826004.1| putative sugar hydrolase [Streptomyces avermitilis MA-4680]
>gi|29831371|ref|NP_826005.1| putative membrane protein [Streptomyces avermitilis MA-4680]
>gi|29831373|ref|NP_826007.1| hypothetical protein SAV4830 [Streptomyces avermitilis MA-4680]
>gi|29831375|ref|NP_826009.1| hypothetical protein SAV4832 [Streptomyces avermitilis MA-4680]
>gi|29831376|ref|NP_826010.1| hypothetical protein SAV4833 [Streptomyces avermitilis MA-4680]
>gi|29831379|ref|NP_826013.1| putative NLP/P60-family protein [Streptomyces avermitilis MA-4680]
>gi|29831395|ref|NP_826029.1| putative oxidoreductase [Streptomyces avermitilis MA-4680]
>gi|29831396|ref|NP_826030.1| putative ABC transporter ATP-binding protein [Streptomyces avermitilis MA-4680]
>gi|29831397|ref|NP_826031.1| putative ABC transporter permease protein [Streptomyces avermitilis MA-4680]
>gi|29831408|ref|NP_826042.1| hypothetical protein SAV4865 [Streptomyces avermitilis MA-4680]
>gi|29831410|ref|NP_826044.1| putative ABC transporter ATP-binding protein [Streptomyces avermitilis MA-4680]
>gi|29831411|ref|NP_826045.1| putative ABC transporter membrane protein [Streptomyces avermitilis MA-4680]
>gi|29831414|ref|NP_826048.1| hypothetical protein SAV4871 [Streptomyces avermitilis MA-4680]
>gi|29831416|ref|NP_826050.1| hypothetical protein SAV4873 [Streptomyces avermitilis MA-4680]
>gi|29831417|ref|NP_826051.1| hypothetical protein SAV4874 [Streptomyces avermitilis MA-4680]
>gi|29831424|ref|NP_826058.1| putative NADH dehydrogenase I chain A [Streptomyces avermitilis MA-4680]
>gi|29831426|ref|NP_826060.1| putative NADH dehydrogenase [Streptomyces avermitilis MA-4680]
>gi|29831428|ref|NP_826062.1| putative NADH dehydrogenase I chain I [Streptomyces avermitilis MA-4680]
>gi|29831431|ref|NP_826065.1| putative NADH dehydrogenase I chain L [Streptomyces avermitilis MA-4680]
>gi|29831433|ref|NP_826067.1| putative NADH dehydrogenase I chain N [Streptomyces avermitilis MA-4680]
>gi|29831437|ref|NP_826071.1| putative amino acid transporter [Streptomyces avermitilis MA-4680]
>gi|29831440|ref|NP_826074.1| putative amidohydrolase [Streptomyces avermitilis MA-4680]
>gi|29831447|ref|NP_826081.1| putative transmembrane efflux protein [Streptomyces avermitilis MA-4680]
>gi|29831452|ref|NP_826086.1| putative transcription antitermination protein [Streptomyces avermitilis MA-4680]
>gi|29831456|ref|NP_826090.1| putative ribosomal protein L7/L12 [Streptomyces avermitilis MA-4680]
>gi|29831465|ref|NP_826099.1| hypothetical protein SAV4922 [Streptomyces avermitilis MA-4680]
>gi|29831475|ref|NP_826109.1| putative ribosomal protein S3 [Streptomyces avermitilis MA-4680]
>gi|29831486|ref|NP_826120.1| putative ribosomal protein S5 [Streptomyces avermitilis MA-4680]
>gi|29831499|ref|NP_826133.1| hypothetical protein SAV4956 [Streptomyces avermitilis MA-4680]
>gi|29831501|ref|NP_826135.1| putative ribosomal protein S9 [Streptomyces avermitilis MA-4680]
>gi|29831503|ref|NP_826137.1| putative membrane protein [Streptomyces avermitilis MA-4680]
>gi|29831508|ref|NP_826142.1| hypothetical protein SAV4965 [Streptomyces avermitilis MA-4680]
>gi|29831509|ref|NP_826143.1| hypothetical protein SAV4966 [Streptomyces avermitilis MA-4680]
>gi|29831510|ref|NP_826144.1| putative alanine racemase [Streptomyces avermitilis MA-4680]
>gi|29831511|ref|NP_826145.1| putative hydrolase [Streptomyces avermitilis MA-4680]
>gi|29831513|ref|NP_826147.1| hypothetical protein SAV4970 [Streptomyces avermitilis MA-4680]
>gi|29831515|ref|NP_826149.1| hypothetical protein SAV4972 [Streptomyces avermitilis MA-4680]
>gi|29831520|ref|NP_826154.1| hypothetical protein SAV4977 [Streptomyces avermitilis MA-4680]
>gi|29831528|ref|NP_826162.1| putative transcriptional regulator [Streptomyces avermitilis MA-4680]
>gi|29831531|ref|NP_826165.1| hypothetical protein SAV4988 [Streptomyces avermitilis MA-4680]
>gi|29831548|ref|NP_826182.1| putative serine/threonine protein kinase [Streptomyces avermitilis MA-4680]
>gi|29831549|ref|NP_826183.1| putative serine/threonine protein kinase [Streptomyces avermitilis MA-4680]
>gi|29831553|ref|NP_826187.1| putative serine/threonine protein kinase [Streptomyces avermitilis MA-4680]

>gi|29831558|ref|NP_826192.1| putative membrane protein [Streptomyces avermitilis MA-4680]
>gi|29831561|ref|NP_826195.1| hypothetical protein SAV5018 [Streptomyces avermitilis MA-4680]
>gi|29831580|ref|NP_826214.1| putative nucleotide phosphorylase [Streptomyces avermitilis MA-4680]
>gi|29831583|ref|NP_826217.1| hypothetical protein SAV5040 [Streptomyces avermitilis MA-4680]
>gi|29831586|ref|NP_826220.1| putative membrane protein [Streptomyces avermitilis MA-4680]
>gi|29831587|ref|NP_826221.1| putative secreted protein [Streptomyces avermitilis MA-4680]
>gi|29831588|ref|NP_826222.1| hypothetical protein SAV5045 [Streptomyces avermitilis MA-4680]
>gi|29831590|ref|NP_826224.1| putative L-lactate permease [Streptomyces avermitilis MA-4680]
>gi|29831593|ref|NP_826227.1| hypothetical protein SAV5050 [Streptomyces avermitilis MA-4680]
>gi|29831595|ref|NP_826229.1| putative transport protein [Streptomyces avermitilis MA-4680]
>gi|29831598|ref|NP_826232.1| putative membrane protein [Streptomyces avermitilis MA-4680]
>gi|29831600|ref|NP_826234.1| hypothetical protein SAV5057 [Streptomyces avermitilis MA-4680]
>gi|29831602|ref|NP_826236.1| putative secreted protein [Streptomyces avermitilis MA-4680]
>gi|29831603|ref|NP_826237.1| putative integral membrane protein [Streptomyces avermitilis MA-4680]
>gi|29831604|ref|NP_826238.1| putative integral membrane protein [Streptomyces avermitilis MA-4680]
>gi|29831605|ref|NP_826239.1| putative initiation factor eIF-2B alpha subunit [Streptomyces avermitilis MA-4680]
>gi|29831609|ref|NP_826243.1| hypothetical protein SAV5066 [Streptomyces avermitilis MA-4680]
>gi|29831616|ref|NP_826250.1| hypothetical protein SAV5073 [Streptomyces avermitilis MA-4680]
>gi|29831620|ref|NP_826254.1| putative integral membrane protein [Streptomyces avermitilis MA-4680]
>gi|29831626|ref|NP_826260.1| putative ribosylglycoyhydrolase [Streptomyces avermitilis MA-4680]
>gi|29831627|ref|NP_826261.1| putative TetR-family transcriptional regulator [Streptomyces avermitilis MA-4680]
>gi|29831632|ref|NP_826266.1| hypothetical protein SAV5089 [Streptomyces avermitilis MA-4680]
>gi|29831633|ref|NP_826267.1| putative MarR-family transcriptional regulator [Streptomyces avermitilis MA-4680]
>gi|29831635|ref|NP_826269.1| putative integral membrane protein [Streptomyces avermitilis MA-4680]
>gi|29831642|ref|NP_826276.1| hypothetical protein SAV5099 [Streptomyces avermitilis MA-4680]
>gi|29831643|ref|NP_826277.1| putative serine/threonine protein kinase [Streptomyces avermitilis MA-4680]
>gi|29831644|ref|NP_826278.1| putative serine/threonine protein kinase [Streptomyces avermitilis MA-4680]
>gi|29831646|ref|NP_826280.1| putative secreted protein [Streptomyces avermitilis MA-4680]
>gi|29831649|ref|NP_826283.1| putative carboxy-terminal processing protease precursor [Streptomyces avermitilis MA-4680]
>gi|29831655|ref|NP_826289.1| putative transmembrane efflux protein [Streptomyces avermitilis MA-4680]
>gi|29831657|ref|NP_826291.1| putative membrane protein [Streptomyces avermitilis MA-4680]
>gi|29831664|ref|NP_826298.1| putative membrane protein [Streptomyces avermitilis MA-4680]
>gi|29831678|ref|NP_826312.1| putative dehydrogenase [Streptomyces avermitilis MA-4680]
>gi|29831679|ref|NP_826313.1| hypothetical protein SAV5136 [Streptomyces avermitilis MA-4680]
>gi|29831682|ref|NP_826316.1| putative multidrug resistance efflux protein [Streptomyces avermitilis MA-4680]
>gi|29831685|ref|NP_826319.1| putative secreted protein [Streptomyces avermitilis MA-4680]
>gi|29831688|ref|NP_826322.1| putative ABC transporter permease protein [Streptomyces avermitilis MA-4680]
>gi|29831693|ref|NP_826327.1| hypothetical protein SAV5150 [Streptomyces avermitilis MA-4680]
>gi|29831695|ref|NP_826329.1| putative membrane protein [Streptomyces avermitilis MA-4680]
>gi|29831697|ref|NP_826331.1| hypothetical protein SAV5154 [Streptomyces avermitilis MA-4680]
>gi|29831699|ref|NP_826333.1| hypothetical protein SAV5156 [Streptomyces avermitilis MA-4680]
>gi|29831708|ref|NP_826342.1| hypothetical protein SAV5165 [Streptomyces avermitilis MA-4680]
>gi|29831714|ref|NP_826348.1| putative secreted protein [Streptomyces avermitilis MA-4680]
>gi|29831718|ref|NP_826352.1| putative osmoprotectant transporter [Streptomyces avermitilis MA-4680]
>gi|29831726|ref|NP_826360.1| hypothetical protein SAV5183 [Streptomyces avermitilis MA-4680]
>gi|29831730|ref|NP_826364.1| putative membrane protein [Streptomyces avermitilis MA-4680]
>gi|29831734|ref|NP_826368.1| putative membrane protein [Streptomyces avermitilis MA-4680]
>gi|29831737|ref|NP_826371.1| hypothetical protein SAV5194 [Streptomyces avermitilis MA-4680]
>gi|29831746|ref|NP_826380.1| hypothetical protein SAV5203 [Streptomyces avermitilis MA-4680]
>gi|29831749|ref|NP_826383.1| putative membrane protein [Streptomyces avermitilis MA-4680]
>gi|29831750|ref|NP_826384.1| hypothetical protein SAV5207 [Streptomyces avermitilis MA-4680]
>gi|29831755|ref|NP_826389.1| putative ROK-family transcriptional regulator [Streptomyces avermitilis MA-4680]
>gi|29831756|ref|NP_826390.1| hypothetical protein SAV5213 [Streptomyces avermitilis MA-4680]
>gi|29831758|ref|NP_826392.1| hypothetical protein SAV5215 [Streptomyces avermitilis MA-4680]
>gi|29831765|ref|NP_826399.1| hypothetical protein SAV5222 [Streptomyces avermitilis MA-4680]
>gi|29831781|ref|NP_826415.1| hypothetical protein SAV5238 [Streptomyces avermitilis MA-4680]
>gi|29831782|ref|NP_826416.1| hypothetical protein SAV5239 [Streptomyces avermitilis MA-4680]
>gi|29831783|ref|NP_826417.1| hypothetical protein SAV5240 [Streptomyces avermitilis MA-4680]
>gi|29831789|ref|NP_826423.1| hypothetical protein SAV5246 [Streptomyces avermitilis MA-4680]
>gi|29831792|ref|NP_826426.1| putative secreted protein [Streptomyces avermitilis MA-4680]
>gi|29831795|ref|NP_826429.1| putative sugar hydrolase [Streptomyces avermitilis MA-4680]
>gi|29831801|ref|NP_826435.1| hypothetical protein SAV5258 [Streptomyces avermitilis MA-4680]
>gi|29831805|ref|NP_826439.1| hypothetical protein SAV5262 [Streptomyces avermitilis MA-4680]
>gi|29831807|ref|NP_826441.1| hypothetical protein SAV5264 [Streptomyces avermitilis MA-4680]
>gi|29831820|ref|NP_826454.1| putative acetyl/propionyl CoA carboxylase alpha subunit [Streptomyces avermitilis MA-4680]
>gi|29831826|ref|NP_826460.1| putative regulatory protein [Streptomyces avermitilis MA-4680]
>gi|29831830|ref|NP_826464.1| hypothetical protein SAV5287 [Streptomyces avermitilis MA-4680]
>gi|29831833|ref|NP_826467.1| putative integral membrane protein [Streptomyces avermitilis MA-4680]
>gi|29831839|ref|NP_826473.1| hypothetical protein SAV5296 [Streptomyces avermitilis MA-4680]
>gi|29831840|ref|NP_826474.1| hypothetical protein SAV5297 [Streptomyces avermitilis MA-4680]

>gi|29831851|ref|NP_826485.1| putative serine/threonine protein kinase [Streptomyces avermitilis MA-4680]
>gi|29831852|ref|NP_826486.1| hypothetical protein SAV5309 [Streptomyces avermitilis MA-4680]
>gi|29831861|ref|NP_826495.1| putative D-ribose ABC transporter permease protein [Streptomyces avermitilis MA-4680]
>gi|29831866|ref|NP_826500.1| hypothetical protein SAV5323 [Streptomyces avermitilis MA-4680]
>gi|29831867|ref|NP_826501.1| hypothetical protein SAV5324 [Streptomyces avermitilis MA-4680]
>gi|29831874|ref|NP_826508.1| putative cation-transporting P-type ATPase [Streptomyces avermitilis MA-4680]
>gi|29831875|ref|NP_826509.1| putative copper chaperone [Streptomyces avermitilis MA-4680]
>gi|29831893|ref|NP_826527.1| hypothetical protein SAV5350 [Streptomyces avermitilis MA-4680]
>gi|29831894|ref|NP_826528.1| putative secreted protein [Streptomyces avermitilis MA-4680]
>gi|29831896|ref|NP_826530.1| hypothetical protein SAV5353 [Streptomyces avermitilis MA-4680]
>gi|29831897|ref|NP_826531.1| putative glycosyl transferase [Streptomyces avermitilis MA-4680]
>gi|29831899|ref|NP_826533.1| putative membrane protein [Streptomyces avermitilis MA-4680]
>gi|29831900|ref|NP_826534.1| hypothetical protein SAV5357 [Streptomyces avermitilis MA-4680]
>gi|29831901|ref|NP_826535.1| putative sugar transferase [Streptomyces avermitilis MA-4680]
>gi|29831902|ref|NP_826536.1| putative glycosyl transferase [Streptomyces avermitilis MA-4680]
>gi|29831903|ref|NP_826537.1| putative secreted protein [Streptomyces avermitilis MA-4680]
>gi|29831907|ref|NP_826541.1| putative ATP-binding protein [Streptomyces avermitilis MA-4680]
>gi|29831915|ref|NP_826549.1| hypothetical protein SAV5372 [Streptomyces avermitilis MA-4680]
>gi|29831930|ref|NP_826564.1| hypothetical protein SAV5387 [Streptomyces avermitilis MA-4680]
>gi|29831933|ref|NP_826567.1| hypothetical protein SAV5390 [Streptomyces avermitilis MA-4680]
>gi|29831935|ref|NP_826569.1| hypothetical protein SAV5392 [Streptomyces avermitilis MA-4680]
>gi|29831937|ref|NP_826571.1| hypothetical protein SAV5394 [Streptomyces avermitilis MA-4680]
>gi|29831942|ref|NP_826576.1| hypothetical protein SAV5399 [Streptomyces avermitilis MA-4680]
>gi|29831949|ref|NP_826583.1| hypothetical protein SAV5406 [Streptomyces avermitilis MA-4680]
>gi|29831952|ref|NP_826586.1| putative regulatory protein [Streptomyces avermitilis MA-4680]
>gi|29831961|ref|NP_826595.1| putative ABC transporter permease protein [Streptomyces avermitilis MA-4680]
>gi|29831963|ref|NP_826597.1| putative ROK-family transcriptional regulator [Streptomyces avermitilis MA-4680]
>gi|29831964|ref|NP_826598.1| putative biotin synthase [Streptomyces avermitilis MA-4680]
>gi|29831967|ref|NP_826601.1| putative serine/threonine protein kinase [Streptomyces avermitilis MA-4680]
>gi|29831973|ref|NP_826607.1| putative ABC transporter ATP-binding protein [Streptomyces avermitilis MA-4680]
>gi|29831974|ref|NP_826608.1| putative ABC transporter transmembrane protein [Streptomyces avermitilis MA-4680]
>gi|29831977|ref|NP_826611.1| hypothetical protein SAV5434 [Streptomyces avermitilis MA-4680]
>gi|29831978|ref|NP_826612.1| hypothetical protein SAV5435 [Streptomyces avermitilis MA-4680]
>gi|29831980|ref|NP_826614.1| hypothetical protein SAV5437 [Streptomyces avermitilis MA-4680]
>gi|29831982|ref|NP_826616.1| hypothetical protein SAV5439 [Streptomyces avermitilis MA-4680]
>gi|29831983|ref|NP_826617.1| hypothetical protein SAV5440 [Streptomyces avermitilis MA-4680]
>gi|29831984|ref|NP_826618.1| hypothetical protein SAV5441 [Streptomyces avermitilis MA-4680]
>gi|29831993|ref|NP_826627.1| putative membrane protein [Streptomyces avermitilis MA-4680]
>gi|29832000|ref|NP_826634.1| putative rod shape-determining protein [Streptomyces avermitilis MA-4680]
>gi|29832003|ref|NP_826637.1| hypothetical protein SAV5460 [Streptomyces avermitilis MA-4680]
>gi|29832006|ref|NP_826640.1| putative integral membrane protein [Streptomyces avermitilis MA-4680]
>gi|29832008|ref|NP_826642.1| hypothetical protein SAV5465 [Streptomyces avermitilis MA-4680]
>gi|29832015|ref|NP_826649.1| hypothetical protein SAV5472 [Streptomyces avermitilis MA-4680]
>gi|29832017|ref|NP_826651.1| hypothetical protein SAV5474 [Streptomyces avermitilis MA-4680]
>gi|29832022|ref|NP_826656.1| putative membrane protein [Streptomyces avermitilis MA-4680]
>gi|29832026|ref|NP_826660.1| putative transmembrane transport protein [Streptomyces avermitilis MA-4680]
>gi|29832028|ref|NP_826662.1| hypothetical protein SAV5485 [Streptomyces avermitilis MA-4680]
>gi|29832034|ref|NP_826668.1| putative DNA-binding protein [Streptomyces avermitilis MA-4680]
>gi|29832035|ref|NP_826669.1| putative membrane protein [Streptomyces avermitilis MA-4680]
>gi|29832062|ref|NP_826696.1| putative phage minor structural protein [Streptomyces avermitilis MA-4680]
>gi|29832073|ref|NP_826707.1| hypothetical protein SAV5530 [Streptomyces avermitilis MA-4680]
>gi|29832074|ref|NP_826708.1| hypothetical protein SAV5531 [Streptomyces avermitilis MA-4680]
>gi|29832075|ref|NP_826709.1| hypothetical protein SAV5532 [Streptomyces avermitilis MA-4680]
>gi|29832076|ref|NP_826710.1| hypothetical protein SAV5533 [Streptomyces avermitilis MA-4680]
>gi|29832095|ref|NP_826729.1| putative transfer protein spdA [Streptomyces avermitilis MA-4680]
>gi|29832096|ref|NP_826730.1| hypothetical protein SAV5553 [Streptomyces avermitilis MA-4680]
>gi|29832100|ref|NP_826734.1| hypothetical protein SAV5557 [Streptomyces avermitilis MA-4680]
>gi|29832103|ref|NP_826737.1| putative ribosomal protein S20 [Streptomyces avermitilis MA-4680]
>gi|29832108|ref|NP_826742.1| putative magnesium or manganese-dependent protein phosphatase [Streptomyces avermitilis MA-4680]
>gi|29832114|ref|NP_826748.1| putative dioxygenase [Streptomyces avermitilis MA-4680]
>gi|29832116|ref|NP_826750.1| putative lipoprotein [Streptomyces avermitilis MA-4680]
>gi|29832121|ref|NP_826755.1| putative transmembrane efflux protein [Streptomyces avermitilis MA-4680]
>gi|29832132|ref|NP_826766.1| hypothetical protein SAV5589 [Streptomyces avermitilis MA-4680]
>gi|29832133|ref|NP_826767.1| hypothetical protein SAV5590 [Streptomyces avermitilis MA-4680]
>gi|29832134|ref|NP_826768.1| hypothetical protein SAV5591 [Streptomyces avermitilis MA-4680]
>gi|29832135|ref|NP_826769.1| hypothetical protein SAV5592 [Streptomyces avermitilis MA-4680]
>gi|29832137|ref|NP_826771.1| putative ammonium transporter [Streptomyces avermitilis MA-4680]
>gi|29832139|ref|NP_826773.1| putative acetyltransferase [Streptomyces avermitilis MA-4680]
>gi|29832140|ref|NP_826774.1| putative membrane protein [Streptomyces avermitilis MA-4680]
>gi|29832146|ref|NP_826780.1| putative membrane protein [Streptomyces avermitilis MA-4680]

>gi|29832147|ref|NP_826781.1| putative two-component system sensor kinase [Streptomyces avermitilis MA-4680]
>gi|29832157|ref|NP_826791.1| putative efflux protein [Streptomyces avermitilis MA-4680]
>gi|29832165|ref|NP_826799.1| putative ABC transporter ATP-binding protein [Streptomyces avermitilis MA-4680]
>gi|29832166|ref|NP_826800.1| putative two-component system sensor kinase [Streptomyces avermitilis MA-4680]
>gi|29832167|ref|NP_826801.1| putative two-component system response regulator [Streptomyces avermitilis MA-4680]
>gi|29832168|ref|NP_826802.1| hypothetical protein SAV5625 [Streptomyces avermitilis MA-4680]
>gi|29832173|ref|NP_826807.1| putative neutral zinc metalloprotease [Streptomyces avermitilis MA-4680]
>gi|29832175|ref|NP_826809.1| putative metal transport system ABC transporter permease [Streptomyces avermitilis MA-4680]
>gi|29832189|ref|NP_826823.1| putative transmembrane efflux protein [Streptomyces avermitilis MA-4680]
>gi|29832199|ref|NP_826833.1| putative two-component system response regulator [Streptomyces avermitilis MA-4680]
>gi|29832201|ref|NP_826835.1| putative transmembrane sulfate transport protein [Streptomyces avermitilis MA-4680]
>gi|29832202|ref|NP_826836.1| putative dehydrogenase [Streptomyces avermitilis MA-4680]
>gi|29832205|ref|NP_826839.1| hypothetical protein SAV5662 [Streptomyces avermitilis MA-4680]
>gi|29832214|ref|NP_826848.1| putative protocatechuate dioxygenase [Streptomyces avermitilis MA-4680]
>gi|29832216|ref|NP_826850.1| hypothetical protein SAV5673 [Streptomyces avermitilis MA-4680]
>gi|29832222|ref|NP_826856.1| putative RNA polymerase sigma factor [Streptomyces avermitilis MA-4680]
>gi|29832225|ref|NP_826859.1| hypothetical protein SAV5682 [Streptomyces avermitilis MA-4680]
>gi|29832229|ref|NP_826863.1| putative ABC transporter ATP-binding protein [Streptomyces avermitilis MA-4680]
>gi|29832230|ref|NP_826864.1| hypothetical protein SAV5687 [Streptomyces avermitilis MA-4680]
>gi|29832234|ref|NP_826868.1| hypothetical protein SAV5691 [Streptomyces avermitilis MA-4680]
>gi|29832236|ref|NP_826870.1| hypothetical protein SAV5693 [Streptomyces avermitilis MA-4680]
>gi|29832238|ref|NP_826872.1| hypothetical protein SAV5695 [Streptomyces avermitilis MA-4680]
>gi|29832241|ref|NP_826875.1| hypothetical protein SAV5698 [Streptomyces avermitilis MA-4680]
>gi|29832242|ref|NP_826876.1| hypothetical protein SAV5699 [Streptomyces avermitilis MA-4680]
>gi|29832267|ref|NP_826901.1| putative TetR-family transcriptional regulator [Streptomyces avermitilis MA-4680]
>gi|29832275|ref|NP_826909.1| hypothetical protein SAV5732 [Streptomyces avermitilis MA-4680]
>gi|29832278|ref|NP_826912.1| putative membrane protein [Streptomyces avermitilis MA-4680]
>gi|29832282|ref|NP_826916.1| hypothetical protein SAV5739 [Streptomyces avermitilis MA-4680]
>gi|29832287|ref|NP_826921.1| hypothetical protein SAV5744 [Streptomyces avermitilis MA-4680]
>gi|29832288|ref|NP_826922.1| hypothetical protein SAV5745 [Streptomyces avermitilis MA-4680]
>gi|29832289|ref|NP_826923.1| putative two-component system sensor kinase [Streptomyces avermitilis MA-4680]
>gi|29832291|ref|NP_826925.1| putative lipoprotein [Streptomyces avermitilis MA-4680]
>gi|29832294|ref|NP_826928.1| hypothetical protein SAV5751 [Streptomyces avermitilis MA-4680]
>gi|29832296|ref|NP_826930.1| hypothetical protein SAV5753 [Streptomyces avermitilis MA-4680]
>gi|29832301|ref|NP_826935.1| putative protocatechuate dioxygenase [Streptomyces avermitilis MA-4680]
>gi|29832302|ref|NP_826936.1| putative monooxygenase [Streptomyces avermitilis MA-4680]
>gi|29832303|ref|NP_826937.1| putative TetR-family transcriptional regulator [Streptomyces avermitilis MA-4680]
>gi|29832304|ref|NP_826938.1| putative protocatechuate dioxygenase [Streptomyces avermitilis MA-4680]
>gi|29832306|ref|NP_826940.1| putative ABC transporter ATP-binding protein [Streptomyces avermitilis MA-4680]
>gi|29832308|ref|NP_826942.1| putative aminotransferase [Streptomyces avermitilis MA-4680]
>gi|29832314|ref|NP_826948.1| putative alcohol dehydrogenase [Streptomyces avermitilis MA-4680]
>gi|29832316|ref|NP_826950.1| hypothetical protein SAV5773 [Streptomyces avermitilis MA-4680]
>gi|29832322|ref|NP_826956.1| hypothetical protein SAV5779 [Streptomyces avermitilis MA-4680]
>gi|29832344|ref|NP_826978.1| hypothetical protein SAV5801 [Streptomyces avermitilis MA-4680]
>gi|29832350|ref|NP_826984.1| hypothetical protein SAV5807 [Streptomyces avermitilis MA-4680]
>gi|29832354|ref|NP_826988.1| hypothetical protein SAV5811 [Streptomyces avermitilis MA-4680]
>gi|29832356|ref|NP_826990.1| hypothetical protein SAV5813 [Streptomyces avermitilis MA-4680]
>gi|29832359|ref|NP_826993.1| hypothetical protein SAV5816 [Streptomyces avermitilis MA-4680]
>gi|29832363|ref|NP_826997.1| hypothetical protein SAV5820 [Streptomyces avermitilis MA-4680]
>gi|29832366|ref|NP_827000.1| hypothetical protein SAV5823 [Streptomyces avermitilis MA-4680]
>gi|29832371|ref|NP_827005.1| hypothetical protein SAV5828 [Streptomyces avermitilis MA-4680]
>gi|29832372|ref|NP_827006.1| putative hydrolase [Streptomyces avermitilis MA-4680]
>gi|29832373|ref|NP_827007.1| putative peptidoglycan-binding membrane protein [Streptomyces avermitilis MA-4680]
>gi|29832374|ref|NP_827008.1| putative multidrug-efflux transporter [Streptomyces avermitilis MA-4680]
>gi|29832375|ref|NP_827009.1| putative acetyltransferase [Streptomyces avermitilis MA-4680]
>gi|29832377|ref|NP_827011.1| hypothetical protein SAV5834 [Streptomyces avermitilis MA-4680]
>gi|29832383|ref|NP_827017.1| putative integral membrane protein [Streptomyces avermitilis MA-4680]
>gi|29832388|ref|NP_827022.1| hypothetical protein SAV5845 [Streptomyces avermitilis MA-4680]
>gi|29832389|ref|NP_827023.1| putative integral membrane protein [Streptomyces avermitilis MA-4680]
>gi|29832390|ref|NP_827024.1| putative ABC transporter ATP-binding subunit [Streptomyces avermitilis MA-4680]
>gi|29832391|ref|NP_827025.1| putative integral membrane protein [Streptomyces avermitilis MA-4680]
>gi|29832393|ref|NP_827027.1| hypothetical protein SAV5850 [Streptomyces avermitilis MA-4680]
>gi|29832402|ref|NP_827036.1| hypothetical protein SAV5859 [Streptomyces avermitilis MA-4680]
>gi|29832404|ref|NP_827038.1| hypothetical protein SAV5861 [Streptomyces avermitilis MA-4680]
>gi|29832406|ref|NP_827040.1| putative integral membrane protein [Streptomyces avermitilis MA-4680]
>gi|29832410|ref|NP_827044.1| putative transmembrane transport protein [Streptomyces avermitilis MA-4680]
>gi|29832411|ref|NP_827045.1| putative two-component system response regulator [Streptomyces avermitilis MA-4680]
>gi|29832417|ref|NP_827051.1| hypothetical protein SAV5874 [Streptomyces avermitilis MA-4680]
>gi|29832420|ref|NP_827054.1| putative bifunctional protein (ribonuclease H/phosphoglycerate mutase) [Streptomyces avermitilis MA-4680]

>gi|29832421|ref|NP_827055.1| putative 2-keto-3-deoxygluconate 6-phosphate aldolase and 2-keto-4-hydroxyglutarate aldolase [Streptomyces avermitilis MA-4680]
>gi|29832425|ref|NP_827059.1| putative integral membrane protein [Streptomyces avermitilis MA-4680]
>gi|29832435|ref|NP_827069.1| hypothetical protein SAV5892 [Streptomyces avermitilis MA-4680]
>gi|29832446|ref|NP_827080.1| putative RNA polymerase ECF-subfamily sigma factor [Streptomyces avermitilis MA-4680]
>gi|29832447|ref|NP_827081.1| putative secreted protein [Streptomyces avermitilis MA-4680]
>gi|29832449|ref|NP_827083.1| hypothetical protein SAV5906 [Streptomyces avermitilis MA-4680]
>gi|29832452|ref|NP_827086.1| hypothetical protein SAV5909 [Streptomyces avermitilis MA-4680]
>gi|29832455|ref|NP_827089.1| putative integral membrane protein [Streptomyces avermitilis MA-4680]
>gi|29832456|ref|NP_827090.1| putative D-alanyl-D-alanine carboxypeptidase [Streptomyces avermitilis MA-4680]
>gi|29832460|ref|NP_827094.1| putative bicyclomycin resistance protein [Streptomyces avermitilis MA-4680]
>gi|29832470|ref|NP_827104.1| hypothetical protein SAV5927 [Streptomyces avermitilis MA-4680]
>gi|29832482|ref|NP_827116.1| putative multi-domain regulatory protein [Streptomyces avermitilis MA-4680]
>gi|29832485|ref|NP_827119.1| hypothetical protein SAV5942 [Streptomyces avermitilis MA-4680]
>gi|29832490|ref|NP_827124.1| putative transmembrane efflux protein [Streptomyces avermitilis MA-4680]
>gi|29832502|ref|NP_827136.1| putative LuxR-family transcriptional regulator [Streptomyces avermitilis MA-4680]
>gi|29832503|ref|NP_827137.1| putative ABC transporter integral membrane protein [Streptomyces avermitilis MA-4680]
>gi|29832505|ref|NP_827139.1| hypothetical protein SAV5962 [Streptomyces avermitilis MA-4680]
>gi|29832513|ref|NP_827147.1| putative transmembrane efflux protein [Streptomyces avermitilis MA-4680]
>gi|29832514|ref|NP_827148.1| putative secreted acid phosphatase [Streptomyces avermitilis MA-4680]
>gi|29832516|ref|NP_827150.1| hypothetical protein SAV5973 [Streptomyces avermitilis MA-4680]
>gi|29832517|ref|NP_827151.1| putative LysR-family transcriptional regulator [Streptomyces avermitilis MA-4680]
>gi|29832529|ref|NP_827163.1| hypothetical protein SAV5986 [Streptomyces avermitilis MA-4680]
>gi|29832533|ref|NP_827167.1| putative secreted protein [Streptomyces avermitilis MA-4680]
>gi|29832535|ref|NP_827169.1| putative two-component system sensor kinase [Streptomyces avermitilis MA-4680]
>gi|29832541|ref|NP_827175.1| putative secreted protein [Streptomyces avermitilis MA-4680]
>gi|29832546|ref|NP_827180.1| putative heat shock protein, protease [Streptomyces avermitilis MA-4680]
>gi|29832547|ref|NP_827181.1| hypothetical protein SAV6004 [Streptomyces avermitilis MA-4680]
>gi|29832549|ref|NP_827183.1| putative membrane protein [Streptomyces avermitilis MA-4680]
>gi|29832559|ref|NP_827193.1| hypothetical protein SAV6016 [Streptomyces avermitilis MA-4680]
>gi|29832565|ref|NP_827199.1| putative dihydrolipoamide S-succinyltransferase [Streptomyces avermitilis MA-4680]
>gi|29832568|ref|NP_827202.1| putative cobalamin (5'-phosphate) synthase [Streptomyces avermitilis MA-4680]
>gi|29832569|ref|NP_827203.1| putative integral membrane protein [Streptomyces avermitilis MA-4680]
>gi|29832572|ref|NP_827206.1| putative nicotinate-nucleotide- dimethylbenzimidazole phosphoribosyltransferase [Streptomyces avermitilis MA-4680]
>gi|29832576|ref|NP_827210.1| putative methyltransferase [Streptomyces avermitilis MA-4680]
>gi|29832578|ref|NP_827212.1| putative phage shock protein A [Streptomyces avermitilis MA-4680]
>gi|29832579|ref|NP_827213.1| hypothetical protein SAV6037 [Streptomyces avermitilis MA-4680]
>gi|29832580|ref|NP_827214.1| putative two-component system sensor kinase [Streptomyces avermitilis MA-4680]
>gi|29832601|ref|NP_827235.1| putative integral membrane protein [Streptomyces avermitilis MA-4680]
>gi|29832603|ref|NP_827237.1| hypothetical protein SAV6061 [Streptomyces avermitilis MA-4680]
>gi|29832604|ref|NP_827238.1| putative NLP/P60-family protein [Streptomyces avermitilis MA-4680]
>gi|29832607|ref|NP_827241.1| putative transporter [Streptomyces avermitilis MA-4680]
>gi|29832608|ref|NP_827242.1| putative membrane protein [Streptomyces avermitilis MA-4680]
>gi|29832614|ref|NP_827248.1| putative ion transporting ATPase [Streptomyces avermitilis MA-4680]
>gi|29832615|ref|NP_827249.1| hypothetical protein SAV6073 [Streptomyces avermitilis MA-4680]
>gi|29832620|ref|NP_827254.1| hypothetical protein SAV6078 [Streptomyces avermitilis MA-4680]
>gi|29832623|ref|NP_827257.1| putative two-component system sensor kinase [Streptomyces avermitilis MA-4680]
>gi|29832643|ref|NP_827277.1| putative membrane protein [Streptomyces avermitilis MA-4680]
>gi|29832645|ref|NP_827279.1| putative membrane protein [Streptomyces avermitilis MA-4680]
>gi|29832648|ref|NP_827282.1| putative ABC transporter ATP-binding protein [Streptomyces avermitilis MA-4680]
>gi|29832649|ref|NP_827283.1| hypothetical protein SAV6107 [Streptomyces avermitilis MA-4680]
>gi|29832650|ref|NP_827284.1| putative methyltransferase [Streptomyces avermitilis MA-4680]
>gi|29832652|ref|NP_827286.1| putative membrane protein [Streptomyces avermitilis MA-4680]
>gi|29832657|ref|NP_827291.1| putative membrane protein [Streptomyces avermitilis MA-4680]
>gi|29832660|ref|NP_827294.1| putative UDP-N-acetylmuramoylalanyl-D-glutamyl- 2,6-diaminopimelate-D-alanyl-D-alanine ligase [Streptomyces avermitilis MA-4680]
>gi|29832667|ref|NP_827301.1| hypothetical protein SAV6125 [Streptomyces avermitilis MA-4680]
>gi|29832671|ref|NP_827305.1| hypothetical protein SAV6129 [Streptomyces avermitilis MA-4680]
>gi|29832673|ref|NP_827307.1| putative DNA-binding protein [Streptomyces avermitilis MA-4680]
>gi|29832674|ref|NP_827308.1| putative lipoprotein signal peptidase [Streptomyces avermitilis MA-4680]
>gi|29832677|ref|NP_827311.1| putative sodium/proton antiporter [Streptomyces avermitilis MA-4680]
>gi|29832678|ref|NP_827312.1| putative 3-oxoacyl-ACP reductase [Streptomyces avermitilis MA-4680]
>gi|29832683|ref|NP_827317.1| putative membrane protein [Streptomyces avermitilis MA-4680]
>gi|29832686|ref|NP_827320.1| hypothetical protein SAV6144 [Streptomyces avermitilis MA-4680]
>gi|29832689|ref|NP_827323.1| putative ABC transporter integral membrane transport protein [Streptomyces avermitilis MA-4680]
>gi|29832690|ref|NP_827324.1| putative membrane protein [Streptomyces avermitilis MA-4680]
>gi|29832693|ref|NP_827327.1| hypothetical protein SAV6151 [Streptomyces avermitilis MA-4680]
>gi|29832704|ref|NP_827338.1| putative integral membrane efflux protein [Streptomyces avermitilis MA-4680]
>gi|29832706|ref|NP_827340.1| putative branched-chain amino acid ABC transporter ATP-binding protein [Streptomyces avermitilis MA-4680]

>gi|29832708|ref|NP_827342.1| putative branched-chain amino acid ABC transporter permease protein [Streptomyces avermitilis MA-4680]
>gi|29832709|ref|NP_827343.1| putative branched-chain amino acid ABC transporter permease protein [Streptomyces avermitilis MA-4680]
>gi|29832714|ref|NP_827348.1| putative membrane protein [Streptomyces avermitilis MA-4680]
>gi|29832716|ref|NP_827350.1| hypothetical protein SAV6174 [Streptomyces avermitilis MA-4680]
>gi|29832717|ref|NP_827351.1| putative indole-3-glycerol phosphate synthase [Streptomyces avermitilis MA-4680]
>gi|29832718|ref|NP_827352.1| hypothetical protein SAV6176 [Streptomyces avermitilis MA-4680]
>gi|29832726|ref|NP_827360.1| hypothetical protein SAV6184 [Streptomyces avermitilis MA-4680]
>gi|29832727|ref|NP_827361.1| hypothetical protein SAV6185 [Streptomyces avermitilis MA-4680]
>gi|29832728|ref|NP_827362.1| hypothetical protein SAV6186 [Streptomyces avermitilis MA-4680]
>gi|29832729|ref|NP_827363.1| hypothetical protein SAV6187 [Streptomyces avermitilis MA-4680]
>gi|29832730|ref|NP_827364.1| putative membrane protein [Streptomyces avermitilis MA-4680]
>gi|29832734|ref|NP_827368.1| putative membrane protein [Streptomyces avermitilis MA-4680]
>gi|29832736|ref|NP_827370.1| hypothetical protein SAV6194 [Streptomyces avermitilis MA-4680]
>gi|29832737|ref|NP_827371.1| putative acyl-CoA dehydrogenase [Streptomyces avermitilis MA-4680]
>gi|29832746|ref|NP_827380.1| putative dehydrogenase [Streptomyces avermitilis MA-4680]
>gi|29832747|ref|NP_827381.1| putative dehydrogenase [Streptomyces avermitilis MA-4680]
>gi|29832748|ref|NP_827382.1| hypothetical protein SAV6206 [Streptomyces avermitilis MA-4680]
>gi|29832755|ref|NP_827389.1| putative amino acid decarboxylase [Streptomyces avermitilis MA-4680]
>gi|29832765|ref|NP_827399.1| putative branched-chain amino acid ABC transporter permease protein [Streptomyces avermitilis MA-4680]
>gi|29832770|ref|NP_827404.1| putative membrane protein [Streptomyces avermitilis MA-4680]
>gi|29832771|ref|NP_827405.1| putative secreted protein [Streptomyces avermitilis MA-4680]
>gi|29832773|ref|NP_827407.1| putative ATP-dependent RNA helicase [Streptomyces avermitilis MA-4680]
>gi|29832778|ref|NP_827412.1| hypothetical protein SAV6236 [Streptomyces avermitilis MA-4680]
>gi|29832782|ref|NP_827416.1| hypothetical protein SAV6240 [Streptomyces avermitilis MA-4680]
>gi|29832783|ref|NP_827417.1| hypothetical protein SAV6241 [Streptomyces avermitilis MA-4680]
>gi|29832788|ref|NP_827422.1| hypothetical protein SAV6246 [Streptomyces avermitilis MA-4680]
>gi|29832790|ref|NP_827424.1| putative integral membrane protein [Streptomyces avermitilis MA-4680]
>gi|29832794|ref|NP_827428.1| putative regulatory protein [Streptomyces avermitilis MA-4680]
>gi|29832802|ref|NP_827436.1| hypothetical protein SAV6260 [Streptomyces avermitilis MA-4680]
>gi|29832808|ref|NP_827442.1| putative sugar kinase [Streptomyces avermitilis MA-4680]
>gi|29832817|ref|NP_827451.1| putative export associated protein [Streptomyces avermitilis MA-4680]
>gi|29832819|ref|NP_827453.1| putative ionic transporter integral membrane protein [Streptomyces avermitilis MA-4680]
>gi|29832820|ref|NP_827454.1| putative transmembrane efflux protein [Streptomyces avermitilis MA-4680]
>gi|29832830|ref|NP_827464.1| putative LacI-family transcriptional regulator [Streptomyces avermitilis MA-4680]
>gi|29832843|ref|NP_827477.1| putative antibiotic transport protein [Streptomyces avermitilis MA-4680]
>gi|29832852|ref|NP_827486.1| putative ABC transporter permease protein [Streptomyces avermitilis MA-4680]
>gi|29832853|ref|NP_827487.1| putative 6-phosphogluconolactonase [Streptomyces avermitilis MA-4680]
>gi|29832863|ref|NP_827497.1| putative ABC transporter permease protein [Streptomyces avermitilis MA-4680]
>gi|29832866|ref|NP_827500.1| putative DNA-binding protein [Streptomyces avermitilis MA-4680]
>gi|29832874|ref|NP_827508.1| putative alpha-L-arabinofuranosidase [Streptomyces avermitilis MA-4680]
>gi|57833864|ref|NP_827510.2| putative integral membrane protein [Streptomyces avermitilis MA-4680]
>gi|57833865|ref|NP_827511.2| putative SMR-type multi-drug efflux transporter [Streptomyces avermitilis MA-4680]
>gi|29832887|ref|NP_827521.1| putative integral membrane protein [Streptomyces avermitilis MA-4680]
>gi|29832888|ref|NP_827522.1| hypothetical protein SAV6346 [Streptomyces avermitilis MA-4680]
>gi|29832895|ref|NP_827529.1| putative transport associated protein [Streptomyces avermitilis MA-4680]
>gi|29832904|ref|NP_827538.1| putative integral membrane efflux protein [Streptomyces avermitilis MA-4680]
>gi|29832924|ref|NP_827558.1| putative secreted pectate lyase [Streptomyces avermitilis MA-4680]
>gi|29832926|ref|NP_827560.1| hypothetical protein SAV6384 [Streptomyces avermitilis MA-4680]
>gi|29832927|ref|NP_827561.1| putative RNA polymerase ECF-subfamily sigma factor [Streptomyces avermitilis MA-4680]
>gi|29832933|ref|NP_827567.1| putative drug/proton antiporter [Streptomyces avermitilis MA-4680]
>gi|29832946|ref|NP_827580.1| putative aminotransferase [Streptomyces avermitilis MA-4680]
>gi|29832952|ref|NP_827586.1| putative permease [Streptomyces avermitilis MA-4680]
>gi|29832954|ref|NP_827588.1| putative cobyrinic acid a,c-diamide synthase [Streptomyces avermitilis MA-4680]
>gi|29832956|ref|NP_827590.1| putative magnesium-chelatase subunit [Streptomyces avermitilis MA-4680]
>gi|29832959|ref|NP_827593.1| putative cobalamin biosynthesis protein [Streptomyces avermitilis MA-4680]
>gi|29832960|ref|NP_827594.1| putative secreted protein [Streptomyces avermitilis MA-4680]
>gi|29832965|ref|NP_827599.1| hypothetical protein SAV6423 [Streptomyces avermitilis MA-4680]
>gi|29832967|ref|NP_827601.1| hypothetical protein SAV6425 [Streptomyces avermitilis MA-4680]
>gi|29832971|ref|NP_827605.1| hypothetical protein SAV6429 [Streptomyces avermitilis MA-4680]
>gi|29832973|ref|NP_827607.1| putative membrane protein [Streptomyces avermitilis MA-4680]
>gi|29832975|ref|NP_827609.1| putative membrane protein [Streptomyces avermitilis MA-4680]
>gi|29832976|ref|NP_827610.1| putative membrane protein [Streptomyces avermitilis MA-4680]
>gi|29832977|ref|NP_827611.1| putative dehydrogenase [Streptomyces avermitilis MA-4680]
>gi|29832982|ref|NP_827616.1| putative TetR-family transcriptional regulator [Streptomyces avermitilis MA-4680]
>gi|29832987|ref|NP_827621.1| putative membrane protein [Streptomyces avermitilis MA-4680]
>gi|29832989|ref|NP_827623.1| hypothetical protein SAV6447 [Streptomyces avermitilis MA-4680]
>gi|29832991|ref|NP_827625.1| putative membrane protein [Streptomyces avermitilis MA-4680]
>gi|29832992|ref|NP_827626.1| hypothetical protein SAV6450 [Streptomyces avermitilis MA-4680]
>gi|29832994|ref|NP_827628.1| putative subtilisin-like protease [Streptomyces avermitilis MA-4680]
>gi|29833008|ref|NP_827642.1| putative transmembrane transport protein [Streptomyces avermitilis MA-4680]

>gi|29833010|ref|NP_827644.1| hypothetical protein SAV6468 [Streptomyces avermitilis MA-4680]
>gi|29833013|ref|NP_827647.1| putative membrane protein [Streptomyces avermitilis MA-4680]
>gi|29833015|ref|NP_827649.1| hypothetical protein SAV6473 [Streptomyces avermitilis MA-4680]
>gi|29833017|ref|NP_827651.1| putative dehydrogenase [Streptomyces avermitilis MA-4680]
>gi|29833018|ref|NP_827652.1| putative two-component system sensor kinase [Streptomyces avermitilis MA-4680]
>gi|29833020|ref|NP_827654.1| putative secreted protein [Streptomyces avermitilis MA-4680]
>gi|29833021|ref|NP_827655.1| hypothetical protein SAV6479 [Streptomyces avermitilis MA-4680]
>gi|29833023|ref|NP_827657.1| putative membrane protein [Streptomyces avermitilis MA-4680]
>gi|29833029|ref|NP_827663.1| hypothetical protein SAV6487 [Streptomyces avermitilis MA-4680]
>gi|29833030|ref|NP_827664.1| hypothetical protein SAV6488 [Streptomyces avermitilis MA-4680]
>gi|29833033|ref|NP_827667.1| putative ferrichrome ABC transport system permease protein [Streptomyces avermitilis MA-4680]
>gi|29833034|ref|NP_827668.1| putative ferrichrome ABC transport system permease protein [Streptomyces avermitilis MA-4680]
>gi|29833037|ref|NP_827671.1| hypothetical protein SAV6495 [Streptomyces avermitilis MA-4680]
>gi|29833040|ref|NP_827674.1| putative membrane protein [Streptomyces avermitilis MA-4680]
>gi|29833041|ref|NP_827675.1| putative DNA recombination and repair protein [Streptomyces avermitilis MA-4680]
>gi|29833042|ref|NP_827676.1| hypothetical protein SAV6500 [Streptomyces avermitilis MA-4680]
>gi|29833044|ref|NP_827678.1| putative regulatory protein [Streptomyces avermitilis MA-4680]
>gi|29833048|ref|NP_827682.1| putative regulatory protein [Streptomyces avermitilis MA-4680]
>gi|29833051|ref|NP_827685.1| hypothetical protein SAV6509 [Streptomyces avermitilis MA-4680]
>gi|29833059|ref|NP_827693.1| putative iron sulphur protein [Streptomyces avermitilis MA-4680]
>gi|29833069|ref|NP_827703.1| putative peptidoglycan-binding protein [Streptomyces avermitilis MA-4680]
>gi|29833070|ref|NP_827704.1| putative peptidoglycan-binding protein [Streptomyces avermitilis MA-4680]
>gi|29833071|ref|NP_827705.1| putative ABC transporter ATP-binding protein [Streptomyces avermitilis MA-4680]
>gi|29833072|ref|NP_827706.1| putative ABC transporter ATP-binding protein [Streptomyces avermitilis MA-4680]
>gi|57833866|ref|YP_187529.1| putative ABC transporter permease protein [Streptomyces avermitilis MA-4680]
>gi|29833073|ref|NP_827707.1| hypothetical protein SAV6531 [Streptomyces avermitilis MA-4680]
>gi|29833081|ref|NP_827715.1| putative integral membrane protein [Streptomyces avermitilis MA-4680]
>gi|29833082|ref|NP_827716.1| putative integral membrane protein [Streptomyces avermitilis MA-4680]
>gi|29833083|ref|NP_827717.1| putative multidrug efflux protein [Streptomyces avermitilis MA-4680]
>gi|29833086|ref|NP_827720.1| putative regulatory protein [Streptomyces avermitilis MA-4680]
>gi|29833088|ref|NP_827722.1| putative ABC transporter permease [Streptomyces avermitilis MA-4680]
>gi|29833096|ref|NP_827730.1| hypothetical protein SAV6554 [Streptomyces avermitilis MA-4680]
>gi|29833102|ref|NP_827736.1| putative cellulose-binding protein [Streptomyces avermitilis MA-4680]
>gi|29833104|ref|NP_827738.1| hypothetical protein SAV6562 [Streptomyces avermitilis MA-4680]
>gi|29833106|ref|NP_827740.1| hypothetical protein SAV6564 [Streptomyces avermitilis MA-4680]
>gi|29833108|ref|NP_827742.1| putative GntR-family transcriptional regulator [Streptomyces avermitilis MA-4680]
>gi|29833112|ref|NP_827746.1| putative membrane protein [Streptomyces avermitilis MA-4680]
>gi|29833117|ref|NP_827751.1| hypothetical protein SAV6575 [Streptomyces avermitilis MA-4680]
>gi|29833120|ref|NP_827754.1| putative serine/threonine protein kinase [Streptomyces avermitilis MA-4680]
>gi|29833121|ref|NP_827755.1| hypothetical protein SAV6579 [Streptomyces avermitilis MA-4680]
>gi|29833122|ref|NP_827756.1| hypothetical protein SAV6580 [Streptomyces avermitilis MA-4680]
>gi|29833125|ref|NP_827759.1| putative membrane protein [Streptomyces avermitilis MA-4680]
>gi|29833130|ref|NP_827764.1| putative secreted protein [Streptomyces avermitilis MA-4680]
>gi|29833134|ref|NP_827768.1| putative integral membrane transport protein [Streptomyces avermitilis MA-4680]
>gi|29833135|ref|NP_827769.1| hypothetical protein SAV6593 [Streptomyces avermitilis MA-4680]
>gi|29833139|ref|NP_827773.1| hypothetical protein SAV6597 [Streptomyces avermitilis MA-4680]
>gi|29833140|ref|NP_827774.1| putative ArsR-family transcriptional regulator [Streptomyces avermitilis MA-4680]
>gi|29833159|ref|NP_827793.1| putative membrane protein [Streptomyces avermitilis MA-4680]
>gi|29833160|ref|NP_827794.1| putative membrane protein [Streptomyces avermitilis MA-4680]
>gi|29833168|ref|NP_827802.1| putative L-idonate 5-dehydrogenase [Streptomyces avermitilis MA-4680]
>gi|29833170|ref|NP_827804.1| putative gluconate permease [Streptomyces avermitilis MA-4680]
>gi|29833177|ref|NP_827811.1| putative secreted protein [Streptomyces avermitilis MA-4680]
>gi|29833178|ref|NP_827812.1| putative secreted protein [Streptomyces avermitilis MA-4680]
>gi|29833181|ref|NP_827815.1| hypothetical protein SAV6639 [Streptomyces avermitilis MA-4680]
>gi|29833190|ref|NP_827824.1| hypothetical protein SAV6648 [Streptomyces avermitilis MA-4680]
>gi|29833196|ref|NP_827830.1| putative sugar ABC transporter permease protein [Streptomyces avermitilis MA-4680]
>gi|29833197|ref|NP_827831.1| putative ABC transporter permease [Streptomyces avermitilis MA-4680]
>gi|29833217|ref|NP_827851.1| hypothetical protein SAV6675 [Streptomyces avermitilis MA-4680]
>gi|29833233|ref|NP_827867.1| hypothetical protein SAV6691 [Streptomyces avermitilis MA-4680]
>gi|29833236|ref|NP_827870.1| hypothetical protein SAV6694 [Streptomyces avermitilis MA-4680]
>gi|29833243|ref|NP_827877.1| putative TetR-family transcriptional regulator [Streptomyces avermitilis MA-4680]
>gi|29833244|ref|NP_827878.1| putative sensor-like histidine kinase [Streptomyces avermitilis MA-4680]
>gi|29833259|ref|NP_827893.1| hypothetical protein SAV6717 [Streptomyces avermitilis MA-4680]
>gi|29833263|ref|NP_827897.1| putative integral membrane protein [Streptomyces avermitilis MA-4680]
>gi|29833264|ref|NP_827898.1| putative LysR-family transcriptional regulator [Streptomyces avermitilis MA-4680]
>gi|29833271|ref|NP_827905.1| putative membrane protein [Streptomyces avermitilis MA-4680]
>gi|29833273|ref|NP_827907.1| hypothetical protein SAV6731 [Streptomyces avermitilis MA-4680]
>gi|29833276|ref|NP_827910.1| putative secreted protein [Streptomyces avermitilis MA-4680]
>gi|29833277|ref|NP_827911.1| hypothetical protein SAV6735 [Streptomyces avermitilis MA-4680]
>gi|29833282|ref|NP_827916.1| putative rRNA methyltransferase [Streptomyces avermitilis MA-4680]

>gi|29833293|ref|NP_827927.1| putative integral membrane protein [Streptomyces avermitilis MA-4680]
>gi|29833296|ref|NP_827930.1| hypothetical protein SAV6754 [Streptomyces avermitilis MA-4680]
>gi|29833311|ref|NP_827945.1| hypothetical protein SAV6769 [Streptomyces avermitilis MA-4680]
>gi|29833313|ref|NP_827947.1| hypothetical protein SAV6771 [Streptomyces avermitilis MA-4680]
>gi|29833319|ref|NP_827953.1| hypothetical protein SAV6777 [Streptomyces avermitilis MA-4680]
>gi|29833323|ref|NP_827957.1| putative TetR-family transcriptional regulator [Streptomyces avermitilis MA-4680]
>gi|29833324|ref|NP_827958.1| putative multidrug resistance efflux protein [Streptomyces avermitilis MA-4680]
>gi|29833327|ref|NP_827961.1| putative RNA polymerase ECF-subfamily sigma factor [Streptomyces avermitilis MA-4680]
>gi|29833336|ref|NP_827970.1| putative precorrin-6Y C5,15-methyltransferase [Streptomyces avermitilis MA-4680]
>gi|29833337|ref|NP_827971.1| putative nicotinate-nucleotide- dimethylbenzimidazole phosphoribosyltransferase [Streptomyces avermitilis MA-4680]
>gi|29833341|ref|NP_827975.1| putative serine/threonine protein kinase [Streptomyces avermitilis MA-4680]
>gi|29833342|ref|NP_827976.1| putative membrane protein [Streptomyces avermitilis MA-4680]
>gi|29833343|ref|NP_827977.1| hypothetical protein SAV6801 [Streptomyces avermitilis MA-4680]
>gi|29833344|ref|NP_827978.1| hypothetical protein SAV6802 [Streptomyces avermitilis MA-4680]
>gi|29833348|ref|NP_827982.1| hypothetical protein SAV6806 [Streptomyces avermitilis MA-4680]
>gi|29833349|ref|NP_827983.1| hypothetical protein SAV6807 [Streptomyces avermitilis MA-4680]
>gi|29833350|ref|NP_827984.1| hypothetical protein SAV6808 [Streptomyces avermitilis MA-4680]
>gi|29833357|ref|NP_827991.1| hypothetical protein SAV6815 [Streptomyces avermitilis MA-4680]
>gi|29833359|ref|NP_827993.1| putative integral membrane protein [Streptomyces avermitilis MA-4680]
>gi|29833371|ref|NP_828005.1| putative secreted protein [Streptomyces avermitilis MA-4680]
>gi|29833375|ref|NP_828009.1| putative Holliday junction nuclease [Streptomyces avermitilis MA-4680]
>gi|29833378|ref|NP_828012.1| putative secreted protein [Streptomyces avermitilis MA-4680]
>gi|29833379|ref|NP_828013.1| putative protein-export membrane protein [Streptomyces avermitilis MA-4680]
>gi|29833384|ref|NP_828018.1| putative peptidyl-prolyl cis-trans isomerase [Streptomyces avermitilis MA-4680]
>gi|29833390|ref|NP_828024.1| putative secreted protein [Streptomyces avermitilis MA-4680]
>gi|29833394|ref|NP_828028.1| putative aminodeoxychorismate lyase [Streptomyces avermitilis MA-4680]
>gi|29833399|ref|NP_828033.1| hypothetical protein SAV6857 [Streptomyces avermitilis MA-4680]
>gi|29833417|ref|NP_828051.1| putative primosomal protein [Streptomyces avermitilis MA-4680]
>gi|29833418|ref|NP_828052.1| hypothetical protein SAV6876 [Streptomyces avermitilis MA-4680]
>gi|29833420|ref|NP_828054.1| putative RNA-binding Sun protein [Streptomyces avermitilis MA-4680]
>gi|29833431|ref|NP_828065.1| putative sensor-like histidine kinase [Streptomyces avermitilis MA-4680]
>gi|29833438|ref|NP_828072.1| putative endo-1,4-beta-glucanase [Streptomyces avermitilis MA-4680]
>gi|29833443|ref|NP_828077.1| putative ROK-family transcriptional regulator [Streptomyces avermitilis MA-4680]
>gi|29833455|ref|NP_828089.1| hypothetical protein SAV6913 [Streptomyces avermitilis MA-4680]
>gi|29833466|ref|NP_828100.1| putative membrane protein [Streptomyces avermitilis MA-4680]
>gi|29833475|ref|NP_828109.1| hypothetical protein SAV6933 [Streptomyces avermitilis MA-4680]
>gi|29833478|ref|NP_828112.1| hypothetical protein SAV6936 [Streptomyces avermitilis MA-4680]
>gi|29833479|ref|NP_828113.1| putative antagonist of KipI [Streptomyces avermitilis MA-4680]
>gi|29833487|ref|NP_828121.1| hypothetical protein SAV6945 [Streptomyces avermitilis MA-4680]
>gi|29833498|ref|NP_828132.1| putative ferredoxin reductase [Streptomyces avermitilis MA-4680]
>gi|29833499|ref|NP_828133.1| putative membrane protein [Streptomyces avermitilis MA-4680]
>gi|29833506|ref|NP_828140.1| putative glycerol uptake facilitator protein [Streptomyces avermitilis MA-4680]
>gi|29833507|ref|NP_828141.1| hypothetical protein SAV6965 [Streptomyces avermitilis MA-4680]
>gi|29833514|ref|NP_828148.1| putative membrane transport protein [Streptomyces avermitilis MA-4680]
>gi|29833521|ref|NP_828155.1| putative membrane protein [Streptomyces avermitilis MA-4680]
>gi|29833527|ref|NP_828161.1| putative DNA damage inducible protein, DNA polymerase IV [Streptomyces avermitilis MA-4680]
>gi|29833536|ref|NP_828170.1| putative PadR-like family transcriptional regulator [Streptomyces avermitilis MA-4680]
>gi|29833538|ref|NP_828172.1| hypothetical protein SAV6996 [Streptomyces avermitilis MA-4680]
>gi|29833540|ref|NP_828174.1| hypothetical protein SAV6998 [Streptomyces avermitilis MA-4680]
>gi|29833547|ref|NP_828181.1| putative iron sulphur protein [Streptomyces avermitilis MA-4680]
>gi|29833548|ref|NP_828182.1| hypothetical protein SAV7006 [Streptomyces avermitilis MA-4680]
>gi|29833554|ref|NP_828188.1| hypothetical protein SAV7012 [Streptomyces avermitilis MA-4680]
>gi|29833555|ref|NP_828189.1| putative lipoprotein [Streptomyces avermitilis MA-4680]
>gi|29833559|ref|NP_828193.1| hypothetical protein SAV7017 [Streptomyces avermitilis MA-4680]
>gi|29833566|ref|NP_828200.1| hypothetical protein SAV7024 [Streptomyces avermitilis MA-4680]
>gi|29833569|ref|NP_828203.1| hypothetical protein SAV7027 [Streptomyces avermitilis MA-4680]
>gi|29833572|ref|NP_828206.1| putative transmembrane efflux protein [Streptomyces avermitilis MA-4680]
>gi|29833573|ref|NP_828207.1| hypothetical protein SAV7031 [Streptomyces avermitilis MA-4680]
>gi|29833574|ref|NP_828208.1| putative membrane protein [Streptomyces avermitilis MA-4680]
>gi|29833577|ref|NP_828211.1| putative sodium:solute symporter [Streptomyces avermitilis MA-4680]
>gi|29833578|ref|NP_828212.1| hypothetical protein SAV7036 [Streptomyces avermitilis MA-4680]
>gi|29833580|ref|NP_828214.1| putative siderophore binding protein [Streptomyces avermitilis MA-4680]
>gi|29833581|ref|NP_828215.1| putative sugar acetyltransferase [Streptomyces avermitilis MA-4680]
>gi|29833582|ref|NP_828216.1| putative membrane protein [Streptomyces avermitilis MA-4680]
>gi|29833585|ref|NP_828219.1| putative cation efflux system protein [Streptomyces avermitilis MA-4680]
>gi|29833586|ref|NP_828220.1| putative ArsR-family transcriptional regulator [Streptomyces avermitilis MA-4680]
>gi|29833590|ref|NP_828224.1| putative cation efflux system protein [Streptomyces avermitilis MA-4680]
>gi|29833592|ref|NP_828226.1| hypothetical protein SAV7050 [Streptomyces avermitilis MA-4680]
>gi|29833593|ref|NP_828227.1| hypothetical protein SAV7051 [Streptomyces avermitilis MA-4680]

>gi|29833598|ref|NP_828232.1| putative exonuclease [Streptomyces avermitilis MA-4680]
>gi|29833600|ref|NP_828234.1| hypothetical protein SAV7058 [Streptomyces avermitilis MA-4680]
>gi|29833607|ref|NP_828241.1| putative GntR-family transcriptional regulator [Streptomyces avermitilis MA-4680]
>gi|29833608|ref|NP_828242.1| putative integral membrane protein [Streptomyces avermitilis MA-4680]
>gi|29833612|ref|NP_828246.1| putative ROK-family transcriptional regulator [Streptomyces avermitilis MA-4680]
>gi|29833616|ref|NP_828250.1| putative ABC transporter integral membrane protein [Streptomyces avermitilis MA-4680]
>gi|29833626|ref|NP_828260.1| putative ABC transporter integral membrane protein [Streptomyces avermitilis MA-4680]
>gi|29833632|ref|NP_828266.1| putative lpsA1 transcriptional activator [Streptomyces avermitilis MA-4680]
>gi|29833633|ref|NP_828267.1| putative dethiobiotin synthetase [Streptomyces avermitilis MA-4680]
>gi|29833636|ref|NP_828270.1| putative 8-amino-7-oxononanoate synthase [Streptomyces avermitilis MA-4680]
>gi|29833640|ref|NP_828274.1| hypothetical protein SAV7098 [Streptomyces avermitilis MA-4680]
>gi|29833641|ref|NP_828275.1| putative NLP/P60-family protein [Streptomyces avermitilis MA-4680]
>gi|29833645|ref|NP_828279.1| hypothetical protein SAV7103 [Streptomyces avermitilis MA-4680]
>gi|29833649|ref|NP_828283.1| putative urease accessroy protein [Streptomyces avermitilis MA-4680]
>gi|29833658|ref|NP_828292.1| putative membrane protein [Streptomyces avermitilis MA-4680]
>gi|29833659|ref|NP_828293.1| putative transmembrane transport protein [Streptomyces avermitilis MA-4680]
>gi|29833662|ref|NP_828296.1| putative serine/threonine protein kinase [Streptomyces avermitilis MA-4680]
>gi|29833663|ref|NP_828297.1| hypothetical protein SAV7121 [Streptomyces avermitilis MA-4680]
>gi|29833681|ref|NP_828315.1| putative dehydrogenase [Streptomyces avermitilis MA-4680]
>gi|29833683|ref|NP_828317.1| putative acyl-CoA dehydrogenase [Streptomyces avermitilis MA-4680]
>gi|29833718|ref|NP_828352.1| hypothetical protein SAV7176 [Streptomyces avermitilis MA-4680]
>gi|29833720|ref|NP_828354.1| putative transcriptional regulator [Streptomyces avermitilis MA-4680]
>gi|29833722|ref|NP_828356.1| putative xylose repressor, ROK-family transcriptional regulator [Streptomyces avermitilis MA-4680]
>gi|57833867|ref|NP_828357.2| putative xylulose kinase [Streptomyces avermitilis MA-4680]
>gi|29833725|ref|NP_828359.1| hypothetical protein SAV7183 [Streptomyces avermitilis MA-4680]
>gi|29833729|ref|NP_828363.1| putative transmembrane efflux protein [Streptomyces avermitilis MA-4680]
>gi|29833730|ref|NP_828364.1| putative oxidoreductase [Streptomyces avermitilis MA-4680]
>gi|29833736|ref|NP_828370.1| putative methyltransferas [Streptomyces avermitilis MA-4680]
>gi|29833740|ref|NP_828374.1| putative hydrolase [Streptomyces avermitilis MA-4680]
>gi|29833742|ref|NP_828376.1| putative transport integral membrane protein [Streptomyces avermitilis MA-4680]
>gi|29833746|ref|NP_828380.1| hypothetical protein SAV7204 [Streptomyces avermitilis MA-4680]
>gi|29833748|ref|NP_828382.1| putative cytosine permease [Streptomyces avermitilis MA-4680]
>gi|29833759|ref|NP_828393.1| putative membrane protein [Streptomyces avermitilis MA-4680]
>gi|29833760|ref|NP_828394.1| putative ABC transporter ATP-binding protein [Streptomyces avermitilis MA-4680]
>gi|29833763|ref|NP_828397.1| hypothetical protein SAV7221 [Streptomyces avermitilis MA-4680]
>gi|29833766|ref|NP_828400.1| hypothetical protein SAV7224 [Streptomyces avermitilis MA-4680]
>gi|29833770|ref|NP_828404.1| hypothetical protein SAV7228 [Streptomyces avermitilis MA-4680]
>gi|29833776|ref|NP_828410.1| putative multidrug efflux protein [Streptomyces avermitilis MA-4680]
>gi|29833780|ref|NP_828414.1| putative GrpE homologue [Streptomyces avermitilis MA-4680]
>gi|29833781|ref|NP_828415.1| putative heat shock protein DnaJ [Streptomyces avermitilis MA-4680]
>gi|29833788|ref|NP_828422.1| hypothetical protein SAV7246 [Streptomyces avermitilis MA-4680]
>gi|29833789|ref|NP_828423.1| hypothetical protein SAV7247 [Streptomyces avermitilis MA-4680]
>gi|29833790|ref|NP_828424.1| hypothetical protein SAV7248 [Streptomyces avermitilis MA-4680]
>gi|29833793|ref|NP_828427.1| hypothetical protein SAV7251 [Streptomyces avermitilis MA-4680]
>gi|29833801|ref|NP_828435.1| putative 3-oxoacyl-ACP reductase [Streptomyces avermitilis MA-4680]
>gi|29833802|ref|NP_828436.1| hypothetical protein SAV7260 [Streptomyces avermitilis MA-4680]
>gi|29833811|ref|NP_828445.1| putative amidase [Streptomyces avermitilis MA-4680]
>gi|29833837|ref|NP_828471.1| hypothetical protein SAV7295 [Streptomyces avermitilis MA-4680]
>gi|29833841|ref|NP_828475.1| putative glycine-rich protein [Streptomyces avermitilis MA-4680]
>gi|29833843|ref|NP_828477.1| putative proline-rich protein [Streptomyces avermitilis MA-4680]
>gi|29833844|ref|NP_828478.1| putative lipoprotein [Streptomyces avermitilis MA-4680]
>gi|29833847|ref|NP_828481.1| putative dehydrogenase [Streptomyces avermitilis MA-4680]
>gi|29833853|ref|NP_828487.1| putative 1-acylglycerol-3-phosphate O-acyltransferase [Streptomyces avermitilis MA-4680]
>gi|29833868|ref|NP_828502.1| putative transcriptional regulator [Streptomyces avermitilis MA-4680]
>gi|29833873|ref|NP_828507.1| putative LuxR-family transcriptional regulator [Streptomyces avermitilis MA-4680]
>gi|29833875|ref|NP_828509.1| putative integral membrane protein [Streptomyces avermitilis MA-4680]
>gi|29833877|ref|NP_828511.1| putative integral membrane protein [Streptomyces avermitilis MA-4680]
>gi|29833884|ref|NP_828518.1| hypothetical protein SAV7342 [Streptomyces avermitilis MA-4680]
>gi|29833886|ref|NP_828520.1| putative membrane protein [Streptomyces avermitilis MA-4680]
>gi|29833887|ref|NP_828521.1| putative amidase [Streptomyces avermitilis MA-4680]
>gi|29833895|ref|NP_828529.1| hypothetical protein SAV7353 [Streptomyces avermitilis MA-4680]
>gi|29833899|ref|NP_828533.1| putative ABC transporter ATP-binding protein [Streptomyces avermitilis MA-4680]
>gi|29833904|ref|NP_828538.1| putative modular polyketide synthase [Streptomyces avermitilis MA-4680]
>gi|29833910|ref|NP_828544.1| hypothetical protein SAV7368 [Streptomyces avermitilis MA-4680]
>gi|29833916|ref|NP_828550.1| putative [NiFe] hydrogenase expression/formation protein [Streptomyces avermitilis MA-4680]
>gi|29833920|ref|NP_828554.1| putative [NiFe] hydrogenase expression/formation protein [Streptomyces avermitilis MA-4680]
>gi|29833921|ref|NP_828555.1| hypothetical protein SAV7379 [Streptomyces avermitilis MA-4680]
>gi|29833922|ref|NP_828556.1| hypothetical protein SAV7380 [Streptomyces avermitilis MA-4680]
>gi|29833923|ref|NP_828557.1| hypothetical protein SAV7381 [Streptomyces avermitilis MA-4680]
>gi|29833924|ref|NP_828558.1| putative oxidoreductase iron-sulphur binding subunit [Streptomyces avermitilis MA-4680]

>gi|29833931|ref|NP_828565.1| putative sodium/proton antiporter [Streptomyces avermitilis MA-4680]
>gi|29833933|ref|NP_828567.1| putative two-component system sensor kinase [Streptomyces avermitilis MA-4680]
>gi|29833934|ref|NP_828568.1| putative two-component system response regulator [Streptomyces avermitilis MA-4680]
>gi|29833936|ref|NP_828570.1| hypothetical protein SAV7394 [Streptomyces avermitilis MA-4680]
>gi|29833948|ref|NP_828582.1| putative ABC transporter permease [Streptomyces avermitilis MA-4680]
>gi|29833949|ref|NP_828583.1| putative ABC transporter permease [Streptomyces avermitilis MA-4680]
>gi|29833959|ref|NP_828593.1| putative ABC transporter permease [Streptomyces avermitilis MA-4680]
>gi|29833960|ref|NP_828594.1| putative ABC transporter permease [Streptomyces avermitilis MA-4680]
>gi|29833967|ref|NP_828601.1| hypothetical protein SAV7425 [Streptomyces avermitilis MA-4680]
>gi|29833969|ref|NP_828603.1| putative TetR-family transcriptional regulator [Streptomyces avermitilis MA-4680]
>gi|29833972|ref|NP_828606.1| putative integral membrane protein [Streptomyces avermitilis MA-4680]
>gi|29833973|ref|NP_828607.1| putative integral membrane protein [Streptomyces avermitilis MA-4680]
>gi|29833974|ref|NP_828608.1| hypothetical protein SAV7432 [Streptomyces avermitilis MA-4680]
>gi|29833977|ref|NP_828611.1| hypothetical protein SAV7435 [Streptomyces avermitilis MA-4680]
>gi|29833985|ref|NP_828619.1| putative integral membrane protein [Streptomyces avermitilis MA-4680]
>gi|29833986|ref|NP_828620.1| putative membrane protein [Streptomyces avermitilis MA-4680]
>gi|29833987|ref|NP_828621.1| putative membrane protein [Streptomyces avermitilis MA-4680]
>gi|29833989|ref|NP_828623.1| putative dehydrogenase [Streptomyces avermitilis MA-4680]
>gi|29833990|ref|NP_828624.1| putative NADH-dehydrogenase [Streptomyces avermitilis MA-4680]
>gi|29833999|ref|NP_828633.1| putative TetR-family transcriptional regulator [Streptomyces avermitilis MA-4680]
>gi|29834008|ref|NP_828642.1| hypothetical protein SAV7466 [Streptomyces avermitilis MA-4680]
>gi|29834017|ref|NP_828651.1| putative hydrolase [Streptomyces avermitilis MA-4680]
>gi|29834018|ref|NP_828652.1| putative hydrolase [Streptomyces avermitilis MA-4680]
>gi|29834019|ref|NP_828653.1| putative membrane efflux protein [Streptomyces avermitilis MA-4680]
>gi|29834022|ref|NP_828656.1| putative hydrolase [Streptomyces avermitilis MA-4680]
>gi|29834027|ref|NP_828661.1| hypothetical protein SAV7485 [Streptomyces avermitilis MA-4680]
>gi|29834028|ref|NP_828662.1| putative subtilisin inhibitor [Streptomyces avermitilis MA-4680]
>gi|29834031|ref|NP_828665.1| hypothetical protein SAV7489 [Streptomyces avermitilis MA-4680]
>gi|29834037|ref|NP_828671.1| hypothetical protein SAV7495 [Streptomyces avermitilis MA-4680]
>gi|29834039|ref|NP_828673.1| putative protease precursor [Streptomyces avermitilis MA-4680]
>gi|29834042|ref|NP_828676.1| putative ABC transporter ATP-binding protein, AmfA [Streptomyces avermitilis MA-4680]
>gi|29834043|ref|NP_828677.1| putative ABC transporter ATP-binding membrane translocator, AmfB [Streptomyces avermitilis MA-4680]
>gi|29834052|ref|NP_828686.1| putative TetR-family transcriptional regulator [Streptomyces avermitilis MA-4680]
>gi|29834057|ref|NP_828691.1| hypothetical protein SAV7515 [Streptomyces avermitilis MA-4680]
>gi|29834058|ref|NP_828692.1| putative mobile element transfer protein SpdB [Streptomyces avermitilis MA-4680]
>gi|29834061|ref|NP_828695.1| hypothetical protein SAV7519 [Streptomyces avermitilis MA-4680]
>gi|29834063|ref|NP_828697.1| putative plasmid transfer protein [Streptomyces avermitilis MA-4680]
>gi|29834064|ref|NP_828698.1| hypothetical protein SAV7522 [Streptomyces avermitilis MA-4680]
>gi|29834067|ref|NP_828701.1| hypothetical protein SAV7525 [Streptomyces avermitilis MA-4680]
>gi|29834078|ref|NP_828712.1| hypothetical protein SAV7536 [Streptomyces avermitilis MA-4680]
>gi|29834083|ref|NP_828717.1| hypothetical protein SAV7541 [Streptomyces avermitilis MA-4680]
>gi|29834086|ref|NP_828720.1| hypothetical protein SAV7544 [Streptomyces avermitilis MA-4680]
>gi|29834105|ref|NP_828739.1| hypothetical protein SAV7563 [Streptomyces avermitilis MA-4680]
>gi|29826444|ref|NP_828750.1| hypothetical protein SAP1p01 [Streptomyces avermitilis MA-4680]
>gi|29826449|ref|NP_828755.1| hypothetical protein SAP1p06 [Streptomyces avermitilis MA-4680]
>gi|29826452|ref|NP_828758.1| hypothetical protein SAP1p09 [Streptomyces avermitilis MA-4680]
>gi|29826453|ref|NP_828759.1| hypothetical protein SAP1p10 [Streptomyces avermitilis MA-4680]
>gi|29826467|ref|NP_828773.1| hypothetical protein SAP1p24 [Streptomyces avermitilis MA-4680]
>gi|29826470|ref|NP_828776.1| hypothetical protein SAP1p27 [Streptomyces avermitilis MA-4680]
>gi|29826471|ref|NP_828777.1| hypothetical protein SAP1p28 [Streptomyces avermitilis MA-4680]
>gi|29826475|ref|NP_828781.1| putative Tra3 protein [Streptomyces avermitilis MA-4680]
>gi|29826476|ref|NP_828782.1| hypothetical protein SAP1p33 [Streptomyces avermitilis MA-4680]
>gi|29826478|ref|NP_828784.1| hypothetical protein SAP1p35 [Streptomyces avermitilis MA-4680]
>gi|29826482|ref|NP_828788.1| hypothetical protein SAP1p39 [Streptomyces avermitilis MA-4680]
>gi|29826487|ref|NP_828793.1| putative large alanine-rich protein [Streptomyces avermitilis MA-4680]
>gi|29826490|ref|NP_828796.1| putative ATP/GTP-binding Gly/Ala-rich protein [Streptomyces avermitilis MA-4680]
>gi|29826499|ref|NP_828805.1| hypothetical protein SAP1p56 [Streptomyces avermitilis MA-4680]
>gi|29826506|ref|NP_828812.1| hypothetical protein SAP1p63 [Streptomyces avermitilis MA-4680]
>gi|29826509|ref|NP_828815.1| hypothetical protein SAP1p66 [Streptomyces avermitilis MA-4680]
>gi|29826519|ref|NP_828825.1| hypothetical protein SAP1p76 [Streptomyces avermitilis MA-4680]
>gi|29826520|ref|NP_828826.1| putative MarR-family transcriptional regulatory protein [Streptomyces avermitilis MA-4680]
>gi|29826522|ref|NP_828828.1| hypothetical protein SAP1p79 [Streptomyces avermitilis MA-4680]
>gi|29826527|ref|NP_828833.1| hypothetical protein SAP1p84 [Streptomyces avermitilis MA-4680]
>gi|49473697|ref|YP_031739.1| Lipoprotein signal peptidase [Bartonella quintana str. Toulouse]
>gi|49473772|ref|YP_031814.1| hypothetical protein BQ00890 [Bartonella quintana str. Toulouse]
>gi|49473776|ref|YP_031818.1| hypothetical protein BQ00960 [Bartonella quintana str. Toulouse]
>gi|49473784|ref|YP_031826.1| Heme exporter protein B [Bartonella quintana str. Toulouse]
>gi|49473802|ref|YP_031844.1| 30s ribosomal protein s21 [Bartonella quintana str. Toulouse]
>gi|49473809|ref|YP_031851.1| hypothetical protein BQ01360 [Bartonella quintana str. Toulouse]
>gi|49473845|ref|YP_031887.1| ABC transporter, permease protein [Bartonella quintana str. Toulouse]

>gi|49473905|ref|YP_031947.1| Hemin binding protein b [Bartonella quintana str. Toulouse]
>gi|49473926|ref|YP_031968.1| Biotin synthase [Bartonella quintana str. Toulouse]
>gi|49473937|ref|YP_031979.1| Large-conductance mechanosensitive channel [Bartonella quintana str. Toulouse]
>gi|49473958|ref|YP_032000.1| hypothetical protein BQ03120 [Bartonella quintana str. Toulouse]
>gi|49473962|ref|YP_032004.1| ATP synthase B chain [Bartonella quintana str. Toulouse]
>gi|49473985|ref|YP_032027.1| hypothetical protein BQ03410 [Bartonella quintana str. Toulouse]
>gi|49474028|ref|YP_032070.1| hypothetical protein BQ03900 [Bartonella quintana str. Toulouse]
>gi|49474068|ref|YP_032110.1| DNA repair protein recO [Bartonella quintana str. Toulouse]
>gi|49474082|ref|YP_032124.1| Colicin v production protein [Bartonella quintana str. Toulouse]
>gi|49474116|ref|YP_032158.1| hypothetical protein BQ04800 [Bartonella quintana str. Toulouse]
>gi|49474121|ref|YP_032163.1| Protein-export membrane protein [Bartonella quintana str. Toulouse]
>gi|49474186|ref|YP_032228.1| NADH dehydrogenase I, J subunit [Bartonella quintana str. Toulouse]
>gi|49474265|ref|YP_032307.1| hypothetical protein BQ06660 [Bartonella quintana str. Toulouse]
>gi|49474308|ref|YP_032350.1| 50s ribosomal protein l7 /l12 [Bartonella quintana str. Toulouse]
>gi|49474440|ref|YP_032482.1| hypothetical protein BQ08690 [Bartonella quintana str. Toulouse]
>gi|49474479|ref|YP_032521.1| hypothetical protein BQ09200 [Bartonella quintana str. Toulouse]
>gi|49474512|ref|YP_032554.1| ABC transporter, permease protein [Bartonella quintana str. Toulouse]
>gi|49474565|ref|YP_032607.1| Ferric anguibactin transport system permease protein [Bartonella quintana str. Toulouse]
>gi|49474575|ref|YP_032617.1| hypothetical protein BQ10500 [Bartonella quintana str. Toulouse]
>gi|49474591|ref|YP_032633.1| hypothetical protein BQ10670 [Bartonella quintana str. Toulouse]
>gi|49474592|ref|YP_032634.1| hypothetical protein BQ10680 [Bartonella quintana str. Toulouse]
>gi|49474637|ref|YP_032679.1| hypothetical signal peptide protein [Bartonella quintana str. Toulouse]
>gi|49474640|ref|YP_032682.1| hypothetical protein BQ11380 [Bartonella quintana str. Toulouse]
>gi|49474649|ref|YP_032691.1| hypothetical protein BQ11490 [Bartonella quintana str. Toulouse]
>gi|49474650|ref|YP_032692.1| phage related protein [Bartonella quintana str. Toulouse]
>gi|49474652|ref|YP_032694.1| hypothetical protein BQ11520 [Bartonella quintana str. Toulouse]
>gi|49474672|ref|YP_032714.1| tolA protein [Bartonella quintana str. Toulouse]
>gi|49474696|ref|YP_032738.1| hypothetical protein BQ12090 [Bartonella quintana str. Toulouse]
>gi|49474803|ref|YP_032845.1| pH adaptation potassium efflux system g [Bartonella quintana str. Toulouse]
>gi|18311645|ref|NP_558312.1| paREP1 [Pyrobaculum aerophilum str. IM2]
>gi|18311659|ref|NP_558326.1| sulfate transport system, permease protein, putative [Pyrobaculum aerophilum str. IM2]
>gi|18311667|ref|NP_558334.1| homoserine kinase [Pyrobaculum aerophilum str. IM2]
>gi|18311673|ref|NP_558340.1| hypothetical protein PAE0044 [Pyrobaculum aerophilum str. IM2]
>gi|18311680|ref|NP_558347.1| hypothetical protein PAE0055 [Pyrobaculum aerophilum str. IM2]
>gi|18311683|ref|NP_558350.1| extracellular solute binding protein, conjectural [Pyrobaculum aerophilum str. IM2]
>gi|18311697|ref|NP_558364.1| hypothetical protein PAE0079 [Pyrobaculum aerophilum str. IM2]
>gi|18311700|ref|NP_558367.1| hypothetical protein PAE0082 [Pyrobaculum aerophilum str. IM2]
>gi|18311701|ref|NP_558368.1| hypothetical protein PAE0084 [Pyrobaculum aerophilum str. IM2]
>gi|18311736|ref|NP_558403.1| hypothetical protein PAE0135 [Pyrobaculum aerophilum str. IM2]
>gi|18311741|ref|NP_558408.1| paREP5a [Pyrobaculum aerophilum str. IM2]
>gi|18311744|ref|NP_558411.1| hypothetical protein PAE0146 [Pyrobaculum aerophilum str. IM2]
>gi|18311748|ref|NP_558415.1| hypothetical protein PAE0152 [Pyrobaculum aerophilum str. IM2]
>gi|18311758|ref|NP_558425.1| paREP11 [Pyrobaculum aerophilum str. IM2]
>gi|18311766|ref|NP_558433.1| metabolite transport protein [Pyrobaculum aerophilum str. IM2]
>gi|18311788|ref|NP_558455.1| phosphoribosylglycinamide formyltransferase [Pyrobaculum aerophilum str. IM2]
>gi|18311816|ref|NP_558483.1| prohibitin homolog (hflK family) [Pyrobaculum aerophilum str. IM2]
>gi|18311817|ref|NP_558484.1| hypothetical protein PAE0263 [Pyrobaculum aerophilum str. IM2]
>gi|18311819|ref|NP_558486.1| hypothetical protein PAE0265 [Pyrobaculum aerophilum str. IM2]
>gi|18311836|ref|NP_558503.1| conserved within P. aerophilum [Pyrobaculum aerophilum str. IM2]
>gi|18311838|ref|NP_558505.1| hypothetical protein PAE0290 [Pyrobaculum aerophilum str. IM2]
>gi|18311853|ref|NP_558520.1| conserved within P. aerophilum [Pyrobaculum aerophilum str. IM2]
>gi|18311858|ref|NP_558525.1| hypothetical protein PAE0313 [Pyrobaculum aerophilum str. IM2]
>gi|18311881|ref|NP_558548.1| cobalamin biosynthesis precorrin-3 methylase (cbiF) [Pyrobaculum aerophilum str. IM2]
>gi|18311893|ref|NP_558560.1| iron (III) dicitrate transport system, permease protein, putative [Pyrobaculum aerophilum str. IM2]
>gi|18311915|ref|NP_558582.1| conserved within P. aerophilum [Pyrobaculum aerophilum str. IM2]
>gi|18311916|ref|NP_558583.1| hypothetical protein PAE0399 [Pyrobaculum aerophilum str. IM2]
>gi|18311917|ref|NP_558584.1| hypothetical protein PAE0401 [Pyrobaculum aerophilum str. IM2]
>gi|18311929|ref|NP_558596.1| glycosyl transferase, putative [Pyrobaculum aerophilum str. IM2]
>gi|18311938|ref|NP_558605.1| hypothetical protein PAE0432 [Pyrobaculum aerophilum str. IM2]
>gi|18311941|ref|NP_558608.1| hypothetical protein PAE0435 [Pyrobaculum aerophilum str. IM2]
>gi|18311942|ref|NP_558609.1| hypothetical protein PAE0436 [Pyrobaculum aerophilum str. IM2]
>gi|18311943|ref|NP_558610.1| glycosyltransferase (type 2) [Pyrobaculum aerophilum str. IM2]
>gi|18311944|ref|NP_558611.1| conserved within P. aerophilum [Pyrobaculum aerophilum str. IM2]
>gi|18311971|ref|NP_558638.1| paREP15, putative coiled-coil protein [Pyrobaculum aerophilum str. IM2]
>gi|18311976|ref|NP_558643.1| paREP1 [Pyrobaculum aerophilum str. IM2]
>gi|18311978|ref|NP_558645.1| paREP15, putative coiled-coil protein [Pyrobaculum aerophilum str. IM2]
>gi|18311982|ref|NP_558649.1| hypothetical protein PAE0499 [Pyrobaculum aerophilum str. IM2]
>gi|18311984|ref|NP_558651.1| paREP15, putative coiled-coil protein [Pyrobaculum aerophilum str. IM2]
>gi|18311992|ref|NP_558659.1| paREP15, putative coiled-coil protein [Pyrobaculum aerophilum str. IM2]
>gi|18312008|ref|NP_558675.1| paREP7 [Pyrobaculum aerophilum str. IM2]

>gi|18312051|ref|NP_558718.1| hypothetical protein PAE0620 [Pyrobaculum aerophilum str. IM2]
>gi|18312067|ref|NP_558734.1| hypothetical protein PAE0643 [Pyrobaculum aerophilum str. IM2]
>gi|18312136|ref|NP_558803.1| hypothetical protein PAE0730 [Pyrobaculum aerophilum str. IM2]
>gi|18312155|ref|NP_558822.1| hypothetical protein PAE0752 [Pyrobaculum aerophilum str. IM2]
>gi|18312157|ref|NP_558824.1| H+-transporting ATP synthase subunit C (atpC) [Pyrobaculum aerophilum str. IM2]
>gi|18312180|ref|NP_558847.1| hypothetical protein PAE0791 [Pyrobaculum aerophilum str. IM2]
>gi|18312207|ref|NP_558874.1| paREP1 [Pyrobaculum aerophilum str. IM2]
>gi|18312213|ref|NP_558880.1| hypothetical protein PAE0831 [Pyrobaculum aerophilum str. IM2]
>gi|18312220|ref|NP_558887.1| hypothetical protein PAE0838 [Pyrobaculum aerophilum str. IM2]
>gi|18312222|ref|NP_558889.1| hypothetical protein PAE0840 [Pyrobaculum aerophilum str. IM2]
>gi|18312227|ref|NP_558894.1| paREP2b [Pyrobaculum aerophilum str. IM2]
>gi|18312228|ref|NP_558895.1| paREP2b [Pyrobaculum aerophilum str. IM2]
>gi|18312232|ref|NP_558899.1| ribosomal protein L14 [Pyrobaculum aerophilum str. IM2]
>gi|18312234|ref|NP_558901.1| hypothetical protein PAE0866 [Pyrobaculum aerophilum str. IM2]
>gi|18312258|ref|NP_558925.1| hypothetical protein PAE0894a [Pyrobaculum aerophilum str. IM2]
>gi|18312264|ref|NP_558931.1| paREP7 [Pyrobaculum aerophilum str. IM2]
>gi|18312268|ref|NP_558935.1| hypothetical protein PAE0910 [Pyrobaculum aerophilum str. IM2]
>gi|18312279|ref|NP_558946.1| hypothetical protein PAE0925 [Pyrobaculum aerophilum str. IM2]
>gi|18312294|ref|NP_558961.1| conserved hypothetical protein part 2, authentic frameshift [Pyrobaculum aerophilum str. IM2]
>gi|18312314|ref|NP_558981.1| hypothetical protein PAE0966 [Pyrobaculum aerophilum str. IM2]
>gi|18312340|ref|NP_559007.1| P. aerophilum family 3 protein [Pyrobaculum aerophilum str. IM2]
>gi|18312351|ref|NP_559018.1| hypothetical protein PAE1016 [Pyrobaculum aerophilum str. IM2]
>gi|18312377|ref|NP_559044.1| hypothetical protein PAE1049 [Pyrobaculum aerophilum str. IM2]
>gi|18312402|ref|NP_559069.1| P. aerophilum family 3 protein [Pyrobaculum aerophilum str. IM2]
>gi|18312405|ref|NP_559072.1| integral membrane protein (rhomboid related), putative [Pyrobaculum aerophilum str. IM2]
>gi|18312409|ref|NP_559076.1| hypothetical protein PAE1105 [Pyrobaculum aerophilum str. IM2]
>gi|18312429|ref|NP_559096.1| hypothetical protein PAE1140 [Pyrobaculum aerophilum str. IM2]
>gi|18312448|ref|NP_559115.1| hypothetical protein PAE1166 [Pyrobaculum aerophilum str. IM2]
>gi|18312463|ref|NP_559130.1| hypothetical protein PAE1188 [Pyrobaculum aerophilum str. IM2]
>gi|18312471|ref|NP_559138.1| hypothetical protein PAE1200 [Pyrobaculum aerophilum str. IM2]
>gi|18312480|ref|NP_559147.1| paREP2b [Pyrobaculum aerophilum str. IM2]
>gi|18312489|ref|NP_559156.1| DNA endonuclease, conjectural [Pyrobaculum aerophilum str. IM2]
>gi|18312495|ref|NP_559162.1| hypothetical protein PAE1238a [Pyrobaculum aerophilum str. IM2]
>gi|18312503|ref|NP_559170.1| hypothetical protein PAE1251 [Pyrobaculum aerophilum str. IM2]
>gi|18312549|ref|NP_559216.1| hypothetical protein PAE1316 [Pyrobaculum aerophilum str. IM2]
>gi|18312550|ref|NP_559217.1| hypothetical protein PAE1318 [Pyrobaculum aerophilum str. IM2]
>gi|18312565|ref|NP_559232.1| cytochrome C oxidase assembly factor [Pyrobaculum aerophilum str. IM2]
>gi|18312572|ref|NP_559239.1| hypothetical protein PAE1345 [Pyrobaculum aerophilum str. IM2]
>gi|18312573|ref|NP_559240.1| conserved within P. aerophilum [Pyrobaculum aerophilum str. IM2]
>gi|18312586|ref|NP_559253.1| hypothetical protein PAE1364 [Pyrobaculum aerophilum str. IM2]
>gi|18312589|ref|NP_559256.1| conserved hypothetical protein part 1, authentic frameshift [Pyrobaculum aerophilum str. IM2]
>gi|18312593|ref|NP_559260.1| hypothetical protein PAE1372 [Pyrobaculum aerophilum str. IM2]
>gi|18312606|ref|NP_559273.1| branched-chain amino acid transport permease protein [Pyrobaculum aerophilum str. IM2]
>gi|18312618|ref|NP_559285.1| hypothetical protein PAE1408 [Pyrobaculum aerophilum str. IM2]
>gi|18312619|ref|NP_559286.1| hypothetical protein PAE1409 [Pyrobaculum aerophilum str. IM2]
>gi|18312625|ref|NP_559292.1| hypothetical protein PAE1421 [Pyrobaculum aerophilum str. IM2]
>gi|18312645|ref|NP_559312.1| hypothetical protein PAE1451 [Pyrobaculum aerophilum str. IM2]
>gi|18312655|ref|NP_559322.1| acetyl/acyl transferase related protein [Pyrobaculum aerophilum str. IM2]
>gi|18312659|ref|NP_559326.1| hypothetical protein PAE1479 [Pyrobaculum aerophilum str. IM2]
>gi|18312671|ref|NP_559338.1| hypothetical protein PAE1500 [Pyrobaculum aerophilum str. IM2]
>gi|18312685|ref|NP_559352.1| conserved protein with 2 CBS domains [Pyrobaculum aerophilum str. IM2]
>gi|18312688|ref|NP_559355.1| hypothetical protein PAE1522 [Pyrobaculum aerophilum str. IM2]
>gi|18312690|ref|NP_559357.1| Glu-tRNA(Gln) amidotransferase subunit A (gatA) [Pyrobaculum aerophilum str. IM2]
>gi|18312694|ref|NP_559361.1| conserved within P. aerophilum [Pyrobaculum aerophilum str. IM2]
>gi|18312702|ref|NP_559369.1| hypothetical protein PAE1545 [Pyrobaculum aerophilum str. IM2]
>gi|18312707|ref|NP_559374.1| transport protein, conjectural [Pyrobaculum aerophilum str. IM2]
>gi|18312720|ref|NP_559387.1| NADH-ubiquinone oxidoreductase subunit [Pyrobaculum aerophilum str. IM2]
>gi|18312727|ref|NP_559394.1| NADH-ubiquinone oxidoreductase subunit [Pyrobaculum aerophilum str. IM2]
>gi|18312728|ref|NP_559395.1| NADH-ubiquinone oxidoreductase subunit [Pyrobaculum aerophilum str. IM2]
>gi|18312732|ref|NP_559399.1| paREP2a [Pyrobaculum aerophilum str. IM2]
>gi|18312771|ref|NP_559438.1| hypothetical protein PAE1636 [Pyrobaculum aerophilum str. IM2]
>gi|18312773|ref|NP_559440.1| ribosomal protein L13 [Pyrobaculum aerophilum str. IM2]
>gi|18312774|ref|NP_559441.1| hypothetical protein PAE1641 [Pyrobaculum aerophilum str. IM2]
>gi|18312779|ref|NP_559446.1| hypothetical protein PAE1647 [Pyrobaculum aerophilum str. IM2]
>gi|18312786|ref|NP_559453.1| paREP2b [Pyrobaculum aerophilum str. IM2]
>gi|18312801|ref|NP_559468.1| hypothetical protein PAE1677 [Pyrobaculum aerophilum str. IM2]
>gi|18312807|ref|NP_559474.1| hypothetical protein PAE1687 [Pyrobaculum aerophilum str. IM2]
>gi|18312824|ref|NP_559491.1| ribosomal protein L12 [Pyrobaculum aerophilum str. IM2]
>gi|18312831|ref|NP_559498.1| P. aerophilum family 550 protein [Pyrobaculum aerophilum str. IM2]
>gi|18312852|ref|NP_559519.1| P. aerophilum family 550 protein [Pyrobaculum aerophilum str. IM2]

>gi|18312872|ref|NP_559539.1| ribosomal protein L29 [Pyrobaculum aerophilum str. IM2]
>gi|18312878|ref|NP_559545.1| hypothetical protein PAE1784 [Pyrobaculum aerophilum str. IM2]
>gi|18312881|ref|NP_559548.1| conserved within P. aerophilum [Pyrobaculum aerophilum str. IM2]
>gi|18312890|ref|NP_559557.1| hypothetical protein PAE1803 [Pyrobaculum aerophilum str. IM2]
>gi|18312911|ref|NP_559578.1| paREP13 [Pyrobaculum aerophilum str. IM2]
>gi|18312921|ref|NP_559588.1| hypothetical protein PAE1842 [Pyrobaculum aerophilum str. IM2]
>gi|18312931|ref|NP_559598.1| hypothetical protein PAE1863 [Pyrobaculum aerophilum str. IM2]
>gi|18312943|ref|NP_559610.1| hypothetical protein PAE1883 [Pyrobaculum aerophilum str. IM2]
>gi|18312944|ref|NP_559611.1| phosphate permease, conjectural [Pyrobaculum aerophilum str. IM2]
>gi|18312952|ref|NP_559619.1| hypothetical protein PAE1897 [Pyrobaculum aerophilum str. IM2]
>gi|18312953|ref|NP_559620.1| hypothetical protein PAE1899 [Pyrobaculum aerophilum str. IM2]
>gi|18312955|ref|NP_559622.1| paREP8 [Pyrobaculum aerophilum str. IM2]
>gi|18312962|ref|NP_559629.1| hypothetical protein PAE1910 [Pyrobaculum aerophilum str. IM2]
>gi|18312986|ref|NP_559653.1| P. aerophilum family 453, possible regulatory protein [Pyrobaculum aerophilum str. IM2]
>gi|18312988|ref|NP_559655.1| hypothetical protein PAE1951 [Pyrobaculum aerophilum str. IM2]
>gi|18313010|ref|NP_559677.1| aerolysin, part 1, authentic frameshift [Pyrobaculum aerophilum str. IM2]
>gi|18313043|ref|NP_559710.1| paREP10, authentic frameshift [Pyrobaculum aerophilum str. IM2]
>gi|18313050|ref|NP_559717.1| hypothetical protein PAE2037 [Pyrobaculum aerophilum str. IM2]
>gi|18313054|ref|NP_559721.1| hypothetical protein PAE2041 [Pyrobaculum aerophilum str. IM2]
>gi|18313063|ref|NP_559730.1| hypothetical protein PAE2054 [Pyrobaculum aerophilum str. IM2]
>gi|18313064|ref|NP_559731.1| hypothetical protein PAE2055 [Pyrobaculum aerophilum str. IM2]
>gi|18313071|ref|NP_559738.1| conserved within P. aerophilum, part 1, authentic frameshift [Pyrobaculum aerophilum str. IM2]
>gi|18313099|ref|NP_559766.1| hypothetical protein PAE2104 [Pyrobaculum aerophilum str. IM2]
>gi|18313116|ref|NP_559783.1| paREP2b [Pyrobaculum aerophilum str. IM2]
>gi|18313165|ref|NP_559832.1| hypothetical protein PAE2187 [Pyrobaculum aerophilum str. IM2]
>gi|18313166|ref|NP_559833.1| hypothetical protein PAE2188 [Pyrobaculum aerophilum str. IM2]
>gi|18313248|ref|NP_559915.1| ABC transporter ATP-binding component, degenerate [Pyrobaculum aerophilum str. IM2]
>gi|18313283|ref|NP_559950.1| hypothetical protein PAE2354 [Pyrobaculum aerophilum str. IM2]
>gi|18313286|ref|NP_559953.1| hypothetical protein PAE2358 [Pyrobaculum aerophilum str. IM2]
>gi|18313294|ref|NP_559961.1| hypothetical protein PAE2367 [Pyrobaculum aerophilum str. IM2]
>gi|18313302|ref|NP_559969.1| hypothetical protein PAE2380 [Pyrobaculum aerophilum str. IM2]
>gi|18313319|ref|NP_559986.1| hypothetical protein PAE2402 [Pyrobaculum aerophilum str. IM2]
>gi|18313322|ref|NP_559989.1| hypothetical protein PAE2406 [Pyrobaculum aerophilum str. IM2]
>gi|18313328|ref|NP_559995.1| hypothetical protein PAE2414 [Pyrobaculum aerophilum str. IM2]
>gi|18313330|ref|NP_559997.1| hypothetical protein PAE2417 [Pyrobaculum aerophilum str. IM2]
>gi|18313353|ref|NP_560020.1| paREP13 [Pyrobaculum aerophilum str. IM2]
>gi|18313388|ref|NP_560055.1| hypothetical protein PAE2498 [Pyrobaculum aerophilum str. IM2]
>gi|18313390|ref|NP_560057.1| paREP13 [Pyrobaculum aerophilum str. IM2]
>gi|18313391|ref|NP_560058.1| conserved hypothetical protein, frameshift [Pyrobaculum aerophilum str. IM2]
>gi|18313396|ref|NP_560063.1| paREP7 [Pyrobaculum aerophilum str. IM2]
>gi|18313399|ref|NP_560066.1| conserved within P. aerophilum [Pyrobaculum aerophilum str. IM2]
>gi|18313401|ref|NP_560068.1| hypothetical protein PAE2516 [Pyrobaculum aerophilum str. IM2]
>gi|18313415|ref|NP_560082.1| hypothetical protein PAE2539 [Pyrobaculum aerophilum str. IM2]
>gi|18313421|ref|NP_560088.1| hypothetical protein PAE2548 [Pyrobaculum aerophilum str. IM2]
>gi|18313426|ref|NP_560093.1| hypothetical protein PAE2553 [Pyrobaculum aerophilum str. IM2]
>gi|18313470|ref|NP_560137.1| paREP13 [Pyrobaculum aerophilum str. IM2]
>gi|18313513|ref|NP_560180.1| hypothetical protein PAE2682 [Pyrobaculum aerophilum str. IM2]
>gi|18313542|ref|NP_560209.1| P. aerophilum family 79 protein [Pyrobaculum aerophilum str. IM2]
>gi|18313545|ref|NP_560212.1| hypothetical protein PAE2728 [Pyrobaculum aerophilum str. IM2]
>gi|18313547|ref|NP_560214.1| hypothetical protein PAE2731 [Pyrobaculum aerophilum str. IM2]
>gi|18313554|ref|NP_560221.1| paREP5a [Pyrobaculum aerophilum str. IM2]
>gi|18313560|ref|NP_560227.1| hypothetical protein PAE2747 [Pyrobaculum aerophilum str. IM2]
>gi|18313566|ref|NP_560233.1| hypothetical protein PAE2755 [Pyrobaculum aerophilum str. IM2]
>gi|18313580|ref|NP_560247.1| hypothetical protein PAE2779 [Pyrobaculum aerophilum str. IM2]
>gi|18313602|ref|NP_560269.1| hypothetical protein PAE2808 [Pyrobaculum aerophilum str. IM2]
>gi|18313610|ref|NP_560277.1| paREP1 [Pyrobaculum aerophilum str. IM2]
>gi|18313623|ref|NP_560290.1| molybdopterin oxidoreductase, membrane subunit [Pyrobaculum aerophilum str. IM2]
>gi|18313635|ref|NP_560302.1| paREP6 [Pyrobaculum aerophilum str. IM2]
>gi|18313637|ref|NP_560304.1| conserved within P. aerophilum [Pyrobaculum aerophilum str. IM2]
>gi|18313642|ref|NP_560309.1| molybdopterin oxidoreductase, membrane subunit [Pyrobaculum aerophilum str. IM2]
>gi|18313650|ref|NP_560317.1| paREP1 [Pyrobaculum aerophilum str. IM2]
>gi|18313665|ref|NP_560332.1| hypothetical protein PAE2889 [Pyrobaculum aerophilum str. IM2]
>gi|18313677|ref|NP_560344.1| purine NTPase, probable [Pyrobaculum aerophilum str. IM2]
>gi|18313681|ref|NP_560348.1| hypothetical protein PAE2910 [Pyrobaculum aerophilum str. IM2]
>gi|18313691|ref|NP_560358.1| resistance protein, conjectural [Pyrobaculum aerophilum str. IM2]
>gi|18313712|ref|NP_560379.1| hypothetical protein PAE2949 [Pyrobaculum aerophilum str. IM2]
>gi|18313717|ref|NP_560384.1| hypothetical protein PAE2958 [Pyrobaculum aerophilum str. IM2]
>gi|18313731|ref|NP_560398.1| hypothetical protein PAE2975 [Pyrobaculum aerophilum str. IM2]
>gi|18313737|ref|NP_560404.1| hypothetical protein PAE2982 [Pyrobaculum aerophilum str. IM2]
>gi|18313746|ref|NP_560413.1| hypothetical protein PAE2992 [Pyrobaculum aerophilum str. IM2]

>gi|18313763|ref|NP_560430.1| conserved within P. aerophilum [Pyrobaculum aerophilum str. IM2]
>gi|18313775|ref|NP_560442.1| translation initiation factor aIF-2 alpha subunit [Pyrobaculum aerophilum str. IM2]
>gi|18313780|ref|NP_560447.1| proliferating-cell nuclear antigen homolog (PCNA) [Pyrobaculum aerophilum str. IM2]
>gi|18313793|ref|NP_560460.1| hypothetical protein PAE3058 [Pyrobaculum aerophilum str. IM2]
>gi|18313798|ref|NP_560465.1| ABC-2 type transport system, membrane protein [Pyrobaculum aerophilum str. IM2]
>gi|18313803|ref|NP_560470.1| P. aerophilum family 1964 protein [Pyrobaculum aerophilum str. IM2]
>gi|18313821|ref|NP_560488.1| hypothetical protein PAE3099 [Pyrobaculum aerophilum str. IM2]
>gi|18313827|ref|NP_560494.1| acidic ribosomal protein P0 (L10E) [Pyrobaculum aerophilum str. IM2]
>gi|18313841|ref|NP_560508.1| hypothetical protein PAE3123 [Pyrobaculum aerophilum str. IM2]
>gi|18313851|ref|NP_560518.1| hypothetical protein PAE3137 [Pyrobaculum aerophilum str. IM2]
>gi|18313852|ref|NP_560519.1| hypothetical protein PAE3138 [Pyrobaculum aerophilum str. IM2]
>gi|18313864|ref|NP_560531.1| hypothetical protein PAE3156 [Pyrobaculum aerophilum str. IM2]
>gi|18313881|ref|NP_560548.1| ribosomal protein S11 [Pyrobaculum aerophilum str. IM2]
>gi|18313888|ref|NP_560555.1| hypothetical protein PAE3188 [Pyrobaculum aerophilum str. IM2]
>gi|18313892|ref|NP_560559.1| possible heat shock protein, htpX homolog part 1, authentic frameshift [Pyrobaculum aerophilum str. IM2]
>gi|18313911|ref|NP_560578.1| hypothetical protein PAE3214 [Pyrobaculum aerophilum str. IM2]
>gi|18313931|ref|NP_560598.1| hypothetical protein PAE3243 [Pyrobaculum aerophilum str. IM2]
>gi|18313939|ref|NP_560606.1| hypothetical protein PAE3252 [Pyrobaculum aerophilum str. IM2]
>gi|18313945|ref|NP_560612.1| P. aerophilum family 550 protein [Pyrobaculum aerophilum str. IM2]
>gi|18313976|ref|NP_560643.1| hypothetical protein PAE3303 [Pyrobaculum aerophilum str. IM2]
>gi|18314033|ref|NP_560700.1| P. aerophilum family 453, possible regulatory protein part 1, authentic frameshift [Pyrobaculum aerophilum str. IM2]
>gi|18314034|ref|NP_560701.1| hypothetical protein PAE3391 [Pyrobaculum aerophilum str. IM2]
>gi|18314037|ref|NP_560704.1| hypothetical protein PAE3397 [Pyrobaculum aerophilum str. IM2]
>gi|18314047|ref|NP_560714.1| ribose ABC transporter, permease protein [Pyrobaculum aerophilum str. IM2]
>gi|18314061|ref|NP_560728.1| hypothetical protein PAE3432 [Pyrobaculum aerophilum str. IM2]
>gi|18314074|ref|NP_560741.1| hypothetical protein PAE3450 [Pyrobaculum aerophilum str. IM2]
>gi|18314106|ref|NP_560773.1| UDP-N-acetylglucosamine--dolichyl-phosphate N-acetylglucosaminephosphotransferase [Pyrobaculum aerophilum str. IM2]
>gi|18314118|ref|NP_560785.1| hypothetical protein PAE3504 [Pyrobaculum aerophilum str. IM2]
>gi|18314136|ref|NP_560803.1| hypothetical protein PAE3533 [Pyrobaculum aerophilum str. IM2]
>gi|18314181|ref|NP_560848.1| cytochrome C oxidase assembly factor [Pyrobaculum aerophilum str. IM2]
>gi|18314182|ref|NP_560849.1| nitrite reductase (cytochrome C), conjectural [Pyrobaculum aerophilum str. IM2]
>gi|18314189|ref|NP_560856.1| hypothetical protein PAE3607 [Pyrobaculum aerophilum str. IM2]
>gi|18314196|ref|NP_560863.1| hypothetical protein PAE3614 [Pyrobaculum aerophilum str. IM2]
>gi|18314199|ref|NP_560866.1| hypothetical protein PAE3618 [Pyrobaculum aerophilum str. IM2]
>gi|18314201|ref|NP_560868.1| transport protein, putative [Pyrobaculum aerophilum str. IM2]
>gi|18314213|ref|NP_560880.1| conserved protein (gufA) [Pyrobaculum aerophilum str. IM2]
>gi|18314215|ref|NP_560882.1| hypothetical protein PAE3640 [Pyrobaculum aerophilum str. IM2]
>gi|18314217|ref|NP_560884.1| conserved within P. aerophilum [Pyrobaculum aerophilum str. IM2]
>gi|18314223|ref|NP_560890.1| hypothetical protein PAE3650 [Pyrobaculum aerophilum str. IM2]
>gi|18314230|ref|NP_560897.1| hypothetical protein PAE3662 [Pyrobaculum aerophilum str. IM2]
>gi|18314234|ref|NP_560901.1| hypothetical protein PAE3667 [Pyrobaculum aerophilum str. IM2]
>gi|18314235|ref|NP_560902.1| hypothetical protein PAE3670 [Pyrobaculum aerophilum str. IM2]
>gi|47458839|ref|YP_015701.1| hypothetical protein MMOB0040 [Mycoplasma mobile 163K]
>gi|47458848|ref|YP_015710.1| xylose ABC transporter permease protein [Mycoplasma mobile 163K]
>gi|47458853|ref|YP_015715.1| expressed protein [Mycoplasma mobile 163K]
>gi|47458856|ref|YP_015718.1| hypothetical protein MMOB0210 [Mycoplasma mobile 163K]
>gi|47458865|ref|YP_015727.1| hypothetical protein MMOB0300 [Mycoplasma mobile 163K]
>gi|47458866|ref|YP_015728.1| expressed protein [Mycoplasma mobile 163K]
>gi|47458874|ref|YP_015736.1| unspecified sugar ABC transport permease protein [Mycoplasma mobile 163K]
>gi|47458875|ref|YP_015737.1| expressed protein [Mycoplasma mobile 163K]
>gi|47458891|ref|YP_015753.1| phosphatidate cytidylyltransferase [Mycoplasma mobile 163K]
>gi|47458932|ref|YP_015794.1| hypothetical protein MMOB0970 [Mycoplasma mobile 163K]
>gi|47458945|ref|YP_015807.1| expressed protein [Mycoplasma mobile 163K]
>gi|47458947|ref|YP_015809.1| heat shock protein GrpE [Mycoplasma mobile 163K]
>gi|47458951|ref|YP_015813.1| expressed protein [Mycoplasma mobile 163K]
>gi|47458964|ref|YP_015826.1| expressed protein [Mycoplasma mobile 163K]
>gi|47458970|ref|YP_015832.1| expressed protein [Mycoplasma mobile 163K]
>gi|47458975|ref|YP_015837.1| 50S ribosomal protein l11 [Mycoplasma mobile 163K]
>gi|47458984|ref|YP_015846.1| hypothetical protein MMOB1490 [Mycoplasma mobile 163K]
>gi|47458990|ref|YP_015852.1| expressed protein [Mycoplasma mobile 163K]
>gi|47458996|ref|YP_015858.1| expressed protein [Mycoplasma mobile 163K]
>gi|47459027|ref|YP_015889.1| NH(3)-dependent NAD(+) synthetase [Mycoplasma mobile 163K]
>gi|47459042|ref|YP_015904.1| ATP synthase epsilon chain [Mycoplasma mobile 163K]
>gi|47459047|ref|YP_015909.1| ATP synthase delta chain [Mycoplasma mobile 163K]
>gi|47459049|ref|YP_015911.1| ATP synthase c chain [Mycoplasma mobile 163K]
>gi|47459071|ref|YP_015933.1| 50S ribosomal protein l4 [Mycoplasma mobile 163K]
>gi|47459072|ref|YP_015934.1| 50S ribosomal protein l23 [Mycoplasma mobile 163K]
>gi|47459107|ref|YP_015969.1| secD- and secF-like type II secretion system protein [Mycoplasma mobile 163K]

>gi|47459115|ref|YP_015977.1| DEAD-box ATP-dependent RNA helicase [Mycoplasma mobile 163K]
>gi|47459124|ref|YP_015986.1| DNA-directed RNA polymerase sigma factor [Mycoplasma mobile 163K]
>gi|47459134|ref|YP_015996.1| expressed protein [Mycoplasma mobile 163K]
>gi|47459137|ref|YP_015999.1| hypothetical protein MMOB3020 [Mycoplasma mobile 163K]
>gi|47459145|ref|YP_016007.1| expressed protein [Mycoplasma mobile 163K]
>gi|47459170|ref|YP_016032.1| truncated DNA polymerase III (exonuclease subunit) protein [Mycoplasma mobile 163K]
>gi|47459176|ref|YP_016038.1| hypothetical protein MMOB3410 [Mycoplasma mobile 163K]
>gi|47459185|ref|YP_016047.1| unspecified toxin/drug ABC transporter ATP-binding and permease protein [Mycoplasma mobile 163K]
>gi|47459188|ref|YP_016050.1| lipoprotein signal peptidase [Mycoplasma mobile 163K]
>gi|47459196|ref|YP_016058.1| glucan 1,6-alpha- (dextran) glucosidase [Mycoplasma mobile 163K]
>gi|47459199|ref|YP_016061.1| hypothetical protein MMOB3640 [Mycoplasma mobile 163K]
>gi|47459210|ref|YP_016072.1| conserved hypothetical membrane protein [Mycoplasma mobile 163K]
>gi|47459218|ref|YP_016080.1| hypothetical protein MMOB3830 [Mycoplasma mobile 163K]
>gi|47459226|ref|YP_016088.1| hypothetical protein MMOB3910 [Mycoplasma mobile 163K]
>gi|47459239|ref|YP_016101.1| hypothetical protein MMOB4040 [Mycoplasma mobile 163K]
>gi|47459272|ref|YP_016134.1| expressed protein [Mycoplasma mobile 163K]
>gi|47459277|ref|YP_016139.1| expressed protein [Mycoplasma mobile 163K]
>gi|47459278|ref|YP_016140.1| conserved hypothetical membrane protein [Mycoplasma mobile 163K]
>gi|47459308|ref|YP_016170.1| expressed protein [Mycoplasma mobile 163K]
>gi|47459313|ref|YP_016175.1| 1-acyl-sn-glycerol-3-phosphate acyltransferase [Mycoplasma mobile 163K]
>gi|47459321|ref|YP_016183.1| expressed protein [Mycoplasma mobile 163K]
>gi|47459335|ref|YP_016197.1| putative glucose/sucrose specific PTS system IIB component [Mycoplasma mobile 163K]
>gi|47459340|ref|YP_016202.1| hypothetical protein MMOB5050 [Mycoplasma mobile 163K]
>gi|47459369|ref|YP_016231.1| 50S ribosomal protein l7/l12 [Mycoplasma mobile 163K]
>gi|47459429|ref|YP_016291.1| expressed protein [Mycoplasma mobile 163K]
>gi|47459430|ref|YP_016292.1| expressed protein [Mycoplasma mobile 163K]
>gi|47459431|ref|YP_016293.1| expressed protein [Mycoplasma mobile 163K]
>gi|47459433|ref|YP_016295.1| expressed protein [Mycoplasma mobile 163K]
>gi|47459454|ref|YP_016316.1| large-conductance mechanosensitive channel [Mycoplasma mobile 163K]
>gi|47459457|ref|YP_016319.1| expressed protein [Mycoplasma mobile 163K]
>gi|47459461|ref|YP_016323.1| expressed protein [Mycoplasma mobile 163K]
>gi|18976376|ref|NP_577733.1| hypothetical protein PF0004 [Pyrococcus furiosus DSM 3638]
>gi|18976399|ref|NP_577756.1| putative integral membrane transport protein [Pyrococcus furiosus DSM 3638]
>gi|18976442|ref|NP_577799.1| hypothetical protein PF0070 [Pyrococcus furiosus DSM 3638]
>gi|18976464|ref|NP_577821.1| replication factor C, large subunit [Pyrococcus furiosus DSM 3638]
>gi|33359450|ref|NP_577859.2| hypothetical protein PF0130 [Pyrococcus furiosus DSM 3638]
>gi|33359451|ref|NP_877881.1| hypothetical protein PF0131.1n [Pyrococcus furiosus DSM 3638]
>gi|33359452|ref|NP_877882.1| hypothetical protein PF0131.2n [Pyrococcus furiosus DSM 3638]
>gi|18976523|ref|NP_577880.1| hypothetical protein PF0151 [Pyrococcus furiosus DSM 3638]
>gi|33359456|ref|NP_577905.2| ATPase subunit B [Pyrococcus furiosus DSM 3638]
>gi|33359457|ref|NP_577908.2| ATPase subunit E [Pyrococcus furiosus DSM 3638]
>gi|18976588|ref|NP_577945.1| maf protein [Pyrococcus furiosus DSM 3638]
>gi|18976635|ref|NP_577992.1| hypothetical protein PF0263 [Pyrococcus furiosus DSM 3638]
>gi|18976653|ref|NP_578010.1| hypothetical protein PF0281 [Pyrococcus furiosus DSM 3638]
>gi|18976707|ref|NP_578064.1| flagella-related protein d, putative [Pyrococcus furiosus DSM 3638]
>gi|18976708|ref|NP_578065.1| hypothetical protein PF0336 [Pyrococcus furiosus DSM 3638]
>gi|18976713|ref|NP_578070.1| hypothetical protein PF0341 [Pyrococcus furiosus DSM 3638]
>gi|18976751|ref|NP_578108.1| LSU ribosomal protein L39E [Pyrococcus furiosus DSM 3638]
>gi|18976782|ref|NP_578139.1| hypothetical protein PF0410 [Pyrococcus furiosus DSM 3638]
>gi|18976818|ref|NP_578175.1| NDP-sugar synthase [Pyrococcus furiosus DSM 3638]
>gi|18976823|ref|NP_578180.1| hypothetical protein PF0451 [Pyrococcus furiosus DSM 3638]
>gi|18976861|ref|NP_578218.1| hypothetical protein PF0489 [Pyrococcus furiosus DSM 3638]
>gi|18976865|ref|NP_578222.1| hypothetical protein PF0493 [Pyrococcus furiosus DSM 3638]
>gi|18976870|ref|NP_578227.1| hypothetical protein PF0498 [Pyrococcus furiosus DSM 3638]
>gi|18976882|ref|NP_578239.1| mazg beta-lactamase regulatory protein - like protein [Pyrococcus furiosus DSM 3638]
>gi|18976891|ref|NP_578248.1| hypothetical protein PF0519 [Pyrococcus furiosus DSM 3638]
>gi|18976917|ref|NP_578274.1| hypothetical protein PF0545 [Pyrococcus furiosus DSM 3638]
>gi|18976945|ref|NP_578302.1| hypothetical protein PF0573 [Pyrococcus furiosus DSM 3638]
>gi|18976999|ref|NP_578356.1| hypothetical protein PF0627 [Pyrococcus furiosus DSM 3638]
>gi|18977016|ref|NP_578373.1| hypothetical protein PF0644 [Pyrococcus furiosus DSM 3638]
>gi|18977028|ref|NP_578385.1| hypothetical protein PF0656 [Pyrococcus furiosus DSM 3638]
>gi|18977037|ref|NP_578394.1| hypothetical protein PF0665 [Pyrococcus furiosus DSM 3638]
>gi|33359497|ref|NP_877897.1| hypothetical protein PF0670.1n [Pyrococcus furiosus DSM 3638]
>gi|18977044|ref|NP_578401.1| hypothetical protein PF0672 [Pyrococcus furiosus DSM 3638]
>gi|18977045|ref|NP_578402.1| methylmalonyl-CoAdecarboxylase gamma chain [Pyrococcus furiosus DSM 3638]
>gi|18977047|ref|NP_578404.1| hypothetical protein PF0675 [Pyrococcus furiosus DSM 3638]
>gi|18977069|ref|NP_578426.1| multidrug resistance protein [Pyrococcus furiosus DSM 3638]
>gi|18977083|ref|NP_578440.1| hypothetical protein PF0711 [Pyrococcus furiosus DSM 3638]
>gi|18977084|ref|NP_578441.1| hypothetical protein PF0712 [Pyrococcus furiosus DSM 3638]
>gi|33359503|ref|NP_877903.1| hypothetical protein PF0736.1n [Pyrococcus furiosus DSM 3638]

>gi|33359504|ref|NP_877904.1| hypothetical protein PF0738.1n [Pyrococcus furiosus DSM 3638]
>gi|18977140|ref|NP_578497.1| acetyl / acyl transferase related protein [Pyrococcus furiosus DSM 3638]
>gi|18977146|ref|NP_578503.1| hypothetical protein PF0774 [Pyrococcus furiosus DSM 3638]
>gi|18977168|ref|NP_578525.1| galactoside o-acetyltransferase [Pyrococcus furiosus DSM 3638]
>gi|18977174|ref|NP_578531.1| hypothetical protein PF0802 [Pyrococcus furiosus DSM 3638]
>gi|18977181|ref|NP_578538.1| hypothetical protein PF0809 [Pyrococcus furiosus DSM 3638]
>gi|18977185|ref|NP_578542.1| hypothetical protein PF0813 [Pyrococcus furiosus DSM 3638]
>gi|18977186|ref|NP_578543.1| hypothetical protein PF0814 [Pyrococcus furiosus DSM 3638]
>gi|18977191|ref|NP_578548.1| LSU ribosomal protein L14E [Pyrococcus furiosus DSM 3638]
>gi|18977202|ref|NP_578559.1| hypothetical protein PF0830 [Pyrococcus furiosus DSM 3638]
>gi|18977230|ref|NP_578587.1| hypothetical protein PF0858 [Pyrococcus furiosus DSM 3638]
>gi|18977248|ref|NP_578605.1| LSU ribosomal protein L15E [Pyrococcus furiosus DSM 3638]
>gi|33359523|ref|NP_877914.1| hypothetical protein PF0897.1n [Pyrococcus furiosus DSM 3638]
>gi|33359524|ref|NP_877915.1| hypothetical protein PF0900.1n [Pyrococcus furiosus DSM 3638]
>gi|18977279|ref|NP_578636.1| hypothetical protein PF0907 [Pyrococcus furiosus DSM 3638]
>gi|18977282|ref|NP_578639.1| iron (III) ABC transporter, permease protein [Pyrococcus furiosus DSM 3638]
>gi|18977295|ref|NP_578652.1| hypothetical protein PF0923 [Pyrococcus furiosus DSM 3638]
>gi|18977298|ref|NP_578655.1| hypothetical protein PF0926 [Pyrococcus furiosus DSM 3638]
>gi|18977368|ref|NP_578725.1| hypothetical protein PF0996 [Pyrococcus furiosus DSM 3638]
>gi|18977397|ref|NP_578754.1| hypothetical protein PF1025 [Pyrococcus furiosus DSM 3638]
>gi|18977409|ref|NP_578766.1| hypothetical protein PF1037 [Pyrococcus furiosus DSM 3638]
>gi|18977410|ref|NP_578767.1| hypothetical protein PF1038 [Pyrococcus furiosus DSM 3638]
>gi|18977416|ref|NP_578773.1| hypothetical protein PF1044 [Pyrococcus furiosus DSM 3638]
>gi|18977434|ref|NP_578791.1| hypothetical protein PF1062 [Pyrococcus furiosus DSM 3638]
>gi|18977441|ref|NP_578798.1| SSU ribosomal protein S8E [Pyrococcus furiosus DSM 3638]
>gi|18977459|ref|NP_578816.1| hypothetical protein PF1087 [Pyrococcus furiosus DSM 3638]
>gi|18977505|ref|NP_578862.1| hypothetical protein PF1133 [Pyrococcus furiosus DSM 3638]
>gi|18977522|ref|NP_578879.1| putative multisubunit Na+/H+ antiporter [Pyrococcus furiosus DSM 3638]
>gi|18977539|ref|NP_578896.1| smc-like [Pyrococcus furiosus DSM 3638]
>gi|18977587|ref|NP_578944.1| hypothetical SUA5 protein [Pyrococcus furiosus DSM 3638]
>gi|18977594|ref|NP_578951.1| hypothetical protein PF1222 [Pyrococcus furiosus DSM 3638]
>gi|18977600|ref|NP_578957.1| hypothetical protein PF1228 [Pyrococcus furiosus DSM 3638]
>gi|18977605|ref|NP_578962.1| putative chitinase [Pyrococcus furiosus DSM 3638]
>gi|18977628|ref|NP_578985.1| hypothetical protein PF1256 [Pyrococcus furiosus DSM 3638]
>gi|18977631|ref|NP_578988.1| hypothetical protein PF1259 [Pyrococcus furiosus DSM 3638]
>gi|18977680|ref|NP_579037.1| hypothetical protein PF1308 [Pyrococcus furiosus DSM 3638]
>gi|18977720|ref|NP_579077.1| hypothetical protein PF1348 [Pyrococcus furiosus DSM 3638]
>gi|18977725|ref|NP_579082.1| hypothetical protein PF1353 [Pyrococcus furiosus DSM 3638]
>gi|18977740|ref|NP_579097.1| SSU ribosomal protein S28E [Pyrococcus furiosus DSM 3638]
>gi|18977745|ref|NP_579102.1| cation transporter, putative [Pyrococcus furiosus DSM 3638]
>gi|18977763|ref|NP_579120.1| hypothetical protein PF1391 [Pyrococcus furiosus DSM 3638]
>gi|18977788|ref|NP_579145.1| hypothetical protein PF1416 [Pyrococcus furiosus DSM 3638]
>gi|18977820|ref|NP_579177.1| hypothetical protein PF1448 [Pyrococcus furiosus DSM 3638]
>gi|18977924|ref|NP_579281.1| hypothetical protein PF1552 [Pyrococcus furiosus DSM 3638]
>gi|18977957|ref|NP_579314.1| hypothetical protein PF1585 [Pyrococcus furiosus DSM 3638]
>gi|18977967|ref|NP_579324.1| hypothetical protein PF1595 [Pyrococcus furiosus DSM 3638]
>gi|18977972|ref|NP_579329.1| ferripyochelin binding protein [Pyrococcus furiosus DSM 3638]
>gi|18977980|ref|NP_579337.1| hypothetical protein PF1608 [Pyrococcus furiosus DSM 3638]
>gi|18977984|ref|NP_579341.1| hypothetical protein PF1612 [Pyrococcus furiosus DSM 3638]
>gi|18978009|ref|NP_579366.1| mevalonate kinase [Pyrococcus furiosus DSM 3638]
>gi|18978046|ref|NP_579403.1| hypothetical protein PF1674 [Pyrococcus furiosus DSM 3638]
>gi|18978049|ref|NP_579406.1| hypothetical protein PF1677 [Pyrococcus furiosus DSM 3638]
>gi|18978053|ref|NP_579410.1| hypothetical protein PF1681 [Pyrococcus furiosus DSM 3638]
>gi|18978069|ref|NP_579426.1| putative ribose/galactose ABC transporter [Pyrococcus furiosus DSM 3638]
>gi|18978098|ref|NP_579455.1| hypothetical protein PF1726 [Pyrococcus furiosus DSM 3638]
>gi|18978104|ref|NP_579461.1| transcriptional regulatory protein, asnC family [Pyrococcus furiosus DSM 3638]
>gi|18978133|ref|NP_579490.1| hypothetical protein PF1761 [Pyrococcus furiosus DSM 3638]
>gi|18978168|ref|NP_579525.1| putative polyferredoxin [Pyrococcus furiosus DSM 3638]
>gi|33359574|ref|NP_579531.2| LSU ribosomal protein L15P [Pyrococcus furiosus DSM 3638]
>gi|18978178|ref|NP_579535.1| LSU ribosomal protein L19E [Pyrococcus furiosus DSM 3638]
>gi|33359575|ref|NP_579557.2| hypothetical protein PF1828 [Pyrococcus furiosus DSM 3638]
>gi|18978208|ref|NP_579565.1| hypothetical protein PF1836 [Pyrococcus furiosus DSM 3638]
>gi|18978213|ref|NP_579570.1| hypothetical protein PF1841 [Pyrococcus furiosus DSM 3638]
>gi|18978214|ref|NP_579571.1| hypothetical protein PF1842 [Pyrococcus furiosus DSM 3638]
>gi|18978215|ref|NP_579572.1| chromosome segregation protein smc [Pyrococcus furiosus DSM 3638]
>gi|33359578|ref|NP_579596.2| hypothetical protein PF1867 [Pyrococcus furiosus DSM 3638]
>gi|18978251|ref|NP_579608.1| o-linked glcnac transferase [Pyrococcus furiosus DSM 3638]
>gi|18978304|ref|NP_579661.1| hypothetical protein PF1932 [Pyrococcus furiosus DSM 3638]
>gi|18978306|ref|NP_579663.1| hypothetical protein PF1934 [Pyrococcus furiosus DSM 3638]
>gi|33359584|ref|NP_877933.1| hypothetical protein PF1940.1n [Pyrococcus furiosus DSM 3638]

>gi|18978320|ref|NP_579677.1| hypothetical protein PF1948 [Pyrococcus furiosus DSM 3638]
>gi|18978325|ref|NP_579682.1| inosine-5'-monophosphate dehydrogenase related protein II [Pyrococcus furiosus DSM 3638]
>gi|18978366|ref|NP_579723.1| LSU ribosomal protein L12A [Pyrococcus furiosus DSM 3638]
>gi|33359588|ref|NP_579746.2| hypothetical protein PF2017 [Pyrococcus furiosus DSM 3638]
>gi|33359589|ref|NP_579747.2| hypothetical protein PF2018 [Pyrococcus furiosus DSM 3638]
>gi|18978397|ref|NP_579754.1| hypothetical protein PF2025 [Pyrococcus furiosus DSM 3638]
>gi|18978415|ref|NP_579772.1| hypothetical protein PF2043 [Pyrococcus furiosus DSM 3638]
>gi|18978430|ref|NP_579787.1| hypothetical protein PF2058 [Pyrococcus furiosus DSM 3638]
>gi|42519941|ref|NP_965856.1| ribosomal protein L7/L12 [Wolbachia endosymbiont of Drosophila melanogaster]
>gi|42519951|ref|NP_965866.1| hypothetical protein WD0034 [Wolbachia endosymbiont of Drosophila melanogaster]
>gi|42519966|ref|NP_965881.1| hypothetical protein WD0051 [Wolbachia endosymbiont of Drosophila melanogaster]
>gi|42519982|ref|NP_965897.1| hypothetical protein WD0069 [Wolbachia endosymbiont of Drosophila melanogaster]
>gi|42519985|ref|NP_965900.1| ankyrin repeat domain protein [Wolbachia endosymbiont of Drosophila melanogaster]
>gi|42520053|ref|NP_965968.1| exopolysaccharide synthesis protein ExoD-related protein [Wolbachia endosymbiont of Drosophila melanogaster]
>gi|42520054|ref|NP_965969.1| exopolysaccharide synthesis protein ExoD-related protein [Wolbachia endosymbiont of Drosophila melanogaster]
>gi|42520083|ref|NP_965998.1| hypothetical protein WD0184 [Wolbachia endosymbiont of Drosophila melanogaster]
>gi|42520176|ref|NP_966091.1| hypothetical protein WD0289 [Wolbachia endosymbiont of Drosophila melanogaster]
>gi|42520198|ref|NP_966113.1| hypothetical protein WD0313 [Wolbachia endosymbiont of Drosophila melanogaster]
>gi|42520218|ref|NP_966133.1| hypothetical protein WD0335 [Wolbachia endosymbiont of Drosophila melanogaster]
>gi|42520224|ref|NP_966139.1| hypothetical protein WD0343 [Wolbachia endosymbiont of Drosophila melanogaster]
>gi|42520233|ref|NP_966148.1| hypothetical protein WD0353 [Wolbachia endosymbiont of Drosophila melanogaster]
>gi|42520242|ref|NP_966157.1| cation ABC transporter, permease protein, putative [Wolbachia endosymbiont of Drosophila melanogaster]
>gi|42520262|ref|NP_966177.1| ankyrin repeat domain protein [Wolbachia endosymbiont of Drosophila melanogaster]
>gi|42520279|ref|NP_966194.1| hypothetical protein WD0403 [Wolbachia endosymbiont of Drosophila melanogaster]
>gi|42520333|ref|NP_966248.1| hypothetical protein WD0462 [Wolbachia endosymbiont of Drosophila melanogaster]
>gi|42520334|ref|NP_966249.1| ATPase, AAA family [Wolbachia endosymbiont of Drosophila melanogaster]
>gi|42520336|ref|NP_966251.1| hypothetical protein WD0465 [Wolbachia endosymbiont of Drosophila melanogaster]
>gi|42520342|ref|NP_966257.1| hypothetical protein WD0471 [Wolbachia endosymbiont of Drosophila melanogaster]
>gi|42520376|ref|NP_966291.1| hypothetical protein WD0511 [Wolbachia endosymbiont of Drosophila melanogaster]
>gi|42520389|ref|NP_966304.1| phosphatidate cytidylyltransferase [Wolbachia endosymbiont of Drosophila melanogaster]
>gi|42520400|ref|NP_966315.1| hypothetical protein WD0539 [Wolbachia endosymbiont of Drosophila melanogaster]
>gi|42520432|ref|NP_966347.1| hypothetical protein WD0576 [Wolbachia endosymbiont of Drosophila melanogaster]
>gi|42520460|ref|NP_966375.1| hypothetical protein WD0608 [Wolbachia endosymbiont of Drosophila melanogaster]
>gi|42520544|ref|NP_966459.1| hypothetical protein WD0696 [Wolbachia endosymbiont of Drosophila melanogaster]
>gi|42520545|ref|NP_966460.1| hypothetical protein WD0697 [Wolbachia endosymbiont of Drosophila melanogaster]
>gi|42520553|ref|NP_966468.1| HesB/YadR/YfhF family protein [Wolbachia endosymbiont of Drosophila melanogaster]
>gi|42520565|ref|NP_966480.1| hypothetical protein WD0722 [Wolbachia endosymbiont of Drosophila melanogaster]
>gi|42520576|ref|NP_966491.1| hypothetical protein WD0733 [Wolbachia endosymbiont of Drosophila melanogaster]
>gi|42520607|ref|NP_966522.1| ankyrin repeat domain protein [Wolbachia endosymbiont of Drosophila melanogaster]
>gi|42520612|ref|NP_966527.1| hypothetical protein WD0772 [Wolbachia endosymbiont of Drosophila melanogaster]
>gi|42520649|ref|NP_966564.1| hypothetical protein WD0811 [Wolbachia endosymbiont of Drosophila melanogaster]
>gi|42520650|ref|NP_966565.1| hypothetical protein WD0812 [Wolbachia endosymbiont of Drosophila melanogaster]
>gi|42520668|ref|NP_966583.1| hypothetical protein WD0830 [Wolbachia endosymbiont of Drosophila melanogaster]
>gi|42520674|ref|NP_966589.1| hypothetical protein WD0837 [Wolbachia endosymbiont of Drosophila melanogaster]
>gi|42520688|ref|NP_966603.1| hypothetical protein WD0853 [Wolbachia endosymbiont of Drosophila melanogaster]
>gi|42520729|ref|NP_966644.1| hypothetical protein WD0898 [Wolbachia endosymbiont of Drosophila melanogaster]
>gi|42520734|ref|NP_966649.1| transposase, degenerate [Wolbachia endosymbiont of Drosophila melanogaster]
>gi|42520778|ref|NP_966693.1| NADH dehydrogenase I, J subunit [Wolbachia endosymbiont of Drosophila melanogaster]
>gi|42520779|ref|NP_966694.1| NADH dehydrogenase I, K subunit [Wolbachia endosymbiont of Drosophila melanogaster]
>gi|42520848|ref|NP_966763.1| hypothetical protein WD1039 [Wolbachia endosymbiont of Drosophila melanogaster]
>gi|42520939|ref|NP_966854.1| hypothetical protein WD1137 [Wolbachia endosymbiont of Drosophila melanogaster]
>gi|42520962|ref|NP_966877.1| hypothetical protein WD1161 [Wolbachia endosymbiont of Drosophila melanogaster]
>gi|42520969|ref|NP_966884.1| hypothetical protein WD1171 [Wolbachia endosymbiont of Drosophila melanogaster]
>gi|42520981|ref|NP_966896.1| hypothetical protein WD1184 [Wolbachia endosymbiont of Drosophila melanogaster]
>gi|42521008|ref|NP_966923.1| ribosomal protein S21, putative [Wolbachia endosymbiont of Drosophila melanogaster]
>gi|42521018|ref|NP_966933.1| succinate dehydrogenase, cytochrome b556 subunit [Wolbachia endosymbiont of Drosophila melanogaster]
>gi|42521095|ref|NP_967010.1| hypothetical protein WD1302 [Wolbachia endosymbiont of Drosophila melanogaster]
>gi|42521109|ref|NP_967024.1| 4-hydroxybenzoate octaprenyltransferase [Wolbachia endosymbiont of Drosophila melanogaster]
>gi|49482266|ref|YP_039490.1| putative membrane protein [Staphylococcus aureus subsp. aureus MRSA252]
>gi|49482340|ref|YP_039564.1| putative membrane protein [Staphylococcus aureus subsp. aureus MRSA252]
>gi|49482354|ref|YP_039578.1| immunoglobulin G binding protein A precursor [Staphylococcus aureus subsp. aureus MRSA252]
>gi|49482357|ref|YP_039581.1| putative siderophore transport system permease [Staphylococcus aureus subsp. aureus MRSA252]
>gi|49482368|ref|YP_039592.1| putative membrane protein [Staphylococcus aureus subsp. aureus MRSA252]
>gi|49482402|ref|YP_039626.1| capsular polysaccharide synthesis enzyme [Staphylococcus aureus subsp. aureus MRSA252]
>gi|49482515|ref|YP_039739.1| putative membrane protein [Staphylococcus aureus subsp. aureus MRSA252]
>gi|49482519|ref|YP_039743.1| hypothetical protein SAR0279 [Staphylococcus aureus subsp. aureus MRSA252]
>gi|49482526|ref|YP_039750.1| hypothetical protein SAR0286 [Staphylococcus aureus subsp. aureus MRSA252]
>gi|49482536|ref|YP_039760.1| putative membrane protein [Staphylococcus aureus subsp. aureus MRSA252]
>gi|49482580|ref|YP_039804.1| putative membrane protein [Staphylococcus aureus subsp. aureus MRSA252]
>gi|49482623|ref|YP_039847.1| putative membrane protein [Staphylococcus aureus subsp. aureus MRSA252]

>gi|49482626|ref|YP_039850.1| putative membrane protein [Staphylococcus aureus subsp. aureus MRSA252]
>gi|49482636|ref|YP_039860.1| putative DNA-binding protein [Staphylococcus aureus subsp. aureus MRSA252]
>gi|49482652|ref|YP_039876.1| exotoxin [Staphylococcus aureus subsp. aureus MRSA252]
>gi|49482653|ref|YP_039877.1| exotoxin [Staphylococcus aureus subsp. aureus MRSA252]
>gi|49482689|ref|YP_039913.1| hypothetical protein SAR0464 [Staphylococcus aureus subsp. aureus MRSA252]
>gi|49482758|ref|YP_039982.1| serine acetyltransferase [Staphylococcus aureus subsp. aureus MRSA252]
>gi|49482770|ref|YP_039994.1| 50S ribosomal protein L7/L12 [Staphylococcus aureus subsp. aureus MRSA252]
>gi|49482791|ref|YP_040015.1| putative surface anchored protein [Staphylococcus aureus subsp. aureus MRSA252]
>gi|49482792|ref|YP_040016.1| bone sialoprotein-binding protein [Staphylococcus aureus subsp. aureus MRSA252]
>gi|49482823|ref|YP_040047.1| hypothetical protein SAR0599 [Staphylococcus aureus subsp. aureus MRSA252]
>gi|49482840|ref|YP_040064.1| FecCD transport family protein [Staphylococcus aureus subsp. aureus MRSA252]
>gi|49482876|ref|YP_040100.1| ferrichrome transport permease [Staphylococcus aureus subsp. aureus MRSA252]
>gi|49482918|ref|YP_040142.1| putative membrane protein [Staphylococcus aureus subsp. aureus MRSA252]
>gi|49482933|ref|YP_040157.1| putative lipoprotein [Staphylococcus aureus subsp. aureus MRSA252]
>gi|49482965|ref|YP_040189.1| putative membrane protein [Staphylococcus aureus subsp. aureus MRSA252]
>gi|49482972|ref|YP_040196.1| putative membrane protein [Staphylococcus aureus subsp. aureus MRSA252]
>gi|49482991|ref|YP_040215.1| FecCD transport family protein [Staphylococcus aureus subsp. aureus MRSA252]
>gi|49483004|ref|YP_040228.1| putative glycosyl transferase [Staphylococcus aureus subsp. aureus MRSA252]
>gi|49483012|ref|YP_040236.1| hypothetical protein SAR0809 [Staphylococcus aureus subsp. aureus MRSA252]
>gi|49483028|ref|YP_040252.1| hypothetical protein SAR0826 [Staphylococcus aureus subsp. aureus MRSA252]
>gi|49483041|ref|YP_040265.1| putative lipoprotein [Staphylococcus aureus subsp. aureus MRSA252]
>gi|49483044|ref|YP_040268.1| clumping factor [Staphylococcus aureus subsp. aureus MRSA252]
>gi|49483057|ref|YP_040281.1| putative LysE type translocator protein [Staphylococcus aureus subsp. aureus MRSA252]
>gi|49483070|ref|YP_040294.1| ABC transporter permease protein [Staphylococcus aureus subsp. aureus MRSA252]
>gi|49483107|ref|YP_040331.1| Na+/H+ antiporter subunit [Staphylococcus aureus subsp. aureus MRSA252]
>gi|49483201|ref|YP_040425.1| hypothetical protein SAR1007 [Staphylococcus aureus subsp. aureus MRSA252]
>gi|49483214|ref|YP_040438.1| glutamyl endopeptidase precursor [Staphylococcus aureus subsp. aureus MRSA252]
>gi|49483266|ref|YP_040490.1| putative membrane protein [Staphylococcus aureus subsp. aureus MRSA252]
>gi|49483268|ref|YP_040492.1| putative manganese transport protein [Staphylococcus aureus subsp. aureus MRSA252]
>gi|49483291|ref|YP_040515.1| iron-regulated heme-iron binding protein [Staphylococcus aureus subsp. aureus MRSA252]
>gi|49483292|ref|YP_040516.1| iron-regulated heme-iron binding protein [Staphylococcus aureus subsp. aureus MRSA252]
>gi|49483318|ref|YP_040542.1| putative membrane protein [Staphylococcus aureus subsp. aureus MRSA252]
>gi|49483323|ref|YP_040547.1| putative membrane protein [Staphylococcus aureus subsp. aureus MRSA252]
>gi|49483515|ref|YP_040739.1| putative membrane protein [Staphylococcus aureus subsp. aureus MRSA252]
>gi|49483535|ref|YP_040759.1| hypothetical protein SAR1353 [Staphylococcus aureus subsp. aureus MRSA252]
>gi|49483587|ref|YP_040811.1| putative tetrahydrodipicolinate acetyltransferase [Staphylococcus aureus subsp. aureus MRSA252]
>gi|49483626|ref|YP_040850.1| transporter protein [Staphylococcus aureus subsp. aureus MRSA252]
>gi|49483667|ref|YP_040891.1| cell surface elastin binding protein [Staphylococcus aureus subsp. aureus MRSA252]
>gi|49483736|ref|YP_040960.1| putative lipoprotein [Staphylococcus aureus subsp. aureus MRSA252]
>gi|49483778|ref|YP_041002.1| putative elongation factor P [Staphylococcus aureus subsp. aureus MRSA252]
>gi|49483781|ref|YP_041005.1| hypothetical protein SAR1609 [Staphylococcus aureus subsp. aureus MRSA252]
>gi|49483817|ref|YP_041041.1| putative diacylglycerol kinase [Staphylococcus aureus subsp. aureus MRSA252]
>gi|49483823|ref|YP_041047.1| 30S ribosomal protein S21 [Staphylococcus aureus subsp. aureus MRSA252]
>gi|49483833|ref|YP_041057.1| putative 30S ribosomal protein S20 [Staphylococcus aureus subsp. aureus MRSA252]
>gi|49483849|ref|YP_041073.1| putative membrane protein [Staphylococcus aureus subsp. aureus MRSA252]
>gi|49483860|ref|YP_041084.1| hypothetical protein SAR1694 [Staphylococcus aureus subsp. aureus MRSA252]
>gi|49483869|ref|YP_041093.1| hypothetical protein SAR1704 [Staphylococcus aureus subsp. aureus MRSA252]
>gi|49483870|ref|YP_041094.1| hypothetical protein SAR1705 [Staphylococcus aureus subsp. aureus MRSA252]
>gi|49483896|ref|YP_041120.1| putative membrane protein [Staphylococcus aureus subsp. aureus MRSA252]
>gi|49483978|ref|YP_041202.1| putative membrane protein [Staphylococcus aureus subsp. aureus MRSA252]
>gi|49483982|ref|YP_041206.1| hypothetical protein SAR1820 [Staphylococcus aureus subsp. aureus MRSA252]
>gi|49484027|ref|YP_041251.1| putative membrane protein [Staphylococcus aureus subsp. aureus MRSA252]
>gi|49484041|ref|YP_041265.1| putative membrane protein [Staphylococcus aureus subsp. aureus MRSA252]
>gi|49484083|ref|YP_041307.1| putative peptidyl-prolyl cis-isomerase [Staphylococcus aureus subsp. aureus MRSA252]
>gi|49484087|ref|YP_041311.1| hypothetical protein SAR1936 [Staphylococcus aureus subsp. aureus MRSA252]
>gi|49484168|ref|YP_041392.1| putative membrane protein [Staphylococcus aureus subsp. aureus MRSA252]
>gi|49484171|ref|YP_041395.1| putative membrane protein [Staphylococcus aureus subsp. aureus MRSA252]
>gi|49484183|ref|YP_041407.1| putative membrane protein [Staphylococcus aureus subsp. aureus MRSA252]
>gi|49484184|ref|YP_041408.1| hypothetical protein SAR2035 [Staphylococcus aureus subsp. aureus MRSA252]
>gi|49484223|ref|YP_041447.1| hypothetical protein SAR2077 [Staphylococcus aureus subsp. aureus MRSA252]
>gi|49484246|ref|YP_041470.1| putative lipoprotein [Staphylococcus aureus subsp. aureus MRSA252]
>gi|49484258|ref|YP_041482.1| membrane anchored protein [Staphylococcus aureus subsp. aureus MRSA252]
>gi|49484264|ref|YP_041488.1| autoinducer sensor protein [Staphylococcus aureus subsp. aureus MRSA252]
>gi|49484287|ref|YP_041511.1| hypothetical protein SAR2149 [Staphylococcus aureus subsp. aureus MRSA252]
>gi|49484352|ref|YP_041576.1| DNA-directed RNA polymerase delta subunit [Staphylococcus aureus subsp. aureus MRSA252]
>gi|49484395|ref|YP_041619.1| FecCD transport family protein [Staphylococcus aureus subsp. aureus MRSA252]
>gi|49484478|ref|YP_041702.1| putative membrane protein [Staphylococcus aureus subsp. aureus MRSA252]
>gi|49484497|ref|YP_041721.1| BioY family protein [Staphylococcus aureus subsp. aureus MRSA252]
>gi|49484514|ref|YP_041738.1| hypothetical protein SAR2383 [Staphylococcus aureus subsp. aureus MRSA252]
>gi|49484518|ref|YP_041742.1| putative membrane protein [Staphylococcus aureus subsp. aureus MRSA252]

>gi|49484519|ref|YP_041743.1| hypothetical protein SAR2388 [Staphylococcus aureus subsp. aureus MRSA252]
>gi|49484533|ref|YP_041757.1| putative membrane protein [Staphylococcus aureus subsp. aureus MRSA252]
>gi|49484542|ref|YP_041766.1| putative membrane protein [Staphylococcus aureus subsp. aureus MRSA252]
>gi|49484568|ref|YP_041792.1| hypothetical protein SAR2438 [Staphylococcus aureus subsp. aureus MRSA252]
>gi|49484586|ref|YP_041810.1| putative lipoprotein [Staphylococcus aureus subsp. aureus MRSA252]
>gi|49484599|ref|YP_041823.1| hypothetical protein SAR2470 [Staphylococcus aureus subsp. aureus MRSA252]
>gi|49484634|ref|YP_041858.1| IgG-binding protein [Staphylococcus aureus subsp. aureus MRSA252]
>gi|49484638|ref|YP_041862.1| putative membrane protein [Staphylococcus aureus subsp. aureus MRSA252]
>gi|49484650|ref|YP_041874.1| putative membrane protein [Staphylococcus aureus subsp. aureus MRSA252]
>gi|49484702|ref|YP_041926.1| putative membrane protein [Staphylococcus aureus subsp. aureus MRSA252]
>gi|49484704|ref|YP_041928.1| fibronectin-binding protein precursor [Staphylococcus aureus subsp. aureus MRSA252]
>gi|49484709|ref|YP_041933.1| MerR family regulatory protein [Staphylococcus aureus subsp. aureus MRSA252]
>gi|49484719|ref|YP_041943.1| putative membrane protein [Staphylococcus aureus subsp. aureus MRSA252]
>gi|49484729|ref|YP_041953.1| sortase A [Staphylococcus aureus subsp. aureus MRSA252]
>gi|49484731|ref|YP_041955.1| putative L-serine dehydratase, alpha chain [Staphylococcus aureus subsp. aureus MRSA252]
>gi|49484766|ref|YP_041990.1| secretory antigen precursor [Staphylococcus aureus subsp. aureus MRSA252]
>gi|49484768|ref|YP_041992.1| immunodominant antigen A [Staphylococcus aureus subsp. aureus MRSA252]
>gi|49484827|ref|YP_042051.1| fibrinogen and keratin-10 binding surface anchored protein [Staphylococcus aureus subsp. aureus MRSA252]
>gi|49484836|ref|YP_042060.1| hypothetical protein SAR2718 [Staphylococcus aureus subsp. aureus MRSA252]
>gi|49484841|ref|YP_042065.1| hypothetical protein SAR2723 [Staphylococcus aureus subsp. aureus MRSA252]
>gi|49484850|ref|YP_042074.1| putative serine rich repeat containing protein [Staphylococcus aureus subsp. aureus MRSA252]
>gi|49484893|ref|YP_042117.1| putative membrane protein [Staphylococcus aureus subsp. aureus MRSA252]
>gi|49484903|ref|YP_042127.1| putative membrane protein [Staphylococcus aureus subsp. aureus MRSA252]
>gi|49484909|ref|YP_042133.1| 50S ribosomal protein L34 [Staphylococcus aureus subsp. aureus MRSA252]
>gi|16802071|ref|NP_463556.1| hypothetical protein lmo0023 [Listeria monocytogenes EGD-e]
>gi|16802093|ref|NP_463578.1| hypothetical protein lmo0045 [Listeria monocytogenes EGD-e]
>gi|16802095|ref|NP_463580.1| hypothetical protein lmo0047 [Listeria monocytogenes EGD-e]
>gi|16802101|ref|NP_463586.1| 50S ribosomal protein L9 [Listeria monocytogenes EGD-e]
>gi|16802106|ref|NP_463591.1| hypothetical protein lmo0058 [Listeria monocytogenes EGD-e]
>gi|16802110|ref|NP_463595.1| hypothetical protein lmo0062 [Listeria monocytogenes EGD-e]
>gi|16802117|ref|NP_463602.1| hypothetical protein lmo0069 [Listeria monocytogenes EGD-e]
>gi|16802122|ref|NP_463607.1| hypothetical protein lmo0074 [Listeria monocytogenes EGD-e]
>gi|16802145|ref|NP_463630.1| hypothetical protein lmo0097 [Listeria monocytogenes EGD-e]
>gi|16802169|ref|NP_463654.1| hypothetical protein lmo0121 [Listeria monocytogenes EGD-e]
>gi|16802207|ref|NP_463692.1| putative peptidoglycan bound protein (LPXTG motif) [Listeria monocytogenes EGD-e]
>gi|16802221|ref|NP_463706.1| putative peptidoglycan bound protein (LPXTG motif) [Listeria monocytogenes EGD-e]
>gi|16802232|ref|NP_463717.1| hypothetical protein lmo0186 [Listeria monocytogenes EGD-e]
>gi|16802284|ref|NP_463769.1| hypothetical protein lmo0238 [Listeria monocytogenes EGD-e]
>gi|16802297|ref|NP_463782.1| ribosomal protein L12 [Listeria monocytogenes EGD-e]
>gi|16802310|ref|NP_463795.1| internalin E [Listeria monocytogenes EGD-e]
>gi|16802365|ref|NP_463850.1| hypothetical protein lmo0320 [Listeria monocytogenes EGD-e]
>gi|16802377|ref|NP_463862.1| hypothetical protein lmo0332 [Listeria monocytogenes EGD-e]
>gi|16802433|ref|NP_463918.1| hypothetical protein lmo0388 [Listeria monocytogenes EGD-e]
>gi|16802434|ref|NP_463919.1| low temperature requirement protein A [Listeria monocytogenes EGD-e]
>gi|16802449|ref|NP_463934.1| hypothetical protein lmo0404 [Listeria monocytogenes EGD-e]
>gi|16802456|ref|NP_463941.1| hypothetical protein lmo0412 [Listeria monocytogenes EGD-e]
>gi|16802461|ref|NP_463946.1| hypothetical protein lmo0417 [Listeria monocytogenes EGD-e]
>gi|16802477|ref|NP_463962.1| Internalin A [Listeria monocytogenes EGD-e]
>gi|16802486|ref|NP_463971.1| hypothetical protein lmo0442 [Listeria monocytogenes EGD-e]
>gi|16802504|ref|NP_463989.1| putative membrane associated lipoprotein [Listeria monocytogenes EGD-e]
>gi|16802594|ref|NP_464079.1| hypothetical protein lmo0551 [Listeria monocytogenes EGD-e]
>gi|16802625|ref|NP_464110.1| P60 extracellular protein, invasion associated protein Iap [Listeria monocytogenes EGD-e]
>gi|16802628|ref|NP_464113.1| putative secreted protein [Listeria monocytogenes EGD-e]
>gi|16802639|ref|NP_464124.1| hypothetical protein lmo0596 [Listeria monocytogenes EGD-e]
>gi|16802652|ref|NP_464137.1| hypothetical protein lmo0610 [Listeria monocytogenes EGD-e]
>gi|16802719|ref|NP_464204.1| hypothetical protein lmo0677 [Listeria monocytogenes EGD-e]
>gi|16802757|ref|NP_464242.1| hypothetical protein lmo0715 [Listeria monocytogenes EGD-e]
>gi|16802773|ref|NP_464258.1| hypothetical protein lmo0731 [Listeria monocytogenes EGD-e]
>gi|16802790|ref|NP_464275.1| hypothetical protein lmo0748 [Listeria monocytogenes EGD-e]
>gi|16802813|ref|NP_464298.1| hypothetical protein lmo0771 [Listeria monocytogenes EGD-e]
>gi|16802847|ref|NP_464332.1| hypothetical protein lmo0805 [Listeria monocytogenes EGD-e]
>gi|16802928|ref|NP_464413.1| hypothetical protein lmo0887 [Listeria monocytogenes EGD-e]
>gi|16802972|ref|NP_464457.1| hypothetical protein lmo0932 [Listeria monocytogenes EGD-e]
>gi|16802977|ref|NP_464462.1| hypothetical protein lmo0937 [Listeria monocytogenes EGD-e]
>gi|16802989|ref|NP_464474.1| hypothetical protein lmo0949 [Listeria monocytogenes EGD-e]
>gi|16802994|ref|NP_464479.1| hypothetical protein lmo0954 [Listeria monocytogenes EGD-e]
>gi|16803012|ref|NP_464497.1| D-alanyl carrier protein [Listeria monocytogenes EGD-e]
>gi|16803051|ref|NP_464536.1| hypothetical protein lmo1011 [Listeria monocytogenes EGD-e]
>gi|16803108|ref|NP_464593.1| hypothetical protein lmo1068 [Listeria monocytogenes EGD-e]
>gi|16803149|ref|NP_464634.1| hypothetical protein lmo1109 [Listeria monocytogenes EGD-e]

>gi|16803176|ref|NP_464661.1| hypothetical protein lmo1136 [Listeria monocytogenes EGD-e]
>gi|16803199|ref|NP_464684.1| hypothetical protein lmo1159 [Listeria monocytogenes EGD-e]
>gi|16803245|ref|NP_464730.1| hypothetical protein lmo1205 [Listeria monocytogenes EGD-e]
>gi|16803285|ref|NP_464770.1| hypothetical protein lmo1245 [Listeria monocytogenes EGD-e]
>gi|16803307|ref|NP_464792.1| trigger factor (prolyl isomerase) [Listeria monocytogenes EGD-e]
>gi|16803346|ref|NP_464831.1| hypothetical protein lmo1306 [Listeria monocytogenes EGD-e]
>gi|16803373|ref|NP_464858.1| hypothetical protein lmo1333 [Listeria monocytogenes EGD-e]
>gi|16803392|ref|NP_464877.1| hypothetical protein lmo1352 [Listeria monocytogenes EGD-e]
>gi|16803396|ref|NP_464881.1| hypothetical protein lmo1356 [Listeria monocytogenes EGD-e]
>gi|16803435|ref|NP_464920.1| hypothetical protein lmo1395 [Listeria monocytogenes EGD-e]
>gi|16803501|ref|NP_464986.1| hypothetical protein lmo1461 [Listeria monocytogenes EGD-e]
>gi|16803509|ref|NP_464994.1| 30S ribosomal protein S21 [Listeria monocytogenes EGD-e]
>gi|16803512|ref|NP_464997.1| heat shock protein DnaJ [Listeria monocytogenes EGD-e]
>gi|16803514|ref|NP_464999.1| heat shock protein GrpE [Listeria monocytogenes EGD-e]
>gi|16803535|ref|NP_465020.1| hypothetical protein lmo1495 [Listeria monocytogenes EGD-e]
>gi|16803582|ref|NP_465067.1| ribosomal protein L21 [Listeria monocytogenes EGD-e]
>gi|16803590|ref|NP_465075.1| hypothetical protein lmo1550 [Listeria monocytogenes EGD-e]
>gi|16803637|ref|NP_465122.1| hypothetical protein lmo1597 [Listeria monocytogenes EGD-e]
>gi|16803641|ref|NP_465126.1| hypothetical protein lmo1601 [Listeria monocytogenes EGD-e]
>gi|16803689|ref|NP_465174.1| hypothetical protein lmo1649 [Listeria monocytogenes EGD-e]
>gi|16803705|ref|NP_465190.1| hypothetical protein lmo1665 [Listeria monocytogenes EGD-e]
>gi|16803763|ref|NP_465248.1| hypothetical protein lmo1723 [Listeria monocytogenes EGD-e]
>gi|16803839|ref|NP_465324.1| putative peptidoglycan bound protein (LPXTG motif) [Listeria monocytogenes EGD-e]
>gi|16803842|ref|NP_465327.1| hypothetical protein lmo1802 [Listeria monocytogenes EGD-e]
>gi|16803852|ref|NP_465337.1| hypothetical protein lmo1812 [Listeria monocytogenes EGD-e]
>gi|16803980|ref|NP_465465.1| hypothetical protein lmo1941 [Listeria monocytogenes EGD-e]
>gi|16803997|ref|NP_465482.1| hypothetical protein lmo1958 [Listeria monocytogenes EGD-e]
>gi|16804040|ref|NP_465525.1| hypothetical protein lmo2001 [Listeria monocytogenes EGD-e]
>gi|16804055|ref|NP_465540.1| hypothetical protein lmo2016 [Listeria monocytogenes EGD-e]
>gi|16804066|ref|NP_465551.1| putative cell surface protein, similar to internalin proteins [Listeria monocytogenes EGD-e]
>gi|16804084|ref|NP_465569.1| hypothetical protein lmo2045 [Listeria monocytogenes EGD-e]
>gi|16804174|ref|NP_465659.1| hypothetical protein lmo2135 [Listeria monocytogenes EGD-e]
>gi|16804184|ref|NP_465669.1| hypothetical protein lmo2145 [Listeria monocytogenes EGD-e]
>gi|16804189|ref|NP_465674.1| hypothetical protein lmo2150 [Listeria monocytogenes EGD-e]
>gi|16804197|ref|NP_465682.1| hypothetical protein lmo2158 [Listeria monocytogenes EGD-e]
>gi|16804267|ref|NP_465752.1| hypothetical protein lmo2228 [Listeria monocytogenes EGD-e]
>gi|16804312|ref|NP_465797.1| protein gp30 [Bacteriophage A118] [Listeria monocytogenes EGD-e]
>gi|16804318|ref|NP_465803.1| holin [Bacteriophage A118] [Listeria monocytogenes EGD-e]
>gi|16804326|ref|NP_465811.1| putative tape-measure [Bacteriophage A118] [Listeria monocytogenes EGD-e]
>gi|16804351|ref|NP_465836.1| hypothetical protein lmo2312 [Listeria monocytogenes EGD-e]
>gi|16804369|ref|NP_465854.1| hypothetical protein lmo2330 [Listeria monocytogenes EGD-e]
>gi|16804373|ref|NP_465858.1| hypothetical protein lmo2335 [Listeria monocytogenes EGD-e]
>gi|16804389|ref|NP_465874.1| hypothetical protein lmo2351 [Listeria monocytogenes EGD-e]
>gi|16804393|ref|NP_465878.1| hypothetical protein lmo2355 [Listeria monocytogenes EGD-e]
>gi|16804395|ref|NP_465880.1| hypothetical protein lmo2357 [Listeria monocytogenes EGD-e]
>gi|16804420|ref|NP_465905.1| hypothetical protein lmo2382 [Listeria monocytogenes EGD-e]
>gi|16804422|ref|NP_465907.1| hypothetical protein lmo2384 [Listeria monocytogenes EGD-e]
>gi|16804447|ref|NP_465932.1| hypothetical protein lmo2409 [Listeria monocytogenes EGD-e]
>gi|16804473|ref|NP_465958.1| hypothetical protein lmo2435 [Listeria monocytogenes EGD-e]
>gi|16804508|ref|NP_465993.1| hypothetical protein lmo2470 [Listeria monocytogenes EGD-e]
>gi|16804522|ref|NP_466007.1| hypothetical protein lmo2484 [Listeria monocytogenes EGD-e]
>gi|16804523|ref|NP_466008.1| hypothetical protein lmo2485 [Listeria monocytogenes EGD-e]
>gi|16804542|ref|NP_466027.1| hypothetical protein lmo2504 [Listeria monocytogenes EGD-e]
>gi|16804543|ref|NP_466028.1| peptidoglycan lytic protein P45 [Listeria monocytogenes EGD-e]
>gi|16804598|ref|NP_466083.1| hypothetical protein lmo2560 [Listeria monocytogenes EGD-e]
>gi|16804633|ref|NP_466118.1| hypothetical protein lmo2595 [Listeria monocytogenes EGD-e]
>gi|16804640|ref|NP_466125.1| hypothetical protein lmo2602 [Listeria monocytogenes EGD-e]
>gi|16804725|ref|NP_466210.1| hypothetical protein lmo2688 [Listeria monocytogenes EGD-e]
>gi|16804728|ref|NP_466213.1| hypothetical protein lmo2691 [Listeria monocytogenes EGD-e]
>gi|16804790|ref|NP_466275.1| hypothetical protein lmo2753 [Listeria monocytogenes EGD-e]
>gi|71064620|ref|YP_263347.1| probable heat shock protein DnaJ [Psychrobacter arcticum 273-4]
>gi|71064636|ref|YP_263363.1| hypothetical protein Psyc_0055 [Psychrobacter arcticum 273-4]
>gi|71064647|ref|YP_263374.1| preprotein translocase subunit SecG [Psychrobacter arcticum 273-4]
>gi|71064709|ref|YP_263436.1| hypothetical protein Psyc_0128 [Psychrobacter arcticum 273-4]
>gi|71064732|ref|YP_263459.1| hypothetical protein Psyc_0151 [Psychrobacter arcticum 273-4]
>gi|71064791|ref|YP_263518.1| ribosomal protein S21 [Psychrobacter arcticum 273-4]
>gi|71064792|ref|YP_263519.1| hypothetical protein Psyc_0212 [Psychrobacter arcticum 273-4]
>gi|71064793|ref|YP_263520.1| hypothetical protein Psyc_0213 [Psychrobacter arcticum 273-4]
>gi|71064811|ref|YP_263538.1| possible type IV pilin protein [Psychrobacter arcticum 273-4]
>gi|71064818|ref|YP_263545.1| probable pilus assembly protein major pilin PilA [Psychrobacter arcticum 273-4]

>gi|71064928|ref|YP_263655.1| possible lytic transglycosylase [Psychrobacter arcticum 273-4]
>gi|71064936|ref|YP_263663.1| conserved hypothetcial protein [Psychrobacter arcticum 273-4]
>gi|71064953|ref|YP_263680.1| hypothetical protein Psyc_0377 [Psychrobacter arcticum 273-4]
>gi|71064978|ref|YP_263705.1| hypothetical protein Psyc_0404 [Psychrobacter arcticum 273-4]
>gi|71064979|ref|YP_263706.1| hypothetical protein Psyc_0405 [Psychrobacter arcticum 273-4]
>gi|71064982|ref|YP_263709.1| hypothetical protein Psyc_0410 [Psychrobacter arcticum 273-4]
>gi|71064995|ref|YP_263722.1| possible cytochrome C, class I [Psychrobacter arcticum 273-4]
>gi|71065006|ref|YP_263733.1| possible Peptidoglycan-binding LysM [Psychrobacter arcticum 273-4]
>gi|71065014|ref|YP_263741.1| probable phage protein tail protein [Psychrobacter arcticum 273-4]
>gi|71065078|ref|YP_263805.1| ribosomal protein L15 [Psychrobacter arcticum 273-4]
>gi|71065088|ref|YP_263815.1| hypothetical protein Psyc_0518 [Psychrobacter arcticum 273-4]
>gi|71065096|ref|YP_263823.1| hypothetical protein Psyc_0526 [Psychrobacter arcticum 273-4]
>gi|71065141|ref|YP_263868.1| macrolide secretion protein MacA, HlyD family [Psychrobacter arcticum 273-4]
>gi|71065266|ref|YP_263993.1| probable FxsA cytoplasmic membrane protein [Psychrobacter arcticum 273-4]
>gi|71065270|ref|YP_263997.1| probable D-alanyl-D-alanine carboxypeptidase [Psychrobacter arcticum 273-4]
>gi|71065273|ref|YP_264000.1| hypothetical protein Psyc_0707 [Psychrobacter arcticum 273-4]
>gi|71065372|ref|YP_264099.1| hypothetical protein Psyc_0812 [Psychrobacter arcticum 273-4]
>gi|71065391|ref|YP_264118.1| probable NUDIX hydrolase [Psychrobacter arcticum 273-4]
>gi|71065396|ref|YP_264123.1| hypothetical protein Psyc_0836 [Psychrobacter arcticum 273-4]
>gi|71065399|ref|YP_264126.1| MFS sugar efflux pump SotB [Psychrobacter arcticum 273-4]
>gi|71065415|ref|YP_264142.1| putative pseudouridine synthase [Psychrobacter arcticum 273-4]
>gi|71065442|ref|YP_264169.1| hypothetical protein Psyc_0882 [Psychrobacter arcticum 273-4]
>gi|71065466|ref|YP_264193.1| hypothetical protein Psyc_0906 [Psychrobacter arcticum 273-4]
>gi|71065481|ref|YP_264208.1| putative Hfq family protein [Psychrobacter arcticum 273-4]
>gi|71065567|ref|YP_264294.1| hypothetical protein Psyc_1007 [Psychrobacter arcticum 273-4]
>gi|71065574|ref|YP_264301.1| hypothetical protein Psyc_1014 [Psychrobacter arcticum 273-4]
>gi|71065641|ref|YP_264368.1| possible ATP-dependent DEAD/DEAH box RNA-helicase [Psychrobacter arcticum 273-4]
>gi|71065660|ref|YP_264387.1| probable acetyl-CoA carboxylase, biotin carboxyl carrier proteine [Psychrobacter arcticum 273-4]
>gi|71065691|ref|YP_264418.1| hypothetical protein Psyc_1133 [Psychrobacter arcticum 273-4]
>gi|71065706|ref|YP_264433.1| malonate/sodium symporter, MadL subunit [Psychrobacter arcticum 273-4]
>gi|71065796|ref|YP_264523.1| possible AEC family tranporter [Psychrobacter arcticum 273-4]
>gi|71065800|ref|YP_264527.1| putative 2-C-methyl-D-erythritol 2,4-cyclodiphosphate synthase [Psychrobacter arcticum 273-4]
>gi|71065828|ref|YP_264555.1| hypothetical protein Psyc_1271 [Psychrobacter arcticum 273-4]
>gi|71065831|ref|YP_264558.1| hypothetical protein Psyc_1274 [Psychrobacter arcticum 273-4]
>gi|71065888|ref|YP_264615.1| hypothetical protein Psyc_1331 [Psychrobacter arcticum 273-4]
>gi|71066047|ref|YP_264774.1| hypothetical protein Psyc_1492 [Psychrobacter arcticum 273-4]
>gi|71066059|ref|YP_264786.1| putative GTP-binding protein, GTP1/Obg family [Psychrobacter arcticum 273-4]
>gi|71066068|ref|YP_264795.1| hypothetical protein Psyc_1513 [Psychrobacter arcticum 273-4]
>gi|71066083|ref|YP_264810.1| probable UDP-3-O-(3-hydroxymyristoyl) glucosamine N-acyltransferase, LpxD [Psychrobacter arcticum 273-4]
>gi|71066130|ref|YP_264857.1| RND membrane fusion protein AcrA [Psychrobacter arcticum 273-4]
>gi|71066145|ref|YP_264872.1| possible copper-resistance protein CopB [Psychrobacter arcticum 273-4]
>gi|71066149|ref|YP_264876.1| hypothetical protein Psyc_1594 [Psychrobacter arcticum 273-4]
>gi|71066156|ref|YP_264883.1| hypothetical protein Psyc_1601 [Psychrobacter arcticum 273-4]
>gi|71066210|ref|YP_264937.1| possible rhomboid family protein [Psychrobacter arcticum 273-4]
>gi|71066219|ref|YP_264946.1| TonB/TolA energy transducing protein [Psychrobacter arcticum 273-4]
>gi|71066238|ref|YP_264965.1| putative single-strand binding protein [Psychrobacter arcticum 273-4]
>gi|71066255|ref|YP_264982.1| hypothetical protein Psyc_1700 [Psychrobacter arcticum 273-4]
>gi|71066290|ref|YP_265017.1| hypothetical protein Psyc_1735 [Psychrobacter arcticum 273-4]
>gi|71066322|ref|YP_265049.1| homoserine/homoserine lactone efflux pump, RhtB family [Psychrobacter arcticum 273-4]
>gi|71066336|ref|YP_265063.1| possible heme-hemopexin-binding/utilization protein [Psychrobacter arcticum 273-4]
>gi|71066346|ref|YP_265073.1| probable transcriptional regulator, ArsR family [Psychrobacter arcticum 273-4]
>gi|71066360|ref|YP_265087.1| outer membrane protein, OmpA/OmpF family [Psychrobacter arcticum 273-4]
>gi|71066383|ref|YP_265110.1| ABC exporter, inner membrane subunit CcmB [Psychrobacter arcticum 273-4]
>gi|71066410|ref|YP_265137.1| hypothetical protein Psyc_1855 [Psychrobacter arcticum 273-4]
>gi|71066419|ref|YP_265146.1| hypothetical protein Psyc_1864 [Psychrobacter arcticum 273-4]
>gi|71066427|ref|YP_265154.1| hypothetical protein Psyc_1872 [Psychrobacter arcticum 273-4]
>gi|71066430|ref|YP_265157.1| hypothetical protein Psyc_1875 [Psychrobacter arcticum 273-4]
>gi|71066443|ref|YP_265170.1| ribosomal protein L7/L12 [Psychrobacter arcticum 273-4]
>gi|71066527|ref|YP_265254.1| hypothetical protein Psyc_1973 [Psychrobacter arcticum 273-4]
>gi|71066529|ref|YP_265256.1| hypothetical protein Psyc_1975 [Psychrobacter arcticum 273-4]
>gi|71066545|ref|YP_265272.1| hypothetical protein Psyc_1991 [Psychrobacter arcticum 273-4]
>gi|71066547|ref|YP_265274.1| hypothetical protein Psyc_1993 [Psychrobacter arcticum 273-4]
>gi|71066582|ref|YP_265309.1| ATP synthase Fo, B subunit [Psychrobacter arcticum 273-4]
>gi|71066636|ref|YP_265363.1| hypothetical protein Psyc_2082 [Psychrobacter arcticum 273-4]
>gi|71066645|ref|YP_265372.1| hypothetical protein Psyc_2091 [Psychrobacter arcticum 273-4]
>gi|50841526|ref|YP_054753.1| hypothetical protein PPA0033 [Propionibacterium acnes KPA171202]
>gi|50841535|ref|YP_054762.1| putative membrane protein [Propionibacterium acnes KPA171202]
>gi|50841536|ref|YP_054763.1| putative membrane protein [Propionibacterium acnes KPA171202]
>gi|50841563|ref|YP_054790.1| membrane associated protein [Propionibacterium acnes KPA171202]
>gi|50841564|ref|YP_054791.1| hypothetical protein PPA0073 [Propionibacterium acnes KPA171202]

>gi|50841571|ref|YP_054798.1| chloride channel protein [Propionibacterium acnes KPA171202]
>gi|50841596|ref|YP_054823.1| transporter (putative iron (III) permease) [Propionibacterium acnes KPA171202]
>gi|50841618|ref|YP_054845.1| hypothetical membrane spanning protein [Propionibacterium acnes KPA171202]
>gi|50841635|ref|YP_054862.1| hypothetical protein PPA0146 [Propionibacterium acnes KPA171202]
>gi|50841661|ref|YP_054888.1| transport ATP-binding protein, CydCD [Propionibacterium acnes KPA171202]
>gi|50841710|ref|YP_054937.1| hypothetical protein PPA2396 [Propionibacterium acnes KPA171202]
>gi|50841714|ref|YP_054941.1| putative translation initiation inhibitor [Propionibacterium acnes KPA171202]
>gi|50841719|ref|YP_054946.1| hypothetical protein PPA0226 [Propionibacterium acnes KPA171202]
>gi|50841724|ref|YP_054951.1| hypothetical protein PPA0231 [Propionibacterium acnes KPA171202]
>gi|50841736|ref|YP_054963.1| ATPase [Propionibacterium acnes KPA171202]
>gi|50841743|ref|YP_054970.1| hypothetical protein PPA0251 [Propionibacterium acnes KPA171202]
>gi|50841754|ref|YP_054981.1| hypothetical protein PPA0264 [Propionibacterium acnes KPA171202]
>gi|50841757|ref|YP_054984.1| hypothetical protein PPA0267 [Propionibacterium acnes KPA171202]
>gi|50841759|ref|YP_054986.1| hypothetical protein PPA0270 [Propionibacterium acnes KPA171202]
>gi|50841797|ref|YP_055024.1| hypothetical protein PPA0310 [Propionibacterium acnes KPA171202]
>gi|50841812|ref|YP_055039.1| cytochrome c biogenesis protein [Propionibacterium acnes KPA171202]
>gi|50841839|ref|YP_055066.1| 2-C-methyl-D-erythritol 4-phosphate cytidylyltransferase [Propionibacterium acnes KPA171202]
>gi|50841848|ref|YP_055075.1| polysaccharide deacetylase [Propionibacterium acnes KPA171202]
>gi|50841866|ref|YP_055093.1| putative tRNA/rRNA methyltransferase [Propionibacterium acnes KPA171202]
>gi|50841871|ref|YP_055098.1| hypothetical protein PPA0388 [Propionibacterium acnes KPA171202]
>gi|50841888|ref|YP_055115.1| ABC transporter-associated permease [Propionibacterium acnes KPA171202]
>gi|50841918|ref|YP_055145.1| hypothetical protein PPA0435 [Propionibacterium acnes KPA171202]
>gi|50841925|ref|YP_055152.1| putative cobalamin-5-phosphate synthase, CobS [Propionibacterium acnes KPA171202]
>gi|50841932|ref|YP_055159.1| hypothetical membrane protein [Propionibacterium acnes KPA171202]
>gi|50841976|ref|YP_055203.1| hypothetical protein PPA2407 [Propionibacterium acnes KPA171202]
>gi|50842036|ref|YP_055263.1| hypothetical protein PPA0552 [Propionibacterium acnes KPA171202]
>gi|50842037|ref|YP_055264.1| hypothetical protein PPA2401 [Propionibacterium acnes KPA171202]
>gi|50842046|ref|YP_055273.1| hypothetical protein PPA0560 [Propionibacterium acnes KPA171202]
>gi|50842135|ref|YP_055362.1| hypothetical protein PPA0648 [Propionibacterium acnes KPA171202]
>gi|50842142|ref|YP_055369.1| hypothetical protein PPA0655 [Propionibacterium acnes KPA171202]
>gi|50842143|ref|YP_055370.1| iron transport system substrate-binding protein [Propionibacterium acnes KPA171202]
>gi|50842144|ref|YP_055371.1| iron transport system, permease protein [Propionibacterium acnes KPA171202]
>gi|50842181|ref|YP_055408.1| dihydrolipoamide acyltransferase [Propionibacterium acnes KPA171202]
>gi|50842185|ref|YP_055412.1| hypothetical protein PPA0697 [Propionibacterium acnes KPA171202]
>gi|50842205|ref|YP_055432.1| conserved protein, putative regulatory protein [Propionibacterium acnes KPA171202]
>gi|50842209|ref|YP_055436.1| putative NPL/P60 family secreted protein [Propionibacterium acnes KPA171202]
>gi|50842211|ref|YP_055438.1| hypothetical protein PPA0723 [Propionibacterium acnes KPA171202]
>gi|50842213|ref|YP_055440.1| conserved protein, putative acyltransferase [Propionibacterium acnes KPA171202]
>gi|50842244|ref|YP_055471.1| cell division protein FtsW [Propionibacterium acnes KPA171202]
>gi|50842271|ref|YP_055498.1| ABC-type transport system, fused ATPase and permease [Propionibacterium acnes KPA171202]
>gi|50842272|ref|YP_055499.1| dephospho-CoA kinase [Propionibacterium acnes KPA171202]
>gi|50842286|ref|YP_055513.1| putative permease [Propionibacterium acnes KPA171202]
>gi|50842313|ref|YP_055540.1| conserved protein, putative ribonuclease [Propionibacterium acnes KPA171202]
>gi|50842316|ref|YP_055543.1| hypothetical protein PPA0830 [Propionibacterium acnes KPA171202]
>gi|50842325|ref|YP_055552.1| hypothetical protein, putative N-acetylmuramoyl-L-alanine amidase [Propionibacterium acnes KPA171202]
>gi|50842326|ref|YP_055553.1| hypothetical protein PPA0840 [Propionibacterium acnes KPA171202]
>gi|50842327|ref|YP_055554.1| hypothetical protein PPA0841 [Propionibacterium acnes KPA171202]
>gi|50842329|ref|YP_055556.1| ABC transporter ATP-binding protein fragment [Propionibacterium acnes KPA171202]
>gi|50842376|ref|YP_055603.1| hypothetical protein, putative glucoamylase S1/S2 precursor [Propionibacterium acnes KPA171202]
>gi|50842380|ref|YP_055607.1| ComE operon protein 1 [Propionibacterium acnes KPA171202]
>gi|50842386|ref|YP_055613.1| O-succinylbenzoate-CoA synthase [Propionibacterium acnes KPA171202]
>gi|50842440|ref|YP_055667.1| hypothetical protein PPA0956 [Propionibacterium acnes KPA171202]
>gi|50842456|ref|YP_055683.1| hypothetical protein PPA0972 [Propionibacterium acnes KPA171202]
>gi|50842458|ref|YP_055685.1| hypothetical protein PPA2378 [Propionibacterium acnes KPA171202]
>gi|50842468|ref|YP_055695.1| malonyl CoA-acyl carrier protein malonyltransferase [Propionibacterium acnes KPA171202]
>gi|50842475|ref|YP_055702.1| hypothetical protein PPA0991 [Propionibacterium acnes KPA171202]
>gi|50842491|ref|YP_055718.1| putative competence-damage inducible protein [Propionibacterium acnes KPA171202]
>gi|50842497|ref|YP_055724.1| hypothetical protein PPA1014 [Propionibacterium acnes KPA171202]
>gi|50842515|ref|YP_055742.1| RNA polymerase principal sigma factor HrdB [Propionibacterium acnes KPA171202]
>gi|50842532|ref|YP_055759.1| hypothetical protein PPA1050 [Propionibacterium acnes KPA171202]
>gi|50842535|ref|YP_055762.1| hypothetical protein PPA1053 [Propionibacterium acnes KPA171202]
>gi|50842569|ref|YP_055796.1| hypothetical membrane associated protein [Propionibacterium acnes KPA171202]
>gi|50842578|ref|YP_055805.1| probable peptidyl-prolyl cis-trans isomerase B [Propionibacterium acnes KPA171202]
>gi|50842618|ref|YP_055845.1| hypothetical protein PPA1139 [Propionibacterium acnes KPA171202]
>gi|50842642|ref|YP_055869.1| translocase subunit SecD [Propionibacterium acnes KPA171202]
>gi|50842682|ref|YP_055909.1| hypothetical protein PPA1202 [Propionibacterium acnes KPA171202]
>gi|50842708|ref|YP_055935.1| cation-efflux transport protein [Propionibacterium acnes KPA171202]
>gi|50842712|ref|YP_055939.1| peptidyl-tRNA hydrolase domain [Propionibacterium acnes KPA171202]
>gi|50842727|ref|YP_055954.1| putative cyanate permease [Propionibacterium acnes KPA171202]
>gi|50842781|ref|YP_056008.1| hypothetical protein PPA1300 [Propionibacterium acnes KPA171202]

>gi|50842782|ref|YP_056009.1| hypothetical protein PPA1301 [Propionibacterium acnes KPA171202]
>gi|50842785|ref|YP_056012.1| hypothetical protein PPA1305 [Propionibacterium acnes KPA171202]
>gi|50842828|ref|YP_056055.1| hypothetical protein PPA1344 [Propionibacterium acnes KPA171202]
>gi|50842848|ref|YP_056075.1| putative transcriptional regulator [Propionibacterium acnes KPA171202]
>gi|50842849|ref|YP_056076.1| conserved protein YyaK [Propionibacterium acnes KPA171202]
>gi|50842866|ref|YP_056093.1| putative peptidyl-prolyl cis-trans isomerase [Propionibacterium acnes KPA171202]
>gi|50842882|ref|YP_056109.1| BioY family protein [Propionibacterium acnes KPA171202]
>gi|50842884|ref|YP_056111.1| hypothetical protein PPA1402 [Propionibacterium acnes KPA171202]
>gi|50842885|ref|YP_056112.1| hypothetical protein PPA1403 [Propionibacterium acnes KPA171202]
>gi|50842895|ref|YP_056122.1| translation initiation factor IF-3 [Propionibacterium acnes KPA171202]
>gi|50842969|ref|YP_056196.1| hypothetical membrane protein [Propionibacterium acnes KPA171202]
>gi|50842971|ref|YP_056198.1| translation initiation factor IF-2 [Propionibacterium acnes KPA171202]
>gi|50842994|ref|YP_056221.1| hypothetical membrane protein [Propionibacterium acnes KPA171202]
>gi|50842998|ref|YP_056225.1| elongation factor Ts (EF-Ts) [Propionibacterium acnes KPA171202]
>gi|50843001|ref|YP_056228.1| conserved membrane spanning protein [Propionibacterium acnes KPA171202]
>gi|50843007|ref|YP_056234.1| hypothetical protein PPA1529 [Propionibacterium acnes KPA171202]
>gi|50843015|ref|YP_056242.1| hypothetical protein PPA1537 [Propionibacterium acnes KPA171202]
>gi|50843033|ref|YP_056260.1| putative ABC transporter-associated permease [Propionibacterium acnes KPA171202]
>gi|50843049|ref|YP_056276.1| trigger factor (chaperone in protein export) [Propionibacterium acnes KPA171202]
>gi|50843071|ref|YP_056298.1| conserved phage-associated protein [Propionibacterium acnes KPA171202]
>gi|50843073|ref|YP_056300.1| hypothetical phage-associated protein [Propionibacterium acnes KPA171202]
>gi|50843114|ref|YP_056341.1| hypothetical protein PPA2356 [Propionibacterium acnes KPA171202]
>gi|50843115|ref|YP_056342.1| hypothetical protein PPA1649 [Propionibacterium acnes KPA171202]
>gi|50843123|ref|YP_056350.1| hypothetical protein PPA1660 [Propionibacterium acnes KPA171202]
>gi|50843127|ref|YP_056354.1| hypothetical adhesion protein [Propionibacterium acnes KPA171202]
>gi|50843129|ref|YP_056356.1| hypothetical adhesion protein fragment [Propionibacterium acnes KPA171202]
>gi|50843157|ref|YP_056384.1| hypothetical protein PPA1695 [Propionibacterium acnes KPA171202]
>gi|50843161|ref|YP_056388.1| cell envelope-related transcriptional attenuator [Propionibacterium acnes KPA171202]
>gi|50843163|ref|YP_056390.1| phosphoribosylaminoimidazole carboxylase catalytic subunit [Propionibacterium acnes KPA171202]
>gi|50843175|ref|YP_056402.1| hypothetical protein PPA1715 [Propionibacterium acnes KPA171202]
>gi|50843202|ref|YP_056429.1| hypothetical protein PPA1742 [Propionibacterium acnes KPA171202]
>gi|50843213|ref|YP_056440.1| hypothetical membrane protein [Propionibacterium acnes KPA171202]
>gi|50843217|ref|YP_056444.1| hypothetical protein PPA1757 [Propionibacterium acnes KPA171202]
>gi|50843220|ref|YP_056447.1| ABC transporter associated permease [Propionibacterium acnes KPA171202]
>gi|50843222|ref|YP_056449.1| hypothetical protein PPA1762 [Propionibacterium acnes KPA171202]
>gi|50843234|ref|YP_056461.1| hypothetical protein PPA1774 [Propionibacterium acnes KPA171202]
>gi|50843247|ref|YP_056474.1| ATP-dependent RNA helicase [Propionibacterium acnes KPA171202]
>gi|50843272|ref|YP_056499.1| ABC transporter associated permease [Propionibacterium acnes KPA171202]
>gi|50843282|ref|YP_056509.1| 50S ribosomal protein L17 [Propionibacterium acnes KPA171202]
>gi|50843301|ref|YP_056528.1| 30S ribosomal protein S5 [Propionibacterium acnes KPA171202]
>gi|50843310|ref|YP_056537.1| 50S ribosomal protein L29 [Propionibacterium acnes KPA171202]
>gi|50843312|ref|YP_056539.1| 30S ribosomal protein S3 [Propionibacterium acnes KPA171202]
>gi|50843331|ref|YP_056558.1| hypothetical protein PPA1877 [Propionibacterium acnes KPA171202]
>gi|50843333|ref|YP_056560.1| hypothetical protein PPA1879 [Propionibacterium acnes KPA171202]
>gi|50843334|ref|YP_056561.1| hypothetical protein, putative adhesion or S-layer protein [Propionibacterium acnes KPA171202]
>gi|50843339|ref|YP_056566.1| 50S ribosomal protein L7/L12 [Propionibacterium acnes KPA171202]
>gi|50843343|ref|YP_056570.1| transcription antitermination protein NusG [Propionibacterium acnes KPA171202]
>gi|50843344|ref|YP_056571.1| putative SecE/Sec61-gamma subunit of protein translocation complex [Propionibacterium acnes KPA171202]
>gi|50843355|ref|YP_056582.1| NADH dehydrogenase subunit [Propionibacterium acnes KPA171202]
>gi|50843373|ref|YP_056600.1| NADH dehydrogenase I chain N [Propionibacterium acnes KPA171202]
>gi|50843390|ref|YP_056617.1| hypothetical protein, putative ABC transporter-associated permease [Propionibacterium acnes KPA171202]
>gi|50843398|ref|YP_056625.1| PTS protein, putative dihydroxyacetone kinase phosphotransfer protein [Propionibacterium acnes KPA171202]
>gi|50843418|ref|YP_056645.1| conserved membrane protein [Propionibacterium acnes KPA171202]
>gi|50843451|ref|YP_056678.1| biotin carboxyl carrier protein of methylmalonyl-CoA carboxyl-transferase (transcarboxylase, 1.3S subunit) [Propionibacterium acnes KPA171202]
>gi|50843471|ref|YP_056698.1| manganese transport protein [Propionibacterium acnes KPA171202]
>gi|50843478|ref|YP_056705.1| conserved membrane protein (putative permease) [Propionibacterium acnes KPA171202]
>gi|50843485|ref|YP_056712.1| hypothetical membrane protein [Propionibacterium acnes KPA171202]
>gi|50843513|ref|YP_056740.1| hypothetical membrane protein [Propionibacterium acnes KPA171202]
>gi|50843545|ref|YP_056772.1| hypothetical membrane-spanning protein [Propionibacterium acnes KPA171202]
>gi|50843549|ref|YP_056776.1| hypothetical protein PPA2111 [Propionibacterium acnes KPA171202]
>gi|50843565|ref|YP_056792.1| hypothetical protein, putative adhesion or S-layer protein [Propionibacterium acnes KPA171202]
>gi|50843571|ref|YP_056798.1| putative aldolase [Propionibacterium acnes KPA171202]
>gi|50843578|ref|YP_056805.1| hypothetical membrane protein [Propionibacterium acnes KPA171202]
>gi|50843612|ref|YP_056839.1| rare lipoprotein A (RlpA) family protein [Propionibacterium acnes KPA171202]
>gi|50843616|ref|YP_056843.1| putative Na+/H+ antiporter [Propionibacterium acnes KPA171202]
>gi|50843620|ref|YP_056847.1| hypothetical protein PPA2183 [Propionibacterium acnes KPA171202]
>gi|50843626|ref|YP_056853.1| hypothetical protein PPA2189 [Propionibacterium acnes KPA171202]
>gi|50843642|ref|YP_056869.1| putative ABC transporter-associated permease [Propionibacterium acnes KPA171202]
>gi|50843645|ref|YP_056872.1| hypothetical protein, putative adhesion or S-layer protein [Propionibacterium acnes KPA171202]

>gi|50843664|ref|YP_056891.1| single-strand binding protein [Propionibacterium acnes KPA171202]
>gi|50843676|ref|YP_056903.1| putative metal-associated protein (copper chaperone) [Propionibacterium acnes KPA171202]
>gi|50843680|ref|YP_056907.1| hypothetical membrane protein [Propionibacterium acnes KPA171202]
>gi|50843696|ref|YP_056923.1| hypothetical membrane protein [Propionibacterium acnes KPA171202]
>gi|50843701|ref|YP_056928.1| alanine dehydrogenase [Propionibacterium acnes KPA171202]
>gi|50843703|ref|YP_056930.1| hypothetical protein PPA2374 [Propionibacterium acnes KPA171202]
>gi|50843711|ref|YP_056938.1| alanine dehydrogenase [Propionibacterium acnes KPA171202]
>gi|50843726|ref|YP_056953.1| hypothetical protein PPA2289 [Propionibacterium acnes KPA171202]
>gi|50843789|ref|YP_057016.1| JAG-like protein [Propionibacterium acnes KPA171202]
>gi|15675950|ref|NP_273068.1| hypothetical protein NMB0002 [Neisseria meningitidis MC58]
>gi|15675966|ref|NP_273084.1| pilin PilE [Neisseria meningitidis MC58]
>gi|15675985|ref|NP_273111.1| signal recognition particle protein [Neisseria meningitidis MC58]
>gi|15676032|ref|NP_273162.1| hypothetical protein NMB0104 [Neisseria meningitidis MC58]
>gi|15676059|ref|NP_273189.1| 50S ribosomal protein L7/L12 [Neisseria meningitidis MC58]
>gi|15676086|ref|NP_273217.1| 30s ribosomal protein S5 [Neisseria meningitidis MC58]
>gi|15676116|ref|NP_273247.1| hypothetical protein NMB0189 [Neisseria meningitidis MC58]
>gi|15676163|ref|NP_273295.1| hypothetical protein NMB0239 [Neisseria meningitidis MC58]
>gi|15676209|ref|NP_273341.1| hypothetical protein NMB0286 [Neisseria meningitidis MC58]
>gi|15676211|ref|NP_273343.1| hypothetical protein NMB0288 [Neisseria meningitidis MC58]
>gi|15676226|ref|NP_273358.1| dihydrofolate reductase [Neisseria meningitidis MC58]
>gi|15676235|ref|NP_273367.1| fatty acid efflux system protein [Neisseria meningitidis MC58]
>gi|15676256|ref|NP_273390.1| tspA protein [Neisseria meningitidis MC58]
>gi|15676296|ref|NP_273431.1| outer membrane protein class 4 [Neisseria meningitidis MC58]
>gi|15676297|ref|NP_273432.1| hypothetical protein NMB0383 [Neisseria meningitidis MC58]
>gi|15676300|ref|NP_273435.1| phosphatidylglycerophosphatase A [Neisseria meningitidis MC58]
>gi|15676305|ref|NP_273440.1| beta-phosphoglucomutase [Neisseria meningitidis MC58]
>gi|15676317|ref|NP_273453.1| hypothetical protein NMB0404 [Neisseria meningitidis MC58]
>gi|15676319|ref|NP_273455.1| hypothetical protein NMB0406 [Neisseria meningitidis MC58]
>gi|15676328|ref|NP_273464.1| UDP-N-acetylmuramoylalanyl-D-glutamyl-2,6-diaminopimelate--D-alanyl-D-alanyl ligase [Neisseria meningitidis MC58]
>gi|15676331|ref|NP_273467.1| hypothetical protein NMB0419 [Neisseria meningitidis MC58]
>gi|15676389|ref|NP_273525.1| hypothetical protein NMB0478 [Neisseria meningitidis MC58]
>gi|15676394|ref|NP_273530.1| hypothetical protein NMB0483 [Neisseria meningitidis MC58]
>gi|15676408|ref|NP_273545.1| hypothetical protein NMB0499 [Neisseria meningitidis MC58]
>gi|15676411|ref|NP_273548.1| hypothetical protein NMB0502 [Neisseria meningitidis MC58]
>gi|15676415|ref|NP_273552.1| hypothetical protein NMB0506 [Neisseria meningitidis MC58]
>gi|15676420|ref|NP_273557.1| hypothetical protein NMB0511 [Neisseria meningitidis MC58]
>gi|15676423|ref|NP_273560.1| hypothetical protein NMB0514 [Neisseria meningitidis MC58]
>gi|15676428|ref|NP_273565.1| hypothetical protein NMB0519 [Neisseria meningitidis MC58]
>gi|15676451|ref|NP_273590.1| hypothetical protein NMB0545 [Neisseria meningitidis MC58]
>gi|15676463|ref|NP_273602.1| hypothetical protein NMB0558 [Neisseria meningitidis MC58]
>gi|15676465|ref|NP_273604.1| serine acetyltransferase [Neisseria meningitidis MC58]
>gi|15676502|ref|NP_273641.1| hypothetical protein NMB0597 [Neisseria meningitidis MC58]
>gi|15676519|ref|NP_273659.1| ammonium transporter AmtB, putative [Neisseria meningitidis MC58]
>gi|15676556|ref|NP_273699.1| hypothetical protein NMB0657 [Neisseria meningitidis MC58]
>gi|15676589|ref|NP_273733.1| colicin V production protein, putative [Neisseria meningitidis MC58]
>gi|15676590|ref|NP_273734.1| tpc protein [Neisseria meningitidis MC58]
>gi|15676598|ref|NP_273742.1| IgA-specific serine endopeptidase [Neisseria meningitidis MC58]
>gi|15676599|ref|NP_273743.1| hypothetical protein NMB0701 [Neisseria meningitidis MC58]
>gi|15676600|ref|NP_273744.1| competence protein ComA [Neisseria meningitidis MC58]
>gi|15676620|ref|NP_273764.1| 50S ribosomal protein L35 [Neisseria meningitidis MC58]
>gi|15676807|ref|NP_273952.1| hypothetical protein NMB0912 [Neisseria meningitidis MC58]
>gi|15676826|ref|NP_273971.1| hypothetical protein NMB0932 [Neisseria meningitidis MC58]
>gi|15676845|ref|NP_273990.1| hypothetical protein NMB0952 [Neisseria meningitidis MC58]
>gi|15676849|ref|NP_273994.1| 2-oxoglutarate dehydrogenase, E2 component, dihydrolipoamide succinyltransferase [Neisseria meningitidis MC58]
>gi|15676865|ref|NP_274010.1| hypothetical protein NMB0973 [Neisseria meningitidis MC58]
>gi|15676871|ref|NP_274016.1| hypothetical protein NMB0979 [Neisseria meningitidis MC58]
>gi|15676922|ref|NP_274069.1| hypothetical protein NMB1035 [Neisseria meningitidis MC58]
>gi|15676946|ref|NP_274095.1| hypothetical protein NMB1062 [Neisseria meningitidis MC58]
>gi|15676949|ref|NP_274098.1| crcB protein [Neisseria meningitidis MC58]
>gi|15676950|ref|NP_274099.1| hypothetical protein NMB1066 [Neisseria meningitidis MC58]
>gi|15676980|ref|NP_274131.1| hypothetical protein NMB1100 [Neisseria meningitidis MC58]
>gi|15676984|ref|NP_274135.1| phage sheath protein [Neisseria meningitidis MC58]
>gi|15677092|ref|NP_274244.1| transporter, putative [Neisseria meningitidis MC58]
>gi|15677094|ref|NP_274246.1| hypothetical protein NMB1221 [Neisseria meningitidis MC58]
>gi|15677133|ref|NP_274286.1| hypothetical protein NMB1265 [Neisseria meningitidis MC58]
>gi|15677151|ref|NP_274304.1| hypothetical protein NMB1284 [Neisseria meningitidis MC58]
>gi|15677199|ref|NP_274352.1| hypothetical protein NMB1333 [Neisseria meningitidis MC58]
>gi|15677208|ref|NP_274361.1| pyruvate dehydrogenase, E2 component, dihydrolipoamide acetyltransferase [Neisseria meningitidis MC58]
>gi|15677234|ref|NP_274387.1| hypothetical protein NMB1369 [Neisseria meningitidis MC58]

>gi|15677248|ref|NP_274401.1| hypothetical protein NMB1387 [Neisseria meningitidis MC58]
>gi|15677267|ref|NP_274420.1| hypothetical protein NMB1406 [Neisseria meningitidis MC58]
>gi|15677268|ref|NP_274421.1| hypothetical protein NMB1408 [Neisseria meningitidis MC58]
>gi|15677274|ref|NP_274427.1| iron-regulated protein FrpC [Neisseria meningitidis MC58]
>gi|15677322|ref|NP_274477.1| hypothetical protein NMB1468 [Neisseria meningitidis MC58]
>gi|15677336|ref|NP_274491.1| lipoprotein NlpD, putative [Neisseria meningitidis MC58]
>gi|15677360|ref|NP_274515.1| hypothetical protein NMB1507 [Neisseria meningitidis MC58]
>gi|15677365|ref|NP_274520.1| YgbB/YacN family protein [Neisseria meningitidis MC58]
>gi|15677376|ref|NP_274531.1| hypothetical protein NMB1523 [Neisseria meningitidis MC58]
>gi|15677385|ref|NP_274540.1| H.8 outer membrane protein [Neisseria meningitidis MC58]
>gi|15677400|ref|NP_274555.1| tspB protein, putative [Neisseria meningitidis MC58]
>gi|15677413|ref|NP_274569.1| hypothetical protein NMB1562 [Neisseria meningitidis MC58]
>gi|15677418|ref|NP_274574.1| macrophage infectivity potentiator [Neisseria meningitidis MC58]
>gi|15677428|ref|NP_274584.1| hypothetical protein NMB1578 [Neisseria meningitidis MC58]
>gi|15677440|ref|NP_274596.1| hypothetical protein NMB1590 [Neisseria meningitidis MC58]
>gi|15677449|ref|NP_274605.1| hypothetical protein NMB1599 [Neisseria meningitidis MC58]
>gi|15677453|ref|NP_274609.1| tellurite resistance protein, putative [Neisseria meningitidis MC58]
>gi|15677492|ref|NP_274648.1| translation initiation factor IF-2 [Neisseria meningitidis MC58]
>gi|15677524|ref|NP_274680.1| hypothetical protein NMB1675 [Neisseria meningitidis MC58]
>gi|15677525|ref|NP_274681.1| cytochrome c5 [Neisseria meningitidis MC58]
>gi|15677545|ref|NP_274701.1| acyl carrier protein, putative [Neisseria meningitidis MC58]
>gi|15677559|ref|NP_274715.1| L-lactate permease-related protein [Neisseria meningitidis MC58]
>gi|15677576|ref|NP_274733.1| TonB protein [Neisseria meningitidis MC58]
>gi|15677588|ref|NP_274745.1| hypothetical protein NMB1744 [Neisseria meningitidis MC58]
>gi|15677612|ref|NP_274771.1| hypothetical protein NMB1771 [Neisseria meningitidis MC58]
>gi|15677613|ref|NP_274772.1| hypothetical protein NMB1772 [Neisseria meningitidis MC58]
>gi|15677616|ref|NP_274775.1| hypothetical protein NMB1775 [Neisseria meningitidis MC58]
>gi|15677624|ref|NP_274783.1| hypothetical protein NMB1784 [Neisseria meningitidis MC58]
>gi|15677654|ref|NP_274815.1| lipopolysaccharide biosynthesis protein, putative [Neisseria meningitidis MC58]
>gi|15677679|ref|NP_274840.1| transcriptional regulator, MarR family [Neisseria meningitidis MC58]
>gi|15677683|ref|NP_274844.1| hypothetical protein NMB1848 [Neisseria meningitidis MC58]
>gi|15677695|ref|NP_274856.1| acetyl-CoA carboxylase, biotin carboxyl carrier protein [Neisseria meningitidis MC58]
>gi|15677718|ref|NP_274879.1| hypothetical protein NMB1883 [Neisseria meningitidis MC58]
>gi|15677723|ref|NP_274884.1| protein-export membrane protein SecG [Neisseria meningitidis MC58]
>gi|15677763|ref|NP_274927.1| ATP synthase F1, epsilon subunit [Neisseria meningitidis MC58]
>gi|15677768|ref|NP_274932.1| ATP synthase F0, B subunit [Neisseria meningitidis MC58]
>gi|15677769|ref|NP_274933.1| ATP synthase F0, C subunit [Neisseria meningitidis MC58]
>gi|15677771|ref|NP_274935.1| hypothetical protein NMB1941 [Neisseria meningitidis MC58]
>gi|15677780|ref|NP_274944.1| 30S ribosomal protein S21 [Neisseria meningitidis MC58]
>gi|15677785|ref|NP_274949.1| cadmium resistance protein [Neisseria meningitidis MC58]
>gi|15677787|ref|NP_274951.1| hypothetical protein NMB1957 [Neisseria meningitidis MC58]
>gi|15677822|ref|NP_274986.1| adhesin/invasin, putative [Neisseria meningitidis MC58]
>gi|15677834|ref|NP_274998.1| chloride channel protein-related protein [Neisseria meningitidis MC58]
>gi|15677852|ref|NP_275019.1| gluconate permease [Neisseria meningitidis MC58]
>gi|15677889|ref|NP_275057.1| hypothetical protein NMB2067 [Neisseria meningitidis MC58]
>gi|15677896|ref|NP_275064.1| hypothetical protein NMB2074 [Neisseria meningitidis MC58]
>gi|15677912|ref|NP_275080.1| hypothetical protein NMB2092 [Neisseria meningitidis MC58]
>gi|15677953|ref|NP_275125.1| hypothetical protein NMB2140 [Neisseria meningitidis MC58]
>gi|15677954|ref|NP_275126.1| hypothetical protein NMB2141 [Neisseria meningitidis MC58]
>gi|15677981|ref|NP_273092.1| pilS cassette [Neisseria meningitidis MC58]
>gi|15678002|ref|NP_274061.1| dnaJ protein [Neisseria meningitidis MC58]
>gi|33864550|ref|NP_896109.1| signal recognition particle docking protein FtsY [Synechococcus sp. WH 8102]
>gi|33864553|ref|NP_896112.1| RNA-binding region RNP-1 (RNA recognition motif) [Synechococcus sp. WH 8102]
>gi|33864570|ref|NP_896129.1| biotin carboxyl carrier protein (BCCP) subunit of acetyl-CoA carboxylase [Synechococcus sp. WH 8102]
>gi|33864572|ref|NP_896131.1| hypothetical protein SYNW0036 [Synechococcus sp. WH 8102]
>gi|33864577|ref|NP_896136.1| hypothetical protein SYNW0041 [Synechococcus sp. WH 8102]
>gi|33864597|ref|NP_896156.1| DNA polymerase, gamma and tau subunits [Synechococcus sp. WH 8102]
>gi|33864605|ref|NP_896164.1| hypothetical protein SYNW0069 [Synechococcus sp. WH 8102]
>gi|33864610|ref|NP_896169.1| putative Precorrin-8X methylmutase CobH [Synechococcus sp. WH 8102]
>gi|33864611|ref|NP_896170.1| possible MesJ homolog [Synechococcus sp. WH 8102]
>gi|33864621|ref|NP_896180.1| SwmA-cell surface protein required for swimming motility [Synechococcus sp. WH 8102]
>gi|33864634|ref|NP_896193.1| hypothetical protein SYNW0098 [Synechococcus sp. WH 8102]
>gi|33864645|ref|NP_896204.1| hypothetical protein SYNW0109 [Synechococcus sp. WH 8102]
>gi|33864660|ref|NP_896219.1| hypothetical protein SYNW0124 [Synechococcus sp. WH 8102]
>gi|33864665|ref|NP_896224.1| hypothetical protein SYNW0129 [Synechococcus sp. WH 8102]
>gi|33864693|ref|NP_896252.1| putative Na+/H+ antiporter, CPA2 family [Synechococcus sp. WH 8102]
>gi|33864694|ref|NP_896253.1| hypothetical protein SYNW0158 [Synechococcus sp. WH 8102]
>gi|33864722|ref|NP_896281.1| possible phosphatase [Synechococcus sp. WH 8102]
>gi|33864725|ref|NP_896284.1| hypothetical protein SYNW0189 [Synechococcus sp. WH 8102]
>gi|33864752|ref|NP_896311.1| possible 2-amino-4-hydroxy-6-hydroxymethyldihydropteridine pyrophosphokinase [Synechococcus sp. WH 8102]

>gi|33864753|ref|NP_896312.1| ABC transporter, ATP binding component [Synechococcus sp. WH 8102]
>gi|33864774|ref|NP_896333.1| Orotate phosphoribosyltransferase [Synechococcus sp. WH 8102]
>gi|33864775|ref|NP_896334.1| hypothetical protein SYNW0239 [Synechococcus sp. WH 8102]
>gi|33864779|ref|NP_896338.1| photosystem II 4 Kda protein psbK precursor [Synechococcus sp. WH 8102]
>gi|33864781|ref|NP_896340.1| Cobalamin-5-phosphate synthase CobS [Synechococcus sp. WH 8102]
>gi|33864791|ref|NP_896350.1| hypothetical protein SYNW0255 [Synechococcus sp. WH 8102]
>gi|33864796|ref|NP_896355.1| putative glycosyltransferase [Synechococcus sp. WH 8102]
>gi|33864800|ref|NP_896359.1| hypothetical protein SYNW0264 [Synechococcus sp. WH 8102]
>gi|33864810|ref|NP_896369.1| hypothetical protein SYNW0274 [Synechococcus sp. WH 8102]
>gi|33864820|ref|NP_896379.1| possible bicarbonate transporter, ICT family [Synechococcus sp. WH 8102]
>gi|33864826|ref|NP_896385.1| hypothetical protein SYNW0290 [Synechococcus sp. WH 8102]
>gi|33864828|ref|NP_896387.1| glutamyl-tRNA (Gln) amidotransferase subunit C [Synechococcus sp. WH 8102]
>gi|33864838|ref|NP_896397.1| hypothetical protein SYNW0302 [Synechococcus sp. WH 8102]
>gi|33864843|ref|NP_896402.1| hypothetical protein SYNW0307 [Synechococcus sp. WH 8102]
>gi|33864868|ref|NP_896427.1| hypothetical protein SYNW0332 [Synechococcus sp. WH 8102]
>gi|33864869|ref|NP_896428.1| hypothetical protein SYNW0333 [Synechococcus sp. WH 8102]
>gi|33864871|ref|NP_896430.1| YGGT family, conserved hypothetical integral membrane protein [Synechococcus sp. WH 8102]
>gi|33864876|ref|NP_896435.1| hypothetical protein SYNW0340 [Synechococcus sp. WH 8102]
>gi|33864877|ref|NP_896436.1| putative chromosome segregation protein, SMC ATPase superfamily [Synechococcus sp. WH 8102]
>gi|33864881|ref|NP_896440.1| hypothetical protein SYNW0345 [Synechococcus sp. WH 8102]
>gi|33864886|ref|NP_896445.1| possible DNA repair protein sms homolog [truncated] [Synechococcus sp. WH 8102]
>gi|33864942|ref|NP_896501.1| hypothetical protein SYNW0406 [Synechococcus sp. WH 8102]
>gi|33864945|ref|NP_896504.1| hypothetical protein SYNW0409 [Synechococcus sp. WH 8102]
>gi|33864949|ref|NP_896508.1| hypothetical protein SYNW0413 [Synechococcus sp. WH 8102]
>gi|33864983|ref|NP_896542.1| putative hexapeptide transferase family protein [Synechococcus sp. WH 8102]
>gi|33864987|ref|NP_896546.1| putative O-acetyltransferase [Synechococcus sp. WH 8102]
>gi|33864993|ref|NP_896552.1| hypothetical protein SYNW0457 [Synechococcus sp. WH 8102]
>gi|33864995|ref|NP_896554.1| possible UDP-glucose 4-epimerase [Synechococcus sp. WH 8102]
>gi|33865000|ref|NP_896559.1| hypothetical protein SYNW0464 [Synechococcus sp. WH 8102]
>gi|33865008|ref|NP_896567.1| putative c-type cytochrome biogenesis protein CcdA [Synechococcus sp. WH 8102]
>gi|33865024|ref|NP_896583.1| possible ATP synthase protein 1 [Synechococcus sp. WH 8102]
>gi|33865026|ref|NP_896585.1| ATP synthase subunit c [Synechococcus sp. WH 8102]
>gi|33865027|ref|NP_896586.1| putative ATP synthase subunit B' [Synechococcus sp. WH 8102]
>gi|33865028|ref|NP_896587.1| putative ATP synthase B chain [Synechococcus sp. WH 8102]
>gi|33865037|ref|NP_896596.1| alanine dehydrogenase [Synechococcus sp. WH 8102]
>gi|33865040|ref|NP_896599.1| possible GTPase [Synechococcus sp. WH 8102]
>gi|33865041|ref|NP_896600.1| hypothetical protein SYNW0505 [Synechococcus sp. WH 8102]
>gi|33865044|ref|NP_896603.1| hypothetical protein SYNW0508 [Synechococcus sp. WH 8102]
>gi|33865046|ref|NP_896605.1| hypothetical protein SYNW0510 [Synechococcus sp. WH 8102]
>gi|33865064|ref|NP_896623.1| hypothetical protein SYNW0528 [Synechococcus sp. WH 8102]
>gi|33865070|ref|NP_896629.1| putative methyltransferase for Ribosomal protein L11 [Synechococcus sp. WH 8102]
>gi|33865076|ref|NP_896635.1| putative N-acetylglucosamine-6-phosphate isomerase [Synechococcus sp. WH 8102]
>gi|33865089|ref|NP_896648.1| hypothetical protein SYNW0553 [Synechococcus sp. WH 8102]
>gi|33865098|ref|NP_896657.1| Leucine aminopeptidase [Synechococcus sp. WH 8102]
>gi|33865113|ref|NP_896672.1| conserved hypothetical membrane protein [Synechococcus sp. WH 8102]
>gi|33865116|ref|NP_896675.1| hypothetical protein SYNW0580 [Synechococcus sp. WH 8102]
>gi|33865117|ref|NP_896676.1| hypothetical protein SYNW0581 [Synechococcus sp. WH 8102]
>gi|33865126|ref|NP_896685.1| hypothetical protein SYNW0592 [Synechococcus sp. WH 8102]
>gi|33865130|ref|NP_896689.1| hypothetical protein SYNW0596 [Synechococcus sp. WH 8102]
>gi|33865132|ref|NP_896691.1| possible N-terminal part of IF-2 [Synechococcus sp. WH 8102]
>gi|33865139|ref|NP_896698.1| hypothetical protein SYNW0605 [Synechococcus sp. WH 8102]
>gi|33865153|ref|NP_896712.1| Sodium:solute symporter family, possibly glucose transporter [Synechococcus sp. WH 8102]
>gi|33865156|ref|NP_896715.1| hypothetical protein SYNW0622 [Synechococcus sp. WH 8102]
>gi|33865161|ref|NP_896720.1| DnaJ-like protein [Synechococcus sp. WH 8102]
>gi|33865169|ref|NP_896728.1| Sodium/bile acid cotransporter family [Synechococcus sp. WH 8102]
>gi|33865184|ref|NP_896743.1| hypothetical protein SYNW0650 [Synechococcus sp. WH 8102]
>gi|33865192|ref|NP_896751.1| possible cobalt transport protein [Synechococcus sp. WH 8102]
>gi|33865205|ref|NP_896764.1| Putative dihydrolipoamide acetyltransferase component (E2) of pyruvate... [Synechococcus sp. WH 8102]
>gi|33865215|ref|NP_896774.1| predicted alpha/beta hydrolase superfamily protein [Synechococcus sp. WH 8102]
>gi|33865224|ref|NP_896783.1| hypothetical protein SYNW0690 [Synechococcus sp. WH 8102]
>gi|33865228|ref|NP_896787.1| putative nicotinamide nucleotide transhydrogenase, subunit beta [Synechococcus sp. WH 8102]
>gi|33865233|ref|NP_896792.1| putative sodium-dependent transporter, NSS family [Synechococcus sp. WH 8102]
>gi|33865253|ref|NP_896812.1| probable tRNA/rRNA methyltransferase [Synechococcus sp. WH 8102]
>gi|33865260|ref|NP_896819.1| hypothetical protein SYNW0726 [Synechococcus sp. WH 8102]
>gi|33865266|ref|NP_896825.1| hypothetical protein SYNW0732 [Synechococcus sp. WH 8102]
>gi|33865273|ref|NP_896832.1| hypothetical protein SYNW0739 [Synechococcus sp. WH 8102]
>gi|33865274|ref|NP_896833.1| putuative bifunctional Methylenetetrahydrofolate dehydrogenase Methenyltetrahydrofolate/cyclohydrolase [Synechococcus sp. WH 8102]
>gi|33865280|ref|NP_896839.1| putative phosphoglycerate mutase family protein [Synechococcus sp. WH 8102]
>gi|33865291|ref|NP_896850.1| hypothetical protein SYNW0757 [Synechococcus sp. WH 8102]

>gi|33865303|ref|NP_896862.1| hypothetical protein SYNW0769 [Synechococcus sp. WH 8102]
>gi|33865305|ref|NP_896864.1| CDP-diacylglycerol-glycerol-3-phosphate 3-phosphatidyltransferase [Synechococcus sp. WH 8102]
>gi|33865309|ref|NP_896868.1| hypothetical protein SYNW0775 [Synechococcus sp. WH 8102]
>gi|33865314|ref|NP_896873.1| hypothetical protein SYNW0780 [Synechococcus sp. WH 8102]
>gi|33865317|ref|NP_896876.1| UDP-3-O-[3-hydroxymyristoyl] glucosamine N-acyltransferase [Synechococcus sp. WH 8102]
>gi|33865321|ref|NP_896880.1| putative type 4 prepilin peptidase [Synechococcus sp. WH 8102]
>gi|33865328|ref|NP_896887.1| hypothetical protein SYNW0794 [Synechococcus sp. WH 8102]
>gi|33865344|ref|NP_896903.1| hypothetical protein SYNW0810 [Synechococcus sp. WH 8102]
>gi|33865367|ref|NP_896926.1| hypothetical protein SYNW0833 [Synechococcus sp. WH 8102]
>gi|33865375|ref|NP_896934.1| ABC transporter for amino acids, membrane component [Synechococcus sp. WH 8102]
>gi|33865376|ref|NP_896935.1| ABC transporter for amino acids, membrane component [Synechococcus sp. WH 8102]
>gi|33865379|ref|NP_896938.1| possible AEC transporter family [Synechococcus sp. WH 8102]
>gi|33865380|ref|NP_896939.1| hypothetical protein SYNW0846 [Synechococcus sp. WH 8102]
>gi|33865392|ref|NP_896951.1| hypothetical protein SYNW0858 [Synechococcus sp. WH 8102]
>gi|33865398|ref|NP_896957.1| hypothetical protein SYNW0864 [Synechococcus sp. WH 8102]
>gi|33865408|ref|NP_896967.1| hypothetical protein SYNW0874 [Synechococcus sp. WH 8102]
>gi|33865415|ref|NP_896974.1| hypothetical protein SYNW0881 [Synechococcus sp. WH 8102]
>gi|33865423|ref|NP_896982.1| hypothetical protein SYNW0889 [Synechococcus sp. WH 8102]
>gi|33865431|ref|NP_896990.1| possible carbonic anhydrase [Synechococcus sp. WH 8102]
>gi|33865434|ref|NP_896993.1| putative cation efflux transporter (CDF family) [Synechococcus sp. WH 8102]
>gi|33865437|ref|NP_896996.1| hypothetical protein SYNW0903 [Synechococcus sp. WH 8102]
>gi|33865473|ref|NP_897032.1| putative glycerol dehydrogenase [Synechococcus sp. WH 8102]
>gi|33865476|ref|NP_897035.1| putative phosphatidate cytidylyltransferase [Synechococcus sp. WH 8102]
>gi|33865478|ref|NP_897037.1| Orn/Lys/Arg decarboxylases family 1 [Synechococcus sp. WH 8102]
>gi|33865480|ref|NP_897039.1| hypothetical protein SYNW0946 [Synechococcus sp. WH 8102]
>gi|33865484|ref|NP_897043.1| hypothetical protein SYNW0950 [Synechococcus sp. WH 8102]
>gi|33865487|ref|NP_897046.1| hypothetical protein SYNW0953 [Synechococcus sp. WH 8102]
>gi|33865495|ref|NP_897054.1| hypothetical protein SYNW0961 [Synechococcus sp. WH 8102]
>gi|33865503|ref|NP_897062.1| ABC transporter component, possibly Mn transport [Synechococcus sp. WH 8102]
>gi|33865506|ref|NP_897065.1| hypothetical protein SYNW0972 [Synechococcus sp. WH 8102]
>gi|33865509|ref|NP_897068.1| possible permease [Synechococcus sp. WH 8102]
>gi|33865513|ref|NP_897072.1| hypothetical protein SYNW0979 [Synechococcus sp. WH 8102]
>gi|33865518|ref|NP_897077.1| putative secreted calcium-binding protein [Synechococcus sp. WH 8102]
>gi|33865531|ref|NP_897090.1| Menaquinone biosynthesis protein; 2-succinyl-6-hydroxy-2,4-cyclohexadiene-1-carboxylate synthase [Synechococcus sp. WH 8102]
>gi|33865535|ref|NP_897094.1| hypothetical protein SYNW1001 [Synechococcus sp. WH 8102]
>gi|33865563|ref|NP_897122.1| possible tRNA/rRNA methyltransferase [Synechococcus sp. WH 8102]
>gi|33865584|ref|NP_897143.1| hypothetical protein SYNW1050 [Synechococcus sp. WH 8102]
>gi|33865587|ref|NP_897146.1| Putative 4-diphosphocytidyl-2C-methyl-D-erythritol kinase (CMK) [Synechococcus sp. WH 8102]
>gi|33865590|ref|NP_897149.1| preprotein translocase subunit [Synechococcus sp. WH 8102]
>gi|33865592|ref|NP_897151.1| hypothetical protein SYNW1058 [Synechococcus sp. WH 8102]
>gi|33865597|ref|NP_897156.1| hypothetical protein SYNW1063 [Synechococcus sp. WH 8102]
>gi|33865598|ref|NP_897157.1| hypothetical protein SYNW1064 [Synechococcus sp. WH 8102]
>gi|33865604|ref|NP_897163.1| hypothetical protein SYNW1070 [Synechococcus sp. WH 8102]
>gi|33865611|ref|NP_897170.1| Pyridoxal-dependent decarboxylase family protein [Synechococcus sp. WH 8102]
>gi|33865614|ref|NP_897173.1| hypothetical protein SYNW1080 [Synechococcus sp. WH 8102]
>gi|33865619|ref|NP_897178.1| possible ABC transporter component [Synechococcus sp. WH 8102]
>gi|33865647|ref|NP_897206.1| possible permease [Synechococcus sp. WH 8102]
>gi|33865660|ref|NP_897219.1| hypothetical protein SYNW1126 [Synechococcus sp. WH 8102]
>gi|33865662|ref|NP_897221.1| hypothetical protein SYNW1128 [Synechococcus sp. WH 8102]
>gi|33865686|ref|NP_897245.1| hypothetical protein SYNW1152 [Synechococcus sp. WH 8102]
>gi|33865691|ref|NP_897250.1| hypothetical protein SYNW1157 [Synechococcus sp. WH 8102]
>gi|33865702|ref|NP_897261.1| putative phosphonate ABC transporter [Synechococcus sp. WH 8102]
>gi|33865706|ref|NP_897265.1| hypothetical protein SYNW1172 [Synechococcus sp. WH 8102]
>gi|33865710|ref|NP_897269.1| hypothetical protein SYNW1176 [Synechococcus sp. WH 8102]
>gi|33865714|ref|NP_897273.1| hypothetical protein SYNW1180 [Synechococcus sp. WH 8102]
>gi|33865724|ref|NP_897283.1| hypothetical protein SYNW1190 [Synechococcus sp. WH 8102]
>gi|33865725|ref|NP_897284.1| TerC family protein [Synechococcus sp. WH 8102]
>gi|33865733|ref|NP_897292.1| hypothetical protein SYNW1199 [Synechococcus sp. WH 8102]
>gi|33865737|ref|NP_897296.1| hypothetical protein SYNW1203 [Synechococcus sp. WH 8102]
>gi|33865738|ref|NP_897297.1| hypothetical protein SYNW1204 [Synechococcus sp. WH 8102]
>gi|33865770|ref|NP_897329.1| hypothetical protein SYNW1236 [Synechococcus sp. WH 8102]
>gi|33865783|ref|NP_897342.1| hypothetical protein SYNW1249 [Synechococcus sp. WH 8102]
>gi|33865801|ref|NP_897360.1| hypothetical protein SYNW1267 [Synechococcus sp. WH 8102]
>gi|33865802|ref|NP_897361.1| DnaJ2 protein [Synechococcus sp. WH 8102]
>gi|33865817|ref|NP_897376.1| ABC transporter for possibly for trehalose/maltose, membrane component [Synechococcus sp. WH 8102]
>gi|33865818|ref|NP_897377.1| ABC transporter, likely for trehalose/maltose, membrane component [Synechococcus sp. WH 8102]
>gi|33865822|ref|NP_897381.1| hypothetical protein SYNW1288 [Synechococcus sp. WH 8102]
>gi|33865823|ref|NP_897382.1| hypothetical protein SYNW1289 [Synechococcus sp. WH 8102]
>gi|33865833|ref|NP_897392.1| possible ABC transporter [Synechococcus sp. WH 8102]

>gi|33865845|ref|NP_897404.1| hypothetical protein SYNW1311 [Synechococcus sp. WH 8102]
>gi|33865857|ref|NP_897416.1| putative chromate transport protein, CHR family [Synechococcus sp. WH 8102]
>gi|33865867|ref|NP_897426.1| hypothetical protein SYNW1333 [Synechococcus sp. WH 8102]
>gi|33865871|ref|NP_897430.1| ABC transporter component, possibly Mn transport [Synechococcus sp. WH 8102]
>gi|33865895|ref|NP_897454.1| hypothetical protein SYNW1361 [Synechococcus sp. WH 8102]
>gi|33865900|ref|NP_897459.1| hypothetical protein SYNW1366 [Synechococcus sp. WH 8102]
>gi|33865904|ref|NP_897463.1| putative glycosyltransferase family 2 [Synechococcus sp. WH 8102]
>gi|33865970|ref|NP_897529.1| hypothetical protein SYNW1436 [Synechococcus sp. WH 8102]
>gi|33865977|ref|NP_897536.1| hypothetical protein SYNW1443 [Synechococcus sp. WH 8102]
>gi|33865978|ref|NP_897537.1| hypothetical protein SYNW1444 [Synechococcus sp. WH 8102]
>gi|33865983|ref|NP_897542.1| possible high light inducible protein [Synechococcus sp. WH 8102]
>gi|33865991|ref|NP_897550.1| hypothetical protein SYNW1457 [Synechococcus sp. WH 8102]
>gi|33866008|ref|NP_897567.1| hypothetical protein SYNW1474 [Synechococcus sp. WH 8102]
>gi|33866021|ref|NP_897580.1| ROK family sugar kinase [Synechococcus sp. WH 8102]
>gi|33866031|ref|NP_897590.1| Type I copper blue protein: plastocyanin [Synechococcus sp. WH 8102]
>gi|33866044|ref|NP_897603.1| hypothetical protein SYNW1510 [Synechococcus sp. WH 8102]
>gi|33866047|ref|NP_897606.1| hypothetical protein SYNW1513 [Synechococcus sp. WH 8102]
>gi|33866083|ref|NP_897642.1| hypothetical protein SYNW1549 [Synechococcus sp. WH 8102]
>gi|33866090|ref|NP_897649.1| hypothetical protein SYNW1556 [Synechococcus sp. WH 8102]
>gi|33866098|ref|NP_897657.1| hypothetical protein SYNW1564 [Synechococcus sp. WH 8102]
>gi|33866099|ref|NP_897658.1| hypothetical protein SYNW1565 [Synechococcus sp. WH 8102]
>gi|33866105|ref|NP_897664.1| hypothetical protein SYNW1571 [Synechococcus sp. WH 8102]
>gi|33866128|ref|NP_897687.1| hypothetical protein SYNW1594 [Synechococcus sp. WH 8102]
>gi|33866129|ref|NP_897688.1| hypothetical protein SYNW1595 [Synechococcus sp. WH 8102]
>gi|33866132|ref|NP_897691.1| possible two-component response regulator or pseudogene [Synechococcus sp. WH 8102]
>gi|33866136|ref|NP_897695.1| hypothetical protein SYNW1602 [Synechococcus sp. WH 8102]
>gi|33866140|ref|NP_897699.1| hypothetical protein SYNW1606 [Synechococcus sp. WH 8102]
>gi|33866149|ref|NP_897708.1| hypothetical protein SYNW1615 [Synechococcus sp. WH 8102]
>gi|33866151|ref|NP_897710.1| 30S Ribosomal protein S16 [Synechococcus sp. WH 8102]
>gi|33866153|ref|NP_897712.1| hypothetical protein SYNW1619 [Synechococcus sp. WH 8102]
>gi|33866157|ref|NP_897716.1| possible apolipoprotein n-acyltransferase [Synechococcus sp. WH 8102]
>gi|33866159|ref|NP_897718.1| FKBP-type peptidyl-prolyl cis-trans isomerase (PPIase) [Synechococcus sp. WH 8102]
>gi|33866162|ref|NP_897721.1| cyanobacterial conserved hypothetical protein [Synechococcus sp. WH 8102]
>gi|33866170|ref|NP_897729.1| hypothetical protein SYNW1636 [Synechococcus sp. WH 8102]
>gi|33866181|ref|NP_897740.1| hypothetical protein SYNW1647 [Synechococcus sp. WH 8102]
>gi|33866214|ref|NP_897773.1| hypothetical protein SYNW1682 [Synechococcus sp. WH 8102]
>gi|33866215|ref|NP_897774.1| putative ABC transporter, oligopeptides [Synechococcus sp. WH 8102]
>gi|33866229|ref|NP_897788.1| hypothetical protein SYNW1697 [Synechococcus sp. WH 8102]
>gi|33866232|ref|NP_897791.1| hypothetical protein SYNW1700 [Synechococcus sp. WH 8102]
>gi|33866233|ref|NP_897792.1| possible mechanosensitive ion channel, MscS family [Synechococcus sp. WH 8102]
>gi|33866243|ref|NP_897802.1| NADH dehydrogenase I chain 5 (or L) [Synechococcus sp. WH 8102]
>gi|33866244|ref|NP_897803.1| possible carbon dioxide concentrating mechanism protein CcmK [Synechococcus sp. WH 8102]
>gi|33866246|ref|NP_897805.1| putative carboxysome peptide A [Synechococcus sp. WH 8102]
>gi|33866262|ref|NP_897821.1| hypothetical protein SYNW1730 [Synechococcus sp. WH 8102]
>gi|33866264|ref|NP_897823.1| hypothetical protein SYNW1732 [Synechococcus sp. WH 8102]
>gi|33866309|ref|NP_897868.1| hypothetical protein SYNW1777 [Synechococcus sp. WH 8102]
>gi|33866313|ref|NP_897872.1| hypothetical protein SYNW1781 [Synechococcus sp. WH 8102]
>gi|33866315|ref|NP_897874.1| Putative principal RNA polymerase sigma factor [Synechococcus sp. WH 8102]
>gi|33866327|ref|NP_897886.1| hypothetical protein SYNW1795 [Synechococcus sp. WH 8102]
>gi|33866328|ref|NP_897887.1| hypothetical protein SYNW1796 [Synechococcus sp. WH 8102]
>gi|33866330|ref|NP_897889.1| putative iron ABC transporter [Synechococcus sp. WH 8102]
>gi|33866333|ref|NP_897892.1| hypothetical protein SYNW1801 [Synechococcus sp. WH 8102]
>gi|33866337|ref|NP_897896.1| hypothetical protein SYNW1805 [Synechococcus sp. WH 8102]
>gi|33866349|ref|NP_897908.1| hypothetical protein SYNW1817 [Synechococcus sp. WH 8102]
>gi|33866359|ref|NP_897918.1| putative Na+/H+ antiporter, CPA1 family [Synechococcus sp. WH 8102]
>gi|33866363|ref|NP_897922.1| homologous to N-terminus of PilT2 protein [Synechococcus sp. WH 8102]
>gi|33866384|ref|NP_897943.1| 3-oxoacyl-[acyl-carrier protein] reductase [Synechococcus sp. WH 8102]
>gi|33866389|ref|NP_897948.1| putative multidrug efflux ABC transporter [Synechococcus sp. WH 8102]
>gi|33866392|ref|NP_897951.1| hypothetical protein SYNW1860 [Synechococcus sp. WH 8102]
>gi|33866402|ref|NP_897961.1| hypothetical protein SYNW1870 [Synechococcus sp. WH 8102]
>gi|33866403|ref|NP_897962.1| hypothetical protein SYNW1871 [Synechococcus sp. WH 8102]
>gi|33866413|ref|NP_897972.1| hypothetical protein SYNW1881 [Synechococcus sp. WH 8102]
>gi|33866415|ref|NP_897974.1| putative SMR family transporter, possible pecM homologue [Synechococcus sp. WH 8102]
>gi|33866419|ref|NP_897978.1| 50S ribosomal protein L34 [Synechococcus sp. WH 8102]
>gi|33866420|ref|NP_897979.1| bacterial ribonuclease P protein component of ribozyme [Synechococcus sp. WH 8102]
>gi|33866425|ref|NP_897984.1| hypothetical protein SYNW1893 [Synechococcus sp. WH 8102]
>gi|33866433|ref|NP_897992.1| hypothetical protein SYNW1901 [Synechococcus sp. WH 8102]
>gi|33866448|ref|NP_898007.1| ABC transporter, glycine betaine/proline family, membrane component [Synechococcus sp. WH 8102]
>gi|33866456|ref|NP_898015.1| conserved hypothetical membrane protein [Synechococcus sp. WH 8102]
>gi|33866466|ref|NP_898025.1| DnaJ3 protein [Synechococcus sp. WH 8102]

>gi|33866506|ref|NP_898065.1| hypothetical protein SYNW1974 [Synechococcus sp. WH 8102]
>gi|33866560|ref|NP_898119.1| possible rare lipoprotein A [Synechococcus sp. WH 8102]
>gi|33866581|ref|NP_898140.1| putative DNA repair and genetic recombination protein RecF [Synechococcus sp. WH 8102]
>gi|33866588|ref|NP_898147.1| hypothetical protein SYNW2056 [Synechococcus sp. WH 8102]
>gi|33866615|ref|NP_898174.1| 30S ribosomal protein S5 [Synechococcus sp. WH 8102]
>gi|33866621|ref|NP_898180.1| 30S ribosomal protein S11 [Synechococcus sp. WH 8102]
>gi|33866657|ref|NP_898216.1| hypothetical protein SYNW2125 [Synechococcus sp. WH 8102]
>gi|33866659|ref|NP_898218.1| putative hydrogenase accessory protein [Synechococcus sp. WH 8102]
>gi|33866681|ref|NP_898240.1| hypothetical protein SYNW2149 [Synechococcus sp. WH 8102]
>gi|33866713|ref|NP_898272.1| possible exodeoxyribonuclease VII large subunit [Synechococcus sp. WH 8102]
>gi|33866714|ref|NP_898273.1| hypothetical protein SYNW2182 [Synechococcus sp. WH 8102]
>gi|33866716|ref|NP_898275.1| hypothetical protein SYNW2184 [Synechococcus sp. WH 8102]
>gi|33866717|ref|NP_898276.1| similar to serum resistance locus BrkB [Synechococcus sp. WH 8102]
>gi|33866722|ref|NP_898281.1| hypothetical protein SYNW2190 [Synechococcus sp. WH 8102]
>gi|33866725|ref|NP_898284.1| hypothetical protein SYNW2193 [Synechococcus sp. WH 8102]
>gi|33866727|ref|NP_898286.1| putative multidrug efflux MFS transporter [Synechococcus sp. WH 8102]
>gi|33866735|ref|NP_898294.1| hypothetical protein SYNW2203 [Synechococcus sp. WH 8102]
>gi|33866752|ref|NP_898311.1| hypothetical protein SYNW2220 [Synechococcus sp. WH 8102]
>gi|33866758|ref|NP_898317.1| hypothetical protein SYNW2228 [Synechococcus sp. WH 8102]
>gi|33866759|ref|NP_898318.1| hypothetical protein SYNW2229 [Synechococcus sp. WH 8102]
>gi|33866760|ref|NP_898319.1| conserved hypothetical membrane protein [Synechococcus sp. WH 8102]
>gi|33866764|ref|NP_898323.1| possible ABC transporter, ATP-binding component [Synechococcus sp. WH 8102]
>gi|33866768|ref|NP_898327.1| Thymidylate kinase [Synechococcus sp. WH 8102]
>gi|33866772|ref|NP_898331.1| hypothetical protein SYNW2242 [Synechococcus sp. WH 8102]
>gi|33866782|ref|NP_898341.1| hypothetical protein SYNW2252 [Synechococcus sp. WH 8102]
>gi|33866785|ref|NP_898344.1| putative RNA-binding protein (RRM domain) [Synechococcus sp. WH 8102]
>gi|33866814|ref|NP_898373.1| hypothetical protein SYNW2284 [Synechococcus sp. WH 8102]
>gi|33866815|ref|NP_898374.1| Phosphoribosylaminoimidazole carboxylase [Synechococcus sp. WH 8102]
>gi|33866822|ref|NP_898381.1| hypothetical protein SYNW2292 [Synechococcus sp. WH 8102]
>gi|33866830|ref|NP_898389.1| hypothetical protein SYNW2300 [Synechococcus sp. WH 8102]
>gi|33866833|ref|NP_898392.1| hypothetical protein SYNW2303 [Synechococcus sp. WH 8102]
>gi|33866836|ref|NP_898395.1| putative O-succinylbenzoate synthase [Synechococcus sp. WH 8102]
>gi|33866837|ref|NP_898396.1| 1,4-dihydroxy-2-naphthoate (DHNA) octaprenyltransferase; UbiA prenyltranferase family [Synechococcus sp. WH 8102]
>gi|33866844|ref|NP_898403.1| possible diacylglycerol kinase [Synechococcus sp. WH 8102]
>gi|33866853|ref|NP_898412.1| hypothetical protein SYNW2323 [Synechococcus sp. WH 8102]
>gi|33866858|ref|NP_898417.1| hypothetical protein SYNW2328 [Synechococcus sp. WH 8102]
>gi|33866865|ref|NP_898424.1| hypothetical protein SYNW2335 [Synechococcus sp. WH 8102]
>gi|33866868|ref|NP_898427.1| possible ribonuclease HI [Synechococcus sp. WH 8102]
>gi|33866870|ref|NP_898429.1| 50S ribosomal protein L7/L12 [Synechococcus sp. WH 8102]
>gi|33866876|ref|NP_898435.1| putative ATP-dependent Clp protease, Hsp 100, ATP-binding subunit ClpB [Synechococcus sp. WH 8102]
>gi|33866881|ref|NP_898440.1| hypothetical protein SYNW2351 [Synechococcus sp. WH 8102]
>gi|33866890|ref|NP_898449.1| Alanyl-tRNA synthetase [Synechococcus sp. WH 8102]
>gi|33866891|ref|NP_898450.1| hypothetical protein SYNW2361 [Synechococcus sp. WH 8102]
>gi|33866896|ref|NP_898455.1| conserved hypothetical putative integral membrane protein [Synechococcus sp. WH 8102]
>gi|33866913|ref|NP_898472.1| hypothetical protein SYNW2383 [Synechococcus sp. WH 8102]
>gi|33866923|ref|NP_898482.1| conserved membrane protein, multidrug efflux associated [Synechococcus sp. WH 8102]
>gi|33866928|ref|NP_898487.1| possible Protein phosphatase 2A regulatory B s [Synechococcus sp. WH 8102]
>gi|33866939|ref|NP_898498.1| putative hemolysin-type calcium-binding protein; similar to HlyA [Synechococcus sp. WH 8102]
>gi|33866947|ref|NP_898506.1| hypothetical protein SYNW2417 [Synechococcus sp. WH 8102]
>gi|33866980|ref|NP_898539.1| hypothetical protein SYNW2450 [Synechococcus sp. WH 8102]
>gi|33867009|ref|NP_898568.1| ABC transporter component, possibly Zn transport. [Synechococcus sp. WH 8102]
>gi|33867024|ref|NP_898583.1| multidrug efflux transporter, MFS family [Synechococcus sp. WH 8102]
>gi|33867027|ref|NP_898586.1| hypothetical protein SYNW2497 [Synechococcus sp. WH 8102]
>gi|33867031|ref|NP_898590.1| putative chloride channel [Synechococcus sp. WH 8102]
>gi|33867033|ref|NP_898592.1| hypothetical protein SYNW2503 [Synechococcus sp. WH 8102]
>gi|33867035|ref|NP_898594.1| hypothetical protein SYNW2505 [Synechococcus sp. WH 8102]
>gi|33867040|ref|NP_898599.1| hypothetical protein SYNW2510 [Synechococcus sp. WH 8102]
>gi|33867044|ref|NP_898603.1| hypothetical protein SYNW2514 [Synechococcus sp. WH 8102]
>gi|33867053|ref|NP_898612.1| putative DNA repair protein [Synechococcus sp. WH 8102]
>gi|15835546|ref|NP_300070.1| hypothetical protein CPj0011 [Chlamydophila pneumoniae J138]
>gi|15835563|ref|NP_300087.1| hypothetical protein CPj0026 [Chlamydophila pneumoniae J138]
>gi|15835565|ref|NP_300089.1| hypothetical protein CPj0028 [Chlamydophila pneumoniae J138]
>gi|15835568|ref|NP_300092.1| S21 ribosomal protein [Chlamydophila pneumoniae J138]
>gi|15835585|ref|NP_300109.1| hypothetical protein CPj0049 [Chlamydophila pneumoniae J138]
>gi|15835586|ref|NP_300110.1| hypothetical protein CPj0050 [Chlamydophila pneumoniae J138]
>gi|15835600|ref|NP_300124.1| hypothetical protein CPj0064 [Chlamydophila pneumoniae J138]
>gi|15835682|ref|NP_300206.1| hypothetical protein CPj0147 [Chlamydophila pneumoniae J138]
>gi|15835701|ref|NP_300225.1| hypothetical protein CPj0166 [Chlamydophila pneumoniae J138]
>gi|15835708|ref|NP_300232.1| hypothetical protein CPj0173 [Chlamydophila pneumoniae J138]

>gi|15835709|ref|NP_300233.1| hypothetical protein CPj0174 [Chlamydophila pneumoniae J138]
>gi|15835746|ref|NP_300270.1| hypothetical protein CPj0211 [Chlamydophila pneumoniae J138]
>gi|15835751|ref|NP_300275.1| hypothetical protein CPj0216 [Chlamydophila pneumoniae J138]
>gi|15835756|ref|NP_300280.1| hypothetical protein CPj0221 [Chlamydophila pneumoniae J138]
>gi|15835760|ref|NP_300284.1| hypothetical protein CPj0225 [Chlamydophila pneumoniae J138]
>gi|15835775|ref|NP_300299.1| hypothetical protein CPj0240 [Chlamydophila pneumoniae J138]
>gi|15835801|ref|NP_300325.1| hypothetical protein CPj0266 [Chlamydophila pneumoniae J138]
>gi|15835803|ref|NP_300327.1| hypothetical protein CPj0268 [Chlamydophila pneumoniae J138]
>gi|15835812|ref|NP_300336.1| hypothetical protein CPj0277 [Chlamydophila pneumoniae J138]
>gi|15835826|ref|NP_300350.1| inclusion membrane protein B [Chlamydophila pneumoniae J138]
>gi|15835837|ref|NP_300361.1| UDP glucosamine N-acyltransferase [Chlamydophila pneumoniae J138]
>gi|15835843|ref|NP_300367.1| hypothetical protein CPj0308 [Chlamydophila pneumoniae J138]
>gi|15835879|ref|NP_300403.1| integral membrane protein [Chlamydophila pneumoniae J138]
>gi|15835886|ref|NP_300410.1| hypothetical protein CPj0353 [Chlamydophila pneumoniae J138]
>gi|15835900|ref|NP_300424.1| hypothetical protein CPj0367 [Chlamydophila pneumoniae J138]
>gi|15835904|ref|NP_300428.1| hypothetical protein CPj0371 [Chlamydophila pneumoniae J138]
>gi|15835917|ref|NP_300441.1| histone-like protein 2 [Chlamydophila pneumoniae J138]
>gi|15835926|ref|NP_300450.1| hypothetical protein CPj0393 [Chlamydophila pneumoniae J138]
>gi|15835938|ref|NP_300462.1| hypothetical protein CPj0405 [Chlamydophila pneumoniae J138]
>gi|15835975|ref|NP_300499.1| hypothetical protein CPj0442 [Chlamydophila pneumoniae J138]
>gi|15836003|ref|NP_300527.1| hypothetical protein CPj0472 [Chlamydophila pneumoniae J138]
>gi|15836016|ref|NP_300540.1| hypothetical protein CPj0485 [Chlamydophila pneumoniae J138]
>gi|15836054|ref|NP_300578.1| hypothetical protein CPj0523 [Chlamydophila pneumoniae J138]
>gi|15836103|ref|NP_300627.1| hypothetical protein CPj0572 [Chlamydophila pneumoniae J138]
>gi|15836133|ref|NP_300657.1| hypothetical protein CPj0601 [Chlamydophila pneumoniae J138]
>gi|15836162|ref|NP_300686.1| L15 ribosomal protein [Chlamydophila pneumoniae J138]
>gi|15836210|ref|NP_300734.1| hypothetical protein CPj0678 [Chlamydophila pneumoniae J138]
>gi|15836238|ref|NP_300762.1| hypothetical protein CPj0706 [Chlamydophila pneumoniae J138]
>gi|15836252|ref|NP_300776.1| hypothetical protein CPj0720 [Chlamydophila pneumoniae J138]
>gi|15836260|ref|NP_300784.1| CHLPN 76 kDa homolog_1 (CT622) [Chlamydophila pneumoniae J138]
>gi|15836341|ref|NP_300865.1| hypothetical protein CPj0808 [Chlamydophila pneumoniae J138]
>gi|15836342|ref|NP_300866.1| hypothetical protein CPj0809 [Chlamydophila pneumoniae J138]
>gi|15836414|ref|NP_300938.1| hypothetical protein CPj0881 [Chlamydophila pneumoniae J138]
>gi|15836419|ref|NP_300943.1| histone-like developmental protein [Chlamydophila pneumoniae J138]
>gi|15836468|ref|NP_300992.1| L34 ribosomal protein [Chlamydophila pneumoniae J138]
>gi|15836535|ref|NP_301059.1| hypothetical protein CPj1004 [Chlamydophila pneumoniae J138]
>gi|15836536|ref|NP_301060.1| hypothetical protein CPj1005 [Chlamydophila pneumoniae J138]
>gi|15836537|ref|NP_301061.1| hypothetical protein CPj1006 [Chlamydophila pneumoniae J138]
>gi|15836545|ref|NP_301069.1| sulfate transporter [Chlamydophila pneumoniae J138]
>gi|15836554|ref|NP_301078.1| hypothetical protein CPj1023 [Chlamydophila pneumoniae J138]
>gi|15836614|ref|NP_297302.1| TonB protein [Xylella fastidiosa 9a5c]
>gi|15836621|ref|NP_297309.1| hypothetical protein XF0016 [Xylella fastidiosa 9a5c]
>gi|15836623|ref|NP_297311.1| hypothetical protein XF0018 [Xylella fastidiosa 9a5c]
>gi|15836656|ref|NP_297344.1| hypothetical protein XF0051 [Xylella fastidiosa 9a5c]
>gi|15836664|ref|NP_297352.1| hypothetical protein XF0059 [Xylella fastidiosa 9a5c]
>gi|15836705|ref|NP_297393.1| hypothetical protein XF0100 [Xylella fastidiosa 9a5c]
>gi|15836738|ref|NP_297426.1| copper resistance protein B precursor [Xylella fastidiosa 9a5c]
>gi|15836785|ref|NP_297473.1| hypothetical protein XF0180 [Xylella fastidiosa 9a5c]
>gi|15836789|ref|NP_297477.1| hypothetical protein XF0184 [Xylella fastidiosa 9a5c]
>gi|15836833|ref|NP_297521.1| 2-amino-4-hydroxy-6-hydroxymethyldihydropteridine pyrophosphokinase [Xylella fastidiosa 9a5c]
>gi|15836842|ref|NP_297530.1| tRNA pseudouridine synthase B [Xylella fastidiosa 9a5c]
>gi|15836846|ref|NP_297534.1| hypothetical protein XF0241 [Xylella fastidiosa 9a5c]
>gi|15836867|ref|NP_297555.1| colicin V precursor [Xylella fastidiosa 9a5c]
>gi|15836868|ref|NP_297556.1| colicin V precursor [Xylella fastidiosa 9a5c]
>gi|15836869|ref|NP_297557.1| hypothetical protein XF0264 [Xylella fastidiosa 9a5c]
>gi|15836871|ref|NP_297559.1| hypothetical protein XF0266 [Xylella fastidiosa 9a5c]
>gi|15836872|ref|NP_297560.1| serine protease [Xylella fastidiosa 9a5c]
>gi|15836886|ref|NP_297574.1| transport protein [Xylella fastidiosa 9a5c]
>gi|15836888|ref|NP_297576.1| hypothetical protein XF0283 [Xylella fastidiosa 9a5c]
>gi|15836889|ref|NP_297577.1| hypothetical protein XF0284 [Xylella fastidiosa 9a5c]
>gi|15836896|ref|NP_297584.1| hypothetical protein XF0291 [Xylella fastidiosa 9a5c]
>gi|15836907|ref|NP_297595.1| protein-export membrane protein [Xylella fastidiosa 9a5c]
>gi|15836917|ref|NP_297605.1| NADH-ubiquinone oxidoreductase, NQO10 subunit [Xylella fastidiosa 9a5c]
>gi|15836932|ref|NP_297620.1| hypothetical protein XF0330 [Xylella fastidiosa 9a5c]
>gi|15836947|ref|NP_297635.1| hypothetical protein XF0345 [Xylella fastidiosa 9a5c]
>gi|15836965|ref|NP_297653.1| hypothetical protein XF0363 [Xylella fastidiosa 9a5c]
>gi|15836993|ref|NP_297681.1| hypothetical protein XF0391 [Xylella fastidiosa 9a5c]
>gi|15836998|ref|NP_297686.1| hypothetical protein XF0396 [Xylella fastidiosa 9a5c]
>gi|15837000|ref|NP_297688.1| hypothetical protein XF0398 [Xylella fastidiosa 9a5c]
>gi|15837023|ref|NP_297711.1| toluene tolerance protein [Xylella fastidiosa 9a5c]

>gi|15837027|ref|NP_297715.1| exodeoxyribonuclease V alpha chain [Xylella fastidiosa 9a5c]
>gi|15837036|ref|NP_297724.1| 30S ribosomal protein S21 [Xylella fastidiosa 9a5c]
>gi|15837056|ref|NP_297744.1| hypothetical protein XF0454 [Xylella fastidiosa 9a5c]
>gi|15837088|ref|NP_297776.1| UDP-3-O-[3-hydroxymyristoyl] glucosamine N-acyltransferase [Xylella fastidiosa 9a5c]
>gi|15837091|ref|NP_297779.1| hypothetical protein XF0489 [Xylella fastidiosa 9a5c]
>gi|15837101|ref|NP_297789.1| hypothetical protein XF0499 [Xylella fastidiosa 9a5c]
>gi|15837106|ref|NP_297794.1| hypothetical protein XF0504 [Xylella fastidiosa 9a5c]
>gi|15837121|ref|NP_297809.1| hypothetical protein XF0519 [Xylella fastidiosa 9a5c]
>gi|15837122|ref|NP_297810.1| hypothetical protein XF0520 [Xylella fastidiosa 9a5c]
>gi|15837126|ref|NP_297814.1| hypothetical protein XF0524 [Xylella fastidiosa 9a5c]
>gi|15837163|ref|NP_297851.1| hypothetical protein XF0561 [Xylella fastidiosa 9a5c]
>gi|15837181|ref|NP_297869.1| hypothetical protein XF0579 [Xylella fastidiosa 9a5c]
>gi|15837191|ref|NP_297879.1| permease [Xylella fastidiosa 9a5c]
>gi|15837196|ref|NP_297884.1| thiamine biosynthesis lipoprotein ApbE precursor [Xylella fastidiosa 9a5c]
>gi|15837202|ref|NP_297890.1| hypothetical protein XF0600 [Xylella fastidiosa 9a5c]
>gi|15837203|ref|NP_297891.1| hypothetical protein XF0601 [Xylella fastidiosa 9a5c]
>gi|15837228|ref|NP_297916.1| hypothetical protein XF0626 [Xylella fastidiosa 9a5c]
>gi|15837241|ref|NP_297929.1| hypothetical protein XF0639 [Xylella fastidiosa 9a5c]
>gi|15837248|ref|NP_297936.1| hypothetical protein XF0646 [Xylella fastidiosa 9a5c]
>gi|15837256|ref|NP_297944.1| hypothetical protein XF0654 [Xylella fastidiosa 9a5c]
>gi|15837260|ref|NP_297948.1| hypothetical protein XF0658 [Xylella fastidiosa 9a5c]
>gi|15837281|ref|NP_297969.1| hypothetical protein XF0679 [Xylella fastidiosa 9a5c]
>gi|15837283|ref|NP_297971.1| hypothetical protein XF0681 [Xylella fastidiosa 9a5c]
>gi|15837295|ref|NP_297983.1| hypothetical protein XF0693 [Xylella fastidiosa 9a5c]
>gi|15837311|ref|NP_297999.1| hypothetical protein XF0709 [Xylella fastidiosa 9a5c]
>gi|15837312|ref|NP_298000.1| hypothetical protein XF0710 [Xylella fastidiosa 9a5c]
>gi|15837321|ref|NP_298009.1| phage-related baseplate assembly protein [Xylella fastidiosa 9a5c]
>gi|15837334|ref|NP_298022.1| phage-related tail protein [Xylella fastidiosa 9a5c]
>gi|15837359|ref|NP_298047.1| hypothetical protein XF0757 [Xylella fastidiosa 9a5c]
>gi|15837374|ref|NP_298062.1| hypothetical protein XF0772 [Xylella fastidiosa 9a5c]
>gi|15837378|ref|NP_298066.1| hypothetical protein XF0776 [Xylella fastidiosa 9a5c]
>gi|15837404|ref|NP_298092.1| cell division protein [Xylella fastidiosa 9a5c]
>gi|15837415|ref|NP_298103.1| hypothetical protein XF0813 [Xylella fastidiosa 9a5c]
>gi|15837420|ref|NP_298108.1| endo-1,4-beta-glucanase [Xylella fastidiosa 9a5c]
>gi|15837429|ref|NP_298117.1| hypothetical protein XF0827 [Xylella fastidiosa 9a5c]
>gi|15837446|ref|NP_298134.1| hypothetical protein XF0844 [Xylella fastidiosa 9a5c]
>gi|15837456|ref|NP_298144.1| hypothetical protein XF0854 [Xylella fastidiosa 9a5c]
>gi|15837458|ref|NP_298146.1| hypothetical protein XF0856 [Xylella fastidiosa 9a5c]
>gi|15837485|ref|NP_298173.1| hypothetical protein XF0883 [Xylella fastidiosa 9a5c]
>gi|15837490|ref|NP_298178.1| hypothetical protein XF0888 [Xylella fastidiosa 9a5c]
>gi|15837491|ref|NP_298179.1| hemagglutinin-like secreted protein [Xylella fastidiosa 9a5c]
>gi|15837495|ref|NP_298183.1| hypothetical protein XF0893 [Xylella fastidiosa 9a5c]
>gi|15837509|ref|NP_298197.1| soluble lytic murein transglycosylase precursor [Xylella fastidiosa 9a5c]
>gi|15837515|ref|NP_298203.1| tropinone reductase [Xylella fastidiosa 9a5c]
>gi|15837520|ref|NP_298208.1| acyl-[ACP]-UDP-N-acetylglucosamine [Xylella fastidiosa 9a5c]
>gi|15837531|ref|NP_298219.1| hypothetical protein XF0929 [Xylella fastidiosa 9a5c]
>gi|15837533|ref|NP_298221.1| hypothetical protein XF0931 [Xylella fastidiosa 9a5c]
>gi|15837549|ref|NP_298237.1| hypothetical protein XF0947 [Xylella fastidiosa 9a5c]
>gi|15837576|ref|NP_298264.1| hypothetical protein XF0974 [Xylella fastidiosa 9a5c]
>gi|15837617|ref|NP_298305.1| manganese transport protein [Xylella fastidiosa 9a5c]
>gi|15837626|ref|NP_298314.1| outer membrane protein H.8 precursor [Xylella fastidiosa 9a5c]
>gi|15837627|ref|NP_298315.1| hypothetical protein XF1025 [Xylella fastidiosa 9a5c]
>gi|15837628|ref|NP_298316.1| serine protease [Xylella fastidiosa 9a5c]
>gi|15837642|ref|NP_298330.1| hypothetical protein XF1040 [Xylella fastidiosa 9a5c]
>gi|15837650|ref|NP_298338.1| 1-deoxy-D-xylulose 5-phosphate reductoisomerase [Xylella fastidiosa 9a5c]
>gi|15837651|ref|NP_298339.1| phosphatidate cytidylyltransferase [Xylella fastidiosa 9a5c]
>gi|15837656|ref|NP_298344.1| hypothetical protein XF1054 [Xylella fastidiosa 9a5c]
>gi|15837673|ref|NP_298361.1| succinate dehydrogenase, membrane anchor subunit [Xylella fastidiosa 9a5c]
>gi|15837693|ref|NP_298381.1| hypothetical protein XF1091 [Xylella fastidiosa 9a5c]
>gi|15837701|ref|NP_298389.1| hypothetical protein XF1099 [Xylella fastidiosa 9a5c]
>gi|15837752|ref|NP_298440.1| hypothetical protein XF1150 [Xylella fastidiosa 9a5c]
>gi|15837775|ref|NP_298463.1| 30S ribosomal protein S13 [Xylella fastidiosa 9a5c]
>gi|15837780|ref|NP_298468.1| hypothetical protein XF1178 [Xylella fastidiosa 9a5c]
>gi|15837804|ref|NP_298492.1| hypothetical protein XF1202 [Xylella fastidiosa 9a5c]
>gi|15837820|ref|NP_298508.1| hypothetical protein XF1218 [Xylella fastidiosa 9a5c]
>gi|15837868|ref|NP_298556.1| 1,4-beta-cellobiosidase [Xylella fastidiosa 9a5c]
>gi|15837897|ref|NP_298585.1| hypothetical protein XF1296 [Xylella fastidiosa 9a5c]
>gi|15837905|ref|NP_298593.1| hypothetical protein XF1304 [Xylella fastidiosa 9a5c]
>gi|15837912|ref|NP_298600.1| rod shape-determining protein [Xylella fastidiosa 9a5c]
>gi|15837940|ref|NP_298628.1| hypothetical protein XF1339 [Xylella fastidiosa 9a5c]

>gi|15837960|ref|NP_298648.1| hypothetical protein XF1359 [Xylella fastidiosa 9a5c]
>gi|15837968|ref|NP_298656.1| hypothetical protein XF1367 [Xylella fastidiosa 9a5c]
>gi|15837993|ref|NP_298681.1| single-stranded DNA binding protein [Xylella fastidiosa 9a5c]
>gi|15838019|ref|NP_298707.1| hypothetical protein XF1418 [Xylella fastidiosa 9a5c]
>gi|15838020|ref|NP_298708.1| acetyltransferase [Xylella fastidiosa 9a5c]
>gi|15838055|ref|NP_298743.1| hypothetical protein XF1454 [Xylella fastidiosa 9a5c]
>gi|15838117|ref|NP_298805.1| surface-exposed outer membrane protein [Xylella fastidiosa 9a5c]
>gi|15838134|ref|NP_298822.1| hypothetical protein XF1533 [Xylella fastidiosa 9a5c]
>gi|15838144|ref|NP_298832.1| hypothetical protein XF1543 [Xylella fastidiosa 9a5c]
>gi|15838148|ref|NP_298836.1| peptidoglycan-associated outer membrane lipoprotein precursor [Xylella fastidiosa 9a5c]
>gi|15838188|ref|NP_298876.1| hypothetical protein XF1587 [Xylella fastidiosa 9a5c]
>gi|15838247|ref|NP_298935.1| UDP-3-O-(R-3-hydroxymyristoyl)-glucosamine N-acyltransferase [Xylella fastidiosa 9a5c]
>gi|15838256|ref|NP_298944.1| hypothetical protein XF1655 [Xylella fastidiosa 9a5c]
>gi|15838293|ref|NP_298981.1| hypothetical protein XF1692 [Xylella fastidiosa 9a5c]
>gi|15838294|ref|NP_298982.1| hypothetical protein XF1693 [Xylella fastidiosa 9a5c]
>gi|15838295|ref|NP_298983.1| hypothetical protein XF1694 [Xylella fastidiosa 9a5c]
>gi|15838311|ref|NP_298999.1| hypothetical protein XF1710 [Xylella fastidiosa 9a5c]
>gi|15838366|ref|NP_299054.1| drug:proton antiporter [Xylella fastidiosa 9a5c]
>gi|15838368|ref|NP_299056.1| hypothetical protein XF1767 [Xylella fastidiosa 9a5c]
>gi|15838381|ref|NP_299069.1| hypothetical protein XF1781 [Xylella fastidiosa 9a5c]
>gi|15838383|ref|NP_299071.1| hypothetical protein XF1783 [Xylella fastidiosa 9a5c]
>gi|15838434|ref|NP_299122.1| hypothetical protein XF1836 [Xylella fastidiosa 9a5c]
>gi|15838449|ref|NP_299137.1| serine protease [Xylella fastidiosa 9a5c]
>gi|15838468|ref|NP_299156.1| hypothetical protein XF1870 [Xylella fastidiosa 9a5c]
>gi|15838473|ref|NP_299161.1| hypothetical protein XF1875 [Xylella fastidiosa 9a5c]
>gi|15838476|ref|NP_299164.1| hypothetical protein XF1878 [Xylella fastidiosa 9a5c]
>gi|15838481|ref|NP_299169.1| hypothetical protein XF1883 [Xylella fastidiosa 9a5c]
>gi|15838513|ref|NP_299201.1| anthranilate synthase component II [Xylella fastidiosa 9a5c]
>gi|15838575|ref|NP_299263.1| surface protein [Xylella fastidiosa 9a5c]
>gi|15838580|ref|NP_299268.1| hypothetical protein XF1986 [Xylella fastidiosa 9a5c]
>gi|15838589|ref|NP_299277.1| hypothetical protein XF1995 [Xylella fastidiosa 9a5c]
>gi|15838591|ref|NP_299279.1| hypothetical protein XF1997 [Xylella fastidiosa 9a5c]
>gi|15838595|ref|NP_299283.1| hypothetical protein XF2001 [Xylella fastidiosa 9a5c]
>gi|15838640|ref|NP_299328.1| conjugal transfer protein [Xylella fastidiosa 9a5c]
>gi|15838654|ref|NP_299342.1| transcriptional repressor [Xylella fastidiosa 9a5c]
>gi|15838694|ref|NP_299382.1| hypothetical protein XF2103 [Xylella fastidiosa 9a5c]
>gi|15838703|ref|NP_299391.1| hypothetical protein XF2112 [Xylella fastidiosa 9a5c]
>gi|15838714|ref|NP_299402.1| hypothetical protein XF2123 [Xylella fastidiosa 9a5c]
>gi|15838766|ref|NP_299454.1| hypothetical protein XF2175 [Xylella fastidiosa 9a5c]
>gi|15838768|ref|NP_299456.1| hypothetical protein XF2177 [Xylella fastidiosa 9a5c]
>gi|15838773|ref|NP_299461.1| hypothetical protein XF2182 [Xylella fastidiosa 9a5c]
>gi|15838787|ref|NP_299475.1| hemagglutinin-like secreted protein [Xylella fastidiosa 9a5c]
>gi|15838788|ref|NP_299476.1| hypothetical protein XF2197 [Xylella fastidiosa 9a5c]
>gi|15838789|ref|NP_299477.1| hypothetical protein XF2198 [Xylella fastidiosa 9a5c]
>gi|15838792|ref|NP_299480.1| ribosomal protein L11 methyltransferase [Xylella fastidiosa 9a5c]
>gi|15838810|ref|NP_299498.1| histidinol dehydrogenase [Xylella fastidiosa 9a5c]
>gi|15838815|ref|NP_299503.1| homoserine kinase [Xylella fastidiosa 9a5c]
>gi|15838822|ref|NP_299510.1| hypothetical protein XF2231 [Xylella fastidiosa 9a5c]
>gi|15838829|ref|NP_299517.1| hypothetical protein XF2238 [Xylella fastidiosa 9a5c]
>gi|15838850|ref|NP_299538.1| polyvinylalcohol dehydrogenase [Xylella fastidiosa 9a5c]
>gi|15838878|ref|NP_299566.1| TonB protein [Xylella fastidiosa 9a5c]
>gi|15838884|ref|NP_299572.1| hypothetical protein XF2293 [Xylella fastidiosa 9a5c]
>gi|15838887|ref|NP_299575.1| hypothetical protein XF2296 [Xylella fastidiosa 9a5c]
>gi|15838899|ref|NP_299587.1| hypothetical protein XF2308 [Xylella fastidiosa 9a5c]
>gi|15838912|ref|NP_299600.1| hypothetical protein XF2321 [Xylella fastidiosa 9a5c]
>gi|15838918|ref|NP_299606.1| TonB protein [Xylella fastidiosa 9a5c]
>gi|15838925|ref|NP_299613.1| diacylglycerol kinase [Xylella fastidiosa 9a5c]
>gi|15838990|ref|NP_299678.1| hypothetical protein XF2399 [Xylella fastidiosa 9a5c]
>gi|15838998|ref|NP_299686.1| bacteriocin [Xylella fastidiosa 9a5c]
>gi|15839025|ref|NP_299713.1| lipopolysaccharide core biosynthesis protein [Xylella fastidiosa 9a5c]
>gi|15839035|ref|NP_299723.1| pheromone shutdown protein [Xylella fastidiosa 9a5c]
>gi|15839047|ref|NP_299735.1| heme ABC transporter membrane protein [Xylella fastidiosa 9a5c]
>gi|15839049|ref|NP_299737.1| hypothetical protein XF2458 [Xylella fastidiosa 9a5c]
>gi|15839051|ref|NP_299739.1| c-type cytochrome biogenesis membrane protein [Xylella fastidiosa 9a5c]
>gi|15839054|ref|NP_299742.1| hypothetical protein XF2463 [Xylella fastidiosa 9a5c]
>gi|15839070|ref|NP_299758.1| phage-related tail protein [Xylella fastidiosa 9a5c]
>gi|15839082|ref|NP_299770.1| phage-related baseplate assembly protein [Xylella fastidiosa 9a5c]
>gi|15839091|ref|NP_299779.1| hypothetical protein XF2501 [Xylella fastidiosa 9a5c]
>gi|15839092|ref|NP_299780.1| hypothetical protein XF2502 [Xylella fastidiosa 9a5c]
>gi|15839104|ref|NP_299792.1| hypothetical protein XF2514 [Xylella fastidiosa 9a5c]

>gi|15839117|ref|NP_299805.1| hypothetical protein XF2527 [Xylella fastidiosa 9a5c]
>gi|15839138|ref|NP_299826.1| hypothetical protein XF2549 [Xylella fastidiosa 9a5c]
>gi|15839148|ref|NP_299836.1| 50S ribosomal protein L9 [Xylella fastidiosa 9a5c]
>gi|15839191|ref|NP_299879.1| hypothetical protein XF2602 [Xylella fastidiosa 9a5c]
>gi|15839216|ref|NP_299904.1| hypothetical protein XF2627 [Xylella fastidiosa 9a5c]
>gi|15839223|ref|NP_299911.1| 50S ribosomal protein L7/L12 [Xylella fastidiosa 9a5c]
>gi|15839248|ref|NP_299936.1| hypothetical protein XF2659 [Xylella fastidiosa 9a5c]
>gi|15839275|ref|NP_299963.1| multidrug efflux protein [Xylella fastidiosa 9a5c]
>gi|15839283|ref|NP_299971.1| cell division protein [Xylella fastidiosa 9a5c]
>gi|15839316|ref|NP_300004.1| hypothetical protein XF2727 [Xylella fastidiosa 9a5c]
>gi|15839351|ref|NP_300039.1| hypothetical protein XF2762 [Xylella fastidiosa 9a5c]
>gi|15839356|ref|NP_300044.1| hypothetical protein XF2767 [Xylella fastidiosa 9a5c]
>gi|15839364|ref|NP_300052.1| hemagglutinin-like secreted protein [Xylella fastidiosa 9a5c]
>gi|10956716|ref|NP_061661.1| conjugal transfer protein [Xylella fastidiosa 9a5c]
>gi|10956725|ref|NP_061670.1| conjugal transfer protein [Xylella fastidiosa 9a5c]
>gi|10956729|ref|NP_061674.1| hypothetical protein XFa0018 [Xylella fastidiosa 9a5c]
>gi|10956741|ref|NP_061686.1| hypothetical protein XFa0030 [Xylella fastidiosa 9a5c]
>gi|10956748|ref|NP_061693.1| conjugal transfer protein [Xylella fastidiosa 9a5c]
>gi|10956750|ref|NP_061695.1| conjugal transfer protein [Xylella fastidiosa 9a5c]
>gi|16124262|ref|NP_418826.1| enoyl-CoA hydratase/isomerase family protein [Caulobacter crescentus CB15]
>gi|16124268|ref|NP_418832.1| DNA mismatch repair protein MutS [Caulobacter crescentus CB15]
>gi|16124296|ref|NP_418860.1| hypothetical protein CC0040 [Caulobacter crescentus CB15]
>gi|16124298|ref|NP_418862.1| translation initiation factor IF-2 [Caulobacter crescentus CB15]
>gi|16124309|ref|NP_418873.1| CBS domain protein [Caulobacter crescentus CB15]
>gi|16124313|ref|NP_418877.1| ribosomal-protein-alanine acetyltransferase, putative [Caulobacter crescentus CB15]
>gi|16124314|ref|NP_418878.1| glycoprotease family protein [Caulobacter crescentus CB15]
>gi|16124320|ref|NP_418884.1| MviN family protein [Caulobacter crescentus CB15]
>gi|16124321|ref|NP_418885.1| methyl-accepting chemotaxis protein McpQ [Caulobacter crescentus CB15]
>gi|16124325|ref|NP_418889.1| glycerol-3-phosphate dehydrogenase (NAD+) [Caulobacter crescentus CB15]
>gi|16124326|ref|NP_418890.1| peptidase M22 family protein [Caulobacter crescentus CB15]
>gi|16124329|ref|NP_418893.1| hypothetical protein CC0074 [Caulobacter crescentus CB15]
>gi|16124330|ref|NP_418894.1| hypothetical protein CC0075 [Caulobacter crescentus CB15]
>gi|16124352|ref|NP_418916.1| asparaginase family protein [Caulobacter crescentus CB15]
>gi|16124358|ref|NP_418922.1| hypothetical protein CC0103 [Caulobacter crescentus CB15]
>gi|16124365|ref|NP_418929.1| thioredoxin [Caulobacter crescentus CB15]
>gi|16124395|ref|NP_418959.1| ComM protein [Caulobacter crescentus CB15]
>gi|16124399|ref|NP_418963.1| tetrapyrrole methylase family protein [Caulobacter crescentus CB15]
>gi|16124404|ref|NP_418968.1| hypothetical protein CC0149 [Caulobacter crescentus CB15]
>gi|16124410|ref|NP_418974.1| hypothetical protein CC0155 [Caulobacter crescentus CB15]
>gi|16124412|ref|NP_418976.1| transcriptional regulator, ArsR family [Caulobacter crescentus CB15]
>gi|16124414|ref|NP_418978.1| recF protein [Caulobacter crescentus CB15]
>gi|16124417|ref|NP_418981.1| hypothetical protein CC0162 [Caulobacter crescentus CB15]
>gi|16124421|ref|NP_418985.1| exopolysaccharide production protein Pss [Caulobacter crescentus CB15]
>gi|16124427|ref|NP_418991.1| hypothetical protein CC0172 [Caulobacter crescentus CB15]
>gi|16124433|ref|NP_418997.1| general secretion pathway protein I [Caulobacter crescentus CB15]
>gi|16124437|ref|NP_419001.1| hypothetical protein CC0182 [Caulobacter crescentus CB15]
>gi|16124441|ref|NP_419005.1| xanthine/uracil permease family protein [Caulobacter crescentus CB15]
>gi|16124456|ref|NP_419020.1| OmpA family protein [Caulobacter crescentus CB15]
>gi|16124471|ref|NP_419035.1| hypothetical protein CC0216 [Caulobacter crescentus CB15]
>gi|16124472|ref|NP_419036.1| thio:disulfide interchange protein, putative [Caulobacter crescentus CB15]
>gi|16124484|ref|NP_419048.1| hypothetical protein CC0229 [Caulobacter crescentus CB15]
>gi|16124485|ref|NP_419049.1| S4 domain protein [Caulobacter crescentus CB15]
>gi|16124486|ref|NP_419050.1| cytidine and deoxycytidylate deaminase family protein [Caulobacter crescentus CB15]
>gi|16124503|ref|NP_419067.1| sensor histidine kinase [Caulobacter crescentus CB15]
>gi|16124526|ref|NP_419090.1| hypothetical protein CC0271 [Caulobacter crescentus CB15]
>gi|16124535|ref|NP_419099.1| hypothetical protein CC0280 [Caulobacter crescentus CB15]
>gi|16124558|ref|NP_419122.1| cation efflux family protein [Caulobacter crescentus CB15]
>gi|16124569|ref|NP_419133.1| glutamate 5-kinase [Caulobacter crescentus CB15]
>gi|16124574|ref|NP_419138.1| ribosomal protein L21 [Caulobacter crescentus CB15]
>gi|16124575|ref|NP_419139.1| hypothetical protein CC0320 [Caulobacter crescentus CB15]
>gi|16124576|ref|NP_419140.1| hypothetical protein CC0321 [Caulobacter crescentus CB15]
>gi|16124584|ref|NP_419148.1| molybdenum ABC transporter, permease protein [Caulobacter crescentus CB15]
>gi|16124585|ref|NP_419149.1| hypothetical protein CC0330 [Caulobacter crescentus CB15]
>gi|16124595|ref|NP_419159.1| 2-oxoglutarate dehydrogenase, E2 component, dihydrolipoamide succinyltransferase [Caulobacter crescentus CB15]
>gi|16124598|ref|NP_419162.1| methyl-accepting chemotaxis protein McpC [Caulobacter crescentus CB15]
>gi|16124599|ref|NP_419163.1| integrase/recombinase XerC, putative [Caulobacter crescentus CB15]
>gi|16124600|ref|NP_419164.1| hypothetical protein CC0345 [Caulobacter crescentus CB15]
>gi|16124605|ref|NP_419169.1| pentapeptide repeat family protein [Caulobacter crescentus CB15]
>gi|16124609|ref|NP_419173.1| hypothetical protein CC0354 [Caulobacter crescentus CB15]
>gi|16124612|ref|NP_419176.1| Fur family protein [Caulobacter crescentus CB15]

>gi|16124620|ref|NP_419184.1| ATP synthase F0, B subunit [Caulobacter crescentus CB15]
>gi|16124621|ref|NP_419185.1| ATP synthase F0, B' subunit [Caulobacter crescentus CB15]
>gi|16124622|ref|NP_419186.1| ATP synthase F0, C subunit [Caulobacter crescentus CB15]
>gi|16124623|ref|NP_419187.1| ATP synthase F0, A subunit [Caulobacter crescentus CB15]
>gi|16124624|ref|NP_419188.1| hypothetical protein CC0369 [Caulobacter crescentus CB15]
>gi|16124626|ref|NP_419190.1| hypothetical protein CC0371 [Caulobacter crescentus CB15]
>gi|16124627|ref|NP_419191.1| hypothetical protein CC0372 [Caulobacter crescentus CB15]
>gi|16124628|ref|NP_419192.1| smc protein [Caulobacter crescentus CB15]
>gi|16124638|ref|NP_419202.1| hypothetical protein CC0383 [Caulobacter crescentus CB15]
>gi|16124644|ref|NP_419208.1| hypothetical protein CC0389 [Caulobacter crescentus CB15]
>gi|16124648|ref|NP_419212.1| thiamine-pyrophosphate-requiring enzyme [Caulobacter crescentus CB15]
>gi|16124654|ref|NP_419218.1| hypothetical protein CC0399 [Caulobacter crescentus CB15]
>gi|16124665|ref|NP_419229.1| TPR domain protein [Caulobacter crescentus CB15]
>gi|16124670|ref|NP_419234.1| hypothetical protein CC0415 [Caulobacter crescentus CB15]
>gi|16124680|ref|NP_419244.1| hypothetical protein CC0425 [Caulobacter crescentus CB15]
>gi|16124688|ref|NP_419252.1| chemotaxis protein CheA [Caulobacter crescentus CB15]
>gi|16124694|ref|NP_419258.1| cheU protein [Caulobacter crescentus CB15]
>gi|16124704|ref|NP_419268.1| PTS system, N-acetylglucosamine-specific IIABC component [Caulobacter crescentus CB15]
>gi|16124707|ref|NP_419271.1| hypothetical protein CC0452 [Caulobacter crescentus CB15]
>gi|16124711|ref|NP_419275.1| efflux protein, LysE family [Caulobacter crescentus CB15]
>gi|16124723|ref|NP_419287.1| hypothetical protein CC0468 [Caulobacter crescentus CB15]
>gi|16124731|ref|NP_419295.1| hypothetical protein CC0476 [Caulobacter crescentus CB15]
>gi|16124738|ref|NP_419302.1| hypothetical protein CC0483 [Caulobacter crescentus CB15]
>gi|16124749|ref|NP_419313.1| pyrroline-5-carboxylate reductase [Caulobacter crescentus CB15]
>gi|16124750|ref|NP_419314.1| transcriptional regulator, TetR family, putative [Caulobacter crescentus CB15]
>gi|16124752|ref|NP_419316.1| ribosomal protein L7/L12 [Caulobacter crescentus CB15]
>gi|16124762|ref|NP_419326.1| hypothetical protein CC0507 [Caulobacter crescentus CB15]
>gi|16124764|ref|NP_419328.1| hypothetical protein CC0509 [Caulobacter crescentus CB15]
>gi|16124770|ref|NP_419334.1| IS511, transposase OrfB [Caulobacter crescentus CB15]
>gi|16124774|ref|NP_419338.1| hydroxyacylglutathione hydrolase, putative [Caulobacter crescentus CB15]
>gi|16124776|ref|NP_419340.1| nitrogen regulatory protein P-II family protein [Caulobacter crescentus CB15]
>gi|16124783|ref|NP_419347.1| dihydroorotate dehydrogenase [Caulobacter crescentus CB15]
>gi|16124790|ref|NP_419354.1| SIS domain protein [Caulobacter crescentus CB15]
>gi|16124793|ref|NP_419357.1| PTS system, N-acetylglucosamine-specific IIABC component [Caulobacter crescentus CB15]
>gi|16124801|ref|NP_419365.1| hypothetical protein CC0546 [Caulobacter crescentus CB15]
>gi|16124804|ref|NP_419368.1| hypothetical protein CC0549 [Caulobacter crescentus CB15]
>gi|16124806|ref|NP_419370.1| hypothetical protein CC0551 [Caulobacter crescentus CB15]
>gi|16124819|ref|NP_419383.1| hypothetical protein CC0565 [Caulobacter crescentus CB15]
>gi|16124822|ref|NP_419386.1| hypothetical protein CC0568 [Caulobacter crescentus CB15]
>gi|16124824|ref|NP_419388.1| hypothetical protein CC0570 [Caulobacter crescentus CB15]
>gi|16124830|ref|NP_419394.1| asparaginase family protein [Caulobacter crescentus CB15]
>gi|16124831|ref|NP_419395.1| hypothetical protein CC0577 [Caulobacter crescentus CB15]
>gi|16124836|ref|NP_419400.1| succinylglutamic semialdehyde dehydrogenase [Caulobacter crescentus CB15]
>gi|16124841|ref|NP_419405.1| pentapeptide repeat family protein [Caulobacter crescentus CB15]
>gi|16124844|ref|NP_419408.1| hypothetical protein CC0590 [Caulobacter crescentus CB15]
>gi|16124867|ref|NP_419431.1| nitrate transporter, NarK/NasA family [Caulobacter crescentus CB15]
>gi|16124915|ref|NP_419479.1| ISCc2, transposase, fusion [Caulobacter crescentus CB15]
>gi|16124919|ref|NP_419483.1| hypothetical protein CC0666 [Caulobacter crescentus CB15]
>gi|16124926|ref|NP_419490.1| hypothetical protein CC0673 [Caulobacter crescentus CB15]
>gi|16124928|ref|NP_419492.1| articulin, putative [Caulobacter crescentus CB15]
>gi|16124929|ref|NP_419493.1| hypothetical protein CC0676 [Caulobacter crescentus CB15]
>gi|16124936|ref|NP_419500.1| HlyD family secretion protein [Caulobacter crescentus CB15]
>gi|16124941|ref|NP_419505.1| hypothetical protein CC0688 [Caulobacter crescentus CB15]
>gi|16124946|ref|NP_419510.1| hypothetical protein CC0693 [Caulobacter crescentus CB15]
>gi|16124953|ref|NP_419517.1| lipoprotein signal peptidase [Caulobacter crescentus CB15]
>gi|16124966|ref|NP_419530.1| transcriptional regulator, AraC family [Caulobacter crescentus CB15]
>gi|16124970|ref|NP_419534.1| nodulin-related protein [Caulobacter crescentus CB15]
>gi|16124971|ref|NP_419535.1| hypothetical protein CC0718 [Caulobacter crescentus CB15]
>gi|16124972|ref|NP_419536.1| hypothetical protein CC0719 [Caulobacter crescentus CB15]
>gi|16124981|ref|NP_419545.1| hypothetical protein CC0728 [Caulobacter crescentus CB15]
>gi|16124983|ref|NP_419547.1| hypothetical protein CC0730 [Caulobacter crescentus CB15]
>gi|16124992|ref|NP_419556.1| hypothetical protein CC0739 [Caulobacter crescentus CB15]
>gi|16125001|ref|NP_419565.1| hypothetical protein CC0748 [Caulobacter crescentus CB15]
>gi|16125002|ref|NP_419566.1| hypothetical protein CC0749 [Caulobacter crescentus CB15]
>gi|16125004|ref|NP_419568.1| multidrug resistance protein, putative [Caulobacter crescentus CB15]
>gi|16125007|ref|NP_419571.1| hypothetical protein CC0754 [Caulobacter crescentus CB15]
>gi|16125010|ref|NP_419574.1| hypothetical protein CC0757 [Caulobacter crescentus CB15]
>gi|16125013|ref|NP_419577.1| ABC transporter, ATP-binding protein Cydc [Caulobacter crescentus CB15]
>gi|16125014|ref|NP_419578.1| ABC transporter, ATP-binding protein CydD [Caulobacter crescentus CB15]
>gi|16125023|ref|NP_419587.1| alcohol dehydrogenase, zinc-containing [Caulobacter crescentus CB15]

>gi|16125028|ref|NP_419592.1| hypothetical protein CC0775 [Caulobacter crescentus CB15]
>gi|16125034|ref|NP_419598.1| hypothetical protein CC0781 [Caulobacter crescentus CB15]
>gi|16125035|ref|NP_419599.1| transcriptional regulator, LuxR family, putative [Caulobacter crescentus CB15]
>gi|16125039|ref|NP_419603.1| hypothetical protein CC0786 [Caulobacter crescentus CB15]
>gi|16125045|ref|NP_419609.1| flagellin FljM [Caulobacter crescentus CB15]
>gi|16125046|ref|NP_419610.1| flagellin FljN [Caulobacter crescentus CB15]
>gi|16125047|ref|NP_419611.1| flagellin FljO [Caulobacter crescentus CB15]
>gi|16125056|ref|NP_419620.1| hypothetical protein CC0803 [Caulobacter crescentus CB15]
>gi|16125059|ref|NP_419623.1| efflux system protein [Caulobacter crescentus CB15]
>gi|16125061|ref|NP_419625.1| HlyD family secretion protein [Caulobacter crescentus CB15]
>gi|16125062|ref|NP_419626.1| transcriptional regulator, TetR family [Caulobacter crescentus CB15]
>gi|16125065|ref|NP_419629.1| hypothetical protein CC0812 [Caulobacter crescentus CB15]
>gi|16125069|ref|NP_419633.1| acetyltransferase, GNAT family [Caulobacter crescentus CB15]
>gi|16125070|ref|NP_419634.1| hypothetical protein CC0817 [Caulobacter crescentus CB15]
>gi|16125078|ref|NP_419642.1| hypothetical protein CC0825 [Caulobacter crescentus CB15]
>gi|16125090|ref|NP_419654.1| hypothetical protein CC0837 [Caulobacter crescentus CB15]
>gi|16125092|ref|NP_419656.1| hypothetical protein CC0839 [Caulobacter crescentus CB15]
>gi|16125101|ref|NP_419665.1| integral membrane protein [Caulobacter crescentus CB15]
>gi|16125103|ref|NP_419667.1| hypothetical protein CC0850 [Caulobacter crescentus CB15]
>gi|16125109|ref|NP_419673.1| peptide chain release factor 1 [Caulobacter crescentus CB15]
>gi|16125123|ref|NP_419687.1| hypothetical protein CC0870 [Caulobacter crescentus CB15]
>gi|16125127|ref|NP_419691.1| hypothetical protein CC0874 [Caulobacter crescentus CB15]
>gi|16125129|ref|NP_419693.1| hypothetical protein CC0876 [Caulobacter crescentus CB15]
>gi|16125134|ref|NP_419698.1| hypothetical protein CC0881 [Caulobacter crescentus CB15]
>gi|16125136|ref|NP_419700.1| hypothetical protein CC0883 [Caulobacter crescentus CB15]
>gi|16125138|ref|NP_419702.1| riboflavin biosynthesis protein RibD [Caulobacter crescentus CB15]
>gi|16125156|ref|NP_419720.1| hypothetical protein CC0904 [Caulobacter crescentus CB15]
>gi|16125159|ref|NP_419723.1| flbE protein [Caulobacter crescentus CB15]
>gi|16125166|ref|NP_419730.1| hypothetical protein CC0914 [Caulobacter crescentus CB15]
>gi|16125174|ref|NP_419738.1| medium-chain-fatty-acid--CoA ligase [Caulobacter crescentus CB15]
>gi|16125176|ref|NP_419740.1| hypothetical protein CC0924 [Caulobacter crescentus CB15]
>gi|16125182|ref|NP_419746.1| HlyD family secretion protein, putative [Caulobacter crescentus CB15]
>gi|16125190|ref|NP_419754.1| hypothetical protein CC0938 [Caulobacter crescentus CB15]
>gi|16125204|ref|NP_419768.1| fliO protein [Caulobacter crescentus CB15]
>gi|16125217|ref|NP_419781.1| copper-binding protein, putative [Caulobacter crescentus CB15]
>gi|16125228|ref|NP_419792.1| hypothetical protein CC0976 [Caulobacter crescentus CB15]
>gi|16125234|ref|NP_419798.1| regulatory protein PupR, putative [Caulobacter crescentus CB15]
>gi|16125249|ref|NP_419813.1| hypothetical protein CC0997 [Caulobacter crescentus CB15]
>gi|16125257|ref|NP_419821.1| hypothetical protein CC1005 [Caulobacter crescentus CB15]
>gi|16125259|ref|NP_419823.1| S-layer protein RsaA [Caulobacter crescentus CB15]
>gi|16125263|ref|NP_419827.1| hexapeptide transferase family protein [Caulobacter crescentus CB15]
>gi|16125275|ref|NP_419839.1| beta/gamma crystallin family protein [Caulobacter crescentus CB15]
>gi|16125276|ref|NP_419840.1| beta/gamma crystallin family protein [Caulobacter crescentus CB15]
>gi|16125282|ref|NP_419846.1| hypothetical protein CC1030 [Caulobacter crescentus CB15]
>gi|16125287|ref|NP_419851.1| hypothetical protein CC1035 [Caulobacter crescentus CB15]
>gi|16125299|ref|NP_419863.1| multidrug resistance protein, putative [Caulobacter crescentus CB15]
>gi|16125315|ref|NP_419879.1| sensor histidine kinase DivJ [Caulobacter crescentus CB15]
>gi|16125325|ref|NP_419889.1| hypothetical protein CC1073 [Caulobacter crescentus CB15]
>gi|16125330|ref|NP_419894.1| cell cycle histidine kinase CckA [Caulobacter crescentus CB15]
>gi|16125332|ref|NP_419896.1| transcriptional regulator, LuxR family [Caulobacter crescentus CB15]
>gi|16125336|ref|NP_419900.1| hypothetical protein CC1084 [Caulobacter crescentus CB15]
>gi|16125346|ref|NP_419910.1| hypothetical protein CC1094 [Caulobacter crescentus CB15]
>gi|16125349|ref|NP_419913.1| chromate transporter, putative [Caulobacter crescentus CB15]
>gi|16125350|ref|NP_419914.1| hypothetical protein CC1098 [Caulobacter crescentus CB15]
>gi|16125354|ref|NP_419918.1| hypothetical protein CC1102 [Caulobacter crescentus CB15]
>gi|16125358|ref|NP_419922.1| hypothetical protein CC1106 [Caulobacter crescentus CB15]
>gi|16125370|ref|NP_419934.1| sirohaem synthase [Caulobacter crescentus CB15]
>gi|16125375|ref|NP_419939.1| hypothetical protein CC1123 [Caulobacter crescentus CB15]
>gi|16125379|ref|NP_419943.1| hypothetical protein CC1127 [Caulobacter crescentus CB15]
>gi|16125382|ref|NP_419946.1| transcriptional regulator, putative [Caulobacter crescentus CB15]
>gi|16125400|ref|NP_419964.1| hypothetical protein CC1148 [Caulobacter crescentus CB15]
>gi|16125401|ref|NP_419965.1| hypothetical protein CC1149 [Caulobacter crescentus CB15]
>gi|16125405|ref|NP_419969.1| hypothetical protein CC1153 [Caulobacter crescentus CB15]
>gi|16125408|ref|NP_419972.1| hypothetical protein CC1156 [Caulobacter crescentus CB15]
>gi|16125413|ref|NP_419977.1| hypothetical protein CC1161 [Caulobacter crescentus CB15]
>gi|16125416|ref|NP_419980.1| hypothetical protein CC1164 [Caulobacter crescentus CB15]
>gi|16125422|ref|NP_419986.1| isochorismatase family protein [Caulobacter crescentus CB15]
>gi|16125431|ref|NP_419995.1| hypothetical protein CC1179 [Caulobacter crescentus CB15]
>gi|16125432|ref|NP_419996.1| hypothetical protein CC1180 [Caulobacter crescentus CB15]
>gi|16125443|ref|NP_420007.1| iron compound ABC transporter, permease protein [Caulobacter crescentus CB15]

>gi|16125444|ref|NP_420008.1| iron compound ABC transporter, ATP-binding protein [Caulobacter crescentus CB15]
>gi|16125451|ref|NP_420015.1| hypothetical protein CC1200 [Caulobacter crescentus CB15]
>gi|16125460|ref|NP_420024.1| cytochrome c family protein [Caulobacter crescentus CB15]
>gi|16125487|ref|NP_420051.1| hypothetical protein CC1238 [Caulobacter crescentus CB15]
>gi|16125494|ref|NP_420058.1| hypothetical protein CC1245 [Caulobacter crescentus CB15]
>gi|16125513|ref|NP_420077.1| ribosomal protein L18 [Caulobacter crescentus CB15]
>gi|16125524|ref|NP_420088.1| hypothetical protein CC1275 [Caulobacter crescentus CB15]
>gi|16125530|ref|NP_420094.1| tetracycline resistance protein [Caulobacter crescentus CB15]
>gi|16125554|ref|NP_420118.1| sensor histidine kinase [Caulobacter crescentus CB15]
>gi|16125556|ref|NP_420120.1| hypothetical protein CC1307 [Caulobacter crescentus CB15]
>gi|16125557|ref|NP_420121.1| hypothetical protein CC1308 [Caulobacter crescentus CB15]
>gi|16125558|ref|NP_420122.1| nicotinate-nucleotide--dimethylbenzimidazole phosphoribosyltransferase [Caulobacter crescentus CB15]
>gi|16125561|ref|NP_420125.1| hypothetical protein CC1312 [Caulobacter crescentus CB15]
>gi|16125568|ref|NP_420132.1| hypothetical protein CC1319 [Caulobacter crescentus CB15]
>gi|16125574|ref|NP_420138.1| hypothetical protein CC1325 [Caulobacter crescentus CB15]
>gi|16125580|ref|NP_420144.1| hypothetical protein CC1331 [Caulobacter crescentus CB15]
>gi|16125582|ref|NP_420146.1| phage SPO1 DNA polymerase-related protein [Caulobacter crescentus CB15]
>gi|16125584|ref|NP_420148.1| TPR domain protein [Caulobacter crescentus CB15]
>gi|16125587|ref|NP_420151.1| ammonium transporter [Caulobacter crescentus CB15]
>gi|16125589|ref|NP_420153.1| hypothetical protein CC1340 [Caulobacter crescentus CB15]
>gi|16125603|ref|NP_420167.1| 3-hydroxyisobutyrate dehydrogenase [Caulobacter crescentus CB15]
>gi|16125623|ref|NP_420187.1| cytochrome c oxidase assembly protein, putative [Caulobacter crescentus CB15]
>gi|16125630|ref|NP_420194.1| hypothetical protein CC1381 [Caulobacter crescentus CB15]
>gi|16125647|ref|NP_420211.1| hypothetical protein CC1398 [Caulobacter crescentus CB15]
>gi|16125648|ref|NP_420212.1| methyl-accepting chemotaxis protein McpP [Caulobacter crescentus CB15]
>gi|16125656|ref|NP_420220.1| cation-transporting ATPase, E1-E2 family [Caulobacter crescentus CB15]
>gi|16125683|ref|NP_420247.1| hypothetical protein CC1434 [Caulobacter crescentus CB15]
>gi|16125685|ref|NP_420249.1| hypothetical protein CC1436 [Caulobacter crescentus CB15]
>gi|16125692|ref|NP_420256.1| hypothetical protein CC1443 [Caulobacter crescentus CB15]
>gi|16125697|ref|NP_420261.1| DedA family protein [Caulobacter crescentus CB15]
>gi|16125699|ref|NP_420263.1| hypothetical protein CC1450 [Caulobacter crescentus CB15]
>gi|16125709|ref|NP_420273.1| flagellin FljL [Caulobacter crescentus CB15]
>gi|16125710|ref|NP_420274.1| flagellin FljK [Caulobacter crescentus CB15]
>gi|16125714|ref|NP_420278.1| hypothetical protein CC1466 [Caulobacter crescentus CB15]
>gi|16125716|ref|NP_420280.1| single-strand binding protein [Caulobacter crescentus CB15]
>gi|16125722|ref|NP_420286.1| transcriptional regulator, LysR family [Caulobacter crescentus CB15]
>gi|16125723|ref|NP_420287.1| hypothetical protein CC1475 [Caulobacter crescentus CB15]
>gi|16125728|ref|NP_420292.1| membrane protein, putative [Caulobacter crescentus CB15]
>gi|16125732|ref|NP_420296.1| hypothetical protein CC1485 [Caulobacter crescentus CB15]
>gi|16125741|ref|NP_420305.1| hypothetical protein CC1494 [Caulobacter crescentus CB15]
>gi|16125760|ref|NP_420324.1| hypothetical protein CC1513 [Caulobacter crescentus CB15]
>gi|16125763|ref|NP_420327.1| penicillin-binding protein, 1A family [Caulobacter crescentus CB15]
>gi|16125766|ref|NP_420330.1| hypothetical protein CC1519 [Caulobacter crescentus CB15]
>gi|16125767|ref|NP_420331.1| hypothetical protein CC1520 [Caulobacter crescentus CB15]
>gi|16125774|ref|NP_420338.1| hypothetical protein CC1527 [Caulobacter crescentus CB15]
>gi|16125777|ref|NP_420341.1| hypothetical protein CC1530 [Caulobacter crescentus CB15]
>gi|16125789|ref|NP_420353.1| hypothetical protein CC1542 [Caulobacter crescentus CB15]
>gi|16125791|ref|NP_420355.1| rod shape-determining protein MreC [Caulobacter crescentus CB15]
>gi|16125811|ref|NP_420375.1| hypothetical protein CC1564 [Caulobacter crescentus CB15]
>gi|16125836|ref|NP_420400.1| hypothetical protein CC1589 [Caulobacter crescentus CB15]
>gi|16125844|ref|NP_420408.1| sulfate ABC transporter, permease protein [Caulobacter crescentus CB15]
>gi|16125852|ref|NP_420416.1| peptidase, M20/M25/M40 family [Caulobacter crescentus CB15]
>gi|16125892|ref|NP_420456.1| RNA polymerase sigma-70 factor, ECF subfamily [Caulobacter crescentus CB15]
>gi|16125893|ref|NP_420457.1| hypothetical protein CC1647 [Caulobacter crescentus CB15]
>gi|16125900|ref|NP_420464.1| GTP-binding protein [Caulobacter crescentus CB15]
>gi|16125919|ref|NP_420483.1| bacterioferritin comigratory protein, putative [Caulobacter crescentus CB15]
>gi|16125928|ref|NP_420492.1| hypothetical protein CC1682 [Caulobacter crescentus CB15]
>gi|16125936|ref|NP_420500.1| hypothetical protein CC1691 [Caulobacter crescentus CB15]
>gi|16125939|ref|NP_420503.1| hypothetical protein CC1694 [Caulobacter crescentus CB15]
>gi|16125945|ref|NP_420509.1| DedA family protein [Caulobacter crescentus CB15]
>gi|16125955|ref|NP_420519.1| DnaA-related protein [Caulobacter crescentus CB15]
>gi|16125963|ref|NP_420527.1| hypothetical protein CC1719 [Caulobacter crescentus CB15]
>gi|16125969|ref|NP_420533.1| hypothetical protein CC1725 [Caulobacter crescentus CB15]
>gi|16125973|ref|NP_420537.1| pyruvate dehydrogenase complex, E2 component, dihydrolipoamide acetyltransferase [Caulobacter crescentus CB15]
>gi|16126002|ref|NP_420566.1| hypothetical protein CC1758 [Caulobacter crescentus CB15]
>gi|16126004|ref|NP_420568.1| acyl-CoA thioesterase I [Caulobacter crescentus CB15]
>gi|16126023|ref|NP_420587.1| ThiJ/PfpI family protein [Caulobacter crescentus CB15]
>gi|16126029|ref|NP_420593.1| efflux system protein [Caulobacter crescentus CB15]
>gi|16126031|ref|NP_420595.1| HlyD family secretion protein [Caulobacter crescentus CB15]

>gi|16126033|ref|NP_420597.1| hypothetical protein CC1789 [Caulobacter crescentus CB15]
>gi|16126037|ref|NP_420601.1| hypothetical protein CC1793 [Caulobacter crescentus CB15]
>gi|16126057|ref|NP_420621.1| enoyl-CoA hydratase/isomerase family protein [Caulobacter crescentus CB15]
>gi|16126062|ref|NP_420626.1| major facilitator family transporter [Caulobacter crescentus CB15]
>gi|16126066|ref|NP_420630.1| DNA polymerase III, delta prime subunit [Caulobacter crescentus CB15]
>gi|16126068|ref|NP_420632.1| rare lipoprotein A [Caulobacter crescentus CB15]
>gi|16126075|ref|NP_420639.1| ABC transporter, permease protein [Caulobacter crescentus CB15]
>gi|16126090|ref|NP_420654.1| ATP-dependent RNA helicase, DEAD/DEAH box family [Caulobacter crescentus CB15]
>gi|16126098|ref|NP_420662.1| transcriptional regulator, TetR family [Caulobacter crescentus CB15]
>gi|16126120|ref|NP_420684.1| ribonuclease, Rne/Rng family protein [Caulobacter crescentus CB15]
>gi|16126126|ref|NP_420690.1| acetyl-CoA carboxylase, biotin carboxyl carrier protein [Caulobacter crescentus CB15]
>gi|16126129|ref|NP_420693.1| hypothetical protein CC1886 [Caulobacter crescentus CB15]
>gi|16126132|ref|NP_420696.1| ribonucleotide reductase-related protein [Caulobacter crescentus CB15]
>gi|16126133|ref|NP_420697.1| pentapeptide repeat family protein [Caulobacter crescentus CB15]
>gi|16126134|ref|NP_420698.1| pentapeptide repeat family protein [Caulobacter crescentus CB15]
>gi|16126141|ref|NP_420705.1| anthranilate phosphoribosyltransferase [Caulobacter crescentus CB15]
>gi|16126147|ref|NP_420711.1| ComEC/Rec2 family protein [Caulobacter crescentus CB15]
>gi|16126151|ref|NP_420715.1| hypothetical protein CC1908 [Caulobacter crescentus CB15]
>gi|16126156|ref|NP_420720.1| UDP-3-O-3-hydroxymyristoyl glucosamine N-acyltransferase [Caulobacter crescentus CB15]
>gi|16126160|ref|NP_420724.1| 1-deoxy-D-xylulose 5-phosphate reductoisomerase [Caulobacter crescentus CB15]
>gi|16126161|ref|NP_420725.1| phosphatidate cytidylyltransferase [Caulobacter crescentus CB15]
>gi|16126180|ref|NP_420744.1| NADH dehydrogenase I, N subunit [Caulobacter crescentus CB15]
>gi|16126184|ref|NP_420748.1| NADH dehydrogenase I, J subunit [Caulobacter crescentus CB15]
>gi|16126196|ref|NP_420760.1| hypothetical protein CC1953 [Caulobacter crescentus CB15]
>gi|16126222|ref|NP_420786.1| hypothetical protein CC1979 [Caulobacter crescentus CB15]
>gi|16126226|ref|NP_420790.1| DNA repair protein RecN [Caulobacter crescentus CB15]
>gi|16126230|ref|NP_420794.1| hypothetical protein CC1987 [Caulobacter crescentus CB15]
>gi|16126239|ref|NP_420803.1| peptidase, M23/M37 family [Caulobacter crescentus CB15]
>gi|16126243|ref|NP_420807.1| hypothetical protein CC2000 [Caulobacter crescentus CB15]
>gi|16126245|ref|NP_420809.1| hypothetical protein CC2002 [Caulobacter crescentus CB15]
>gi|16126250|ref|NP_420814.1| hypothetical protein CC2007 [Caulobacter crescentus CB15]
>gi|16126255|ref|NP_420819.1| hypothetical protein CC2012 [Caulobacter crescentus CB15]
>gi|16126257|ref|NP_420821.1| hypothetical protein CC2014 [Caulobacter crescentus CB15]
>gi|16126263|ref|NP_420827.1| MATE efflux family protein [Caulobacter crescentus CB15]
>gi|16126273|ref|NP_420837.1| hypothetical protein CC2030 [Caulobacter crescentus CB15]
>gi|16126281|ref|NP_420845.1| hypothetical protein CC2038 [Caulobacter crescentus CB15]
>gi|16126300|ref|NP_420864.1| hypothetical protein CC2059 [Caulobacter crescentus CB15]
>gi|16126301|ref|NP_420865.1| hypothetical protein CC2060 [Caulobacter crescentus CB15]
>gi|16126304|ref|NP_420868.1| distal basal-body ring component protein FlaD [Caulobacter crescentus CB15]
>gi|16126311|ref|NP_420875.1| histone deacetylase family protein [Caulobacter crescentus CB15]
>gi|16126314|ref|NP_420878.1| hypothetical protein CC2075 [Caulobacter crescentus CB15]
>gi|16126320|ref|NP_420884.1| hypothetical protein CC2081 [Caulobacter crescentus CB15]
>gi|16126331|ref|NP_420895.1| HlyD family secretion protein [Caulobacter crescentus CB15]
>gi|16126341|ref|NP_420905.1| hypothetical protein CC2102 [Caulobacter crescentus CB15]
>gi|16126345|ref|NP_420909.1| hypothetical protein CC2106 [Caulobacter crescentus CB15]
>gi|16126347|ref|NP_420911.1| hypothetical protein CC2108 [Caulobacter crescentus CB15]
>gi|16126349|ref|NP_420913.1| acetyltransferase, GNAT family [Caulobacter crescentus CB15]
>gi|16126360|ref|NP_420924.1| hypothetical protein CC2121 [Caulobacter crescentus CB15]
>gi|16126362|ref|NP_420926.1| hypothetical protein CC2123 [Caulobacter crescentus CB15]
>gi|16126364|ref|NP_420928.1| alcohol dehydrogenase, zinc-containing [Caulobacter crescentus CB15]
>gi|16126372|ref|NP_420936.1| hypothetical protein CC2133 [Caulobacter crescentus CB15]
>gi|16126374|ref|NP_420938.1| hypothetical protein CC2135 [Caulobacter crescentus CB15]
>gi|16126385|ref|NP_420949.1| hypothetical protein CC2146 [Caulobacter crescentus CB15]
>gi|16126390|ref|NP_420954.1| hypothetical protein CC2151 [Caulobacter crescentus CB15]
>gi|16126394|ref|NP_420958.1| hypothetical protein CC2155 [Caulobacter crescentus CB15]
>gi|16126399|ref|NP_420963.1| hypothetical protein CC2160 [Caulobacter crescentus CB15]
>gi|16126405|ref|NP_420969.1| pantoate--beta-alanine ligase [Caulobacter crescentus CB15]
>gi|16126412|ref|NP_420976.1| lysozyme family protein [Caulobacter crescentus CB15]
>gi|16126420|ref|NP_420984.1| glcG protein, putative [Caulobacter crescentus CB15]
>gi|16126423|ref|NP_420987.1| hypothetical protein CC2184 [Caulobacter crescentus CB15]
>gi|16126427|ref|NP_420991.1| transcriptional regulator, MarR family [Caulobacter crescentus CB15]
>gi|16126429|ref|NP_420993.1| hypothetical protein CC2190 [Caulobacter crescentus CB15]
>gi|16126432|ref|NP_420996.1| hypothetical protein CC2193 [Caulobacter crescentus CB15]
>gi|16126441|ref|NP_421005.1| transcription elongation factor GreA/GreB, family [Caulobacter crescentus CB15]
>gi|16126446|ref|NP_421010.1| hypothetical protein CC2207 [Caulobacter crescentus CB15]
>gi|16126451|ref|NP_421015.1| hypothetical protein CC2212 [Caulobacter crescentus CB15]
>gi|16126454|ref|NP_421018.1| hypothetical protein CC2215 [Caulobacter crescentus CB15]
>gi|16126456|ref|NP_421020.1| hypothetical protein CC2217 [Caulobacter crescentus CB15]
>gi|16126474|ref|NP_421038.1| hypothetical protein CC2235 [Caulobacter crescentus CB15]
>gi|16126484|ref|NP_421048.1| hypothetical protein CC2245 [Caulobacter crescentus CB15]

>gi|16126498|ref|NP_421062.1| pentapeptide repeat family protein [Caulobacter crescentus CB15]
>gi|16126499|ref|NP_421063.1| pentapeptide repeat family protein [Caulobacter crescentus CB15]
>gi|16126520|ref|NP_421084.1| methyl-accepting chemotaxis protein McpE [Caulobacter crescentus CB15]
>gi|16126529|ref|NP_421093.1| IS511, transposase OrfB [Caulobacter crescentus CB15]
>gi|16126535|ref|NP_421099.1| hypothetical protein CC2296 [Caulobacter crescentus CB15]
>gi|16126536|ref|NP_421100.1| peptidase, M23/M37 family [Caulobacter crescentus CB15]
>gi|16126563|ref|NP_421127.1| sensor histidine kinase/response regulator [Caulobacter crescentus CB15]
>gi|16126566|ref|NP_421130.1| TonB protein, putative [Caulobacter crescentus CB15]
>gi|16126568|ref|NP_421132.1| D-amino acid oxidase family protein [Caulobacter crescentus CB15]
>gi|16126573|ref|NP_421137.1| hypothetical protein CC2334 [Caulobacter crescentus CB15]
>gi|16126575|ref|NP_421139.1| MotA/TolQ/ExbB proton channel family protein [Caulobacter crescentus CB15]
>gi|16126576|ref|NP_421140.1| oxidoreductase, short-chain dehydrogenase/reductase family [Caulobacter crescentus CB15]
>gi|16126581|ref|NP_421145.1| maf protein [Caulobacter crescentus CB15]
>gi|16126590|ref|NP_421154.1| hypothetical protein CC2351 [Caulobacter crescentus CB15]
>gi|16126619|ref|NP_421183.1| hypothetical protein CC2380 [Caulobacter crescentus CB15]
>gi|16126624|ref|NP_421188.1| hypothetical protein CC2385 [Caulobacter crescentus CB15]
>gi|16126627|ref|NP_421191.1| metal ion efflux outer membrane factor protein family [Caulobacter crescentus CB15]
>gi|16126630|ref|NP_421194.1| transcriptional regulator, MarR family [Caulobacter crescentus CB15]
>gi|16126631|ref|NP_421195.1| transcriptional regulator, GntR family [Caulobacter crescentus CB15]
>gi|16126651|ref|NP_421215.1| major facilitator family transporter [Caulobacter crescentus CB15]
>gi|16126656|ref|NP_421220.1| hypothetical protein CC2417 [Caulobacter crescentus CB15]
>gi|16126659|ref|NP_421223.1| hypothetical protein CC2420 [Caulobacter crescentus CB15]
>gi|16126661|ref|NP_421225.1| type IV secretion system protein B10, putative [Caulobacter crescentus CB15]
>gi|16126664|ref|NP_421228.1| hypothetical protein CC2425 [Caulobacter crescentus CB15]
>gi|16126665|ref|NP_421229.1| polysaccharide biosynthesis protein, putative [Caulobacter crescentus CB15]
>gi|16126674|ref|NP_421238.1| hypothetical protein CC2435 [Caulobacter crescentus CB15]
>gi|16126680|ref|NP_421244.1| hypothetical protein CC2441 [Caulobacter crescentus CB15]
>gi|16126681|ref|NP_421245.1| hypothetical protein CC2442 [Caulobacter crescentus CB15]
>gi|16126684|ref|NP_421248.1| hypothetical protein CC2445 [Caulobacter crescentus CB15]
>gi|16126685|ref|NP_421249.1| hypothetical protein CC2446 [Caulobacter crescentus CB15]
>gi|16126688|ref|NP_421252.1| hypothetical protein CC2449 [Caulobacter crescentus CB15]
>gi|16126691|ref|NP_421255.1| hypothetical protein CC2452 [Caulobacter crescentus CB15]
>gi|16126696|ref|NP_421260.1| hypothetical protein CC2457 [Caulobacter crescentus CB15]
>gi|16126708|ref|NP_421272.1| hypothetical protein CC2469 [Caulobacter crescentus CB15]
>gi|16126710|ref|NP_421274.1| hypothetical protein CC2471 [Caulobacter crescentus CB15]
>gi|16126714|ref|NP_421278.1| hypothetical protein CC2475 [Caulobacter crescentus CB15]
>gi|16126721|ref|NP_421285.1| non-motile and phage-resistance protein [Caulobacter crescentus CB15]
>gi|16126724|ref|NP_421288.1| major facilitator family transporter [Caulobacter crescentus CB15]
>gi|16126732|ref|NP_421296.1| transcriptional regulator, TetR family [Caulobacter crescentus CB15]
>gi|16126737|ref|NP_421301.1| hypothetical protein CC2498 [Caulobacter crescentus CB15]
>gi|16126749|ref|NP_421313.1| hypothetical protein CC2510 [Caulobacter crescentus CB15]
>gi|16126753|ref|NP_421317.1| hypothetical protein CC2514 [Caulobacter crescentus CB15]
>gi|16126758|ref|NP_421322.1| hypothetical protein CC2519 [Caulobacter crescentus CB15]
>gi|16126763|ref|NP_421327.1| hypothetical protein CC2524 [Caulobacter crescentus CB15]
>gi|16126775|ref|NP_421339.1| hypothetical protein CC2536 [Caulobacter crescentus CB15]
>gi|16126779|ref|NP_421343.1| cell division protein FtsZ [Caulobacter crescentus CB15]
>gi|16126781|ref|NP_421345.1| cell division protein FtsQ [Caulobacter crescentus CB15]
>gi|16126794|ref|NP_421358.1| voltage gated chloride channel family protein [Caulobacter crescentus CB15]
>gi|16126799|ref|NP_421363.1| hypothetical protein CC2561 [Caulobacter crescentus CB15]
>gi|16126803|ref|NP_421367.1| hypothetical protein CC2565 [Caulobacter crescentus CB15]
>gi|16126812|ref|NP_421376.1| hypothetical protein CC2574 [Caulobacter crescentus CB15]
>gi|16126819|ref|NP_421383.1| fliX protein [Caulobacter crescentus CB15]
>gi|16126822|ref|NP_421386.1| flagellar basal-body protein FlbY [Caulobacter crescentus CB15]
>gi|16126823|ref|NP_421387.1| hypothetical protein CC2585 [Caulobacter crescentus CB15]
>gi|16126825|ref|NP_421389.1| ABC transporter, ATP-binding protein [Caulobacter crescentus CB15]
>gi|16126830|ref|NP_421394.1| hypothetical protein CC2593 [Caulobacter crescentus CB15]
>gi|16126832|ref|NP_421396.1| hypothetical protein CC2595 [Caulobacter crescentus CB15]
>gi|16126833|ref|NP_421397.1| hypothetical protein CC2596 [Caulobacter crescentus CB15]
>gi|16126834|ref|NP_421398.1| hypothetical protein CC2597 [Caulobacter crescentus CB15]
>gi|16126839|ref|NP_421403.1| hypothetical protein CC2602 [Caulobacter crescentus CB15]
>gi|16126847|ref|NP_421411.1| calcium-binding protein [Caulobacter crescentus CB15]
>gi|16126869|ref|NP_421433.1| ABC transporter, ATP-binding protein [Caulobacter crescentus CB15]
>gi|16126870|ref|NP_421434.1| ABC transporter, permease protein, putative [Caulobacter crescentus CB15]
>gi|16126871|ref|NP_421435.1| hypothetical protein CC2636 [Caulobacter crescentus CB15]
>gi|16126874|ref|NP_421438.1| hypothetical protein CC2639 [Caulobacter crescentus CB15]
>gi|16126878|ref|NP_421442.1| bacterial transferase family protein [Caulobacter crescentus CB15]
>gi|16126890|ref|NP_421454.1| oxidoreductase, short-chain dehydrogenase/reductase family [Caulobacter crescentus CB15]
>gi|16126894|ref|NP_421458.1| hypothetical protein CC2659 [Caulobacter crescentus CB15]
>gi|16126897|ref|NP_421461.1| transcriptional regulator, TetR family [Caulobacter crescentus CB15]
>gi|16126903|ref|NP_421467.1| ABC transporter, permease protein [Caulobacter crescentus CB15]

>gi|16126923|ref|NP_421487.1| IS511, transposase OrfB [Caulobacter crescentus CB15]
>gi|16126949|ref|NP_421513.1| hypothetical protein CC2716 [Caulobacter crescentus CB15]
>gi|16126952|ref|NP_421516.1| hypothetical protein CC2719 [Caulobacter crescentus CB15]
>gi|16126954|ref|NP_421518.1| metal ion efflux outer membrane factor protein family [Caulobacter crescentus CB15]
>gi|16126955|ref|NP_421519.1| metal ion efflux membrane fusion protein family [Caulobacter crescentus CB15]
>gi|16126956|ref|NP_421520.1| hypothetical protein CC2723 [Caulobacter crescentus CB15]
>gi|16126963|ref|NP_421527.1| hypothetical protein CC2730 [Caulobacter crescentus CB15]
>gi|16126964|ref|NP_421528.1| hypothetical protein CC2731 [Caulobacter crescentus CB15]
>gi|16126969|ref|NP_421533.1| transcriptional regulator, Cro/CI family [Caulobacter crescentus CB15]
>gi|16126975|ref|NP_421539.1| IS511, transposase OrfB [Caulobacter crescentus CB15]
>gi|16126981|ref|NP_421545.1| hypothetical protein CC2749 [Caulobacter crescentus CB15]
>gi|16126982|ref|NP_421546.1| hypothetical protein CC2750 [Caulobacter crescentus CB15]
>gi|16126994|ref|NP_421558.1| cytochrome c-type biogenesis protein CycH [Caulobacter crescentus CB15]
>gi|16127000|ref|NP_421564.1| hypothetical protein CC2768 [Caulobacter crescentus CB15]
>gi|16127001|ref|NP_421565.1| hypothetical protein CC2769 [Caulobacter crescentus CB15]
>gi|16127004|ref|NP_421568.1| dnaJ family protein [Caulobacter crescentus CB15]
>gi|16127005|ref|NP_421569.1| hypothetical protein CC2773 [Caulobacter crescentus CB15]
>gi|16127006|ref|NP_421570.1| hypothetical protein CC2774 [Caulobacter crescentus CB15]
>gi|16127009|ref|NP_421573.1| hypothetical protein CC2777 [Caulobacter crescentus CB15]
>gi|16127011|ref|NP_421575.1| hypothetical protein CC2779 [Caulobacter crescentus CB15]
>gi|16127012|ref|NP_421576.1| hypothetical protein CC2780 [Caulobacter crescentus CB15]
>gi|16127014|ref|NP_421578.1| hypothetical protein CC2782 [Caulobacter crescentus CB15]
>gi|16127016|ref|NP_421580.1| hypothetical protein CC2784 [Caulobacter crescentus CB15]
>gi|16127019|ref|NP_421583.1| hypothetical protein CC2787 [Caulobacter crescentus CB15]
>gi|16127022|ref|NP_421586.1| hypothetical protein CC2790 [Caulobacter crescentus CB15]
>gi|16127026|ref|NP_421590.1| transcriptional regulator, TetR family [Caulobacter crescentus CB15]
>gi|16127042|ref|NP_421606.1| methyl-accepting chemotaxis protein McpR, putative [Caulobacter crescentus CB15]
>gi|16127050|ref|NP_421614.1| TPR domain protein [Caulobacter crescentus CB15]
>gi|16127054|ref|NP_421618.1| hypothetical protein CC2822 [Caulobacter crescentus CB15]
>gi|16127060|ref|NP_421624.1| hypothetical protein CC2828 [Caulobacter crescentus CB15]
>gi|16127061|ref|NP_421625.1| hypothetical protein CC2829 [Caulobacter crescentus CB15]
>gi|16127062|ref|NP_421626.1| hypothetical protein CC2830 [Caulobacter crescentus CB15]
>gi|16127071|ref|NP_421635.1| hypothetical protein CC2839 [Caulobacter crescentus CB15]
>gi|16127073|ref|NP_421637.1| hypothetical protein CC2841 [Caulobacter crescentus CB15]
>gi|16127074|ref|NP_421638.1| methyl-accepting chemotaxis protein McpO [Caulobacter crescentus CB15]
>gi|16127077|ref|NP_421641.1| OmpA family protein [Caulobacter crescentus CB15]
>gi|16127078|ref|NP_421642.1| hypothetical protein CC2846 [Caulobacter crescentus CB15]
>gi|16127079|ref|NP_421643.1| methyl-accepting chemotaxis protein McpI [Caulobacter crescentus CB15]
>gi|16127086|ref|NP_421650.1| flagellin modification protein FlmD [Caulobacter crescentus CB15]
>gi|16127094|ref|NP_421658.1| hypothetical protein CC2862 [Caulobacter crescentus CB15]
>gi|16127095|ref|NP_421659.1| rhodanese family protein [Caulobacter crescentus CB15]
>gi|16127102|ref|NP_421666.1| hypothetical protein CC2870 [Caulobacter crescentus CB15]
>gi|16127108|ref|NP_421672.1| transcriptional regulator, TetR family [Caulobacter crescentus CB15]
>gi|16127122|ref|NP_421686.1| hypothetical protein CC2890 [Caulobacter crescentus CB15]
>gi|16127125|ref|NP_421689.1| hypothetical protein CC2893 [Caulobacter crescentus CB15]
>gi|16127129|ref|NP_421693.1| hypothetical protein CC2899 [Caulobacter crescentus CB15]
>gi|16127134|ref|NP_421698.1| hypothetical protein CC2904 [Caulobacter crescentus CB15]
>gi|16127137|ref|NP_421701.1| hypothetical protein CC2907 [Caulobacter crescentus CB15]
>gi|16127143|ref|NP_421707.1| L-aspartate oxidase [Caulobacter crescentus CB15]
>gi|16127146|ref|NP_421710.1| hypothetical protein CC2916 [Caulobacter crescentus CB15]
>gi|16127151|ref|NP_421715.1| hypothetical protein CC2921 [Caulobacter crescentus CB15]
>gi|16127152|ref|NP_421716.1| hypothetical protein CC2922 [Caulobacter crescentus CB15]
>gi|16127165|ref|NP_421729.1| cytochrome c, membrane-bound [Caulobacter crescentus CB15]
>gi|16127168|ref|NP_421732.1| TPR domain protein [Caulobacter crescentus CB15]
>gi|16127173|ref|NP_421737.1| pilus assembly protein CpaE [Caulobacter crescentus CB15]
>gi|16127174|ref|NP_421738.1| pilus assembly protein CpaD [Caulobacter crescentus CB15]
>gi|16127176|ref|NP_421740.1| pilus assembly protein CpaB [Caulobacter crescentus CB15]
>gi|16127177|ref|NP_421741.1| pilus assembly protein CpaA [Caulobacter crescentus CB15]
>gi|16127178|ref|NP_421742.1| pilus subunit protein PilA [Caulobacter crescentus CB15]
>gi|16127179|ref|NP_421743.1| hypothetical protein CC2949 [Caulobacter crescentus CB15]
>gi|16127182|ref|NP_421746.1| 4-amino-4-deoxychorismate lyase, putative [Caulobacter crescentus CB15]
>gi|16127200|ref|NP_421764.1| cytochrome c family protein [Caulobacter crescentus CB15]
>gi|16127208|ref|NP_421772.1| hypothetical protein CC2978 [Caulobacter crescentus CB15]
>gi|16127219|ref|NP_421783.1| hypothetical protein CC2989 [Caulobacter crescentus CB15]
>gi|16127221|ref|NP_421785.1| hypothetical protein CC2991 [Caulobacter crescentus CB15]
>gi|16127223|ref|NP_421787.1| sensor histidine kinase/response regulator [Caulobacter crescentus CB15]
>gi|16127234|ref|NP_421798.1| hypothetical protein CC3004 [Caulobacter crescentus CB15]
>gi|16127235|ref|NP_421799.1| phnA protein [Caulobacter crescentus CB15]
>gi|16127237|ref|NP_421801.1| hypothetical protein CC3007 [Caulobacter crescentus CB15]
>gi|16127249|ref|NP_421813.1| PAP2 superfamily protein [Caulobacter crescentus CB15]

>gi|16127252|ref|NP_421816.1| hypothetical protein CC3022 [Caulobacter crescentus CB15]
>gi|16127253|ref|NP_421817.1| hypothetical protein CC3023 [Caulobacter crescentus CB15]
>gi|16127254|ref|NP_421818.1| hypothetical protein CC3024 [Caulobacter crescentus CB15]
>gi|16127256|ref|NP_421820.1| hypothetical protein CC3026 [Caulobacter crescentus CB15]
>gi|16127263|ref|NP_421827.1| integral membrane protein [Caulobacter crescentus CB15]
>gi|16127271|ref|NP_421835.1| FliJ protein [Caulobacter crescentus CB15]
>gi|16127276|ref|NP_421840.1| hypothetical protein CC3046 [Caulobacter crescentus CB15]
>gi|16127280|ref|NP_421844.1| hypothetical protein CC3050 [Caulobacter crescentus CB15]
>gi|16127285|ref|NP_421849.1| hypothetical protein CC3055 [Caulobacter crescentus CB15]
>gi|16127294|ref|NP_421858.1| hypothetical protein CC3064 [Caulobacter crescentus CB15]
>gi|16127299|ref|NP_421863.1| major facilitator family transporter [Caulobacter crescentus CB15]
>gi|16127303|ref|NP_421867.1| hypothetical protein CC3073 [Caulobacter crescentus CB15]
>gi|16127330|ref|NP_421894.1| response regulator [Caulobacter crescentus CB15]
>gi|16127332|ref|NP_421896.1| sensor histidine kinase/response regulator [Caulobacter crescentus CB15]
>gi|16127349|ref|NP_421913.1| efflux system protein, putative [Caulobacter crescentus CB15]
>gi|16127353|ref|NP_421917.1| transcriptional regulator, TetR family [Caulobacter crescentus CB15]
>gi|16127372|ref|NP_421936.1| transcriptional regulator, GntR family [Caulobacter crescentus CB15]
>gi|16127381|ref|NP_421945.1| hypothetical protein CC3151 [Caulobacter crescentus CB15]
>gi|16127386|ref|NP_421950.1| hypothetical protein CC3156 [Caulobacter crescentus CB15]
>gi|16127412|ref|NP_421976.1| hypothetical protein CC3182 [Caulobacter crescentus CB15]
>gi|16127413|ref|NP_421977.1| hypothetical protein CC3183 [Caulobacter crescentus CB15]
>gi|16127417|ref|NP_421981.1| hypothetical protein CC3187 [Caulobacter crescentus CB15]
>gi|16127425|ref|NP_421989.1| efflux system protein [Caulobacter crescentus CB15]
>gi|16127433|ref|NP_421997.1| hypothetical protein CC3203 [Caulobacter crescentus CB15]
>gi|16127450|ref|NP_422014.1| hypothetical protein CC3220 [Caulobacter crescentus CB15]
>gi|16127457|ref|NP_422021.1| hypothetical protein CC3227 [Caulobacter crescentus CB15]
>gi|16127458|ref|NP_422022.1| hypothetical protein CC3228 [Caulobacter crescentus CB15]
>gi|16127459|ref|NP_422023.1| OmpA family protein [Caulobacter crescentus CB15]
>gi|16127461|ref|NP_422025.1| hypothetical protein CC3231 [Caulobacter crescentus CB15]
>gi|16127465|ref|NP_422029.1| hypothetical protein CC3235 [Caulobacter crescentus CB15]
>gi|16127482|ref|NP_422046.1| hypothetical protein CC3252 [Caulobacter crescentus CB15]
>gi|16127484|ref|NP_422048.1| hypothetical protein CC3254 [Caulobacter crescentus CB15]
>gi|16127486|ref|NP_422050.1| hypothetical protein CC3256 [Caulobacter crescentus CB15]
>gi|16127490|ref|NP_422054.1| hypothetical protein CC3260 [Caulobacter crescentus CB15]
>gi|16127497|ref|NP_422061.1| hypothetical protein CC3267 [Caulobacter crescentus CB15]
>gi|16127521|ref|NP_422085.1| hypothetical protein CC3291 [Caulobacter crescentus CB15]
>gi|16127526|ref|NP_422090.1| hypothetical protein CC3296 [Caulobacter crescentus CB15]
>gi|16127527|ref|NP_422091.1| ribosomal protein S21 [Caulobacter crescentus CB15]
>gi|16127528|ref|NP_422092.1| hypothetical protein CC3298 [Caulobacter crescentus CB15]
>gi|16127532|ref|NP_422096.1| hypothetical protein CC3302 [Caulobacter crescentus CB15]
>gi|16127534|ref|NP_422098.1| NAD(P) transhydrogenase, alpha2 subunit [Caulobacter crescentus CB15]
>gi|16127535|ref|NP_422099.1| NAD(P) transhydrogenase, alpha subunit [Caulobacter crescentus CB15]
>gi|16127536|ref|NP_422100.1| hypothetical protein CC3306 [Caulobacter crescentus CB15]
>gi|16127548|ref|NP_422112.1| hypothetical protein CC3318 [Caulobacter crescentus CB15]
>gi|16127556|ref|NP_422120.1| peptidoglycan binding domain protein [Caulobacter crescentus CB15]
>gi|16127565|ref|NP_422129.1| hypothetical protein CC3335 [Caulobacter crescentus CB15]
>gi|16127567|ref|NP_422131.1| peptidase, M20/M25/M40 family [Caulobacter crescentus CB15]
>gi|16127572|ref|NP_422136.1| hypothetical protein CC3342 [Caulobacter crescentus CB15]
>gi|16127578|ref|NP_422142.1| hypothetical protein CC3348 [Caulobacter crescentus CB15]
>gi|16127580|ref|NP_422144.1| hypothetical protein CC3350 [Caulobacter crescentus CB15]
>gi|16127581|ref|NP_422145.1| hypothetical protein CC3351 [Caulobacter crescentus CB15]
>gi|16127586|ref|NP_422150.1| hypothetical protein CC3356 [Caulobacter crescentus CB15]
>gi|16127588|ref|NP_422152.1| methyl-accepting chemotaxis protein McpL [Caulobacter crescentus CB15]
>gi|16127590|ref|NP_422154.1| hypothetical protein CC3360 [Caulobacter crescentus CB15]
>gi|16127605|ref|NP_422169.1| band 7/Mec-2 family protein [Caulobacter crescentus CB15]
>gi|16127608|ref|NP_422172.1| hypothetical protein CC3378 [Caulobacter crescentus CB15]
>gi|16127609|ref|NP_422173.1| hypothetical protein CC3379 [Caulobacter crescentus CB15]
>gi|16127621|ref|NP_422185.1| hypothetical protein CC3391 [Caulobacter crescentus CB15]
>gi|16127626|ref|NP_422190.1| GGDEF family protein [Caulobacter crescentus CB15]
>gi|16127627|ref|NP_422191.1| ribosomal-protein-alanine acetyltransferase, putative [Caulobacter crescentus CB15]
>gi|16127634|ref|NP_422198.1| hypothetical protein CC3404 [Caulobacter crescentus CB15]
>gi|16127640|ref|NP_422204.1| hypothetical protein CC3410 [Caulobacter crescentus CB15]
>gi|16127649|ref|NP_422213.1| hypothetical protein CC3419 [Caulobacter crescentus CB15]
>gi|16127650|ref|NP_422214.1| ABC transporter, HlyB/MsbA family [Caulobacter crescentus CB15]
>gi|16127669|ref|NP_422233.1| hypothetical protein CC3439 [Caulobacter crescentus CB15]
>gi|16127671|ref|NP_422235.1| arylesterase-related protein [Caulobacter crescentus CB15]
>gi|16127674|ref|NP_422238.1| hypothetical protein CC3444 [Caulobacter crescentus CB15]
>gi|16127681|ref|NP_422245.1| hypothetical protein CC3451 [Caulobacter crescentus CB15]
>gi|16127683|ref|NP_422247.1| hypothetical protein CC3453 [Caulobacter crescentus CB15]
>gi|16127686|ref|NP_422250.1| hypothetical protein CC3456 [Caulobacter crescentus CB15]

>gi|16127688|ref|NP_422252.1| hypothetical protein CC3458 [Caulobacter crescentus CB15]
>gi|16127709|ref|NP_422273.1| hypothetical protein CC3479 [Caulobacter crescentus CB15]
>gi|16127720|ref|NP_422284.1| hypothetical protein CC3490 [Caulobacter crescentus CB15]
>gi|16127723|ref|NP_422287.1| hypothetical protein CC3493 [Caulobacter crescentus CB15]
>gi|16127724|ref|NP_422288.1| OmpA family protein [Caulobacter crescentus CB15]
>gi|16127726|ref|NP_422290.1| TrkA family protein [Caulobacter crescentus CB15]
>gi|16127729|ref|NP_422293.1| hypothetical protein CC3499 [Caulobacter crescentus CB15]
>gi|16127735|ref|NP_422299.1| hypothetical protein CC3505 [Caulobacter crescentus CB15]
>gi|16127736|ref|NP_422300.1| transcriptional regulator, AraC family [Caulobacter crescentus CB15]
>gi|16127738|ref|NP_422302.1| hypothetical protein CC3508 [Caulobacter crescentus CB15]
>gi|16127739|ref|NP_422303.1| hypothetical protein CC3509 [Caulobacter crescentus CB15]
>gi|16127740|ref|NP_422304.1| transcriptional regulator, LysR family [Caulobacter crescentus CB15]
>gi|16127742|ref|NP_422306.1| hypothetical protein CC3512 [Caulobacter crescentus CB15]
>gi|16127752|ref|NP_422316.1| hypothetical protein CC3522 [Caulobacter crescentus CB15]
>gi|16127764|ref|NP_422328.1| hypothetical protein CC3534 [Caulobacter crescentus CB15]
>gi|16127767|ref|NP_422331.1| hypothetical protein CC3537 [Caulobacter crescentus CB15]
>gi|16127777|ref|NP_422341.1| hypothetical protein CC3547 [Caulobacter crescentus CB15]
>gi|16127780|ref|NP_422344.1| dihydrodipicolinate reductase [Caulobacter crescentus CB15]
>gi|16127781|ref|NP_422345.1| hypothetical protein CC3551 [Caulobacter crescentus CB15]
>gi|16127782|ref|NP_422346.1| hypothetical protein CC3552 [Caulobacter crescentus CB15]
>gi|16127784|ref|NP_422348.1| hypothetical protein CC3554 [Caulobacter crescentus CB15]
>gi|16127785|ref|NP_422349.1| hypothetical protein CC3555 [Caulobacter crescentus CB15]
>gi|16127788|ref|NP_422352.1| hypothetical protein CC3558 [Caulobacter crescentus CB15]
>gi|16127789|ref|NP_422353.1| hypothetical protein CC3559 [Caulobacter crescentus CB15]
>gi|16127793|ref|NP_422357.1| hypothetical protein CC3563 [Caulobacter crescentus CB15]
>gi|16127805|ref|NP_422369.1| hypothetical protein CC3575 [Caulobacter crescentus CB15]
>gi|16127818|ref|NP_422382.1| cytidylate kinase [Caulobacter crescentus CB15]
>gi|16127820|ref|NP_422384.1| hypothetical protein CC3590 [Caulobacter crescentus CB15]
>gi|16127826|ref|NP_422390.1| PTS system, nitrogen regulatory IIA component [Caulobacter crescentus CB15]
>gi|16127828|ref|NP_422392.1| hypothetical protein CC3598 [Caulobacter crescentus CB15]
>gi|16127838|ref|NP_422402.1| hypothetical protein CC3608 [Caulobacter crescentus CB15]
>gi|16127839|ref|NP_422403.1| hypothetical protein CC3609 [Caulobacter crescentus CB15]
>gi|16127845|ref|NP_422409.1| dTDP-4-dehydrorhamnose reductase [Caulobacter crescentus CB15]
>gi|16127857|ref|NP_422421.1| hypothetical protein CC3627 [Caulobacter crescentus CB15]
>gi|16127871|ref|NP_422435.1| cation efflux family protein [Caulobacter crescentus CB15]
>gi|16127873|ref|NP_422437.1| citrate synthase [Caulobacter crescentus CB15]
>gi|16127882|ref|NP_422446.1| ribosomal protein S16 [Caulobacter crescentus CB15]
>gi|16127888|ref|NP_422452.1| hypothetical protein CC3658 [Caulobacter crescentus CB15]
>gi|16127898|ref|NP_422462.1| heme exporter protein A [Caulobacter crescentus CB15]
>gi|16127899|ref|NP_422463.1| heme exporter protein B [Caulobacter crescentus CB15]
>gi|16127900|ref|NP_422464.1| integral membrane protein [Caulobacter crescentus CB15]
>gi|16127901|ref|NP_422465.1| hypothetical protein CC3671 [Caulobacter crescentus CB15]
>gi|16127906|ref|NP_422470.1| hypothetical protein CC3676 [Caulobacter crescentus CB15]
>gi|16127910|ref|NP_422474.1| YghA protein [Caulobacter crescentus CB15]
>gi|16127911|ref|NP_422475.1| hypothetical protein CC3681 [Caulobacter crescentus CB15]
>gi|16127915|ref|NP_422479.1| oxidoreductase, short-chain dehydrogenase/reductase family [Caulobacter crescentus CB15]
>gi|16127923|ref|NP_422487.1| hypothetical protein CC3693 [Caulobacter crescentus CB15]
>gi|16127928|ref|NP_422492.1| alkyl hydroperoxide reductase D [Caulobacter crescentus CB15]
>gi|16127929|ref|NP_422493.1| hypothetical protein CC3699 [Caulobacter crescentus CB15]
>gi|16127930|ref|NP_422494.1| hypothetical protein CC3700 [Caulobacter crescentus CB15]
>gi|16127944|ref|NP_422508.1| HlyD family secretion protein [Caulobacter crescentus CB15]
>gi|16127948|ref|NP_422512.1| TPR domain protein [Caulobacter crescentus CB15]
>gi|16127960|ref|NP_422524.1| hypothetical protein CC3730 [Caulobacter crescentus CB15]
>gi|16127968|ref|NP_422532.1| phosphoribosyl-ATP pyrophosphatase [Caulobacter crescentus CB15]
>gi|15899975|ref|NP_344579.1| hypothetical protein SP0029 [Streptococcus pneumoniae TIGR4]
>gi|15899986|ref|NP_344590.1| bacteriocin BlpU [Streptococcus pneumoniae TIGR4]
>gi|15900014|ref|NP_344618.1| choline binding protein I [Streptococcus pneumoniae TIGR4]
>gi|15900040|ref|NP_344644.1| hypothetical protein SP0097 [Streptococcus pneumoniae TIGR4]
>gi|15900042|ref|NP_344646.1| hypothetical protein SP0099 [Streptococcus pneumoniae TIGR4]
>gi|15900044|ref|NP_344648.1| transporter, putative [Streptococcus pneumoniae TIGR4]
>gi|15900050|ref|NP_344654.1| LysM domain protein [Streptococcus pneumoniae TIGR4]
>gi|15900059|ref|NP_344663.1| pneumococcal surface protein A [Streptococcus pneumoniae TIGR4]
>gi|15900092|ref|NP_344696.1| hypothetical protein SP0154 [Streptococcus pneumoniae TIGR4]
>gi|15900134|ref|NP_344738.1| hypothetical protein SP0198 [Streptococcus pneumoniae TIGR4]
>gi|15900153|ref|NP_344757.1| ribosomal protein L29 [Streptococcus pneumoniae TIGR4]
>gi|15900165|ref|NP_344769.1| ribosomal protein L15 [Streptococcus pneumoniae TIGR4]
>gi|15900297|ref|NP_344901.1| hypothetical protein SP0374 [Streptococcus pneumoniae TIGR4]
>gi|15900308|ref|NP_344912.1| hypothetical protein SP0385 [Streptococcus pneumoniae TIGR4]
>gi|15900324|ref|NP_344928.1| hypothetical protein SP0405 [Streptococcus pneumoniae TIGR4]
>gi|15900342|ref|NP_344946.1| acetyl-CoA carboxylase, bitoin carboxyl carrier protein [Streptococcus pneumoniae TIGR4]

>gi|15900402|ref|NP_345006.1| hypothetical protein SP0488 [Streptococcus pneumoniae TIGR4]
>gi|15900407|ref|NP_345011.1| DNA-directed RNA polymerase, delta subunit, putative [Streptococcus pneumoniae TIGR4]
>gi|15900437|ref|NP_345041.1| ABC transporter, permease protein, putative [Streptococcus pneumoniae TIGR4]
>gi|15900444|ref|NP_345048.1| bacteriocin BlpI [Streptococcus pneumoniae TIGR4]
>gi|15900445|ref|NP_345049.1| bacteriocin BlpJ [Streptococcus pneumoniae TIGR4]
>gi|15900446|ref|NP_345050.1| bacteriocin BlpK [Streptococcus pneumoniae TIGR4]
>gi|15900452|ref|NP_345056.1| bacteriocin BlpM [Streptococcus pneumoniae TIGR4]
>gi|15900453|ref|NP_345057.1| BlpN protein [Streptococcus pneumoniae TIGR4]
>gi|15900468|ref|NP_345072.1| translation initiation factor IF-2 [Streptococcus pneumoniae TIGR4]
>gi|15900476|ref|NP_345080.1| hypothetical protein SP0564 [Streptococcus pneumoniae TIGR4]
>gi|15900554|ref|NP_345158.1| hypothetical protein SP0653 [Streptococcus pneumoniae TIGR4]
>gi|15900556|ref|NP_345160.1| sodium/hydrogen exchanger family protein [Streptococcus pneumoniae TIGR4]
>gi|15900558|ref|NP_345162.1| ribonuclease BN, putative [Streptococcus pneumoniae TIGR4]
>gi|15900559|ref|NP_345163.1| cytochrome c-type biogenesis protein CcdA [Streptococcus pneumoniae TIGR4]
>gi|15900571|ref|NP_345175.1| hypothetical protein SP0670 [Streptococcus pneumoniae TIGR4]
>gi|15900583|ref|NP_345187.1| hypothetical protein SP0682 [Streptococcus pneumoniae TIGR4]
>gi|15900591|ref|NP_345195.1| cell division protein DivIB [Streptococcus pneumoniae TIGR4]
>gi|15900597|ref|NP_345201.1| hypothetical protein SP0696 [Streptococcus pneumoniae TIGR4]
>gi|15900646|ref|NP_345250.1| branched-chain amino acid ABC transporter, permease protein [Streptococcus pneumoniae TIGR4]
>gi|15900693|ref|NP_345297.1| hypothetical protein SP0800 [Streptococcus pneumoniae TIGR4]
>gi|15900719|ref|NP_345323.1| hypothetical protein SP0832 [Streptococcus pneumoniae TIGR4]
>gi|15900762|ref|NP_345366.1| hypothetical protein SP0879 [Streptococcus pneumoniae TIGR4]
>gi|15900867|ref|NP_345471.1| hypothetical protein SP0992 [Streptococcus pneumoniae TIGR4]
>gi|15900872|ref|NP_345476.1| cytochrome c-type biogenesis protein CcdA [Streptococcus pneumoniae TIGR4]
>gi|15900972|ref|NP_345576.1| ribosomal protein L21 [Streptococcus pneumoniae TIGR4]
>gi|15900978|ref|NP_345582.1| hypothetical protein SP1111 [Streptococcus pneumoniae TIGR4]
>gi|15900980|ref|NP_345584.1| DNA-binding protein HU [Streptococcus pneumoniae TIGR4]
>gi|15901012|ref|NP_345616.1| hypothetical protein SP1146 [Streptococcus pneumoniae TIGR4]
>gi|15901051|ref|NP_345655.1| PTS system, lactose-specific IIA component [Streptococcus pneumoniae TIGR4]
>gi|15901105|ref|NP_345709.1| signal recognition particle-docking protein FtsY [Streptococcus pneumoniae TIGR4]
>gi|15901155|ref|NP_345759.1| crcB protein [Streptococcus pneumoniae TIGR4]
>gi|15901166|ref|NP_345770.1| hypothetical protein SP1307 [Streptococcus pneumoniae TIGR4]
>gi|15901208|ref|NP_345812.1| ribosomal protein L7/L12 [Streptococcus pneumoniae TIGR4]
>gi|15901268|ref|NP_345872.1| ribosomal protein S21 [Streptococcus pneumoniae TIGR4]
>gi|15901277|ref|NP_345881.1| hypothetical protein SP1424 [Streptococcus pneumoniae TIGR4]
>gi|15901284|ref|NP_345888.1| hypothetical protein SP1432 [Streptococcus pneumoniae TIGR4]
>gi|15901340|ref|NP_345944.1| cell wall surface anchor family protein [Streptococcus pneumoniae TIGR4]
>gi|15901354|ref|NP_345958.1| ATP synthase F1, epsilon subunit [Streptococcus pneumoniae TIGR4]
>gi|15901376|ref|NP_345980.1| hypothetical protein SP1531 [Streptococcus pneumoniae TIGR4]
>gi|15901444|ref|NP_346048.1| hypothetical protein SP1604 [Streptococcus pneumoniae TIGR4]
>gi|15901464|ref|NP_346068.1| hypothetical protein SP1628 [Streptococcus pneumoniae TIGR4]
>gi|15901477|ref|NP_346081.1| hypothetical protein SP1641 [Streptococcus pneumoniae TIGR4]
>gi|15901493|ref|NP_346097.1| hypothetical protein SP1658 [Streptococcus pneumoniae TIGR4]
>gi|15901528|ref|NP_346132.1| hypothetical protein SP1694 [Streptococcus pneumoniae TIGR4]
>gi|15901569|ref|NP_346173.1| DNA-directed RNA polymerase, omega subunit, putative [Streptococcus pneumoniae TIGR4]
>gi|15901602|ref|NP_346206.1| cell wall surface anchor family protein [Streptococcus pneumoniae TIGR4]
>gi|15901634|ref|NP_346238.1| hypothetical protein SP1805 [Streptococcus pneumoniae TIGR4]
>gi|15901637|ref|NP_346241.1| type IV prepilin peptidase, putative [Streptococcus pneumoniae TIGR4]
>gi|15901659|ref|NP_346263.1| phosphate transport system regulatory protein PhoU, putative [Streptococcus pneumoniae TIGR4]
>gi|15901684|ref|NP_346288.1| transcriptional regulator, MerR family [Streptococcus pneumoniae TIGR4]
>gi|15901815|ref|NP_346419.1| cell wall surface anchor family protein [Streptococcus pneumoniae TIGR4]
>gi|15901845|ref|NP_346449.1| PTS system, IIA component [Streptococcus pneumoniae TIGR4]
>gi|15901908|ref|NP_346512.1| hypothetical protein SP2093 [Streptococcus pneumoniae TIGR4]
>gi|15901912|ref|NP_346516.1| 2,3,4,5-tetrahydropyridine-2-carboxylate N-succinyltransferase, putative [Streptococcus pneumoniae TIGR4]
>gi|15901917|ref|NP_346521.1| hypothetical protein SP2102 [Streptococcus pneumoniae TIGR4]
>gi|15901950|ref|NP_346554.1| choline binding protein PcpA [Streptococcus pneumoniae TIGR4]
>gi|15901997|ref|NP_346601.1| choline binding protein A [Streptococcus pneumoniae TIGR4]
>gi|15901998|ref|NP_346602.1| hypothetical protein SP2191 [Streptococcus pneumoniae TIGR4]
>gi|15902020|ref|NP_346624.1| secreted 45 kd protein [Streptococcus pneumoniae TIGR4]
>gi|15902021|ref|NP_346625.1| rod shpae-determining protein MreD, putative [Streptococcus pneumoniae TIGR4]
>gi|15902027|ref|NP_346631.1| hypothetical protein SP2223 [Streptococcus pneumoniae TIGR4]
>gi|58038257|ref|YP_190226.1| hypothetical protein GOX2504 [Gluconobacter oxydans 621H]
>gi|58038268|ref|YP_190237.1| hypothetical protein GOX2515 [Gluconobacter oxydans 621H]
>gi|58038352|ref|YP_190321.1| Hypothetical membrane protein [Gluconobacter oxydans 621H]
>gi|58038353|ref|YP_190322.1| Hypothetical membrane protein [Gluconobacter oxydans 621H]
>gi|58038370|ref|YP_190339.1| hypothetical protein GOX2619 [Gluconobacter oxydans 621H]
>gi|58038376|ref|YP_190345.1| PilN [Gluconobacter oxydans 621H]
>gi|58038377|ref|YP_190346.1| hypothetical protein GOX2626 [Gluconobacter oxydans 621H]
>gi|58038490|ref|YP_190455.1| PemK-like protein [Gluconobacter oxydans 621H]
>gi|58038519|ref|YP_190483.1| hypothetical protein GOX0029 [Gluconobacter oxydans 621H]

>gi|58038525|ref|YP_190489.1| hypothetical protein GOX0035 [Gluconobacter oxydans 621H]
>gi|58038540|ref|YP_190504.1| Cobalamin biosynthesis protein CobT [Gluconobacter oxydans 621H]
>gi|58038548|ref|YP_190512.1| Putative transcriptional regulator cold shock protein [Gluconobacter oxydans 621H]
>gi|58038579|ref|YP_190543.1| ATP-dependent RNA helicase [Gluconobacter oxydans 621H]
>gi|58038599|ref|YP_190563.1| hypothetical protein GOX0113 [Gluconobacter oxydans 621H]
>gi|58038601|ref|YP_190565.1| 3-Oxoacyl-[acyl-carrier-protein] synthase III [Gluconobacter oxydans 621H]
>gi|58038610|ref|YP_190574.1| SSU ribosomal protein S21P [Gluconobacter oxydans 621H]
>gi|58038624|ref|YP_190588.1| LSU ribosomal protein L21P [Gluconobacter oxydans 621H]
>gi|58038636|ref|YP_190600.1| hypothetical protein GOX0151 [Gluconobacter oxydans 621H]
>gi|58038645|ref|YP_190609.1| UDP-N-acetylenolpyruvoylglucosamine reductase [Gluconobacter oxydans 621H]
>gi|58038649|ref|YP_190613.1| Cell division protein FtsZ [Gluconobacter oxydans 621H]
>gi|58038652|ref|YP_190616.1| DNA repair protein RecN [Gluconobacter oxydans 621H]
>gi|58038664|ref|YP_190628.1| hypothetical protein GOX0182 [Gluconobacter oxydans 621H]
>gi|58038676|ref|YP_190640.1| LSU ribosomal protein L19P [Gluconobacter oxydans 621H]
>gi|58038706|ref|YP_190670.1| hypothetical protein GOX0231 [Gluconobacter oxydans 621H]
>gi|58038713|ref|YP_190677.1| hypothetical protein GOX0238 [Gluconobacter oxydans 621H]
>gi|58038726|ref|YP_190690.1| Dimethylallyltransferase [Gluconobacter oxydans 621H]
>gi|58038747|ref|YP_190711.1| hypothetical protein GOX0272 [Gluconobacter oxydans 621H]
>gi|58038762|ref|YP_190726.1| hypothetical protein GOX0287 [Gluconobacter oxydans 621H]
>gi|58038763|ref|YP_190727.1| hypothetical protein GOX0288 [Gluconobacter oxydans 621H]
>gi|58038764|ref|YP_190728.1| hypothetical protein GOX0289 [Gluconobacter oxydans 621H]
>gi|58038779|ref|YP_190743.1| LSU ribosomal protein L9P [Gluconobacter oxydans 621H]
>gi|58038786|ref|YP_190750.1| NAD(P) transhydrogenase subunit alpha [Gluconobacter oxydans 621H]
>gi|58038791|ref|YP_190755.1| hypothetical protein GOX0316 [Gluconobacter oxydans 621H]
>gi|58038806|ref|YP_190770.1| hypothetical protein GOX0331 [Gluconobacter oxydans 621H]
>gi|58038818|ref|YP_190782.1| hypothetical protein GOX0343 [Gluconobacter oxydans 621H]
>gi|58038827|ref|YP_190791.1| hypothetical protein GOX0352 [Gluconobacter oxydans 621H]
>gi|58038844|ref|YP_190808.1| LSU ribosomal protein L24P [Gluconobacter oxydans 621H]
>gi|58038862|ref|YP_190826.1| LSU ribosomal protein L12P (L7/L12) [Gluconobacter oxydans 621H]
>gi|58038900|ref|YP_190864.1| Basal-body rod modification protein FlgD [Gluconobacter oxydans 621H]
>gi|58038911|ref|YP_190875.1| Biotin carboxyl carrier protein of acetyl-CoA carboxylase [Gluconobacter oxydans 621H]
>gi|58038934|ref|YP_190898.1| hypothetical protein GOX0460 [Gluconobacter oxydans 621H]
>gi|58038940|ref|YP_190904.1| Nitrogen assimilation regulatory protein NtrC [Gluconobacter oxydans 621H]
>gi|58038966|ref|YP_190930.1| hypothetical protein GOX0495 [Gluconobacter oxydans 621H]
>gi|58038989|ref|YP_190953.1| Hypothetical outer membrane protein [Gluconobacter oxydans 621H]
>gi|58039000|ref|YP_190964.1| TonB protein [Gluconobacter oxydans 621H]
>gi|58039053|ref|YP_191017.1| ABC-type Fe3+-siderophore transport system, permease component [Gluconobacter oxydans 621H]
>gi|58039060|ref|YP_191024.1| hypothetical protein GOX0590 [Gluconobacter oxydans 621H]
>gi|58039069|ref|YP_191033.1| hypothetical protein GOX0599 [Gluconobacter oxydans 621H]
>gi|58039074|ref|YP_191038.1| DNA polymerase III subunit gamma/tau [Gluconobacter oxydans 621H]
>gi|58039086|ref|YP_191050.1| hypothetical protein GOX0616 [Gluconobacter oxydans 621H]
>gi|58039089|ref|YP_191053.1| hypothetical protein GOX0619 [Gluconobacter oxydans 621H]
>gi|58039093|ref|YP_191057.1| hypothetical protein GOX0624 [Gluconobacter oxydans 621H]
>gi|58039104|ref|YP_191068.1| hypothetical protein GOX0635 [Gluconobacter oxydans 621H]
>gi|58039163|ref|YP_191127.1| hypothetical protein GOX0694 [Gluconobacter oxydans 621H]
>gi|58039180|ref|YP_191144.1| hypothetical protein GOX0711 [Gluconobacter oxydans 621H]
>gi|58039185|ref|YP_191149.1| Putative oxidoreductase [Gluconobacter oxydans 621H]
>gi|58039211|ref|YP_191175.1| hypothetical protein GOX0742 [Gluconobacter oxydans 621H]
>gi|58039221|ref|YP_191185.1| Putative acetyltransferase [Gluconobacter oxydans 621H]
>gi|58039234|ref|YP_191198.1| Transcriptional regulator [Gluconobacter oxydans 621H]
>gi|58039253|ref|YP_191217.1| Flagellin B [Gluconobacter oxydans 621H]
>gi|58039271|ref|YP_191235.1| hypothetical protein GOX0805 [Gluconobacter oxydans 621H]
>gi|58039304|ref|YP_191268.1| hypothetical protein GOX0838 [Gluconobacter oxydans 621H]
>gi|58039305|ref|YP_191269.1| hypothetical protein GOX0839 [Gluconobacter oxydans 621H]
>gi|58039312|ref|YP_191276.1| Probable MFS family transport protein [Gluconobacter oxydans 621H]
>gi|58039324|ref|YP_191288.1| Chaperone protein DnaJ [Gluconobacter oxydans 621H]
>gi|58039355|ref|YP_191319.1| Single-strand DNA binding protein [Gluconobacter oxydans 621H]
>gi|58039378|ref|YP_191342.1| Putative transport protein [Gluconobacter oxydans 621H]
>gi|58039379|ref|YP_191343.1| hypothetical protein GOX0915 [Gluconobacter oxydans 621H]
>gi|58039386|ref|YP_191350.1| hypothetical protein GOX0922 [Gluconobacter oxydans 621H]
>gi|58039397|ref|YP_191361.1| hypothetical protein GOX0933 [Gluconobacter oxydans 621H]
>gi|58039421|ref|YP_191385.1| Putative chloride channel protein [Gluconobacter oxydans 621H]
>gi|58039426|ref|YP_191390.1| hypothetical protein GOX0962 [Gluconobacter oxydans 621H]
>gi|58039464|ref|YP_191428.1| hypothetical protein GOX1000 [Gluconobacter oxydans 621H]
>gi|58039471|ref|YP_191435.1| hypothetical protein GOX1008 [Gluconobacter oxydans 621H]
>gi|58039475|ref|YP_191439.1| Ferrichrome ABC transporter ATP-binding protein [Gluconobacter oxydans 621H]
>gi|58039476|ref|YP_191440.1| Ferrichrome ABC transporter permease protein [Gluconobacter oxydans 621H]
>gi|58039488|ref|YP_191452.1| Flagellar hook-associated protein 3 FlgL [Gluconobacter oxydans 621H]
>gi|58039489|ref|YP_191453.1| Flagellar hook-associated protein 1 FlgK [Gluconobacter oxydans 621H]
>gi|58039490|ref|YP_191454.1| Flagellar hook protein FlgE [Gluconobacter oxydans 621H]

>gi|58039507|ref|YP_191471.1| Metal ABC transport system ATP-binding protein [Gluconobacter oxydans 621H]
>gi|58039508|ref|YP_191472.1| Metal ABC transport system permease protein [Gluconobacter oxydans 621H]
>gi|58039530|ref|YP_191494.1| O6-methylguanine-DNA methyltransferase [Gluconobacter oxydans 621H]
>gi|58039534|ref|YP_191498.1| Dihydrolipoamide succinyl transferase (E2) of 2-oxoglutarate dehydrogenase [Gluconobacter oxydans 621H]
>gi|58039538|ref|YP_191502.1| hypothetical protein GOX1077 [Gluconobacter oxydans 621H]
>gi|58039540|ref|YP_191504.1| hypothetical protein GOX1079 [Gluconobacter oxydans 621H]
>gi|58039559|ref|YP_191523.1| hypothetical protein GOX1098 [Gluconobacter oxydans 621H]
>gi|58039563|ref|YP_191527.1| hypothetical protein GOX1102 [Gluconobacter oxydans 621H]
>gi|58039570|ref|YP_191534.1| ATP synthase B' chain [Gluconobacter oxydans 621H]
>gi|58039571|ref|YP_191535.1| ATP synthase B' chain [Gluconobacter oxydans 621H]
>gi|58039574|ref|YP_191538.1| Vitamin B12-dependent ribonucleotide reductase [Gluconobacter oxydans 621H]
>gi|58039577|ref|YP_191541.1| hypothetical protein GOX1117 [Gluconobacter oxydans 621H]
>gi|58039584|ref|YP_191548.1| hypothetical protein GOX1124 [Gluconobacter oxydans 621H]
>gi|58039656|ref|YP_191620.1| hypothetical protein GOX1203 [Gluconobacter oxydans 621H]
>gi|58039670|ref|YP_191634.1| hypothetical protein GOX1217 [Gluconobacter oxydans 621H]
>gi|58039675|ref|YP_191639.1| hypothetical protein GOX1222 [Gluconobacter oxydans 621H]
>gi|58039677|ref|YP_191641.1| Phage-related baseplate assembly protein [Gluconobacter oxydans 621H]
>gi|58039682|ref|YP_191646.1| hypothetical protein GOX1229 [Gluconobacter oxydans 621H]
>gi|58039739|ref|YP_191703.1| hypothetical protein GOX1286 [Gluconobacter oxydans 621H]
>gi|58039740|ref|YP_191704.1| Biopolymer transport ExbB protein [Gluconobacter oxydans 621H]
>gi|58039778|ref|YP_191742.1| Amino acid efflux protein [Gluconobacter oxydans 621H]
>gi|58039805|ref|YP_191769.1| Two component response regulator [Gluconobacter oxydans 621H]
>gi|58039821|ref|YP_191785.1| Na+/H+ Antiporter NhaA [Gluconobacter oxydans 621H]
>gi|58039841|ref|YP_191805.1| hypothetical protein GOX1397 [Gluconobacter oxydans 621H]
>gi|58039850|ref|YP_191814.1| hypothetical protein GOX1407 [Gluconobacter oxydans 621H]
>gi|58039857|ref|YP_191821.1| Chaperone protein DnaJ [Gluconobacter oxydans 621H]
>gi|58039864|ref|YP_191828.1| Murein transglycosylase [Gluconobacter oxydans 621H]
>gi|58039876|ref|YP_191840.1| hypothetical protein GOX1434 [Gluconobacter oxydans 621H]
>gi|58039884|ref|YP_191848.1| hypothetical protein GOX1442 [Gluconobacter oxydans 621H]
>gi|58039953|ref|YP_191917.1| hypothetical protein GOX1519 [Gluconobacter oxydans 621H]
>gi|58039960|ref|YP_191924.1| Flagellar hook-basal body protein FleE [Gluconobacter oxydans 621H]
>gi|58039965|ref|YP_191929.1| Flagellar biosynthetic protein FliP [Gluconobacter oxydans 621H]
>gi|58039966|ref|YP_191930.1| Gamma-glutamyltranspeptidase [Gluconobacter oxydans 621H]
>gi|58039994|ref|YP_191958.1| hypothetical protein GOX1561 [Gluconobacter oxydans 621H]
>gi|58039997|ref|YP_191961.1| hypothetical protein GOX1564 [Gluconobacter oxydans 621H]
>gi|58040003|ref|YP_191967.1| Heme exporter protein B [Gluconobacter oxydans 621H]
>gi|58040004|ref|YP_191968.1| Heme exporter protein A [Gluconobacter oxydans 621H]
>gi|58040011|ref|YP_191975.1| hypothetical protein GOX1578 [Gluconobacter oxydans 621H]
>gi|58040015|ref|YP_191979.1| Bacterial Protein Translation Initiation Factor 2 (IF2) [Gluconobacter oxydans 621H]
>gi|58040024|ref|YP_191988.1| hypothetical protein GOX1591 [Gluconobacter oxydans 621H]
>gi|58040047|ref|YP_192011.1| Putative hexosyltransferase [Gluconobacter oxydans 621H]
>gi|58040091|ref|YP_192055.1| hypothetical protein GOX1658 [Gluconobacter oxydans 621H]
>gi|58040093|ref|YP_192057.1| hypothetical protein GOX1660 [Gluconobacter oxydans 621H]
>gi|58040100|ref|YP_192064.1| hypothetical protein GOX1667 [Gluconobacter oxydans 621H]
>gi|58040118|ref|YP_192082.1| hypothetical protein GOX1686 [Gluconobacter oxydans 621H]
>gi|58040121|ref|YP_192085.1| hypothetical protein GOX1689 [Gluconobacter oxydans 621H]
>gi|58040127|ref|YP_192091.1| hypothetical protein GOX1695 [Gluconobacter oxydans 621H]
>gi|58040162|ref|YP_192126.1| Ribonuclease E [Gluconobacter oxydans 621H]
>gi|58040165|ref|YP_192129.1| Hypothetical transmembrane protein [Gluconobacter oxydans 621H]
>gi|58040166|ref|YP_192130.1| hypothetical protein GOX1735 [Gluconobacter oxydans 621H]
>gi|58040167|ref|YP_192131.1| hypothetical protein GOX1736 [Gluconobacter oxydans 621H]
>gi|58040176|ref|YP_192140.1| hypothetical protein GOX1745 [Gluconobacter oxydans 621H]
>gi|58040180|ref|YP_192144.1| hypothetical protein GOX1749 [Gluconobacter oxydans 621H]
>gi|58040185|ref|YP_192149.1| hypothetical protein GOX1754 [Gluconobacter oxydans 621H]
>gi|58040198|ref|YP_192162.1| hypothetical protein GOX1767 [Gluconobacter oxydans 621H]
>gi|58040220|ref|YP_192184.1| hypothetical protein GOX1789 [Gluconobacter oxydans 621H]
>gi|58040237|ref|YP_192201.1| hypothetical protein GOX1806 [Gluconobacter oxydans 621H]
>gi|58040257|ref|YP_192221.1| Ribonuclease P protein component [Gluconobacter oxydans 621H]
>gi|58040264|ref|YP_192228.1| hypothetical protein GOX1833 [Gluconobacter oxydans 621H]
>gi|58040288|ref|YP_192252.1| hypothetical protein GOX1858 [Gluconobacter oxydans 621H]
>gi|58040295|ref|YP_192259.1| Putative transmembrane protein [Gluconobacter oxydans 621H]
>gi|58040306|ref|YP_192270.1| hypothetical protein GOX1876 [Gluconobacter oxydans 621H]
>gi|58040310|ref|YP_192274.1| Glycerol-3-phosphate dehydrogenase [NAD(P)+] [Gluconobacter oxydans 621H]
>gi|58040314|ref|YP_192278.1| Putative uroporphyrinogen-III synthase [Gluconobacter oxydans 621H]
>gi|58040354|ref|YP_192318.1| Putative permease [Gluconobacter oxydans 621H]
>gi|58040363|ref|YP_192327.1| Phosphoribosylglycinamide formyltransferase protein [Gluconobacter oxydans 621H]
>gi|58040369|ref|YP_192333.1| Putative transmembrane efflux protein [Gluconobacter oxydans 621H]
>gi|58040372|ref|YP_192336.1| hypothetical protein GOX1942 [Gluconobacter oxydans 621H]
>gi|58040379|ref|YP_192343.1| hypothetical protein GOX1951 [Gluconobacter oxydans 621H]
>gi|58040387|ref|YP_192351.1| Shikimate 5-dehydrogenase [Gluconobacter oxydans 621H]

>gi|58040394|ref|YP_192358.1| hypothetical protein GOX1966 [Gluconobacter oxydans 621H]
>gi|58040403|ref|YP_192367.1| 4-Hydroxybenzoate octaprenyltransferase [Gluconobacter oxydans 621H]
>gi|58040409|ref|YP_192373.1| hypothetical protein GOX1981 [Gluconobacter oxydans 621H]
>gi|58040431|ref|YP_192395.1| Chromosome partition protein Smc [Gluconobacter oxydans 621H]
>gi|58040492|ref|YP_192456.1| hypothetical protein GOX2064 [Gluconobacter oxydans 621H]
>gi|58040507|ref|YP_192471.1| hypothetical protein GOX2079 [Gluconobacter oxydans 621H]
>gi|58040512|ref|YP_192476.1| Ribokinase [Gluconobacter oxydans 621H]
>gi|58040513|ref|YP_192477.1| hypothetical protein GOX2085 [Gluconobacter oxydans 621H]
>gi|58040514|ref|YP_192478.1| hypothetical protein GOX2086 [Gluconobacter oxydans 621H]
>gi|58040549|ref|YP_192513.1| Dipeptide ABC transport system permease protein DppC [Gluconobacter oxydans 621H]
>gi|58040550|ref|YP_192514.1| Dipeptide ABC transport system permease protein DppB [Gluconobacter oxydans 621H]
>gi|58040558|ref|YP_192522.1| hypothetical protein GOX2130 [Gluconobacter oxydans 621H]
>gi|58040583|ref|YP_192547.1| ABC transporter permease protein [Gluconobacter oxydans 621H]
>gi|58040584|ref|YP_192548.1| Putative ABC transporter permease protein [Gluconobacter oxydans 621H]
>gi|58040598|ref|YP_192562.1| Transmembrane protein [Gluconobacter oxydans 621H]
>gi|58040601|ref|YP_192565.1| ATP synthase subunit b [Gluconobacter oxydans 621H]
>gi|58040616|ref|YP_192580.1| Gluconate permease [Gluconobacter oxydans 621H]
>gi|58040648|ref|YP_192612.1| Ribose ABC transporter, permease protein [Gluconobacter oxydans 621H]
>gi|58040657|ref|YP_192621.1| Phosphomethylpyrimidine kinase ThiD [Gluconobacter oxydans 621H]
>gi|58040675|ref|YP_192639.1| Aspartyl-tRNA synthetase [Gluconobacter oxydans 621H]
>gi|58040720|ref|YP_192684.1| hypothetical protein GOX2295 [Gluconobacter oxydans 621H]
>gi|58040732|ref|YP_192696.1| hypothetical protein GOX2307 [Gluconobacter oxydans 621H]
>gi|58040765|ref|YP_192729.1| hypothetical protein GOX2341 [Gluconobacter oxydans 621H]
>gi|58040775|ref|YP_192739.1| Bacteriophage protein [Gluconobacter oxydans 621H]
>gi|58040783|ref|YP_192747.1| hypothetical protein GOX2359 [Gluconobacter oxydans 621H]
>gi|58040788|ref|YP_192752.1| hypothetical protein GOX2364 [Gluconobacter oxydans 621H]
>gi|58040814|ref|YP_192778.1| DNA repair protein RadC [Gluconobacter oxydans 621H]
>gi|58040818|ref|YP_192782.1| hypothetical protein GOX2395 [Gluconobacter oxydans 621H]
>gi|58040841|ref|YP_192805.1| Putative oxidoreductase [Gluconobacter oxydans 621H]
>gi|58040843|ref|YP_192807.1| hypothetical protein GOX2420 [Gluconobacter oxydans 621H]
>gi|58040845|ref|YP_192809.1| hypothetical protein GOX2422 [Gluconobacter oxydans 621H]
>gi|58040851|ref|YP_192815.1| hypothetical protein GOX2428 [Gluconobacter oxydans 621H]
>gi|58040858|ref|YP_192822.1| hypothetical protein GOX2435 [Gluconobacter oxydans 621H]
>gi|58040860|ref|YP_192824.1| hypothetical protein GOX2437 [Gluconobacter oxydans 621H]
>gi|58040861|ref|YP_192825.1| hypothetical protein GOX2438 [Gluconobacter oxydans 621H]
>gi|58040902|ref|YP_192866.1| hypothetical protein GOX2479 [Gluconobacter oxydans 621H]
>gi|58040910|ref|YP_192874.1| hypothetical protein GOX2488 [Gluconobacter oxydans 621H]
>gi|58040918|ref|YP_192882.1| Transport protein [Gluconobacter oxydans 621H]
>gi|27904515|ref|NP_777641.1| ATP synthase A chain [Buchnera aphidicola str. Bp (Baizongia pistaciae)]
>gi|27904517|ref|NP_777643.1| ATP synthase B chain [Buchnera aphidicola str. Bp (Baizongia pistaciae)]
>gi|27904553|ref|NP_777679.1| preprotein translocase SecE subunit [Buchnera aphidicola str. Bp (Baizongia pistaciae)]
>gi|27904578|ref|NP_777704.1| flagellar FliK protein [Buchnera aphidicola str. Bp (Baizongia pistaciae)]
>gi|27904583|ref|NP_777709.1| flagellar biosynthetic protein FliR [Buchnera aphidicola str. Bp (Baizongia pistaciae)]
>gi|27904619|ref|NP_777745.1| hypothetical protein bbp116 [Buchnera aphidicola str. Bp (Baizongia pistaciae)]
>gi|27904643|ref|NP_777769.1| 30S ribosomal protein S20 [Buchnera aphidicola str. Bp (Baizongia pistaciae)]
>gi|27904659|ref|NP_777785.1| putative transport protein [Buchnera aphidicola str. Bp (Baizongia pistaciae)]
>gi|27904661|ref|NP_777787.1| colicin V production-like protein [Buchnera aphidicola str. Bp (Baizongia pistaciae)]
>gi|27904694|ref|NP_777820.1| HesB/YadR/YfhF family protein [Buchnera aphidicola str. Bp (Baizongia pistaciae)]
>gi|27904706|ref|NP_777832.1| cell division protein FtsL homolog [Buchnera aphidicola str. Bp (Baizongia pistaciae)]
>gi|27904744|ref|NP_777870.1| putative membrane protein [Buchnera aphidicola str. Bp (Baizongia pistaciae)]
>gi|27904752|ref|NP_777878.1| hypothetical protein bbp256 [Buchnera aphidicola str. Bp (Baizongia pistaciae)]
>gi|27904806|ref|NP_777932.1| flagellar P-ring protein precursor [Buchnera aphidicola str. Bp (Baizongia pistaciae)]
>gi|27904837|ref|NP_777963.1| UDP-N-acetylglucosamine 1-carboxyvinyltransferase [Buchnera aphidicola str. Bp (Baizongia pistaciae)]
>gi|27904877|ref|NP_778003.1| DNA polymerase III delta subunit [Buchnera aphidicola str. Bp (Baizongia pistaciae)]
>gi|27904880|ref|NP_778006.1| putative membrane protein [Buchnera aphidicola str. Bp (Baizongia pistaciae)]
>gi|27904883|ref|NP_778009.1| putative membrane protein [Buchnera aphidicola str. Bp (Baizongia pistaciae)]
>gi|27904887|ref|NP_778013.1| 6,7-dimethyl-8-ribityllumazine synthase [Buchnera aphidicola str. Bp (Baizongia pistaciae)]
>gi|27904892|ref|NP_778018.1| protoheme IX farnesyltransferase [Buchnera aphidicola str. Bp (Baizongia pistaciae)]
>gi|27904894|ref|NP_778020.1| cytochrome O ubiquinol oxidase subunit III [Buchnera aphidicola str. Bp (Baizongia pistaciae)]
>gi|27904897|ref|NP_778023.1| BolA [Buchnera aphidicola str. Bp (Baizongia pistaciae)]
>gi|27904901|ref|NP_778027.1| peptidyl-prolyl cis-trans isomerase D [Buchnera aphidicola str. Bp (Baizongia pistaciae)]
>gi|27904923|ref|NP_778049.1| 50S ribosomal protein L15 [Buchnera aphidicola str. Bp (Baizongia pistaciae)]
>gi|27904931|ref|NP_778057.1| ribosomal protein L24 [Buchnera aphidicola str. Bp (Baizongia pistaciae)]
>gi|27904952|ref|NP_778078.1| putative transport [Buchnera aphidicola str. Bp (Baizongia pistaciae)]
>gi|27904985|ref|NP_778111.1| HflK [Buchnera aphidicola str. Bp (Baizongia pistaciae)]
>gi|27905013|ref|NP_778139.1| chaperone protein HscB [Buchnera aphidicola str. Bp (Baizongia pistaciae)]
>gi|14590018|ref|NP_142082.1| hypothetical protein PH0064 [Pyrococcus horikoshii OT3]
>gi|14590019|ref|NP_142083.1| hypothetical protein PH0065 [Pyrococcus horikoshii OT3]
>gi|33359250|ref|NP_877729.1| hypothetical protein PH0065.1n [Pyrococcus horikoshii OT3]
>gi|14590041|ref|NP_142105.1| hypothetical protein PH0089 [Pyrococcus horikoshii OT3]

>gi|33359257|ref|NP_877735.1| hypothetical protein PH0097.2n [Pyrococcus horikoshii OT3]
>gi|14590077|ref|NP_142141.1| hypothetical protein PH0134 [Pyrococcus horikoshii OT3]
>gi|33359259|ref|NP_877736.1| hypothetical protein PH0144.3n [Pyrococcus horikoshii OT3]
>gi|14590097|ref|NP_142161.1| hypothetical protein PH0159 [Pyrococcus horikoshii OT3]
>gi|14590109|ref|NP_142173.1| hypothetical protein PH0172 [Pyrococcus horikoshii OT3]
>gi|14590147|ref|NP_142212.1| hypothetical protein PH0214 [Pyrococcus horikoshii OT3]
>gi|33359265|ref|NP_877740.1| hypothetical protein PH0216.1n [Pyrococcus horikoshii OT3]
>gi|14590201|ref|NP_142266.1| hypothetical protein PH0277 [Pyrococcus horikoshii OT3]
>gi|14590202|ref|NP_142267.1| hypothetical protein PH0278 [Pyrococcus horikoshii OT3]
>gi|14590207|ref|NP_142272.1| hypothetical protein PH0283 [Pyrococcus horikoshii OT3]
>gi|33359271|ref|NP_877744.1| hypothetical protein PH0317.1n [Pyrococcus horikoshii OT3]
>gi|14590247|ref|NP_142313.1| hypothetical protein PH0332 [Pyrococcus horikoshii OT3]
>gi|14590257|ref|NP_142323.1| hypothetical protein PH0345 [Pyrococcus horikoshii OT3]
>gi|14590263|ref|NP_142329.1| hypothetical protein PH0353 [Pyrococcus horikoshii OT3]
>gi|14590265|ref|NP_142331.1| hypothetical protein PH0355 [Pyrococcus horikoshii OT3]
>gi|33359275|ref|NP_877747.1| hypothetical protein PH0358.1n [Pyrococcus horikoshii OT3]
>gi|14590286|ref|NP_142352.1| hypothetical protein PHS007 [Pyrococcus horikoshii OT3]
>gi|33359278|ref|NP_877750.1| hypothetical protein PH0381.1n [Pyrococcus horikoshii OT3]
>gi|33359281|ref|NP_877752.1| hypothetical protein PH0389.1n [Pyrococcus horikoshii OT3]
>gi|33359284|ref|NP_877755.1| hypothetical protein PHS010.2n [Pyrococcus horikoshii OT3]
>gi|14590323|ref|NP_142389.1| hypothetical protein PHS014 [Pyrococcus horikoshii OT3]
>gi|14590327|ref|NP_142393.1| hypothetical protein PHS017 [Pyrococcus horikoshii OT3]
>gi|14590356|ref|NP_142422.1| hypothetical protein PH0441 [Pyrococcus horikoshii OT3]
>gi|14590357|ref|NP_142423.1| hypothetical protein PH0442 [Pyrococcus horikoshii OT3]
>gi|14590367|ref|NP_142433.1| hypothetical protein PH0456 [Pyrococcus horikoshii OT3]
>gi|14590383|ref|NP_142449.1| membrane protein [Pyrococcus horikoshii OT3]
>gi|14590384|ref|NP_142450.1| hypothetical protein PH0471 [Pyrococcus horikoshii OT3]
>gi|14590387|ref|NP_142453.1| hypothetical protein PH0475 [Pyrococcus horikoshii OT3]
>gi|14590389|ref|NP_142455.1| hypothetical protein PH0477 [Pyrococcus horikoshii OT3]
>gi|14590392|ref|NP_142458.1| hypothetical protein PH0480 [Pyrococcus horikoshii OT3]
>gi|14590394|ref|NP_142460.1| chemotaxis protein (cheY) [Pyrococcus horikoshii OT3]
>gi|14590418|ref|NP_142484.1| hypothetical protein PH0516 [Pyrococcus horikoshii OT3]
>gi|33359300|ref|NP_142497.2| S ribosomal protein L31E [Pyrococcus horikoshii OT3]
>gi|14591742|ref|NP_142498.1| Ribosomal protein L39E [Pyrococcus horikoshii OT3]
>gi|14590444|ref|NP_142512.1| hypothetical protein PH0543 [Pyrococcus horikoshii OT3]
>gi|14590452|ref|NP_142520.1| hypothetical protein PH0552 [Pyrococcus horikoshii OT3]
>gi|14590453|ref|NP_142521.1| hypothetical protein PH0553 [Pyrococcus horikoshii OT3]
>gi|14590454|ref|NP_142522.1| hypothetical protein similar to diverse transmembrane protein set [Pyrococcus horikoshii OT3]
>gi|14590459|ref|NP_142527.1| hypothetical protein PH0560 [Pyrococcus horikoshii OT3]
>gi|14590495|ref|NP_142563.1| hypothetical protein PH0599 [Pyrococcus horikoshii OT3]
>gi|33359308|ref|NP_877772.1| hypothetical protein PH0604.1n [Pyrococcus horikoshii OT3]
>gi|14590509|ref|NP_142577.1| hypothetical protein PH0617 [Pyrococcus horikoshii OT3]
>gi|14590512|ref|NP_142580.1| hypothetical protein PH0620 [Pyrococcus horikoshii OT3]
>gi|14590530|ref|NP_142598.1| phosphate permease [Pyrococcus horikoshii OT3]
>gi|14590536|ref|NP_142604.1| hypothetical protein PH0646 [Pyrococcus horikoshii OT3]
>gi|14590537|ref|NP_142605.1| hypothetical protein PH0648 [Pyrococcus horikoshii OT3]
>gi|14590583|ref|NP_142651.1| hypothetical protein PH0705 [Pyrococcus horikoshii OT3]
>gi|14590620|ref|NP_142688.1| hypothetical protein PH0749 [Pyrococcus horikoshii OT3]
>gi|14590622|ref|NP_142690.1| hypothetical protein PH0752 [Pyrococcus horikoshii OT3]
>gi|14590628|ref|NP_142696.1| hypothetical protein PH0758 [Pyrococcus horikoshii OT3]
>gi|33359328|ref|NP_877791.1| hypothetical protein PH0771.1n [Pyrococcus horikoshii OT3]
>gi|14590642|ref|NP_142710.1| hypothetical protein PH0773 [Pyrococcus horikoshii OT3]
>gi|14590648|ref|NP_142716.1| hypothetical protein PH0780 [Pyrococcus horikoshii OT3]
>gi|14590672|ref|NP_142740.1| hypothetical protein PH0805 [Pyrococcus horikoshii OT3]
>gi|14590728|ref|NP_142798.1| hypothetical protein PH0869 [Pyrococcus horikoshii OT3]
>gi|33359340|ref|NP_877800.1| hypothetical protein PH0894.1n [Pyrococcus horikoshii OT3]
>gi|14590782|ref|NP_142852.1| purine NTPase [Pyrococcus horikoshii OT3]
>gi|33359347|ref|NP_142864.2| Multisubunit Na+/H+ antiporter, MnhC subunit [Pyrococcus horikoshii OT3]
>gi|14590794|ref|NP_142865.1| hypothetical protein PH0942 [Pyrococcus horikoshii OT3]
>gi|14590807|ref|NP_142878.1| hypothetical protein PH0955 [Pyrococcus horikoshii OT3]
>gi|14590856|ref|NP_142928.1| hypothetical protein PH1016 [Pyrococcus horikoshii OT3]
>gi|14590867|ref|NP_142939.1| hypothetical protein PH1029 [Pyrococcus horikoshii OT3]
>gi|14590868|ref|NP_142940.1| hypothetical protein PH1030 [Pyrococcus horikoshii OT3]
>gi|14590888|ref|NP_142960.1| hypothetical protein PH1051 [Pyrococcus horikoshii OT3]
>gi|14590890|ref|NP_142962.1| hypothetical protein PH1053 [Pyrococcus horikoshii OT3]
>gi|14590897|ref|NP_142969.1| hypothetical protein PH1060 [Pyrococcus horikoshii OT3]
>gi|14590912|ref|NP_142985.1| hypothetical protein PH1077 [Pyrococcus horikoshii OT3]
>gi|14590932|ref|NP_143005.1| hypothetical protein PH1101 [Pyrococcus horikoshii OT3]
>gi|14590934|ref|NP_143007.1| 30S ribosomal protein S8 [Pyrococcus horikoshii OT3]
>gi|14590935|ref|NP_143008.1| hypothetical protein PH1104 [Pyrococcus horikoshii OT3]

>gi|14590937|ref|NP_143010.1| hypothetical protein PH1106 [Pyrococcus horikoshii OT3]
>gi|14590948|ref|NP_143022.1| hypothetical protein PH1118 [Pyrococcus horikoshii OT3]
>gi|33359362|ref|NP_877815.1| hypothetical protein PH1126.1n [Pyrococcus horikoshii OT3]
>gi|14590955|ref|NP_143030.1| hypothetical protein PH1127 [Pyrococcus horikoshii OT3]
>gi|14590960|ref|NP_143035.1| sodium dependent phosphate transporter [Pyrococcus horikoshii OT3]
>gi|33359365|ref|NP_143038.2| hypothetical protein PH1135 [Pyrococcus horikoshii OT3]
>gi|14590989|ref|NP_143064.1| hypothetical protein PH1162 [Pyrococcus horikoshii OT3]
>gi|14590995|ref|NP_143070.1| hypothetical protein PH1169 [Pyrococcus horikoshii OT3]
>gi|14590996|ref|NP_143071.1| hypothetical protein PH1170 [Pyrococcus horikoshii OT3]
>gi|14591014|ref|NP_143089.1| hypothetical protein PH1189 [Pyrococcus horikoshii OT3]
>gi|33359372|ref|NP_877822.1| hypothetical protein PH1192.1n [Pyrococcus horikoshii OT3]
>gi|14591022|ref|NP_143097.1| hypothetical protein PH1199 [Pyrococcus horikoshii OT3]
>gi|33359375|ref|NP_877825.1| hypothetical protein PH1205.1n [Pyrococcus horikoshii OT3]
>gi|33359376|ref|NP_877826.1| hypothetical protein PH1210.1n [Pyrococcus horikoshii OT3]
>gi|14591046|ref|NP_143121.1| hypothetical protein PH1227 [Pyrococcus horikoshii OT3]
>gi|14591051|ref|NP_143126.1| hypothetical protein PH1232 [Pyrococcus horikoshii OT3]
>gi|14591052|ref|NP_143127.1| hypothetical protein PH1233 [Pyrococcus horikoshii OT3]
>gi|14591072|ref|NP_143147.1| hypothetical protein PH1254 [Pyrococcus horikoshii OT3]
>gi|33359382|ref|NP_877831.1| hypothetical protein PH1253.1n [Pyrococcus horikoshii OT3]
>gi|14591081|ref|NP_143156.1| hypothetical protein PH1264 [Pyrococcus horikoshii OT3]
>gi|33359385|ref|NP_877834.1| hypothetical protein PH1282.1n [Pyrococcus horikoshii OT3]
>gi|14591097|ref|NP_143172.1| methylmalonyl-CoA decarboxylase gamma chain [Pyrococcus horikoshii OT3]
>gi|33359390|ref|NP_877839.1| hypothetical protein PH1300.1n [Pyrococcus horikoshii OT3]
>gi|33359391|ref|NP_877840.1| hypothetical protein PHS037.1n [Pyrococcus horikoshii OT3]
>gi|33359396|ref|NP_143240.2| hypothetical protein PH1358 [Pyrococcus horikoshii OT3]
>gi|14591214|ref|NP_143290.1| hypothetical protein PH1419 [Pyrococcus horikoshii OT3]
>gi|14591749|ref|NP_143297.1| Multisubunit Na+/H+ antiporter, MnhF subunit [Pyrococcus horikoshii OT3]
>gi|14591222|ref|NP_143299.1| hypothetical protein PHS039 [Pyrococcus horikoshii OT3]
>gi|14591243|ref|NP_143320.1| hypothetical protein PH1453 [Pyrococcus horikoshii OT3]
>gi|33359406|ref|NP_877852.1| hypothetical Na+/H+ antiporter [Pyrococcus horikoshii OT3]
>gi|14591250|ref|NP_143327.1| hypothetical protein PH1461 [Pyrococcus horikoshii OT3]
>gi|14591276|ref|NP_143353.1| hypothetical protein PH1491 [Pyrococcus horikoshii OT3]
>gi|14591278|ref|NP_143355.1| hypothetical protein PH1493 [Pyrococcus horikoshii OT3]
>gi|14591281|ref|NP_143359.1| 50S ribosomal protein S28 [Pyrococcus horikoshii OT3]
>gi|14591283|ref|NP_143361.1| hypothetical protein PH1497 [Pyrococcus horikoshii OT3]
>gi|14591304|ref|NP_143382.1| hypothetical protein PH1521 [Pyrococcus horikoshii OT3]
>gi|14591306|ref|NP_143384.1| hypothetical protein PH1523 [Pyrococcus horikoshii OT3]
>gi|14591307|ref|NP_143385.1| hypothetical protein PH1524 [Pyrococcus horikoshii OT3]
>gi|14591318|ref|NP_143396.1| hypothetical protein PH1536 [Pyrococcus horikoshii OT3]
>gi|14591321|ref|NP_143399.1| hypothetical protein PH1539 [Pyrococcus horikoshii OT3]
>gi|14591339|ref|NP_143417.1| hypothetical protein PH1556 [Pyrococcus horikoshii OT3]
>gi|14591369|ref|NP_143447.1| ferripyochelin binding protein [Pyrococcus horikoshii OT3]
>gi|14591376|ref|NP_143454.1| hypothetical protein PH1599 [Pyrococcus horikoshii OT3]
>gi|33359417|ref|NP_877859.1| hypothetical protein PH1600.1n [Pyrococcus horikoshii OT3]
>gi|14591435|ref|NP_143515.1| hypothetical protein PH1668 [Pyrococcus horikoshii OT3]
>gi|14591436|ref|NP_143516.1| signal recognition particle protein [Pyrococcus horikoshii OT3]
>gi|14591461|ref|NP_143541.1| hypothetical protein PH1698 [Pyrococcus horikoshii OT3]
>gi|14591465|ref|NP_143545.1| archaeal histon [Pyrococcus horikoshii OT3]
>gi|14591467|ref|NP_143547.1| hypothetical protein PH1703 [Pyrococcus horikoshii OT3]
>gi|14591475|ref|NP_143555.1| hypothetical protein PH1712 [Pyrococcus horikoshii OT3]
>gi|33359423|ref|NP_877865.1| hypothetical protein PH1721.1n [Pyrococcus horikoshii OT3]
>gi|14591507|ref|NP_143588.1| ferredoxin [Pyrococcus horikoshii OT3]
>gi|33359426|ref|NP_143593.2| 50S ribosomal protein L15 [Pyrococcus horikoshii OT3]
>gi|14591516|ref|NP_143597.1| 50S ribosomal protein L19 [Pyrococcus horikoshii OT3]
>gi|14591538|ref|NP_143620.1| hypothetical protein PH1781 [Pyrococcus horikoshii OT3]
>gi|14591550|ref|NP_143632.1| hypothetical protein PH1795 [Pyrococcus horikoshii OT3]
>gi|14591552|ref|NP_143634.1| hypothetical protein PH1797 [Pyrococcus horikoshii OT3]
>gi|14591553|ref|NP_143635.1| chromosome assembly protein [Pyrococcus horikoshii OT3]
>gi|14591570|ref|NP_143652.1| hypothetical protein PH1819 [Pyrococcus horikoshii OT3]
>gi|33359430|ref|NP_877869.1| hypothetical protein PH1824.1n [Pyrococcus horikoshii OT3]
>gi|33359431|ref|NP_877870.1| hypothetical protein PH1827.1n [Pyrococcus horikoshii OT3]
>gi|14591582|ref|NP_143664.1| hypothetical protein PH1832 [Pyrococcus horikoshii OT3]
>gi|14591585|ref|NP_143667.1| hypothetical protein PH1836 [Pyrococcus horikoshii OT3]
>gi|33359432|ref|NP_877871.1| hypothetical protein PH1842.1n [Pyrococcus horikoshii OT3]
>gi|14591636|ref|NP_143718.1| hypothetical protein PH1892 [Pyrococcus horikoshii OT3]
>gi|14591638|ref|NP_143720.1| hypothetical protein PH1894 [Pyrococcus horikoshii OT3]
>gi|14591639|ref|NP_143721.1| hypothetical protein PH1895 [Pyrococcus horikoshii OT3]
>gi|14591668|ref|NP_143755.1| hypothetical protein PH1924 [Pyrococcus horikoshii OT3]
>gi|14591710|ref|NP_143798.1| hypothetical protein PH1973 [Pyrococcus horikoshii OT3]
>gi|14591715|ref|NP_143803.1| hypothetical protein PH1978 [Pyrococcus horikoshii OT3]

>gi|14591716|ref|NP_143804.1| hypothetical protein PH1980 [Pyrococcus horikoshii OT3]
>gi|14591718|ref|NP_143806.1| hypothetical protein PH1983 [Pyrococcus horikoshii OT3]
>gi|14591732|ref|NP_143820.1| 50S ribosomal protein L12 [Pyrococcus horikoshii OT3]
>gi|49183052|ref|YP_026304.1| hypothetical protein BAS0016 [Bacillus anthracis str. Sterne]
>gi|49183093|ref|YP_026345.1| membrane protein, putative [Bacillus anthracis str. Sterne]
>gi|49183134|ref|YP_026386.1| ribosomal protein L7/L12 [Bacillus anthracis str. Sterne]
>gi|49183185|ref|YP_026437.1| hypothetical protein BAS0151 [Bacillus anthracis str. Sterne]
>gi|49183206|ref|YP_026458.1| lipoprotein, putative [Bacillus anthracis str. Sterne]
>gi|49183299|ref|YP_026551.1| bacitracin ABC transporter, permease protein, putative [Bacillus anthracis str. Sterne]
>gi|49183316|ref|YP_026568.1| SPFH domain/band 7 family protein [Bacillus anthracis str. Sterne]
>gi|49183363|ref|YP_026615.1| iron compound ABC transporter, permease protein [Bacillus anthracis str. Sterne]
>gi|49183402|ref|YP_026654.1| hypothetical protein BAS0374 [Bacillus anthracis str. Sterne]
>gi|49183411|ref|YP_026663.1| LPXTG-motif cell wall anchor domain protein [Bacillus anthracis str. Sterne]
>gi|49183424|ref|YP_026676.1| hypothetical protein BAS0396 [Bacillus anthracis str. Sterne]
>gi|49183486|ref|YP_026738.1| hypothetical protein BAS0458 [Bacillus anthracis str. Sterne]
>gi|49183493|ref|YP_026745.1| hypothetical protein BAS0465 [Bacillus anthracis str. Sterne]
>gi|49183521|ref|YP_026773.1| small acid-soluble spore protein, gamma-type [Bacillus anthracis str. Sterne]
>gi|49183546|ref|YP_026798.1| internalin, putative [Bacillus anthracis str. Sterne]
>gi|49183551|ref|YP_026803.1| SPFH domain/band 7 family protein [Bacillus anthracis str. Sterne]
>gi|49183568|ref|YP_026820.1| hypothetical protein BAS0542 [Bacillus anthracis str. Sterne]
>gi|49183618|ref|YP_026870.1| hypothetical protein BAS0593 [Bacillus anthracis str. Sterne]
>gi|49183619|ref|YP_026871.1| hypothetical protein BAS0594 [Bacillus anthracis str. Sterne]
>gi|49183644|ref|YP_026896.1| hypothetical protein BAS0619 [Bacillus anthracis str. Sterne]
>gi|49183676|ref|YP_026928.1| hypothetical protein BAS0651 [Bacillus anthracis str. Sterne]
>gi|49183781|ref|YP_027033.1| hypothetical protein BAS0757 [Bacillus anthracis str. Sterne]
>gi|49183799|ref|YP_027051.1| hypothetical protein BAS0775 [Bacillus anthracis str. Sterne]
>gi|49183810|ref|YP_027062.1| hypothetical protein BAS0786 [Bacillus anthracis str. Sterne]
>gi|49183846|ref|YP_027098.1| hypothetical protein BAS0822 [Bacillus anthracis str. Sterne]
>gi|49183888|ref|YP_027140.1| hypothetical protein BAS0864 [Bacillus anthracis str. Sterne]
>gi|49183902|ref|YP_027154.1| hypothetical protein BAS0878 [Bacillus anthracis str. Sterne]
>gi|49183906|ref|YP_027158.1| lipoprotein, putative [Bacillus anthracis str. Sterne]
>gi|49183907|ref|YP_027159.1| hypothetical protein BAS0883 [Bacillus anthracis str. Sterne]
>gi|49183915|ref|YP_027167.1| protein-export membrane protein-related protein [Bacillus anthracis str. Sterne]
>gi|49183922|ref|YP_027174.1| hypothetical protein BAS0899 [Bacillus anthracis str. Sterne]
>gi|49183934|ref|YP_027186.1| CAAX amino terminal protease family protein [Bacillus anthracis str. Sterne]
>gi|49183945|ref|YP_027197.1| hypothetical protein BAS0923 [Bacillus anthracis str. Sterne]
>gi|49183956|ref|YP_027208.1| hypothetical protein BAS0934 [Bacillus anthracis str. Sterne]
>gi|49183960|ref|YP_027212.1| hypothetical protein BAS0938 [Bacillus anthracis str. Sterne]
>gi|49183996|ref|YP_027248.1| hypothetical protein BAS0975 [Bacillus anthracis str. Sterne]
>gi|49184011|ref|YP_027263.1| lipoprotein, putative [Bacillus anthracis str. Sterne]
>gi|49184120|ref|YP_027372.1| hypothetical protein BAS1100 [Bacillus anthracis str. Sterne]
>gi|49184130|ref|YP_027382.1| membrane protein, putative [Bacillus anthracis str. Sterne]
>gi|49184150|ref|YP_027402.1| hypothetical protein BAS1130 [Bacillus anthracis str. Sterne]
>gi|49184160|ref|YP_027412.1| hypothetical protein BAS1140 [Bacillus anthracis str. Sterne]
>gi|49184163|ref|YP_027415.1| hypothetical protein BAS1143 [Bacillus anthracis str. Sterne]
>gi|49184182|ref|YP_027434.1| hypothetical protein BAS1163 [Bacillus anthracis str. Sterne]
>gi|49184211|ref|YP_027463.1| hypothetical protein BAS1193 [Bacillus anthracis str. Sterne]
>gi|49184226|ref|YP_027478.1| hypothetical protein BAS1208 [Bacillus anthracis str. Sterne]
>gi|49184315|ref|YP_027567.1| hypothetical protein BAS1298 [Bacillus anthracis str. Sterne]
>gi|49184356|ref|YP_027608.1| peptidase, M23/M37 family [Bacillus anthracis str. Sterne]
>gi|49184371|ref|YP_027623.1| hypothetical protein BAS1354 [Bacillus anthracis str. Sterne]
>gi|49184481|ref|YP_027733.1| hypothetical protein BAS1464 [Bacillus anthracis str. Sterne]
>gi|49184482|ref|YP_027734.1| spore coat protein D, putative [Bacillus anthracis str. Sterne]
>gi|49184502|ref|YP_027754.1| lipoprotein, putative [Bacillus anthracis str. Sterne]
>gi|49184507|ref|YP_027759.1| acyltransferase family protein [Bacillus anthracis str. Sterne]
>gi|49184545|ref|YP_027797.1| membrane protein, putative [Bacillus anthracis str. Sterne]
>gi|49184550|ref|YP_027802.1| hypothetical protein BAS1535 [Bacillus anthracis str. Sterne]
>gi|49184560|ref|YP_027812.1| hypothetical protein BAS1545 [Bacillus anthracis str. Sterne]
>gi|49184577|ref|YP_027829.1| hypothetical protein BAS1562 [Bacillus anthracis str. Sterne]
>gi|49184597|ref|YP_027849.1| flagellin [Bacillus anthracis str. Sterne]
>gi|49184622|ref|YP_027874.1| lipoprotein, putative [Bacillus anthracis str. Sterne]
>gi|49184637|ref|YP_027889.1| transcriptional regulator, MarR family [Bacillus anthracis str. Sterne]
>gi|49184688|ref|YP_027940.1| hypothetical protein BAS1674 [Bacillus anthracis str. Sterne]
>gi|49184778|ref|YP_028030.1| membrane protein, putative [Bacillus anthracis str. Sterne]
>gi|49184824|ref|YP_028076.1| NLP/P60 family protein [Bacillus anthracis str. Sterne]
>gi|49184880|ref|YP_028132.1| hypothetical protein BAS1868 [Bacillus anthracis str. Sterne]
>gi|49184897|ref|YP_028149.1| hypothetical protein BAS1885 [Bacillus anthracis str. Sterne]
>gi|49184910|ref|YP_028162.1| hypothetical protein BAS1898 [Bacillus anthracis str. Sterne]
>gi|49184939|ref|YP_028191.1| SPFH domain/Band 7 family protein [Bacillus anthracis str. Sterne]
>gi|49184994|ref|YP_028246.1| hypothetical protein BAS1983 [Bacillus anthracis str. Sterne]

>gi|49185011|ref|YP_028263.1| hypothetical protein BAS2000 [Bacillus anthracis str. Sterne]
>gi|49185015|ref|YP_028267.1| stage V sporulation protein S [Bacillus anthracis str. Sterne]
>gi|49185019|ref|YP_028271.1| hypothetical protein BAS2008 [Bacillus anthracis str. Sterne]
>gi|49185051|ref|YP_028303.1| hypothetical protein BAS2040 [Bacillus anthracis str. Sterne]
>gi|49185086|ref|YP_028338.1| protein ecsB, C-terminal [Bacillus anthracis str. Sterne]
>gi|49185160|ref|YP_028412.1| hypothetical protein BAS2151 [Bacillus anthracis str. Sterne]
>gi|49185184|ref|YP_028436.1| hypothetical protein BAS2175 [Bacillus anthracis str. Sterne]
>gi|49185194|ref|YP_028446.1| penicillin-binding protein 1A [Bacillus anthracis str. Sterne]
>gi|49185247|ref|YP_028499.1| hypothetical protein BAS2238 [Bacillus anthracis str. Sterne]
>gi|49185275|ref|YP_028527.1| hypothetical protein BAS2266 [Bacillus anthracis str. Sterne]
>gi|49185289|ref|YP_028541.1| hypothetical protein BAS2280 [Bacillus anthracis str. Sterne]
>gi|49185290|ref|YP_028542.1| hypothetical protein BAS2281 [Bacillus anthracis str. Sterne]
>gi|49185304|ref|YP_028556.1| membrane protein, putative [Bacillus anthracis str. Sterne]
>gi|49185327|ref|YP_028579.1| hypothetical protein BAS2318 [Bacillus anthracis str. Sterne]
>gi|49185376|ref|YP_028628.1| hypothetical protein BAS2367 [Bacillus anthracis str. Sterne]
>gi|49185446|ref|YP_028698.1| hypothetical protein BAS2438 [Bacillus anthracis str. Sterne]
>gi|49185453|ref|YP_028705.1| hypothetical protein BAS2445 [Bacillus anthracis str. Sterne]
>gi|49185461|ref|YP_028713.1| hypothetical protein BAS2453 [Bacillus anthracis str. Sterne]
>gi|49185476|ref|YP_028728.1| permease, putative [Bacillus anthracis str. Sterne]
>gi|49185501|ref|YP_028753.1| hypothetical protein BAS2493 [Bacillus anthracis str. Sterne]
>gi|49185566|ref|YP_028818.1| lipoprotein, putative [Bacillus anthracis str. Sterne]
>gi|49185637|ref|YP_028889.1| hypothetical protein BAS2630 [Bacillus anthracis str. Sterne]
>gi|49185657|ref|YP_028909.1| hypothetical protein BAS2650 [Bacillus anthracis str. Sterne]
>gi|49185669|ref|YP_028921.1| hypothetical protein BAS2662 [Bacillus anthracis str. Sterne]
>gi|49185672|ref|YP_028924.1| hypothetical protein BAS2665 [Bacillus anthracis str. Sterne]
>gi|49185698|ref|YP_028950.1| hypothetical protein BAS2692 [Bacillus anthracis str. Sterne]
>gi|49185731|ref|YP_028983.1| lipoprotein, putative [Bacillus anthracis str. Sterne]
>gi|49185739|ref|YP_028991.1| lipoprotein, putative [Bacillus anthracis str. Sterne]
>gi|49185746|ref|YP_028998.1| hypothetical protein BAS2740 [Bacillus anthracis str. Sterne]
>gi|49185762|ref|YP_029014.1| hypothetical protein BAS2756 [Bacillus anthracis str. Sterne]
>gi|49185770|ref|YP_029022.1| ribose ABC transporter, permease protein, putative [Bacillus anthracis str. Sterne]
>gi|49185798|ref|YP_029050.1| hypothetical protein BAS2792 [Bacillus anthracis str. Sterne]
>gi|49185811|ref|YP_029063.1| hypothetical protein BAS2805 [Bacillus anthracis str. Sterne]
>gi|49185815|ref|YP_029067.1| hypothetical protein BAS2809 [Bacillus anthracis str. Sterne]
>gi|49185851|ref|YP_029103.1| hypothetical protein BAS2845 [Bacillus anthracis str. Sterne]
>gi|49185913|ref|YP_029165.1| amino acid permease [Bacillus anthracis str. Sterne]
>gi|49185927|ref|YP_029179.1| pyrroline-5-carboxylate reductase [Bacillus anthracis str. Sterne]
>gi|49185928|ref|YP_029180.1| hypothetical protein BAS2922 [Bacillus anthracis str. Sterne]
>gi|49185996|ref|YP_029248.1| hypothetical protein BAS2991 [Bacillus anthracis str. Sterne]
>gi|49186000|ref|YP_029252.1| hypothetical protein BAS2995 [Bacillus anthracis str. Sterne]
>gi|49186006|ref|YP_029258.1| hypothetical protein BAS3001 [Bacillus anthracis str. Sterne]
>gi|49186008|ref|YP_029261.1| hypothetical protein BAS3003 [Bacillus anthracis str. Sterne]
>gi|49186016|ref|YP_029268.1| hypothetical protein BAS3011 [Bacillus anthracis str. Sterne]
>gi|49186026|ref|YP_029278.1| LPXTG-motif cell wall anchor domain protein [Bacillus anthracis str. Sterne]
>gi|49186048|ref|YP_029300.1| hypothetical protein BAS3043 [Bacillus anthracis str. Sterne]
>gi|49186111|ref|YP_029363.1| hypothetical protein BAS3106 [Bacillus anthracis str. Sterne]
>gi|49186125|ref|YP_029377.1| lipoprotein, putative [Bacillus anthracis str. Sterne]
>gi|49186140|ref|YP_029392.1| hypothetical protein BAS3135 [Bacillus anthracis str. Sterne]
>gi|49186142|ref|YP_029394.1| lipoprotein, putative [Bacillus anthracis str. Sterne]
>gi|49186167|ref|YP_029419.1| hypothetical protein BAS3162 [Bacillus anthracis str. Sterne]
>gi|49186180|ref|YP_029432.1| pentapeptide repeats domain protein [Bacillus anthracis str. Sterne]
>gi|49186235|ref|YP_029487.1| hypothetical protein BAS3230 [Bacillus anthracis str. Sterne]
>gi|49186240|ref|YP_029492.1| hypothetical protein BAS3235 [Bacillus anthracis str. Sterne]
>gi|49186242|ref|YP_029494.1| transcriptional regulator, DeoR family [Bacillus anthracis str. Sterne]
>gi|49186244|ref|YP_029496.1| ABC transporter, efflux permease protein [Bacillus anthracis str. Sterne]
>gi|49186295|ref|YP_029547.1| hypothetical protein BAS3290 [Bacillus anthracis str. Sterne]
>gi|49186306|ref|YP_029558.1| hypothetical protein BAS3301 [Bacillus anthracis str. Sterne]
>gi|49186319|ref|YP_029571.1| bile acid transporter family protein [Bacillus anthracis str. Sterne]
>gi|49186333|ref|YP_029585.1| hypothetical protein BAS3328 [Bacillus anthracis str. Sterne]
>gi|49186380|ref|YP_029632.1| hypothetical protein BAS3375 [Bacillus anthracis str. Sterne]
>gi|49186424|ref|YP_029676.1| bioY family protein [Bacillus anthracis str. Sterne]
>gi|49186428|ref|YP_029680.1| hypothetical protein BAS3424 [Bacillus anthracis str. Sterne]
>gi|49186434|ref|YP_029686.1| membrane protein, putative [Bacillus anthracis str. Sterne]
>gi|49186456|ref|YP_029708.1| conserved repeat domain protein [Bacillus anthracis str. Sterne]
>gi|49186471|ref|YP_029723.1| conserved hypothetical protein UPF0154 [Bacillus anthracis str. Sterne]
>gi|49186520|ref|YP_029772.1| hypothetical protein BAS3518 [Bacillus anthracis str. Sterne]
>gi|49186529|ref|YP_029781.1| hypothetical protein BAS3527 [Bacillus anthracis str. Sterne]
>gi|49186542|ref|YP_029794.1| hypothetical protein BAS3540 [Bacillus anthracis str. Sterne]
>gi|49186559|ref|YP_029811.1| hypothetical protein BAS3557 [Bacillus anthracis str. Sterne]
>gi|49186563|ref|YP_029815.1| hypothetical protein BAS3562 [Bacillus anthracis str. Sterne]

>gi|49186564|ref|YP_029816.1| PTS system, fructose-specific IIABC component [Bacillus anthracis str. Sterne]
>gi|49186600|ref|YP_029852.1| hypothetical protein BAS3599 [Bacillus anthracis str. Sterne]
>gi|49186626|ref|YP_029878.1| stage V sporulation protein S [Bacillus anthracis str. Sterne]
>gi|49186632|ref|YP_029884.1| hypothetical protein BAS3632 [Bacillus anthracis str. Sterne]
>gi|49186693|ref|YP_029945.1| KH domain protein [Bacillus anthracis str. Sterne]
>gi|49186696|ref|YP_029948.1| hypothetical protein BAS3697 [Bacillus anthracis str. Sterne]
>gi|49186832|ref|YP_030084.1| hypothetical protein BAS3834 [Bacillus anthracis str. Sterne]
>gi|49186833|ref|YP_030085.1| hypothetical protein BAS3835 [Bacillus anthracis str. Sterne]
>gi|49186867|ref|YP_030119.1| hypothetical protein BAS3869 [Bacillus anthracis str. Sterne]
>gi|49186889|ref|YP_030141.1| 2,3,4,5-tetrahydropyridine-2-carboxylate N-succinyltransferase, putative [Bacillus anthracis str. Sterne]
>gi|49186900|ref|YP_030152.1| hypothetical protein BAS3902 [Bacillus anthracis str. Sterne]
>gi|49186904|ref|YP_030156.1| lipoprotein, putative [Bacillus anthracis str. Sterne]
>gi|49187006|ref|YP_030258.1| hypothetical protein BAS4008 [Bacillus anthracis str. Sterne]
>gi|49187027|ref|YP_030279.1| hypothetical protein BAS4029 [Bacillus anthracis str. Sterne]
>gi|49187032|ref|YP_030284.1| hypothetical protein BAS4034 [Bacillus anthracis str. Sterne]
>gi|49187044|ref|YP_030296.1| L-serine dehydratase, iron-sulfur-dependent, alpha subunit [Bacillus anthracis str. Sterne]
>gi|49187087|ref|YP_030339.1| acetyl-CoA carboxylase, biotin carboxyl carrier protein [Bacillus anthracis str. Sterne]
>gi|49187088|ref|YP_030340.1| stage III sporulation protein AH [Bacillus anthracis str. Sterne]
>gi|49187092|ref|YP_030344.1| stage III sporulation protein AD [Bacillus anthracis str. Sterne]
>gi|49187108|ref|YP_030360.1| hypothetical protein BAS4110 [Bacillus anthracis str. Sterne]
>gi|49187113|ref|YP_030365.1| sugE protein [Bacillus anthracis str. Sterne]
>gi|49187115|ref|YP_030367.1| hypothetical protein BAS4117 [Bacillus anthracis str. Sterne]
>gi|49187136|ref|YP_030388.1| hypothetical protein BAS4139 [Bacillus anthracis str. Sterne]
>gi|49187161|ref|YP_030413.1| hypothetical protein BAS4164 [Bacillus anthracis str. Sterne]
>gi|49187185|ref|YP_030437.1| VrrA protein [Bacillus anthracis str. Sterne]
>gi|49187204|ref|YP_030456.1| ribosomal protein S21 [Bacillus anthracis str. Sterne]
>gi|49187210|ref|YP_030462.1| GrpE protein [Bacillus anthracis str. Sterne]
>gi|49187217|ref|YP_030469.1| ribosomal protein S20 [Bacillus anthracis str. Sterne]
>gi|49187233|ref|YP_030485.1| hypothetical protein BAS4237 [Bacillus anthracis str. Sterne]
>gi|49187253|ref|YP_030505.1| hypothetical protein BAS4257 [Bacillus anthracis str. Sterne]
>gi|49187269|ref|YP_030521.1| hypothetical protein BAS4273 [Bacillus anthracis str. Sterne]
>gi|49187318|ref|YP_030570.1| forespore-specific protein, putative [Bacillus anthracis str. Sterne]
>gi|49187319|ref|YP_030571.1| lysM domain protein [Bacillus anthracis str. Sterne]
>gi|49187331|ref|YP_030583.1| Bacillus cereus group-specific protein; possible two-component sensor kinase [Bacillus anthracis str. Sterne]
>gi|49187352|ref|YP_030604.1| stage VI sporulation protein D, putative [Bacillus anthracis str. Sterne]
>gi|49187379|ref|YP_030631.1| hypothetical protein BAS4384 [Bacillus anthracis str. Sterne]
>gi|49187393|ref|YP_030645.1| membrane protein, putative [Bacillus anthracis str. Sterne]
>gi|49187418|ref|YP_030670.1| conserved hypothetical protein, collagen triple helix repeat domain [Bacillus anthracis str. Sterne]
>gi|49187437|ref|YP_030689.1| hypothetical protein BAS4442 [Bacillus anthracis str. Sterne]
>gi|49187458|ref|YP_030710.1| hypothetical protein BAS4463 [Bacillus anthracis str. Sterne]
>gi|49187475|ref|YP_030727.1| membrane protein, putative [Bacillus anthracis str. Sterne]
>gi|49187484|ref|YP_030737.1| hypothetical protein BAS4489 [Bacillus anthracis str. Sterne]
>gi|49187512|ref|YP_030765.1| hypothetical protein BAS4518 [Bacillus anthracis str. Sterne]
>gi|49187532|ref|YP_030785.1| hypothetical protein BAS4538 [Bacillus anthracis str. Sterne]
>gi|49187578|ref|YP_030831.1| FtsK/SpoIIIE family protein [Bacillus anthracis str. Sterne]
>gi|49187603|ref|YP_030856.1| hypothetical protein BAS4610 [Bacillus anthracis str. Sterne]
>gi|49187616|ref|YP_030869.1| collagen triple helix repeat domain protein [Bacillus anthracis str. Sterne]
>gi|49187620|ref|YP_030873.1| hypothetical protein BAS4627 [Bacillus anthracis str. Sterne]
>gi|49187622|ref|YP_030875.1| spore germination protein GerHA [Bacillus anthracis str. Sterne]
>gi|49187627|ref|YP_030880.1| vrrB protein [Bacillus anthracis str. Sterne]
>gi|49187636|ref|YP_030889.1| hypothetical protein BAS4645 [Bacillus anthracis str. Sterne]
>gi|49187688|ref|YP_030941.1| lipoprotein, putative [Bacillus anthracis str. Sterne]
>gi|49187698|ref|YP_030951.1| DNA-binding response regulator [Bacillus anthracis str. Sterne]
>gi|49187700|ref|YP_030953.1| cell wall surface anchor family protein [Bacillus anthracis str. Sterne]
>gi|49187706|ref|YP_030959.1| hypothetical protein BAS4715 [Bacillus anthracis str. Sterne]
>gi|49187725|ref|YP_030978.1| hypothetical protein BAS4734 [Bacillus anthracis str. Sterne]
>gi|49187807|ref|YP_031060.1| hypothetical protein BAS4817 [Bacillus anthracis str. Sterne]
>gi|49187844|ref|YP_031097.1| ABC transporter, permease protein [Bacillus anthracis str. Sterne]
>gi|49187876|ref|YP_031129.1| hypothetical protein BAS4888 [Bacillus anthracis str. Sterne]
>gi|49187884|ref|YP_031137.1| hypothetical protein BAS4896 [Bacillus anthracis str. Sterne]
>gi|49187930|ref|YP_031183.1| crcB protein [Bacillus anthracis str. Sterne]
>gi|49188029|ref|YP_031282.1| endopeptidase lytE, putative [Bacillus anthracis str. Sterne]
>gi|49188070|ref|YP_031323.1| N-acetylmuramoyl-L-alanine amidase, C-terminus [Bacillus anthracis str. Sterne]
>gi|49188076|ref|YP_031329.1| hypothetical protein BAS5090 [Bacillus anthracis str. Sterne]
>gi|49188134|ref|YP_031387.1| NADH dehydrogenase I, C subunit, putative [Bacillus anthracis str. Sterne]
>gi|49188162|ref|YP_031415.1| stage II sporulation protein R, putative [Bacillus anthracis str. Sterne]
>gi|49188174|ref|YP_031427.1| DNA-directed RNA polymerase, delta subunit [Bacillus anthracis str. Sterne]
>gi|49188217|ref|YP_031470.1| ABC transporter permease, C-terminal [Bacillus anthracis str. Sterne]
>gi|49188227|ref|YP_031480.1| hypothetical protein BAS5242 [Bacillus anthracis str. Sterne]
>gi|49188269|ref|YP_031522.1| membrane protein, putative [Bacillus anthracis str. Sterne]

>gi|49188310|ref|YP_031563.1| single-stranded DNA-binding protein [Bacillus anthracis str. Sterne]
>gi|24378550|ref|NP_720505.1| putative secreted antigen GbpB/SagA; putative peptidoglycan hydrolase [Streptococcus mutans UA159]
>gi|24378589|ref|NP_720544.1| hypothetical protein SMU.63c [Streptococcus mutans UA159]
>gi|24378605|ref|NP_720560.1| heat shock protein GrpE (HSP-70 cofactor) [Streptococcus mutans UA159]
>gi|24378618|ref|NP_720573.1| putative DNA-directed RNA polymerase, delta subunit [Streptococcus mutans UA159]
>gi|24378662|ref|NP_720617.1| hypothetical protein SMU.145 [Streptococcus mutans UA159]
>gi|24378665|ref|NP_720620.1| hypothetical protein SMU.150 [Streptococcus mutans UA159]
>gi|24378667|ref|NP_720622.1| hypothetical protein SMU.152 [Streptococcus mutans UA159]
>gi|24378703|ref|NP_720658.1| hypothetical protein SMU.189 [Streptococcus mutans UA159]
>gi|24378747|ref|NP_720702.1| putative integral membrane protein [Streptococcus mutans UA159]
>gi|24378749|ref|NP_720704.1| hypothetical protein SMU.239c [Streptococcus mutans UA159]
>gi|24378783|ref|NP_720738.1| hypothetical protein SMU.277 [Streptococcus mutans UA159]
>gi|24378786|ref|NP_720741.1| hypothetical protein SMU.281 [Streptococcus mutans UA159]
>gi|24378787|ref|NP_720742.1| hypothetical protein SMU.283 [Streptococcus mutans UA159]
>gi|24378791|ref|NP_720746.1| putative ComB, accessory factor for ComA [Streptococcus mutans UA159]
>gi|24378817|ref|NP_720772.1| putative tetrahydrodipicolinate succinylase [Streptococcus mutans UA159]
>gi|24378831|ref|NP_720786.1| hypothetical protein SMU.333 [Streptococcus mutans UA159]
>gi|24378847|ref|NP_720802.1| hypothetical protein SMU.350 [Streptococcus mutans UA159]
>gi|24378868|ref|NP_720823.1| conserved hypothetical protein; inner membrane protein [Streptococcus mutans UA159]
>gi|24378898|ref|NP_720853.1| putative transcriptional regulator [Streptococcus mutans UA159]
>gi|24378902|ref|NP_720857.1| hypothetical protein SMU.409 [Streptococcus mutans UA159]
>gi|24378907|ref|NP_720862.1| putative ABC transporter, permease protein [Streptococcus mutans UA159]
>gi|24378914|ref|NP_720869.1| translation initiation factor 2 [Streptococcus mutans UA159]
>gi|24378916|ref|NP_720871.1| hypothetical protein SMU.423 [Streptococcus mutans UA159]
>gi|24378960|ref|NP_720915.1| hypothetical protein SMU.473 [Streptococcus mutans UA159]
>gi|24378964|ref|NP_720919.1| RNA polymerase-associated protein RpoZ, omega subunit [Streptococcus mutans UA159]
>gi|24378978|ref|NP_720933.1| putative transaldolase [Streptococcus mutans UA159]
>gi|24378987|ref|NP_720942.1| hypothetical protein SMU.503c [Streptococcus mutans UA159]
>gi|24379005|ref|NP_720960.1| hypothetical protein SMU.523 [Streptococcus mutans UA159]
>gi|24379031|ref|NP_720986.1| putative cell division protein FtsQ (DivIB) [Streptococcus mutans UA159]
>gi|24379035|ref|NP_720990.1| hypothetical protein SMU.554 [Streptococcus mutans UA159]
>gi|24379054|ref|NP_721009.1| putative membrane protein [Streptococcus mutans UA159]
>gi|24379072|ref|NP_721027.1| hypothetical protein SMU.594 [Streptococcus mutans UA159]
>gi|24379087|ref|NP_721042.1| cell surface antigen SpaP [Streptococcus mutans UA159]
>gi|24379089|ref|NP_721044.1| hypothetical protein SMU.613 [Streptococcus mutans UA159]
>gi|24379092|ref|NP_721047.1| hypothetical protein SMU.618 [Streptococcus mutans UA159]
>gi|24379100|ref|NP_721055.1| hypothetical protein SMU.627 [Streptococcus mutans UA159]
>gi|24379110|ref|NP_721065.1| hypothetical protein SMU.637c [Streptococcus mutans UA159]
>gi|24379150|ref|NP_721105.1| putative transcriptional regulator (MerR family) [Streptococcus mutans UA159]
>gi|24379162|ref|NP_721117.1| hypothetical protein SMU.690 [Streptococcus mutans UA159]
>gi|24379166|ref|NP_721121.1| hypothetical protein SMU.695 [Streptococcus mutans UA159]
>gi|24379203|ref|NP_721158.1| hypothetical protein SMU.739c [Streptococcus mutans UA159]
>gi|24379211|ref|NP_721166.1| hypothetical protein SMU.748 [Streptococcus mutans UA159]
>gi|24379249|ref|NP_721204.1| hypothetical protein SMU.791c [Streptococcus mutans UA159]
>gi|24379259|ref|NP_721214.1| hypothetical protein SMU.802 [Streptococcus mutans UA159]
>gi|24379273|ref|NP_721228.1| 30S ribosomal protein S21 [Streptococcus mutans UA159]
>gi|24379295|ref|NP_721250.1| hypothetical protein SMU.840c [Streptococcus mutans UA159]
>gi|24379301|ref|NP_721256.1| hypothetical protein SMU.847c [Streptococcus mutans UA159]
>gi|24379302|ref|NP_721257.1| 50S ribosomal protein L21 [Streptococcus mutans UA159]
>gi|24379316|ref|NP_721271.1| conserved hypothetical protein; putative permease [Streptococcus mutans UA159]
>gi|24379359|ref|NP_721314.1| hypothetical protein SMU.911c [Streptococcus mutans UA159]
>gi|24379370|ref|NP_721325.1| hypothetical protein SMU.925 [Streptococcus mutans UA159]
>gi|24379374|ref|NP_721329.1| hypothetical protein SMU.929c [Streptococcus mutans UA159]
>gi|24379401|ref|NP_721356.1| hypothetical protein SMU.958 [Streptococcus mutans UA159]
>gi|24379402|ref|NP_721357.1| hypothetical protein SMU.959c [Streptococcus mutans UA159]
>gi|24379403|ref|NP_721358.1| 50S ribosomal protein L7/L12 [Streptococcus mutans UA159]
>gi|24379427|ref|NP_721382.1| cell wall-associated protein precursor WapA [Streptococcus mutans UA159]
>gi|24379436|ref|NP_721391.1| putative ABC transporter, permease protein; possible ferrichrome transport system [Streptococcus mutans UA159]
>gi|24379553|ref|NP_721508.1| putative ABC transporter [Streptococcus mutans UA159]
>gi|24379559|ref|NP_721514.1| putative 30S ribosomal protein S20 [Streptococcus mutans UA159]
>gi|24379562|ref|NP_721517.1| hypothetical protein SMU.1131c [Streptococcus mutans UA159]
>gi|24379617|ref|NP_721572.1| hypothetical protein SMU.1189c [Streptococcus mutans UA159]
>gi|24379623|ref|NP_721578.1| conserved hypothetical protein; possible permease [Streptococcus mutans UA159]
>gi|24379710|ref|NP_721665.1| putative flavodoxin [Streptococcus mutans UA159]
>gi|24379732|ref|NP_721687.1| hypothetical protein SMU.1316c [Streptococcus mutans UA159]
>gi|24379801|ref|NP_721756.1| glucan-binding protein C, GbpC [Streptococcus mutans UA159]
>gi|24379804|ref|NP_721759.1| hypothetical protein SMU.1399 [Streptococcus mutans UA159]
>gi|24379806|ref|NP_721761.1| hypothetical protein SMU.1402c [Streptococcus mutans UA159]
>gi|24379852|ref|NP_721807.1| putative membrane protein; possible permease [Streptococcus mutans UA159]
>gi|24379873|ref|NP_721828.1| hypothetical protein SMU.1479 [Streptococcus mutans UA159]

>gi|24379959|ref|NP_721914.1| hypothetical protein SMU.1575c [Streptococcus mutans UA159]
>gi|24380019|ref|NP_721974.1| hypothetical protein SMU.1641c [Streptococcus mutans UA159]
>gi|24380026|ref|NP_721981.1| hypothetical protein SMU.1648c [Streptococcus mutans UA159]
>gi|24380075|ref|NP_722030.1| hypothetical protein SMU.1701c [Streptococcus mutans UA159]
>gi|24380077|ref|NP_722032.1| hypothetical protein SMU.1703c [Streptococcus mutans UA159]
>gi|24380079|ref|NP_722034.1| hypothetical protein SMU.1705 [Streptococcus mutans UA159]
>gi|24380164|ref|NP_722119.1| hypothetical protein SMU.1800c [Streptococcus mutans UA159]
>gi|24380190|ref|NP_722145.1| putative biotin biosynthesis protein [Streptococcus mutans UA159]
>gi|24380219|ref|NP_722174.1| putative single-stranded DNA-binding protein [Streptococcus mutans UA159]
>gi|24380221|ref|NP_722176.1| hypothetical protein SMU.1861c [Streptococcus mutans UA159]
>gi|24380222|ref|NP_722177.1| hypothetical protein SMU.1862 [Streptococcus mutans UA159]
>gi|24380237|ref|NP_722192.1| hypothetical protein SMU.1882c [Streptococcus mutans UA159]
>gi|24380242|ref|NP_722197.1| hypothetical protein SMU.1889c [Streptococcus mutans UA159]
>gi|24380248|ref|NP_722203.1| hypothetical protein SMU.1896c [Streptococcus mutans UA159]
>gi|24380252|ref|NP_722207.1| hypothetical protein SMU.1900 [Streptococcus mutans UA159]
>gi|24380255|ref|NP_722210.1| hypothetical protein SMU.1904c [Streptococcus mutans UA159]
>gi|24380257|ref|NP_722212.1| hypothetical protein SMU.1906c [Streptococcus mutans UA159]
>gi|24380264|ref|NP_722219.1| hypothetical protein SMU.1914c [Streptococcus mutans UA159]
>gi|24380319|ref|NP_722274.1| conserved hypothetical protein; possible membrane protein [Streptococcus mutans UA159]
>gi|24380398|ref|NP_722353.1| hypothetical protein SMU.2061 [Streptococcus mutans UA159]
>gi|24380416|ref|NP_722371.1| hypothetical protein SMU.2081 [Streptococcus mutans UA159]
>gi|24380417|ref|NP_722372.1| hypothetical protein SMU.2083c [Streptococcus mutans UA159]
>gi|24380463|ref|NP_722418.1| putative membrane protein [Streptococcus mutans UA159]
>gi|24380469|ref|NP_722424.1| 50S ribosomal protein L9 [Streptococcus mutans UA159]
>gi|24380474|ref|NP_722429.1| hypothetical protein SMU.2146c [Streptococcus mutans UA159]
>gi|24380475|ref|NP_722430.1| hypothetical protein SMU.2147c [Streptococcus mutans UA159]
>gi|72383756|ref|YP_293110.1| hypothetical protein Reut_C5911 [Ralstonia eutropha JMP134]
>gi|72383758|ref|YP_293112.1| putative membrane protein [Ralstonia eutropha JMP134]
>gi|72383769|ref|YP_293123.1| hypothetical protein Reut_C5925 [Ralstonia eutropha JMP134]
>gi|72383796|ref|YP_293150.1| inner-membrane translocator [Ralstonia eutropha JMP134]
>gi|72383812|ref|YP_293166.1| VCBS [Ralstonia eutropha JMP134]
>gi|72383831|ref|YP_293185.1| Histone-like bacterial DNA-binding protein [Ralstonia eutropha JMP134]
>gi|72383844|ref|YP_293198.1| histone H1-like protein [Ralstonia eutropha JMP134]
>gi|72383846|ref|YP_293200.1| probable signal peptide protein [Ralstonia eutropha JMP134]
>gi|72383847|ref|YP_293201.1| GCN5-related N-acetyltransferase [Ralstonia eutropha JMP134]
>gi|72383860|ref|YP_293214.1| putative Uncharacterized conserved protein [Ralstonia eutropha JMP134]
>gi|72383902|ref|YP_293256.1| hypothetical protein Reut_C6073 [Ralstonia eutropha JMP134]
>gi|72383946|ref|YP_293300.1| putative membrane protein [Ralstonia eutropha JMP134]
>gi|72383952|ref|YP_293306.1| Major facilitator superfamily MFS_1 [Ralstonia eutropha JMP134]
>gi|72383962|ref|YP_293316.1| hypothetical protein Reut_C6139 [Ralstonia eutropha JMP134]
>gi|72383975|ref|YP_293329.1| hypothetical protein Reut_C6154 [Ralstonia eutropha JMP134]
>gi|72383976|ref|YP_293330.1| hypothetical protein Reut_C6156 [Ralstonia eutropha JMP134]
>gi|72383983|ref|YP_293337.1| hypothetical protein Reut_C6165 [Ralstonia eutropha JMP134]
>gi|72383994|ref|YP_293348.1| hypothetical protein Reut_C6176 [Ralstonia eutropha JMP134]
>gi|72383997|ref|YP_293351.1| Sulfate transporter/antisigma-factor antagonist STAS:Xanthine/uracil/vitamin C permease:Sulphate transporter [Ralstonia eutropha JMP134]
>gi|72383999|ref|YP_293353.1| hypothetical protein Reut_C6181 [Ralstonia eutropha JMP134]
>gi|72384006|ref|YP_293360.1| General substrate transporter:Major facilitator superfamily MFS_1 [Ralstonia eutropha JMP134]
>gi|72384025|ref|YP_293379.1| inner-membrane translocator [Ralstonia eutropha JMP134]
>gi|72384033|ref|YP_293387.1| transferase hexapeptide repeat [Ralstonia eutropha JMP134]
>gi|72384077|ref|YP_293431.1| inner-membrane translocator [Ralstonia eutropha JMP134]
>gi|72384131|ref|YP_293485.1| Short-chain dehydrogenase/reductase SDR [Ralstonia eutropha JMP134]
>gi|72384149|ref|YP_293503.1| hypothetical protein Reut_C6343 [Ralstonia eutropha JMP134]
>gi|72384152|ref|YP_293506.1| hypothetical protein Reut_C6346 [Ralstonia eutropha JMP134]
>gi|72384153|ref|YP_293507.1| Histone-like nucleoid-structuring protein H-NS [Ralstonia eutropha JMP134]
>gi|72384186|ref|YP_293540.1| hypothetical protein Reut_C6381 [Ralstonia eutropha JMP134]
>gi|72384187|ref|YP_293541.1| Histone-like nucleoid-structuring protein H-NS [Ralstonia eutropha JMP134]
>gi|72384188|ref|YP_293542.1| hypothetical protein Reut_C6383 [Ralstonia eutropha JMP134]
>gi|72384197|ref|YP_293551.1| putative signal peptide protein [Ralstonia eutropha JMP134]
>gi|72384238|ref|YP_293592.1| inner-membrane translocator [Ralstonia eutropha JMP134]
>gi|72384245|ref|YP_293598.1| hypothetical protein Reut_D6441 [Ralstonia eutropha JMP134]
>gi|72384247|ref|YP_293600.1| hypothetical protein Reut_D6443 [Ralstonia eutropha JMP134]
>gi|72384251|ref|YP_293604.1| hypothetical protein Reut_D6447 [Ralstonia eutropha JMP134]
>gi|72384262|ref|YP_293615.1| hypothetical protein Reut_D6458 [Ralstonia eutropha JMP134]
>gi|72384271|ref|YP_293624.1| Benzoate transport [Ralstonia eutropha JMP134]
>gi|72384273|ref|YP_293626.1| Iron-containing alcohol dehydrogenase [Ralstonia eutropha JMP134]
>gi|72384288|ref|YP_293641.1| Short-chain dehydrogenase/reductase SDR [Ralstonia eutropha JMP134]
>gi|72384290|ref|YP_293643.1| inner-membrane translocator [Ralstonia eutropha JMP134]
>gi|72384296|ref|YP_293649.1| Mercuric resistence transcriptional repressor protein MerD [Ralstonia eutropha JMP134]
>gi|72384310|ref|YP_293663.1| Conjugal transfer TrbH [Ralstonia eutropha JMP134]

>gi|72384312|ref|YP_293665.1| TrbJ protein of DNA transfer system [Ralstonia eutropha JMP134]
>gi|72384314|ref|YP_293667.1| TrbL/VirB6 plasmid conjugal transfer protein [Ralstonia eutropha JMP134]
>gi|72384316|ref|YP_293669.1| Lytic transglycosylase, catalytic [Ralstonia eutropha JMP134]
>gi|72384317|ref|YP_293670.1| hypothetical protein Reut_D6515 [Ralstonia eutropha JMP134]
>gi|72384318|ref|YP_293671.1| TraX [Ralstonia eutropha JMP134]
>gi|72384319|ref|YP_293672.1| hypothetical protein Reut_D6517 [Ralstonia eutropha JMP134]
>gi|72384330|ref|YP_293683.1| TraK protein of DNA transfer system [Ralstonia eutropha JMP134]
>gi|72384332|ref|YP_293685.1| TraM protein [Ralstonia eutropha JMP134]
>gi|73539707|ref|YP_294227.1| chromosomal replication initiator protein, DnaA [Ralstonia eutropha JMP134]
>gi|73539715|ref|YP_294235.1| hypothetical protein Reut_A0009 [Ralstonia eutropha JMP134]
>gi|73539743|ref|YP_294263.1| probable lipoprotein [Ralstonia eutropha JMP134]
>gi|73539747|ref|YP_294267.1| Drug resistance transporter EmrB/QacA subfamily [Ralstonia eutropha JMP134]
>gi|73539748|ref|YP_294268.1| Glutathione peroxidase [Ralstonia eutropha JMP134]
>gi|73539770|ref|YP_294290.1| Septum formation inhibitor MinC [Ralstonia eutropha JMP134]
>gi|73539772|ref|YP_294292.1| Sel1-like repeat [Ralstonia eutropha JMP134]
>gi|73539784|ref|YP_294304.1| probable rod shape-determining MreD transmembrane protein [Ralstonia eutropha JMP134]
>gi|73539808|ref|YP_294328.1| Biotin--acetyl-CoA-carboxylase ligase [Ralstonia eutropha JMP134]
>gi|73539815|ref|YP_294335.1| hypothetical protein Reut_A0109 [Ralstonia eutropha JMP134]
>gi|73539820|ref|YP_294340.1| putative prolin-rich signal peptide protein [Ralstonia eutropha JMP134]
>gi|73539826|ref|YP_294346.1| cell wall surface anchor family protein [Ralstonia eutropha JMP134]
>gi|73539830|ref|YP_294350.1| Protein of unknown function DUF1328 [Ralstonia eutropha JMP134]
>gi|73539834|ref|YP_294354.1| Sporulation related [Ralstonia eutropha JMP134]
>gi|73539839|ref|YP_294359.1| regulatory protein, MerR [Ralstonia eutropha JMP134]
>gi|73539867|ref|YP_294387.1| Cobalamin synthesis protein/P47K:Cobalamin synthesis CobW, C-terminal [Ralstonia eutropha JMP134]
>gi|73539869|ref|YP_294389.1| Cobalamin synthesis protein/P47K:Cobalamin synthesis CobW, C-terminal [Ralstonia eutropha JMP134]
>gi|73539877|ref|YP_294397.1| hypothetical protein Reut_A0171 [Ralstonia eutropha JMP134]
>gi|73539882|ref|YP_294402.1| Protein of unknown function DUF606 [Ralstonia eutropha JMP134]
>gi|73539889|ref|YP_294409.1| Inner membrane CreD [Ralstonia eutropha JMP134]
>gi|73539913|ref|YP_294433.1| ThiS, thiamine-biosynthesis [Ralstonia eutropha JMP134]
>gi|73539920|ref|YP_294440.1| Membrane protein of unknown function [Ralstonia eutropha JMP134]
>gi|73539937|ref|YP_294457.1| hypothetical protein Reut_A0231 [Ralstonia eutropha JMP134]
>gi|73539942|ref|YP_294462.1| RND efflux system, outer membrane lipoprotein, NodT [Ralstonia eutropha JMP134]
>gi|73539952|ref|YP_294472.1| Cation efflux protein [Ralstonia eutropha JMP134]
>gi|73539962|ref|YP_294482.1| putative membrane protein [Ralstonia eutropha JMP134]
>gi|73539979|ref|YP_294499.1| General substrate transporter:Major facilitator superfamily MFS_1 [Ralstonia eutropha JMP134]
>gi|73539991|ref|YP_294511.1| hypothetical protein Reut_A0285 [Ralstonia eutropha JMP134]
>gi|73539995|ref|YP_294515.1| Protein of unknown function DUF526 [Ralstonia eutropha JMP134]
>gi|73540040|ref|YP_294560.1| Cell division transporter substrate-binding protein FtsY [Ralstonia eutropha JMP134]
>gi|73540057|ref|YP_294577.1| PsiF [Ralstonia eutropha JMP134]
>gi|73540066|ref|YP_294586.1| TrkA-N:TrkA-C:Sodium/hydrogen exchanger [Ralstonia eutropha JMP134]
>gi|73540075|ref|YP_294595.1| Single-strand binding protein [Ralstonia eutropha JMP134]
>gi|73540078|ref|YP_294598.1| Outer membrane autotransporter barrel [Ralstonia eutropha JMP134]
>gi|73540083|ref|YP_294603.1| YadA-like, C-terminal:Haemagluttinin motif:Hep_Hag [Ralstonia eutropha JMP134]
>gi|73540094|ref|YP_294614.1| hypothetical protein Reut_A0388 [Ralstonia eutropha JMP134]
>gi|73540098|ref|YP_294618.1| hypothetical protein Reut_A0392 [Ralstonia eutropha JMP134]
>gi|73540107|ref|YP_294627.1| probable type 4 fimbrial biogenesis related transmembrane protein [Ralstonia eutropha JMP134]
>gi|73540112|ref|YP_294632.1| RND efflux system, outer membrane lipoprotein, NodT [Ralstonia eutropha JMP134]
>gi|73540115|ref|YP_294635.1| Secretion protein HlyD [Ralstonia eutropha JMP134]
>gi|73540124|ref|YP_294644.1| hypothetical protein Reut_A0418 [Ralstonia eutropha JMP134]
>gi|73540138|ref|YP_294658.1| Mitochondrial import inner membrane translocase, subunit Tim44 [Ralstonia eutropha JMP134]
>gi|73540143|ref|YP_294663.1| putative Uncharacterized protein conserved in bacteria [Ralstonia eutropha JMP134]
>gi|73540146|ref|YP_294666.1| probable transmembrane protein [Ralstonia eutropha JMP134]
>gi|73540208|ref|YP_294728.1| Protein of unknown function DUF6, transmembrane [Ralstonia eutropha JMP134]
>gi|73540222|ref|YP_294742.1| CBS:Cl- channel, voltage gated [Ralstonia eutropha JMP134]
>gi|73540223|ref|YP_294743.1| Helicase, C-terminal:Type III restriction enzyme, res subunit:DEAD/DEAH box helicase, N-terminal [Ralstonia eutropha JMP134]
>gi|73540238|ref|YP_294758.1| Integral membrane protein TerC [Ralstonia eutropha JMP134]
>gi|73540239|ref|YP_294759.1| general secretion pathway protein H [Ralstonia eutropha JMP134]
>gi|73540241|ref|YP_294761.1| proline-rich region [Ralstonia eutropha JMP134]
>gi|73540242|ref|YP_294762.1| hypothetical protein Reut_A0536 [Ralstonia eutropha JMP134]
>gi|73540256|ref|YP_294776.1| hypothetical protein Reut_A0550 [Ralstonia eutropha JMP134]
>gi|73540268|ref|YP_294788.1| Protein of unknown function DUF931 [Ralstonia eutropha JMP134]
>gi|73540273|ref|YP_294793.1| regulatory protein, LysR:LysR, substrate-binding [Ralstonia eutropha JMP134]
>gi|73540278|ref|YP_294798.1| Secretion protein HlyD [Ralstonia eutropha JMP134]
>gi|73540294|ref|YP_294814.1| Helix-turn-helix motif [Ralstonia eutropha JMP134]
>gi|73540318|ref|YP_294838.1| conserved hypothetical transmembrane protein [Ralstonia eutropha JMP134]
>gi|73540339|ref|YP_294859.1| Molybdate ABC transporter, permease protein [Ralstonia eutropha JMP134]
>gi|73540349|ref|YP_294869.1| Protein of unknown function DUF898, transmembrane bacterial [Ralstonia eutropha JMP134]
>gi|73540353|ref|YP_294873.1| Enoyl-CoA hydratase/isomerase [Ralstonia eutropha JMP134]
>gi|73540361|ref|YP_294881.1| transport system permease protein [Ralstonia eutropha JMP134]

>gi|73540365|ref|YP_294885.1| Cobalamin-5-phosphate synthase CobS [Ralstonia eutropha JMP134]
>gi|73540369|ref|YP_294889.1| Cobalamin biosynthesis protein CbiB [Ralstonia eutropha JMP134]
>gi|73540381|ref|YP_294901.1| OmpA [Ralstonia eutropha JMP134]
>gi|73540384|ref|YP_294904.1| proline rich protein [Ralstonia eutropha JMP134]
>gi|73540387|ref|YP_294907.1| hypothetical protein Reut_A0681 [Ralstonia eutropha JMP134]
>gi|73540389|ref|YP_294909.1| 4Fe-4S ferredoxin, iron-sulfur binding [Ralstonia eutropha JMP134]
>gi|73540392|ref|YP_294912.1| conserved hypothetical signal peptide protein [Ralstonia eutropha JMP134]
>gi|73540403|ref|YP_294923.1| Sigma-70 region 2:Sigma-70 region 4 [Ralstonia eutropha JMP134]
>gi|73540408|ref|YP_294928.1| HemY, N-terminal:Tetratricopeptide TPR_4 [Ralstonia eutropha JMP134]
>gi|73540431|ref|YP_294951.1| Glycosyl transferase, family 4 [Ralstonia eutropha JMP134]
>gi|73540439|ref|YP_294959.1| hypothetical protein Reut_A0733 [Ralstonia eutropha JMP134]
>gi|73540470|ref|YP_294990.1| Lytic transglycosylase, catalytic [Ralstonia eutropha JMP134]
>gi|73540477|ref|YP_294997.1| hypothetical protein Reut_A0773 [Ralstonia eutropha JMP134]
>gi|73540481|ref|YP_295001.1| putative type 4 fimbrial pilin related transmembrane protein [Ralstonia eutropha JMP134]
>gi|73540486|ref|YP_295006.1| hypothetical protein Reut_A0782 [Ralstonia eutropha JMP134]
>gi|73540498|ref|YP_295018.1| TonB, C-terminal [Ralstonia eutropha JMP134]
>gi|73540506|ref|YP_295026.1| probable transmembrane protein [Ralstonia eutropha JMP134]
>gi|73540507|ref|YP_295027.1| Protein of unknown function DUF883, ElaB [Ralstonia eutropha JMP134]
>gi|73540520|ref|YP_295040.1| AsmA [Ralstonia eutropha JMP134]
>gi|73540525|ref|YP_295045.1| Thioredoxin [Ralstonia eutropha JMP134]
>gi|73540543|ref|YP_295063.1| Fimbrial protein [Ralstonia eutropha JMP134]
>gi|73540548|ref|YP_295068.1| Protein of unknown function DUF1468 [Ralstonia eutropha JMP134]
>gi|73540549|ref|YP_295069.1| Transporter-associated region:TrkA-C:Sodium/hydrogen exchanger [Ralstonia eutropha JMP134]
>gi|73540574|ref|YP_295094.1| putative transmembrane protein [Ralstonia eutropha JMP134]
>gi|73540579|ref|YP_295099.1| Zinc transporter ZIP [Ralstonia eutropha JMP134]
>gi|73540583|ref|YP_295103.1| Exonuclease VII, small subunit [Ralstonia eutropha JMP134]
>gi|73540589|ref|YP_295109.1| GatB/Yqey [Ralstonia eutropha JMP134]
>gi|73540591|ref|YP_295111.1| Sigma-70 region 1.2 [Ralstonia eutropha JMP134]
>gi|73540622|ref|YP_295142.1| Helicase, C-terminal:DEAD/DEAH box helicase, N-terminal [Ralstonia eutropha JMP134]
>gi|73540625|ref|YP_295145.1| Protein of unknown function DUF323 [Ralstonia eutropha JMP134]
>gi|73540636|ref|YP_295156.1| probable transmembrane protein [Ralstonia eutropha JMP134]
>gi|73540637|ref|YP_295157.1| Protein of unknown function DUF214 [Ralstonia eutropha JMP134]
>gi|73540647|ref|YP_295167.1| probable transmembrane protein [Ralstonia eutropha JMP134]
>gi|73540655|ref|YP_295175.1| Peptidoglycan-binding LysM:Transport-associated [Ralstonia eutropha JMP134]
>gi|73540663|ref|YP_295183.1| Preprotein translocase SecG subunit [Ralstonia eutropha JMP134]
>gi|73540673|ref|YP_295193.1| NADH dehydrogenase (quinone) [Ralstonia eutropha JMP134]
>gi|73540696|ref|YP_295216.1| HupE/UreJ protein [Ralstonia eutropha JMP134]
>gi|73540699|ref|YP_295219.1| UreE urease accessory, N-terminal:UreE urease accessory, C-terminal [Ralstonia eutropha JMP134]
>gi|73540707|ref|YP_295227.1| Allergen V5/Tpx-1 related [Ralstonia eutropha JMP134]
>gi|73540719|ref|YP_295239.1| probable lipoprotein transmembrane [Ralstonia eutropha JMP134]
>gi|73540734|ref|YP_295254.1| probable transmembrane protein [Ralstonia eutropha JMP134]
>gi|73540747|ref|YP_295267.1| Protein of unknown function DUF6, transmembrane [Ralstonia eutropha JMP134]
>gi|73540750|ref|YP_295270.1| Electron transport complex, RnfABCDGE type, B subunit [Ralstonia eutropha JMP134]
>gi|73540756|ref|YP_295276.1| hypothetical protein Reut_A1053 [Ralstonia eutropha JMP134]
>gi|73540770|ref|YP_295290.1| putative ABC transporter permease protein [Ralstonia eutropha JMP134]
>gi|73540781|ref|YP_295301.1| ComEC/Rec2-related protein:DNA internalization-related competence protein ComEC/Rec2 [Ralstonia eutropha JMP134]
>gi|73540795|ref|YP_295315.1| hypothetical protein Reut_A1093 [Ralstonia eutropha JMP134]
>gi|73540797|ref|YP_295317.1| transferase hexapeptide repeat [Ralstonia eutropha JMP134]
>gi|73540809|ref|YP_295329.1| hypothetical protein Reut_A1107 [Ralstonia eutropha JMP134]
>gi|73540827|ref|YP_295347.1| PP-loop [Ralstonia eutropha JMP134]
>gi|73540830|ref|YP_295350.1| transferase hexapeptide repeat [Ralstonia eutropha JMP134]
>gi|73540840|ref|YP_295360.1| hypothetical protein Reut_A1140 [Ralstonia eutropha JMP134]
>gi|73540856|ref|YP_295376.1| hypothetical protein Reut_A1157 [Ralstonia eutropha JMP134]
>gi|73540869|ref|YP_295389.1| putative DNA-binding protein H-NS [Ralstonia eutropha JMP134]
>gi|73540877|ref|YP_295397.1| hypothetical protein Reut_A1181 [Ralstonia eutropha JMP134]
>gi|73540884|ref|YP_295404.1| hypothetical protein Reut_A1189 [Ralstonia eutropha JMP134]
>gi|73540903|ref|YP_295423.1| Sulphate anion transporter [Ralstonia eutropha JMP134]
>gi|73540905|ref|YP_295425.1| hypothetical protein Reut_A1210 [Ralstonia eutropha JMP134]
>gi|73540920|ref|YP_295440.1| RND efflux system, outer membrane lipoprotein, NodT [Ralstonia eutropha JMP134]
>gi|73540931|ref|YP_295451.1| Transport-associated [Ralstonia eutropha JMP134]
>gi|73540937|ref|YP_295457.1| Binding-protein-dependent transport systems inner membrane component [Ralstonia eutropha JMP134]
>gi|73540955|ref|YP_295475.1| regulatory protein GntR, HTH:GntR, C-terminal [Ralstonia eutropha JMP134]
>gi|73540960|ref|YP_295480.1| TRAP dicarboxylate transporter, DctM subunit [Ralstonia eutropha JMP134]
>gi|73540971|ref|YP_295491.1| Ribosomal protein L35 [Ralstonia eutropha JMP134]
>gi|73540980|ref|YP_295500.1| hypothetical protein Reut_A1286 [Ralstonia eutropha JMP134]
>gi|73540985|ref|YP_295505.1| conserved hypothetical signal peptide protein [Ralstonia eutropha JMP134]
>gi|73540990|ref|YP_295510.1| Sulfate transporter/antisigma-factor antagonist STAS:Xanthine/uracil/vitamin C permease:Sulphate transporter [Ralstonia eutropha JMP134]
>gi|73540994|ref|YP_295514.1| putative membrane protein [Ralstonia eutropha JMP134]

>gi|73540998|ref|YP_295518.1| Dihydrolipoamide acetyltransferase [Ralstonia eutropha JMP134]
>gi|73541002|ref|YP_295522.1| regulatory protein GntR, HTH:GntR, C-terminal [Ralstonia eutropha JMP134]
>gi|73541008|ref|YP_295528.1| inner-membrane translocator [Ralstonia eutropha JMP134]
>gi|73541025|ref|YP_295545.1| inner-membrane translocator [Ralstonia eutropha JMP134]
>gi|73541034|ref|YP_295554.1| hypothetical protein Reut_A1340 [Ralstonia eutropha JMP134]
>gi|73541048|ref|YP_295568.1| putative signal peptide protein [Ralstonia eutropha JMP134]
>gi|73541063|ref|YP_295583.1| putative transmembrane protein [Ralstonia eutropha JMP134]
>gi|73541068|ref|YP_295588.1| hypothetical protein Reut_A1374 [Ralstonia eutropha JMP134]
>gi|73541103|ref|YP_295623.1| Short-chain dehydrogenase/reductase SDR [Ralstonia eutropha JMP134]
>gi|73541111|ref|YP_295631.1| Appr-1-p processing [Ralstonia eutropha JMP134]
>gi|73541122|ref|YP_295642.1| regulatory protein GntR, HTH:GntR, C-terminal [Ralstonia eutropha JMP134]
>gi|73541139|ref|YP_295659.1| Cell divisionFtsK/SpoIIIE [Ralstonia eutropha JMP134]
>gi|73541142|ref|YP_295662.1| putative acyl coenzyme A thioester hydrolase protein [Ralstonia eutropha JMP134]
>gi|73541148|ref|YP_295668.1| Twin-arginine translocation pathway signal [Ralstonia eutropha JMP134]
>gi|73541183|ref|YP_295703.1| ProQ activator of osmoprotectant transporter ProP [Ralstonia eutropha JMP134]
>gi|73541187|ref|YP_295707.1| hypothetical protein Reut_A1496 [Ralstonia eutropha JMP134]
>gi|73541206|ref|YP_295726.1| regulatory protein, TetR [Ralstonia eutropha JMP134]
>gi|73541207|ref|YP_295727.1| hypothetical protein Reut_A1516 [Ralstonia eutropha JMP134]
>gi|73541213|ref|YP_295733.1| Protein of unknown function DUF6, transmembrane [Ralstonia eutropha JMP134]
>gi|73541227|ref|YP_295747.1| Lipolytic enzyme [Ralstonia eutropha JMP134]
>gi|73541228|ref|YP_295748.1| Short-chain dehydrogenase/reductase SDR:Glucose/ribitol dehydrogenase [Ralstonia eutropha JMP134]
>gi|73541254|ref|YP_295774.1| Acyl-CoA dehydrogenase, C-terminal [Ralstonia eutropha JMP134]
>gi|73541258|ref|YP_295778.1| Acyl-CoA dehydrogenase, C-terminal [Ralstonia eutropha JMP134]
>gi|73541274|ref|YP_295794.1| Flavin reductase-like, FMN-binding [Ralstonia eutropha JMP134]
>gi|73541301|ref|YP_295821.1| Malonate transporter MadL subunit [Ralstonia eutropha JMP134]
>gi|73541310|ref|YP_295830.1| Major facilitator superfamily MFS_1 [Ralstonia eutropha JMP134]
>gi|73541316|ref|YP_295836.1| hypothetical protein Reut_A1626 [Ralstonia eutropha JMP134]
>gi|73541320|ref|YP_295840.1| hypothetical protein Reut_A1630 [Ralstonia eutropha JMP134]
>gi|73541328|ref|YP_295848.1| conserved hypothetical signal peptide protein [Ralstonia eutropha JMP134]
>gi|73541333|ref|YP_295853.1| hypothetical protein Reut_A1643 [Ralstonia eutropha JMP134]
>gi|73541336|ref|YP_295856.1| von Willebrand factor, type A [Ralstonia eutropha JMP134]
>gi|73541342|ref|YP_295862.1| inner-membrane translocator [Ralstonia eutropha JMP134]
>gi|73541369|ref|YP_295889.1| NADH/Ubiquinone/plastoquinone (complex I) [Ralstonia eutropha JMP134]
>gi|73541370|ref|YP_295890.1| cation antiporter [Ralstonia eutropha JMP134]
>gi|73541396|ref|YP_295916.1| hypothetical protein Reut_A1707 [Ralstonia eutropha JMP134]
>gi|73541398|ref|YP_295918.1| Major facilitator superfamily MFS_1 [Ralstonia eutropha JMP134]
>gi|73541408|ref|YP_295928.1| Sodium:dicarboxylate symporter [Ralstonia eutropha JMP134]
>gi|73541411|ref|YP_295931.1| extracellular solute-binding protein, family 3 [Ralstonia eutropha JMP134]
>gi|73541412|ref|YP_295932.1| hypothetical protein Reut_A1723 [Ralstonia eutropha JMP134]
>gi|73541417|ref|YP_295937.1| Protein of unknown function DUF1305 [Ralstonia eutropha JMP134]
>gi|73541427|ref|YP_295947.1| membrane protein, putative [Ralstonia eutropha JMP134]
>gi|73541440|ref|YP_295960.1| hypothetical protein Reut_A1751 [Ralstonia eutropha JMP134]
>gi|73541455|ref|YP_295975.1| histone protein [Ralstonia eutropha JMP134]
>gi|73541457|ref|YP_295977.1| hypothetical protein Reut_A1768 [Ralstonia eutropha JMP134]
>gi|73541461|ref|YP_295981.1| 17 kDa surface antigen-like protein [Ralstonia eutropha JMP134]
>gi|73541465|ref|YP_295985.1| extracellular solute-binding protein, family 3 [Ralstonia eutropha JMP134]
>gi|73541475|ref|YP_295995.1| ATP-binding region, ATPase-like:Histidine kinase, HAMP region:Histidine kinase, dimerisation and phosphoacceptor region [Ralstonia eutropha JMP134]
>gi|73541490|ref|YP_296010.1| MscS Mechanosensitive ion channel [Ralstonia eutropha JMP134]
>gi|73541506|ref|YP_296026.1| Secretion protein HlyD [Ralstonia eutropha JMP134]
>gi|73541544|ref|YP_296064.1| probable transmembrane protein [Ralstonia eutropha JMP134]
>gi|73541547|ref|YP_296067.1| probable transmembrane protein [Ralstonia eutropha JMP134]
>gi|73541551|ref|YP_296071.1| Band 7 protein [Ralstonia eutropha JMP134]
>gi|73541552|ref|YP_296072.1| Protein of unknown function DUF107 [Ralstonia eutropha JMP134]
>gi|73541561|ref|YP_296081.1| Outer membrane chaperone Skp (OmpH) [Ralstonia eutropha JMP134]
>gi|73541573|ref|YP_296093.1| Pseudouridine synthase, Rsu [Ralstonia eutropha JMP134]
>gi|73541576|ref|YP_296096.1| conserved hypothetical transmembrane protein [Ralstonia eutropha JMP134]
>gi|73541579|ref|YP_296099.1| Protein of unknown function DUF6, transmembrane [Ralstonia eutropha JMP134]
>gi|73541582|ref|YP_296102.1| Twin-arginine translocation pathway signal [Ralstonia eutropha JMP134]
>gi|73541587|ref|YP_296107.1| hypothetical protein Reut_A1898 [Ralstonia eutropha JMP134]
>gi|73541630|ref|YP_296150.1| Drug resistance transporter EmrB/QacA subfamily:Twin-arginine translocation pathway signal [Ralstonia eutropha JMP134]
>gi|73541638|ref|YP_296158.1| hypothetical protein Reut_A1950 [Ralstonia eutropha JMP134]
>gi|73541645|ref|YP_296165.1| chemotaxis sensory transducer [Ralstonia eutropha JMP134]
>gi|73541648|ref|YP_296168.1| Peptidase M48, Ste24p [Ralstonia eutropha JMP134]
>gi|73541663|ref|YP_296183.1| Binding-protein-dependent transport systems inner membrane component [Ralstonia eutropha JMP134]
>gi|73541671|ref|YP_296191.1| inner-membrane translocator [Ralstonia eutropha JMP134]
>gi|73541678|ref|YP_296198.1| Molybdopterin converting factor, subunit 1 [Ralstonia eutropha JMP134]
>gi|73541716|ref|YP_296236.1| Initiation factor 2:Small GTP-binding protein domain [Ralstonia eutropha JMP134]
>gi|73541719|ref|YP_296239.1| Pseudouridine synthase, Rsu [Ralstonia eutropha JMP134]

>gi|73541720|ref|YP_296240.1| Prokaryotic chromosome segregation and condensation protein ScpB [Ralstonia eutropha JMP134]
>gi|73541721|ref|YP_296241.1| probable transmembrane protein [Ralstonia eutropha JMP134]
>gi|73541734|ref|YP_296254.1| Dihydrolipoamide succinyltransferase [Ralstonia eutropha JMP134]
>gi|73541749|ref|YP_296269.1| probable transmembrane protein [Ralstonia eutropha JMP134]
>gi|73541775|ref|YP_296295.1| Helix-turn-helix motif [Ralstonia eutropha JMP134]
>gi|73541784|ref|YP_296304.1| Peptidoglycan-binding LysM:Peptidase M23B [Ralstonia eutropha JMP134]
>gi|73541785|ref|YP_296305.1| Protein-L-isoaspartate(D-aspartate) O-methyltransferase [Ralstonia eutropha JMP134]
>gi|73541800|ref|YP_296320.1| AAA ATPase, central region [Ralstonia eutropha JMP134]
>gi|73541820|ref|YP_296340.1| General substrate transporter:Major facilitator superfamily MFS_1 [Ralstonia eutropha JMP134]
>gi|73541843|ref|YP_296363.1| conserved hypothetical transmembrane protein [Ralstonia eutropha JMP134]
>gi|73541864|ref|YP_296384.1| Lysine exporter protein (LYSE/YGGA) [Ralstonia eutropha JMP134]
>gi|73541896|ref|YP_296416.1| ABC transporter related [Ralstonia eutropha JMP134]
>gi|73541905|ref|YP_296425.1| probable signal peptide protein [Ralstonia eutropha JMP134]
>gi|73541922|ref|YP_296442.1| TonB, C-terminal [Ralstonia eutropha JMP134]
>gi|73541925|ref|YP_296445.1| hypothetical protein Reut_A2238 [Ralstonia eutropha JMP134]
>gi|73541927|ref|YP_296447.1| conserved hypothetical signal peptide protein [Ralstonia eutropha JMP134]
>gi|73541936|ref|YP_296456.1| Pyridoxal phosphate biosynthetic protein PdxJ [Ralstonia eutropha JMP134]
>gi|73541962|ref|YP_296482.1| Ribonuclease E and G [Ralstonia eutropha JMP134]
>gi|73541982|ref|YP_296502.1| ABC-2 type transporter, NodJ [Ralstonia eutropha JMP134]
>gi|73541983|ref|YP_296503.1| hypothetical protein Reut_A2297 [Ralstonia eutropha JMP134]
>gi|73541986|ref|YP_296506.1| Colicin V production protein [Ralstonia eutropha JMP134]
>gi|73541995|ref|YP_296515.1| Peptidoglycan-binding LysM [Ralstonia eutropha JMP134]
>gi|73542007|ref|YP_296527.1| Protein of unknown function DUF339 [Ralstonia eutropha JMP134]
>gi|73542033|ref|YP_296553.1| Protein of unknown function DUF6, transmembrane [Ralstonia eutropha JMP134]
>gi|73542038|ref|YP_296558.1| Lysine exporter protein (LYSE/YGGA) [Ralstonia eutropha JMP134]
>gi|73542041|ref|YP_296561.1| Chromate transporter [Ralstonia eutropha JMP134]
>gi|73542048|ref|YP_296568.1| Benzoate membrane transport protein [Ralstonia eutropha JMP134]
>gi|73542060|ref|YP_296580.1| hypothetical protein Reut_A2374 [Ralstonia eutropha JMP134]
>gi|73542069|ref|YP_296589.1| hypothetical protein Reut_A2383 [Ralstonia eutropha JMP134]
>gi|73542098|ref|YP_296618.1| hypothetical protein Reut_A2412 [Ralstonia eutropha JMP134]
>gi|73542105|ref|YP_296625.1| hypothetical protein Reut_A2419 [Ralstonia eutropha JMP134]
>gi|73542124|ref|YP_296644.1| hypothetical protein Reut_A2438 [Ralstonia eutropha JMP134]
>gi|73542128|ref|YP_296648.1| probable lipoprotein transmembrane [Ralstonia eutropha JMP134]
>gi|73542132|ref|YP_296652.1| probable signal peptide protein [Ralstonia eutropha JMP134]
>gi|73542138|ref|YP_296658.1| PAS [Ralstonia eutropha JMP134]
>gi|73542166|ref|YP_296686.1| 17 kDa surface antigen-like protein [Ralstonia eutropha JMP134]
>gi|73542171|ref|YP_296691.1| Copper resistance D [Ralstonia eutropha JMP134]
>gi|73542172|ref|YP_296692.1| Copper resistance protein CopC [Ralstonia eutropha JMP134]
>gi|73542175|ref|YP_296695.1| Heavy metal response regulator [Ralstonia eutropha JMP134]
>gi|73542181|ref|YP_296701.1| Binding-protein-dependent transport systems inner membrane component [Ralstonia eutropha JMP134]
>gi|73542202|ref|YP_296722.1| Cytochrome c, class I [Ralstonia eutropha JMP134]
>gi|73542211|ref|YP_296731.1| Iojap-related protein [Ralstonia eutropha JMP134]
>gi|73542234|ref|YP_296754.1| Electron transfer flavoprotein beta-subunit:Electron transfer flavoprotein, alpha subunit [Ralstonia eutropha JMP134]
>gi|73542237|ref|YP_296757.1| Binding-protein-dependent transport systems inner membrane component [Ralstonia eutropha JMP134]
>gi|73542247|ref|YP_296767.1| hypothetical protein Reut_A2562 [Ralstonia eutropha JMP134]
>gi|73542252|ref|YP_296772.1| probable transmembrane protein [Ralstonia eutropha JMP134]
>gi|73542264|ref|YP_296784.1| OmpA/MotB [Ralstonia eutropha JMP134]
>gi|73542277|ref|YP_296797.1| Phosphoesterase, PA-phosphatase related [Ralstonia eutropha JMP134]
>gi|73542278|ref|YP_296798.1| putative Uncharacterized protein conserved in bacteria [Ralstonia eutropha JMP134]
>gi|73542282|ref|YP_296802.1| hypothetical protein Reut_A2597 [Ralstonia eutropha JMP134]
>gi|73542303|ref|YP_296823.1| hypothetical protein Reut_A2618 [Ralstonia eutropha JMP134]
>gi|73542304|ref|YP_296824.1| Response regulator receiver:CheW-like protein:ATP-binding region, ATPase-like:Hpt [Ralstonia eutropha JMP134]
>gi|73542325|ref|YP_296845.1| Flp/Fap pilin component [Ralstonia eutropha JMP134]
>gi|73542326|ref|YP_296846.1| Peptidase A24A, prepilin type IV [Ralstonia eutropha JMP134]
>gi|73542329|ref|YP_296849.1| type II and III secretion system protein:Transport-associated [Ralstonia eutropha JMP134]
>gi|73542336|ref|YP_296856.1| putative Spermidine synthase [Ralstonia eutropha JMP134]
>gi|73542338|ref|YP_296858.1| TadE-like [Ralstonia eutropha JMP134]
>gi|73542343|ref|YP_296863.1| conserved hypothetical signal peptide protein [Ralstonia eutropha JMP134]
>gi|73542348|ref|YP_296868.1| Acyltransferase 3 [Ralstonia eutropha JMP134]
>gi|73542359|ref|YP_296879.1| CBS:Cl- channel, voltage gated [Ralstonia eutropha JMP134]
>gi|73542361|ref|YP_296881.1| Protein of unknown function UPF0118 [Ralstonia eutropha JMP134]
>gi|73542375|ref|YP_296895.1| DoxX [Ralstonia eutropha JMP134]
>gi|73542409|ref|YP_296929.1| Calcium-binding EF-hand [Ralstonia eutropha JMP134]
>gi|73542443|ref|YP_296963.1| hypothetical protein Reut_A2758 [Ralstonia eutropha JMP134]
>gi|73542450|ref|YP_296970.1| Ribosomal protein S20p [Ralstonia eutropha JMP134]
>gi|73542459|ref|YP_296979.1| Protein of unknown function UPF0118 [Ralstonia eutropha JMP134]
>gi|73542471|ref|YP_296991.1| Cobalamin adenosyltransferase [Ralstonia eutropha JMP134]
>gi|73542482|ref|YP_297002.1| putative transcriptional regulator [Ralstonia eutropha JMP134]
>gi|73542521|ref|YP_297041.1| SmpA/OmlA [Ralstonia eutropha JMP134]

>gi|73542529|ref|YP_297049.1| Protein of unknown function DUF937, bacterial [Ralstonia eutropha JMP134]
>gi|73542537|ref|YP_297057.1| Protein of unknown function DUF6, transmembrane [Ralstonia eutropha JMP134]
>gi|73542550|ref|YP_297070.1| Acetyl-CoA biotin carboxyl carrier [Ralstonia eutropha JMP134]
>gi|73542553|ref|YP_297073.1| MJ0042 finger-like region [Ralstonia eutropha JMP134]
>gi|73542556|ref|YP_297076.1| 17 kDa surface antigen-like protein [Ralstonia eutropha JMP134]
>gi|73542557|ref|YP_297077.1| probable histone H1 protein [Ralstonia eutropha JMP134]
>gi|73542558|ref|YP_297078.1| hypothetical protein Reut_A2873 [Ralstonia eutropha JMP134]
>gi|73542569|ref|YP_297089.1| Protein of unknown function DUF894, DitE:Major facilitator superfamily MFS_1 [Ralstonia eutropha JMP134]
>gi|73542571|ref|YP_297091.1| hypothetical protein Reut_A2886 [Ralstonia eutropha JMP134]
>gi|73542574|ref|YP_297094.1| hypothetical protein Reut_A2889 [Ralstonia eutropha JMP134]
>gi|73542577|ref|YP_297097.1| Spermine synthase [Ralstonia eutropha JMP134]
>gi|73542590|ref|YP_297110.1| Sodium/hydrogen exchanger [Ralstonia eutropha JMP134]
>gi|73542597|ref|YP_297117.1| hypothetical protein Reut_A2913 [Ralstonia eutropha JMP134]
>gi|73542600|ref|YP_297120.1| hypothetical protein Reut_A2916 [Ralstonia eutropha JMP134]
>gi|73542602|ref|YP_297122.1| conserved hypothetical signal peptide protein [Ralstonia eutropha JMP134]
>gi|73542603|ref|YP_297123.1| hypothetical protein Reut_A2919 [Ralstonia eutropha JMP134]
>gi|73542607|ref|YP_297127.1| hypothetical protein Reut_A2923 [Ralstonia eutropha JMP134]
>gi|73542614|ref|YP_297134.1| Filamentous haemagglutinin, N-terminal:Adhesin HecA 20-residue repeat x2 [Ralstonia eutropha JMP134]
>gi|73542618|ref|YP_297138.1| hypothetical protein Reut_A2934 [Ralstonia eutropha JMP134]
>gi|73542627|ref|YP_297147.1| Helix-turn-helix, Fis-type [Ralstonia eutropha JMP134]
>gi|73542630|ref|YP_297150.1| Cytochrome c assembly protein [Ralstonia eutropha JMP134]
>gi|73542646|ref|YP_297166.1| Prepilin peptidase [Ralstonia eutropha JMP134]
>gi|73542650|ref|YP_297170.1| Oligopeptide transporter OPT superfamily:Oligopeptide transporter OPT [Ralstonia eutropha JMP134]
>gi|73542670|ref|YP_297190.1| Cell division protein, FtsL -like [Ralstonia eutropha JMP134]
>gi|73542674|ref|YP_297194.1| Porin, Gram-negative type [Ralstonia eutropha JMP134]
>gi|73542677|ref|YP_297197.1| hypothetical protein Reut_A2993 [Ralstonia eutropha JMP134]
>gi|73542697|ref|YP_297217.1| Amino acid ABC transporter, permease protein, 3-TM region, His/Glu/Gln/Arg/opine [Ralstonia eutropha JMP134]
>gi|73542699|ref|YP_297219.1| Phenylacetate degradation probable enoyl-CoA hydratase paaB [Ralstonia eutropha JMP134]
>gi|73542723|ref|YP_297243.1| hypothetical protein Reut_A3039 [Ralstonia eutropha JMP134]
>gi|73542724|ref|YP_297244.1| putative membrane protein [Ralstonia eutropha JMP134]
>gi|73542726|ref|YP_297246.1| conserved hypothetical transmembrane protein [Ralstonia eutropha JMP134]
>gi|73542729|ref|YP_297249.1| outer membrane protein [Ralstonia eutropha JMP134]
>gi|73542733|ref|YP_297253.1| hypothetical protein Reut_A3049 [Ralstonia eutropha JMP134]
>gi|73542738|ref|YP_297258.1| conserved hypothetical signal peptide protein [Ralstonia eutropha JMP134]
>gi|73542744|ref|YP_297264.1| Multi antimicrobial extrusion protein MatE [Ralstonia eutropha JMP134]
>gi|73542748|ref|YP_297268.1| Carbohydrate-binding family V/XII [Ralstonia eutropha JMP134]
>gi|73542766|ref|YP_297286.1| Cytochrome c, class I [Ralstonia eutropha JMP134]
>gi|73542801|ref|YP_297321.1| Sulfate transporter/antisigma-factor antagonist STAS [Ralstonia eutropha JMP134]
>gi|73542803|ref|YP_297323.1| VacJ-like lipoprotein [Ralstonia eutropha JMP134]
>gi|73542807|ref|YP_297327.1| Uncharacterized protein UPF0065 [Ralstonia eutropha JMP134]
>gi|73542835|ref|YP_297355.1| Protein-disulfide reductase [Ralstonia eutropha JMP134]
>gi|73542846|ref|YP_297366.1| Ribosomal protein S5, bacterial and organelle form [Ralstonia eutropha JMP134]
>gi|73542847|ref|YP_297367.1| Ribosomal protein L18 [Ralstonia eutropha JMP134]
>gi|73542874|ref|YP_297394.1| Ribosomal protein L7/L12 [Ralstonia eutropha JMP134]
>gi|73542879|ref|YP_297399.1| SecE subunit of protein translocation complex [Ralstonia eutropha JMP134]
>gi|73542886|ref|YP_297406.1| hypothetical protein Reut_A3202 [Ralstonia eutropha JMP134]
>gi|73542897|ref|YP_297417.1| Potassium efflux system protein [Ralstonia eutropha JMP134]
>gi|73542902|ref|YP_297422.1| hypothetical protein Reut_A3218 [Ralstonia eutropha JMP134]
>gi|73542910|ref|YP_297430.1| General secretion pathway L [Ralstonia eutropha JMP134]
>gi|73542912|ref|YP_297432.1| probable general secretory pathway N transmembrane protein [Ralstonia eutropha JMP134]
>gi|73542916|ref|YP_297436.1| hypothetical protein Reut_A3232 [Ralstonia eutropha JMP134]
>gi|73542921|ref|YP_297441.1| Major facilitator superfamily MFS_1 [Ralstonia eutropha JMP134]
>gi|73542922|ref|YP_297442.1| Major facilitator superfamily MFS_1 [Ralstonia eutropha JMP134]
>gi|73542927|ref|YP_297447.1| ABC transporter related [Ralstonia eutropha JMP134]
>gi|73542928|ref|YP_297448.1| Cation efflux protein [Ralstonia eutropha JMP134]
>gi|73542945|ref|YP_297465.1| Cytochrome c, class I [Ralstonia eutropha JMP134]
>gi|73542949|ref|YP_297469.1| Protein of unknown function UPF0011 [Ralstonia eutropha JMP134]
>gi|73542973|ref|YP_297493.1| hypothetical protein Reut_A3290 [Ralstonia eutropha JMP134]
>gi|73542980|ref|YP_297500.1| hypothetical protein Reut_A3297 [Ralstonia eutropha JMP134]
>gi|73542985|ref|YP_297505.1| putative transmembrane protein [Ralstonia eutropha JMP134]
>gi|73542989|ref|YP_297509.1| hypothetical protein Reut_A3306 [Ralstonia eutropha JMP134]
>gi|73543003|ref|YP_297523.1| hypothetical protein Reut_A3321 [Ralstonia eutropha JMP134]
>gi|73543004|ref|YP_297524.1| Cytochrome c, class I [Ralstonia eutropha JMP134]
>gi|73543007|ref|YP_297527.1| ATP-binding region, ATPase-like:Histidine kinase, HAMP region:Histidine kinase A, N-terminal [Ralstonia eutropha JMP134]
>gi|73543010|ref|YP_297530.1| hypothetical protein Reut_A3328 [Ralstonia eutropha JMP134]
>gi|73543017|ref|YP_297537.1| hypothetical protein Reut_A3335 [Ralstonia eutropha JMP134]
>gi|73543019|ref|YP_297539.1| Sodium/hydrogen exchanger [Ralstonia eutropha JMP134]
>gi|73543032|ref|YP_297552.1| ATP synthase F0, subunit B [Ralstonia eutropha JMP134]
>gi|73543045|ref|YP_297565.1| inner-membrane translocator [Ralstonia eutropha JMP134]

>gi|73543047|ref|YP_297567.1| Protein of unknown function DUF81 [Ralstonia eutropha JMP134]
>gi|73543048|ref|YP_297568.1| conserved hypothetical transmembrane protein [Ralstonia eutropha JMP134]
>gi|73543050|ref|YP_297570.1| inner-membrane translocator [Ralstonia eutropha JMP134]
>gi|73543058|ref|YP_297578.1| ATPase, E1-E2 type:Copper-translocating P-type ATPase:Heavy metal translocating P-type ATPase [Ralstonia eutropha JMP134]
>gi|73543077|ref|YP_297597.1| RarD protein [Ralstonia eutropha JMP134]
>gi|73543079|ref|YP_297599.1| hypothetical protein Reut_A3397 [Ralstonia eutropha JMP134]
>gi|73543080|ref|YP_297600.1| Major intrinsic protein [Ralstonia eutropha JMP134]
>gi|73543081|ref|YP_297601.1| Lysine exporter protein (LYSE/YGGA) [Ralstonia eutropha JMP134]
>gi|73543084|ref|YP_297604.1| Helix-turn-helix, Fis-type [Ralstonia eutropha JMP134]
>gi|73543086|ref|YP_297606.1| probable proline rich signal peptide protein [Ralstonia eutropha JMP134]
>gi|73543096|ref|YP_297616.1| hypothetical protein Reut_A3414 [Ralstonia eutropha JMP134]
>gi|73543097|ref|YP_297617.1| 17 kDa surface antigen-like protein [Ralstonia eutropha JMP134]
>gi|73543104|ref|YP_297624.1| Major facilitator superfamily MFS_1 [Ralstonia eutropha JMP134]
>gi|73543122|ref|YP_297642.1| Secretion protein HlyD [Ralstonia eutropha JMP134]
>gi|73543128|ref|YP_297648.1| regulatory protein, TetR [Ralstonia eutropha JMP134]
>gi|73543134|ref|YP_297654.1| Glycosyl transferase, family 28 [Ralstonia eutropha JMP134]
>gi|73543144|ref|YP_297664.1| ribonuclease P protein [Ralstonia eutropha JMP134]
>gi|73537310|ref|YP_297677.1| Molybdenum-pterin binding protein [Ralstonia eutropha JMP134]
>gi|73537315|ref|YP_297682.1| Cytochrome c, class I [Ralstonia eutropha JMP134]
>gi|73537317|ref|YP_297684.1| MscS Mechanosensitive ion channel [Ralstonia eutropha JMP134]
>gi|73537318|ref|YP_297685.1| hypothetical protein Reut_B3483 [Ralstonia eutropha JMP134]
>gi|73537321|ref|YP_297688.1| NAD-dependent epimerase/dehydratase:Short-chain dehydrogenase/reductase SDR [Ralstonia eutropha JMP134]
>gi|73537337|ref|YP_297704.1| Secretion protein HlyD [Ralstonia eutropha JMP134]
>gi|73537339|ref|YP_297706.1| hypothetical protein Reut_B3504 [Ralstonia eutropha JMP134]
>gi|73537358|ref|YP_297725.1| hypothetical protein Reut_B3523 [Ralstonia eutropha JMP134]
>gi|73537374|ref|YP_297741.1| HpcH/HpaI aldolase [Ralstonia eutropha JMP134]
>gi|73537426|ref|YP_297793.1| Protein of unknown function DUF81 [Ralstonia eutropha JMP134]
>gi|73537432|ref|YP_297799.1| Carbon monoxide dehydrogenase subunit G [Ralstonia eutropha JMP134]
>gi|73537458|ref|YP_297825.1| hypothetical protein Reut_B3623 [Ralstonia eutropha JMP134]
>gi|73537461|ref|YP_297828.1| Esterase, PHB depolymerase [Ralstonia eutropha JMP134]
>gi|73537465|ref|YP_297832.1| putative membrane protein [Ralstonia eutropha JMP134]
>gi|73537467|ref|YP_297834.1| Cytochrome c oxidase, subunit II:Cytochrome c, class I [Ralstonia eutropha JMP134]
>gi|73537469|ref|YP_297836.1| cytochrome c oxidase subunit III [Ralstonia eutropha JMP134]
>gi|73537478|ref|YP_297845.1| Protein of unknown function DUF1468 [Ralstonia eutropha JMP134]
>gi|73537486|ref|YP_297853.1| Protein-glutamate O-methyltransferase [Ralstonia eutropha JMP134]
>gi|73537490|ref|YP_297857.1| putative Chromosome segregation ATPases [Ralstonia eutropha JMP134]
>gi|73537498|ref|YP_297865.1| Short-chain dehydrogenase/reductase SDR [Ralstonia eutropha JMP134]
>gi|73537500|ref|YP_297867.1| Major facilitator superfamily MFS_1 [Ralstonia eutropha JMP134]
>gi|73537507|ref|YP_297874.1| Arsenical pump membrane protein [Ralstonia eutropha JMP134]
>gi|73537510|ref|YP_297877.1| hypothetical protein Reut_B3675 [Ralstonia eutropha JMP134]
>gi|73537514|ref|YP_297881.1| Protein of unknown function UPF0060 [Ralstonia eutropha JMP134]
>gi|73537529|ref|YP_297896.1| Lysine exporter protein (LYSE/YGGA) [Ralstonia eutropha JMP134]
>gi|73537543|ref|YP_297910.1| Enoyl-CoA hydratase/isomerase [Ralstonia eutropha JMP134]
>gi|73537545|ref|YP_297912.1| Acyl-CoA dehydrogenase, C-terminal [Ralstonia eutropha JMP134]
>gi|73537605|ref|YP_297972.1| Outer membrane autotransporter barrel [Ralstonia eutropha JMP134]
>gi|73537614|ref|YP_297981.1| inner-membrane translocator [Ralstonia eutropha JMP134]
>gi|73537615|ref|YP_297982.1| inner-membrane translocator [Ralstonia eutropha JMP134]
>gi|73537626|ref|YP_297993.1| Molybdopterin dehydrogenase, FAD-binding [Ralstonia eutropha JMP134]
>gi|73537631|ref|YP_297998.1| Protein of unknown function DUF305 [Ralstonia eutropha JMP134]
>gi|73537636|ref|YP_298003.1| hypothetical protein Reut_B3801 [Ralstonia eutropha JMP134]
>gi|73537640|ref|YP_298007.1| Major facilitator superfamily MFS_1 [Ralstonia eutropha JMP134]
>gi|73537641|ref|YP_298008.1| Outer membrane autotransporter barrel [Ralstonia eutropha JMP134]
>gi|73537645|ref|YP_298012.1| Major facilitator superfamily MFS_1 [Ralstonia eutropha JMP134]
>gi|73537646|ref|YP_298013.1| hypothetical protein Reut_B3811 [Ralstonia eutropha JMP134]
>gi|73537651|ref|YP_298018.1| Protein of unknown function DUF6, transmembrane [Ralstonia eutropha JMP134]
>gi|73537652|ref|YP_298019.1| hypothetical protein Reut_B3817 [Ralstonia eutropha JMP134]
>gi|73537668|ref|YP_298035.1| hypothetical protein Reut_B3833 [Ralstonia eutropha JMP134]
>gi|73537671|ref|YP_298038.1| Acyl-CoA dehydrogenase, C-terminal:Acyl-CoA dehydrogenase, central region [Ralstonia eutropha JMP134]
>gi|73537686|ref|YP_298053.1| Short-chain dehydrogenase/reductase SDR:Glucose/ribitol dehydrogenase [Ralstonia eutropha JMP134]
>gi|73537699|ref|YP_298066.1| Phasin [Ralstonia eutropha JMP134]
>gi|73537741|ref|YP_298108.1| hypothetical protein Reut_B3907 [Ralstonia eutropha JMP134]
>gi|73537762|ref|YP_298129.1| Polysaccharide deacetylase [Ralstonia eutropha JMP134]
>gi|73537763|ref|YP_298130.1| Phosphoglucomutase/phosphomannomutase alpha/beta/alpha domain III [Ralstonia eutropha JMP134]
>gi|73537766|ref|YP_298133.1| AMP-dependent synthetase and ligase [Ralstonia eutropha JMP134]
>gi|73537767|ref|YP_298134.1| probable transmembrane protein [Ralstonia eutropha JMP134]
>gi|73537771|ref|YP_298138.1| Cupin region [Ralstonia eutropha JMP134]
>gi|73537777|ref|YP_298144.1| Rickettsia 17 kDa surface antigen [Ralstonia eutropha JMP134]
>gi|73537787|ref|YP_298154.1| Sodium/hydrogen exchanger [Ralstonia eutropha JMP134]
>gi|73537791|ref|YP_298158.1| hypothetical protein Reut_B3958 [Ralstonia eutropha JMP134]

>gi|73537793|ref|YP_298160.1| hypothetical protein Reut_B3960 [Ralstonia eutropha JMP134]
>gi|73537795|ref|YP_298162.1| Heavy metal sensor kinase [Ralstonia eutropha JMP134]
>gi|73537805|ref|YP_298172.1| ATPase, E1-E2 type:Heavy metal-(Cd/Co/Hg/Pb/Zn)-translocating P-type ATPase:Heavy metal translocating P-type ATPase [Ralstonia eutropha JMP134]
>gi|73537811|ref|YP_298178.1| putative small multidrug resistance transmembrane protein [Ralstonia eutropha JMP134]
>gi|73537813|ref|YP_298180.1| conserved hypothetical transmembrane protein [Ralstonia eutropha JMP134]
>gi|73537815|ref|YP_298182.1| hypothetical protein Reut_B3982 [Ralstonia eutropha JMP134]
>gi|73537824|ref|YP_298191.1| inner-membrane translocator [Ralstonia eutropha JMP134]
>gi|73537825|ref|YP_298192.1| inner-membrane translocator [Ralstonia eutropha JMP134]
>gi|73537828|ref|YP_298195.1| hypothetical protein Reut_B3995 [Ralstonia eutropha JMP134]
>gi|73537834|ref|YP_298201.1| Histidine kinase, HAMP region:Bacterial chemotaxis sensory transducer [Ralstonia eutropha JMP134]
>gi|73537835|ref|YP_298202.1| Major facilitator superfamily MFS_1 [Ralstonia eutropha JMP134]
>gi|73537841|ref|YP_298208.1| inner-membrane translocator [Ralstonia eutropha JMP134]
>gi|73537847|ref|YP_298214.1| hypothetical protein Reut_B4014 [Ralstonia eutropha JMP134]
>gi|73537865|ref|YP_298232.1| conserved hypothetical signal peptide protein [Ralstonia eutropha JMP134]
>gi|73537868|ref|YP_298235.1| hypothetical protein Reut_B4035 [Ralstonia eutropha JMP134]
>gi|73537869|ref|YP_298236.1| Drug resistance transporter Bcr/CflA subfamily [Ralstonia eutropha JMP134]
>gi|73537872|ref|YP_298239.1| Histone-like bacterial DNA-binding protein [Ralstonia eutropha JMP134]
>gi|73537880|ref|YP_298247.1| ATP-binding region, ATPase-like:Histidine kinase, HAMP region:Histidine kinase A, N-terminal [Ralstonia eutropha JMP134]
>gi|73537884|ref|YP_298251.1| Histone-like bacterial DNA-binding protein [Ralstonia eutropha JMP134]
>gi|73537885|ref|YP_298252.1| 5-carboxymethyl-2-hydroxymuconate isomerase [Ralstonia eutropha JMP134]
>gi|73537886|ref|YP_298253.1| MgtC/SapB transporter [Ralstonia eutropha JMP134]
>gi|73537891|ref|YP_298258.1| regulatory protein, TetR [Ralstonia eutropha JMP134]
>gi|73537892|ref|YP_298259.1| lipoprotein, putative [Ralstonia eutropha JMP134]
>gi|73537896|ref|YP_298263.1| hypothetical protein Reut_B4063 [Ralstonia eutropha JMP134]
>gi|73537906|ref|YP_298273.1| Amidase [Ralstonia eutropha JMP134]
>gi|73537912|ref|YP_298279.1| hypothetical protein Reut_B4079 [Ralstonia eutropha JMP134]
>gi|73537914|ref|YP_298281.1| RND efflux system, outer membrane lipoprotein, NodT [Ralstonia eutropha JMP134]
>gi|73537916|ref|YP_298283.1| Fusaric acid resistance protein conserved region [Ralstonia eutropha JMP134]
>gi|73537931|ref|YP_298298.1| probable efflux transporter [Ralstonia eutropha JMP134]
>gi|73537932|ref|YP_298299.1| hypothetical protein Reut_B4099 [Ralstonia eutropha JMP134]
>gi|73537938|ref|YP_298305.1| hypothetical protein Reut_B4105 [Ralstonia eutropha JMP134]
>gi|73537948|ref|YP_298315.1| PAS [Ralstonia eutropha JMP134]
>gi|73537961|ref|YP_298328.1| UDP-glucose/GDP-mannose dehydrogenase:Prephenate dehydrogenase:NADP oxidoreductase, coenzyme F420-dependent:6-phosphogluconate dehydrogenase, NAD-binding [Ralstonia eutropha JMP134]
>gi|73537965|ref|YP_298332.1| inner-membrane translocator [Ralstonia eutropha JMP134]
>gi|73537966|ref|YP_298333.1| inner-membrane translocator [Ralstonia eutropha JMP134]
>gi|73537973|ref|YP_298340.1| Uncharacterized protein UPF0065 [Ralstonia eutropha JMP134]
>gi|73538016|ref|YP_298383.1| alkylphosphonate utilization protein PhnG [Ralstonia eutropha JMP134]
>gi|73538020|ref|YP_298387.1| ATPase [Ralstonia eutropha JMP134]
>gi|73538026|ref|YP_298393.1| ABC transporter related [Ralstonia eutropha JMP134]
>gi|73538032|ref|YP_298399.1| hypothetical protein Reut_B4202 [Ralstonia eutropha JMP134]
>gi|73538050|ref|YP_298417.1| General substrate transporter:Major facilitator superfamily MFS_1 [Ralstonia eutropha JMP134]
>gi|73538054|ref|YP_298421.1| putative membrane protein [Ralstonia eutropha JMP134]
>gi|73538066|ref|YP_298433.1| Transport-associated [Ralstonia eutropha JMP134]
>gi|73538067|ref|YP_298434.1| hypothetical protein Reut_B4237 [Ralstonia eutropha JMP134]
>gi|73538069|ref|YP_298436.1| hypothetical protein Reut_B4239 [Ralstonia eutropha JMP134]
>gi|73538103|ref|YP_298470.1| Transcriptional regulator PadR-like [Ralstonia eutropha JMP134]
>gi|73538122|ref|YP_298489.1| hypothetical protein Reut_B4292 [Ralstonia eutropha JMP134]
>gi|73538124|ref|YP_298491.1| hypothetical protein Reut_B4294 [Ralstonia eutropha JMP134]
>gi|73538135|ref|YP_298502.1| hypothetical protein Reut_B4305 [Ralstonia eutropha JMP134]
>gi|73538143|ref|YP_298510.1| Major facilitator superfamily MFS_1 [Ralstonia eutropha JMP134]
>gi|73538144|ref|YP_298511.1| hypothetical protein Reut_B4314 [Ralstonia eutropha JMP134]
>gi|73538146|ref|YP_298513.1| Glyoxalase/bleomycin resistance protein/dioxygenase [Ralstonia eutropha JMP134]
>gi|73538148|ref|YP_298515.1| Protein of unknown function DUF1228:Major facilitator superfamily MFS_1 [Ralstonia eutropha JMP134]
>gi|73538153|ref|YP_298520.1| Major facilitator superfamily MFS_1 [Ralstonia eutropha JMP134]
>gi|73538158|ref|YP_298525.1| hypothetical protein Reut_B4328 [Ralstonia eutropha JMP134]
>gi|73538161|ref|YP_298528.1| hypothetical protein Reut_B4331 [Ralstonia eutropha JMP134]
>gi|73538168|ref|YP_298535.1| Copper/Zinc superoxide dismutase [Ralstonia eutropha JMP134]
>gi|73538176|ref|YP_298543.1| Heat shock protein DnaJ, N-terminal [Ralstonia eutropha JMP134]
>gi|73538184|ref|YP_298551.1| hypothetical protein Reut_B4355 [Ralstonia eutropha JMP134]
>gi|73538190|ref|YP_298557.1| hypothetical protein Reut_B4361 [Ralstonia eutropha JMP134]
>gi|73538204|ref|YP_298571.1| Transport-associated [Ralstonia eutropha JMP134]
>gi|73538215|ref|YP_298582.1| Lipopolysaccharide heptosyltransferase II [Ralstonia eutropha JMP134]
>gi|73538228|ref|YP_298595.1| Sodium/calcium exchanger membrane region [Ralstonia eutropha JMP134]
>gi|73538253|ref|YP_298620.1| TonB, C-terminal [Ralstonia eutropha JMP134]
>gi|73538260|ref|YP_298627.1| conserved hypothetical protein-signal peptide and transmembrane prediction [Ralstonia eutropha JMP134]
>gi|73538266|ref|YP_298633.1| Caa(3)-type oxidase, subunit IV [Ralstonia eutropha JMP134]
>gi|73538315|ref|YP_298682.1| Major facilitator superfamily MFS_1 [Ralstonia eutropha JMP134]

>gi|73538332|ref|YP_298699.1| Twin-arginine translocation pathway signal [Ralstonia eutropha JMP134]
>gi|73538336|ref|YP_298703.1| hypothetical protein Reut_B4509 [Ralstonia eutropha JMP134]
>gi|73538352|ref|YP_298719.1| hypothetical protein Reut_B4525 [Ralstonia eutropha JMP134]
>gi|73538354|ref|YP_298721.1| regulatory protein, MarR [Ralstonia eutropha JMP134]
>gi|73538371|ref|YP_298738.1| Protein of unknown function DUF81 [Ralstonia eutropha JMP134]
>gi|73538385|ref|YP_298752.1| Acyl-CoA dehydrogenase, C-terminal [Ralstonia eutropha JMP134]
>gi|73538387|ref|YP_298754.1| Enoyl-CoA hydratase/isomerase [Ralstonia eutropha JMP134]
>gi|73538388|ref|YP_298755.1| Thiolase [Ralstonia eutropha JMP134]
>gi|73538389|ref|YP_298756.1| Acyl-CoA dehydrogenase, C-terminal [Ralstonia eutropha JMP134]
>gi|73538401|ref|YP_298768.1| hypothetical protein Reut_B4574 [Ralstonia eutropha JMP134]
>gi|73538413|ref|YP_298780.1| hypothetical protein Reut_B4586 [Ralstonia eutropha JMP134]
>gi|73538418|ref|YP_298785.1| Transport-associated [Ralstonia eutropha JMP134]
>gi|73538419|ref|YP_298786.1| hypothetical protein Reut_B4592 [Ralstonia eutropha JMP134]
>gi|73538435|ref|YP_298802.1| hypothetical protein Reut_B4608 [Ralstonia eutropha JMP134]
>gi|73538458|ref|YP_298825.1| MscS Mechanosensitive ion channel [Ralstonia eutropha JMP134]
>gi|73538459|ref|YP_298826.1| hypothetical protein Reut_B4632 [Ralstonia eutropha JMP134]
>gi|73538474|ref|YP_298841.1| hypothetical protein Reut_B4648 [Ralstonia eutropha JMP134]
>gi|73538476|ref|YP_298843.1| hypothetical protein Reut_B4650 [Ralstonia eutropha JMP134]
>gi|73538493|ref|YP_298860.1| NAD(P) transhydrogenase, alpha subunit [Ralstonia eutropha JMP134]
>gi|73538532|ref|YP_298899.1| hypothetical protein Reut_B4707 [Ralstonia eutropha JMP134]
>gi|73538564|ref|YP_298931.1| hypothetical protein Reut_B4739 [Ralstonia eutropha JMP134]
>gi|73538567|ref|YP_298934.1| TrkA-N:Sodium/hydrogen exchanger [Ralstonia eutropha JMP134]
>gi|73538592|ref|YP_298959.1| Cytochrome c-type biogenesis protein CcmB [Ralstonia eutropha JMP134]
>gi|73538595|ref|YP_298962.1| Cytochrome c-type biogenesis protein CcmF [Ralstonia eutropha JMP134]
>gi|73538597|ref|YP_298964.1| Cytochrome C biogenesis protein [Ralstonia eutropha JMP134]
>gi|73538617|ref|YP_298984.1| regulatory protein GntR, HTH:GntR, C-terminal [Ralstonia eutropha JMP134]
>gi|73538641|ref|YP_299008.1| Electron transfer flavoprotein beta-subunit [Ralstonia eutropha JMP134]
>gi|73538642|ref|YP_299009.1| Electron transfer flavoprotein beta-subunit:Electron transfer flavoprotein, alpha subunit [Ralstonia eutropha JMP134]
>gi|73538664|ref|YP_299031.1| hypothetical protein Reut_B4839 [Ralstonia eutropha JMP134]
>gi|73538670|ref|YP_299037.1| General substrate transporter:Major facilitator superfamily MFS_1 [Ralstonia eutropha JMP134]
>gi|73538672|ref|YP_299039.1| DoxX [Ralstonia eutropha JMP134]
>gi|73538676|ref|YP_299043.1| Protein of unknown function DUF1275 [Ralstonia eutropha JMP134]
>gi|73538682|ref|YP_299049.1| GCN5-related N-acetyltransferase [Ralstonia eutropha JMP134]
>gi|73538684|ref|YP_299051.1| Major facilitator superfamily MFS_1 [Ralstonia eutropha JMP134]
>gi|73538702|ref|YP_299069.1| hypothetical protein Reut_B4877 [Ralstonia eutropha JMP134]
>gi|73538705|ref|YP_299072.1| Protein of unknown function DUF6, transmembrane [Ralstonia eutropha JMP134]
>gi|73538728|ref|YP_299095.1| hypothetical protein Reut_B4903 [Ralstonia eutropha JMP134]
>gi|73538730|ref|YP_299097.1| membrane protein, putative [Ralstonia eutropha JMP134]
>gi|73538732|ref|YP_299099.1| Peptidylprolyl isomerase, FKBP-type [Ralstonia eutropha JMP134]
>gi|73538748|ref|YP_299115.1| hypothetical protein Reut_B4923 [Ralstonia eutropha JMP134]
>gi|73538751|ref|YP_299118.1| Triphosphoribosyl-dephospho-CoA synthase [Ralstonia eutropha JMP134]
>gi|73538761|ref|YP_299128.1| regulatory protein, TetR [Ralstonia eutropha JMP134]
>gi|73538762|ref|YP_299129.1| acyl-CoA dehydrogenase-related protein [Ralstonia eutropha JMP134]
>gi|73538767|ref|YP_299134.1| Peptidoglycan-binding LysM [Ralstonia eutropha JMP134]
>gi|73538785|ref|YP_299152.1| GGDEF [Ralstonia eutropha JMP134]
>gi|73538786|ref|YP_299153.1| regulatory protein, IclR [Ralstonia eutropha JMP134]
>gi|73538787|ref|YP_299154.1| hypothetical protein Reut_B4962 [Ralstonia eutropha JMP134]
>gi|73538822|ref|YP_299189.1| Outer membrane autotransporter barrel [Ralstonia eutropha JMP134]
>gi|73538824|ref|YP_299191.1| NnrS [Ralstonia eutropha JMP134]
>gi|73538836|ref|YP_299203.1| Cytochrome c, class I [Ralstonia eutropha JMP134]
>gi|73538848|ref|YP_299215.1| Delta crystallin [Ralstonia eutropha JMP134]
>gi|73538852|ref|YP_299219.1| General substrate transporter:Major facilitator superfamily MFS_1 [Ralstonia eutropha JMP134]
>gi|73538865|ref|YP_299232.1| hypothetical protein Reut_B5040 [Ralstonia eutropha JMP134]
>gi|73538873|ref|YP_299240.1| hypothetical protein Reut_B5048 [Ralstonia eutropha JMP134]
>gi|73538888|ref|YP_299255.1| hypothetical protein Reut_B5063 [Ralstonia eutropha JMP134]
>gi|73538892|ref|YP_299259.1| hypothetical protein Reut_B5067 [Ralstonia eutropha JMP134]
>gi|73538898|ref|YP_299265.1| putative membrane protein [Ralstonia eutropha JMP134]
>gi|73538899|ref|YP_299266.1| hypothetical protein Reut_B5074 [Ralstonia eutropha JMP134]
>gi|73538905|ref|YP_299272.1| hypothetical protein Reut_B5080 [Ralstonia eutropha JMP134]
>gi|73538909|ref|YP_299276.1| hypothetical protein Reut_B5084 [Ralstonia eutropha JMP134]
>gi|73538912|ref|YP_299279.1| Histidine kinase, HAMP region:Bacterial chemotaxis sensory transducer [Ralstonia eutropha JMP134]
>gi|73538913|ref|YP_299280.1| hypothetical protein Reut_B5088 [Ralstonia eutropha JMP134]
>gi|73538922|ref|YP_299289.1| Flagellar FliF M-ring protein [Ralstonia eutropha JMP134]
>gi|73538927|ref|YP_299294.1| Flagellar hook-length control protein [Ralstonia eutropha JMP134]
>gi|73538931|ref|YP_299298.1| Protein of unknown function DUF81 [Ralstonia eutropha JMP134]
>gi|73538932|ref|YP_299299.1| Protein of unknown function DUF938 [Ralstonia eutropha JMP134]
>gi|73538934|ref|YP_299301.1| Histidine kinase, HAMP region:Bacterial chemotaxis sensory transducer [Ralstonia eutropha JMP134]
>gi|73538940|ref|YP_299307.1| putative cytochrome p450 oxidoreductase [Ralstonia eutropha JMP134]
>gi|73538953|ref|YP_299320.1| 2-nitropropane dioxygenase, NPD [Ralstonia eutropha JMP134]

>gi|73538960|ref|YP_299327.1| Metal-dependent phosphohydrolase, HD subdomain [Ralstonia eutropha JMP134]
>gi|73538972|ref|YP_299339.1| 4Fe-4S ferredoxin, iron-sulfur binding [Ralstonia eutropha JMP134]
>gi|73538985|ref|YP_299352.1| hypothetical protein Reut_B5161 [Ralstonia eutropha JMP134]
>gi|73538986|ref|YP_299353.1| probable signal peptide protein [Ralstonia eutropha JMP134]
>gi|73538995|ref|YP_299362.1| Secretion protein HlyD [Ralstonia eutropha JMP134]
>gi|73539000|ref|YP_299367.1| probable signal peptide protein [Ralstonia eutropha JMP134]
>gi|73539004|ref|YP_299371.1| Endoribonuclease L-PSP [Ralstonia eutropha JMP134]
>gi|73539025|ref|YP_299392.1| Phospholipase D/Transphosphatidylase [Ralstonia eutropha JMP134]
>gi|73539038|ref|YP_299405.1| PepSY-associated TM helix [Ralstonia eutropha JMP134]
>gi|73539039|ref|YP_299406.1| Porin, Gram-negative type [Ralstonia eutropha JMP134]
>gi|73539051|ref|YP_299418.1| Major facilitator superfamily MFS_1 [Ralstonia eutropha JMP134]
>gi|73539052|ref|YP_299419.1| Chromate transporter [Ralstonia eutropha JMP134]
>gi|73539061|ref|YP_299428.1| hypothetical protein Reut_B5238 [Ralstonia eutropha JMP134]
>gi|73539073|ref|YP_299440.1| regulatory protein, LysR:LysR, substrate-binding [Ralstonia eutropha JMP134]
>gi|73539076|ref|YP_299443.1| Protein of unknown function DUF6, transmembrane [Ralstonia eutropha JMP134]
>gi|73539128|ref|YP_299495.1| putative membrane protein [Ralstonia eutropha JMP134]
>gi|73539143|ref|YP_299510.1| Oligopeptide transporter OPT superfamily [Ralstonia eutropha JMP134]
>gi|73539174|ref|YP_299541.1| hypothetical protein Reut_B5352 [Ralstonia eutropha JMP134]
>gi|73539175|ref|YP_299542.1| regulatory protein, LuxR [Ralstonia eutropha JMP134]
>gi|73539203|ref|YP_299570.1| probable signal peptide protein [Ralstonia eutropha JMP134]
>gi|73539204|ref|YP_299571.1| Major facilitator superfamily MFS_1 [Ralstonia eutropha JMP134]
>gi|73539212|ref|YP_299579.1| probable glycin-rich signal peptide protein [Ralstonia eutropha JMP134]
>gi|73539213|ref|YP_299580.1| conserved hypothetical signal peptide protein [Ralstonia eutropha JMP134]
>gi|73539215|ref|YP_299582.1| hypothetical protein Reut_B5393 [Ralstonia eutropha JMP134]
>gi|73539216|ref|YP_299583.1| hypothetical protein Reut_B5394 [Ralstonia eutropha JMP134]
>gi|73539225|ref|YP_299592.1| putative hemagglutinin-related transmembrane protein [Ralstonia eutropha JMP134]
>gi|73539226|ref|YP_299593.1| putative hemagglutinin-related transmembrane protein [Ralstonia eutropha JMP134]
>gi|73539227|ref|YP_299594.1| Flp/Fap pilin component [Ralstonia eutropha JMP134]
>gi|73539228|ref|YP_299595.1| Peptidase A24A, prepilin type IV [Ralstonia eutropha JMP134]
>gi|73539229|ref|YP_299596.1| TadE-like [Ralstonia eutropha JMP134]
>gi|73539235|ref|YP_299602.1| type II secretion system protein [Ralstonia eutropha JMP134]
>gi|73539236|ref|YP_299603.1| TPR repeat [Ralstonia eutropha JMP134]
>gi|73539237|ref|YP_299604.1| putative lipoprotein [Ralstonia eutropha JMP134]
>gi|73539238|ref|YP_299605.1| probable transmembrane protein [Ralstonia eutropha JMP134]
>gi|73539245|ref|YP_299612.1| NADP oxidoreductase, coenzyme F420-dependent:6-phosphogluconate dehydrogenase, NAD-binding [Ralstonia eutropha JMP134]
>gi|73539249|ref|YP_299616.1| hypothetical protein Reut_B5427 [Ralstonia eutropha JMP134]
>gi|73539250|ref|YP_299617.1| putative transmembrane protein [Ralstonia eutropha JMP134]
>gi|73539269|ref|YP_299636.1| Protein of unknown function DUF162 [Ralstonia eutropha JMP134]
>gi|73539276|ref|YP_299643.1| hypothetical protein Reut_B5454 [Ralstonia eutropha JMP134]
>gi|73539280|ref|YP_299647.1| hypothetical protein Reut_B5458 [Ralstonia eutropha JMP134]
>gi|73539303|ref|YP_299670.1| General substrate transporter:Major facilitator superfamily MFS_1 [Ralstonia eutropha JMP134]
>gi|73539341|ref|YP_299708.1| putative cytoplasmic protein [Ralstonia eutropha JMP134]
>gi|73539366|ref|YP_299733.1| hypothetical protein Reut_B5544 [Ralstonia eutropha JMP134]
>gi|73539387|ref|YP_299754.1| Transglutaminase-like [Ralstonia eutropha JMP134]
>gi|73539389|ref|YP_299756.1| ATPase associated with various cellular activities, AAA_3 [Ralstonia eutropha JMP134]
>gi|73539394|ref|YP_299761.1| hypothetical protein Reut_B5572 [Ralstonia eutropha JMP134]
>gi|73539395|ref|YP_299762.1| Protein of unknown function YGGT [Ralstonia eutropha JMP134]
>gi|73539397|ref|YP_299764.1| Rickettsia 17 kDa surface antigen [Ralstonia eutropha JMP134]
>gi|73539405|ref|YP_299772.1| exonuclease SbcC, putative [Ralstonia eutropha JMP134]
>gi|73539424|ref|YP_299791.1| Histidine kinase, HAMP region:Bacterial chemotaxis sensory transducer [Ralstonia eutropha JMP134]
>gi|73539462|ref|YP_299829.1| Secretion protein HlyD [Ralstonia eutropha JMP134]
>gi|73539471|ref|YP_299838.1| Major facilitator superfamily MFS_1 [Ralstonia eutropha JMP134]
>gi|73539476|ref|YP_299843.1| Secretion protein HlyD [Ralstonia eutropha JMP134]
>gi|73539478|ref|YP_299845.1| hypothetical protein Reut_B5656 [Ralstonia eutropha JMP134]
>gi|73539510|ref|YP_299877.1| Protein of unknown function DUF336 [Ralstonia eutropha JMP134]
>gi|73539514|ref|YP_299881.1| 4-oxalocrotonate decarboxylase [Ralstonia eutropha JMP134]
>gi|73539518|ref|YP_299885.1| Carboxymuconolactone decarboxylase [Ralstonia eutropha JMP134]
>gi|73539568|ref|YP_299935.1| hypothetical protein Reut_B5748 [Ralstonia eutropha JMP134]
>gi|73539617|ref|YP_299984.1| Protein of unknown function DUF894, DitE:Major facilitator superfamily MFS_1 [Ralstonia eutropha JMP134]
>gi|73539645|ref|YP_300012.1| hypothetical protein Reut_B5825 [Ralstonia eutropha JMP134]
>gi|73539666|ref|YP_300033.1| YadA-like, C-terminal:Haemagluttinin motif:Hep_Hag [Ralstonia eutropha JMP134]
>gi|73539680|ref|YP_300047.1| General substrate transporter:Major facilitator superfamily MFS_1 [Ralstonia eutropha JMP134]
>gi|73539701|ref|YP_300068.1| Flagellar transport protein FliP [Ralstonia eutropha JMP134]
>gi|73539702|ref|YP_300069.1| Flagellar biosynthesis protein, FliO [Ralstonia eutropha JMP134]
>gi|73539703|ref|YP_300070.1| Type III secretion system outer membrane O protein [Ralstonia eutropha JMP134]
>gi|51593953|ref|YP_068519.1| TriE protein [Yersinia pseudotuberculosis IP 32953]
>gi|51593960|ref|YP_068526.1| hypothetical protein pYptb0018 [Yersinia pseudotuberculosis IP 32953]
>gi|51593962|ref|YP_068528.1| DNA-binding protein [Yersinia pseudotuberculosis IP 32953]
>gi|51594417|ref|YP_068608.1| putative membrane protein [Yersinia pseudotuberculosis IP 32953]

>gi|51594450|ref|YP_068641.1| hypothetical protein YPTB0092 [Yersinia pseudotuberculosis IP 32953]
>gi|51594457|ref|YP_068648.1| cell division protein [Yersinia pseudotuberculosis IP 32953]
>gi|51594488|ref|YP_068679.1| ABC ribose/sugar transporter, permease domain [Yersinia pseudotuberculosis IP 32953]
>gi|51594538|ref|YP_068729.1| putative uroporphyrin-III C-methyltransferase [Yersinia pseudotuberculosis IP 32953]
>gi|51594571|ref|YP_068762.1| putative membrane protein [Yersinia pseudotuberculosis IP 32953]
>gi|51594573|ref|YP_068764.1| putative membrane protein [Yersinia pseudotuberculosis IP 32953]
>gi|51594576|ref|YP_068767.1| cell division protein [Yersinia pseudotuberculosis IP 32953]
>gi|51594583|ref|YP_068774.1| ABC type branched-chain aa transport system, permease [Yersinia pseudotuberculosis IP 32953]
>gi|51594608|ref|YP_068799.1| putative membrane protein [Yersinia pseudotuberculosis IP 32953]
>gi|51594609|ref|YP_068800.1| putative DNA recombination protein [Yersinia pseudotuberculosis IP 32953]
>gi|51594614|ref|YP_068805.1| Sec-independent protein translocase protein TatB [Yersinia pseudotuberculosis IP 32953]
>gi|51594632|ref|YP_068823.1| preprotein translocase SecE subunit [Yersinia pseudotuberculosis IP 32953]
>gi|51594636|ref|YP_068827.1| 50S ribosomal protein L10 [Yersinia pseudotuberculosis IP 32953]
>gi|51594637|ref|YP_068828.1| 50S ribosomal protein L7/L12 [Yersinia pseudotuberculosis IP 32953]
>gi|51594675|ref|YP_068866.1| hypothetical protein YPTB0320 [Yersinia pseudotuberculosis IP 32953]
>gi|51594676|ref|YP_068867.1| putative type III secretion system apparatus protein [Yersinia pseudotuberculosis IP 32953]
>gi|51594679|ref|YP_068870.1| type III secretion system apparatus protein [Yersinia pseudotuberculosis IP 32953]
>gi|51594692|ref|YP_068883.1| ABC hemin transporter, permease subunit HmuU [Yersinia pseudotuberculosis IP 32953]
>gi|51594727|ref|YP_068918.1| hypothetical protein YPTB0373 [Yersinia pseudotuberculosis IP 32953]
>gi|51594734|ref|YP_068925.1| single-strand binding protein [Yersinia pseudotuberculosis IP 32953]
>gi|51594743|ref|YP_068934.1| hypothetical protein YPTB0389 [Yersinia pseudotuberculosis IP 32953]
>gi|51594755|ref|YP_068946.1| putative membrane protein [Yersinia pseudotuberculosis IP 32953]
>gi|51594761|ref|YP_068952.1| putative SugE efflux pump [Yersinia pseudotuberculosis IP 32953]
>gi|51594779|ref|YP_068970.1| putative membrane protein [Yersinia pseudotuberculosis IP 32953]
>gi|51594829|ref|YP_069020.1| protein-export membrane protein segG [Yersinia pseudotuberculosis IP 32953]
>gi|51594838|ref|YP_069029.1| cold-shock dead-box protein A [Yersinia pseudotuberculosis IP 32953]
>gi|51594872|ref|YP_069063.1| ABC oligo-dipeptide/nickel transporter, ATP binding subunit [Yersinia pseudotuberculosis IP 32953]
>gi|51594902|ref|YP_069093.1| ABC sugar transporter, permease subunit [Yersinia pseudotuberculosis IP 32953]
>gi|51594922|ref|YP_069113.1| putative membrane protein [Yersinia pseudotuberculosis IP 32953]
>gi|51594928|ref|YP_069119.1| hypothetical protein YPTB0577 [Yersinia pseudotuberculosis IP 32953]
>gi|51594931|ref|YP_069122.1| putative Na+ dependent nucleoside transporter-family protein [Yersinia pseudotuberculosis IP 32953]
>gi|51594963|ref|YP_069154.1| heat shock protein, DnaJ and GrpE stimulates ATPase activity of DnaK [Yersinia pseudotuberculosis IP 32953]
>gi|51594999|ref|YP_069190.1| hypothetical protein YPTB0648 [Yersinia pseudotuberculosis IP 32953]
>gi|51595064|ref|YP_069255.1| pyruvate dehydrogenase, dihydrolipoyltransacetylase component [Yersinia pseudotuberculosis IP 32953]
>gi|51595091|ref|YP_069282.1| ABC type hydroxamate-dependent iron uptake, with duplicated permease domains [Yersinia pseudotuberculosis IP 32953]
>gi|51595093|ref|YP_069284.1| putative CLC family, chlorine transport protein [Yersinia pseudotuberculosis IP 32953]
>gi|51595150|ref|YP_069341.1| putative ABC sugar transporter, permease subunit [Yersinia pseudotuberculosis IP 32953]
>gi|51595174|ref|YP_069365.1| putative regulatory protein [Yersinia pseudotuberculosis IP 32953]
>gi|51595178|ref|YP_069369.1| putative integral membrane protein [Yersinia pseudotuberculosis IP 32953]
>gi|51595204|ref|YP_069395.1| putative tRNA/rRNA methyltransferase [Yersinia pseudotuberculosis IP 32953]
>gi|51595265|ref|YP_069456.1| putative ATP-dependent dsDNA exonuclease [Yersinia pseudotuberculosis IP 32953]
>gi|51595327|ref|YP_069518.1| putative 6-O-methylguanine DNA methyltransferase family protein [Yersinia pseudotuberculosis IP 32953]
>gi|51595373|ref|YP_069564.1| putative membrane protein [Yersinia pseudotuberculosis IP 32953]
>gi|51595374|ref|YP_069565.1| hypothetical protein YPTB1025 [Yersinia pseudotuberculosis IP 32953]
>gi|51595375|ref|YP_069566.1| putative thioredoxin [Yersinia pseudotuberculosis IP 32953]
>gi|51595379|ref|YP_069570.1| putative permease [Yersinia pseudotuberculosis IP 32953]
>gi|51595423|ref|YP_069614.1| ABC type sugar transport system permease protein [Yersinia pseudotuberculosis IP 32953]
>gi|51595437|ref|YP_069628.1| sec-independent protein translocase protein [Yersinia pseudotuberculosis IP 32953]
>gi|51595502|ref|YP_069693.1| TolA colicin import membrane protein [Yersinia pseudotuberculosis IP 32953]
>gi|51595505|ref|YP_069696.1| conserved putative exported protein [Yersinia pseudotuberculosis IP 32953]
>gi|51595516|ref|YP_069707.1| putative membrane protein [Yersinia pseudotuberculosis IP 32953]
>gi|51595559|ref|YP_069750.1| hypothetical protein YPTB1215 [Yersinia pseudotuberculosis IP 32953]
>gi|51595566|ref|YP_069757.1| putative membrane protein [Yersinia pseudotuberculosis IP 32953]
>gi|51595573|ref|YP_069764.1| ABC type branched-chain amino acid transporter, permease protein [Yersinia pseudotuberculosis IP 32953]
>gi|51595574|ref|YP_069765.1| ABC type branched-chain amino acid transporter, permease protein [Yersinia pseudotuberculosis IP 32953]
>gi|51595581|ref|YP_069772.1| ABC type choline/glycine/betaine transporter, permease subunit [Yersinia pseudotuberculosis IP 32953]
>gi|51595752|ref|YP_069943.1| hypothetical protein YPTB1412 [Yersinia pseudotuberculosis IP 32953]
>gi|51595753|ref|YP_069944.1| hypothetical protein YPTB1413 [Yersinia pseudotuberculosis IP 32953]
>gi|51595786|ref|YP_069977.1| putative lipoprotein protein [Yersinia pseudotuberculosis IP 32953]
>gi|51595794|ref|YP_069985.1| putative cell division inhibitor [Yersinia pseudotuberculosis IP 32953]
>gi|51595842|ref|YP_070033.1| hypothetical protein YPTB1505 [Yersinia pseudotuberculosis IP 32953]
>gi|51595870|ref|YP_070061.1| putative membrane protein [Yersinia pseudotuberculosis IP 32953]
>gi|51595884|ref|YP_070075.1| putative ABC iron-siderophore transporter, permease subunit [Yersinia pseudotuberculosis IP 32953]
>gi|51595937|ref|YP_070128.1| putative signal transducer or MFS Superfamily transporter [Yersinia pseudotuberculosis IP 32953]
>gi|51595954|ref|YP_070145.1| hypothetical protein YPTB1618 [Yersinia pseudotuberculosis IP 32953]
>gi|51595958|ref|YP_070149.1| hypothetical protein YPTB1622 [Yersinia pseudotuberculosis IP 32953]
>gi|51596028|ref|YP_070219.1| flagellar biosynthetic protein FliQ [Yersinia pseudotuberculosis IP 32953]
>gi|51596034|ref|YP_070225.1| flagellar hook-length control protein FliK [Yersinia pseudotuberculosis IP 32953]
>gi|51596089|ref|YP_070280.1| putative Orf27; P2 LysB homolog; control of lysis [Ente... [Yersinia pseudotuberculosis IP 32953]

>gi|51596091|ref|YP_070282.1| putative prophage Hp1 family Holin [Yersinia pseudotuberculosis IP 32953]
>gi|51596145|ref|YP_070336.1| putative small subunit bacteriophage terminase [Yersinia pseudotuberculosis IP 32953]
>gi|51596148|ref|YP_070339.1| putative phage l protein [Yersinia pseudotuberculosis IP 32953]
>gi|51596164|ref|YP_070355.1| bacteriophage hypothetical protein [Yersinia pseudotuberculosis IP 32953]
>gi|51596186|ref|YP_070377.1| putative (AB008550) orf12; similar to LysB gene of P2:l... [Yersinia pseudotuberculosis IP 32953]
>gi|51596201|ref|YP_070392.1| hypothetical protein YPTB1866 [Yersinia pseudotuberculosis IP 32953]
>gi|51596212|ref|YP_070403.1| hypothetical protein YPTB1880 [Yersinia pseudotuberculosis IP 32953]
>gi|51596348|ref|YP_070539.1| MFS multidrug efflux antiporter, yceL [Yersinia pseudotuberculosis IP 32953]
>gi|51596404|ref|YP_070595.1| possible DMT superfamliy Ni++ efflux protein [Yersinia pseudotuberculosis IP 32953]
>gi|51596427|ref|YP_070618.1| DNA-binding protein Hns [Yersinia pseudotuberculosis IP 32953]
>gi|51596443|ref|YP_070634.1| TonB protein [Yersinia pseudotuberculosis IP 32953]
>gi|51596477|ref|YP_070668.1| osmotically inducible lipoprotein B precursor [Yersinia pseudotuberculosis IP 32953]
>gi|51596487|ref|YP_070678.1| putative membrane protein [Yersinia pseudotuberculosis IP 32953]
>gi|51596491|ref|YP_070682.1| putative iron-sulfur binding NADH dehydrogenase [Yersinia pseudotuberculosis IP 32953]
>gi|51596494|ref|YP_070685.1| putative membrane protein [Yersinia pseudotuberculosis IP 32953]
>gi|51596561|ref|YP_070752.1| acid shock protein [Yersinia pseudotuberculosis IP 32953]
>gi|51596574|ref|YP_070765.1| putative lipoprotein [Yersinia pseudotuberculosis IP 32953]
>gi|51596611|ref|YP_070802.1| putative lipoprotein [Yersinia pseudotuberculosis IP 32953]
>gi|51596622|ref|YP_070813.1| hypothetical protein YPTB2298 [Yersinia pseudotuberculosis IP 32953]
>gi|51596631|ref|YP_070822.1| major outer membrane lipoprotein [Yersinia pseudotuberculosis IP 32953]
>gi|51596648|ref|YP_070839.1| putative membrane protein [Yersinia pseudotuberculosis IP 32953]
>gi|51596664|ref|YP_070855.1| 50S ribosomal protein L35 [Yersinia pseudotuberculosis IP 32953]
>gi|51596698|ref|YP_070889.1| putative fimbrial protein [Yersinia pseudotuberculosis IP 32953]
>gi|51596701|ref|YP_070892.1| ProP effector homologue [Yersinia pseudotuberculosis IP 32953]
>gi|51596705|ref|YP_070896.1| hypothetical protein YPTB2381 [Yersinia pseudotuberculosis IP 32953]
>gi|51596717|ref|YP_070908.1| putative surface protein [Yersinia pseudotuberculosis IP 32953]
>gi|51596729|ref|YP_070920.1| chemotaxis MotB protein [Yersinia pseudotuberculosis IP 32953]
>gi|51596766|ref|YP_070957.1| hypothetical protein YPTB2444 [Yersinia pseudotuberculosis IP 32953]
>gi|51596773|ref|YP_070964.1| putative lipoprotein [Yersinia pseudotuberculosis IP 32953]
>gi|51596787|ref|YP_070978.1| DNA polymerase III, delta' subunit [Yersinia pseudotuberculosis IP 32953]
>gi|51596801|ref|YP_070992.1| ribonuclease E [Yersinia pseudotuberculosis IP 32953]
>gi|51596814|ref|YP_071005.1| hypothetical protein YPTB2492 [Yersinia pseudotuberculosis IP 32953]
>gi|51596897|ref|YP_071088.1| NADH dehydrogenase I chain K [Yersinia pseudotuberculosis IP 32953]
>gi|51596933|ref|YP_071124.1| putative membrane protein [Yersinia pseudotuberculosis IP 32953]
>gi|51596949|ref|YP_071140.1| putative membrane protein [Yersinia pseudotuberculosis IP 32953]
>gi|51596965|ref|YP_071156.1| putative heme exporter protein D [Yersinia pseudotuberculosis IP 32953]
>gi|51596967|ref|YP_071158.1| ABC type heme exporter, permease subunit ccmB [Yersinia pseudotuberculosis IP 32953]
>gi|51596971|ref|YP_071162.1| putative membrane protein [Yersinia pseudotuberculosis IP 32953]
>gi|51596978|ref|YP_071169.1| hypothetical protein YPTB2659 [Yersinia pseudotuberculosis IP 32953]
>gi|51596996|ref|YP_071187.1| putative membrane protein [Yersinia pseudotuberculosis IP 32953]
>gi|51597005|ref|YP_071196.1| hypothetical protein YPTB2686 [Yersinia pseudotuberculosis IP 32953]
>gi|51597023|ref|YP_071214.1| hypothetical protein YPTB2704 [Yersinia pseudotuberculosis IP 32953]
>gi|51597054|ref|YP_071245.1| MFS family sialic acid transporter [Yersinia pseudotuberculosis IP 32953]
>gi|51597058|ref|YP_071249.1| putative antigenic leucine-rich repeat protein [Yersinia pseudotuberculosis IP 32953]
>gi|51597063|ref|YP_071254.1| hypothetical protein YPTB2745 [Yersinia pseudotuberculosis IP 32953]
>gi|51597083|ref|YP_071274.1| putative membrane protein [Yersinia pseudotuberculosis IP 32953]
>gi|51597095|ref|YP_071286.1| putative membrane protein [Yersinia pseudotuberculosis IP 32953]
>gi|51597143|ref|YP_071334.1| hypothetical protein YPTB2827 [Yersinia pseudotuberculosis IP 32953]
>gi|51597146|ref|YP_071337.1| hypothetical protein YPTB2830 [Yersinia pseudotuberculosis IP 32953]
>gi|51597150|ref|YP_071341.1| exodeoxyribonuclease VII large subunit [Yersinia pseudotuberculosis IP 32953]
>gi|51597158|ref|YP_071349.1| putative DNA-binding protein [Yersinia pseudotuberculosis IP 32953]
>gi|51597164|ref|YP_071355.1| putative pertactin family virulence factor/autotransporter [Yersinia pseudotuberculosis IP 32953]
>gi|51597191|ref|YP_071382.1| putative lipoprotein [Yersinia pseudotuberculosis IP 32953]
>gi|51597198|ref|YP_071389.1| putative membrane protein [Yersinia pseudotuberculosis IP 32953]
>gi|51597210|ref|YP_071401.1| sigma E factor regulatory protein [Yersinia pseudotuberculosis IP 32953]
>gi|51597224|ref|YP_071415.1| hypothetical protein YPTB2908 [Yersinia pseudotuberculosis IP 32953]
>gi|51597237|ref|YP_071428.1| putative BAT family (DMT superfamily) transporter [Yersinia pseudotuberculosis IP 32953]
>gi|51597269|ref|YP_071460.1| putative acid shock protein [Yersinia pseudotuberculosis IP 32953]
>gi|51597308|ref|YP_071499.1| UDP-3-o-[3-hydroxymyristoyl] glucosamine N-acyltransferase [Yersinia pseudotuberculosis IP 32953]
>gi|51597331|ref|YP_071522.1| hypothetical protein YPTB3016 [Yersinia pseudotuberculosis IP 32953]
>gi|51597343|ref|YP_071534.1| putative prepilin peptidase dependent protein [Yersinia pseudotuberculosis IP 32953]
>gi|51597404|ref|YP_071595.1| hypothetical protein YPTB3090 [Yersinia pseudotuberculosis IP 32953]
>gi|51597452|ref|YP_071643.1| hypothetical protein YPTB3141 [Yersinia pseudotuberculosis IP 32953]
>gi|51597460|ref|YP_071651.1| possible phage P2 holin-like protein [Yersinia pseudotuberculosis IP 32953]
>gi|51597470|ref|YP_071661.1| possible bacteriophage protein. [Yersinia pseudotuberculosis IP 32953]
>gi|51597480|ref|YP_071671.1| putative membrane protein [Yersinia pseudotuberculosis IP 32953]
>gi|51597525|ref|YP_071716.1| hypothetical protein YPTB3215 [Yersinia pseudotuberculosis IP 32953]
>gi|51597532|ref|YP_071723.1| hypothetical protein YPTB3222 [Yersinia pseudotuberculosis IP 32953]
>gi|51597564|ref|YP_071755.1| MFS family efflux protein. [Yersinia pseudotuberculosis IP 32953]
>gi|51597566|ref|YP_071757.1| putative siderophore biosynthesis protein IucB [Yersinia pseudotuberculosis IP 32953]

>gi|51597584|ref|YP_071775.1| hypothetical protein YPTB3284 [Yersinia pseudotuberculosis IP 32953]
>gi|51597585|ref|YP_071776.1| putative autotransporter secreted protein [Yersinia pseudotuberculosis IP 32953]
>gi|51597603|ref|YP_071794.1| putative pertactin family virulence factor/autotransporter [Yersinia pseudotuberculosis IP 32953]
>gi|51597607|ref|YP_071798.1| HlyD family drug efflux protein [Yersinia pseudotuberculosis IP 32953]
>gi|51597613|ref|YP_071804.1| putative outer membrane lipoprotein Pcp precursor [Yersinia pseudotuberculosis IP 32953]
>gi|51597622|ref|YP_071813.1| putative flagellar hook-length control protein [Yersinia pseudotuberculosis IP 32953]
>gi|51597640|ref|YP_071831.1| putative flagellar basal-body rod protein [Yersinia pseudotuberculosis IP 32953]
>gi|51597681|ref|YP_071872.1| possible MotA/TolQ/ExbB proton channel family protein [Yersinia pseudotuberculosis IP 32953]
>gi|51597683|ref|YP_071874.1| putative DedA-family membrane protein. [Yersinia pseudotuberculosis IP 32953]
>gi|51597760|ref|YP_071951.1| putative HdeD-like membrane protein. [Yersinia pseudotuberculosis IP 32953]
>gi|51597776|ref|YP_071967.1| hypothetical protein YPTB3484 [Yersinia pseudotuberculosis IP 32953]
>gi|51597778|ref|YP_071969.1| hypothetical protein YPTB3486 [Yersinia pseudotuberculosis IP 32953]
>gi|51597780|ref|YP_071971.1| putative membrane protein. [Yersinia pseudotuberculosis IP 32953]
>gi|51597847|ref|YP_072038.1| hypothetical protein YPTB3556 [Yersinia pseudotuberculosis IP 32953]
>gi|51597860|ref|YP_072051.1| putative membrane protein [Yersinia pseudotuberculosis IP 32953]
>gi|51597862|ref|YP_072053.1| biotin carboxyl carrier protein of acetyl-CoA carboxylase [Yersinia pseudotuberculosis IP 32953]
>gi|51597875|ref|YP_072066.1| outer membrane lipoprotein [Yersinia pseudotuberculosis IP 32953]
>gi|51597980|ref|YP_072171.1| 50S ribosomal protein L29 [Yersinia pseudotuberculosis IP 32953]
>gi|51597988|ref|YP_072179.1| 50S ribosomal protein L3 [Yersinia pseudotuberculosis IP 32953]
>gi|51597996|ref|YP_072187.1| hypothetical protein YPTB3706 [Yersinia pseudotuberculosis IP 32953]
>gi|51598002|ref|YP_072193.1| peptidyl-prolyl cis-trans isomerase [Yersinia pseudotuberculosis IP 32953]
>gi|51598044|ref|YP_072235.1| putative membrane protein [Yersinia pseudotuberculosis IP 32953]
>gi|51598079|ref|YP_072270.1| possible Bacterial Ig-like domain (group 1) [Yersinia pseudotuberculosis IP 32953]
>gi|51598081|ref|YP_072272.1| possible MarC family transporter [Yersinia pseudotuberculosis IP 32953]
>gi|51598110|ref|YP_072301.1| GntP family symporter [Yersinia pseudotuberculosis IP 32953]
>gi|51598130|ref|YP_072321.1| ABC dipeptide transporter, permease subunit [Yersinia pseudotuberculosis IP 32953]
>gi|51598136|ref|YP_072327.1| putative sensor protein uhpB [Yersinia pseudotuberculosis IP 32953]
>gi|51598143|ref|YP_072334.1| putative membrane protein [Yersinia pseudotuberculosis IP 32953]
>gi|51598197|ref|YP_072388.1| OmpA-OmpF family porin [Yersinia pseudotuberculosis IP 32953]
>gi|51598219|ref|YP_072410.1| putative membrane protein [Yersinia pseudotuberculosis IP 32953]
>gi|51598235|ref|YP_072426.1| hypothetical protein YPTB3951 [Yersinia pseudotuberculosis IP 32953]
>gi|51598255|ref|YP_072446.1| ATP synthase subunit B protein [Yersinia pseudotuberculosis IP 32953]
>gi|51598256|ref|YP_072447.1| ATP synthase subunit C protein [Yersinia pseudotuberculosis IP 32953]
>gi|15923074|ref|NP_370608.1| hypothetical protein SAV0084 [Staphylococcus aureus subsp. aureus Mu50]
>gi|15923101|ref|NP_370635.1| Immunoglobulin G binding protein A precursor [Staphylococcus aureus subsp. aureus Mu50]
>gi|15923104|ref|NP_370638.1| lipoprotein [Staphylococcus aureus subsp. aureus Mu50]
>gi|15923115|ref|NP_370649.1| hypothetical protein SAV0125 [Staphylococcus aureus subsp. aureus Mu50]
>gi|15923124|ref|NP_370658.1| hypothetical protein SAV0134 [Staphylococcus aureus subsp. aureus Mu50]
>gi|15923268|ref|NP_370802.1| hypothetical protein SAV0278 [Staphylococcus aureus subsp. aureus Mu50]
>gi|15923272|ref|NP_370806.1| hypothetical protein SAV0282 [Staphylococcus aureus subsp. aureus Mu50]
>gi|15923286|ref|NP_370820.1| hypothetical protein SAV0296 [Staphylococcus aureus subsp. aureus Mu50]
>gi|15923340|ref|NP_370874.1| hypothetical protein SAV0350 [Staphylococcus aureus subsp. aureus Mu50]
>gi|15923364|ref|NP_370898.1| hypothetical protein SAV0374 [Staphylococcus aureus subsp. aureus Mu50]
>gi|15923367|ref|NP_370901.1| hypothetical protein SAV0377 [Staphylococcus aureus subsp. aureus Mu50]
>gi|15923397|ref|NP_370931.1| hypothetical protein SAV0407 [Staphylococcus aureus subsp. aureus Mu50]
>gi|15923414|ref|NP_370948.1| exotoxin 8 [Staphylococcus aureus subsp. aureus Mu50]
>gi|15923519|ref|NP_371053.1| Serine acetyltransferase homolog [Staphylococcus aureus subsp. aureus Mu50]
>gi|15923530|ref|NP_371064.1| 50S ribosomal protein L7/L12 [Staphylococcus aureus subsp. aureus Mu50]
>gi|15923551|ref|NP_371085.1| Ser-Asp rich fibrinogen-binding, bone sialoprotein-binding protein [Staphylococcus aureus subsp. aureus Mu50]
>gi|15923552|ref|NP_371086.1| Ser-Asp rich fibrinogen-binding, bone sialoprotein-binding protein [Staphylococcus aureus subsp. aureus Mu50]
>gi|15923553|ref|NP_371087.1| Ser-Asp rich fibrinogen-binding, bone sialoprotein-binding protein [Staphylococcus aureus subsp. aureus Mu50]
>gi|15923583|ref|NP_371117.1| hypothetical protein SAV0593 [Staphylococcus aureus subsp. aureus Mu50]
>gi|15923600|ref|NP_371134.1| similar to iron(III) ABC transporter permease protein [Staphylococcus aureus subsp. aureus Mu50]
>gi|15923607|ref|NP_371141.1| hypothetical protein SAV0617 [Staphylococcus aureus subsp. aureus Mu50]
>gi|15923638|ref|NP_371172.1| ferrichrome transport permease [Staphylococcus aureus subsp. aureus Mu50]
>gi|15923667|ref|NP_371201.1| hypothetical protein SAV0677 [Staphylococcus aureus subsp. aureus Mu50]
>gi|15923672|ref|NP_371206.1| hypothetical protein SAV0682 [Staphylococcus aureus subsp. aureus Mu50]
>gi|15923699|ref|NP_371233.1| hypothetical protein SAV0709 [Staphylococcus aureus subsp. aureus Mu50]
>gi|15923706|ref|NP_371240.1| hypothetical protein SAV0716 [Staphylococcus aureus subsp. aureus Mu50]
>gi|15923724|ref|NP_371258.1| similar to ferrichrome ABC transporter permease [Staphylococcus aureus subsp. aureus Mu50]
>gi|15923737|ref|NP_371271.1| lipophilic protein affecting bacterial lysis rate and methicillin resistance level [Staphylococcus aureus subsp. aureus Mu50]
>gi|15923745|ref|NP_371279.1| hypothetical protein SAV0755 [Staphylococcus aureus subsp. aureus Mu50]
>gi|15923760|ref|NP_371294.1| hypothetical protein SAV0770 [Staphylococcus aureus subsp. aureus Mu50]
>gi|15923798|ref|NP_371332.1| hypothetical protein SAV0808 [Staphylococcus aureus subsp. aureus Mu50]
>gi|15923801|ref|NP_371335.1| fibrinogen-binding protein [Staphylococcus aureus subsp. aureus Mu50]
>gi|15923828|ref|NP_371362.1| similar to ABC transporter, permease protein homolog [Staphylococcus aureus subsp. aureus Mu50]
>gi|15923854|ref|NP_371388.1| single strand DNA binding protein [Staphylococcus aureus subsp. aureus Mu50]
>gi|15923858|ref|NP_371392.1| hypothetical protein SAV0868 [Staphylococcus aureus subsp. aureus Mu50]
>gi|15923863|ref|NP_371397.1| phi PVL ORF 50 [Staphylococcus aureus subsp. aureus Mu50]

>gi|15923880|ref|NP_371414.1| hypothetical protein SAV0890 [Staphylococcus aureus subsp. aureus Mu50]
>gi|15923891|ref|NP_371425.1| hypothetical protein SAV0901 [Staphylococcus aureus subsp. aureus Mu50]
>gi|15923937|ref|NP_371471.1| Na+/H+ antiporter subunit [Staphylococcus aureus subsp. aureus Mu50]
>gi|15924093|ref|NP_371627.1| hypothetical protein SAV1103 [Staphylococcus aureus subsp. aureus Mu50]
>gi|15924095|ref|NP_371629.1| Mn2+transport protein [Staphylococcus aureus subsp. aureus Mu50]
>gi|15924119|ref|NP_371653.1| iron-regulated cell wall-anchored protein SirH [Staphylococcus aureus subsp. aureus Mu50]
>gi|15924120|ref|NP_371654.1| cell surface protein [Staphylococcus aureus subsp. aureus Mu50]
>gi|15924147|ref|NP_371681.1| hypothetical protein SAV1157 [Staphylococcus aureus subsp. aureus Mu50]
>gi|15924313|ref|NP_371847.1| hypothetical protein SAV1323 [Staphylococcus aureus subsp. aureus Mu50]
>gi|15924333|ref|NP_371867.1| hypothetical protein SAV1343 [Staphylococcus aureus subsp. aureus Mu50]
>gi|15924366|ref|NP_371900.1| hypothetical protein SAV1376 [Staphylococcus aureus subsp. aureus Mu50]
>gi|15924387|ref|NP_371921.1| tetrahydrodipicolinate acetyltransferase [Staphylococcus aureus subsp. aureus Mu50]
>gi|15924471|ref|NP_372005.1| elastin binding protein [Staphylococcus aureus subsp. aureus Mu50]
>gi|15924518|ref|NP_372052.1| translation elongation factor EF-P [Staphylococcus aureus subsp. aureus Mu50]
>gi|15924522|ref|NP_372056.1| hypothetical protein SAV1532 [Staphylococcus aureus subsp. aureus Mu50]
>gi|15924559|ref|NP_372093.1| similar to diacylglycerol kinase [Staphylococcus aureus subsp. aureus Mu50]
>gi|15924565|ref|NP_372099.1| 30S ribosomal protein S21 [Staphylococcus aureus subsp. aureus Mu50]
>gi|15924576|ref|NP_372110.1| 30S ribosomal protein S20 [Staphylococcus aureus subsp. aureus Mu50]
>gi|15924594|ref|NP_372128.1| probable transmembrane transport protein [Staphylococcus aureus subsp. aureus Mu50]
>gi|15924605|ref|NP_372139.1| hypothetical protein SAV1615 [Staphylococcus aureus subsp. aureus Mu50]
>gi|15924614|ref|NP_372148.1| hypothetical protein SAV1624 [Staphylococcus aureus subsp. aureus Mu50]
>gi|15924615|ref|NP_372149.1| sigmaB-controlled gene product [Staphylococcus aureus subsp. aureus Mu50]
>gi|15924641|ref|NP_372175.1| hypothetical protein SAV1651 [Staphylococcus aureus subsp. aureus Mu50]
>gi|15924721|ref|NP_372255.1| hypothetical protein SAV1731 [Staphylococcus aureus subsp. aureus Mu50]
>gi|15924728|ref|NP_372262.1| hypothetical protein SAV1738 [Staphylococcus aureus subsp. aureus Mu50]
>gi|15924772|ref|NP_372306.1| hypothetical protein SAV1782 [Staphylococcus aureus subsp. aureus Mu50]
>gi|15924789|ref|NP_372323.1| hypothetical protein SAV1799 [Staphylococcus aureus subsp. aureus Mu50]
>gi|15924790|ref|NP_372324.1| hypothetical protein SAV1800 [Staphylococcus aureus subsp. aureus Mu50]
>gi|15924812|ref|NP_372346.1| hypothetical protein SAV1822 [Staphylococcus aureus subsp. aureus Mu50]
>gi|15924831|ref|NP_372365.1| peptidyl-prolyl cis/trans isomerase [Staphylococcus aureus subsp. aureus Mu50]
>gi|15924835|ref|NP_372369.1| putative transcriptional regulator [Staphylococcus aureus subsp. aureus Mu50]
>gi|15924917|ref|NP_372451.1| hypothetical protein SAV1927 [Staphylococcus aureus subsp. aureus Mu50]
>gi|15924920|ref|NP_372454.1| hypothetical protein SAV1930 [Staphylococcus aureus subsp. aureus Mu50]
>gi|15924932|ref|NP_372466.1| hypothetical protein SAV1942 [Staphylococcus aureus subsp. aureus Mu50]
>gi|15924969|ref|NP_372503.1| phi PVL ORF 50 homolog [Staphylococcus aureus subsp. aureus Mu50]
>gi|15924991|ref|NP_372525.1| hypothetical protein SAV2001 [Staphylococcus aureus subsp. aureus Mu50]
>gi|15925000|ref|NP_372534.1| hypothetical protein SAV2010 [Staphylococcus aureus subsp. aureus Mu50]
>gi|15925014|ref|NP_372548.1| hypothetical protein SAV2024 [Staphylococcus aureus subsp. aureus Mu50]
>gi|15925022|ref|NP_372556.1| hypothetical protein SAV2032 [Staphylococcus aureus subsp. aureus Mu50]
>gi|15925026|ref|NP_372560.1| accessory gene regulator B [Staphylococcus aureus subsp. aureus Mu50]
>gi|15925118|ref|NP_372652.1| probable DNA-directed RNA polymerase delta subunit [Staphylococcus aureus subsp. aureus Mu50]
>gi|15925150|ref|NP_372684.1| FmtB protein [Staphylococcus aureus subsp. aureus Mu50]
>gi|15925165|ref|NP_372699.1| heme transport system permease [Staphylococcus aureus subsp. aureus Mu50]
>gi|15925253|ref|NP_372787.1| hypothetical protein SAV2263 [Staphylococcus aureus subsp. aureus Mu50]
>gi|15925272|ref|NP_372806.1| similar to biotin biosynthesis protein [Staphylococcus aureus subsp. aureus Mu50]
>gi|15925289|ref|NP_372823.1| secretory antigen precursor SsaA homolog [Staphylococcus aureus subsp. aureus Mu50]
>gi|15925293|ref|NP_372827.1| hypothetical protein SAV2303 [Staphylococcus aureus subsp. aureus Mu50]
>gi|15925294|ref|NP_372828.1| hypothetical protein SAV2304 [Staphylococcus aureus subsp. aureus Mu50]
>gi|15925307|ref|NP_372841.1| hypothetical protein SAV2317 [Staphylococcus aureus subsp. aureus Mu50]
>gi|15925317|ref|NP_372851.1| hypothetical protein SAV2327 [Staphylococcus aureus subsp. aureus Mu50]
>gi|15925343|ref|NP_372877.1| similar to multidrug resistance protein A [Staphylococcus aureus subsp. aureus Mu50]
>gi|15925358|ref|NP_372892.1| hypothetical protein SAV2368 [Staphylococcus aureus subsp. aureus Mu50]
>gi|15925373|ref|NP_372907.1| hypothetical protein SAV2383 [Staphylococcus aureus subsp. aureus Mu50]
>gi|15925412|ref|NP_372946.1| hypothetical protein SAV2422 [Staphylococcus aureus subsp. aureus Mu50]
>gi|15925423|ref|NP_372957.1| hypothetical protein SAV2433 [Staphylococcus aureus subsp. aureus Mu50]
>gi|15925484|ref|NP_373018.1| hypothetical protein SAV2494 [Staphylococcus aureus subsp. aureus Mu50]
>gi|57634654|ref|NP_373020.2| hypothetical protein SAV2496 [Staphylococcus aureus subsp. aureus Mu50]
>gi|15925492|ref|NP_373026.1| fibronectin-binding protein homolog [Staphylococcus aureus subsp. aureus Mu50]
>gi|15925493|ref|NP_373027.1| fibronectin-binding protein homolog [Staphylococcus aureus subsp. aureus Mu50]
>gi|15925505|ref|NP_373039.1| probable transmembrane protein smpB [Staphylococcus aureus subsp. aureus Mu50]
>gi|15925517|ref|NP_373051.1| hypothetical protein SAV2527 [Staphylococcus aureus subsp. aureus Mu50]
>gi|15925518|ref|NP_373052.1| sortase [Staphylococcus aureus subsp. aureus Mu50]
>gi|15925520|ref|NP_373054.1| putative L-serine dehydratase [Staphylococcus aureus subsp. aureus Mu50]
>gi|15925556|ref|NP_373090.1| similar to secretory antigen precursor SsaA [Staphylococcus aureus subsp. aureus Mu50]
>gi|15925559|ref|NP_373093.1| immunodominant antigen A [Staphylococcus aureus subsp. aureus Mu50]
>gi|15925620|ref|NP_373154.1| Clumping factor B [Staphylococcus aureus subsp. aureus Mu50]
>gi|15925629|ref|NP_373163.1| hypothetical protein SAV2639 [Staphylococcus aureus subsp. aureus Mu50]
>gi|15925634|ref|NP_373168.1| similar to autolysin precursor [Staphylococcus aureus subsp. aureus Mu50]
>gi|15925644|ref|NP_373178.1| serine-threoinine rich antigen [Staphylococcus aureus subsp. aureus Mu50]
>gi|15925681|ref|NP_373215.1| hypothetical protein SAV2691 [Staphylococcus aureus subsp. aureus Mu50]

>gi|15925685|ref|NP_373219.1| 2-oxoglutarate/malate translocator [Staphylococcus aureus subsp. aureus Mu50]
>gi|15925690|ref|NP_373224.1| hypothetical protein SAV2700 [Staphylococcus aureus subsp. aureus Mu50]
>gi|15925697|ref|NP_373231.1| hypothetical protein SAV2707 [Staphylococcus aureus subsp. aureus Mu50]
>gi|15925704|ref|NP_373238.1| 50S ribosomal protein L34 [Staphylococcus aureus subsp. aureus Mu50]
>gi|14141829|ref|NP_115295.1| hypothetical protein SAVP006 [Staphylococcus aureus subsp. aureus Mu50]
>gi|14141842|ref|NP_115308.1| truncated hypothetical protein [Staphylococcus aureus subsp. aureus Mu50]
>gi|14141843|ref|NP_115309.1| truncated hypothetical protein [Staphylococcus aureus subsp. aureus Mu50]
>gi|14141857|ref|NP_115323.1| replication-associated protein [Staphylococcus aureus subsp. aureus Mu50]
>gi|15605618|ref|NP_212991.1| ribosomal protein L23 [Aquifex aeolicus VF5]
>gi|15605634|ref|NP_213009.1| transporter (Pho87 family) [Aquifex aeolicus VF5]
>gi|15605660|ref|NP_213035.1| single stranded DNA-binding protein [Aquifex aeolicus VF5]
>gi|15605662|ref|NP_213037.1| hypothetical protein aq_064b [Aquifex aeolicus VF5]
>gi|15605663|ref|NP_213038.1| hypothetical protein aq_064c [Aquifex aeolicus VF5]
>gi|15605682|ref|NP_213057.1| Molybdenum transport system permease [Aquifex aeolicus VF5]
>gi|15605693|ref|NP_213069.1| hypothetical protein aq_106 [Aquifex aeolicus VF5]
>gi|15605708|ref|NP_213085.1| hypothetical protein aq_125 [Aquifex aeolicus VF5]
>gi|15605730|ref|NP_213107.1| hypothetical protein aq_157 [Aquifex aeolicus VF5]
>gi|15605746|ref|NP_213123.1| ribosomal protein L34 [Aquifex aeolicus VF5]
>gi|15605747|ref|NP_213124.1| ATP synthase F0 subunit c [Aquifex aeolicus VF5]
>gi|15605760|ref|NP_213137.1| hypothetical protein aq_197 [Aquifex aeolicus VF5]
>gi|15605775|ref|NP_213152.1| deoxyuridine 5'triphosphate nucleotidohydrolase [Aquifex aeolicus VF5]
>gi|15605807|ref|NP_213184.1| hypothetical protein aq_267 [Aquifex aeolicus VF5]
>gi|15605826|ref|NP_213203.1| hypothetical protein aq_293 [Aquifex aeolicus VF5]
>gi|15605827|ref|NP_213204.1| hypothetical protein aq_294 [Aquifex aeolicus VF5]
>gi|15605866|ref|NP_213243.1| anti sigma factor FlgM [Aquifex aeolicus VF5]
>gi|15605906|ref|NP_213283.1| hypothetical protein aq_406 [Aquifex aeolicus VF5]
>gi|15605922|ref|NP_213299.1| hypothetical protein aq_423 [Aquifex aeolicus VF5]
>gi|15605979|ref|NP_213356.1| hypothetical protein aq_507 [Aquifex aeolicus VF5]
>gi|15605996|ref|NP_213373.1| hypothetical protein aq_531 [Aquifex aeolicus VF5]
>gi|15606000|ref|NP_213377.1| UDP-3-O-[3-hydroxymyristoyl] glucosamine N acyltransferase [Aquifex aeolicus VF5]
>gi|15606017|ref|NP_213394.1| recombination protein RecN [Aquifex aeolicus VF5]
>gi|15606028|ref|NP_213405.1| hypothetical protein aq_577 [Aquifex aeolicus VF5]
>gi|15606037|ref|NP_213414.1| hypothetical protein aq_592 [Aquifex aeolicus VF5]
>gi|15606041|ref|NP_213418.1| RNA polymerase sigma factor RpoN [Aquifex aeolicus VF5]
>gi|15606043|ref|NP_213420.1| hypothetical protein aq_601 [Aquifex aeolicus VF5]
>gi|15606045|ref|NP_213422.1| acyl-[acyl-carrier-protein]-UDP-N-acetylglucosamine acyltransferase [Aquifex aeolicus VF5]
>gi|15606050|ref|NP_213427.1| hypothetical protein aq_615 [Aquifex aeolicus VF5]
>gi|15606061|ref|NP_213438.1| chromosome assembly protein homolog [Aquifex aeolicus VF5]
>gi|15606073|ref|NP_213450.1| lipoprotein [Aquifex aeolicus VF5]
>gi|15606074|ref|NP_213451.1| flagellar switch protein FliG [Aquifex aeolicus VF5]
>gi|15606081|ref|NP_213458.1| HupD hydrogenase related function [Aquifex aeolicus VF5]
>gi|15606086|ref|NP_213463.1| ATP synthase F1 epsilon subunit [Aquifex aeolicus VF5]
>gi|15606111|ref|NP_213488.1| ribosome recycling factor [Aquifex aeolicus VF5]
>gi|15606125|ref|NP_213502.1| cytochrome c-type biogenesis protein [Aquifex aeolicus VF5]
>gi|15606140|ref|NP_213517.1| hypothetical protein aq_754 [Aquifex aeolicus VF5]
>gi|15606143|ref|NP_213520.1| hypothetical protein aq_757 [Aquifex aeolicus VF5]
>gi|15606154|ref|NP_213531.1| hypothetical protein aq_771 [Aquifex aeolicus VF5]
>gi|15606165|ref|NP_213542.1| cytochrome c552 [Aquifex aeolicus VF5]
>gi|15606177|ref|NP_213554.1| hypothetical protein aq_811 [Aquifex aeolicus VF5]
>gi|15606183|ref|NP_213560.1| hypothetical protein aq_818 [Aquifex aeolicus VF5]
>gi|15606201|ref|NP_213578.1| hypothetical protein aq_850 [Aquifex aeolicus VF5]
>gi|15606212|ref|NP_213589.1| ribosomal protein S21 [Aquifex aeolicus VF5]
>gi|15606216|ref|NP_213594.1| peptide chain release factor RF-1 [Aquifex aeolicus VF5]
>gi|15606218|ref|NP_213596.1| septum site-determining protein MinC [Aquifex aeolicus VF5]
>gi|15606243|ref|NP_213621.1| hypothetical protein aq_914 [Aquifex aeolicus VF5]
>gi|15606246|ref|NP_213624.1| hypothetical protein aq_917 [Aquifex aeolicus VF5]
>gi|15606250|ref|NP_213628.1| cell division protein FtsY [Aquifex aeolicus VF5]
>gi|15606290|ref|NP_213669.1| hypothetical protein aq_978 [Aquifex aeolicus VF5]
>gi|15606295|ref|NP_213674.1| hypothetical protein aq_986 [Aquifex aeolicus VF5]
>gi|15606308|ref|NP_213687.1| hypothetical protein aq_1006 [Aquifex aeolicus VF5]
>gi|15606341|ref|NP_213720.1| transcriptional regulator (TetR/AcrR family) [Aquifex aeolicus VF5]
>gi|15606343|ref|NP_213722.1| hypothetical protein aq_1060 [Aquifex aeolicus VF5]
>gi|15606363|ref|NP_213742.1| hemolysin [Aquifex aeolicus VF5]
>gi|15606376|ref|NP_213756.1| glycine cleavage system protein H [Aquifex aeolicus VF5]
>gi|15606388|ref|NP_213768.1| hypothetical protein aq_1127 [Aquifex aeolicus VF5]
>gi|15606432|ref|NP_213812.1| hypothetical protein aq_1188 [Aquifex aeolicus VF5]
>gi|15606441|ref|NP_213821.1| hypothetical protein aq_1201 [Aquifex aeolicus VF5]
>gi|15606457|ref|NP_213837.1| hypothetical protein aq_1223 [Aquifex aeolicus VF5]
>gi|15606468|ref|NP_213848.1| hypothetical protein aq_1239 [Aquifex aeolicus VF5]
>gi|15606487|ref|NP_213867.1| hypothetical protein aq_1264 [Aquifex aeolicus VF5]

>gi|15606500|ref|NP_213880.1| small heat shock protein (class I) [Aquifex aeolicus VF5]
>gi|15606522|ref|NP_213902.1| NADH dehydrogenase I chain J [Aquifex aeolicus VF5]
>gi|15606534|ref|NP_213914.1| cation efflux system (czcB-like) [Aquifex aeolicus VF5]
>gi|15606542|ref|NP_213922.1| trigger factor [Aquifex aeolicus VF5]
>gi|15606545|ref|NP_213925.1| hypothetical protein aq_1345 [Aquifex aeolicus VF5]
>gi|15606547|ref|NP_213927.1| hypothetical protein aq_1348 [Aquifex aeolicus VF5]
>gi|15606548|ref|NP_213928.1| hypothetical protein aq_1349 [Aquifex aeolicus VF5]
>gi|15606569|ref|NP_213949.1| NADH dehydrogenase I chain H [Aquifex aeolicus VF5]
>gi|15606572|ref|NP_213952.1| NADH dehydrogenase I chain K [Aquifex aeolicus VF5]
>gi|15606588|ref|NP_213968.1| hypothetical protein aq_1403 [Aquifex aeolicus VF5]
>gi|15606608|ref|NP_213988.1| pilin [Aquifex aeolicus VF5]
>gi|15606618|ref|NP_213998.1| hypothetical protein aq_1446 [Aquifex aeolicus VF5]
>gi|15606628|ref|NP_214008.1| hypothetical protein aq_1462 [Aquifex aeolicus VF5]
>gi|15606632|ref|NP_214012.1| hypothetical protein aq_1467 [Aquifex aeolicus VF5]
>gi|15606638|ref|NP_214018.1| hypothetical protein aq_1476 [Aquifex aeolicus VF5]
>gi|15606641|ref|NP_214021.1| hypothetical protein aq_1480 [Aquifex aeolicus VF5]
>gi|15606657|ref|NP_214037.1| hypothetical protein aq_1502 [Aquifex aeolicus VF5]
>gi|15606667|ref|NP_214047.1| hypothetical protein aq_1516 [Aquifex aeolicus VF5]
>gi|15606680|ref|NP_214060.1| flagellar switch protein FliN [Aquifex aeolicus VF5]
>gi|15606693|ref|NP_214073.1| hypothetical protein aq_1558 [Aquifex aeolicus VF5]
>gi|15606699|ref|NP_214079.1| hypothetical protein aq_1570 [Aquifex aeolicus VF5]
>gi|15606710|ref|NP_214090.1| ATP synthase F0 subunit b [Aquifex aeolicus VF5]
>gi|15606711|ref|NP_214091.1| ATP synthase F0 subunit b [Aquifex aeolicus VF5]
>gi|15606712|ref|NP_214092.1| ATP synthase F1 delta chain [Aquifex aeolicus VF5]
>gi|15606717|ref|NP_214097.1| hypothetical protein aq_1596 [Aquifex aeolicus VF5]
>gi|15606722|ref|NP_214102.1| hypothetical protein aq_1603 [Aquifex aeolicus VF5]
>gi|15606730|ref|NP_214110.1| hypothetical protein aq_1618 [Aquifex aeolicus VF5]
>gi|15606738|ref|NP_214118.1| hypothetical protein aq_1631 [Aquifex aeolicus VF5]
>gi|15606777|ref|NP_214157.1| hypothetical protein aq_1680 [Aquifex aeolicus VF5]
>gi|15606785|ref|NP_214165.1| hypothetical protein aq_1694 [Aquifex aeolicus VF5]
>gi|15606808|ref|NP_214188.1| hypothetical protein aq_1732 [Aquifex aeolicus VF5]
>gi|15606819|ref|NP_214199.1| hypothetical protein aq_1752 [Aquifex aeolicus VF5]
>gi|15606820|ref|NP_214200.1| lipoprotein NlpD fragment [Aquifex aeolicus VF5]
>gi|15606846|ref|NP_214226.1| hypothetical protein aq_1791 [Aquifex aeolicus VF5]
>gi|15606848|ref|NP_214228.1| hypothetical protein aq_1793 [Aquifex aeolicus VF5]
>gi|15606850|ref|NP_214230.1| hypothetical protein aq_1795 [Aquifex aeolicus VF5]
>gi|15606868|ref|NP_214248.1| nodulation competitiveness protein NfeD [Aquifex aeolicus VF5]
>gi|15606883|ref|NP_214264.1| hypothetical protein aq_1842 [Aquifex aeolicus VF5]
>gi|15606899|ref|NP_214280.1| flagellar biosynthesis FliL [Aquifex aeolicus VF5]
>gi|15606907|ref|NP_214288.1| hypothetical protein aq_1873 [Aquifex aeolicus VF5]
>gi|15606921|ref|NP_214302.1| hypothetical protein aq_1894 [Aquifex aeolicus VF5]
>gi|15606925|ref|NP_214306.1| hypothetical protein aq_1899a [Aquifex aeolicus VF5]
>gi|15606948|ref|NP_214330.1| ribosomal protein L7/L12 [Aquifex aeolicus VF5]
>gi|15606954|ref|NP_214336.1| ribosomal protein L19 [Aquifex aeolicus VF5]
>gi|15606960|ref|NP_214342.1| flagellar biosynthetic protein FliR [Aquifex aeolicus VF5]
>gi|15606971|ref|NP_214353.1| hypothetical protein aq_1974 [Aquifex aeolicus VF5]
>gi|15606977|ref|NP_214359.1| hypothetical protein aq_1982 [Aquifex aeolicus VF5]
>gi|15606991|ref|NP_214373.1| hypothetical protein aq_2000 [Aquifex aeolicus VF5]
>gi|15607005|ref|NP_214387.1| hypothetical protein aq_2020 [Aquifex aeolicus VF5]
>gi|15607009|ref|NP_214391.1| hypothetical protein aq_2028 [Aquifex aeolicus VF5]
>gi|15607012|ref|NP_214394.1| initiation factor IF-2 [Aquifex aeolicus VF5]
>gi|15607071|ref|NP_214453.1| hemolysin homolog protein [Aquifex aeolicus VF5]
>gi|15607088|ref|NP_214470.1| hypothetical protein aq_2145 [Aquifex aeolicus VF5]
>gi|15607095|ref|NP_214477.1| phosphotidylglycerophosphate synthase [Aquifex aeolicus VF5]
>gi|15607133|ref|NP_212997.1| 50S Ribosomal protein L29 [Aquifex aeolicus VF5]
>gi|15607140|ref|NP_214262.1| Copper chaperone [Aquifex aeolicus VF5]
>gi|10957046|ref|NP_046402.1| hypothetical protein aq_aa07 [Aquifex aeolicus VF5]
>gi|10957049|ref|NP_046405.1| hypothetical protein aq_aa11 [Aquifex aeolicus VF5]
>gi|10957050|ref|NP_046406.1| hypothetical protein aq_aa13 [Aquifex aeolicus VF5]
>gi|10957056|ref|NP_046412.1| hypothetical protein aq_aa21 [Aquifex aeolicus VF5]
>gi|10957067|ref|NP_046423.1| hypothetical protein aq_aa34 [Aquifex aeolicus VF5]
>gi|53723371|ref|YP_102846.1| nitrite/sulfite reductase domain protein [Burkholderia mallei ATCC 23344]
>gi|53723374|ref|YP_102849.1| cbiG protein/precorrin-3B C17-methyltransferase [Burkholderia mallei ATCC 23344]
>gi|53723376|ref|YP_102851.1| hypothetical protein BMA1166 [Burkholderia mallei ATCC 23344]
>gi|53723379|ref|YP_102808.1| hypothetical protein BMA1122 [Burkholderia mallei ATCC 23344]
>gi|53723383|ref|YP_102812.1| transcriptional regulator, AraC family [Burkholderia mallei ATCC 23344]
>gi|53723385|ref|YP_102814.1| membrane protein, putative [Burkholderia mallei ATCC 23344]
>gi|53723390|ref|YP_102856.1| cbiG protein [Burkholderia mallei ATCC 23344]
>gi|53723392|ref|YP_102858.1| cobyrinic acid a,c-diamide synthase [Burkholderia mallei ATCC 23344]
>gi|53723398|ref|YP_102820.1| drug resistance transporter, EmrB/QacA family [Burkholderia mallei ATCC 23344]

>gi|53723403|ref|YP_102862.1| non-ribosomal peptide synthetase, putative [Burkholderia mallei ATCC 23344]
>gi|53723404|ref|YP_102863.1| hypothetical protein BMA1182 [Burkholderia mallei ATCC 23344]
>gi|53723406|ref|YP_102865.1| hypothetical protein BMA1184 [Burkholderia mallei ATCC 23344]
>gi|53723408|ref|YP_102824.1| ABC transporter, permease protein [Burkholderia mallei ATCC 23344]
>gi|53723410|ref|YP_102826.1| ABC transporter, permease protein [Burkholderia mallei ATCC 23344]
>gi|53723415|ref|YP_102867.1| ferric iron reductase protein FhuF [Burkholderia mallei ATCC 23344]
>gi|53723416|ref|YP_102868.1| iron compound ABC transporter, permease protein [Burkholderia mallei ATCC 23344]
>gi|53723423|ref|YP_102834.1| hypothetical protein BMA1149 [Burkholderia mallei ATCC 23344]
>gi|53723426|ref|YP_102837.1| transcriptional regulator, MarR family [Burkholderia mallei ATCC 23344]
>gi|53723428|ref|YP_102873.1| hypothetical protein BMA1192 [Burkholderia mallei ATCC 23344]
>gi|53723432|ref|YP_102877.1| ribose ABC transporter, permease protein [Burkholderia mallei ATCC 23344]
>gi|53723433|ref|YP_102879.1| ribose ABC transporter, periplasmic ribose-binding protein [Burkholderia mallei ATCC 23344]
>gi|53723436|ref|YP_102840.1| major facilitator family transporter [Burkholderia mallei ATCC 23344]
>gi|53723439|ref|YP_102843.1| precorrin-6x reductase [Burkholderia mallei ATCC 23344]
>gi|53723440|ref|YP_102845.1| precorrin-6Y C5,15-methyltransferase (decarboxylating) [Burkholderia mallei ATCC 23344]
>gi|53723443|ref|YP_102881.1| hypothetical protein BMA1200 [Burkholderia mallei ATCC 23344]
>gi|53723448|ref|YP_102887.1| sulfate ABC transporter, ATP-binding protein [Burkholderia mallei ATCC 23344]
>gi|53723449|ref|YP_102916.1| hypothetical protein BMA1240 [Burkholderia mallei ATCC 23344]
>gi|53723450|ref|YP_102917.1| enoyl-CoA hydratase/isomerase family protein [Burkholderia mallei ATCC 23344]
>gi|53723459|ref|YP_102893.1| hypothetical protein BMA1214 [Burkholderia mallei ATCC 23344]
>gi|53723484|ref|YP_102904.1| hypothetical protein BMA1225 [Burkholderia mallei ATCC 23344]
>gi|53723491|ref|YP_102939.1| sulfate permease family protein [Burkholderia mallei ATCC 23344]
>gi|53723495|ref|YP_102943.1| transcriptional regulator, TetR family [Burkholderia mallei ATCC 23344]
>gi|53723499|ref|YP_102913.1| aliphatic sulfonate ABC transporter, ATP-binding protein [Burkholderia mallei ATCC 23344]
>gi|53723500|ref|YP_102914.1| aliphatic sulfonate ABC transporter, permease protein [Burkholderia mallei ATCC 23344]
>gi|53723504|ref|YP_102946.1| hypothetical protein BMA1278 [Burkholderia mallei ATCC 23344]
>gi|53723505|ref|YP_102947.1| hfq protein [Burkholderia mallei ATCC 23344]
>gi|53723506|ref|YP_102948.1| hypothetical protein BMA1281 [Burkholderia mallei ATCC 23344]
>gi|53723508|ref|YP_102950.1| hypothetical protein BMA1283 [Burkholderia mallei ATCC 23344]
>gi|53723509|ref|YP_102951.1| TPR domain protein [Burkholderia mallei ATCC 23344]
>gi|53723510|ref|YP_102983.1| conserved hypothetical protein TIGR00726 [Burkholderia mallei ATCC 23344]
>gi|53723515|ref|YP_102988.1| lipoprotein, putative [Burkholderia mallei ATCC 23344]
>gi|53723518|ref|YP_102952.1| membrane protein, putative [Burkholderia mallei ATCC 23344]
>gi|53723521|ref|YP_102955.1| hypothetical protein BMA1291 [Burkholderia mallei ATCC 23344]
>gi|53723529|ref|YP_102997.1| ftsH protease activity modulator HflC [Burkholderia mallei ATCC 23344]
>gi|53723530|ref|YP_102998.1| ftsH protease activity modulator HflK [Burkholderia mallei ATCC 23344]
>gi|53723532|ref|YP_102959.1| hypothetical protein BMA1297 [Burkholderia mallei ATCC 23344]
>gi|53723540|ref|YP_103003.1| hypothetical protein BMA1343 [Burkholderia mallei ATCC 23344]
>gi|53723543|ref|YP_103006.1| hypothetical protein BMA1346 [Burkholderia mallei ATCC 23344]
>gi|53723550|ref|YP_102969.1| AsmA family protein [Burkholderia mallei ATCC 23344]
>gi|53723551|ref|YP_102970.1| ABC transporter, ATP-binding protein [Burkholderia mallei ATCC 23344]
>gi|53723559|ref|YP_103015.1| lipoprotein NlpD, putative [Burkholderia mallei ATCC 23344]
>gi|53723560|ref|YP_102971.1| membrane protein, putative [Burkholderia mallei ATCC 23344]
>gi|53723562|ref|YP_102973.1| phosphoserine phosphatase [Burkholderia mallei ATCC 23344]
>gi|53723566|ref|YP_102977.1| carbohydrate kinase, PfkB family [Burkholderia mallei ATCC 23344]
>gi|53723568|ref|YP_103016.1| protein-L-isoaspartate O-methyltransferase [Burkholderia mallei ATCC 23344]
>gi|53723573|ref|YP_103021.1| DNA polymerase III, subunit gamma, putative [Burkholderia mallei ATCC 23344]
>gi|53723582|ref|YP_103061.1| hypothetical protein BMA1405 [Burkholderia mallei ATCC 23344]
>gi|53723583|ref|YP_103062.1| RNA polymerase sigma-70 factor, ECF subfamily [Burkholderia mallei ATCC 23344]
>gi|53723591|ref|YP_103031.1| hypothetical protein BMA1372 [Burkholderia mallei ATCC 23344]
>gi|53723593|ref|YP_103064.1| cytochrome c oxidase family protein [Burkholderia mallei ATCC 23344]
>gi|53723595|ref|YP_103067.1| hypothetical protein BMA1412 [Burkholderia mallei ATCC 23344]
>gi|53723596|ref|YP_103068.1| asparaginase family protein [Burkholderia mallei ATCC 23344]
>gi|53723602|ref|YP_103034.1| hypothetical protein BMA1375 [Burkholderia mallei ATCC 23344]
>gi|53723607|ref|YP_103073.1| hypothetical protein BMA1418 [Burkholderia mallei ATCC 23344]
>gi|53723613|ref|YP_103079.1| DNA polymerase III, delta prime subunit [Burkholderia mallei ATCC 23344]
>gi|53723621|ref|YP_103082.1| hypothetical protein BMA1427 [Burkholderia mallei ATCC 23344]
>gi|53723622|ref|YP_103083.1| acetyltransferase, GNAT family [Burkholderia mallei ATCC 23344]
>gi|53723631|ref|YP_103052.1| PhoH family protein [Burkholderia mallei ATCC 23344]
>gi|53723632|ref|YP_103053.1| lipoprotein, NLP/P60 family [Burkholderia mallei ATCC 23344]
>gi|53723634|ref|YP_103087.1| hypothetical protein BMA1434 [Burkholderia mallei ATCC 23344]
>gi|53723650|ref|YP_103095.1| BolA-like protein [Burkholderia mallei ATCC 23344]
>gi|53723652|ref|YP_103097.1| acetyltransferase, GNAT family [Burkholderia mallei ATCC 23344]
>gi|53723655|ref|YP_103100.1| carbohydrate kinase, putative [Burkholderia mallei ATCC 23344]
>gi|53723659|ref|YP_103104.1| hypothetical protein BMA1452 [Burkholderia mallei ATCC 23344]
>gi|53723670|ref|YP_103115.1| glycerate kinase 1 [Burkholderia mallei ATCC 23344]
>gi|53723672|ref|YP_103117.1| 2-dehydropantoate 2-reductase [Burkholderia mallei ATCC 23344]
>gi|53723675|ref|YP_103120.1| membrane protein, putative [Burkholderia mallei ATCC 23344]
>gi|53723690|ref|YP_103142.1| PspA/IM30 family protein [Burkholderia mallei ATCC 23344]
>gi|53723691|ref|YP_103143.1| hypothetical protein BMA1499 [Burkholderia mallei ATCC 23344]

>gi|53723709|ref|YP_103164.1| hypothetical protein BMA1523 [Burkholderia mallei ATCC 23344]
>gi|53723714|ref|YP_103169.1| membrane protein, putative [Burkholderia mallei ATCC 23344]
>gi|53723717|ref|YP_103173.1| SPFH domain/band 7 family protein [Burkholderia mallei ATCC 23344]
>gi|53723718|ref|YP_103174.1| hypothetical protein BMA1534 [Burkholderia mallei ATCC 23344]
>gi|53723720|ref|YP_103176.1| hypothetical protein BMA1536 [Burkholderia mallei ATCC 23344]
>gi|53723729|ref|YP_103185.1| UDP-3-O-3-hydroxymyristoyl glucosamine N-acyltransferase [Burkholderia mallei ATCC 23344]
>gi|53723730|ref|YP_103186.1| outer membrane protein, OmpH/HlpA family [Burkholderia mallei ATCC 23344]
>gi|53723734|ref|YP_103190.1| phosphatidate cytidylyltransferase [Burkholderia mallei ATCC 23344]
>gi|53723745|ref|YP_103201.1| hypothetical protein BMA1561 [Burkholderia mallei ATCC 23344]
>gi|53723746|ref|YP_103202.1| hypothetical protein BMA1562 [Burkholderia mallei ATCC 23344]
>gi|53723747|ref|YP_103203.1| chromosome segregation protein SMC [Burkholderia mallei ATCC 23344]
>gi|53723749|ref|YP_103274.1| hypothetical protein BMA1645 [Burkholderia mallei ATCC 23344]
>gi|53723752|ref|YP_103277.1| hypothetical protein BMA1650 [Burkholderia mallei ATCC 23344]
>gi|53723754|ref|YP_103279.1| cell cycle protein mesJ [Burkholderia mallei ATCC 23344]
>gi|53723758|ref|YP_103283.1| TPR domain protein [Burkholderia mallei ATCC 23344]
>gi|53723763|ref|YP_103288.1| serine O-acetyltransferase [Burkholderia mallei ATCC 23344]
>gi|53723770|ref|YP_103295.1| lipoprotein, putative [Burkholderia mallei ATCC 23344]
>gi|53723780|ref|YP_103305.1| hypothetical protein BMA1685 [Burkholderia mallei ATCC 23344]
>gi|53723781|ref|YP_103306.1| bacterial transferase hexapeptide repeat protein [Burkholderia mallei ATCC 23344]
>gi|53723788|ref|YP_103313.1| DNA internalization-related competence protein ComEC/Rec2 [Burkholderia mallei ATCC 23344]
>gi|53723792|ref|YP_103317.1| hypothetical protein BMA1697 [Burkholderia mallei ATCC 23344]
>gi|53723796|ref|YP_103321.1| hypothetical protein BMA1701 [Burkholderia mallei ATCC 23344]
>gi|53723805|ref|YP_103330.1| low molecular weight protein-tyrosine-phosphatase [Burkholderia mallei ATCC 23344]
>gi|53723806|ref|YP_103331.1| hypothetical protein BMA1711 [Burkholderia mallei ATCC 23344]
>gi|53723810|ref|YP_103336.1| phasin family protein [Burkholderia mallei ATCC 23344]
>gi|53723811|ref|YP_103337.1| D-alanyl-D-alanine endopeptidase, putative [Burkholderia mallei ATCC 23344]
>gi|53723813|ref|YP_103340.1| pyruvate dehydrogenase, E2 component, dihydrolipoamide acetyltransferase [Burkholderia mallei ATCC 23344]
>gi|53723825|ref|YP_104137.1| thiol:disulfide interchange protein DsbD, putative [Burkholderia mallei ATCC 23344]
>gi|53723863|ref|YP_104175.1| ribosomal protein L7/L12 [Burkholderia mallei ATCC 23344]
>gi|53723873|ref|YP_104185.1| hypothetical protein BMA2657 [Burkholderia mallei ATCC 23344]
>gi|53723875|ref|YP_104188.1| oxidoreductase, short-chain dehydrogenase/reductase family [Burkholderia mallei ATCC 23344]
>gi|53723880|ref|YP_104193.1| branched-chain amino acid ABC transporter, permease protein, putative [Burkholderia mallei ATCC 23344]
>gi|53723881|ref|YP_104194.1| branched-chain amino acid ABC transporter, ATP-binding protein [Burkholderia mallei ATCC 23344]
>gi|53723883|ref|YP_104268.1| fimbrial assembly protein PilN, putative [Burkholderia mallei ATCC 23344]
>gi|53723884|ref|YP_104269.1| hypothetical protein BMA2751 [Burkholderia mallei ATCC 23344]
>gi|53723886|ref|YP_104272.1| hypothetical protein BMA2754 [Burkholderia mallei ATCC 23344]
>gi|53723893|ref|YP_103204.1| membrane protein, putative [Burkholderia mallei ATCC 23344]
>gi|53723900|ref|YP_104280.1| flagellar protein FliO [Burkholderia mallei ATCC 23344]
>gi|53723903|ref|YP_104283.1| flagellar protein FliL [Burkholderia mallei ATCC 23344]
>gi|53723904|ref|YP_104284.1| hypothetical protein BMA2766 [Burkholderia mallei ATCC 23344]
>gi|53723905|ref|YP_104286.1| LrgA family protein [Burkholderia mallei ATCC 23344]
>gi|53723914|ref|YP_103218.1| hypothetical protein BMA1578 [Burkholderia mallei ATCC 23344]
>gi|53723915|ref|YP_103219.1| hypothetical protein BMA1579 [Burkholderia mallei ATCC 23344]
>gi|53723920|ref|YP_104291.1| RND efflux system, outer membrane lipoprotein, NodT family [Burkholderia mallei ATCC 23344]
>gi|53723922|ref|YP_104294.1| general secretory pathway protein L [Burkholderia mallei ATCC 23344]
>gi|53723923|ref|YP_104293.1| general secretion pathway protein M [Burkholderia mallei ATCC 23344]
>gi|53723943|ref|YP_104315.1| transporter, putative [Burkholderia mallei ATCC 23344]
>gi|53723944|ref|YP_104316.1| phosphoheptose isomerase [Burkholderia mallei ATCC 23344]
>gi|53723946|ref|YP_104318.1| tetrapyrrole methylase family protein [Burkholderia mallei ATCC 23344]
>gi|53723951|ref|YP_104323.1| transcriptional regulator, TetR family [Burkholderia mallei ATCC 23344]
>gi|53723952|ref|YP_104324.1| hypothetical protein BMA2808 [Burkholderia mallei ATCC 23344]
>gi|53723958|ref|YP_104330.1| hypothetical protein BMA2814 [Burkholderia mallei ATCC 23344]
>gi|53723965|ref|YP_104337.1| hypothetical protein BMA2823 [Burkholderia mallei ATCC 23344]
>gi|53723967|ref|YP_104339.1| hypothetical protein BMA2825 [Burkholderia mallei ATCC 23344]
>gi|53723969|ref|YP_104341.1| OmpA domain protein [Burkholderia mallei ATCC 23344]
>gi|53723984|ref|YP_104429.1| hypothetical protein BMA2922 [Burkholderia mallei ATCC 23344]
>gi|53723992|ref|YP_104437.1| transcriptional regulator, LysR family [Burkholderia mallei ATCC 23344]
>gi|53723994|ref|YP_104439.1| hypothetical protein BMA2932 [Burkholderia mallei ATCC 23344]
>gi|53723998|ref|YP_104443.1| branched-chain amino acid ABC transporter, permease protein [Burkholderia mallei ATCC 23344]
>gi|53724000|ref|YP_104446.1| branched-chain amino acid ABC transporter, permease protein [Burkholderia mallei ATCC 23344]
>gi|53724007|ref|YP_104453.1| transporter, putative [Burkholderia mallei ATCC 23344]
>gi|53724011|ref|YP_104457.1| ATP synthase F0, C subunit [Burkholderia mallei ATCC 23344]
>gi|53724012|ref|YP_104458.1| ATP synthase F0, B subunit [Burkholderia mallei ATCC 23344]
>gi|53724025|ref|YP_104471.1| feruloyl-CoA synthetase [Burkholderia mallei ATCC 23344]
>gi|53724026|ref|YP_104472.1| acyl-CoA dehydrogenase domain protein [Burkholderia mallei ATCC 23344]
>gi|53724028|ref|YP_104474.1| hypothetical protein BMA2970 [Burkholderia mallei ATCC 23344]
>gi|53724030|ref|YP_104477.1| transcriptional regulator, PadR family [Burkholderia mallei ATCC 23344]
>gi|53724032|ref|YP_104479.1| cadmium-translocating P-type ATPase [Burkholderia mallei ATCC 23344]
>gi|53724033|ref|YP_104480.1| hypothetical protein BMA2977 [Burkholderia mallei ATCC 23344]
>gi|53724035|ref|YP_104482.1| acyltransferase family protein [Burkholderia mallei ATCC 23344]

>gi|53724036|ref|YP_104483.1| hypothetical protein BMA2980 [Burkholderia mallei ATCC 23344]
>gi|53724045|ref|YP_104563.1| ABC transporter, permease protein [Burkholderia mallei ATCC 23344]
>gi|53724046|ref|YP_104564.1| hypothetical protein BMA3073 [Burkholderia mallei ATCC 23344]
>gi|53724047|ref|YP_104565.1| ABC transporter, permease protein [Burkholderia mallei ATCC 23344]
>gi|53724050|ref|YP_104568.1| hypothetical protein BMA3078 [Burkholderia mallei ATCC 23344]
>gi|53724051|ref|YP_104569.1| hypothetical protein BMA3079 [Burkholderia mallei ATCC 23344]
>gi|53724052|ref|YP_104570.1| membrane protein, putative [Burkholderia mallei ATCC 23344]
>gi|53724053|ref|YP_104571.1| hypothetical protein BMA3081 [Burkholderia mallei ATCC 23344]
>gi|53724054|ref|YP_104572.1| major facilitator family transporter [Burkholderia mallei ATCC 23344]
>gi|53724055|ref|YP_104573.1| membrane protein, putative [Burkholderia mallei ATCC 23344]
>gi|53724058|ref|YP_104576.1| hypothetical protein BMA3086 [Burkholderia mallei ATCC 23344]
>gi|53724059|ref|YP_104577.1| hypothetical protein BMA3087 [Burkholderia mallei ATCC 23344]
>gi|53724062|ref|YP_104581.1| single-strand binding protein [Burkholderia mallei ATCC 23344]
>gi|53724082|ref|YP_104602.1| lipoprotein, putative [Burkholderia mallei ATCC 23344]
>gi|53724085|ref|YP_104605.1| ribosomal protein L25 [Burkholderia mallei ATCC 23344]
>gi|53724093|ref|YP_104613.1| hypothetical protein BMA3129 [Burkholderia mallei ATCC 23344]
>gi|53724098|ref|YP_104618.1| acetyltransferase, GNAT family [Burkholderia mallei ATCC 23344]
>gi|53724105|ref|YP_104625.1| ABC transporter, permease protein [Burkholderia mallei ATCC 23344]
>gi|53724107|ref|YP_103222.1| glycoprotease family protein [Burkholderia mallei ATCC 23344]
>gi|53724110|ref|YP_103224.1| ATP-dependent RNA helicase RhlE, putative [Burkholderia mallei ATCC 23344]
>gi|53724114|ref|YP_103229.1| haloacid dehalogenase, type II [Burkholderia mallei ATCC 23344]
>gi|53724116|ref|YP_103231.1| hypothetical protein BMA1591 [Burkholderia mallei ATCC 23344]
>gi|53724117|ref|YP_103232.1| hypothetical protein BMA1592 [Burkholderia mallei ATCC 23344]
>gi|53724130|ref|YP_103245.1| hypothetical protein BMA1609 [Burkholderia mallei ATCC 23344]
>gi|53724131|ref|YP_103246.1| hypothetical protein BMA1610 [Burkholderia mallei ATCC 23344]
>gi|53724134|ref|YP_103248.1| molybdenum cofactor biosynthesis protein A, putative [Burkholderia mallei ATCC 23344]
>gi|53724149|ref|YP_103264.1| hypothetical protein BMA1630 [Burkholderia mallei ATCC 23344]
>gi|53724153|ref|YP_103268.1| membrane protein, putative [Burkholderia mallei ATCC 23344]
>gi|53724154|ref|YP_103269.1| peptide synthetase-domain protein [Burkholderia mallei ATCC 23344]
>gi|53724157|ref|YP_103272.1| multidrug efflux RND membrane fusion protein MexE [Burkholderia mallei ATCC 23344]
>gi|53724161|ref|YP_103345.1| hypothetical protein BMA1725 [Burkholderia mallei ATCC 23344]
>gi|53724168|ref|YP_103352.1| nitrate reductase, delta subunit [Burkholderia mallei ATCC 23344]
>gi|53724171|ref|YP_103355.1| DNA-binding response regulator NarL [Burkholderia mallei ATCC 23344]
>gi|53724172|ref|YP_103356.1| hypothetical protein BMA1737 [Burkholderia mallei ATCC 23344]
>gi|53724181|ref|YP_103366.1| hypothetical protein BMA1747 [Burkholderia mallei ATCC 23344]
>gi|53724188|ref|YP_103372.1| CoA transferase, CAIB/BAIF family [Burkholderia mallei ATCC 23344]
>gi|53724190|ref|YP_103377.1| lipoprotein, putative [Burkholderia mallei ATCC 23344]
>gi|53724191|ref|YP_103378.1| membrane protein, putative [Burkholderia mallei ATCC 23344]
>gi|53724194|ref|YP_103381.1| EAL domain protein [Burkholderia mallei ATCC 23344]
>gi|53724196|ref|YP_103383.1| exodeoxyribonuclease V, alpha subunit [Burkholderia mallei ATCC 23344]
>gi|53724197|ref|YP_103384.1| exodeoxyribonuclease V, beta subunit [Burkholderia mallei ATCC 23344]
>gi|53724198|ref|YP_103386.1| hypothetical protein BMA1771 [Burkholderia mallei ATCC 23344]
>gi|53724204|ref|YP_103392.1| iron compound ABC transporter, permease protein, putative [Burkholderia mallei ATCC 23344]
>gi|53724207|ref|YP_103395.1| hypothetical protein BMA1783 [Burkholderia mallei ATCC 23344]
>gi|53724209|ref|YP_103397.1| hypothetical protein BMA1785 [Burkholderia mallei ATCC 23344]
>gi|53724223|ref|YP_104121.1| hypothetical protein BMA2583 [Burkholderia mallei ATCC 23344]
>gi|53724225|ref|YP_104123.1| hypothetical protein BMA2585 [Burkholderia mallei ATCC 23344]
>gi|53724231|ref|YP_104198.1| hypothetical protein BMA2672 [Burkholderia mallei ATCC 23344]
>gi|53724237|ref|YP_104204.1| ABC transporter, permease protein [Burkholderia mallei ATCC 23344]
>gi|53724241|ref|YP_104208.1| flagellar biosynthetic protein FliR [Burkholderia mallei ATCC 23344]
>gi|53724242|ref|YP_104209.1| flagellar biosynthetic protein FliQ [Burkholderia mallei ATCC 23344]
>gi|53724246|ref|YP_104215.1| stringent starvation protein B [Burkholderia mallei ATCC 23344]
>gi|53724272|ref|YP_104241.1| hypothetical protein BMA2720 [Burkholderia mallei ATCC 23344]
>gi|53724274|ref|YP_104243.1| lipoprotein VacJ [Burkholderia mallei ATCC 23344]
>gi|53724278|ref|YP_104249.1| thiamine biosynthesis protein ThiS, putative [Burkholderia mallei ATCC 23344]
>gi|53724280|ref|YP_104250.1| oxidoreductase, FAD-binding [Burkholderia mallei ATCC 23344]
>gi|53724289|ref|YP_104259.1| glycerophosphoryl diester phosphodiesterase [Burkholderia mallei ATCC 23344]
>gi|53724301|ref|YP_104347.1| oxidoreductase, 2-nitropropane dioxygenase family [Burkholderia mallei ATCC 23344]
>gi|53724303|ref|YP_104349.1| carboxymethylenebutenolidase [Burkholderia mallei ATCC 23344]
>gi|53724304|ref|YP_104350.1| 6-aminohexanoate-cyclic-dimer hydrolase [Burkholderia mallei ATCC 23344]
>gi|53724316|ref|YP_104364.1| hypothetical protein BMA2853 [Burkholderia mallei ATCC 23344]
>gi|53724318|ref|YP_104366.1| chemotaxis protein CheD [Burkholderia mallei ATCC 23344]
>gi|53724320|ref|YP_104368.1| methyl-accepting chemotaxis protein, putative [Burkholderia mallei ATCC 23344]
>gi|53724322|ref|YP_104370.1| chemotaxis protein CheA [Burkholderia mallei ATCC 23344]
>gi|53724332|ref|YP_104380.1| hypothetical protein BMA2870 [Burkholderia mallei ATCC 23344]
>gi|53724337|ref|YP_104705.1| hypothetical protein BMA3230 [Burkholderia mallei ATCC 23344]
>gi|53724340|ref|YP_104708.1| fatty oxidation complex, alpha subunit, putative [Burkholderia mallei ATCC 23344]
>gi|53724342|ref|YP_104383.1| flagellin [Burkholderia mallei ATCC 23344]
>gi|53724343|ref|YP_104384.1| flagellar hook-associated protein 2 [Burkholderia mallei ATCC 23344]
>gi|53724348|ref|YP_104389.1| hypothetical protein BMA2879 [Burkholderia mallei ATCC 23344]

>gi|53724350|ref|YP_104391.1| oxidoreductase, short chain dehydrogenase/reductase family [Burkholderia mallei ATCC 23344]
>gi|53724351|ref|YP_104392.1| hexapeptide transferase family protein [Burkholderia mallei ATCC 23344]
>gi|53724354|ref|YP_104395.1| LysE family protein [Burkholderia mallei ATCC 23344]
>gi|53724362|ref|YP_104710.1| hypothetical protein BMA3236 [Burkholderia mallei ATCC 23344]
>gi|53724375|ref|YP_104409.1| amino acid ABC transporter, permease protein, putative [Burkholderia mallei ATCC 23344]
>gi|53724389|ref|YP_104488.1| hypothetical protein BMA2985 [Burkholderia mallei ATCC 23344]
>gi|53724394|ref|YP_104493.1| hypothetical protein BMA2990 [Burkholderia mallei ATCC 23344]
>gi|53724399|ref|YP_104499.1| hypothetical protein BMA2996 [Burkholderia mallei ATCC 23344]
>gi|53724400|ref|YP_104500.1| lipoprotein, putative [Burkholderia mallei ATCC 23344]
>gi|53724402|ref|YP_104502.1| hypothetical protein BMA2999 [Burkholderia mallei ATCC 23344]
>gi|53724404|ref|YP_104504.1| membrane protein, putative [Burkholderia mallei ATCC 23344]
>gi|53724406|ref|YP_104506.1| cytochrome c family protein [Burkholderia mallei ATCC 23344]
>gi|53724409|ref|YP_104510.1| lipoprotein, putative [Burkholderia mallei ATCC 23344]
>gi|53724416|ref|YP_104517.1| hypothetical protein BMA3015 [Burkholderia mallei ATCC 23344]
>gi|53724418|ref|YP_104520.1| lipoprotein, putative [Burkholderia mallei ATCC 23344]
>gi|53724419|ref|YP_104521.1| ecotin [Burkholderia mallei ATCC 23344]
>gi|53724420|ref|YP_104522.1| D-alanyl-D-alanine carboxypeptidase family protein [Burkholderia mallei ATCC 23344]
>gi|53724424|ref|YP_104527.1| hypothetical protein BMA3028 [Burkholderia mallei ATCC 23344]
>gi|53724426|ref|YP_104529.1| acyl transferase domain protein [Burkholderia mallei ATCC 23344]
>gi|53724427|ref|YP_104530.1| triphosphoribosyl-dephospho-CoA synthase [Burkholderia mallei ATCC 23344]
>gi|53724428|ref|YP_104531.1| mdcG protein [Burkholderia mallei ATCC 23344]
>gi|53724429|ref|YP_104532.1| malonate decarboxylase, gamma subunit [Burkholderia mallei ATCC 23344]
>gi|53724430|ref|YP_104533.1| malonate decarboxylase, beta subunit [Burkholderia mallei ATCC 23344]
>gi|53724434|ref|YP_104537.1| malonate transporter, L subunit [Burkholderia mallei ATCC 23344]
>gi|53724438|ref|YP_104541.1| hypothetical protein BMA3043 [Burkholderia mallei ATCC 23344]
>gi|53724439|ref|YP_104543.1| hypothetical protein BMA3046 [Burkholderia mallei ATCC 23344]
>gi|53724445|ref|YP_104549.1| oxidoreductase, short-chain dehydrogenase/reductase family [Burkholderia mallei ATCC 23344]
>gi|53724446|ref|YP_104550.1| hypothetical protein BMA3057 [Burkholderia mallei ATCC 23344]
>gi|53724455|ref|YP_104730.1| cobalamin synthesis protein/P47K family protein [Burkholderia mallei ATCC 23344]
>gi|53724456|ref|YP_104731.1| hypothetical protein BMA3257 [Burkholderia mallei ATCC 23344]
>gi|53724458|ref|YP_104733.1| hypothetical protein BMA3259 [Burkholderia mallei ATCC 23344]
>gi|53724462|ref|YP_104737.1| hypothetical protein BMA3263 [Burkholderia mallei ATCC 23344]
>gi|53724466|ref|YP_104741.1| membrane protein, putative [Burkholderia mallei ATCC 23344]
>gi|53724467|ref|YP_104742.1| major facilitator family transporter [Burkholderia mallei ATCC 23344]
>gi|53724468|ref|YP_104743.1| hypothetical protein BMA3270 [Burkholderia mallei ATCC 23344]
>gi|53724474|ref|YP_104556.1| membrane protein, putative [Burkholderia mallei ATCC 23344]
>gi|53724476|ref|YP_104558.1| hypothetical protein BMA3065 [Burkholderia mallei ATCC 23344]
>gi|53724478|ref|YP_104630.1| major facilitator family transporter [Burkholderia mallei ATCC 23344]
>gi|53724481|ref|YP_104633.1| hypothetical protein BMA3150 [Burkholderia mallei ATCC 23344]
>gi|53724482|ref|YP_104634.1| ABC transporter, permease protein, putative [Burkholderia mallei ATCC 23344]
>gi|53724488|ref|YP_104640.1| hypothetical protein BMA3157 [Burkholderia mallei ATCC 23344]
>gi|53724497|ref|YP_104651.1| PTS system, N-acetylglucosamine-specific IIABC component [Burkholderia mallei ATCC 23344]
>gi|53724518|ref|YP_104676.1| ComF family protein [Burkholderia mallei ATCC 23344]
>gi|53724520|ref|YP_104678.1| hypothetical protein BMA3202 [Burkholderia mallei ATCC 23344]
>gi|53724521|ref|YP_104679.1| hypothetical protein BMA3203 [Burkholderia mallei ATCC 23344]
>gi|53724522|ref|YP_104680.1| glycerol-3-phosphate dehydrogenase (NAD(P)+) [Burkholderia mallei ATCC 23344]
>gi|53724537|ref|YP_104696.1| Mg chelatase, subunit D/I family protein [Burkholderia mallei ATCC 23344]
>gi|53724538|ref|YP_104697.1| hypothetical protein BMA3221 [Burkholderia mallei ATCC 23344]
>gi|53724541|ref|YP_104700.1| hypothetical protein BMA3224 [Burkholderia mallei ATCC 23344]
>gi|53724553|ref|YP_104785.1| NADH-ubiquinone oxidoreductase, putative [Burkholderia mallei ATCC 23344]
>gi|53724556|ref|YP_104788.1| hypothetical protein BMA3318 [Burkholderia mallei ATCC 23344]
>gi|53724559|ref|YP_104792.1| negative regulator of flagellin synthesis FlgM, putative [Burkholderia mallei ATCC 23344]
>gi|53724560|ref|YP_104793.1| flagella basal body P-ring formation protein FlgA [Burkholderia mallei ATCC 23344]
>gi|53724561|ref|YP_104794.1| hypothetical protein BMA3324 [Burkholderia mallei ATCC 23344]
>gi|53724565|ref|YP_104798.1| flagellar hook protein FlgE [Burkholderia mallei ATCC 23344]
>gi|53724577|ref|YP_104810.1| alkylphosphonate utilization operon protein PhnA, putative [Burkholderia mallei ATCC 23344]
>gi|53724580|ref|YP_104813.1| lipoprotein, putative [Burkholderia mallei ATCC 23344]
>gi|53724582|ref|YP_104815.1| glycosyl transferase, group 2 family protein [Burkholderia mallei ATCC 23344]
>gi|53724584|ref|YP_104818.1| hypothetical protein BMA3353 [Burkholderia mallei ATCC 23344]
>gi|53724586|ref|YP_104820.1| hypothetical protein BMA3355 [Burkholderia mallei ATCC 23344]
>gi|53724597|ref|YP_104832.1| outer membrane efflux protein [Burkholderia mallei ATCC 23344]
>gi|53724600|ref|YP_104835.1| hypothetical protein BMA3377 [Burkholderia mallei ATCC 23344]
>gi|53724606|ref|YP_104841.1| hypothetical protein BMA3383 [Burkholderia mallei ATCC 23344]
>gi|53724611|ref|YP_101917.1| hypothetical protein BMA0068 [Burkholderia mallei ATCC 23344]
>gi|53724612|ref|YP_101918.1| biotin--acetyl-CoA-carboxylase ligase [Burkholderia mallei ATCC 23344]
>gi|53724614|ref|YP_101920.1| hypothetical protein BMA0071 [Burkholderia mallei ATCC 23344]
>gi|53724617|ref|YP_101923.1| hypothetical protein BMA0075 [Burkholderia mallei ATCC 23344]
>gi|53724621|ref|YP_101927.1| hypothetical protein BMA0079 [Burkholderia mallei ATCC 23344]
>gi|53724624|ref|YP_101931.1| sporulation-related repeat protein [Burkholderia mallei ATCC 23344]
>gi|53724626|ref|YP_101933.1| oxidoreductase, short-chain dehydrogenase/reductase family [Burkholderia mallei ATCC 23344]

>gi|53724631|ref|YP_101938.1| peptide ABC transporter, ATP-binding protein [Burkholderia mallei ATCC 23344]
>gi|53724636|ref|YP_101943.1| oxidoreductase, short chain dehydrogenase/reductase family [Burkholderia mallei ATCC 23344]
>gi|53724640|ref|YP_101947.1| dethiobiotin synthetase [Burkholderia mallei ATCC 23344]
>gi|53724642|ref|YP_101949.1| cutC family protein [Burkholderia mallei ATCC 23344]
>gi|53724644|ref|YP_101950.1| hypothetical protein BMA0105 [Burkholderia mallei ATCC 23344]
>gi|53724646|ref|YP_101953.1| hypothetical protein BMA0109 [Burkholderia mallei ATCC 23344]
>gi|53724648|ref|YP_101956.1| hypothetical protein BMA0112 [Burkholderia mallei ATCC 23344]
>gi|53724652|ref|YP_101960.1| DNA-3-methyladenine glycosylase II [Burkholderia mallei ATCC 23344]
>gi|53724654|ref|YP_101962.1| RNA polymerase sigma factor RpoD, putative [Burkholderia mallei ATCC 23344]
>gi|53724661|ref|YP_101969.1| hypothetical protein BMA0127 [Burkholderia mallei ATCC 23344]
>gi|53724673|ref|YP_101981.1| DNA processing protein DprA, putative [Burkholderia mallei ATCC 23344]
>gi|53724675|ref|YP_102054.1| apolipoprotein N-acyltransferase [Burkholderia mallei ATCC 23344]
>gi|53724678|ref|YP_102058.1| hypothetical protein BMA0224 [Burkholderia mallei ATCC 23344]
>gi|53724683|ref|YP_102062.1| major facilitator family transporter [Burkholderia mallei ATCC 23344]
>gi|53724684|ref|YP_102063.1| DNA-binding protein [Burkholderia mallei ATCC 23344]
>gi|53724691|ref|YP_102070.1| hypothetical protein BMA0238 [Burkholderia mallei ATCC 23344]
>gi|53724692|ref|YP_102071.1| glycerol uptake facilitator protein [Burkholderia mallei ATCC 23344]
>gi|53724700|ref|YP_102079.1| ATP-dependent RNA helicase RhlE [Burkholderia mallei ATCC 23344]
>gi|53724704|ref|YP_102083.1| copper resistance protein, putative [Burkholderia mallei ATCC 23344]
>gi|53724705|ref|YP_102084.1| hypothetical protein BMA0253 [Burkholderia mallei ATCC 23344]
>gi|53724707|ref|YP_102086.1| hypothetical protein BMA0255 [Burkholderia mallei ATCC 23344]
>gi|53724719|ref|YP_102099.1| regulatory protein RecX, putative [Burkholderia mallei ATCC 23344]
>gi|53724727|ref|YP_102107.1| hypothetical protein BMA0281 [Burkholderia mallei ATCC 23344]
>gi|53724730|ref|YP_102112.1| hypothetical protein BMA0287 [Burkholderia mallei ATCC 23344]
>gi|53724737|ref|YP_102119.1| AzlC family protein [Burkholderia mallei ATCC 23344]
>gi|53724738|ref|YP_102120.1| membrane protein, putative [Burkholderia mallei ATCC 23344]
>gi|53724739|ref|YP_102122.1| hypothetical protein BMA0297 [Burkholderia mallei ATCC 23344]
>gi|53724746|ref|YP_102200.1| hypothetical protein BMA0384 [Burkholderia mallei ATCC 23344]
>gi|53724748|ref|YP_102202.1| dihydrofolate reductase [Burkholderia mallei ATCC 23344]
>gi|53724765|ref|YP_102219.1| membrane protein, putative [Burkholderia mallei ATCC 23344]
>gi|53724772|ref|YP_102226.1| D-methionine ABC transporter, permease protein [Burkholderia mallei ATCC 23344]
>gi|53724776|ref|YP_102230.1| murein transglycosylase domain protein [Burkholderia mallei ATCC 23344]
>gi|53724778|ref|YP_102232.1| hypothetical protein BMA0419 [Burkholderia mallei ATCC 23344]
>gi|53724779|ref|YP_102233.1| competence protein ComE [Burkholderia mallei ATCC 23344]
>gi|53724784|ref|YP_102238.1| hypothetical protein BMA0425 [Burkholderia mallei ATCC 23344]
>gi|53724790|ref|YP_102244.1| hypothetical protein BMA0431 [Burkholderia mallei ATCC 23344]
>gi|53724793|ref|YP_102247.1| hypothetical protein BMA0434 [Burkholderia mallei ATCC 23344]
>gi|53724795|ref|YP_102249.1| OmpA family protein [Burkholderia mallei ATCC 23344]
>gi|53724800|ref|YP_102255.1| hypothetical protein BMA0445 [Burkholderia mallei ATCC 23344]
>gi|53724807|ref|YP_102262.1| TPR domain protein [Burkholderia mallei ATCC 23344]
>gi|53724809|ref|YP_102264.1| glutamine amidotransferase, class I [Burkholderia mallei ATCC 23344]
>gi|53724810|ref|YP_102265.1| hypothetical protein BMA0456 [Burkholderia mallei ATCC 23344]
>gi|53724822|ref|YP_102764.1| segregation and condensation protein B [Burkholderia mallei ATCC 23344]
>gi|53724825|ref|YP_102760.1| translation initiation factor IF-2 [Burkholderia mallei ATCC 23344]
>gi|53724835|ref|YP_102750.1| 2-oxoglutarate dehydrogenase, E2 component, dihydrolipoamide succinyltransferase [Burkholderia mallei ATCC 23344]
>gi|53724843|ref|YP_102742.1| lipoprotein, putative [Burkholderia mallei ATCC 23344]
>gi|53724844|ref|YP_102741.1| hypothetical protein BMA1042 [Burkholderia mallei ATCC 23344]
>gi|53724849|ref|YP_102735.1| hypothetical protein BMA1028 [Burkholderia mallei ATCC 23344]
>gi|53724850|ref|YP_102734.1| outer membrane protein, putative [Burkholderia mallei ATCC 23344]
>gi|53724856|ref|YP_102728.1| hypothetical protein BMA1017 [Burkholderia mallei ATCC 23344]
>gi|53724860|ref|YP_104748.1| flagellar hook-length control protein [Burkholderia mallei ATCC 23344]
>gi|53724861|ref|YP_104749.1| flagellar FliJ protein [Burkholderia mallei ATCC 23344]
>gi|53724863|ref|YP_104751.1| flagellar assembly protein FliH [Burkholderia mallei ATCC 23344]
>gi|53724866|ref|YP_104754.1| flagellar hook-basal body complex protein FliE, putative [Burkholderia mallei ATCC 23344]
>gi|53724869|ref|YP_104757.1| hypothetical protein BMA3285 [Burkholderia mallei ATCC 23344]
>gi|53724874|ref|YP_104762.1| DNA-binding response regulator, LuxR family [Burkholderia mallei ATCC 23344]
>gi|53724879|ref|YP_104767.1| hypothetical protein BMA3295 [Burkholderia mallei ATCC 23344]
>gi|53724887|ref|YP_104842.1| hypothetical protein BMA3384 [Burkholderia mallei ATCC 23344]
>gi|53724892|ref|YP_104847.1| methyl-accepting chemotaxis protein, putative [Burkholderia mallei ATCC 23344]
>gi|53724894|ref|YP_104849.1| hypothetical protein BMA3391 [Burkholderia mallei ATCC 23344]
>gi|53724901|ref|YP_101862.1| chromosomal replication initiator protein DnaA [Burkholderia mallei ATCC 23344]
>gi|53724908|ref|YP_101871.1| hypothetical protein BMA0010 [Burkholderia mallei ATCC 23344]
>gi|53724911|ref|YP_101874.1| protein kinase domain protein [Burkholderia mallei ATCC 23344]
>gi|53724913|ref|YP_101876.1| hypothetical protein BMA0023 [Burkholderia mallei ATCC 23344]
>gi|53724916|ref|YP_101879.1| EAL/GGDEF domain protein [Burkholderia mallei ATCC 23344]
>gi|53724923|ref|YP_101886.1| PAP2 family protein [Burkholderia mallei ATCC 23344]
>gi|53724936|ref|YP_101899.1| hypothetical protein BMA0050 [Burkholderia mallei ATCC 23344]
>gi|53724945|ref|YP_101908.1| hypothetical protein BMA0059 [Burkholderia mallei ATCC 23344]
>gi|53724948|ref|YP_101911.1| membrane protein, putative [Burkholderia mallei ATCC 23344]

>gi|53724956|ref|YP_101989.1| DNA-binding response regulator [Burkholderia mallei ATCC 23344]
>gi|53724961|ref|YP_101994.1| hypothetical protein BMA0155 [Burkholderia mallei ATCC 23344]
>gi|53724963|ref|YP_101996.1| hypothetical protein BMA0157 [Burkholderia mallei ATCC 23344]
>gi|53724965|ref|YP_101998.1| penicillin-binding protein 2 [Burkholderia mallei ATCC 23344]
>gi|53724967|ref|YP_102000.1| rod shape-determining protein MreC [Burkholderia mallei ATCC 23344]
>gi|53724970|ref|YP_102003.1| glutamyl-tRNA(Gln) amidotransferase, A subunit [Burkholderia mallei ATCC 23344]
>gi|53724975|ref|YP_102008.1| hypothetical protein BMA0169 [Burkholderia mallei ATCC 23344]
>gi|53724977|ref|YP_102010.1| esterase, putative [Burkholderia mallei ATCC 23344]
>gi|53724989|ref|YP_102021.1| hypothetical protein BMA0182 [Burkholderia mallei ATCC 23344]
>gi|53724992|ref|YP_102025.1| hypothetical protein BMA0187 [Burkholderia mallei ATCC 23344]
>gi|53724994|ref|YP_102027.1| thiopurine S-methyltransferase family protein [Burkholderia mallei ATCC 23344]
>gi|53725002|ref|YP_102724.1| polyhydroxyalkanoate depolymerase domain protein [Burkholderia mallei ATCC 23344]
>gi|53725004|ref|YP_102722.1| hypothetical protein BMA1007 [Burkholderia mallei ATCC 23344]
>gi|53725012|ref|YP_102040.1| hypothetical protein BMA0205 [Burkholderia mallei ATCC 23344]
>gi|53725013|ref|YP_102041.1| nucleotidyltransferase family protein [Burkholderia mallei ATCC 23344]
>gi|53725017|ref|YP_102045.1| 4-hydroxythreonine-4-phosphate dehydrogenase [Burkholderia mallei ATCC 23344]
>gi|53725024|ref|YP_102648.1| hypothetical protein BMA0921 [Burkholderia mallei ATCC 23344]
>gi|53725032|ref|YP_102640.1| iolD protein [Burkholderia mallei ATCC 23344]
>gi|53725043|ref|YP_102636.1| branched-chain amino acid ABC transporter, permease/ATP binding protein, putative [Burkholderia mallei ATCC 23344]
>gi|53725044|ref|YP_102635.1| branched-chain amino acid ABC transporter, permease protein, putative [Burkholderia mallei ATCC 23344]
>gi|53725055|ref|YP_102130.1| hypothetical protein BMA0306 [Burkholderia mallei ATCC 23344]
>gi|53725059|ref|YP_102625.1| CAIB/BAIF family protein [Burkholderia mallei ATCC 23344]
>gi|53725060|ref|YP_102624.1| hypothetical protein BMA0893 [Burkholderia mallei ATCC 23344]
>gi|53725074|ref|YP_102616.1| hypothetical protein BMA0884 [Burkholderia mallei ATCC 23344]
>gi|53725075|ref|YP_102615.1| multidrug resistance protein, putative [Burkholderia mallei ATCC 23344]
>gi|53725078|ref|YP_102139.1| RND efflux system, outer membrane lipoprotein, NodT family [Burkholderia mallei ATCC 23344]
>gi|53725083|ref|YP_102612.1| sensor histidine kinase/response regulator [Burkholderia mallei ATCC 23344]
>gi|53725089|ref|YP_102146.1| cation ABC transporter, permease protein, putative [Burkholderia mallei ATCC 23344]
>gi|53725097|ref|YP_102605.1| hypothetical protein BMA0865 [Burkholderia mallei ATCC 23344]
>gi|53725099|ref|YP_102603.1| lipoprotein, putative [Burkholderia mallei ATCC 23344]
>gi|53725104|ref|YP_102602.1| hypothetical protein BMA0862 [Burkholderia mallei ATCC 23344]
>gi|53725111|ref|YP_102594.1| hypothetical protein BMA0853 [Burkholderia mallei ATCC 23344]
>gi|53725115|ref|YP_102158.1| esterase, putative [Burkholderia mallei ATCC 23344]
>gi|53725116|ref|YP_102159.1| major facilitator family transporter [Burkholderia mallei ATCC 23344]
>gi|53725118|ref|YP_102161.1| xylulokinase [Burkholderia mallei ATCC 23344]
>gi|53725121|ref|YP_102592.1| hypothetical protein BMA0848 [Burkholderia mallei ATCC 23344]
>gi|53725126|ref|YP_102165.1| aldehyde dehydrogenase family protein [Burkholderia mallei ATCC 23344]
>gi|53725128|ref|YP_102167.1| hypothetical protein BMA0349 [Burkholderia mallei ATCC 23344]
>gi|53725129|ref|YP_102168.1| 4-hydroxybenzoate transporter, putative [Burkholderia mallei ATCC 23344]
>gi|53725134|ref|YP_102524.1| hypothetical protein BMA0761 [Burkholderia mallei ATCC 23344]
>gi|53725144|ref|YP_102175.1| hypothetical protein BMA0357 [Burkholderia mallei ATCC 23344]
>gi|53725147|ref|YP_102180.1| membrane protein, putative [Burkholderia mallei ATCC 23344]
>gi|53725150|ref|YP_102515.1| hypothetical protein BMA0749 [Burkholderia mallei ATCC 23344]
>gi|53725152|ref|YP_102181.1| hypothetical protein BMA0364 [Burkholderia mallei ATCC 23344]
>gi|53725164|ref|YP_102507.1| hypothetical protein BMA0739 [Burkholderia mallei ATCC 23344]
>gi|53725172|ref|YP_102194.1| ribosomal protein S20 [Burkholderia mallei ATCC 23344]
>gi|53725173|ref|YP_102506.1| endo/excinuclease domain protein [Burkholderia mallei ATCC 23344]
>gi|53725177|ref|YP_102502.1| major facilitator family transporter [Burkholderia mallei ATCC 23344]
>gi|53725178|ref|YP_102501.1| hypothetical protein BMA0732 [Burkholderia mallei ATCC 23344]
>gi|53725179|ref|YP_102500.1| uroporphyrinogen-III synthase HemD/uroporphyrinogen III methylase HemX, putative [Burkholderia mallei ATCC 23344]
>gi|53725180|ref|YP_102499.1| porphobilinogen deaminase [Burkholderia mallei ATCC 23344]
>gi|53725183|ref|YP_102496.1| hypothetical protein BMA0727 [Burkholderia mallei ATCC 23344]
>gi|53725184|ref|YP_102495.1| methlytransferase, UbiE/COQ5 family [Burkholderia mallei ATCC 23344]
>gi|53725187|ref|YP_102266.1| amidase family protein [Burkholderia mallei ATCC 23344]
>gi|53725189|ref|YP_102268.1| hypothetical protein BMA0459 [Burkholderia mallei ATCC 23344]
>gi|53725203|ref|YP_102274.1| lipoprotein, putative [Burkholderia mallei ATCC 23344]
>gi|53725209|ref|YP_102280.1| hypothetical protein BMA0472 [Burkholderia mallei ATCC 23344]
>gi|53725211|ref|YP_102482.1| ParA family protein [Burkholderia mallei ATCC 23344]
>gi|53725212|ref|YP_102481.1| ompA family protein [Burkholderia mallei ATCC 23344]
>gi|53725213|ref|YP_102480.1| hypothetical protein BMA0710 [Burkholderia mallei ATCC 23344]
>gi|53725216|ref|YP_102477.1| hypothetical protein BMA0707 [Burkholderia mallei ATCC 23344]
>gi|53725217|ref|YP_102476.1| hypothetical protein BMA0706 [Burkholderia mallei ATCC 23344]
>gi|53725225|ref|YP_102473.1| hypothetical protein BMA0702 [Burkholderia mallei ATCC 23344]
>gi|53725229|ref|YP_102469.1| DoxD-like family protein [Burkholderia mallei ATCC 23344]
>gi|53725234|ref|YP_102290.1| mulitcopper oxidase domain protein [Burkholderia mallei ATCC 23344]
>gi|53725238|ref|YP_102466.1| cobalamin biosynthesis protein CobD [Burkholderia mallei ATCC 23344]
>gi|53725239|ref|YP_102465.1| alpha-ribazole-5`-phosphate phosphatase [Burkholderia mallei ATCC 23344]
>gi|53725242|ref|YP_102461.1| cobalamin-5-phosphate synthase [Burkholderia mallei ATCC 23344]

>gi|53725243|ref|YP_102462.1| phosphoglycerate mutase family protein [Burkholderia mallei ATCC 23344]
>gi|53725246|ref|YP_102296.1| hypothetical protein BMA0494 [Burkholderia mallei ATCC 23344]
>gi|53725250|ref|YP_102459.1| iron compound ABC transporter, ATP-binding protein [Burkholderia mallei ATCC 23344]
>gi|53725251|ref|YP_102458.1| iron compound ABC transporter, permease protein [Burkholderia mallei ATCC 23344]
>gi|53725257|ref|YP_102302.1| multidrug resistance protein NorM, putative [Burkholderia mallei ATCC 23344]
>gi|53725259|ref|YP_102304.1| hypothetical protein BMA0505 [Burkholderia mallei ATCC 23344]
>gi|53725282|ref|YP_102795.1| polysaccharide deacetylase family protein [Burkholderia mallei ATCC 23344]
>gi|53725284|ref|YP_102792.1| hypothetical protein BMA1102 [Burkholderia mallei ATCC 23344]
>gi|53725285|ref|YP_102791.1| hypothetical protein BMA1101 [Burkholderia mallei ATCC 23344]
>gi|53725290|ref|YP_102374.1| hypothetical protein BMA0583 [Burkholderia mallei ATCC 23344]
>gi|53725298|ref|YP_102785.1| ribosomal protein L35 [Burkholderia mallei ATCC 23344]
>gi|53725302|ref|YP_102370.1| syl transferase, group 2 family protein [Burkholderia mallei ATCC 23344]
>gi|53725305|ref|YP_102367.1| hypothetical protein BMA0575 [Burkholderia mallei ATCC 23344]
>gi|53725314|ref|YP_102717.1| dihydroxyacetone kinase [Burkholderia mallei ATCC 23344]
>gi|53725315|ref|YP_102716.1| hypothetical protein BMA1000 [Burkholderia mallei ATCC 23344]
>gi|53725316|ref|YP_102361.1| hypothetical protein BMA0569 [Burkholderia mallei ATCC 23344]
>gi|53725325|ref|YP_102714.1| transporter, putative [Burkholderia mallei ATCC 23344]
>gi|53725338|ref|YP_102708.1| copper ABC transporter, permease protein NosY, putative [Burkholderia mallei ATCC 23344]
>gi|53725341|ref|YP_102705.1| cytochrome c family protein [Burkholderia mallei ATCC 23344]
>gi|53725343|ref|YP_102703.1| mechanosensitive ion channel family [Burkholderia mallei ATCC 23344]
>gi|53725351|ref|YP_102339.1| ribonuclease III/hypothetical protein, fusion [Burkholderia mallei ATCC 23344]
>gi|53725353|ref|YP_102700.1| hypothetical protein BMA0982 [Burkholderia mallei ATCC 23344]
>gi|53725354|ref|YP_102699.1| hypothetical protein BMA0981 [Burkholderia mallei ATCC 23344]
>gi|53725363|ref|YP_102333.1| sigma factor algU regulatory protein MucA, putative [Burkholderia mallei ATCC 23344]
>gi|53725365|ref|YP_102331.1| lipoprotein, putative [Burkholderia mallei ATCC 23344]
>gi|53725374|ref|YP_102693.1| transcriptional regulator, AsnC family [Burkholderia mallei ATCC 23344]
>gi|53725377|ref|YP_102322.1| Maf family protein [Burkholderia mallei ATCC 23344]
>gi|53725386|ref|YP_102688.1| hypothetical protein BMA0964 [Burkholderia mallei ATCC 23344]
>gi|53725389|ref|YP_102685.1| hypothetical protein BMA0960 [Burkholderia mallei ATCC 23344]
>gi|53725392|ref|YP_103460.1| glucose 1-dehydrogenase [Burkholderia mallei ATCC 23344]
>gi|53725393|ref|YP_103461.1| hypothetical protein BMA1860 [Burkholderia mallei ATCC 23344]
>gi|53725397|ref|YP_103465.1| amidase family protein [Burkholderia mallei ATCC 23344]
>gi|53725399|ref|YP_102683.1| transcriptional regulator, LysR family [Burkholderia mallei ATCC 23344]
>gi|53725403|ref|YP_102679.1| efflux transporter, RND family, MFP subunit [Burkholderia mallei ATCC 23344]
>gi|53725405|ref|YP_102677.1| RND efflux system, outer membrane lipoprotein, NodT family [Burkholderia mallei ATCC 23344]
>gi|53725406|ref|YP_103466.1| hypothetical protein BMA1865 [Burkholderia mallei ATCC 23344]
>gi|53725410|ref|YP_103470.1| hypothetical protein BMA1871 [Burkholderia mallei ATCC 23344]
>gi|53725412|ref|YP_102676.1| hypothetical protein BMA0951 [Burkholderia mallei ATCC 23344]
>gi|53725426|ref|YP_102667.1| hypothetical protein BMA0940 [Burkholderia mallei ATCC 23344]
>gi|53725428|ref|YP_102665.1| Ser/Thr protein phosphatase family protein [Burkholderia mallei ATCC 23344]
>gi|53725429|ref|YP_102664.1| hypothetical protein BMA0937 [Burkholderia mallei ATCC 23344]
>gi|53725431|ref|YP_102662.1| RND efflux system, outer membrane lipoprotein, NodT family protein [Burkholderia mallei ATCC 23344]
>gi|53725433|ref|YP_103478.1| oxidoreductase, short chain dehydrogenase/reductase family [Burkholderia mallei ATCC 23344]
>gi|53725440|ref|YP_102659.1| hypothetical protein BMA0932 [Burkholderia mallei ATCC 23344]
>gi|53725449|ref|YP_103486.1| maf protein [Burkholderia mallei ATCC 23344]
>gi|53725456|ref|YP_102590.1| hypothetical protein BMA0844 [Burkholderia mallei ATCC 23344]
>gi|53725459|ref|YP_102587.1| ompA family protein [Burkholderia mallei ATCC 23344]
>gi|53725460|ref|YP_102586.1| haemagluttinin family protein [Burkholderia mallei ATCC 23344]
>gi|53725461|ref|YP_102585.1| hypothetical protein BMA0839 [Burkholderia mallei ATCC 23344]
>gi|53725465|ref|YP_103496.1| hypothetical protein BMA1901 [Burkholderia mallei ATCC 23344]
>gi|53725466|ref|YP_103497.1| major facilitator superfamily protein [Burkholderia mallei ATCC 23344]
>gi|53725467|ref|YP_103498.1| hypothetical protein BMA1903 [Burkholderia mallei ATCC 23344]
>gi|53725470|ref|YP_102583.1| hypothetical protein BMA0837 [Burkholderia mallei ATCC 23344]
>gi|53725485|ref|YP_102575.1| hypothetical protein BMA0823 [Burkholderia mallei ATCC 23344]
>gi|53725490|ref|YP_103511.1| hypothetical protein BMA1916 [Burkholderia mallei ATCC 23344]
>gi|53725491|ref|YP_103513.1| hypothetical protein BMA1918 [Burkholderia mallei ATCC 23344]
>gi|53725493|ref|YP_103514.1| hypothetical protein BMA1920 [Burkholderia mallei ATCC 23344]
>gi|53725496|ref|YP_102571.1| 4-alpha-glucanotransferase [Burkholderia mallei ATCC 23344]
>gi|53725497|ref|YP_102570.1| maltooligosyl trehalose synthase, putative [Burkholderia mallei ATCC 23344]
>gi|53725502|ref|YP_103518.1| cell division protein FtsK, putative [Burkholderia mallei ATCC 23344]
>gi|53725503|ref|YP_103519.1| hypothetical protein BMA1925 [Burkholderia mallei ATCC 23344]
>gi|53725511|ref|YP_102562.1| lipoprotein NlpD, putative [Burkholderia mallei ATCC 23344]
>gi|53725514|ref|YP_102559.1| lipoprotein, putative [Burkholderia mallei ATCC 23344]
>gi|53725518|ref|YP_102554.1| hypothetical protein BMA0792 [Burkholderia mallei ATCC 23344]
>gi|53725520|ref|YP_102552.1| PAP2 family protein [Burkholderia mallei ATCC 23344]
>gi|53725521|ref|YP_103524.1| iron-sulfur cluster-binding protein [Burkholderia mallei ATCC 23344]
>gi|53725525|ref|YP_103599.1| hypothetical protein BMA2017 [Burkholderia mallei ATCC 23344]
>gi|53725528|ref|YP_102549.1| hypothetical protein BMA0787 [Burkholderia mallei ATCC 23344]
>gi|53725535|ref|YP_103602.1| acetyltransferase, GNAT family [Burkholderia mallei ATCC 23344]
>gi|53725538|ref|YP_103605.1| drug resistance transporter, EmrB/QacA subfamily [Burkholderia mallei ATCC 23344]

>gi|53725539|ref|YP_103606.1| membrane protein, putative [Burkholderia mallei ATCC 23344]
>gi|53725540|ref|YP_103607.1| hypothetical protein BMA2025 [Burkholderia mallei ATCC 23344]
>gi|53725541|ref|YP_102544.1| phosphate ABC transporter, permease protein [Burkholderia mallei ATCC 23344]
>gi|53725550|ref|YP_103611.1| transport-associated domain protein [Burkholderia mallei ATCC 23344]
>gi|53725554|ref|YP_103615.1| iron compound ABC transporter, permease protein [Burkholderia mallei ATCC 23344]
>gi|53725557|ref|YP_103618.1| hypothetical protein BMA2037 [Burkholderia mallei ATCC 23344]
>gi|53725560|ref|YP_102538.1| hypothetical protein BMA0775 [Burkholderia mallei ATCC 23344]
>gi|53725561|ref|YP_102537.1| membrane protein, putative [Burkholderia mallei ATCC 23344]
>gi|53725564|ref|YP_102534.1| homoserine/threonine efflux protein, putative [Burkholderia mallei ATCC 23344]
>gi|53725569|ref|YP_103623.1| transporter, putative [Burkholderia mallei ATCC 23344]
>gi|53725570|ref|YP_103624.1| hypothetical protein BMA2044 [Burkholderia mallei ATCC 23344]
>gi|53725574|ref|YP_103628.1| hypothetical protein BMA2048 [Burkholderia mallei ATCC 23344]
>gi|53725578|ref|YP_103632.1| major facilitator family transporter [Burkholderia mallei ATCC 23344]
>gi|53725579|ref|YP_103633.1| transporter, putative [Burkholderia mallei ATCC 23344]
>gi|53725580|ref|YP_103634.1| hypothetical protein BMA2054 [Burkholderia mallei ATCC 23344]
>gi|53725582|ref|YP_102530.1| major facilitator family tranporter [Burkholderia mallei ATCC 23344]
>gi|53725586|ref|YP_102456.1| hypothetical protein BMA0684 [Burkholderia mallei ATCC 23344]
>gi|53725597|ref|YP_102453.1| isoleucine biosynthesis transcriptional activator [Burkholderia mallei ATCC 23344]
>gi|53725601|ref|YP_102449.1| hypothetical protein BMA0674 [Burkholderia mallei ATCC 23344]
>gi|53725602|ref|YP_103643.1| transcriptional regulator, IclR family [Burkholderia mallei ATCC 23344]
>gi|53725608|ref|YP_103649.1| type IV pilus biogenesis protein, putative [Burkholderia mallei ATCC 23344]
>gi|53725613|ref|YP_102444.1| cbiX protein [Burkholderia mallei ATCC 23344]
>gi|53725617|ref|YP_103650.1| hypothetical protein BMA2071 [Burkholderia mallei ATCC 23344]
>gi|53725620|ref|YP_103655.1| oxidoreductase, short-chain dehydrogenase/reductase family [Burkholderia mallei ATCC 23344]
>gi|53725624|ref|YP_103659.1| hypothetical protein BMA2080 [Burkholderia mallei ATCC 23344]
>gi|53725629|ref|YP_102436.1| hypothetical protein BMA0661 [Burkholderia mallei ATCC 23344]
>gi|53725643|ref|YP_102429.1| hypothetical protein BMA0651 [Burkholderia mallei ATCC 23344]
>gi|53725648|ref|YP_104024.1| thiamine-monophosphate kinase [Burkholderia mallei ATCC 23344]
>gi|53725650|ref|YP_104022.1| ribonuclease SA [Burkholderia mallei ATCC 23344]
>gi|53725651|ref|YP_104021.1| hypothetical protein BMA2475 [Burkholderia mallei ATCC 23344]
>gi|53725653|ref|YP_104019.1| hypothetical protein BMA2473 [Burkholderia mallei ATCC 23344]
>gi|53725654|ref|YP_102426.1| hypothetical protein BMA0648 [Burkholderia mallei ATCC 23344]
>gi|53725665|ref|YP_104013.1| transporter, putative [Burkholderia mallei ATCC 23344]
>gi|53725672|ref|YP_102416.1| hypothetical protein BMA0634 [Burkholderia mallei ATCC 23344]
>gi|53725675|ref|YP_104009.1| hypothetical protein BMA2463 [Burkholderia mallei ATCC 23344]
>gi|53725680|ref|YP_104004.1| outer membrane lipoprotein, putatitve [Burkholderia mallei ATCC 23344]
>gi|53725682|ref|YP_102413.1| membrane protein, putative [Burkholderia mallei ATCC 23344]
>gi|53725687|ref|YP_104002.1| MotA/TolQ/ExbB proton channel family protein [Burkholderia mallei ATCC 23344]
>gi|53725688|ref|YP_104001.1| biopolymer transport protein, ExbD/TolR family [Burkholderia mallei ATCC 23344]
>gi|53725696|ref|YP_102405.1| membrane protein, putative [Burkholderia mallei ATCC 23344]
>gi|53725699|ref|YP_102402.1| methyl-accepting chemotaxis protein, putative [Burkholderia mallei ATCC 23344]
>gi|53725700|ref|YP_103996.1| membrane protein, putative [Burkholderia mallei ATCC 23344]
>gi|53725701|ref|YP_103995.1| hypothetical protein BMA2448 [Burkholderia mallei ATCC 23344]
>gi|53725707|ref|YP_102401.1| hypothetical protein BMA0615 [Burkholderia mallei ATCC 23344]
>gi|53725708|ref|YP_102400.1| membrane protein, putative [Burkholderia mallei ATCC 23344]
>gi|53725710|ref|YP_102398.1| sufD domain protein [Burkholderia mallei ATCC 23344]
>gi|53725715|ref|YP_103988.1| nitrate transporter, putative [Burkholderia mallei ATCC 23344]
>gi|53725727|ref|YP_102317.1| ribonuclease E [Burkholderia mallei ATCC 23344]
>gi|53725731|ref|YP_103977.1| hypothetical protein BMA2424 [Burkholderia mallei ATCC 23344]
>gi|53725734|ref|YP_103974.1| hypothetical protein BMA2421 [Burkholderia mallei ATCC 23344]
>gi|53725736|ref|YP_102315.1| molybdopterin-guanine dinucleotide biosynthesis protein [Burkholderia mallei ATCC 23344]
>gi|53725737|ref|YP_102314.1| molybdopterin biosynthesis moeA protein [Burkholderia mallei ATCC 23344]
>gi|53725738|ref|YP_102313.1| membrane protein, putative [Burkholderia mallei ATCC 23344]
>gi|53725742|ref|YP_102309.1| membrane protein, putative [Burkholderia mallei ATCC 23344]
>gi|53725752|ref|YP_103964.1| conserved hypothetical protein TIGR00044 [Burkholderia mallei ATCC 23344]
>gi|53725753|ref|YP_103963.1| pyrroline-5-carboxylate reductase [Burkholderia mallei ATCC 23344]
>gi|53725755|ref|YP_103961.1| phosphonates ABC transporter, ATP-binding protein [Burkholderia mallei ATCC 23344]
>gi|53725763|ref|YP_103404.1| membrane protein, putative [Burkholderia mallei ATCC 23344]
>gi|53725766|ref|YP_103887.1| co-chaperone GrpE [Burkholderia mallei ATCC 23344]
>gi|53725767|ref|YP_103886.1| hypothetical protein BMA2327 [Burkholderia mallei ATCC 23344]
>gi|53725787|ref|YP_103413.1| hypothetical protein BMA1807 [Burkholderia mallei ATCC 23344]
>gi|53725826|ref|YP_103435.1| preprotein translocase, SecG subunit [Burkholderia mallei ATCC 23344]
>gi|53725839|ref|YP_103441.1| hypothetical protein BMA1837 [Burkholderia mallei ATCC 23344]
>gi|53725854|ref|YP_103452.1| hypothetical protein BMA1849 [Burkholderia mallei ATCC 23344]
>gi|53725856|ref|YP_103454.1| hypothetical protein BMA1851 [Burkholderia mallei ATCC 23344]
>gi|53725858|ref|YP_103839.1| hypothetical protein BMA2276 [Burkholderia mallei ATCC 23344]
>gi|53725862|ref|YP_103835.1| exodeoxyribonuclease VII, large subunit [Burkholderia mallei ATCC 23344]
>gi|53725865|ref|YP_103831.1| chromate transport protein [Burkholderia mallei ATCC 23344]
>gi|53725867|ref|YP_103829.1| membrane protein, putative [Burkholderia mallei ATCC 23344]
>gi|53725868|ref|YP_103828.1| 2-dehydropantoate 2-reductase [Burkholderia mallei ATCC 23344]

>gi|53725869|ref|YP_103827.1| hypothetical protein BMA2263 [Burkholderia mallei ATCC 23344]
>gi|53725871|ref|YP_103526.1| membrane protein, putative [Burkholderia mallei ATCC 23344]
>gi|53725876|ref|YP_103531.1| benzoate transport protein [Burkholderia mallei ATCC 23344]
>gi|53725877|ref|YP_103532.1| hypothetical protein BMA1939 [Burkholderia mallei ATCC 23344]
>gi|53725879|ref|YP_103824.1| hypothetical protein BMA2260 [Burkholderia mallei ATCC 23344]
>gi|53725881|ref|YP_103822.1| citrate synthase family protein [Burkholderia mallei ATCC 23344]
>gi|53725884|ref|YP_103819.1| lipoprotein, putative [Burkholderia mallei ATCC 23344]
>gi|53725885|ref|YP_103817.1| hypothetical protein BMA2253 [Burkholderia mallei ATCC 23344]
>gi|53725900|ref|YP_103732.1| transcriptional regulator, Sir2 family [Burkholderia mallei ATCC 23344]
>gi|53725903|ref|YP_103542.1| hypothetical protein BMA1950 [Burkholderia mallei ATCC 23344]
>gi|53725915|ref|YP_103726.1| membrane protein, putative [Burkholderia mallei ATCC 23344]
>gi|53725916|ref|YP_103725.1| hypothetical protein BMA2157 [Burkholderia mallei ATCC 23344]
>gi|53725921|ref|YP_103554.1| copper resistance protein, putative [Burkholderia mallei ATCC 23344]
>gi|53725923|ref|YP_103724.1| hypothetical protein BMA2155 [Burkholderia mallei ATCC 23344]
>gi|53725925|ref|YP_103722.1| LysE family protein [Burkholderia mallei ATCC 23344]
>gi|53725928|ref|YP_103719.1| transglycosylase SLT domain protein [Burkholderia mallei ATCC 23344]
>gi|53725945|ref|YP_103565.1| glycosyl transferase family protein [Burkholderia mallei ATCC 23344]
>gi|53725947|ref|YP_103710.1| hypothetical protein BMA2140 [Burkholderia mallei ATCC 23344]
>gi|53725949|ref|YP_103708.1| hypothetical protein BMA2138 [Burkholderia mallei ATCC 23344]
>gi|53725954|ref|YP_103703.1| hypothetical protein BMA2133 [Burkholderia mallei ATCC 23344]
>gi|53725956|ref|YP_103701.1| 6-phosphogluconolactonase [Burkholderia mallei ATCC 23344]
>gi|53725970|ref|YP_103695.1| Smr domain protein [Burkholderia mallei ATCC 23344]
>gi|53725985|ref|YP_103685.1| septum site-determining protein MinC [Burkholderia mallei ATCC 23344]
>gi|53725987|ref|YP_103582.1| pyrimidine operon regulatory protein PyrR [Burkholderia mallei ATCC 23344]
>gi|53725990|ref|YP_103585.1| hypothetical protein BMA1998 [Burkholderia mallei ATCC 23344]
>gi|53725994|ref|YP_103590.1| hypothetical protein BMA2003 [Burkholderia mallei ATCC 23344]
>gi|53725996|ref|YP_103682.1| hypothetical protein BMA2110 [Burkholderia mallei ATCC 23344]
>gi|53725997|ref|YP_103681.1| voltage gated chloride channel family protein [Burkholderia mallei ATCC 23344]
>gi|53725999|ref|YP_103679.1| hypothetical protein BMA2107 [Burkholderia mallei ATCC 23344]
>gi|53726000|ref|YP_103678.1| metabolite:proton symporter family protein [Burkholderia mallei ATCC 23344]
>gi|53726006|ref|YP_103671.1| conserved hypothetical protein TIGR00255 [Burkholderia mallei ATCC 23344]
>gi|53726012|ref|YP_103593.1| hypothetical protein BMA2007 [Burkholderia mallei ATCC 23344]
>gi|53726013|ref|YP_103594.1| hypothetical protein BMA2008 [Burkholderia mallei ATCC 23344]
>gi|53726023|ref|YP_104110.1| hypothetical protein BMA2572 [Burkholderia mallei ATCC 23344]
>gi|53726025|ref|YP_104101.1| cell division protein FtsL, putative [Burkholderia mallei ATCC 23344]
>gi|53726028|ref|YP_104098.1| UDP-N-acetylmuramoyl-tripeptide--D-alanyl-D-alanine ligase [Burkholderia mallei ATCC 23344]
>gi|53726030|ref|YP_104096.1| UDP-N-acetylmuramoylalanine--D-glutamate ligase [Burkholderia mallei ATCC 23344]
>gi|53726032|ref|YP_103692.1| outer membrane lipoprotein carrier protein LolA [Burkholderia mallei ATCC 23344]
>gi|53726041|ref|YP_104627.1| hypothetical protein BMA3143 [Burkholderia mallei ATCC 23344]
>gi|53726042|ref|YP_104267.1| hypothetical protein BMA2749 [Burkholderia mallei ATCC 23344]
>gi|53726052|ref|YP_104084.1| hypothetical protein BMA2541 [Burkholderia mallei ATCC 23344]
>gi|53726057|ref|YP_103041.1| hypothetical protein BMA1383 [Burkholderia mallei ATCC 23344]
>gi|53726058|ref|YP_101870.1| hypothetical protein BMA0009 [Burkholderia mallei ATCC 23344]
>gi|53726060|ref|YP_102362.1| hypothetical protein BMA0570 [Burkholderia mallei ATCC 23344]
>gi|53726065|ref|YP_104077.1| type IV pilus prepilin peptidase PilD [Burkholderia mallei ATCC 23344]
>gi|53726066|ref|YP_104076.1| type IV pilus biogenesis protein PilC [Burkholderia mallei ATCC 23344]
>gi|53726072|ref|YP_102794.1| hypothetical protein BMA1104 [Burkholderia mallei ATCC 23344]
>gi|53726083|ref|YP_104242.1| hypothetical protein BMA2721 [Burkholderia mallei ATCC 23344]
>gi|53726085|ref|YP_104361.1| hypothetical protein BMA2850 [Burkholderia mallei ATCC 23344]
>gi|53726091|ref|YP_104059.1| cytochrome C assembly family protein [Burkholderia mallei ATCC 23344]
>gi|53726092|ref|YP_104058.1| hypothetical protein BMA2512 [Burkholderia mallei ATCC 23344]
>gi|53726093|ref|YP_104444.1| hypothetical protein BMA2939 [Burkholderia mallei ATCC 23344]
>gi|53726099|ref|YP_102827.1| hypothetical protein BMA1141.1 [Burkholderia mallei ATCC 23344]
>gi|53726103|ref|YP_104054.1| hypothetical protein BMA2508 [Burkholderia mallei ATCC 23344]
>gi|53726104|ref|YP_104053.1| outer membrane lipoprotein [Burkholderia mallei ATCC 23344]
>gi|53726107|ref|YP_104050.1| hypothetical protein BMA2504 [Burkholderia mallei ATCC 23344]
>gi|53726109|ref|YP_102920.1| hypothetical protein BMA1247.1 [Burkholderia mallei ATCC 23344]
>gi|53726112|ref|YP_103492.1| hypothetical protein BMA1894.2 [Burkholderia mallei ATCC 23344]
>gi|53726113|ref|YP_103491.1| hypothetical protein BMA1894.1 [Burkholderia mallei ATCC 23344]
>gi|53726114|ref|YP_103651.1| hypothetical protein BMA2071.1 [Burkholderia mallei ATCC 23344]
>gi|53726115|ref|YP_103652.1| hypothetical protein BMA2073 [Burkholderia mallei ATCC 23344]
>gi|53726116|ref|YP_103847.1| hypothetical protein BMA2284.1 [Burkholderia mallei ATCC 23344]
>gi|53726119|ref|YP_104047.1| acetyl-CoA carboxylase, biotin carboxyl carrier protein [Burkholderia mallei ATCC 23344]
>gi|53726121|ref|YP_104045.1| thioredoxin family protein, putative [Burkholderia mallei ATCC 23344]
>gi|53726125|ref|YP_104519.1| hypothetical protein BMA3016.1 [Burkholderia mallei ATCC 23344]
>gi|53726127|ref|YP_104644.1| hypothetical protein BMA3164.1 [Burkholderia mallei ATCC 23344]
>gi|53726128|ref|YP_104653.1| hypothetical protein BMA3173.1 [Burkholderia mallei ATCC 23344]
>gi|53726138|ref|YP_104037.1| hypothetical protein BMA2491 [Burkholderia mallei ATCC 23344]
>gi|53726140|ref|YP_103385.1| exodeoxyribonuclease V, gamma subunit [Burkholderia mallei ATCC 23344]
>gi|53726141|ref|YP_103515.1| hypothetical protein BMA1920.1 [Burkholderia mallei ATCC 23344]

>gi|53726144|ref|YP_104035.1| 2-dehydro-3-deoxygalactonokinase [Burkholderia mallei ATCC 23344]
>gi|53726146|ref|YP_104033.1| oxidoreductase, short-chain dehydrogenase/reductase family [Burkholderia mallei ATCC 23344]
>gi|53726149|ref|YP_104030.1| L-arabinose ABC transporter, permease protein [Burkholderia mallei ATCC 23344]
>gi|53726157|ref|YP_103955.1| alkylphosphonate utilization operon protein PhnG [Burkholderia mallei ATCC 23344]
>gi|53726159|ref|YP_103953.1| hypothetical protein BMA2398 [Burkholderia mallei ATCC 23344]
>gi|53726164|ref|YP_103948.1| hypothetical protein BMA2393 [Burkholderia mallei ATCC 23344]
>gi|53726165|ref|YP_103947.1| hypothetical protein BMA2392 [Burkholderia mallei ATCC 23344]
>gi|53726176|ref|YP_103936.1| hypothetical protein BMA2381 [Burkholderia mallei ATCC 23344]
>gi|53726179|ref|YP_103933.1| lipoprotein, putative [Burkholderia mallei ATCC 23344]
>gi|53726180|ref|YP_103932.1| hypothetical protein BMA2376 [Burkholderia mallei ATCC 23344]
>gi|53726183|ref|YP_103929.1| hypothetical protein BMA2373 [Burkholderia mallei ATCC 23344]
>gi|53726185|ref|YP_103927.1| hypothetical protein BMA2369 [Burkholderia mallei ATCC 23344]
>gi|53726188|ref|YP_103924.1| NAD(P) transhydrogenase, alpha-1 subunit [Burkholderia mallei ATCC 23344]
>gi|53726219|ref|YP_103892.1| DNA repair protein RecN [Burkholderia mallei ATCC 23344]
>gi|53726224|ref|YP_103811.1| hypothetical protein BMA2247 [Burkholderia mallei ATCC 23344]
>gi|53726241|ref|YP_103794.1| DNA repair protein RadC [Burkholderia mallei ATCC 23344]
>gi|53726245|ref|YP_103790.1| hypothetical protein BMA2226 [Burkholderia mallei ATCC 23344]
>gi|53726248|ref|YP_103786.1| phenazine biosynthesis protein phzF, putative [Burkholderia mallei ATCC 23344]
>gi|53726250|ref|YP_103784.1| chromate transport protein, putative [Burkholderia mallei ATCC 23344]
>gi|53726256|ref|YP_103778.1| membrane protein, putative [Burkholderia mallei ATCC 23344]
>gi|53726259|ref|YP_103775.1| hypothetical protein BMA2210 [Burkholderia mallei ATCC 23344]
>gi|53726269|ref|YP_103765.1| hypothetical protein BMA2200 [Burkholderia mallei ATCC 23344]
>gi|53726274|ref|YP_103758.1| membrane protein, putative [Burkholderia mallei ATCC 23344]
>gi|53726279|ref|YP_103754.1| 3-deoxy-D-manno-octulosonic-acid transferase [Burkholderia mallei ATCC 23344]
>gi|53726281|ref|YP_103752.1| urease accessory protein UreF [Burkholderia mallei ATCC 23344]
>gi|53726282|ref|YP_103751.1| urease accessory protein UreE [Burkholderia mallei ATCC 23344]
>gi|53726290|ref|YP_103743.1| branched-chain amino acid ABC transporter, permease protein, putative [Burkholderia mallei ATCC 23344]
>gi|53726360|ref|YP_102763.1| RNA pseudouridylate synthase family protein [Burkholderia mallei ATCC 23344]
>gi|53726362|ref|YP_102348.1| excinuclease ABC, C subunit [Burkholderia mallei ATCC 23344]
>gi|53726366|ref|YP_104542.1| hypothetical protein BMA3045 [Burkholderia mallei ATCC 23344]
>gi|53715878|ref|YP_106611.1| esterase EstC [Burkholderia mallei ATCC 23344]
>gi|53715879|ref|YP_106610.1| hypothetical protein BMAA2103 [Burkholderia mallei ATCC 23344]
>gi|53715888|ref|YP_106598.1| membrane protein, putative [Burkholderia mallei ATCC 23344]
>gi|53715889|ref|YP_106597.1| acyl transferase domain protein [Burkholderia mallei ATCC 23344]
>gi|53715890|ref|YP_106596.1| polyketide synthase, putative [Burkholderia mallei ATCC 23344]
>gi|53715903|ref|YP_106580.1| DNA-binding response regulator, LuxR family [Burkholderia mallei ATCC 23344]
>gi|53715905|ref|YP_106578.1| hypothetical protein BMAA2067 [Burkholderia mallei ATCC 23344]
>gi|53715909|ref|YP_106573.1| amidase family protein [Burkholderia mallei ATCC 23344]
>gi|53715913|ref|YP_106568.1| lipoprotein, putative [Burkholderia mallei ATCC 23344]
>gi|53715915|ref|YP_106566.1| hypothetical protein BMAA2054 [Burkholderia mallei ATCC 23344]
>gi|53715916|ref|YP_106565.1| hypothetical protein BMAA2053 [Burkholderia mallei ATCC 23344]
>gi|53715917|ref|YP_106564.1| polysaccharide deacetylase family protein [Burkholderia mallei ATCC 23344]
>gi|53715918|ref|YP_106561.1| membrane protein, putative [Burkholderia mallei ATCC 23344]
>gi|53715919|ref|YP_106560.1| iron-sulfur cluster-binding protein [Burkholderia mallei ATCC 23344]
>gi|53715920|ref|YP_106558.1| major facilitator family transporter [Burkholderia mallei ATCC 23344]
>gi|53715924|ref|YP_106554.1| hypothetical protein BMAA2041 [Burkholderia mallei ATCC 23344]
>gi|53715925|ref|YP_106553.1| transcriptional regulator, LuxR family [Burkholderia mallei ATCC 23344]
>gi|53715926|ref|YP_106552.1| hypothetical protein BMAA2038 [Burkholderia mallei ATCC 23344]
>gi|53715930|ref|YP_106547.1| hypothetical protein BMAA2032 [Burkholderia mallei ATCC 23344]
>gi|53715931|ref|YP_106546.1| sulfate permease family protein [Burkholderia mallei ATCC 23344]
>gi|53715932|ref|YP_106544.1| hypothetical protein BMAA2028 [Burkholderia mallei ATCC 23344]
>gi|53715933|ref|YP_106543.1| hypothetical protein BMAA2027 [Burkholderia mallei ATCC 23344]
>gi|53715935|ref|YP_106540.1| C4-type zinc finger protein, DksA/TraR family [Burkholderia mallei ATCC 23344]
>gi|53715939|ref|YP_106464.1| hypothetical protein BMAA1939 [Burkholderia mallei ATCC 23344]
>gi|53715940|ref|YP_106463.1| hypothetical protein BMAA1938 [Burkholderia mallei ATCC 23344]
>gi|53715942|ref|YP_106461.1| hypothetical protein BMAA1935 [Burkholderia mallei ATCC 23344]
>gi|53715950|ref|YP_104910.1| hypothetical protein BMAA0057 [Burkholderia mallei ATCC 23344]
>gi|53715956|ref|YP_106453.1| hypothetical protein BMAA1925 [Burkholderia mallei ATCC 23344]
>gi|53715958|ref|YP_104908.1| hypothetical protein BMAA0054 [Burkholderia mallei ATCC 23344]
>gi|53715959|ref|YP_104907.1| membrane protein, putative [Burkholderia mallei ATCC 23344]
>gi|53715977|ref|YP_106445.1| OmpA domain protein [Burkholderia mallei ATCC 23344]
>gi|53715983|ref|YP_104891.1| hypothetical protein BMAA0034 [Burkholderia mallei ATCC 23344]
>gi|53715984|ref|YP_104890.1| hypothetical protein BMAA0033 [Burkholderia mallei ATCC 23344]
>gi|53715996|ref|YP_106441.1| hypothetical protein BMAA1902 [Burkholderia mallei ATCC 23344]
>gi|53715998|ref|YP_106439.1| pentapeptide repeat family protein [Burkholderia mallei ATCC 23344]
>gi|53716006|ref|YP_104879.1| transcriptional regulator, PadR family [Burkholderia mallei ATCC 23344]
>gi|53716013|ref|YP_106431.1| hypothetical protein BMAA1891 [Burkholderia mallei ATCC 23344]
>gi|53716017|ref|YP_106428.1| hypothetical protein BMAA1887 [Burkholderia mallei ATCC 23344]
>gi|53716019|ref|YP_104872.1| hypothetical protein BMAA0015 [Burkholderia mallei ATCC 23344]
>gi|53716044|ref|YP_106416.1| lipoprotein, putative [Burkholderia mallei ATCC 23344]

>gi|53716045|ref|YP_106415.1| hypothetical protein BMAA1873 [Burkholderia mallei ATCC 23344]
>gi|53716047|ref|YP_106413.1| propionate catabolism operon regulatory protein [Burkholderia mallei ATCC 23344]
>gi|53716055|ref|YP_106537.1| hypothetical protein BMAA2021 [Burkholderia mallei ATCC 23344]
>gi|53716063|ref|YP_106529.1| 2-oxoisovalerate dehydrogenase, E2 component, dihydrolipamide acetyltransferase [Burkholderia mallei ATCC 23344]
>gi|53716067|ref|YP_106339.1| hypothetical protein BMAA1782 [Burkholderia mallei ATCC 23344]
>gi|53716069|ref|YP_106527.1| hypothetical protein BMAA2008 [Burkholderia mallei ATCC 23344]
>gi|53716074|ref|YP_106521.1| hypothetical protein BMAA2002 [Burkholderia mallei ATCC 23344]
>gi|53716084|ref|YP_106518.1| hypothetical protein BMAA1999 [Burkholderia mallei ATCC 23344]
>gi|53716087|ref|YP_106515.1| hypothetical protein BMAA1995 [Burkholderia mallei ATCC 23344]
>gi|53716088|ref|YP_106514.1| ABC transporter, ATP-binding protein, putative [Burkholderia mallei ATCC 23344]
>gi|53716095|ref|YP_106325.1| membrane protein, putative [Burkholderia mallei ATCC 23344]
>gi|53716097|ref|YP_106511.1| oxidoreductase, short chain dehydrogenase/reductase family [Burkholderia mallei ATCC 23344]
>gi|53716099|ref|YP_106509.1| heptosyltransferase family protein [Burkholderia mallei ATCC 23344]
>gi|53716106|ref|YP_106502.1| hypothetical protein BMAA1980 [Burkholderia mallei ATCC 23344]
>gi|53716108|ref|YP_106324.1| oxidoreductase, 2OG-Fe(II) oxygenase family [Burkholderia mallei ATCC 23344]
>gi|53716109|ref|YP_106323.1| CDP-alcohol phosphatidyltransferase family protein [Burkholderia mallei ATCC 23344]
>gi|53716111|ref|YP_106321.1| hypothetical protein BMAA1762 [Burkholderia mallei ATCC 23344]
>gi|53716118|ref|YP_106496.1| hypothetical protein BMAA1973 [Burkholderia mallei ATCC 23344]
>gi|53716119|ref|YP_106495.1| hypothetical protein BMAA1972 [Burkholderia mallei ATCC 23344]
>gi|53716128|ref|YP_106308.1| succinate dehydrogenase, cytochrome b556 subunit [Burkholderia mallei ATCC 23344]
>gi|53716130|ref|YP_106492.1| hypothetical protein BMAA1969 [Burkholderia mallei ATCC 23344]
>gi|53716132|ref|YP_106490.1| hypothetical protein BMAA1967 [Burkholderia mallei ATCC 23344]
>gi|53716134|ref|YP_106488.1| hypothetical protein BMAA1965 [Burkholderia mallei ATCC 23344]
>gi|53716141|ref|YP_106304.1| hypothetical protein BMAA1745 [Burkholderia mallei ATCC 23344]
>gi|53716143|ref|YP_106302.1| transcriptional regulator, AraC family [Burkholderia mallei ATCC 23344]
>gi|53716146|ref|YP_106482.1| hypothetical protein BMAA1959 [Burkholderia mallei ATCC 23344]
>gi|53716149|ref|YP_106479.1| rarD protein [Burkholderia mallei ATCC 23344]
>gi|53716150|ref|YP_106478.1| hypothetical protein BMAA1955 [Burkholderia mallei ATCC 23344]
>gi|53716159|ref|YP_106474.1| citrate lyase, beta subunit, putative [Burkholderia mallei ATCC 23344]
>gi|53716163|ref|YP_106469.1| hypothetical protein BMAA1945 [Burkholderia mallei ATCC 23344]
>gi|53716169|ref|YP_106291.1| entericidin [Burkholderia mallei ATCC 23344]
>gi|53716173|ref|YP_106287.1| hypothetical protein BMAA1724 [Burkholderia mallei ATCC 23344]
>gi|53716181|ref|YP_106279.1| sporulation-related repeat protein [Burkholderia mallei ATCC 23344]
>gi|53716182|ref|YP_106278.1| CvpA family protein [Burkholderia mallei ATCC 23344]
>gi|53716191|ref|YP_106404.1| transcriptional regulator, LysR family [Burkholderia mallei ATCC 23344]
>gi|53716195|ref|YP_106270.1| polysaccharide biosynthesis family protein [Burkholderia mallei ATCC 23344]
>gi|53716197|ref|YP_106197.1| hypothetical protein BMAA1619 [Burkholderia mallei ATCC 23344]
>gi|53716199|ref|YP_106194.1| hypothetical protein BMAA1616 [Burkholderia mallei ATCC 23344]
>gi|53716202|ref|YP_106191.1| type II/III secretion system family protein [Burkholderia mallei ATCC 23344]
>gi|53716204|ref|YP_106189.1| pilL domain protein [Burkholderia mallei ATCC 23344]
>gi|53716205|ref|YP_106188.1| type IV pilus biogenesis protein PilN, putative [Burkholderia mallei ATCC 23344]
>gi|53716209|ref|YP_106401.1| hypothetical protein BMAA1854 [Burkholderia mallei ATCC 23344]
>gi|53716211|ref|YP_106399.1| hypothetical protein BMAA1852 [Burkholderia mallei ATCC 23344]
>gi|53716212|ref|YP_106398.1| copper-translocating P-type ATPase [Burkholderia mallei ATCC 23344]
>gi|53716213|ref|YP_106397.1| hypothetical protein BMAA1849 [Burkholderia mallei ATCC 23344]
>gi|53716222|ref|YP_106394.1| hypothetical protein BMAA1845 [Burkholderia mallei ATCC 23344]
>gi|53716224|ref|YP_106392.1| hypothetical protein BMAA1842 [Burkholderia mallei ATCC 23344]
>gi|53716228|ref|YP_106177.1| hypothetical protein BMAA1594 [Burkholderia mallei ATCC 23344]
>gi|53716229|ref|YP_106175.1| hipB domain protein [Burkholderia mallei ATCC 23344]
>gi|53716233|ref|YP_106172.1| endo-1,4-D-glucanase [Burkholderia mallei ATCC 23344]
>gi|53716234|ref|YP_106171.1| cellulose synthase operon protein C [Burkholderia mallei ATCC 23344]
>gi|53716242|ref|YP_106381.1| hemin ABC transporter, ATP-binding protein [Burkholderia mallei ATCC 23344]
>gi|53716243|ref|YP_106170.1| hypothetical protein BMAA1587 [Burkholderia mallei ATCC 23344]
>gi|53716244|ref|YP_106169.1| cellulose biosynthesis protein, putative [Burkholderia mallei ATCC 23344]
>gi|53716245|ref|YP_106168.1| glycosyl transferase, group 2 family protein [Burkholderia mallei ATCC 23344]
>gi|53716246|ref|YP_106167.1| hypothetical protein BMAA1584 [Burkholderia mallei ATCC 23344]
>gi|53716250|ref|YP_106380.1| hemin ABC transporter, permease protein [Burkholderia mallei ATCC 23344]
>gi|53716264|ref|YP_106373.1| ABC transporter, permease protein, putative [Burkholderia mallei ATCC 23344]
>gi|53716267|ref|YP_106370.1| ABC transporter, permease protein, putative [Burkholderia mallei ATCC 23344]
>gi|53716268|ref|YP_106369.1| conserved hypothetical protein TIGR00725 [Burkholderia mallei ATCC 23344]
>gi|53716272|ref|YP_106155.1| hypothetical protein BMAA1571 [Burkholderia mallei ATCC 23344]
>gi|53716274|ref|YP_106153.1| LysE family protein [Burkholderia mallei ATCC 23344]
>gi|53716276|ref|YP_106151.1| hypothetical protein BMAA1567 [Burkholderia mallei ATCC 23344]
>gi|53716281|ref|YP_106364.1| sugar ABC transporter, permease protein [Burkholderia mallei ATCC 23344]
>gi|53716285|ref|YP_106360.1| dihydroxyacetone kinase family protein [Burkholderia mallei ATCC 23344]
>gi|53716286|ref|YP_106359.1| aminoglycoside 6'-N-acetyltransferase Iz [Burkholderia mallei ATCC 23344]
>gi|53716287|ref|YP_106358.1| hypothetical protein BMAA1805 [Burkholderia mallei ATCC 23344]
>gi|53716291|ref|YP_106354.1| TonB protein, putative [Burkholderia mallei ATCC 23344]
>gi|53716300|ref|YP_106145.1| transcriptional regulator, MarR family [Burkholderia mallei ATCC 23344]

>gi|53716308|ref|YP_106142.1| transcriptional regulator, putative [Burkholderia mallei ATCC 23344]
>gi|53716309|ref|YP_106141.1| hypothetical protein BMAA1553 [Burkholderia mallei ATCC 23344]
>gi|53716310|ref|YP_106140.1| hypothetical protein BMAA1552 [Burkholderia mallei ATCC 23344]
>gi|53716311|ref|YP_106139.1| oxygen-regulated invasion protein OrgA [Burkholderia mallei ATCC 23344]
>gi|53716312|ref|YP_106138.1| type III secretion system BasJ [Burkholderia mallei ATCC 23344]
>gi|53716315|ref|YP_106341.1| hypothetical protein BMAA1784 [Burkholderia mallei ATCC 23344]
>gi|53716316|ref|YP_106340.1| nitroreductase family protein [Burkholderia mallei ATCC 23344]
>gi|53716319|ref|YP_106265.1| DNA-binding protein BpH2 [Burkholderia mallei ATCC 23344]
>gi|53716320|ref|YP_106266.1| acyltransferase family protein [Burkholderia mallei ATCC 23344]
>gi|53716321|ref|YP_106264.1| hypothetical protein BMAA1699 [Burkholderia mallei ATCC 23344]
>gi|53716323|ref|YP_106135.1| type III secretion system protein BsaM [Burkholderia mallei ATCC 23344]
>gi|53716324|ref|YP_106134.1| type III secretion system transcriptional regulator BsaN [Burkholderia mallei ATCC 23344]
>gi|53716326|ref|YP_106132.1| type III secretion system protein BsaP [Burkholderia mallei ATCC 23344]
>gi|53716329|ref|YP_106262.1| ribonuclease BN, putative [Burkholderia mallei ATCC 23344]
>gi|53716331|ref|YP_106260.1| lipoprotein, putative [Burkholderia mallei ATCC 23344]
>gi|53716332|ref|YP_106259.1| hypothetical protein BMAA1694 [Burkholderia mallei ATCC 23344]
>gi|53716334|ref|YP_106257.1| acetyltransferase, GNAT family [Burkholderia mallei ATCC 23344]
>gi|53716338|ref|YP_106253.1| amino acid ABC transporter, ATP-binding/permease protein [Burkholderia mallei ATCC 23344]
>gi|53716339|ref|YP_106252.1| hypothetical protein BMAA1687 [Burkholderia mallei ATCC 23344]
>gi|53716341|ref|YP_106250.1| hypothetical protein BMAA1685 [Burkholderia mallei ATCC 23344]
>gi|53716345|ref|YP_106248.1| formate dehydrogenase, gamma subunit [Burkholderia mallei ATCC 23344]
>gi|53716352|ref|YP_105191.1| hypothetical protein BMAA0395 [Burkholderia mallei ATCC 23344]
>gi|53716354|ref|YP_105189.1| FHA domain protein [Burkholderia mallei ATCC 23344]
>gi|53716357|ref|YP_105186.1| hypothetical protein BMAA0389 [Burkholderia mallei ATCC 23344]
>gi|53716364|ref|YP_106241.1| transporter, putative [Burkholderia mallei ATCC 23344]
>gi|53716365|ref|YP_106240.1| hypothetical protein BMAA1670 [Burkholderia mallei ATCC 23344]
>gi|53716372|ref|YP_105177.1| hypothetical protein BMAA0377 [Burkholderia mallei ATCC 23344]
>gi|53716374|ref|YP_105175.1| transcriptional regulator, LysR family [Burkholderia mallei ATCC 23344]
>gi|53716376|ref|YP_106236.1| hypothetical protein BMAA1666 [Burkholderia mallei ATCC 23344]
>gi|53716378|ref|YP_106233.1| hypothetical protein BMAA1663 [Burkholderia mallei ATCC 23344]
>gi|53716382|ref|YP_106228.1| drug resistance transporter, EmrB/QacA family [Burkholderia mallei ATCC 23344]
>gi|53716384|ref|YP_105172.1| hypothetical protein BMAA0372 [Burkholderia mallei ATCC 23344]
>gi|53716387|ref|YP_105169.1| indole-3-acetamide hydrolase-related protein [Burkholderia mallei ATCC 23344]
>gi|53716391|ref|YP_106225.1| citrate lyase, beta subunit, putative [Burkholderia mallei ATCC 23344]
>gi|53716397|ref|YP_105165.1| hypothetical protein BMAA0364 [Burkholderia mallei ATCC 23344]
>gi|53716398|ref|YP_105164.1| sigma-54 dependent DNA-binding transcriptional regulator [Burkholderia mallei ATCC 23344]
>gi|53716407|ref|YP_106214.1| hypothetical protein BMAA1641 [Burkholderia mallei ATCC 23344]
>gi|53716409|ref|YP_106212.1| type III secretion inner membrane protein SctT, putative [Burkholderia mallei ATCC 23344]
>gi|53716416|ref|YP_106210.1| type III secretion inner membrane protein SctL [Burkholderia mallei ATCC 23344]
>gi|53716418|ref|YP_106209.1| hypothetical protein BMAA1635 [Burkholderia mallei ATCC 23344]
>gi|53716419|ref|YP_106208.1| HrpB2-like protein [Burkholderia mallei ATCC 23344]
>gi|53716422|ref|YP_105154.1| hypothetical protein BMAA0349 [Burkholderia mallei ATCC 23344]
>gi|53716424|ref|YP_105152.1| hypothetical protein BMAA0347 [Burkholderia mallei ATCC 23344]
>gi|53716425|ref|YP_105151.1| lipoprotein, putative [Burkholderia mallei ATCC 23344]
>gi|53716432|ref|YP_106204.1| type III secretion inner membrane protein SctQ [Burkholderia mallei ATCC 23344]
>gi|53716434|ref|YP_106202.1| type III secretion inner membrane protein SctS [Burkholderia mallei ATCC 23344]
>gi|53716435|ref|YP_106201.1| hypothetical protein BMAA1626 [Burkholderia mallei ATCC 23344]
>gi|53716436|ref|YP_105144.1| GatB/Yqey family protein [Burkholderia mallei ATCC 23344]
>gi|53716439|ref|YP_105141.1| O-sialoglycoprotein endopeptidase [Burkholderia mallei ATCC 23344]
>gi|53716440|ref|YP_105140.1| hypothetical protein BMAA0333 [Burkholderia mallei ATCC 23344]
>gi|53716445|ref|YP_105135.1| exonuclease VII, small subunit [Burkholderia mallei ATCC 23344]
>gi|53716447|ref|YP_105069.1| hypothetical protein BMAA0249 [Burkholderia mallei ATCC 23344]
>gi|53716448|ref|YP_105068.1| hypothetical protein BMAA0248 [Burkholderia mallei ATCC 23344]
>gi|53716454|ref|YP_106128.1| BsaU protein [Burkholderia mallei ATCC 23344]
>gi|53716455|ref|YP_106127.1| type III secretion system protein BsaV [Burkholderia mallei ATCC 23344]
>gi|53716463|ref|YP_106125.1| type III secretion system protein BsaX [Burkholderia mallei ATCC 23344]
>gi|53716467|ref|YP_106121.1| BipB protein [Burkholderia mallei ATCC 23344]
>gi|53716468|ref|YP_106120.1| type III secretion target BipC [Burkholderia mallei ATCC 23344]
>gi|53716476|ref|YP_106119.1| DNA-binding protein BprA [Burkholderia mallei ATCC 23344]
>gi|53716478|ref|YP_106117.1| BapA protein [Burkholderia mallei ATCC 23344]
>gi|53716479|ref|YP_106116.1| BapB protein [Burkholderia mallei ATCC 23344]
>gi|53716480|ref|YP_106115.1| BapC protein [Burkholderia mallei ATCC 23344]
>gi|53716482|ref|YP_106113.1| hypothetical protein BMAA1522 [Burkholderia mallei ATCC 23344]
>gi|53716487|ref|YP_105049.1| voltage-gated chloride channel [Burkholderia mallei ATCC 23344]
>gi|53716490|ref|YP_105045.1| CheR methyltransferase, SAM binding/TPR domain protein [Burkholderia mallei ATCC 23344]
>gi|53716502|ref|YP_105037.1| Na+/H+ antiporter [Burkholderia mallei ATCC 23344]
>gi|53716503|ref|YP_105036.1| hypothetical protein BMAA0209 [Burkholderia mallei ATCC 23344]
>gi|53716504|ref|YP_105230.1| hypothetical protein BMAA0456 [Burkholderia mallei ATCC 23344]
>gi|53716509|ref|YP_105033.1| ferredoxin reductase [Burkholderia mallei ATCC 23344]
>gi|53716513|ref|YP_105029.1| transcriptional regulator, AraC family [Burkholderia mallei ATCC 23344]

>gi|53716515|ref|YP_105027.1| muconate cycloisomerase [Burkholderia mallei ATCC 23344]
>gi|53716518|ref|YP_105024.1| ubiquinol oxidase, subunit IV [Burkholderia mallei ATCC 23344]
>gi|53716522|ref|YP_105020.1| hypothetical protein BMAA0193 [Burkholderia mallei ATCC 23344]
>gi|53716530|ref|YP_105012.1| oxidoreductase, short chain dehydrogenase/reductase family [Burkholderia mallei ATCC 23344]
>gi|53716534|ref|YP_105008.1| membrane protein, putative [Burkholderia mallei ATCC 23344]
>gi|53716542|ref|YP_104931.1| membrane protein, putative [Burkholderia mallei ATCC 23344]
>gi|53716549|ref|YP_105223.1| Rhs element Vgr protein [Burkholderia mallei ATCC 23344]
>gi|53716559|ref|YP_104920.1| hypothetical protein BMAA0072 [Burkholderia mallei ATCC 23344]
>gi|53716568|ref|YP_105215.1| lipase chaperone [Burkholderia mallei ATCC 23344]
>gi|53716571|ref|YP_105615.1| serine O-acetyltransferase, putative [Burkholderia mallei ATCC 23344]
>gi|53716574|ref|YP_105618.1| cysteine desulfurase, putative [Burkholderia mallei ATCC 23344]
>gi|53716576|ref|YP_105620.1| hypothetical protein BMAA0935 [Burkholderia mallei ATCC 23344]
>gi|53716578|ref|YP_105213.1| hypothetical protein BMAA0426 [Burkholderia mallei ATCC 23344]
>gi|53716585|ref|YP_105206.1| hypothetical protein BMAA0419 [Burkholderia mallei ATCC 23344]
>gi|53716586|ref|YP_105622.1| YeeE/YedE family protein [Burkholderia mallei ATCC 23344]
>gi|53716587|ref|YP_105623.1| membrane protein, putative [Burkholderia mallei ATCC 23344]
>gi|53716591|ref|YP_105628.1| di-haem cytochrome c peroxidase family protein [Burkholderia mallei ATCC 23344]
>gi|53716594|ref|YP_105631.1| major facilitator family transporter [Burkholderia mallei ATCC 23344]
>gi|53716596|ref|YP_105633.1| major facilitator family transporter [Burkholderia mallei ATCC 23344]
>gi|53716608|ref|YP_105638.1| hypothetical protein BMAA0955 [Burkholderia mallei ATCC 23344]
>gi|53716612|ref|YP_105642.1| membrane protein, putative [Burkholderia mallei ATCC 23344]
>gi|53716617|ref|YP_105129.1| hypothetical protein BMAA0321 [Burkholderia mallei ATCC 23344]
>gi|53716621|ref|YP_105644.1| hypothetical protein BMAA0964 [Burkholderia mallei ATCC 23344]
>gi|53716628|ref|YP_105125.1| membrane protein, putative [Burkholderia mallei ATCC 23344]
>gi|53716632|ref|YP_105651.1| asparaginase family protein [Burkholderia mallei ATCC 23344]
>gi|53716636|ref|YP_105655.1| dipeptide transport protein, putative [Burkholderia mallei ATCC 23344]
>gi|53716639|ref|YP_105120.1| hypothetical protein BMAA0308 [Burkholderia mallei ATCC 23344]
>gi|53716646|ref|YP_105113.1| polyhydroxybutyrate depolymerase domain protein [Burkholderia mallei ATCC 23344]
>gi|53716647|ref|YP_105112.1| PAP2 family protein [Burkholderia mallei ATCC 23344]
>gi|53716649|ref|YP_105110.1| molybdenum ABC transporter, permease protein [Burkholderia mallei ATCC 23344]
>gi|53716651|ref|YP_105108.1| hypothetical protein BMAA0296 [Burkholderia mallei ATCC 23344]
>gi|53716654|ref|YP_105658.1| transcriptional regulator, LysR family [Burkholderia mallei ATCC 23344]
>gi|53716655|ref|YP_105659.1| hypothetical protein BMAA0983 [Burkholderia mallei ATCC 23344]
>gi|53716657|ref|YP_105729.1| hypothetical protein BMAA1064 [Burkholderia mallei ATCC 23344]
>gi|53716658|ref|YP_105107.1| hypothetical protein BMAA0295 [Burkholderia mallei ATCC 23344]
>gi|53716660|ref|YP_105105.1| hypothetical protein BMAA0293 [Burkholderia mallei ATCC 23344]
>gi|53716662|ref|YP_105103.1| transcriptional regulator, LysR family [Burkholderia mallei ATCC 23344]
>gi|53716664|ref|YP_105101.1| sensor histidine kinase [Burkholderia mallei ATCC 23344]
>gi|53716666|ref|YP_105099.1| thiamine-phosphate pyrophosphorylase ThiE, putative [Burkholderia mallei ATCC 23344]
>gi|53716669|ref|YP_105732.1| hypothetical protein BMAA1068 [Burkholderia mallei ATCC 23344]
>gi|53716670|ref|YP_105733.1| transcriptional regulator, DeoR family [Burkholderia mallei ATCC 23344]
>gi|53716671|ref|YP_105734.1| transporter, putative [Burkholderia mallei ATCC 23344]
>gi|53716673|ref|YP_105736.1| hypothetical protein BMAA1072 [Burkholderia mallei ATCC 23344]
>gi|53716687|ref|YP_105743.1| hypothetical protein BMAA1080 [Burkholderia mallei ATCC 23344]
>gi|53716694|ref|YP_105744.1| D-alanyl-D-alanine carboxypeptidase family protein [Burkholderia mallei ATCC 23344]
>gi|53716696|ref|YP_105746.1| hypothetical protein BMAA1083 [Burkholderia mallei ATCC 23344]
>gi|53716700|ref|YP_105750.1| nitrate transporter, putative [Burkholderia mallei ATCC 23344]
>gi|53716706|ref|YP_105751.1| hypothetical protein BMAA1088 [Burkholderia mallei ATCC 23344]
>gi|53716707|ref|YP_105752.1| uroporphyrin-III C-methyltransferase [Burkholderia mallei ATCC 23344]
>gi|53716711|ref|YP_105756.1| hypothetical protein BMAA1093 [Burkholderia mallei ATCC 23344]
>gi|53716712|ref|YP_105758.1| hypothetical protein BMAA1095 [Burkholderia mallei ATCC 23344]
>gi|53716716|ref|YP_105077.1| hypothetical protein BMAA0258 [Burkholderia mallei ATCC 23344]
>gi|53716717|ref|YP_105076.1| hypothetical protein BMAA0256 [Burkholderia mallei ATCC 23344]
>gi|53716719|ref|YP_105074.1| hypothetical protein BMAA0254 [Burkholderia mallei ATCC 23344]
>gi|53716722|ref|YP_105760.1| hypothetical protein BMAA1097 [Burkholderia mallei ATCC 23344]
>gi|53716724|ref|YP_105762.1| N-carbamyl-L-amino acid amidohydrolase [Burkholderia mallei ATCC 23344]
>gi|53716727|ref|YP_105765.1| hypothetical protein BMAA1102 [Burkholderia mallei ATCC 23344]
>gi|53716730|ref|YP_105070.1| major facilitator family transporter [Burkholderia mallei ATCC 23344]
>gi|53716736|ref|YP_105767.1| oxidoreductase, short chain dehydrogenase/reductase family [Burkholderia mallei ATCC 23344]
>gi|53716744|ref|YP_104990.1| sulfotransferase domain protein [Burkholderia mallei ATCC 23344]
>gi|53716747|ref|YP_105772.1| hypothetical protein BMAA1113 [Burkholderia mallei ATCC 23344]
>gi|53716749|ref|YP_105774.1| hypothetical protein BMAA1116 [Burkholderia mallei ATCC 23344]
>gi|53716751|ref|YP_105777.1| multidrug resistance protein [Burkholderia mallei ATCC 23344]
>gi|53716753|ref|YP_104988.1| methyl-accepting chemotaxis protein [Burkholderia mallei ATCC 23344]
>gi|53716754|ref|YP_104987.1| hypothetical protein BMAA0151 [Burkholderia mallei ATCC 23344]
>gi|53716755|ref|YP_104985.1| membrane protein [Burkholderia mallei ATCC 23344]
>gi|53716758|ref|YP_104982.1| hypothetical protein BMAA0145 [Burkholderia mallei ATCC 23344]
>gi|53716761|ref|YP_104979.1| transcriptional regulator, TetR family [Burkholderia mallei ATCC 23344]
>gi|53716765|ref|YP_105782.1| MOSC domain protein [Burkholderia mallei ATCC 23344]
>gi|53716770|ref|YP_104977.1| RND efflux system, cytoplasmic membrane extrusion protein [Burkholderia mallei ATCC 23344]

>gi|53716777|ref|YP_105789.1| transcriptional regulator, AraC family [Burkholderia mallei ATCC 23344]
>gi|53716782|ref|YP_104972.1| hypothetical protein BMAA0134 [Burkholderia mallei ATCC 23344]
>gi|53716785|ref|YP_104969.1| ATP synthase F1, gamma subunit, putative [Burkholderia mallei ATCC 23344]
>gi|53716786|ref|YP_104968.1| ATP synthase F1, alpha subunit [Burkholderia mallei ATCC 23344]
>gi|53716787|ref|YP_104967.1| ATP synthase F0, B subunit [Burkholderia mallei ATCC 23344]
>gi|53716791|ref|YP_105797.1| hypothetical protein BMAA1142 [Burkholderia mallei ATCC 23344]
>gi|53716793|ref|YP_105865.1| histidine kinase [Burkholderia mallei ATCC 23344]
>gi|53716794|ref|YP_105866.1| hypothetical protein BMAA1229 [Burkholderia mallei ATCC 23344]
>gi|53716795|ref|YP_105867.1| transporter, putative [Burkholderia mallei ATCC 23344]
>gi|53716797|ref|YP_104964.1| hypothetical protein BMAA0126 [Burkholderia mallei ATCC 23344]
>gi|53716798|ref|YP_104961.1| ATP synthase F1, beta subunit [Burkholderia mallei ATCC 23344]
>gi|53716802|ref|YP_104957.1| hypothetical protein BMAA0119 [Burkholderia mallei ATCC 23344]
>gi|53716803|ref|YP_105868.1| hypothetical protein BMAA1234 [Burkholderia mallei ATCC 23344]
>gi|53716808|ref|YP_104956.1| 6-phosphofructokinase [Burkholderia mallei ATCC 23344]
>gi|53716810|ref|YP_104953.1| thymidine phosphorylase [Burkholderia mallei ATCC 23344]
>gi|53716811|ref|YP_104952.1| hypothetical protein BMAA0111 [Burkholderia mallei ATCC 23344]
>gi|53716812|ref|YP_104951.1| deoxyribose-phosphate aldolase [Burkholderia mallei ATCC 23344]
>gi|53716819|ref|YP_105878.1| oxidoreductase, putative [Burkholderia mallei ATCC 23344]
>gi|53716821|ref|YP_104949.1| hypothetical protein BMAA0108 [Burkholderia mallei ATCC 23344]
>gi|53716822|ref|YP_104948.1| glutathione S-transferase [Burkholderia mallei ATCC 23344]
>gi|53716825|ref|YP_104944.1| hypothetical protein BMAA0103 [Burkholderia mallei ATCC 23344]
>gi|53716828|ref|YP_105880.1| serine metalloprotease MrpA [Burkholderia mallei ATCC 23344]
>gi|53716836|ref|YP_104939.1| hypothetical protein BMAA0098 [Burkholderia mallei ATCC 23344]
>gi|53716840|ref|YP_105887.1| hypothetical protein BMAA1257 [Burkholderia mallei ATCC 23344]
>gi|53716845|ref|YP_105892.1| serine protease, subtilase family [Burkholderia mallei ATCC 23344]
>gi|53716846|ref|YP_105893.1| hypothetical protein BMAA1264 [Burkholderia mallei ATCC 23344]
>gi|53716848|ref|YP_104934.1| lipoprotein, putative [Burkholderia mallei ATCC 23344]
>gi|53716850|ref|YP_104932.1| hypothetical protein BMAA0088 [Burkholderia mallei ATCC 23344]
>gi|53716856|ref|YP_105895.1| hypothetical protein BMAA1267 [Burkholderia mallei ATCC 23344]
>gi|53716861|ref|YP_105665.1| amino acid permease [Burkholderia mallei ATCC 23344]
>gi|53716863|ref|YP_105668.1| ATP-dependent helicase, DEAD/DEAH family [Burkholderia mallei ATCC 23344]
>gi|53716864|ref|YP_105669.1| hypothetical protein BMAA0994 [Burkholderia mallei ATCC 23344]
>gi|53716870|ref|YP_105903.1| hypothetical protein BMAA1279 [Burkholderia mallei ATCC 23344]
>gi|53716877|ref|YP_105679.1| transcriptional regulator, TetR family [Burkholderia mallei ATCC 23344]
>gi|53716879|ref|YP_105906.1| lipoprotein NlpD, putative [Burkholderia mallei ATCC 23344]
>gi|53716883|ref|YP_105910.1| opgC protein, putative [Burkholderia mallei ATCC 23344]
>gi|53716885|ref|YP_105912.1| ornithine cyclodeaminase/mu-crystallin family protein [Burkholderia mallei ATCC 23344]
>gi|53716887|ref|YP_105681.1| membrane protein [Burkholderia mallei ATCC 23344]
>gi|53716888|ref|YP_105683.1| hypothetical protein BMAA1011 [Burkholderia mallei ATCC 23344]
>gi|53716900|ref|YP_105687.1| acyl-CoA dehydrogenase domain protein [Burkholderia mallei ATCC 23344]
>gi|53716903|ref|YP_105690.1| thiotemplate mechanism natural product synthetase [Burkholderia mallei ATCC 23344]
>gi|53716904|ref|YP_105691.1| polyketide synthase, putative [Burkholderia mallei ATCC 23344]
>gi|53716908|ref|YP_105921.1| membrane protein, putative [Burkholderia mallei ATCC 23344]
>gi|53716911|ref|YP_105925.1| hypothetical protein BMAA1304 [Burkholderia mallei ATCC 23344]
>gi|53716912|ref|YP_105694.1| hypothetical protein BMAA1025 [Burkholderia mallei ATCC 23344]
>gi|53716918|ref|YP_105700.1| heavy metal resistance protein CzcC [Burkholderia mallei ATCC 23344]
>gi|53716919|ref|YP_105701.1| hypothetical protein BMAA1032 [Burkholderia mallei ATCC 23344]
>gi|53716921|ref|YP_106000.1| hypothetical protein BMAA1388 [Burkholderia mallei ATCC 23344]
>gi|53716924|ref|YP_106003.1| RND efflux system, outer membrane lipoprotein, NodT family [Burkholderia mallei ATCC 23344]
>gi|53716927|ref|YP_105704.1| amino acid permease [Burkholderia mallei ATCC 23344]
>gi|53716928|ref|YP_105705.1| serine protease, rhomboid family [Burkholderia mallei ATCC 23344]
>gi|53716931|ref|YP_106005.1| cyclic nucleotide-binding domain protein [Burkholderia mallei ATCC 23344]
>gi|53716936|ref|YP_105707.1| hypothetical protein BMAA1041 [Burkholderia mallei ATCC 23344]
>gi|53716939|ref|YP_105710.1| efflux transporter, RND family, MFP subunit [Burkholderia mallei ATCC 23344]
>gi|53716941|ref|YP_105712.1| RND efflux system, outer membrane lipoprotein, NodT family [Burkholderia mallei ATCC 23344]
>gi|53716952|ref|YP_106010.1| membrane protein, putative [Burkholderia mallei ATCC 23344]
>gi|53716954|ref|YP_106012.1| hypothetical protein BMAA1404 [Burkholderia mallei ATCC 23344]
>gi|53716957|ref|YP_106015.1| hypothetical protein BMAA1407 [Burkholderia mallei ATCC 23344]
>gi|53716959|ref|YP_105724.1| oxidoreductase, short chain dehydrogenase/reductase family [Burkholderia mallei ATCC 23344]
>gi|53716960|ref|YP_105725.1| potassium efflux system protein, putative [Burkholderia mallei ATCC 23344]
>gi|53716961|ref|YP_105726.1| hypothetical protein BMAA1060 [Burkholderia mallei ATCC 23344]
>gi|53716969|ref|YP_106022.1| oxidoreductase, putative [Burkholderia mallei ATCC 23344]
>gi|53716972|ref|YP_106024.1| oxidoreductase, FAD-binding family protein [Burkholderia mallei ATCC 23344]
>gi|53716974|ref|YP_105801.1| acyl-CoA dehydrogenase domain protein [Burkholderia mallei ATCC 23344]
>gi|53716975|ref|YP_105802.1| hypothetical protein BMAA1149 [Burkholderia mallei ATCC 23344]
>gi|53716982|ref|YP_106028.1| aldehyde dehydrogenase family protein [Burkholderia mallei ATCC 23344]
>gi|53716985|ref|YP_106031.1| methylenomycin A resistance protein, putative [Burkholderia mallei ATCC 23344]
>gi|53716987|ref|YP_105808.1| hypothetical protein BMAA1157 [Burkholderia mallei ATCC 23344]
>gi|53716991|ref|YP_105812.1| cytochrome c oxidase, subunit III family protein [Burkholderia mallei ATCC 23344]
>gi|53716992|ref|YP_106032.1| DoxD-like family protein [Burkholderia mallei ATCC 23344]

>gi|53716993|ref|YP_106033.1| hypothetical protein BMAA1427 [Burkholderia mallei ATCC 23344]
>gi|53716994|ref|YP_106034.1| lactate permease family protein [Burkholderia mallei ATCC 23344]
>gi|53716995|ref|YP_106035.1| hypothetical protein BMAA1429 [Burkholderia mallei ATCC 23344]
>gi|53717003|ref|YP_105816.1| hypothetical protein BMAA1167 [Burkholderia mallei ATCC 23344]
>gi|53717009|ref|YP_106040.1| hypothetical protein BMAA1434 [Burkholderia mallei ATCC 23344]
>gi|53717010|ref|YP_106041.1| monoxygenase [Burkholderia mallei ATCC 23344]
>gi|53717015|ref|YP_106047.1| hypothetical protein BMAA1442 [Burkholderia mallei ATCC 23344]
>gi|53717018|ref|YP_105826.1| branched-chain amino acid ABC transporter, permease protein, putative [Burkholderia mallei ATCC 23344]
>gi|53717020|ref|YP_105828.1| FecR family protein [Burkholderia mallei ATCC 23344]
>gi|53717022|ref|YP_106049.1| thiotemplate mechanism natural product synthetase [Burkholderia mallei ATCC 23344]
>gi|53717026|ref|YP_106053.1| aldehyde dehydrogenase family protein [Burkholderia mallei ATCC 23344]
>gi|53717027|ref|YP_105830.1| hypothetical protein BMAA1183 [Burkholderia mallei ATCC 23344]
>gi|53717029|ref|YP_105832.1| YceI like family protein [Burkholderia mallei ATCC 23344]
>gi|53717031|ref|YP_105835.1| lipoprotein, putative [Burkholderia mallei ATCC 23344]
>gi|53717032|ref|YP_105833.1| hypothetical protein BMAA1186 [Burkholderia mallei ATCC 23344]
>gi|53717033|ref|YP_105836.1| transcriptional regulator, AraC family [Burkholderia mallei ATCC 23344]
>gi|53717036|ref|YP_106055.1| FkbH domain protein [Burkholderia mallei ATCC 23344]
>gi|53717043|ref|YP_105838.1| CAAX protease family protein [Burkholderia mallei ATCC 23344]
>gi|53717047|ref|YP_105842.1| calcium/proton exchanger [Burkholderia mallei ATCC 23344]
>gi|53717049|ref|YP_105844.1| HPP family protein [Burkholderia mallei ATCC 23344]
>gi|53717051|ref|YP_105846.1| voltage-gated chloride channel/CBS domain protein [Burkholderia mallei ATCC 23344]
>gi|53717052|ref|YP_105847.1| drug resistance transporter, EmrB/QacA family [Burkholderia mallei ATCC 23344]
>gi|53717058|ref|YP_105282.1| hypothetical protein BMAA0511 [Burkholderia mallei ATCC 23344]
>gi|53717064|ref|YP_105851.1| malonyl CoA-acyl carrier protein transacylase [Burkholderia mallei ATCC 23344]
>gi|53717068|ref|YP_105277.1| hypothetical protein BMAA0506 [Burkholderia mallei ATCC 23344]
>gi|53717083|ref|YP_105267.1| hypothetical protein BMAA0495 [Burkholderia mallei ATCC 23344]
>gi|53717091|ref|YP_105926.1| membrane protein, putative [Burkholderia mallei ATCC 23344]
>gi|53717102|ref|YP_105931.1| N-hydroxyarylamine O-acetyltransferase [Burkholderia mallei ATCC 23344]
>gi|53717103|ref|YP_105932.1| hypothetical protein BMAA1314 [Burkholderia mallei ATCC 23344]
>gi|53717104|ref|YP_105933.1| hypothetical protein BMAA1315 [Burkholderia mallei ATCC 23344]
>gi|53717112|ref|YP_105249.1| hypothetical protein BMAA0477 [Burkholderia mallei ATCC 23344]
>gi|53717118|ref|YP_105940.1| haemagluttinin family protein [Burkholderia mallei ATCC 23344]
>gi|53717121|ref|YP_105248.1| electron transfer flavoprotein, subunit alpha, putative [Burkholderia mallei ATCC 23344]
>gi|53717131|ref|YP_105945.1| hypothetical protein BMAA1329 [Burkholderia mallei ATCC 23344]
>gi|53717132|ref|YP_105946.1| acetolactate synthase, large subunit, putative [Burkholderia mallei ATCC 23344]
>gi|53717135|ref|YP_105949.1| hydrolase, alpha/beta fold family [Burkholderia mallei ATCC 23344]
>gi|53717140|ref|YP_105611.1| multidrug resistance protein [Burkholderia mallei ATCC 23344]
>gi|53717141|ref|YP_105610.1| RND efflux system, outer membrane lipoprotein, NodT family [Burkholderia mallei ATCC 23344]
>gi|53717144|ref|YP_105951.1| oxidoreductase, short chain dehydrogenase/reductase family [Burkholderia mallei ATCC 23344]
>gi|53717154|ref|YP_105602.1| regulatory protein betI [Burkholderia mallei ATCC 23344]
>gi|53717163|ref|YP_105964.1| hypothetical protein BMAA1348 [Burkholderia mallei ATCC 23344]
>gi|53717176|ref|YP_105970.1| hypothetical protein BMAA1354 [Burkholderia mallei ATCC 23344]
>gi|53717179|ref|YP_105973.1| acetyltransferase, GNAT family [Burkholderia mallei ATCC 23344]
>gi|53717185|ref|YP_105585.1| ribosomal protein S21 [Burkholderia mallei ATCC 23344]
>gi|53717186|ref|YP_105584.1| hypothetical protein BMAA0895 [Burkholderia mallei ATCC 23344]
>gi|53717187|ref|YP_105974.1| LysE family protein [Burkholderia mallei ATCC 23344]
>gi|53717190|ref|YP_105978.1| hypothetical protein BMAA1362 [Burkholderia mallei ATCC 23344]
>gi|53717203|ref|YP_105983.1| oxidoreductase, short chain dehydrogenase/reductase family [Burkholderia mallei ATCC 23344]
>gi|53717204|ref|YP_105985.1| hypothetical protein BMAA1369 [Burkholderia mallei ATCC 23344]
>gi|53717206|ref|YP_105987.1| hypothetical protein BMAA1372 [Burkholderia mallei ATCC 23344]
>gi|53717230|ref|YP_105321.1| hypothetical protein BMAA0559 [Burkholderia mallei ATCC 23344]
>gi|53717232|ref|YP_105324.1| NADPH-dependent FMN reductase family protein [Burkholderia mallei ATCC 23344]
>gi|53717234|ref|YP_105995.1| hypothetical protein BMAA1382 [Burkholderia mallei ATCC 23344]
>gi|53717240|ref|YP_106070.1| hypothetical protein BMAA1469 [Burkholderia mallei ATCC 23344]
>gi|53717241|ref|YP_106071.1| hypothetical protein BMAA1470 [Burkholderia mallei ATCC 23344]
>gi|53717246|ref|YP_105331.1| hypothetical protein BMAA0570 [Burkholderia mallei ATCC 23344]
>gi|53717249|ref|YP_106073.1| hypothetical protein BMAA1472 [Burkholderia mallei ATCC 23344]
>gi|53717251|ref|YP_106075.1| membrane protein, putative [Burkholderia mallei ATCC 23344]
>gi|53717254|ref|YP_106078.1| C4-dicarboxylate anaerobic carrier family protein [Burkholderia mallei ATCC 23344]
>gi|53717258|ref|YP_105336.1| hypothetical protein BMAA0576 [Burkholderia mallei ATCC 23344]
>gi|53717259|ref|YP_105337.1| 2-hydroxy-3-oxopropionate reductase [Burkholderia mallei ATCC 23344]
>gi|53717260|ref|YP_105338.1| transcriptional regulator, GntR family [Burkholderia mallei ATCC 23344]
>gi|53717269|ref|YP_105348.1| hypothetical protein BMAA0588 [Burkholderia mallei ATCC 23344]
>gi|53717274|ref|YP_106081.1| hypothetical protein BMAA1482 [Burkholderia mallei ATCC 23344]
>gi|53717277|ref|YP_105351.1| amino acid permease [Burkholderia mallei ATCC 23344]
>gi|53717280|ref|YP_105354.1| hypothetical protein BMAA0594 [Burkholderia mallei ATCC 23344]
>gi|53717283|ref|YP_105357.1| hypothetical protein BMAA0597 [Burkholderia mallei ATCC 23344]
>gi|53717297|ref|YP_105364.1| membrane protein [Burkholderia mallei ATCC 23344]
>gi|53717302|ref|YP_106092.1| hypothetical protein BMAA1497 [Burkholderia mallei ATCC 23344]
>gi|53717303|ref|YP_106093.1| O-antigen acetylase, putative [Burkholderia mallei ATCC 23344]

>gi|53717307|ref|YP_105368.1| voltage gated chloride channel family protein [Burkholderia mallei ATCC 23344]
>gi|53717308|ref|YP_105370.1| di-haem cytochrome c peroxidase family protein [Burkholderia mallei ATCC 23344]
>gi|53717312|ref|YP_105437.1| hypothetical protein BMAA0695 [Burkholderia mallei ATCC 23344]
>gi|53717314|ref|YP_105439.1| hypothetical protein BMAA0697 [Burkholderia mallei ATCC 23344]
>gi|53717335|ref|YP_105448.1| hypothetical protein BMAA0710 [Burkholderia mallei ATCC 23344]
>gi|53717337|ref|YP_105450.1| major facilitator superfamily protein [Burkholderia mallei ATCC 23344]
>gi|53717349|ref|YP_105454.1| hypothetical protein BMAA0718 [Burkholderia mallei ATCC 23344]
>gi|53717364|ref|YP_105467.1| ATP-dependent Clp protease, ATP-binding subunit ClpB [Burkholderia mallei ATCC 23344]
>gi|53717365|ref|YP_105468.1| hypothetical protein BMAA0739 [Burkholderia mallei ATCC 23344]
>gi|53717366|ref|YP_105469.1| sensor histidine kinase [Burkholderia mallei ATCC 23344]
>gi|53717373|ref|YP_105284.1| hypothetical protein BMAA0514 [Burkholderia mallei ATCC 23344]
>gi|53717376|ref|YP_105471.1| hypothetical protein BMAA0747 [Burkholderia mallei ATCC 23344]
>gi|53717377|ref|YP_105472.1| hemagglutinin domain protein [Burkholderia mallei ATCC 23344]
>gi|53717383|ref|YP_105373.1| hypothetical protein BMAA0613 [Burkholderia mallei ATCC 23344]
>gi|53717386|ref|YP_105376.1| hypothetical protein BMAA0616 [Burkholderia mallei ATCC 23344]
>gi|53717388|ref|YP_105476.1| SCO1/SenC family protein [Burkholderia mallei ATCC 23344]
>gi|53717390|ref|YP_105480.1| NH3-dependent NAD+ synthetase [Burkholderia mallei ATCC 23344]
>gi|53717392|ref|YP_105482.1| membrane protein, putative [Burkholderia mallei ATCC 23344]
>gi|53717395|ref|YP_105379.1| hypothetical protein BMAA0623 [Burkholderia mallei ATCC 23344]
>gi|53717398|ref|YP_105382.1| hypothetical protein BMAA0626 [Burkholderia mallei ATCC 23344]
>gi|53717400|ref|YP_105384.1| aldehyde dehydrogenase (NADP) family protein [Burkholderia mallei ATCC 23344]
>gi|53717405|ref|YP_105488.1| sugar transporter, putative [Burkholderia mallei ATCC 23344]
>gi|53717407|ref|YP_105490.1| hypothetical protein BMAA0771 [Burkholderia mallei ATCC 23344]
>gi|53717409|ref|YP_105492.1| oxidoreductase, FAD-binding [Burkholderia mallei ATCC 23344]
>gi|53717410|ref|YP_105493.1| hypothetical protein BMAA0775 [Burkholderia mallei ATCC 23344]
>gi|53717413|ref|YP_105385.1| 3-hydroxyphenylpropionic acid transporter [Burkholderia mallei ATCC 23344]
>gi|53717416|ref|YP_105388.1| hypothetical protein BMAA0635 [Burkholderia mallei ATCC 23344]
>gi|53717427|ref|YP_105392.1| sugar ABC transporter, permease protein [Burkholderia mallei ATCC 23344]
>gi|53717435|ref|YP_105401.1| Hep_Hag family protein/haemagluttinin motif family protein/YadA-like domain protein [Burkholderia mallei ATCC 23344]
>gi|53717436|ref|YP_105402.1| hypothetical protein BMAA0650 [Burkholderia mallei ATCC 23344]
>gi|53717437|ref|YP_105403.1| H-NS histone family protein [Burkholderia mallei ATCC 23344]
>gi|53717454|ref|YP_105411.1| hypothetical protein BMAA0661 [Burkholderia mallei ATCC 23344]
>gi|53717470|ref|YP_105418.1| hypothetical protein BMAA0669 [Burkholderia mallei ATCC 23344]
>gi|53717486|ref|YP_105427.1| penicillin-binding protein [Burkholderia mallei ATCC 23344]
>gi|53717488|ref|YP_105235.1| RND efflux system, outer membrane lipoprotein, NodT family [Burkholderia mallei ATCC 23344]
>gi|53717490|ref|YP_106599.1| lipoprotein, putative [Burkholderia mallei ATCC 23344]
>gi|53717493|ref|YP_105236.1| multidrug resistance protein, putative [Burkholderia mallei ATCC 23344]
>gi|53717496|ref|YP_105431.1| hypothetical protein BMAA0688 [Burkholderia mallei ATCC 23344]
>gi|53717509|ref|YP_105435.1| hypothetical protein BMAA0693 [Burkholderia mallei ATCC 23344]
>gi|53717512|ref|YP_105498.1| ApbE family protein [Burkholderia mallei ATCC 23344]
>gi|53717516|ref|YP_105047.1| chemotaxis sensor histidine kinase, putative [Burkholderia mallei ATCC 23344]
>gi|53717518|ref|YP_105501.1| sensor histidine kinase [Burkholderia mallei ATCC 23344]
>gi|53717519|ref|YP_105502.1| hypothetical protein BMAA0786 [Burkholderia mallei ATCC 23344]
>gi|53717522|ref|YP_105505.1| hypothetical protein BMAA0789 [Burkholderia mallei ATCC 23344]
>gi|53717524|ref|YP_105506.1| hypothetical protein BMAA0792 [Burkholderia mallei ATCC 23344]
>gi|53717545|ref|YP_105520.1| YadA-like C-terminal region protein [Burkholderia mallei ATCC 23344]
>gi|53717547|ref|YP_105523.1| hypothetical protein BMAA0813 [Burkholderia mallei ATCC 23344]
>gi|53717556|ref|YP_105532.1| hypothetical protein BMAA0823 [Burkholderia mallei ATCC 23344]
>gi|53717560|ref|YP_105537.1| DNA-binding response regulator [Burkholderia mallei ATCC 23344]
>gi|53717561|ref|YP_105536.1| sensor histidine kinase [Burkholderia mallei ATCC 23344]
>gi|53717562|ref|YP_105538.1| hypothetical protein BMAA0832 [Burkholderia mallei ATCC 23344]
>gi|53717577|ref|YP_105041.1| hypothetical protein BMAA0215 [Burkholderia mallei ATCC 23344]
>gi|53717578|ref|YP_105052.1| hypothetical protein BMAA0228 [Burkholderia mallei ATCC 23344]
>gi|53717587|ref|YP_105463.1| hypothetical protein BMAA0733 [Burkholderia mallei ATCC 23344]
>gi|53717588|ref|YP_105465.1| hypothetical protein BMAA0735 [Burkholderia mallei ATCC 23344]
>gi|53717589|ref|YP_105539.1| immunity protein, putative [Burkholderia mallei ATCC 23344]
>gi|53717594|ref|YP_105999.1| hypothetical protein BMAA1387 [Burkholderia mallei ATCC 23344]
>gi|53717601|ref|YP_106438.1| hypothetical protein BMAA1899 [Burkholderia mallei ATCC 23344]
>gi|53717602|ref|YP_106513.1| response regulator [Burkholderia mallei ATCC 23344]
>gi|53717604|ref|YP_106606.1| hypothetical protein BMAA2099 [Burkholderia mallei ATCC 23344]
>gi|53717605|ref|YP_106608.1| hypothetical protein BMAA2100.1 [Burkholderia mallei ATCC 23344]
>gi|53717606|ref|YP_106574.1| hypothetical protein BMAA2061.1 [Burkholderia mallei ATCC 23344]
>gi|53717607|ref|YP_106562.1| hypothetical protein BMAA2049.1 [Burkholderia mallei ATCC 23344]
>gi|53717609|ref|YP_106545.1| hypothetical protein BMAA2028.1 [Burkholderia mallei ATCC 23344]
>gi|53717610|ref|YP_106541.1| hypothetical protein BMAA2024.1 [Burkholderia mallei ATCC 23344]
>gi|53717611|ref|YP_106498.1| hypothetical protein BMAA1975.1 [Burkholderia mallei ATCC 23344]
>gi|53717612|ref|YP_104911.1| hypothetical protein BMAA0057.1 [Burkholderia mallei ATCC 23344]
>gi|53717618|ref|YP_105590.1| hypothetical protein BMAA0902.1 [Burkholderia mallei ATCC 23344]
>gi|53717619|ref|YP_105757.1| hypothetical protein BMAA1093.1 [Burkholderia mallei ATCC 23344]

>gi|53717620|ref|YP_105879.1| hypothetical protein BMAA1246.1 [Burkholderia mallei ATCC 23344]
>gi|53717621|ref|YP_105881.1| hypothetical protein BMAA1248.1 [Burkholderia mallei ATCC 23344]
>gi|53717623|ref|YP_106042.1| hypothetical protein BMAA1436.1 [Burkholderia mallei ATCC 23344]
>gi|53717624|ref|YP_106180.1| hypothetical protein BMAA1597.1 [Burkholderia mallei ATCC 23344]
>gi|53717625|ref|YP_106235.1| hypothetical protein BMAA1664.1 [Burkholderia mallei ATCC 23344]
>gi|53717628|ref|YP_106526.1| hypothetical protein BMAA2006.1 [Burkholderia mallei ATCC 23344]
>gi|53717629|ref|YP_106570.1| hypothetical protein BMAA2057.1 [Burkholderia mallei ATCC 23344]
>gi|53717631|ref|YP_105196.1| serine/threonine protein kinase [Burkholderia mallei ATCC 23344]
>gi|53717633|ref|YP_106246.1| L-seryl-tRNA selenium transferase [Burkholderia mallei ATCC 23344]
>gi|53717635|ref|YP_104963.1| ATP synthase gene 1, putative [Burkholderia mallei ATCC 23344]
>gi|53717637|ref|YP_105825.1| ribose transport system permease protein RbsC, internal deletion [Burkholderia mallei ATCC 23344]
>gi|46198309|ref|YP_003976.1| hypothetical protein TTC0001 [Thermus thermophilus HB27]
>gi|46198312|ref|YP_003979.1| hypothetical protein TTC0004 [Thermus thermophilus HB27]
>gi|46198316|ref|YP_003983.1| O-sialoglycoprotein endopeptidase [Thermus thermophilus HB27]
>gi|46198317|ref|YP_003984.1| sugar ABC transporter, permease protein [Thermus thermophilus HB27]
>gi|46198321|ref|YP_003988.1| hypothetical protein TTC0013 [Thermus thermophilus HB27]
>gi|46198322|ref|YP_003989.1| hypothetical protein TTC0014 [Thermus thermophilus HB27]
>gi|46198326|ref|YP_003993.1| Sec-independent protein translocase protein tatA [Thermus thermophilus HB27]
>gi|46198327|ref|YP_003994.1| Sec-independent protein translocase protein tatC [Thermus thermophilus HB27]
>gi|46198332|ref|YP_003999.1| probable methylmalonyl-coA epimerase [Thermus thermophilus HB27]
>gi|46198333|ref|YP_004000.1| hypothetical membrane spanning protein [Thermus thermophilus HB27]
>gi|46198337|ref|YP_004004.1| hypothetical membrane spanning protein [Thermus thermophilus HB27]
>gi|46198338|ref|YP_004005.1| hypothetical membrane spanning protein [Thermus thermophilus HB27]
>gi|46198339|ref|YP_004006.1| hypothetical protein TTC0031 [Thermus thermophilus HB27]
>gi|46198342|ref|YP_004009.1| hypothetical protein TTC0034 [Thermus thermophilus HB27]
>gi|46198344|ref|YP_004011.1| hypothetical protein TTC0036 [Thermus thermophilus HB27]
>gi|46198345|ref|YP_004012.1| hypothetical protein TTC0037 [Thermus thermophilus HB27]
>gi|46198352|ref|YP_004019.1| dNTP triphosphohydrolase [Thermus thermophilus HB27]
>gi|46198361|ref|YP_004028.1| hypothetical protein TTC0053 [Thermus thermophilus HB27]
>gi|46198363|ref|YP_004030.1| hypothetical protein TTC0055 [Thermus thermophilus HB27]
>gi|46198365|ref|YP_004032.1| NADH dehydrogenase [Thermus thermophilus HB27]
>gi|46198366|ref|YP_004033.1| hypothetical protein TTC0058 [Thermus thermophilus HB27]
>gi|46198367|ref|YP_004034.1| hypothetical protein TTC0059 [Thermus thermophilus HB27]
>gi|46198369|ref|YP_004036.1| imidazoleglycerol-phosphate dehydratase [Thermus thermophilus HB27]
>gi|46198373|ref|YP_004040.1| hypothetical protein TTC0065 [Thermus thermophilus HB27]
>gi|46198374|ref|YP_004041.1| hypothetical protein TTC0066 [Thermus thermophilus HB27]
>gi|46198377|ref|YP_004044.1| hypothetical protein TTC0069 [Thermus thermophilus HB27]
>gi|46198380|ref|YP_004047.1| hypothetical protein TTC0072 [Thermus thermophilus HB27]
>gi|46198381|ref|YP_004048.1| methyltransferase [Thermus thermophilus HB27]
>gi|46198382|ref|YP_004049.1| probable jag protein [Thermus thermophilus HB27]
>gi|46198388|ref|YP_004055.1| branched-chain amino acid transport system permease protein livH [Thermus thermophilus HB27]
>gi|46198389|ref|YP_004056.1| branched-chain amino acid transport system permease protein livM [Thermus thermophilus HB27]
>gi|46198393|ref|YP_004060.1| manganese transport system membrane protein mntB [Thermus thermophilus HB27]
>gi|46198395|ref|YP_004062.1| cysteine desulfhydrase/selenocysteine lyase [Thermus thermophilus HB27]
>gi|46198397|ref|YP_004064.1| cytidylate kinase [Thermus thermophilus HB27]
>gi|46198398|ref|YP_004065.1| hypothetical protein TTC0090 [Thermus thermophilus HB27]
>gi|46198408|ref|YP_004075.1| oligopeptide transport system permease protein appC [Thermus thermophilus HB27]
>gi|46198411|ref|YP_004078.1| acetoin utilization acuB protein [Thermus thermophilus HB27]
>gi|46198413|ref|YP_004080.1| hypothetical protein TTC0105 [Thermus thermophilus HB27]
>gi|46198418|ref|YP_004085.1| apolipoprotein N-acyltransferase [Thermus thermophilus HB27]
>gi|46198421|ref|YP_004088.1| hypothetical protein TTC0113 [Thermus thermophilus HB27]
>gi|46198423|ref|YP_004090.1| homoserine dehydrogenase [Thermus thermophilus HB27]
>gi|46198427|ref|YP_004094.1| hypothetical protein TTC0119 [Thermus thermophilus HB27]
>gi|46198428|ref|YP_004095.1| hypothetical protein TTC0120 [Thermus thermophilus HB27]
>gi|46198436|ref|YP_004103.1| (S)-2-hydroxy-acid oxidase subunit glcE [Thermus thermophilus HB27]
>gi|46198446|ref|YP_004113.1| phosphoribosylaminoimidazole carboxylase catalytic subunit [Thermus thermophilus HB27]
>gi|46198447|ref|YP_004114.1| sodium bicarbonate cotransporter [Thermus thermophilus HB27]
>gi|46198449|ref|YP_004116.1| hypothetical protein TTC0141 [Thermus thermophilus HB27]
>gi|46198453|ref|YP_004120.1| hypothetical protein TTC0145 [Thermus thermophilus HB27]
>gi|46198461|ref|YP_004128.1| hypothetical membrane spanning protein [Thermus thermophilus HB27]
>gi|46198466|ref|YP_004133.1| probable two-component sensor [Thermus thermophilus HB27]
>gi|46198471|ref|YP_004138.1| hypothetical membrane spanning protein [Thermus thermophilus HB27]
>gi|46198480|ref|YP_004147.1| hypothetical protein TTC0172 [Thermus thermophilus HB27]
>gi|46198481|ref|YP_004148.1| DNA repair protein radA [Thermus thermophilus HB27]
>gi|46198484|ref|YP_004151.1| hypothetical protein TTC0176 [Thermus thermophilus HB27]
>gi|46198486|ref|YP_004153.1| hemolysin [Thermus thermophilus HB27]
>gi|46198488|ref|YP_004155.1| hypothetical protein TTC0180 [Thermus thermophilus HB27]
>gi|46198494|ref|YP_004161.1| hypothetical protein TTC0186 [Thermus thermophilus HB27]
>gi|46198495|ref|YP_004162.1| hypothetical membrane spanning protein [Thermus thermophilus HB27]
>gi|46198496|ref|YP_004163.1| hypothetical protein TTC0188 [Thermus thermophilus HB27]

>gi|46198504|ref|YP_004171.1| hypothetical protein TTC0196 [Thermus thermophilus HB27]
>gi|46198508|ref|YP_004175.1| hypothetical protein TTC0200 [Thermus thermophilus HB27]
>gi|46198514|ref|YP_004181.1| hypothetical protein TTC0206 [Thermus thermophilus HB27]
>gi|46198515|ref|YP_004182.1| hypothetical protein TTC0207 [Thermus thermophilus HB27]
>gi|46198517|ref|YP_004184.1| hypothetical protein TTC0209 [Thermus thermophilus HB27]
>gi|46198520|ref|YP_004187.1| hypothetical protein TTC0212 [Thermus thermophilus HB27]
>gi|46198524|ref|YP_004191.1| branched-chain amino acid transport system permease protein livM [Thermus thermophilus HB27]
>gi|46198526|ref|YP_004193.1| branched-chain amino acid transport ATP-binding protein livG [Thermus thermophilus HB27]
>gi|46198528|ref|YP_004195.1| branched-chain amino acid transport system permease protein livH [Thermus thermophilus HB27]
>gi|46198537|ref|YP_004204.1| hypothetical membrane spanning protein [Thermus thermophilus HB27]
>gi|46198541|ref|YP_004208.1| probable peptidyl-prolyl cis-trans isomerase [Thermus thermophilus HB27]
>gi|46198542|ref|YP_004209.1| hypothetical protein TTC0234 [Thermus thermophilus HB27]
>gi|46198548|ref|YP_004215.1| 4-hydroxybenzoate octaprenyltransferase [Thermus thermophilus HB27]
>gi|46198557|ref|YP_004224.1| trigger factor, ppiase [Thermus thermophilus HB27]
>gi|46198564|ref|YP_004231.1| hypothetical protein TTC0256 [Thermus thermophilus HB27]
>gi|46198566|ref|YP_004233.1| hypothetical protein TTC0258 [Thermus thermophilus HB27]
>gi|46198568|ref|YP_004235.1| phosphoenolpyruvate carboxylase [Thermus thermophilus HB27]
>gi|46198571|ref|YP_004238.1| hypothetical protein TTC0263 [Thermus thermophilus HB27]
>gi|46198577|ref|YP_004244.1| nucleotidyltransferase [Thermus thermophilus HB27]
>gi|46198582|ref|YP_004249.1| hypothetical protein TTC0274 [Thermus thermophilus HB27]
>gi|46198583|ref|YP_004250.1| undecaprenyl-phosphate galactosephosphotransferase [Thermus thermophilus HB27]
>gi|46198584|ref|YP_004251.1| second mannosyl transferase [Thermus thermophilus HB27]
>gi|46198589|ref|YP_004256.1| hypothetical protein TTC0281 [Thermus thermophilus HB27]
>gi|46198591|ref|YP_004258.1| hypothetical protein TTC0283 [Thermus thermophilus HB27]
>gi|46198602|ref|YP_004269.1| hypothetical protein TTC0294 [Thermus thermophilus HB27]
>gi|46198604|ref|YP_004271.1| probable transcriptional regulator [Thermus thermophilus HB27]
>gi|46198607|ref|YP_004274.1| hypothetical protein TTC0299 [Thermus thermophilus HB27]
>gi|46198608|ref|YP_004275.1| transporter [Thermus thermophilus HB27]
>gi|46198610|ref|YP_004277.1| hypothetical protein TTC0302 [Thermus thermophilus HB27]
>gi|46198613|ref|YP_004280.1| quinone oxidoreductase [Thermus thermophilus HB27]
>gi|46198617|ref|YP_004284.1| hypothetical membrane spanning protein [Thermus thermophilus HB27]
>gi|46198620|ref|YP_004287.1| uroporphyrinogen-III synthase [Thermus thermophilus HB27]
>gi|46198622|ref|YP_004289.1| hypothetical protein TTC0314 [Thermus thermophilus HB27]
>gi|46198626|ref|YP_004293.1| putative oxidoreductase [Thermus thermophilus HB27]
>gi|46198628|ref|YP_004295.1| sugar transport protein [Thermus thermophilus HB27]
>gi|46198629|ref|YP_004296.1| phosphomethylpyrimidine kinase/hydroxymethylpyrimidine kinase [Thermus thermophilus HB27]
>gi|46198631|ref|YP_004298.1| hypothetical protein TTC0323 [Thermus thermophilus HB27]
>gi|46198632|ref|YP_004299.1| putative biopolymer transport exbD protein [Thermus thermophilus HB27]
>gi|46198637|ref|YP_004304.1| transcriptional repressor [Thermus thermophilus HB27]
>gi|46198642|ref|YP_004309.1| branched-chain amino acid transport ATP-binding protein livF [Thermus thermophilus HB27]
>gi|46198643|ref|YP_004310.1| branched-chain amino acid transport ATP-binding protein livG [Thermus thermophilus HB27]
>gi|46198644|ref|YP_004311.1| branched amino acid transport system permease [Thermus thermophilus HB27]
>gi|46198645|ref|YP_004312.1| branched-chain amino acid transport system permease protein livH [Thermus thermophilus HB27]
>gi|46198649|ref|YP_004316.1| putative hydrolase [Thermus thermophilus HB27]
>gi|46198652|ref|YP_004319.1| hypothetical protein TTC0344 [Thermus thermophilus HB27]
>gi|46198654|ref|YP_004321.1| hypothetical protein TTC0346 [Thermus thermophilus HB27]
>gi|46198661|ref|YP_004328.1| transcriptional repressor smtB [Thermus thermophilus HB27]
>gi|46198662|ref|YP_004329.1| cation-transporting ATPase [Thermus thermophilus HB27]
>gi|46198663|ref|YP_004330.1| pyridoxine biosynthesis amidotransferase [Thermus thermophilus HB27]
>gi|46198665|ref|YP_004332.1| hydrolase (HAD superfamily) [Thermus thermophilus HB27]
>gi|46198668|ref|YP_004335.1| histidyl-tRNA synthetase [Thermus thermophilus HB27]
>gi|46198669|ref|YP_004336.1| hypothetical protein TTC0361 [Thermus thermophilus HB27]
>gi|46198670|ref|YP_004337.1| hypothetical protein TTC0362 [Thermus thermophilus HB27]
>gi|46198671|ref|YP_004338.1| ABC transporter ATP-binding protein [Thermus thermophilus HB27]
>gi|46198672|ref|YP_004339.1| molybdenum transport system permease protein modB [Thermus thermophilus HB27]
>gi|46198673|ref|YP_004340.1| molybdate-binding protein [Thermus thermophilus HB27]
>gi|46198675|ref|YP_004342.1| hypothetical protein TTC0367 [Thermus thermophilus HB27]
>gi|46198676|ref|YP_004343.1| hypothetical protein TTC0368 [Thermus thermophilus HB27]
>gi|46198677|ref|YP_004344.1| hypothetical protein TTC0369 [Thermus thermophilus HB27]
>gi|46198684|ref|YP_004351.1| carboxymethylenebutenolidase-related protein [Thermus thermophilus HB27]
>gi|46198685|ref|YP_004352.1| acriflavin resistance protein B [Thermus thermophilus HB27]
>gi|46198686|ref|YP_004353.1| hypothetical membrane spanning protein [Thermus thermophilus HB27]
>gi|46198687|ref|YP_004354.1| hypothetical protein TTC0379 [Thermus thermophilus HB27]
>gi|46198688|ref|YP_004355.1| hypothetical protein TTC0380 [Thermus thermophilus HB27]
>gi|46198689|ref|YP_004356.1| transcriptional regulator, marR family [Thermus thermophilus HB27]
>gi|46198690|ref|YP_004357.1| probable hemolysin-related protein/CBS domain containing protein [Thermus thermophilus HB27]
>gi|46198692|ref|YP_004359.1| hypothetical protein TTC0384 [Thermus thermophilus HB27]
>gi|46198694|ref|YP_004361.1| hypothetical protein TTC0386 [Thermus thermophilus HB27]
>gi|46198696|ref|YP_004363.1| sensory transduction protein kinase [Thermus thermophilus HB27]
>gi|46198699|ref|YP_004366.1| iron(III) dicitrate transport system permease protein fecD [Thermus thermophilus HB27]

>gi|46198700|ref|YP_004367.1| iron(III) dicitrate transport ATP-binding protein fecE [Thermus thermophilus HB27]
>gi|46198703|ref|YP_004370.1| hypothetical protein TTC0395 [Thermus thermophilus HB27]
>gi|46198705|ref|YP_004372.1| L-allo-threonine aldolase [Thermus thermophilus HB27]
>gi|46198706|ref|YP_004373.1| transcriptional repressor [Thermus thermophilus HB27]
>gi|46198708|ref|YP_004375.1| hypothetical protein TTC0400 [Thermus thermophilus HB27]
>gi|46198717|ref|YP_004384.1| sufE protein [Thermus thermophilus HB27]
>gi|46198719|ref|YP_004386.1| dehydrogenase family protein (alcohol) [Thermus thermophilus HB27]
>gi|46198721|ref|YP_004388.1| endo-type 6-aminohexanoate oligomer hydrolase [Thermus thermophilus HB27]
>gi|46198727|ref|YP_004394.1| ABC transporter permease protein [Thermus thermophilus HB27]
>gi|46198730|ref|YP_004397.1| sensory transduction histidine kinase [Thermus thermophilus HB27]
>gi|46198731|ref|YP_004398.1| hypothetical protein TTC0423 [Thermus thermophilus HB27]
>gi|46198734|ref|YP_004401.1| dihydroorotase [Thermus thermophilus HB27]
>gi|46198737|ref|YP_004404.1| hypothetical membrane spanning protein [Thermus thermophilus HB27]
>gi|46198738|ref|YP_004405.1| putative permease [Thermus thermophilus HB27]
>gi|46198740|ref|YP_004407.1| pmbA protein [Thermus thermophilus HB27]
>gi|46198742|ref|YP_004409.1| hypothetical cytosolic protein [Thermus thermophilus HB27]
>gi|46198744|ref|YP_004411.1| acetyltransferase [Thermus thermophilus HB27]
>gi|46198745|ref|YP_004412.1| hypothetical membrane associated protein [Thermus thermophilus HB27]
>gi|46198746|ref|YP_004413.1| hypothetical cytosolic protein [Thermus thermophilus HB27]
>gi|46198754|ref|YP_004421.1| putative response regulator [Thermus thermophilus HB27]
>gi|46198755|ref|YP_004422.1| prephenate dehydrogenase [Thermus thermophilus HB27]
>gi|46198759|ref|YP_004426.1| hypothetical cytosolic protein [Thermus thermophilus HB27]
>gi|46198762|ref|YP_004429.1| hypothetical membrane spanning protein [Thermus thermophilus HB27]
>gi|46198767|ref|YP_004434.1| phosphoribosylglycinamide formyltransferase [Thermus thermophilus HB27]
>gi|46198769|ref|YP_004436.1| DNA polymerase III epsilon chain-like protein [Thermus thermophilus HB27]
>gi|46198770|ref|YP_004437.1| transcriptional regulator [Thermus thermophilus HB27]
>gi|46198771|ref|YP_004438.1| sensory transduction histidine kinase [Thermus thermophilus HB27]
>gi|46198772|ref|YP_004439.1| hypothetical protein TTC0464 [Thermus thermophilus HB27]
>gi|46198774|ref|YP_004441.1| hypothetical protein TTC0466 [Thermus thermophilus HB27]
>gi|46198778|ref|YP_004445.1| glutathione-regulated potassium-efflux system protein kefC [Thermus thermophilus HB27]
>gi|46198782|ref|YP_004449.1| cell division protein ftsK [Thermus thermophilus HB27]
>gi|46198784|ref|YP_004451.1| hypothetical protein TTC0476 [Thermus thermophilus HB27]
>gi|46198786|ref|YP_004453.1| hypothetical protein TTC0478 [Thermus thermophilus HB27]
>gi|46198787|ref|YP_004454.1| tRNA nucleotidyltransferase [Thermus thermophilus HB27]
>gi|46198789|ref|YP_004456.1| membrane metalloprotease [Thermus thermophilus HB27]
>gi|46198791|ref|YP_004458.1| putative permease [Thermus thermophilus HB27]
>gi|46198796|ref|YP_004463.1| thiol:disulfide interchange protein tlpA [Thermus thermophilus HB27]
>gi|46198798|ref|YP_004465.1| glycosyltransferase [Thermus thermophilus HB27]
>gi|46198800|ref|YP_004467.1| hypothetical protein TTC0492 [Thermus thermophilus HB27]
>gi|46198802|ref|YP_004469.1| putative Zn-dependent hydrolase (beta-lactamase superfamily) [Thermus thermophilus HB27]
>gi|46198804|ref|YP_004471.1| regulatory protein recX [Thermus thermophilus HB27]
>gi|46198806|ref|YP_004473.1| stage V sporulation protein S [Thermus thermophilus HB27]
>gi|46198807|ref|YP_004474.1| 16S rRNA m(5)C 967 methyltransferase [Thermus thermophilus HB27]
>gi|46198809|ref|YP_004476.1| hypothetical protein TTC0501 [Thermus thermophilus HB27]
>gi|46198811|ref|YP_004478.1| membrane metalloprotease [Thermus thermophilus HB27]
>gi|46198812|ref|YP_004479.1| 1-deoxy-D-xylulose 5-phosphate reductoisomerase [Thermus thermophilus HB27]
>gi|46198824|ref|YP_004491.1| hypothetical membrane spanning protein [Thermus thermophilus HB27]
>gi|46198829|ref|YP_004496.1| glutamyl-tRNA(Gln) amidotransferase subunit C [Thermus thermophilus HB27]
>gi|46198830|ref|YP_004497.1| hypothetical membrane spanning protein [Thermus thermophilus HB27]
>gi|46198831|ref|YP_004498.1| methyltransferase [Thermus thermophilus HB27]
>gi|46198833|ref|YP_004500.1| hypothetical protein TTC0525 [Thermus thermophilus HB27]
>gi|46198838|ref|YP_004505.1| lipopolysaccharide core biosynthesis protein rfaG [Thermus thermophilus HB27]
>gi|46198847|ref|YP_004514.1| hypothetical protein TTC0539 [Thermus thermophilus HB27]
>gi|46198848|ref|YP_004515.1| hypothetical membrane spanning protein [Thermus thermophilus HB27]
>gi|46198849|ref|YP_004516.1| hypothetical protein TTC0541 [Thermus thermophilus HB27]
>gi|46198851|ref|YP_004518.1| chromosome partition protein smc [Thermus thermophilus HB27]
>gi|46198853|ref|YP_004520.1| hypothetical protein TTC0545 [Thermus thermophilus HB27]
>gi|46198855|ref|YP_004522.1| hypothetical protein TTC0547 [Thermus thermophilus HB27]
>gi|46198856|ref|YP_004523.1| probable bioY protein [Thermus thermophilus HB27]
>gi|46198859|ref|YP_004526.1| hypothetical protein TTC0551 [Thermus thermophilus HB27]
>gi|46198863|ref|YP_004530.1| hypothetical protein TTC0555 [Thermus thermophilus HB27]
>gi|46198864|ref|YP_004531.1| poly(A) polymerase/tRNA nucleotidyl transferase [Thermus thermophilus HB27]
>gi|46198865|ref|YP_004532.1| dephospho-CoA kinase [Thermus thermophilus HB27]
>gi|46198866|ref|YP_004533.1| hypothetical protein TTC0558 [Thermus thermophilus HB27]
>gi|46198870|ref|YP_004537.1| thiophene and furan oxidation protein thdF [Thermus thermophilus HB27]
>gi|46198871|ref|YP_004538.1| Na(+)/H(+) antiporter [Thermus thermophilus HB27]
>gi|46198873|ref|YP_004540.1| quaternary ammonium compound-resistance protein [Thermus thermophilus HB27]
>gi|46198874|ref|YP_004541.1| hypothetical protein TTC0566 [Thermus thermophilus HB27]
>gi|46198876|ref|YP_004543.1| pyruvate dehydrogenase E1 component beta subunit [Thermus thermophilus HB27]
>gi|46198877|ref|YP_004544.1| pyruvate decarboxylase alpha subunit-like protein [Thermus thermophilus HB27]

>gi|46198878|ref|YP_004545.1| hypothetical protein TTC0570 [Thermus thermophilus HB27]
>gi|46198881|ref|YP_004548.1| ABC transporter ATP-binding protein [Thermus thermophilus HB27]
>gi|46198882|ref|YP_004549.1| hypothetical protein TTC0574 [Thermus thermophilus HB27]
>gi|46198883|ref|YP_004550.1| hypothetical protein TTC0575 [Thermus thermophilus HB27]
>gi|46198887|ref|YP_004554.1| hypothetical protein TTC0579 [Thermus thermophilus HB27]
>gi|46198897|ref|YP_004564.1| mannosyl-3-phosphoglycerate phosphatase [Thermus thermophilus HB27]
>gi|46198898|ref|YP_004565.1| hypothetical protein TTC0590 [Thermus thermophilus HB27]
>gi|46198903|ref|YP_004570.1| probable oxidoreductase [Thermus thermophilus HB27]
>gi|46198906|ref|YP_004573.1| branched-chain amino acid transport system permease protein livH [Thermus thermophilus HB27]
>gi|46198907|ref|YP_004574.1| branched-chain amino acid transport system permease protein livM/branched-chain amino acid transport ATP-binding protein livG [Thermus thermophilus HB27]
>gi|46198908|ref|YP_004575.1| branched-chain amino acid transport ATP-binding protein livF [Thermus thermophilus HB27]
>gi|46198913|ref|YP_004580.1| phenylacetic acid degradation protein paaD [Thermus thermophilus HB27]
>gi|46198921|ref|YP_004588.1| sugar ABC transporter, permease protein [Thermus thermophilus HB27]
[truncated: 3,049,479 more chars]
